# Supplementary material for: Discovery of diverse chimeric peptides in a eukaryotic proteome sets the stage for experimental validation of the mosaic translation hypothesis
Source: Comput Struct Biotechnol J. 2025 Sep 12;27:4048–64. doi: 10.1016/j.csbj.2025.09.019 (PMC12481079; doi:10.1016/j.csbj.2025.09.019)
Supplement: Supplementary file 1 — Supplementary material [file mmc1.zip › Supplementary Datasets/Supplementary Dataset S2 Graphical summary.pdf]

CP1: MtrunA17_Chr0c01g0489091_1F_199-492_294_MtrunA17_Chr0c01g0489091_3F_384-629_246_-1_iteration_2

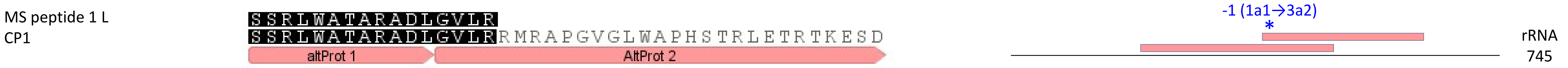

CP2: MtrunA17_Chr0c28g0493951_2F_422-661_240_MtrunA17_Chr0c28g0493951_3F_498-1307_810_-2_iteration_6

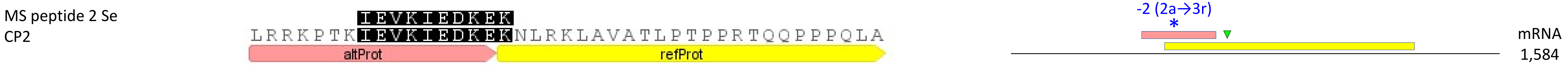

CP3: MtrunA17_Chr1g0148371_1F_2233-2337_105_MtrunA17_Chr1g0148371_3F_198-2927_2730_-1_iteration_16

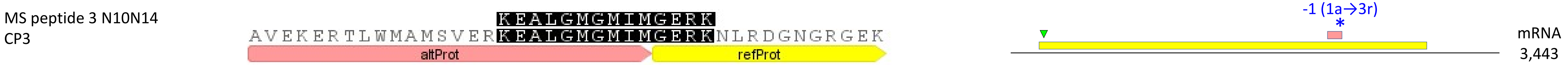

CP4: MtrunA17_Chr1g0149991_2F_1847-2362_516_MtrunA17_Chr1g0149991_3F_171-1910_1740_+2_iteration_0

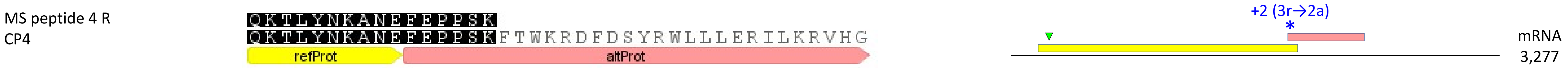

CP5: MtrunA17_Chr1g0150571_3F_1035-1250_216_MtrunA17_Chr1g0150571_2F_224-1192_969_-2_iteration_15

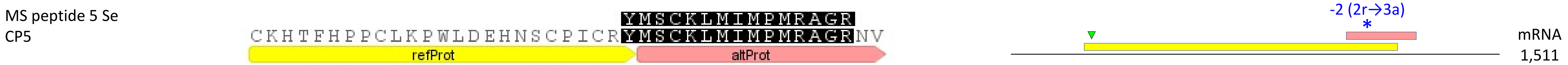

CP6: MtrunA17_Chr1g0152521_2F_290-436_147_MtrunA17_Chr1g0152521_1F_1-969_969_-2_iteration_20

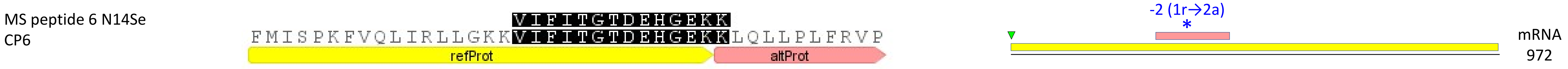

CP7: MtrunA17_Chr1g0153001_1F_178-519_342_MtrunA17_Chr1g0153001_3F_396-806_411_-1_iteration_9

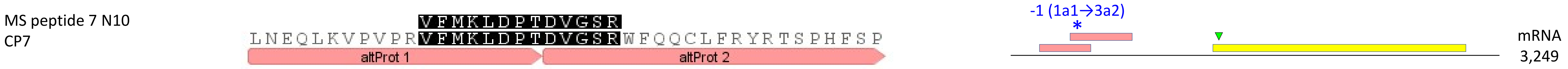

CP8: MtrunA17_Chr1g0155251_1F_1228-1467_240_MtrunA17_Chr1g0155251_3F_138-2090_1953_-1_iteration_16

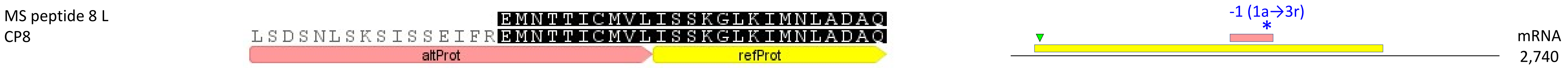

CP9: MtrunA17_Chr1g0158341_2F_758-859_102_MtrunA17_Chr1g0158341_1F_1-1251_1251_+2_iteration_17

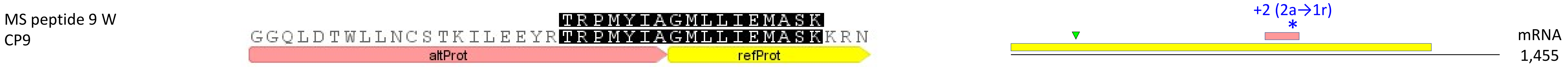

CP10: MtrunA17_Chr1g0162101_3F_3-221_219_MtrunA17_Chr1g0162101_1F_1-255_255_+2_iteration_1

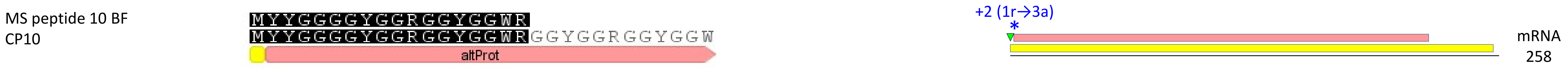

CP11: MtrunA17_Chr1g0164591_3F_3-338_336_MtrunA17_Chr1g0164591_2F_314-1369_1056_-1_iteration_2

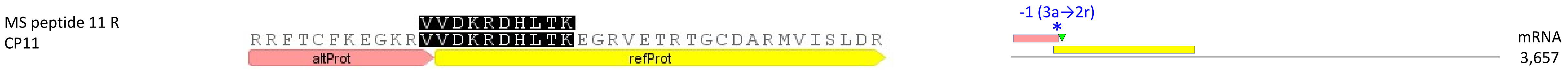

CP12: MtrunA17_Chr1g0178361_1F_529-618_90_MtrunA17_Chr1g0178361_3F_201-1124_924_-1_iteration_9

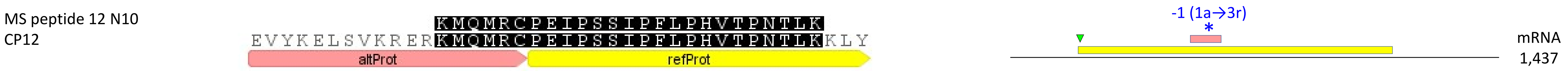

CP13: MtrunA17_Chr1g0181761_1F_445-570_126_MtrunA17_Chr1g0181761_3F_18-1262_1245_+2_iteration_0

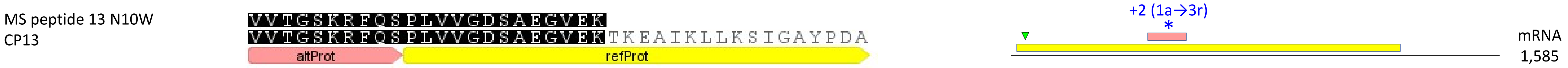

CP14: MtrunA17_Chr1g0182591_1F_4162-4257_96_MtrunA17_Chr1g0182591_2F_11-4228_4218_-1_iteration_11

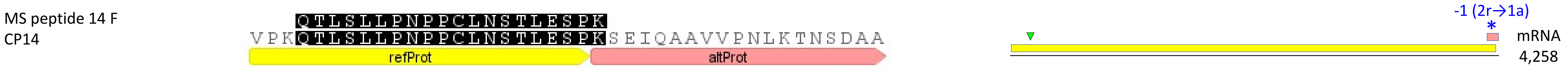

CP15: MtrunA17_Chr1g0183001_1F_2749-2943_195_MtrunA17_Chr1g0183001_3F_375-4916_4542_-2_iteration_16

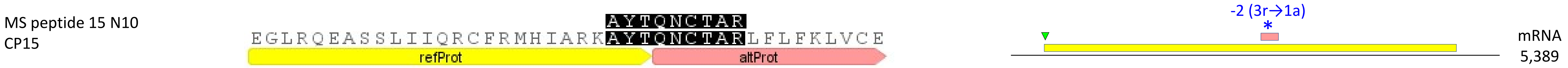

CP16: MtrunA17_Chr1g0185811_1F_535-621_87_MtrunA17_Chr1g0185811_2F_2-1774_1773_+1_iteration_10

MS peptide 16 Se: FNPQSIYLGGNPEVEFPETQER

CP16: SSRHLQLCKPARFNPQSIYLGGNPEVEFPETQERQQGR

altProt | refProt

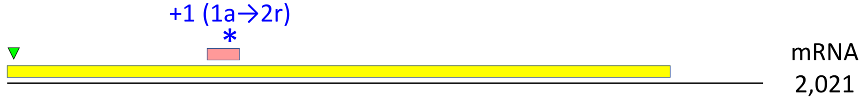

CP17: MtrunA17_Chr1g0185811_1F_535-621_87_MtrunA17_Chr1g0185811_2F_2-1774_1773_+1_iteration_2

MS peptide 17 Se: PLDSTPRVFYLGGNPEVEFPETQER

CP17: SSRHLQLCKPLDSTPRVFYLGGNPEVEFPETQERQQGR

altProt | refProt

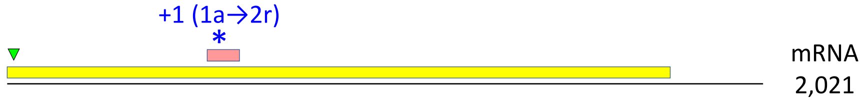

CP18: MtrunA17_Chr1g0185811_1F_535-621_87_MtrunA17_Chr1g0185811_2F_2-1774_1773_+1_iteration_9

MS peptide 18 Se: FNPQSFYLGGNPEVEFPETQER

CP18: SSRHLQLCKPARFNPQSFYLGGNPEVEFPETQERQQGR

altProt | refProt

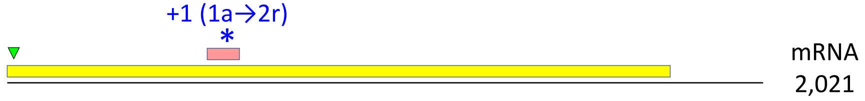

CP19: MtrunA17_Chr1g0185871_1F_1447-1602_156_MtrunA17_Chr1g0185871_3F_309-3371_3063_-2_iteration_3

MS peptide 19 RoCRoDPD: DGGNLIGK

CP19: TAKGMWTKMVKDGGNLIGKISLLDPACPHHLERHYVLLLQL

refProt | altProt

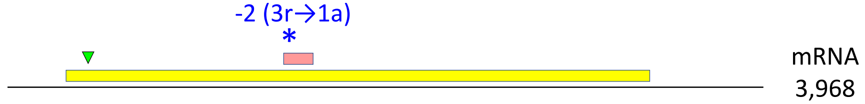

CP20: MtrunA17_Chr1g0190571_2F_62-262_201_MtrunA17_Chr1g0190571_1F_1-2274_2274_+2_iteration_10

MS peptide 20 Se: LVIFKLDLHGSK

CP20: MQIAANGKEFNATTKLVIFKLDLHGSKTRYLRGKINPLIT

altProt | refProt

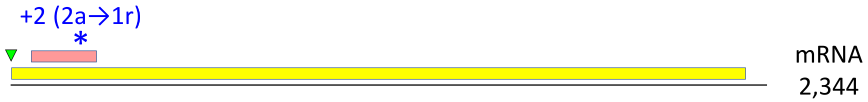

CP21: MtrunA17_Chr1g0191411_1F_1660-1869_210_MtrunA17_Chr1g0191411_2F_1601-1732_132_+2_iteration_22

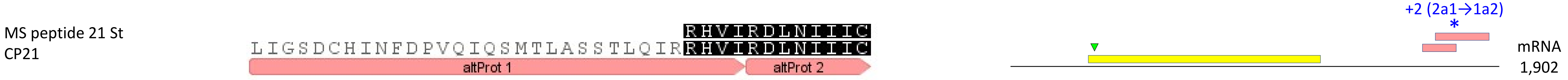

CP22: MtrunA17_Chr1g0198091_3F_255-443_189_MtrunA17_Chr1g0198091_1F_379-507_129_+1_iteration_2

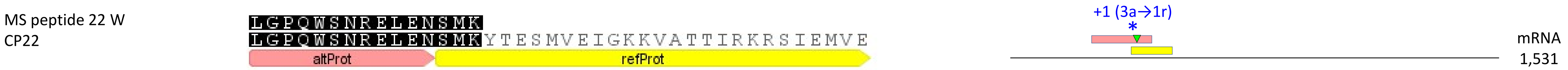

CP23: MtrunA17_Chr1g0200071_3F_828-1001_174_MtrunA17_Chr1g0200071_2F_218-1567_1350_+2_iteration_1

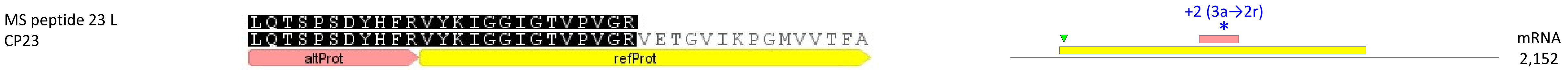

CP24: MtrunA17_Chr1g0200071_3F_828-1001_174_MtrunA17_Chr1g0200071_2F_218-1567_1350_-2_iteration_11

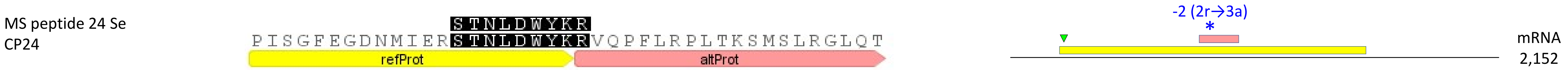

CP25: MtrunA17_Chr1g0202001_2F_149-421_273_MtrunA17_Chr1g0202001_1F_1-2331_2331_-1_iteration_0

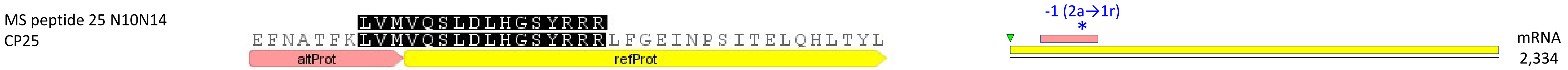

CP26: MtrunA17_Chr1g0205601_3F_321-395_75_MtrunA17_Chr1g0205601_1F_13-369_357_-1_iteration_17

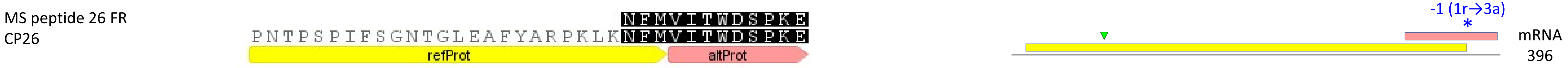

CP27: MtrunA17_Chr1g0207811_1F_1462-1824_363_MtrunA17_Chr1g0207811_3F_207-1913_1707_+1_iteration_19

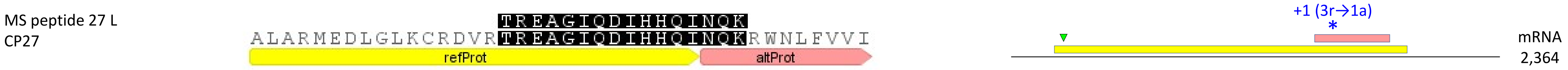

CP28: MtrunA17_Chr1g0207921_2F_260-391_132_MtrunA17_Chr1g0207921_1F_1-597_597_+1_iteration_6

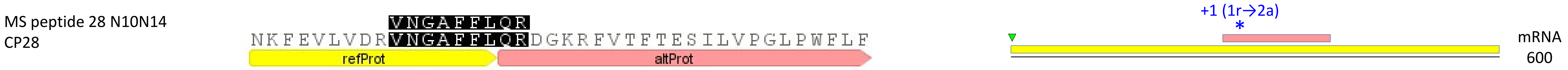

CP29: MtrunA17_Chr1g0209791_2F_2-130_129_MtrunA17_Chr1g0209791_1F_1-288_288_+2_iteration_5

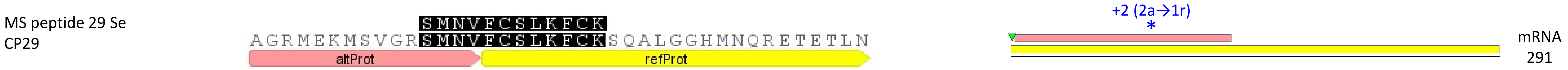

CP30: MtrunA17_Chr1g0210521_2F_782-928_147_MtrunA17_Chr1g0210521_3F_192-917_726_-1_iteration_23

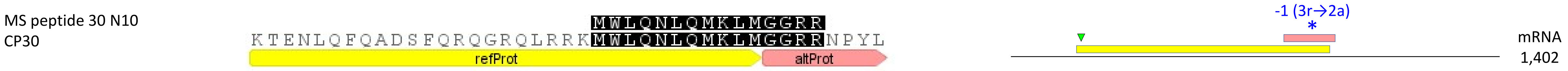

CP31: MtrunA17_Chr1g0212961_2F_617-703_87_MtrunA17_Chr1g0212961_3F_63-1136_1074_-2_iteration_2

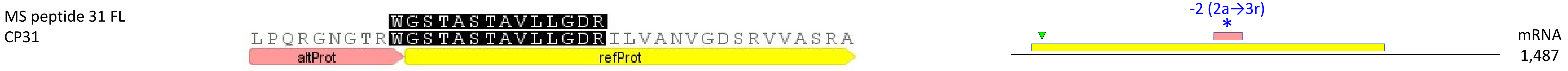

CP32: MtrunA17_Chr1g1004575_2F_299-379_81_MtrunA17_Chr1g1004575_3F_333-416_84_-2_iteration_11

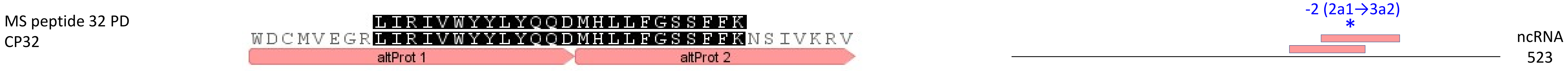

CP33: MtrunA17_Chr2g0283311_1F_766-849_84_MtrunA17_Chr2g0283311_2F_263-868_606_-2_iteration_14

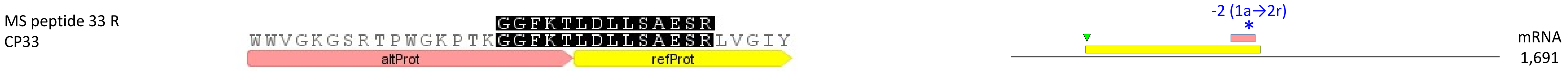

CP34: MtrunA17_Chr2g0285461_2F_1553-1795_243_MtrunA17_Chr2g0285461_1F_838-3573_2736_+2_iteration_0

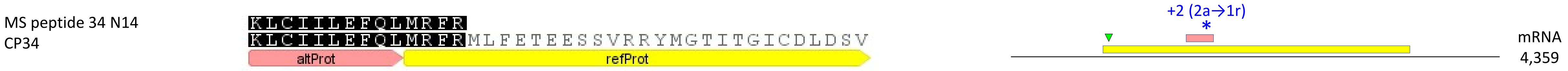

CP35: MtrunA17_Chr2g0292921_2F_1202-1453_252_MtrunA17_Chr2g0292921_1F_187-1545_1359_-2_iteration_6

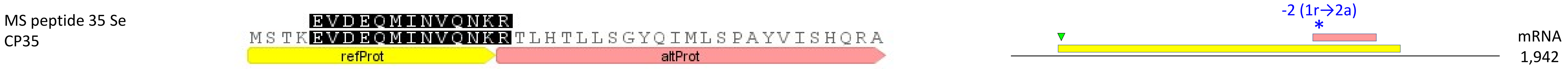

CP36: MtrunA17_Chr2g0298731_1F_3127-3255_129_MtrunA17_Chr2g0298731_3F_105-3674_3570_-1_iteration_7

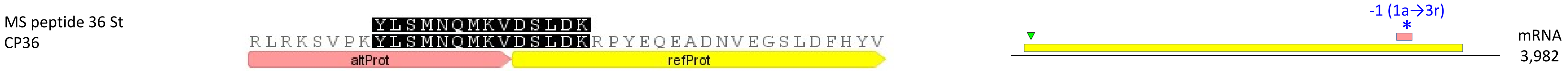

CP37: MtrunA17_Chr2g0299561_2F_3347-3535_189_MtrunA17_Chr2g0299561_1F_97-3969_3873_-1_iteration_17

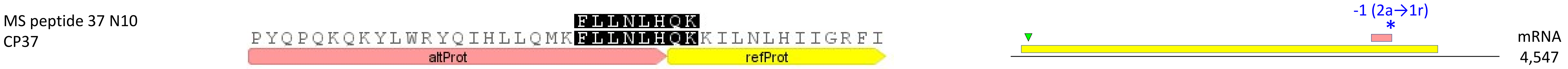

CP38: MtrunA17_Chr2g0304891_3F_627-725_99_MtrunA17_Chr2g0304891_1F_643-1512_870_-2_iteration_1

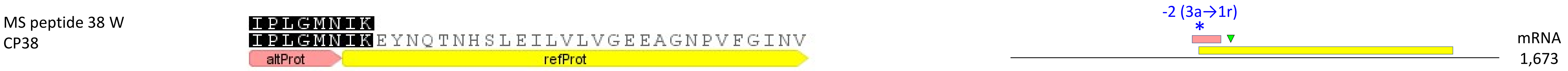

CP39: MtrunA17_Chr2g0305951_3F_3-113_111_MtrunA17_Chr2g0305951_1F_1-153_153_-1_iteration_14

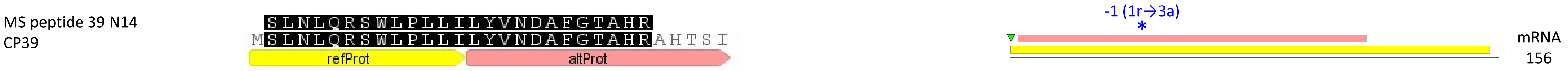

CP40: MtrunA17_Chr2g0309251_1F_1300-1383_84_MtrunA17_Chr2g0309251_3F_795-2144_1350_-1_iteration_5

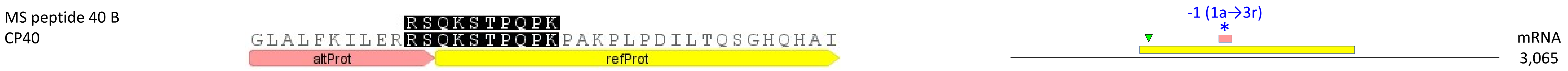

CP41: MtrunA17_Chr2g0310041_3F_3-95_93_MtrunA17_Chr2g0310041_1F_1-540_540_-2_iteration_20

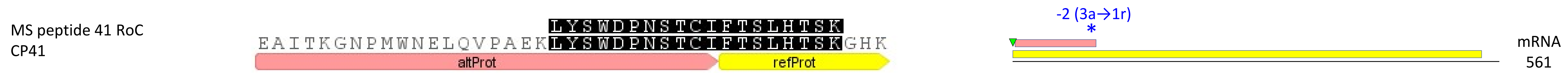

CP42: MtrunA17_Chr2g0312631_1F_2020-2094_75_MtrunA17_Chr2g0312631_3F_213-3398_3186_+2_iteration_11

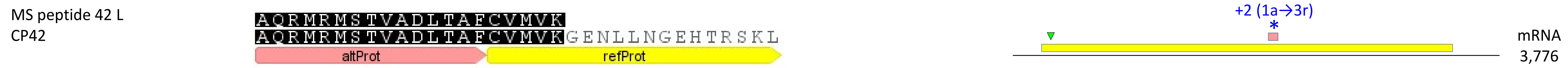

CP43: MtrunA17_Chr2g0316291_3F_1647-1745_99_MtrunA17_Chr2g0316291_2F_101-3394_3294_-2_iteration_1

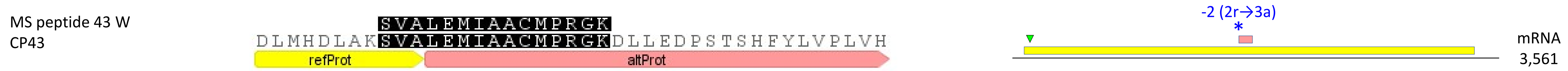

CP44: MtrunA17_Chr2g0326801_1F_2404-2493_90_MtrunA17_Chr2g0326801_3F_2394-2507_114_-2_iteration_19

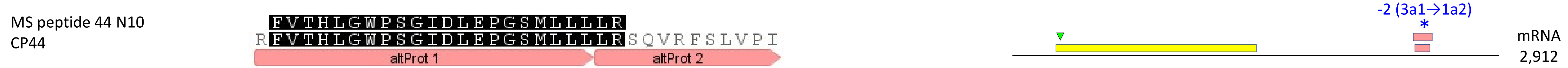

CP45: MtrunA17_Chr2g0328091_3F_87-245_159_MtrunA17_Chr2g0328091_2F_107-1243_1137_-1_iteration_5

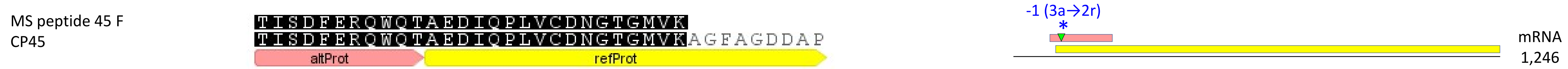

CP46: MtrunA17_Chr2g0329031_3F_1209-1379_171_MtrunA17_Chr2g0329031_2F_197-2440_2244_+2_iteration_20

MS peptide 46 B
CP46

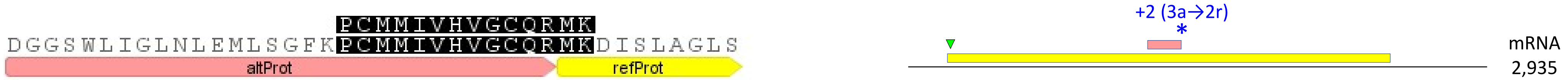

CP47: MtrunA17_Chr3g0079521_2F_1580-1747_168_MtrunA17_Chr3g0079521_1F_25-2292_2268_-1_iteration_10

MS peptide 47 St
CP47

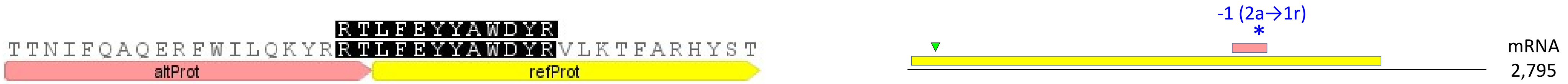

CP48: MtrunA17_Chr3g0083801_1F_76-174_99_MtrunA17_Chr3g0083801_3F_12-737_726_+1_iteration_3

MS peptide 48 N10
CP48

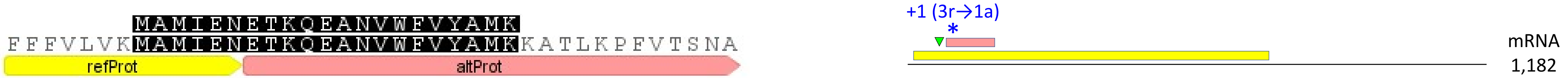

CP49: MtrunA17_Chr3g0091141_1F_976-1071_96_MtrunA17_Chr3g0091141_2F_170-1891_1722_-2_iteration_11

MS peptide 49 B
CP49

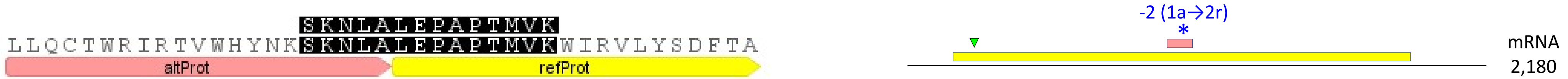

CP50: MtrunA17_Chr3g0091671_2F_14-130_117_MtrunA17_Chr3g0091671_1F_1-153_153_-2_iteration_14

MS peptide 50 PD
CP50

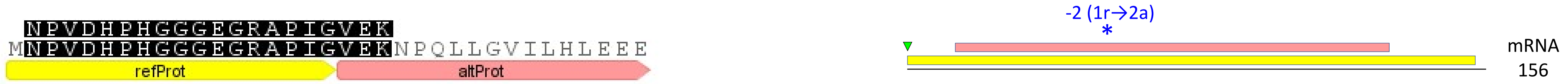

CP51: MtrunA17_Chr3g0096421_3F_54-173_120_MtrunA17_Chr3g0096421_1F_46-1086_1041_-1_iteration_7

MS peptide 51 Se QILIAGSREEFLWQEGHSAFASEEEADAESP
CP51 AVRQILIAGSREEFLWQEGHSAFASEEEADAESP
refProt altProt

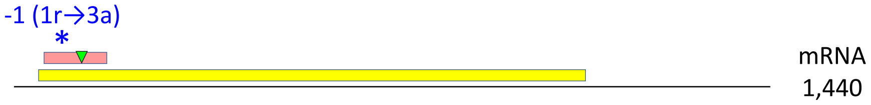

CP52: MtrunA17_Chr3g0100221_2F_803-922_120_MtrunA17_Chr3g0100221_1F_34-852_819_+1_iteration_6

MS peptide 52 R TNLVIVDDLWK
CP52 ERAQRLWLSLKEKKTNLVIVDDLWKKFNLTSIGIRIDSVN
refProt altProt

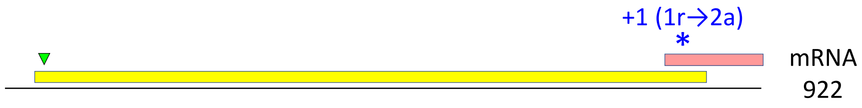

CP53: MtrunA17_Chr3g0102171_2F_236-379_144_MtrunA17_Chr3g0102171_1F_73-3423_3351_-2_iteration_5

MS peptide 53 Se KKILKPLWTTMSFK
CP53 NLTSHLYDALSRKKILKPLWTTMSFKKEMRSRQHSSKPLRS
refProt altProt

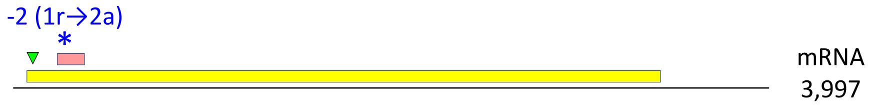

CP54: MtrunA17_Chr3g0105981_2F_1454-1618_165_MtrunA17_Chr3g0105981_3F_27-1478_1452_-1_iteration_5

MS peptide 54 N14BSe AESCDFGGLGLITTVDPSDDR
CP54 YYQEHDQKAESCDFGGLGLITTVDPSDDRCRFPIEIHTSNV
refProt altProt

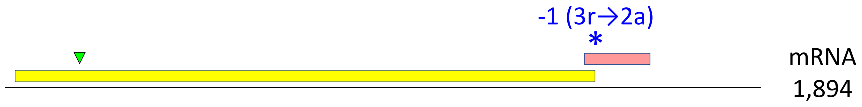

CP55: MtrunA17_Chr3g0110451_1F_538-711_174_MtrunA17_Chr3g0110451_2F_620-886_267_-2_iteration_4

MS peptide 55 PhC IYTAPCILSLTSFHIPR
CP55 IYTAPCILSLTSFHIPRAEPPNSQGRPWPTVTEQYLWKSNS
altProt 1 altProt 2

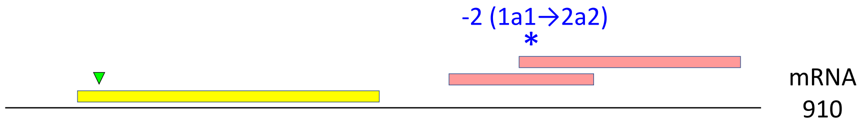

CP56: MtrunA17_Chr3g0113591_2F_1295-1465_171_MtrunA17_Chr3g0113591_1F_124-2748_2625_-2_iteration_19

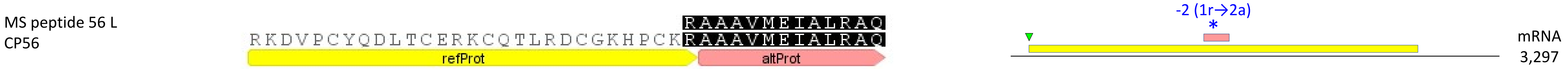

CP57: MtrunA17_Chr3g0124631_2F_1127-1249_123_MtrunA17_Chr3g0124631_1F_130-2304_2175_+2_iteration_19

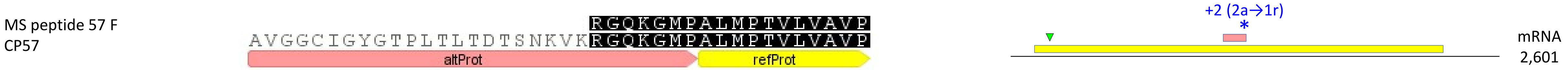

CP58: MtrunA17_Chr3g0127321_2F_1055-1303_249_MtrunA17_Chr3g0127321_1F_1-1329_1329_+2_iteration_18

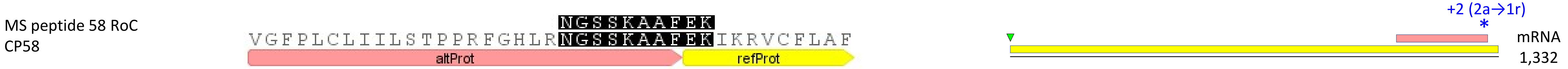

CP59: MtrunA17_Chr3g0130671_3F_1026-1190_165_MtrunA17_Chr3g0130671_1F_334-1179_846_-1_iteration_5

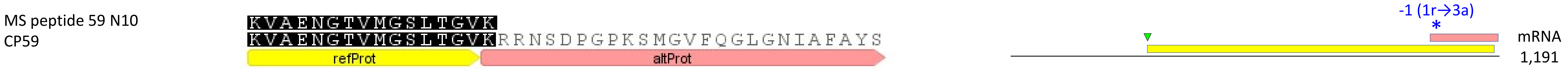

CP60: MtrunA17_Chr3g0135761_2F_734-958_225_MtrunA17_Chr3g0135761_1F_1-2115_2115_+2_iteration_0

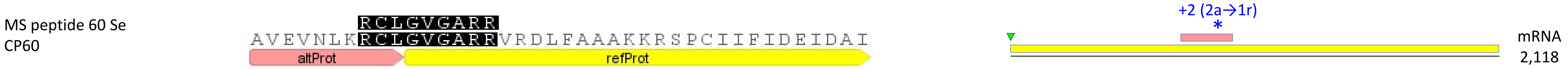

CP61: MtrunA17_Chr3g0137391_3F_915-1070_156_MtrunA17_Chr3g0137391_2F_23-1768_1746_-1_iteration_8

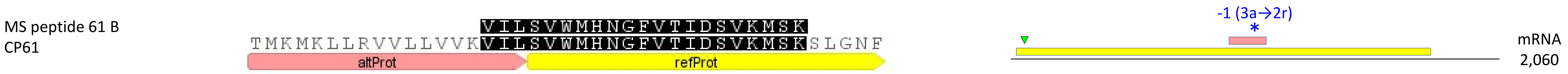

CP62: MtrunA17_Chr3g0141901_2F_1316-1585_270_MtrunA17_Chr3g0141901_1F_70-1371_1302_-2_iteration_17

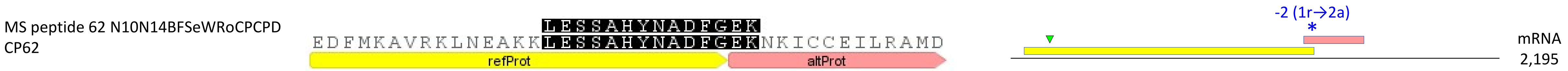

CP63: MtrunA17_Chr3g0144151_1F_1222-1311_90_MtrunA17_Chr3g0144151_3F_33-1415_1383_+2_iteration_3

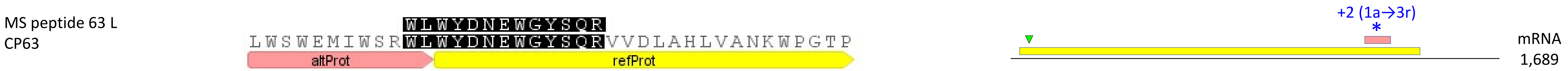

<u>CP64</u>: MtrunA17_Chr3g0144151_1F_1222-1311_90_MtrunA17_Chr3g0144151_3F_33-1415_1383_-1_iteration_5

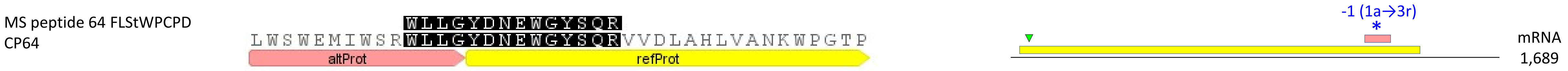

<u>CP65</u>: MtrunA17_Chr3g0144151_1F_1222-1311_90_MtrunA17_Chr3g0144151_3F_33-1415_1383_-1_iteration_6

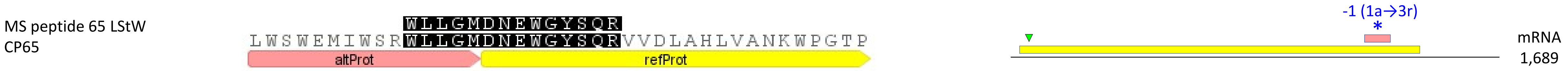

CP66: MtrunA17_Chr3g1011650_2F_26-340_315_MtrunA17_Chr3g1011650_3F_108-224_117_-2_iteration_2

MS peptide 66 RoC LSRKTVETFIDNTMSFK
CP66 LSRKTVETFIDNTMSFKKEMRSRQHSSKPLRSHMHLLSSSQ
altProt 1 | altProt 2

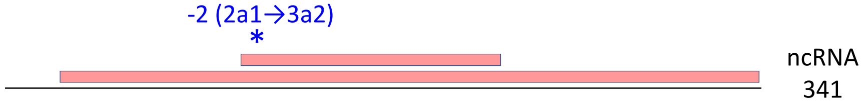

CP67: MtrunA17_Chr4g0000131_1F_1471-1644_174_MtrunA17_Chr4g0000131_3F_159-2459_2301_+1_iteration_7

MS peptide 67 N10 AVQALLAANLNDEIEPALPK
CP67 AVQALLAANLNDEIEPALPKDMISLRNLRLQRTLLEILRV
refProt | altProt

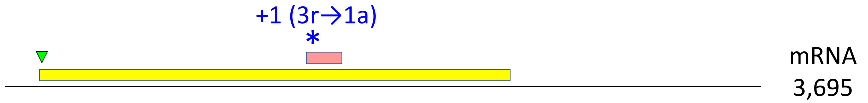

CP68: MtrunA17_Chr4g0000891_3F_3-473_471_MtrunA17_Chr4g0000891_1F_1-471_471_+2_iteration_6

MS peptide 68 N14 RLSQIFGTRIAFFHK
CP68 MKRLKRLSQIFGTRIAFFHKINNGCCTKLL
refProt | altProt

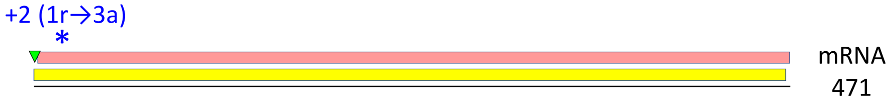

CP69: MtrunA17_Chr4g0004721_1F_634-813_180_MtrunA17_Chr4g0004721_3F_171-1157_987_+1_iteration_9

MS peptide 69 N10 TVALLGWEGLAQPLPR
CP69 GNWKHGDYKLTTKFSGKTVALLGWEGLAQPLPRELKALTV
refProt | altProt

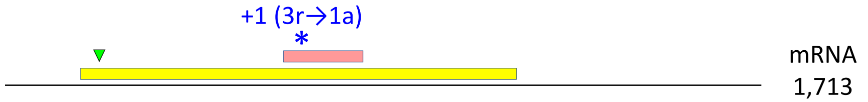

CP70: MtrunA17_Chr4g0014251_3F_45-242_198_MtrunA17_Chr4g0014251_1F_1-327_327_+1_iteration_4

MS peptide 70 N10 QPFNDHPAFFTSSPRCHRHR
CP70 QPFNDHPAFFTSSPRCHRHRLRLLEKLLILIETYLRNMNA
altProt | refProt

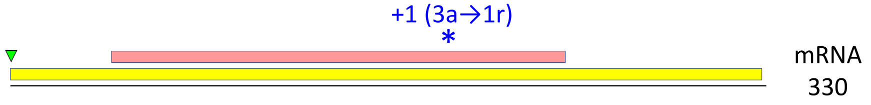

CP71: MtrunA17_Chr4g0022491_3F_762-923_162_MtrunA17_Chr4g0022491_2F_413-1177_765_+2_iteration_9

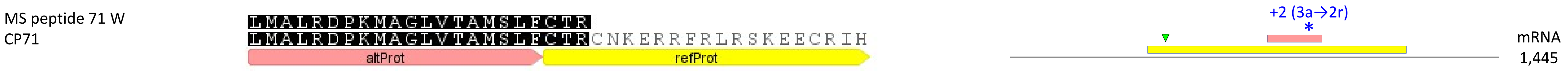

CP72: MtrunA17_Chr4g0023791_1F_1-84_84_MtrunA17_Chr4g0023791_2F_2-82_81_-1_iteration_5

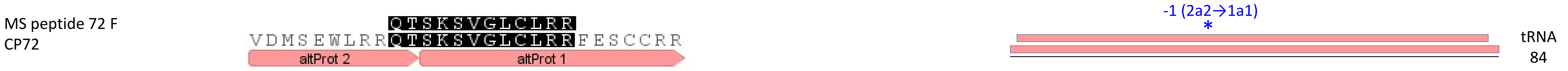

CP73: MtrunA17_Chr4g0034271_3F_15-344_330_MtrunA17_Chr4g0034271_1F_1-738_738_-1_iteration_7

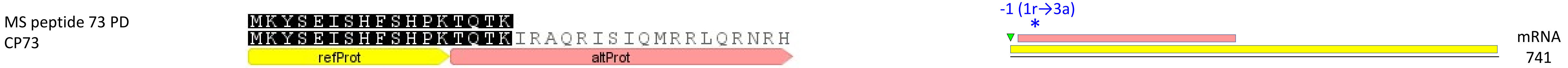

CP74: MtrunA17_Chr4g0034491_2F_1151-1708_558_MtrunA17_Chr4g0034491_1F_88-2415_2328_-2_iteration_1

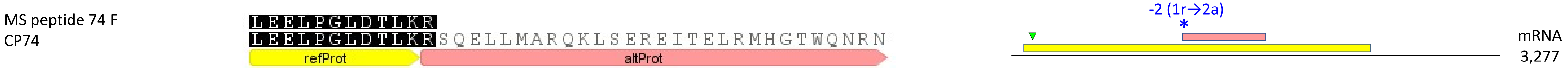

CP75: MtrunA17_Chr4g0037381_1F_880-1014_135_MtrunA17_Chr4g0037381_2F_422-1024_603_-1_iteration_16

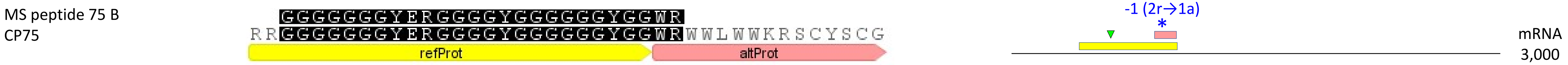

CP76: MtrunA17_Chr4g0040471_3F_2295-2474_180_MtrunA17_Chr4g0040471_1F_85-2868_2784_-1_iteration_14

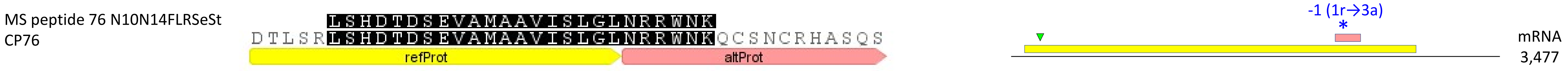

CP77: MtrunA17_Chr4g0055331_2F_62-127_66_MtrunA17_Chr4g0055331_1F_1-738_738_+2_iteration_9

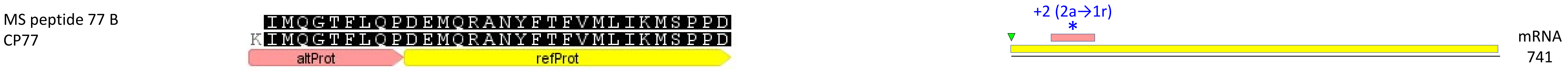

CP78: MtrunA17_Chr4g0059001_2F_83-277_195_MtrunA17_Chr4g0059001_1F_1-840_840_-2_iteration_11

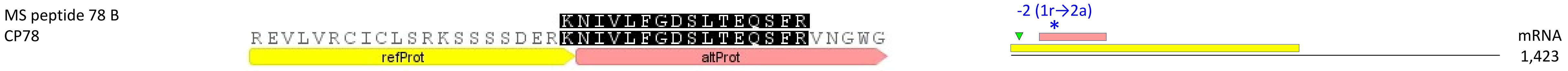

CP79: MtrunA17_Chr4g0063201_2F_446-535_90_MtrunA17_Chr4g0063201_1F_262-522_261_-2_iteration_18

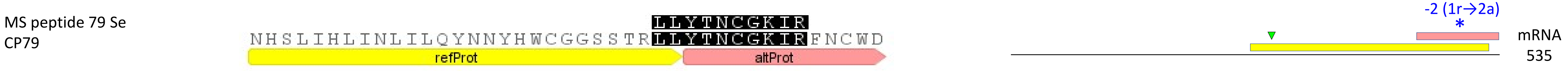

CP80: MtrunA17_Chr4g0070011_3F_1743-1832_90_MtrunA17_Chr4g0070011_1F_58-2481_2424_-1_iteration_16

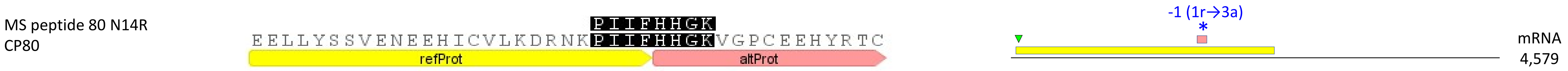

CP81: MtrunA17_Chr5g0393401_2F_1649-1765_117_MtrunA17_Chr5g0393401_3F_282-1781_1500_-2_iteration_10

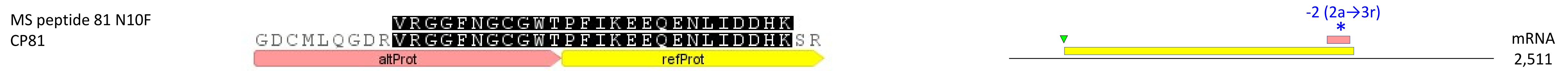

CP82: MtrunA17_Chr5g0400181_1F_859-1017_159_MtrunA17_Chr5g0400181_3F_312-2192_1881_+1_iteration_14

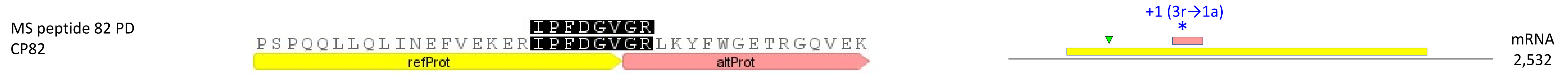

CP83: MtrunA17_Chr5g0405061_2F_182-412_231_MtrunA17_Chr5g0405061_3F_228-2174_1947_-2_iteration_20

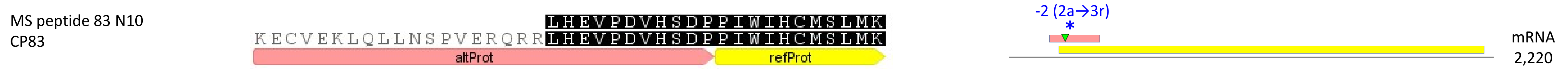

CP84: MtrunA17_Chr5g0408581_2F_2615-2860_246_MtrunA17_Chr5g0408581_1F_1-3168_3168_-2_iteration_12

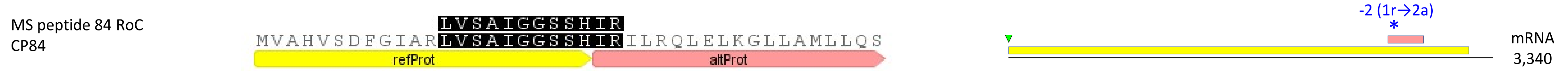

CP85: MtrunA17_Chr5g0415031_1F_931-1152_222_MtrunA17_Chr5g0415031_3F_114-4400_4287_+1_iteration_19

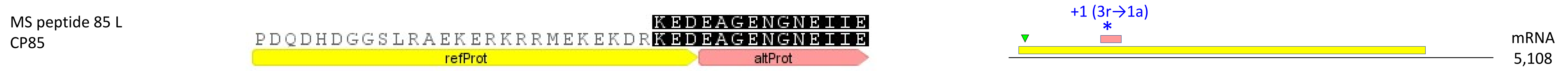

CP86: MtrunA17_Chr5g0415911_1F_3553-3948_396_MtrunA17_Chr5g0415911_3F_2832-3632_801_-2_iteration_6

MS peptide 86 N10
CP86

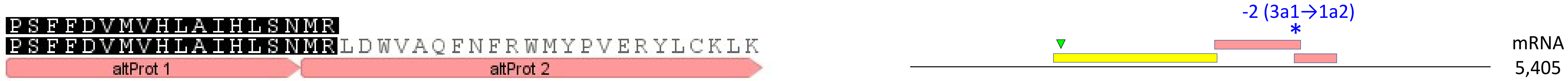

CP87: MtrunA17_Chr5g0421761_2F_1835-1918_84_MtrunA17_Chr5g0421761_3F_1818-1925_108_-1_iteration_4

MS peptide 87 Se
CP87

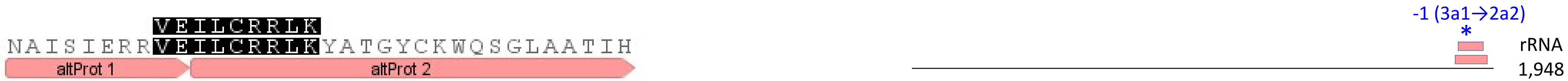

CP88: MtrunA17_Chr5g0422291_2F_3056-3259_204_MtrunA17_Chr5g0422291_3F_3120-3197_78_-1_iteration_5

MS peptide 88 B
CP88

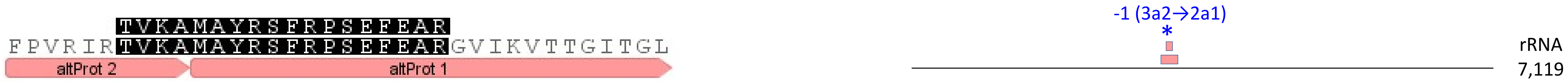

CP89: MtrunA17_Chr5g0422291_2F_4808-5617_810_MtrunA17_Chr5g0422291_1F_4921-5037_117_-1_iteration_29

MS peptide 89 PhC
CP89

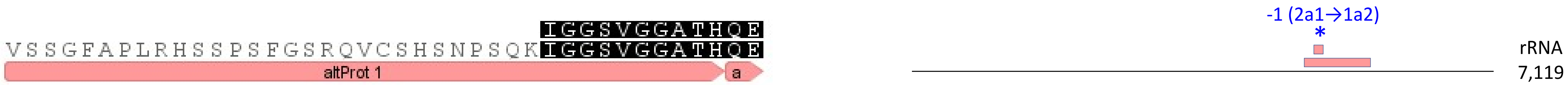

CP90: MtrunA17_Chr5g0430341_2F_2501-2623_123_MtrunA17_Chr5g0430341_3F_2298-2531_234_+2_iteration_3

MS peptide 90 L
CP90

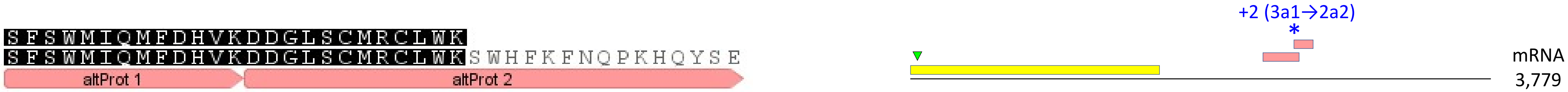

CP91: MtrunA17_Chr5g0430341_3F_1059-1172_114_MtrunA17_Chr5g0430341_1F_1-1620_1620_-2_iteration_9

MS peptide 91 W
CP91

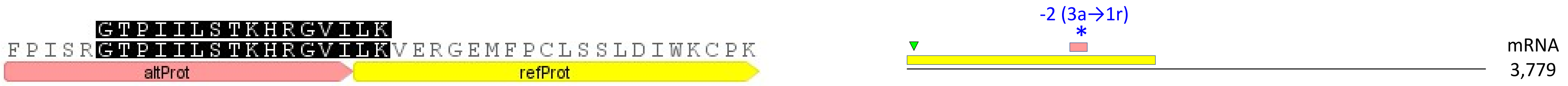

CP92: MtrunA17_Chr5g0431401_3F_1149-1388_240_MtrunA17_Chr5g0431401_2F_2-1354_1353_+1_iteration_18

MS peptide 92 RSt
CP92

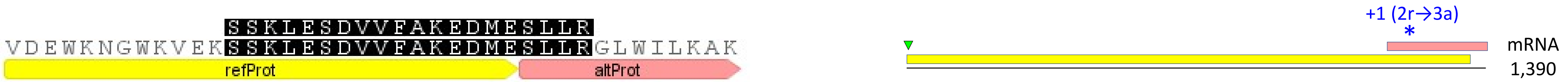

CP93: MtrunA17_Chr5g0435191_1F_883-1032_150_MtrunA17_Chr5g0435191_2F_677-979_303_-1_iteration_25

MS peptide 93 Se
CP93

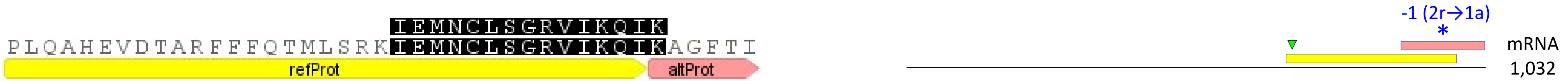

CP94: MtrunA17_Chr5g0444231_2F_758-967_210_MtrunA17_Chr5g0444231_3F_3-902_900_+2_iteration_11

MS peptide 94 F
CP94

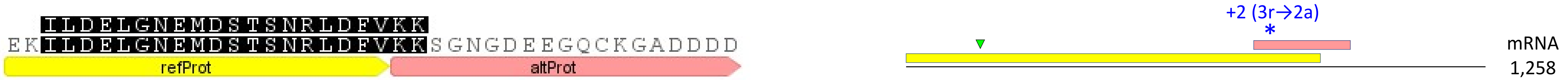

CP95: MtrunA17_Chr6g0451601_2F_434-577_144_MtrunA17_Chr6g0451601_1F_91-543_453_-2_iteration_29

MS peptide 95 F
CP95

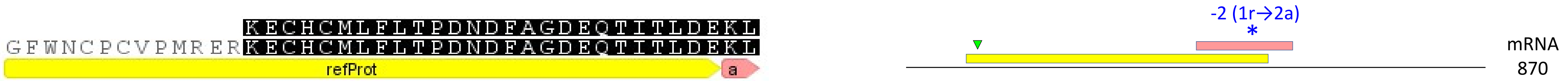

CP96: MtrunA17_Chr6g0452781_3F_4245-4397_153_MtrunA17_Chr6g0452781_1F_1-4458_4458_-1_iteration_15

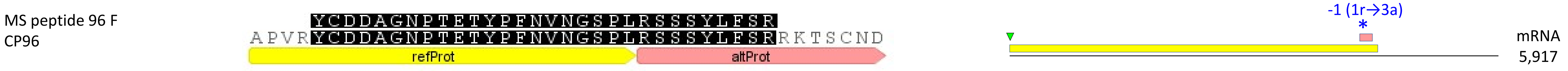

CP97: MtrunA17_Chr6g0457351_1F_406-636_231_MtrunA17_Chr6g0457351_3F_279-575_297_-2_iteration_22

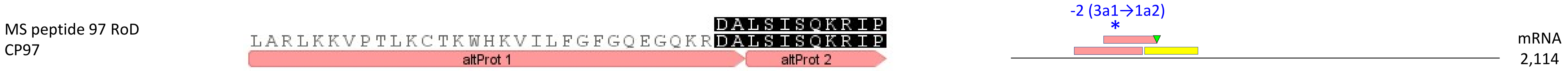

CP98: MtrunA17_Chr6g0457461_3F_438-596_159_MtrunA17_Chr6g0457461_2F_170-715_546_+2_iteration_6

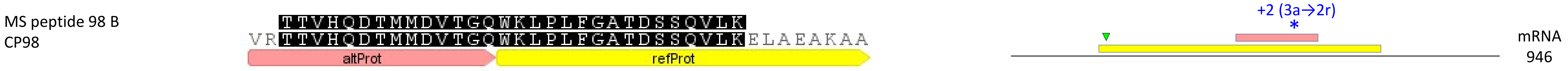

CP99: MtrunA17_Chr6g0457461_3F_438-596_159_MtrunA17_Chr6g0457461_2F_170-715_546_-2_iteration_17

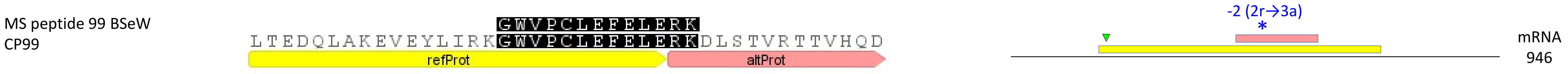

<u>CP100</u>: MtrunA17_Chr6g0457461_3F_438-596_159_MtrunA17_Chr6g0457461_2F_170-715_546_-2_iteration_18

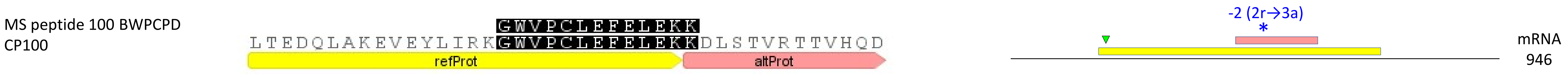

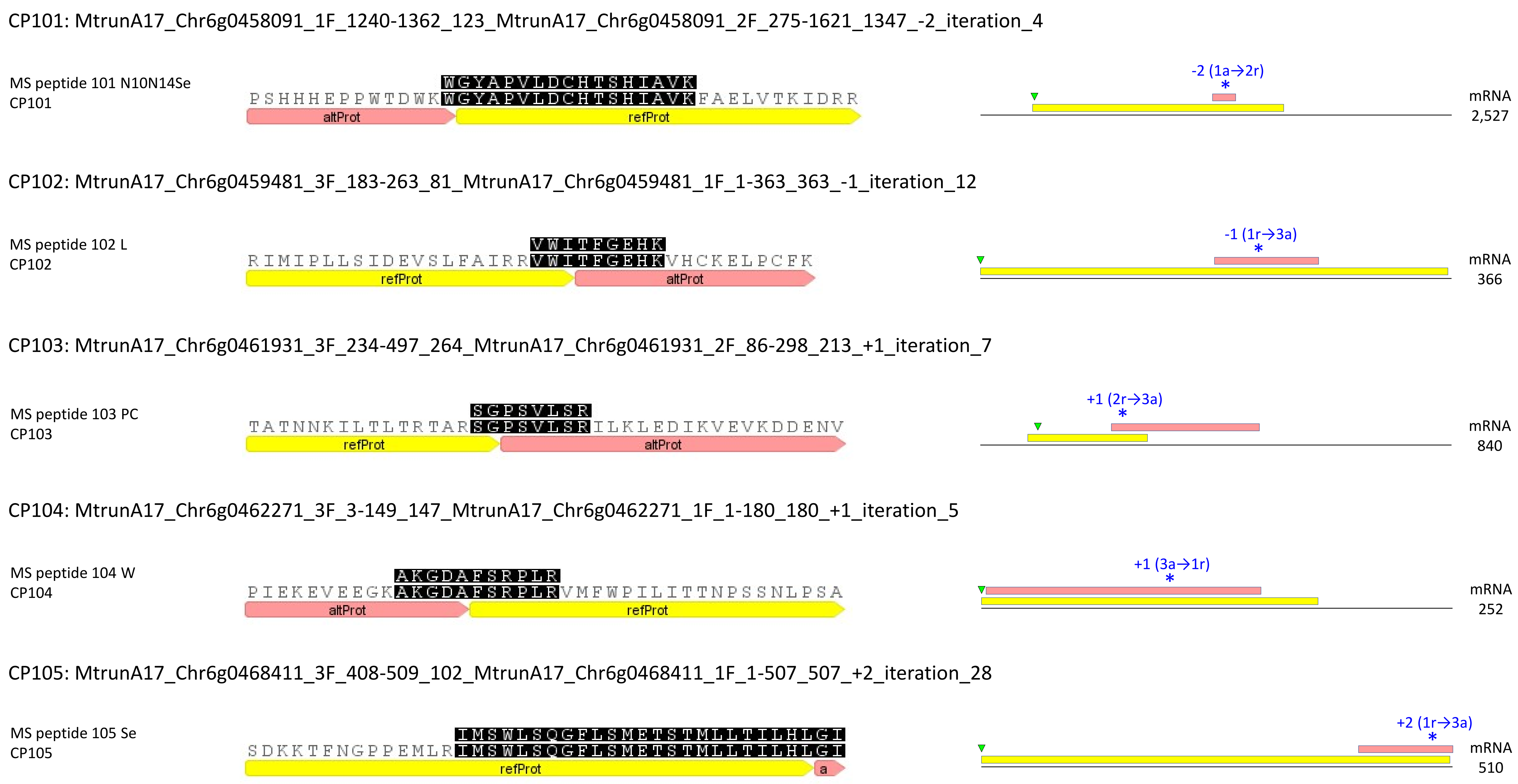
CP101: MtrunA17_Chr6g0458091_1F_1240-1362_123_MtrunA17_Chr6g0458091_2F_275-1621_1347_-2_iteration_4
MS peptide 101 N10N14Se
CP101
WGYAPVLDCHTSHIAVK
PSHHHEPPWTDWKWGYAPVLDCHTSHIAVKFAELVTKIDRR
altProt
refProt
-2 (1a→2r)
*
mRNA
2,527
CP102: MtrunA17_Chr6g0459481_3F_183-263_81_MtrunA17_Chr6g0459481_1F_1-363_363_-1_iteration_12
MS peptide 102 L
CP102
VWITFGEHK
RIMIPLLSIDEVSLFAIRRVWITFGEHKVHCKELPCFK
refProt
altProt
-1 (1r→3a)
*
mRNA
366
CP103: MtrunA17_Chr6g0461931_3F_234-497_264_MtrunA17_Chr6g0461931_2F_86-298_213_+1_iteration_7
MS peptide 103 PC
CP103
SGPSVLSR
TATNNKILTLTRTARSGPSVLSRILKLEDIKVEVKDDENV
refProt
altProt
+1 (2r→3a)
*
mRNA
840
CP104: MtrunA17_Chr6g0462271_3F_3-149_147_MtrunA17_Chr6g0462271_1F_1-180_180_+1_iteration_5
MS peptide 104 W
CP104
AKGDAFSRPLR
PIEKEVEEGKAKGDAFSRPLRVMFWPILITTNPSSNLPSA
altProt
refProt
+1 (3a→1r)
*
mRNA
252
CP105: MtrunA17_Chr6g0468411_3F_408-509_102_MtrunA17_Chr6g0468411_1F_1-507_507_+2_iteration_28
MS peptide 105 Se
CP105
IMSWLSQGFLSMETSTMLLTILHLGI
SDKKTFNGPPEMLRIMSWLSQGFLSMETSTMLLTILHLGI
refProt
a
+2 (1r→3a)
*
mRNA
510

CP106: MtrunA17_Chr6g0476751_3F_2274-2426_153_MtrunA17_Chr6g0476751_2F_359-2653_2295_+2_iteration_5

MS peptide 106 R
CP106

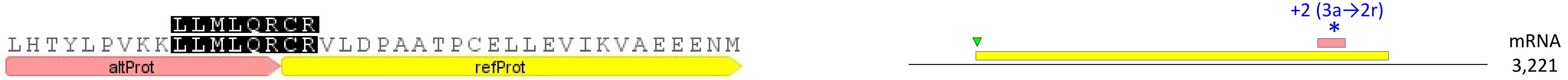

CP107: MtrunA17_Chr6g0479001_1F_3799-3981_183_MtrunA17_Chr6g0479001_2F_3701-3817_117_+2_iteration_5

MS peptide 107 PD
CP107

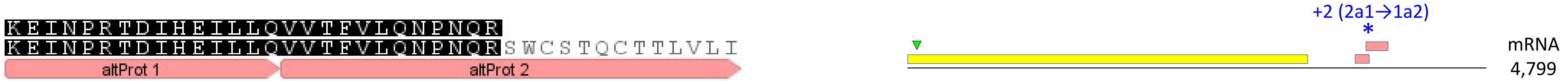

CP108: MtrunA17_Chr6g0485321_1F_1768-1881_114_MtrunA17_Chr6g0485321_3F_1674-1892_219_+2_iteration_13

MS peptide 108 R
CP108

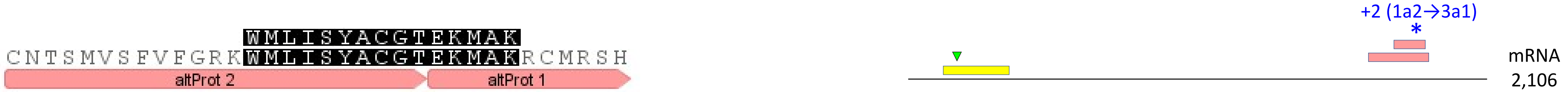

CP109: MtrunA17_Chr6g0486961_3F_1875-2120_246_MtrunA17_Chr6g0486961_1F_1-2943_2943_-2_iteration_12

MS peptide 109 Se
CP109

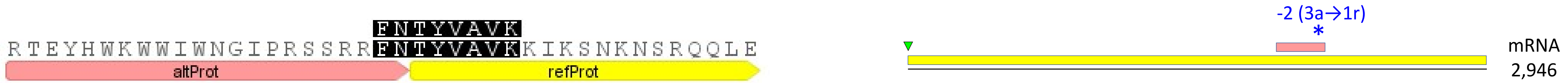

CP110: MtrunA17_Chr7g0214741_1F_340-462_123_MtrunA17_Chr7g0214741_3F_132-1478_1347_+1_iteration_8

MS peptide 110 Se
CP110

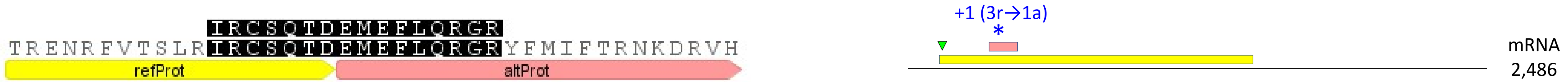

CP111: MtrunA17_Chr7g0214911_3F_183-368_186_MtrunA17_Chr7g0214911_2F_140-1999_1860_-2_iteration_6

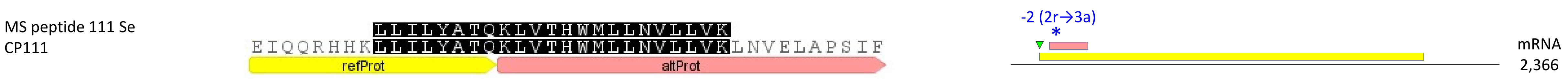

CP112: MtrunA17_Chr7g0219691_1F_1507-1602_96_MtrunA17_Chr7g0219691_3F_12-2699_2688_-2_iteration_2

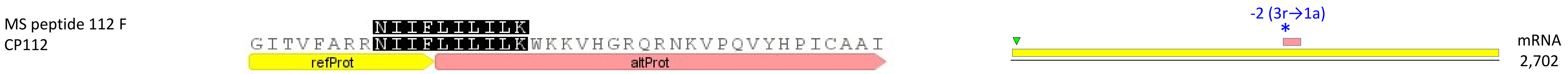

CP113: MtrunA17_Chr7g0221631_3F_3-125_123_MtrunA17_Chr7g0221631_1F_1-351_351_-1_iteration_13

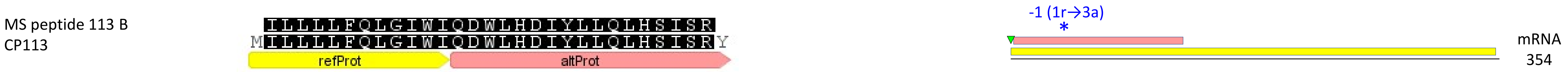

CP114: MtrunA17_Chr7g0229401_2F_2402-2755_354_MtrunA17_Chr7g0229401_3F_2268-2465_198_+2_iteration_1

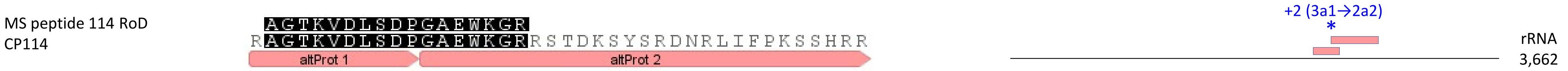

CP115: MtrunA17_Chr7g0230341_2F_2-307_306_MtrunA17_Chr7g0230341_1F_16-186_171_-1_iteration_3

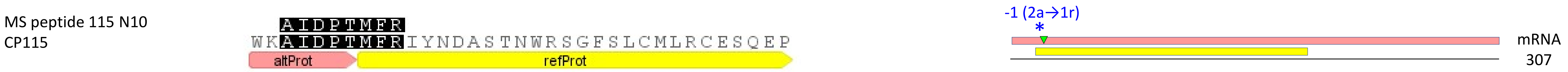

CP116: MtrunA17_Chr7g0232851_1F_502-684_183_MtrunA17_Chr7g0232851_2F_587-775_189_-2_iteration_22

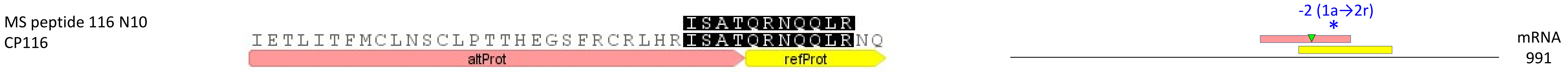

CP117: MtrunA17_Chr7g0237331_2F_1172-1372_201_MtrunA17_Chr7g0237331_1F_88-2508_2421_+2_iteration_18

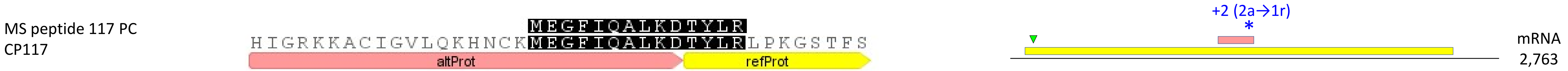

CP118: MtrunA17_Chr7g0251971_3F_3-161_159_MtrunA17_Chr7g0251971_1F_1-189_189_-1_iteration_4

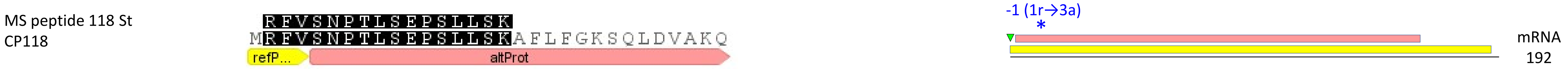

CP119: MtrunA17_Chr7g0259611_2F_185-292_108_MtrunA17_Chr7g0259611_3F_201-779_579_-2_iteration_6

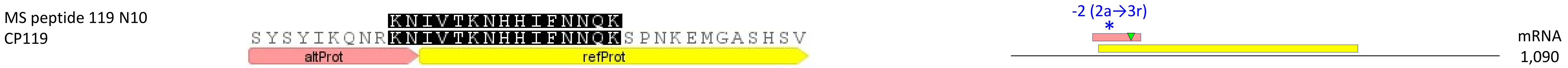

CP120: MtrunA17_Chr7g0262821_3F_1662-1847_186_MtrunA17_Chr7g0262821_2F_86-2131_2046_-1_iteration_13

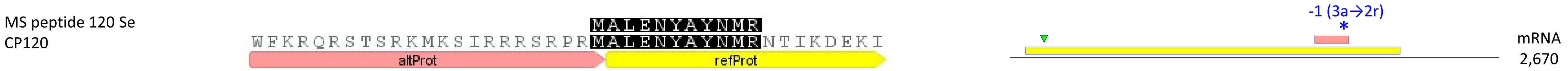

CP121: MtrunA17_Chr7g0270811_3F_1536-1718_183_MtrunA17_Chr7g0270811_2F_8-1711_1704_-2_iteration_10

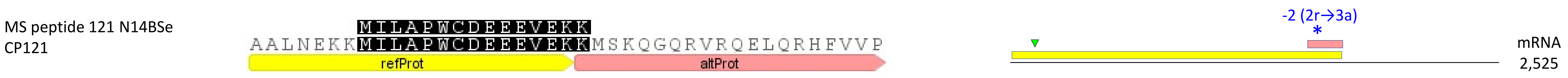

CP122: MtrunA17_Chr7g1034306_1F_502-681_180_MtrunA17_Chr7g1034306_2F_533-703_171_-2_iteration_17

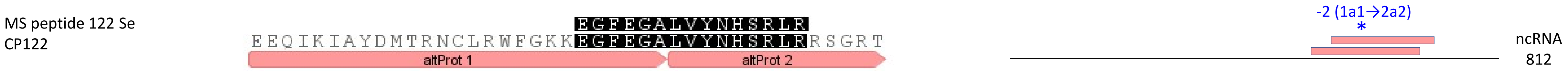

CP123: MtrunA17_Chr8g0338111_1F_436-534_99_MtrunA17_Chr8g0338111_2F_476-733_258_-2_iteration_11

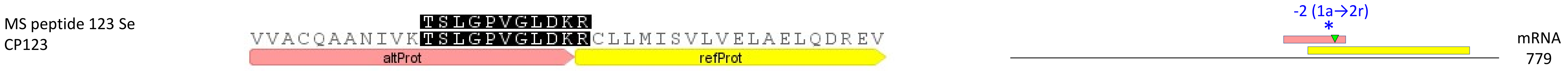

CP124: MtrunA17_Chr8g0338301_2F_1574-1681_108_MtrunA17_Chr8g0338301_1F_1-1680_1680_+1_iteration_17

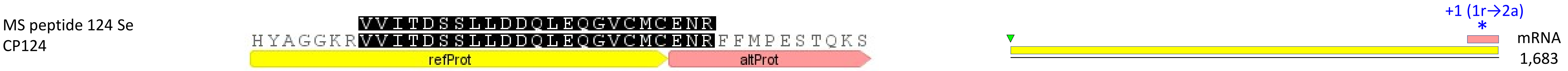

CP125: MtrunA17_Chr8g0339891_2F_998-1174_177_MtrunA17_Chr8g0339891_3F_426-1067_642_+2_iteration_11

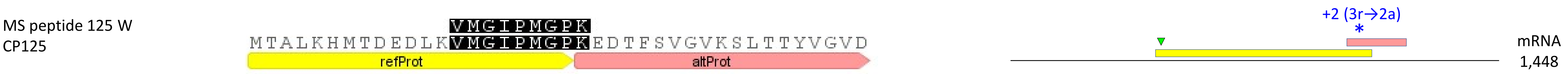

CP126: MtrunA17_Chr8g0342881_2F_656-892_237_MtrunA17_Chr8g0342881_1F_94-2667_2574_-2_iteration_1

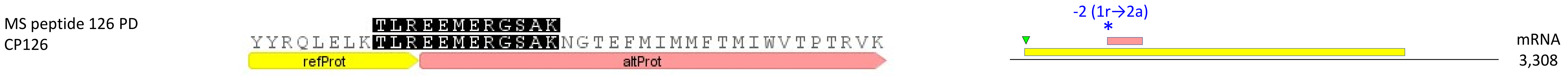

CP127: MtrunA17_Chr8g0345421_3F_2838-2963_126_MtrunA17_Chr8g0345421_2F_83-3610_3528_-2_iteration_6

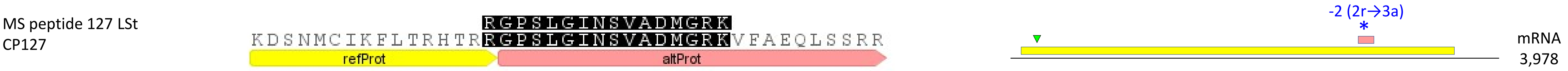

CP128: MtrunA17_Chr8g0353711_2F_110-283_174_MtrunA17_Chr8g0353711_1F_1-282_282_+1_iteration_21

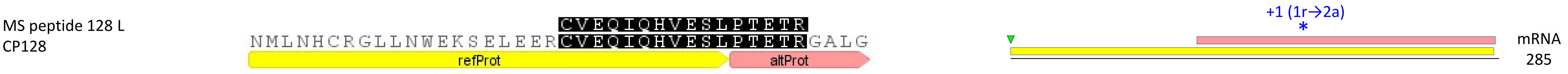

CP129: MtrunA17_Chr8g0355501_1F_1552-1761_210_MtrunA17_Chr8g0355501_3F_63-1898_1836_-2_iteration_6

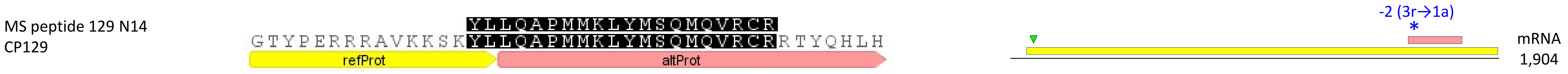

CP130: MtrunA17_Chr8g0356581_1F_1387-1464_78_MtrunA17_Chr8g0356581_2F_1394-1516_123_+1_iteration_5

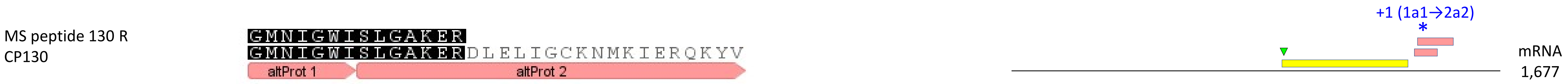

CP131: MtrunA17_Chr8g0365341_2F_773-1216_444_MtrunA17_Chr8g0365341_1F_169-2331_2163_+2_iteration_7

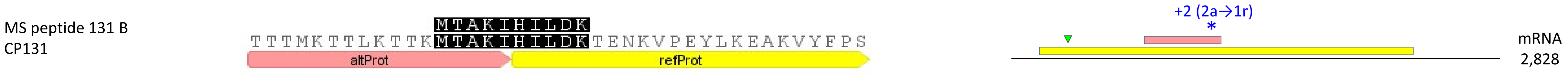

CP132: MtrunA17_Chr8g0368731_2F_1343-1540_198_MtrunA17_Chr8g0368731_1F_1-1875_1875_+1_iteration_0

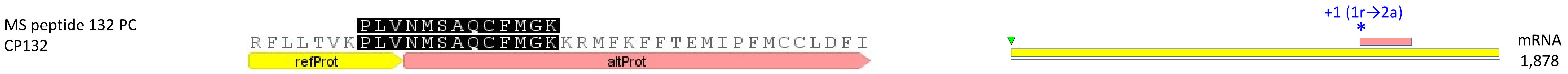

CP133: MtrunA17_Chr8g0368931_1F_889-1059_171_MtrunA17_Chr8g0368931_3F_3-1043_1041_-2_iteration_12

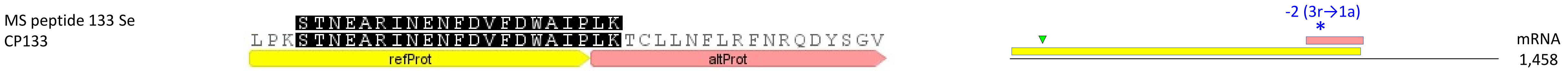

CP134: MtrunA17_Chr8g0371281_3F_696-851_156_MtrunA17_Chr8g0371281_2F_122-955_834_-2_iteration_4

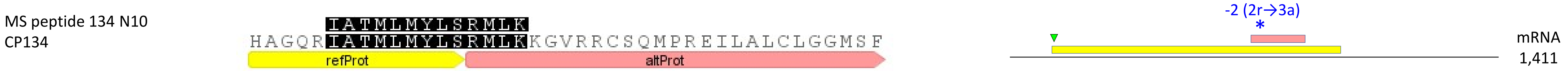

CP135: MtrunA17_Chr8g0371741_1F_691-888_198_MtrunA17_Chr8g0371741_3F_189-794_606_+1_iteration_16

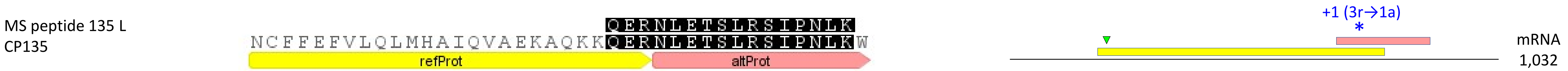

CP136: MtrunA17_Chr8g0373091_1F_457-702_246_MtrunA17_Chr8g0373091_3F_234-1106_873_+1_iteration_3

MS peptide 136 F
CP136

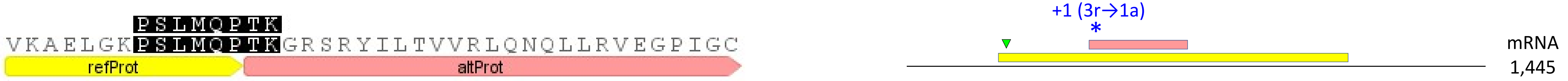

CP137: MtrunA17_Chr8g0376411_1F_433-582_150_MtrunA17_Chr8g0376411_3F_156-1463_1308_-1_iteration_6

MS peptide 137 BF
CP137

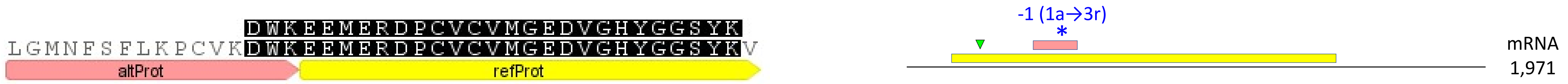

CP138: MtrunA17_Chr8g0377071_2F_1406-1693_288_MtrunA17_Chr8g0377071_1F_511-2715_2205_-1_iteration_17

MS peptide 138 L
CP138

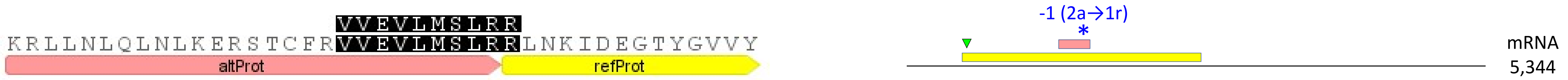

CP139: MtrunA17_Chr8g0385331_1F_1456-1719_264_MtrunA17_Chr8g0385331_3F_255-1670_1416_-2_iteration_8

MS peptide 139 B
CP139

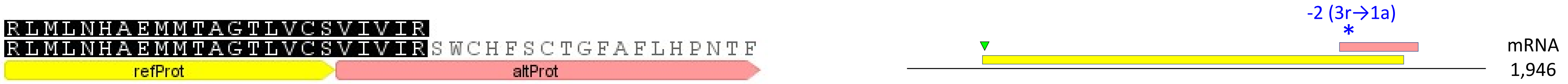

CP140: MtrunA17_Chr8g0392351_3F_96-272_177_MtrunA17_Chr8g0392351_1F_1-105_105_+2_iteration_4

MS peptide 140 PD
CP140

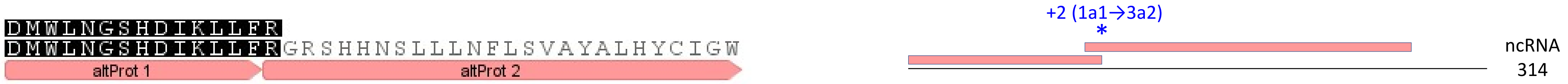

CP141: MtrunA17_CPg0492331_2F_584-874_291_MtrunA17_CPg0492331_3F_603-704_102_-1_iteration_19

MS peptide 141 LSt
CP141

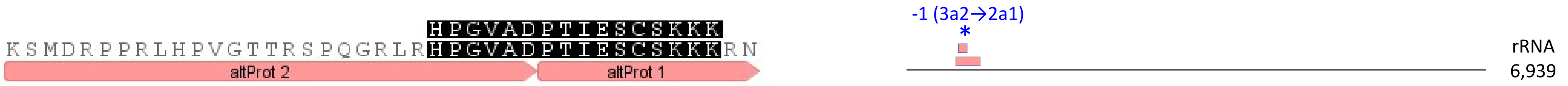

CP142: MtrunA17_CPg0492381_1F_631-750_120_MtrunA17_CPg0492381_2F_671-820_150_+1_iteration_1

MS peptide 142 N14
CP142

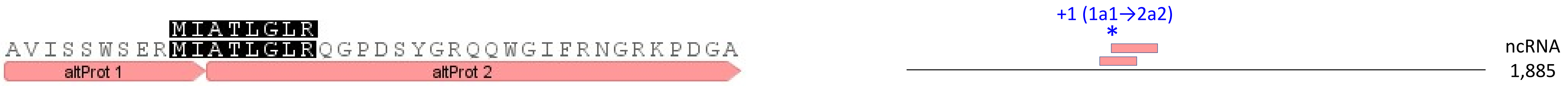

CP143: MtrunA17_CPg0492461_1F_3232-3426_195_MtrunA17_CPg0492461_3F_1413-7163_5751_+1_iteration_12

MS peptide 143 F
CP143

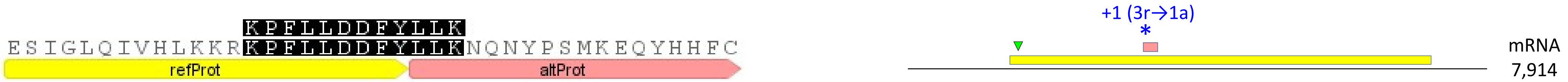

CP144: MtrunA17_CPg0492851_2F_209-328_120_MtrunA17_CPg0492851_3F_129-1157_1029_+1_iteration_6

MS peptide 144 FPD
CP144

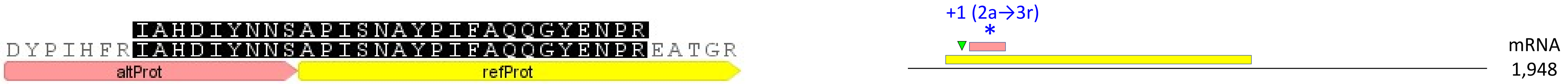

<u>CP145</u>: MtrunA17_CPg0492941_3F_825-947_123_MtrunA17_CPg0492941_1F_31-1572_1542_-1_iteration_2

MS peptide 145 N14BFLSeWPCPD
CP145

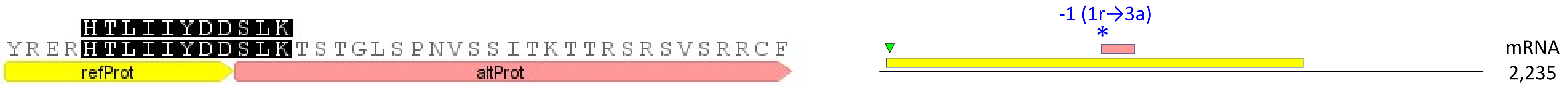

CP146: MtrunA17_CPg0493291_1F_733-804_72_MtrunA17_CPg0493291_2F_698-799_102_+2_iteration_18

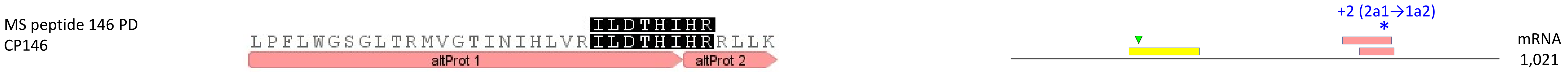

CP147: MtrunA17_CPg0493401_2F_1223-1450_228_MtrunA17_CPg0493401_3F_261-1778_1518_-2_iteration_7

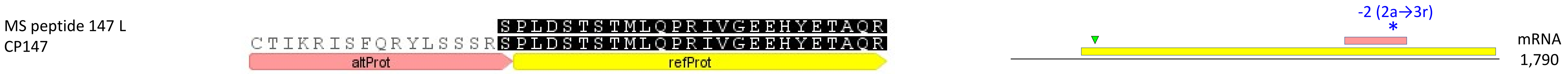

CP148: MtrunA17_MTg0490471_2F_1031-1147_117_MtrunA17_MTg0490471_1F_220-1740_1521_+1_iteration_7

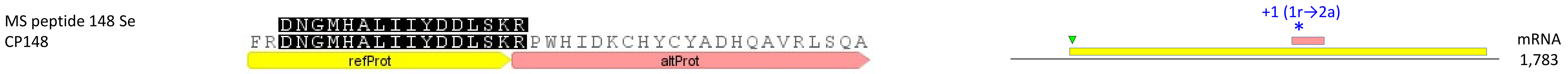

CP149: MtrunA17_MTg0490471_2F_422-538_117_MtrunA17_MTg0490471_1F_220-1740_1521_+1_iteration_16

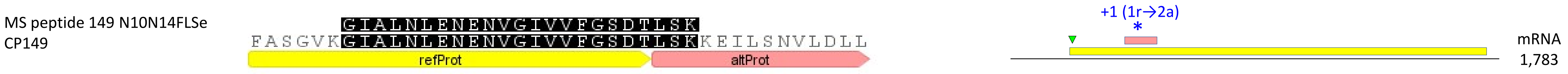

CP150: MtrunA17_MTg0490971_2F_1562-1699_138_MtrunA17_MTg0490971_3F_1542-1727_186_+1_iteration_12

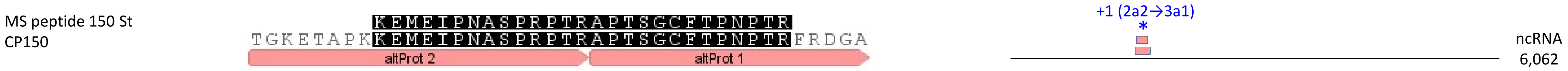

CP151: MtrunA17_MTg0490971_2F_476-628_153_MtrunA17_MTg0490971_1F_277-534_258_+1_iteration_0

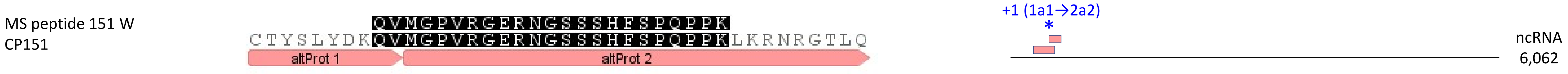

CP152: MtrunA17_MTg0491151_1F_4534-4659_126_MtrunA17_MTg0491151_2F_4511-4612_102_+2_iteration_15

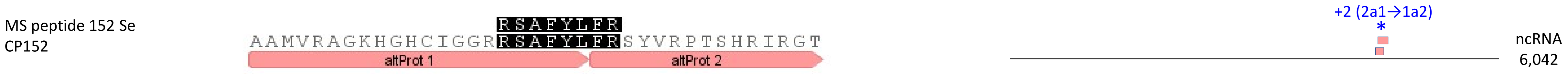

CP153: MtrunA17_MTg0491291_2F_1358-1447_90_MtrunA17_MTg0491291_3F_1329-1421_93_-1_iteration_18

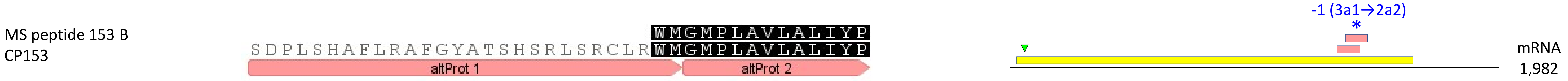

CP154: MtrunA17_MTg0491501_1F_661-825_165_MtrunA17_MTg0491501_3F_624-734_111_-2_iteration_6

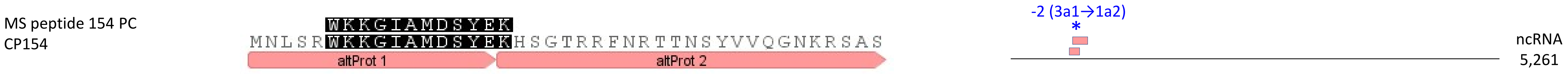

CP155: MtrunA17_MTg0491621_1F_1660-1836_177_MtrunA17_MTg0491621_3F_1377-1772_396_+1_iteration_16

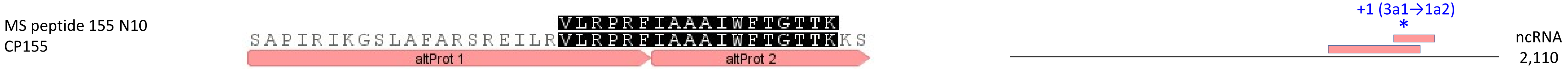

CP156: MtrunA17_MTg0491711_1F_2815-2910_96_MtrunA17_MTg0491711_3F_2664-2906_243_+1_iteration_24

MS peptide 156 L
CP156

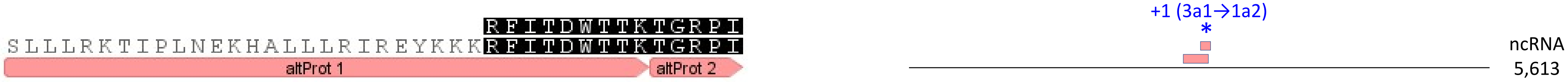

Supplementary Dataset S2. A graphical summary on alignments between 156 chimeric peptide models (CPs) and corresponding MS peptides (left) together with transcript models (right) that feature the transcript type, length in nucleotides, and relative positions of ORFs (to scale) involved in the production of CPs. Reference ORFs (refORFs) and reference proteins (refProts) are shown in yellow. Alternative ORFs (altORFs) and alternative proteins (altProts) are shown in pink. The first in-frame start codon (AUG) in each refORF is marked with a green triangle. If an altORF and a refORF are in the same frame, they are shown in the same level (e.g. CP7). If they are in different reading frames, they are shown in different levels (e.g. CP21). Codes of CPs modeled with MS-supported altProts are shown in a bold font. Codes of CPs with “confident” MS peptide detection in at least one sample are underlined. “Confident” is not the same as validated. For a short chimeric model sequence (mostly 40 amino acids or shorter in our dataset), it is very difficult to receive the status "confident" due to the nature of the MS method. Transcript models also show the positions and characteristics of programmed ribosomal frameshifting (PRF) events, which are mapped with an asterisk. The description of a PRF event should be interpreted as follows. For example, CP1, -1 (1a1→3a2): a minus 1 frameshift changes the translation from frame 1 to frame 3, which corresponds to the change from altORF1 to altORF2. An altORF1 in this study is defined as the one that starts earlier than an altORF2. Switches from altORF2 to altORF1 are also found in this dataset but are five times less frequent (e.g. CP108 and four other CPs). Throughout this study, unique numbers are assigned to chimeric models and their matching chimeric MS peptides. MS peptide identifiers also contain codes of biological samples in which they were detected. For example, the chimeric MS peptide of CP3 was identified in nodules of two developmental stages (10 and 14 days post inoculation). Thus, the identifier of MS peptide 3 ends with “N10N14”.
