# Supplementary material for: Discovery of diverse chimeric peptides in a eukaryotic proteome sets the stage for experimental validation of the mosaic translation hypothesis
Source: Comput Struct Biotechnol J. 2025 Sep 12;27:4048–64. doi: 10.1016/j.csbj.2025.09.019 (PMC12481079; doi:10.1016/j.csbj.2025.09.019)
Supplement: Supplementary file 1 — Supplementary material [file mmc1.zip › Supplementary Datasets/Supplementary Dataset S26 Expression profiles of non-overlapping repeat elements.pdf]

INRAE/CNRS Medicago Express | Expression Atlas Server

lipm-browsers.toulouse.inra.fr/pub/expressionAtlas/app/v3/aa_reference_dataset/MtrunA17_Chr2R0106940

MtExpress V3 | Core (20220901)

! Switch to another dataset using the left menu

lipme INRAE cnrs

TMM | METADATA | SYNONYMOUS | ANNOTATION | GENOME PORTAL | LEGOO

repeat_region: MtrunA17_Chr2R0106940;

Log2 TMM Normalisation using EdgeR (Core [20220901])

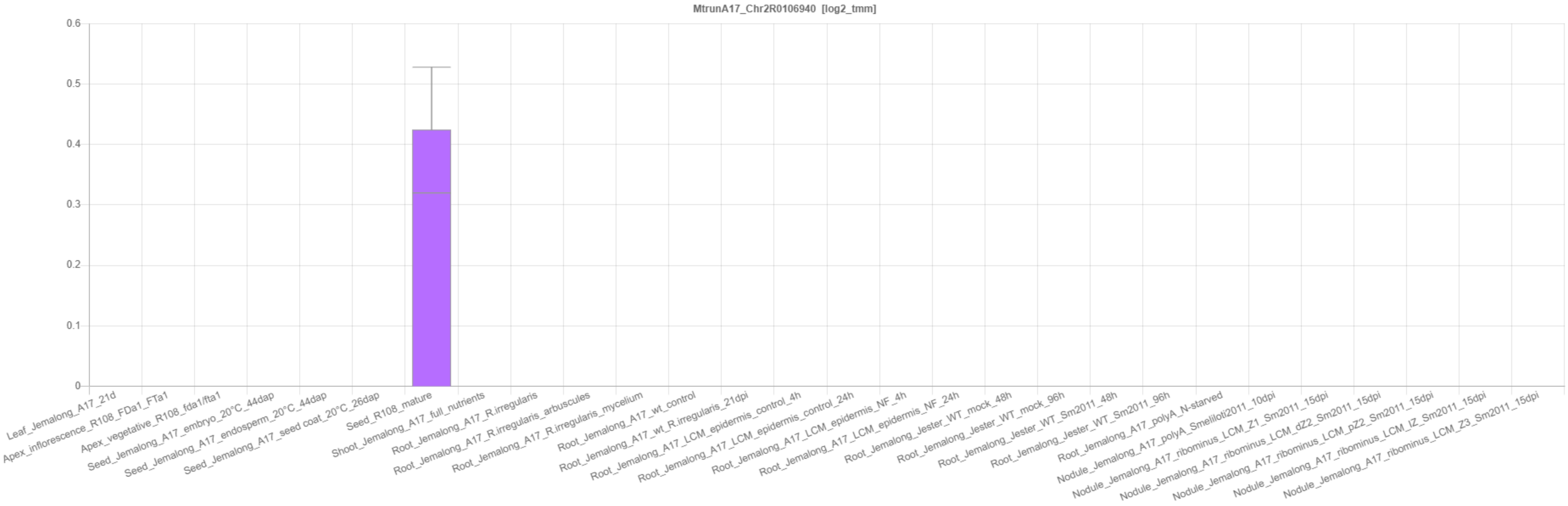

INRAE/CNRS Medicago Express

Expression Atlas Server

lipm-browsers.toulouse.inra.fr/pub/expressionAtlas/app/v3/aa_reference_dataset/MtrunA17_Chr3R0310480

MtExpress V3

Core (20220901)

! Switch to another dataset using the left menu

TMM METADATA SYNONYMOUS ANNOTATION GENOME PORTAL LEGOO

repeat_region: MtrunA17_Chr3R0310480;

Log2 TMM Normalisation using EdgeR (Core [20220901])

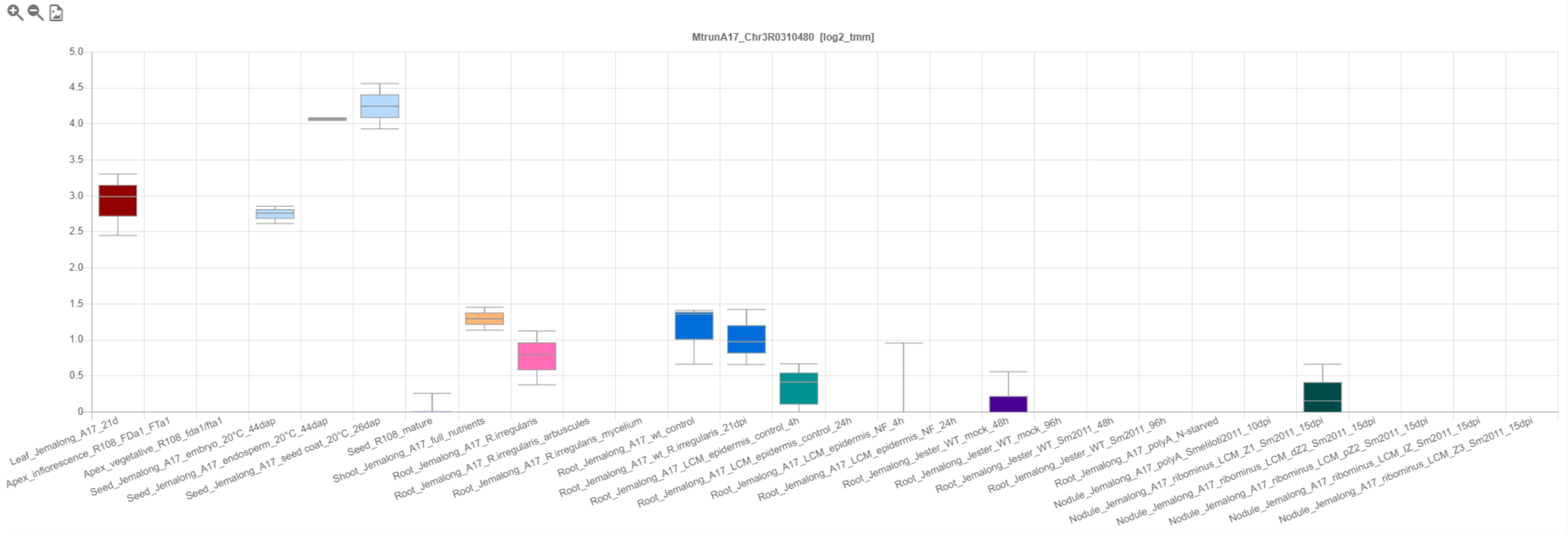

INRAE/CNRS Medicago Express | Expression Atlas Server

lipm-browsers.toulouse.inra.fr/pub/expressionAtlas/app/v3/aa_reference_dataset/MtrunA17_Chr4R0052500

MtExpress V3 | Core (20220901)

! Switch to another dataset using the left menu

TMM | METADATA | SYNONYMOUS | ANNOTATION | GENOME PORTAL | LEGOO

repeat_region: MtrunA17_Chr4R0052500;

Log2 TMM Normalisation using EdgeR (Core [20220901])

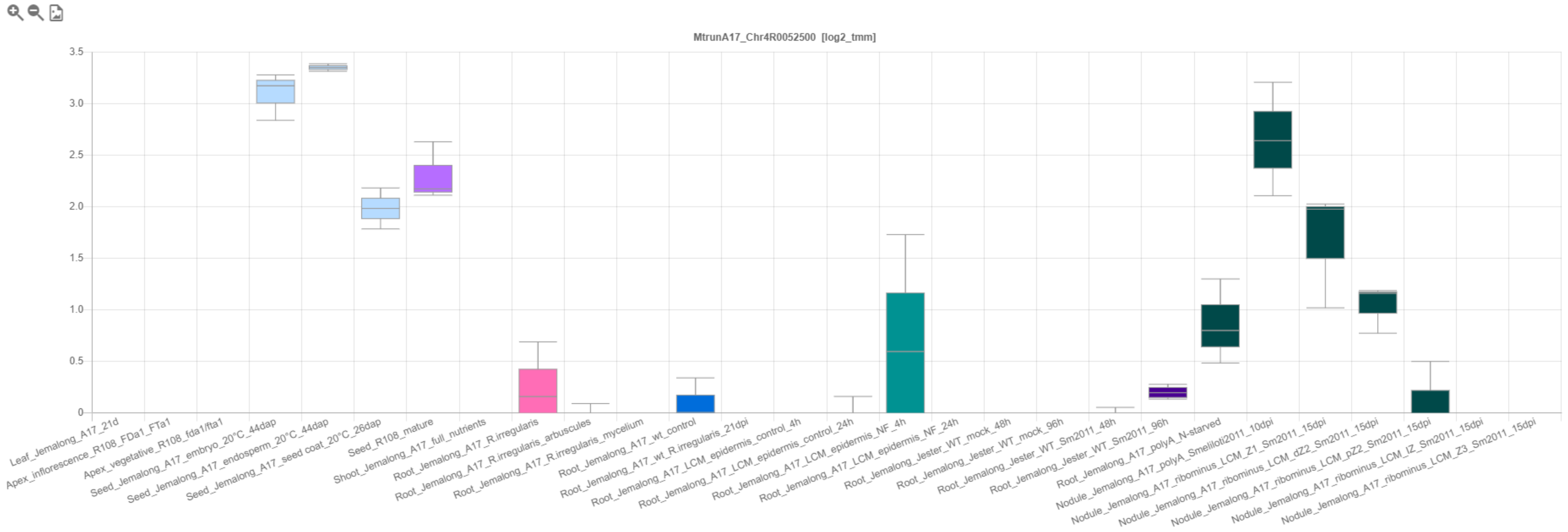

INRAE/CNRS Medicago Express
Expression Atlas Server

lipm-browsers.toulouse.inra.fr/pub/expressionAtlas/app/v3/aa_reference_dataset/MtrunA17_Chr1R0148110

MtExpress V3 | Core (20220901)

! Switch to another dataset using the left menu

TMM | METADATA | SYNONYMOUS | ANNOTATION | GENOME PORTAL | LEGOO

repeat_region: MtrunA17_Chr1R0148110;

Log2 TMM Normalisation using EdgeR (Core [20220901])

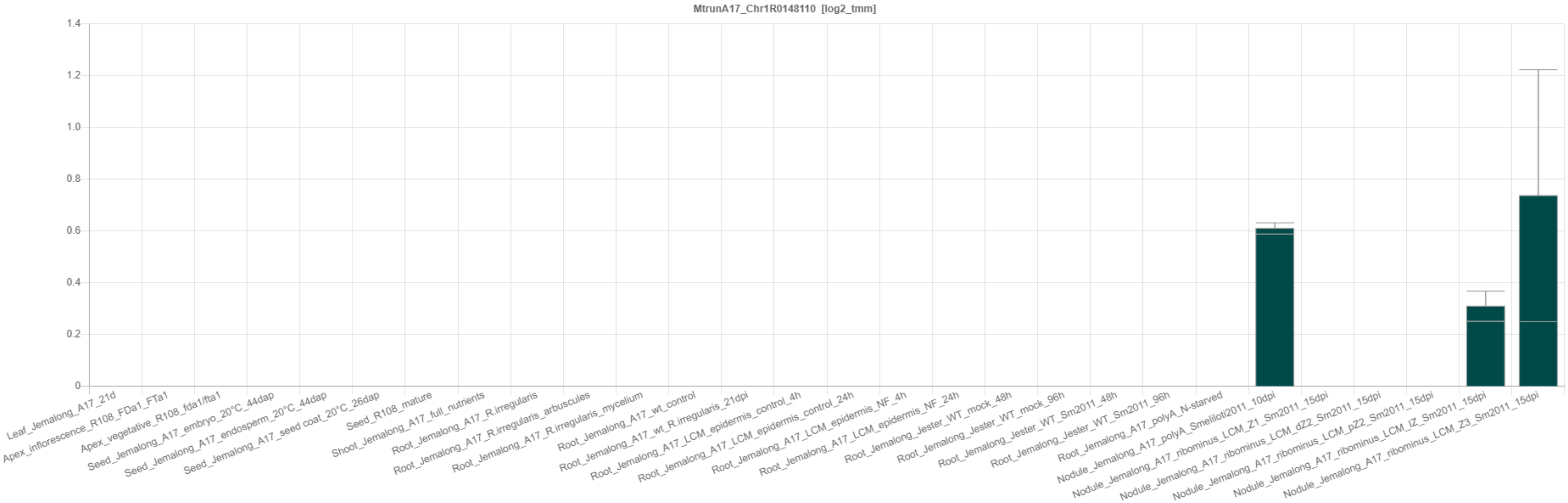

INRAE/CNRS Medicago Express | Expression Atlas Server

lipm-browsers.toulouse.inra.fr/pub/expressionAtlas/app/v3/aa_reference_dataset/MtrunA17_Chr1R0014150

MtExpress V3 | Core (20220901)

! Switch to another dataset using the left menu

TMM | METADATA | SYNONYMOUS | ANNOTATION | GENOME PORTAL | LEGOO

repeat_region: MtrunA17_Chr1R0014150;

Log2 TMM Normalisation using EdgeR (Core [20220901])

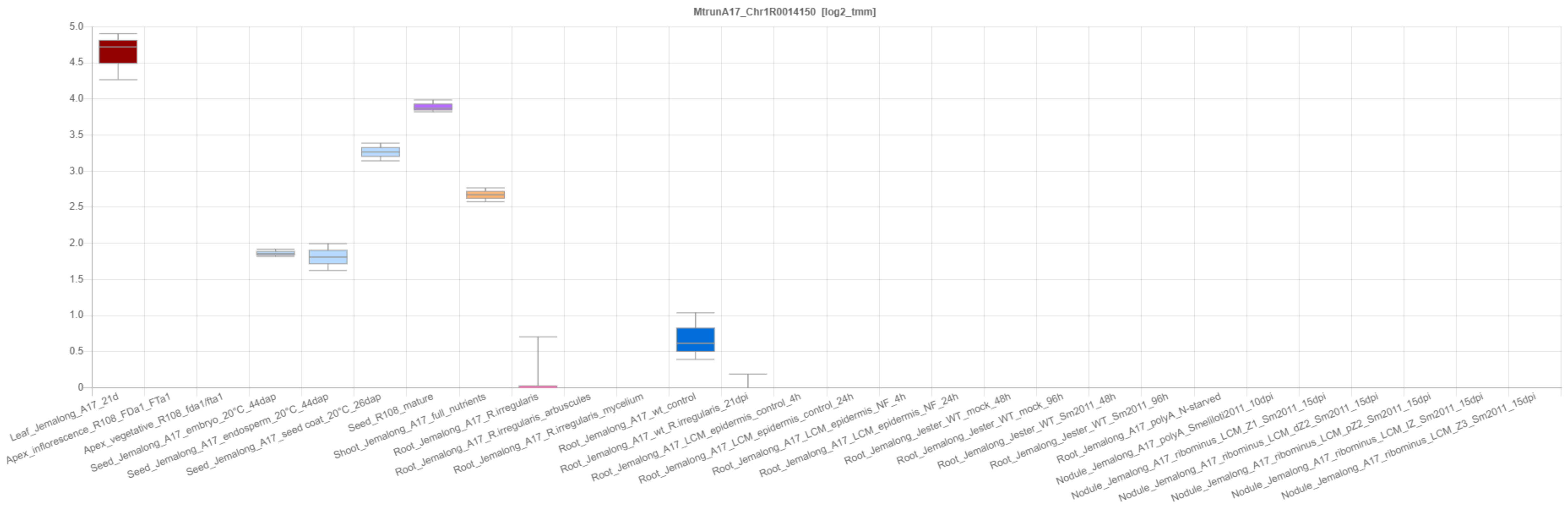

INRAE/CNRS Medicago Express | Expression Atlas Server

lipm-browsers.toulouse.inra.fr/pub/expressionAtlas/app/v3/aa_reference_dataset/MtrunA17_Chr7R0161930

MtExpress V3 | Core (20220901)

! Switch to another dataset using the left menu

TMM | METADATA | SYNONYMOUS | ANNOTATION | GENOME PORTAL | LEGOO

repeat_region: MtrunA17_Chr7R0161930;

Log2 TMM Normalisation using EdgeR (Core [20220901])

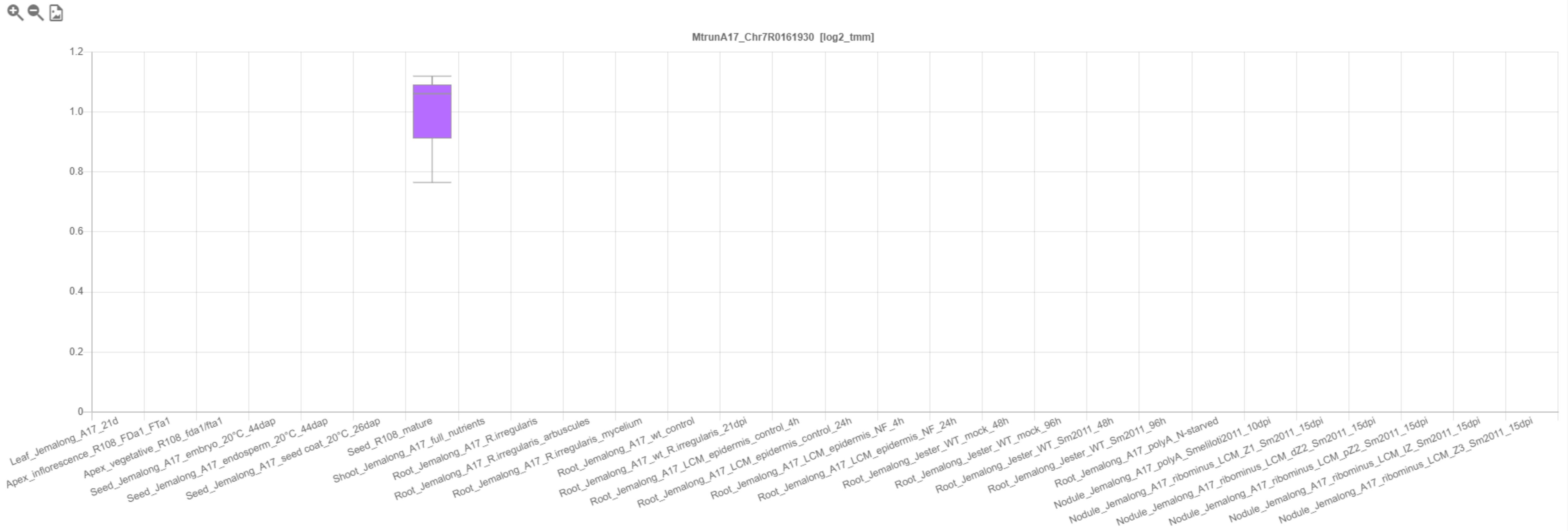

INRAE/CNRS Medicago Express
Expression Atlas Server

lipm-browsers.toulouse.inra.fr/pub/expressionAtlas/app/v3/aa_reference_dataset/MtrunA17_Chr3R0062580

MtExpress V3 | Core (20220901)

! Switch to another dataset using the left menu

TMM | METADATA | SYNONYMOUS | ANNOTATION | GENOME PORTAL | LEGOO

repeat_region: MtrunA17_Chr3R0062580;

Log2 TMM Normalisation using EdgeR (Core [20220901])

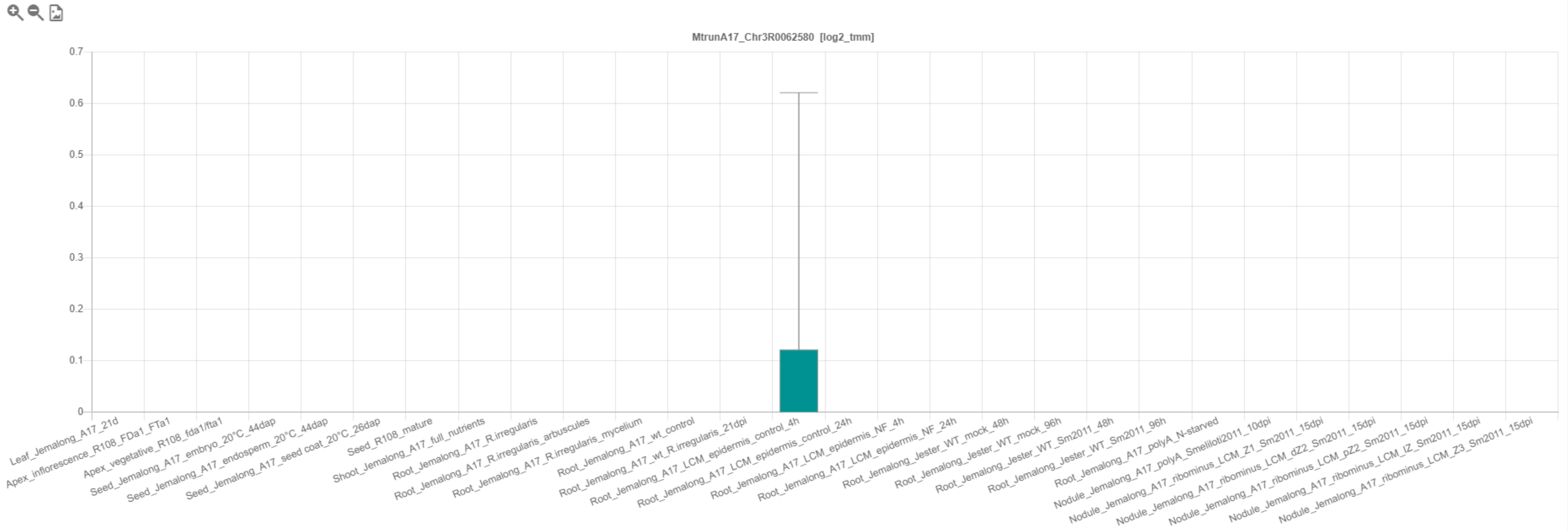

INRAE/CNRS Medicago Express

Expression Atlas Server

lipm-browsers.toulouse.inra.fr/pub/expressionAtlas/app/v3/aa_reference_dataset/MtrunA17_Chr0c04R0002520

MtExpress V3

Core (20220901)

! Switch to another dataset using the left menu

TMM METADATA SYNONYMOUS ANNOTATION GENOME PORTAL LEGOO

repeat_region: MtrunA17_Chr0c04R0002520;

Log2 TMM Normalisation using EdgeR (Core [20220901])

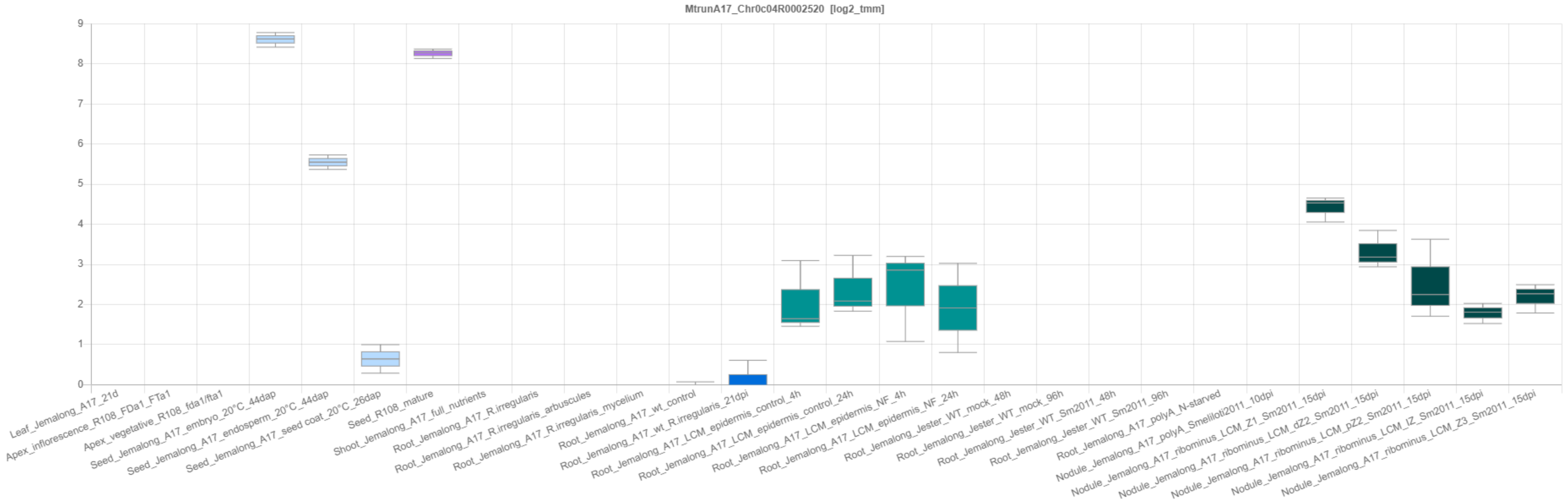

INRAE/CNRS Medicago Express | Expression Atlas Server

lipm-browsers.toulouse.inra.fr/pub/expressionAtlas/app/v3/aa_reference_dataset/MtrunA17_Chr3R0074220

MtExpress V3 | Core (20220901)

! Switch to another dataset using the left menu

lipme INRAE cnrs

TMM | METADATA | SYNONYMOUS | ANNOTATION | GENOME PORTAL | LEGOO

repeat_region: MtrunA17_Chr3R0074220;

Log2 TMM Normalisation using EdgeR (Core [20220901])

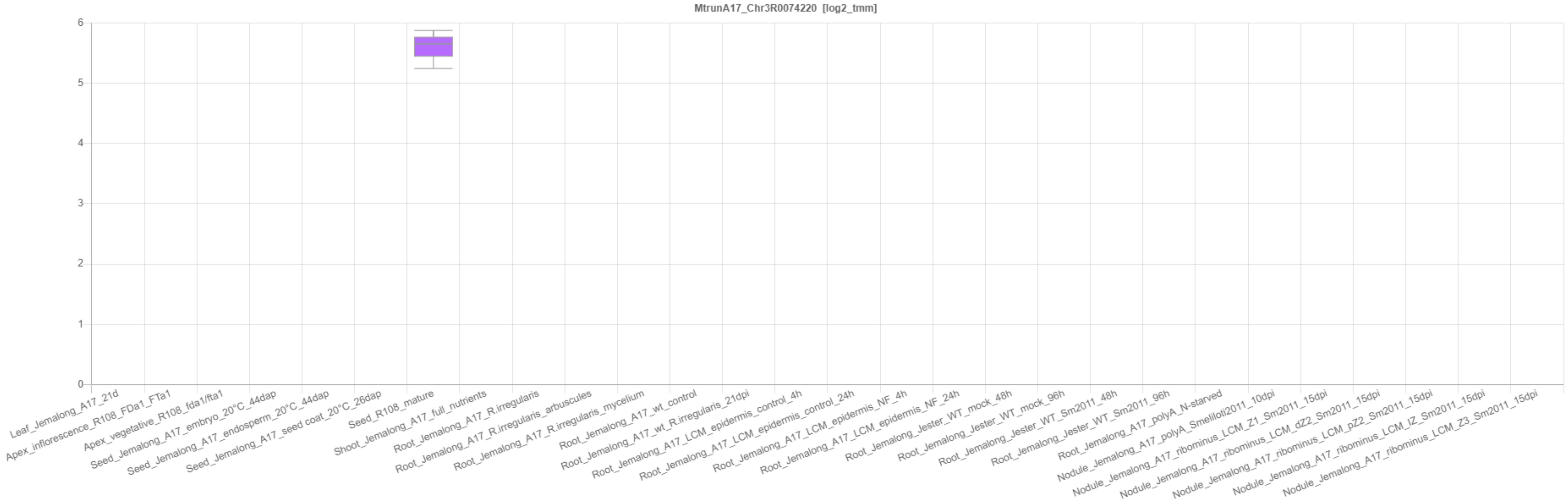

INRAE/CNRS Medicago Express | Expression Atlas Server

lipm-browsers.toulouse.inra.fr/pub/expressionAtlas/app/v3/aa_reference_dataset/MtrunA17_Chr1R0265150

MtExpress V3 | Core (20220901)

! Switch to another dataset using the left menu

lipme INRAE cnrs

TMM | METADATA | SYNONYMOUS | ANNOTATION | GENOME PORTAL | LEGOO

repeat_region: MtrunA17_Chr1R0265150;

Log2 TMM Normalisation using EdgeR (Core [20220901])

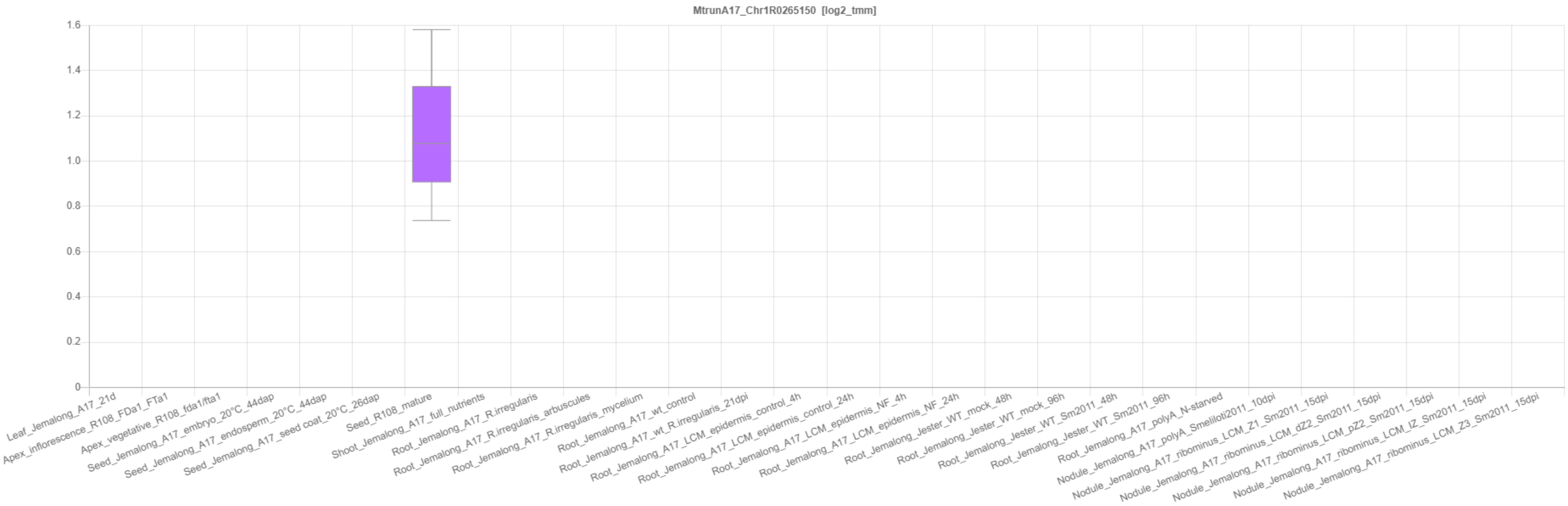

Supplementary Dataset S26. Expression profiles of ten non-overlapping repeat elements that have log2 TMM values above zero. Screenshots are from the RNA-Seq-based gene expression atlas of *Medicago truncatula* (MtExpress v. 3, https://medicago.toulouse.inrae.fr/GEA). The list corresponds to repeat elements marked with "Yes" in column B of Supplementary Dataset S25. For clarity and better visibility, only the core sample set is shown. Note that some repeat elements in this list have tissue- or sample-specific expression.
