# Supplementary material for: Discovery of diverse chimeric peptides in a eukaryotic proteome sets the stage for experimental validation of the mosaic translation hypothesis
Source: Comput Struct Biotechnol J. 2025 Sep 12;27:4048–64. doi: 10.1016/j.csbj.2025.09.019 (PMC12481079; doi:10.1016/j.csbj.2025.09.019)
Supplement: Supplementary file 1 — Supplementary material [file mmc1.zip › Supplementary Datasets/Supplementary Dataset S27 Expression profiles of 156 primary-source transcripts.pdf]

CP1: MtrunA17_Chr0c01g0489091

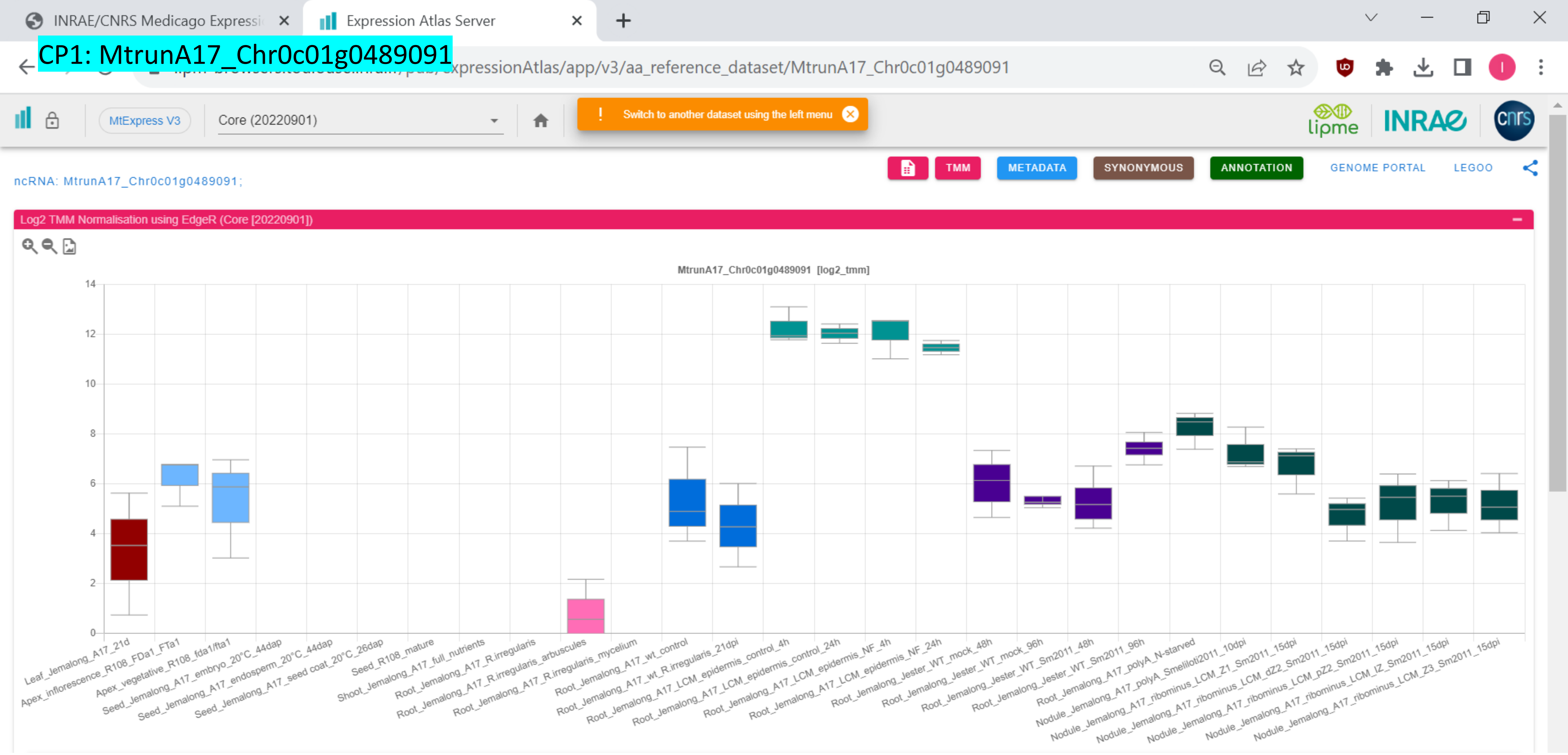

CP2: MtrunA17_Chr0c28g0493951

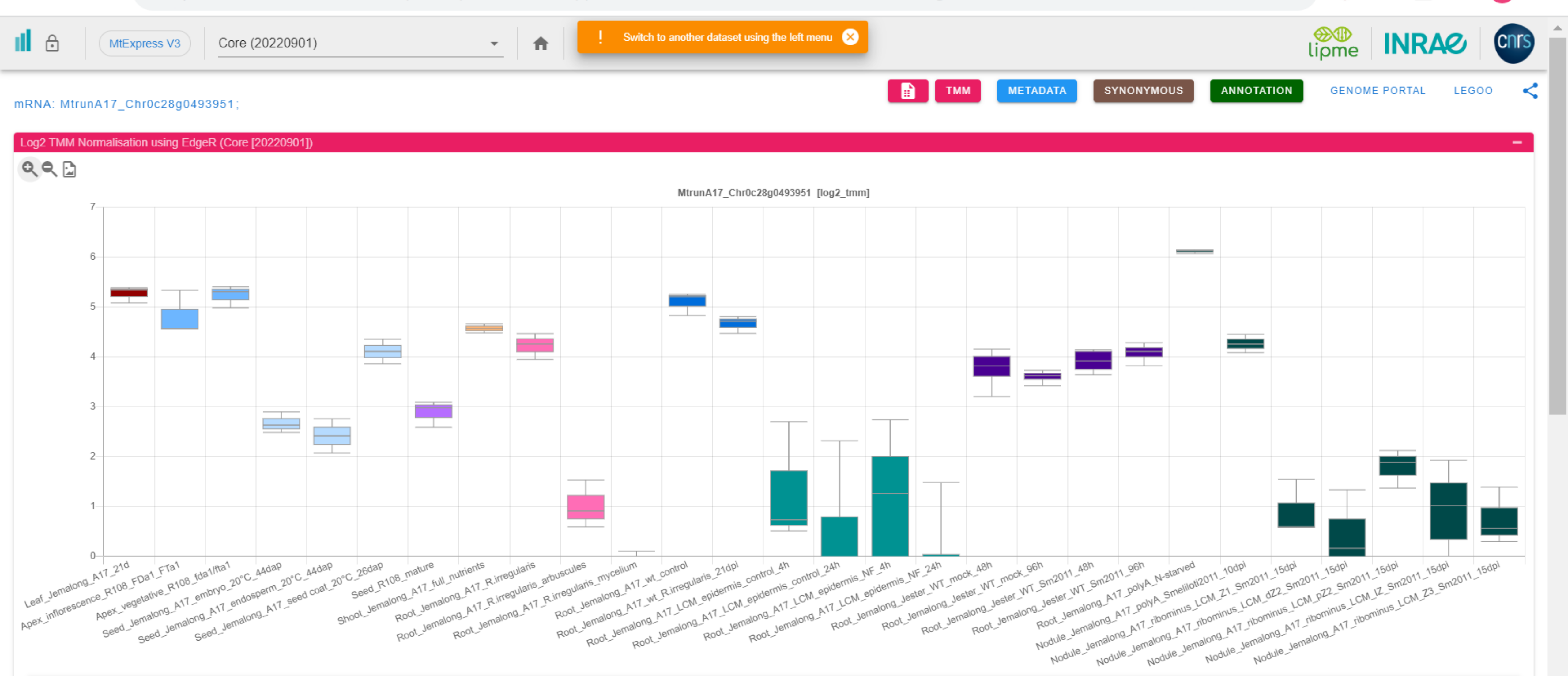

CP3: MtrunA17_Chr1g0148371

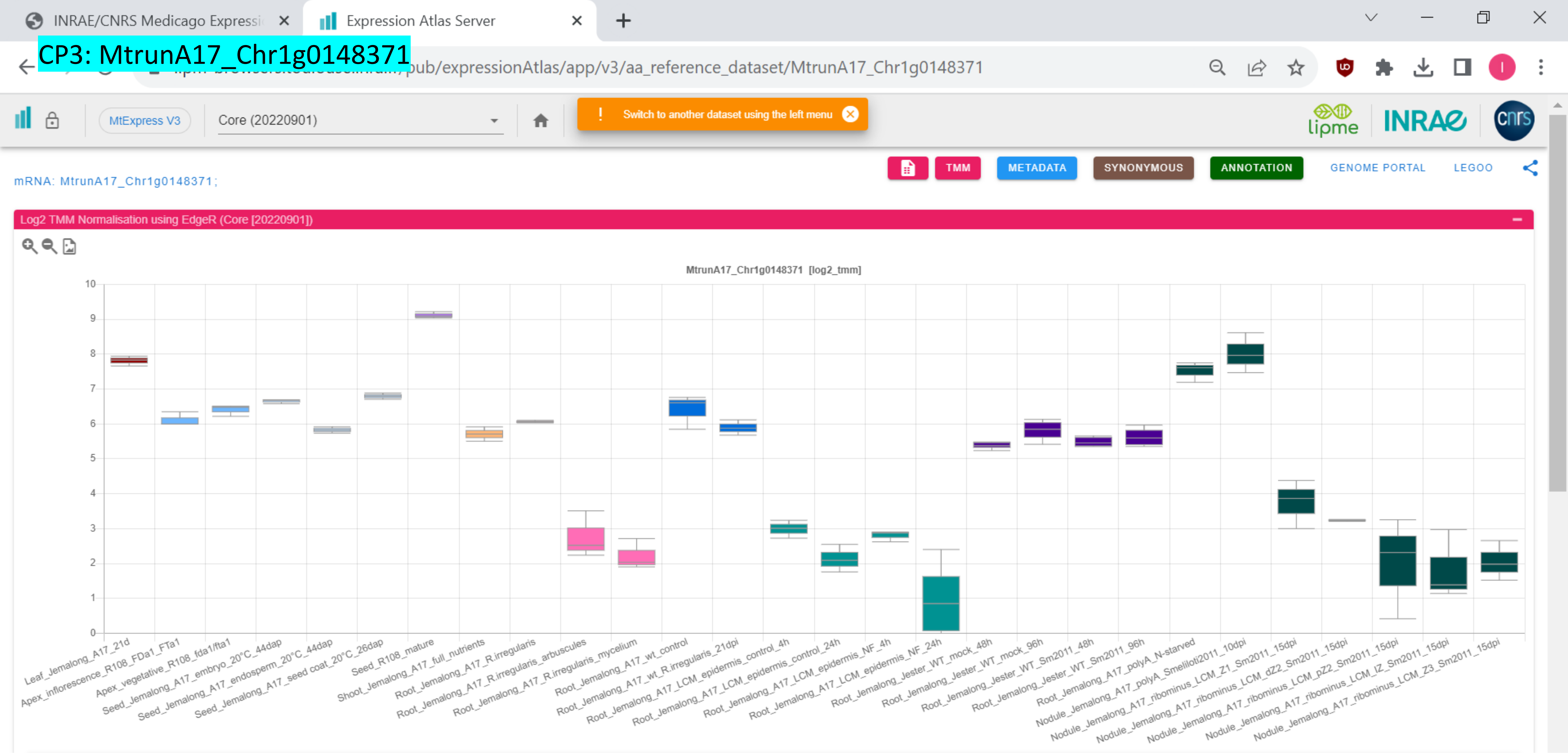

CP4: MtrunA17_Chr1g0149991

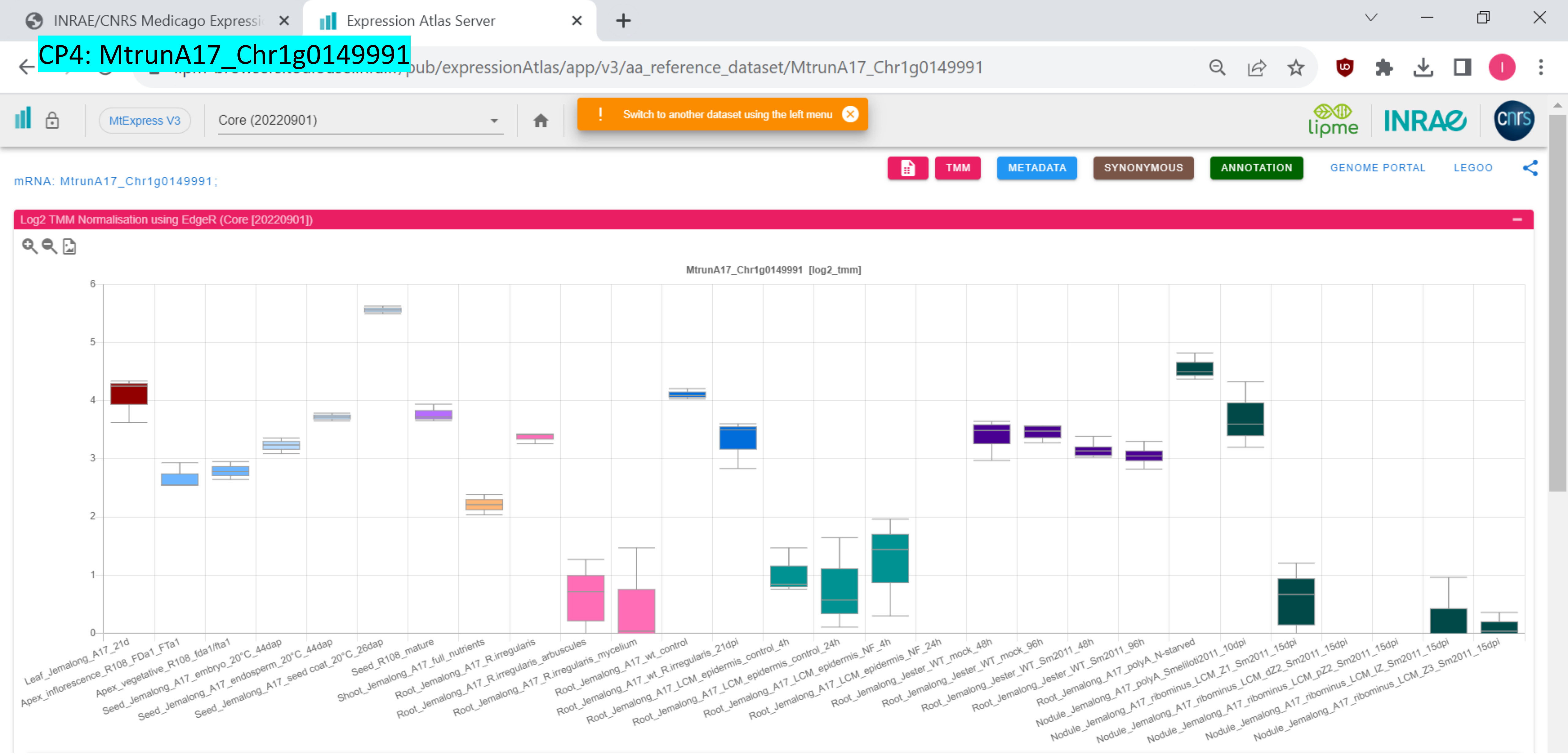

CP5: MtrunA17_Chr1g0150571

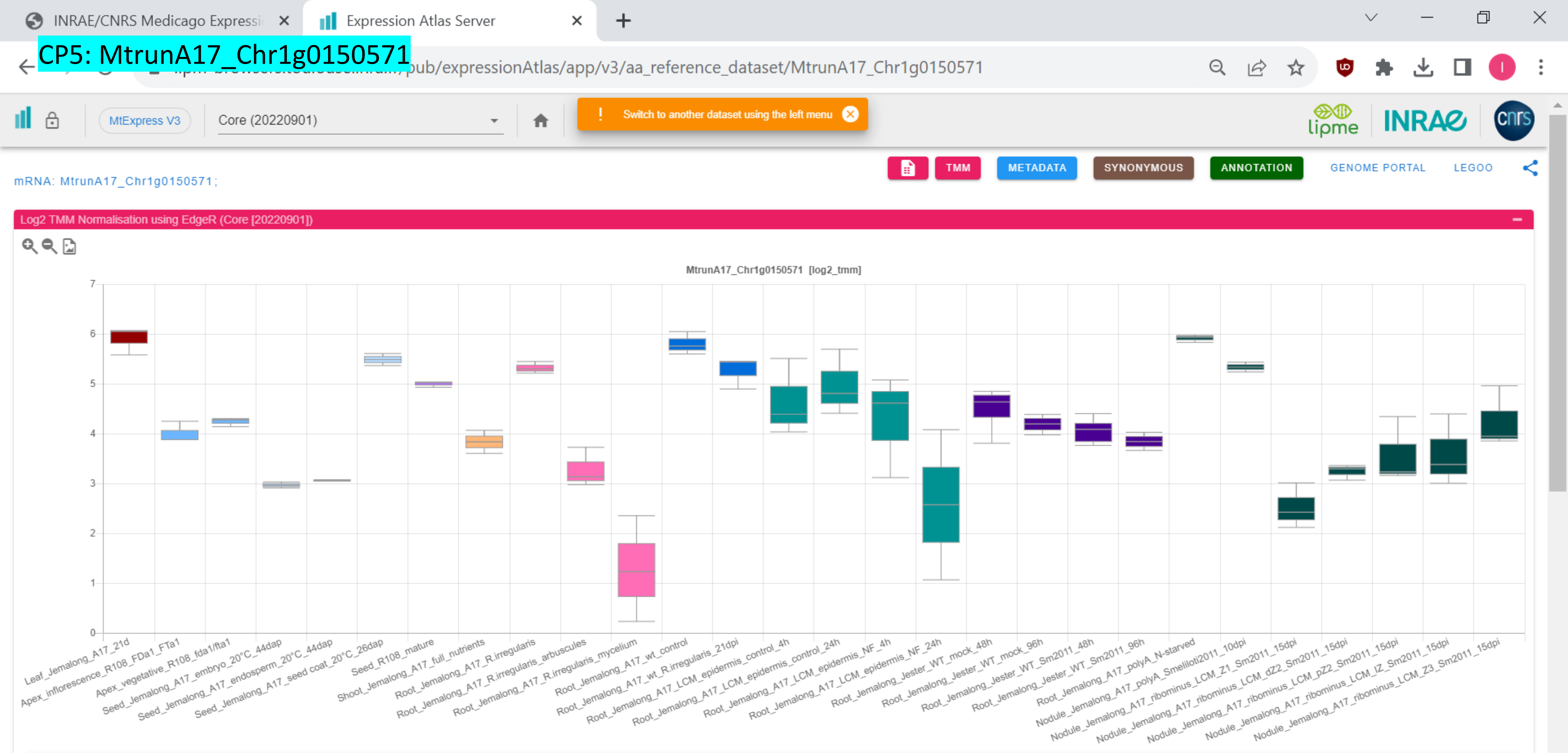

*CP6: MtrunA17_Chr1g0152521

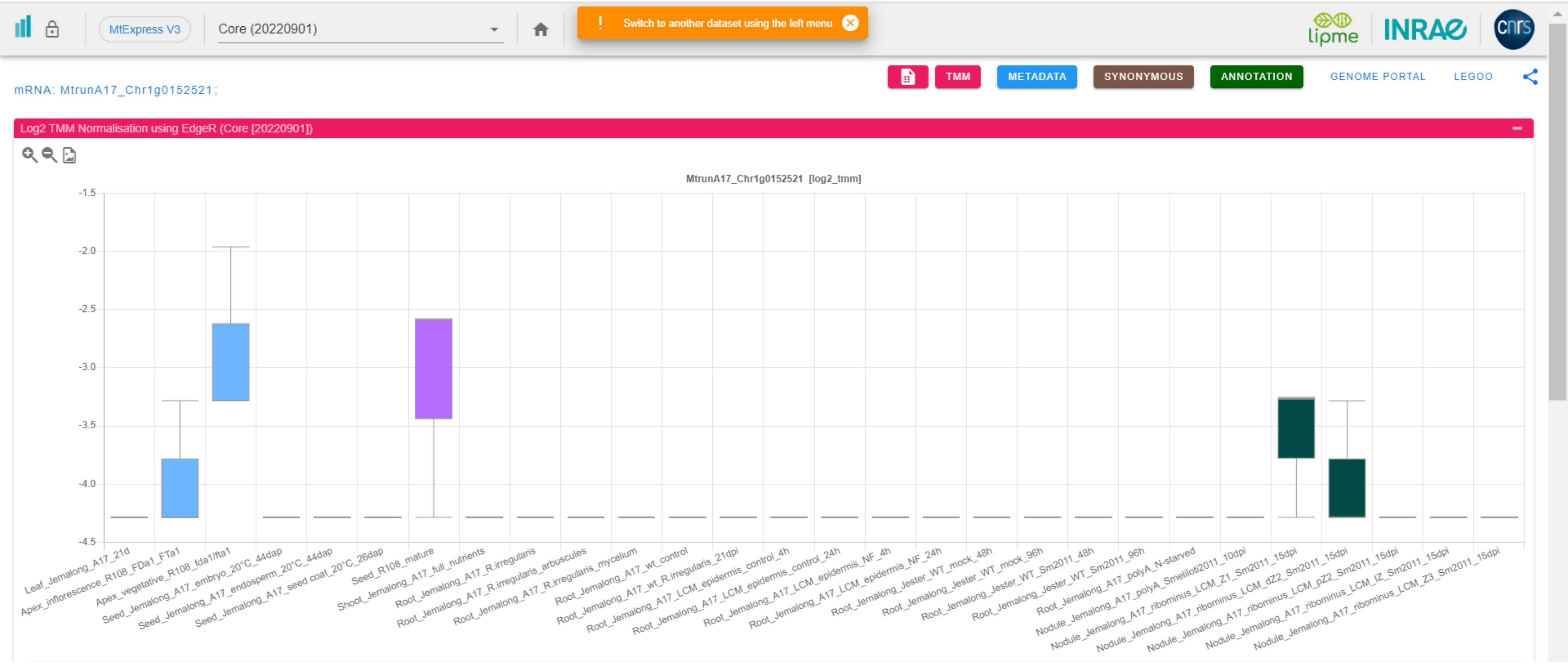

CP7: MtrunA17_Chr1g0153001

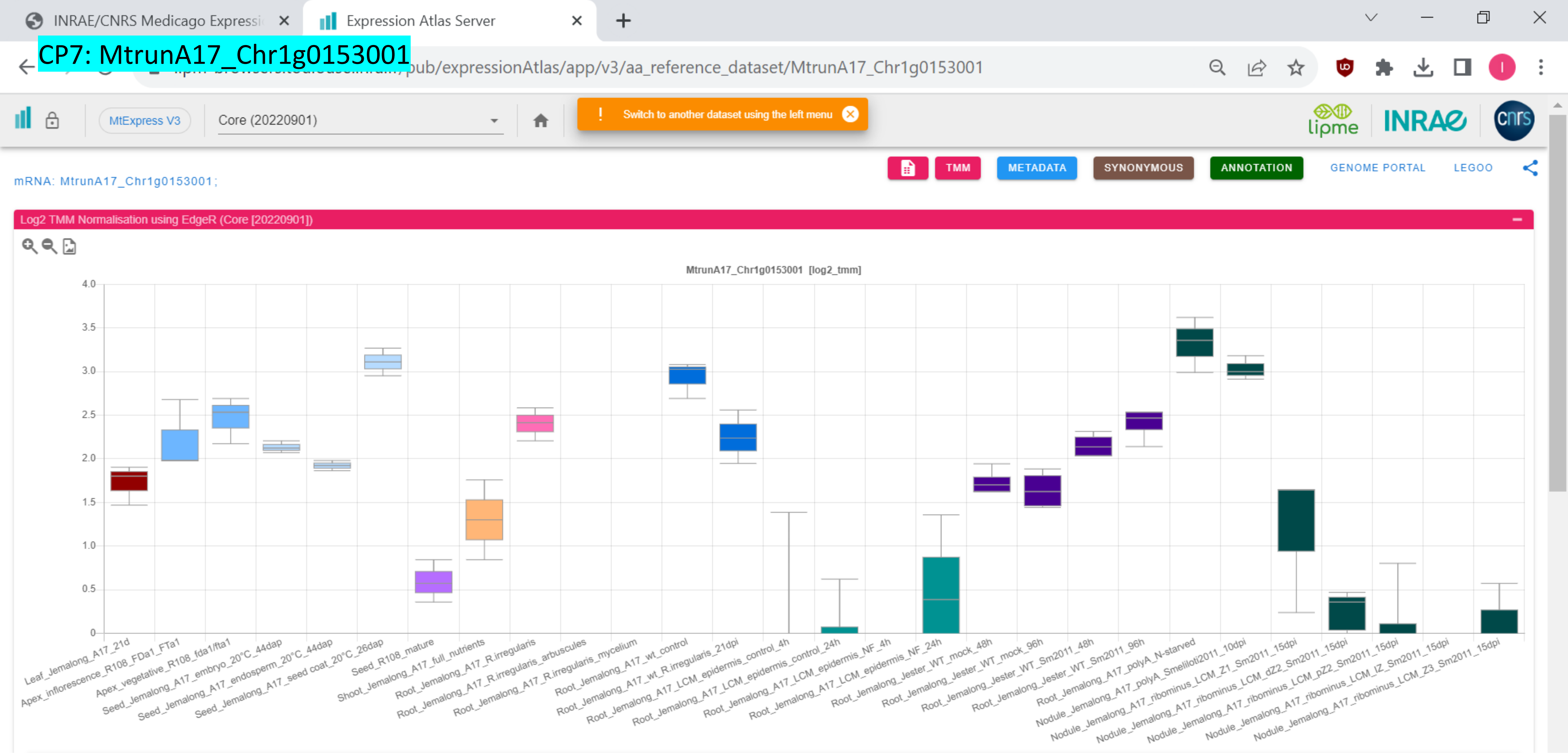

CP8: MtrunA17_Chr1g0155251

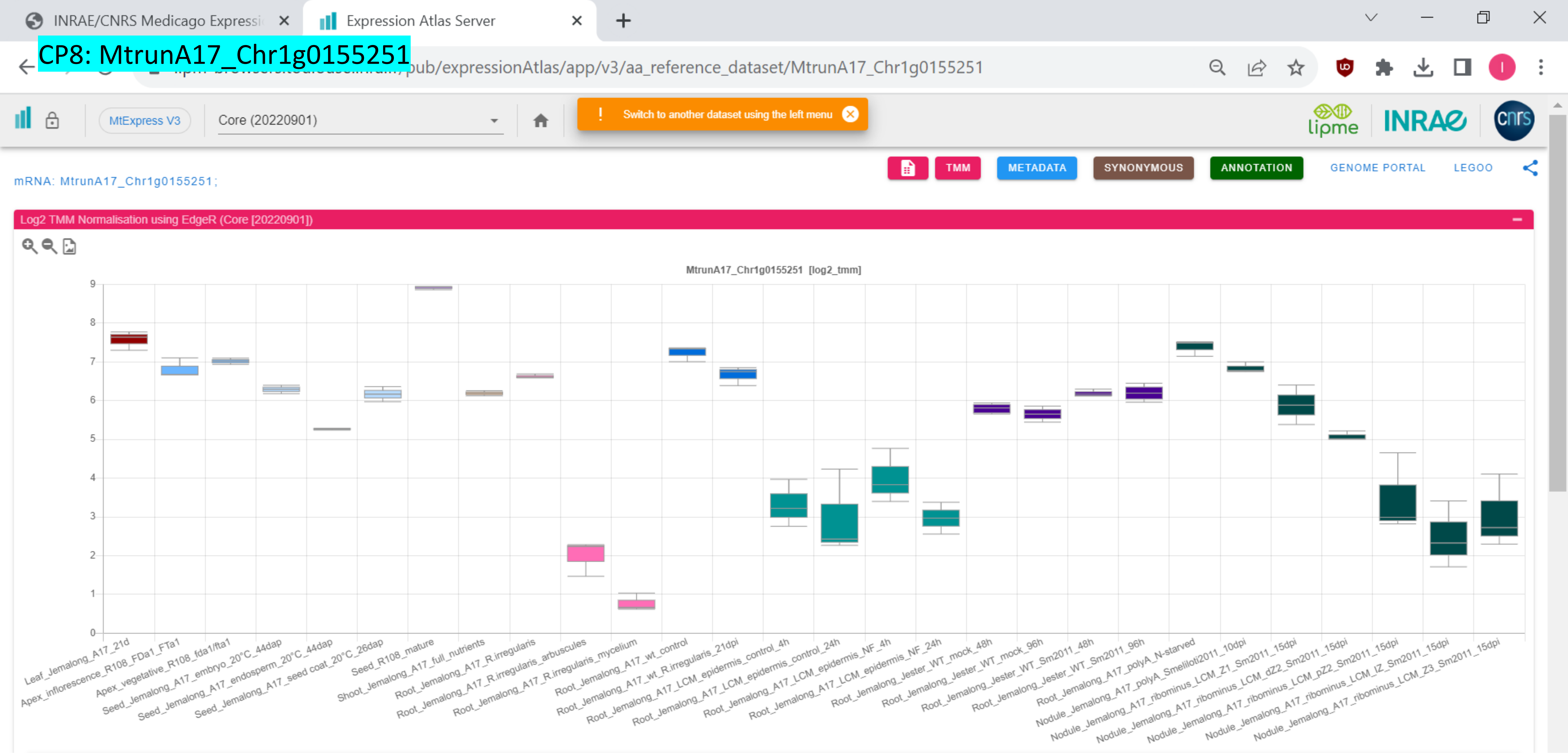

CP9: MtrunA17_Chr1g0158341

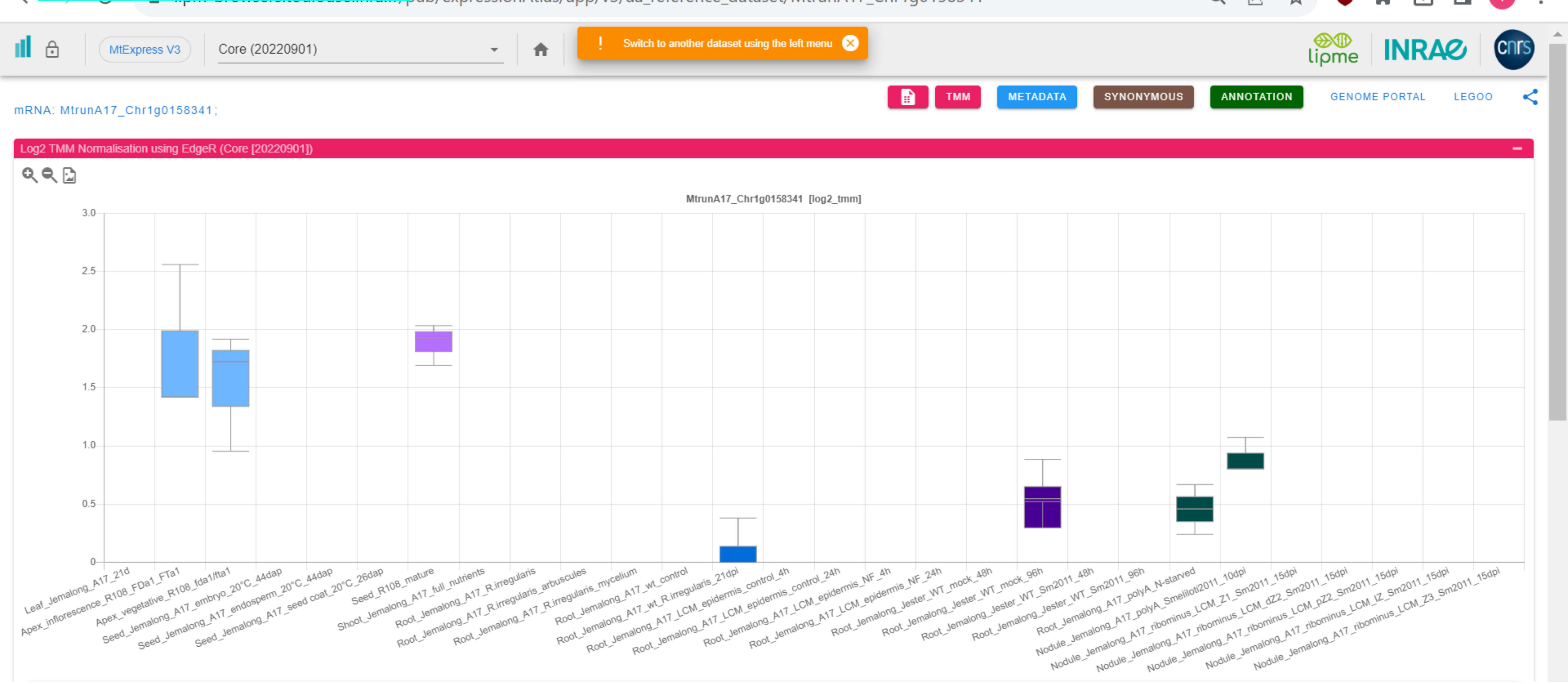

CP10: MtrunA17_Chr1g0162101

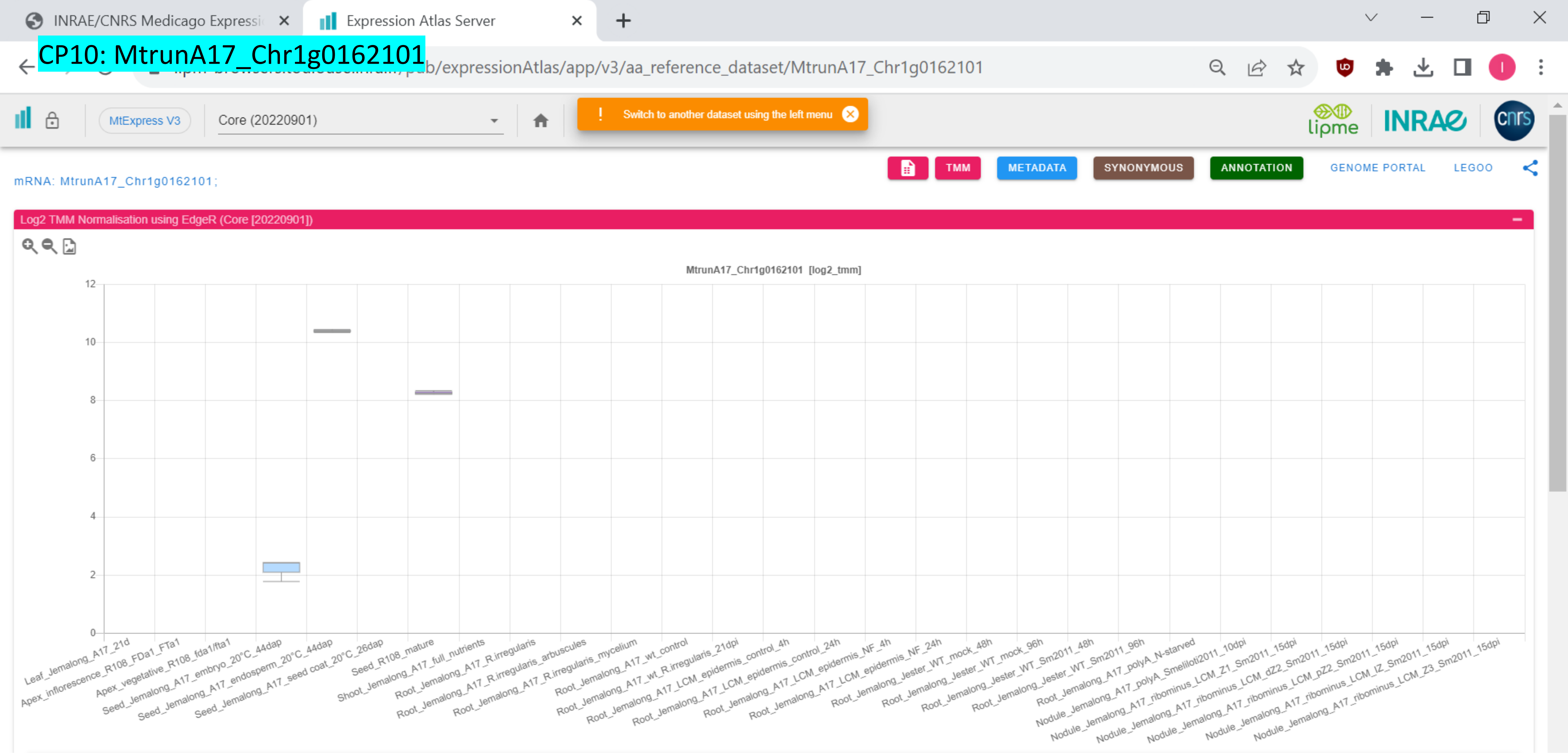

CP11: MtrunA17_Chr1g0164591

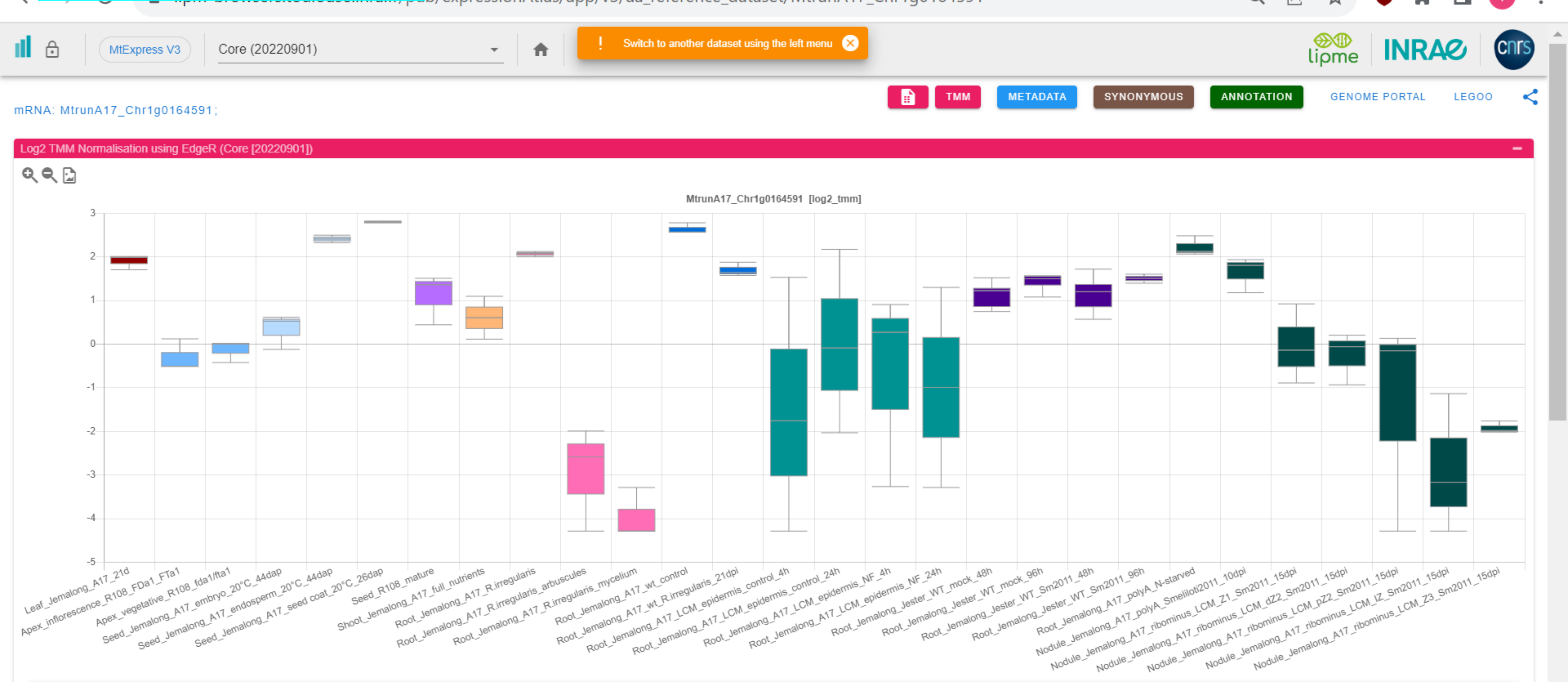

CP12: MtrunA17_Chr1g0178361

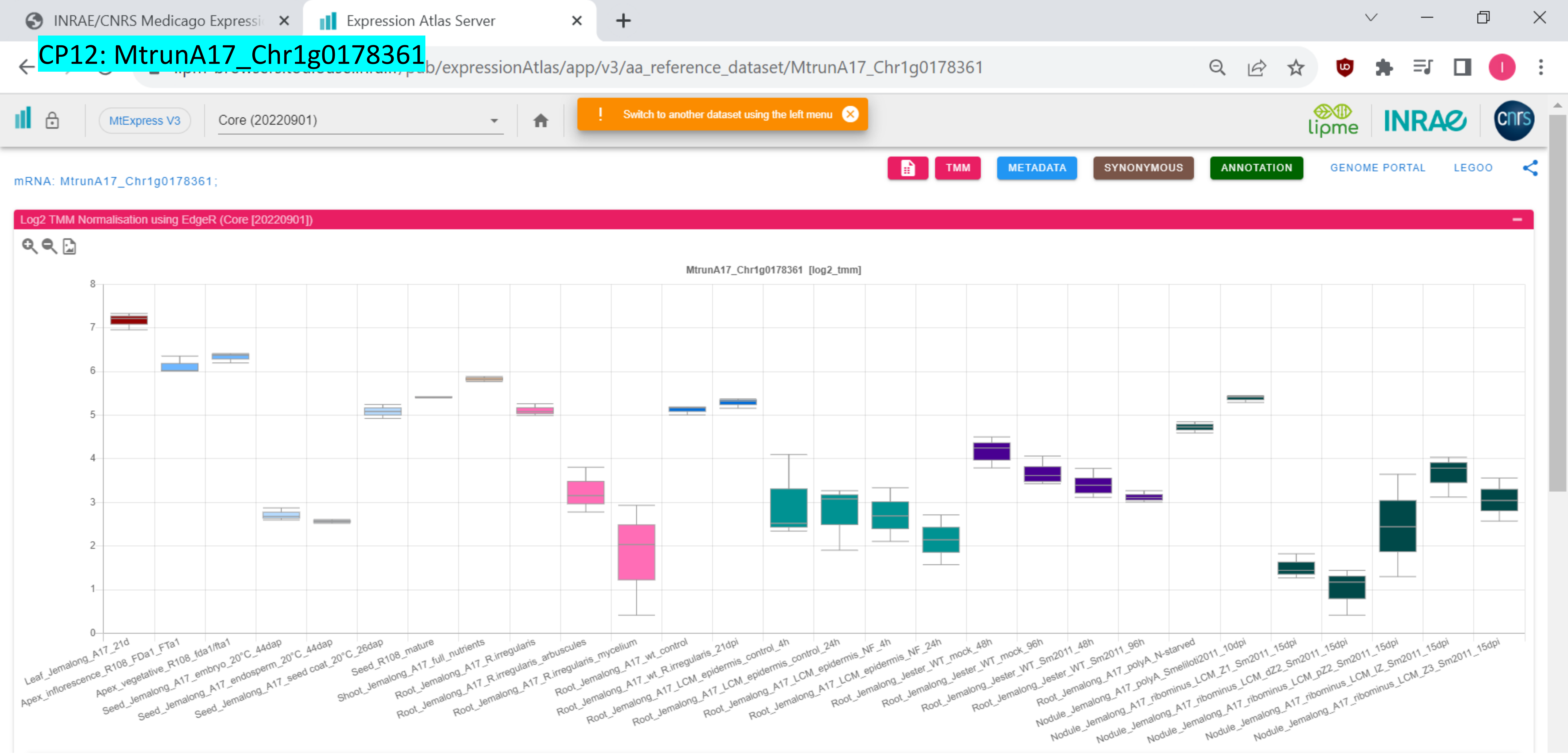

CP13: MtrunA17_Chr1g0181761

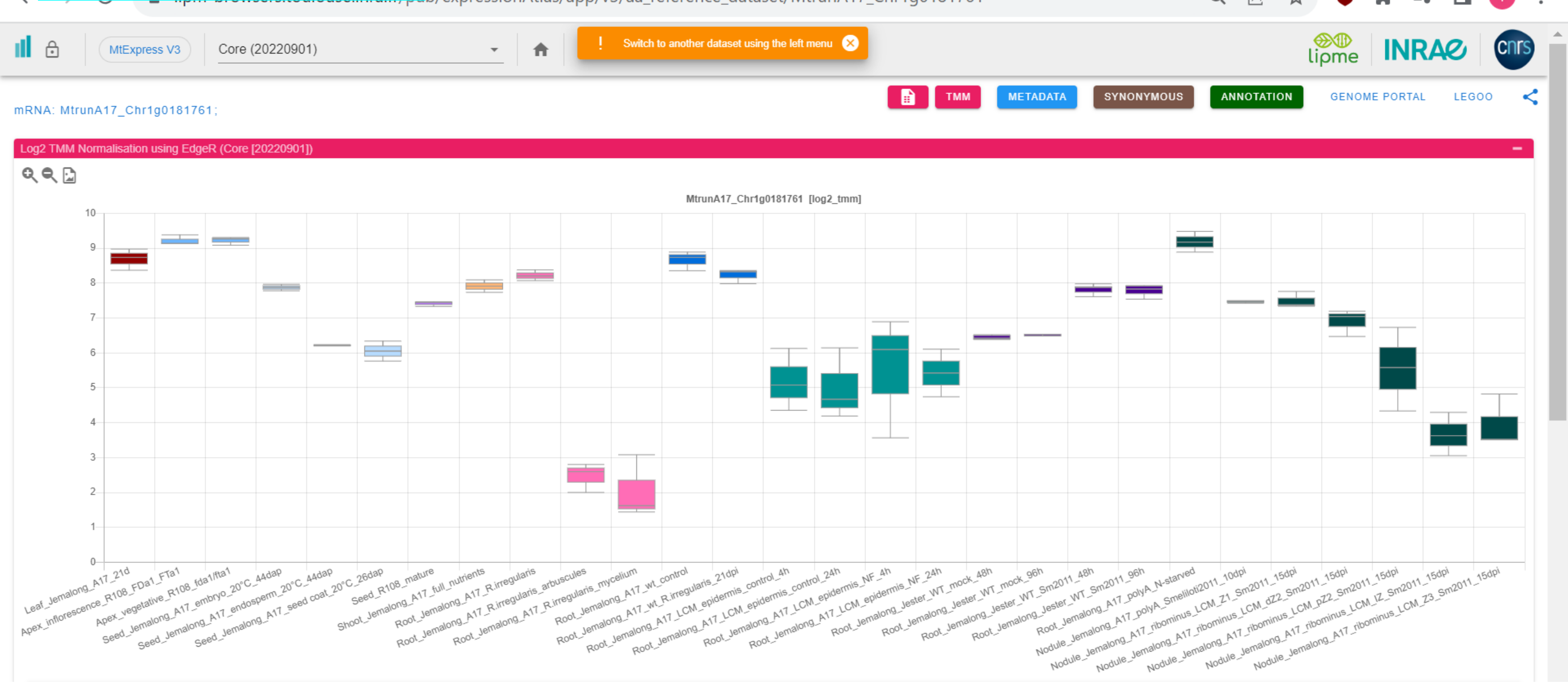

CP14: MtrunA17_Chr1g0182591

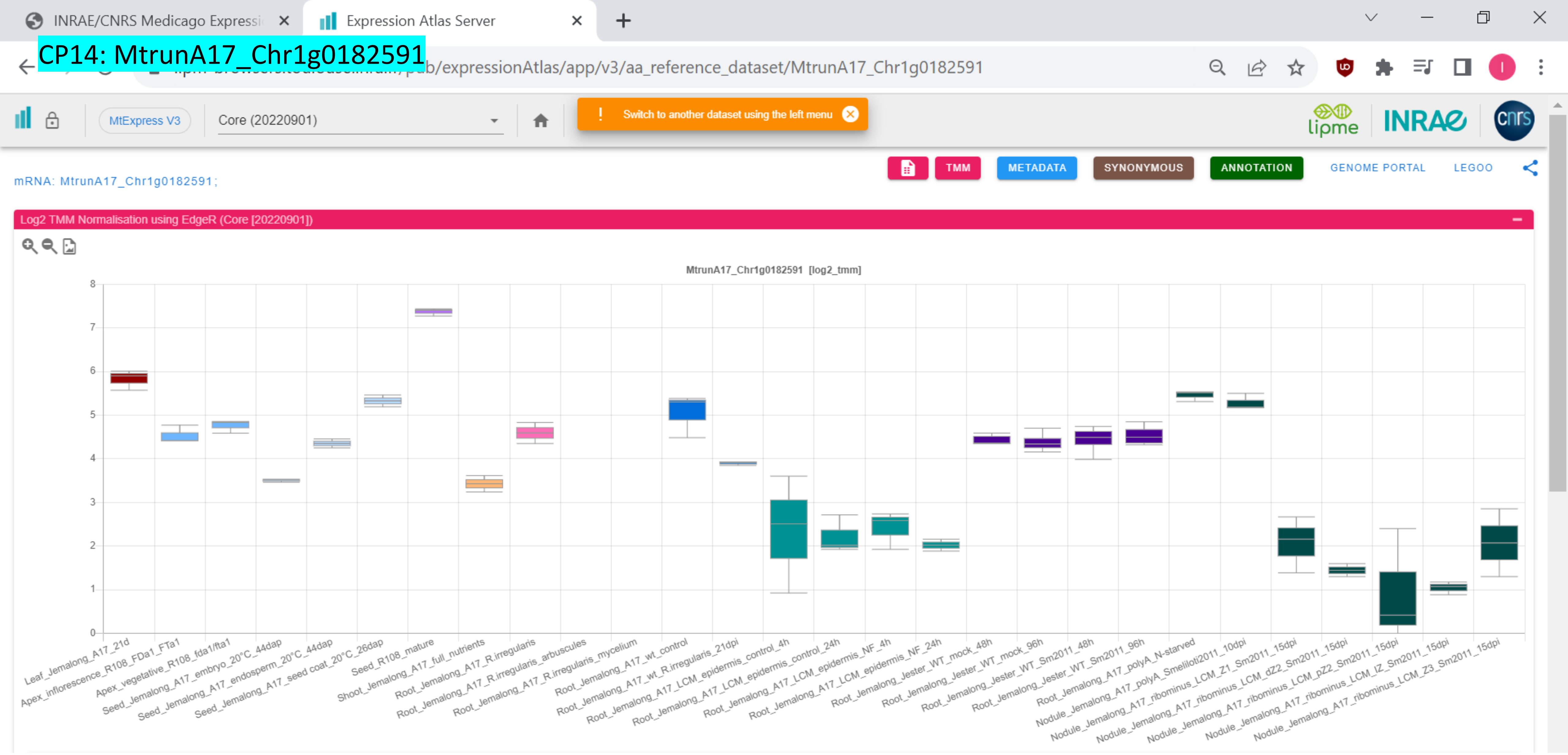

CP15: MtrunA17_Chr1g0183001

INRAE/CNRS Medicago Expressi | Expression Atlas Server

…pub/expressionAtlas/app/v3/aa_reference_dataset/MtrunA17_Chr1g0183001

MtExpress V3 | Core (20220901)

! Switch to another dataset using the left menu

TMM | METADATA | SYNONYMOUS | ANNOTATION | GENOME PORTAL | LEGOO

mRNA: MtrunA17_Chr1g0183001;

Log2 TMM Normalisation using EdgeR (Core [20220901])

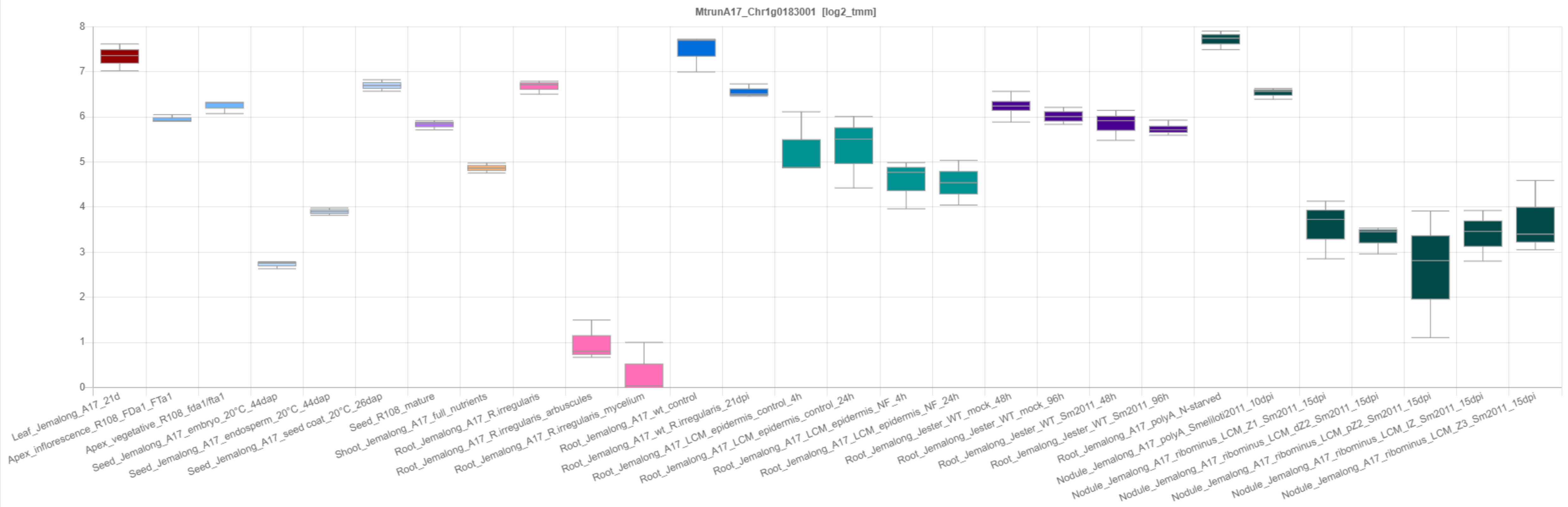

CP16: MtrunA17_Chr1g0185811

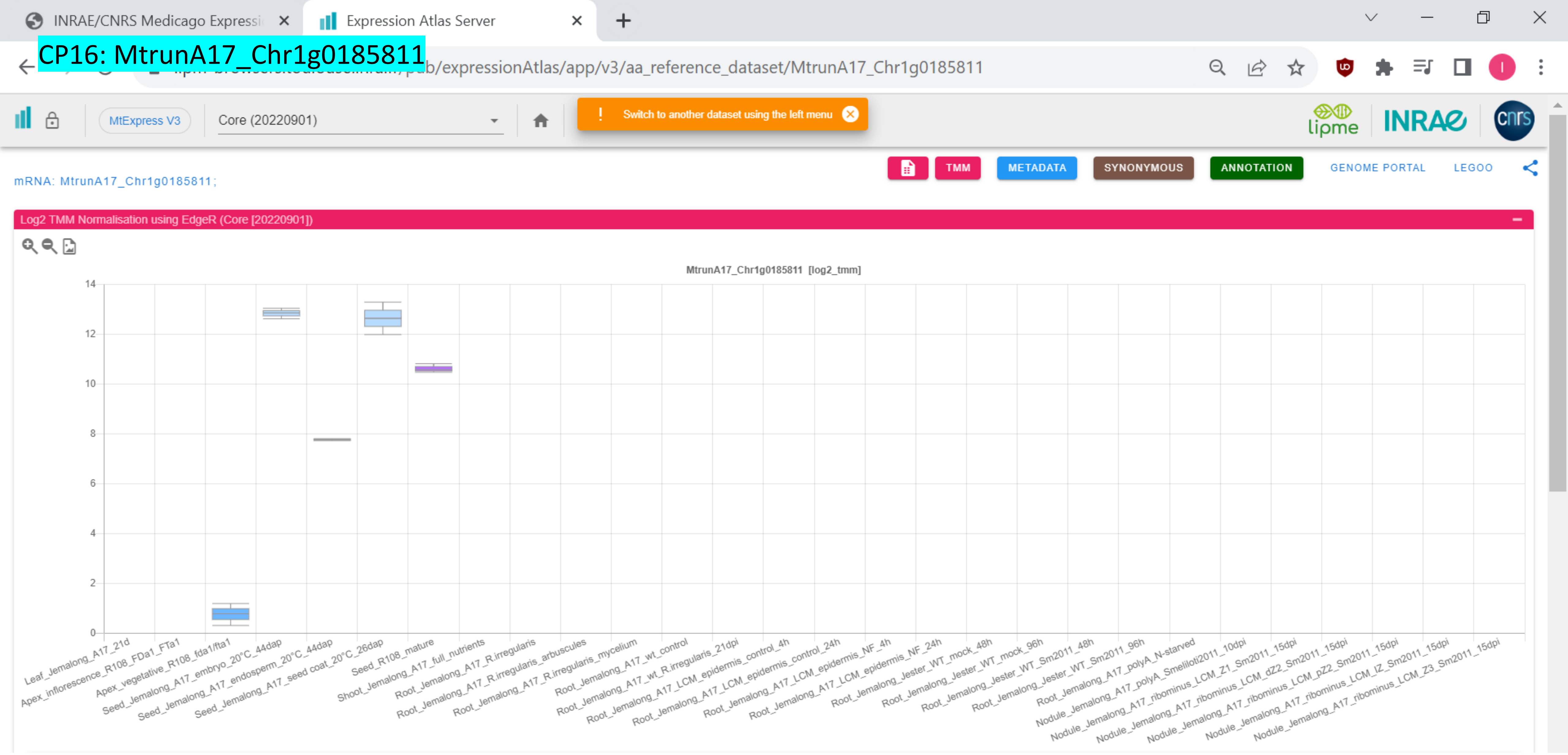

CP17: MtrunA17_Chr1g0185811

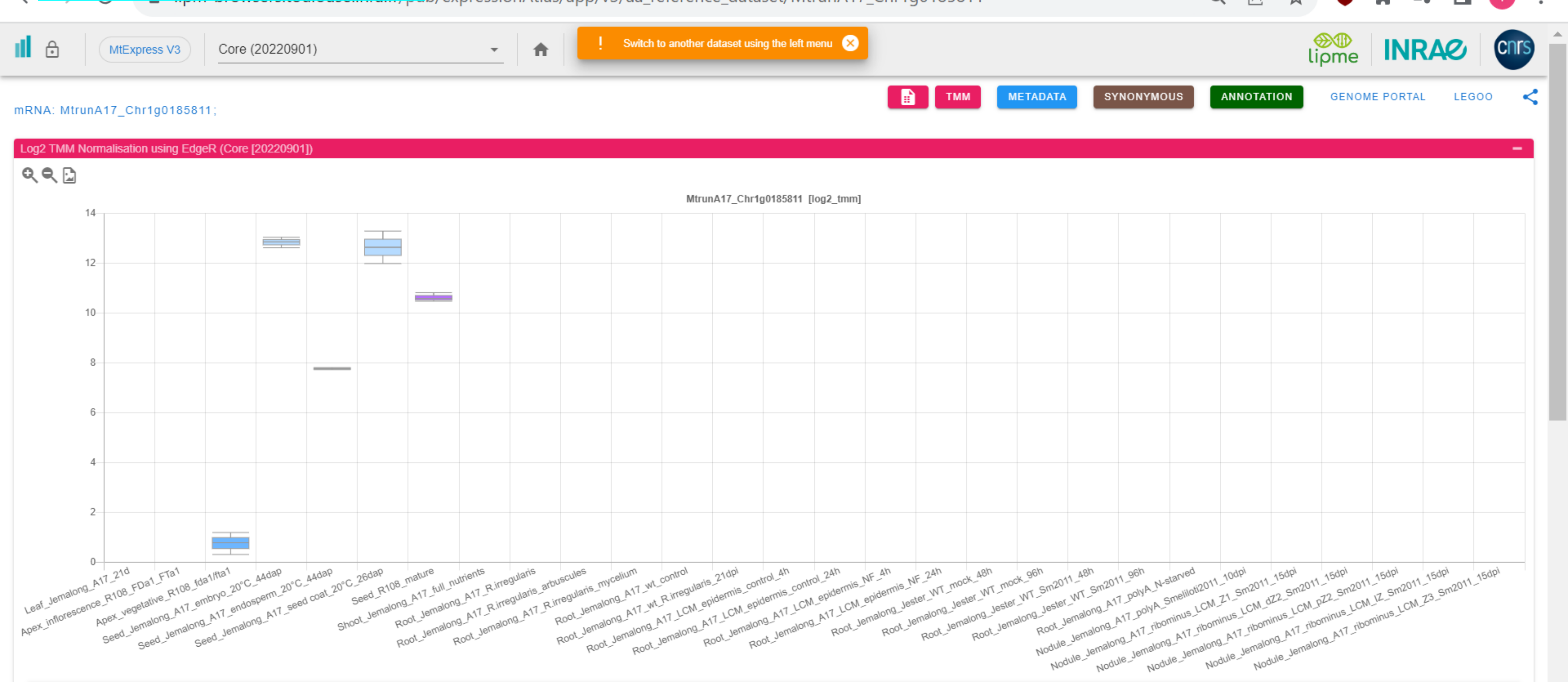

CP18: MtrunA17_Chr1g0185811

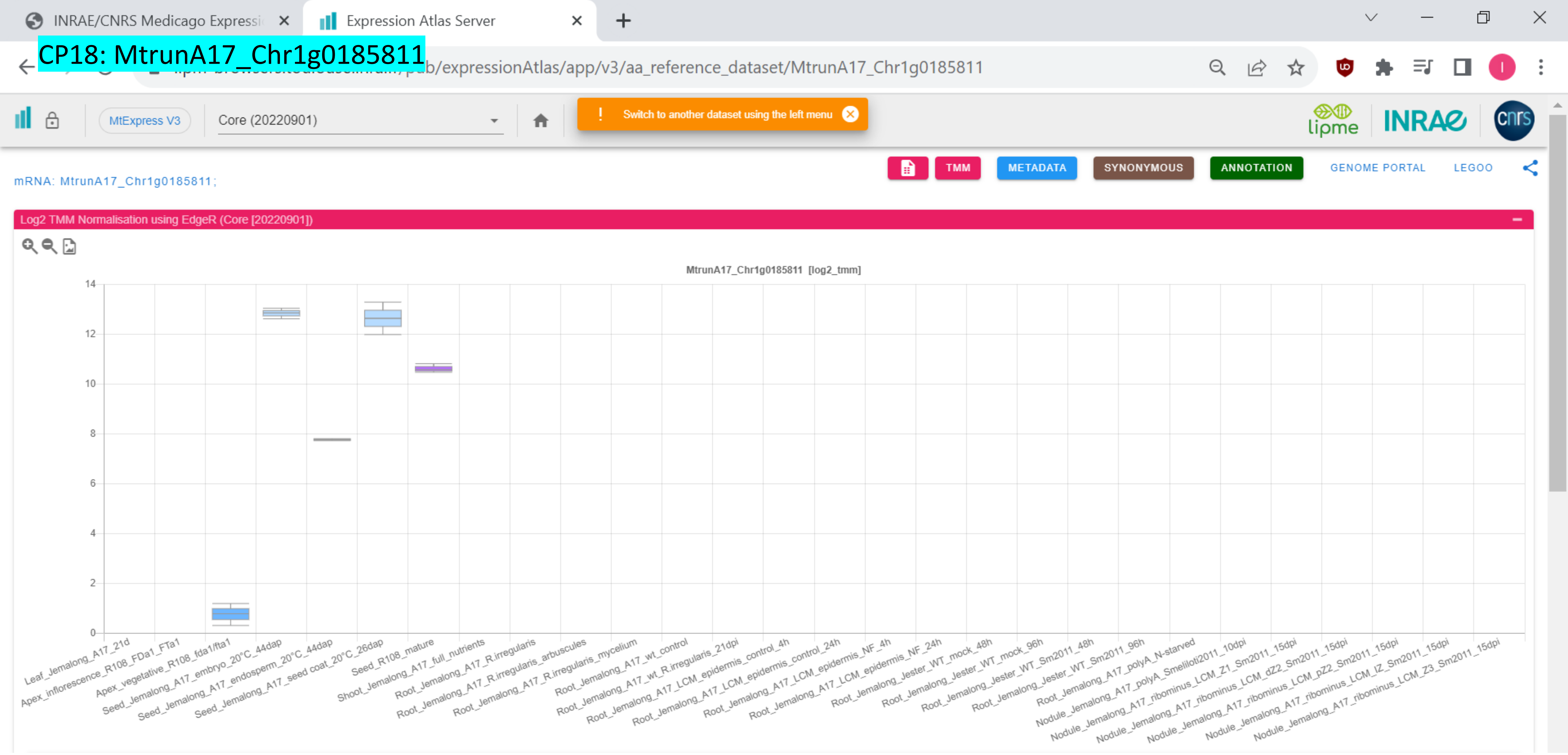

CP19: MtrunA17_Chr1g0185871

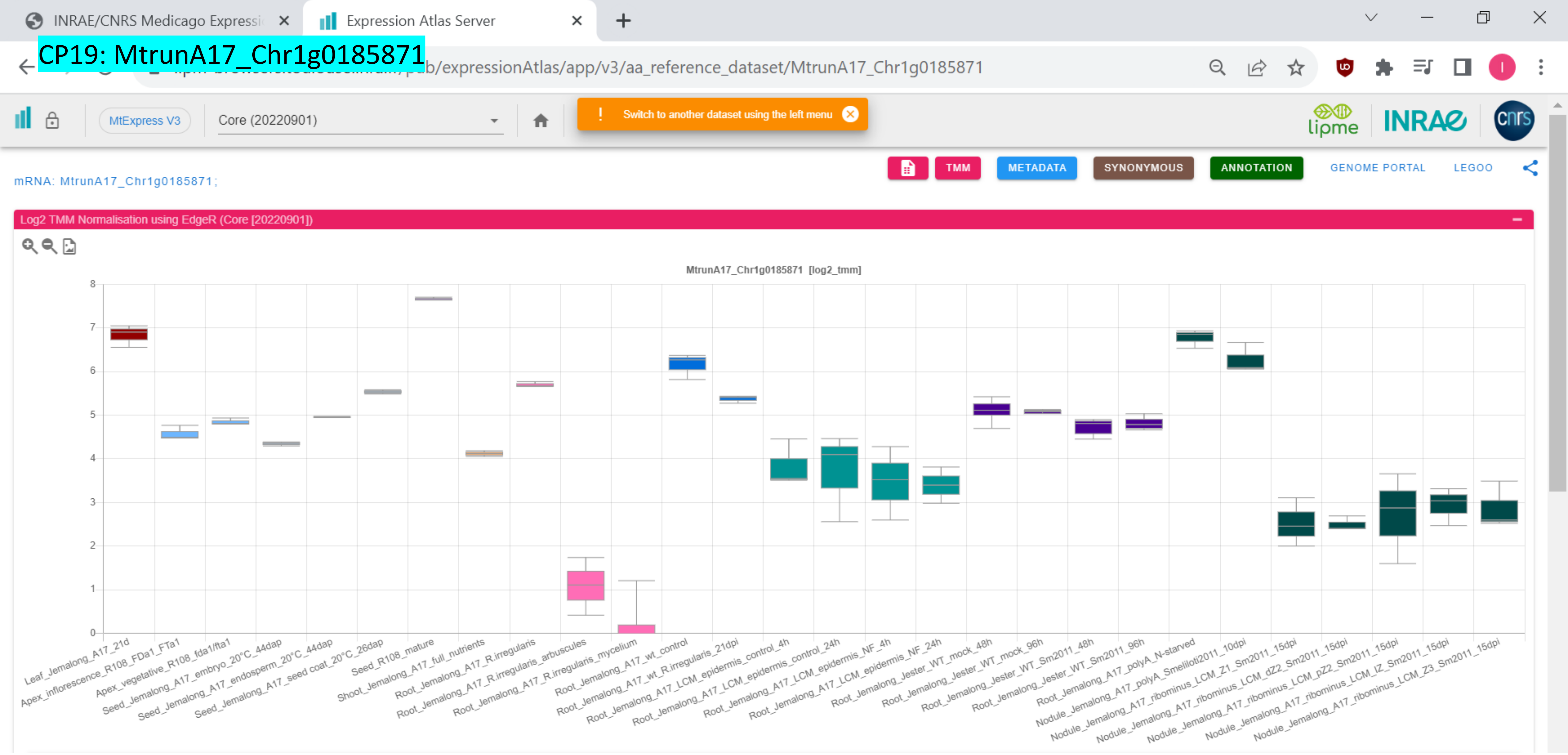

*CP20: MtrunA17_Chr1g0190571

INRAE/CNRS Medicago Expressi | Expression Atlas Server

expressionAtlas/app/v3/aa_reference_dataset/MtrunA17_Chr1g0190571

MtExpress V3 | Core (20220901) | Switch to another dataset using the left menu

TMM | METADATA | SYNONYMOUS | ANNOTATION | GENOME PORTAL | LEGOO

mRNA: MtrunA17_Chr1g0190571;

Log2 TMM Normalisation using EdgeR (Core [20220901])

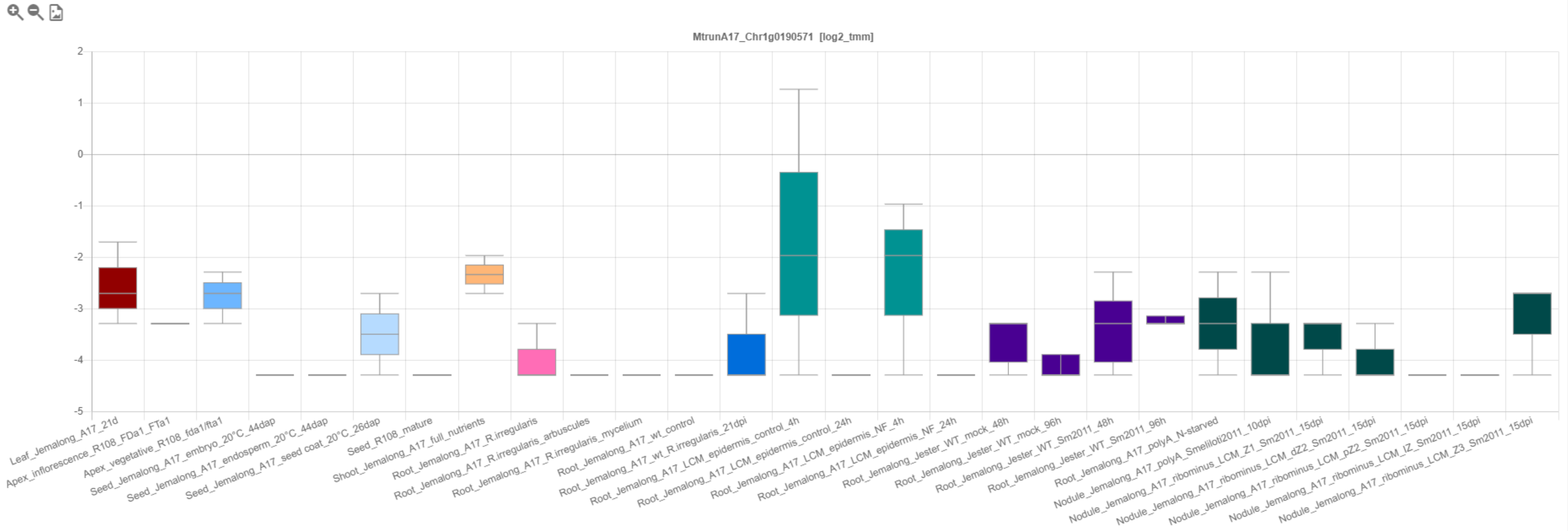

CP21: MtrunA17_Chr1g0191411

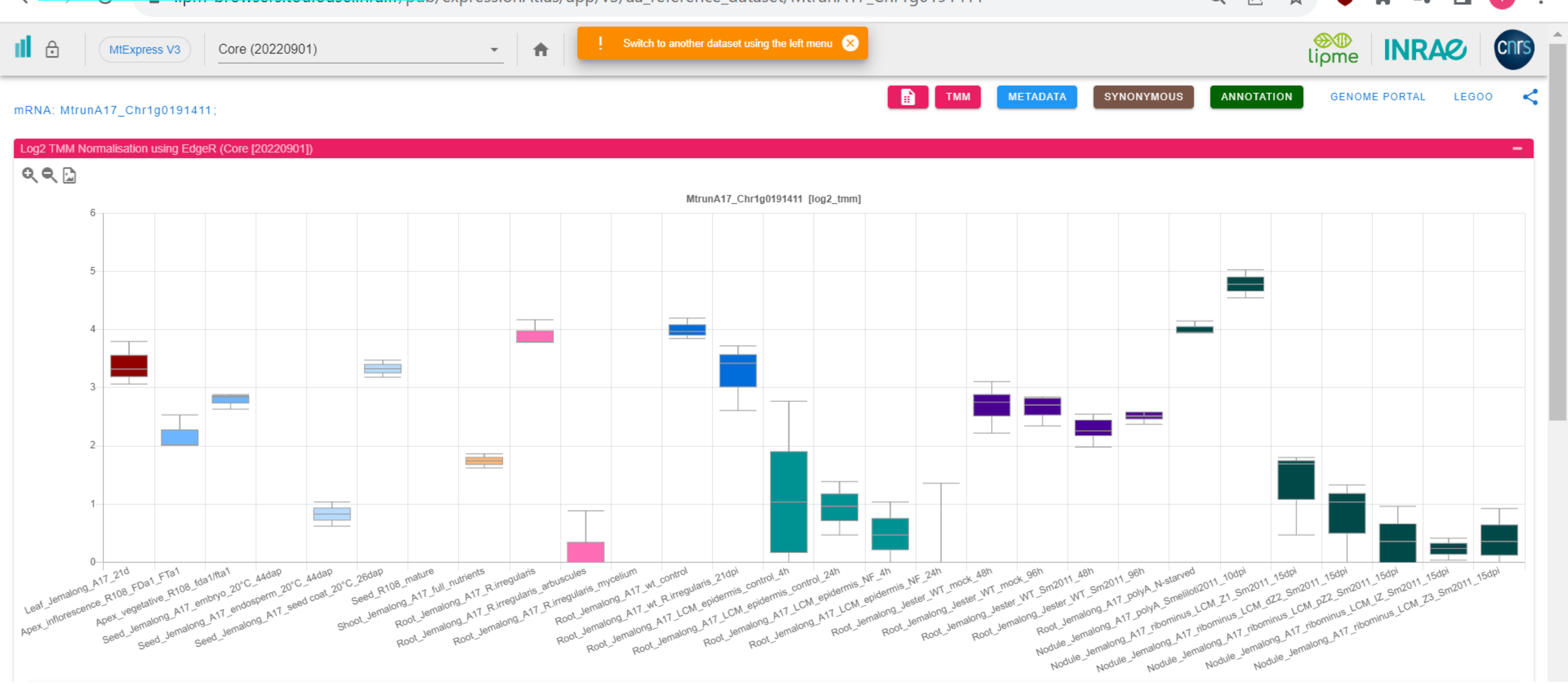

CP22: MtrunA17_Chr1g0198091

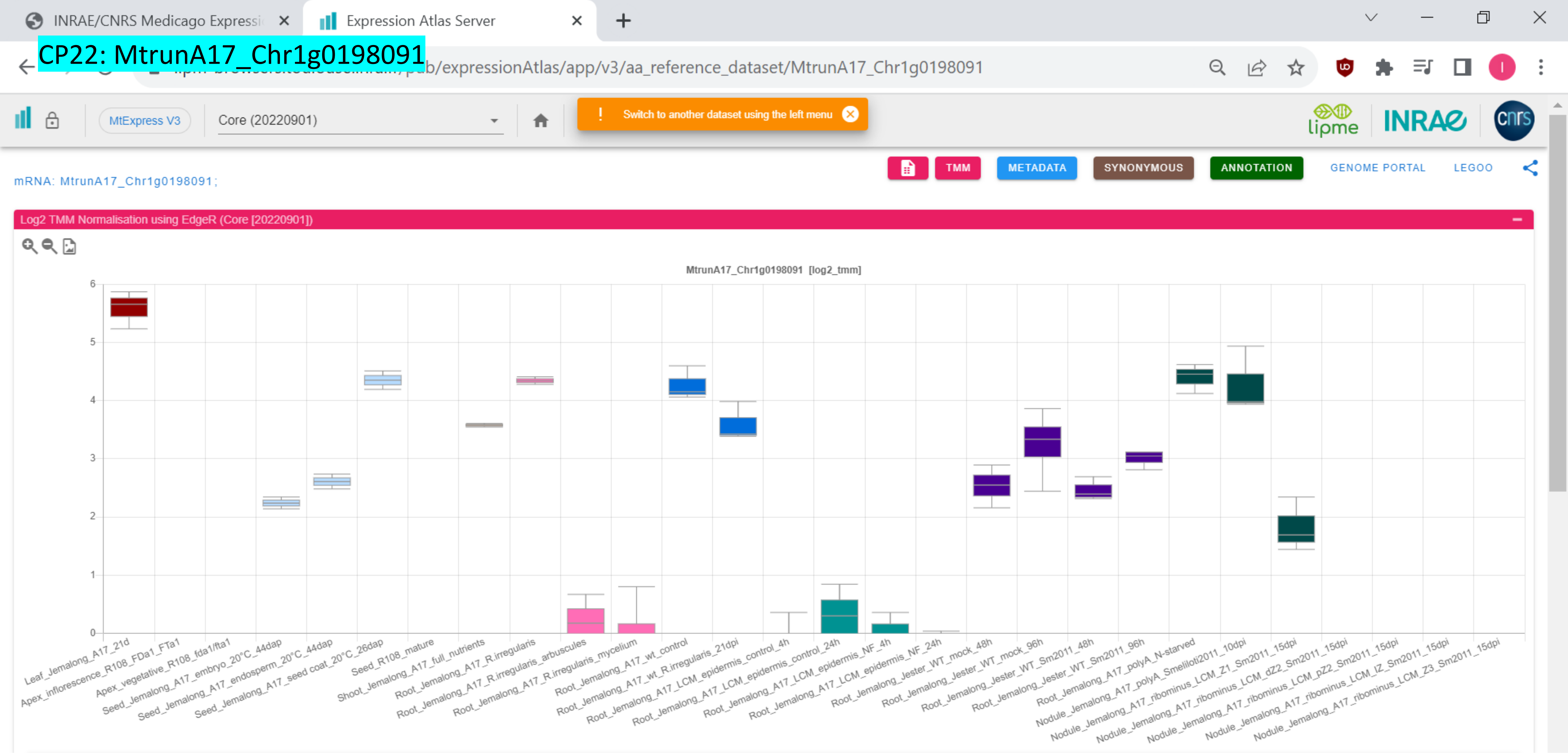

CP23: MtrunA17_Chr1g0200071

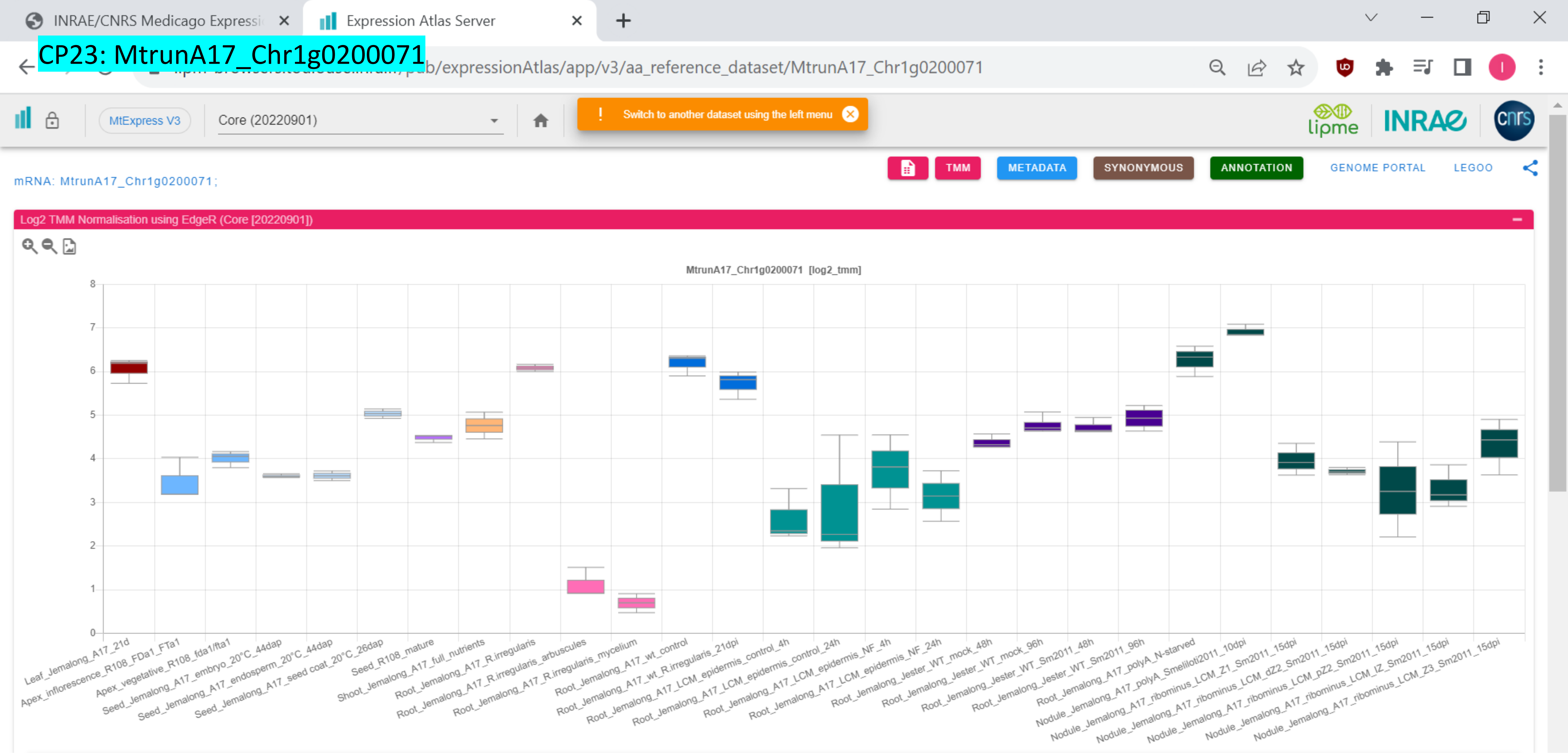

CP24: MtrunA17_Chr1g0200071

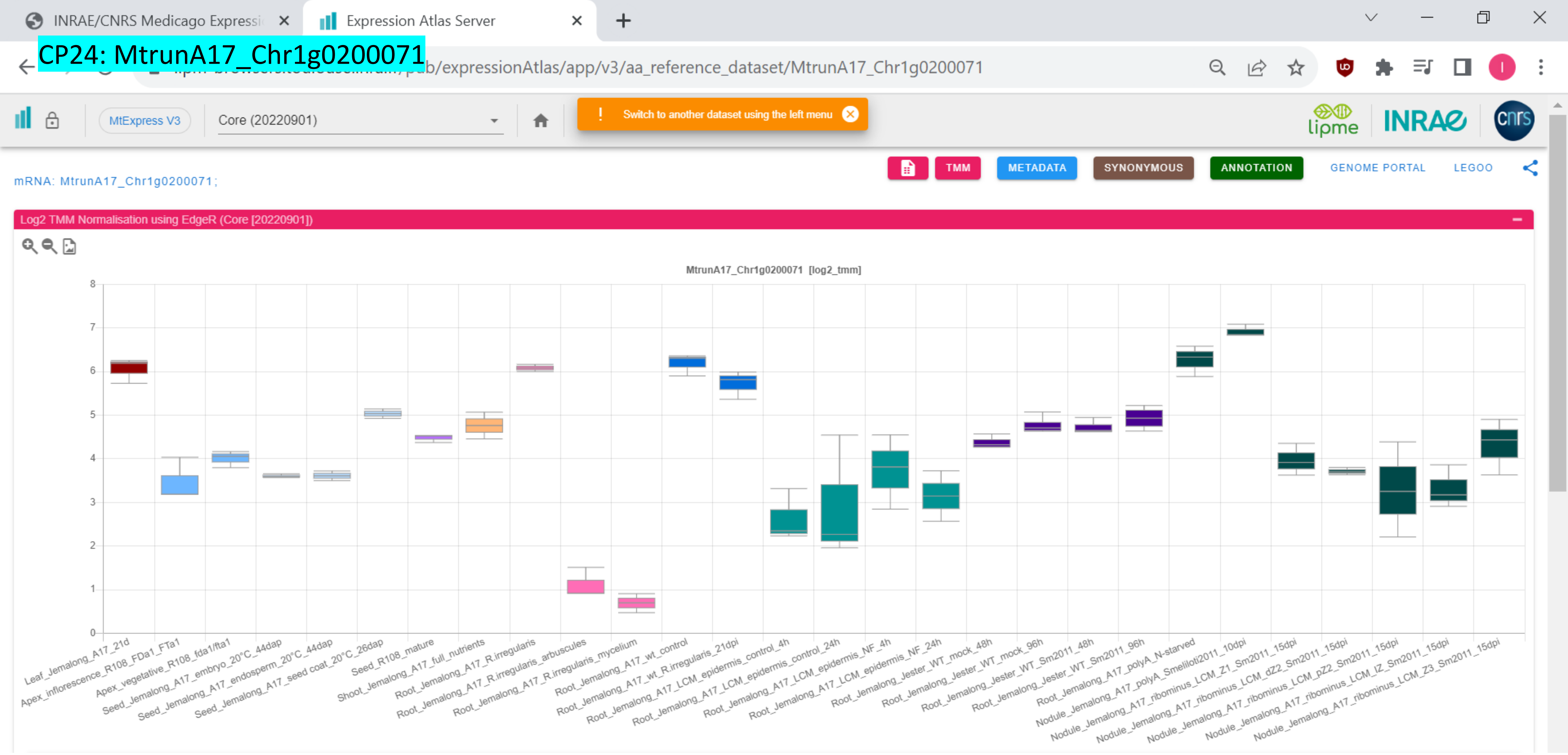

*CP25: MtrunA17_Chr1g0202001

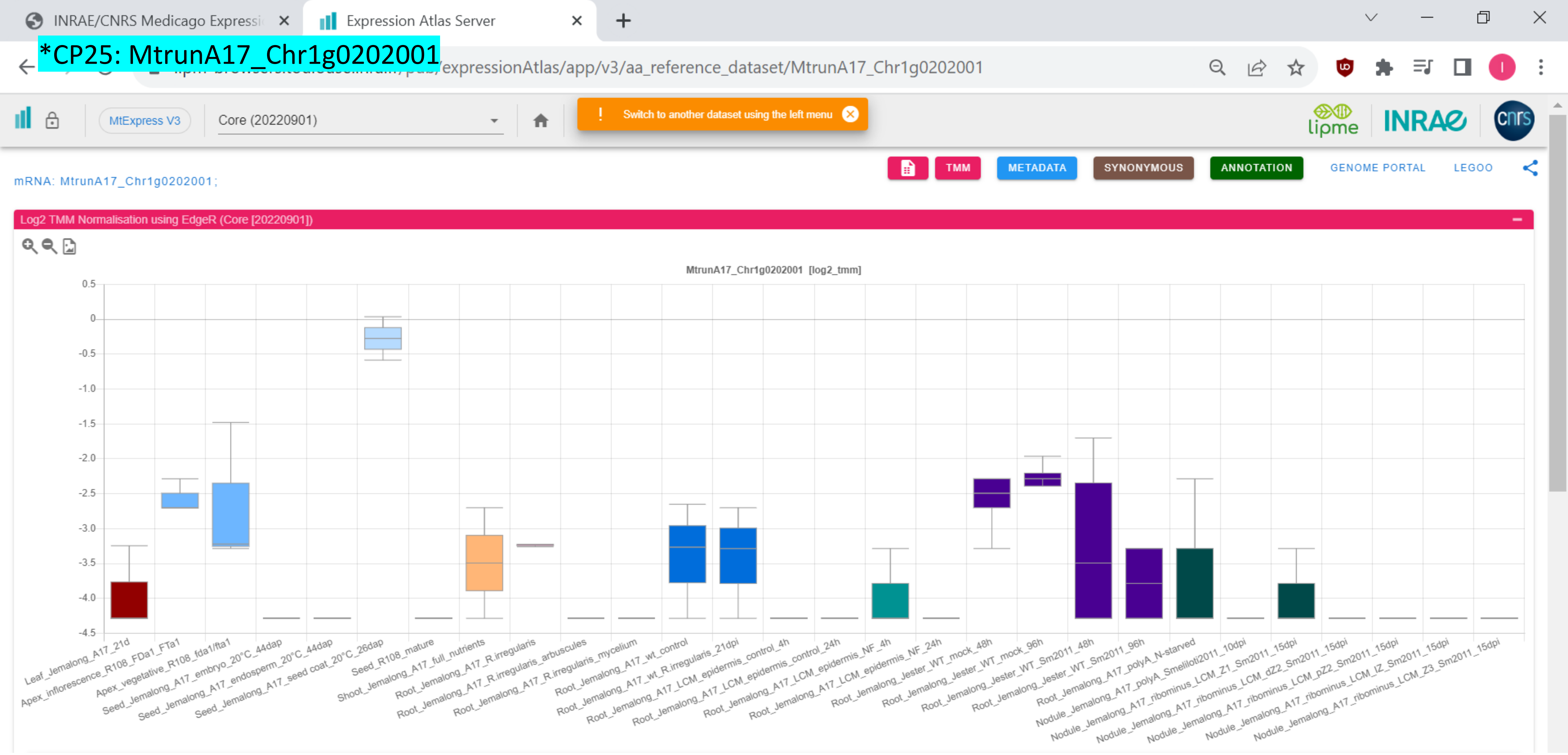

CP26: MtrunA17_Chr1g0205601

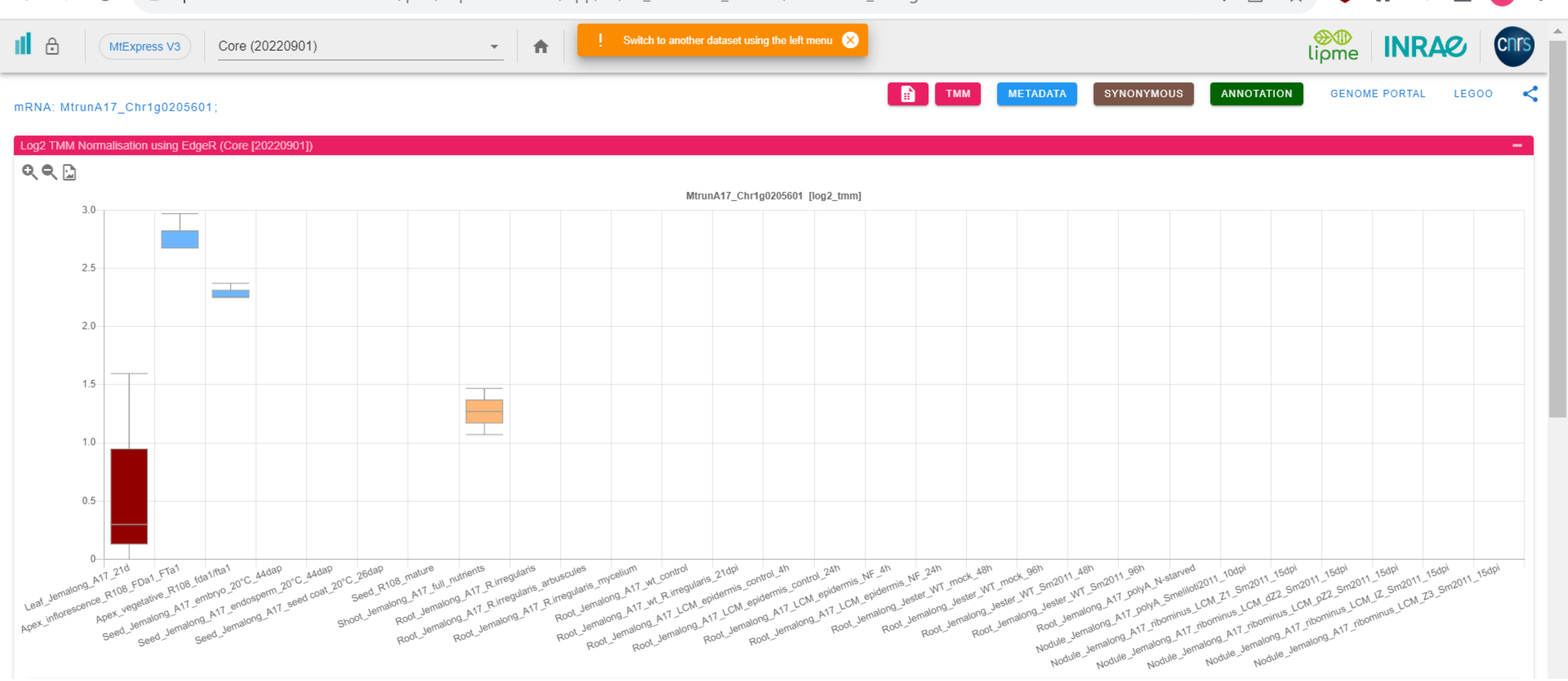

CP27: MtrunA17_Chr1g0207811

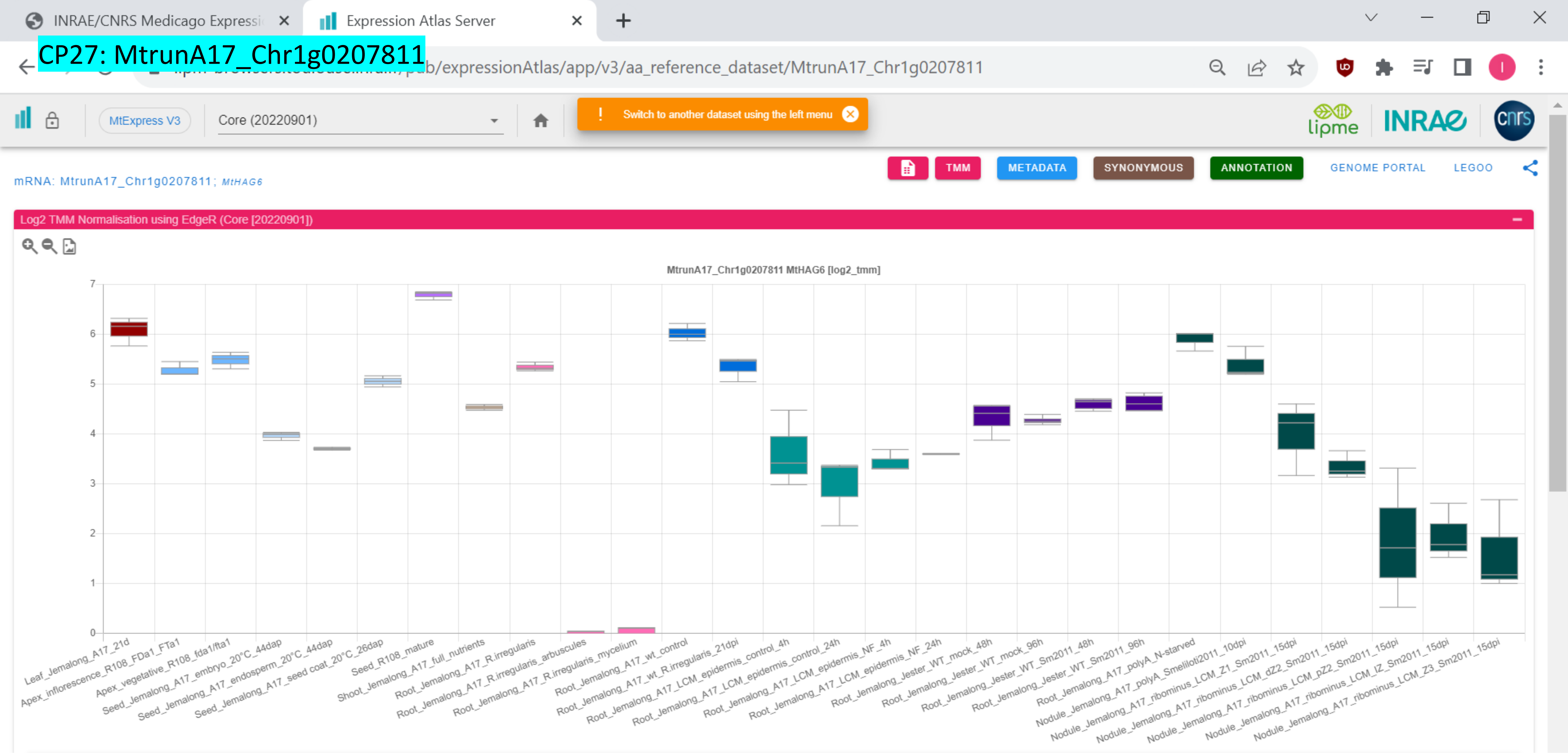

*CP28: MtrunA17_Chr1g0207921

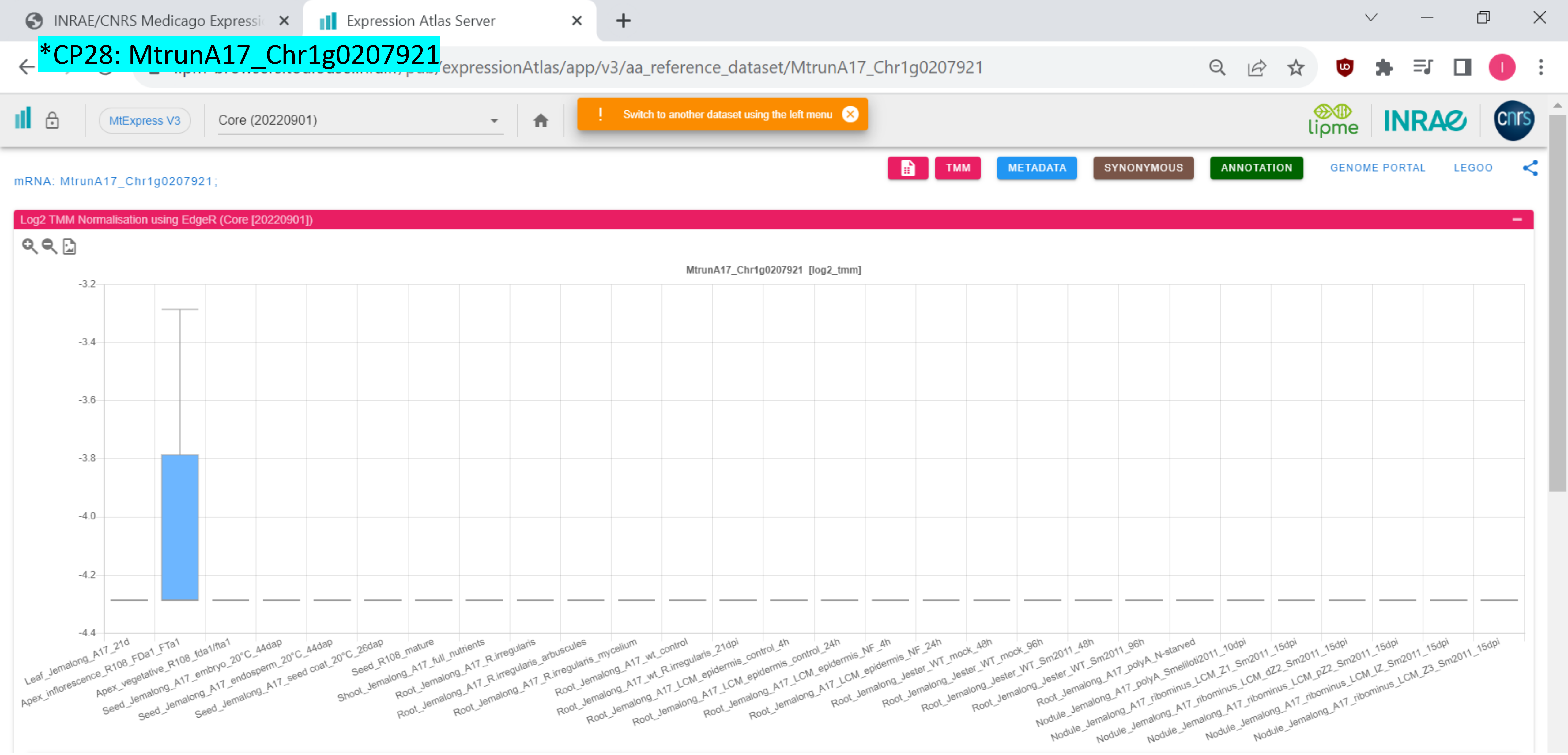

*CP29: MtrunA17_Chr1g0209791

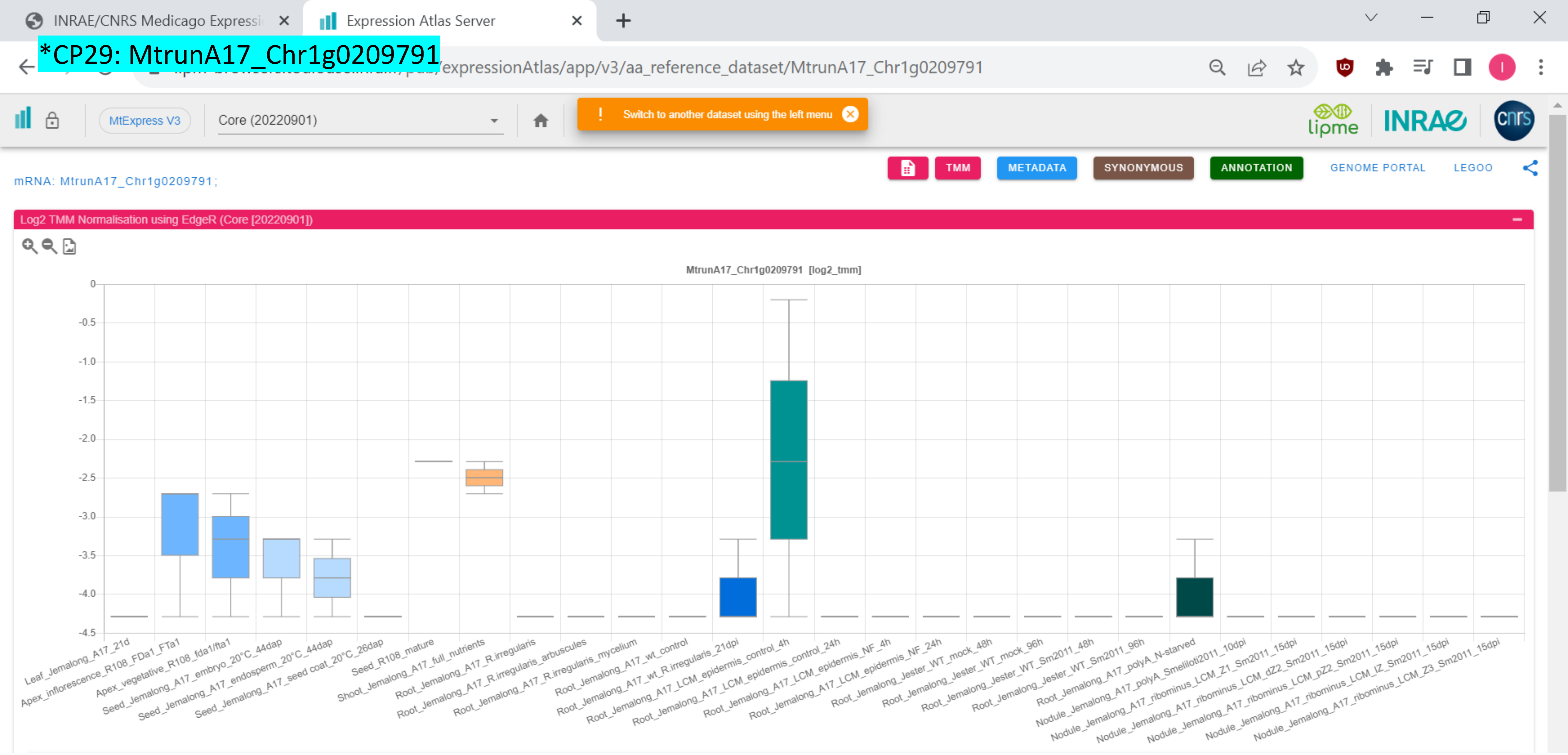

CP30: MtrunA17_Chr1g0210521

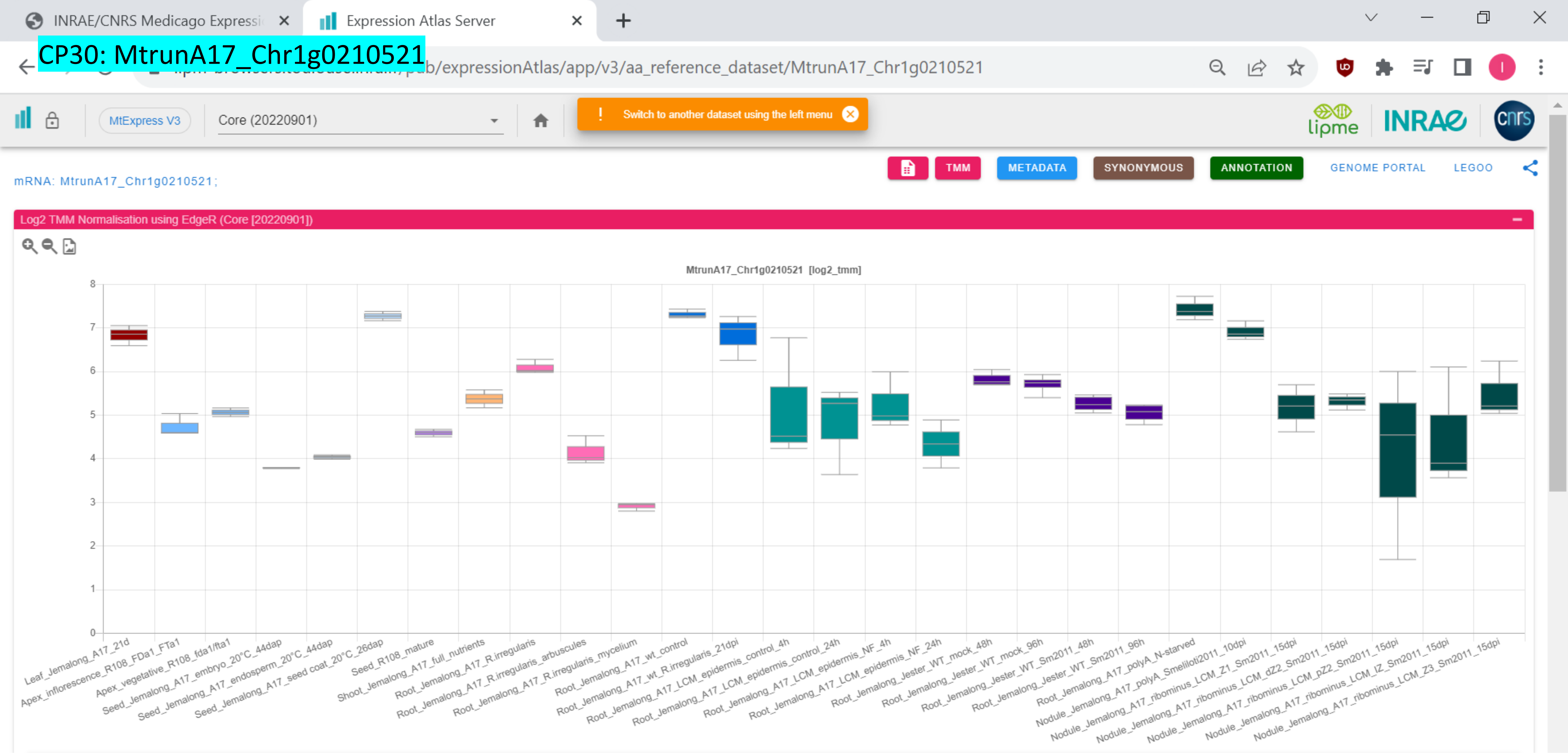

CP31: MtrunA17_Chr1g0212961

INRAE/CNRS Medicago Expressi | Expression Atlas Server

…pub/expressionAtlas/app/v3/aa_reference_dataset/MtrunA17_Chr1g0212961

MtExpress V3 | Core (20220901)

Switch to another dataset using the left menu

TMM | METADATA | SYNONYMOUS | ANNOTATION | GENOME PORTAL | LEGOO

mRNA: MtrunA17_Chr1g0212961;

Log2 TMM Normalisation using EdgeR (Core [20220901])

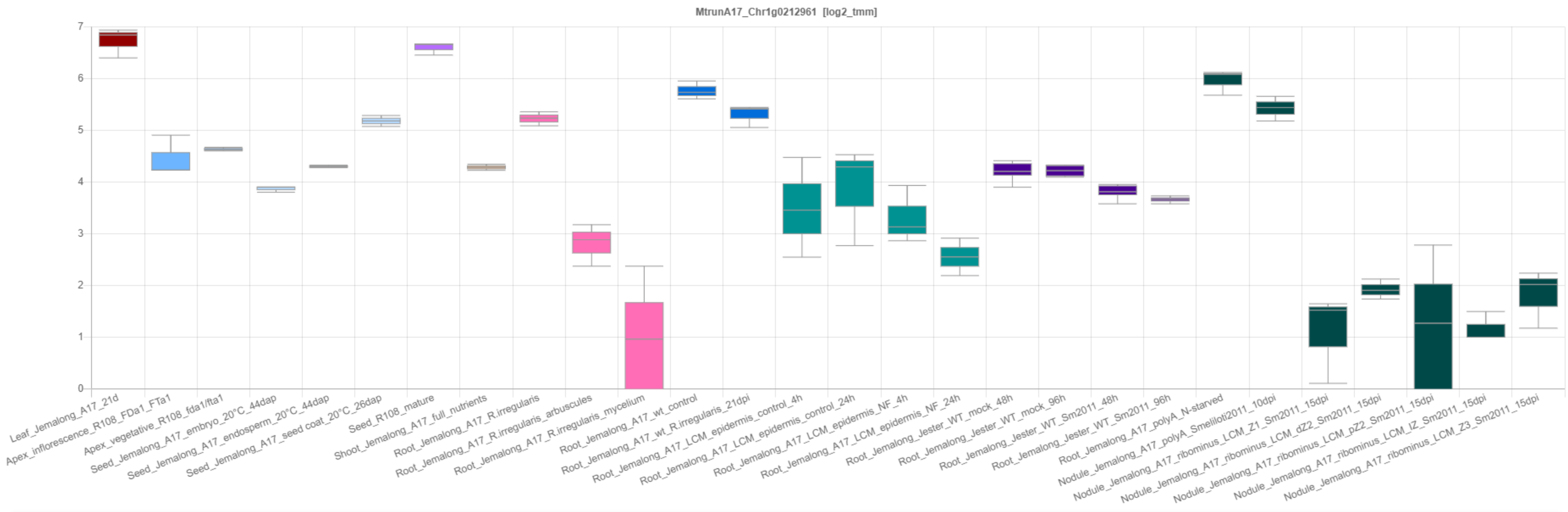

CP32: MtrunA17_Chr1g1004575

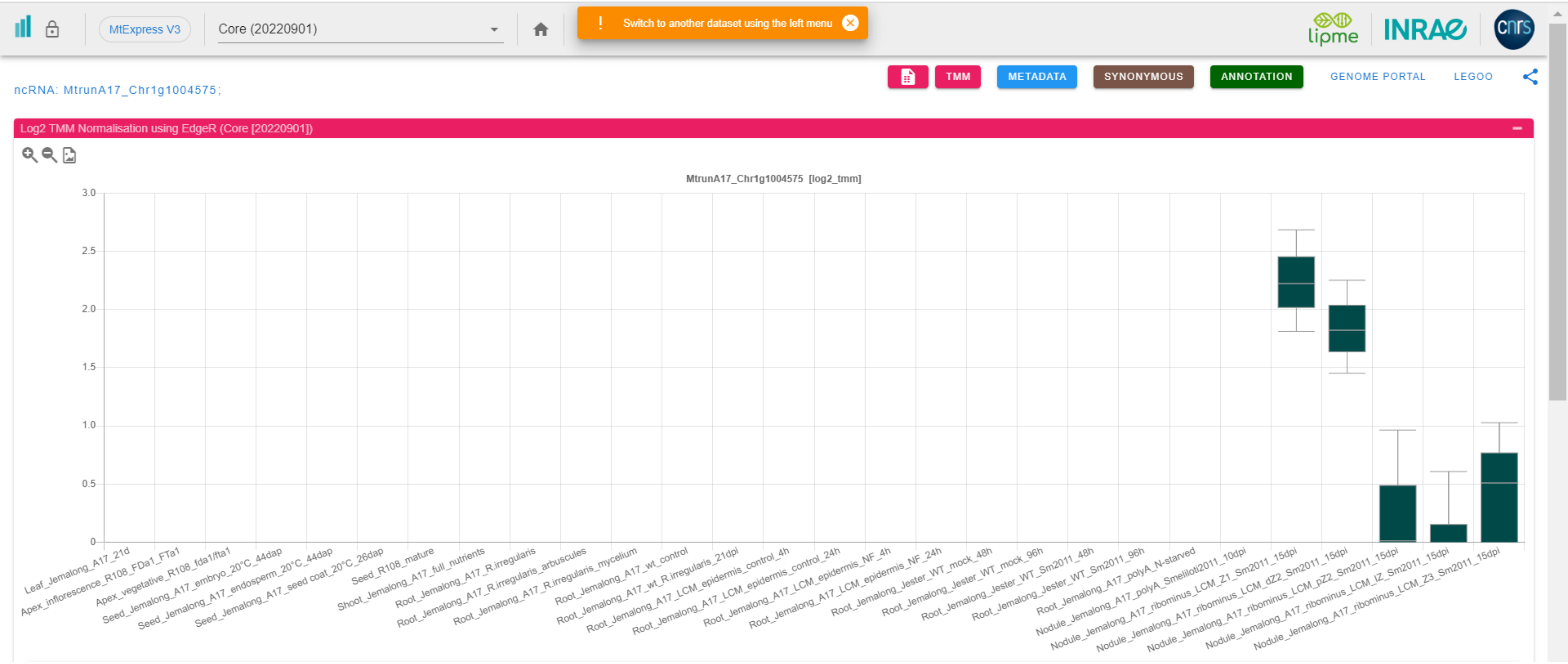

CP33: MtrunA17_Chr2g0283311

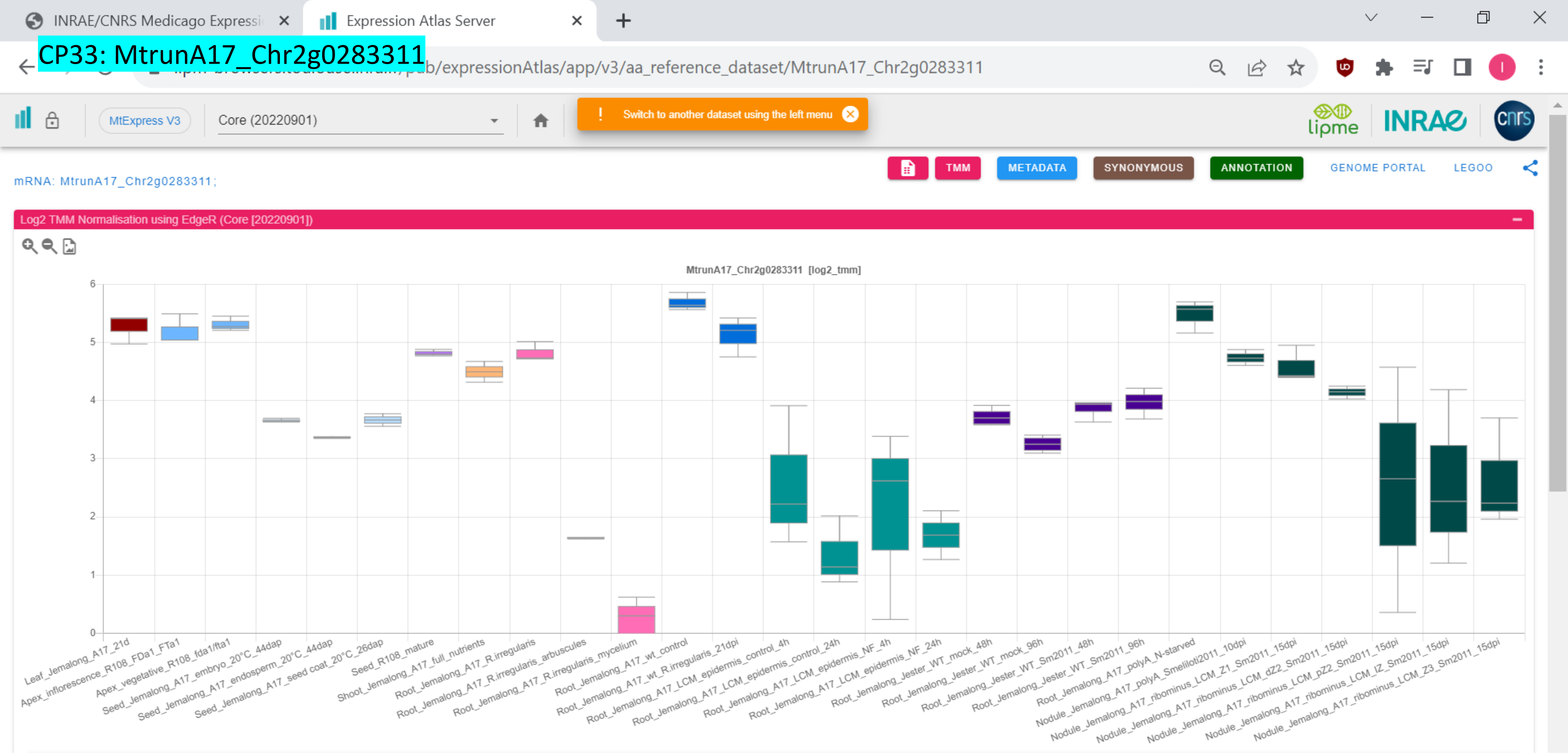

CP34: MtrunA17_Chr2g0285461

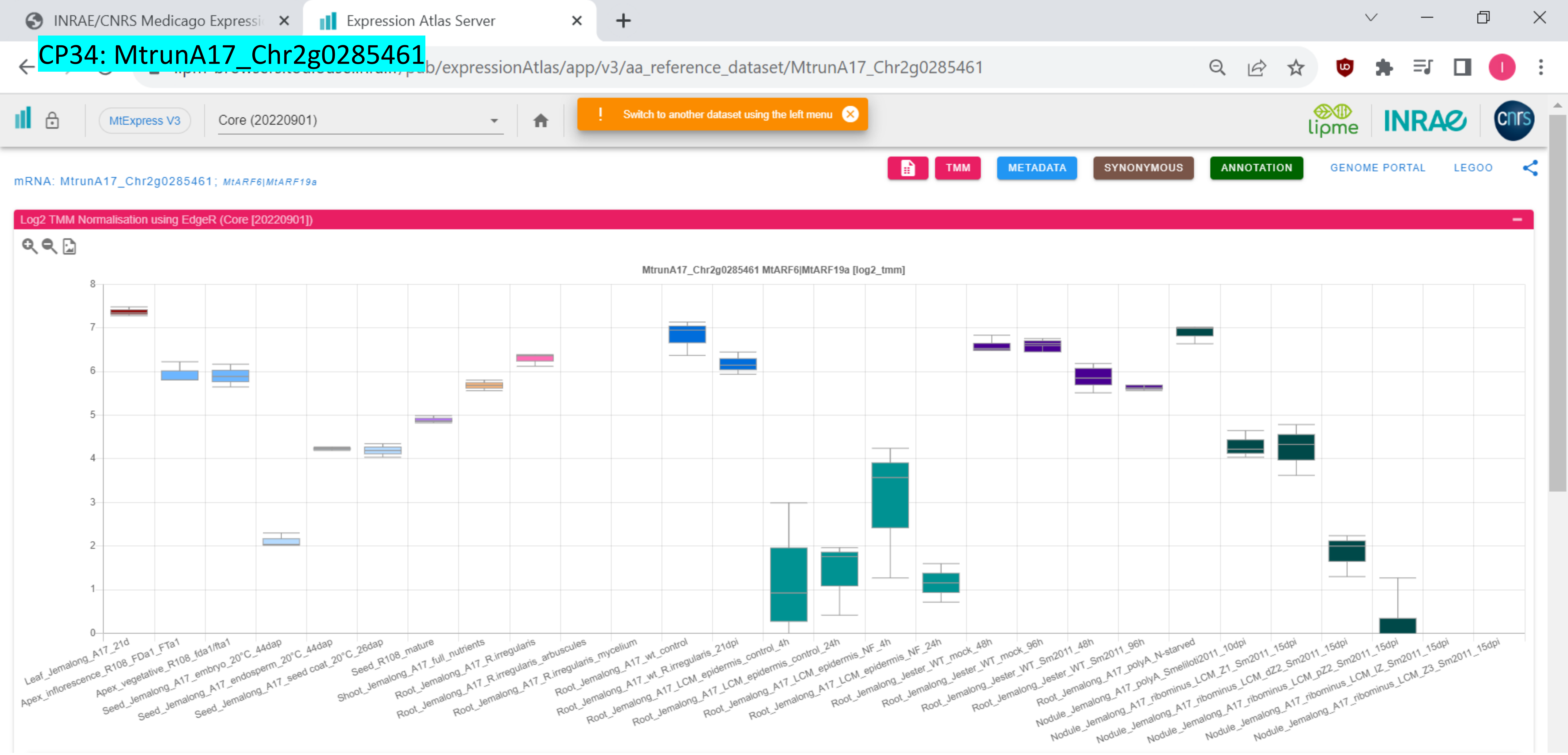

CP35: MtrunA17_Chr2g0292921

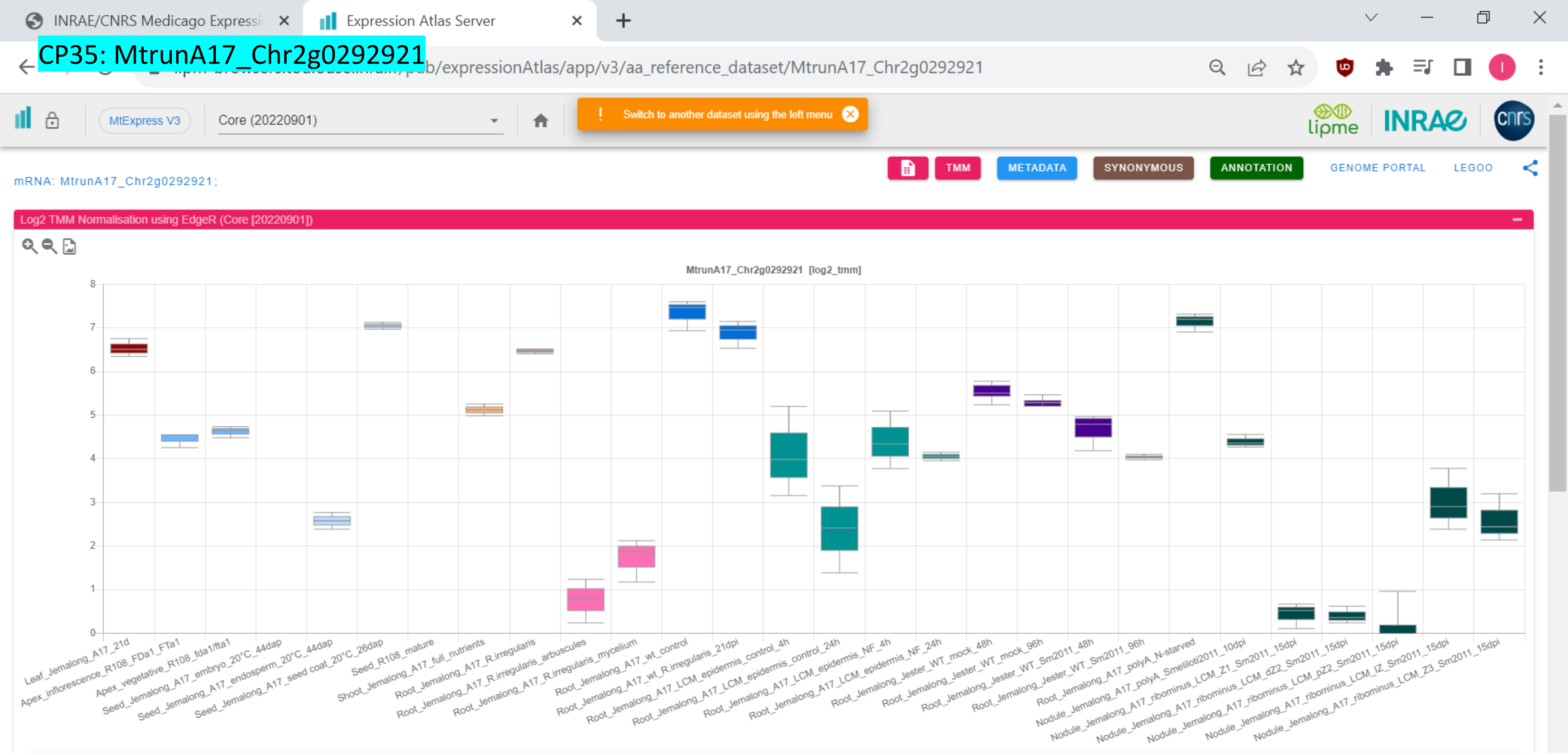

CP36: MtrunA17_Chr2g0298731

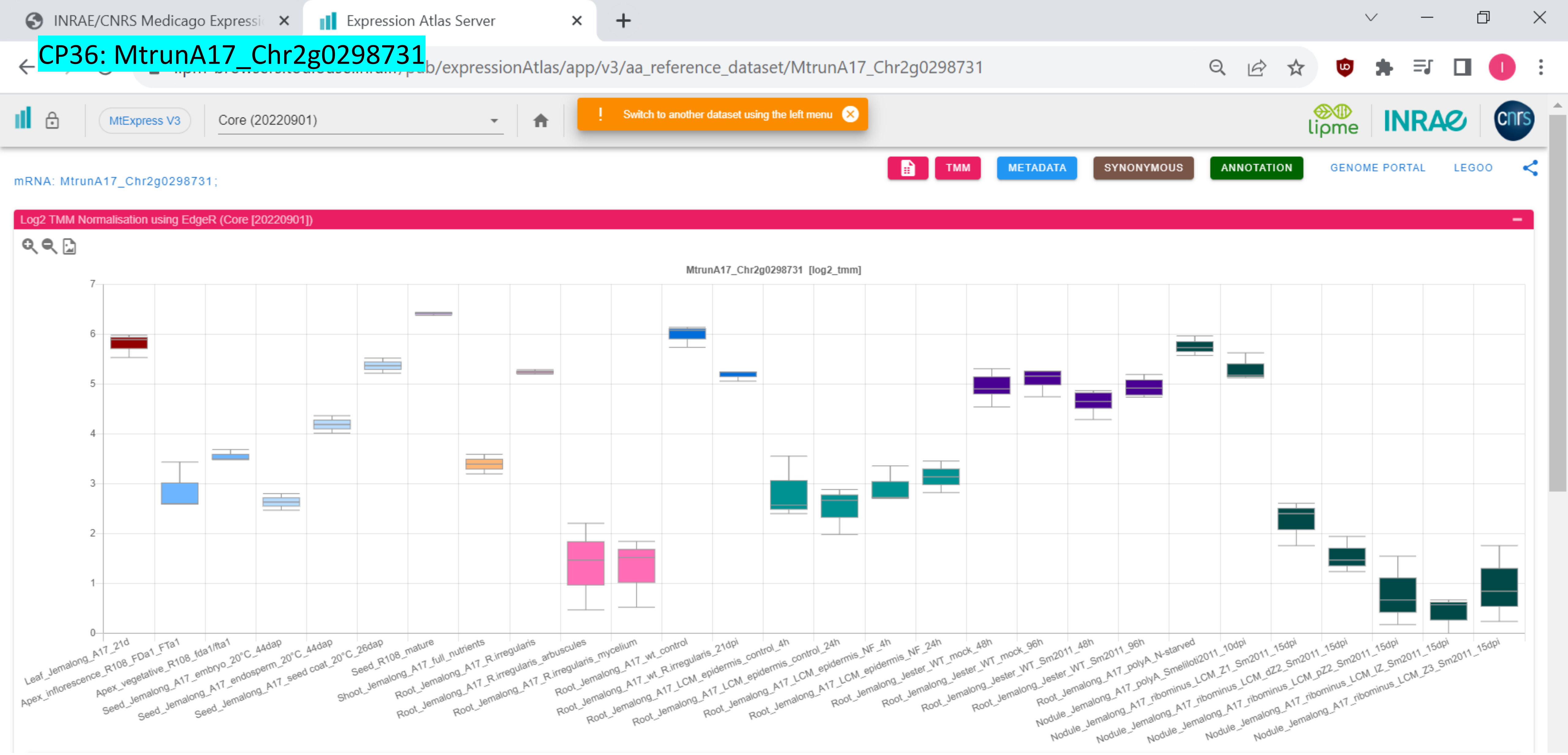

CP37: MtrunA17_Chr2g0299561

MtExpress V3 | Core (20220901)

Switch to another dataset using the left menu

TMM | METADATA | SYNONYMOUS | ANNOTATION | GENOME PORTAL | LEGOO

mRNA: MtrunA17_Chr2g0299561 ;

Log2 TMM Normalisation using EdgeR (Core [20220901])

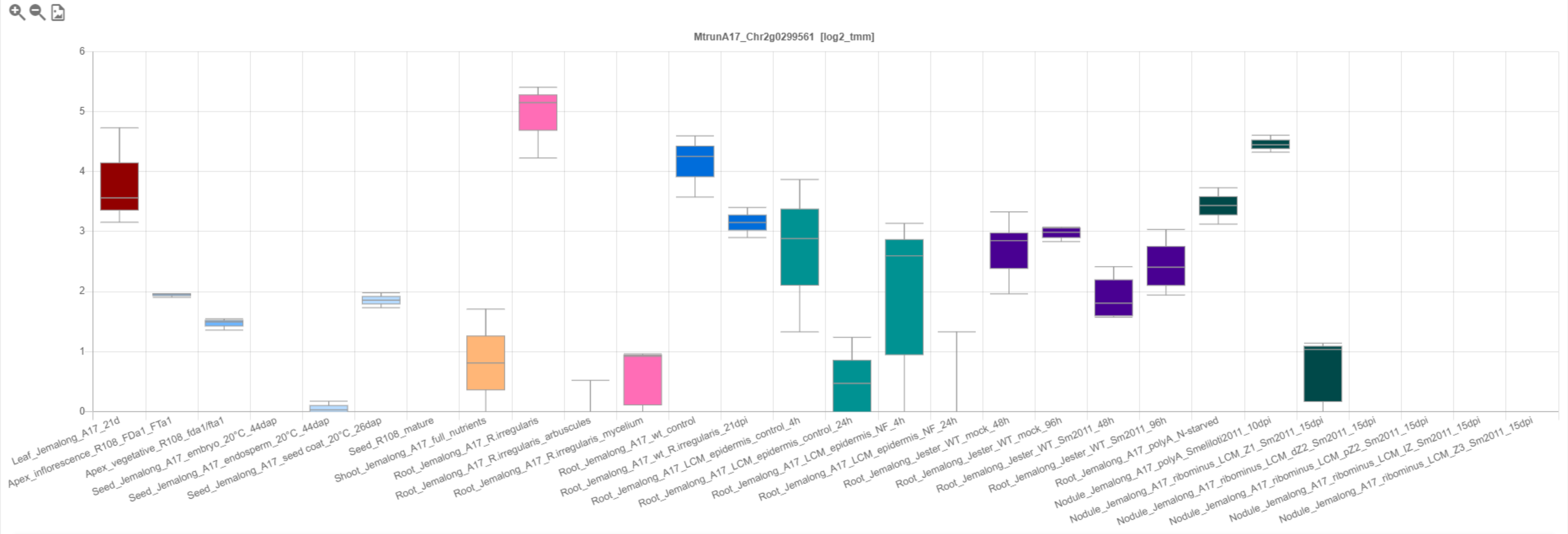

CP38: MtrunA17_Chr2g0304891

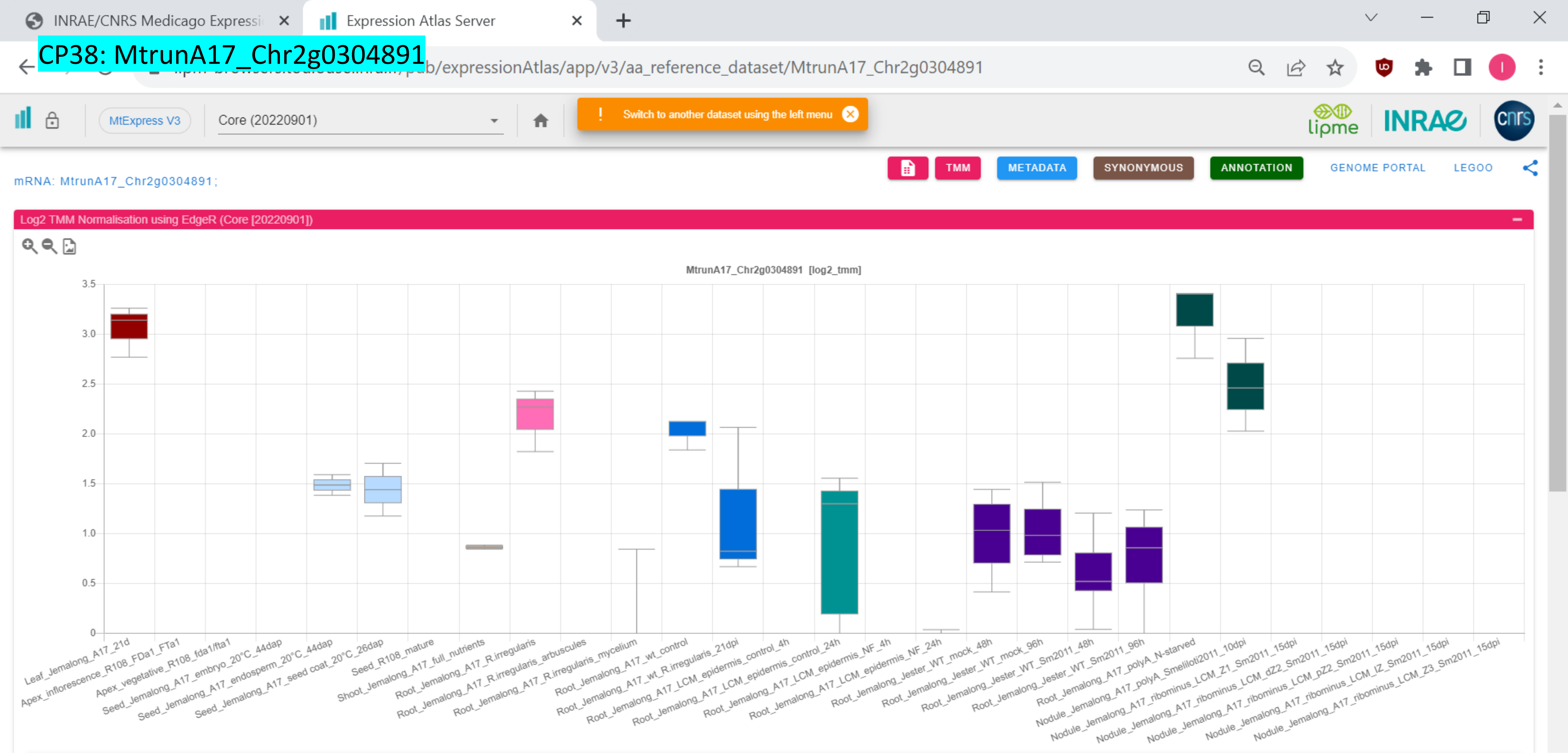

*CP39: MtrunA17_Chr2g0305951

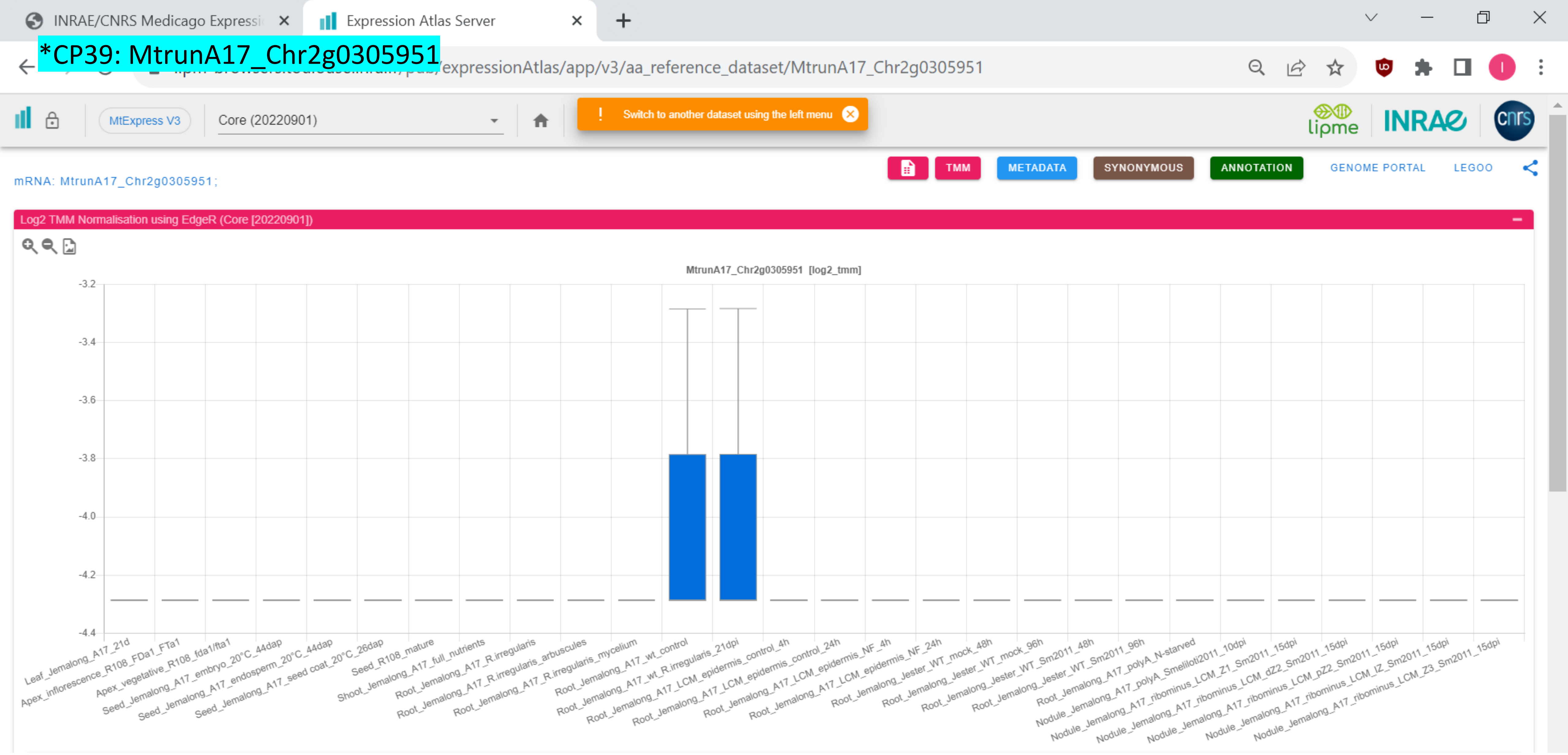

CP40: MtrunA17_Chr2g0309251

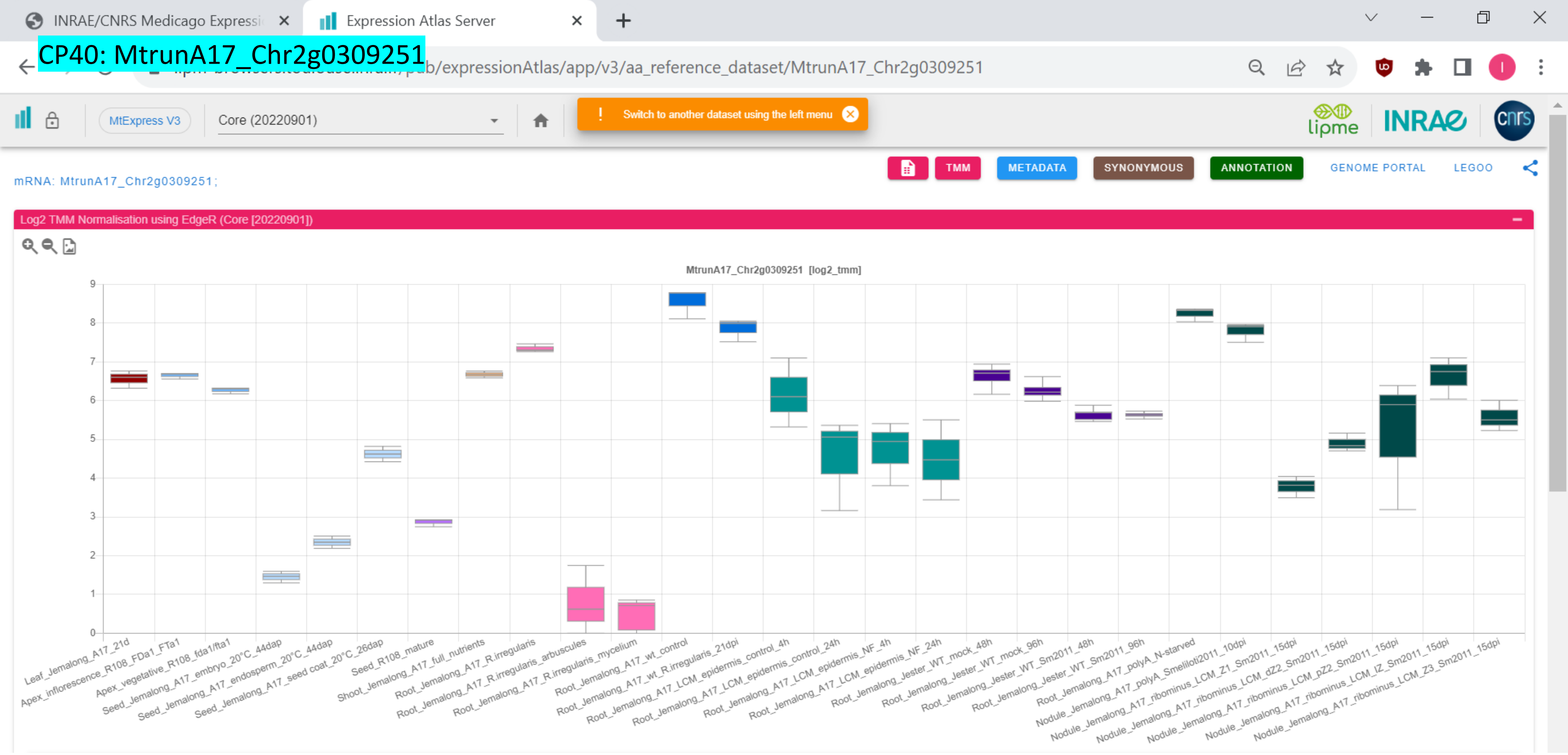

CP41: MtrunA17_Chr2g0310041

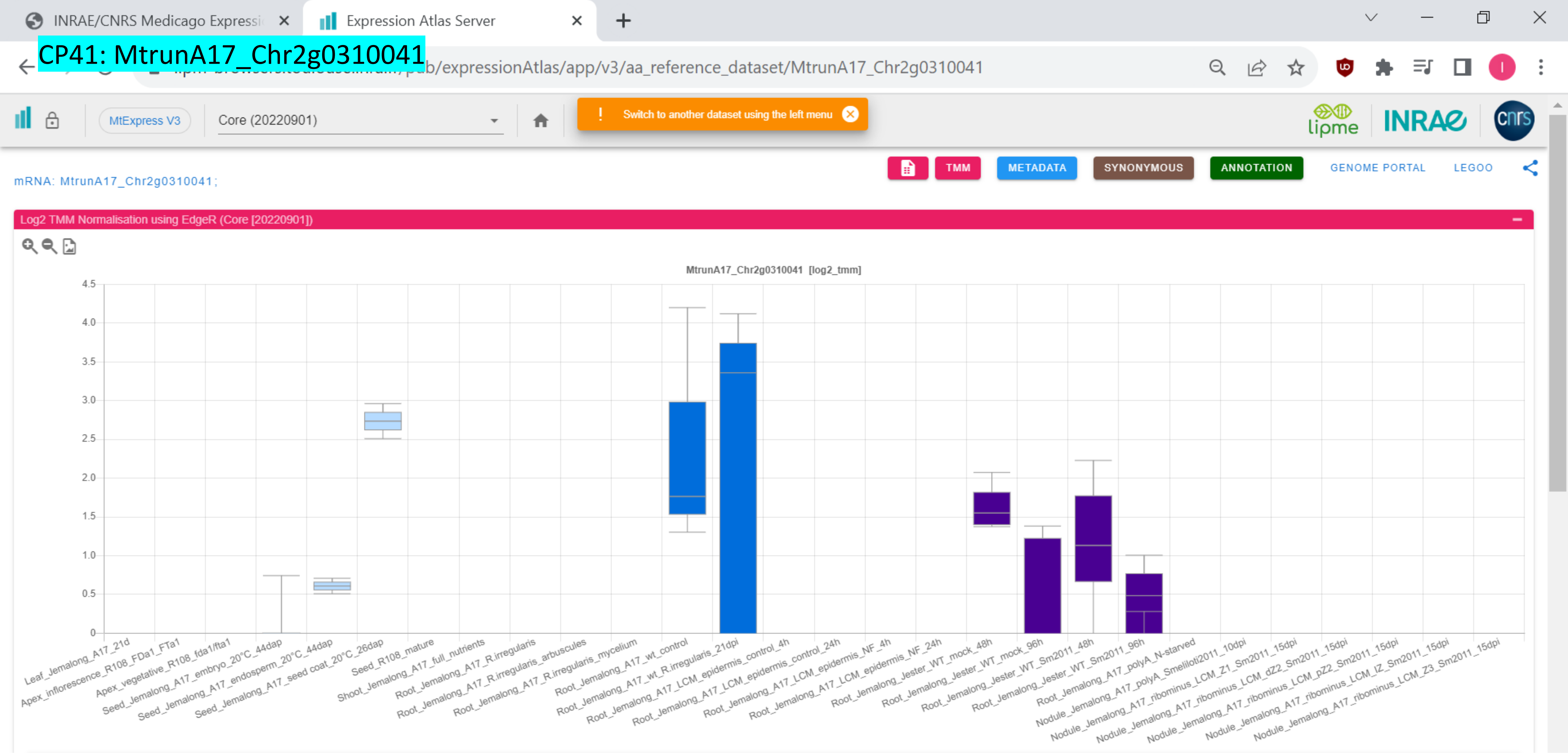

CP42: MtrunA17_Chr2g0312631

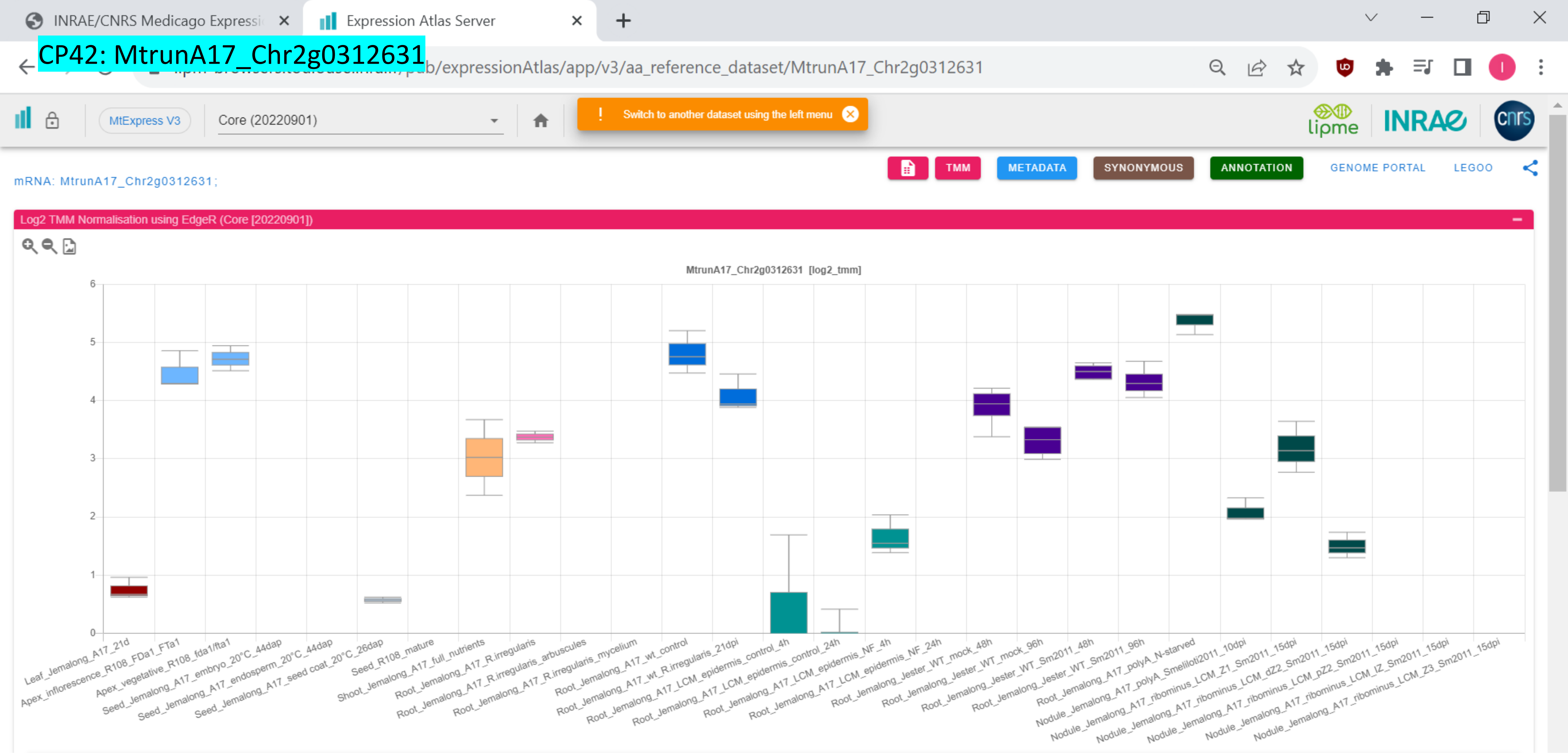

CP43: MtrunA17_Chr2g0316291

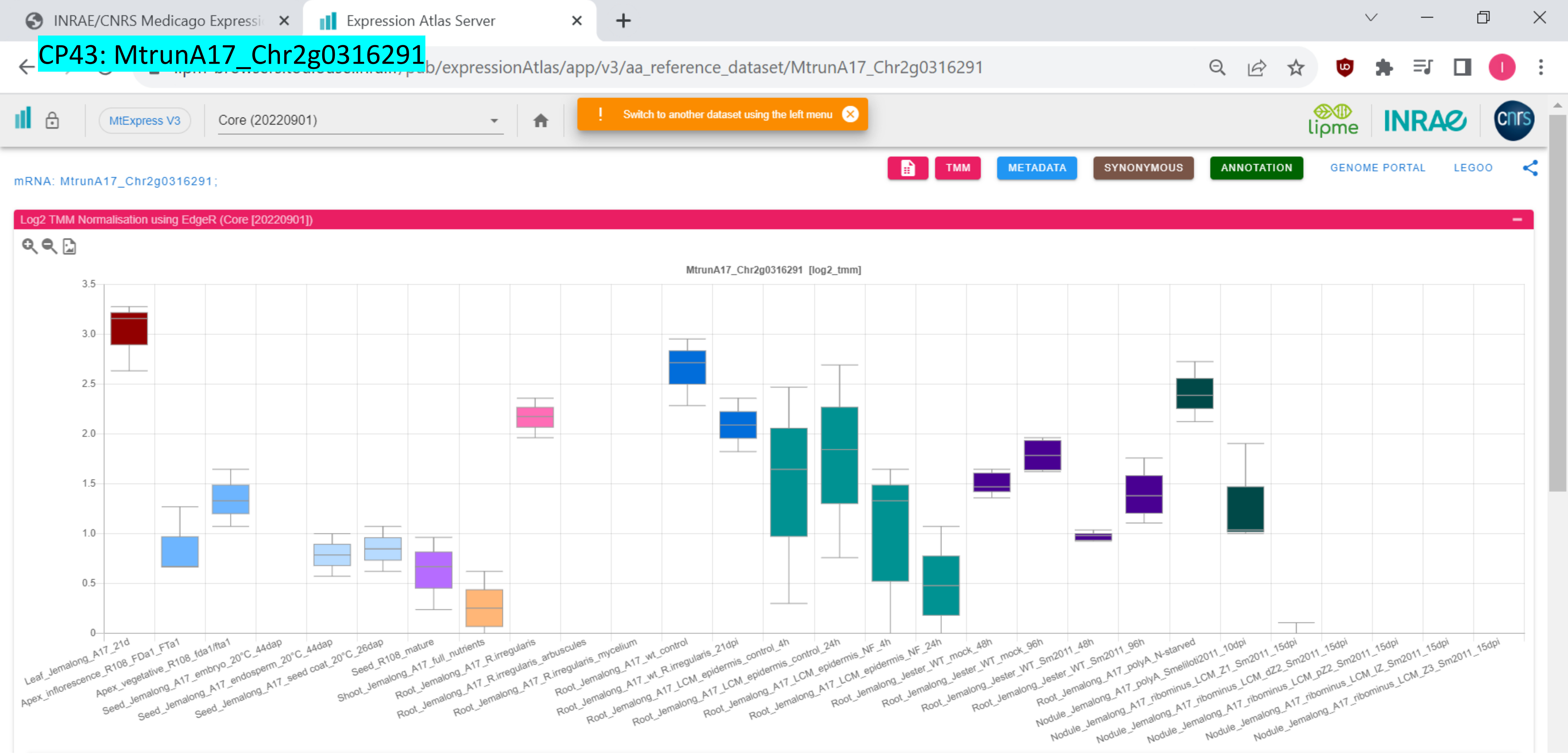

CP44: MtrunA17_Chr2g0326801

INRAE/CNRS Medicago Expressi | Expression Atlas Server

.../pub/expressionAtlas/app/v3/aa_reference_dataset/MtrunA17_Chr2g0326801

MtExpress V3 | Core (20220901)

! Switch to another dataset using the left menu

lipme INRAE cnrs

TMM | METADATA | SYNONYMOUS | ANNOTATION | GENOME PORTAL | LEGOO

mRNA: MtrunA17_Chr2g0326801;

Log2 TMM Normalisation using EdgeR (Core [20220901])

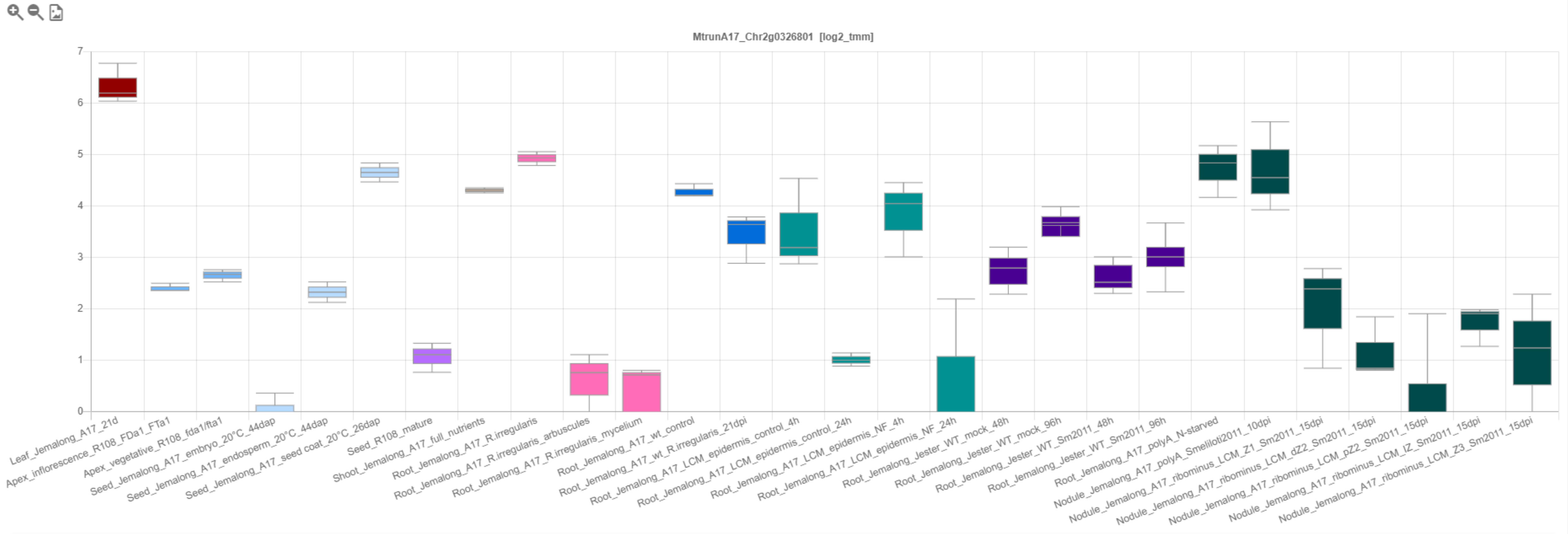

CP45: MtrunA17_Chr2g0328091

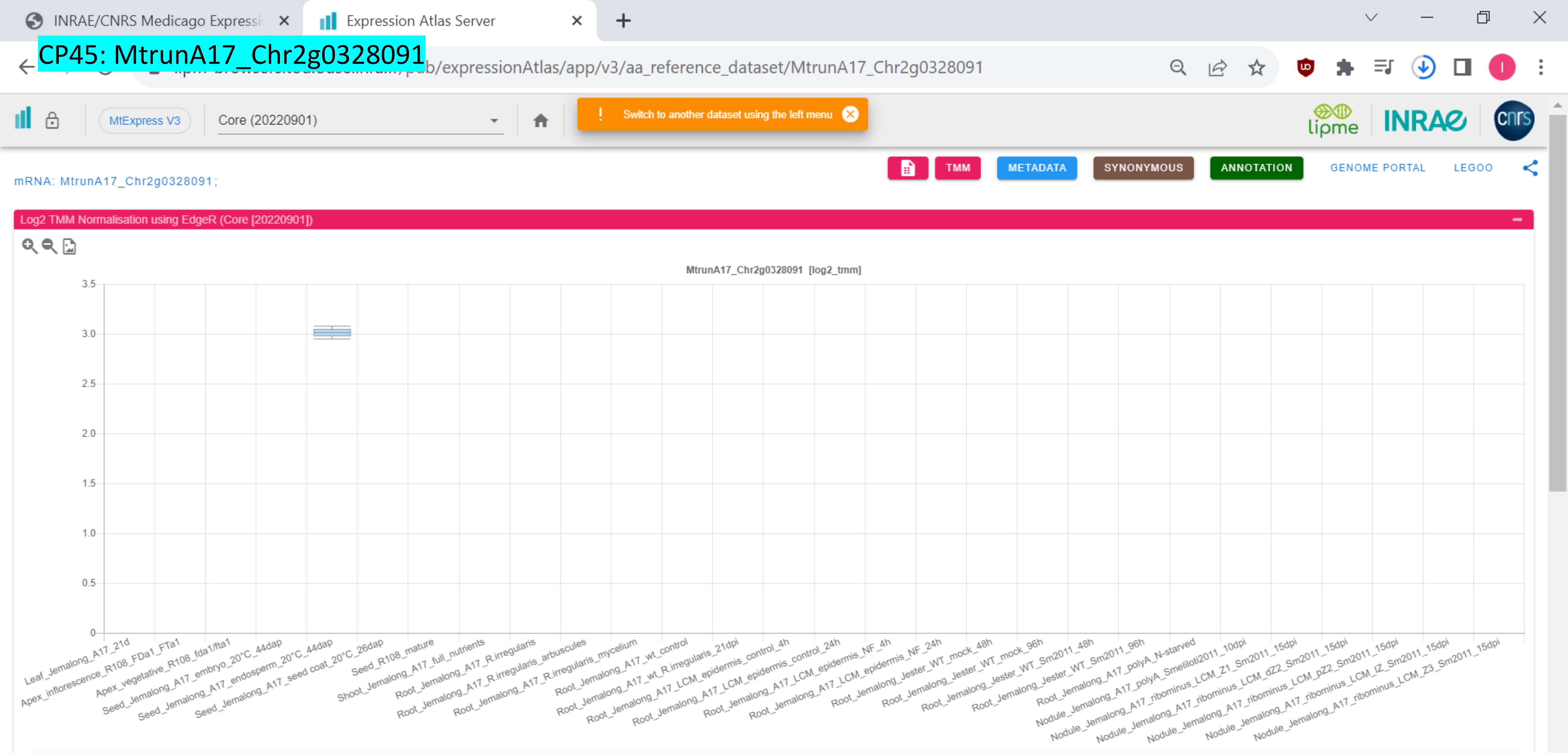

CP46: MtrunA17_Chr2g0329031

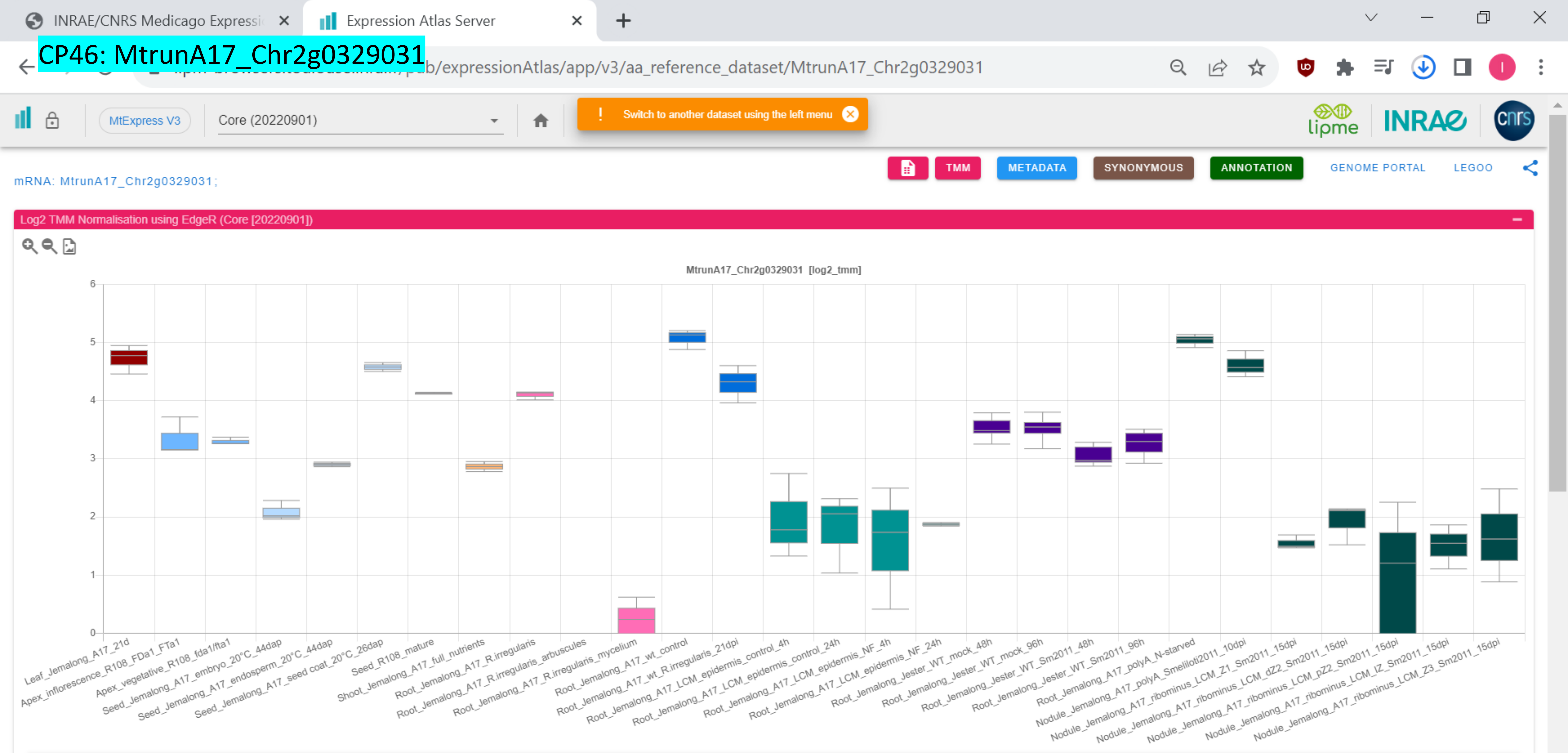

CP47: MtrunA17_Chr3g0079521

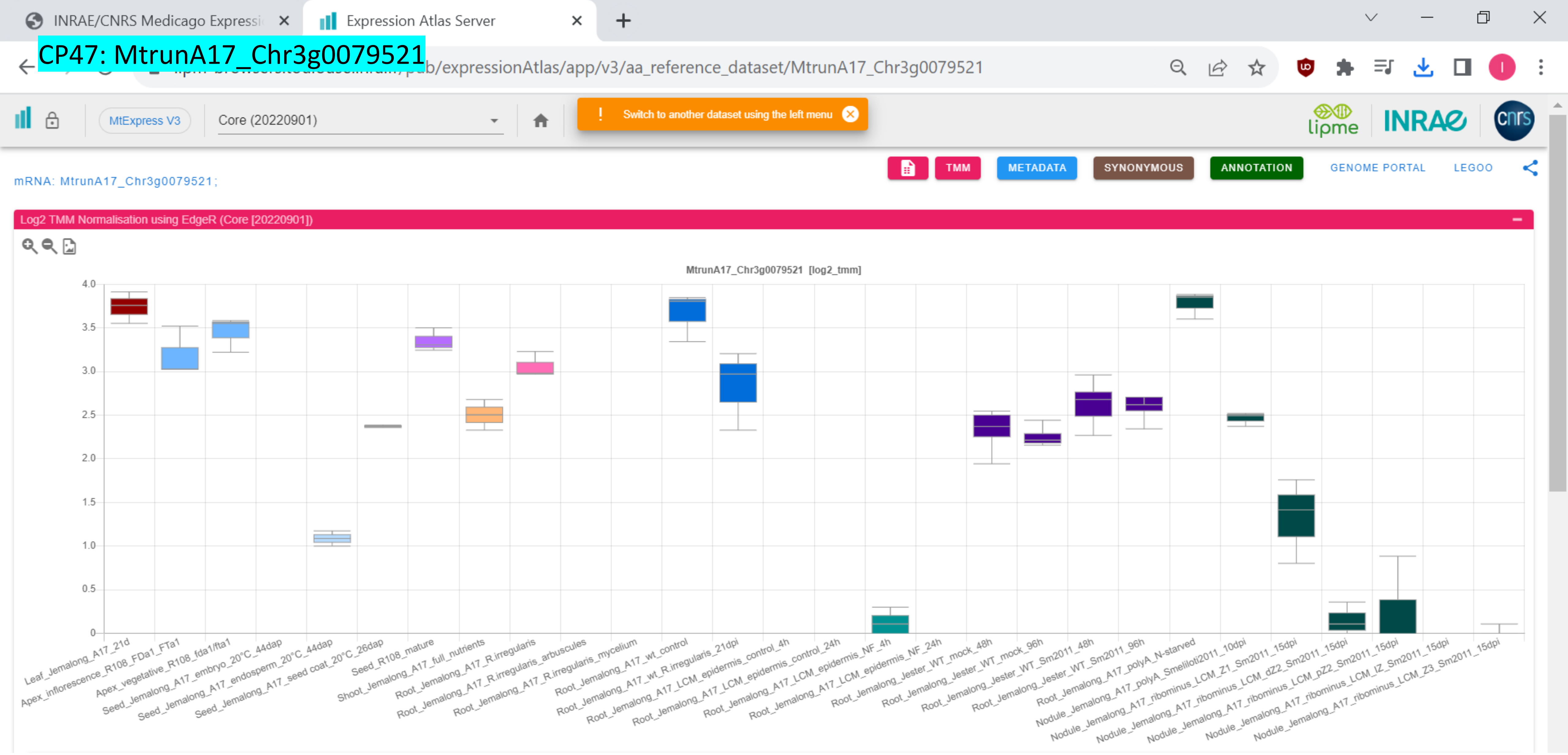

CP48: MtrunA17_Chr3g0083801

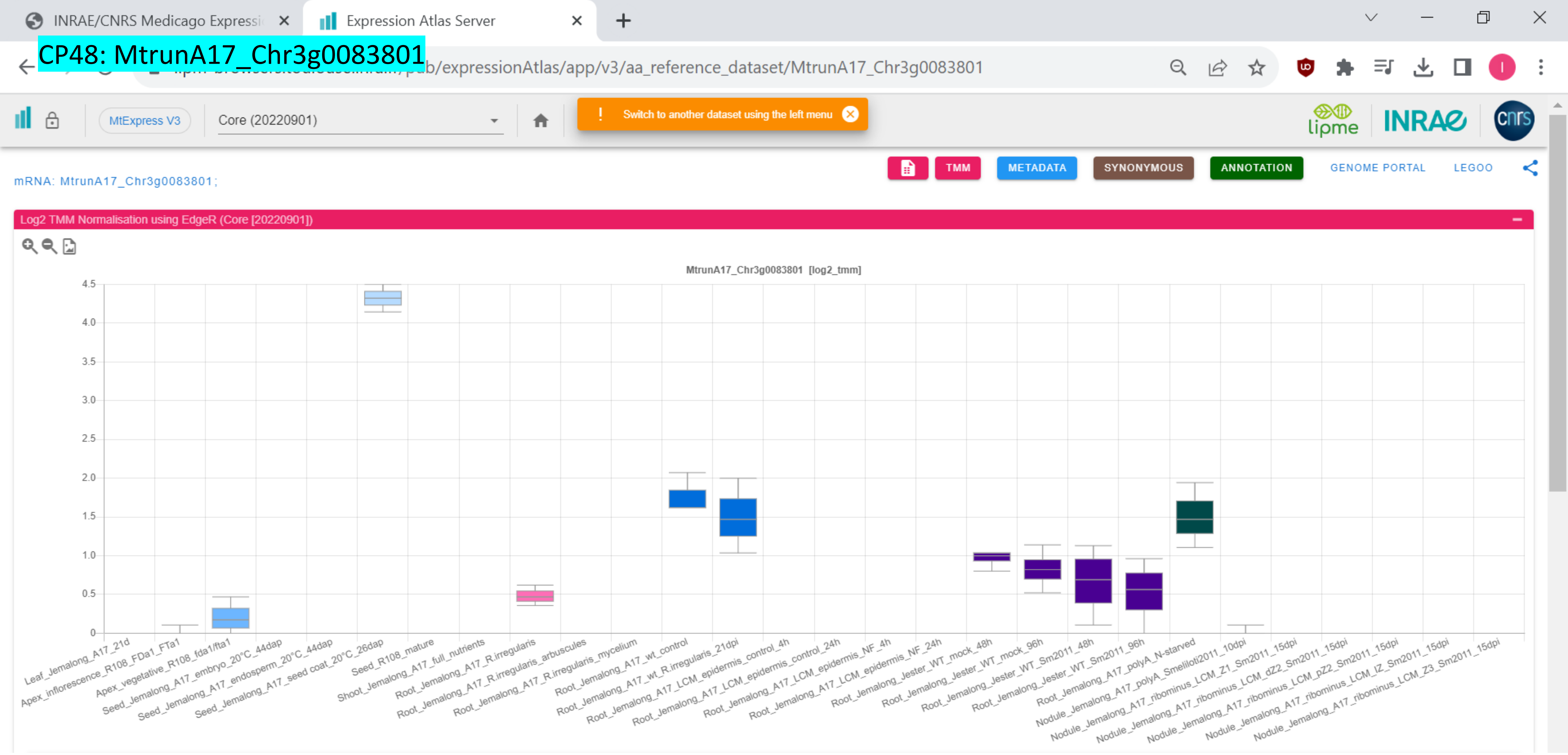

CP49: MtrunA17_Chr3g0091141

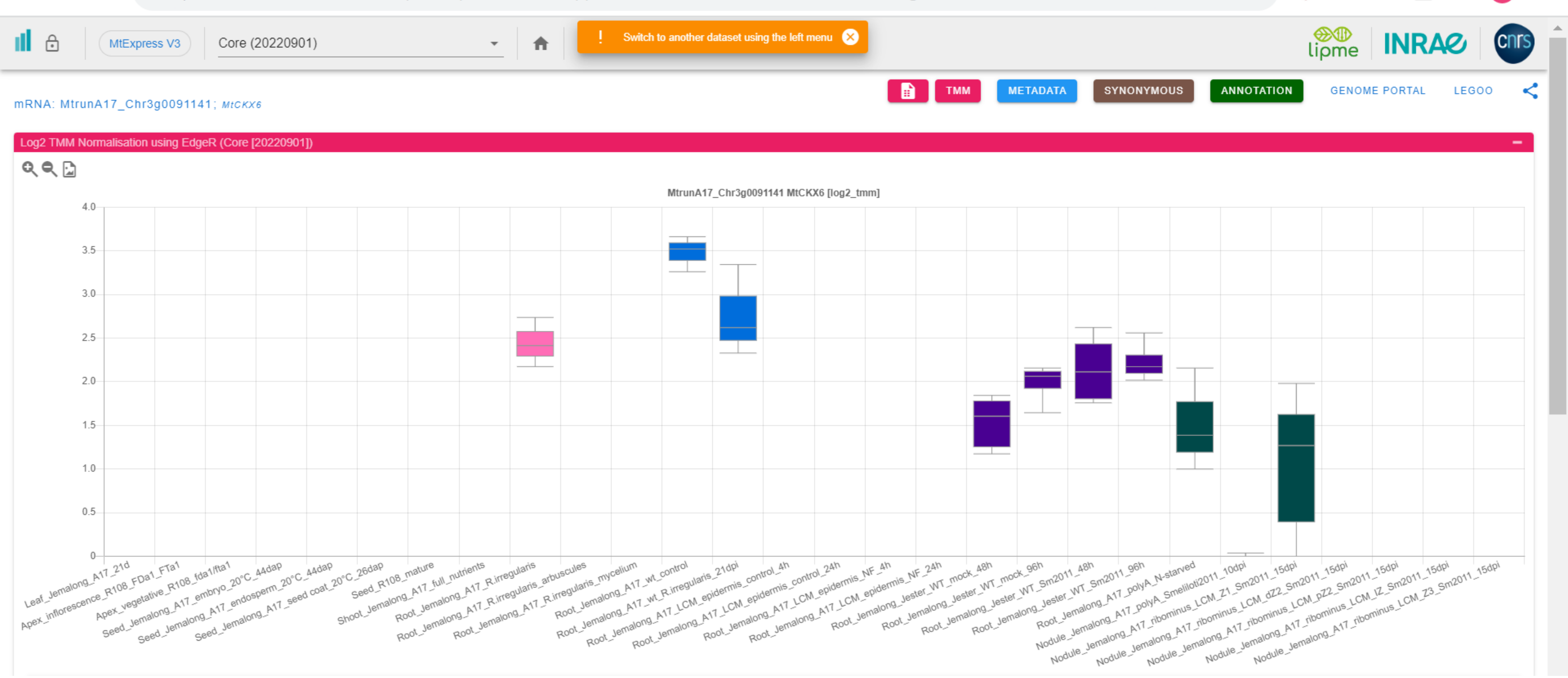

*CP50: MtrunA17_Chr3g0091671

INRAE/CNRS Medicago Expressi… | Expression Atlas Server

…/expressionAtlas/app/v3/aa_reference_dataset/MtrunA17_Chr3g0091671

MtExpress V3 | Core (20220901)

! Switch to another dataset using the left menu

TMM | METADATA | SYNONYMOUS | ANNOTATION | GENOME PORTAL | LEGOO

mRNA: MtrunA17_Chr3g0091671;

Log2 TMM Normalisation using EdgeR (Core [20220901])

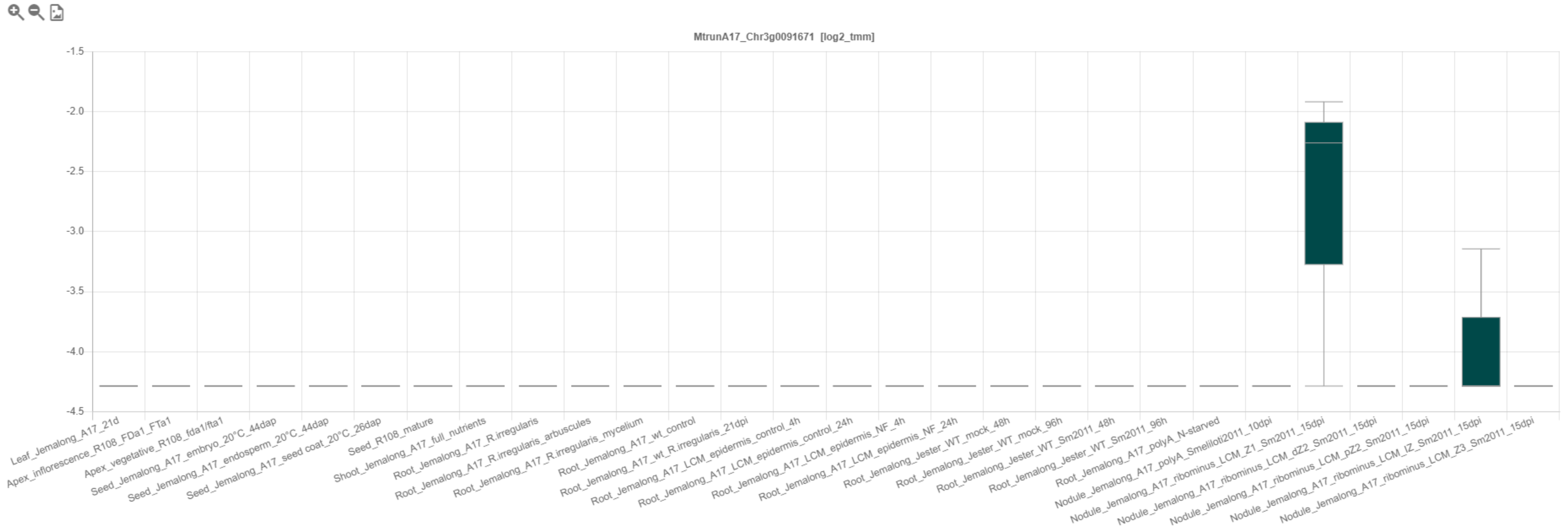

CP51: MtrunA17_Chr3g0096421

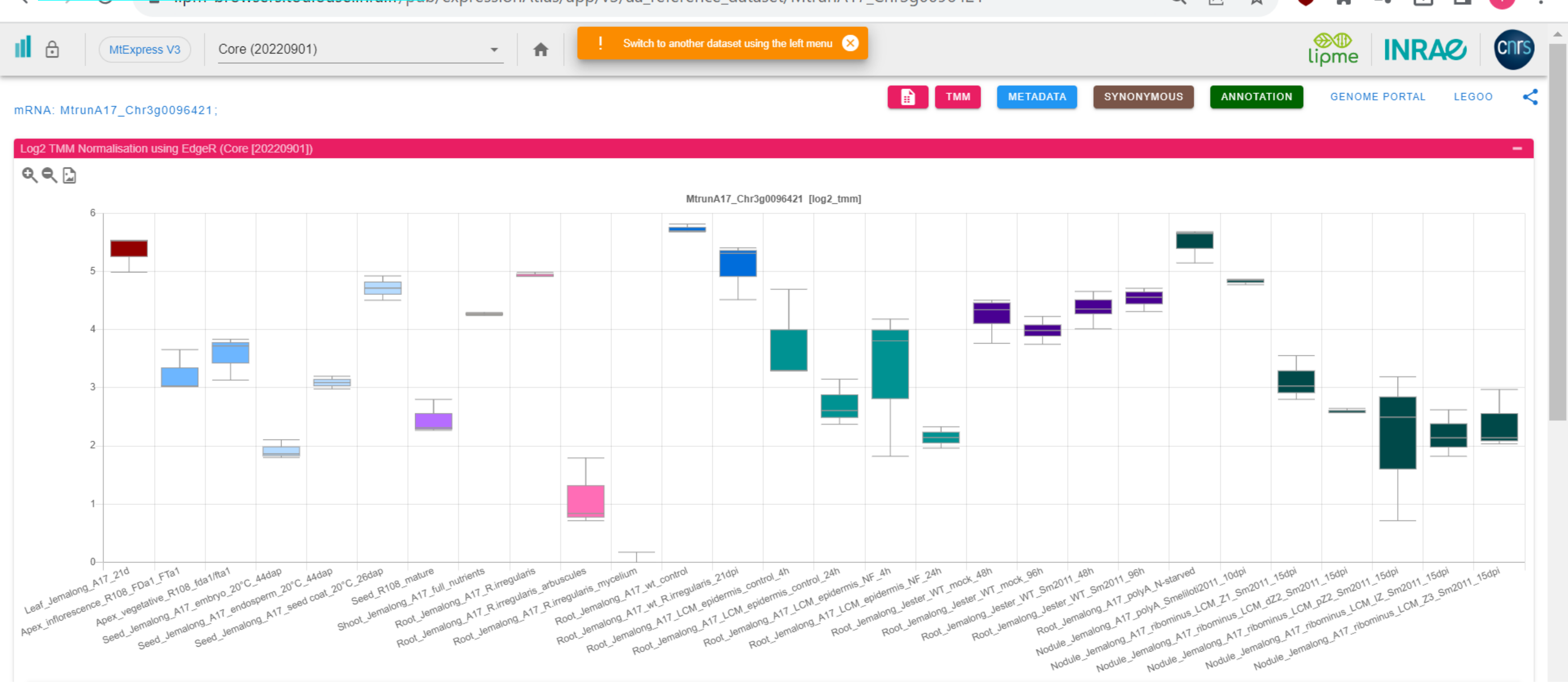

*CP52: MtrunA17_Chr3g0100221

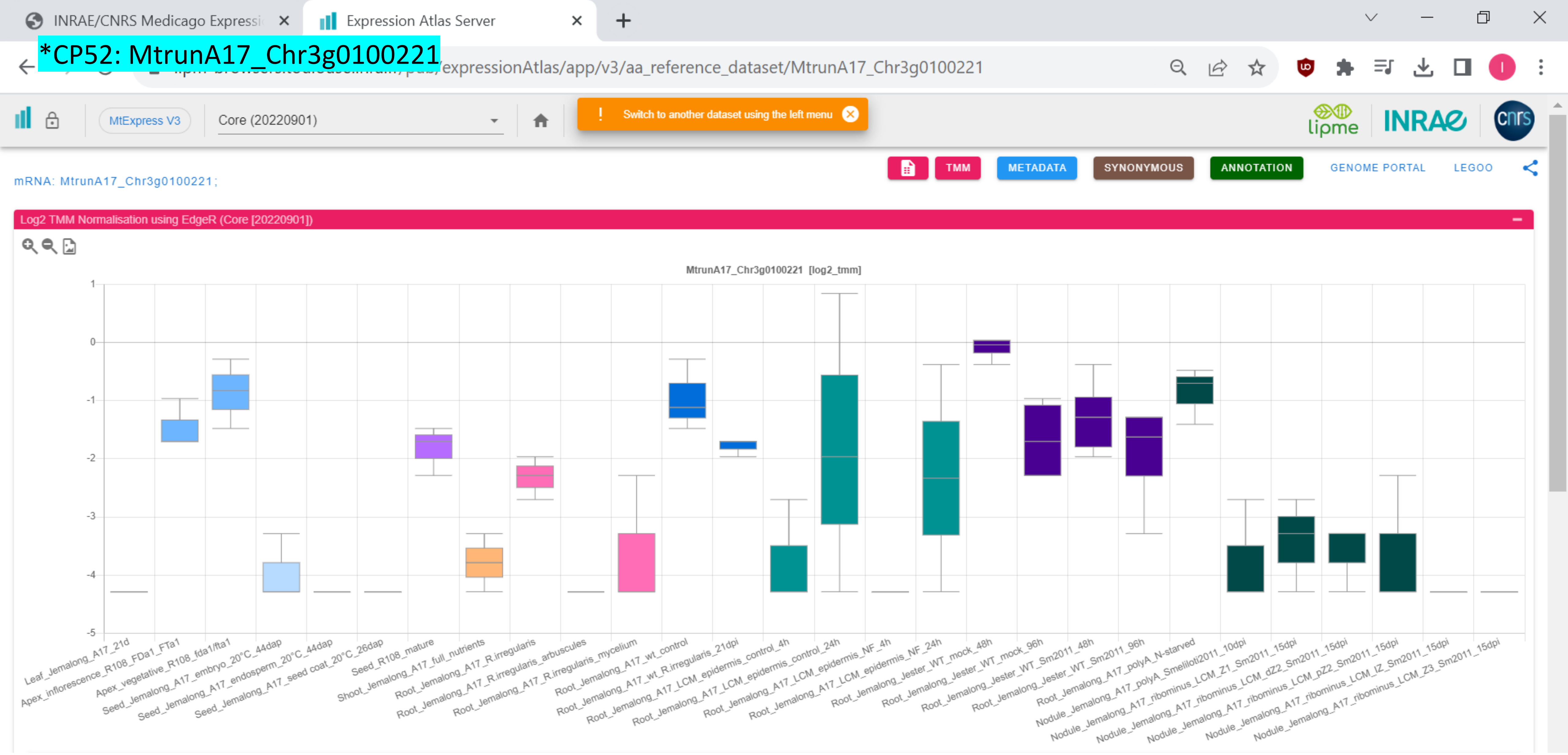

CP53: MtrunA17_Chr3g0102171

INRAE/CNRS Medicago Expressi

Expression Atlas Server

[illegible]/pub/expressionAtlas/app/v3/aa_reference_dataset/MtrunA17_Chr3g0102171

MtExpress V3

Core (20220901)

! Switch to another dataset using the left menu

lipme INRAE cnrs

TMM METADATA SYNONYMOUS ANNOTATION GENOME PORTAL LEGOO

mRNA: MtrunA17_Chr3g0102171;

Log2 TMM Normalisation using EdgeR (Core [20220901])

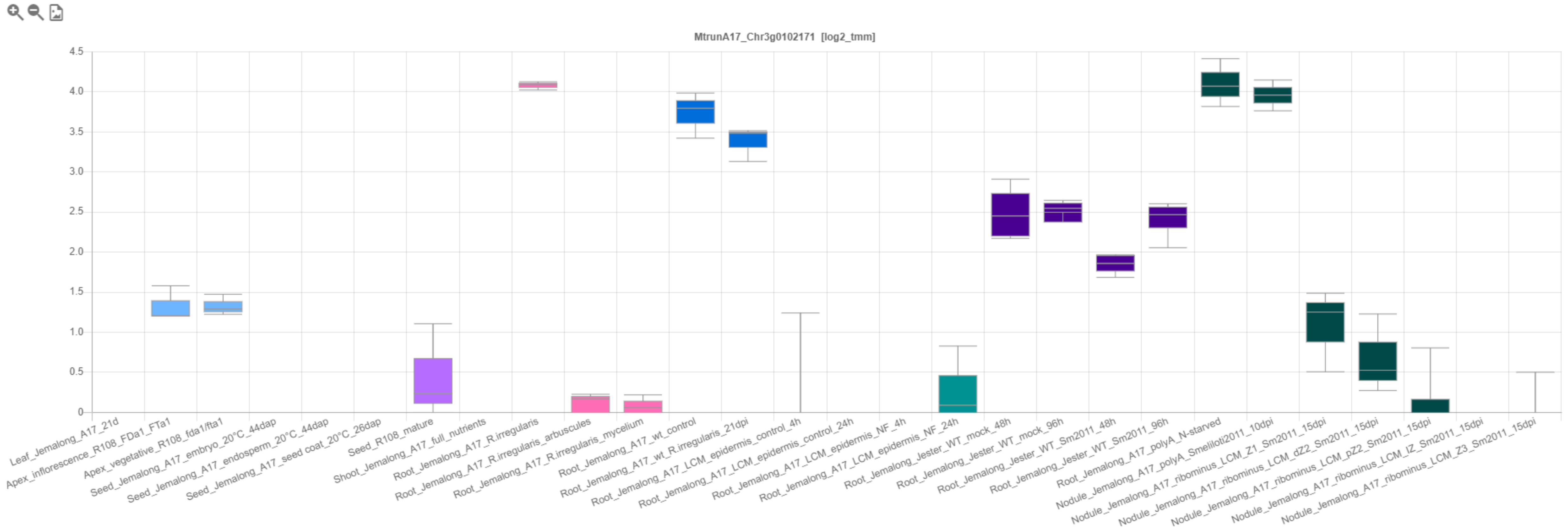

CP54: MtrunA17_Chr3g0105981

INRAE/CNRS Medicago Expressi | Expression Atlas Server

…pub/expressionAtlas/app/v3/aa_reference_dataset/MtrunA17_Chr3g0105981

MtExpress V3 | Core (20220901) | Switch to another dataset using the left menu

TMM | METADATA | SYNONYMOUS | ANNOTATION | GENOME PORTAL | LEGOO

mRNA: MtrunA17_Chr3g0105981;

Log2 TMM Normalisation using EdgeR (Core [20220901])

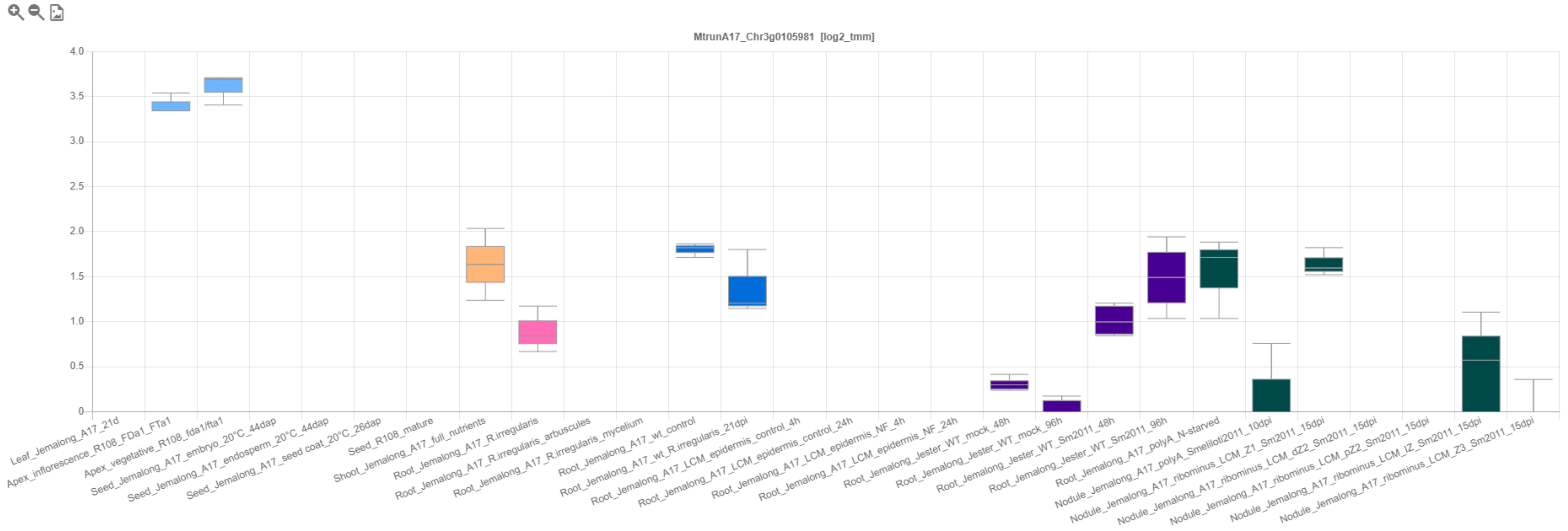

CP55: MtrunA17_Chr3g0110451

Expression Atlas Server

.../pub/expressionAtlas/app/v3/aa_reference_dataset/MtrunA17_Chr3g0110451

MtExpress V3 | Core (20220901)

! Switch to another dataset using the left menu

TMM | METADATA | SYNONYMOUS | ANNOTATION | GENOME PORTAL | LEGOO

mRNA: MtrunA17_Chr3g0110451;

Log2 TMM Normalisation using EdgeR (Core [20220901])

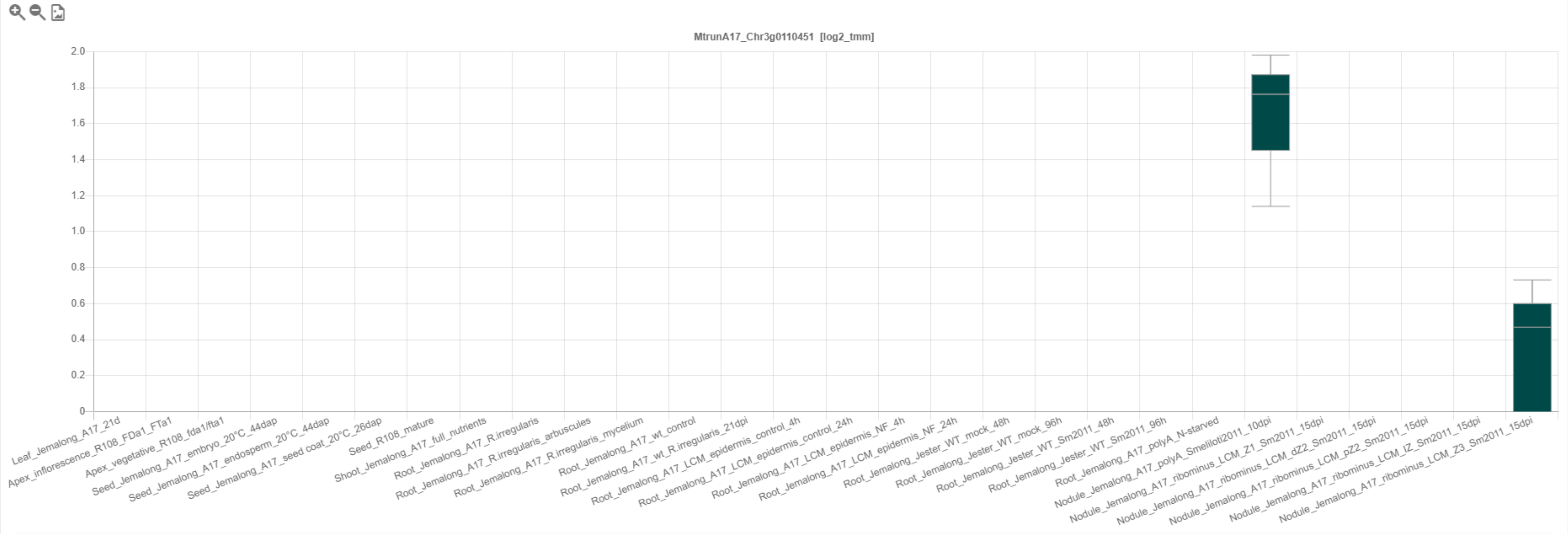

CP56: MtrunA17_Chr3g0113591

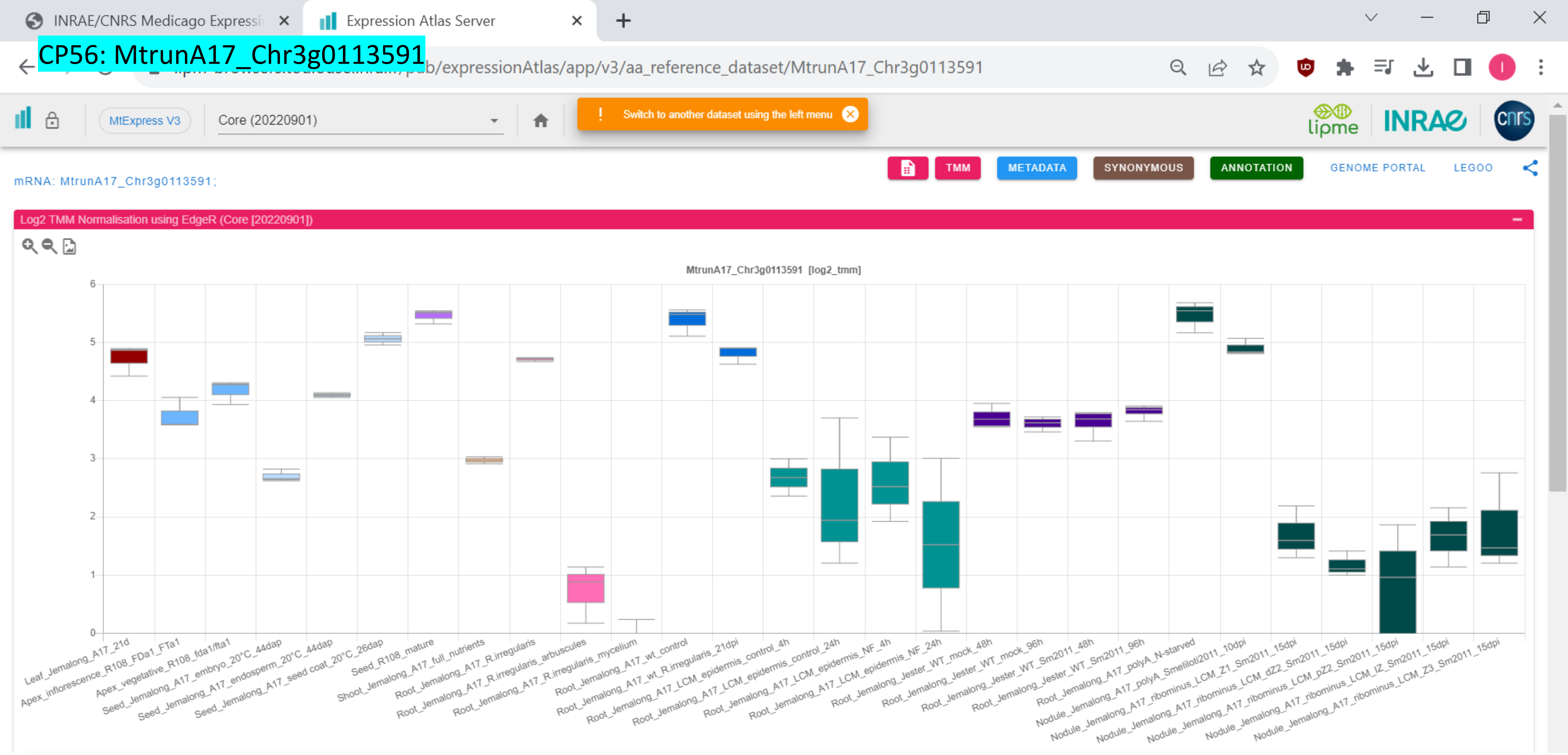

CP57: MtrunA17_Chr3g0124631

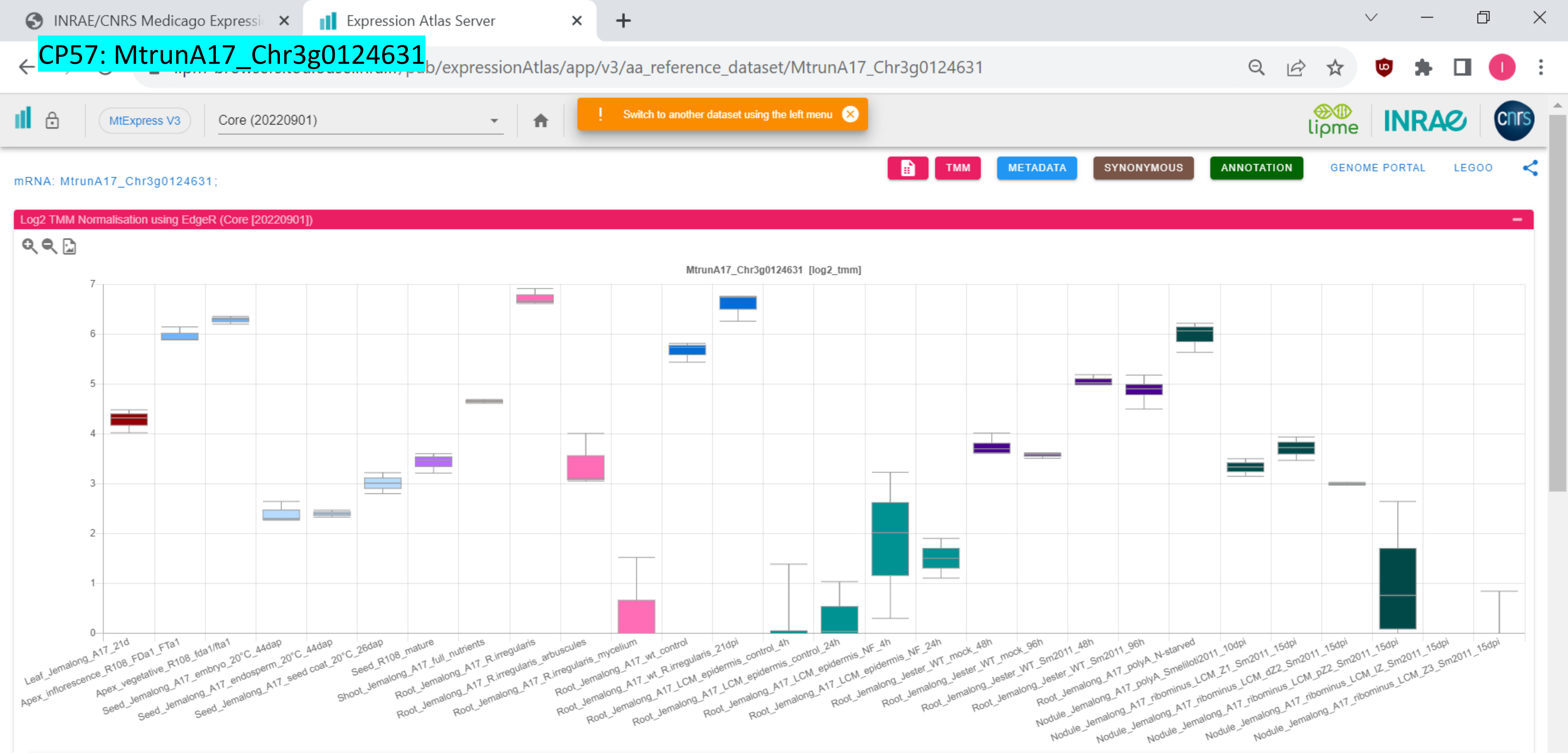

*CP58: MtrunA17_Chr3g0127321

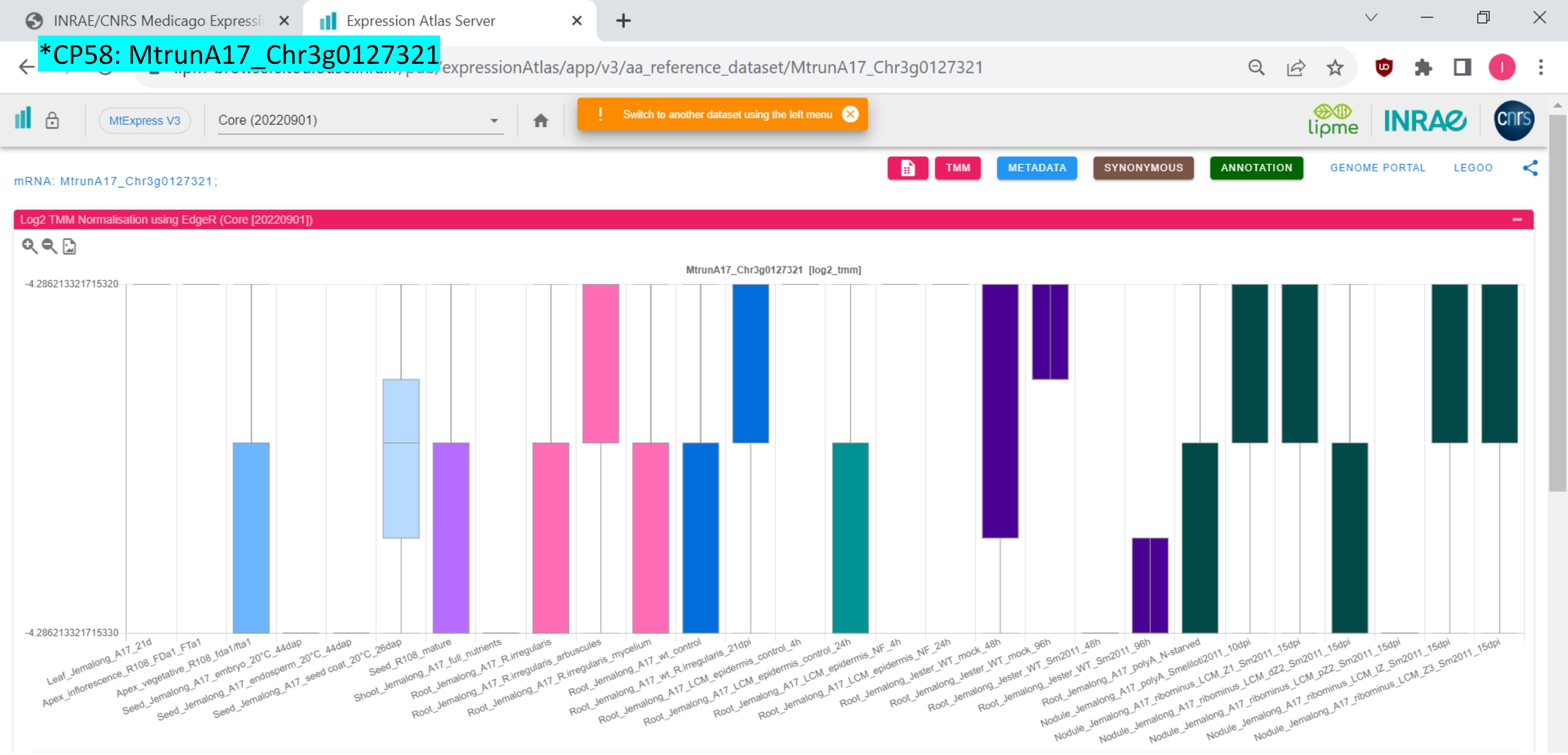

CP59: MtrunA17_Chr3g0130671

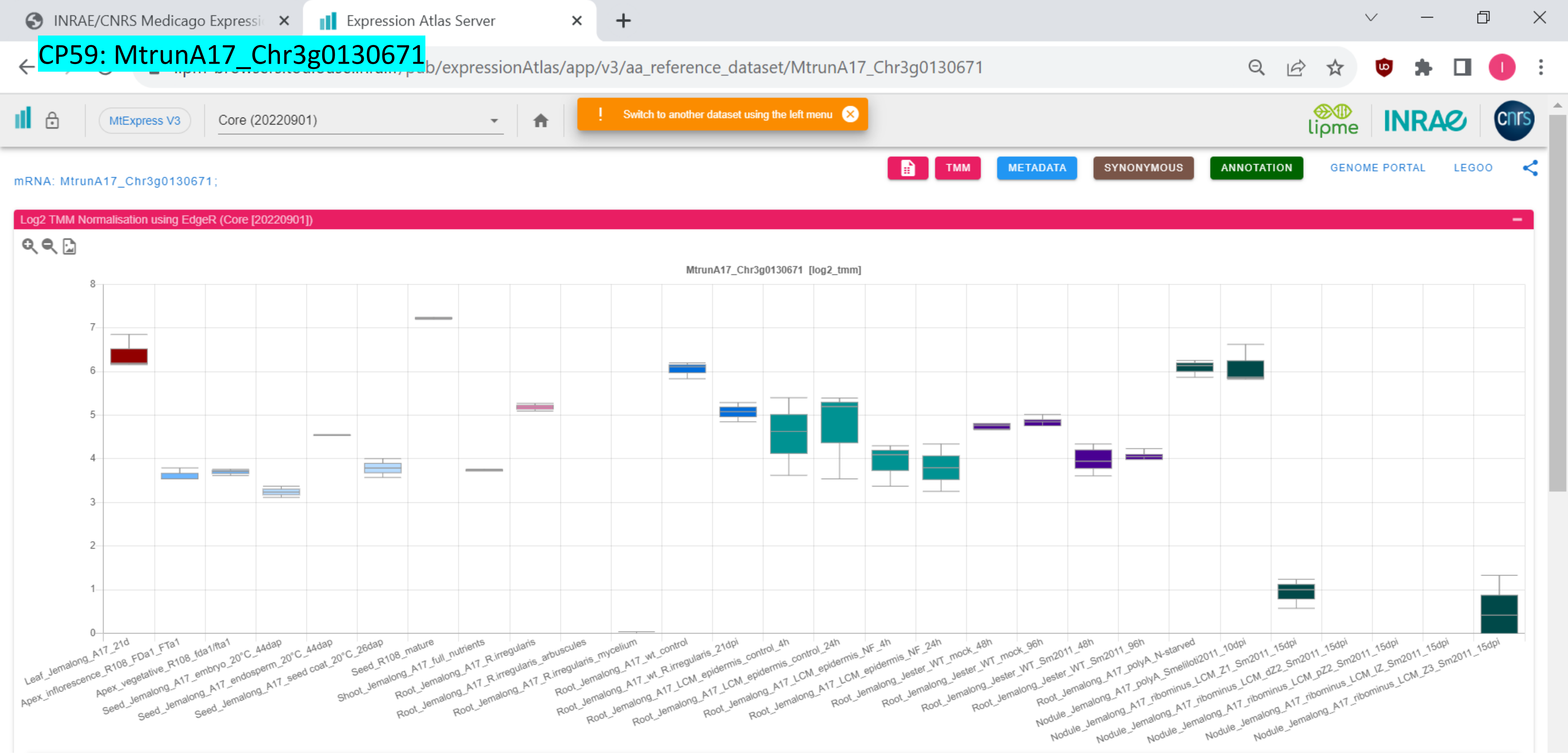

*CP60: MtrunA17_Chr3g0135761

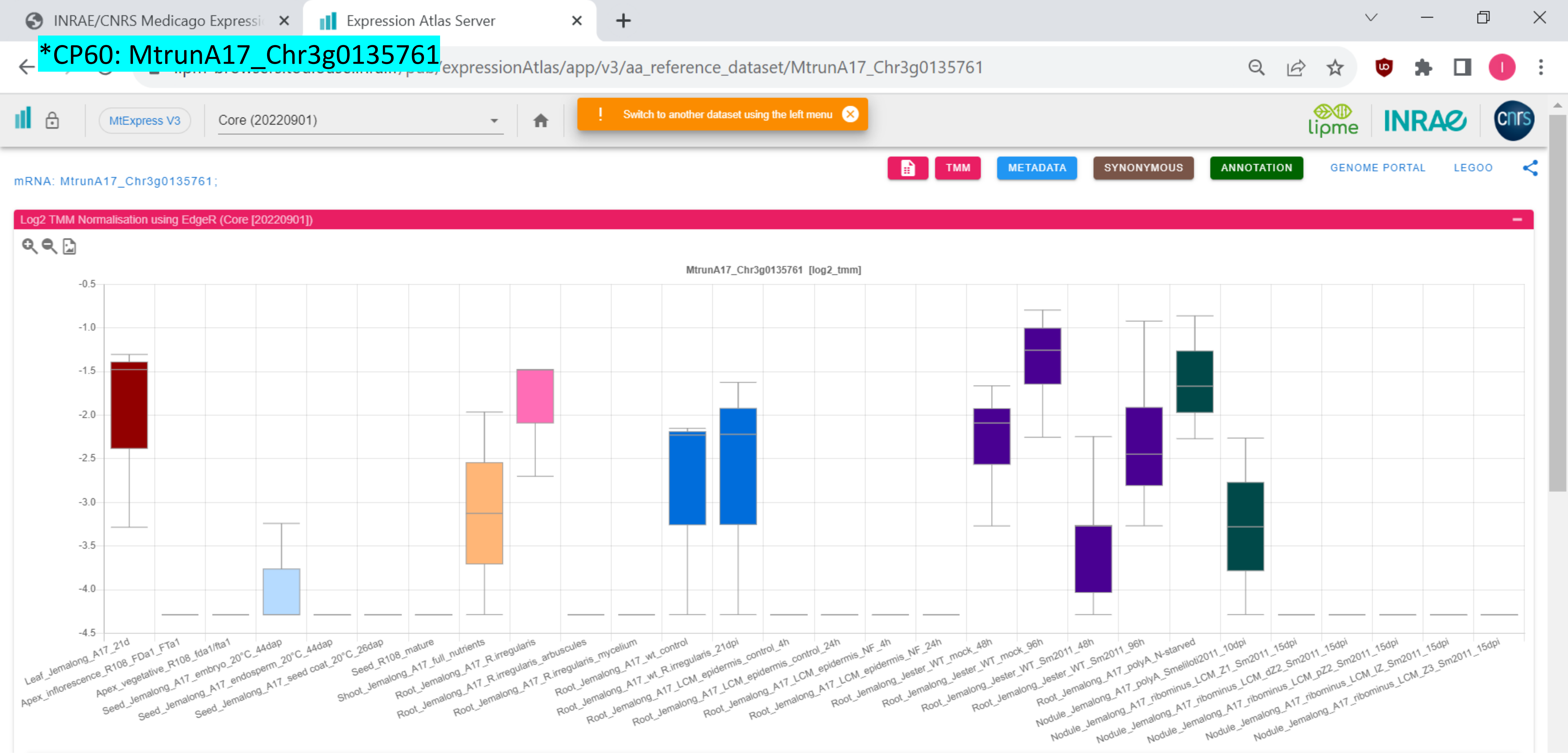

CP61: MtrunA17_Chr3g0137391

INRAE/CNRS Medicago Expressi | Expression Atlas Server

[illegible]/pub/expressionAtlas/app/v3/aa_reference_dataset/MtrunA17_Chr3g0137391

MtExpress V3 | Core (20220901) | Switch to another dataset using the left menu

TMM | METADATA | SYNONYMOUS | ANNOTATION | GENOME PORTAL | LEGOO

mRNA: MtrunA17_Chr3g0137391;

Log2 TMM Normalisation using EdgeR (Core [20220901])

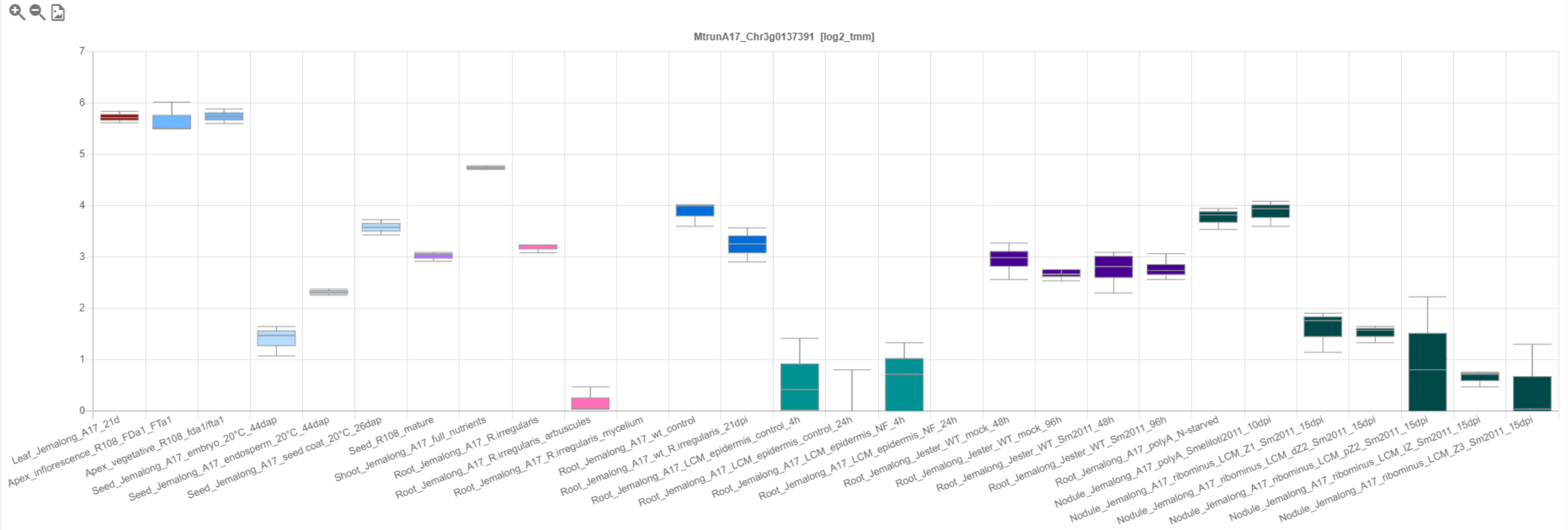

CP62: MtrunA17_Chr3g0141901

INRAE/CNRS Medicago Expressi | Expression Atlas Server

…b/expressionAtlas/app/v3/aa_reference_dataset/MtrunA17_Chr3g0141901

MtExpress V3 | Core (20220901)

! Switch to another dataset using the left menu

TMM | METADATA | SYNONYMOUS | ANNOTATION | GENOME PORTAL | LEGOO

mRNA: MtrunA17_Chr3g0141901;

Log2 TMM Normalisation using EdgeR (Core [20220901])

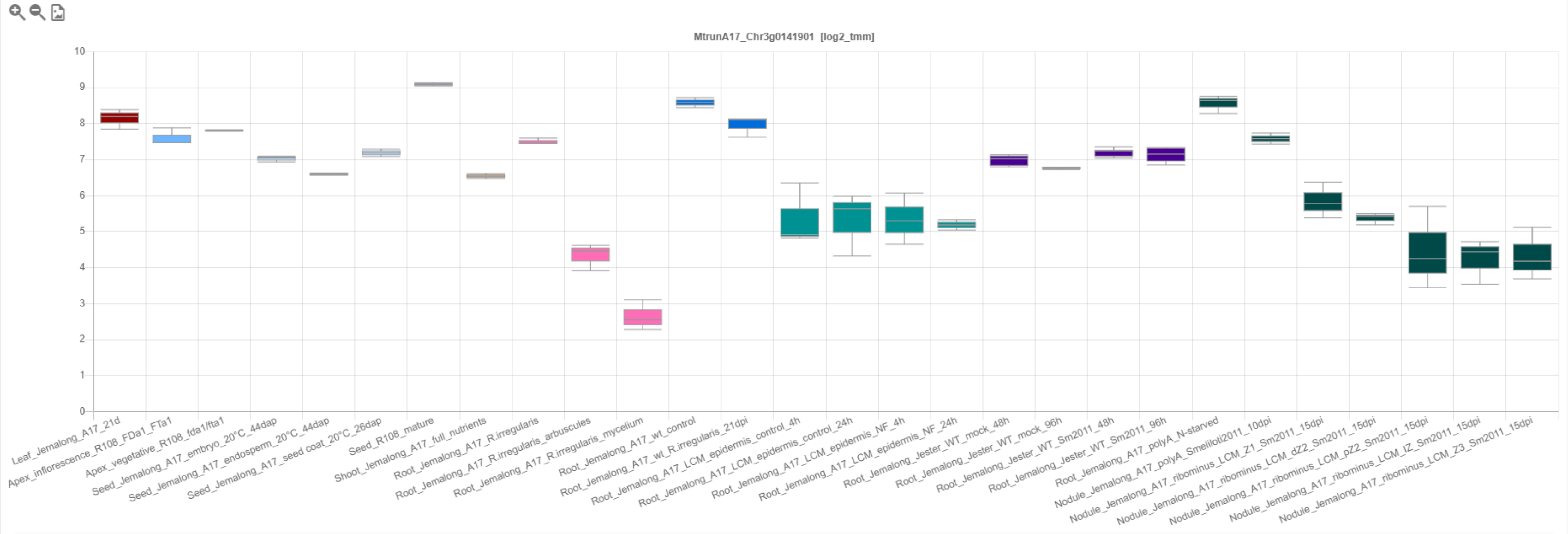

CP63: MtrunA17_Chr3g0144151

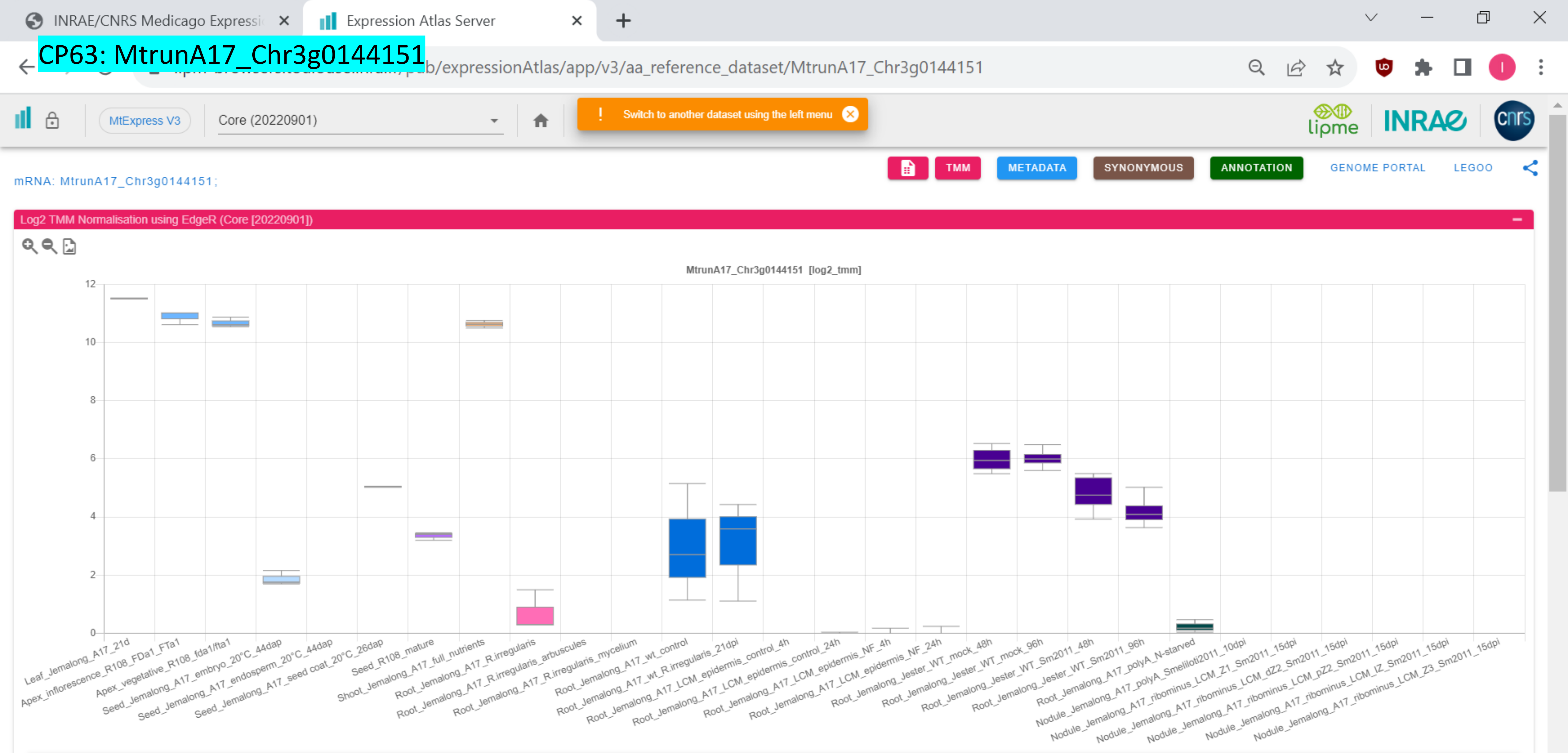

CP64: MtrunA17_Chr3g0144151

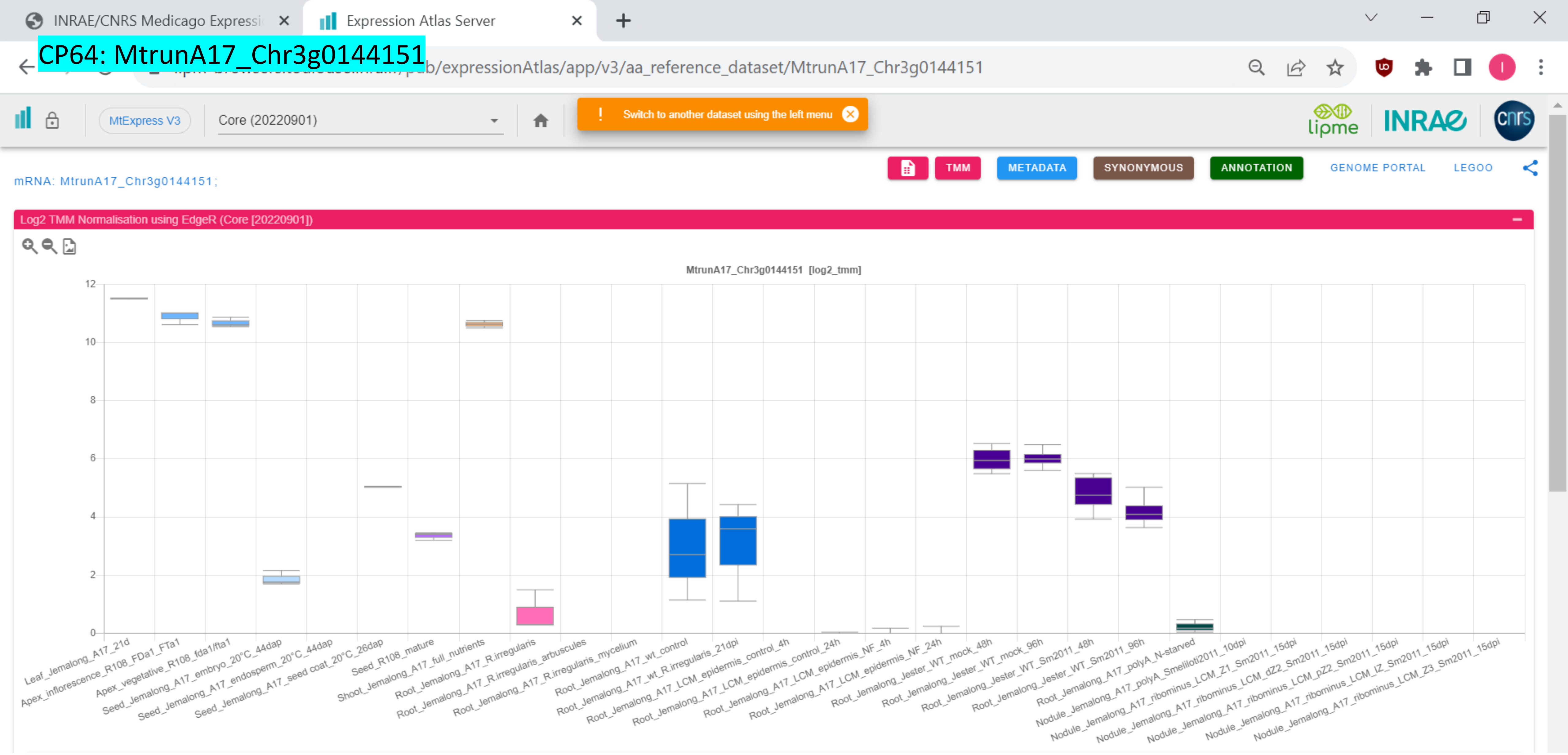

CP65: MtrunA17_Chr3g0144151

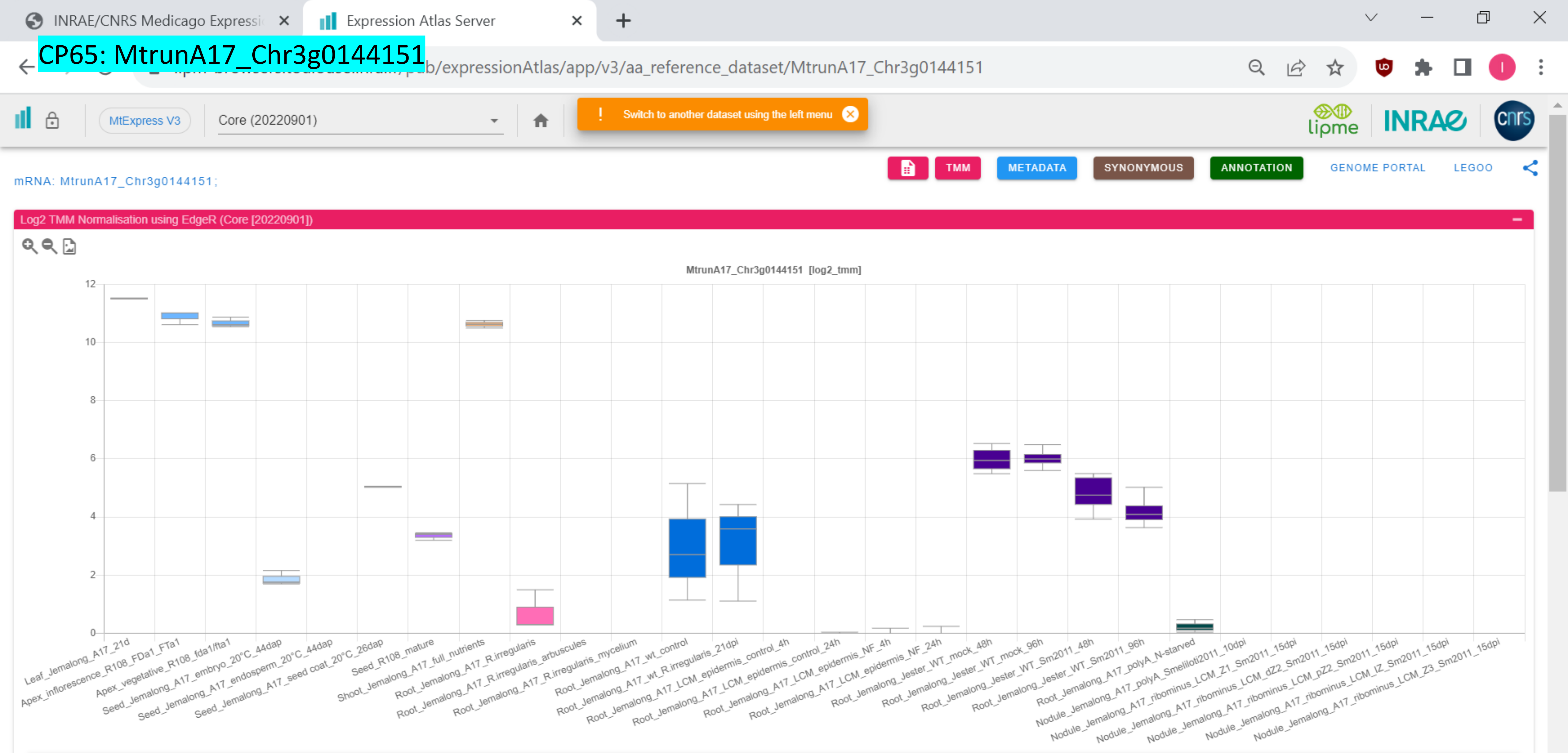

*CP66: MtrunA17_Chr3g1011650

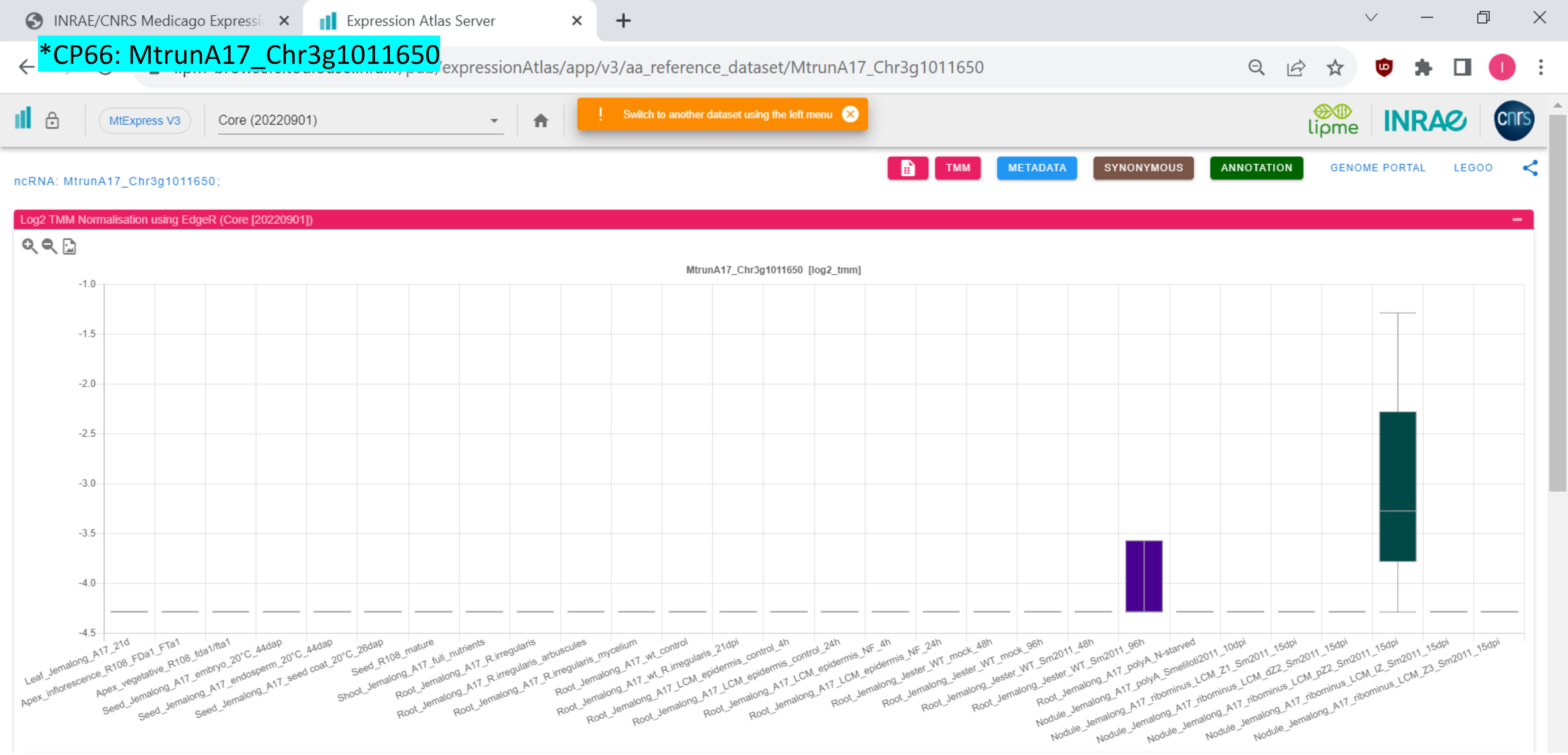

CP67: MtrunA17_Chr4g0000131

INRAE/CNRS Medicago Expressi | Expression Atlas Server

[illegible]/pub/expressionAtlas/app/v3/aa_reference_dataset/MtrunA17_Chr4g0000131

MtExpress V3 | Core (20220901)

! Switch to another dataset using the left menu

TMM | METADATA | SYNONYMOUS | ANNOTATION | GENOME PORTAL | LEGOO

mRNA: MtrunA17_Chr4g0000131;

Log2 TMM Normalisation using EdgeR (Core [20220901])

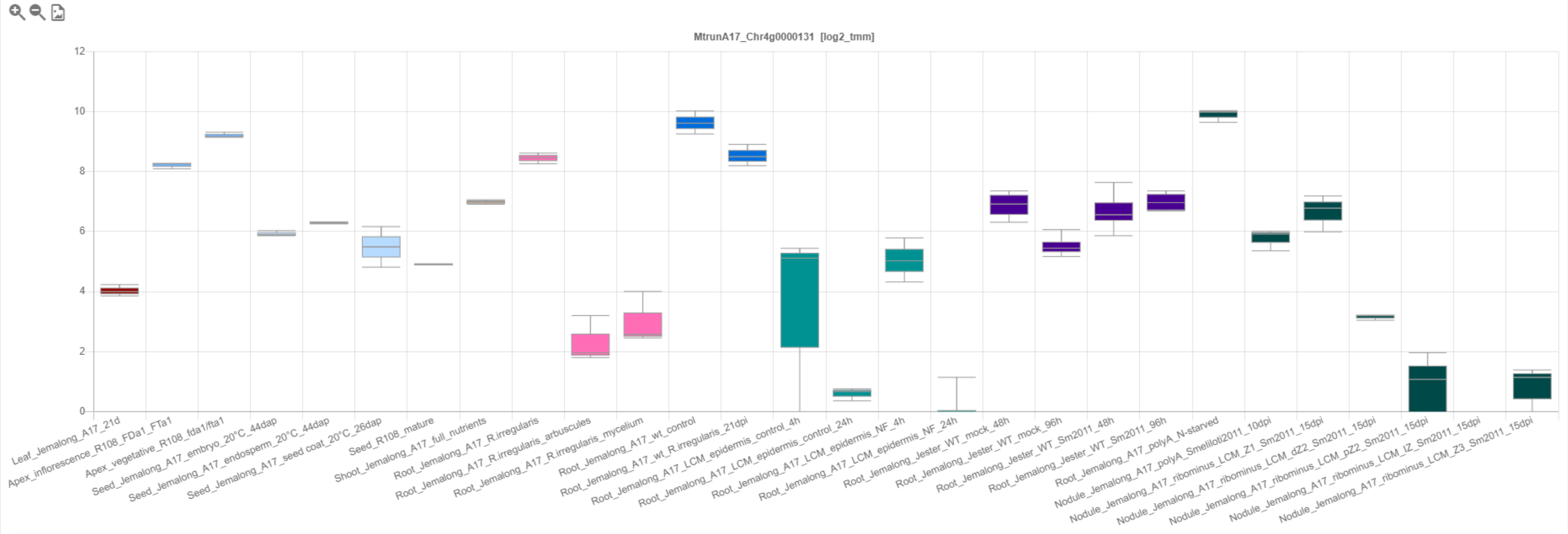

*CP68: MtrunA17_Chr4g0000891

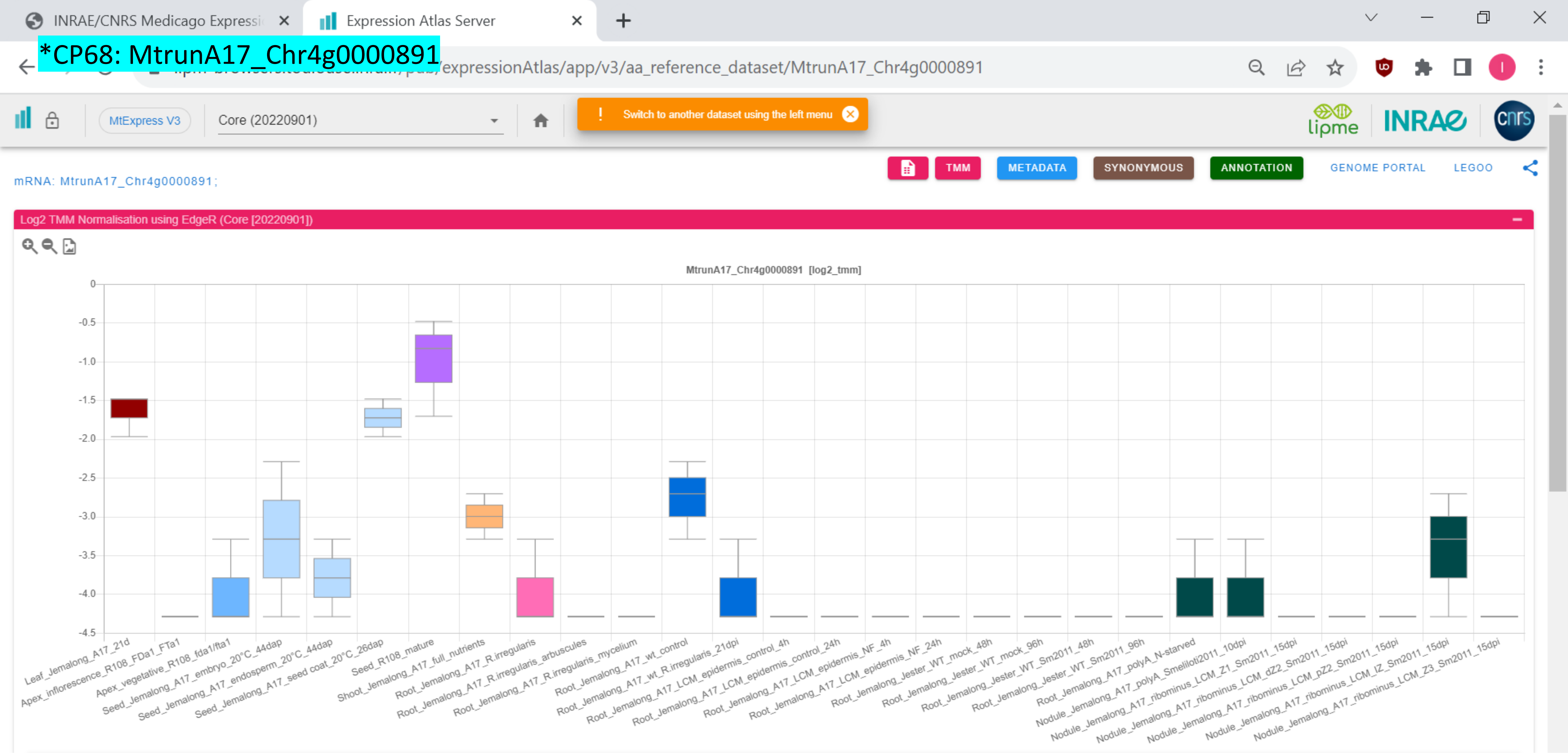

CP69: MtrunA17_Chr4g0004721

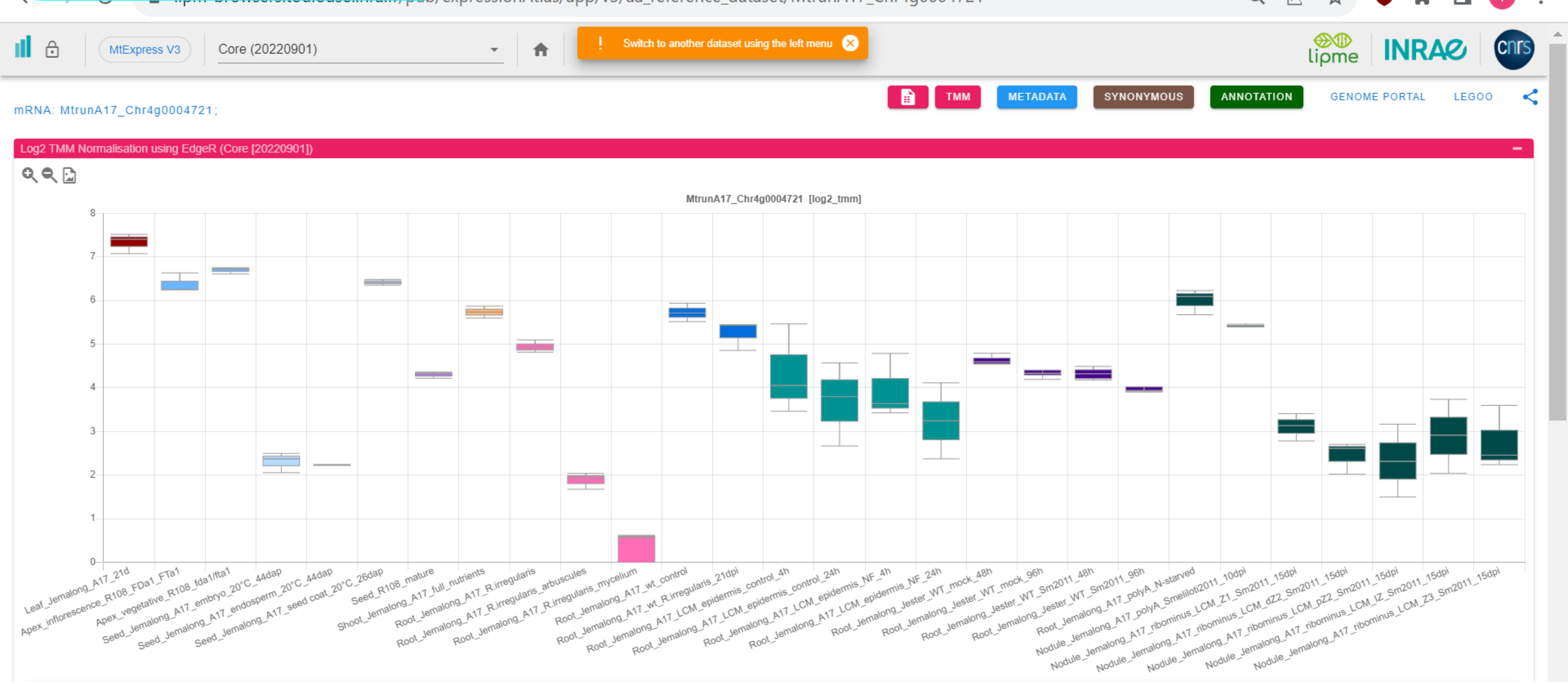

*CP70: MtrunA17_Chr4g0014251

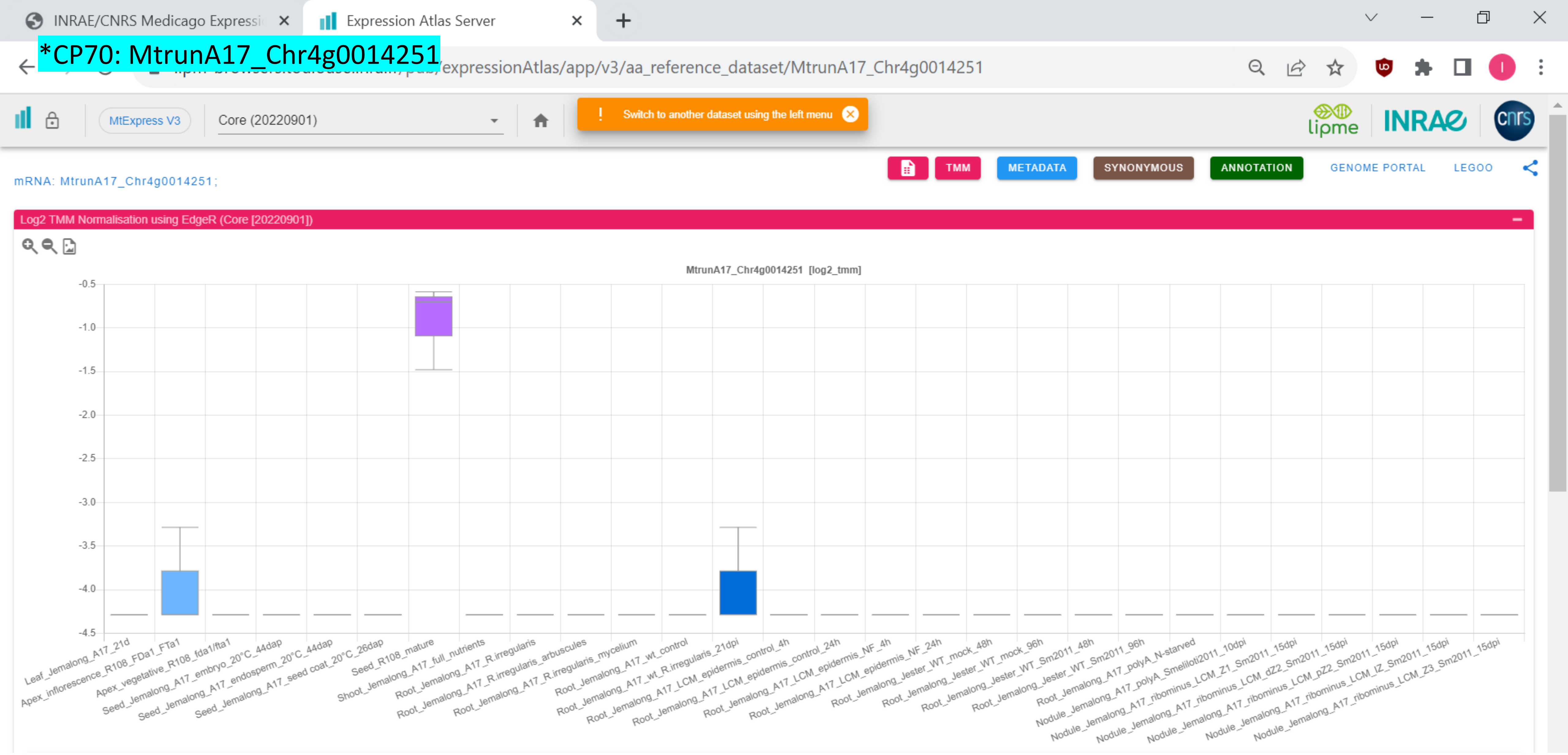

CP71: MtrunA17_Chr4g0022491

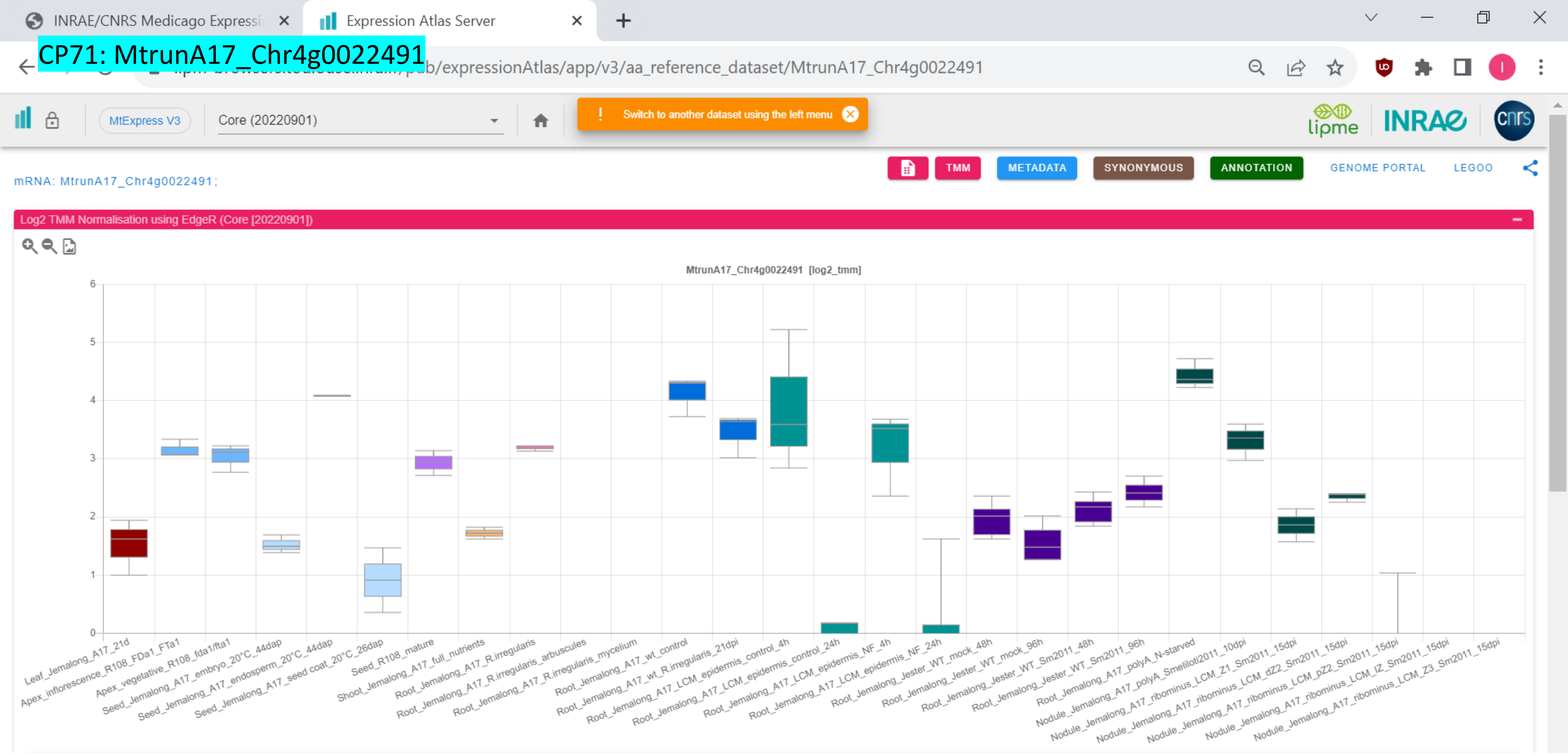

*CP72: MtrunA17_Chr4g0023791

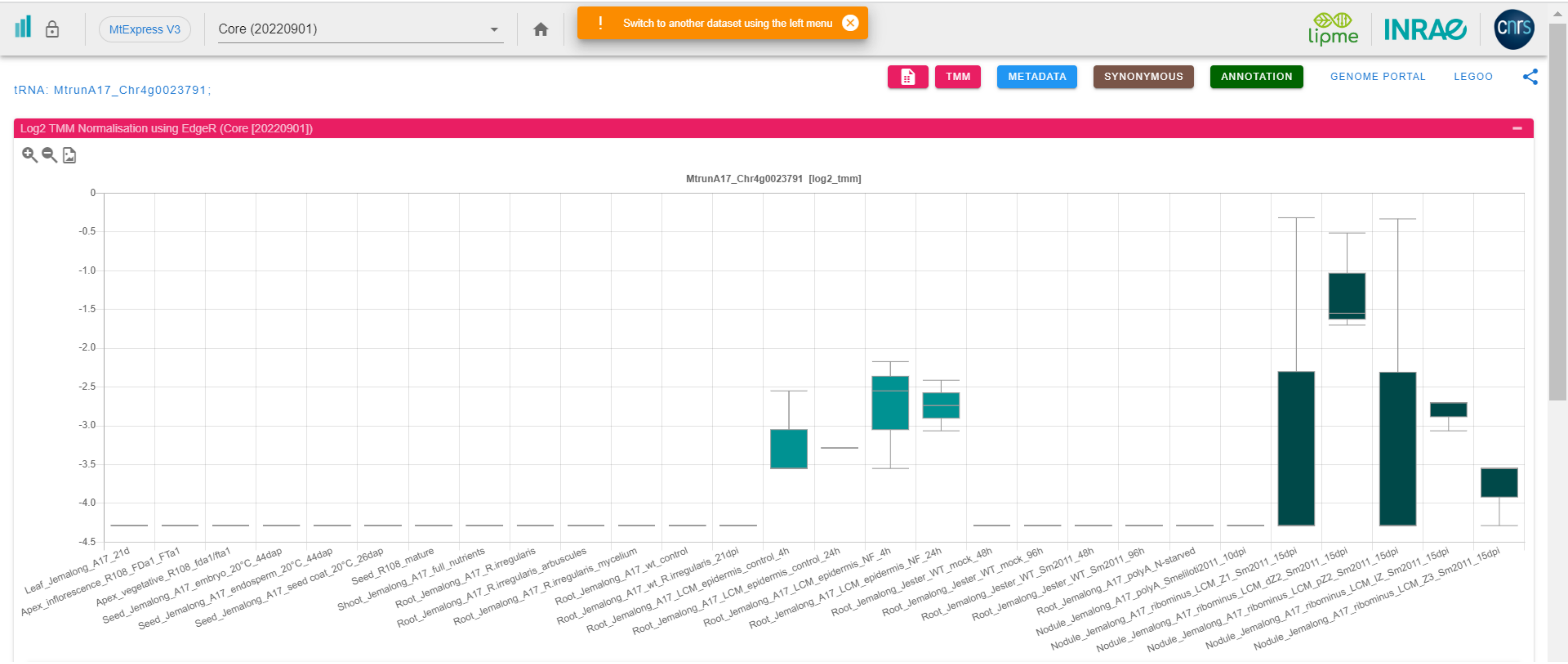

CP73: MtrunA17_Chr4g0034271

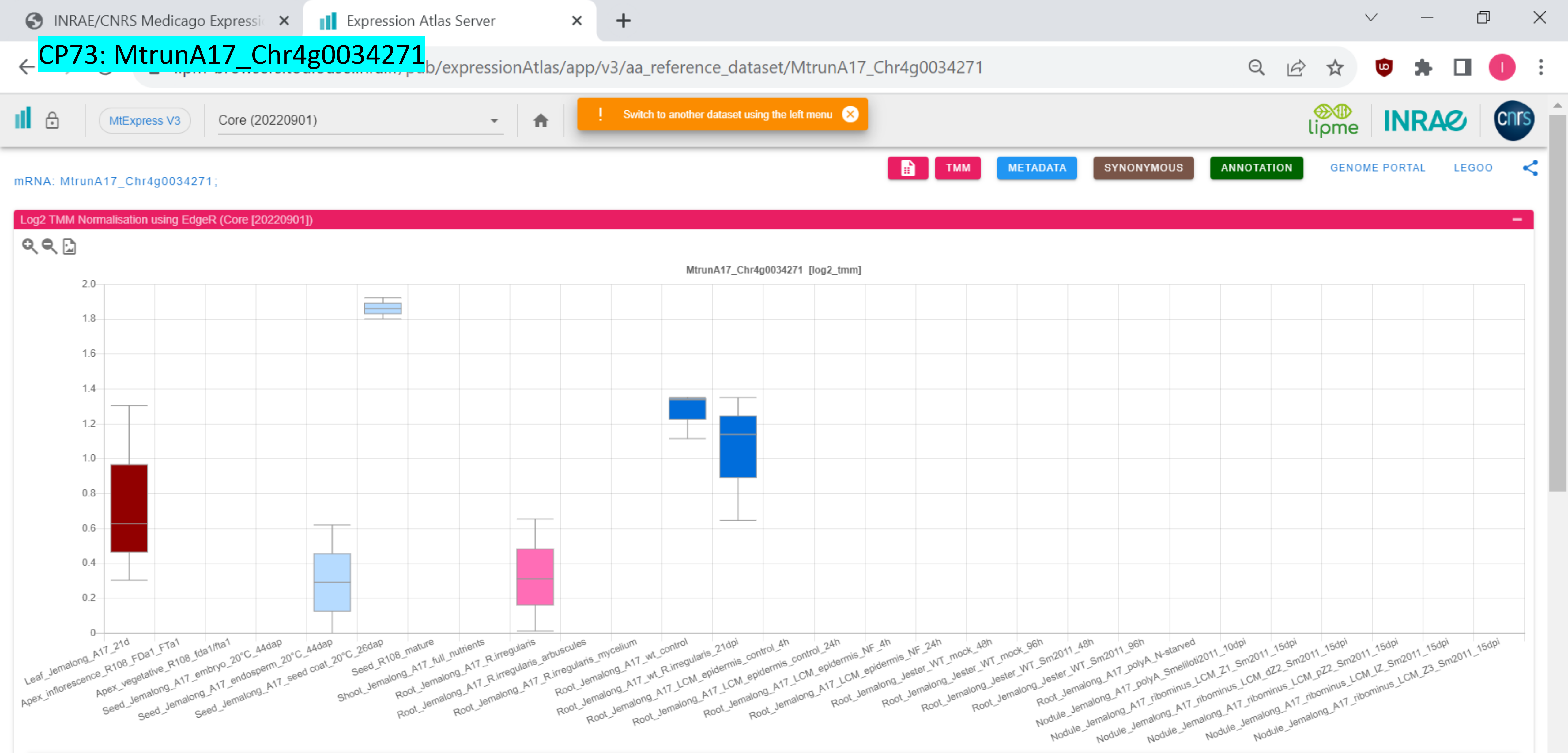

CP74: MtrunA17_Chr4g0034491

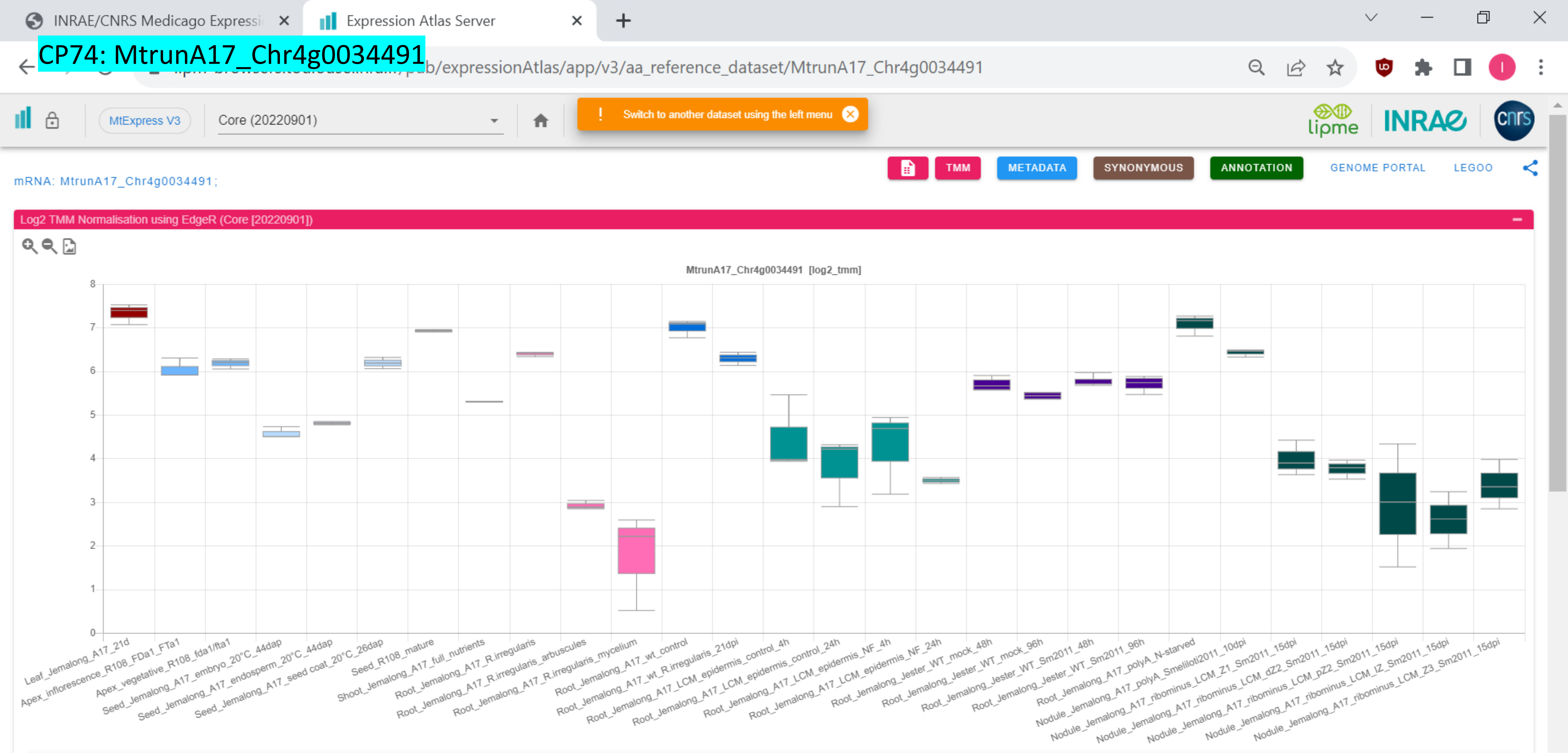

CP75: MtrunA17_Chr4g0037381

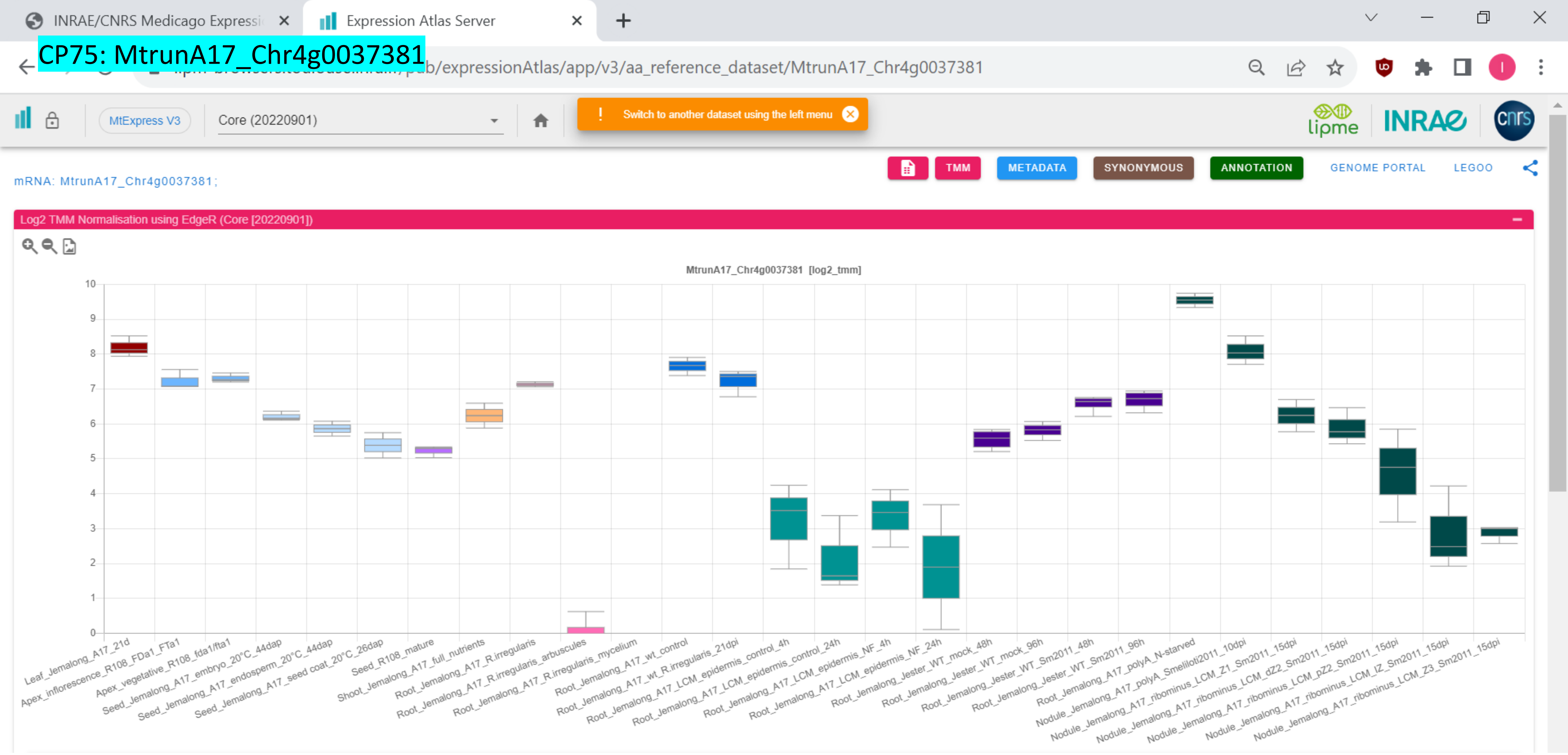

CP76: MtrunA17_Chr4g0040471

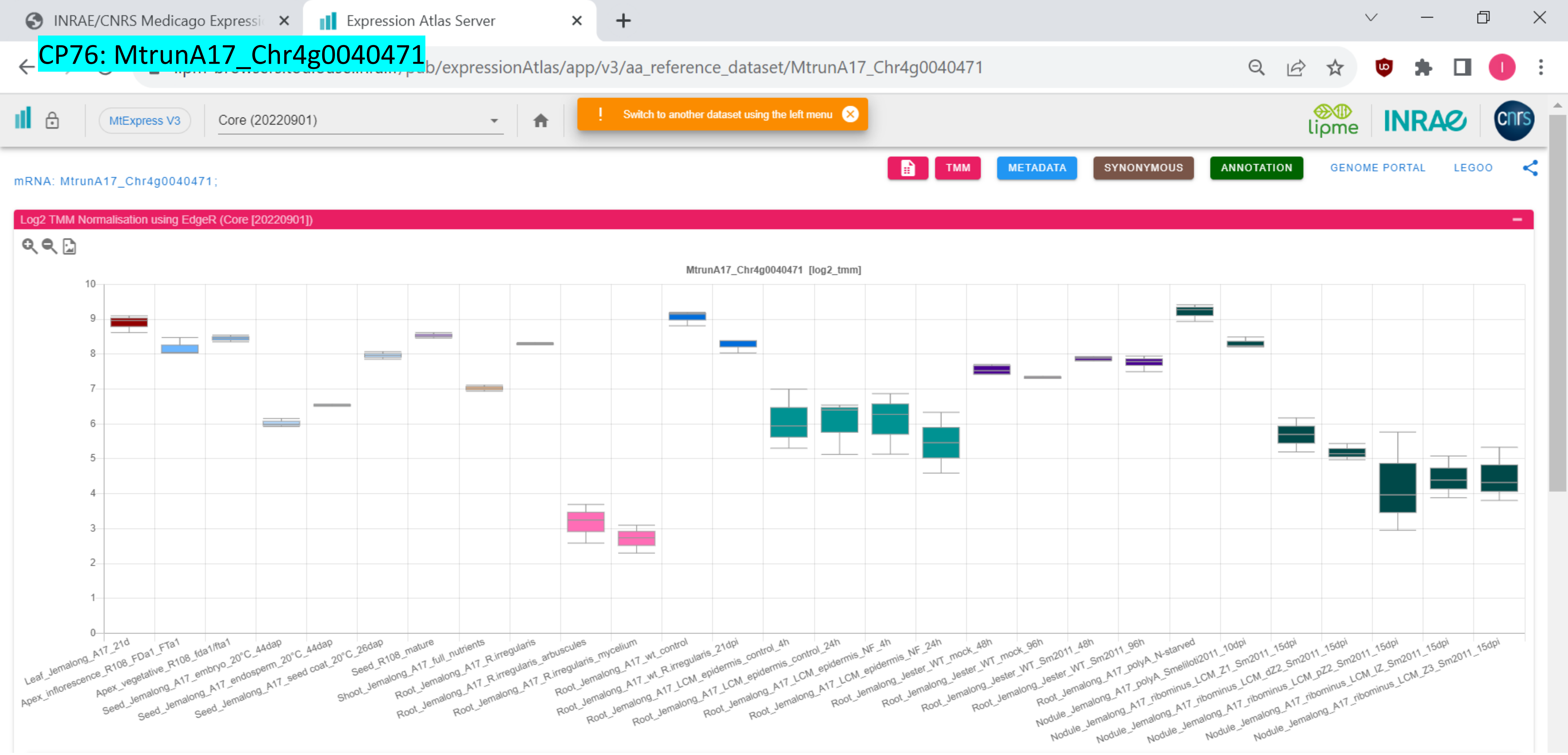

CP77: MtrunA17_Chr4g0055331

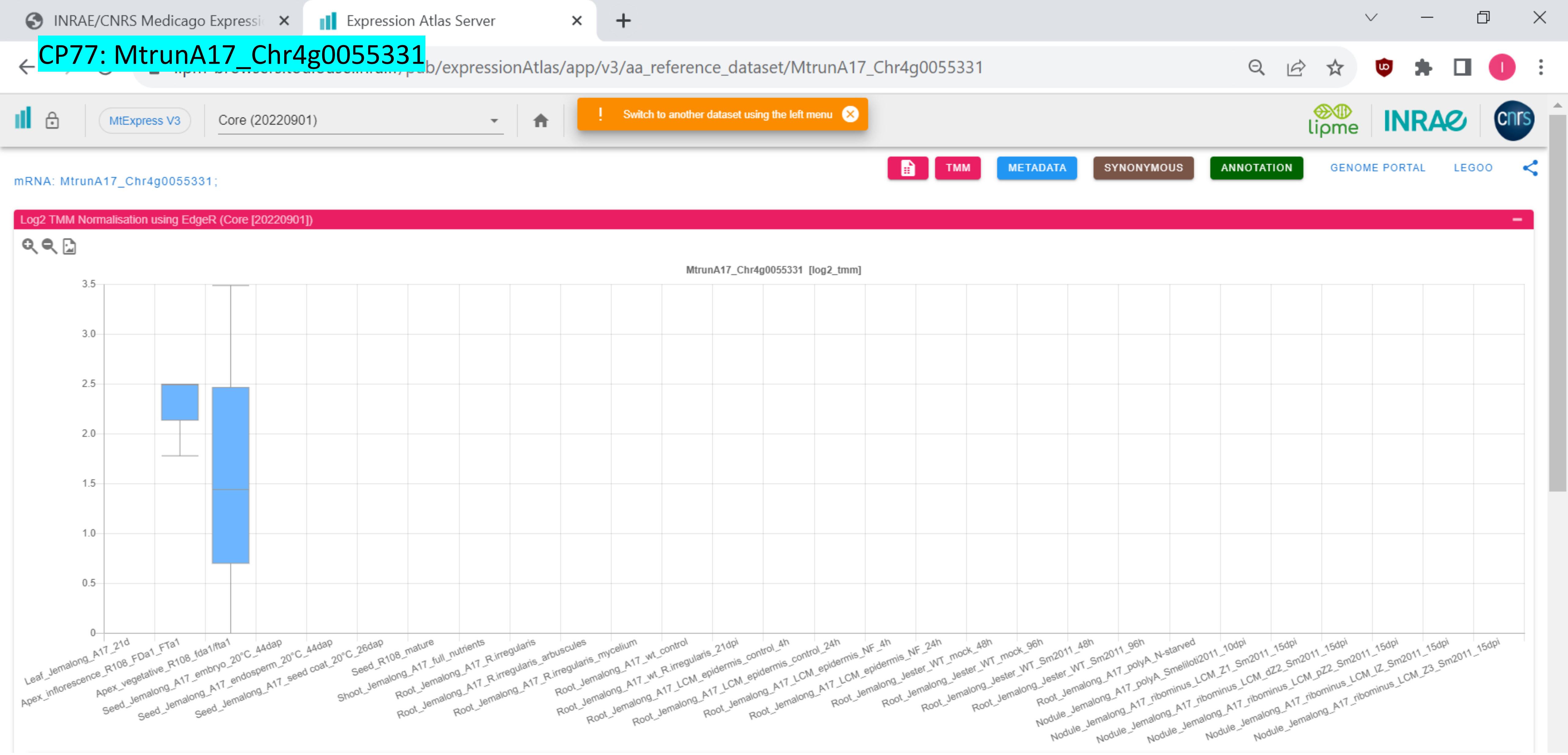

CP78: MtrunA17_Chr4g0059001

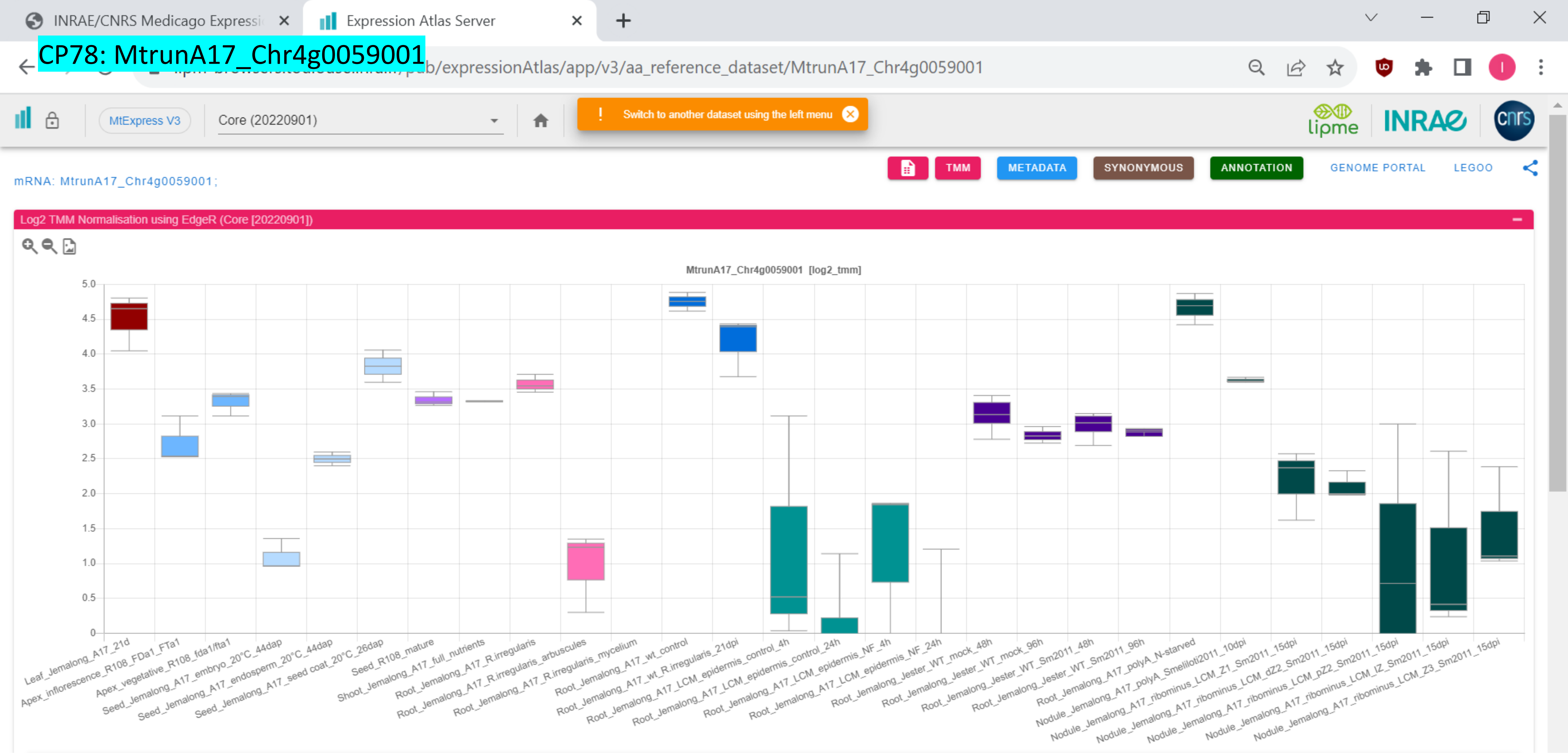

*CP79: MtrunA17_Chr4g0063201

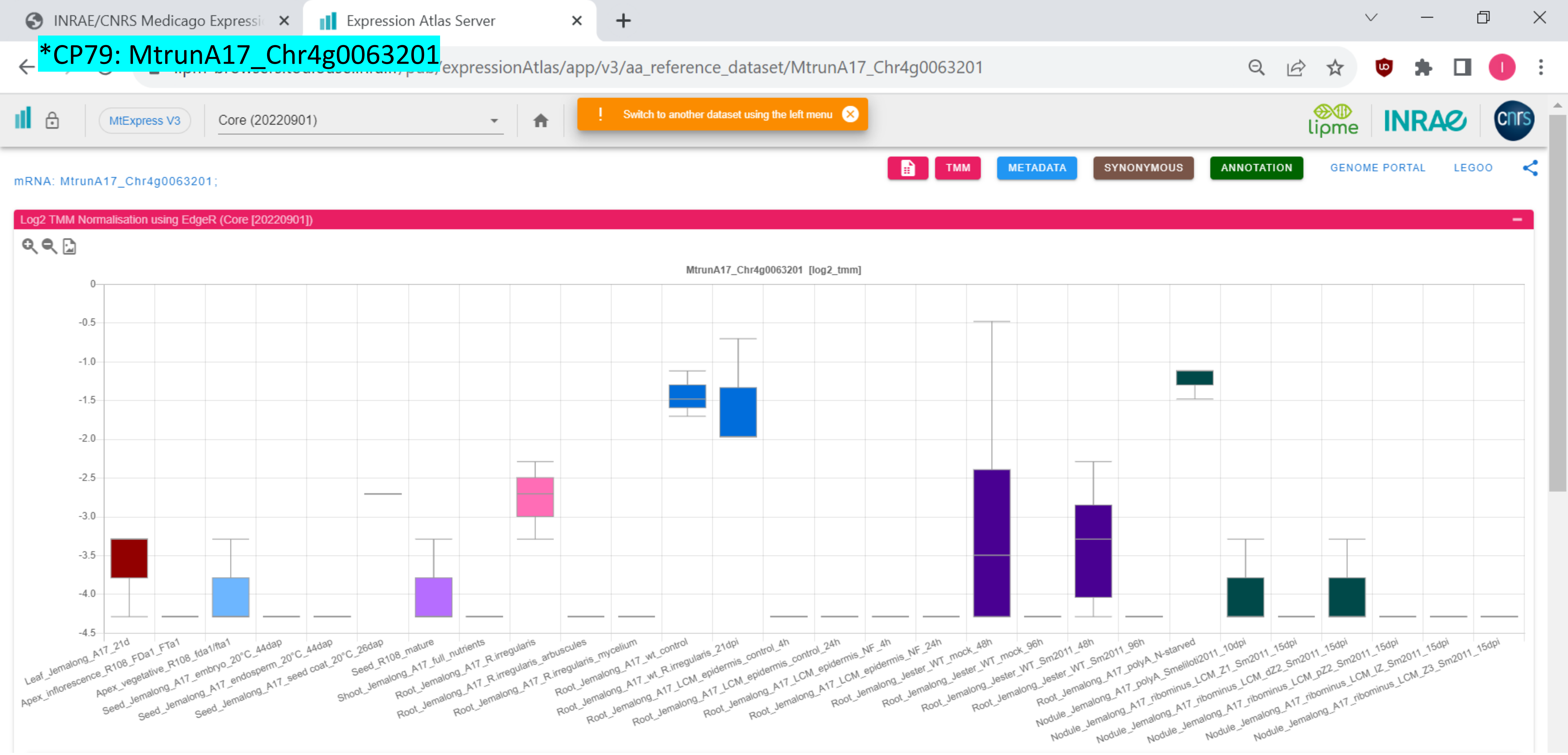

CP80: MtrunA17_Chr4g0070011

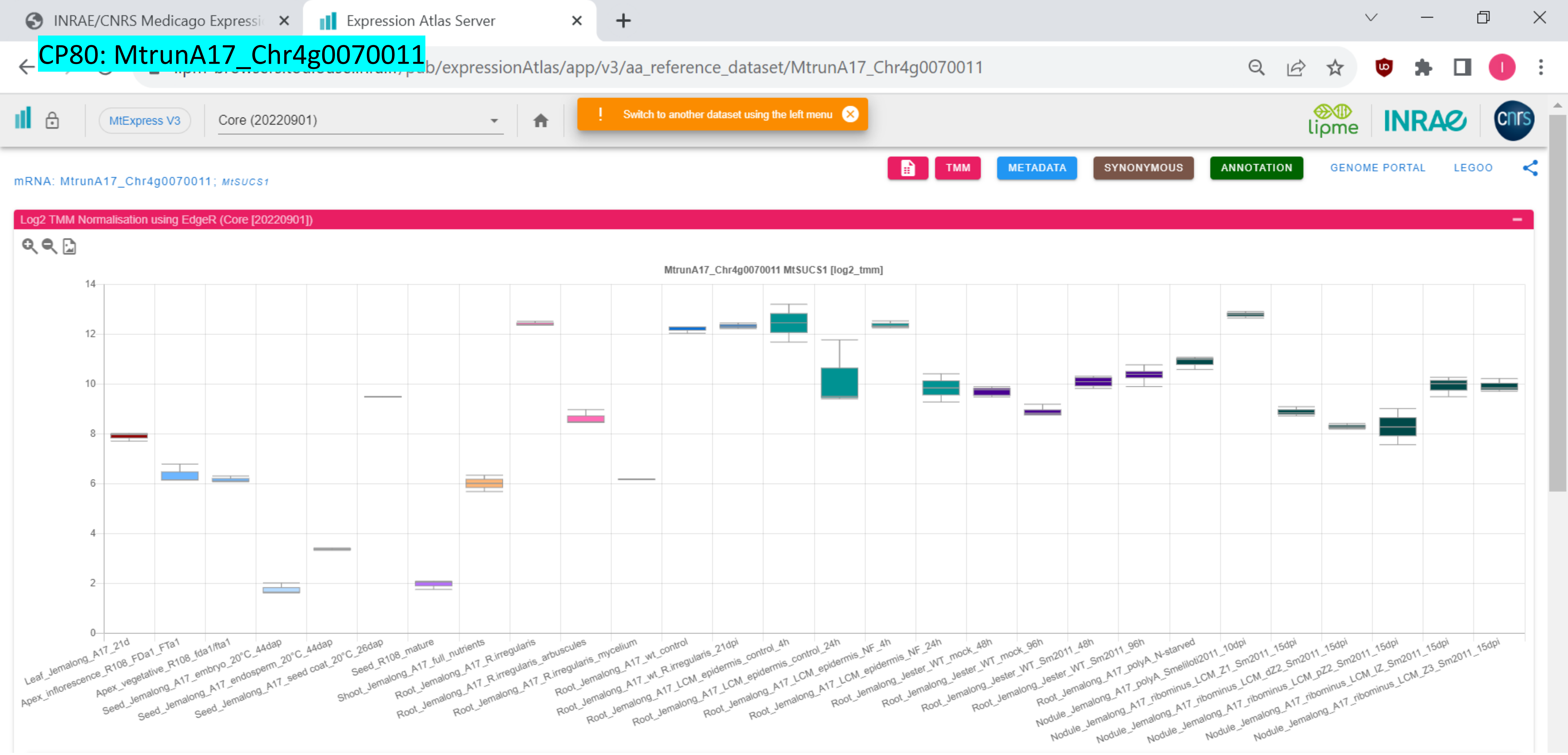

CP81: MtrunA17_Chr5g0393401

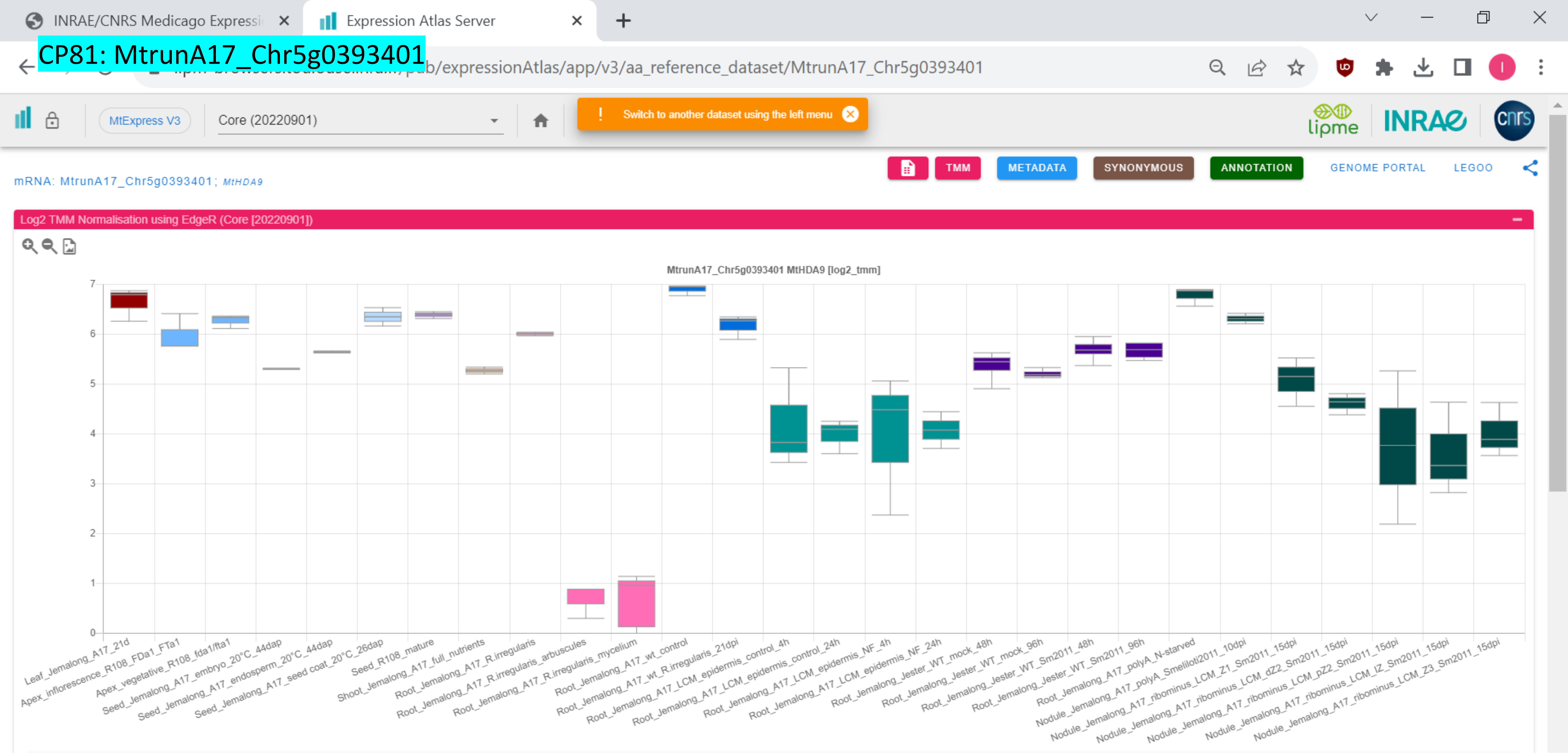

CP82: MtrunA17_Chr5g0400181

INRAE/CNRS Medicago Expressi | Expression Atlas Server

.../pub/expressionAtlas/app/v3/aa_reference_dataset/MtrunA17_Chr5g0400181

MtExpress V3 | Core (20220901) | Switch to another dataset using the left menu

lipme INRAE cnrs

TMM | METADATA | SYNONYMOUS | ANNOTATION | GENOME PORTAL | LEGOO

mRNA: MtrunA17_Chr5g0400181;

Log2 TMM Normalisation using EdgeR (Core [20220901])

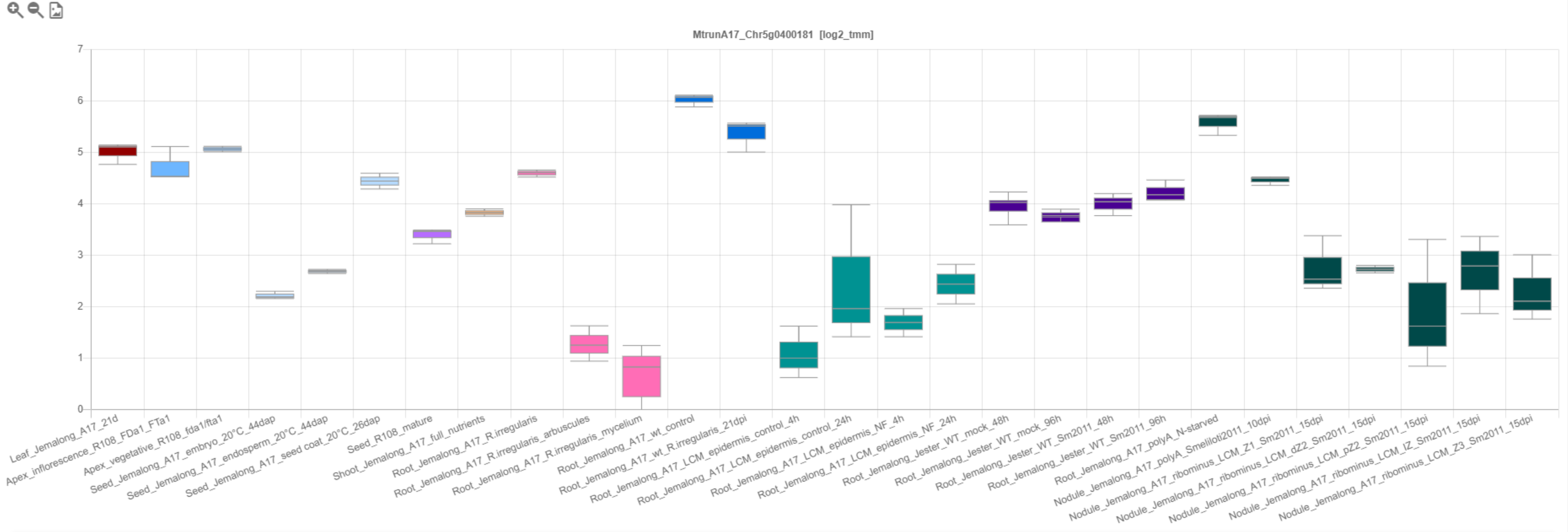

CP83: MtrunA17_Chr5g0405061

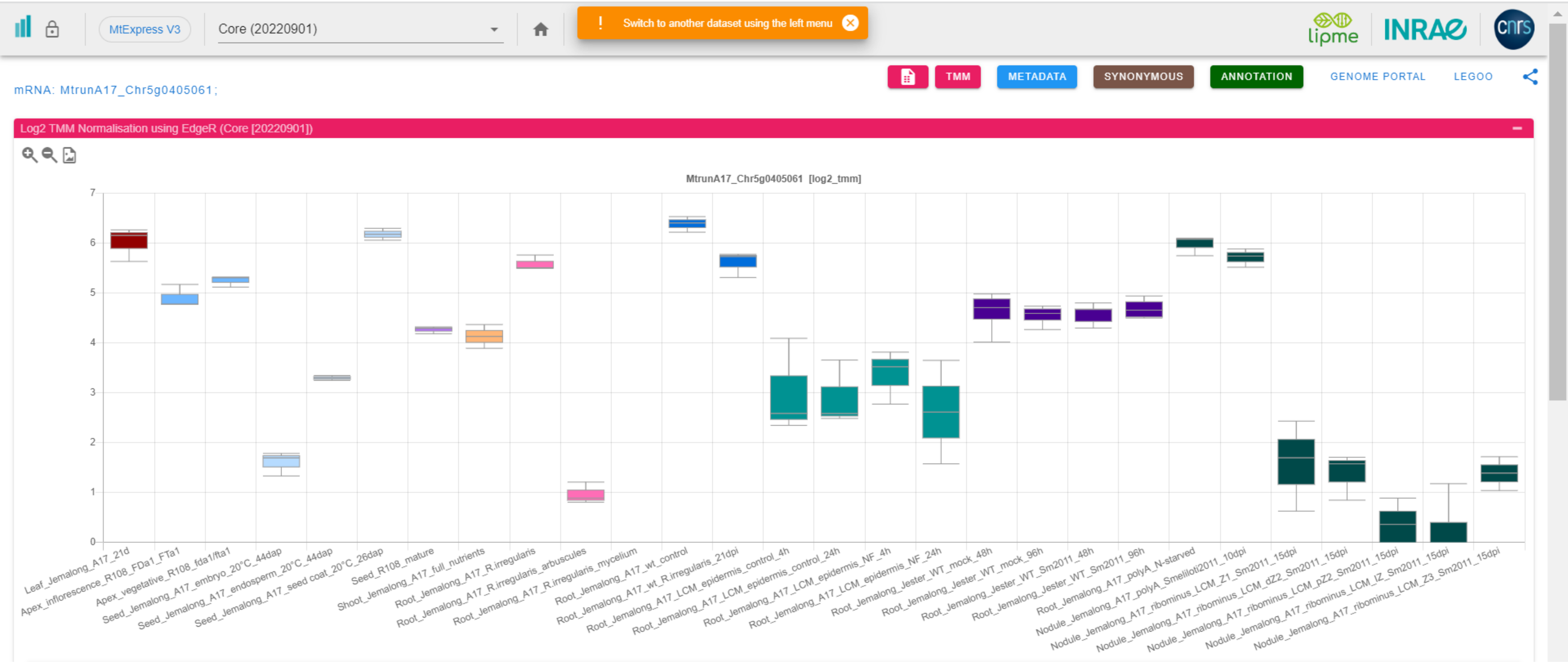

CP84: MtrunA17_Chr5g0408581

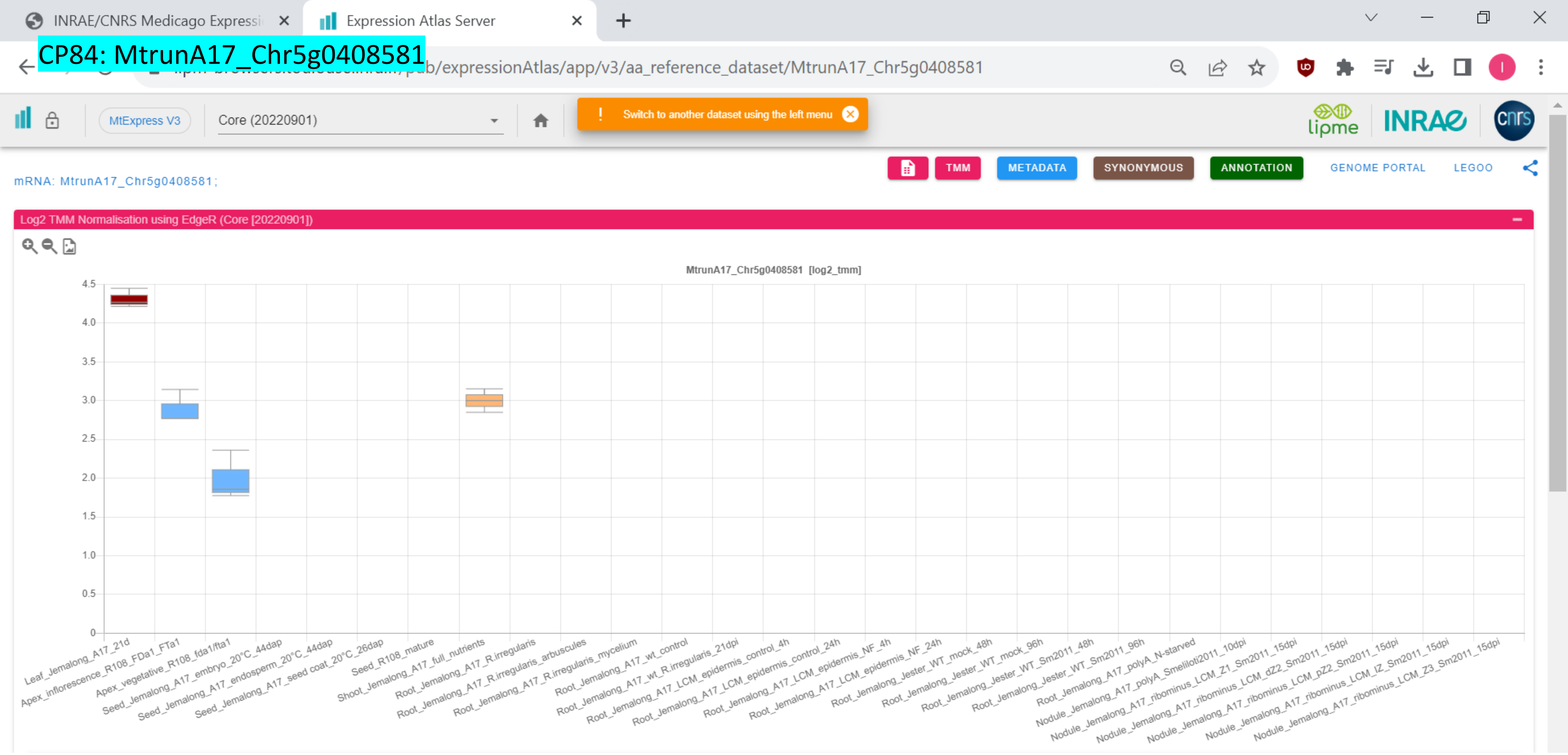

CP85: MtrunA17_Chr5g0415031

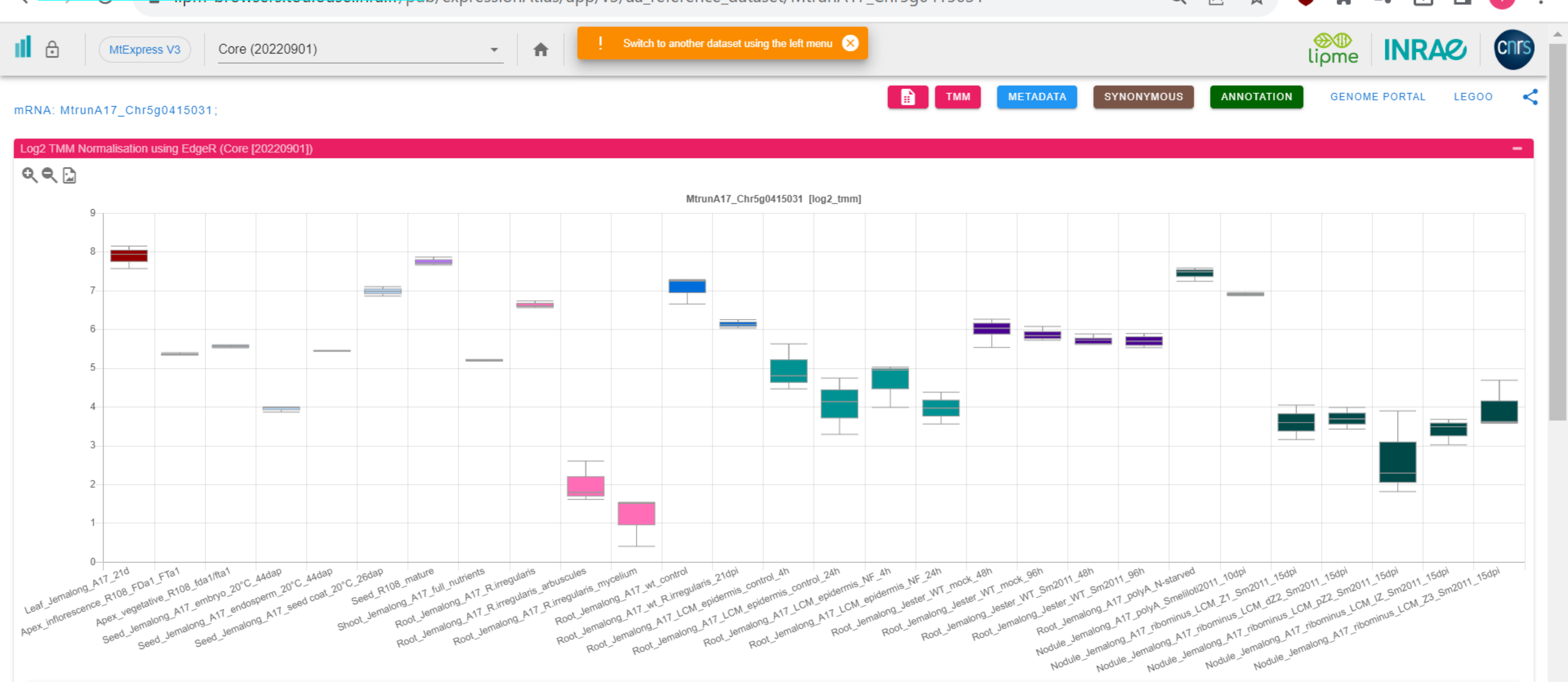

CP86: MtrunA17_Chr5g0415911

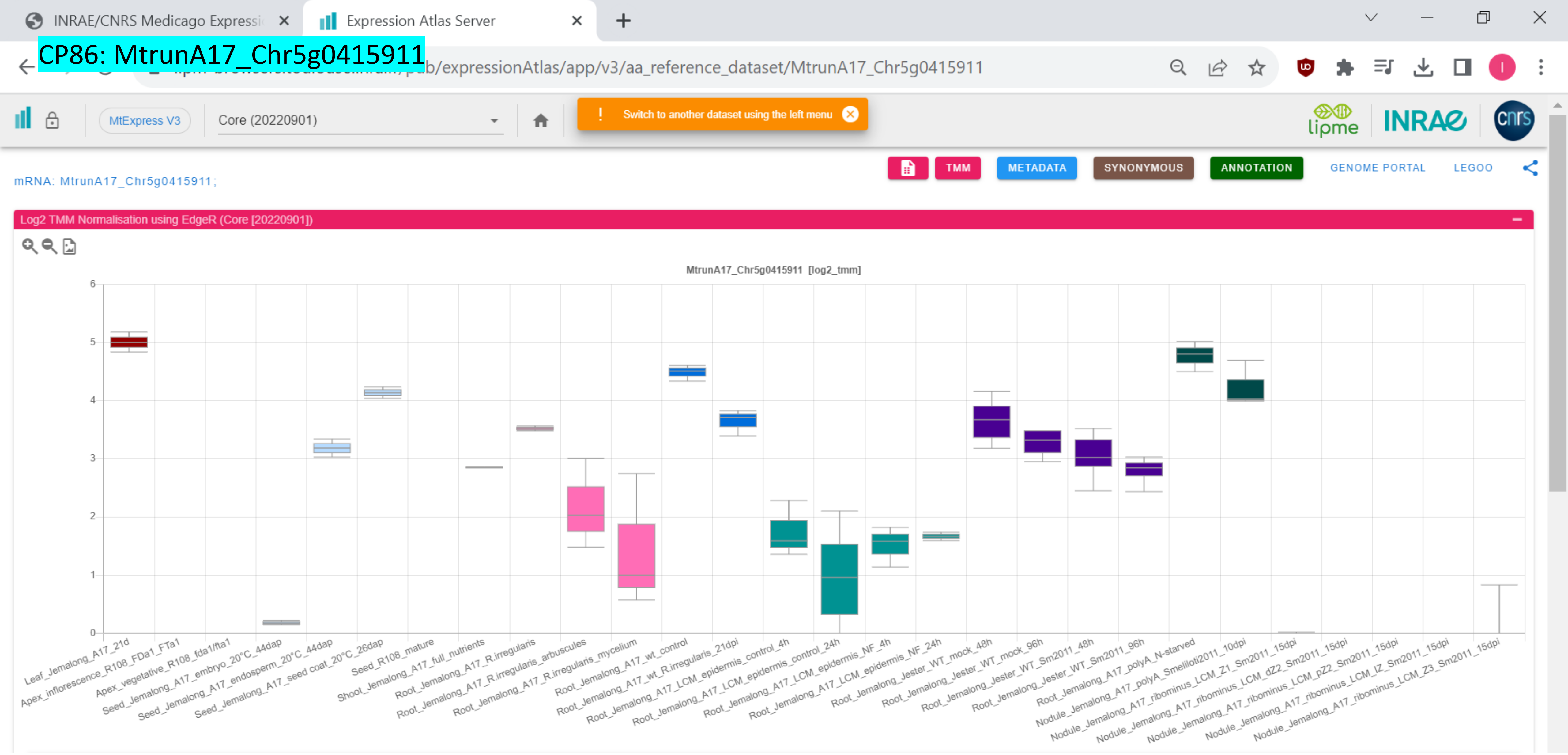

CP87: MtrunA17_Chr5g0421761

INRAE/CNRS Medicago Expressi | Expression Atlas Server

[illegible]/pub/expressionAtlas/app/v3/aa_reference_dataset/MtrunA17_Chr5g0421761

MtExpress V3 | Core (20220901)

! Switch to another dataset using the left menu

TMM | METADATA | SYNONYMOUS | ANNOTATION | GENOME PORTAL | LEGOO

ncRNA: MtrunA17_Chr5g0421761;

Log2 TMM Normalisation using EdgeR (Core [20220901])

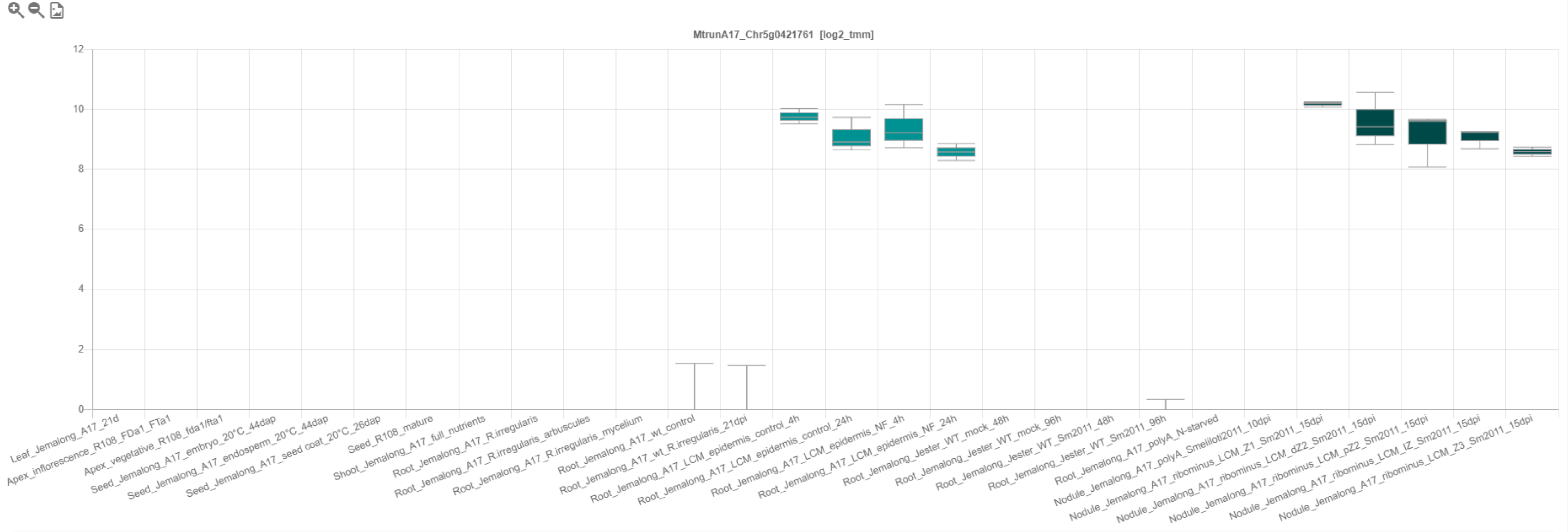

CP88: MtrunA17_Chr5g0422291

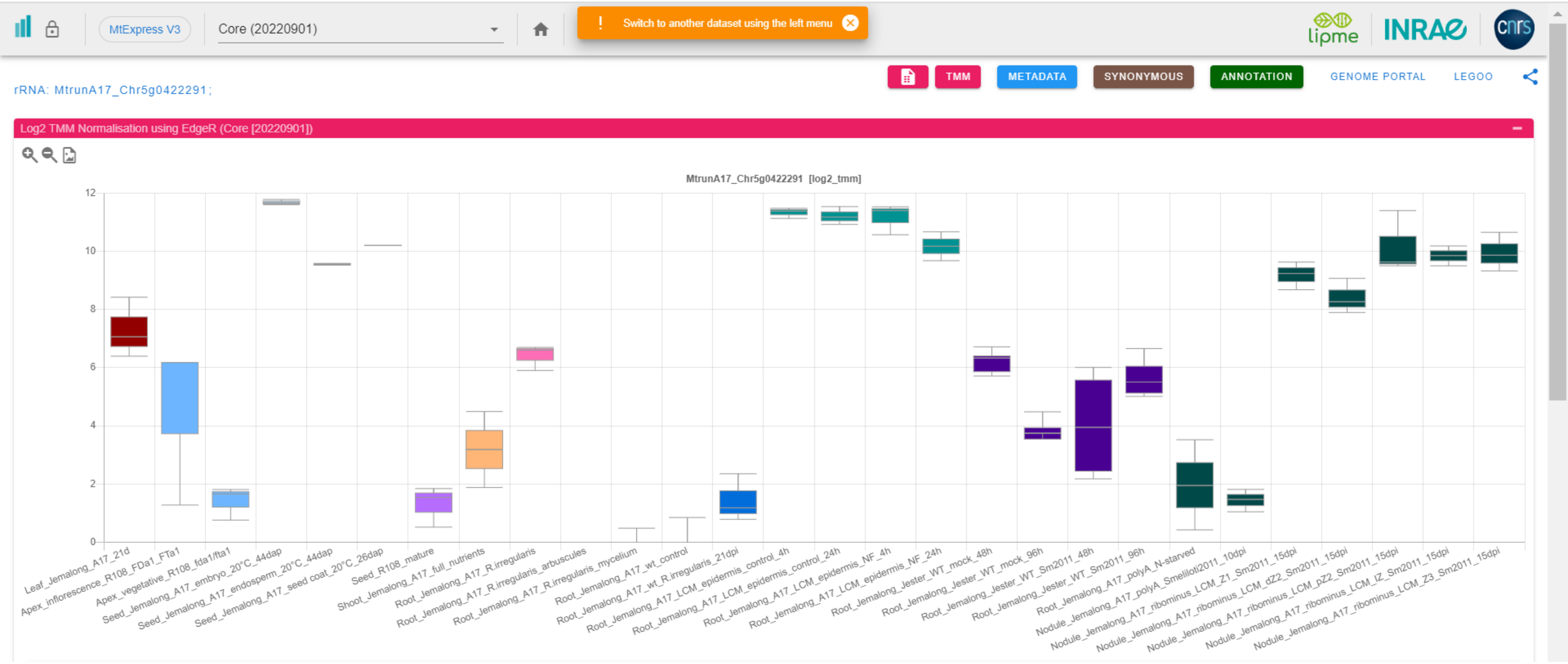

CP89: MtrunA17_Chr5g0422291

INRAE/CNRS Medicago Expressi | Expression Atlas Server

.../pub/expressionAtlas/app/v3/aa_reference_dataset/MtrunA17_Chr5g0422291

MtExpress V3 | Core (20220901)

! Switch to another dataset using the left menu

TMM | METADATA | SYNONYMOUS | ANNOTATION | GENOME PORTAL | LEGOO

rRNA: MtrunA17_Chr5g0422291 ;

Log2 TMM Normalisation using EdgeR (Core [20220901])

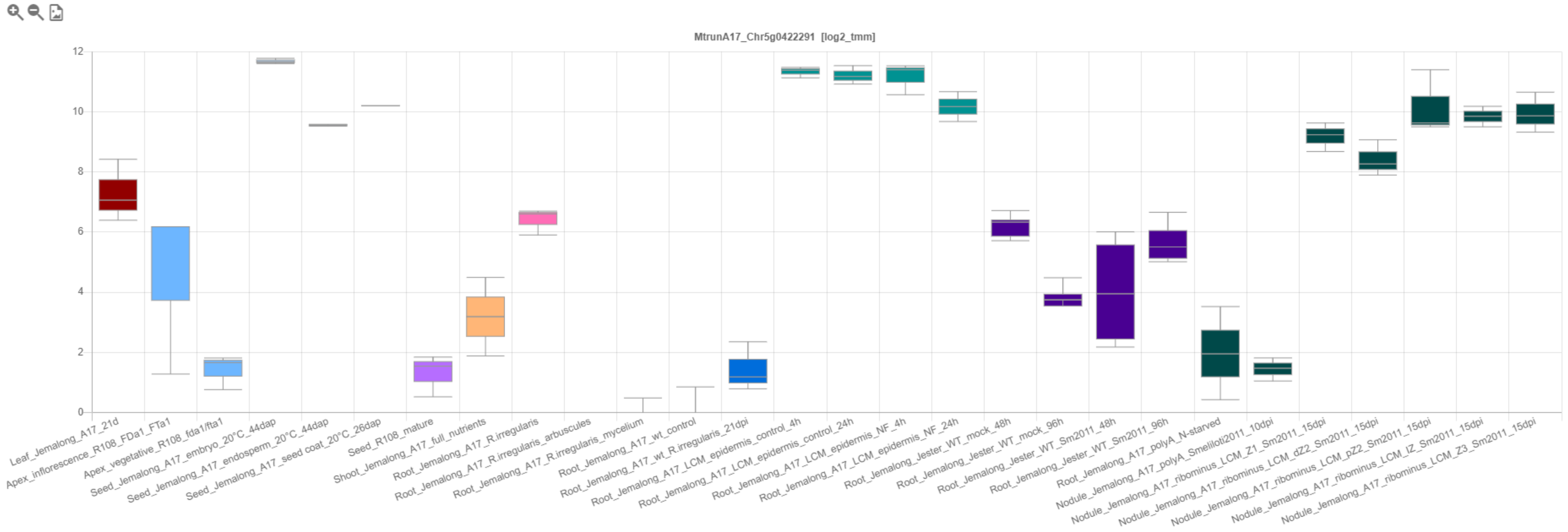

CP90: MtrunA17_Chr5g0430341

INRAE/CNRS Medicago Expressi | Expression Atlas Server

.../pub/expressionAtlas/app/v3/aa_reference_dataset/MtrunA17_Chr5g0430341

MtExpress V3 | Core (20220901)

! Switch to another dataset using the left menu

TMM | METADATA | SYNONYMOUS | ANNOTATION | GENOME PORTAL | LEGOO

mRNA: MtrunA17_Chr5g0430341;

Log2 TMM Normalisation using EdgeR (Core [20220901])

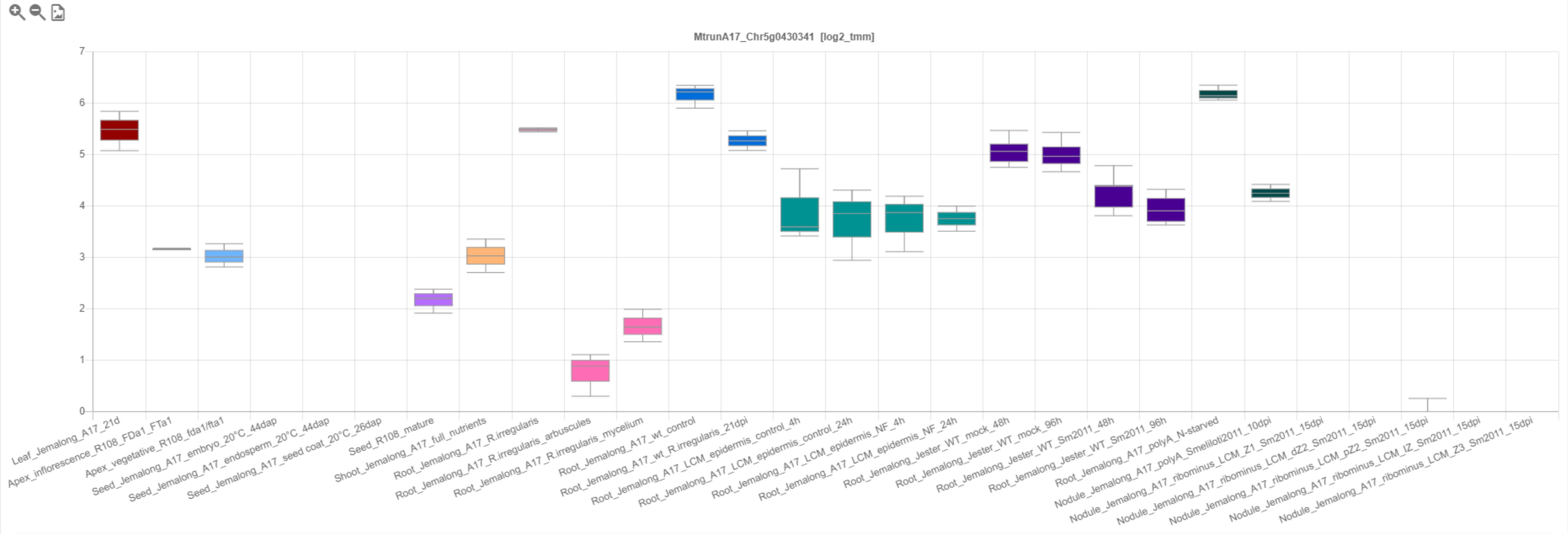

CP91: MtrunA17_Chr5g0430341

INRAE/CNRS Medicago Expressi
Expression Atlas Server
…pub/expressionAtlas/app/v3/aa_reference_dataset/MtrunA17_Chr5g0430341
MtExpress V3
Core (20220901)
Switch to another dataset using the left menu
lipme INRAE cnrs
TMM METADATA SYNONYMOUS ANNOTATION GENOME PORTAL LEGOO

mRNA: MtrunA17_Chr5g0430341;

Log2 TMM Normalisation using EdgeR (Core [20220901])

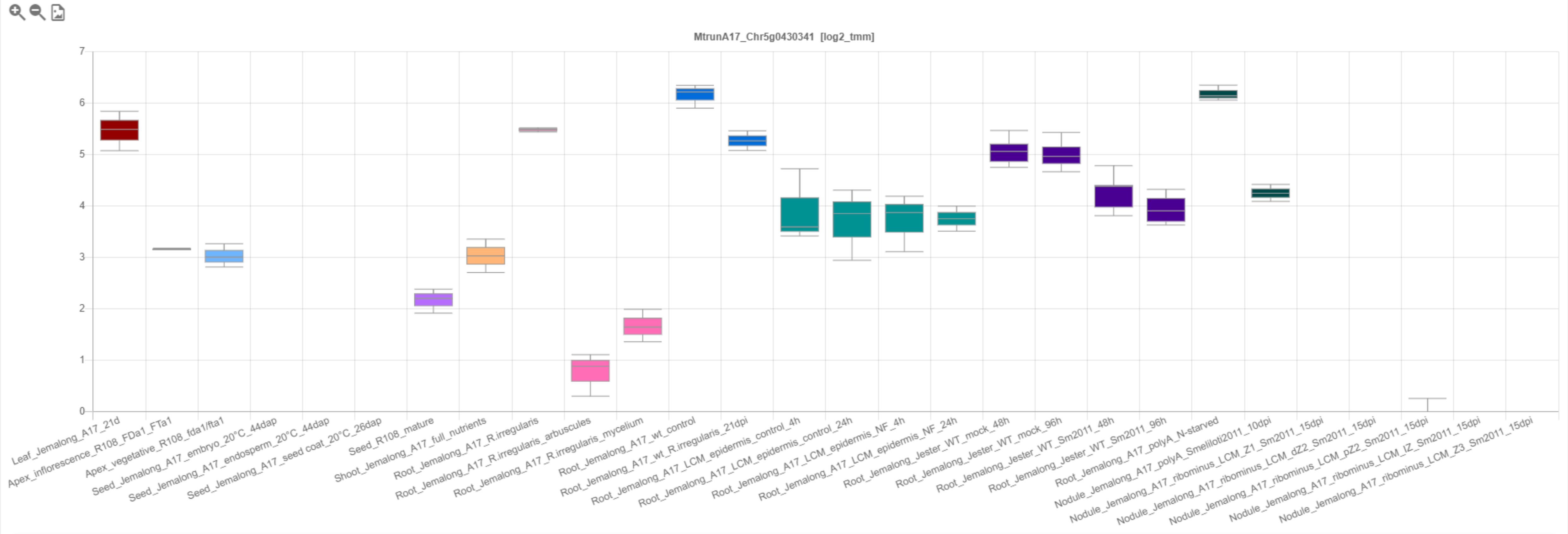

CP92: MtrunA17_Chr5g0431401

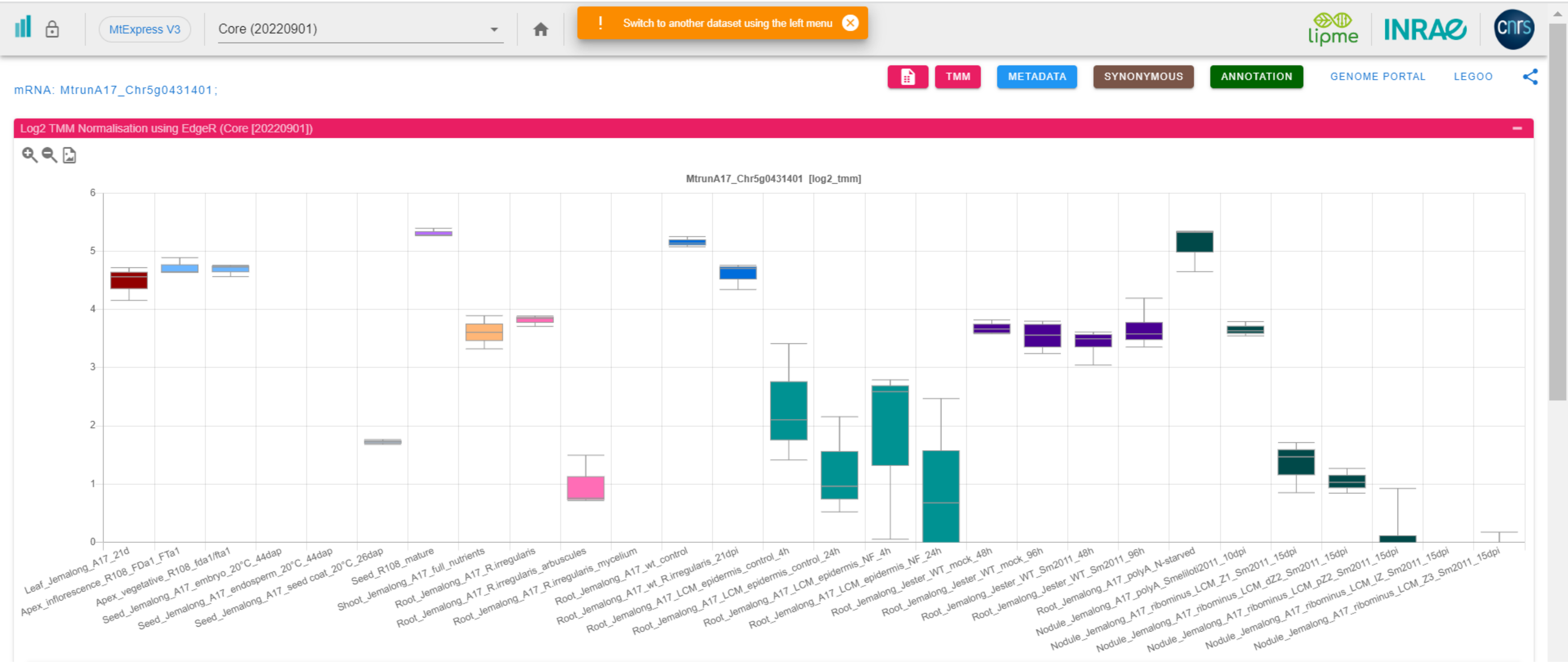

CP93: MtrunA17_Chr5g0435191

INRAE/CNRS Medicago Expressi | Expression Atlas Server

.../pub/expressionAtlas/app/v3/aa_reference_dataset/MtrunA17_Chr5g0435191

MtExpress V3 | Core (20220901)

! Switch to another dataset using the left menu

TMM | METADATA | SYNONYMOUS | ANNOTATION | GENOME PORTAL | LEGOO

mRNA: MtrunA17_Chr5g0435191 ;

Log2 TMM Normalisation using EdgeR (Core [20220901])

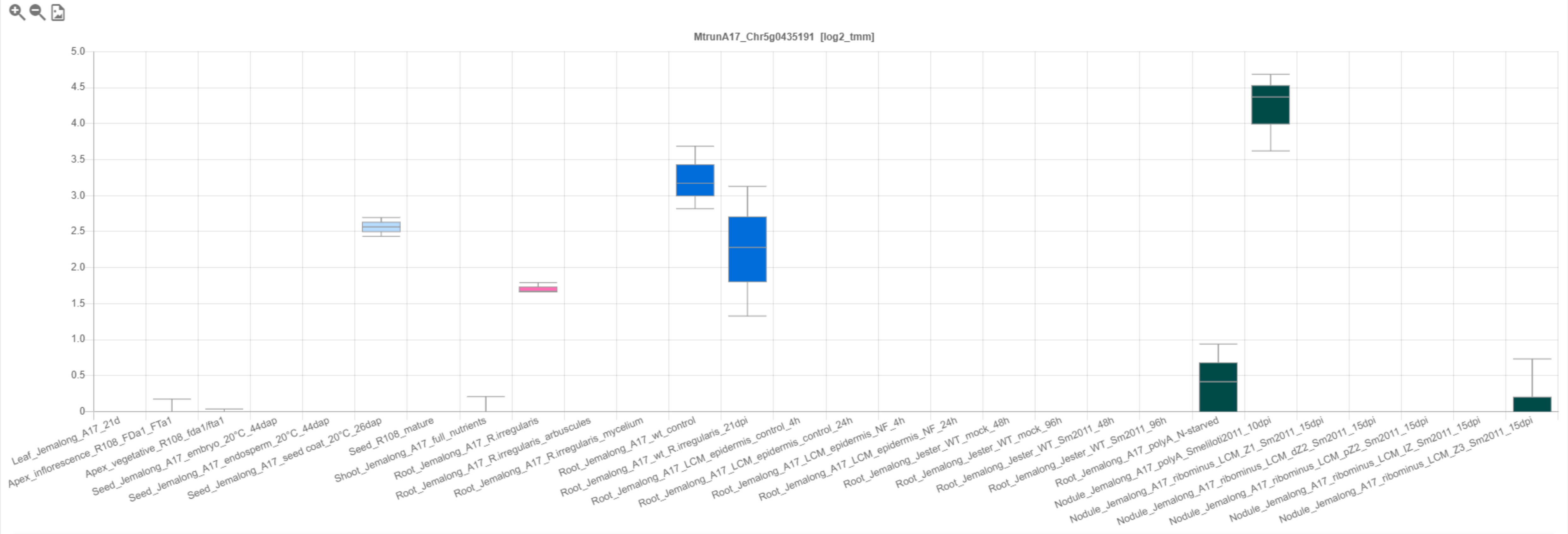

CP94: MtrunA17_Chr5g0444231

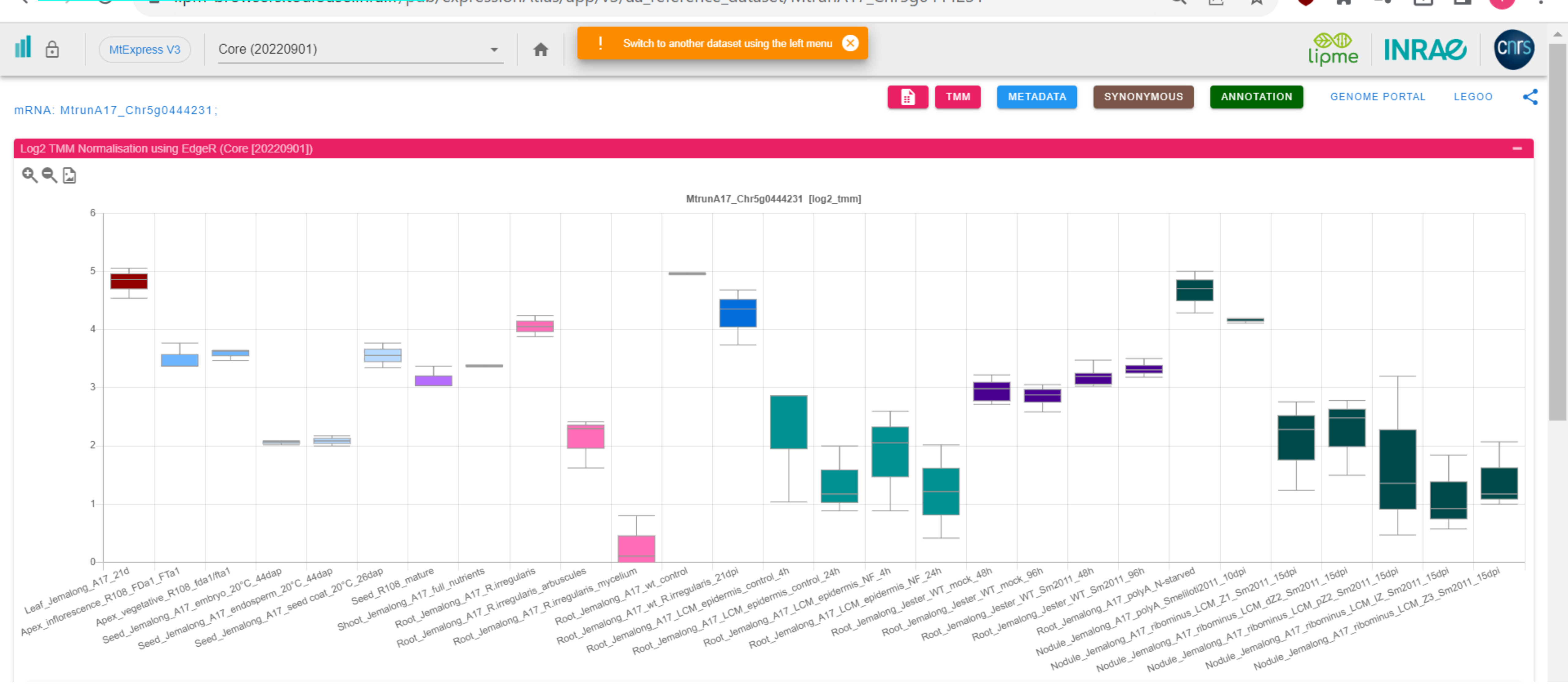

CP95: MtrunA17_Chr6g0451601

INRAE/CNRS Medicago Expressi… | Expression Atlas Server

…/pub/expressionAtlas/app/v3/aa_reference_dataset/MtrunA17_Chr6g0451601

MtExpress V3 | Core (20220901)

! Switch to another dataset using the left menu

TMM | METADATA | SYNONYMOUS | ANNOTATION | GENOME PORTAL | LEGOO

mRNA: MtrunA17_Chr6g0451601;

Log2 TMM Normalisation using EdgeR (Core [20220901])

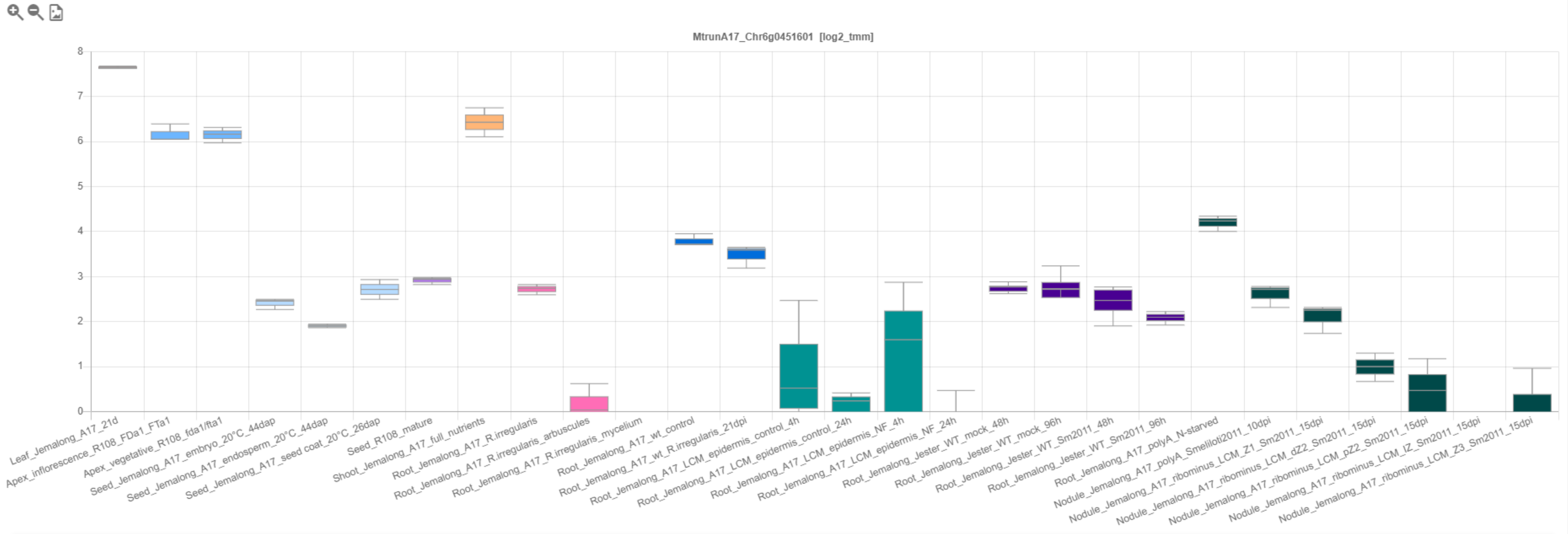

CP96: MtrunA17_Chr6g0452781

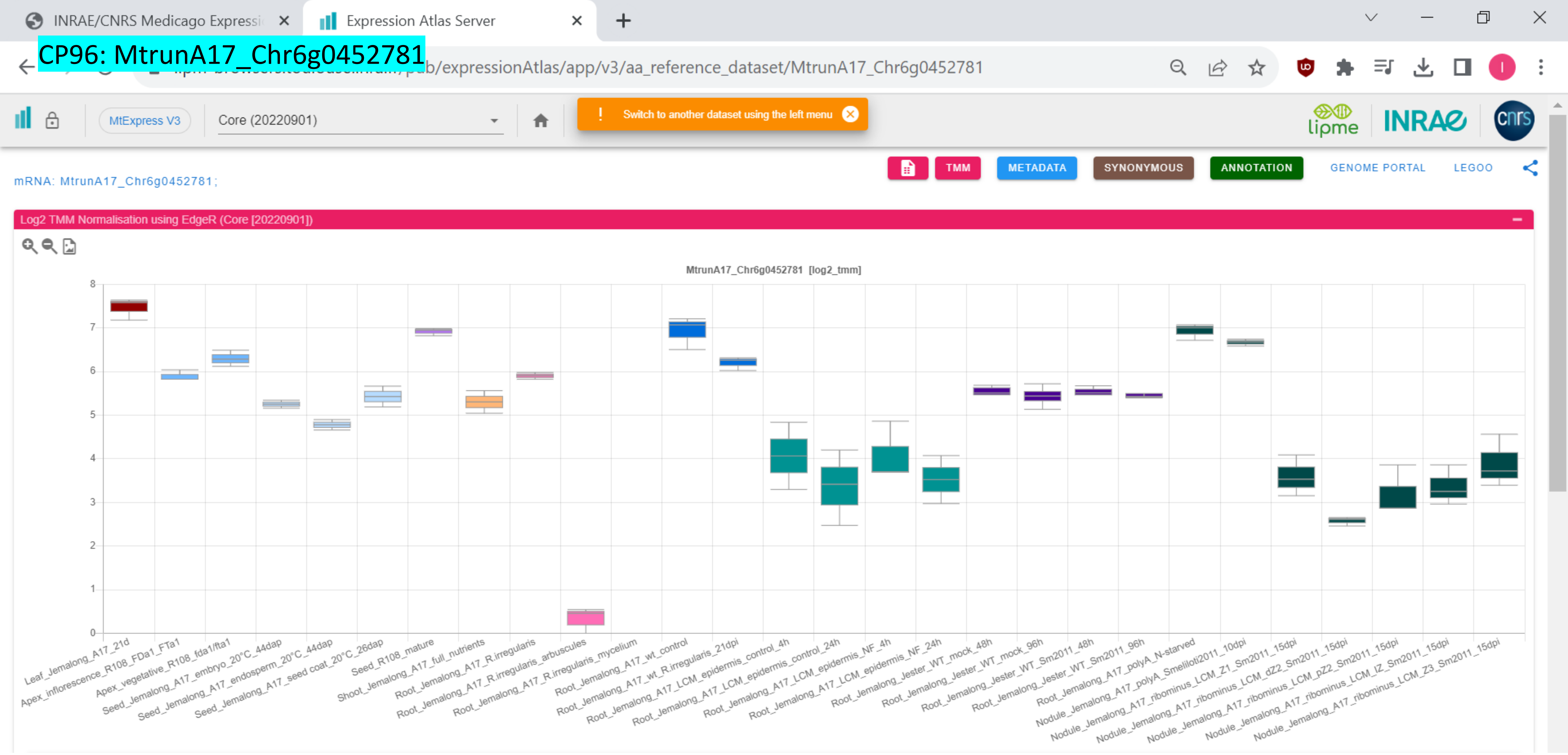

CP97: MtrunA17_Chr6g0457351

INRAE/CNRS Medicago Expressi | Expression Atlas Server

[illegible]/pub/expressionAtlas/app/v3/aa_reference_dataset/MtrunA17_Chr6g0457351

MtExpress V3 | Core (20220901)

! Switch to another dataset using the left menu

TMM | METADATA | SYNONYMOUS | ANNOTATION | GENOME PORTAL | LEGOO

mRNA: MtrunA17_Chr6g0457351;

Log2 TMM Normalisation using EdgeR (Core [20220901])

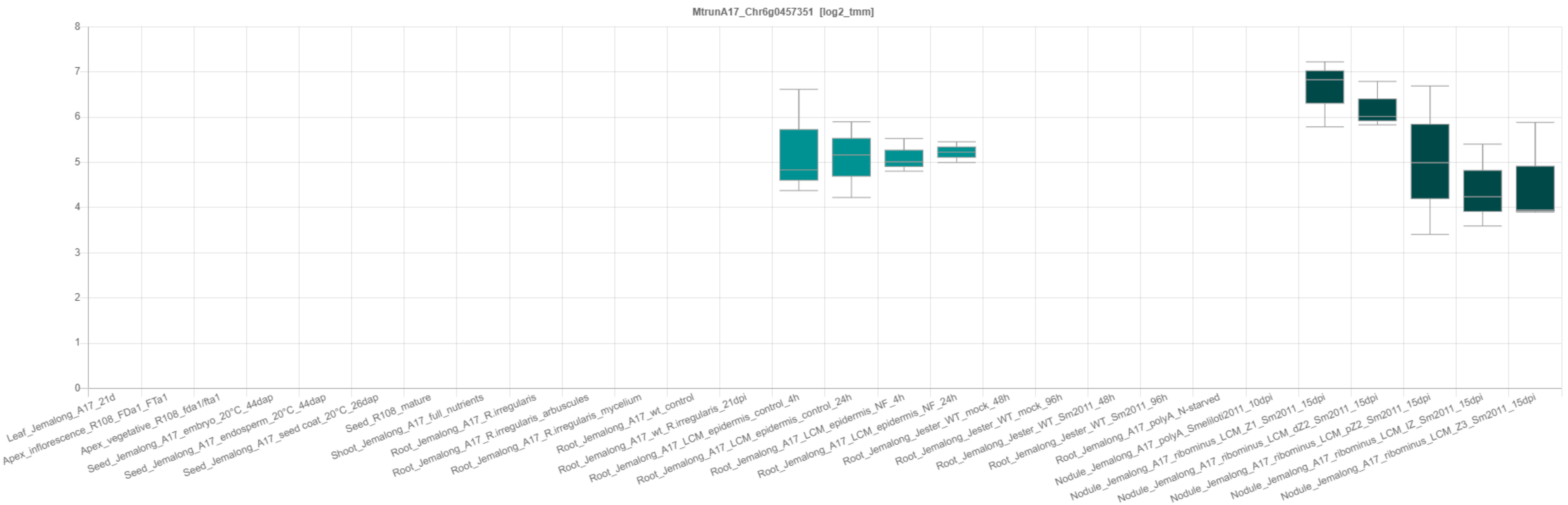

CP98: MtrunA17_Chr6g0457461

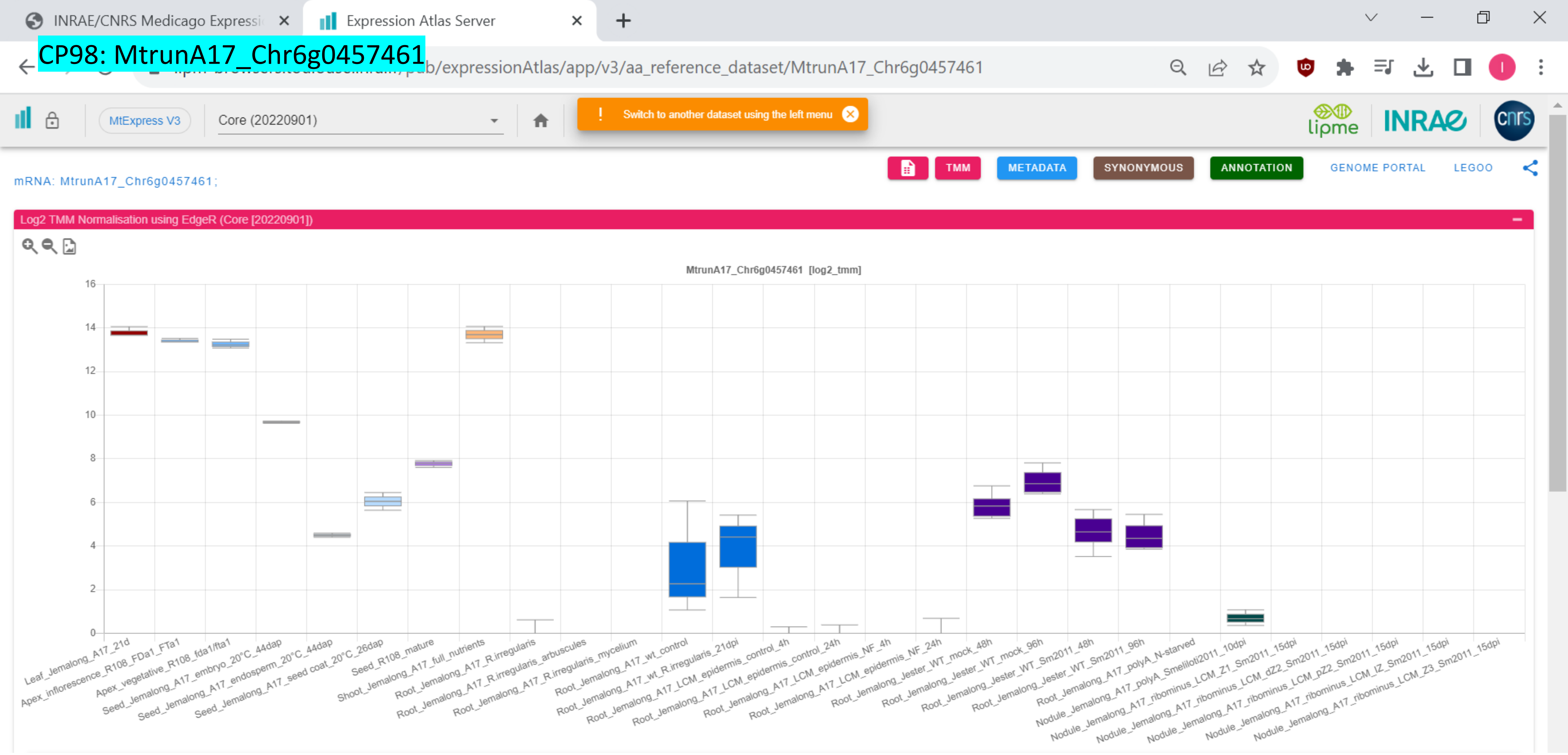

CP99: MtrunA17_Chr6g0457461

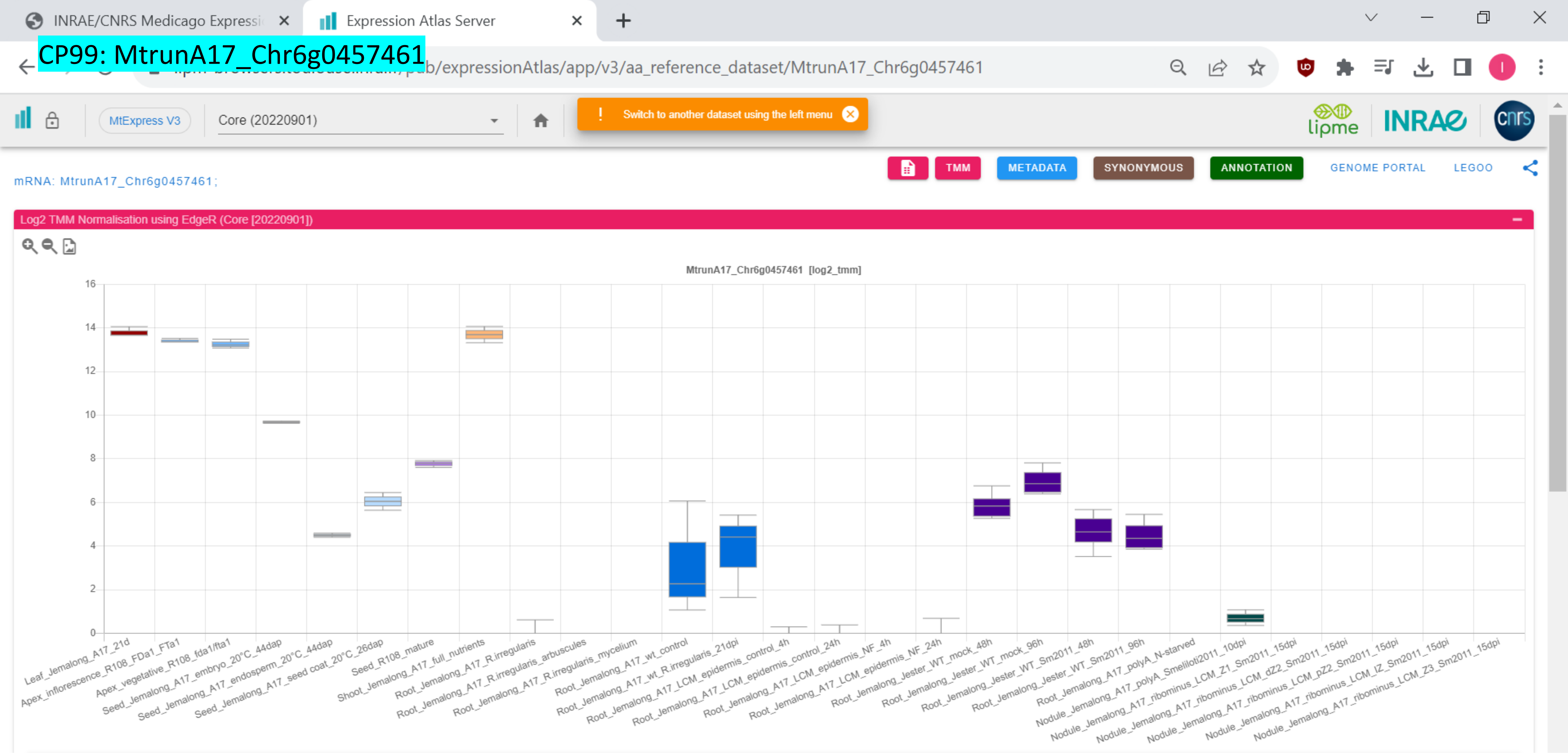

CP100: MtrunA17_Chr6g0457461

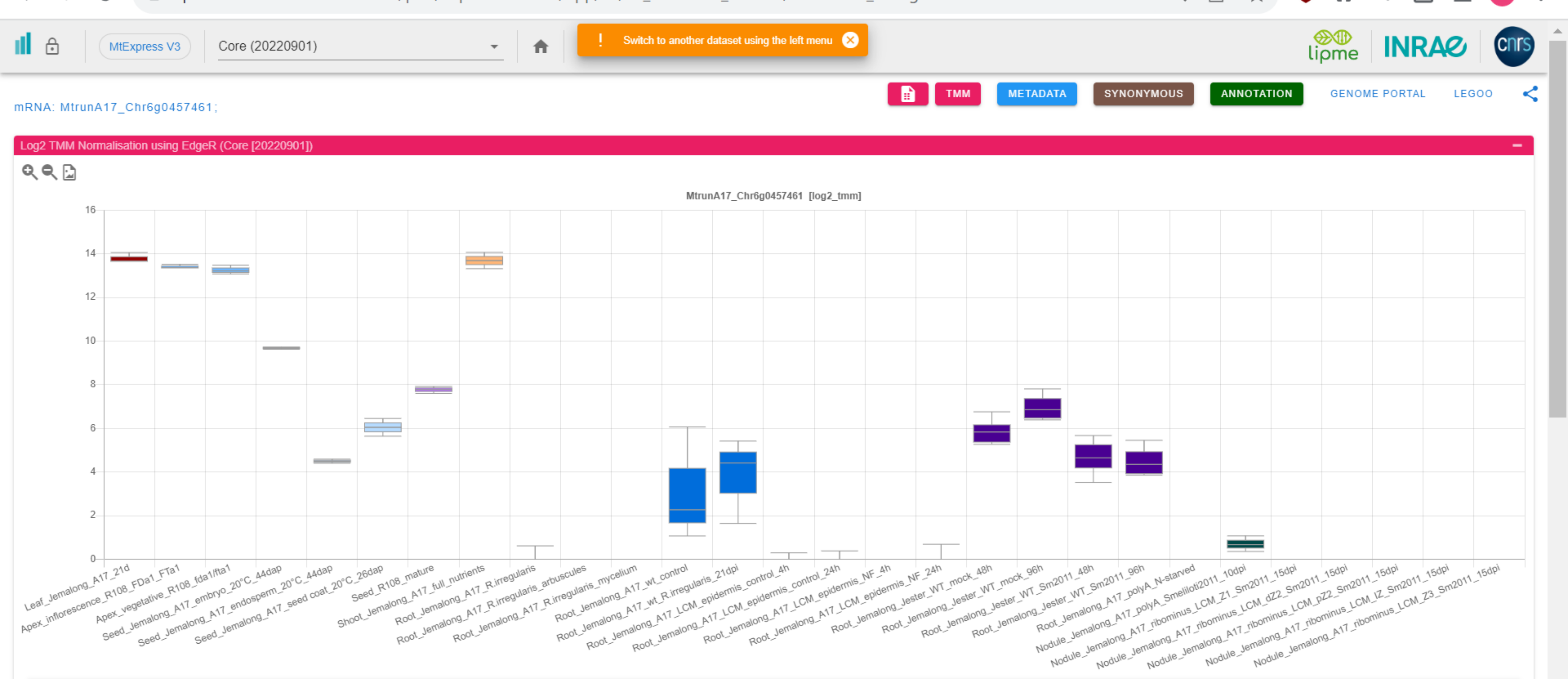

CP101: MtrunA17_Chr6g0458091

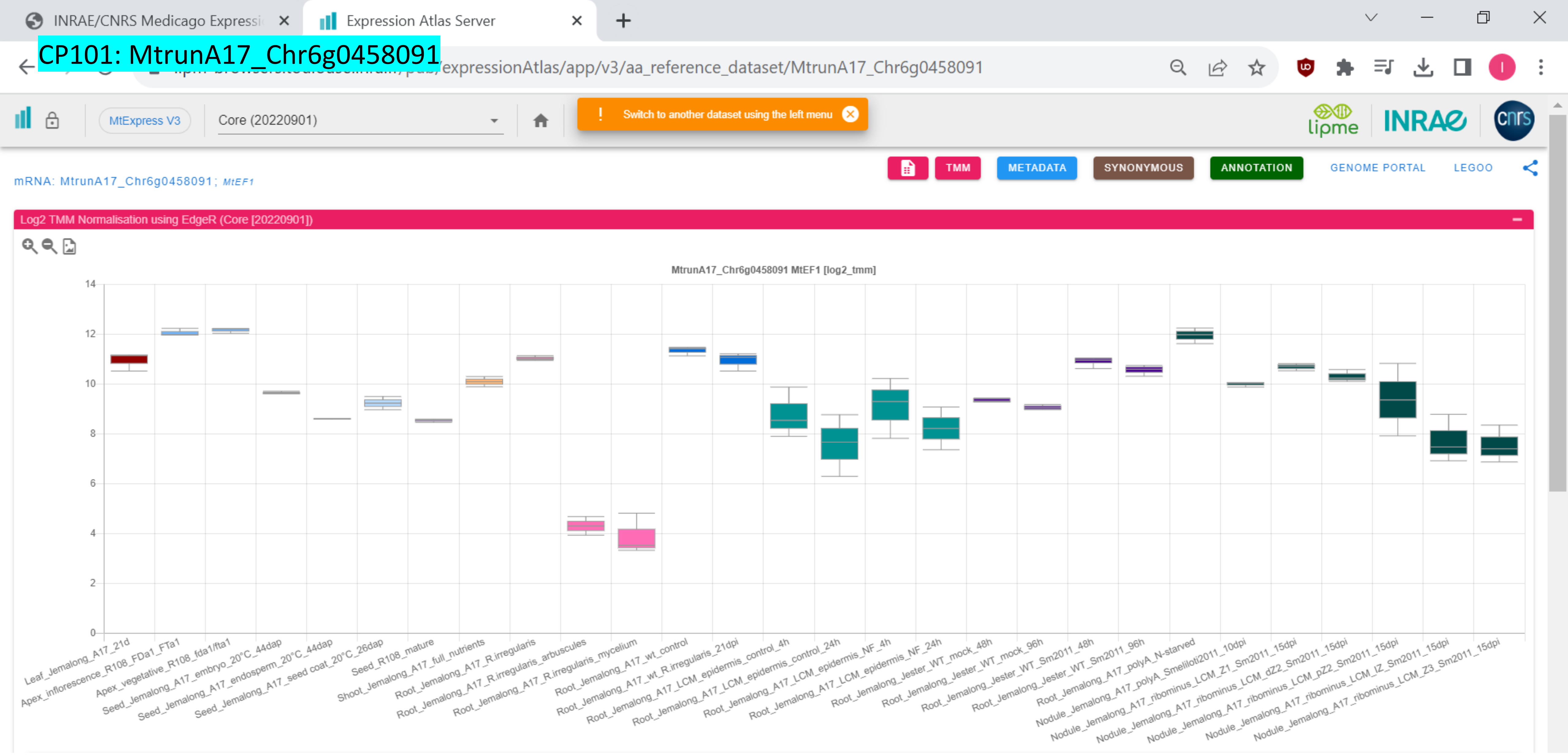

*CP102: MtrunA17_Chr6g0459481

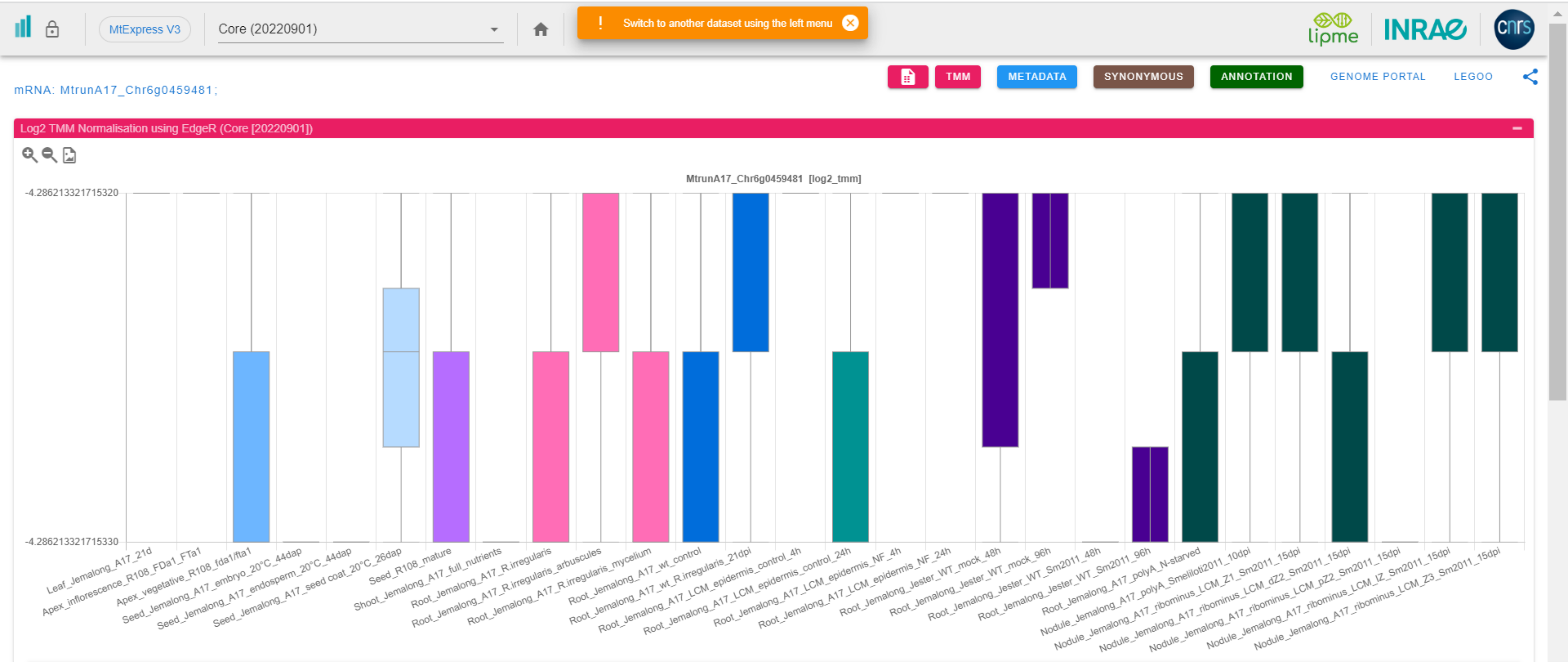

CP103: MtrunA17_Chr6g0461931

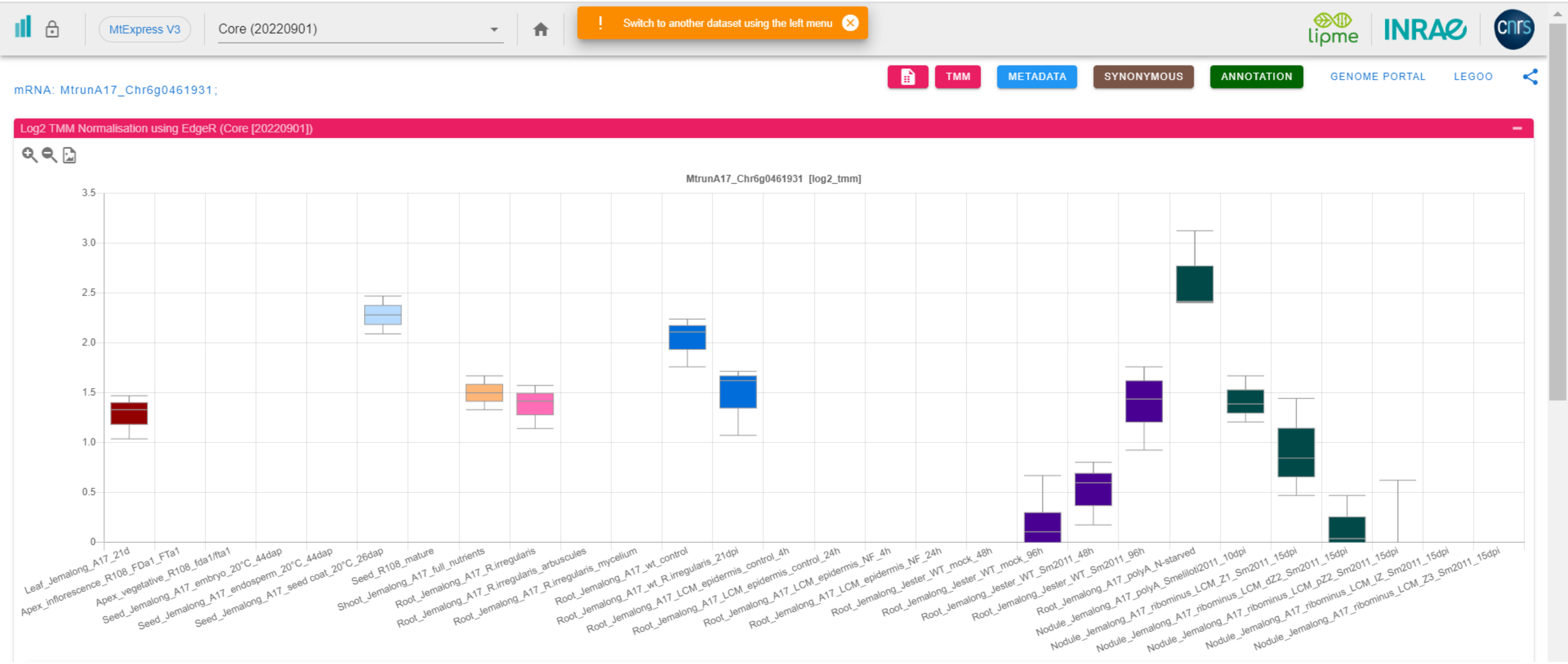

CP104: MtrunA17_Chr6g0462271

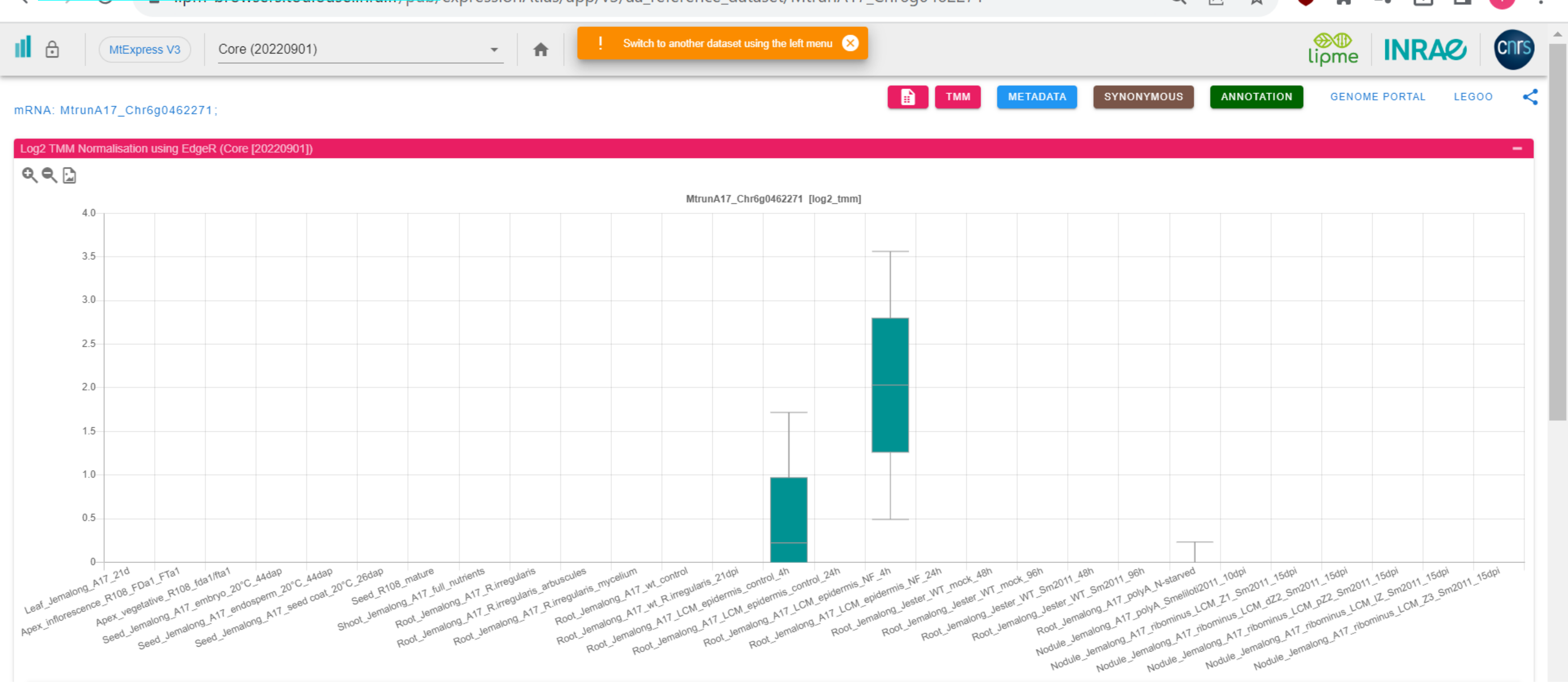

*CP105: MtrunA17_Chr6g0468411

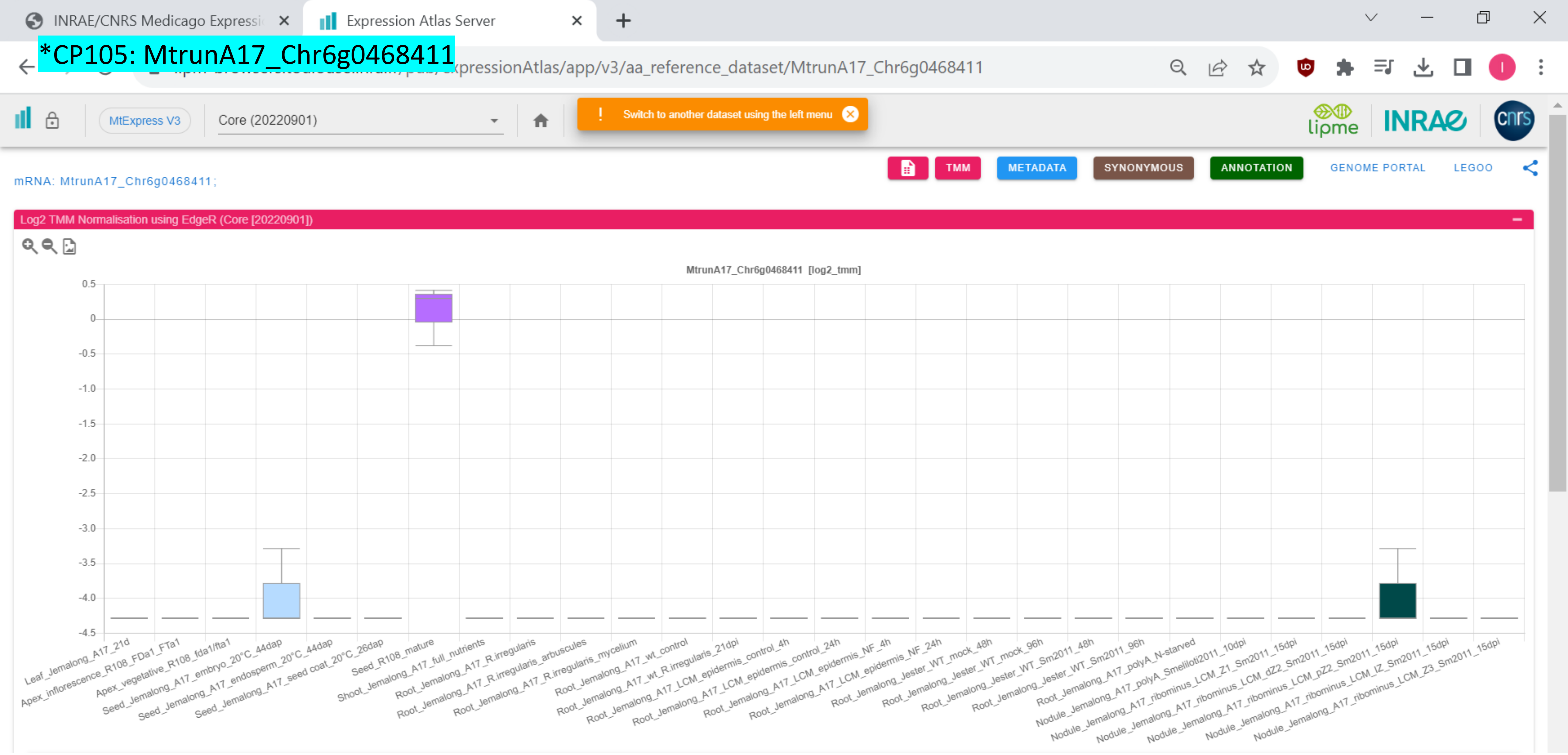

CP106: MtrunA17_Chr6g0476751

INRAE/CNRS Medicago Expressi… | Expression Atlas Server

…/expressionAtlas/app/v3/aa_reference_dataset/MtrunA17_Chr6g0476751

MtExpress V3 | Core (20220901)

! Switch to another dataset using the left menu

TMM | METADATA | SYNONYMOUS | ANNOTATION | GENOME PORTAL | LEGOO

mRNA: MtrunA17_Chr6g0476751;

Log2 TMM Normalisation using EdgeR (Core [20220901])

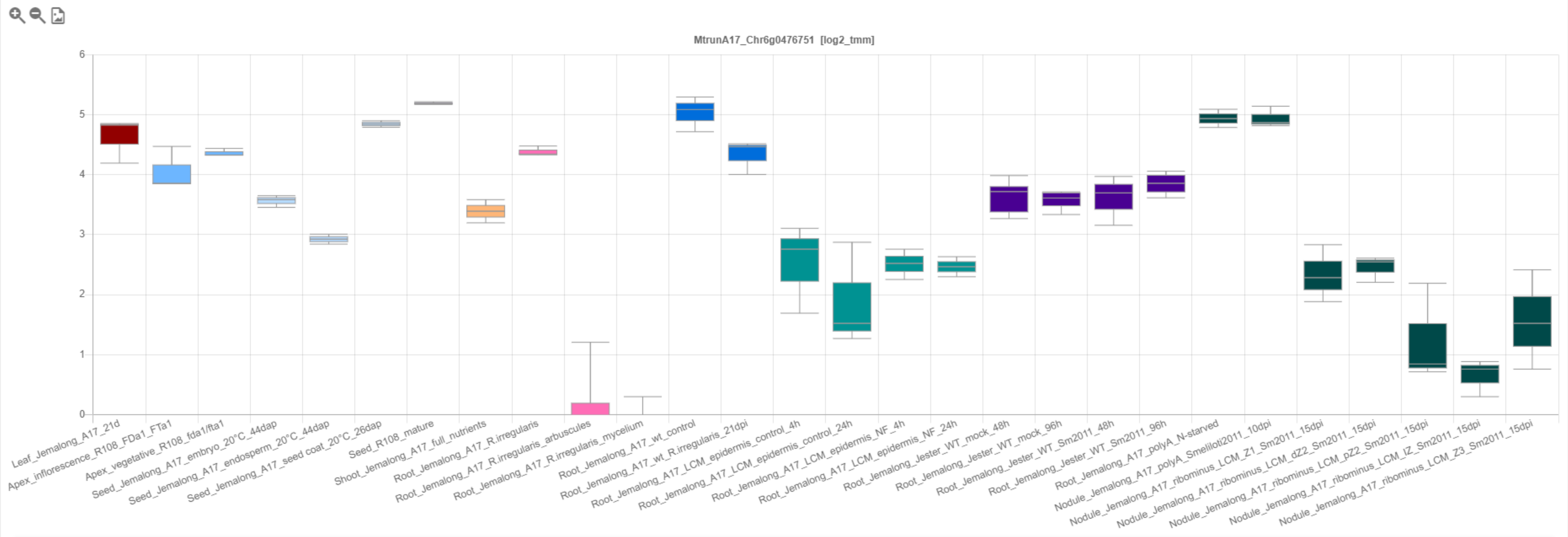

CP107: MtrunA17_Chr6g0479001

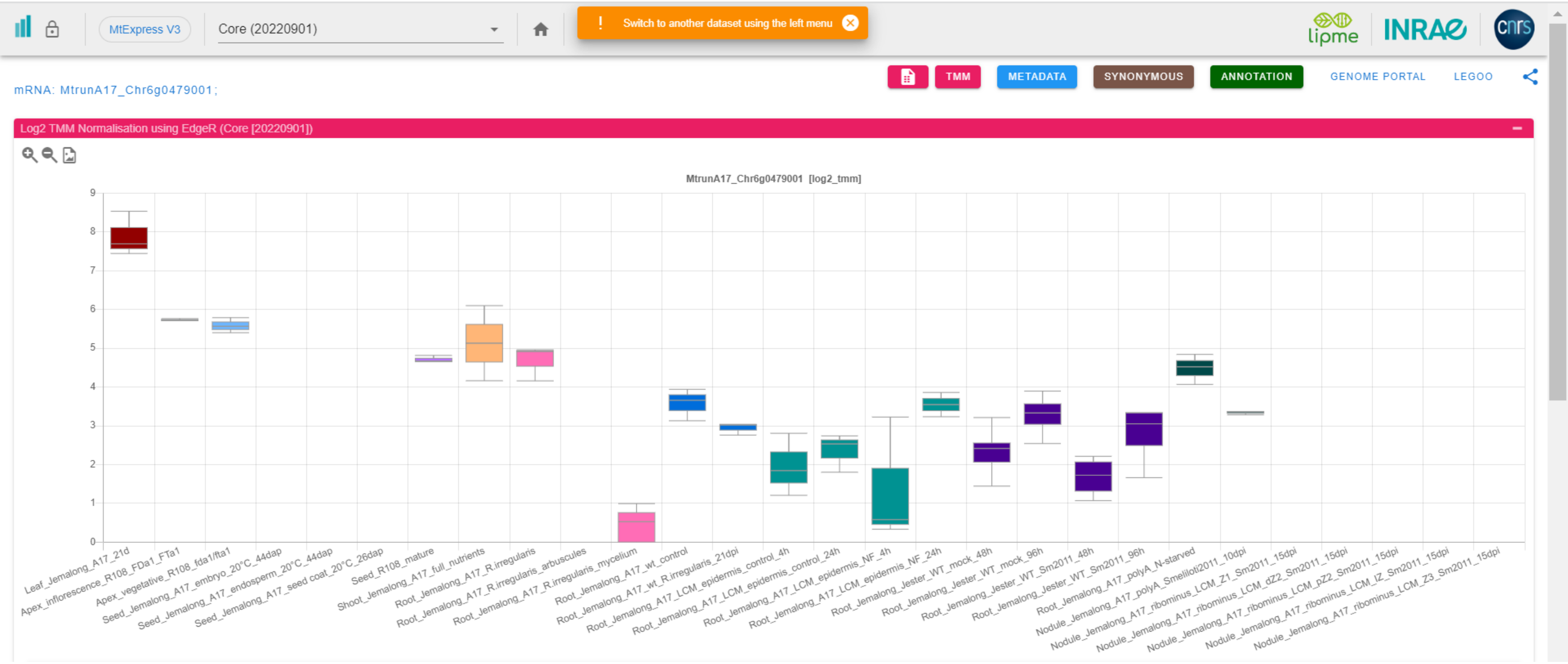

CP108: MtrunA17_Chr6g0485321

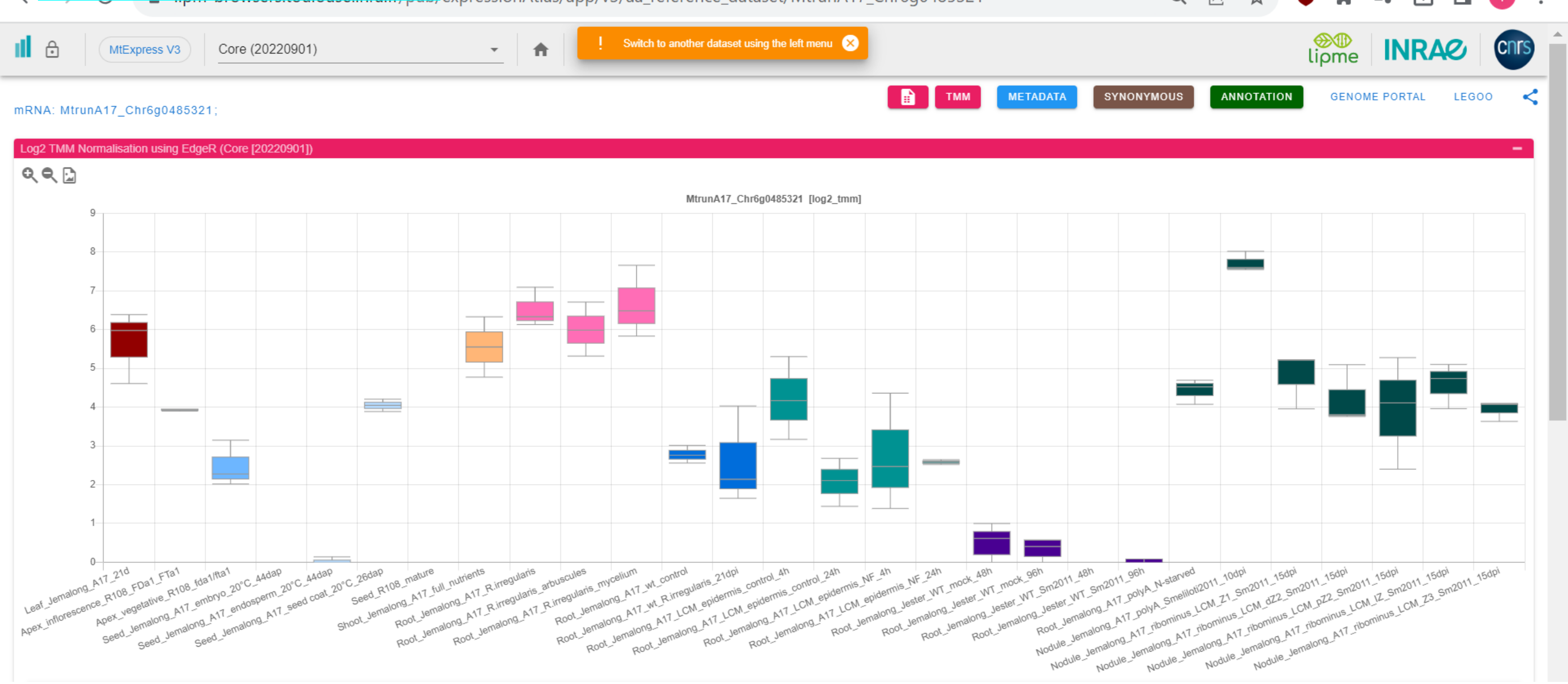

CP109: MtrunA17_Chr6g0486961

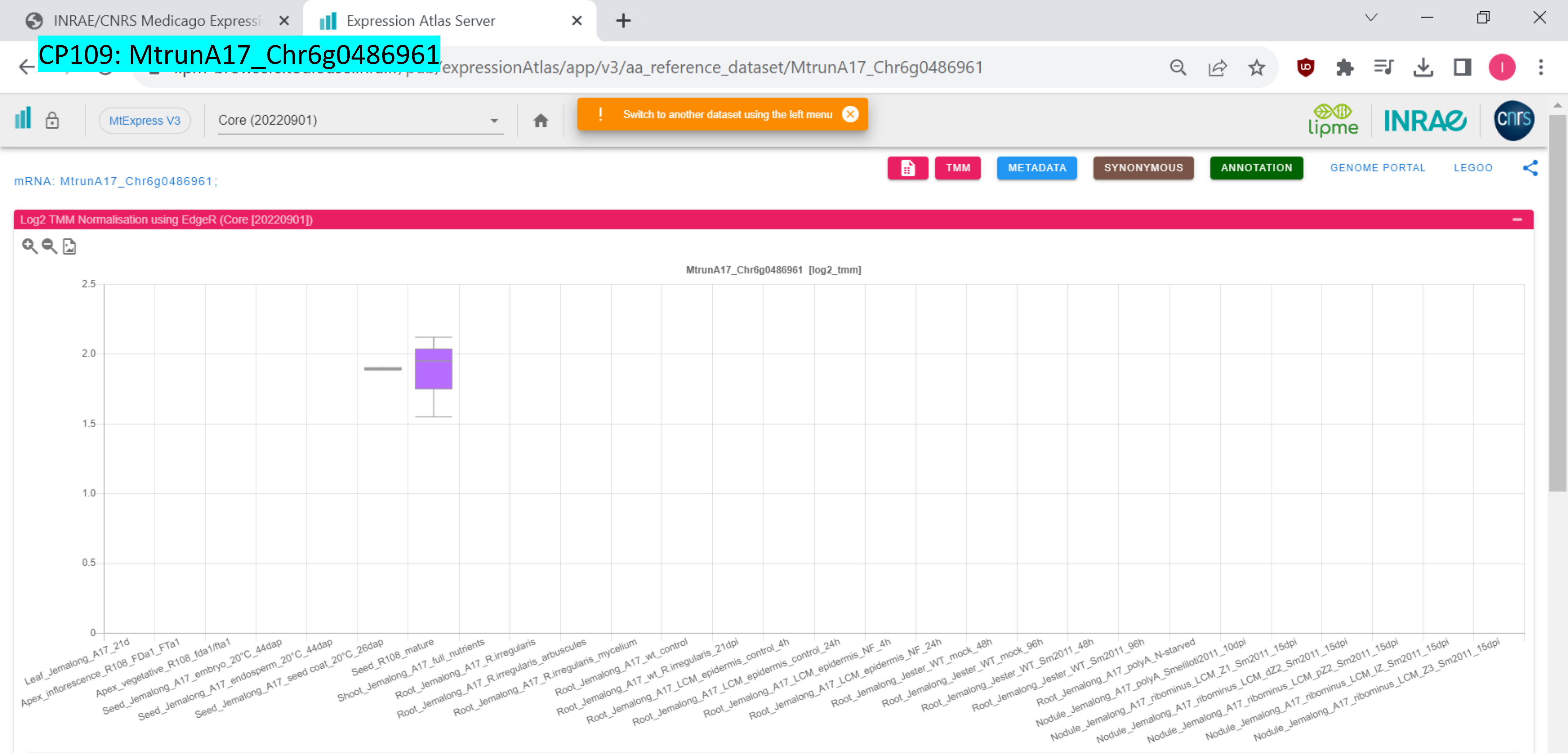

CP110: MtrunA17_Chr7g0214741

mRNA: MtrunA17_Chr7g0214741;

Log2 TMM Normalisation using EdgeR (Core [20220901])

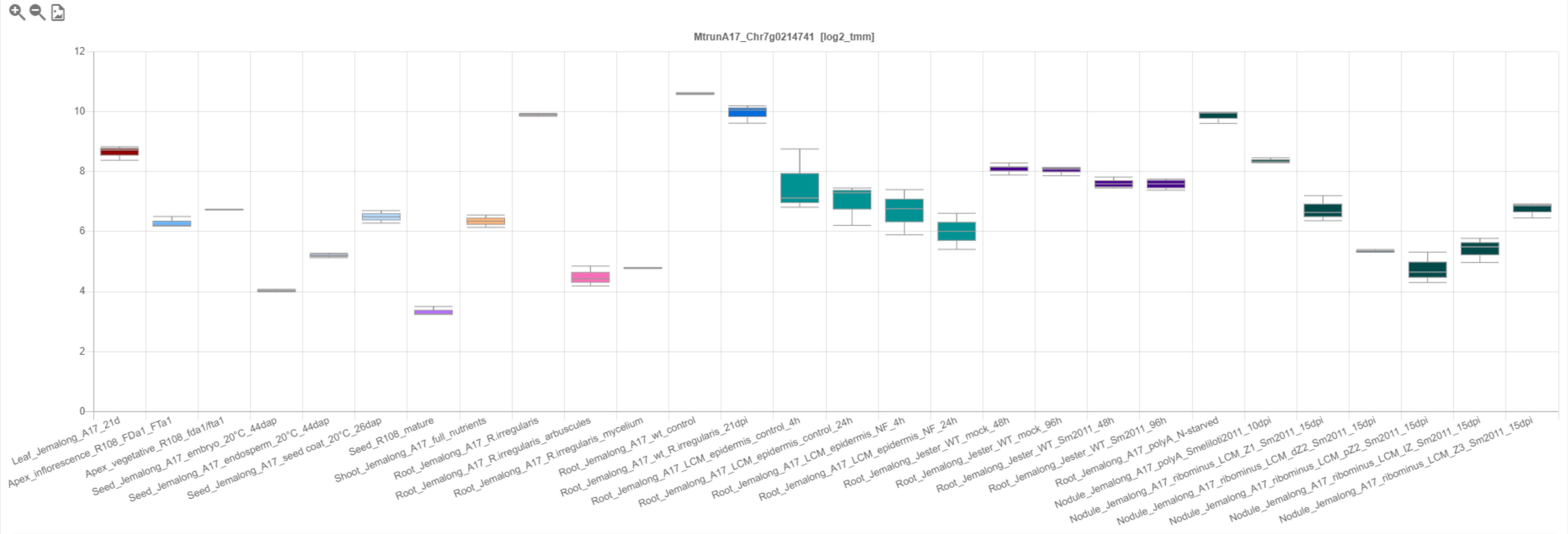

CP111: MtrunA17_Chr7g0214911

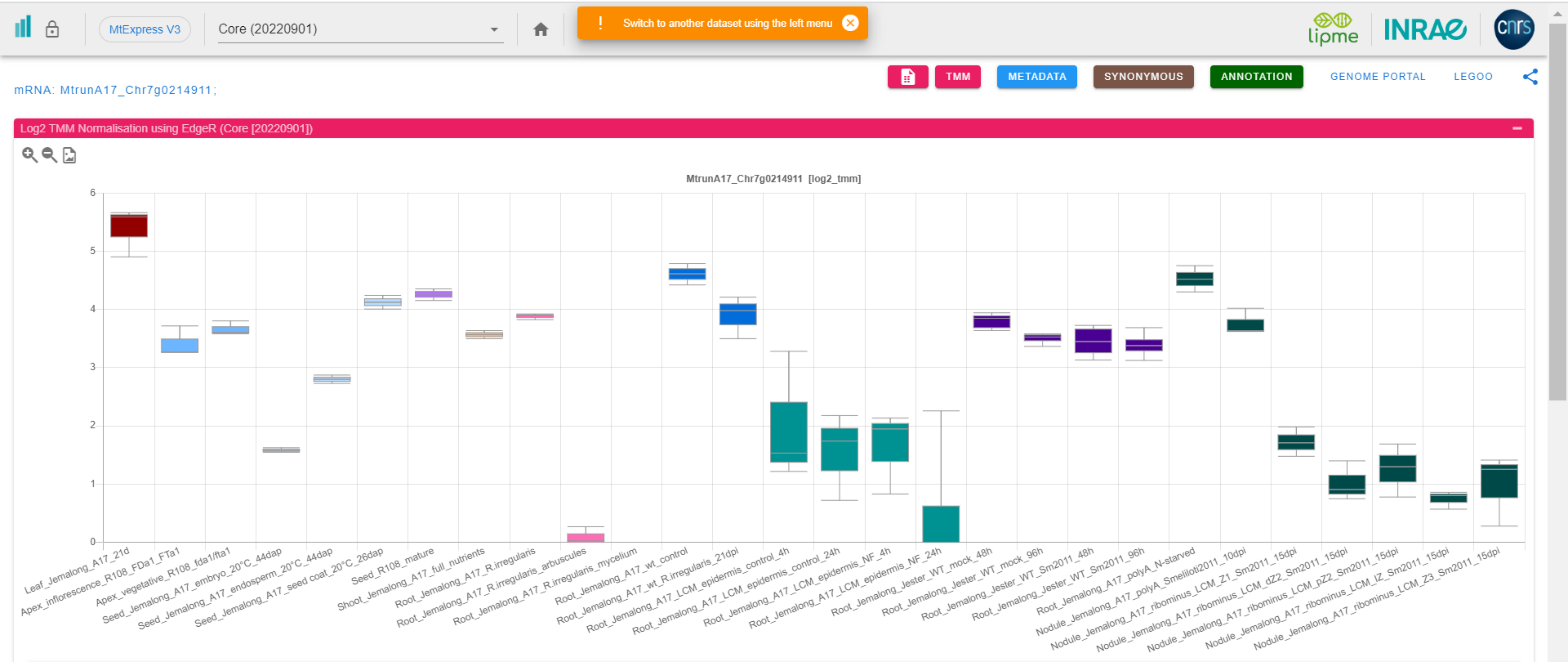

CP112: MtrunA17_Chr7g0219691

INRAE/CNRS Medicago Expressi | Expression Atlas Server

[illegible]/expressionAtlas/app/v3/aa_reference_dataset/MtrunA17_Chr7g0219691

MtExpress V3 | Core (20220901)

! Switch to another dataset using the left menu

lipme INRAE cnrs

TMM | METADATA | SYNONYMOUS | ANNOTATION | GENOME PORTAL | LEGOO

mRNA: MtrunA17_Chr7g0219691;

Log2 TMM Normalisation using EdgeR (Core [20220901])

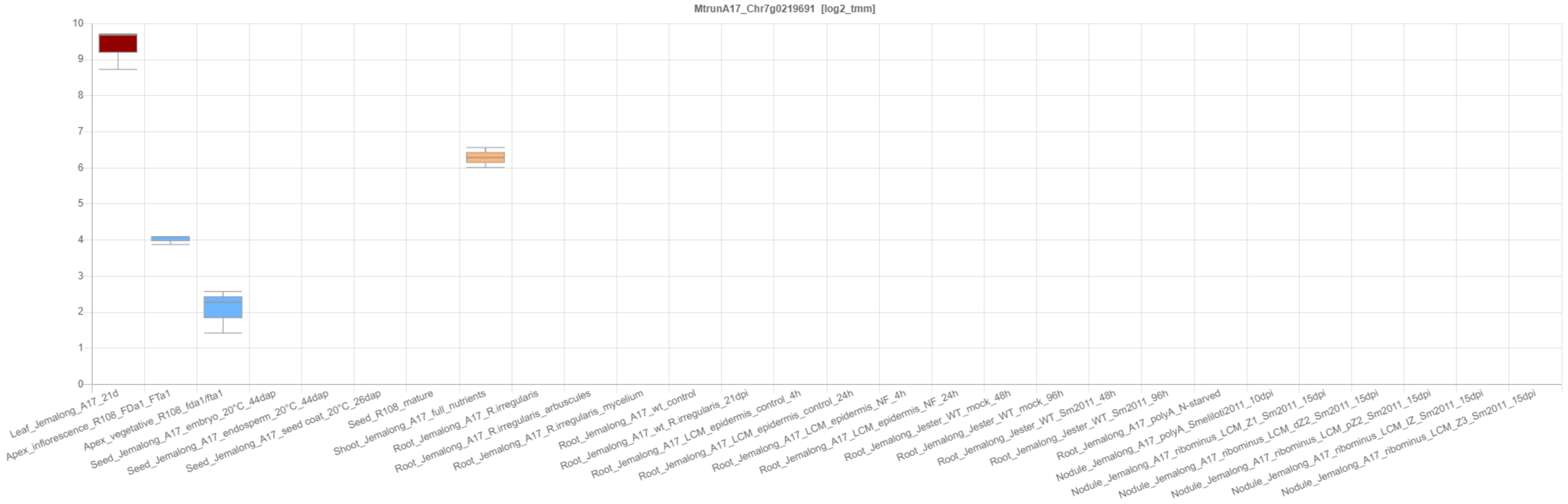

*CP113: MtrunA17_Chr7g0221631

INRAE/CNRS Medicago Expressi | Expression Atlas Server

...expressionAtlas/app/v3/aa_reference_dataset/MtrunA17_Chr7g0221631

MtExpress V3 | Core (20220901) | Switch to another dataset using the left menu

TMM | METADATA | SYNONYMOUS | ANNOTATION | GENOME PORTAL | LEGOO

mRNA: MtrunA17_Chr7g0221631;

Log2 TMM Normalisation using EdgeR (Core [20220901])

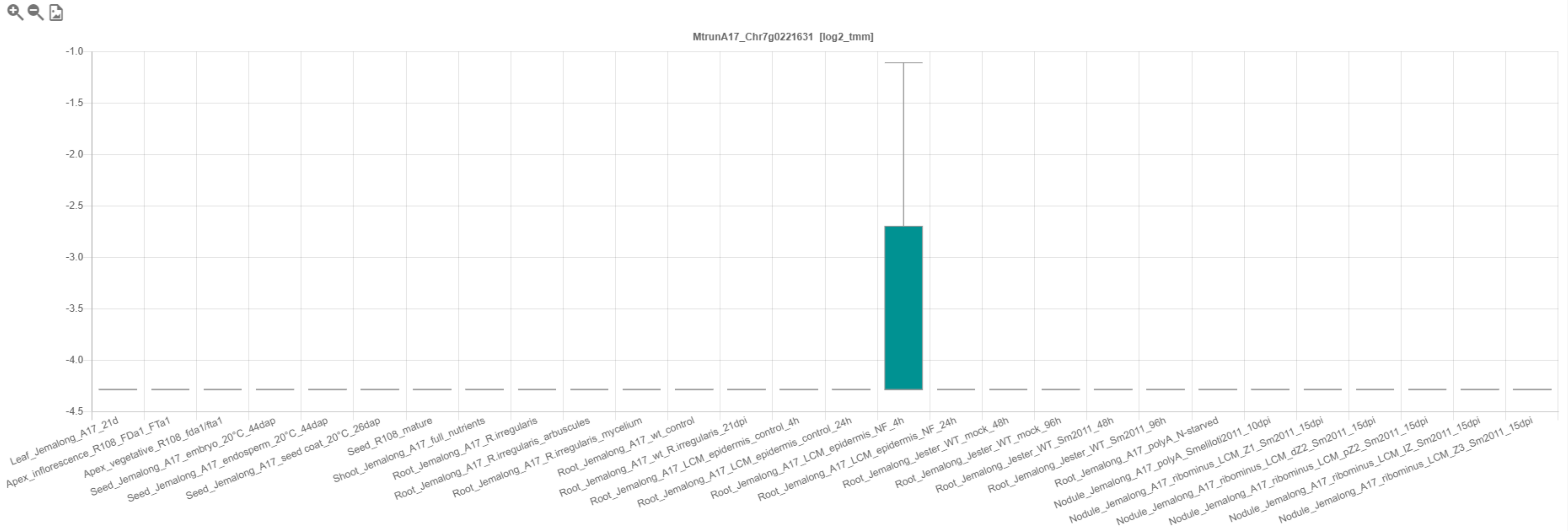

CP114: MtrunA17_Chr7g0229401

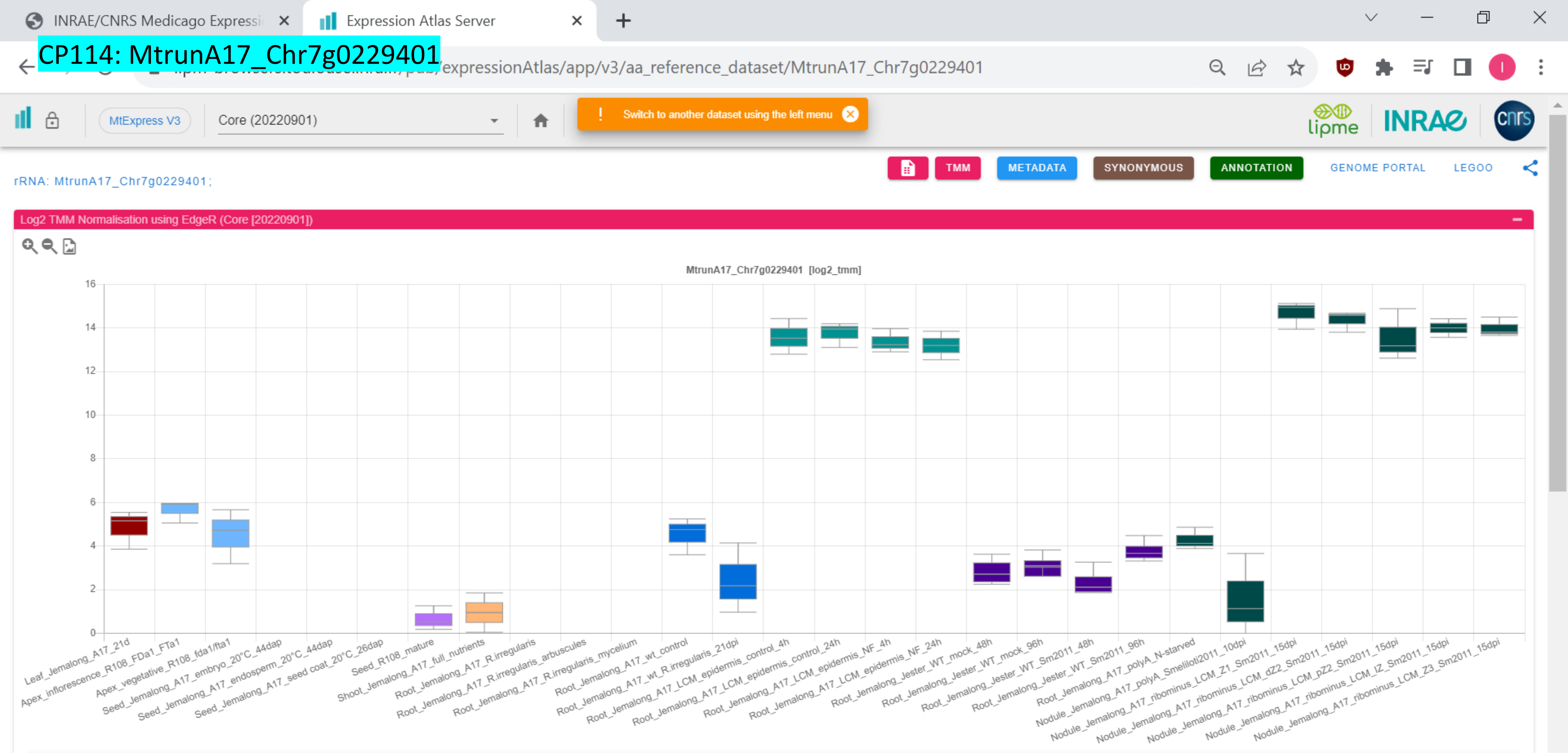

CP115: MtrunA17_Chr7g0230341

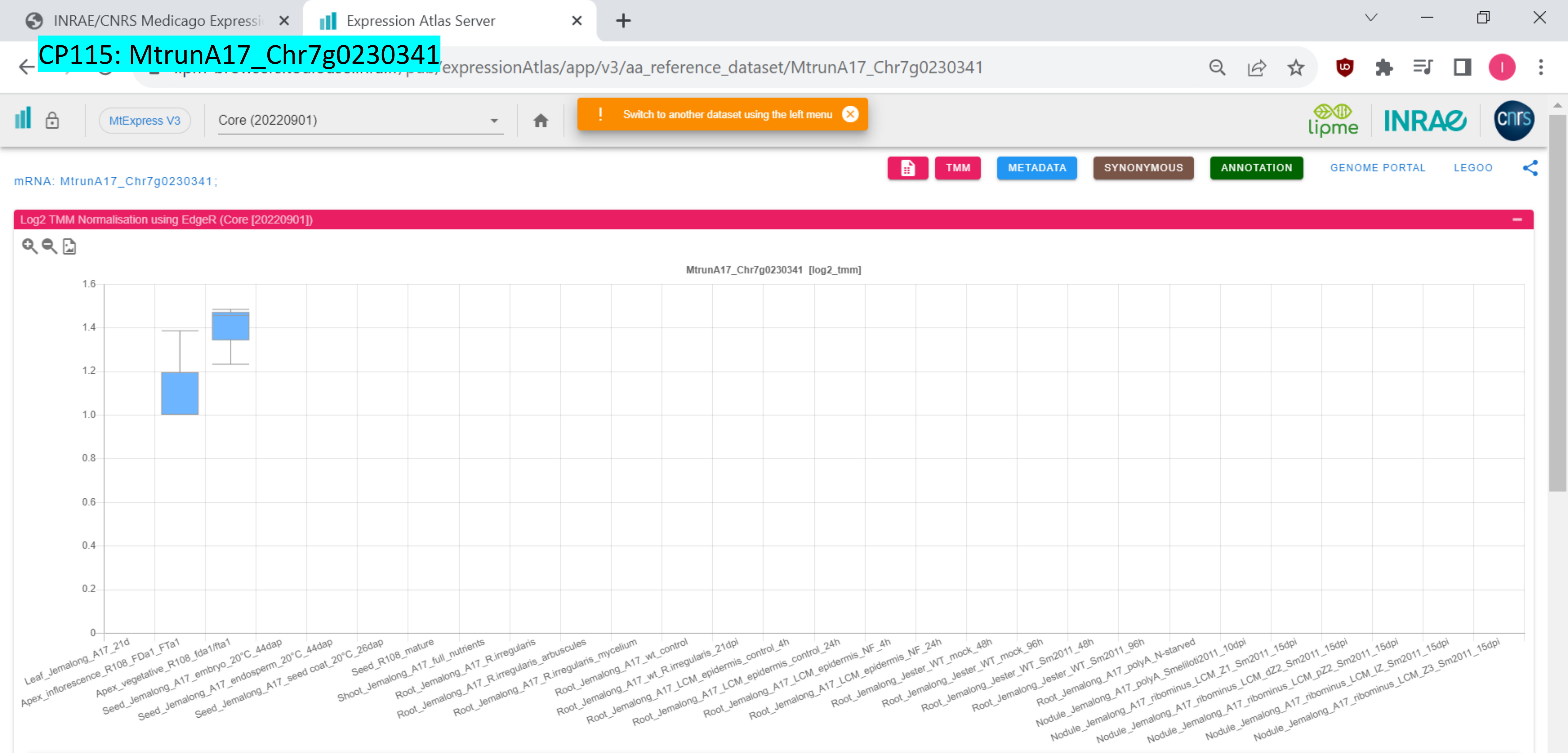

CP116: MtrunA17_Chr7g0232851

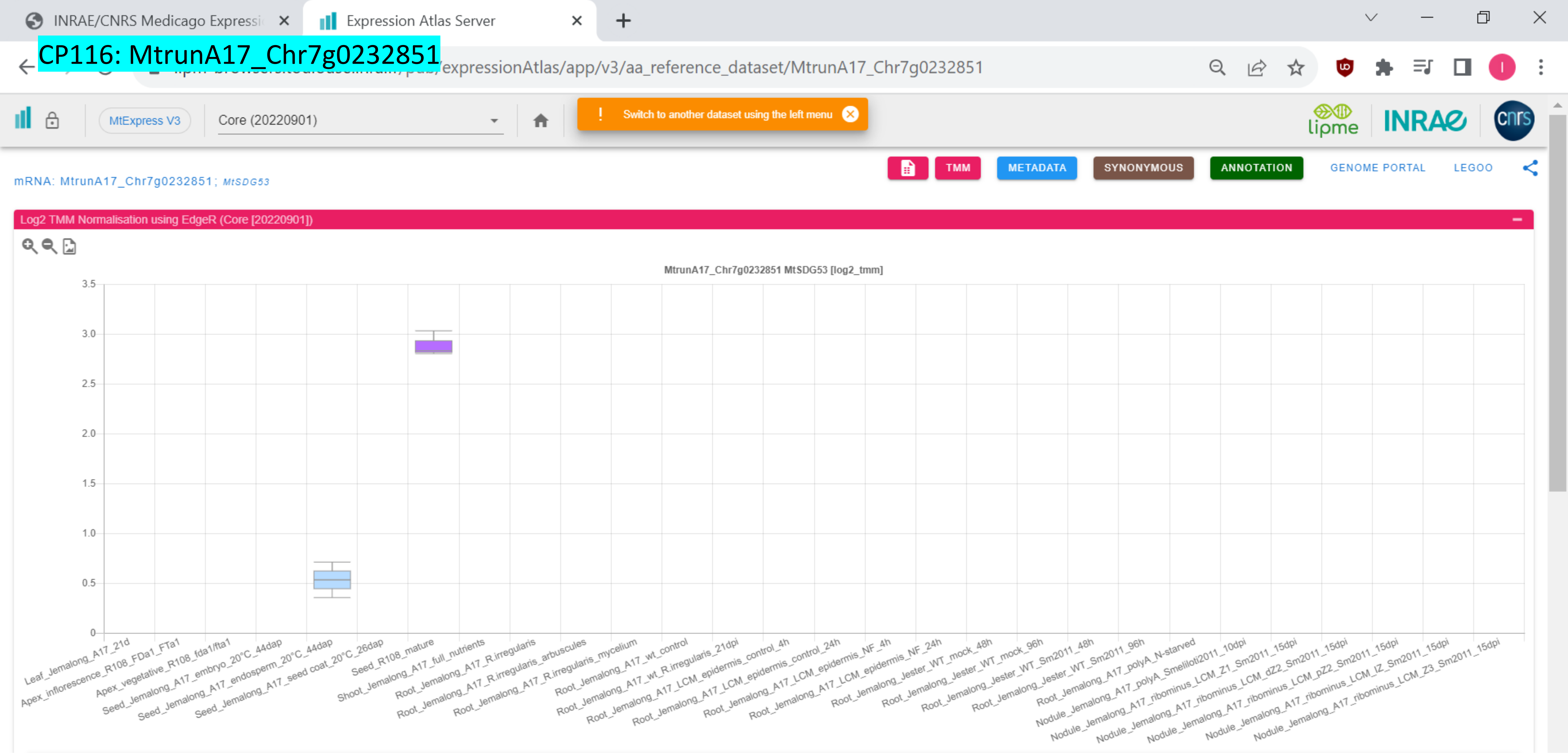

CP117: MtrunA17_Chr7g0237331

INRAE/CNRS Medicago Expressi | Expression Atlas Server

.../expressionAtlas/app/v3/aa_reference_dataset/MtrunA17_Chr7g0237331

MtExpress V3 | Core (20220901)

! Switch to another dataset using the left menu

TMM | METADATA | SYNONYMOUS | ANNOTATION | GENOME PORTAL | LEGOO

mRNA: MtrunA17_Chr7g0237331;

Log2 TMM Normalisation using EdgeR (Core [20220901])

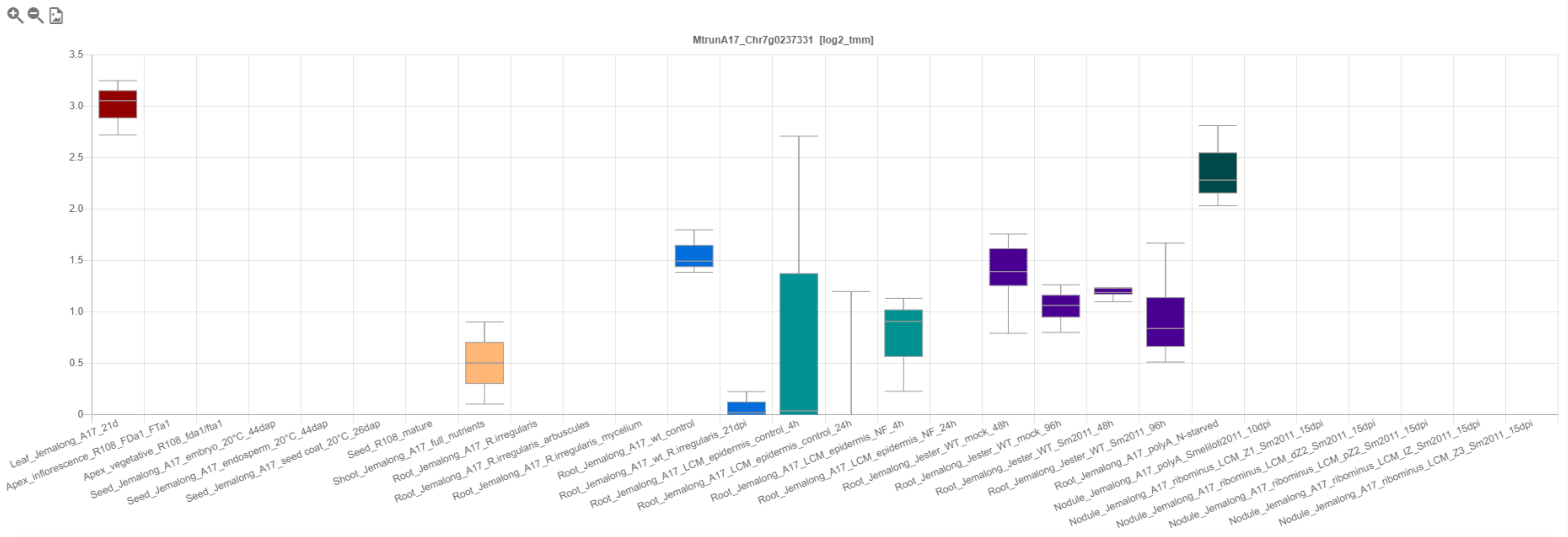

*CP118: MtrunA17_Chr7g0251971

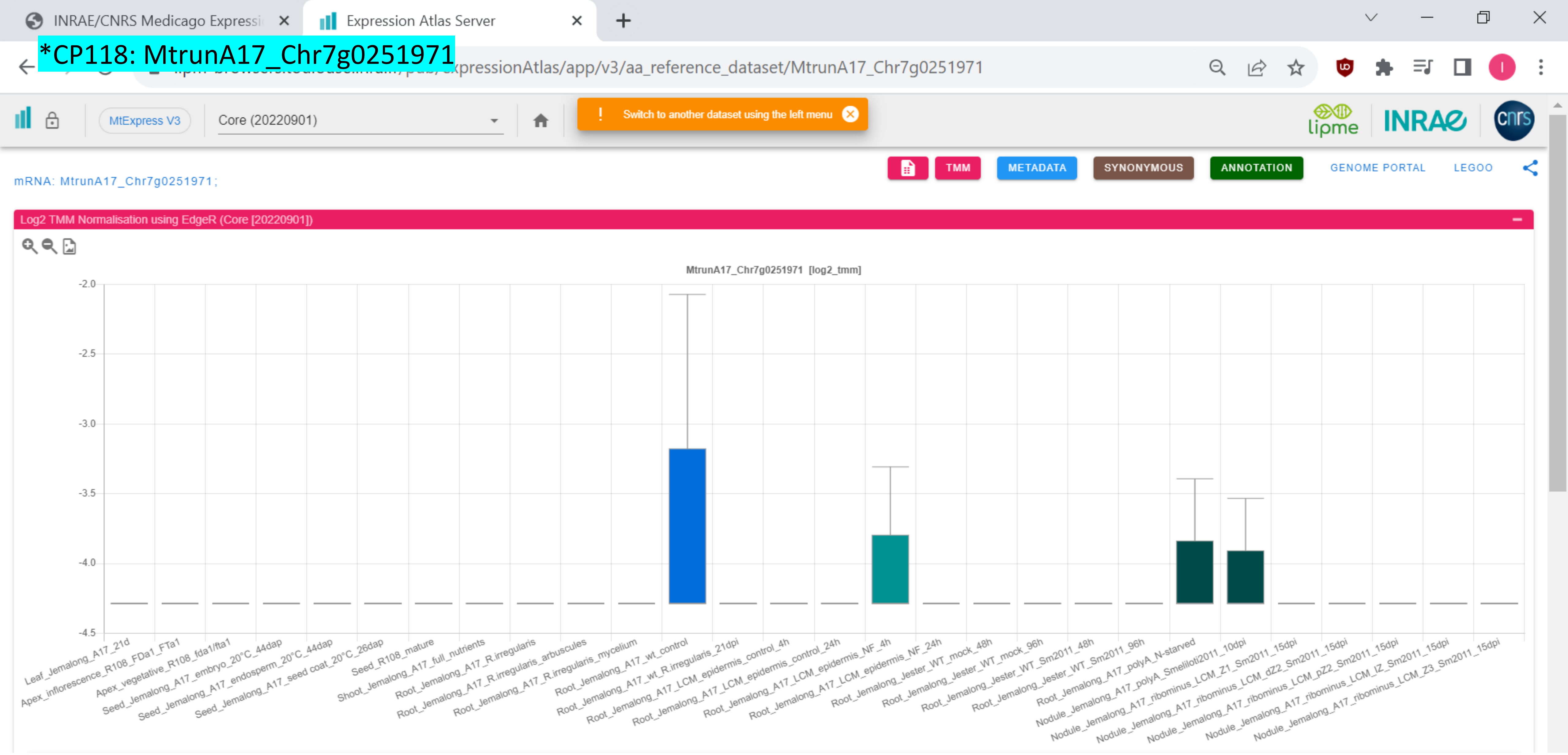

CP119: MtrunA17_Chr7g0259611

INRAE/CNRS Medicago Expressi | Expression Atlas Server

.../expressionAtlas/app/v3/aa_reference_dataset/MtrunA17_Chr7g0259611

MtExpress V3 | Core (20220901) | Switch to another dataset using the left menu

TMM | METADATA | SYNONYMOUS | ANNOTATION | GENOME PORTAL | LEGOO

mRNA: MtrunA17_Chr7g0259611;

Log2 TMM Normalisation using EdgeR (Core [20220901])

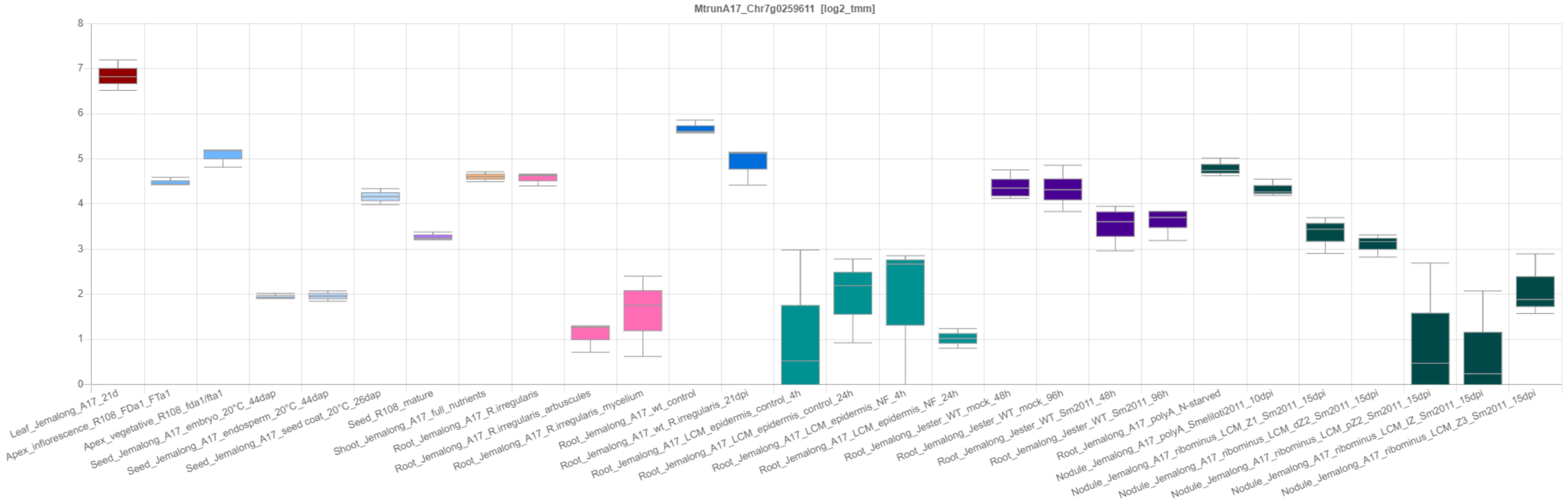

CP120: MtrunA17_Chr7g0262821

INRAE/CNRS Medicago Expressi | Expression Atlas Server

.../expressionAtlas/app/v3/aa_reference_dataset/MtrunA17_Chr7g0262821

MtExpress V3 | Core (20220901)

! Switch to another dataset using the left menu

lipme INRAE cnrs

TMM | METADATA | SYNONYMOUS | ANNOTATION | GENOME PORTAL | LEGOO

mRNA: MtrunA17_Chr7g0262821;

Log2 TMM Normalisation using EdgeR (Core [20220901])

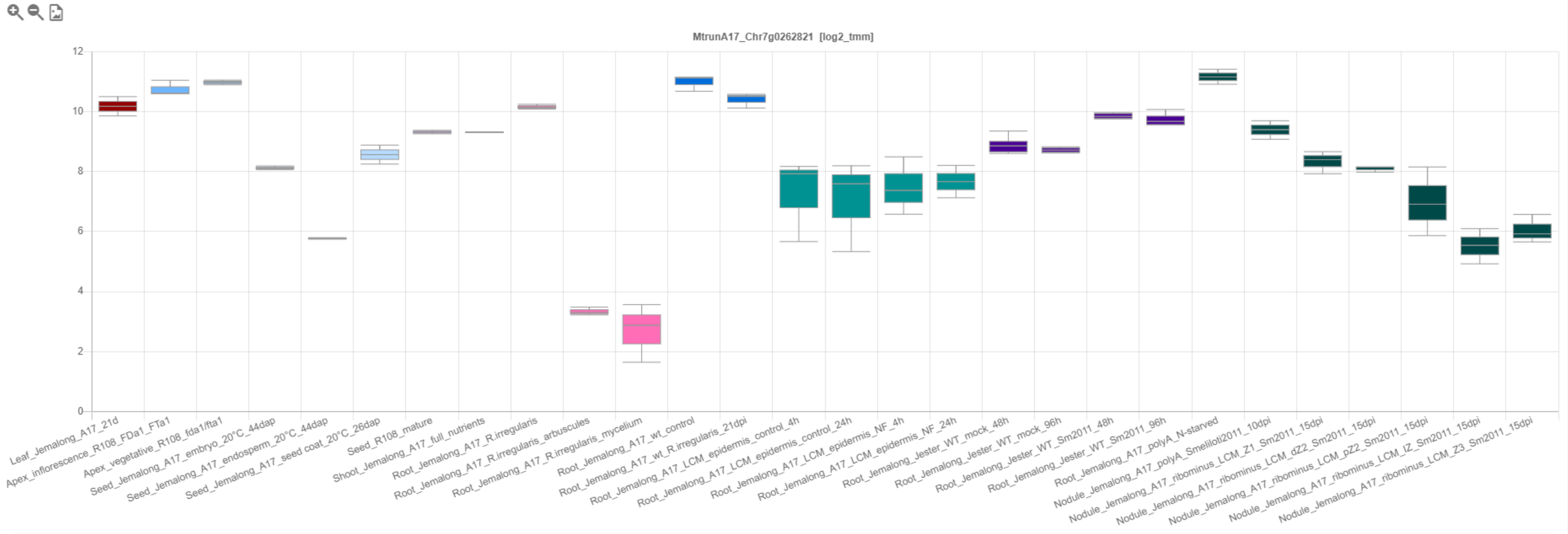

CP121: MtrunA17_Chr7g0270811

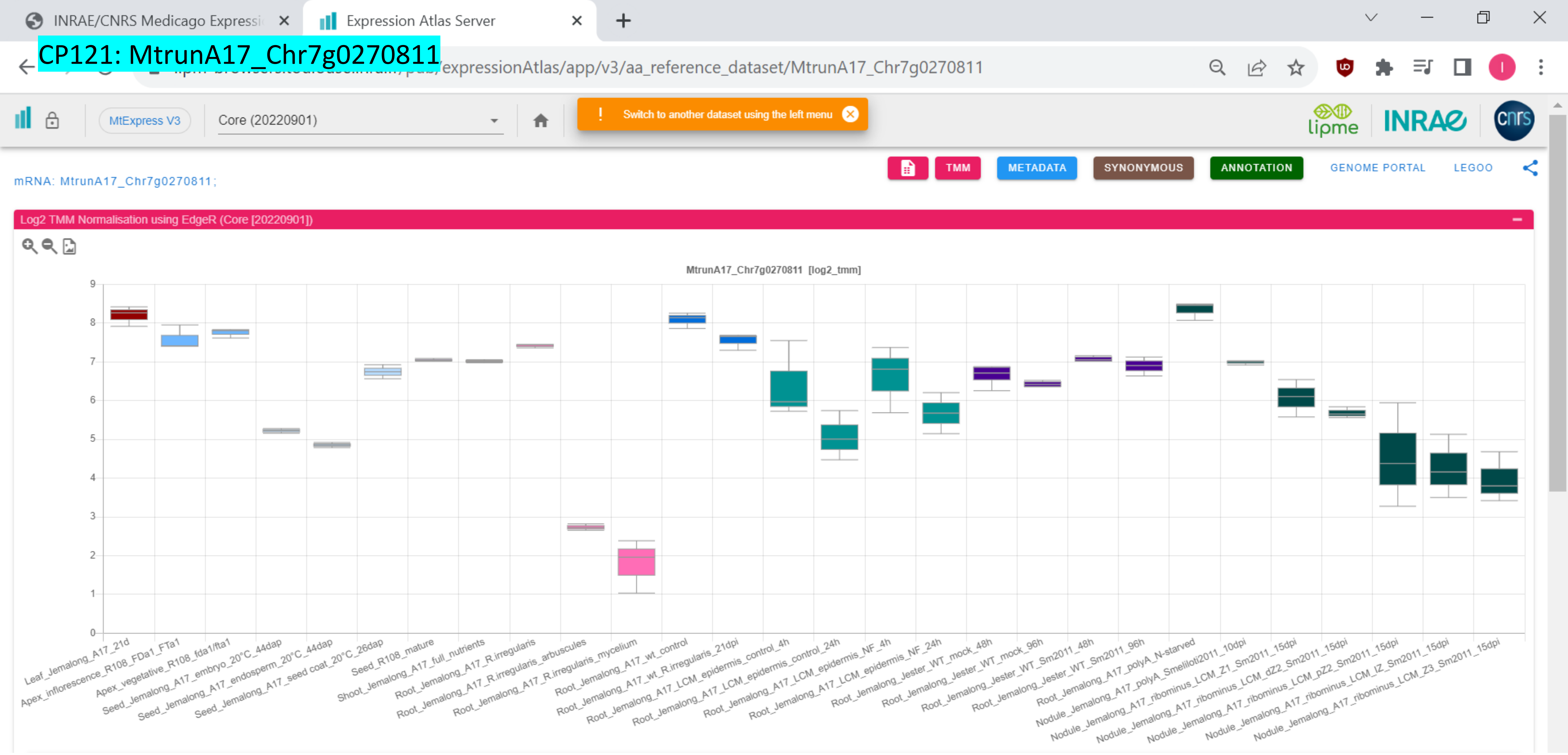

CP122: MtrunA17_Chr7g1034306

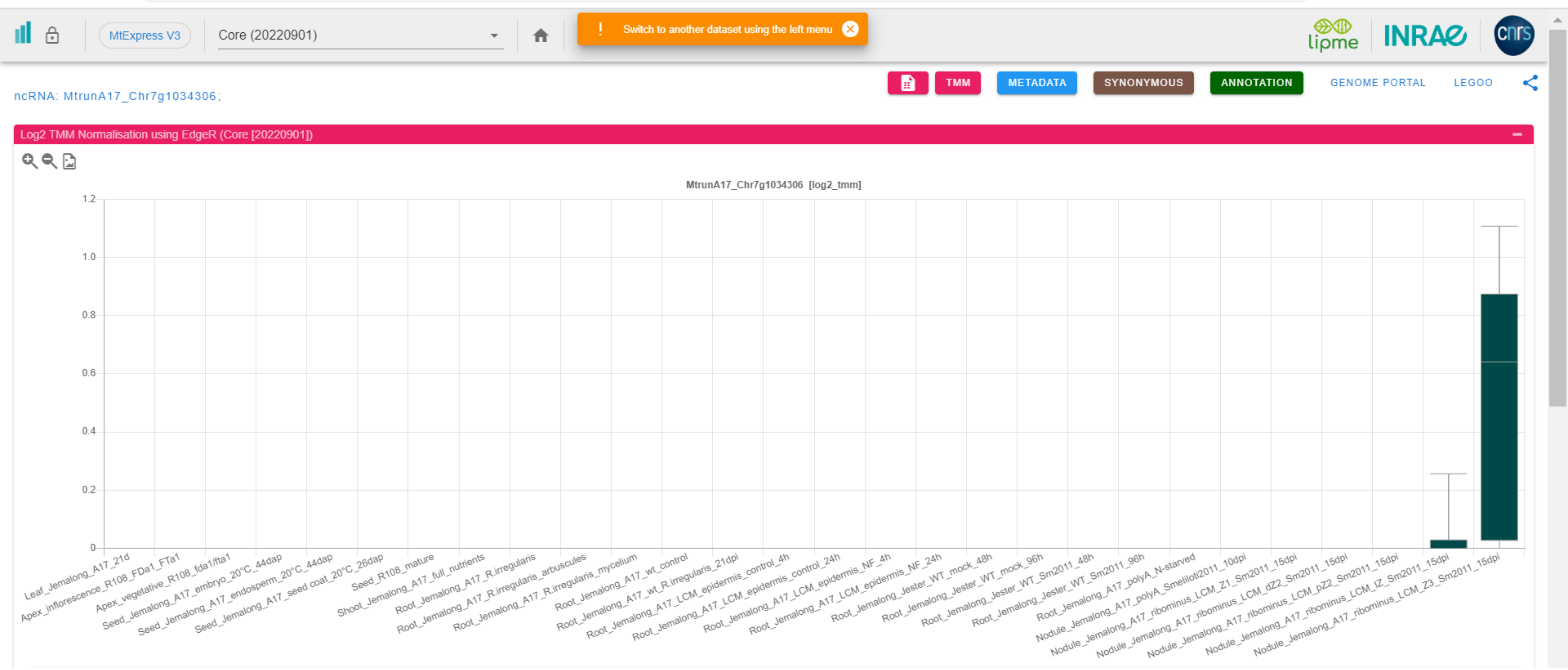

CP123: MtrunA17_Chr8g0338111

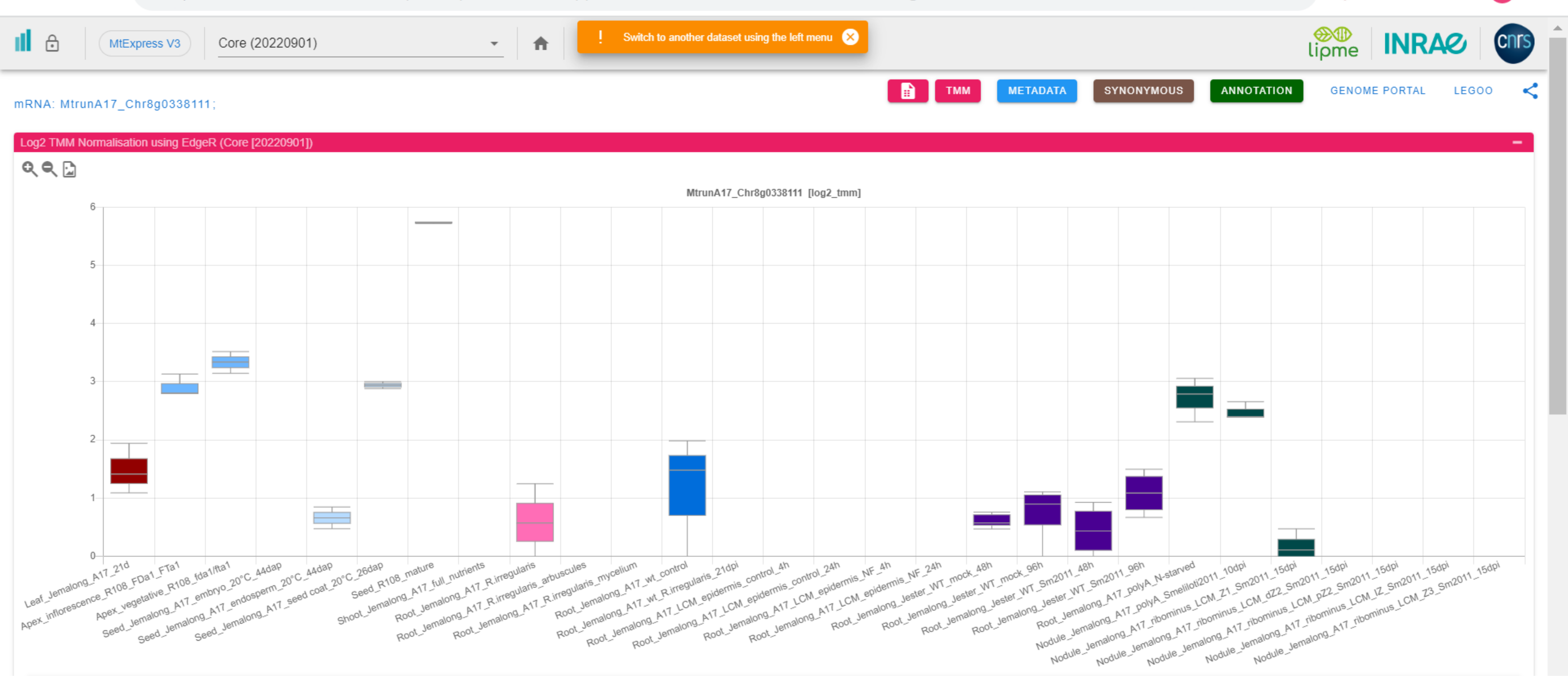

*CP124: MtrunA17_Chr8g0338301

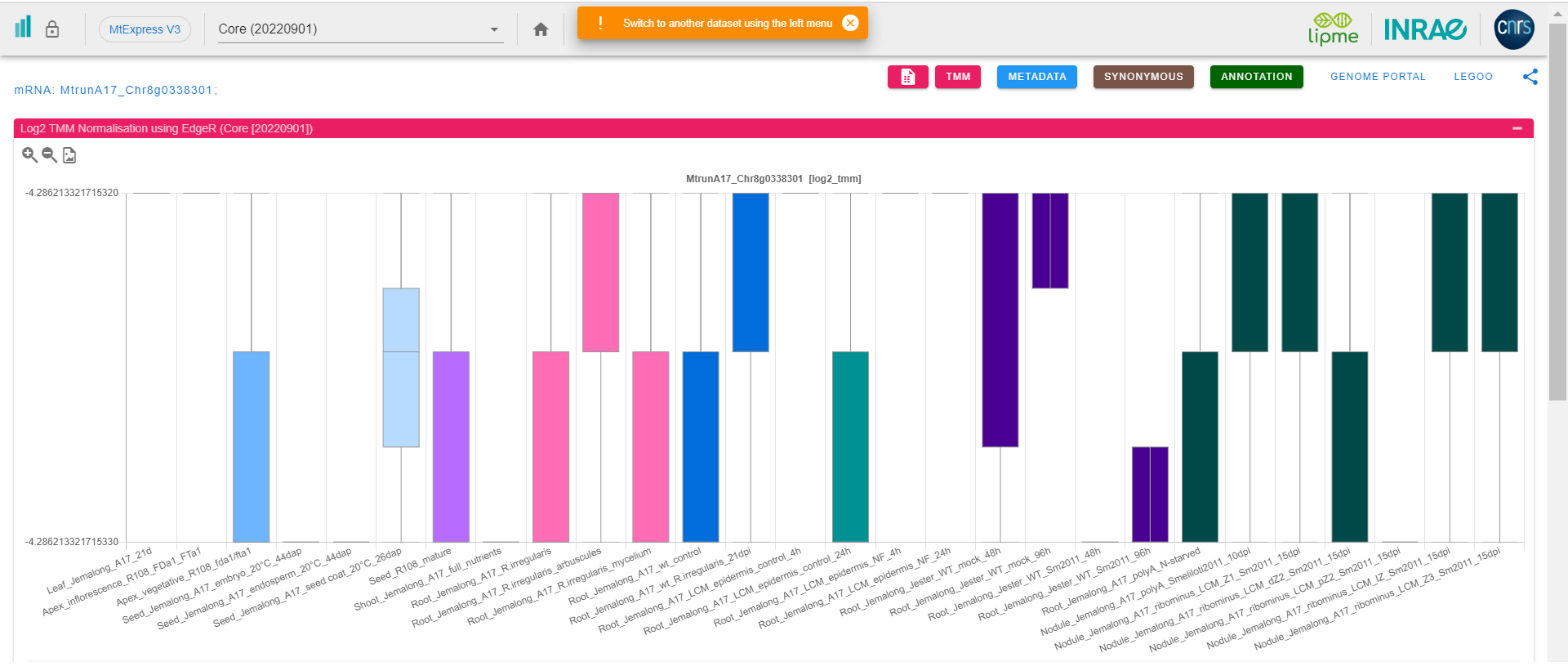

CP125: MtrunA17_Chr8g0339891

INRAE/CNRS Medicago Expressi | Expression Atlas Server

[illegible]/expressionAtlas/app/v3/aa_reference_dataset/MtrunA17_Chr8g0339891

MtExpress V3 | Core (20220901) | Switch to another dataset using the left menu

TMM | METADATA | SYNONYMOUS | ANNOTATION | GENOME PORTAL | LEGOO

mRNA: MtrunA17_Chr8g0339891;

Log2 TMM Normalisation using EdgeR (Core [20220901])

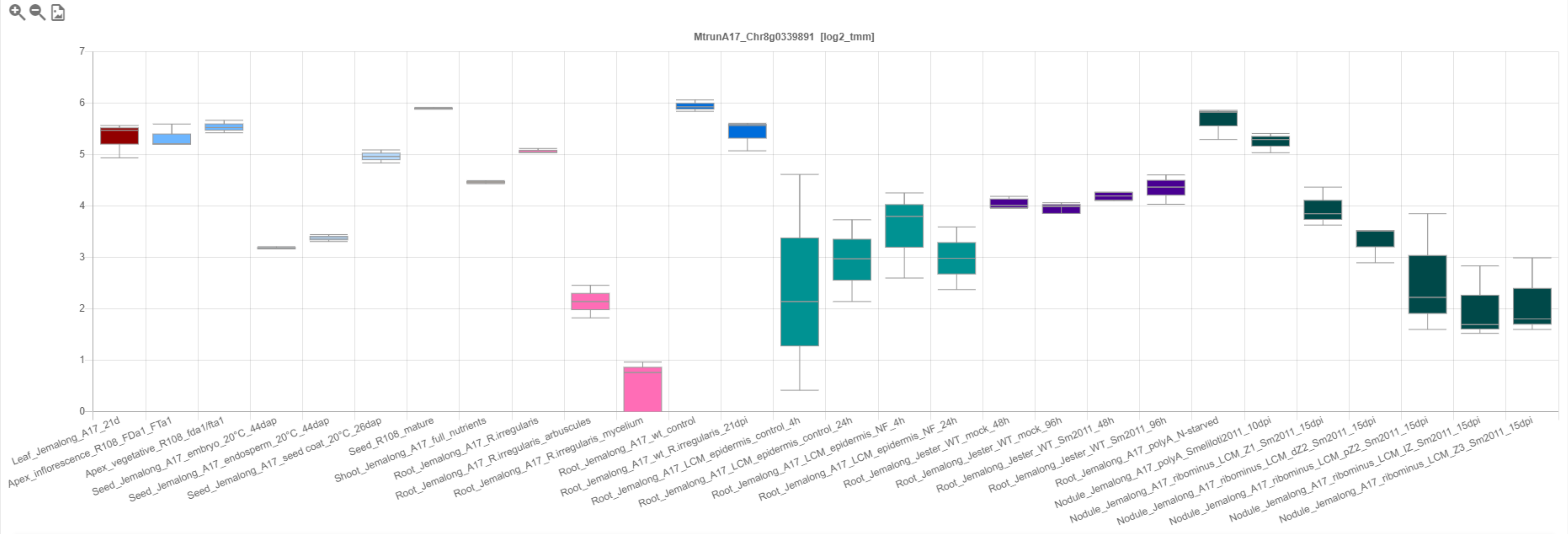

CP126: MtrunA17_Chr8g0342881

INRAE/CNRS Medicago Expressi | Expression Atlas Server

.../expressionAtlas/app/v3/aa_reference_dataset/MtrunA17_Chr8g0342881

MtExpress V3 | Core (20220901)

Switch to another dataset using the left menu

TMM | METADATA | SYNONYMOUS | ANNOTATION | GENOME PORTAL | LEGOO

mRNA: MtrunA17_Chr8g0342881;

Log2 TMM Normalisation using EdgeR (Core [20220901])

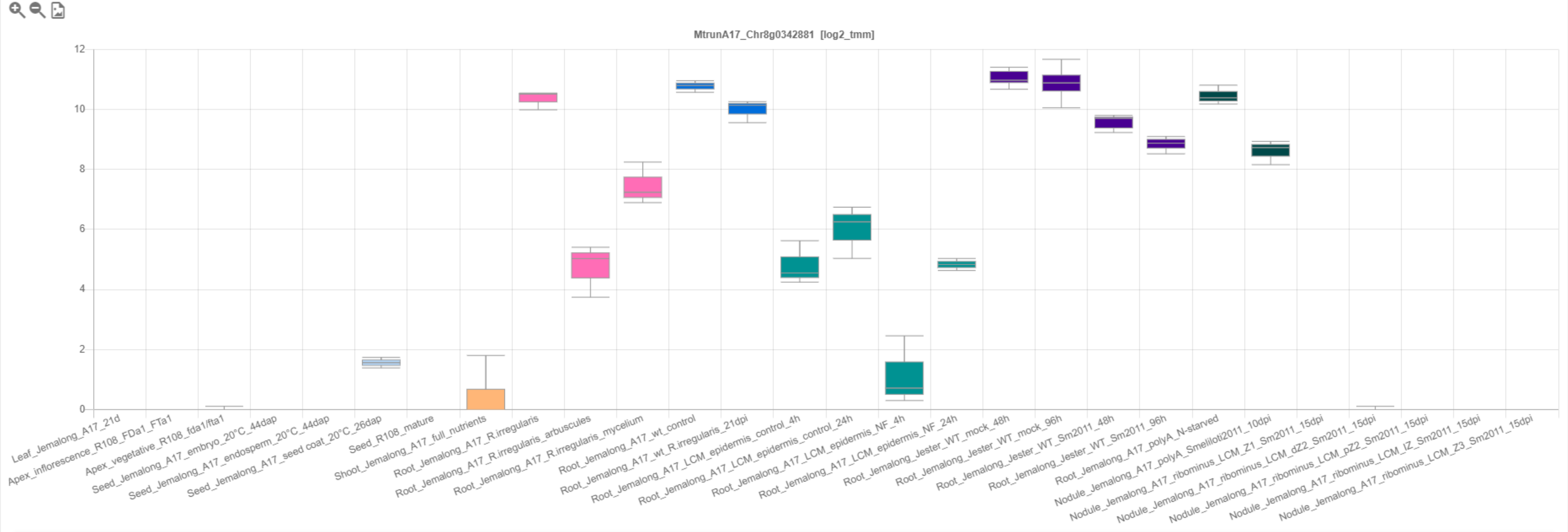

CP127: MtrunA17_Chr8g0345421

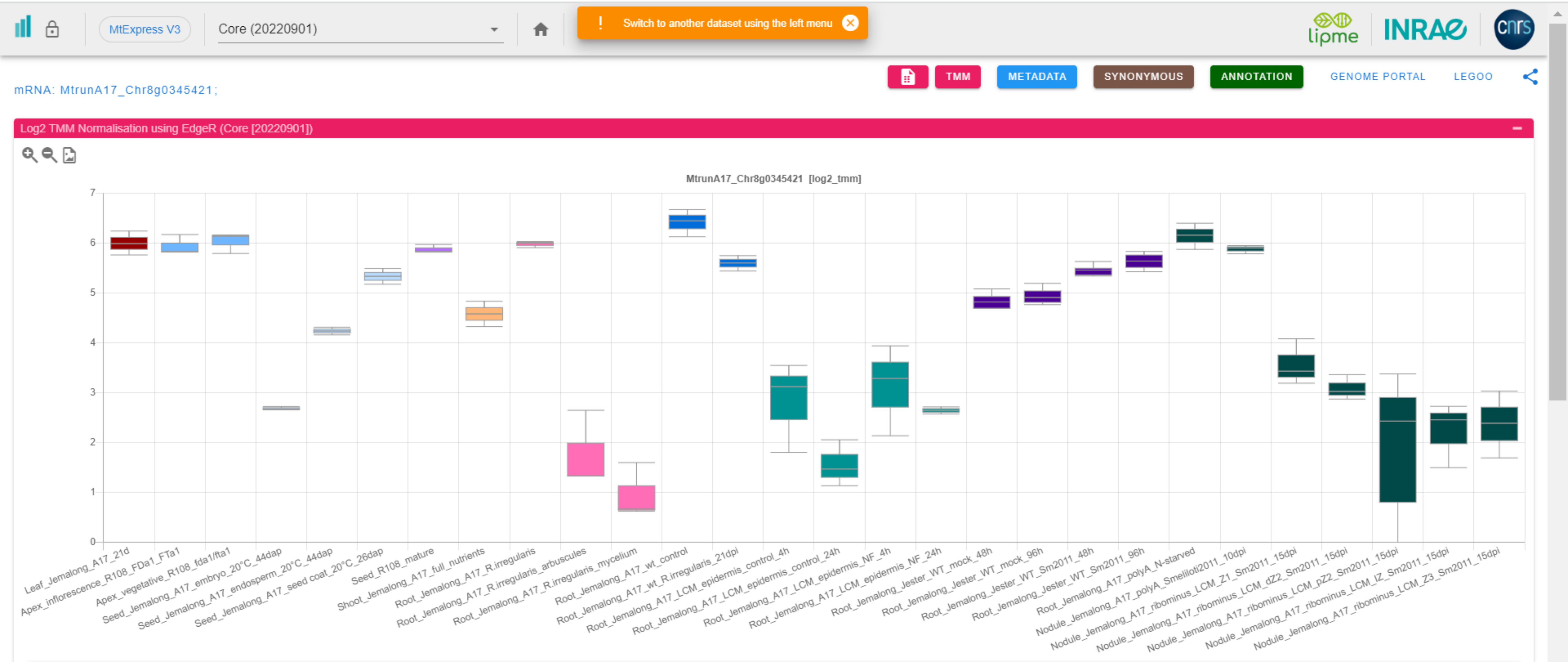

*CP128: MtrunA17_Chr8g0353711

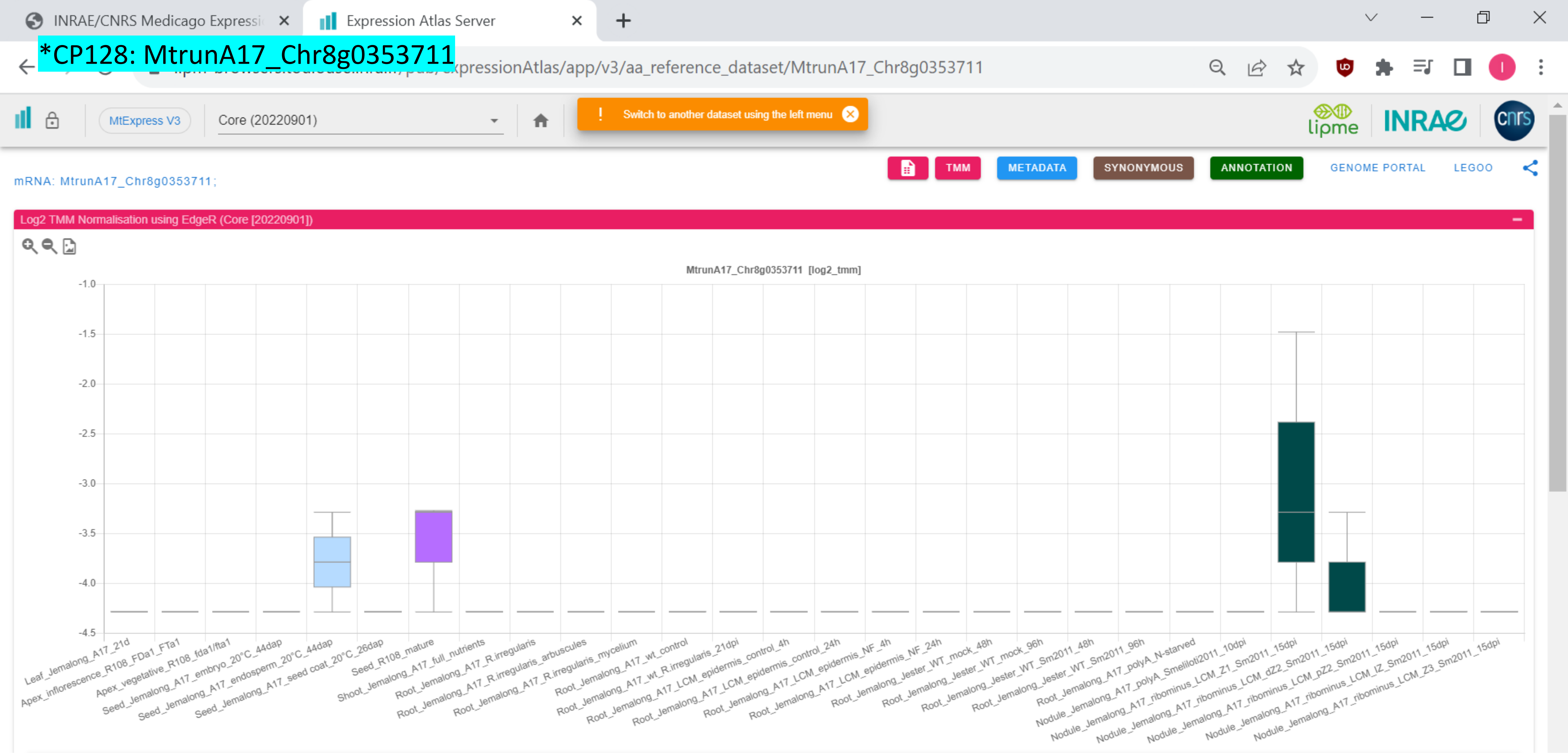

CP129: MtrunA17_Chr8g0355501

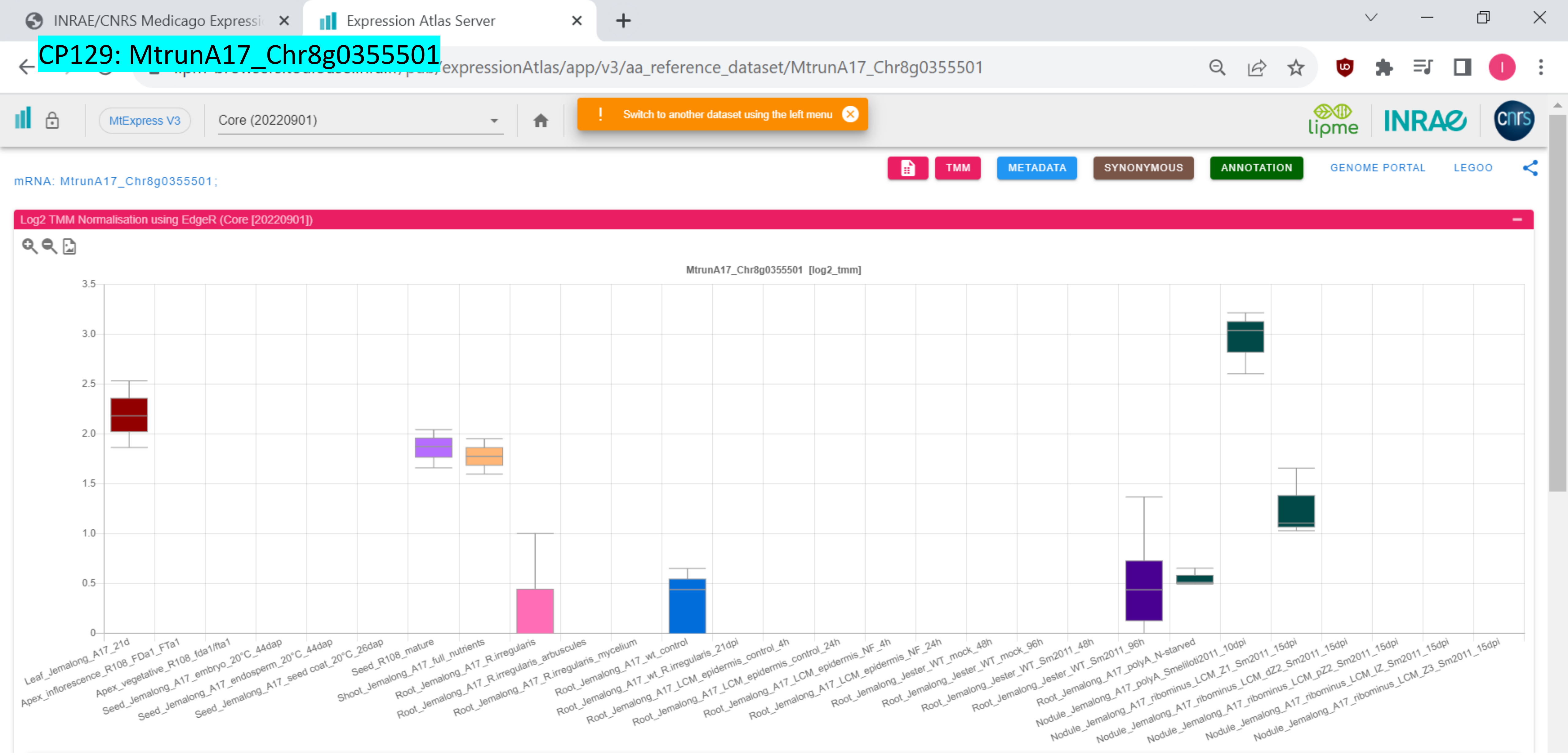

*CP130: MtrunA17_Chr8g0356581

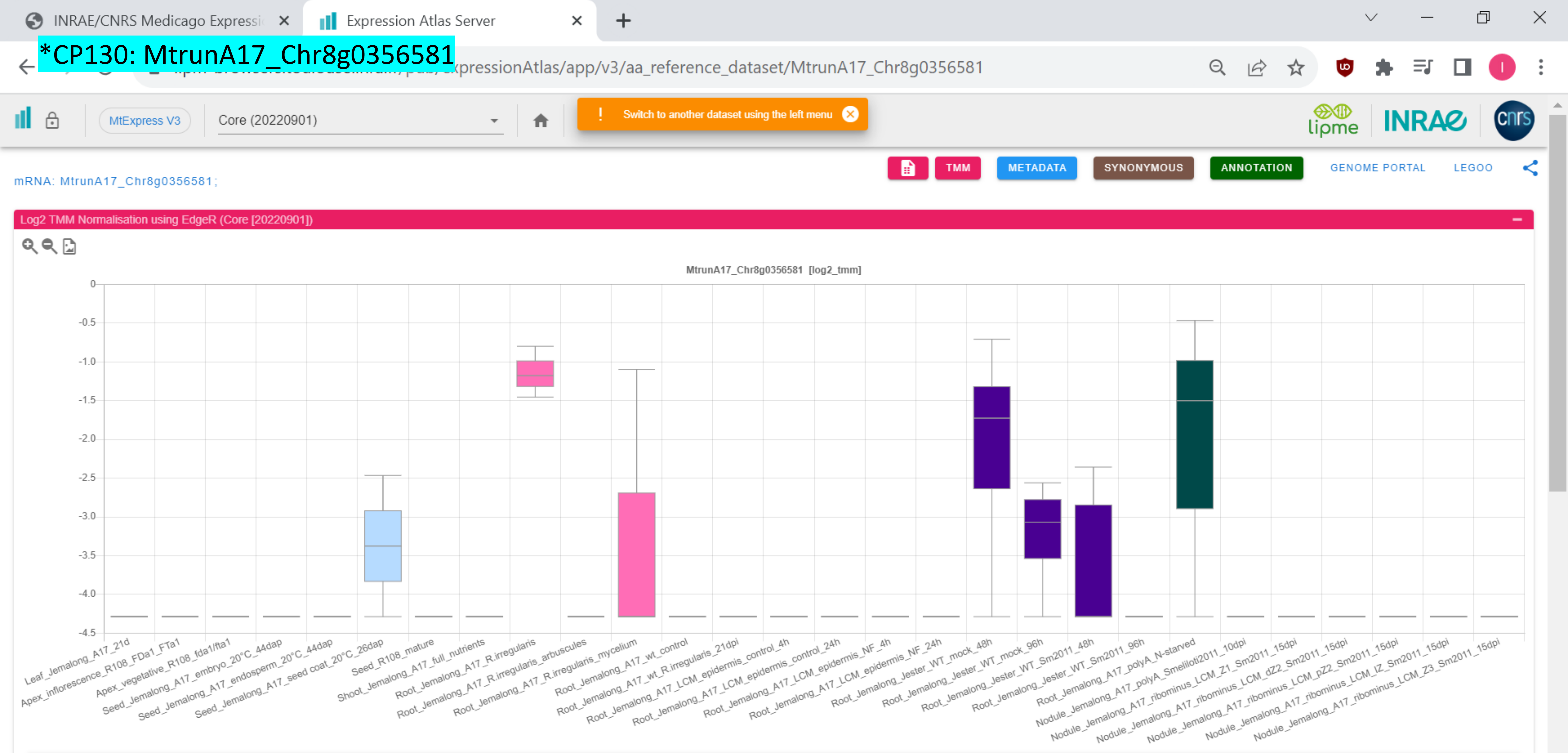

CP131: MtrunA17_Chr8g0365341

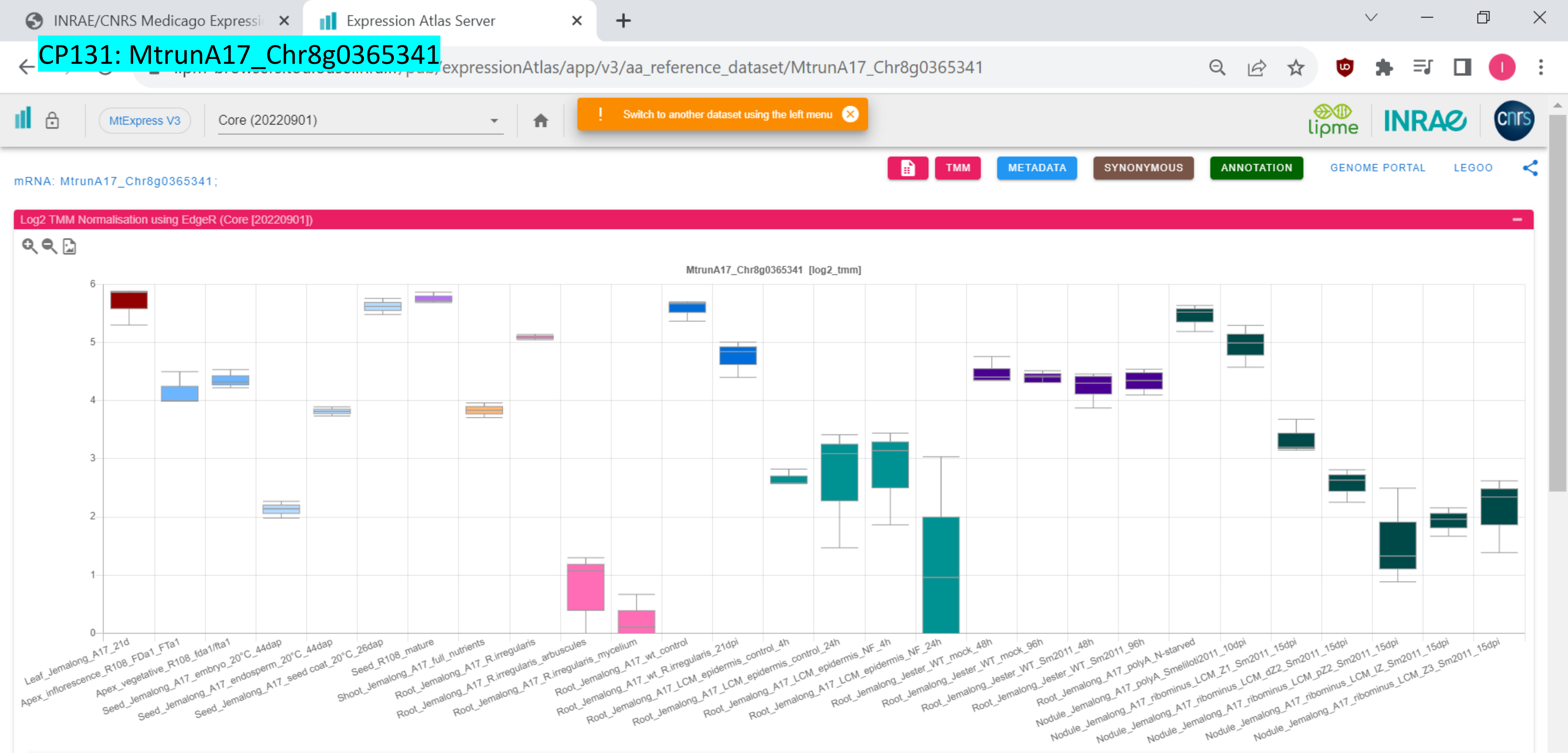

CP132: MtrunA17_Chr8g0368731

INRAE/CNRS Medicago Expressi
Expression Atlas Server

/expressionAtlas/app/v3/aa_reference_dataset/MtrunA17_Chr8g0368731

MtExpress V3
Core (20220901)
Switch to another dataset using the left menu

TMM
METADATA
SYNONYMOUS
ANNOTATION
GENOME PORTAL
LEGOO

mRNA: MtrunA17_Chr8g0368731;

Log2 TMM Normalisation using EdgeR (Core [20220901])

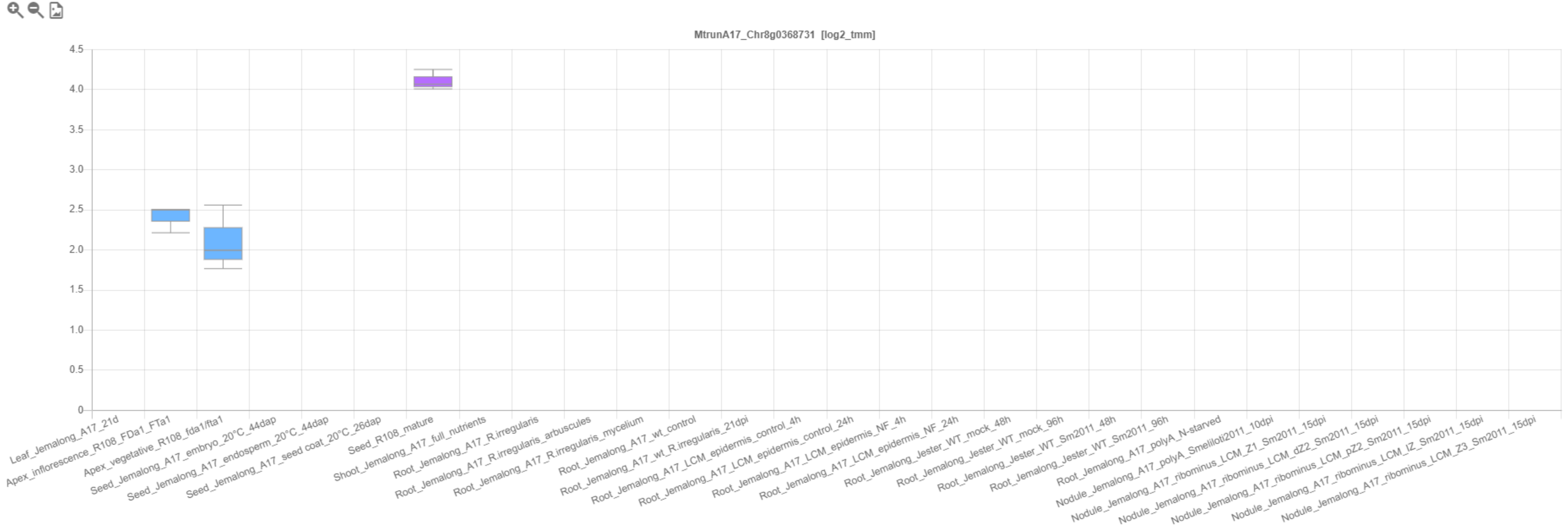

INRAE/CNRS Medicago Expressi

Expression Atlas Server

CP133: MtrunA17_Chr8g0368931

/expressionAtlas/app/v3/aa_reference_dataset/MtrunA17_Chr8g0368931

MtExpress V3

Core (20220901)

Switch to another dataset using the left menu

TMM METADATA SYNONYMOUS ANNOTATION GENOME PORTAL LEGOO

mRNA: MtrunA17_Chr8g0368931;

Log2 TMM Normalisation using EdgeR (Core [20220901])

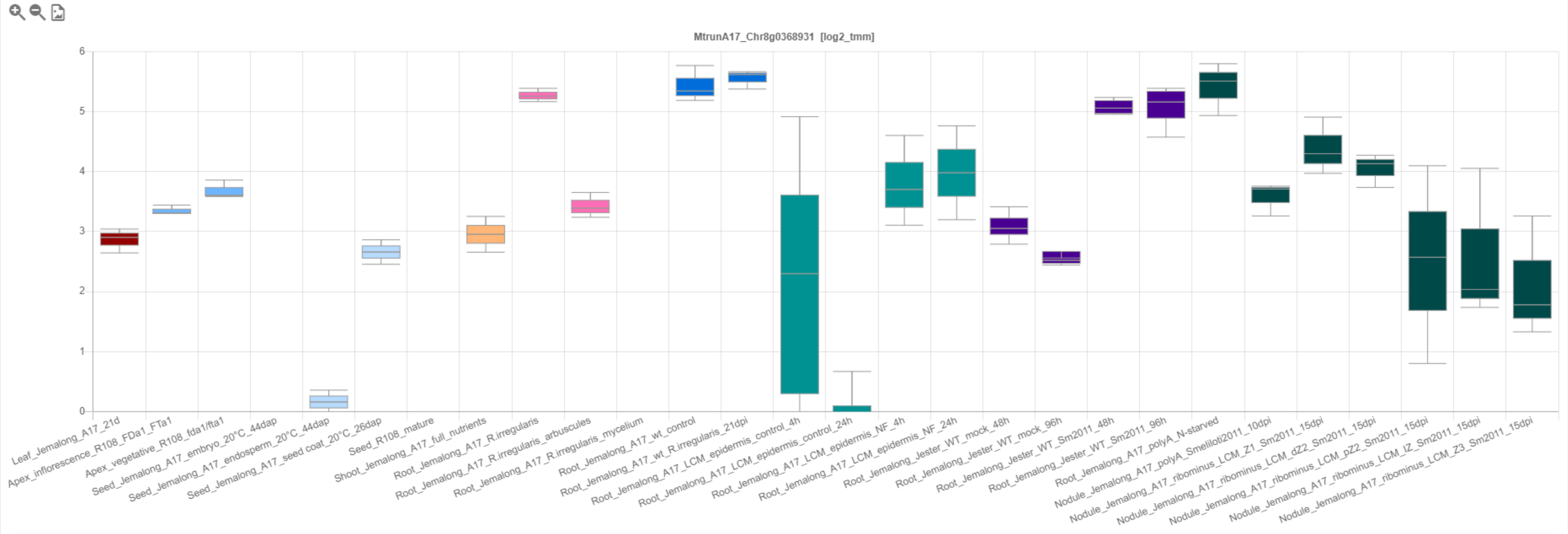

CP134: MtrunA17_Chr8g0371281

INRAE/CNRS Medicago Expressi | Expression Atlas Server

…/expressionAtlas/app/v3/aa_reference_dataset/MtrunA17_Chr8g0371281

MtExpress V3 | Core (20220901)

Switch to another dataset using the left menu

TMM | METADATA | SYNONYMOUS | ANNOTATION | GENOME PORTAL | LEGOO

mRNA: MtrunA17_Chr8g0371281;

Log2 TMM Normalisation using EdgeR (Core [20220901])

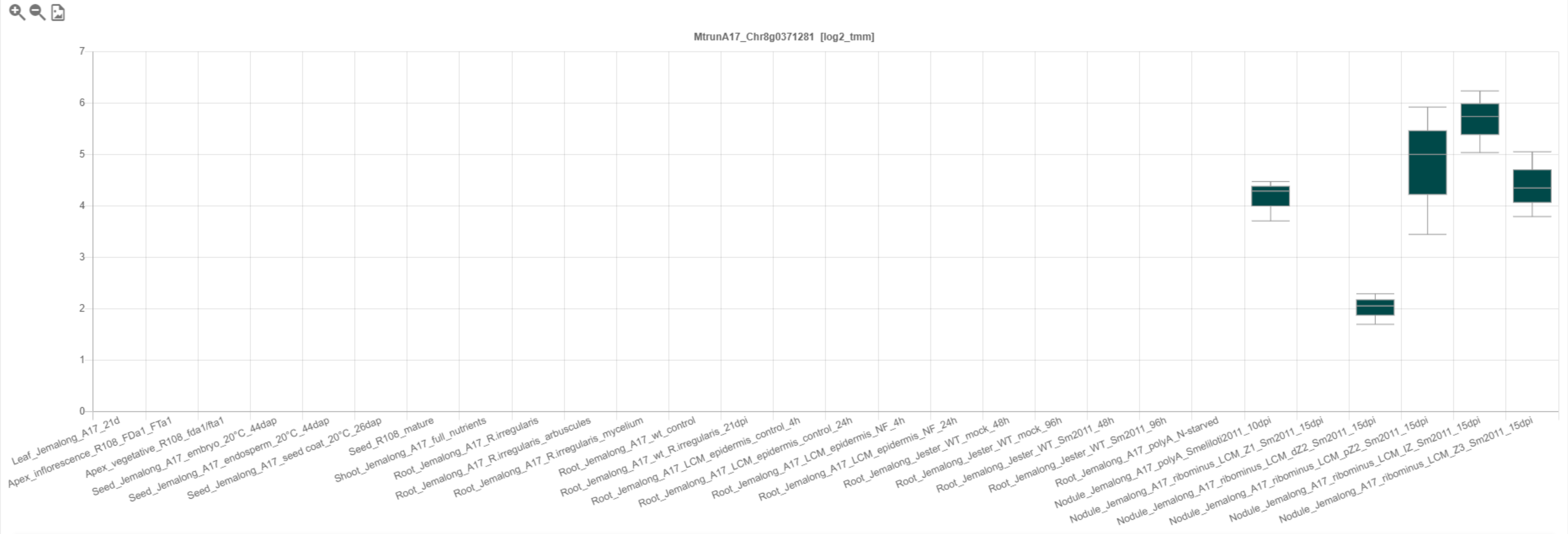

CP135: MtrunA17_Chr8g0371741

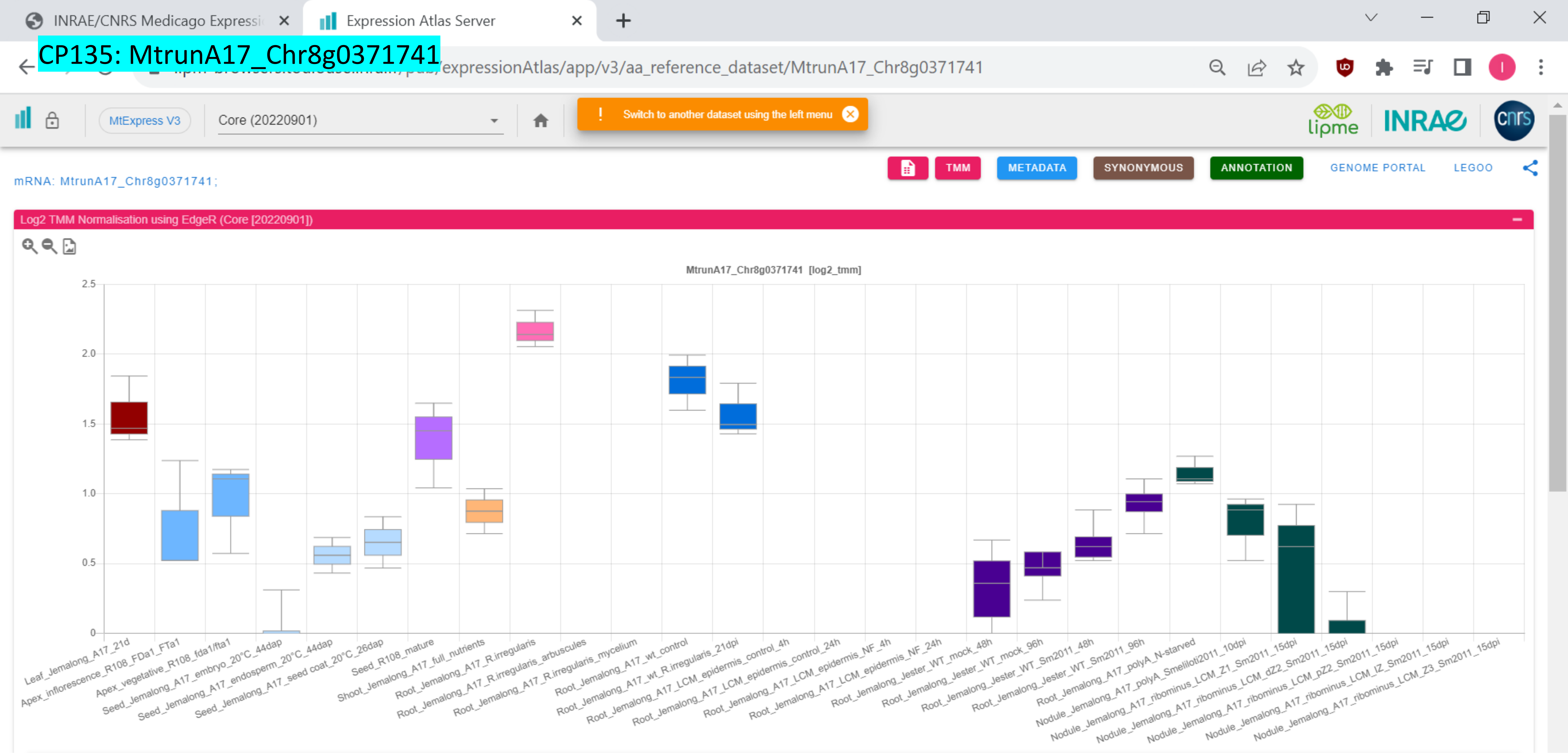

CP136: MtrunA17_Chr8g0373091

expressionAtlas/app/v3/aa_reference_dataset/MtrunA17_Chr8g0373091

MtExpress V3 | Core (20220901) | Switch to another dataset using the left menu

TMM | METADATA | SYNONYMOUS | ANNOTATION | GENOME PORTAL | LEGOO

mRNA: MtrunA17_Chr8g0373091;

Log2 TMM Normalisation using EdgeR (Core [20220901])

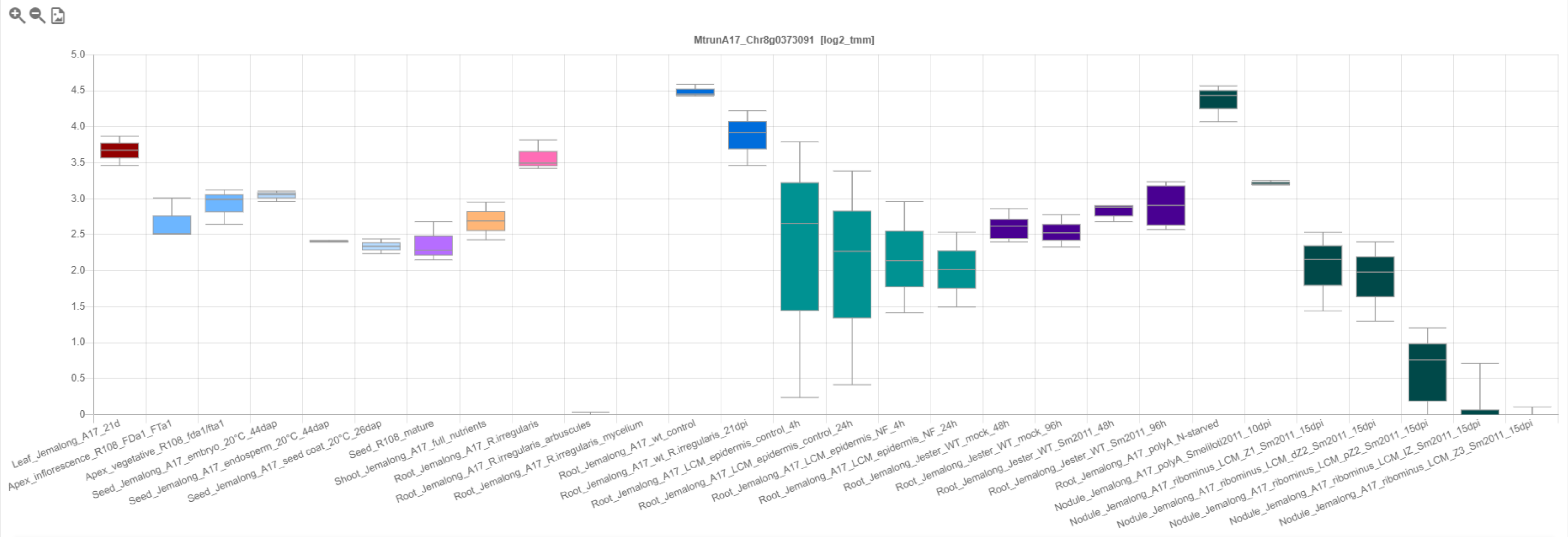

CP137: MtrunA17_Chr8g0376411

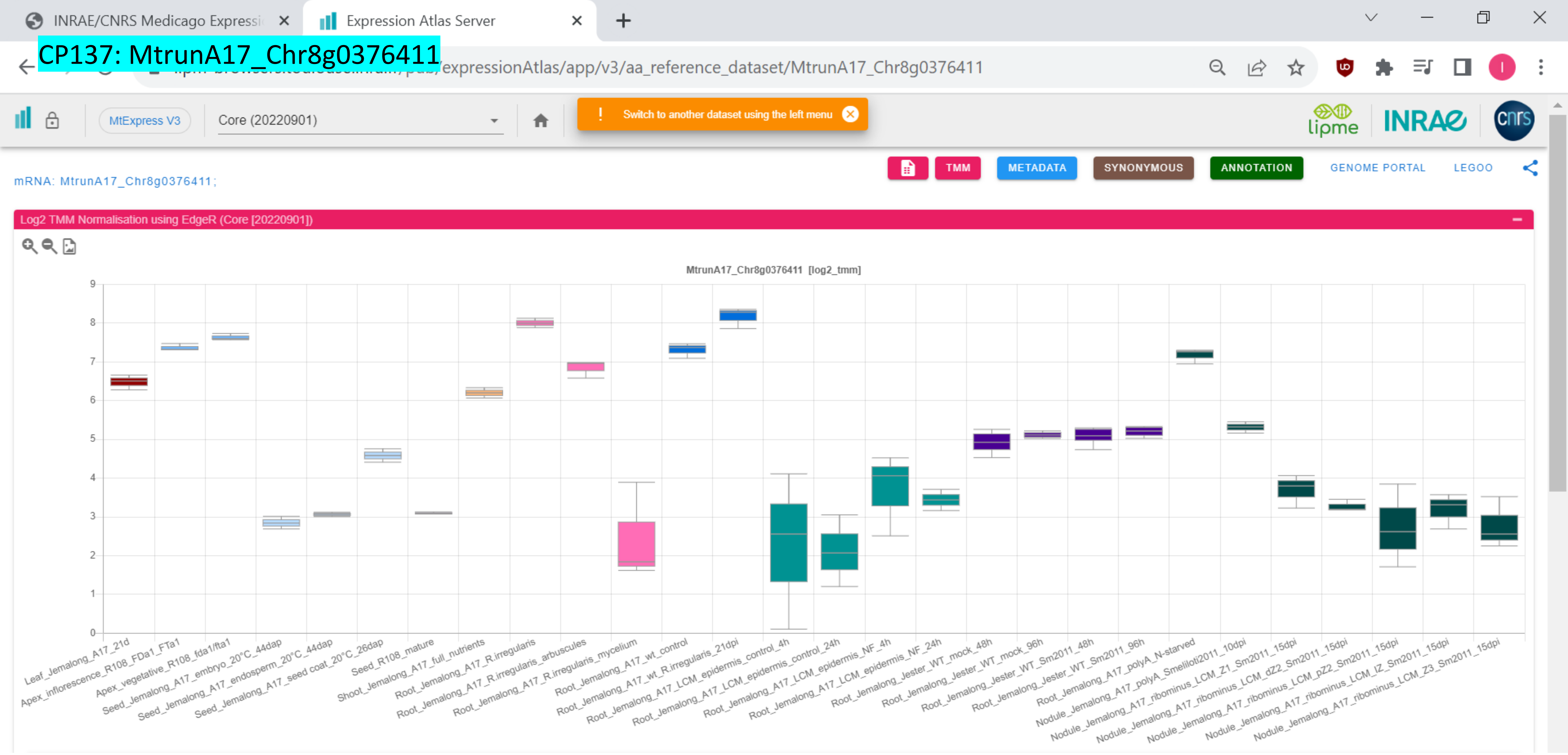

CP138: MtrunA17_Chr8g0377071

INRAE/CNRS Medicago Expressi | Expression Atlas Server

.../expressionAtlas/app/v3/aa_reference_dataset/MtrunA17_Chr8g0377071

MtExpress V3 | Core (20220901)

! Switch to another dataset using the left menu

TMM | METADATA | SYNONYMOUS | ANNOTATION | GENOME PORTAL | LEGOO

mRNA: MtrunA17_Chr8g0377071;

Log2 TMM Normalisation using EdgeR (Core [20220901])

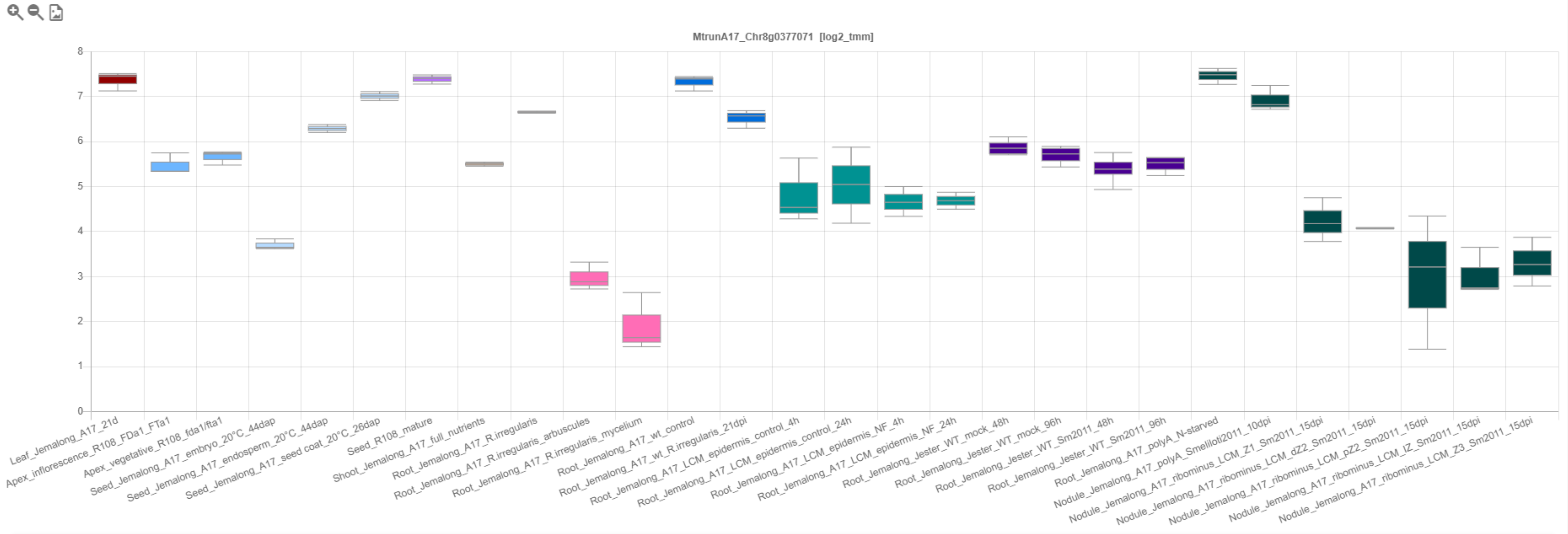

CP139: MtrunA17_Chr8g0385331

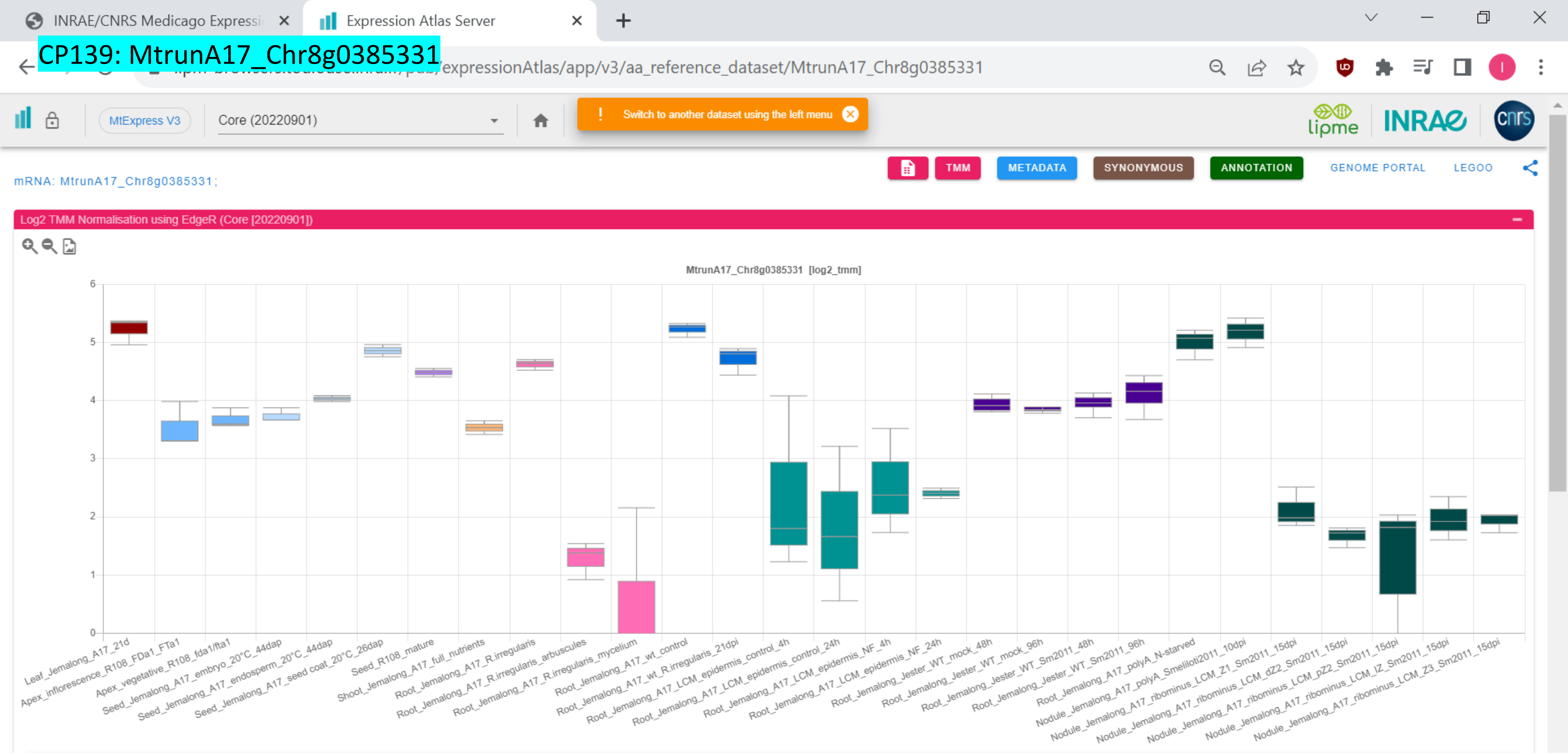

CP140: MtrunA17_Chr8g0392351

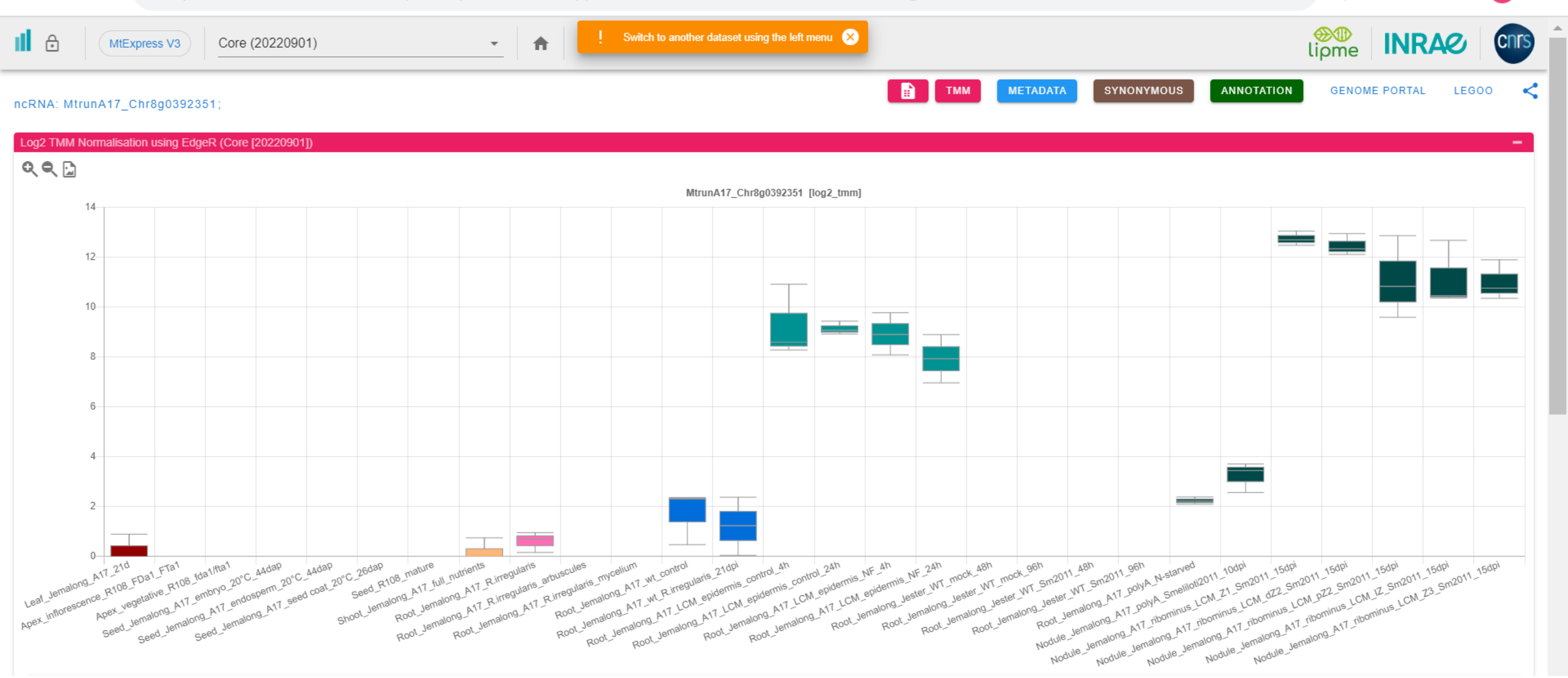

CP141: MtrunA17_CPg0492331

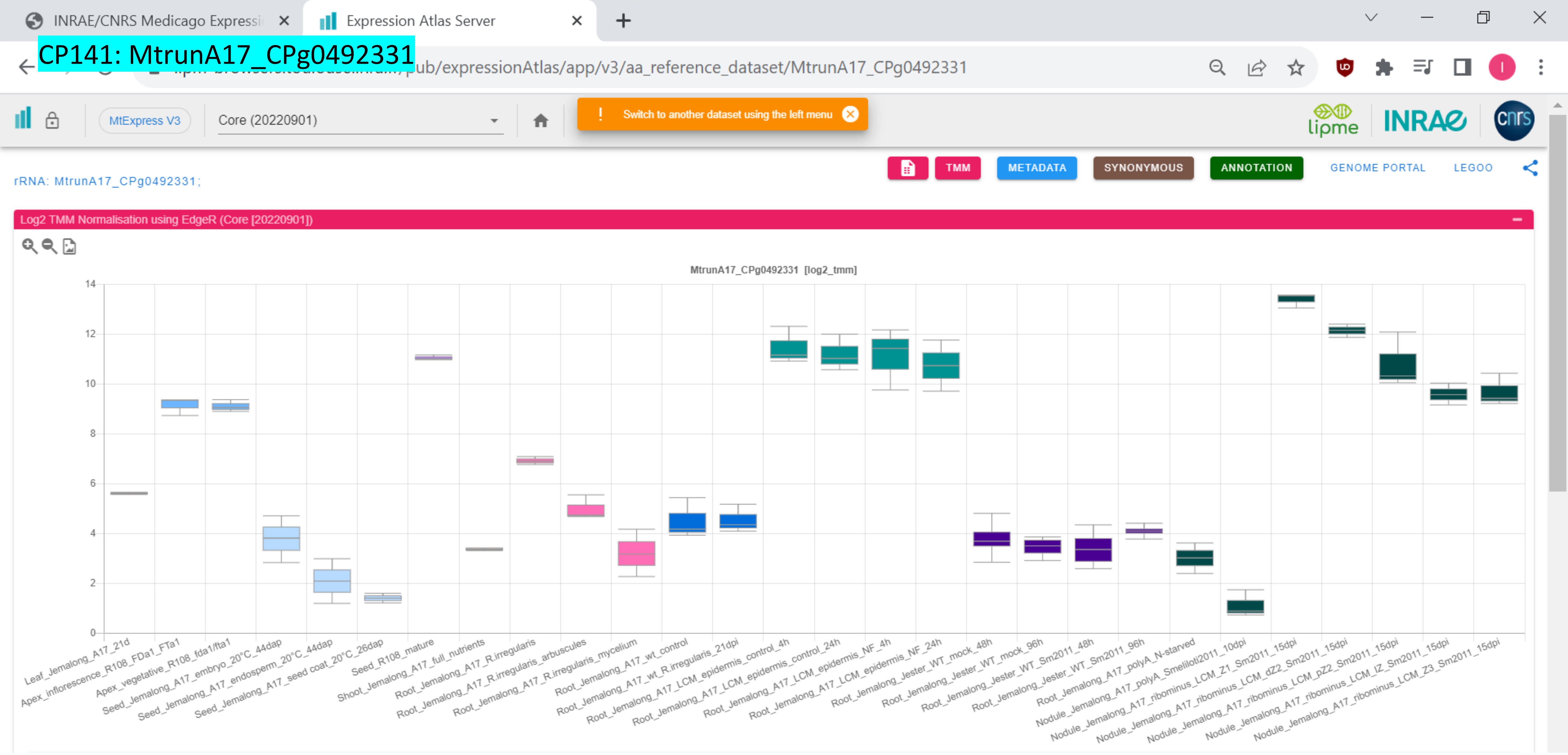

CP142: MtrunA17_CPg0492381

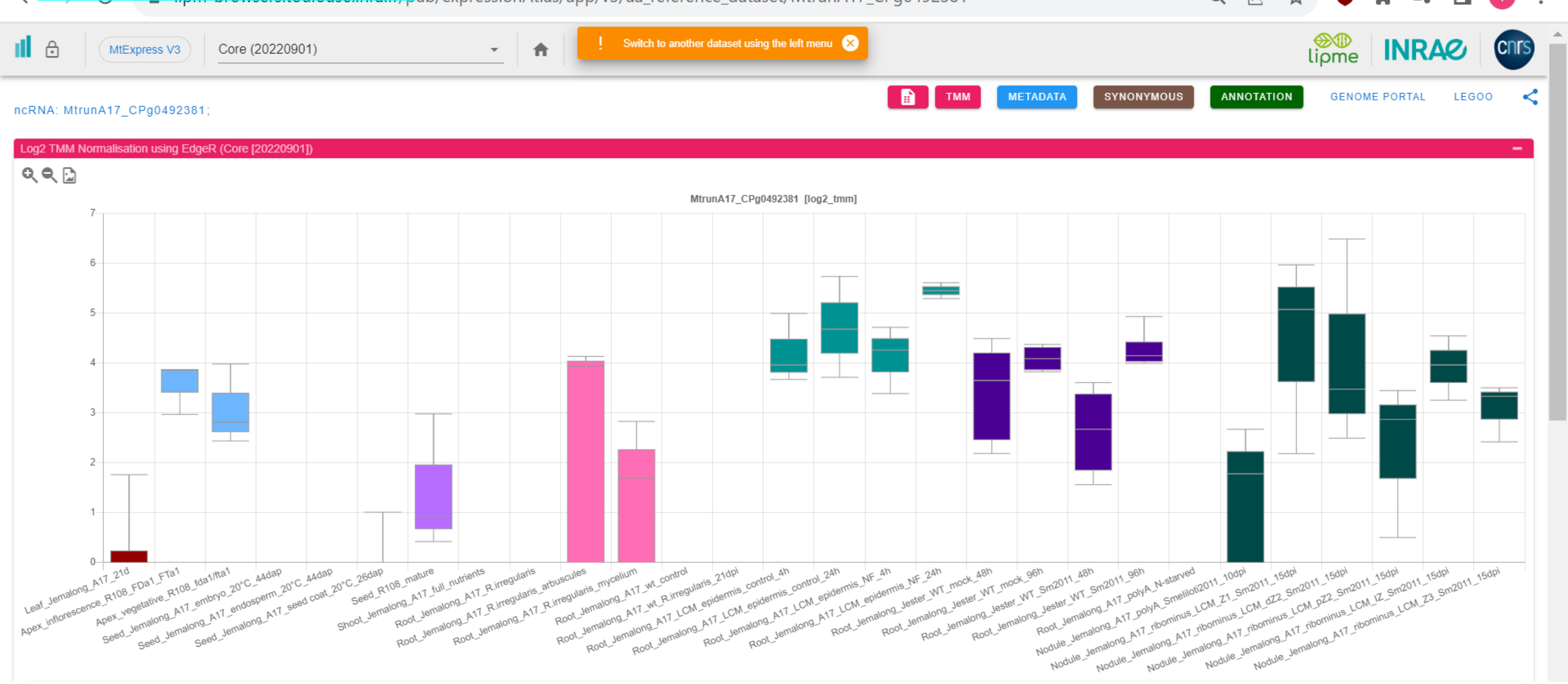

CP143: MtrunA17_CPg0492461

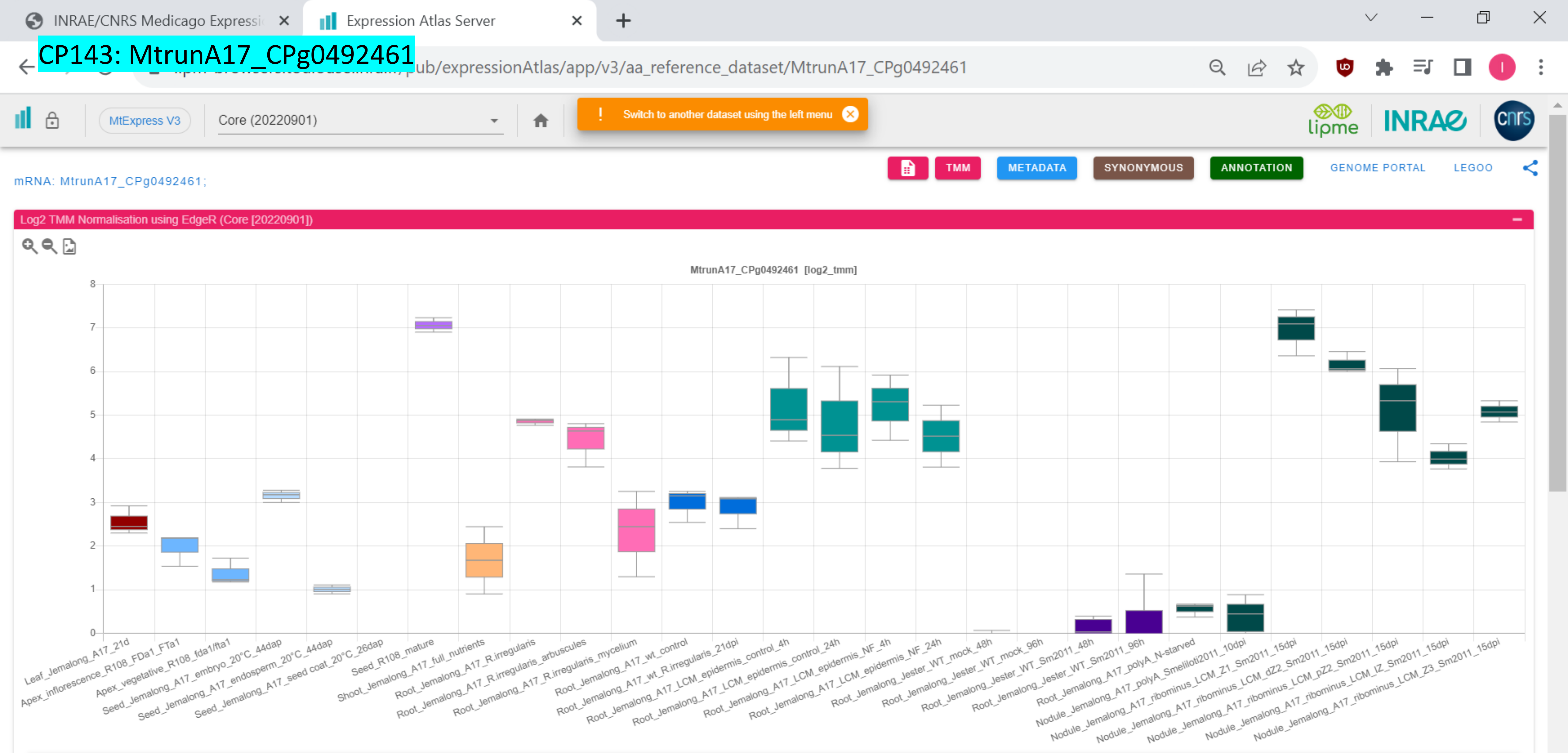

CP144: MtrunA17_CPg0492851

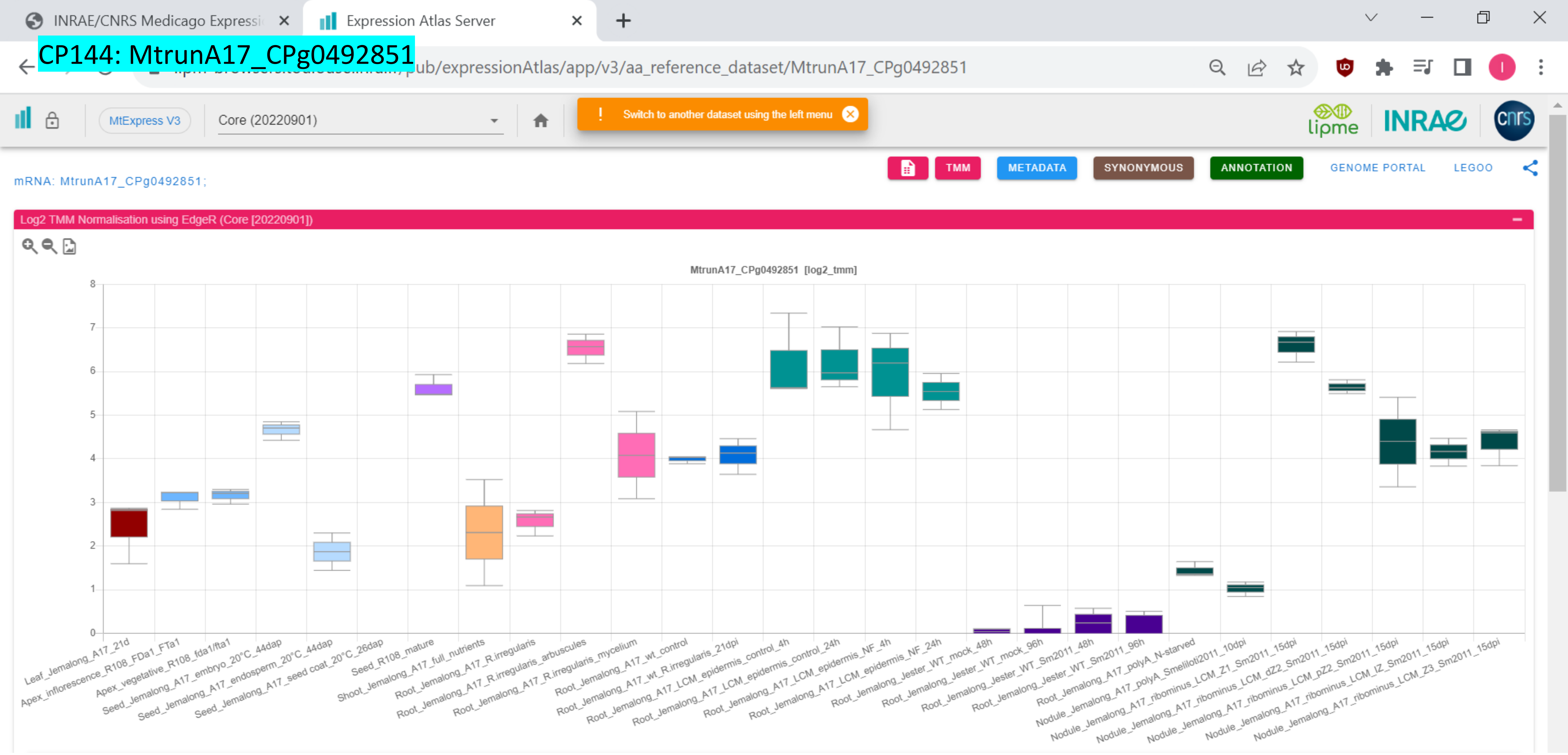

CP145: MtrunA17_CPg0492941

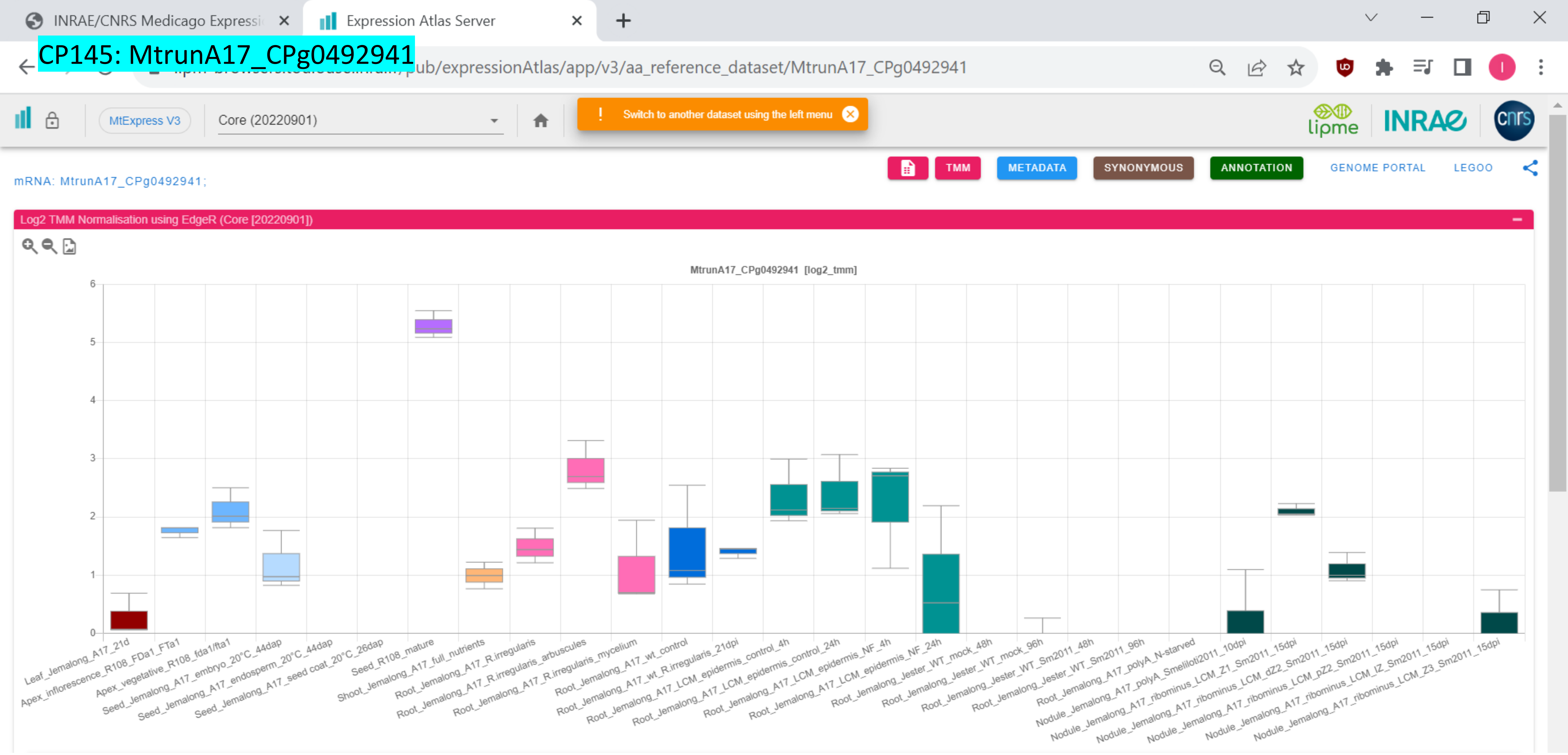

CP146: MtrunA17_CPg0493291

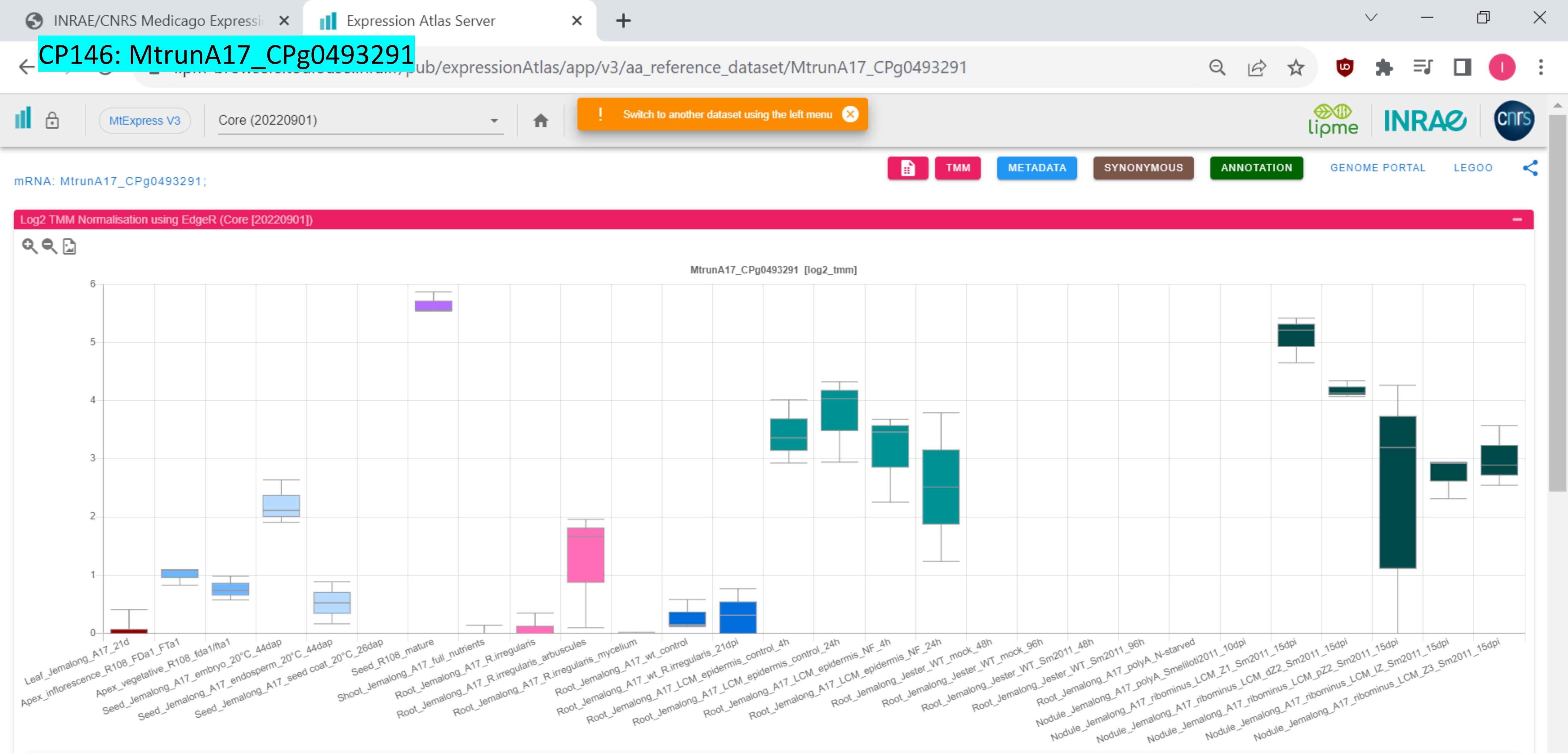

CP147: MtrunA17_CPg0493401

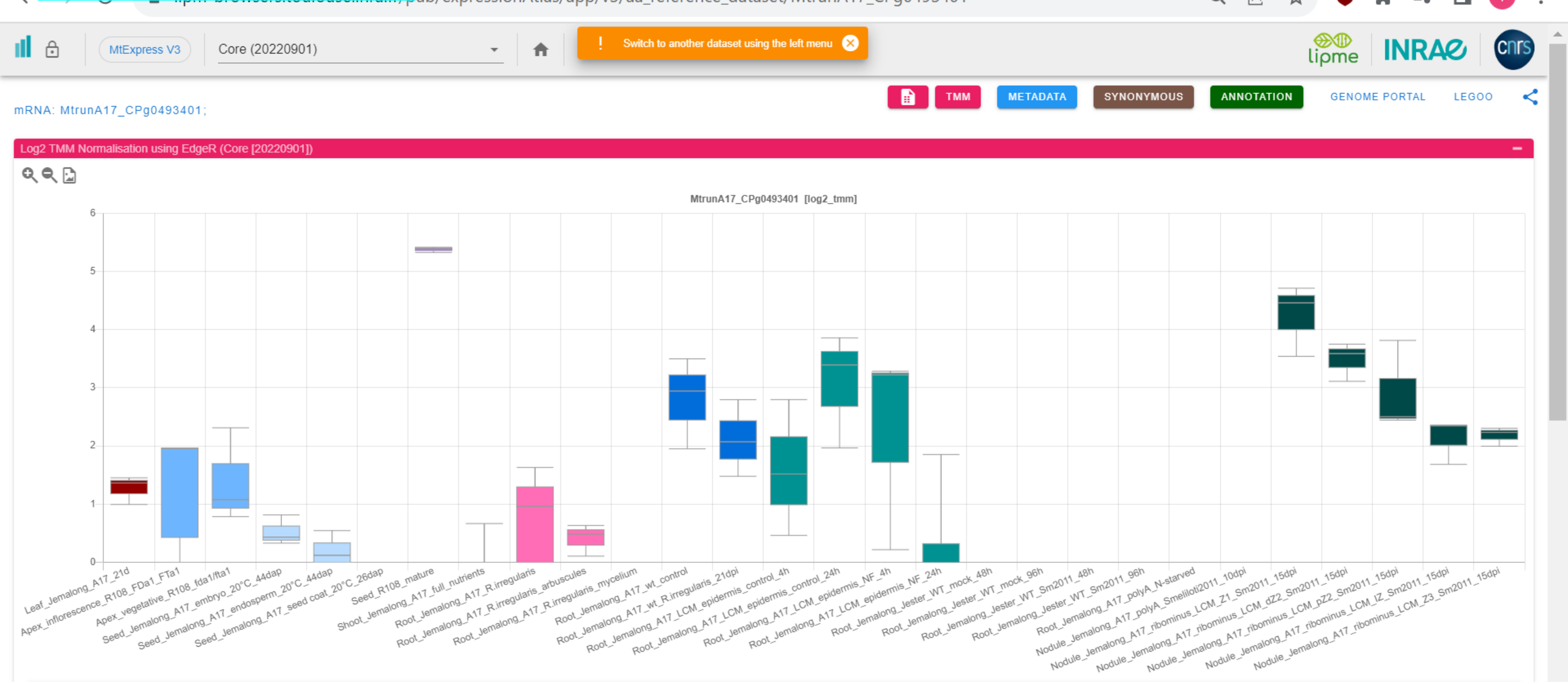

CP148: MtrunA17_MTg0490471

INRAE/CNRS Medicago Expressi | Expression Atlas Server

.../pub/expressionAtlas/app/v3/aa_reference_dataset/MtrunA17_MTg0490471

MtExpress V3 | Core (20220901) | Switch to another dataset using the left menu

TMM | METADATA | SYNONYMOUS | ANNOTATION | GENOME PORTAL | LEGOO

mRNA: MtrunA17_MTg0490471;

Log2 TMM Normalisation using EdgeR (Core [20220901])

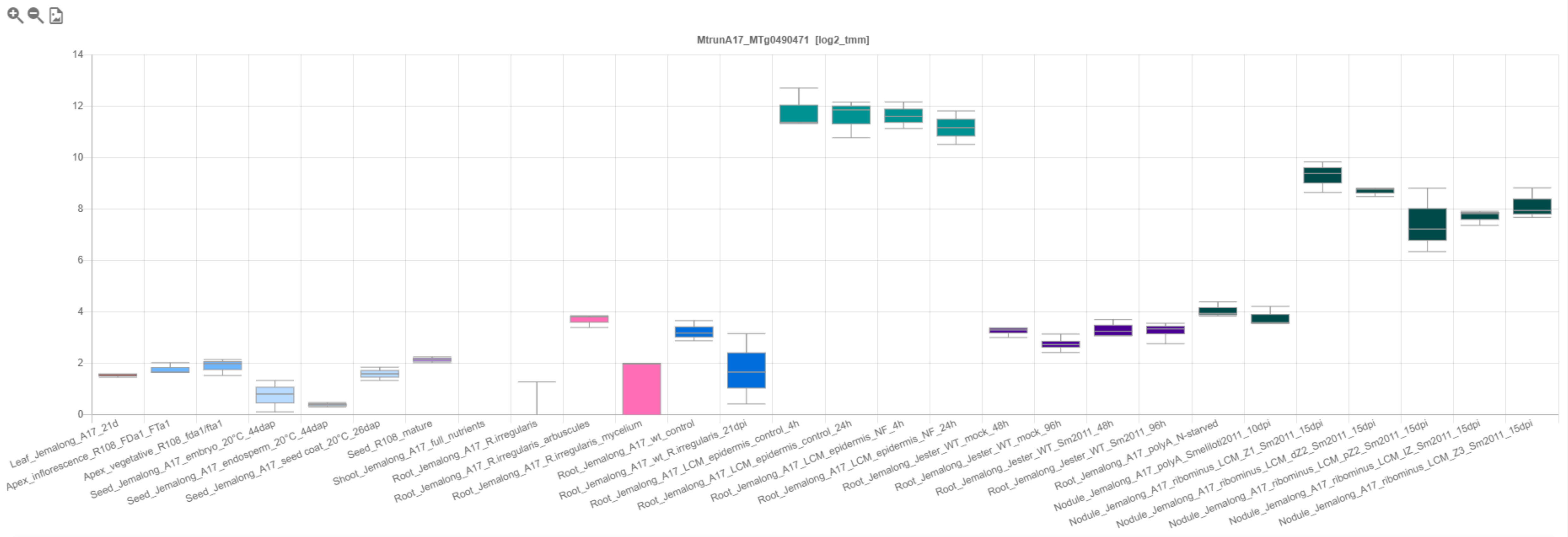

CP149: MtrunA17_MTg0490471

INRAE/CNRS Medicago Expressi | Expression Atlas Server

…/pub/expressionAtlas/app/v3/aa_reference_dataset/MtrunA17_MTg0490471

MtExpress V3 | Core (20220901)

! Switch to another dataset using the left menu

TMM | METADATA | SYNONYMOUS | ANNOTATION | GENOME PORTAL | LEGOO

mRNA: MtrunA17_MTg0490471;

Log2 TMM Normalisation using EdgeR (Core [20220901])

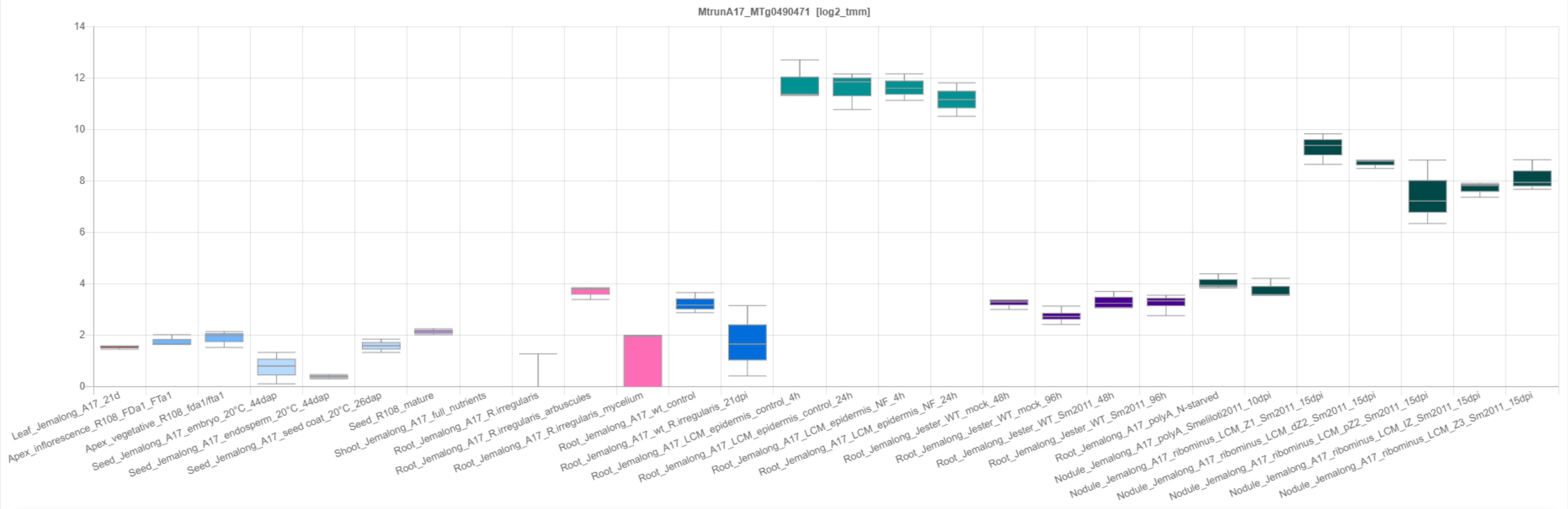

CP150: MtrunA17_MTg0490971

ncRNA: MtrunA17_MTg0490971;

Log2 TMM Normalisation using EdgeR (Core [20220901])

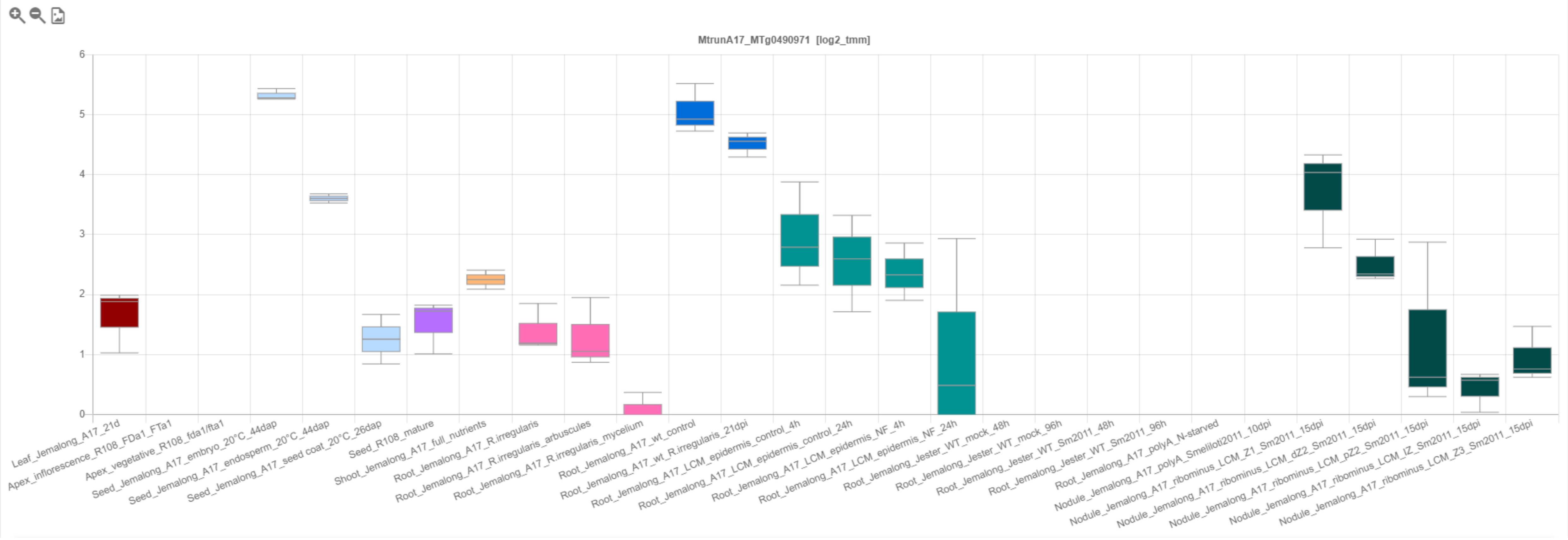

CP151: MtrunA17_MTg0490971

INRAE/CNRS Medicago Expressi | Expression Atlas Server

pub/expressionAtlas/app/v3/aa_reference_dataset/MtrunA17_MTg0490971

MtExpress V3 | Core (20220901) | Switch to another dataset using the left menu

TMM | METADATA | SYNONYMOUS | ANNOTATION | GENOME PORTAL | LEGOO

ncRNA: MtrunA17_MTg0490971;

Log2 TMM Normalisation using EdgeR (Core [20220901])

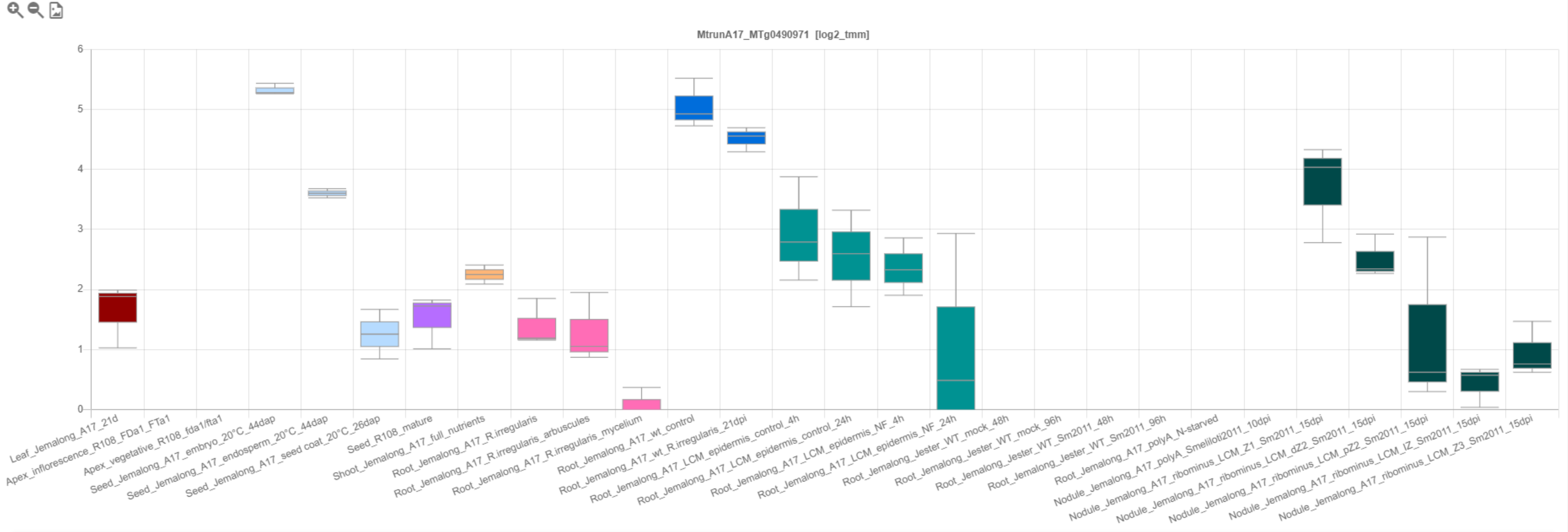

CP152: MtrunA17_MTg0491151

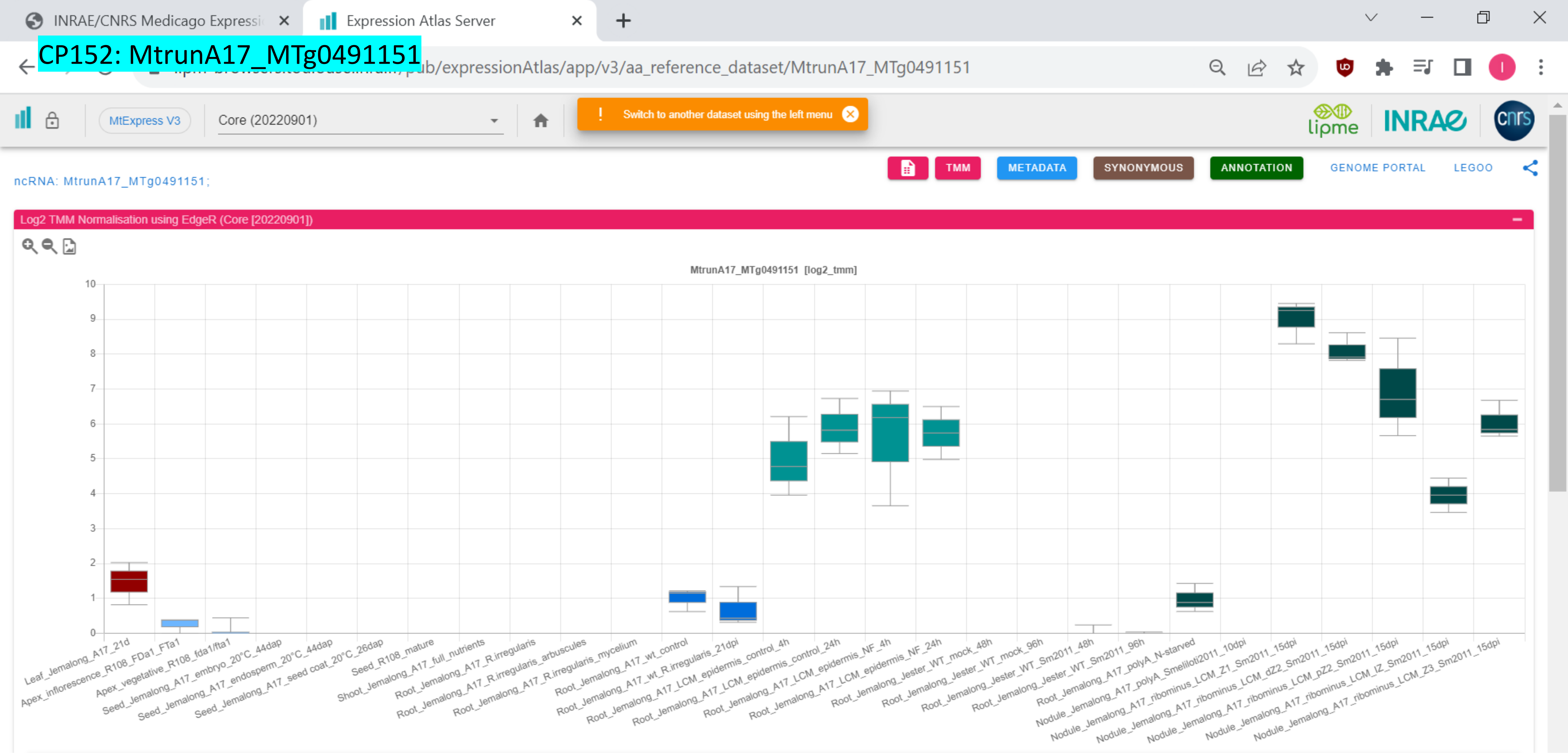

CP153: MtrunA17_MTg0491291

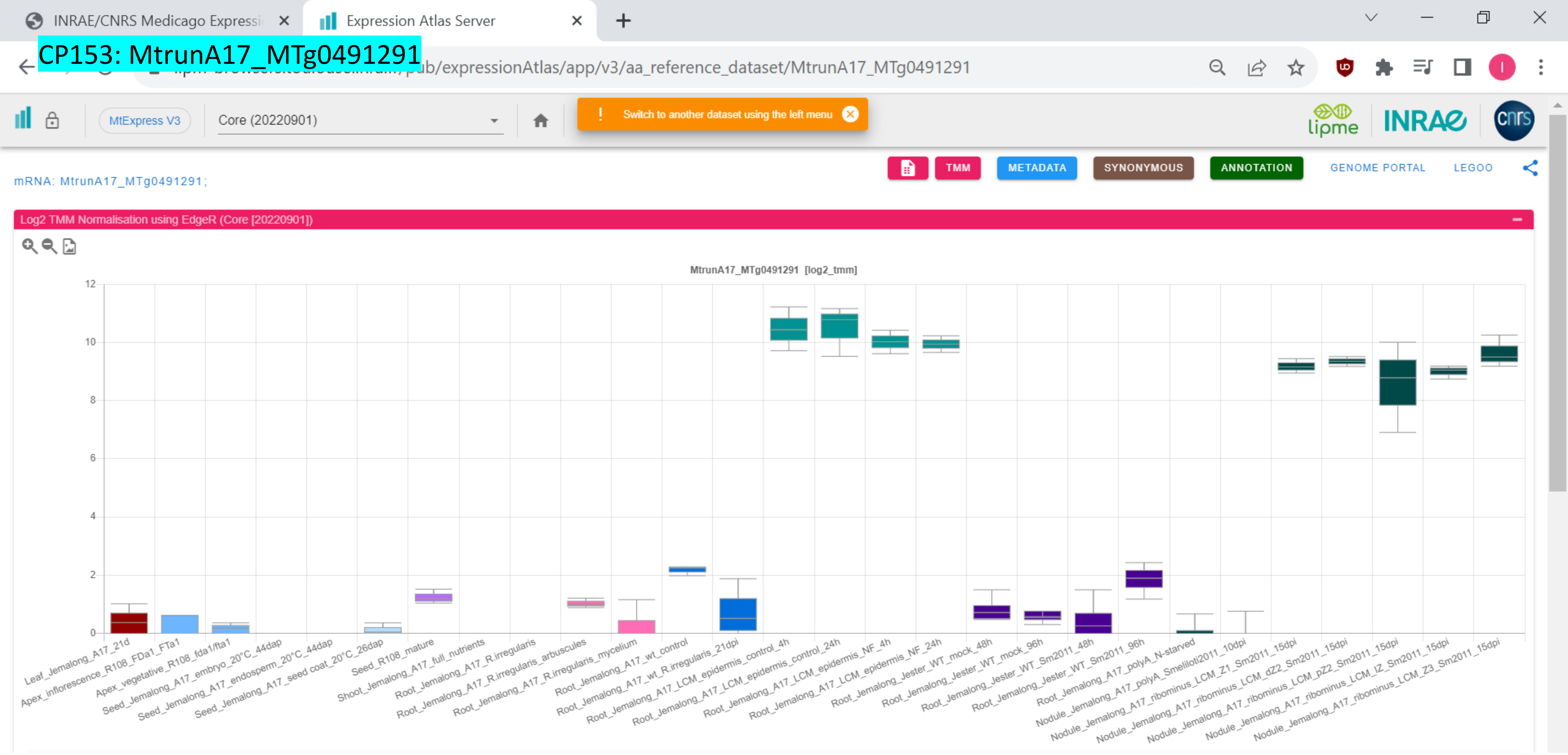

CP154: MtrunA17_MTg0491501

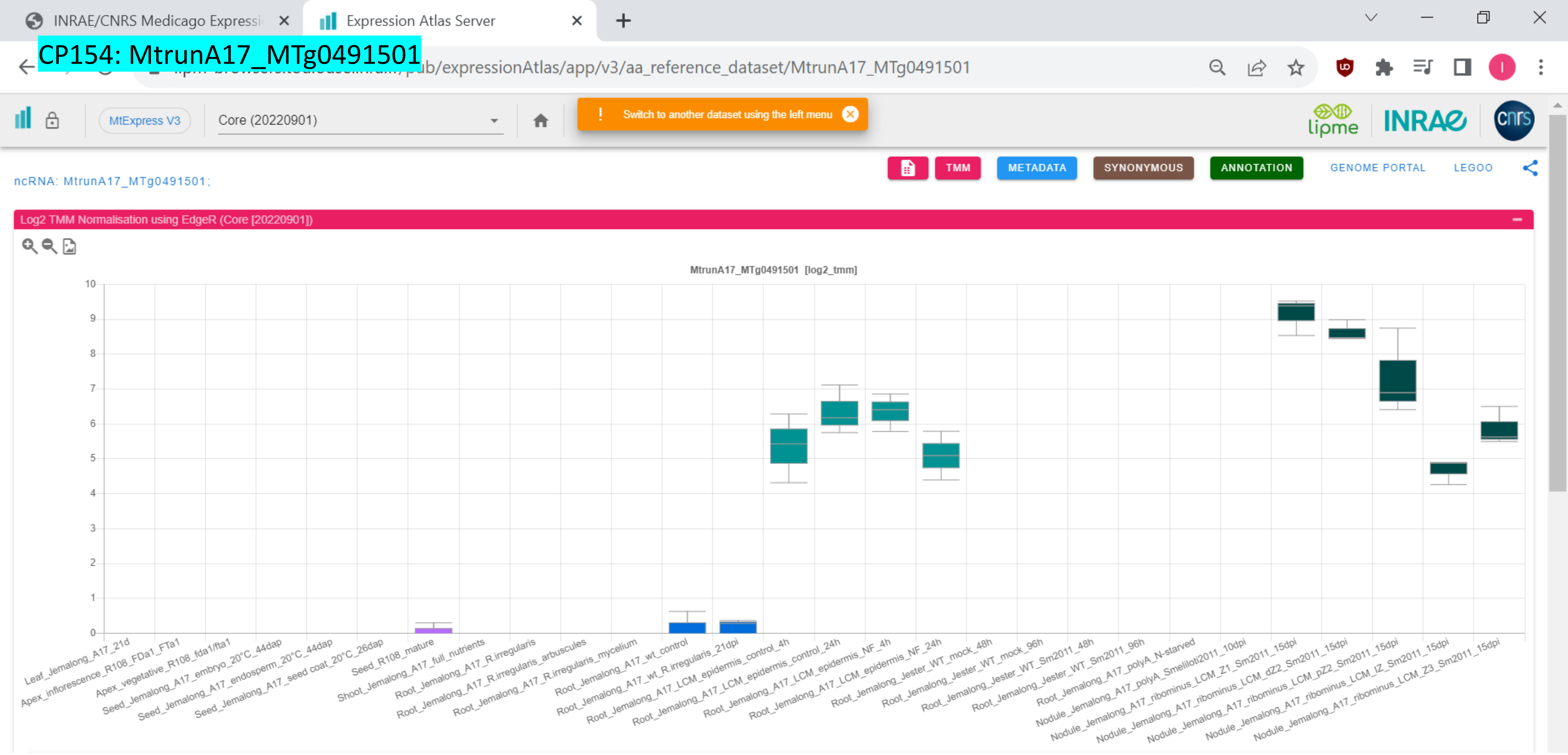

CP155: MtrunA17_MTg0491621

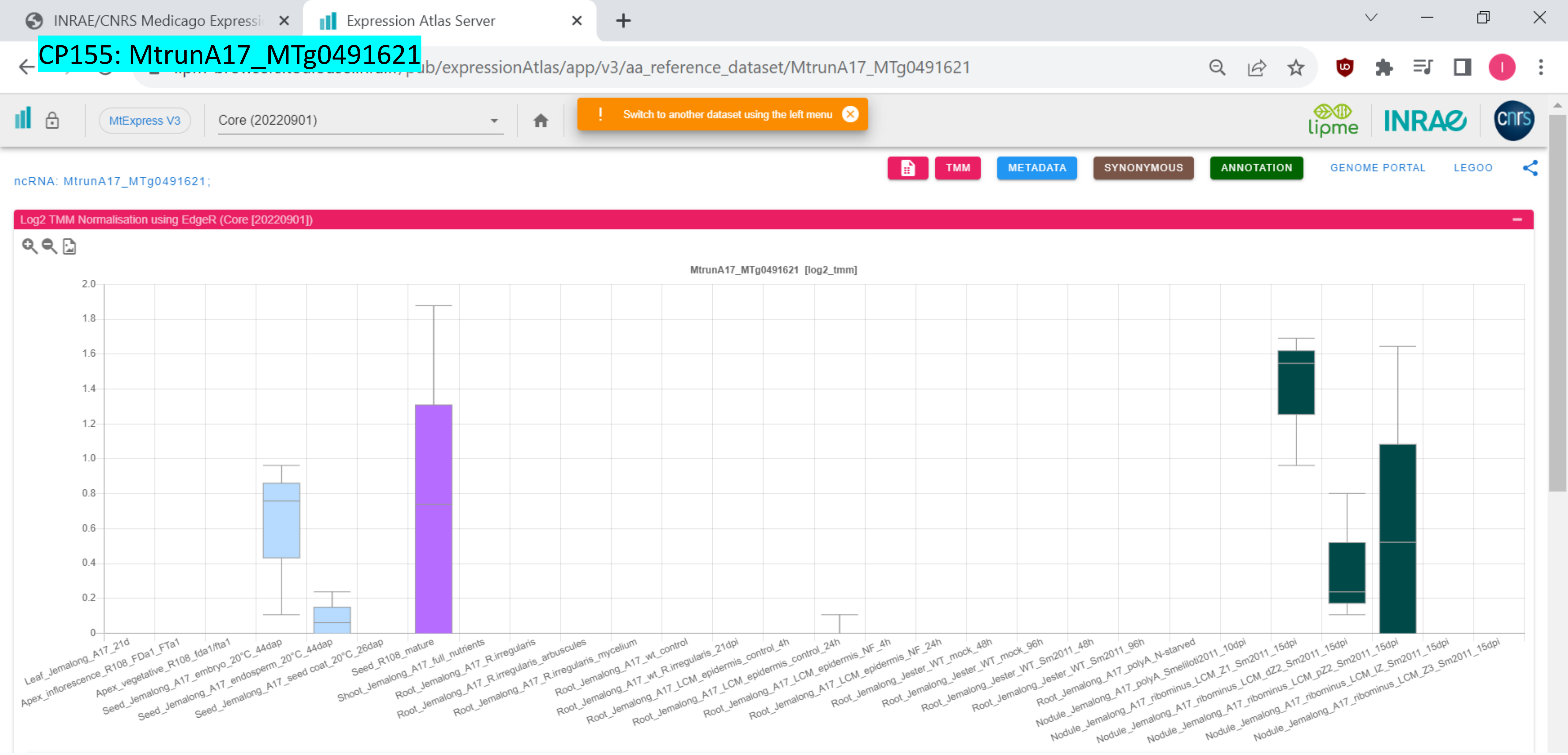

CP156: MtrunA17_MTg0491711

INRAE/CNRS Medicago Expressi | Expression Atlas Server

pub/expressionAtlas/app/v3/aa_reference_dataset/MtrunA17_MTg0491711

MtExpress V3 | Core (20220901) | Switch to another dataset using the left menu

TMM | METADATA | SYNONYMOUS | ANNOTATION | GENOME PORTAL | LEGOO

ncRNA: MtrunA17_MTg0491711;

Log2 TMM Normalisation using EdgeR (Core [20220901])

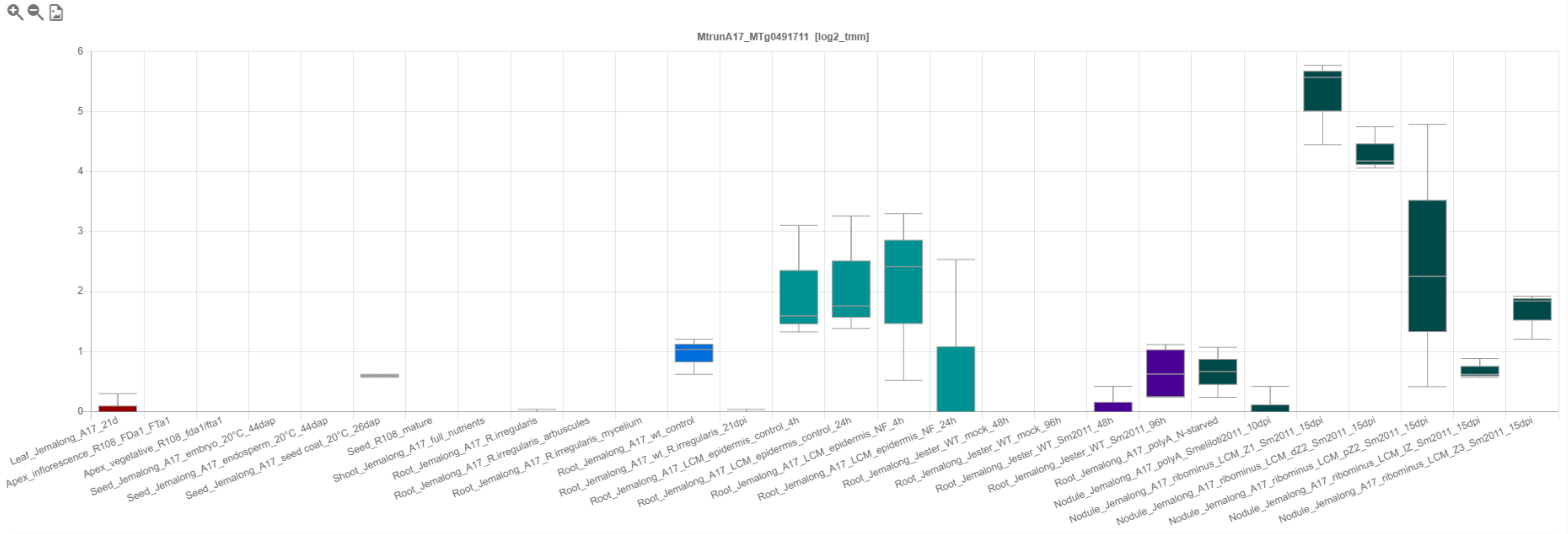

Supplementary Dataset S27. Expression profiles of 156 primary-source transcripts. Screenshots are from the RNA-Seq-based gene expression atlas of *Medicago truncatula* (MtExpress v. 3, https://medicago.toulouse.inrae.fr/GEA). For clarity and better visibility, only the core sample set is shown. Chimeric peptide identifiers that begin with an asterisk correspond to transcripts with log2 TMM values below zero.
