# Supplementary material for: Discovery of diverse chimeric peptides in a eukaryotic proteome sets the stage for experimental validation of the mosaic translation hypothesis
Source: Comput Struct Biotechnol J. 2025 Sep 12;27:4048–64. doi: 10.1016/j.csbj.2025.09.019 (PMC12481079; doi:10.1016/j.csbj.2025.09.019)
Supplement: Supplementary file 1 — Supplementary material [file mmc1.zip › Supplementary Datasets/Supplementary Dataset S31 Alignments of rRNA transcripts with known rRNA-like mRNA transcripts.pdf]

Nucleotide alignment (Alignment)

CP1: MtrunA17_Chr0c01g0489091

Mus musculus mRNA for testin2, X78990.1

58.6% in a region of 256 nt

Predict Primer Design Save

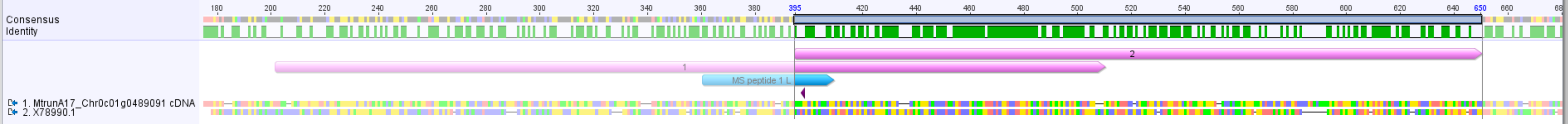

Selected 256 bases from base 395 to 650 (256 ungapped bases from base 395 to 650).

4 new notifications

CP1: MtrunA17_Chr0c01g0489091, altORF2

Mus musculus testin2, X78990.1

32.9% in a region of 70 aa

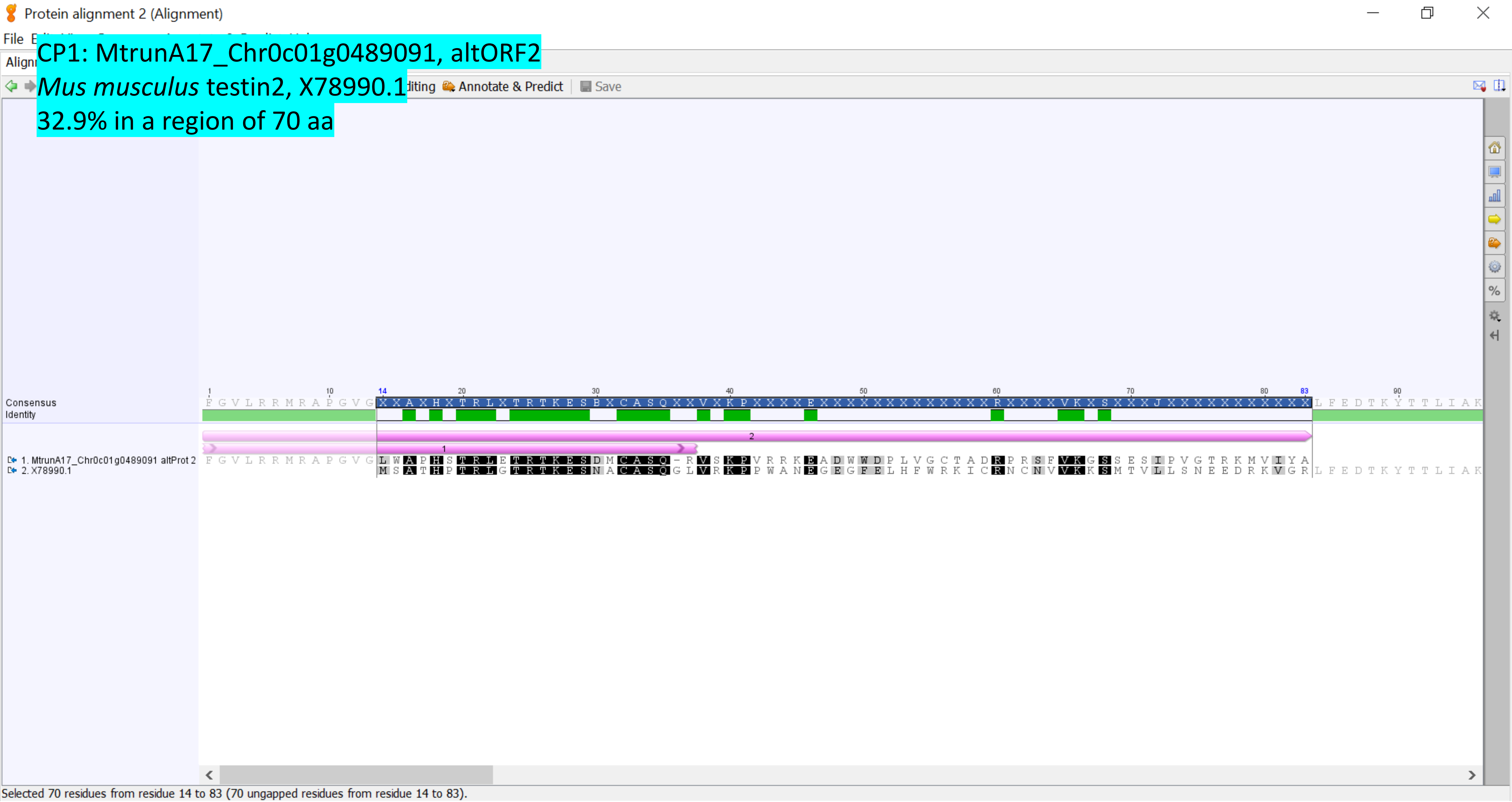

CP114: MtrunA17_Chr7g0229401

Homo sapiens Humanin (HN1) mRNA, complete cds, AY029066.1

67.4% in a region of 196 nt

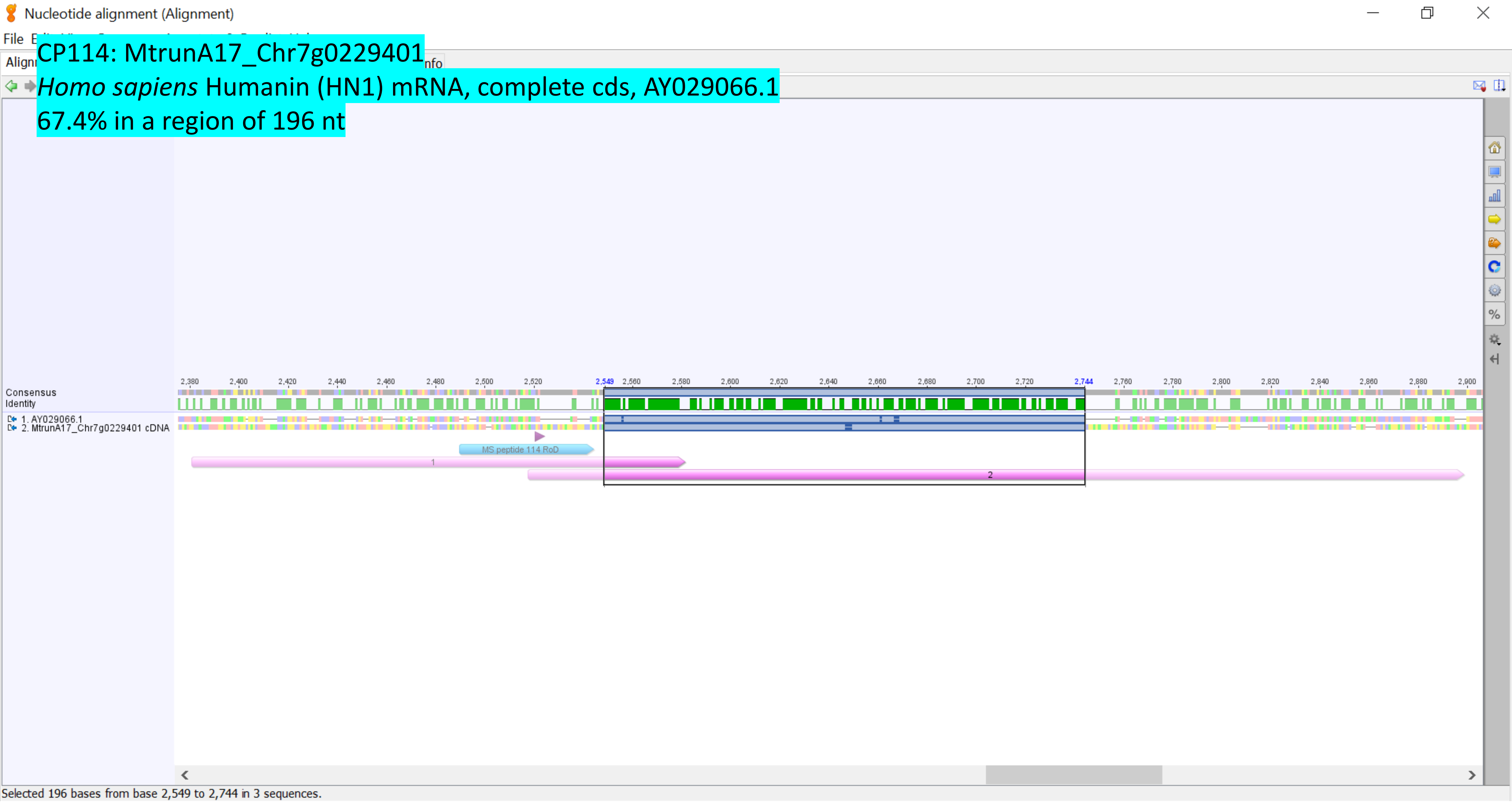

CP89: MtrunA17_Chr5g0422291

Candida albicans SC5314 Tar1p (TAR1), partial mRNA, XM_019475565.1

73.3% in a region of 285 nt

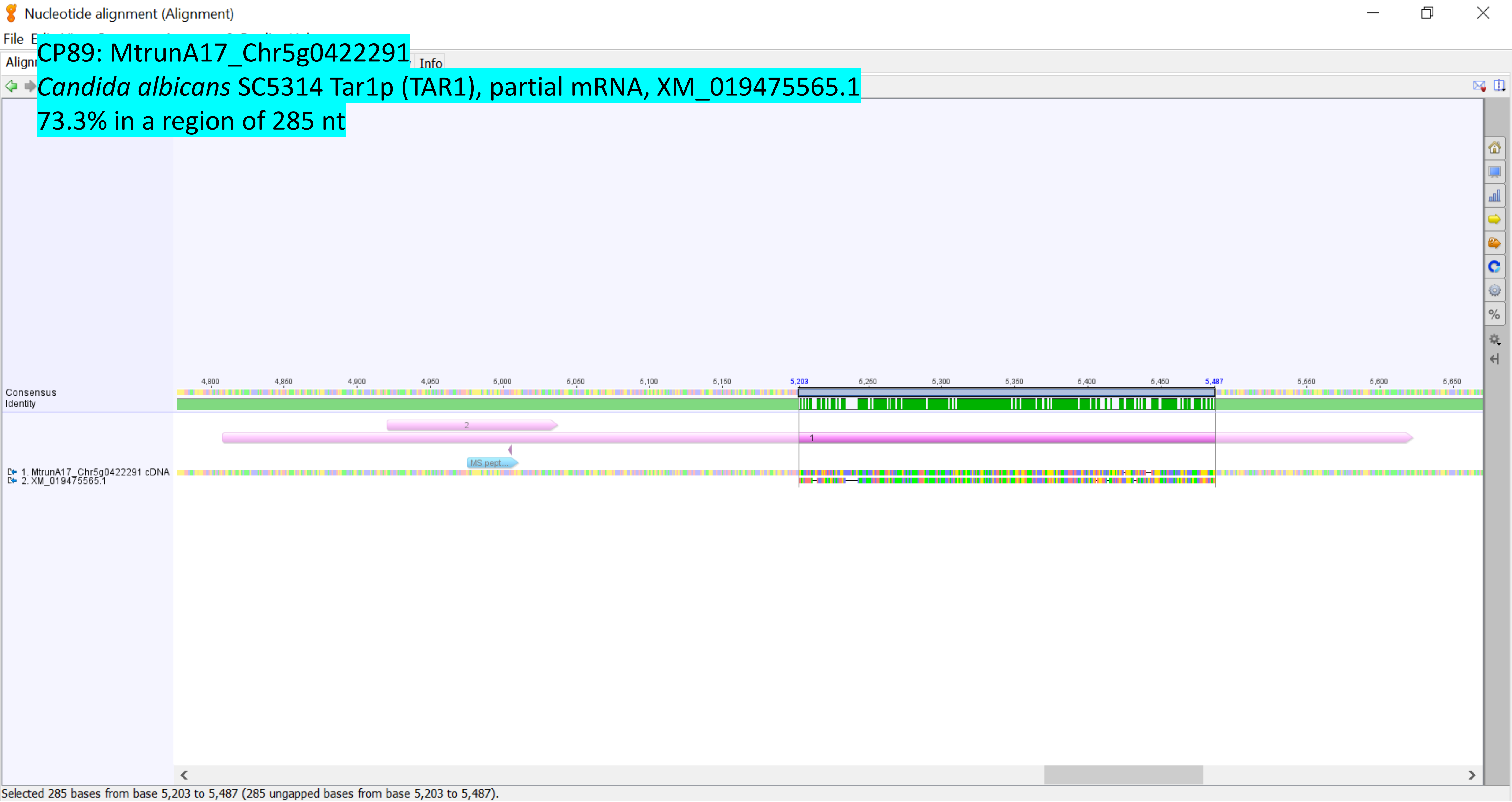

CP87: MtrunA17_Chr5g0421761

Reticulitermes flavipes rRNA-like mRNA, partial sequence, AY572860.1

80.5% in a region of 41 nt

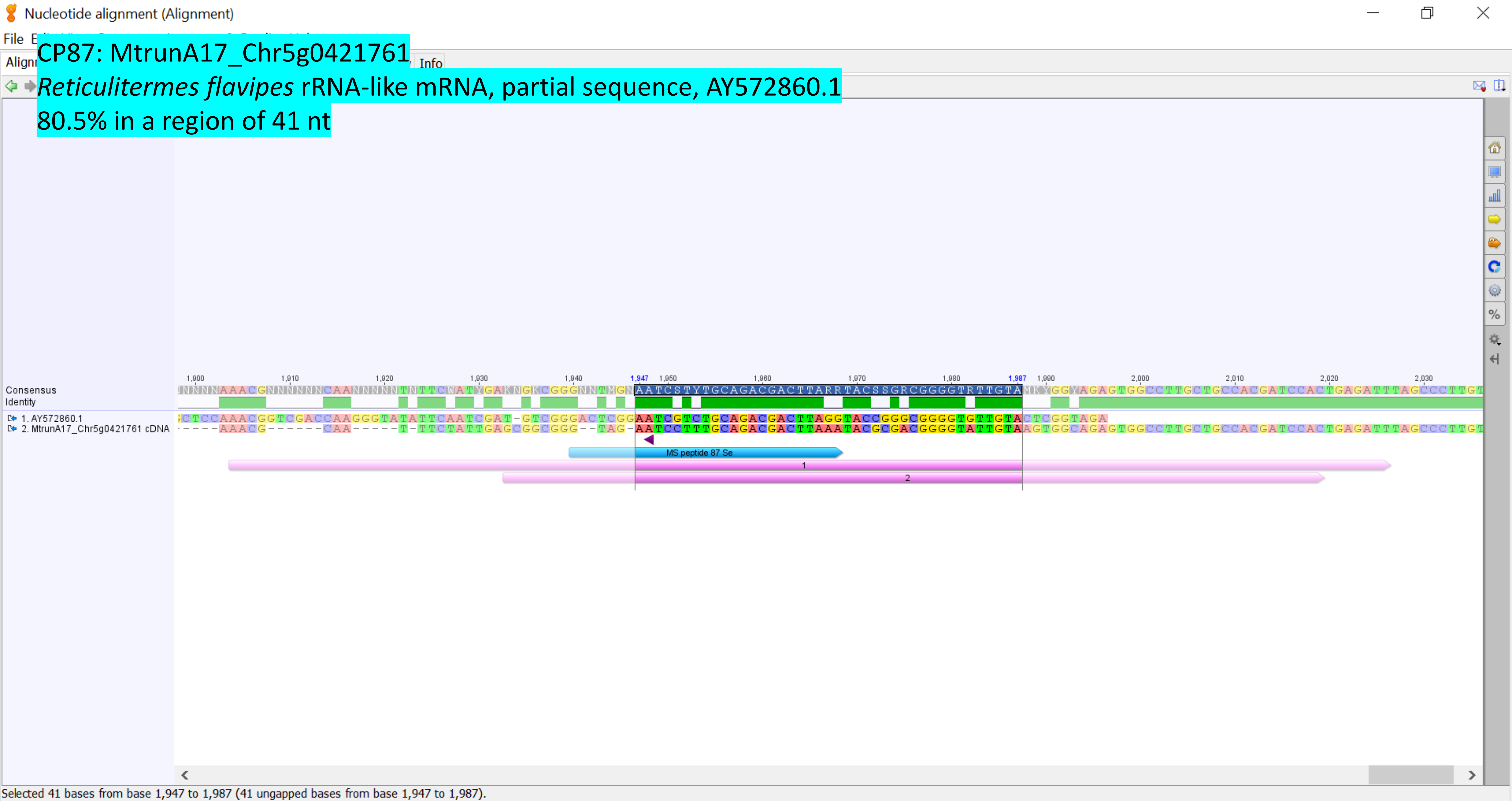

CP88: MtrunA17_Chr5g0422291

Reticulitermes flavipes rRNA-like mRNA, partial sequence, AY572860.1

88.3% in a region of 417 nt

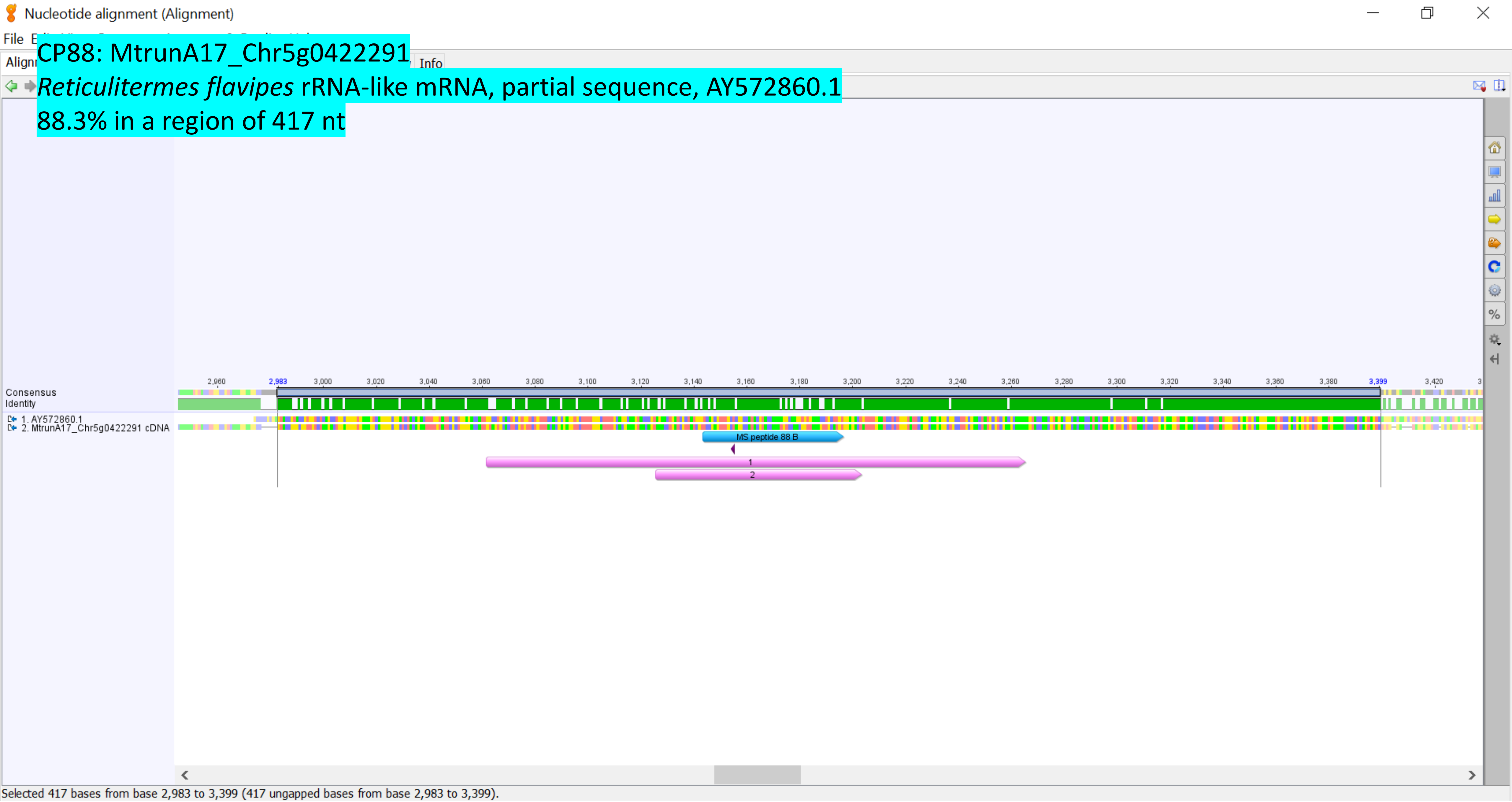

Supplementary Dataset S31. Alignments of four PRF-related rRNA transcripts with known rRNA-like mRNA transcripts. Percent identity values are based on Geneious® alignments (Geneious® v. 7.1, Dotmatics Ltd., MA, USA, https://www.geneious.com). Codes at the end of the annotated gene names are GenBank identifiers. Alternative ORFs (altORFs) are shown in pink. Numbers 1 and 2 on altORFs indicate the following. An altORF1 in this study is defined as the one that starts earlier than an altORF2. Switches from altORF2 to altORF1 are also found in this dataset but are five times less frequent (e.g. CP108 and four other CPs). Positions and the direction of programmed ribosomal frameshifting (PRF) events are labeled with deep purple arrowheads.
