# Supplementary material for: Discovery of diverse chimeric peptides in a eukaryotic proteome sets the stage for experimental validation of the mosaic translation hypothesis
Source: Comput Struct Biotechnol J. 2025 Sep 12;27:4048–64. doi: 10.1016/j.csbj.2025.09.019 (PMC12481079; doi:10.1016/j.csbj.2025.09.019)
Supplement: Supplementary file 1 — Supplementary material [file mmc1.zip › Supplementary Datasets/Supplementary Dataset S4 Primary-source transcripts associated with multiple PRF events.pdf]

MtrunA17_Chr1g0185811

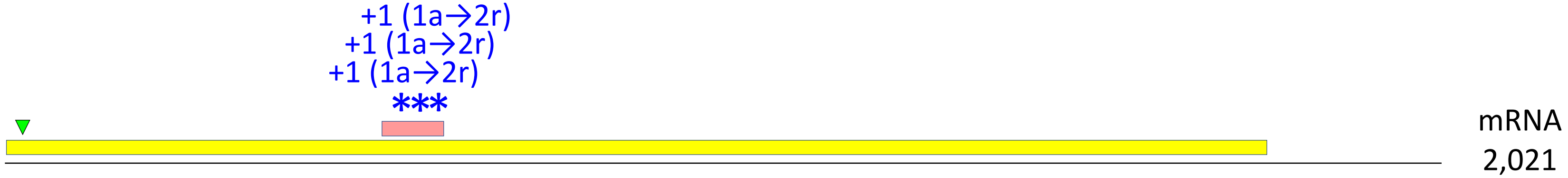

CP17: MtrunA17_Chr1g0185811_1F_535-621_87_MtrunA17_Chr1g0185811_2F_2-1774_1773_+1_iteration_2

MS peptide 17 Se
CP17

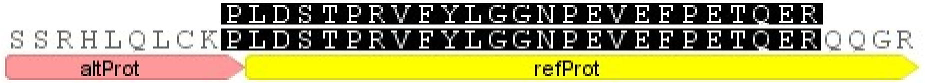

CP18: MtrunA17_Chr1g0185811_1F_535-621_87_MtrunA17_Chr1g0185811_2F_2-1774_1773_+1_iteration_9

MS peptide 18 Se
CP18

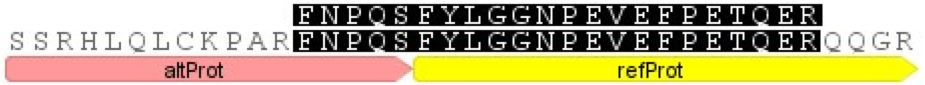

CP16: MtrunA17_Chr1g0185811_1F_535-621_87_MtrunA17_Chr1g0185811_2F_2-1774_1773_+1_iteration_10

MS peptide 16 Se
CP16

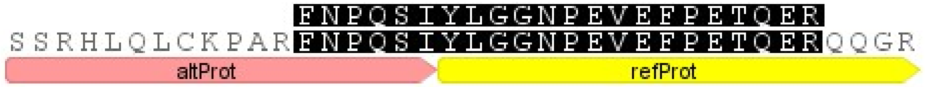

MtrunA17_Chr1g0200071

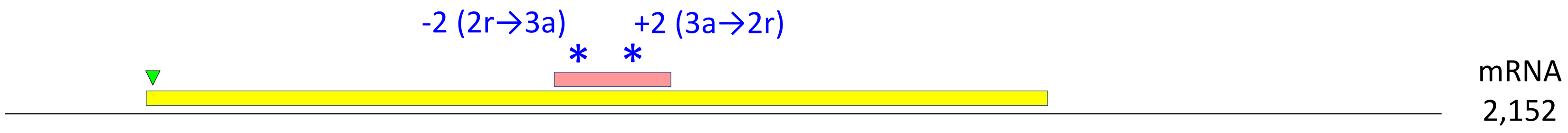

CP24: MtrunA17_Chr1g0200071_3F_828-1001_174_MtrunA17_Chr1g0200071_2F_218-1567_1350_-2_iteration_11

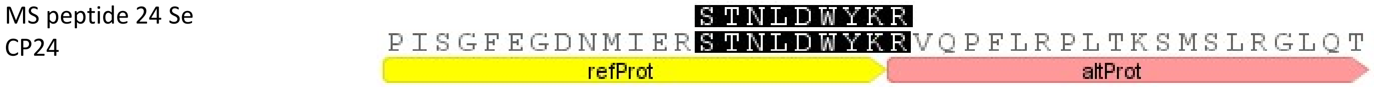

CP23: MtrunA17_Chr1g0200071_3F_828-1001_174_MtrunA17_Chr1g0200071_2F_218-1567_1350_+2_iteration_1

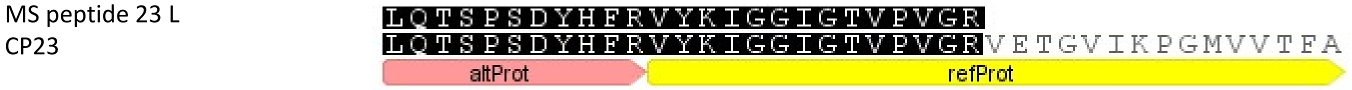

MtrunA17_Chr3g0144151

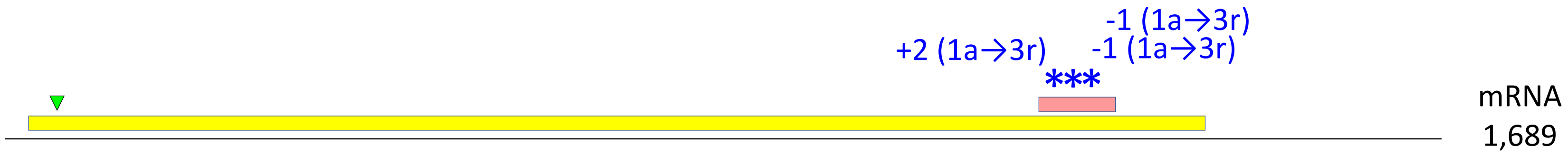

CP63: MtrunA17_Chr3g0144151_1F_1222-1311_90_MtrunA17_Chr3g0144151_3F_33-1415_1383_+2_iteration_3

MS peptide 63 L
CP63

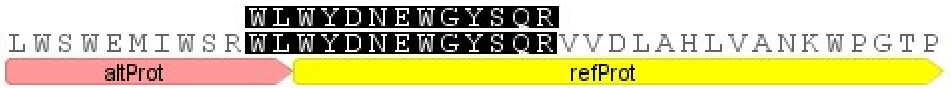

CP64: MtrunA17_Chr3g0144151_1F_1222-1311_90_MtrunA17_Chr3g0144151_3F_33-1415_1383_-1_iteration_5

MS peptide 64 FLStWPCPD
CP64

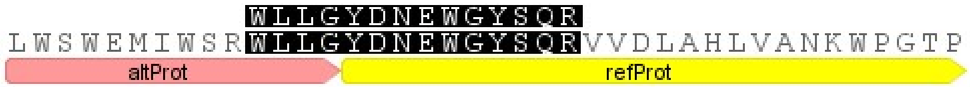

CP65: MtrunA17_Chr3g0144151_1F_1222-1311_90_MtrunA17_Chr3g0144151_3F_33-1415_1383_-1_iteration_6

MS peptide 65 LStW
CP65

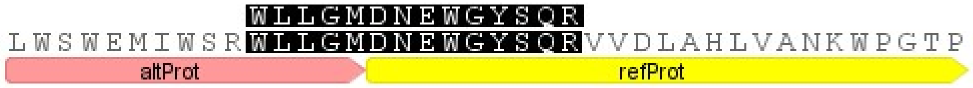

MtrunA17_Chr5g0422291

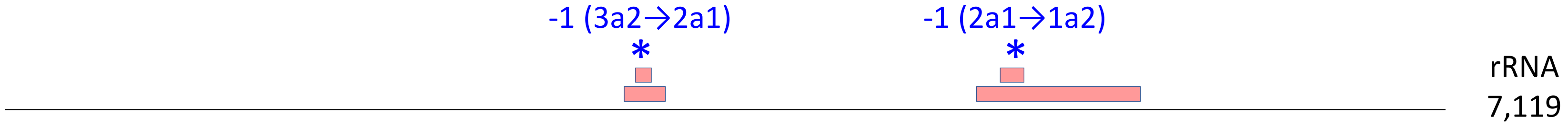

CP88: MtrunA17_Chr5g0422291_2F_3056-3259_204_MtrunA17_Chr5g0422291_3F_3120-3197_78_-1_iteration_5

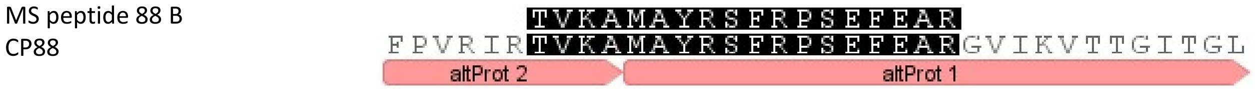

CP89: MtrunA17_Chr5g0422291_2F_4808-5617_810_MtrunA17_Chr5g0422291_1F_4921-5037_117_-1_iteration_29

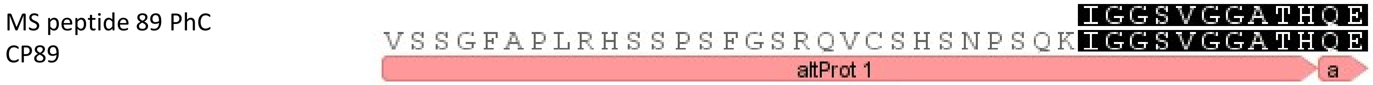

MtrunA17_Chr5g0430341

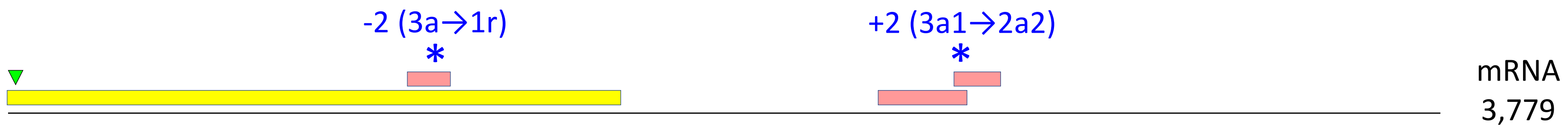

CP91: MtrunA17_Chr5g0430341_3F_1059-1172_114_MtrunA17_Chr5g0430341_1F_1-1620_1620_-2_iteration_9

MS peptide 91 W
CP91

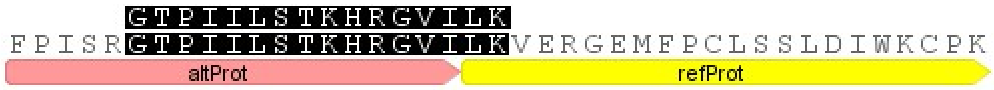

CP90: MtrunA17_Chr5g0430341_2F_2501-2623_123_MtrunA17_Chr5g0430341_3F_2298-2531_234_+2_iteration_3

MS peptide 90 L
CP90

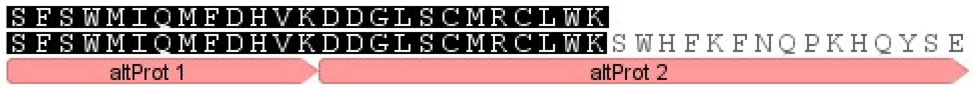

MtrunA17_Chr6g0457461

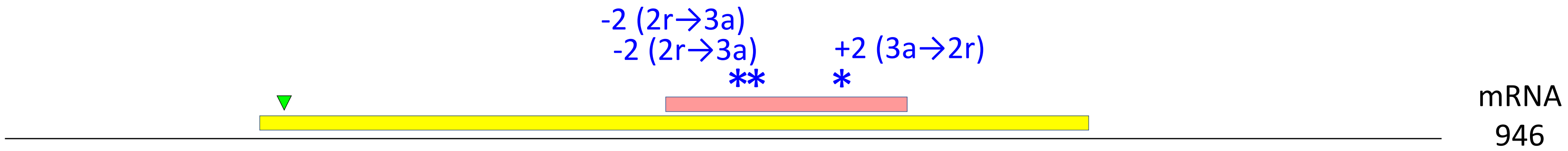

CP99: MtrunA17_Chr6g0457461_3F_438-596_159_MtrunA17_Chr6g0457461_2F_170-715_546_-2_iteration_17

MS peptide 99 BSeW
CP99

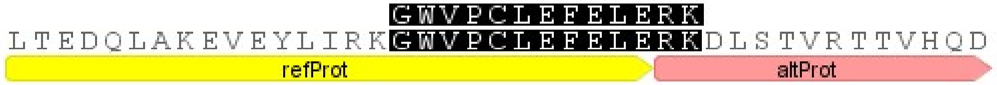

<u>CP100</u>: MtrunA17_Chr6g0457461_3F_438-596_159_MtrunA17_Chr6g0457461_2F_170-715_546_-2_iteration_18

MS peptide 100 BWPCPD
CP100

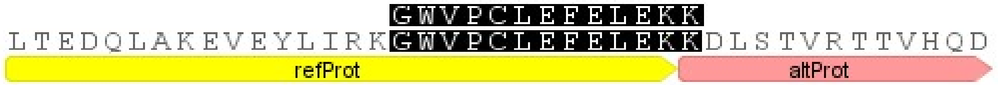

CP98: MtrunA17_Chr6g0457461_3F_438-596_159_MtrunA17_Chr6g0457461_2F_170-715_546_+2_iteration_6

MS peptide 98 B
CP98

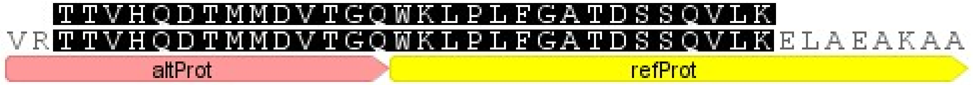

MtrunA17_MTg0490471

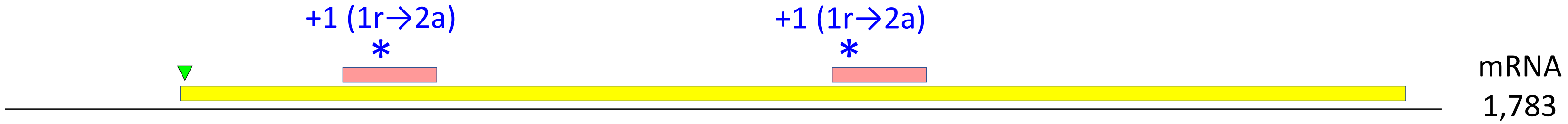

CP149: MtrunA17_MTg0490471_2F_422-538_117_MtrunA17_MTg0490471_1F_220-1740_1521_+1_iteration_16

MS peptide 149 N10N14FLSe
CP149

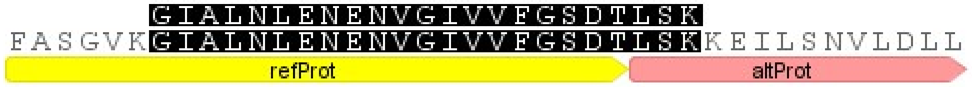

<u>CP148</u>: MtrunA17_MTg0490471_2F_1031-1147_117_MtrunA17_MTg0490471_1F_220-1740_1521_+1_iteration_7

MS peptide 148 Se
CP148

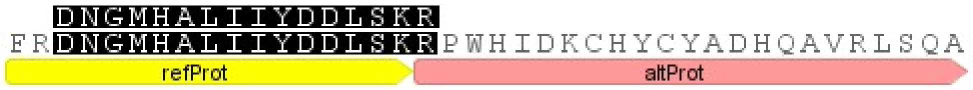

MtrunA17_MTg0490971

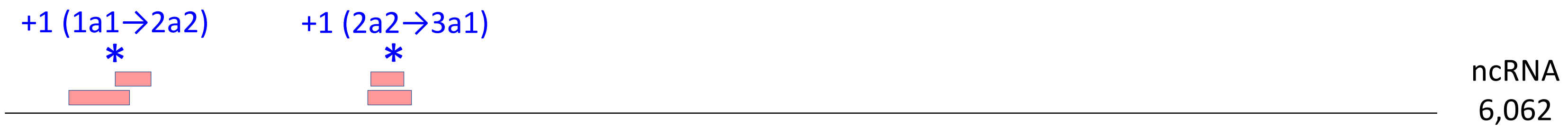

CP151: MtrunA17_MTg0490971_2F_476-628_153_MtrunA17_MTg0490971_1F_277-534_258_+1_iteration_0

MS peptide 151 W
CP151

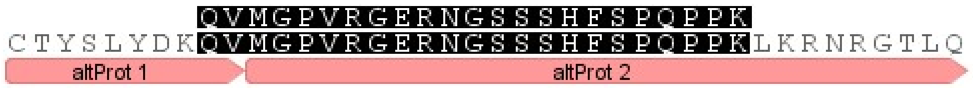

CP150: MtrunA17_MTg0490971_2F_1562-1699_138_MtrunA17_MTg0490971_3F_1542-1727_186_+1_iteration_12

MS peptide 150 St
CP150

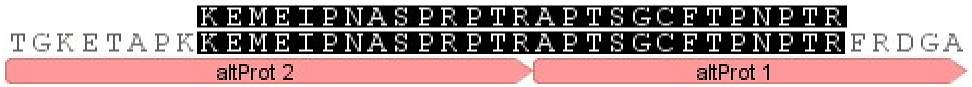

Supplementary Dataset S4. Eight primary-source transcripts associated with multiple PRF events. Chimeric peptide models (CPs) and corresponding MS peptides (bottom) are shown together with transcript models (top) that feature the transcript type, length in nucleotides, and relative positions of ORFs (to scale) involved in the production of CPs. Reference ORFs (refORFs) and reference proteins (refProts) are shown in yellow. Alternative ORFs (altORFs) and alternative proteins (altProts) are shown in pink. The first in-frame start codon (AUG) in each refORF is marked with a green triangle. Codes of CPs modeled with MS-supported altProts are shown in bold. Codes of CPs with "confident" MS peptide detection in at least one sample are underlined. Transcript models also show the positions and characteristics of PRF events, which are mapped with an asterisk. The description of a PRF event should be interpreted as follows. For example, CP88, -1 (3a2→2a1): a minus 1 frameshift changes the translation from frame 3 to frame 2, which corresponds to the change from altORF2 to altORF1. An altORF1 in this study is defined as the one that starts earlier than an altORF2. Throughout this study, unique numbers are assigned to chimeric models and their matching chimeric MS peptides. MS peptide identifiers also contain codes of biological samples in which they were detected. For example, the chimeric MS peptide of CP65 was identified in leaves, stems, and the whole plant. Thus, the identifier of MS peptide 65 ends with "LStW".
