# Supplementary material for: Discovery of diverse chimeric peptides in a eukaryotic proteome sets the stage for experimental validation of the mosaic translation hypothesis
Source: Comput Struct Biotechnol J. 2025 Sep 12;27:4048–64. doi: 10.1016/j.csbj.2025.09.019 (PMC12481079; doi:10.1016/j.csbj.2025.09.019)
Supplement: Supplementary file 1 — Supplementary material [file mmc1.zip › Supplementary Datasets/Supplementary Dataset S7 Folding of chimeric protein models Part 2.pdf]

CP51: MtrunA17_Chr3g0096421

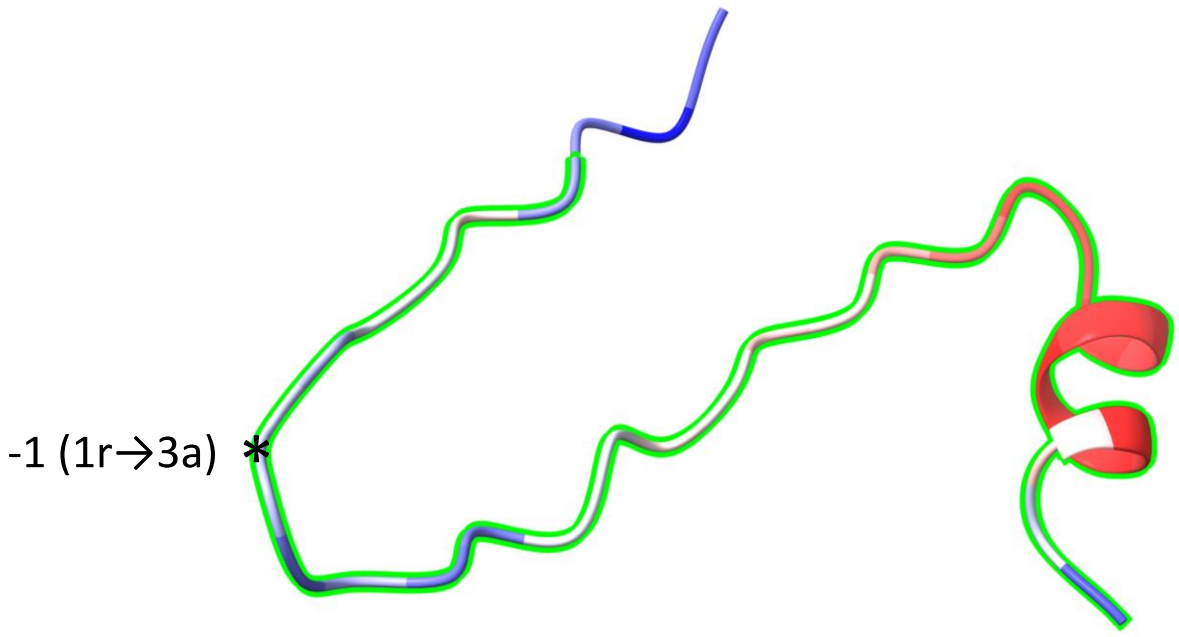

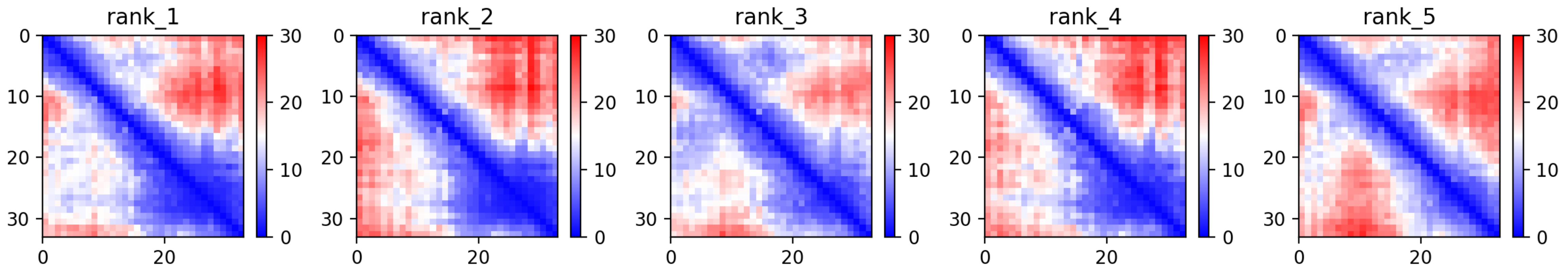

CP51: MtrunA17_Chr3g0096421

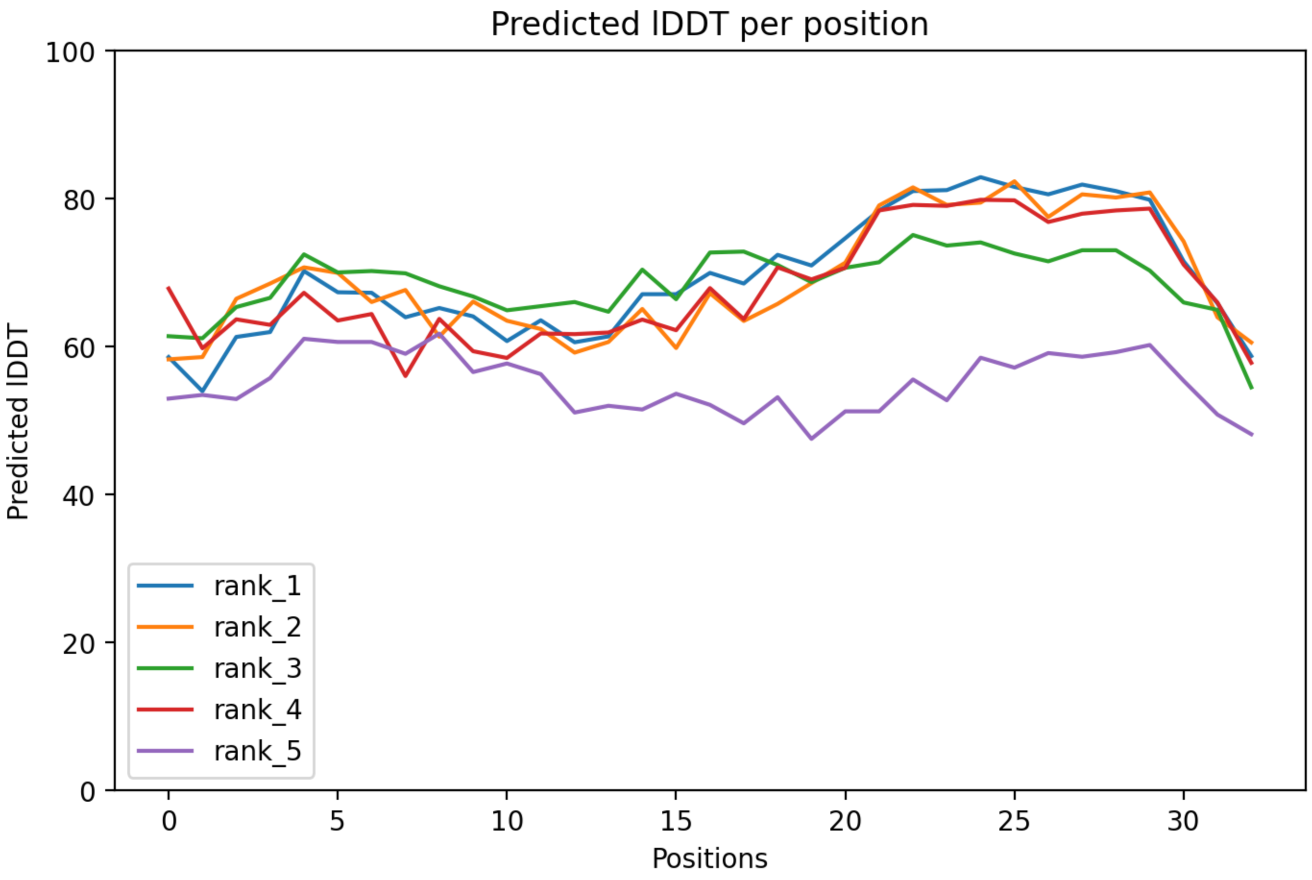

CP51: MtrunA17_Chr3g0096421

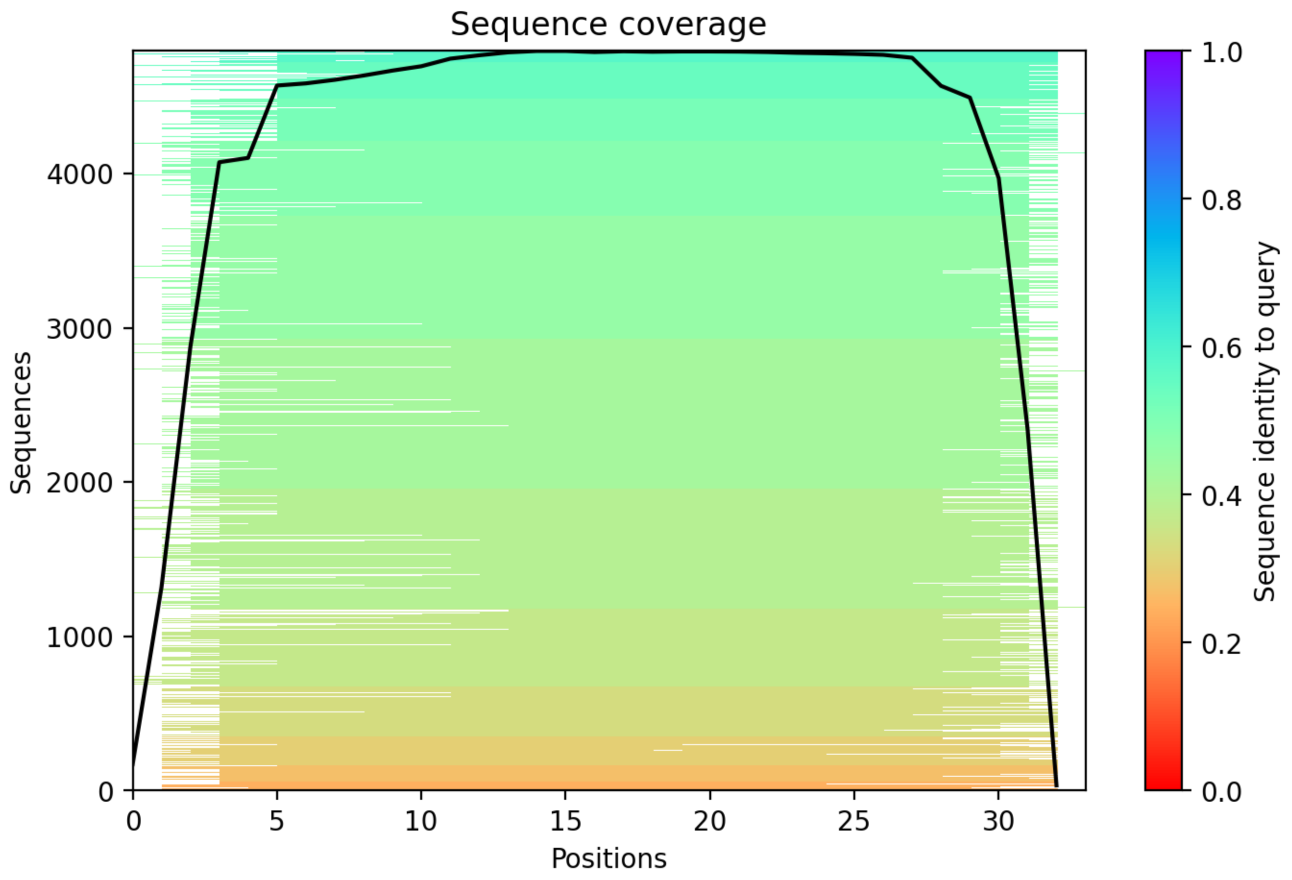

CP52: MtrunA17_Chr3g0100221

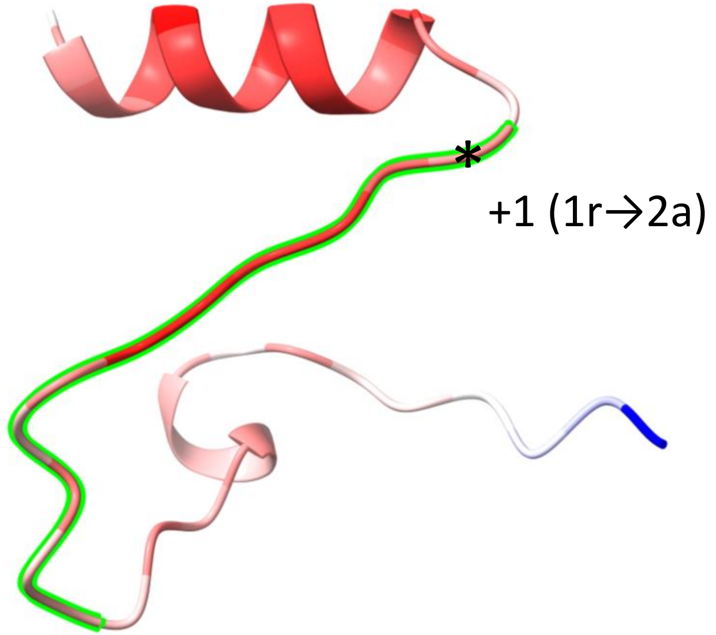

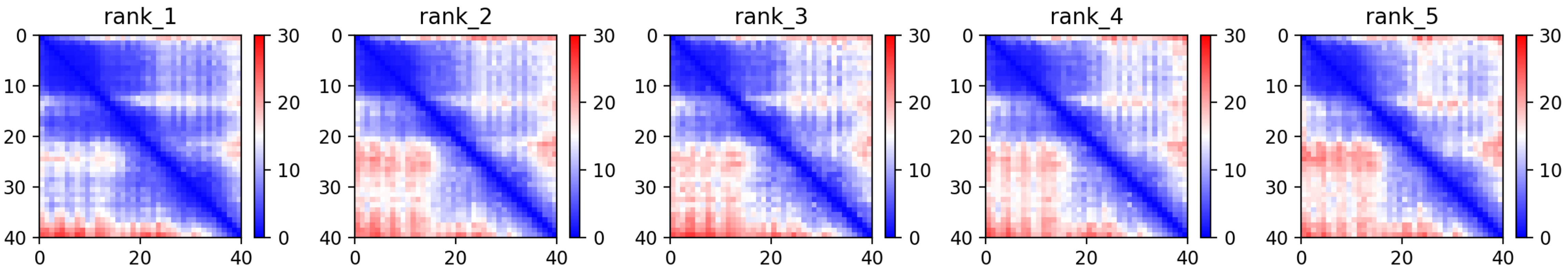

CP52: MtrunA17_Chr3g0100221

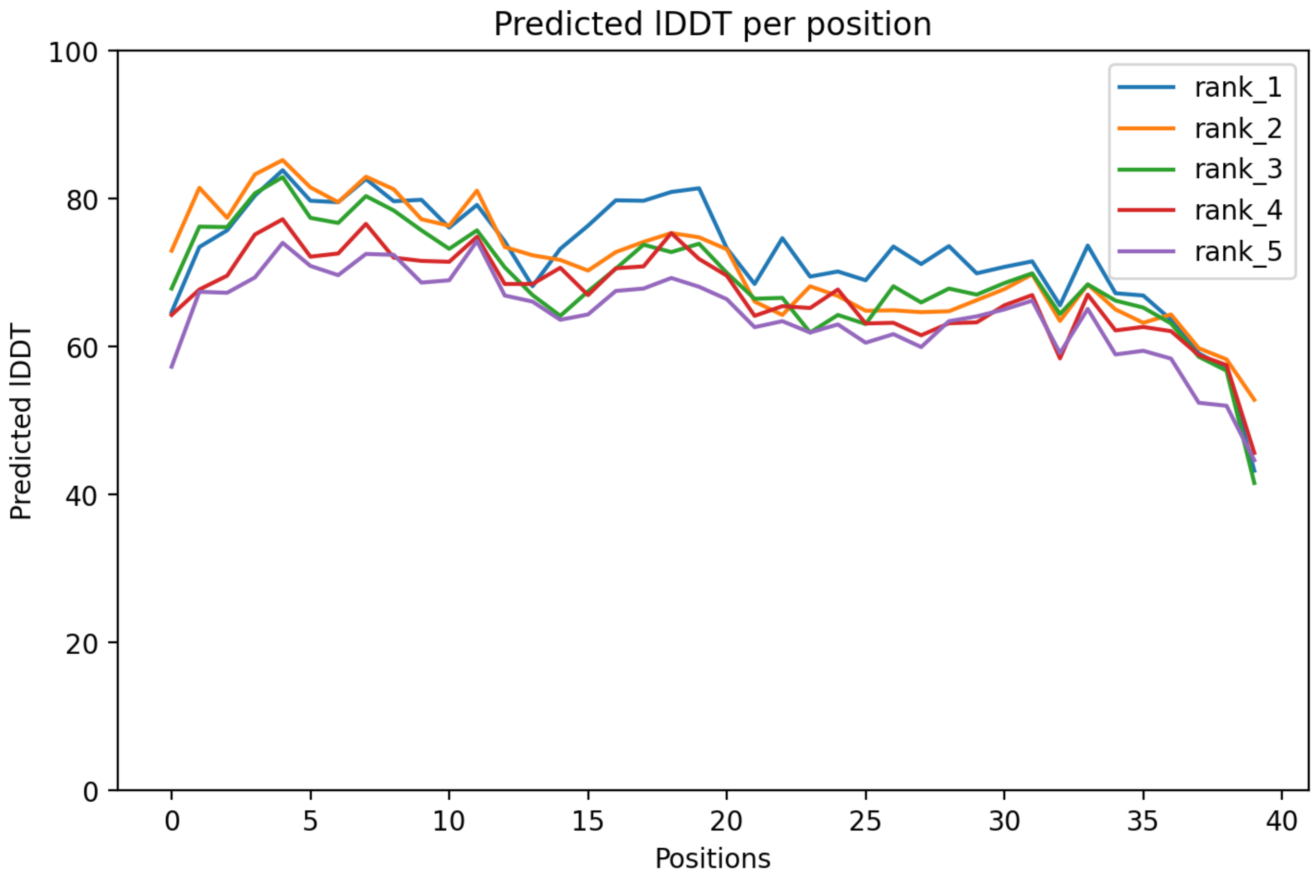

CP52: MtrunA17_Chr3g0100221

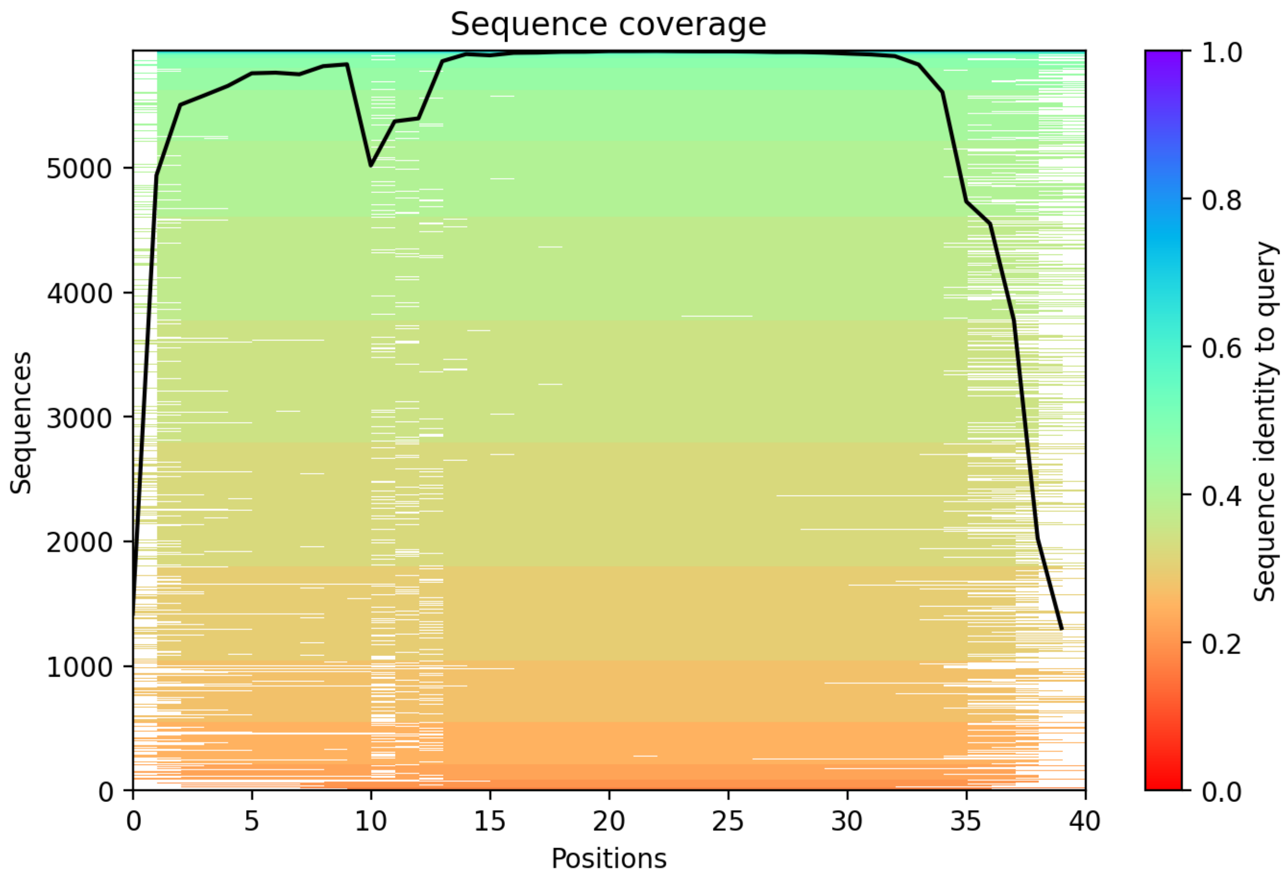

CP53: MtrunA17_Chr3g0102171

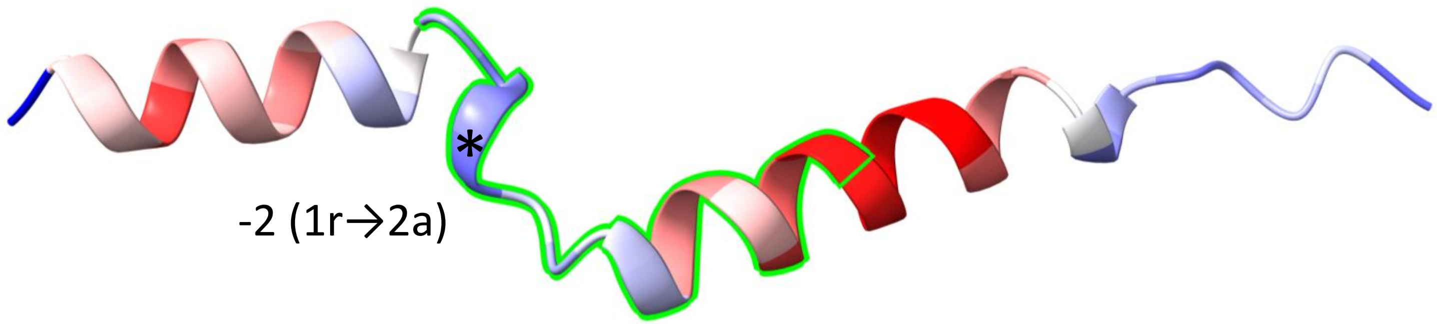

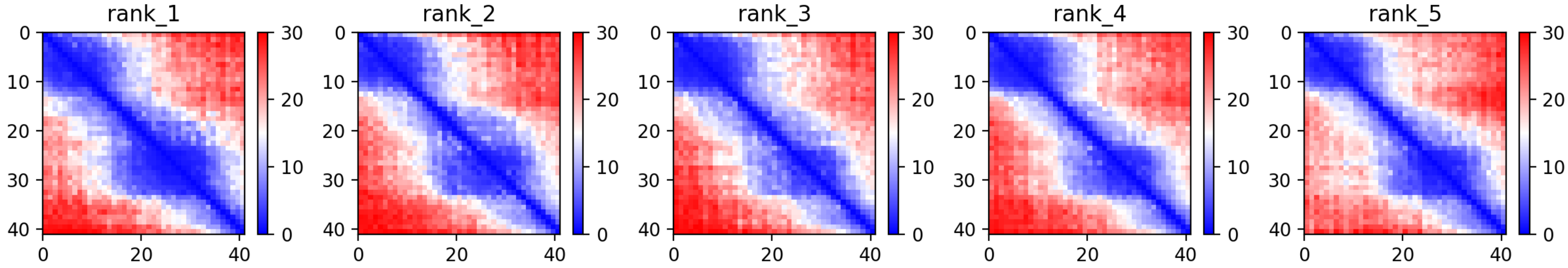

CP53: MtrunA17_Chr3g0102171

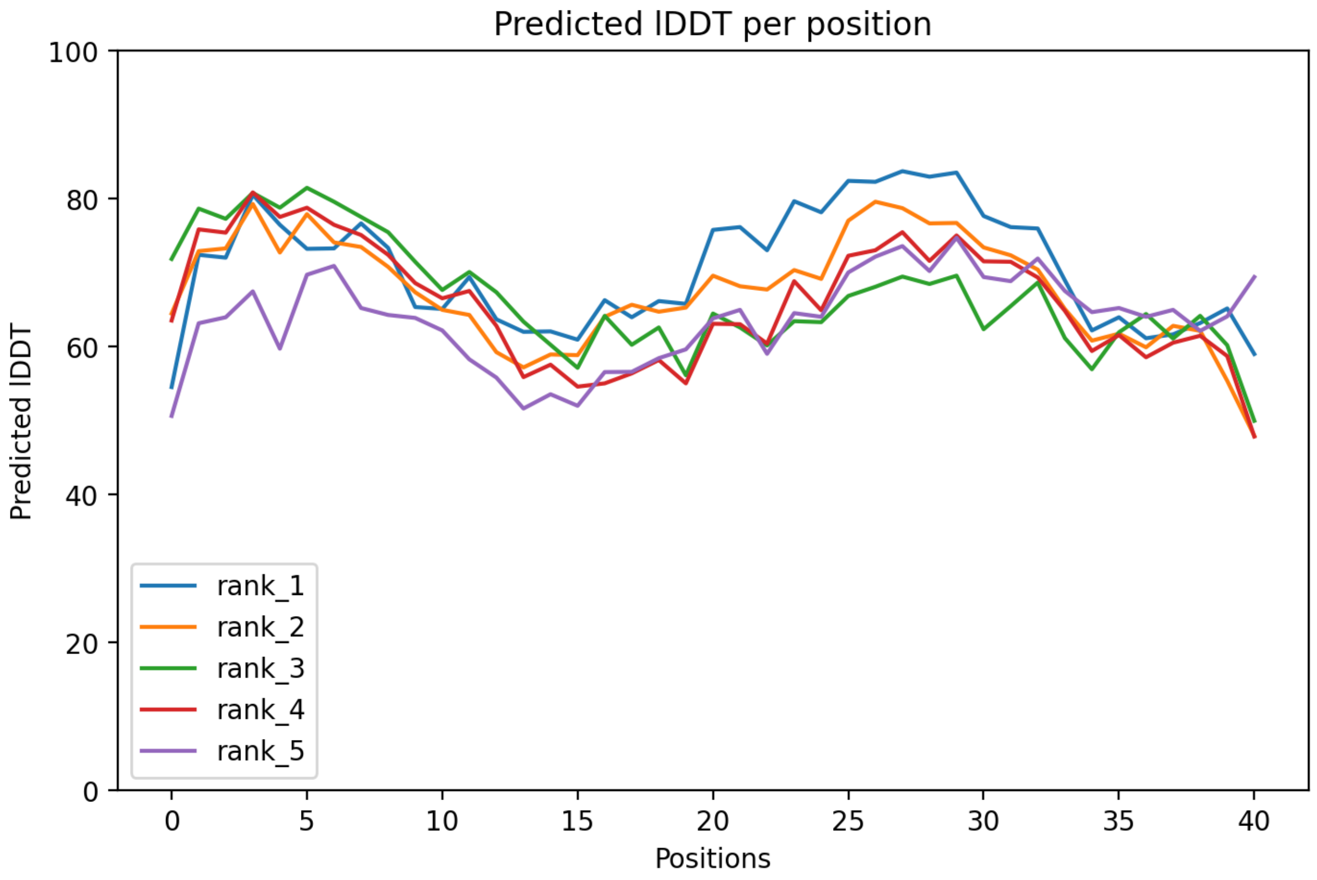

CP53: MtrunA17_Chr3g0102171

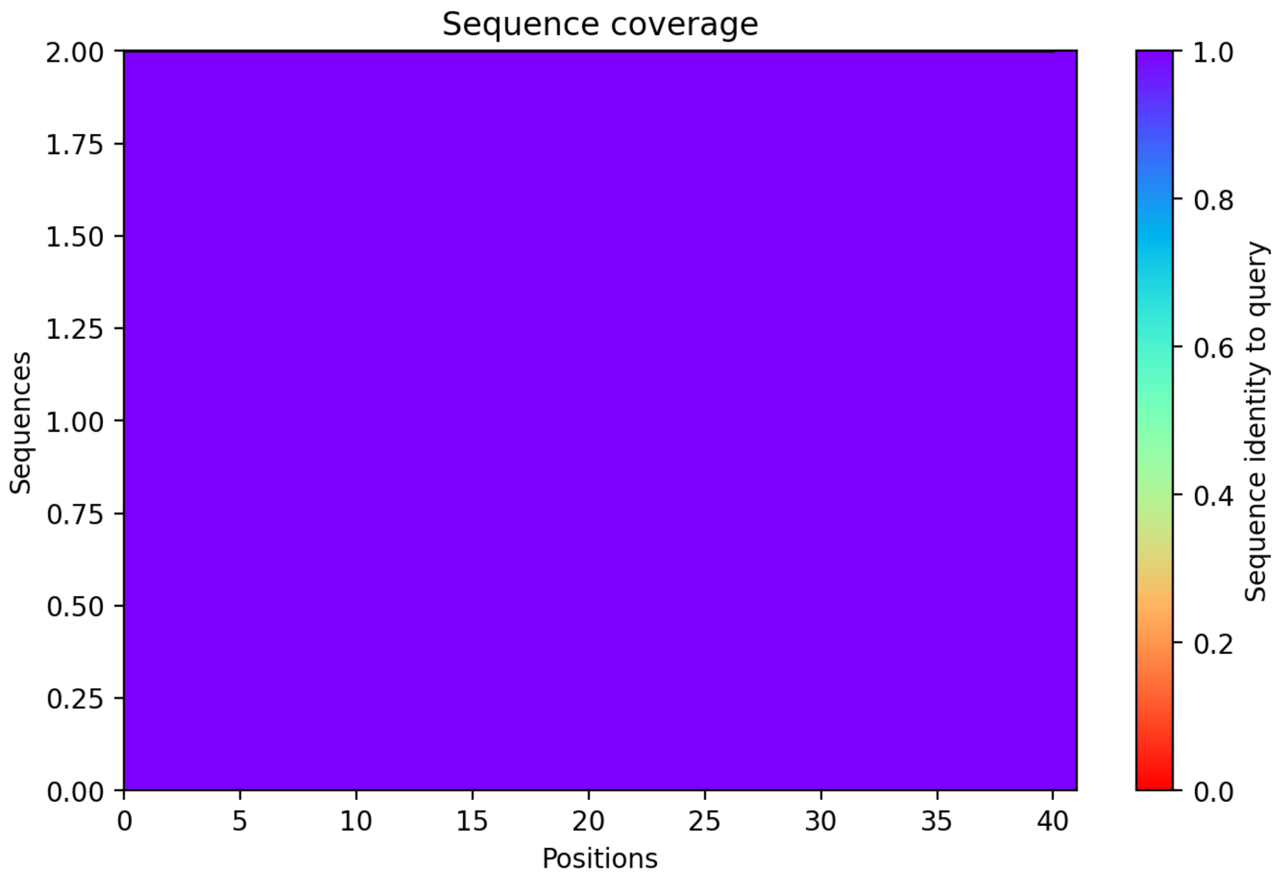

CP54: MtrunA17_Chr3g0105981

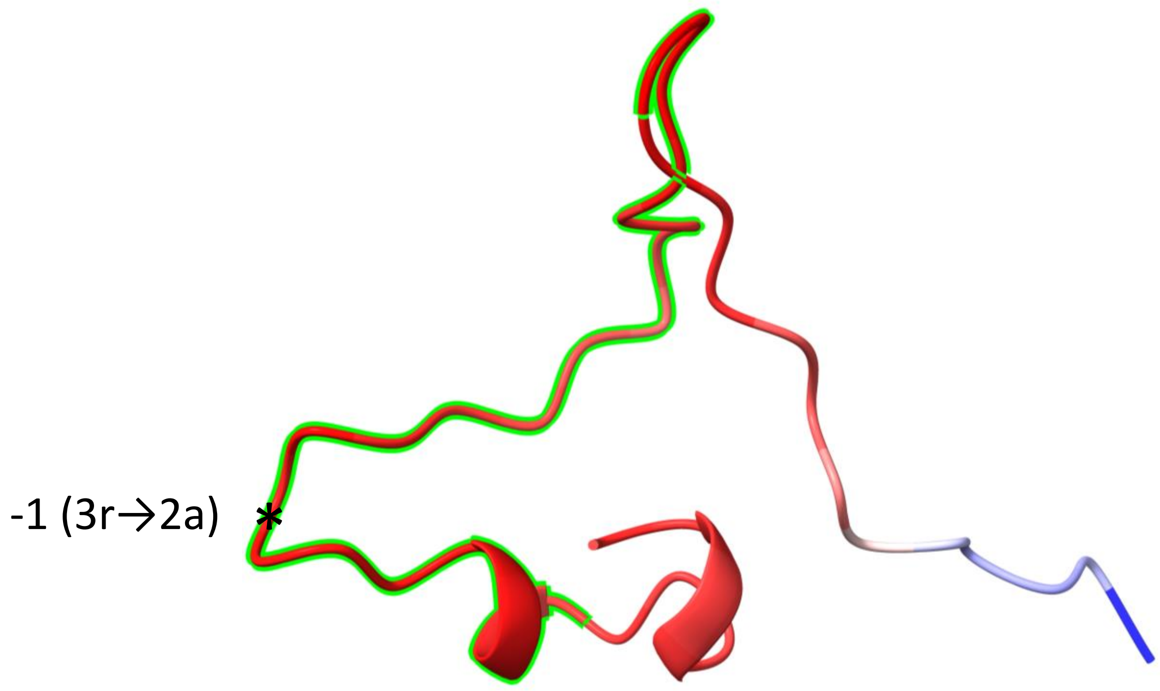

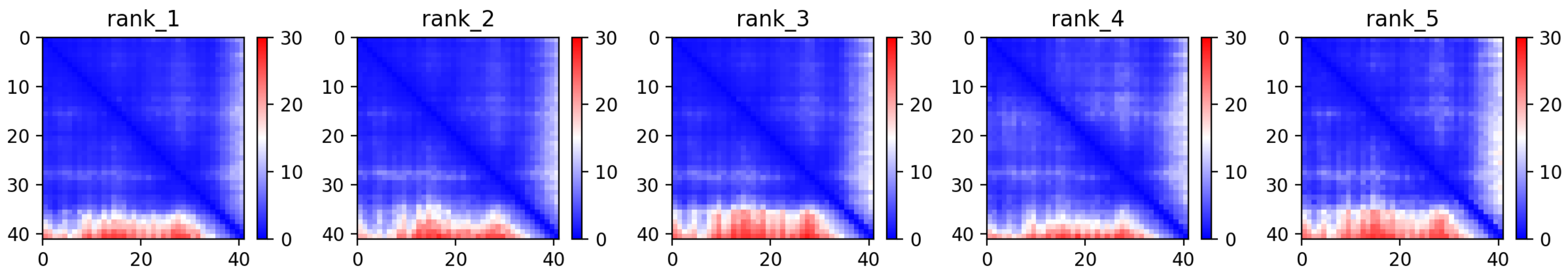

CP54: MtrunA17_Chr3g0105981

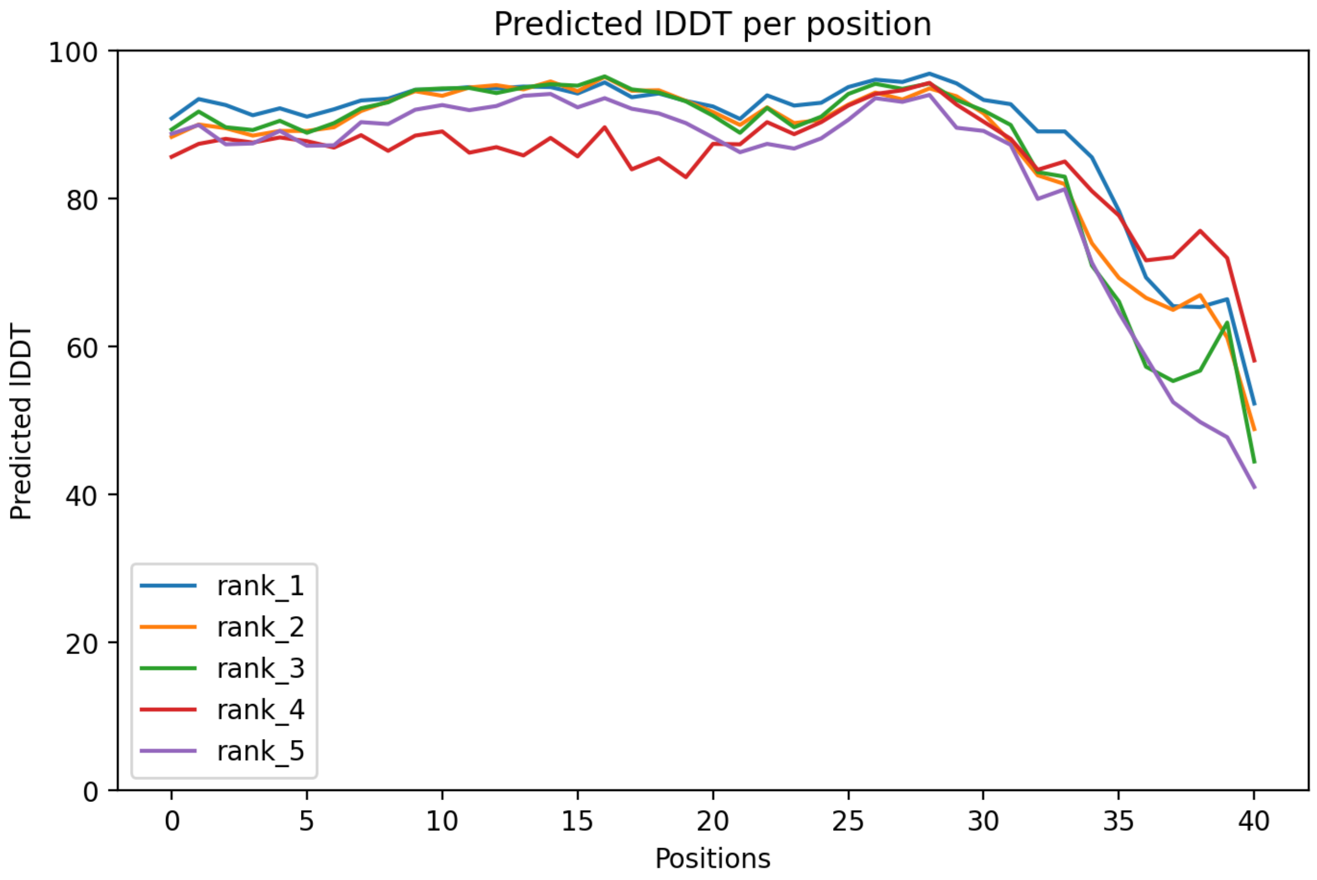

CP54: MtrunA17_Chr3g0105981

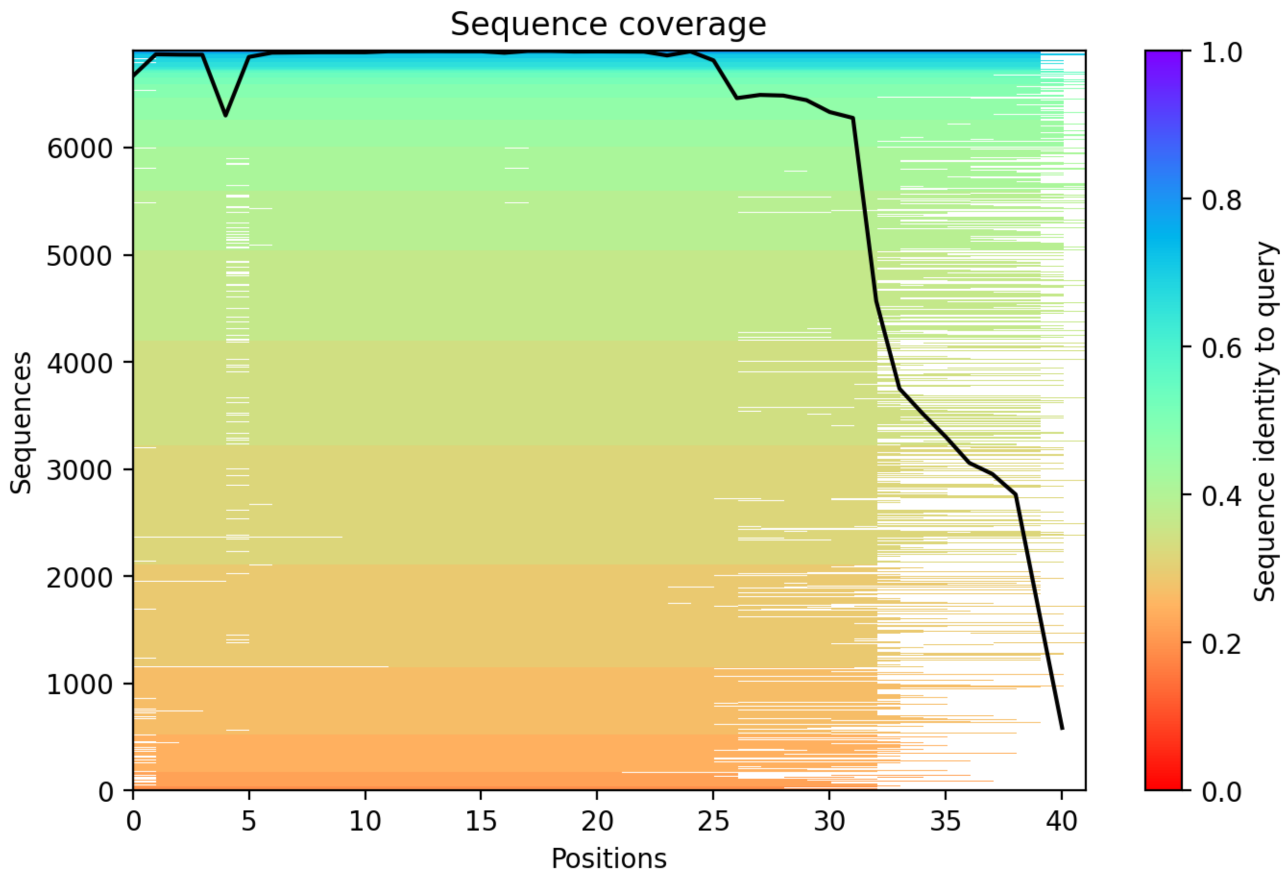

CP55: MtrunA17_Chr3g0110451

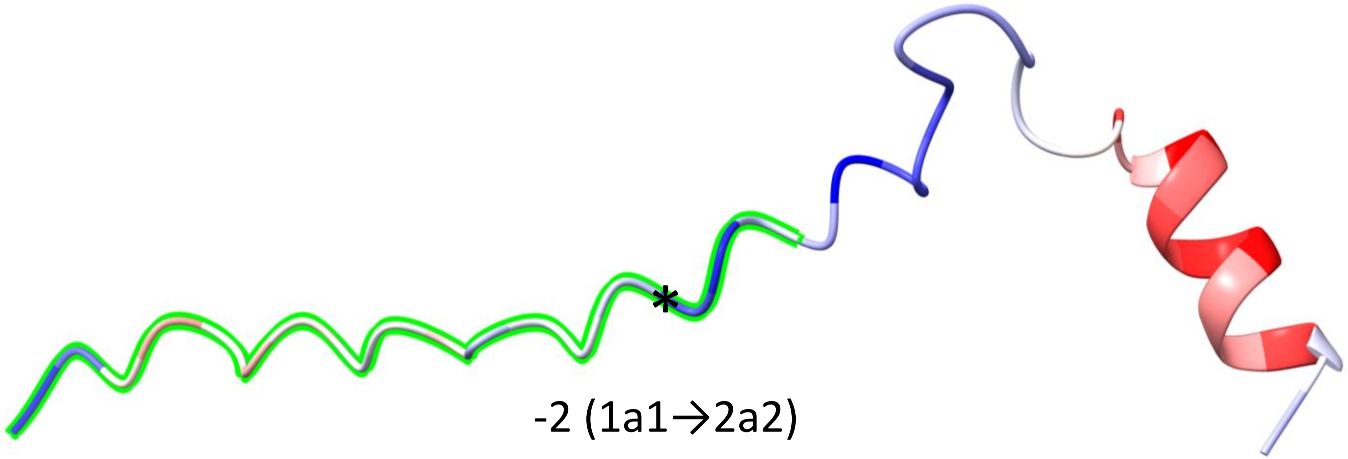

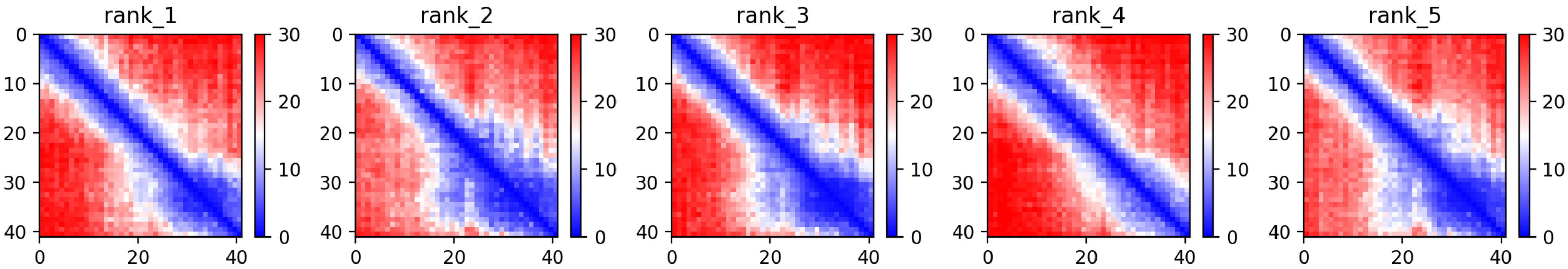

CP55: MtrunA17_Chr3g0110451

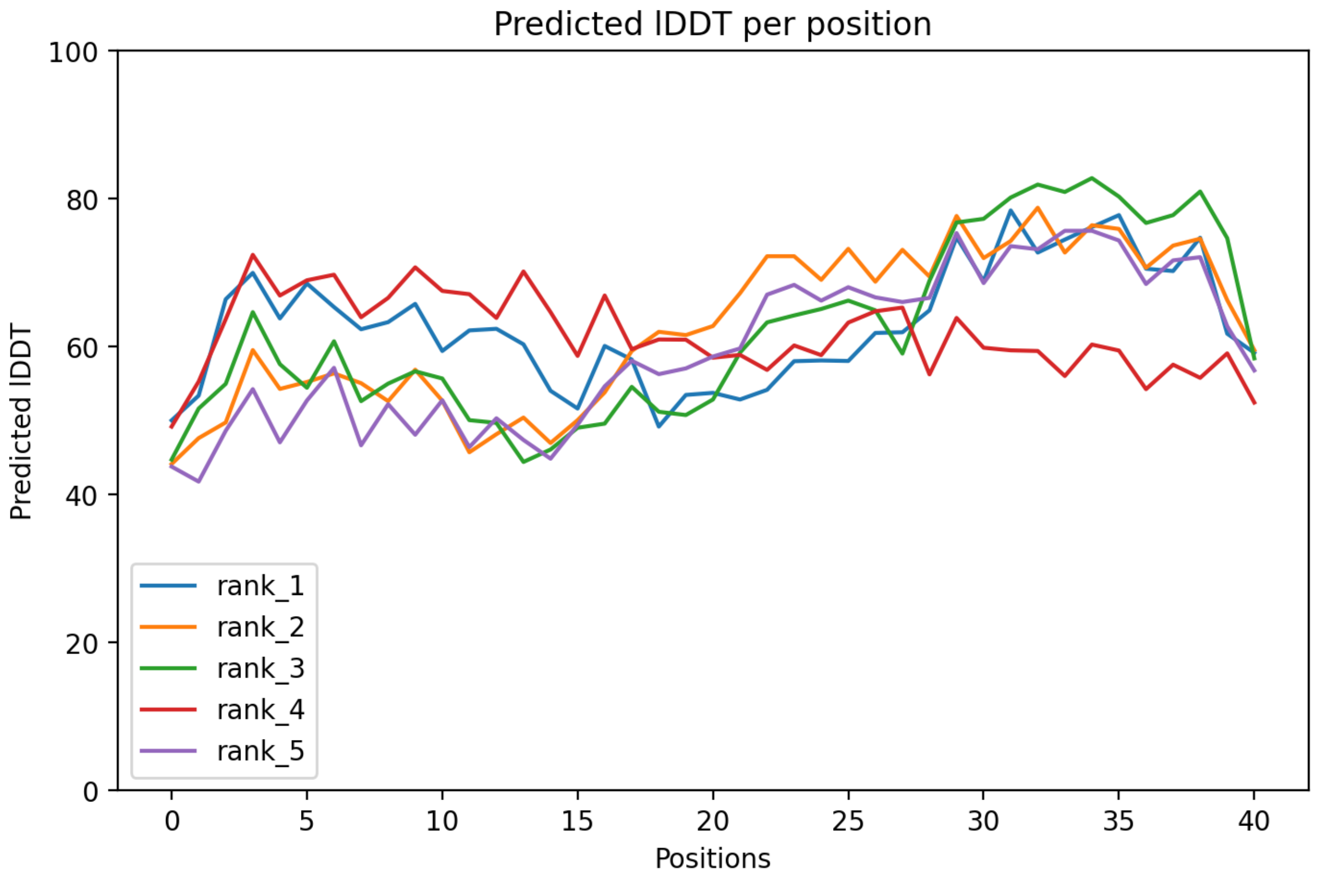

CP55: MtrunA17_Chr3g0110451

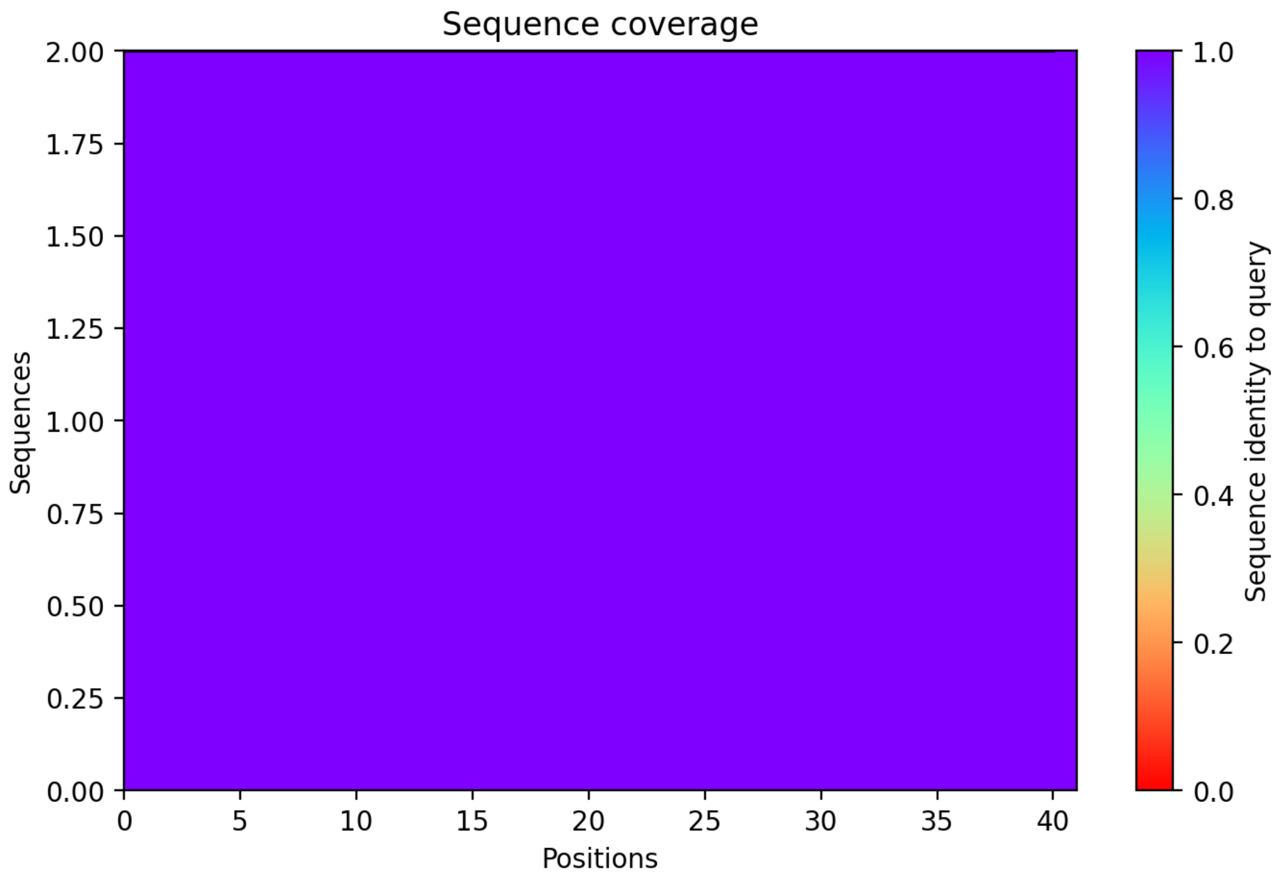

CP56: MtrunA17_Chr3g0113591

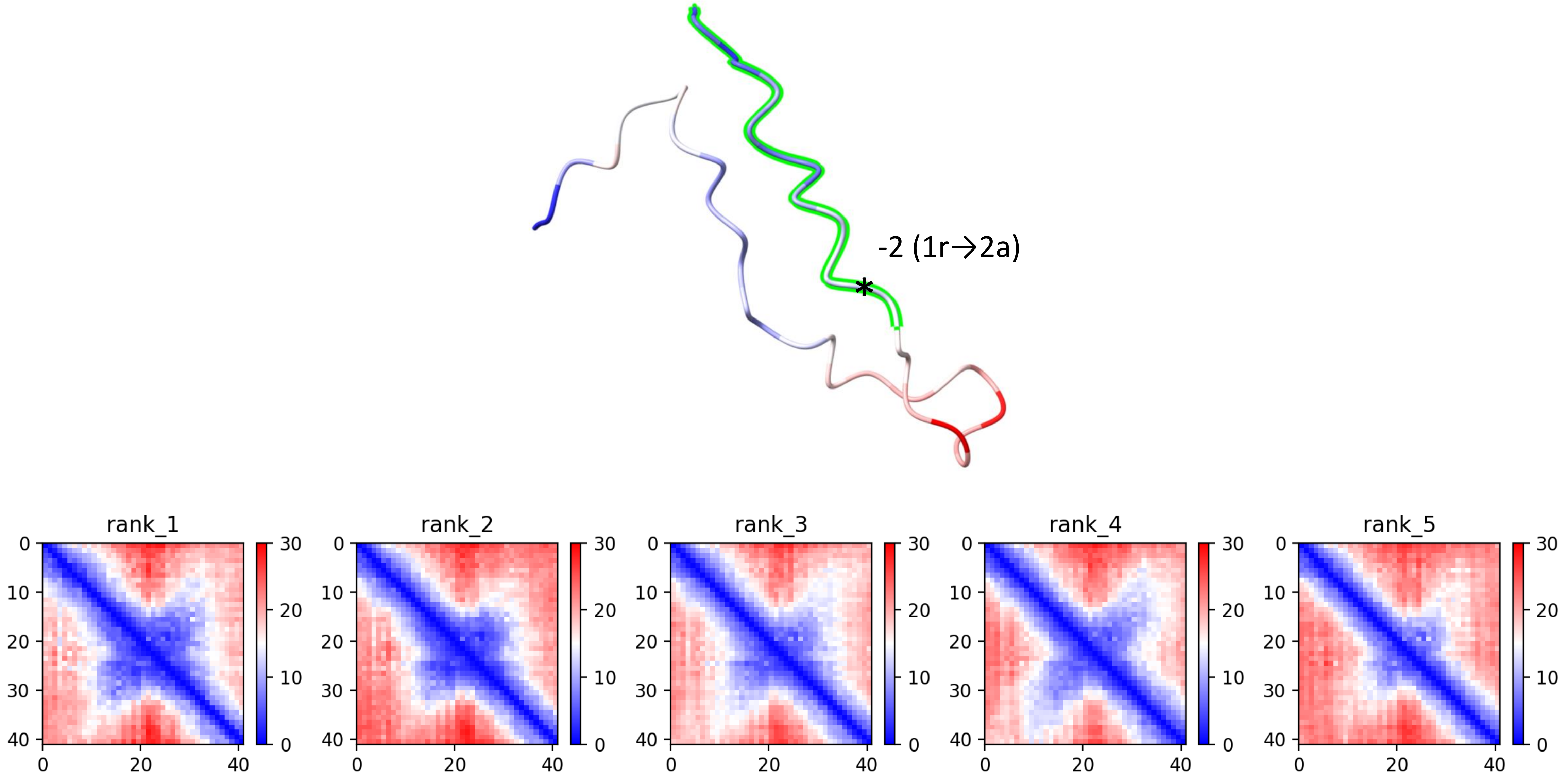

CP56: MtrunA17_Chr3g0113591

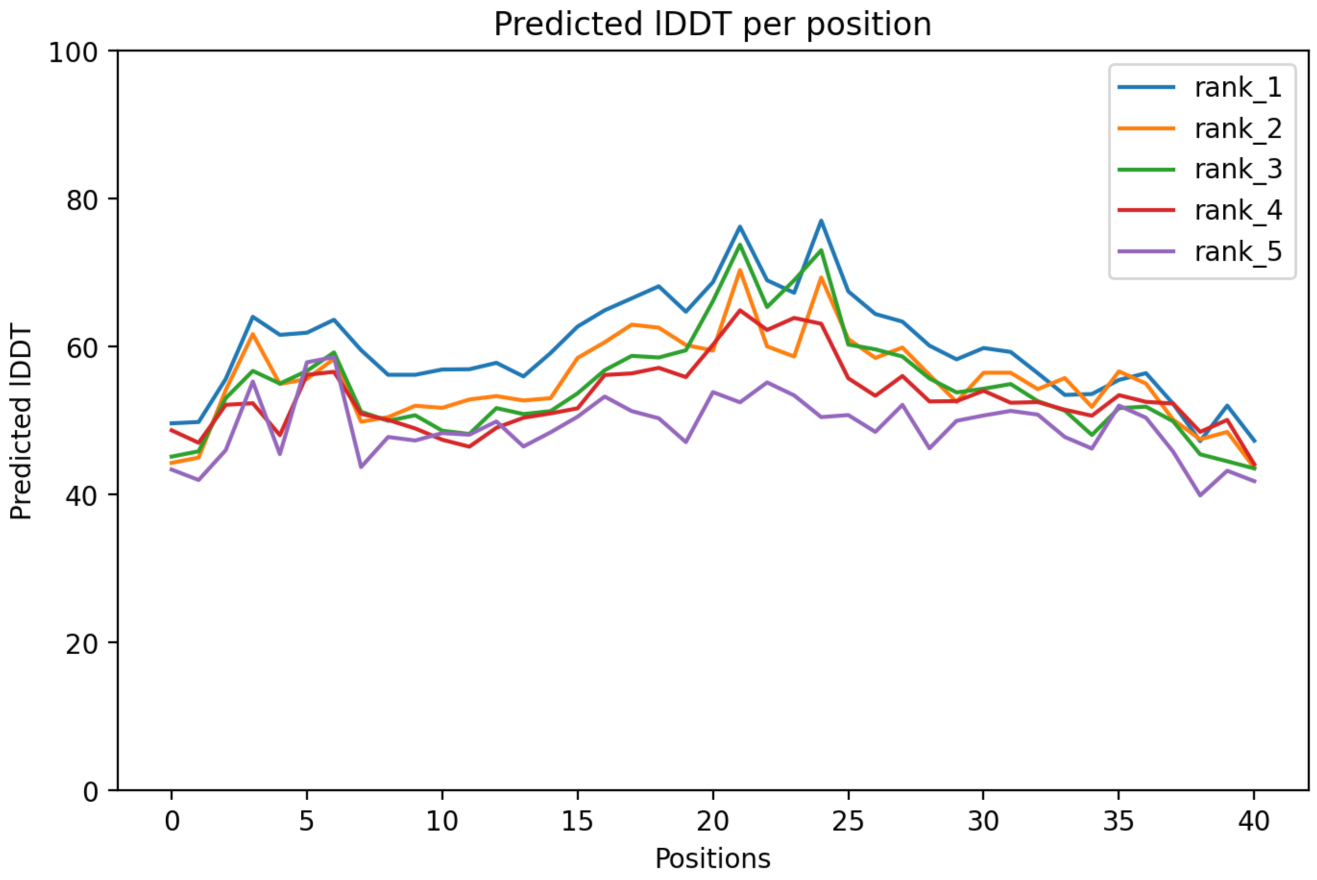

CP56: MtrunA17_Chr3g0113591

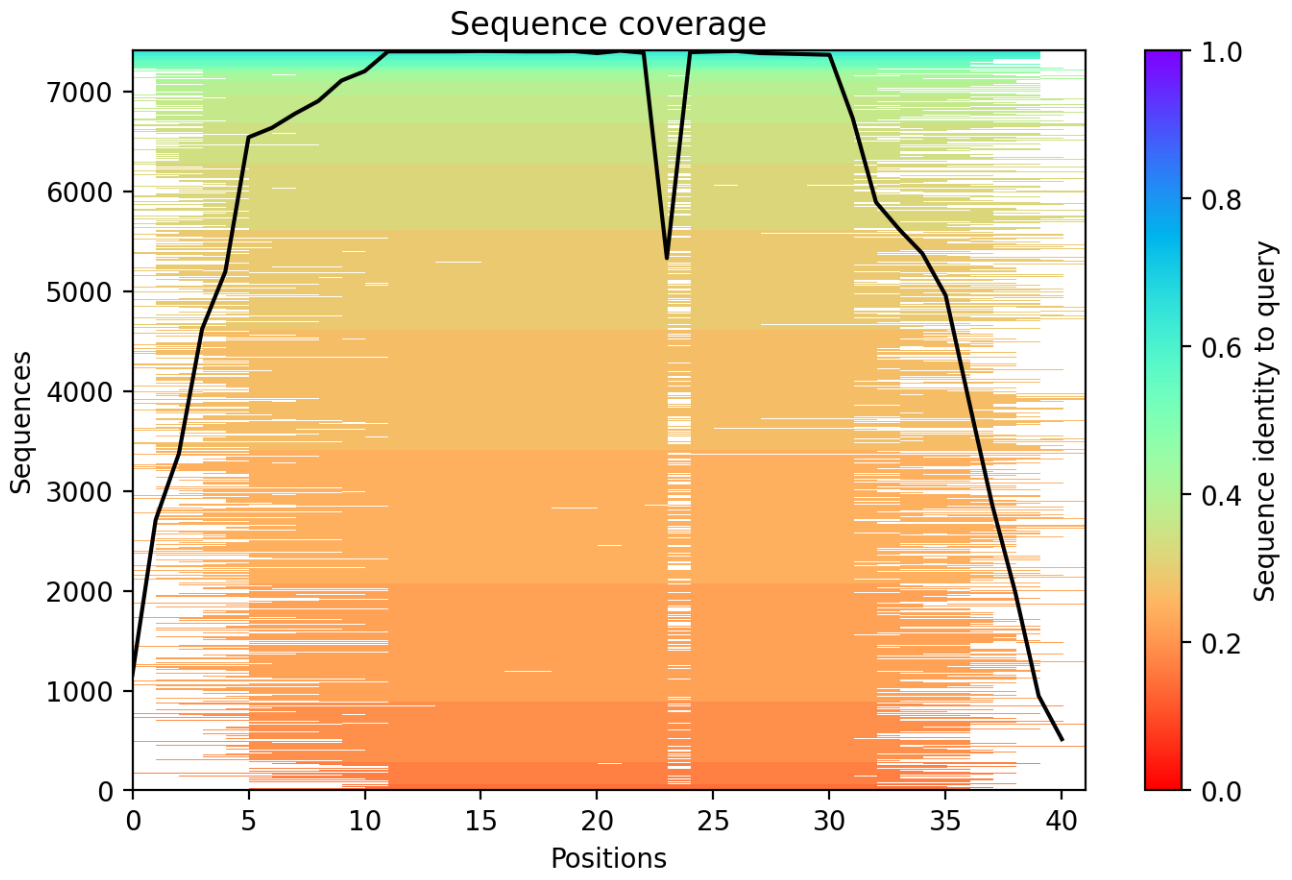

CP57: MtrunA17_Chr3g0124631

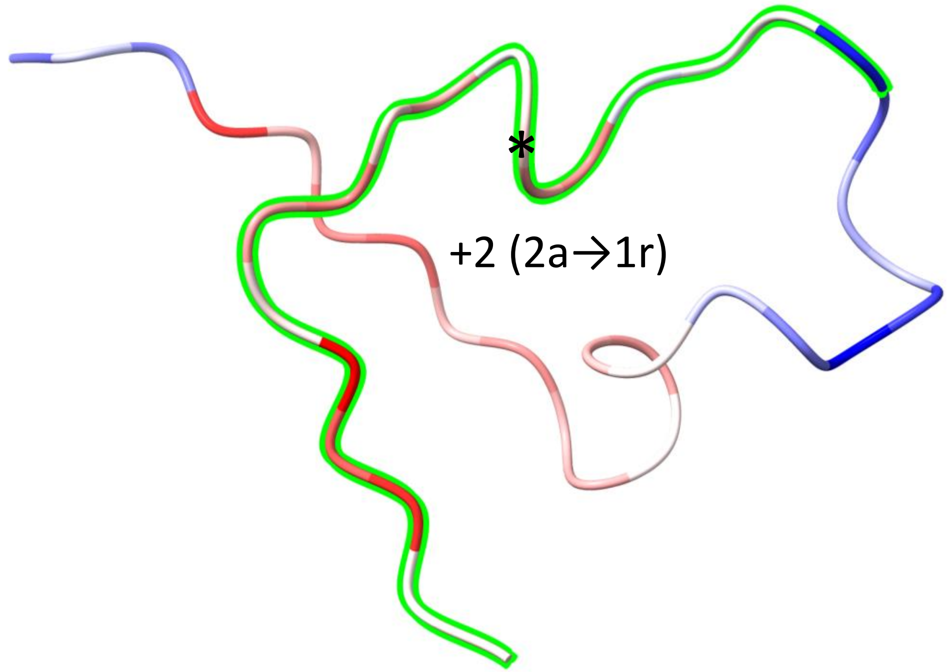

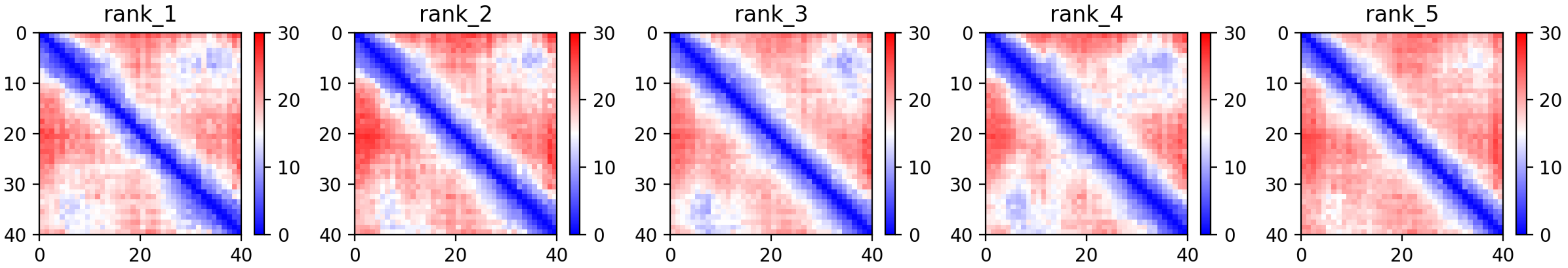

CP57: MtrunA17_Chr3g0124631

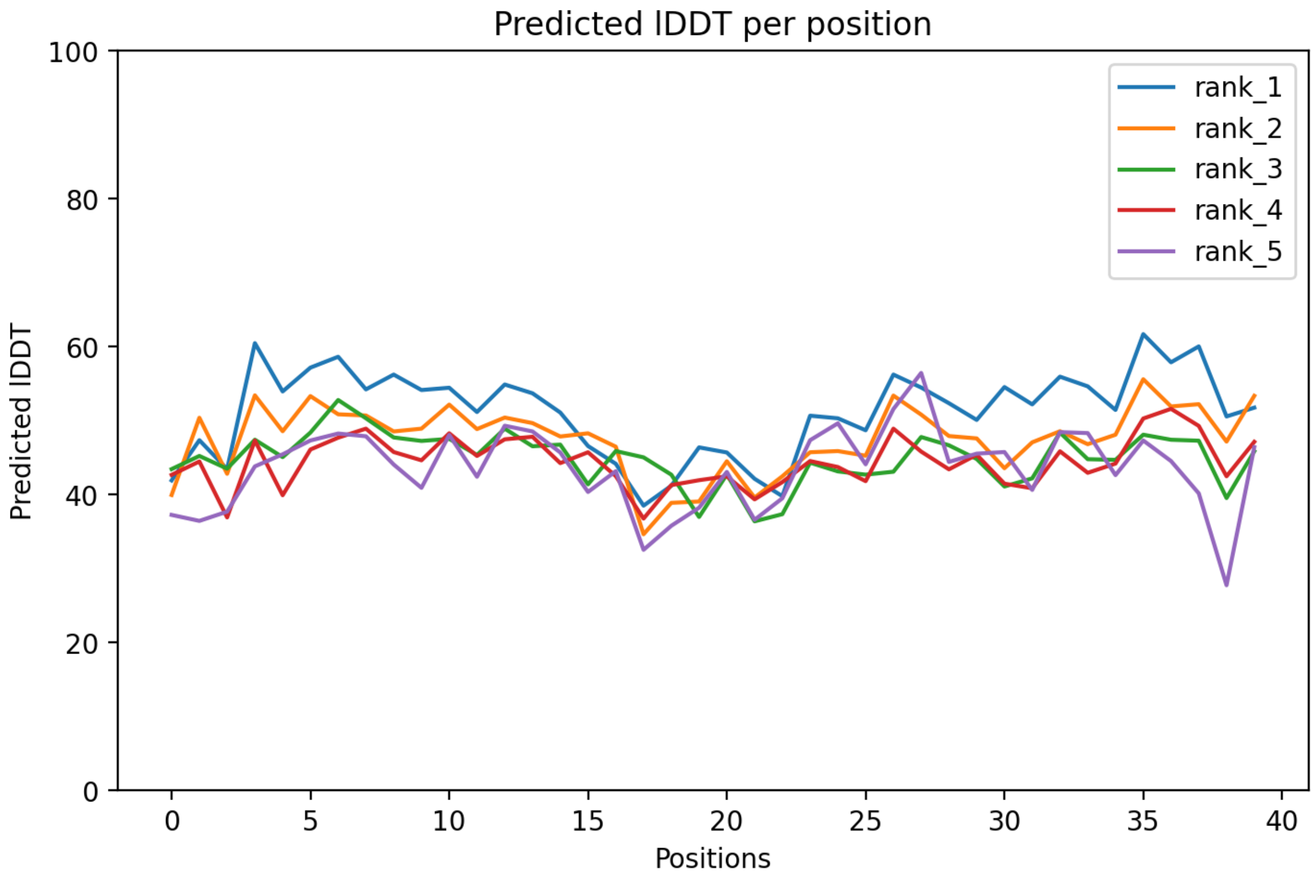

CP57: MtrunA17_Chr3g0124631

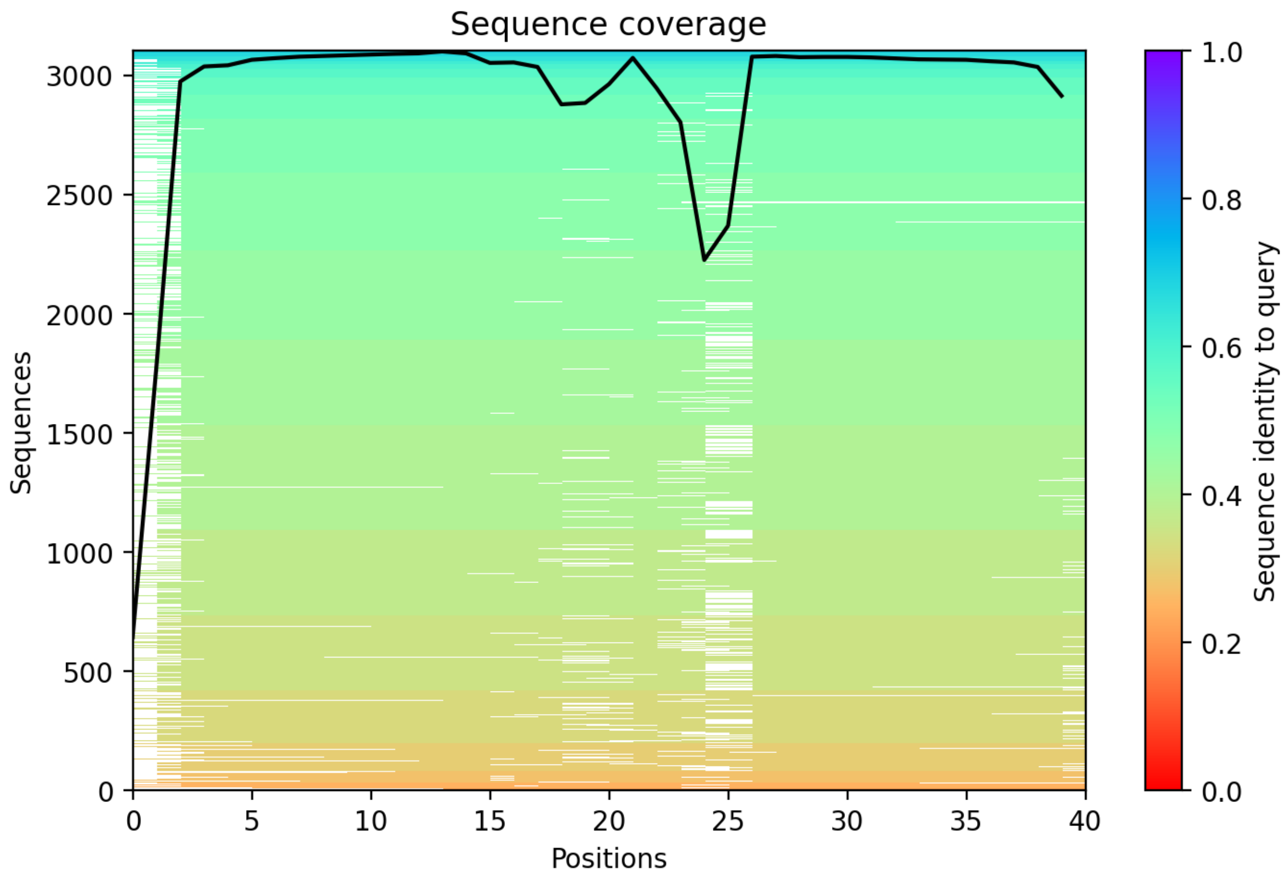

CP58: MtrunA17_Chr3g0127321

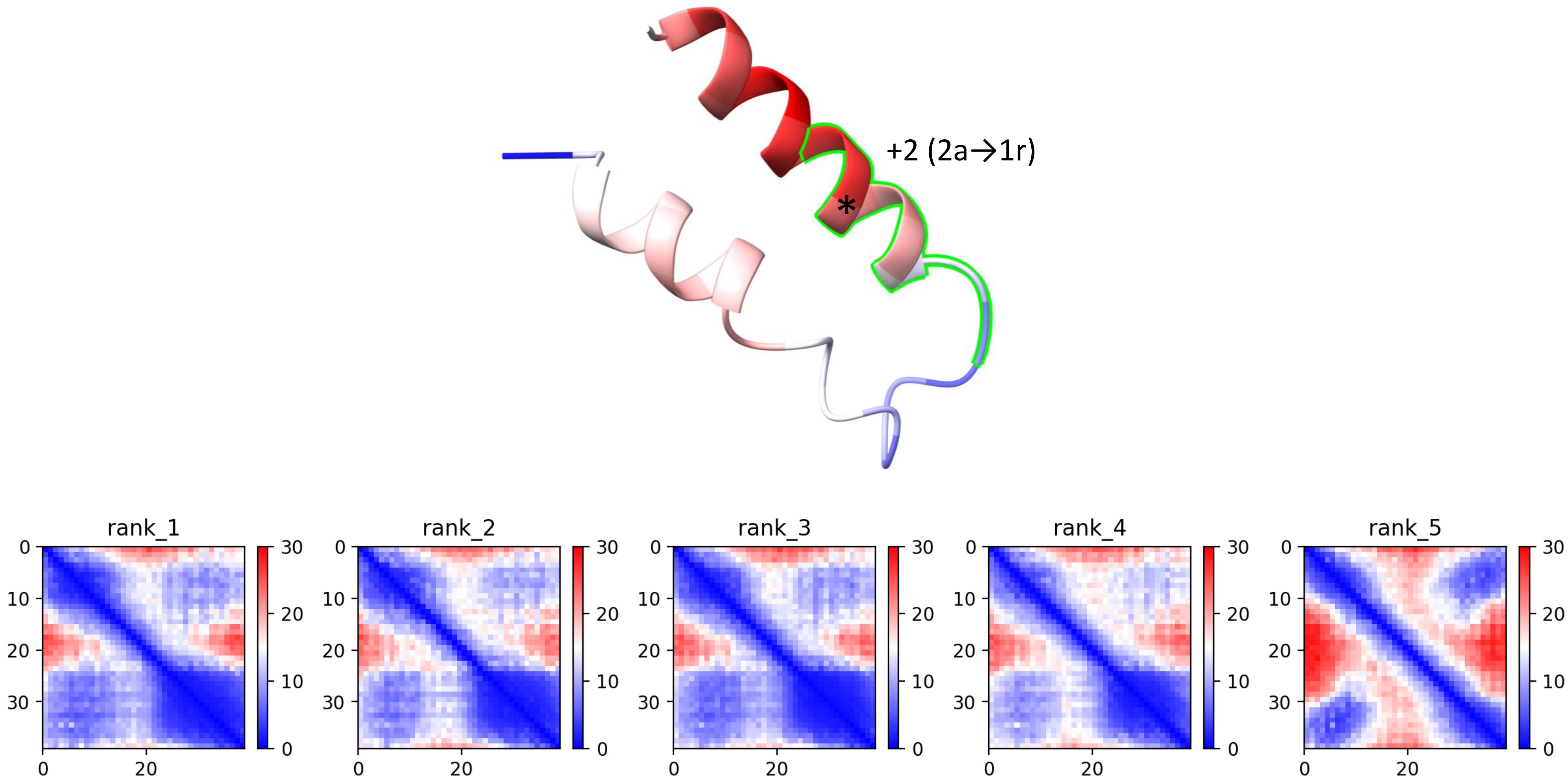

CP58: MtrunA17_Chr3g0127321

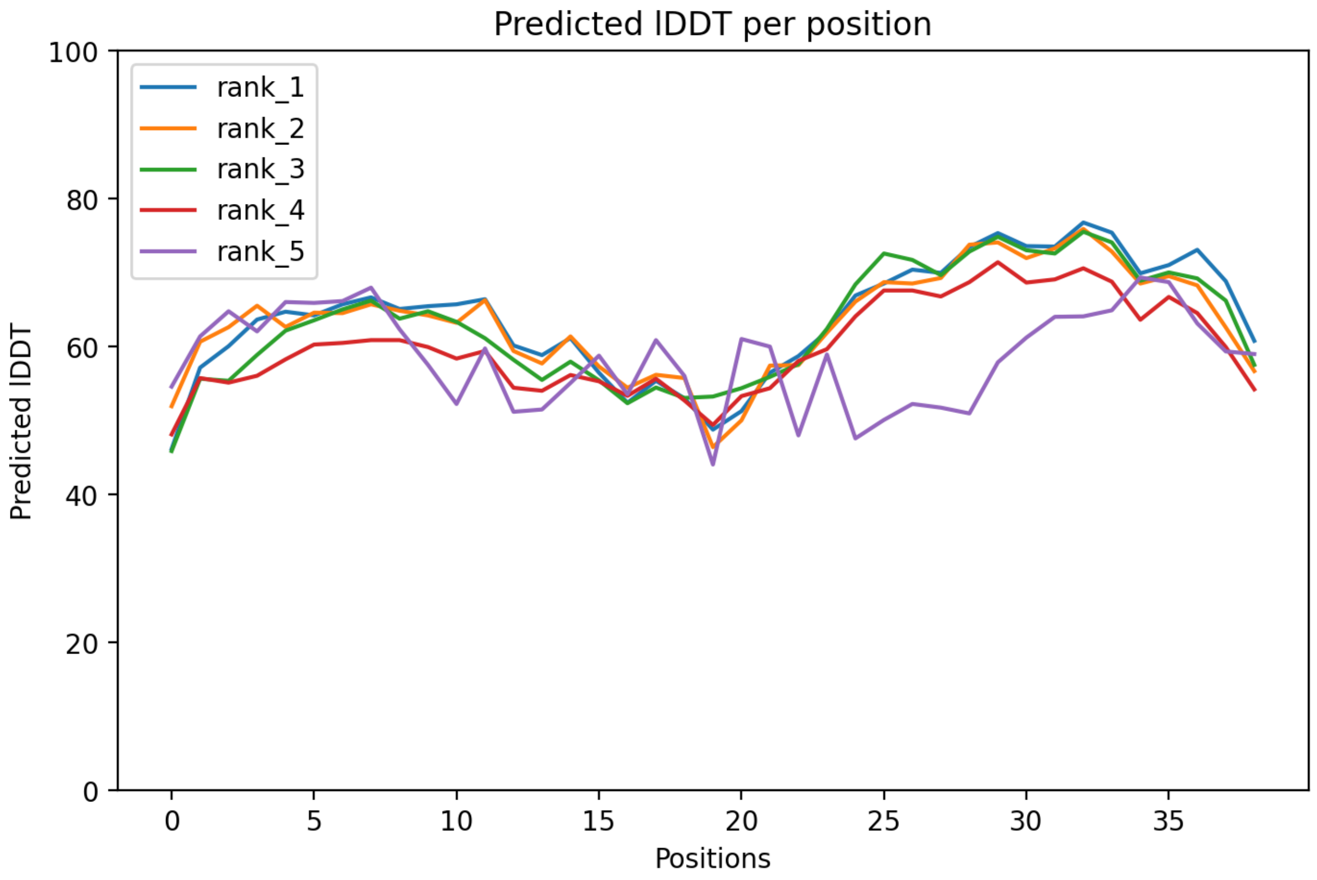

CP58: MtrunA17_Chr3g0127321

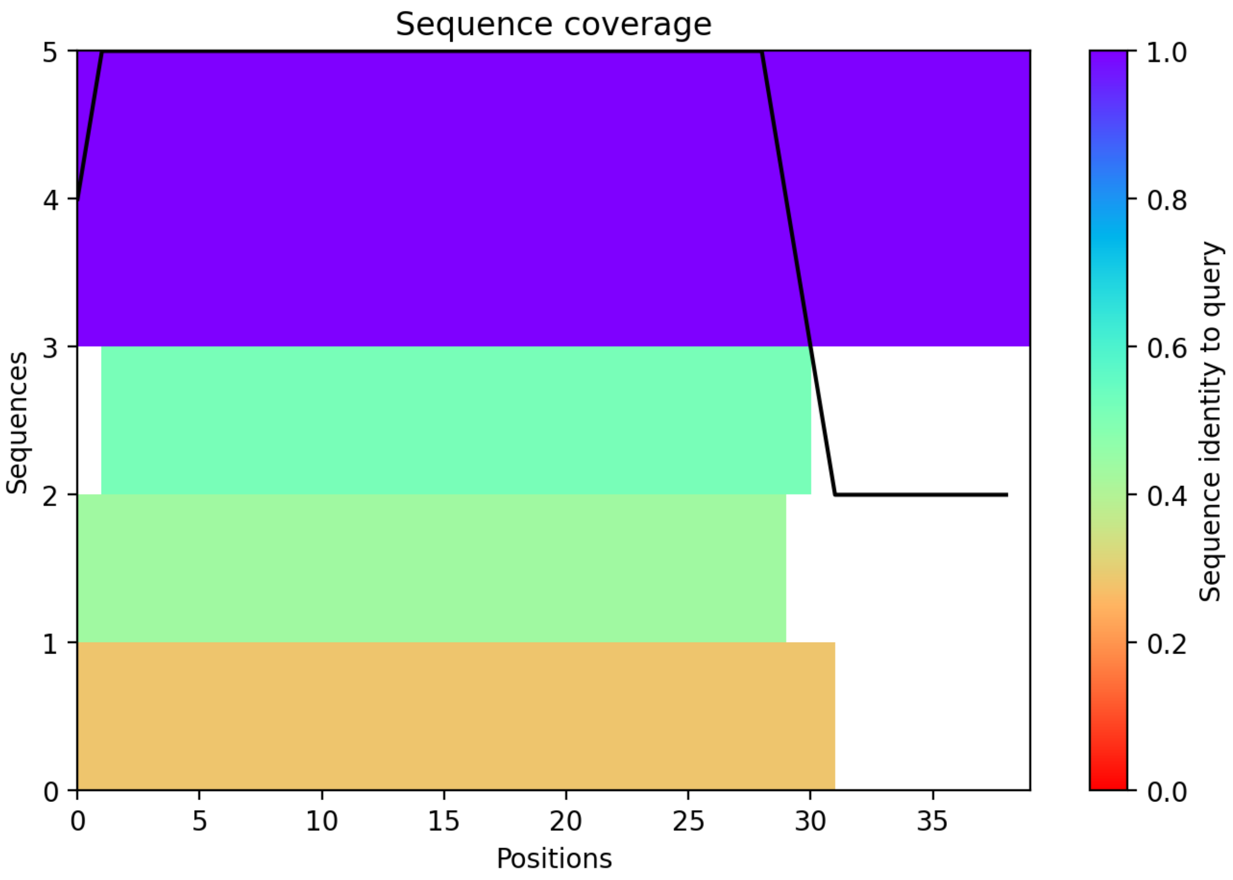

CP59: MtrunA17_Chr3g0130671

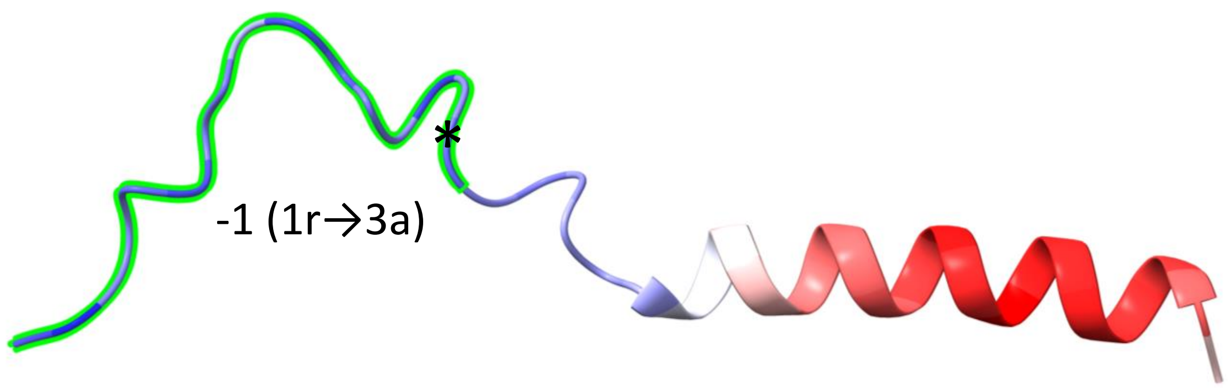

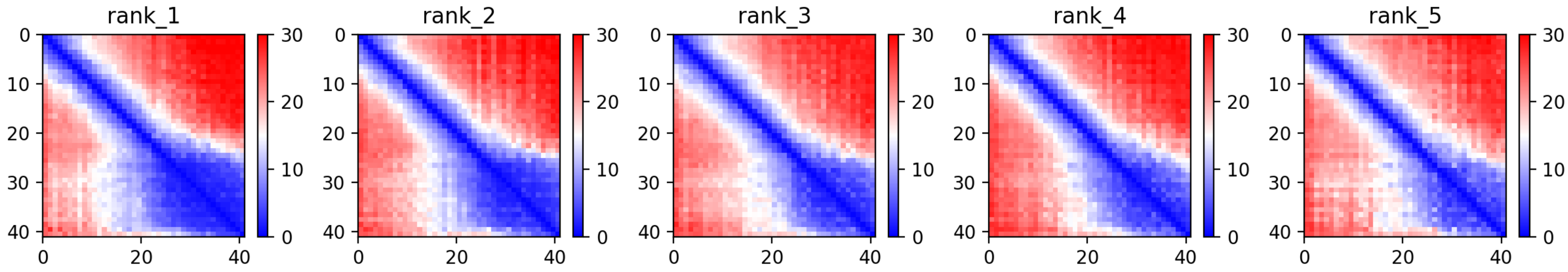

CP59: MtrunA17_Chr3g0130671

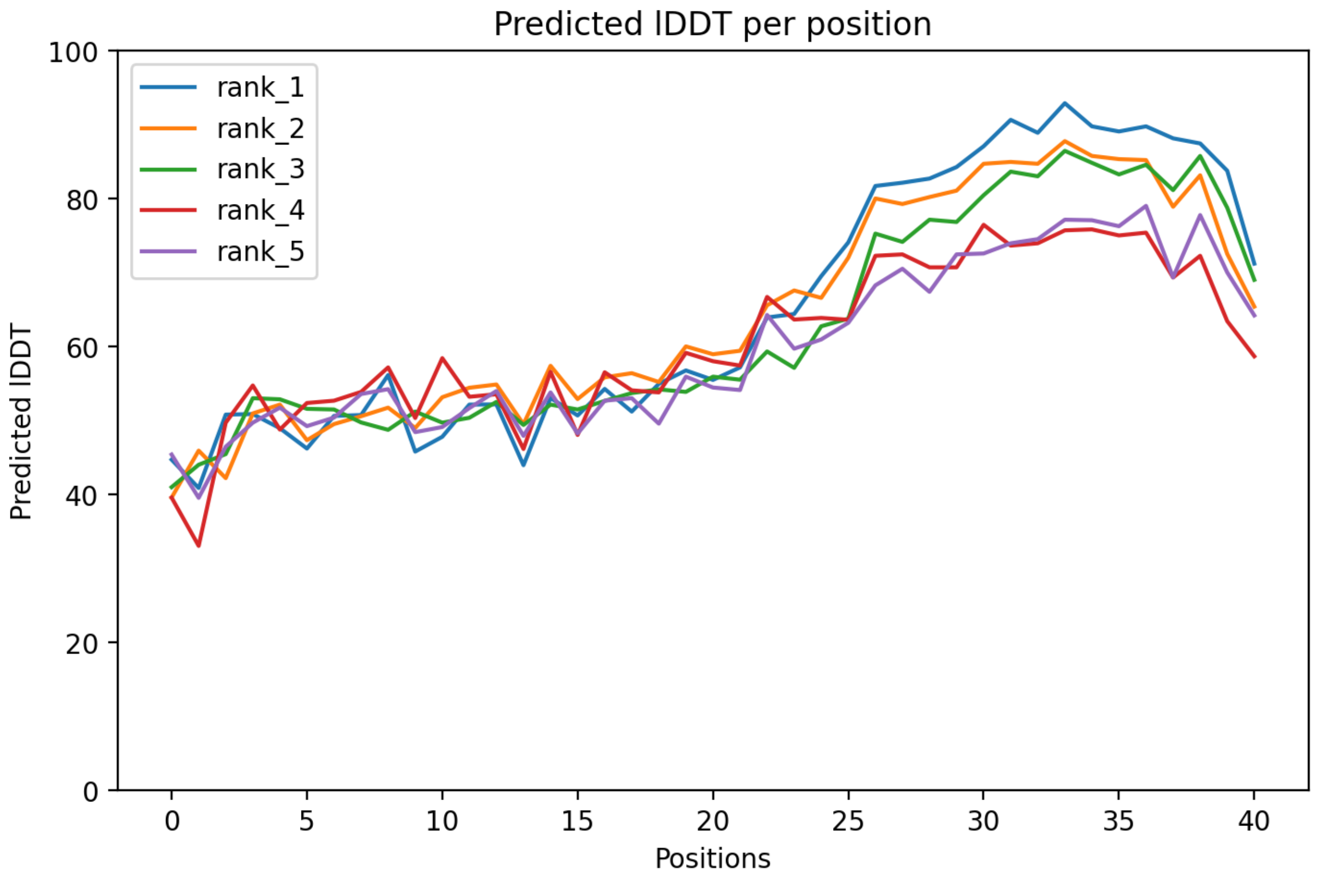

CP59: MtrunA17_Chr3g0130671

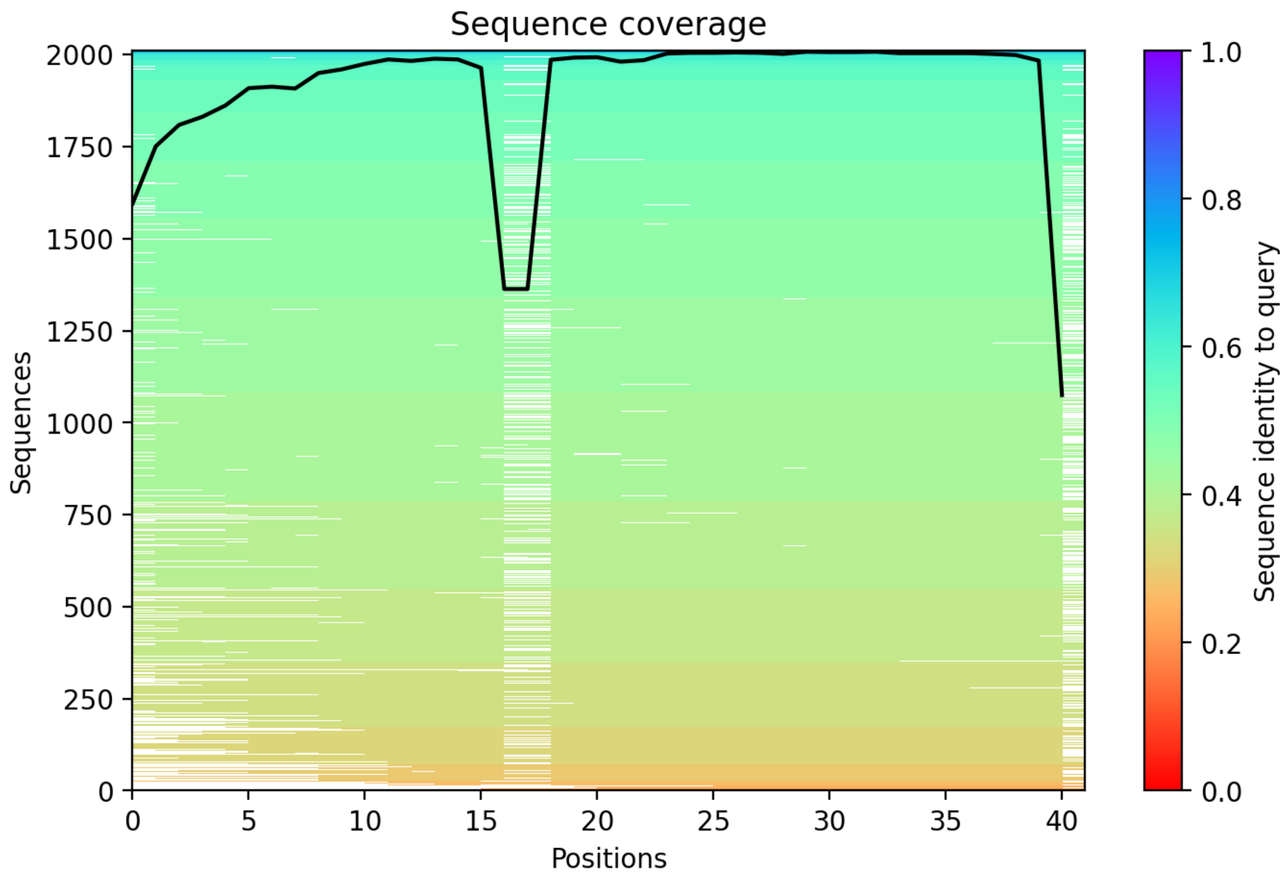

CP60: MtrunA17_Chr3g0135761

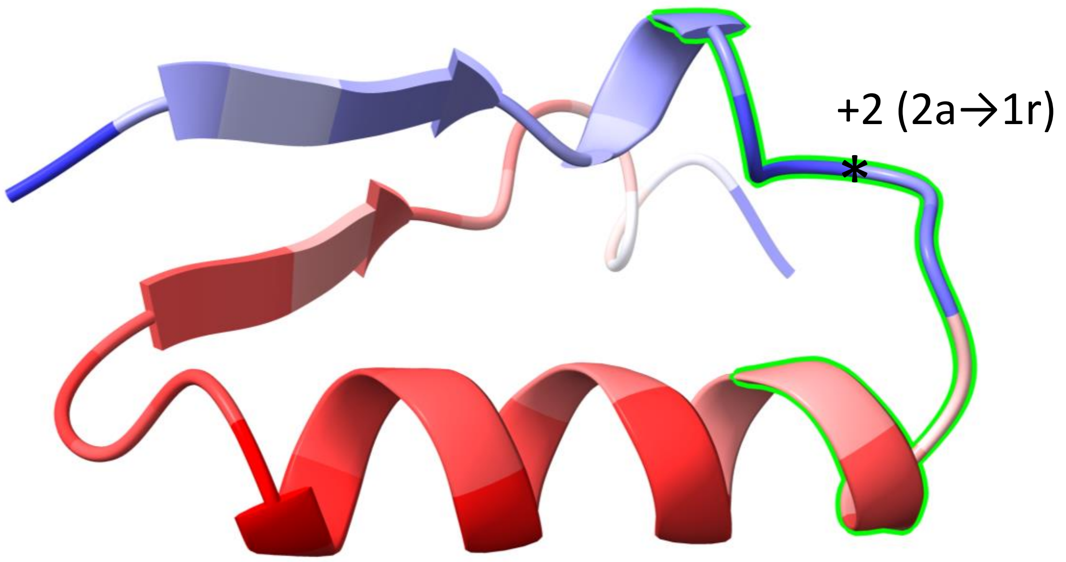

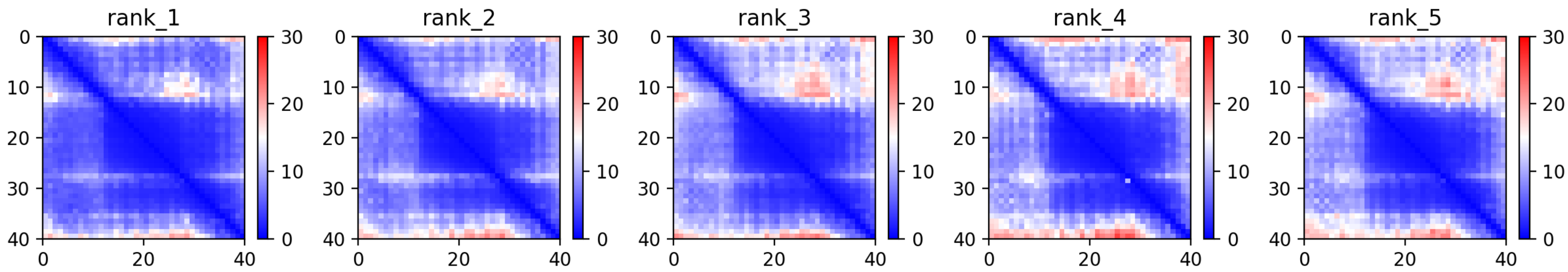

CP60: MtrunA17_Chr3g0135761

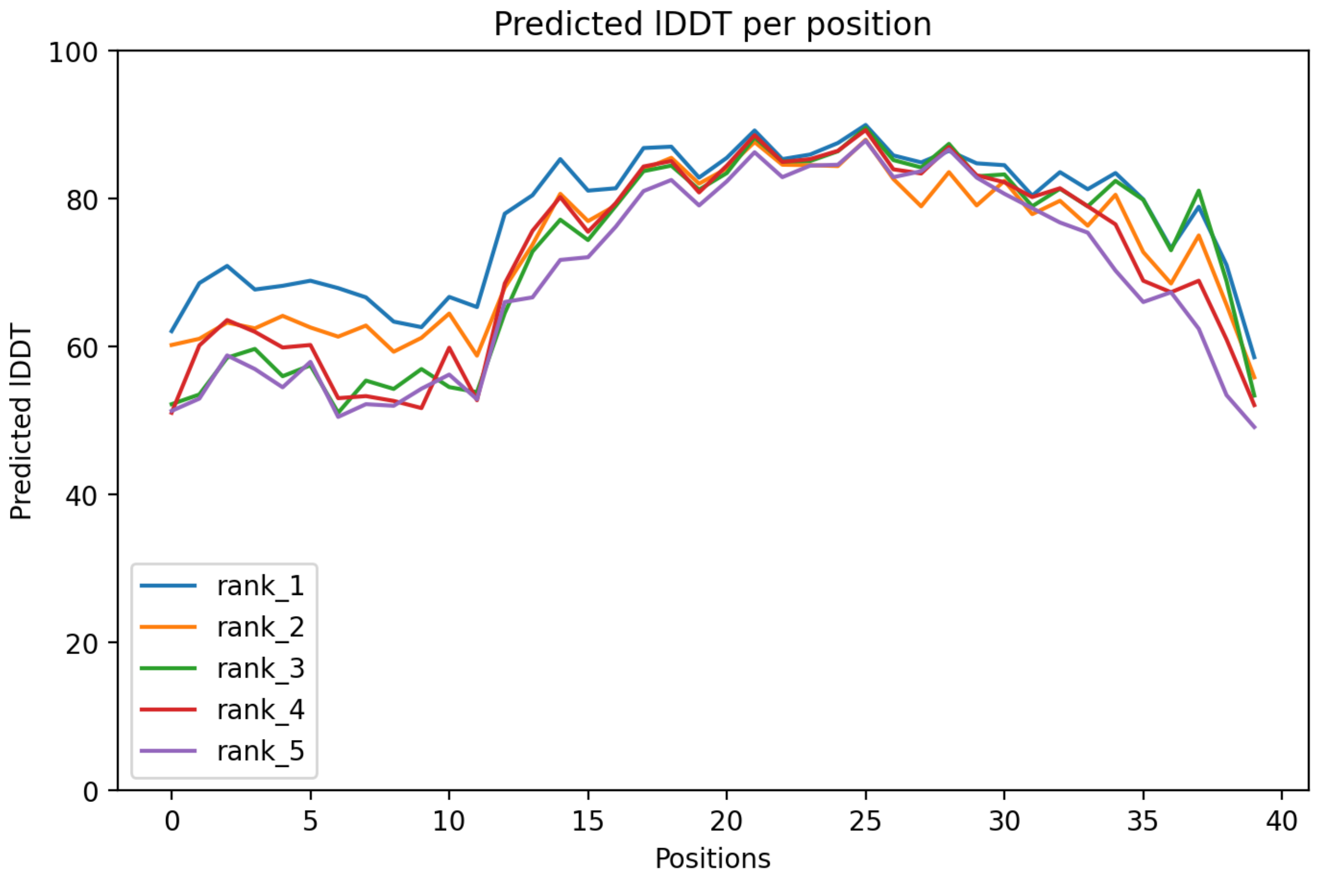

CP60: MtrunA17_Chr3g0135761

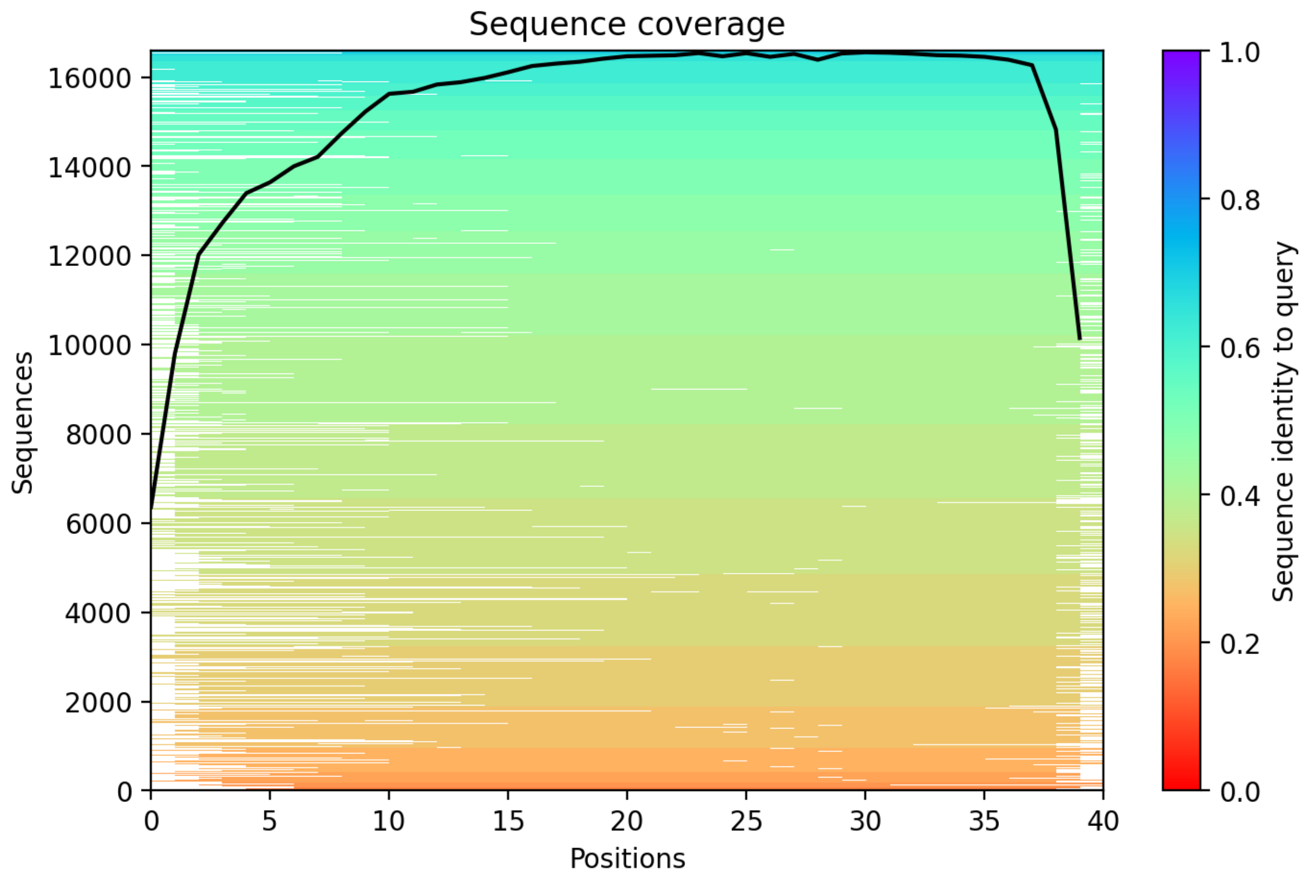

CP61: MtrunA17_Chr3g0137391

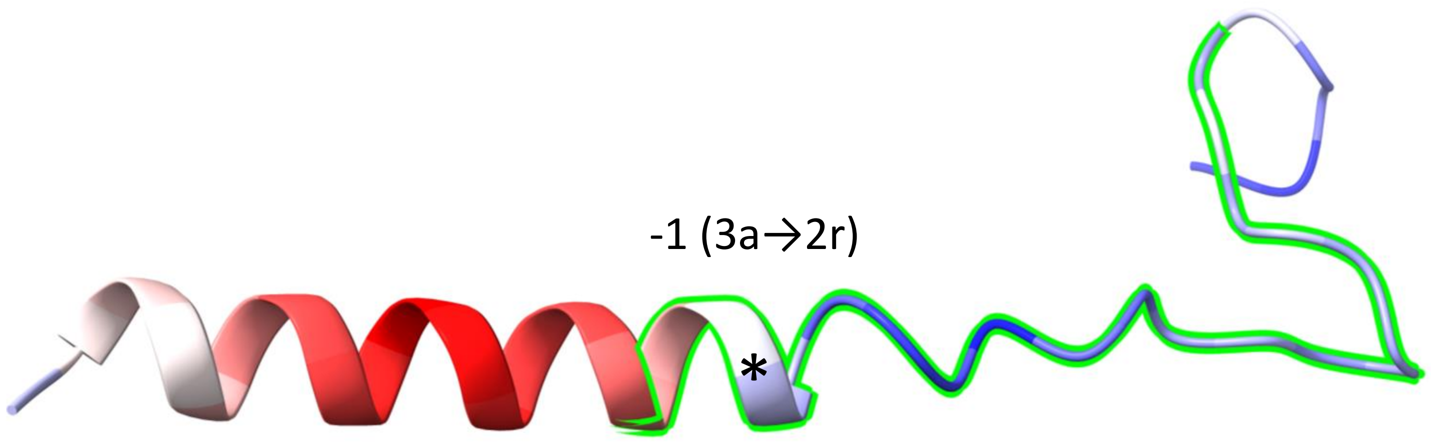

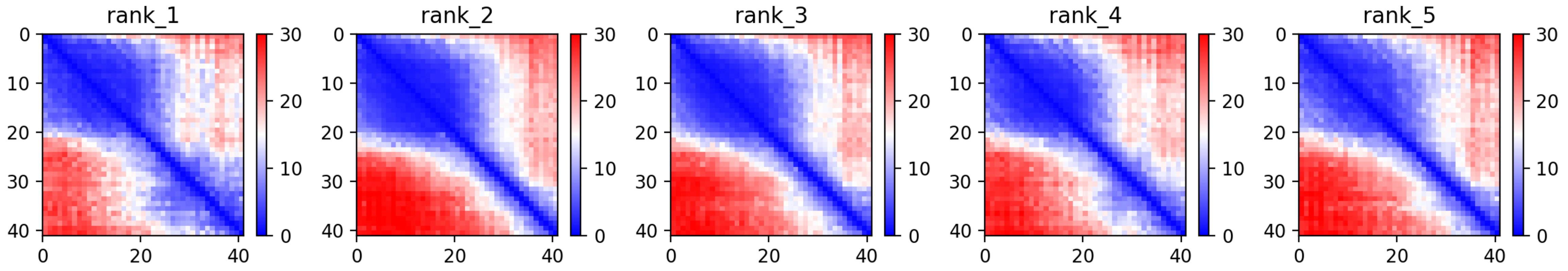

CP61: MtrunA17_Chr3g0137391

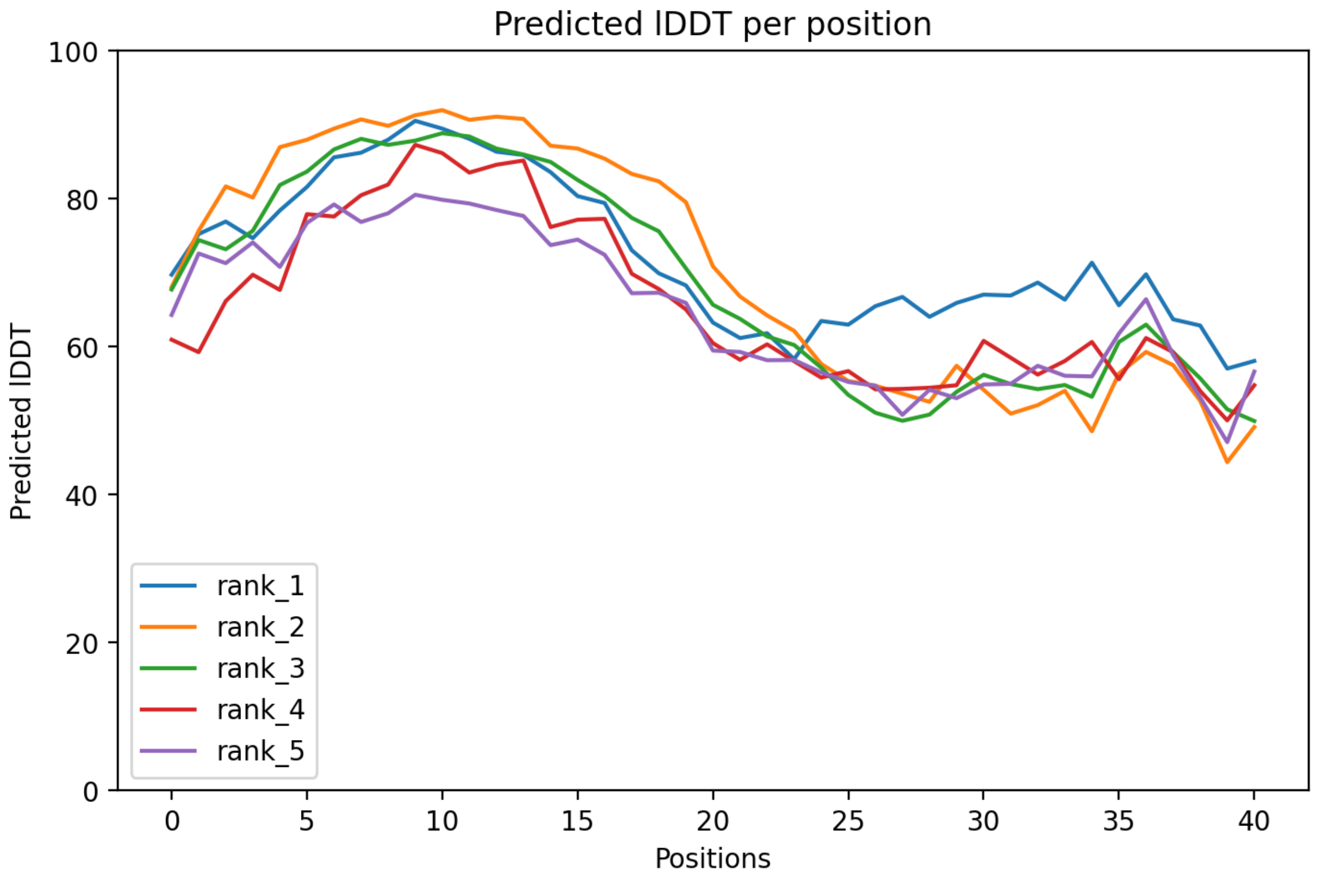

CP61: MtrunA17_Chr3g0137391

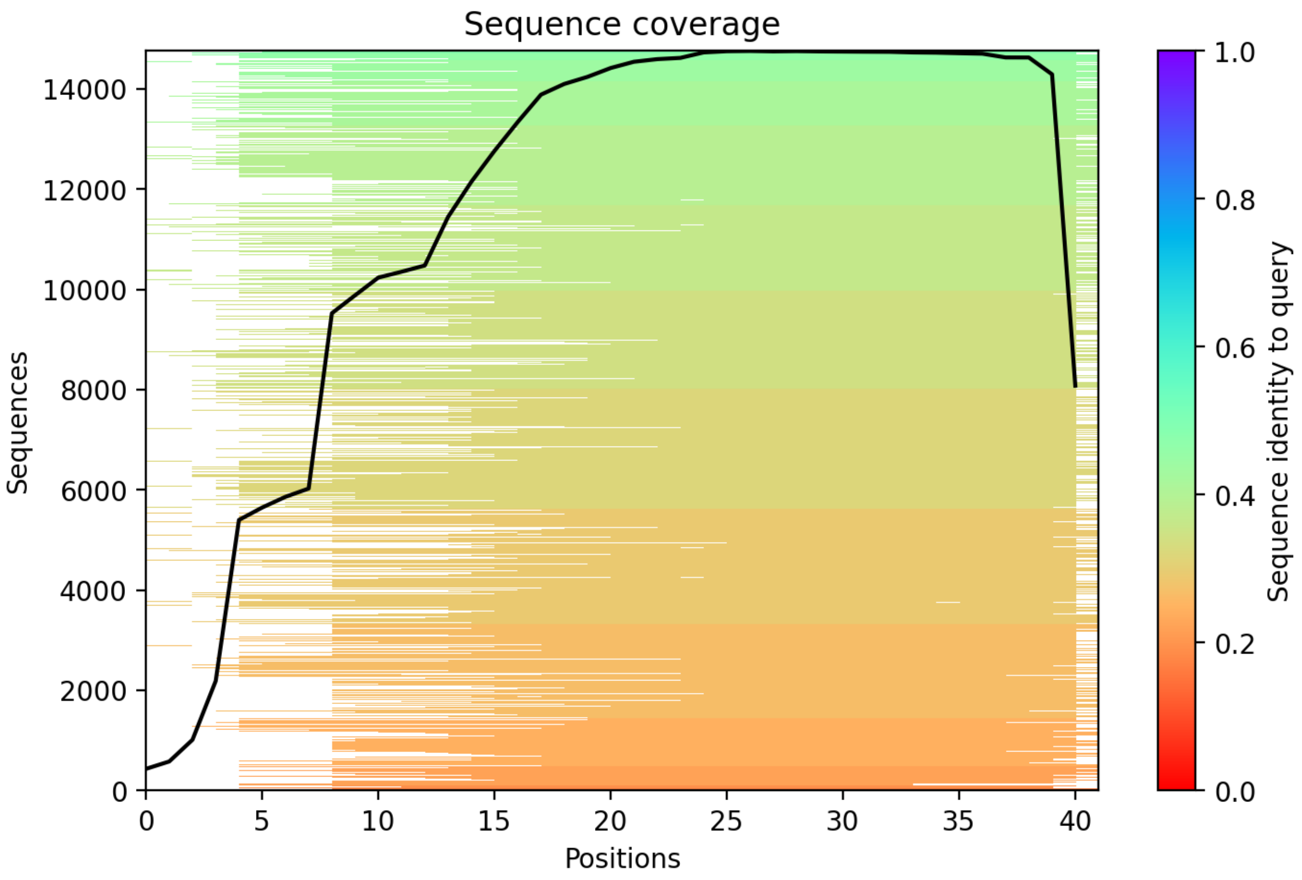

CP62: MtrunA17_Chr3g0141901

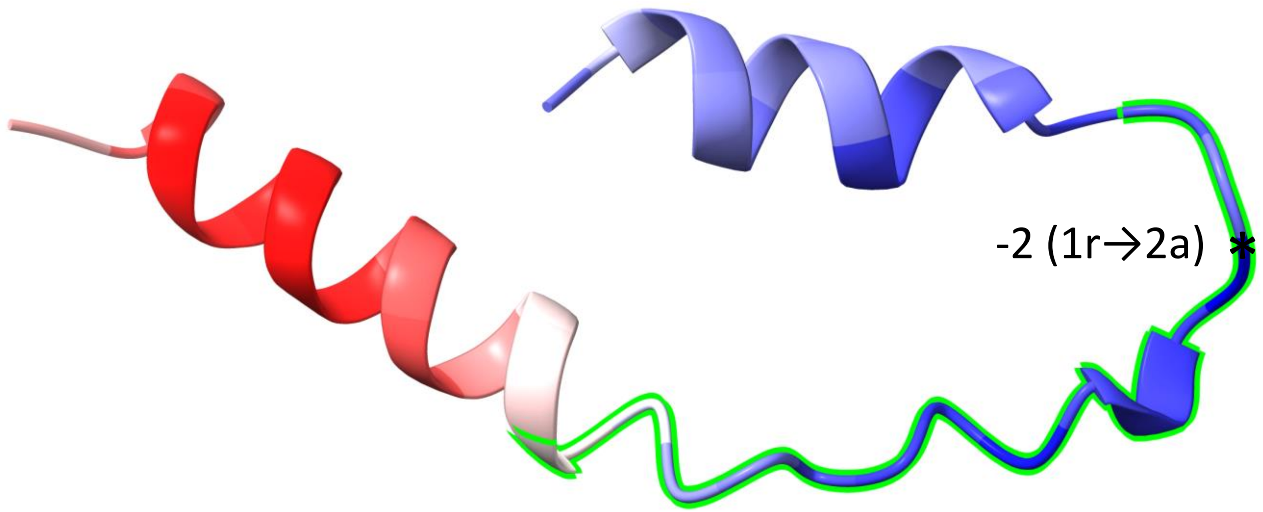

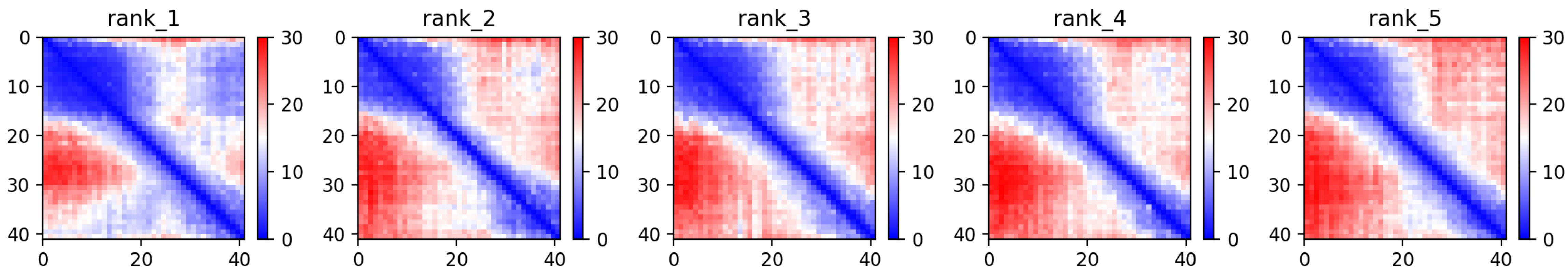

CP62: MtrunA17_Chr3g0141901

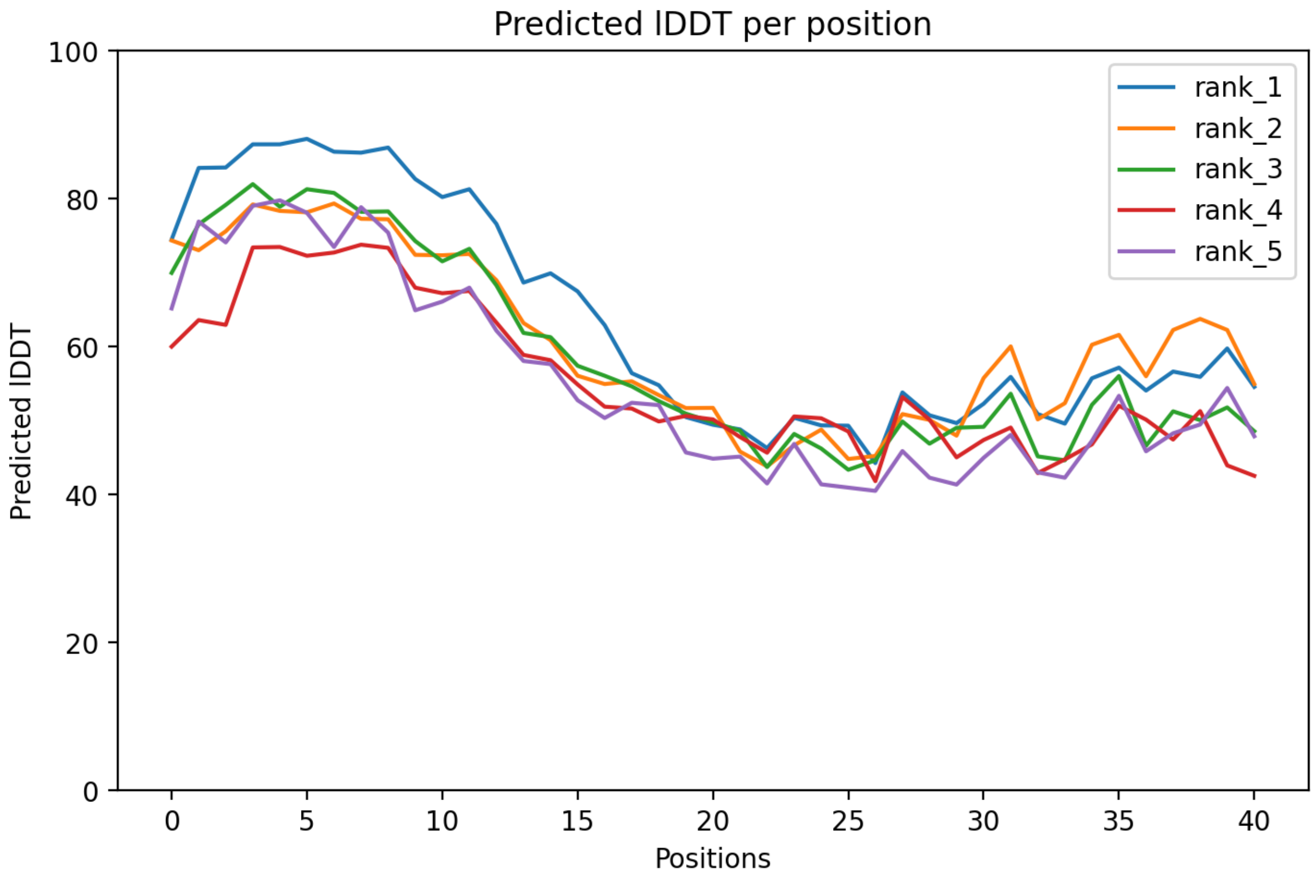

CP62: MtrunA17_Chr3g0141901

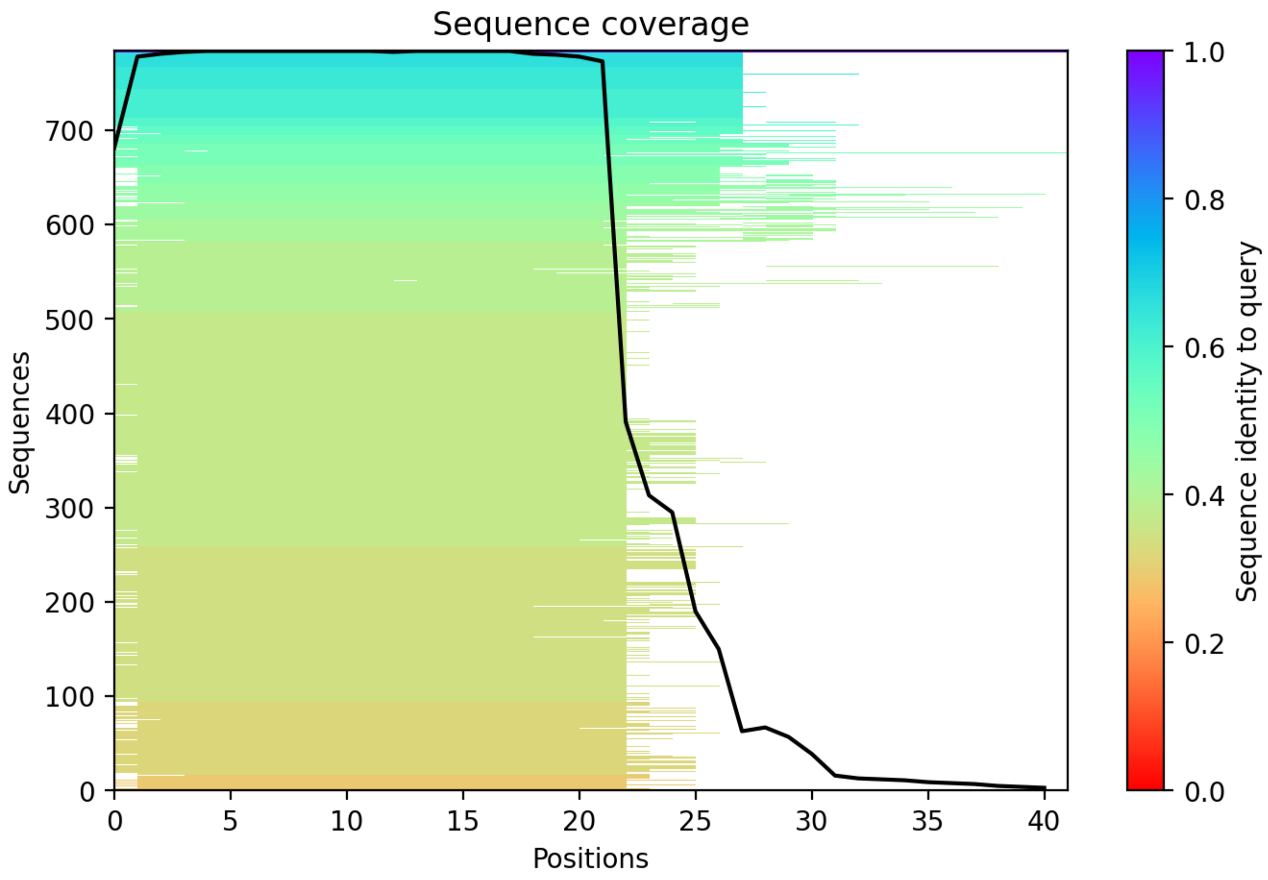

CP63: MtrunA17_Chr3g0144151

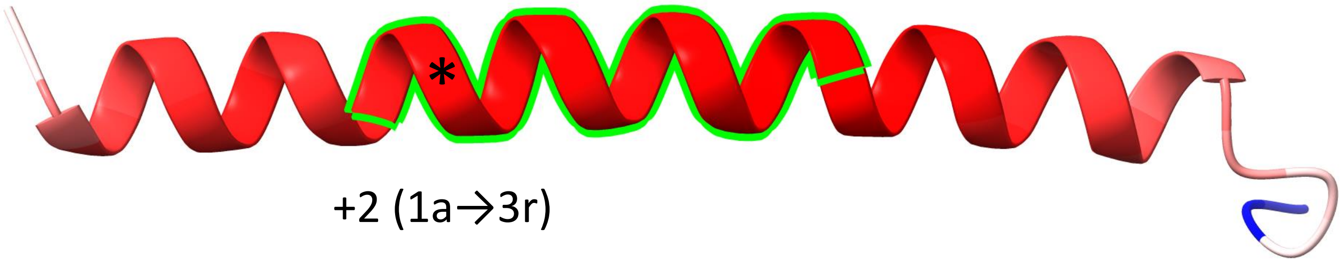

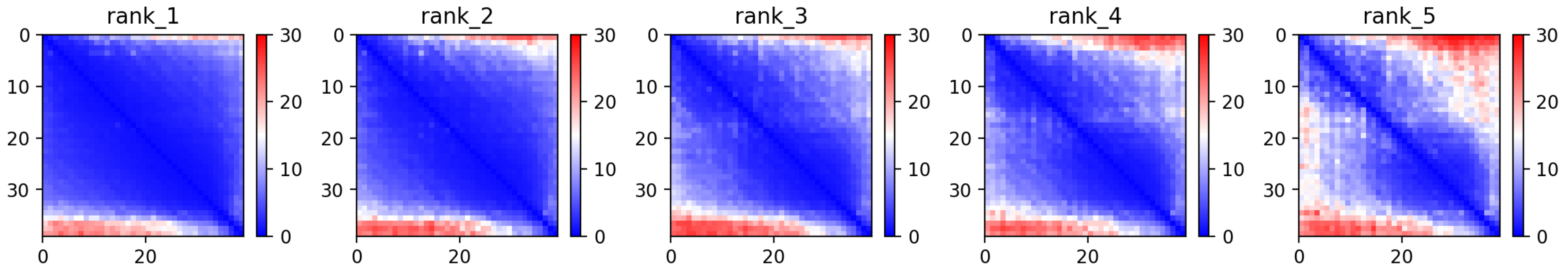

CP63: MtrunA17_Chr3g0144151

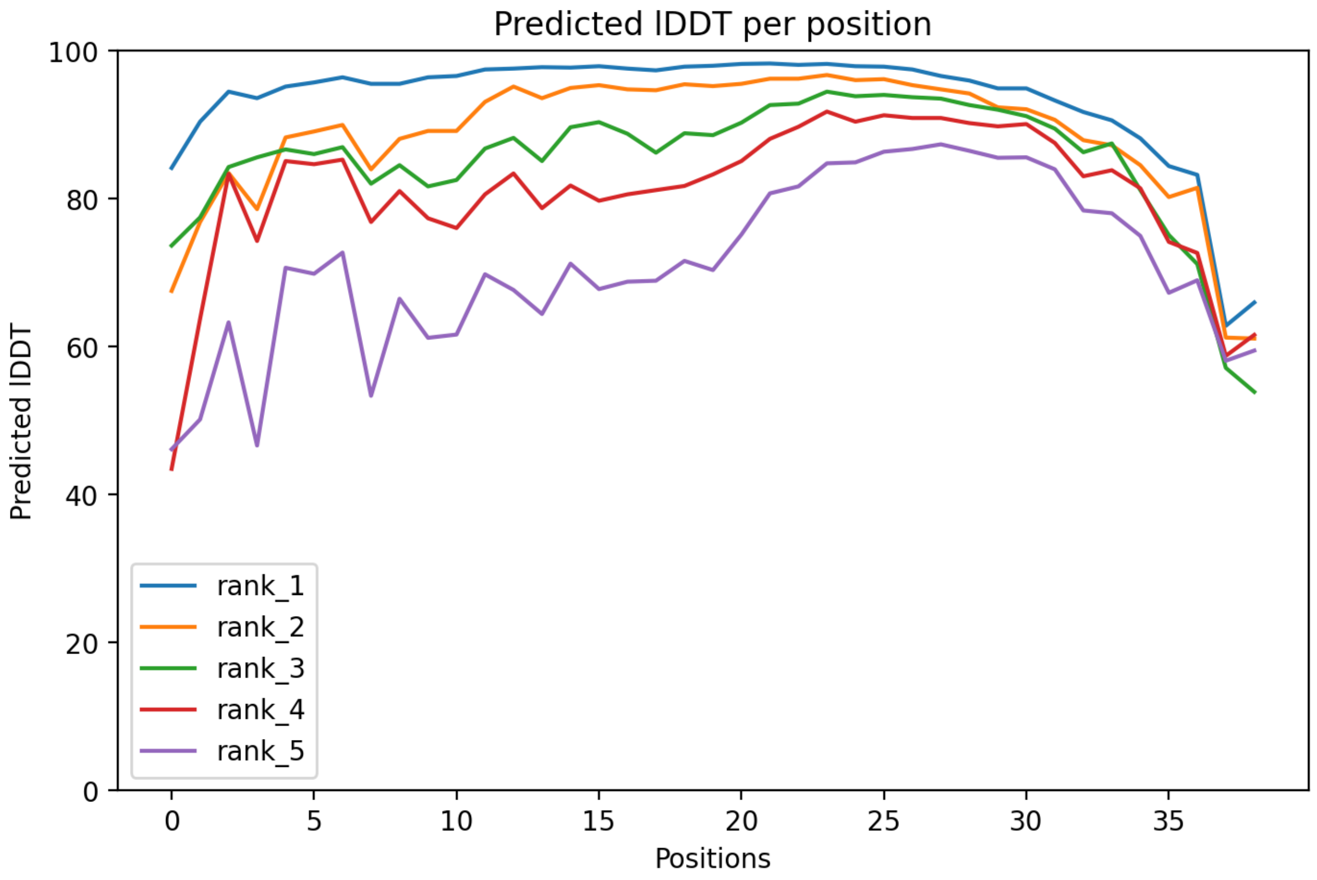

CP63: MtrunA17_Chr3g0144151

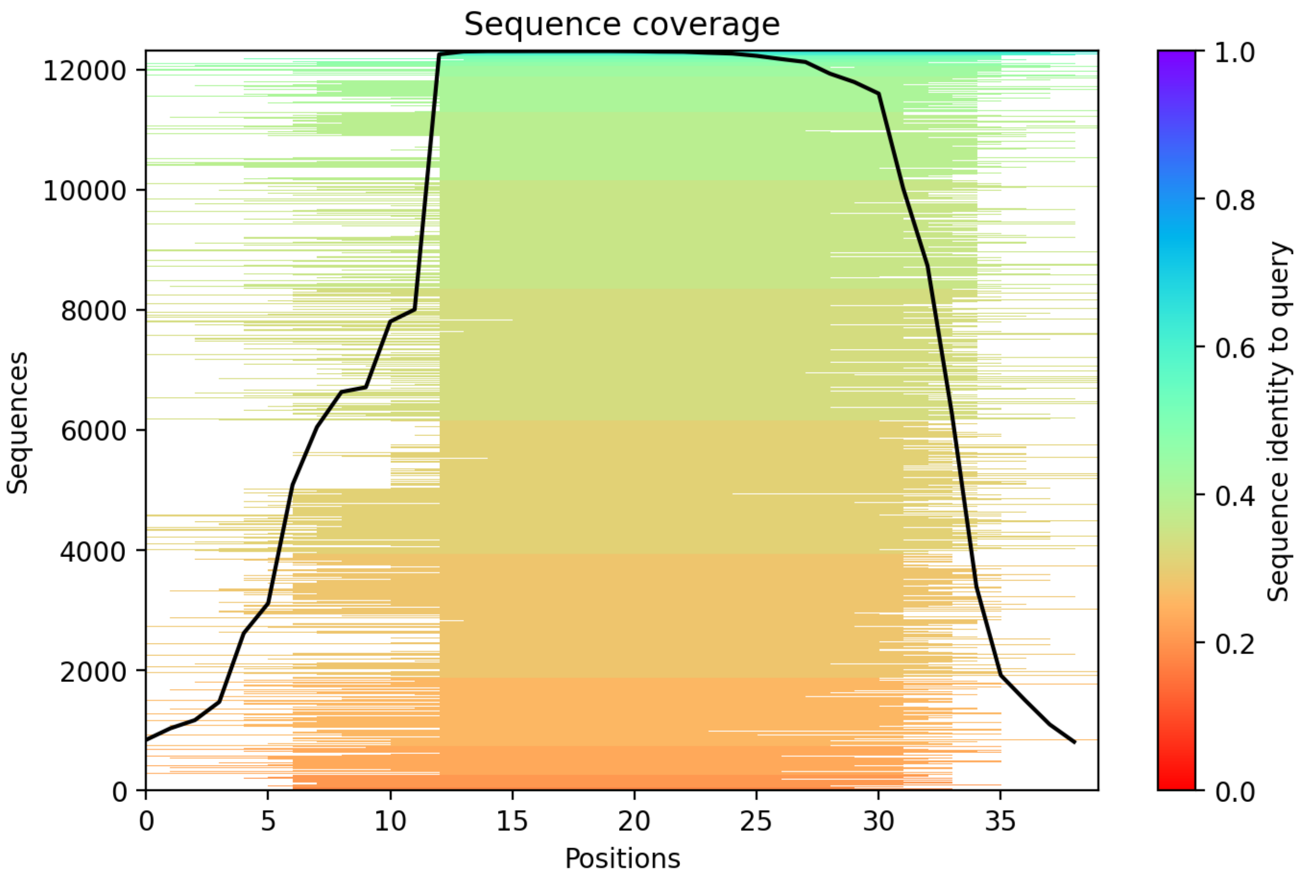

CP64: MtrunA17_Chr3g0144151

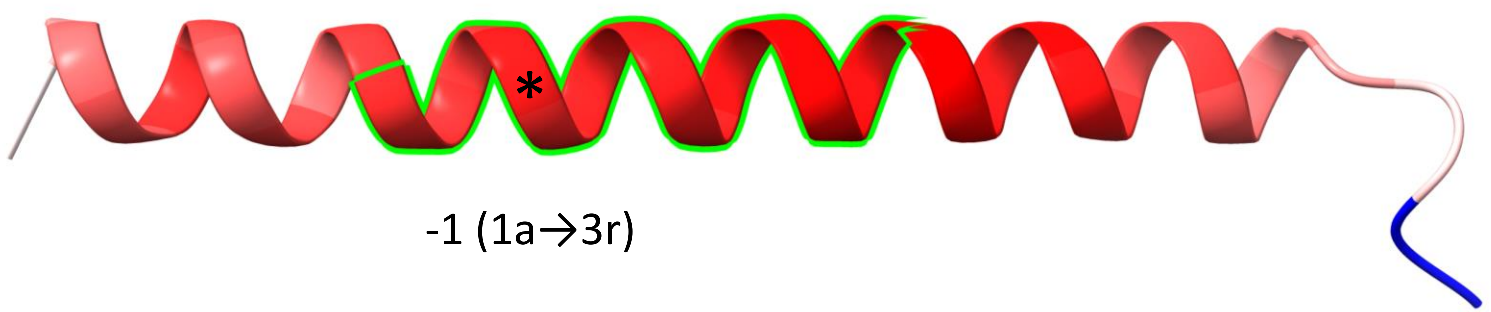

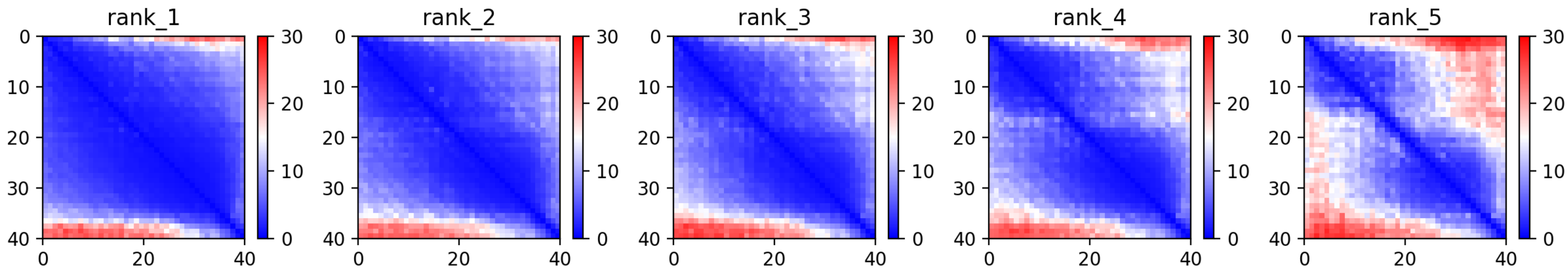

CP64: MtrunA17_Chr3g0144151

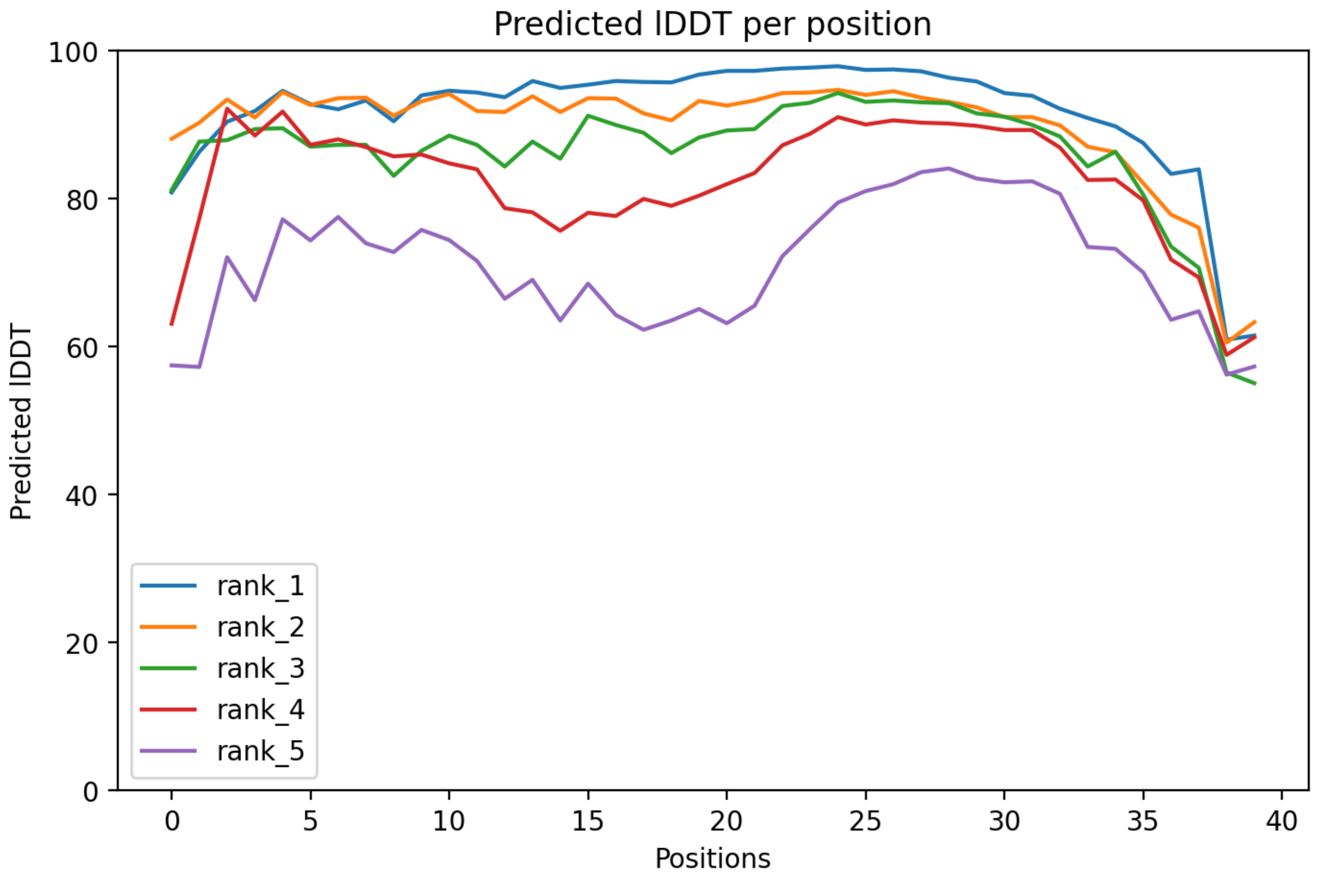

CP64: MtrunA17_Chr3g0144151

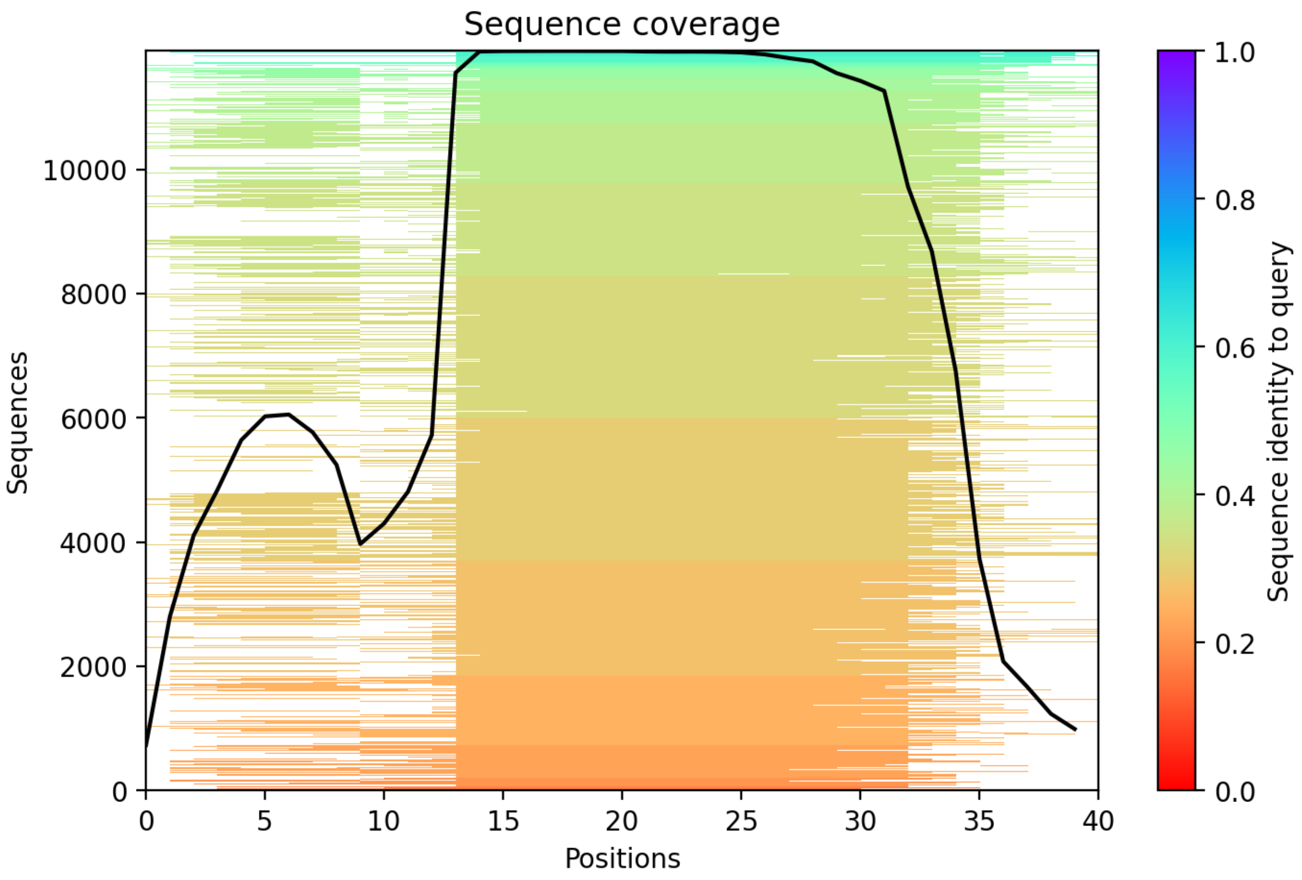

CP65: MtrunA17_Chr3g0144151

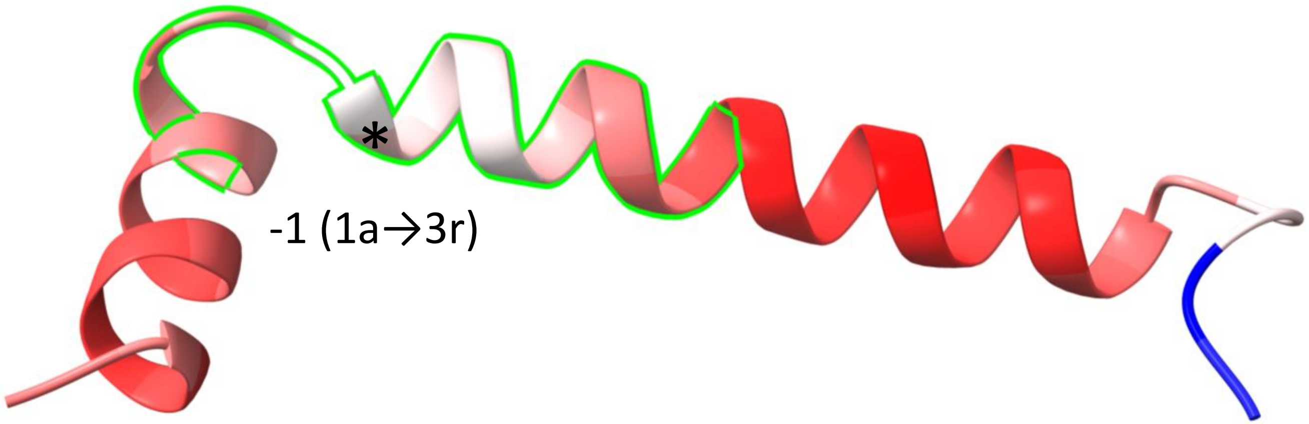

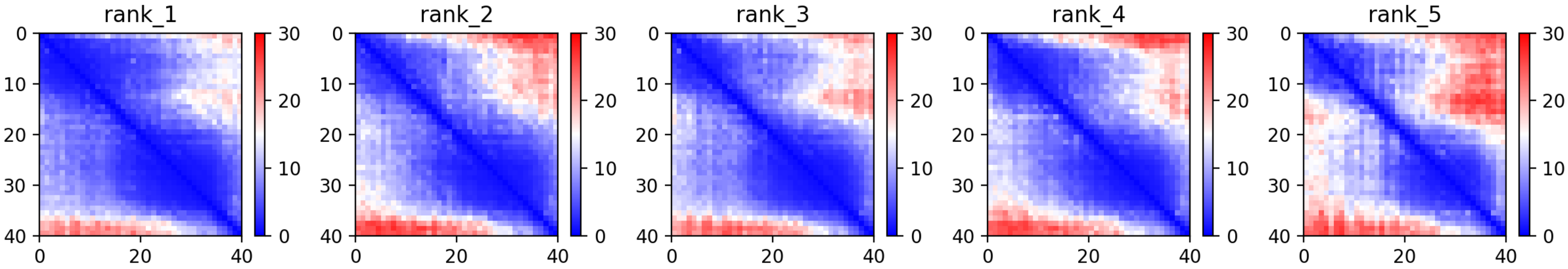

CP65: MtrunA17_Chr3g0144151

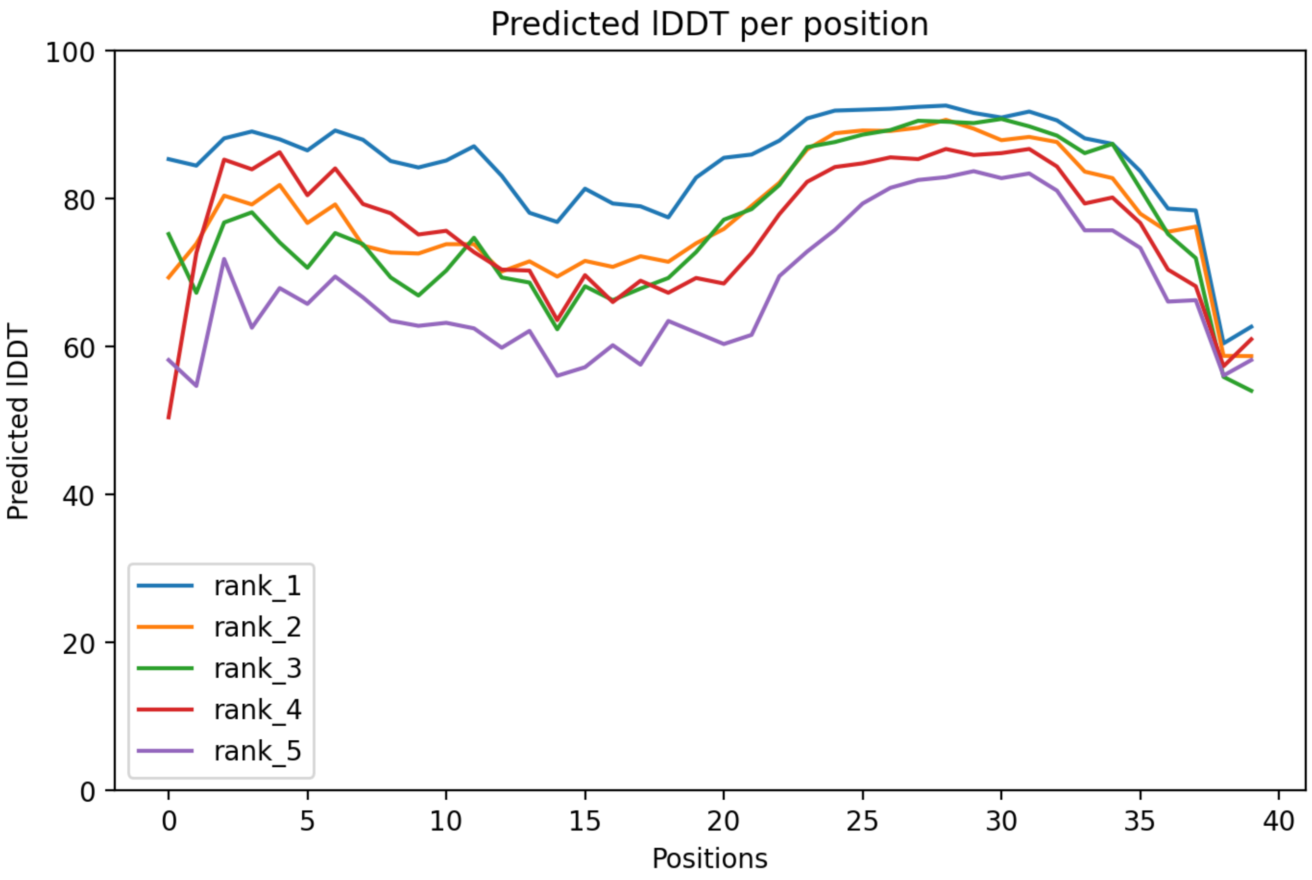

CP65: MtrunA17_Chr3g0144151

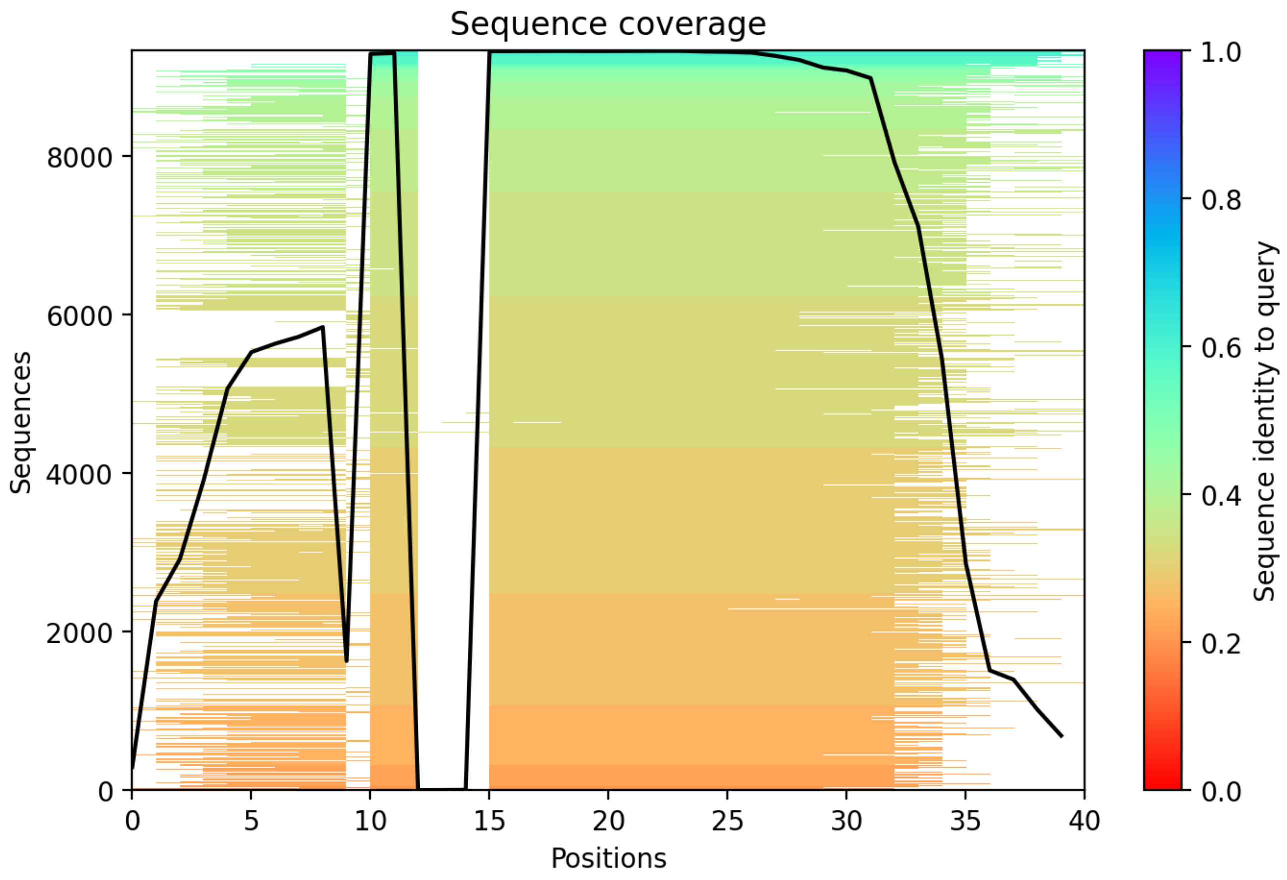

CP66: MtrunA17_Chr3g1011650

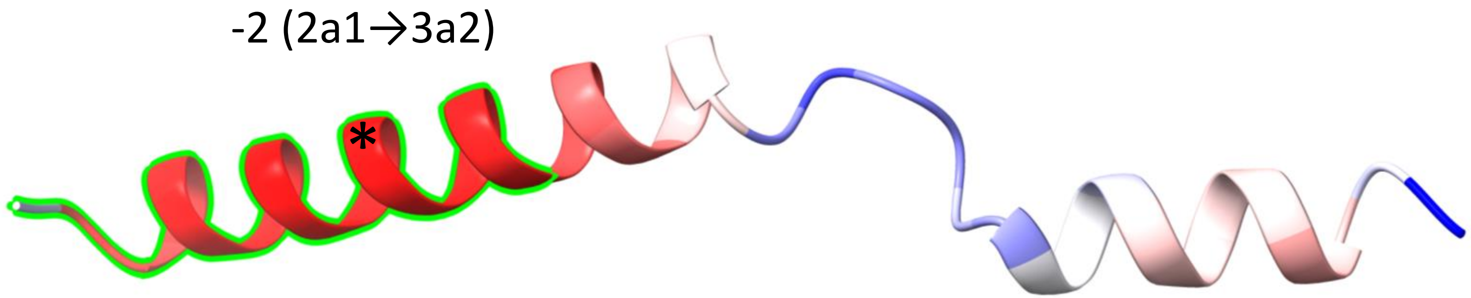

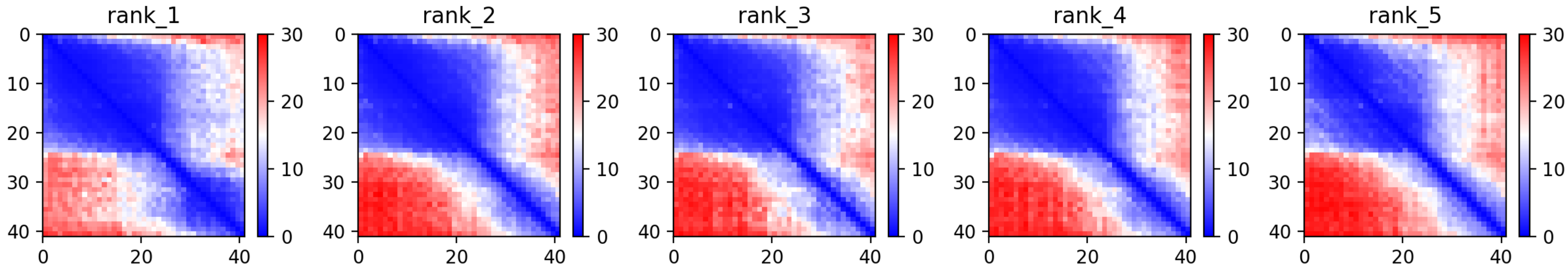

CP66: MtrunA17_Chr3g1011650

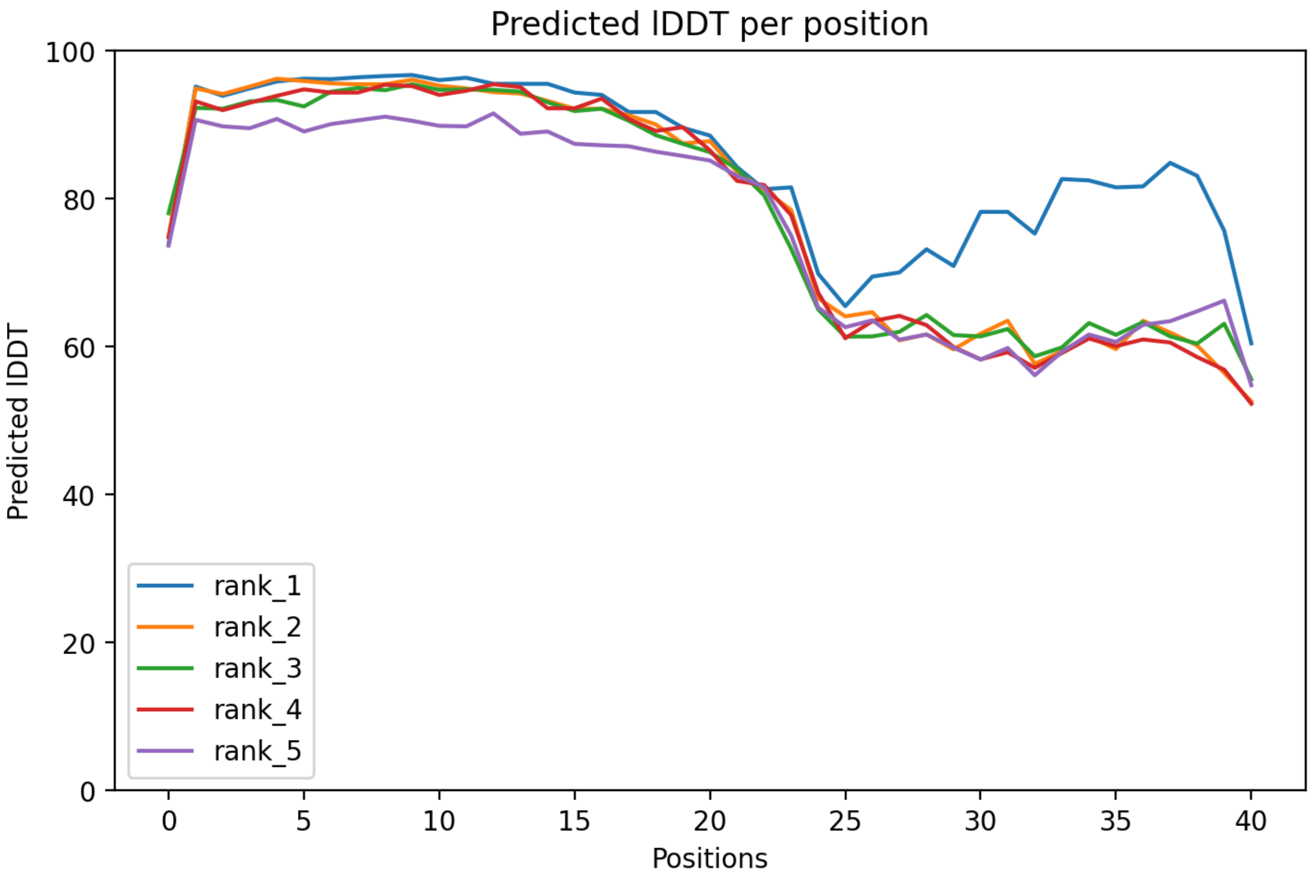

CP66: MtrunA17_Chr3g1011650

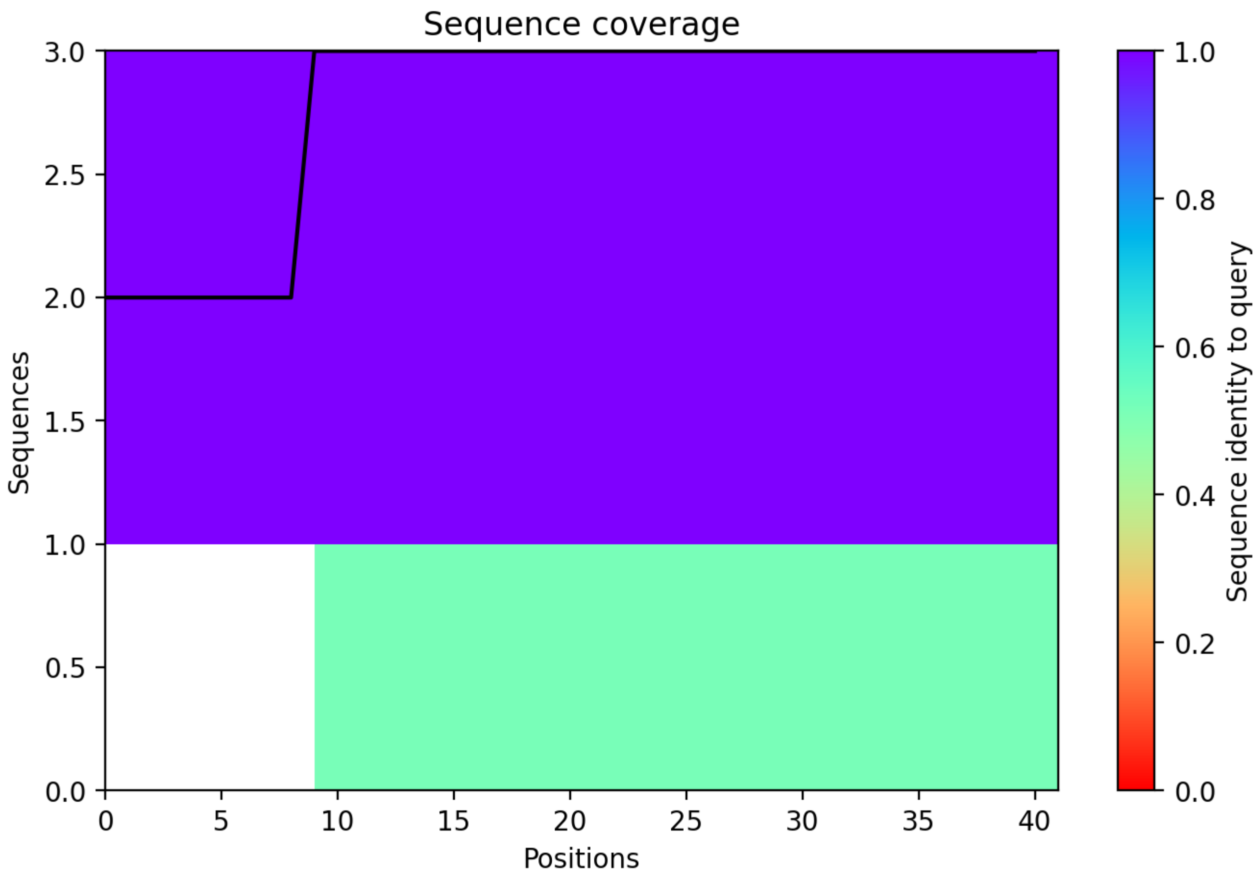

CP67: MtrunA17_Chr4g0000131

+1 (3r→1a)

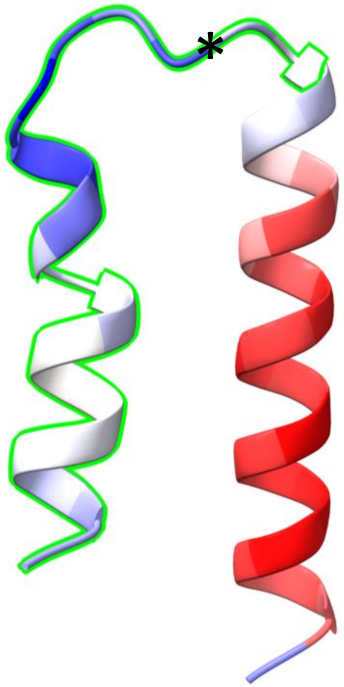

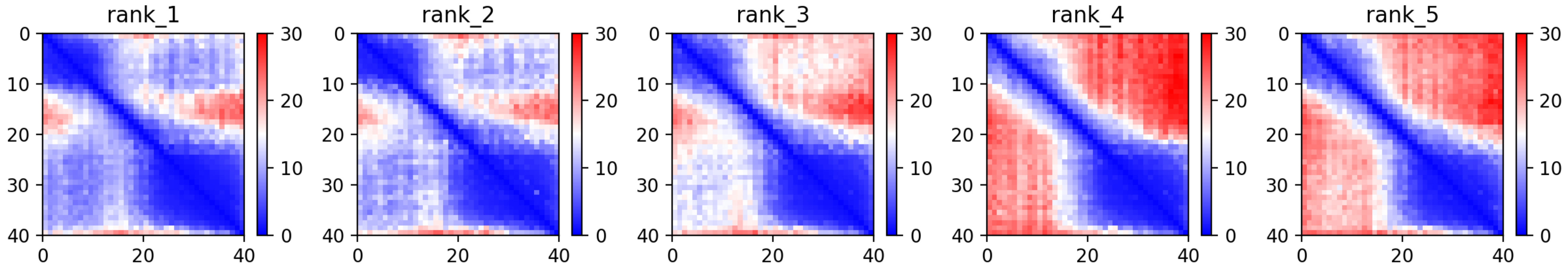

CP67: MtrunA17_Chr4g0000131

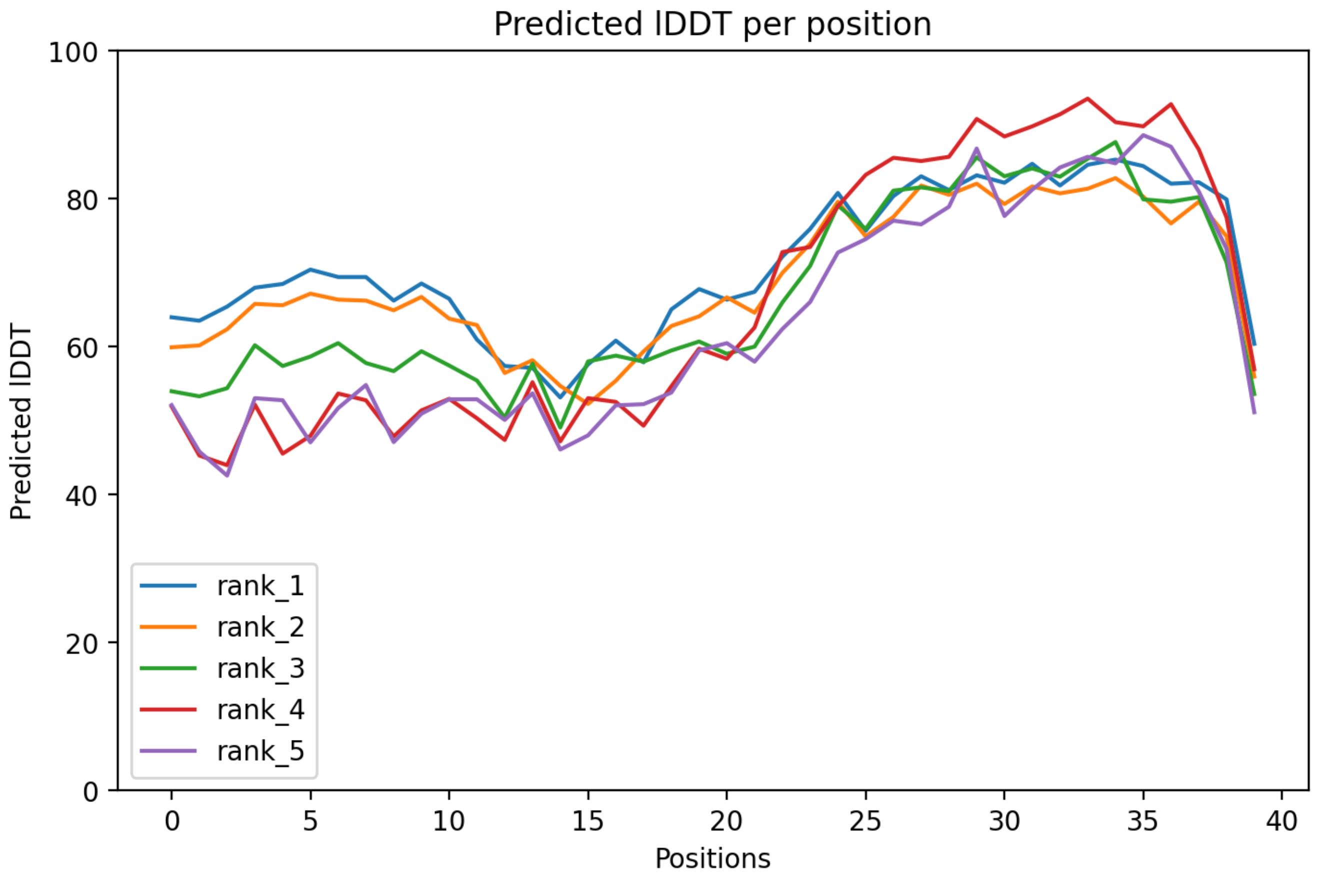

CP67: MtrunA17_Chr4g0000131

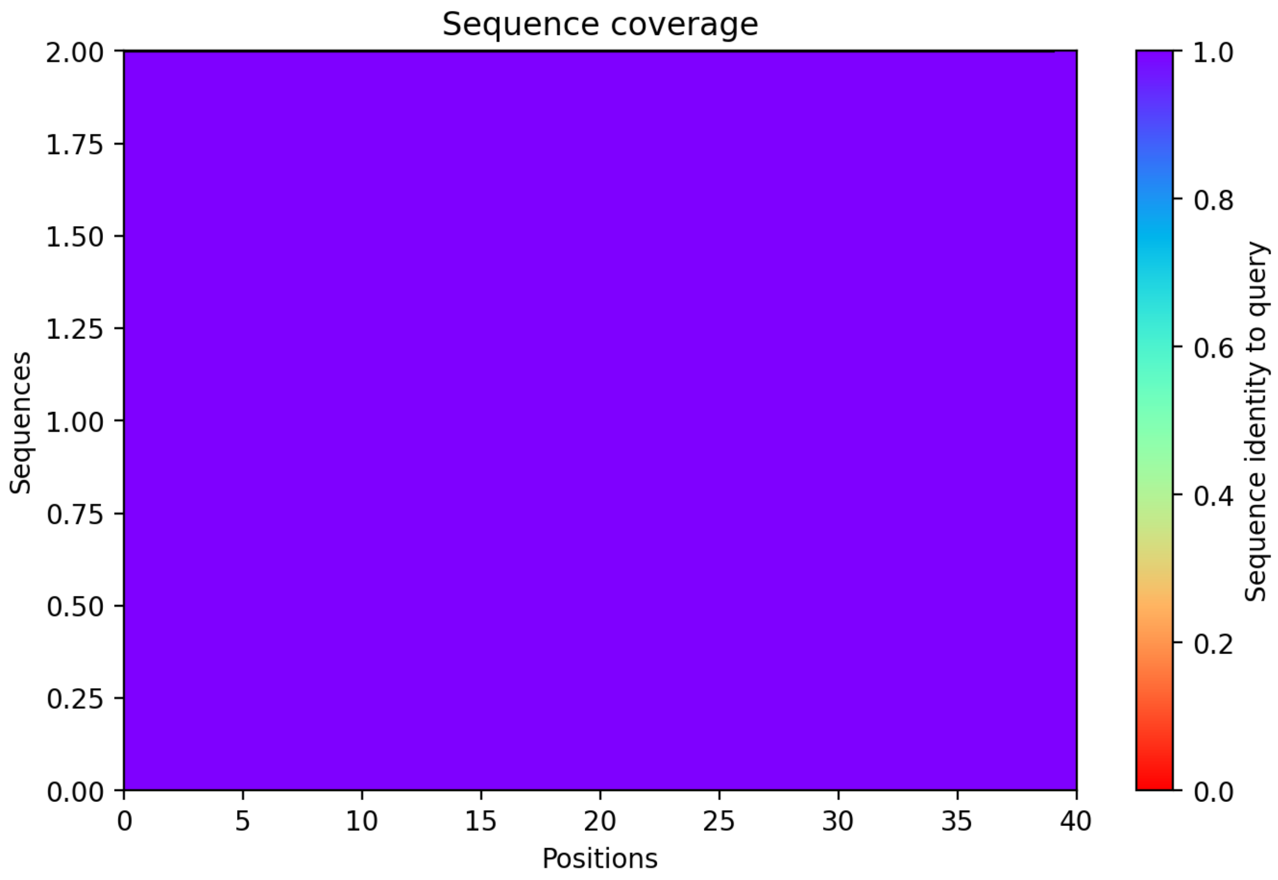

CP68: MtrunA17_Chr4g0000891

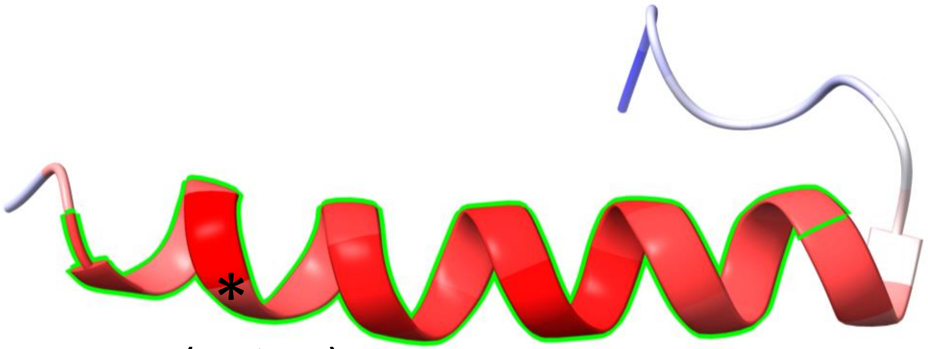

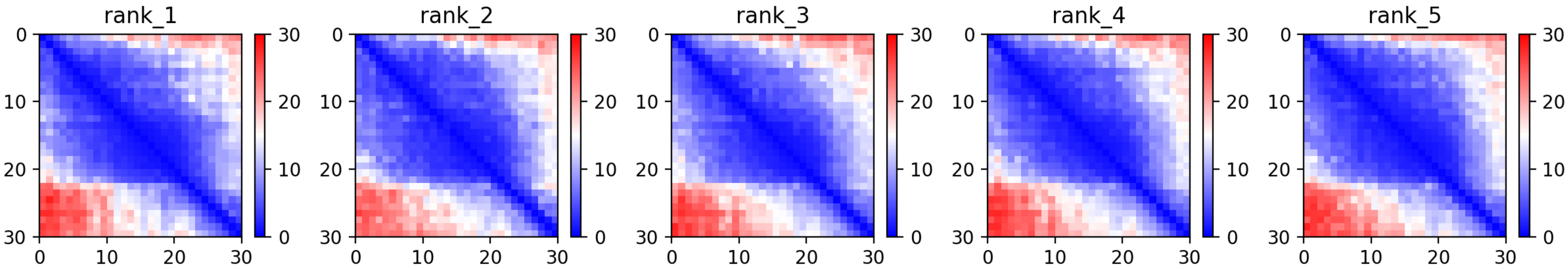

CP68: MtrunA17_Chr4g0000891

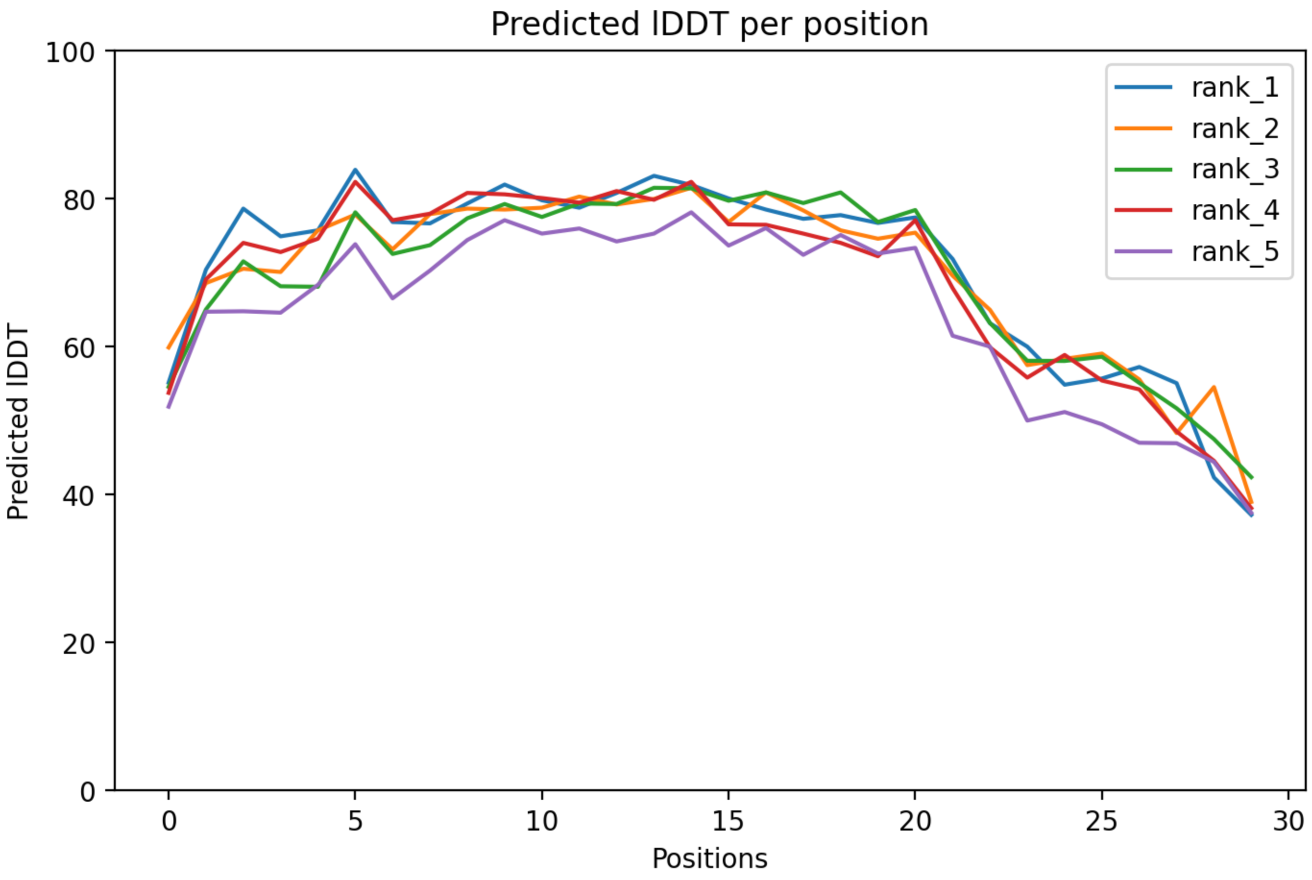

CP68: MtrunA17_Chr4g0000891

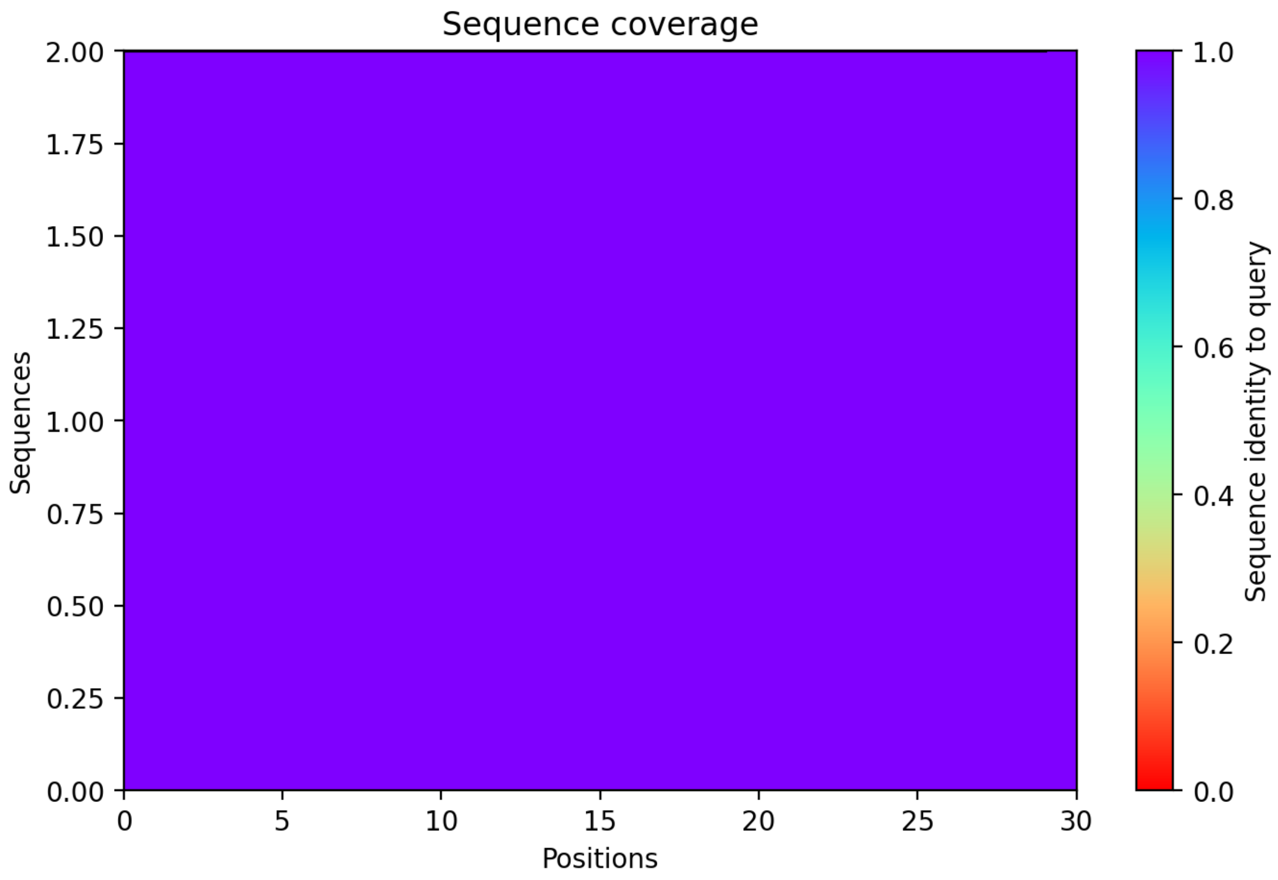

CP69: MtrunA17_Chr4g0004721

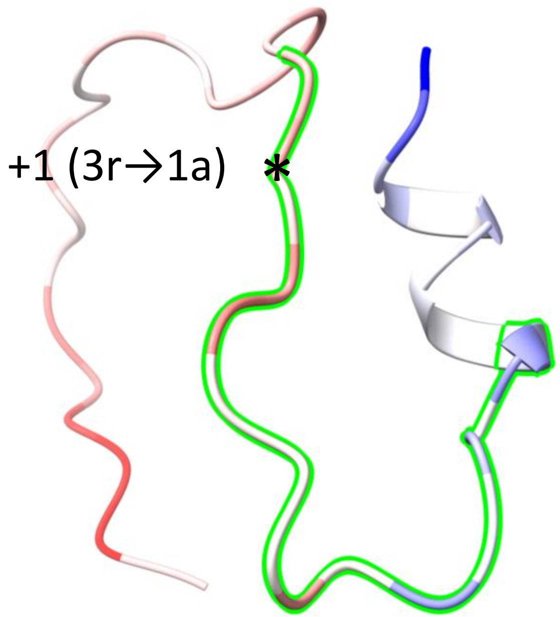

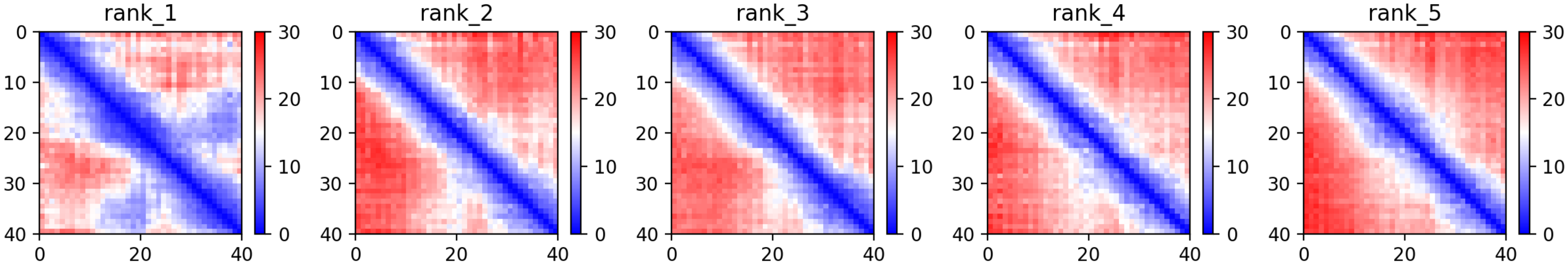

CP69: MtrunA17_Chr4g0004721

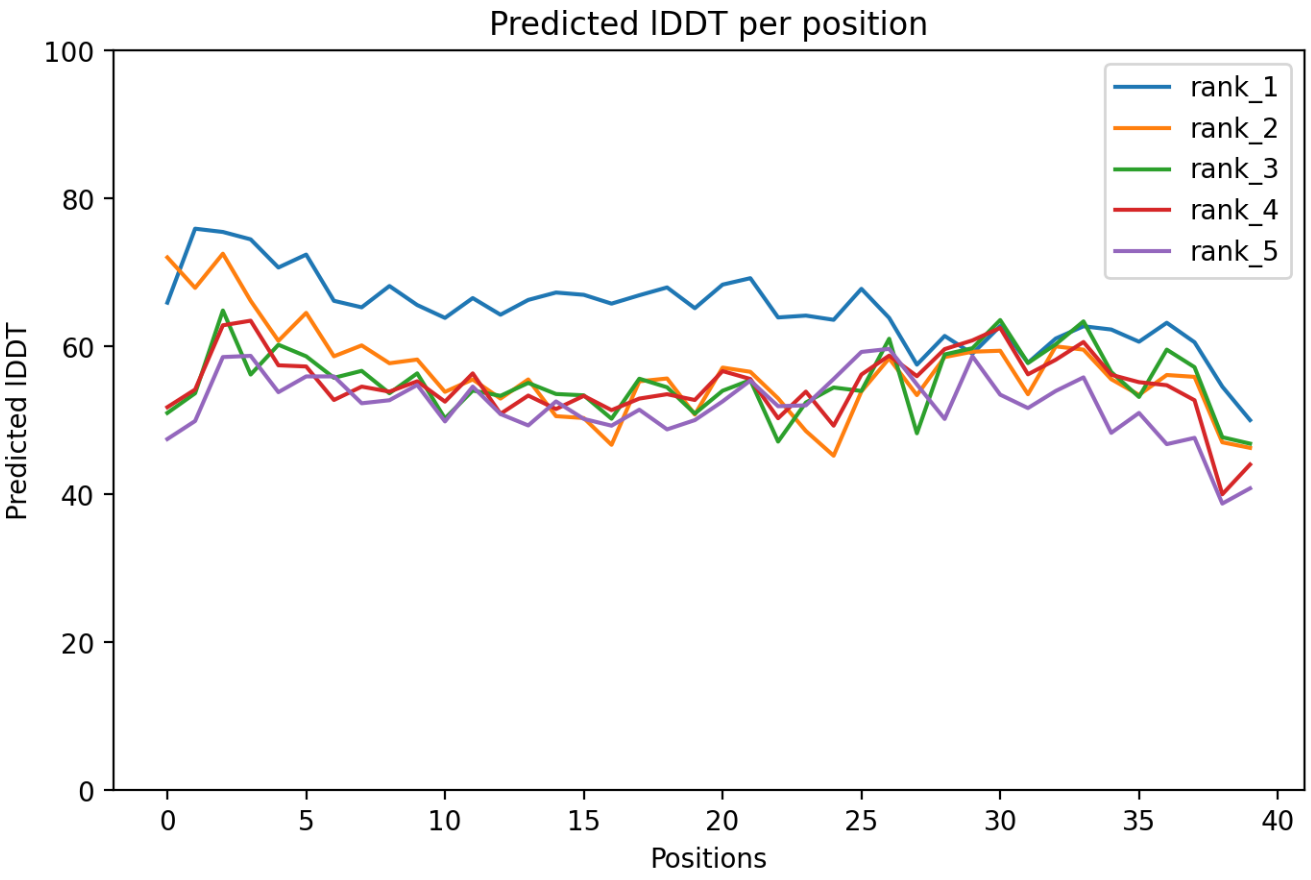

CP69: MtrunA17_Chr4g0004721

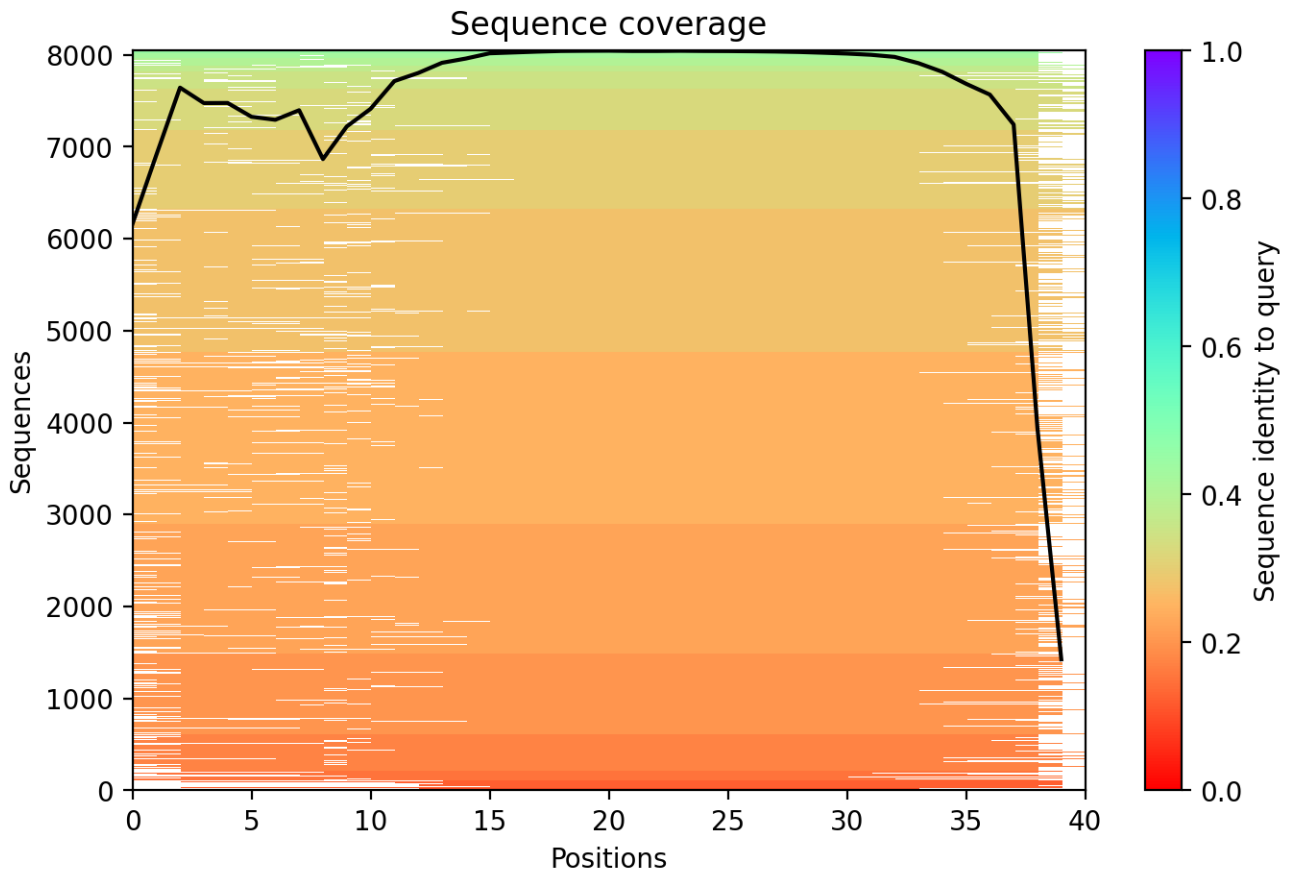

CP70: MtrunA17_Chr4g0014251

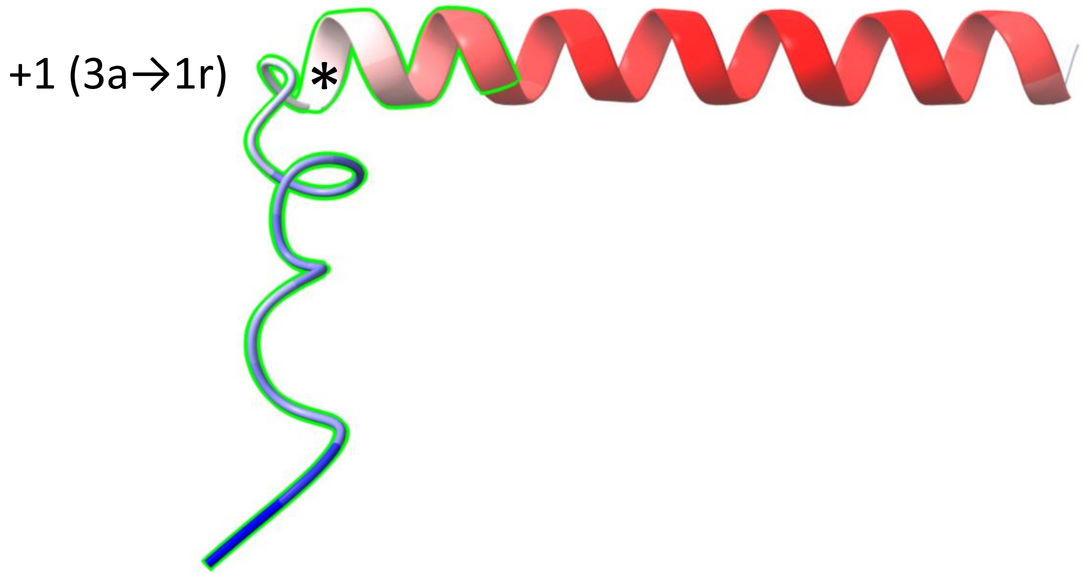

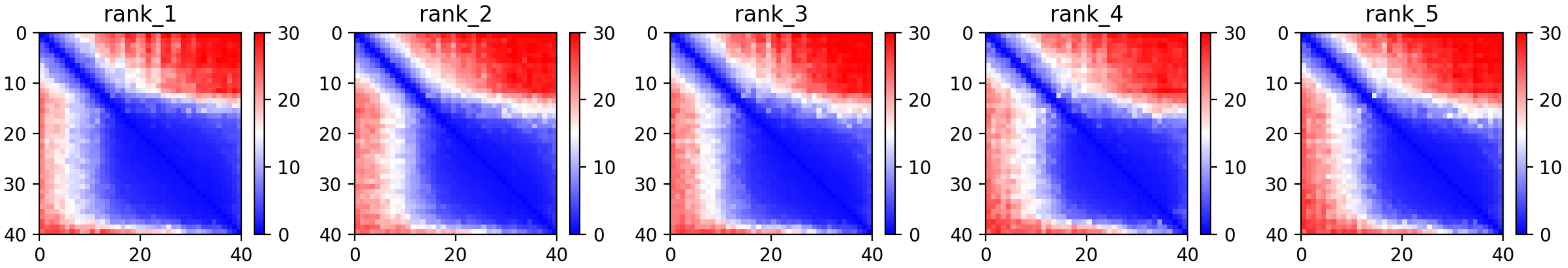

CP70: MtrunA17_Chr4g0014251

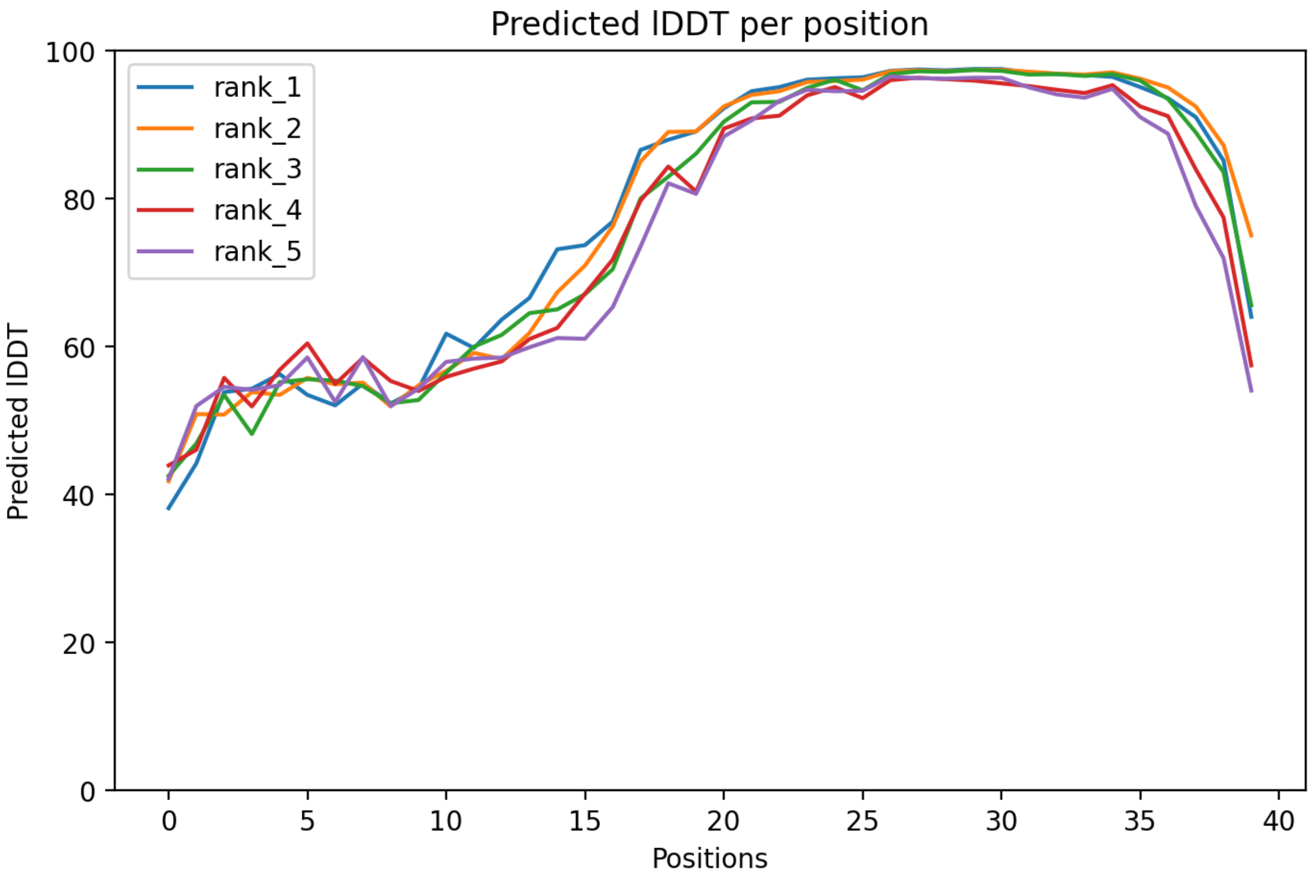

CP70: MtrunA17_Chr4g0014251

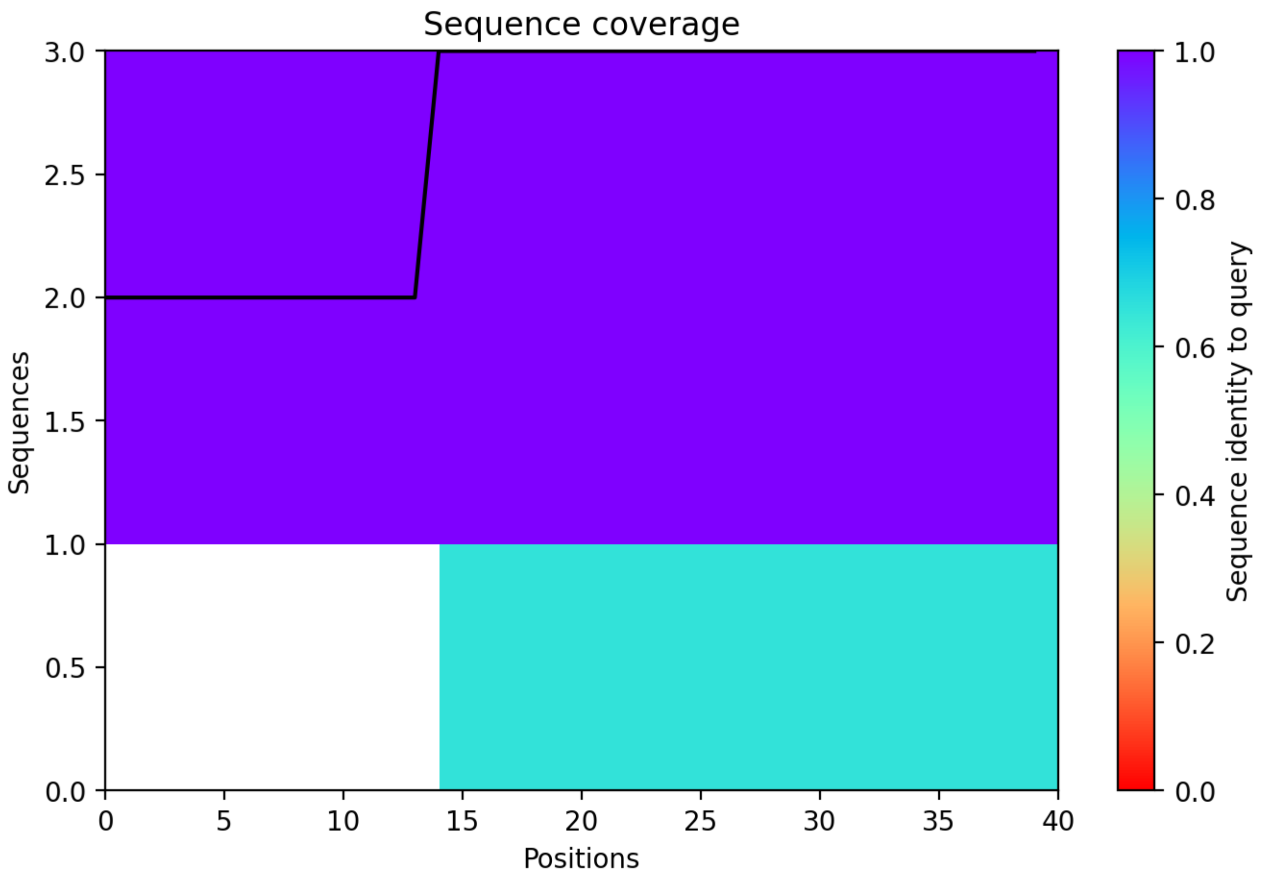

CP71: MtrunA17_Chr4g0022491

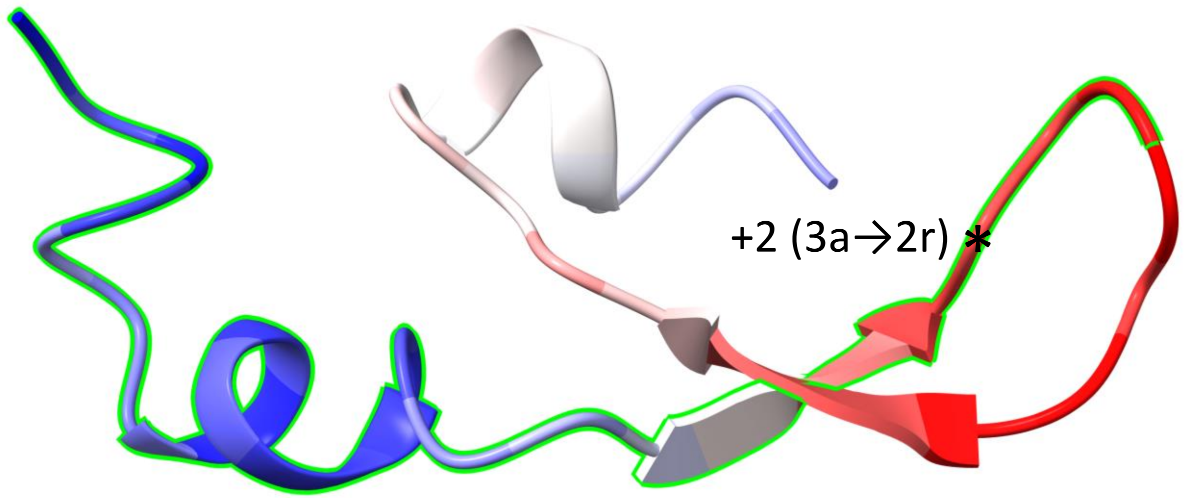

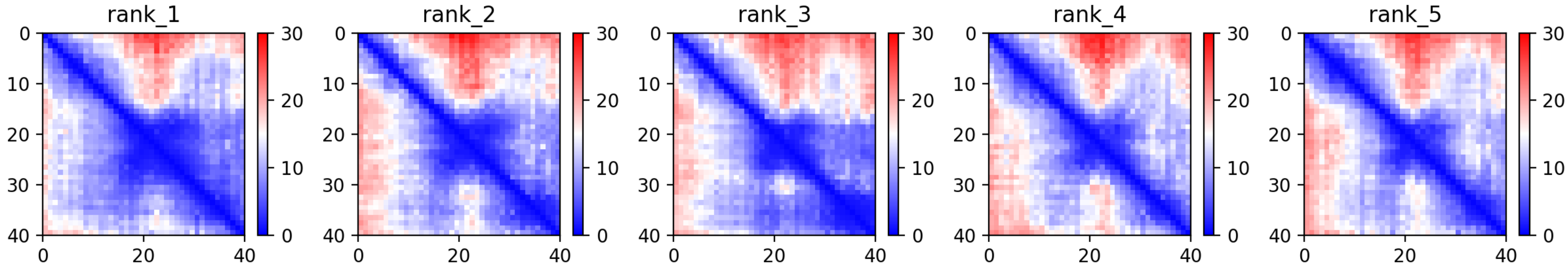

CP71: MtrunA17_Chr4g0022491

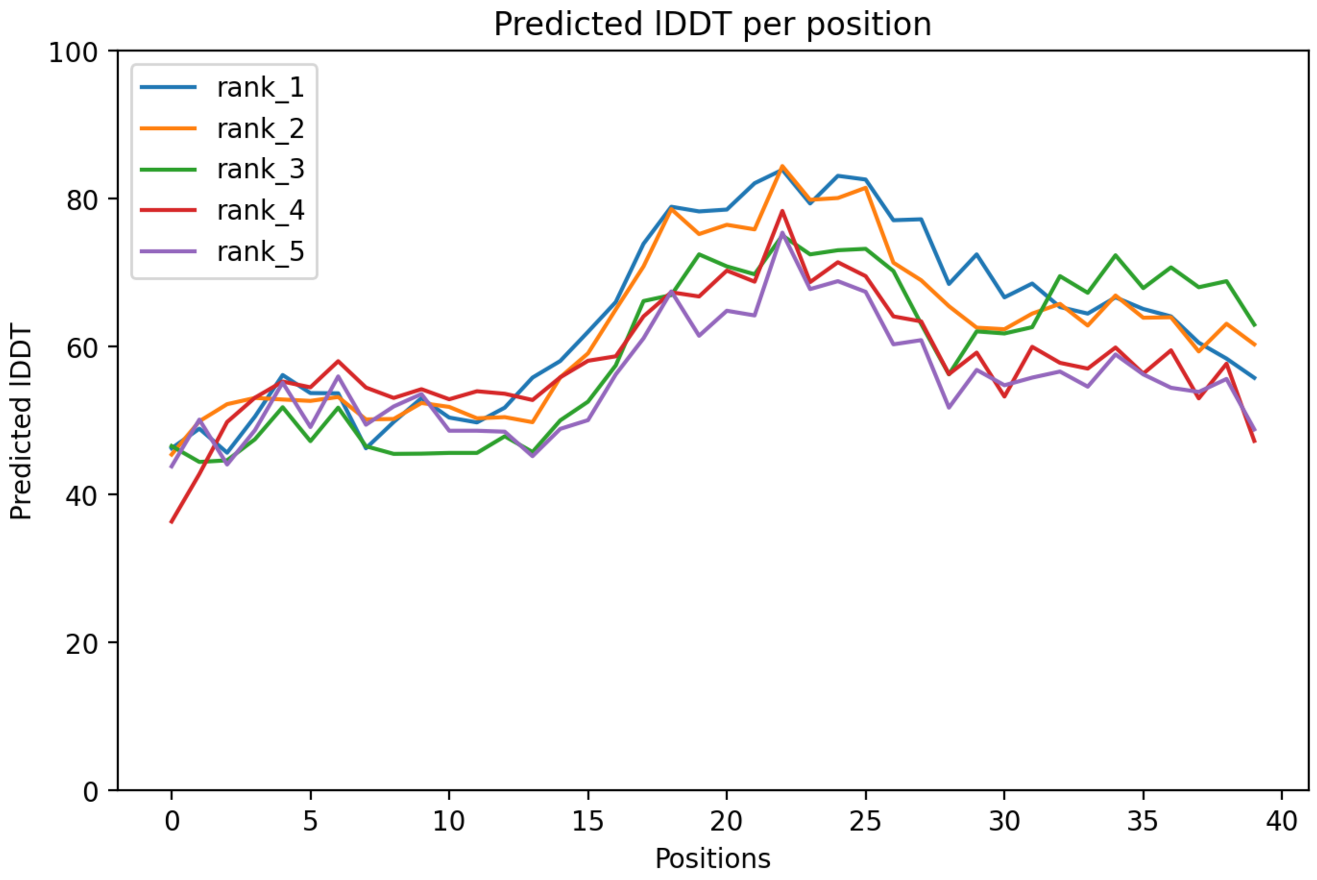

CP71: MtrunA17_Chr4g0022491

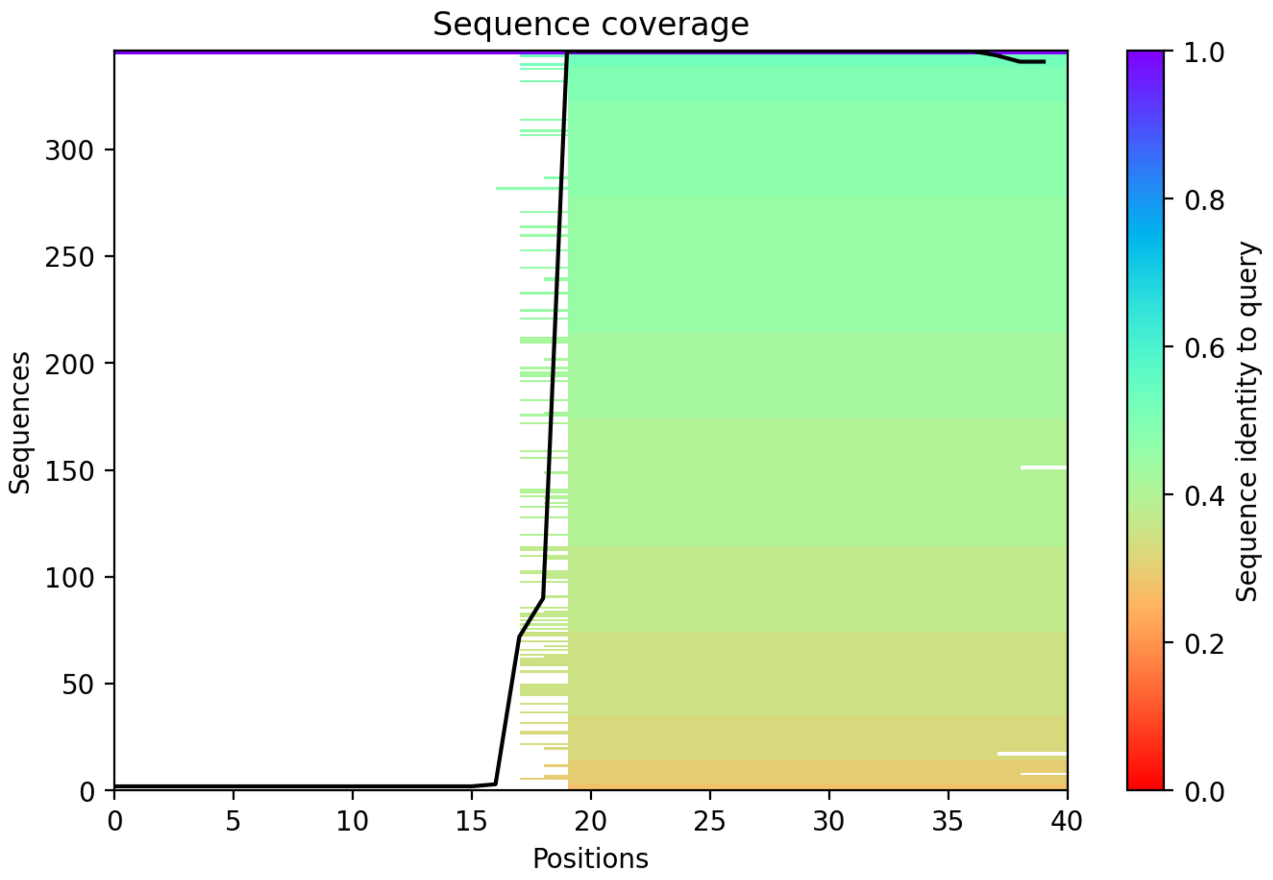

CP72: MtrunA17_Chr4g0023791

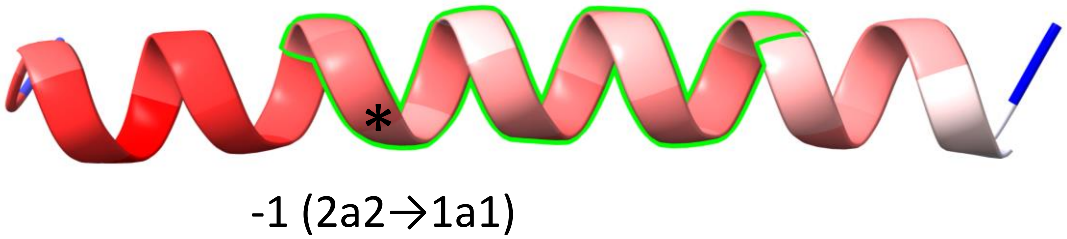

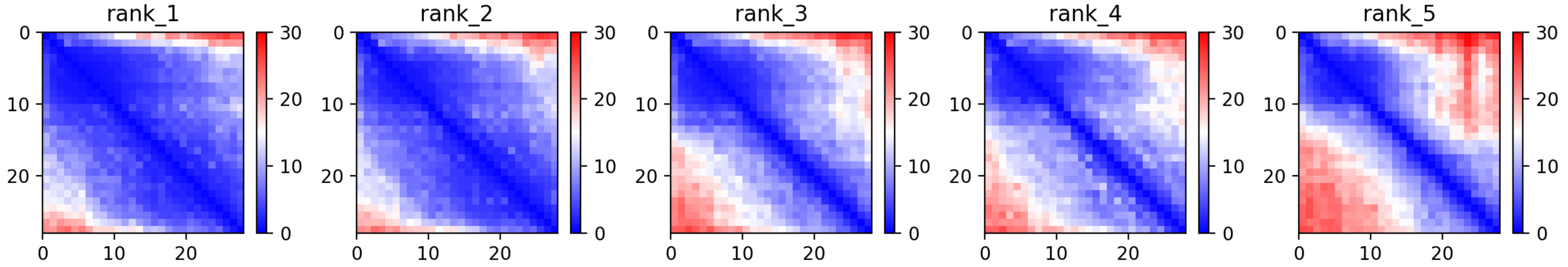

CP72: MtrunA17_Chr4g0023791

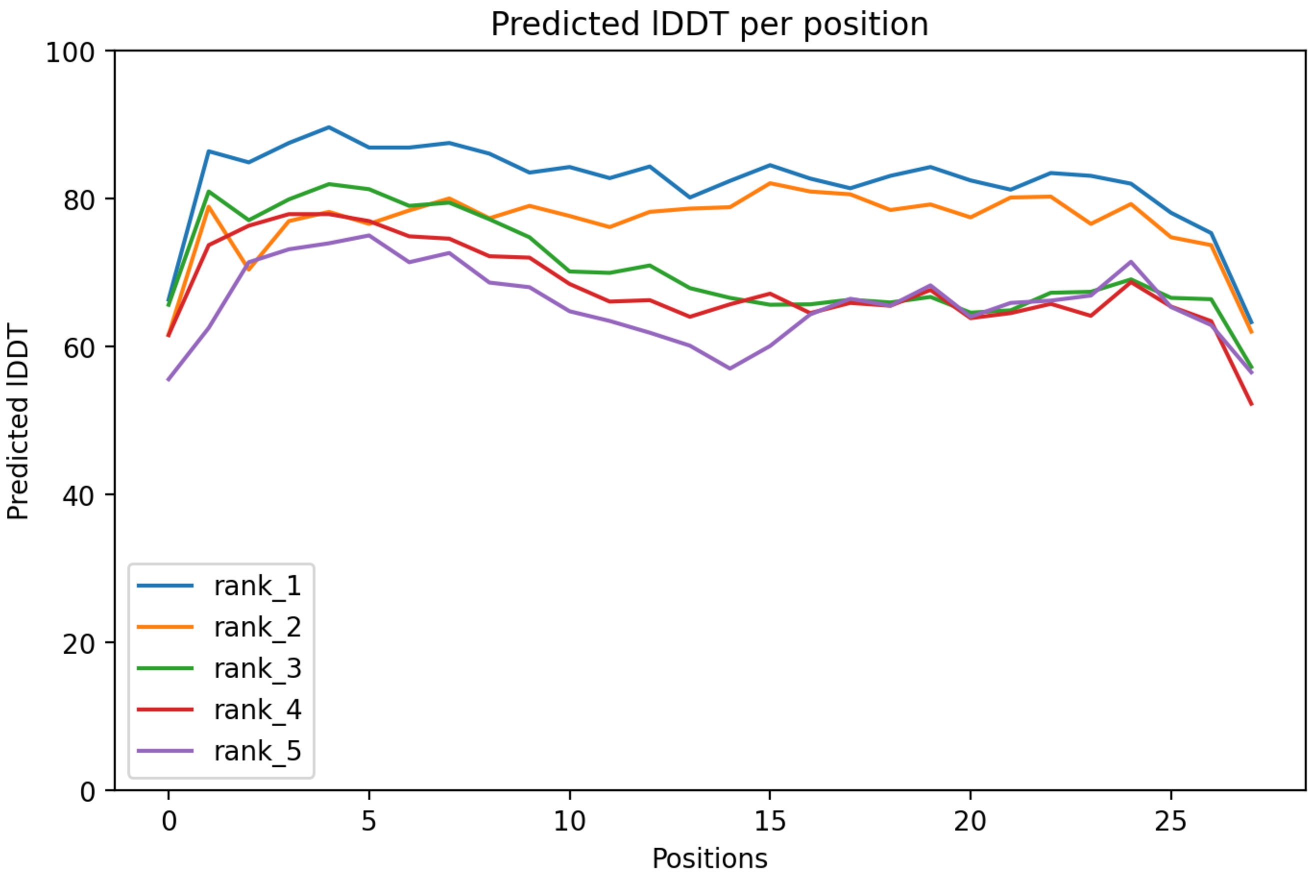

CP72: MtrunA17_Chr4g0023791

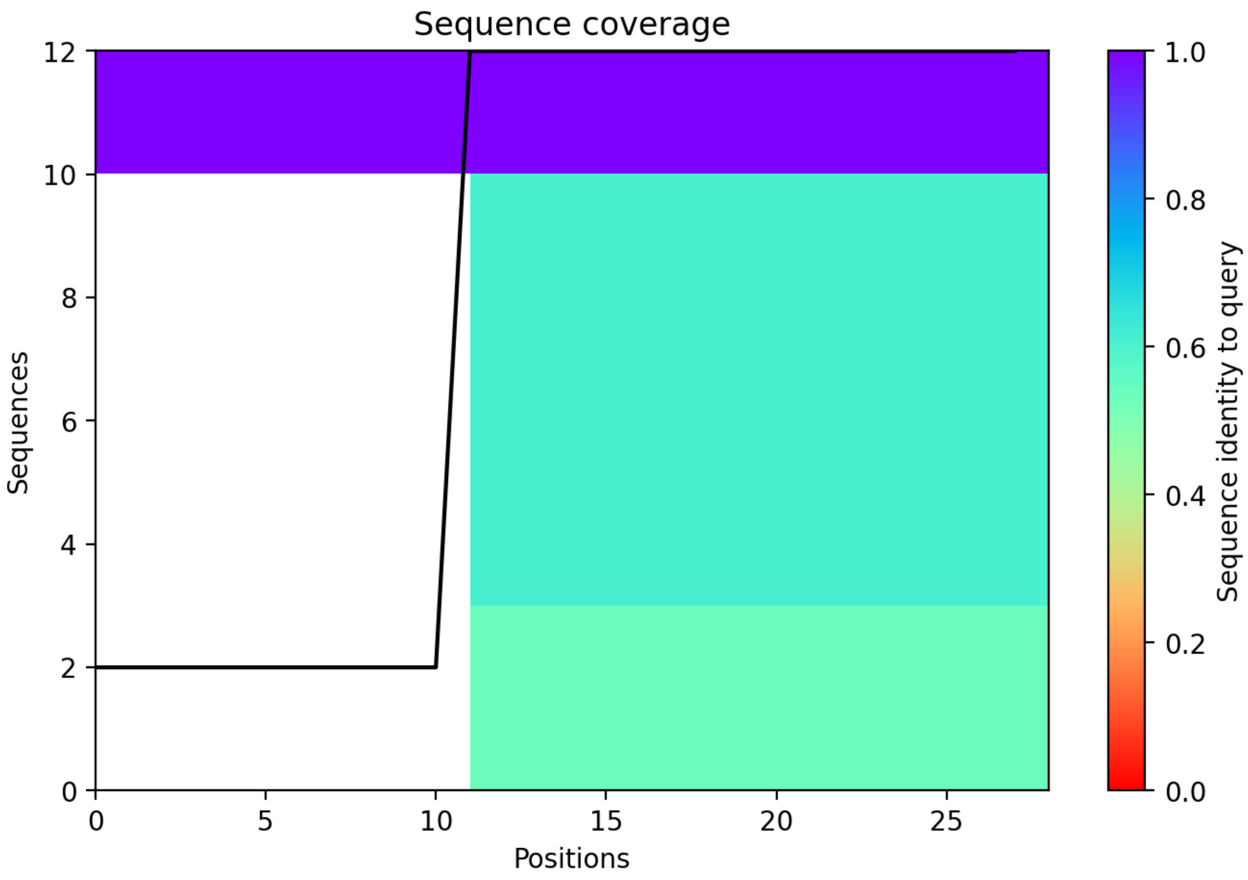

CP73: MtrunA17_Chr4g0034271

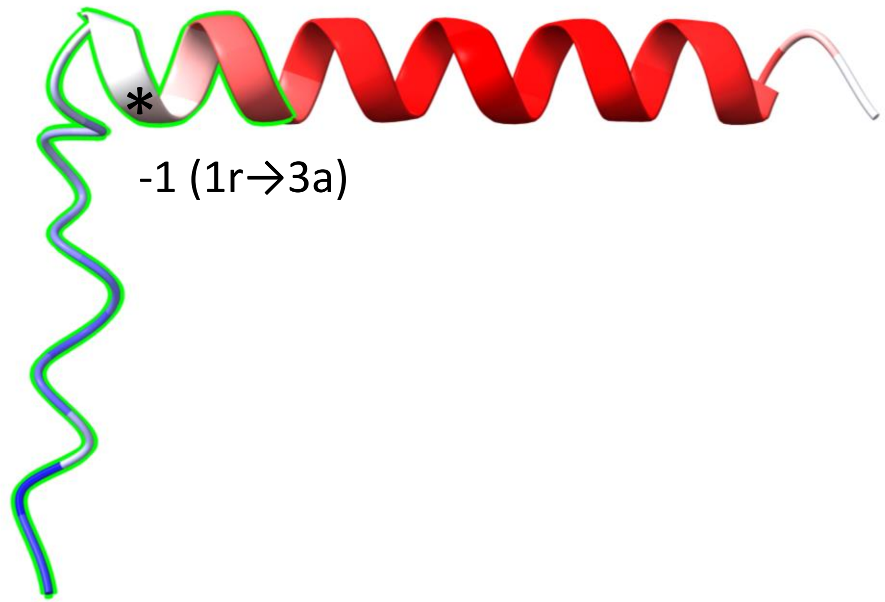

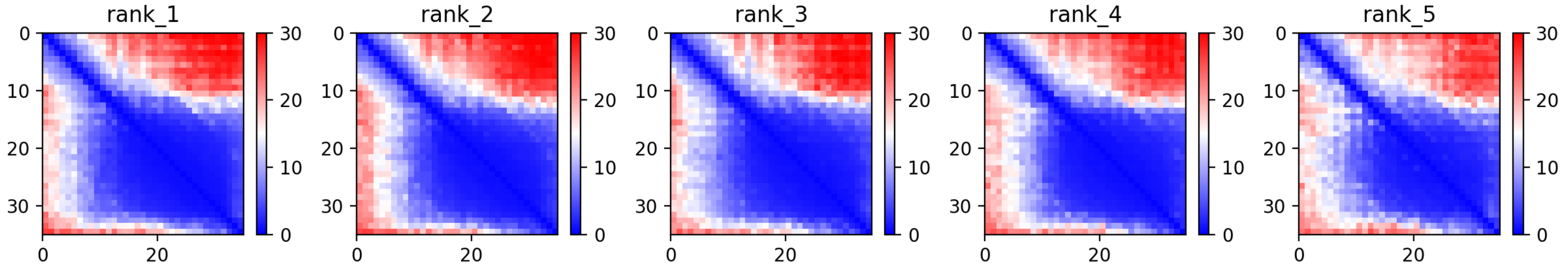

CP73: MtrunA17_Chr4g0034271

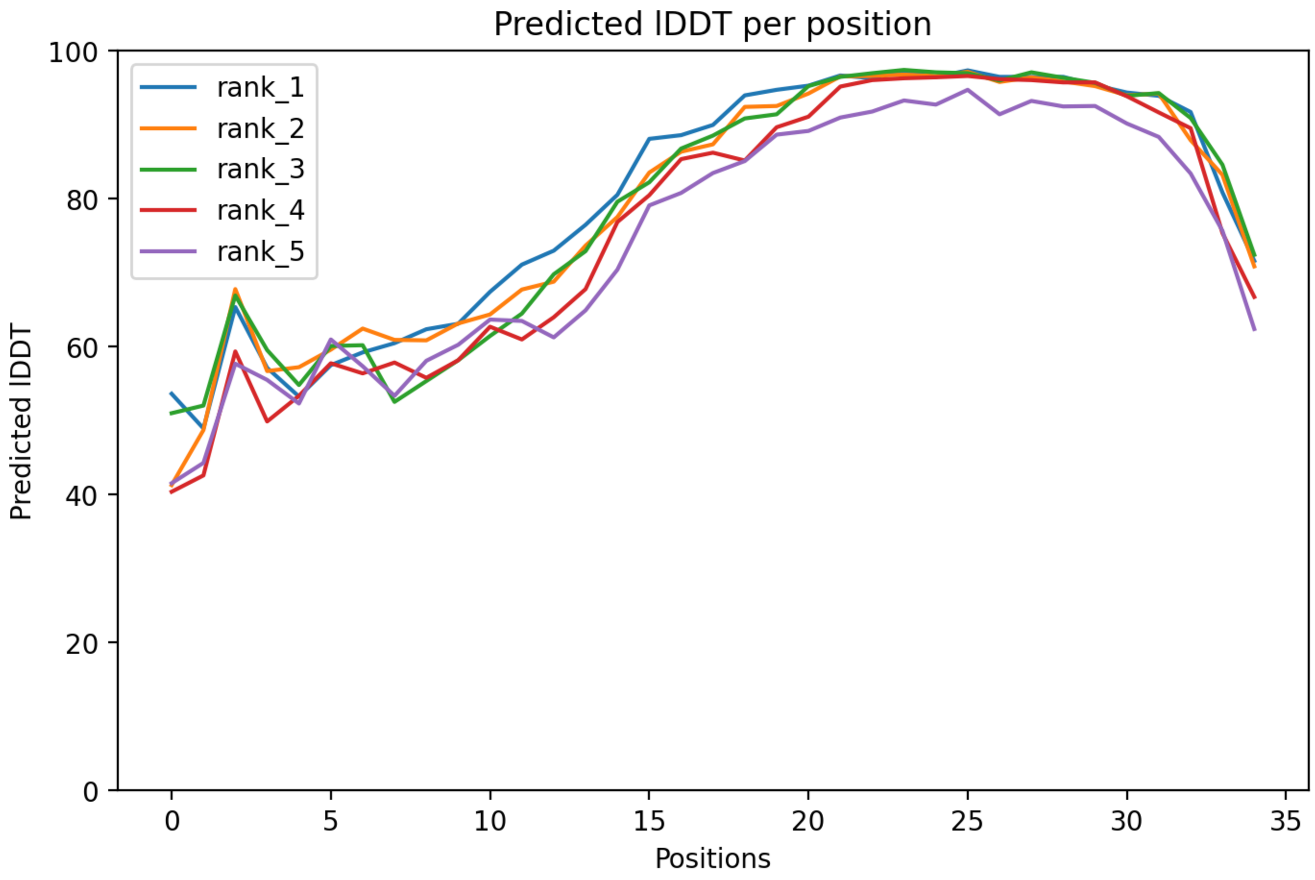

CP73: MtrunA17_Chr4g0034271

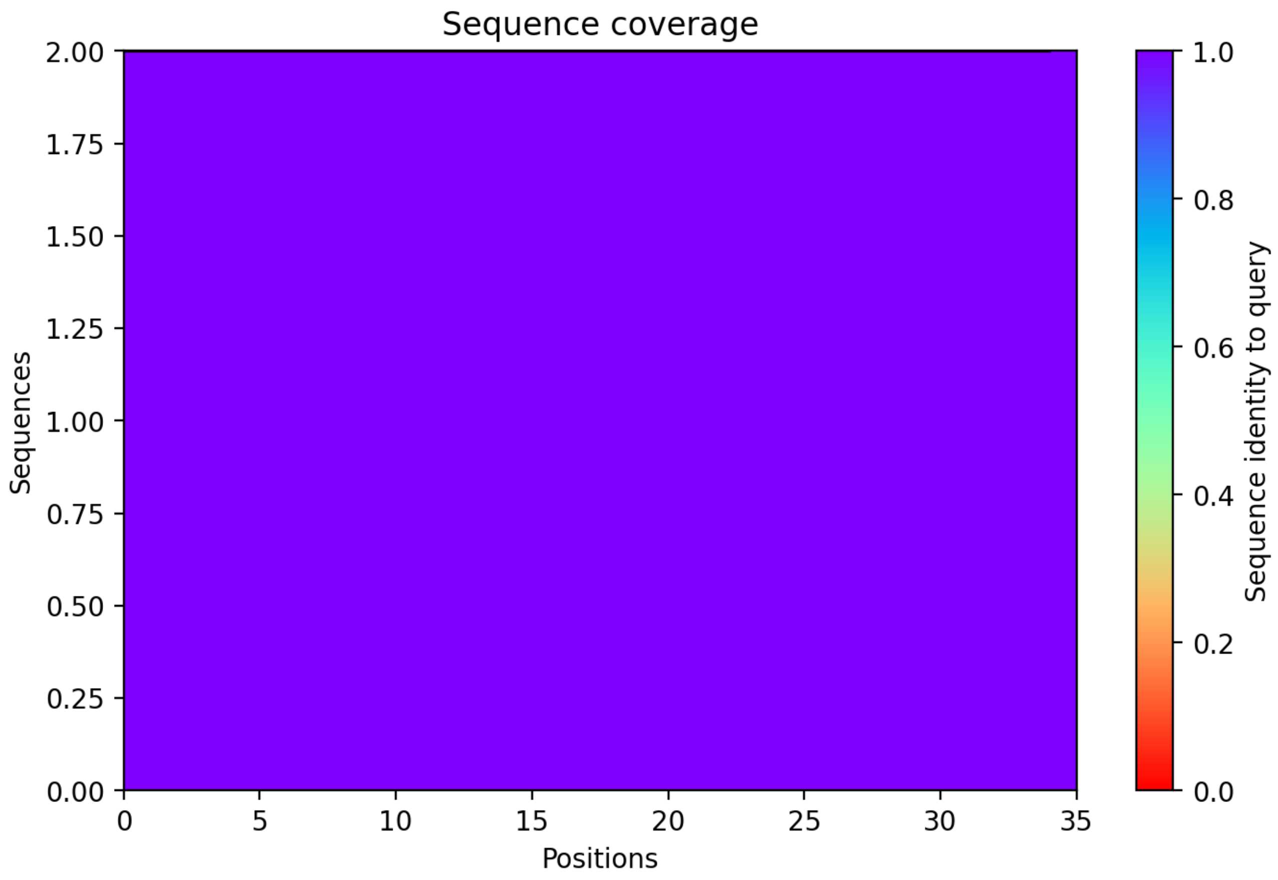

CP74: MtrunA17_Chr4g0034491

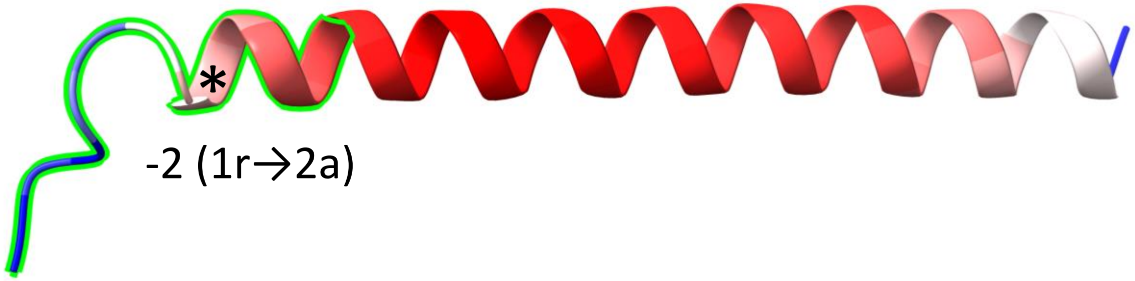

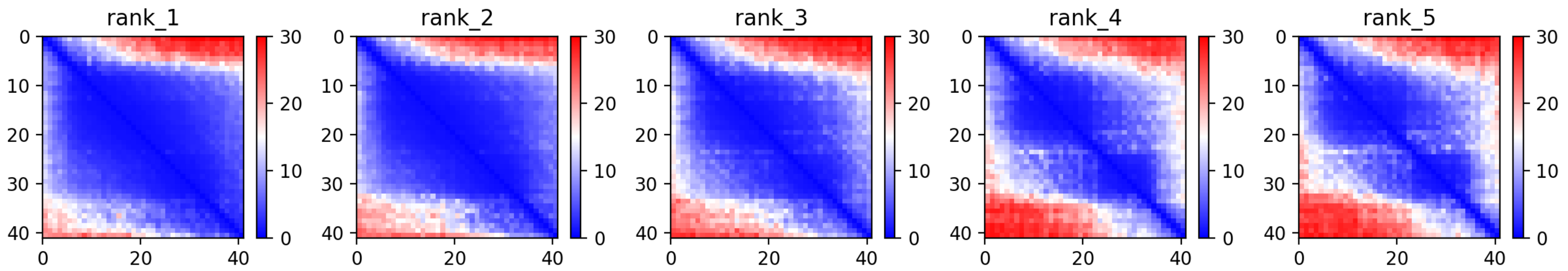

CP74: MtrunA17_Chr4g0034491

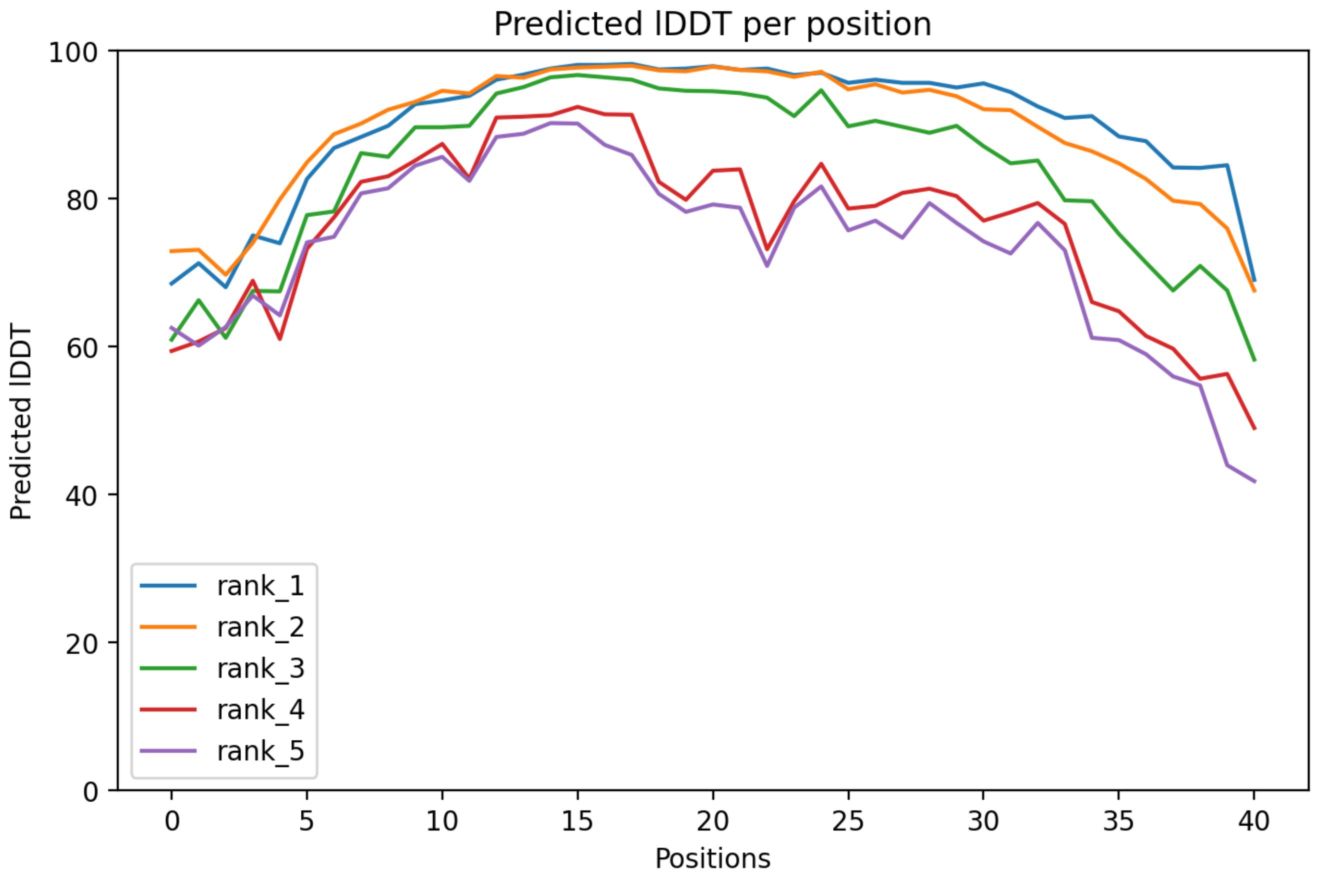

CP74: MtrunA17_Chr4g0034491

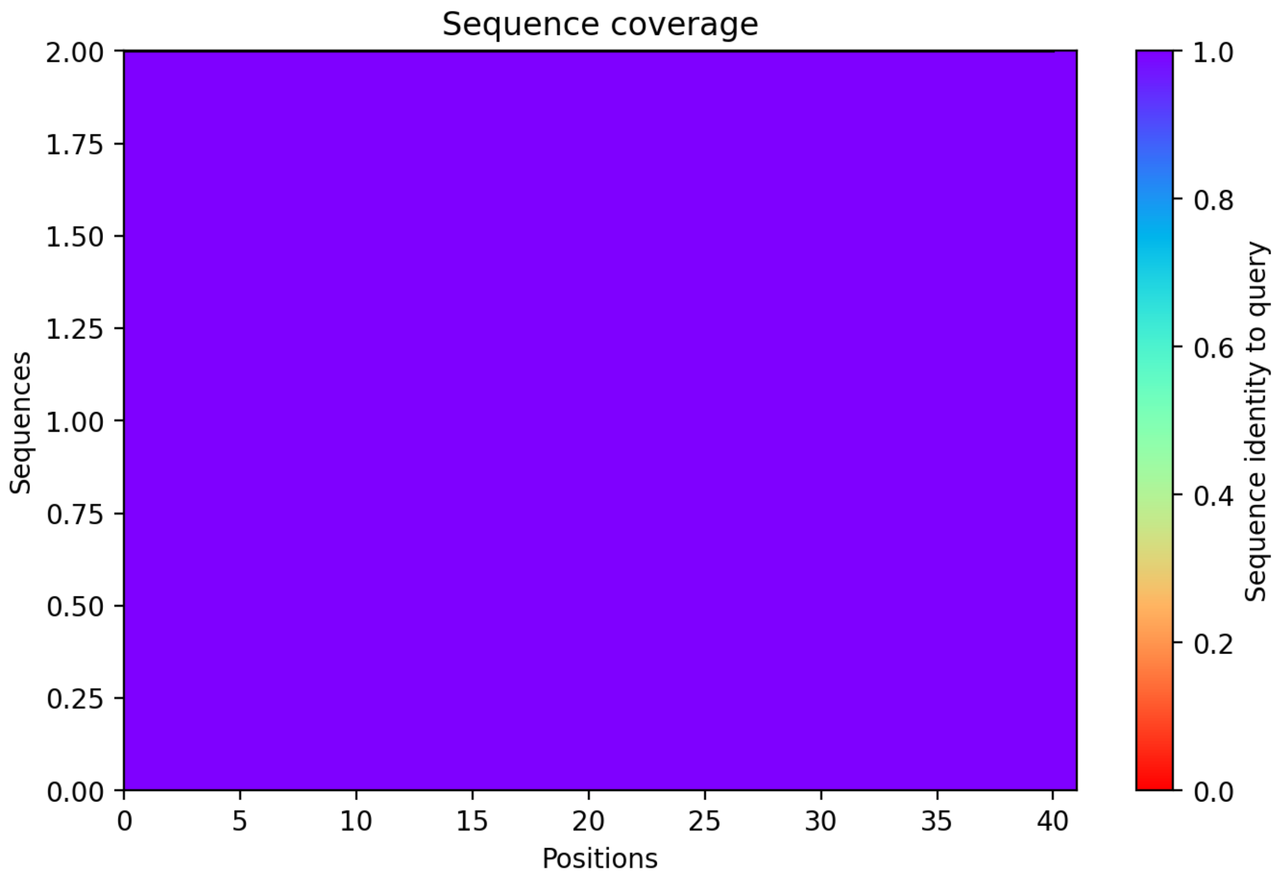

CP75: MtrunA17_Chr4g0037381

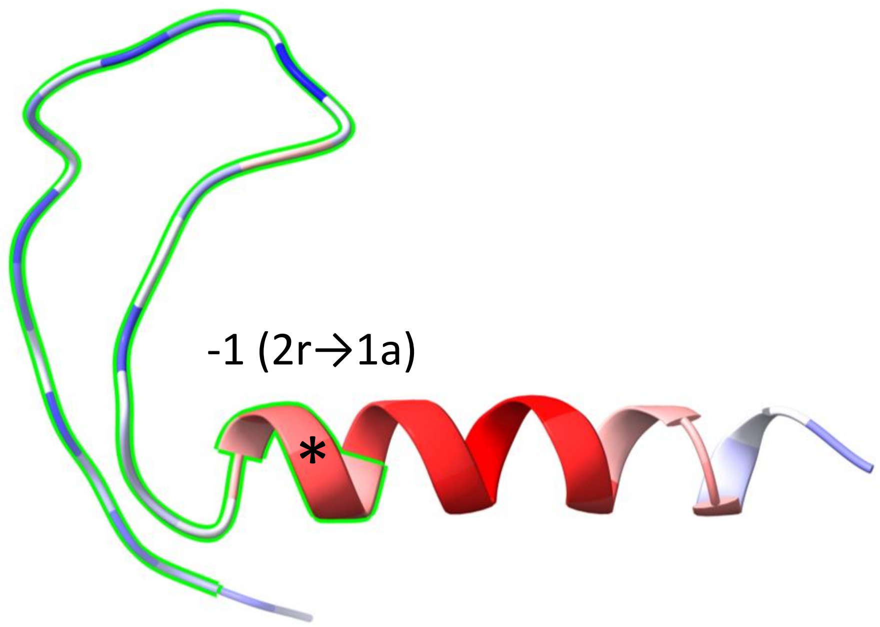

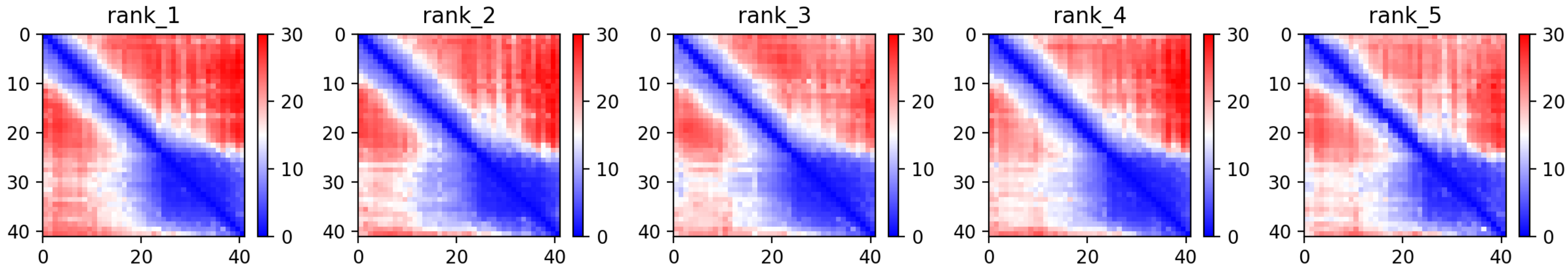

CP75: MtrunA17_Chr4g0037381

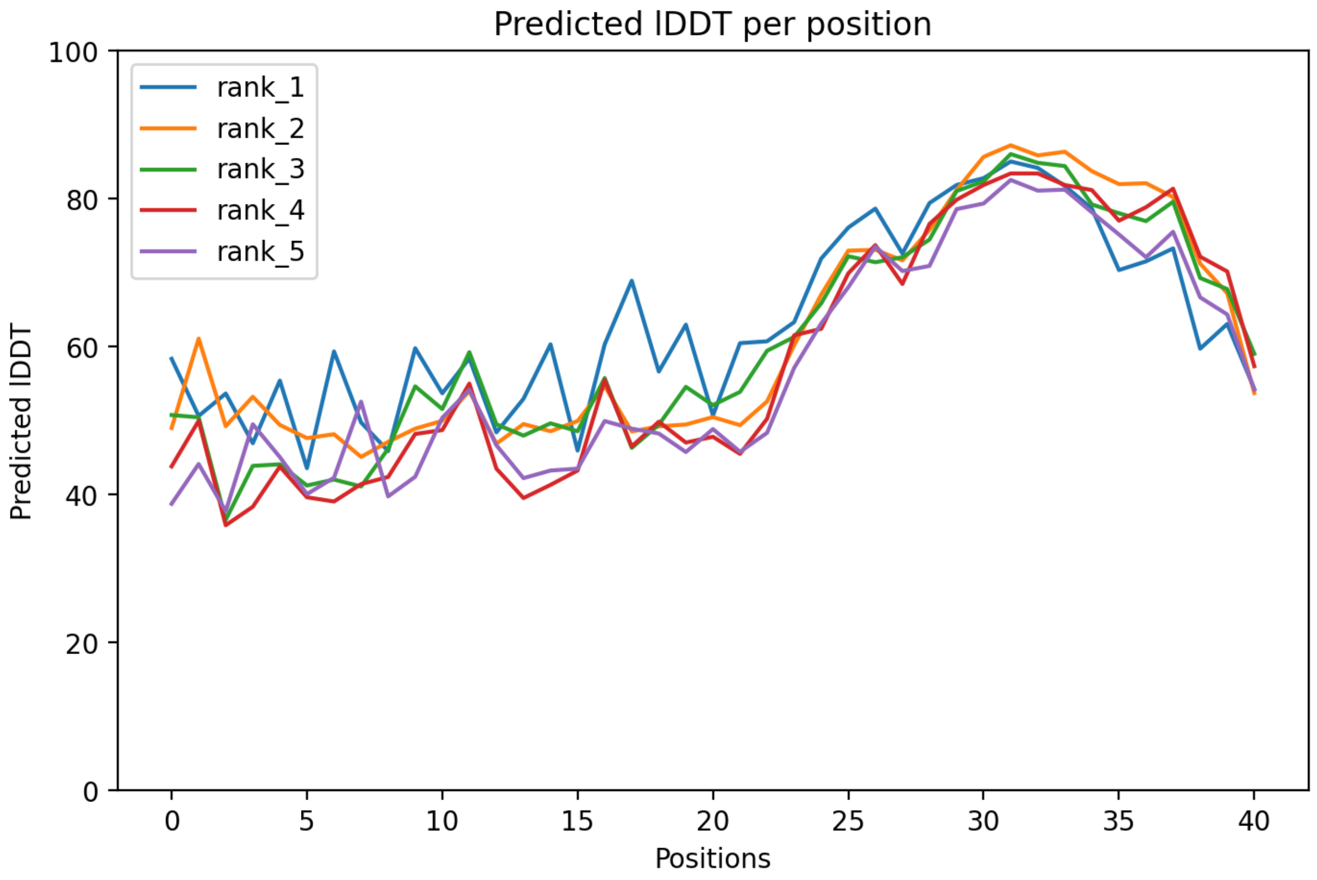

CP75: MtrunA17_Chr4g0037381

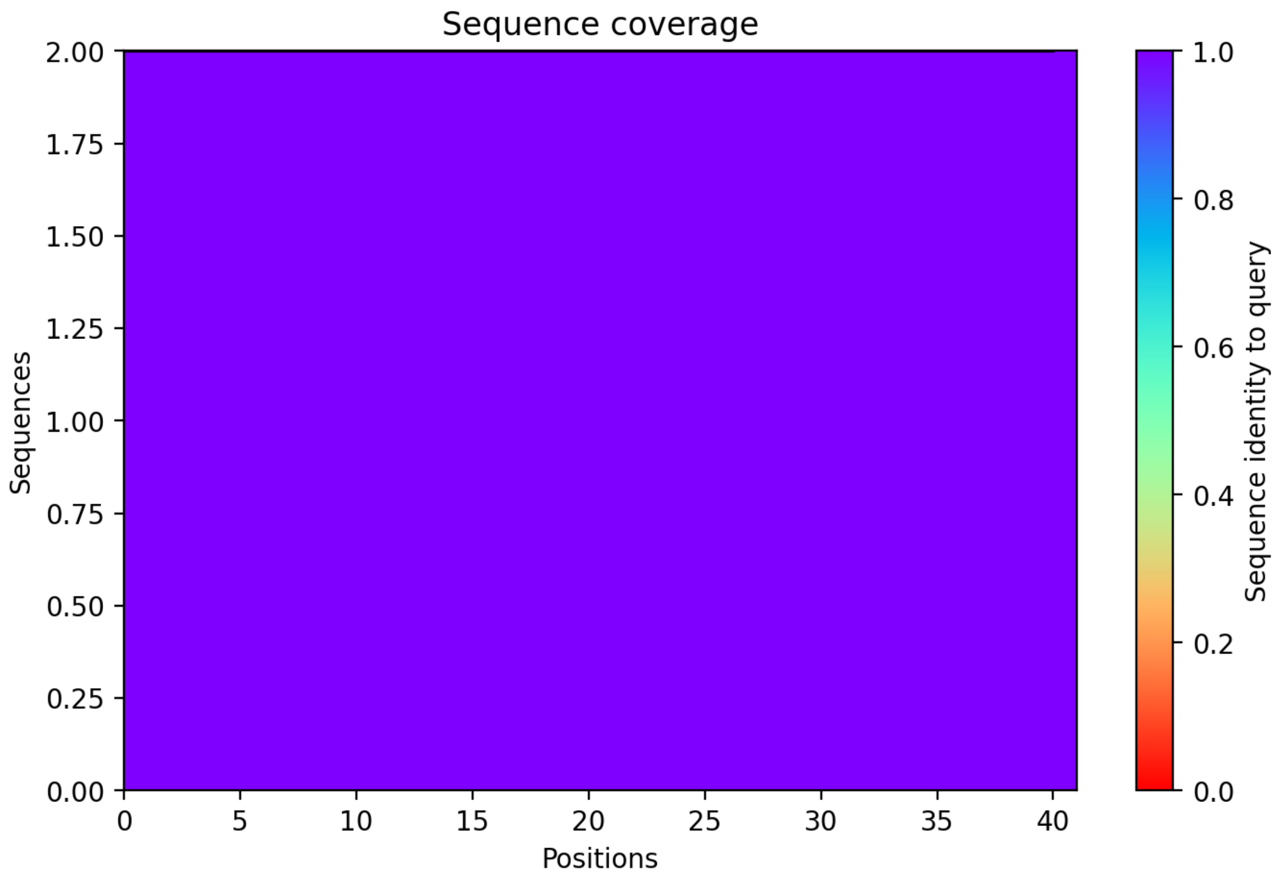

CP76: MtrunA17_Chr4g0040471

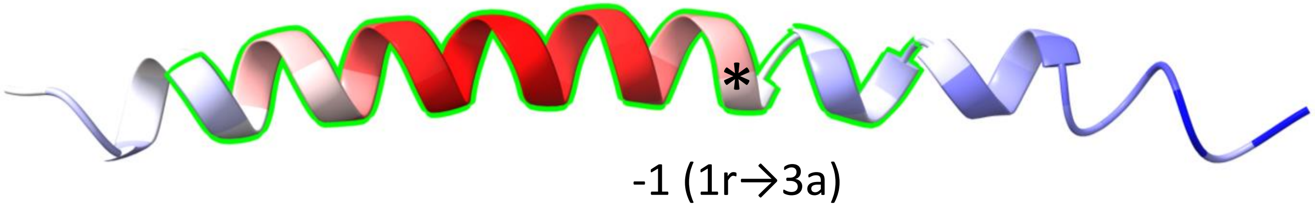

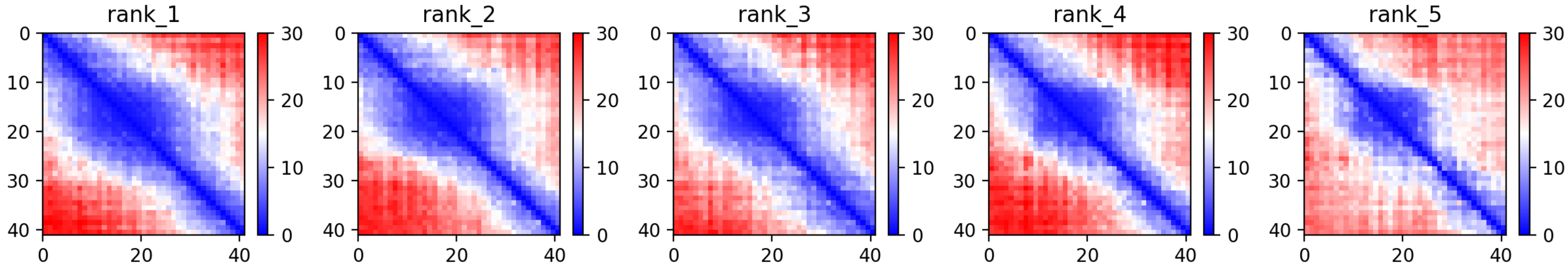

CP76: MtrunA17_Chr4g0040471

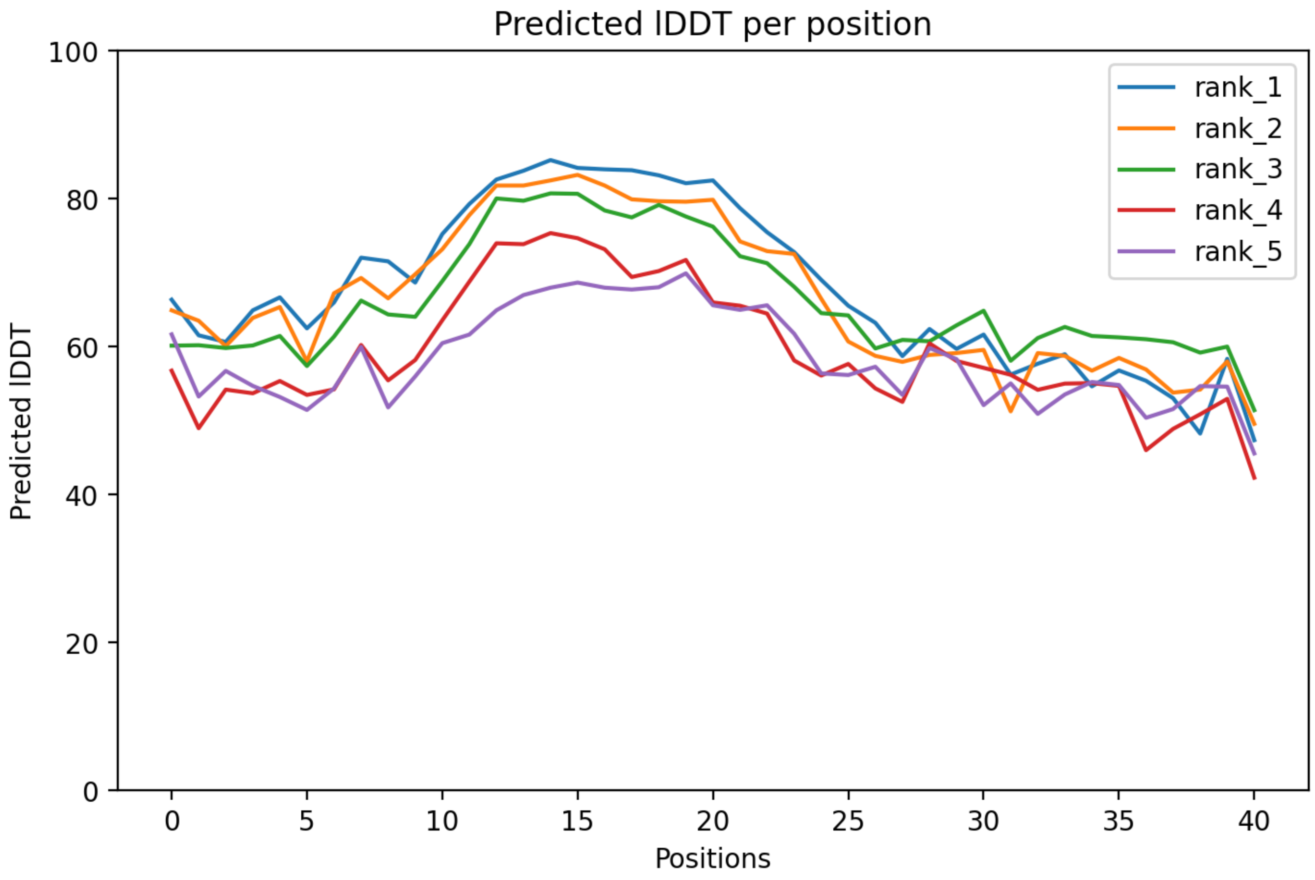

CP76: MtrunA17_Chr4g0040471

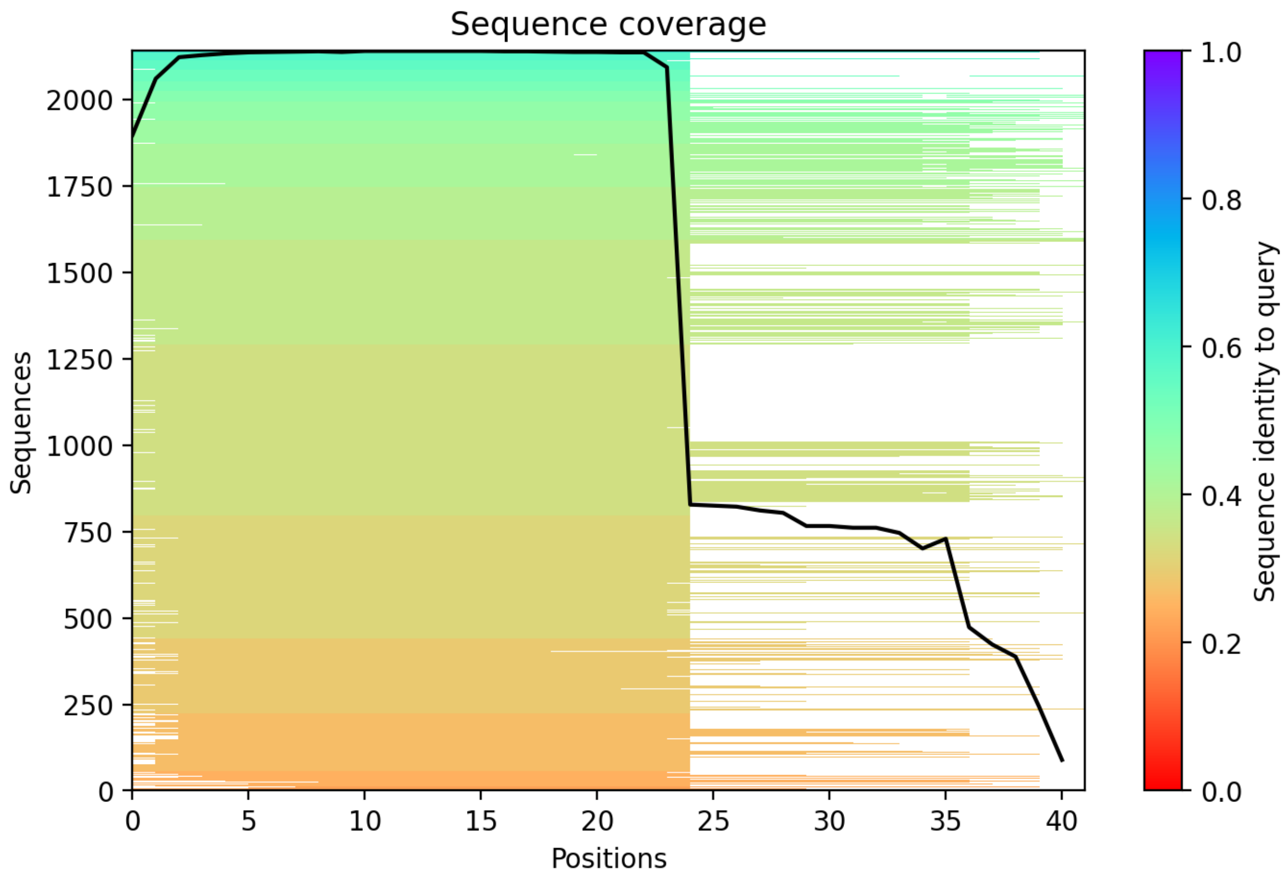

CP77: MtrunA17_Chr4g0055331

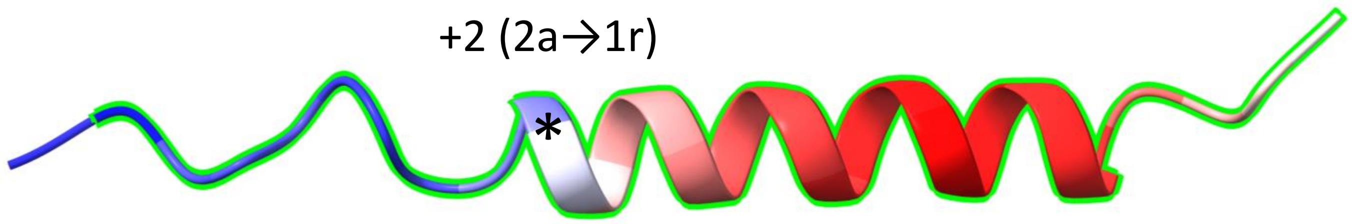

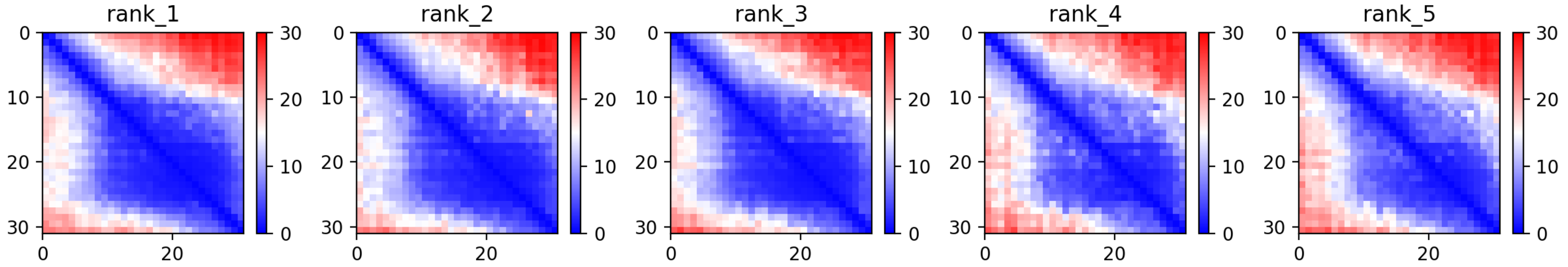

CP77: MtrunA17_Chr4g0055331

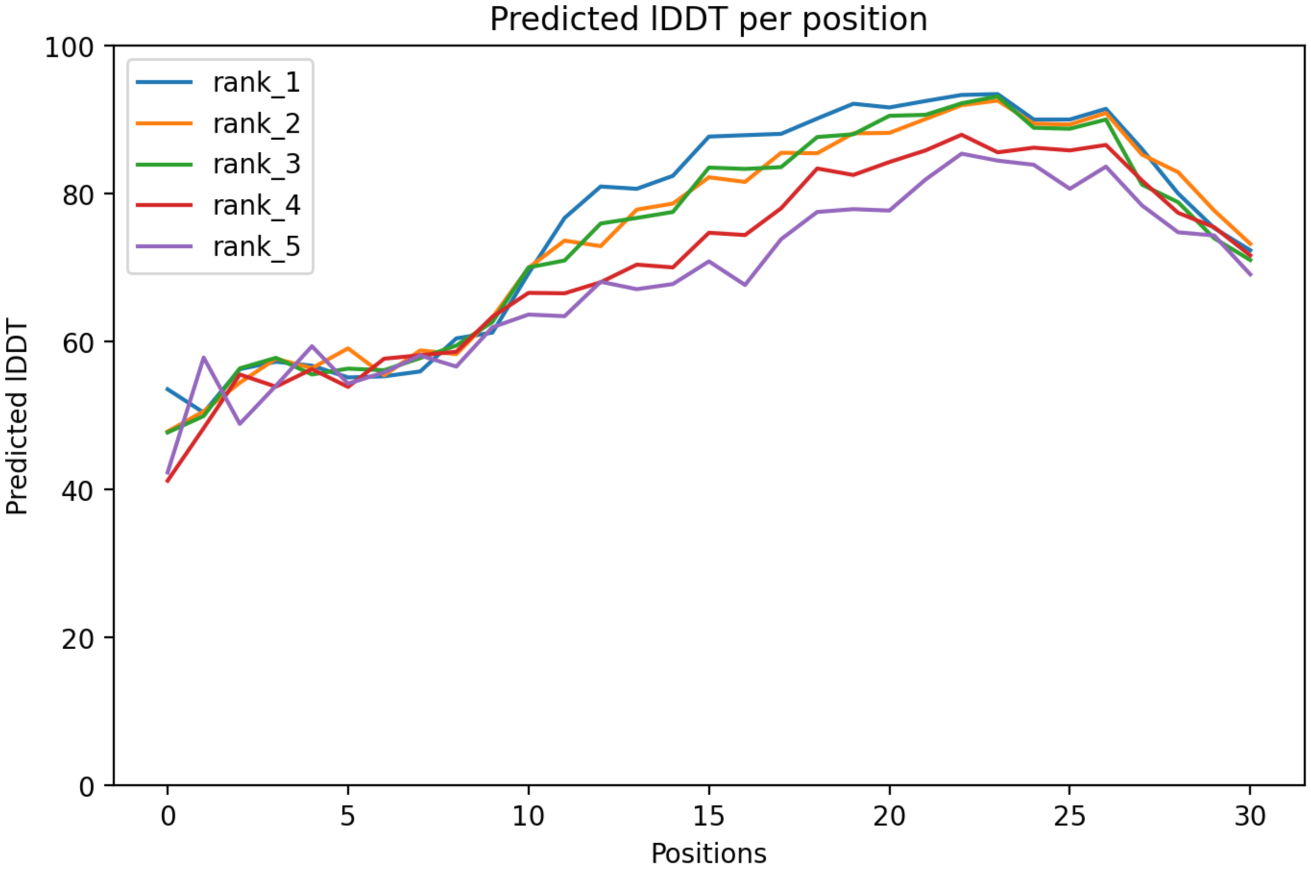

CP77: MtrunA17_Chr4g0055331

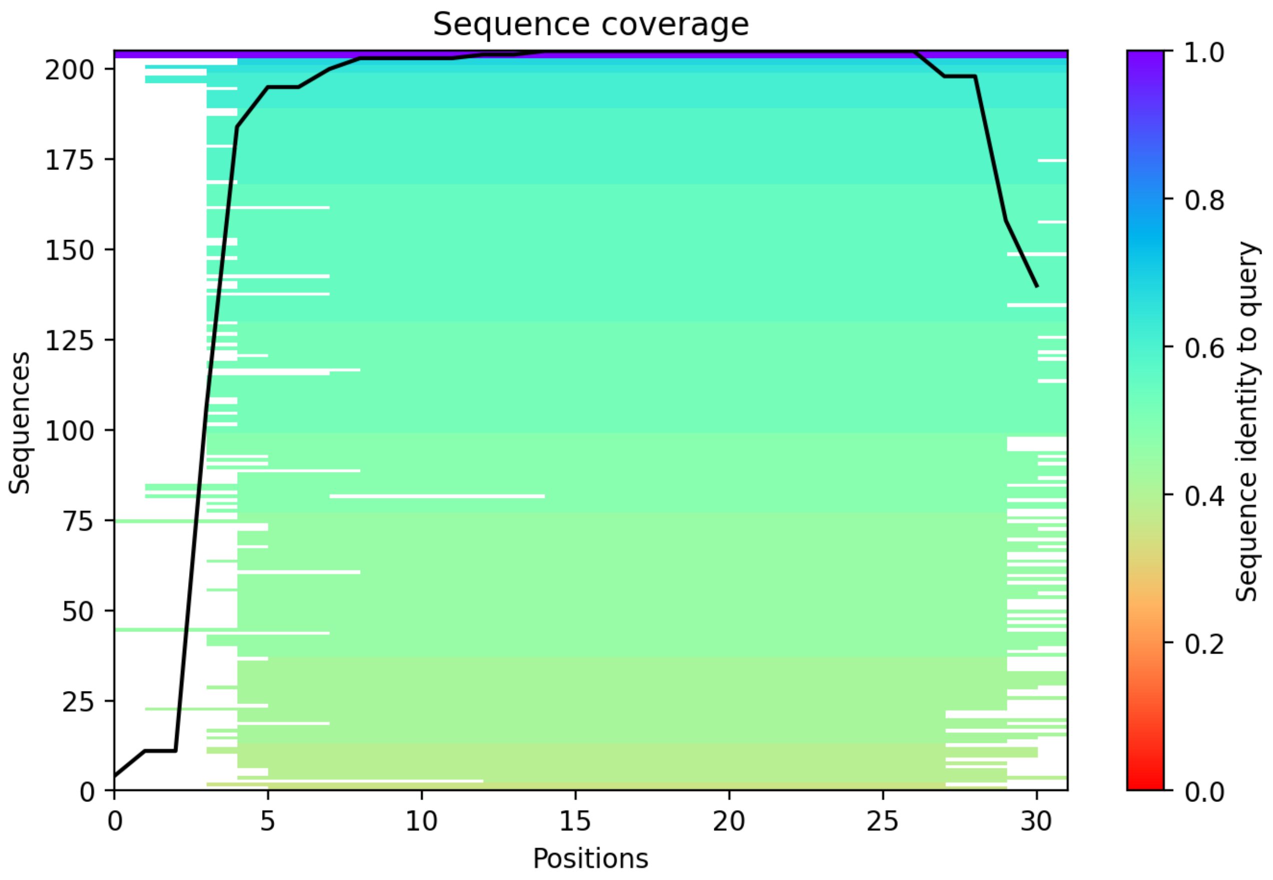

CP78: MtrunA17_Chr4g0059001

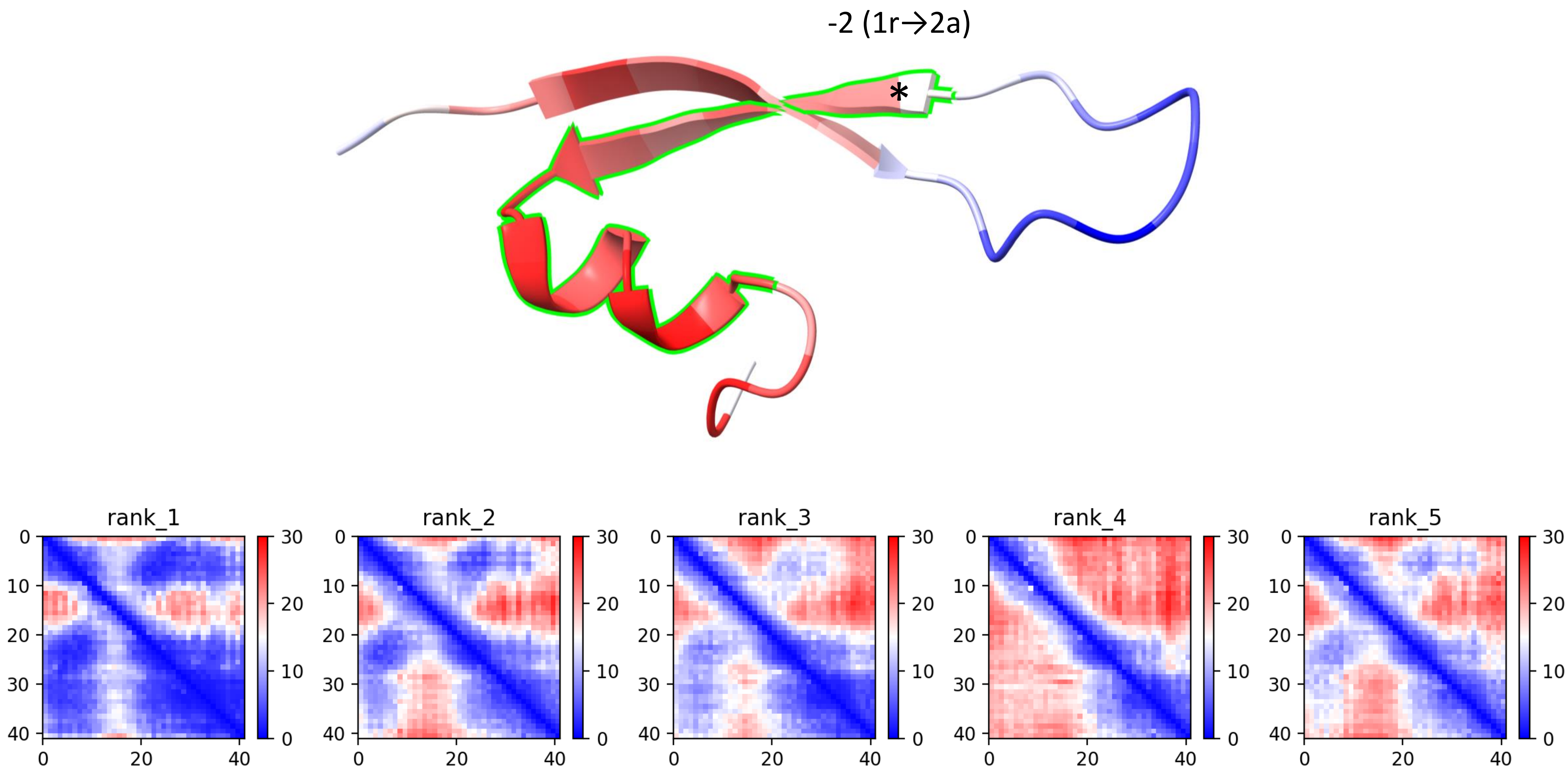

CP78: MtrunA17_Chr4g0059001

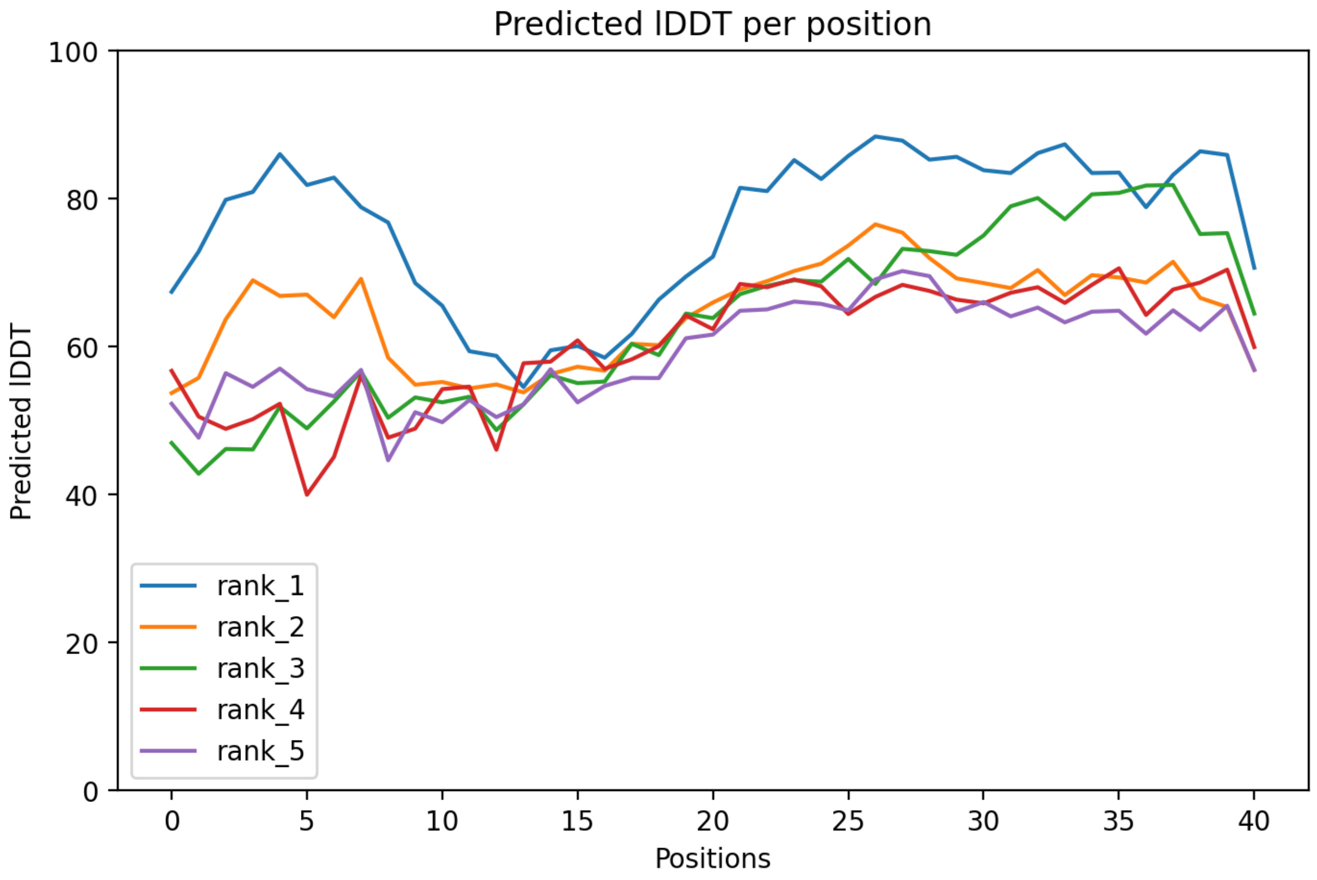

CP78: MtrunA17_Chr4g0059001

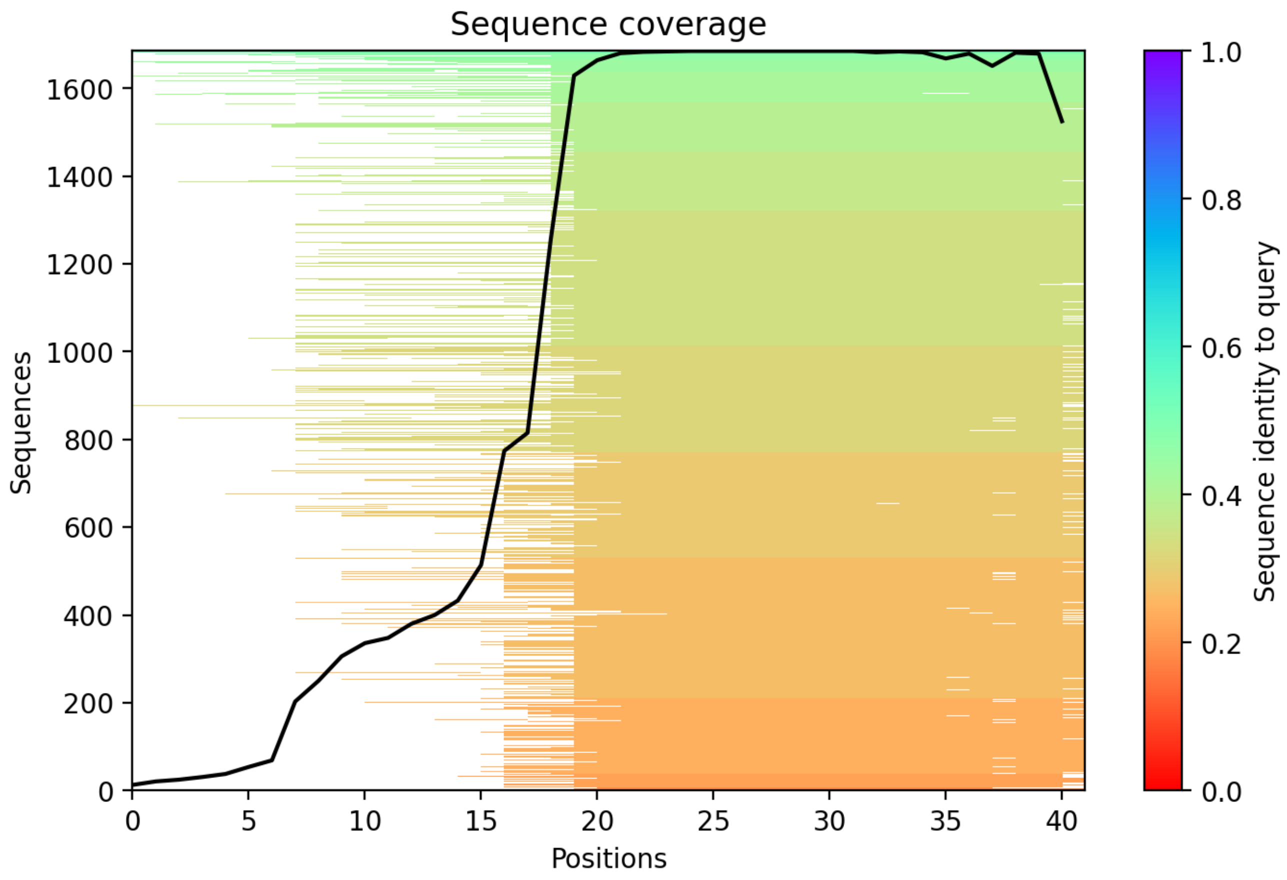

CP79: MtrunA17_Chr4g0063201

-2 (1r→2a)

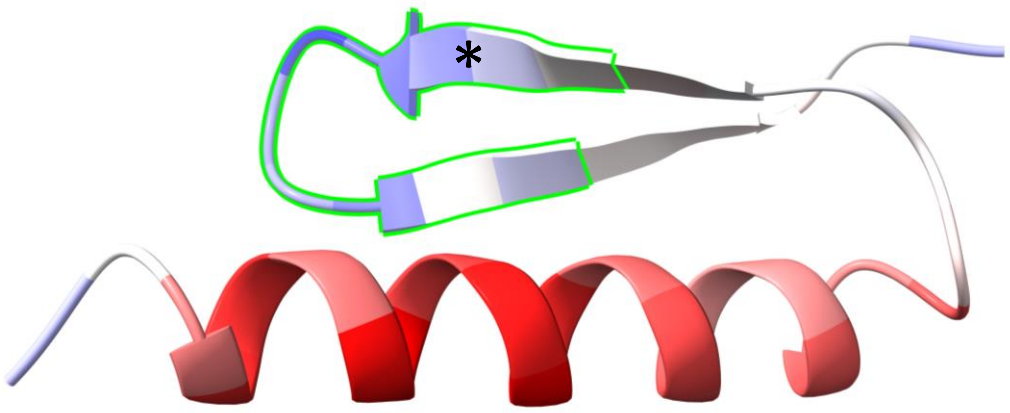

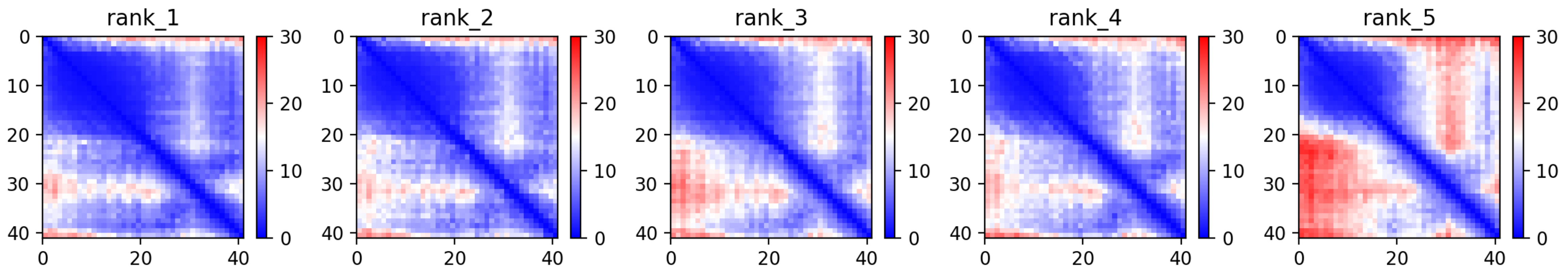

CP79: MtrunA17_Chr4g0063201

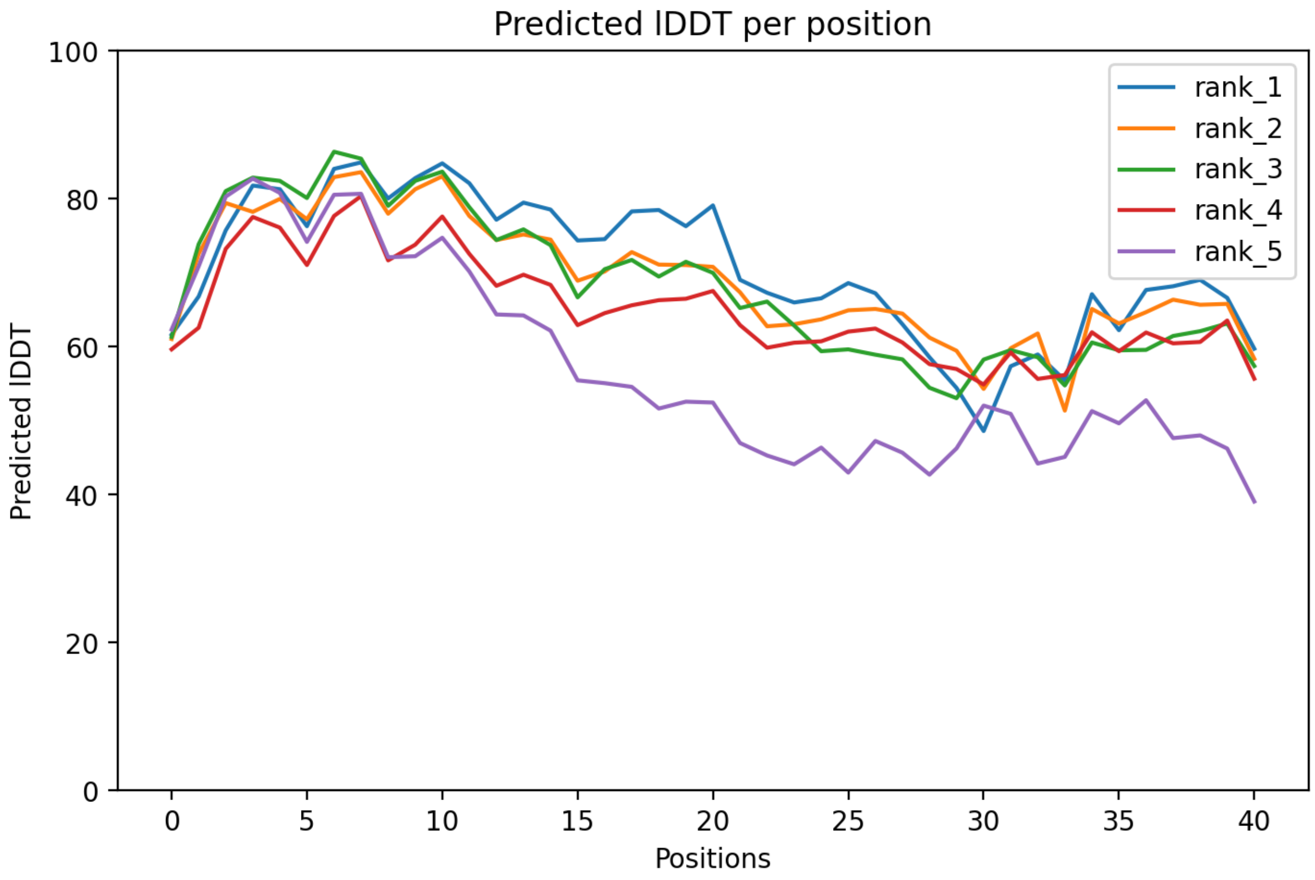

CP79: MtrunA17_Chr4g0063201

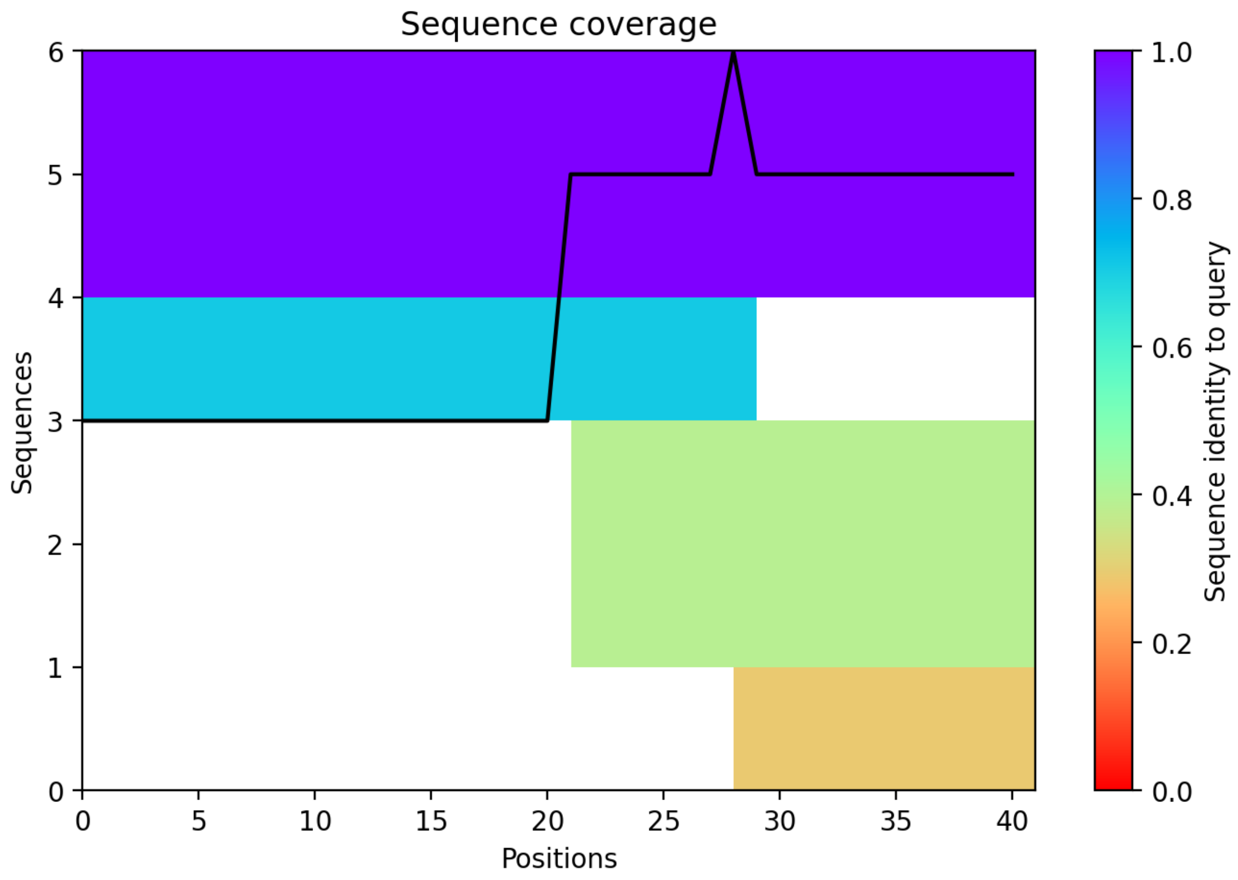

CP80: MtrunA17_Chr4g0070011

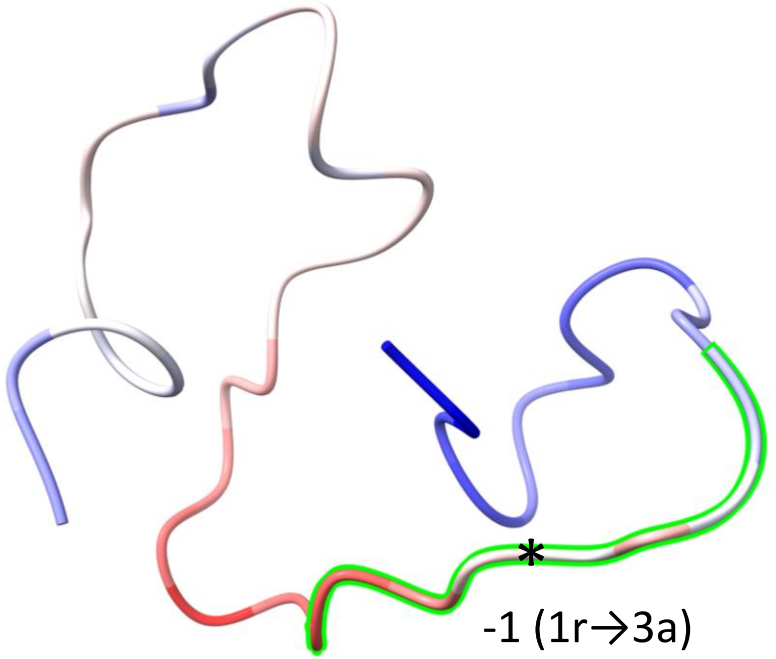

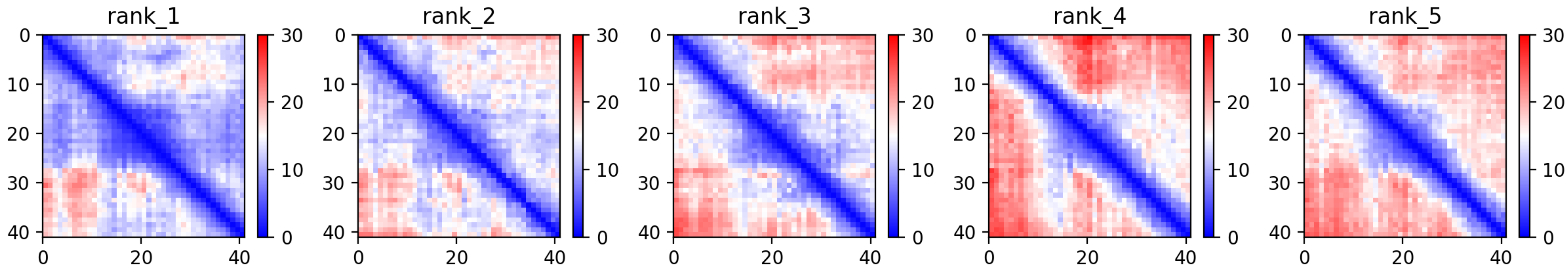

CP80: MtrunA17_Chr4g0070011

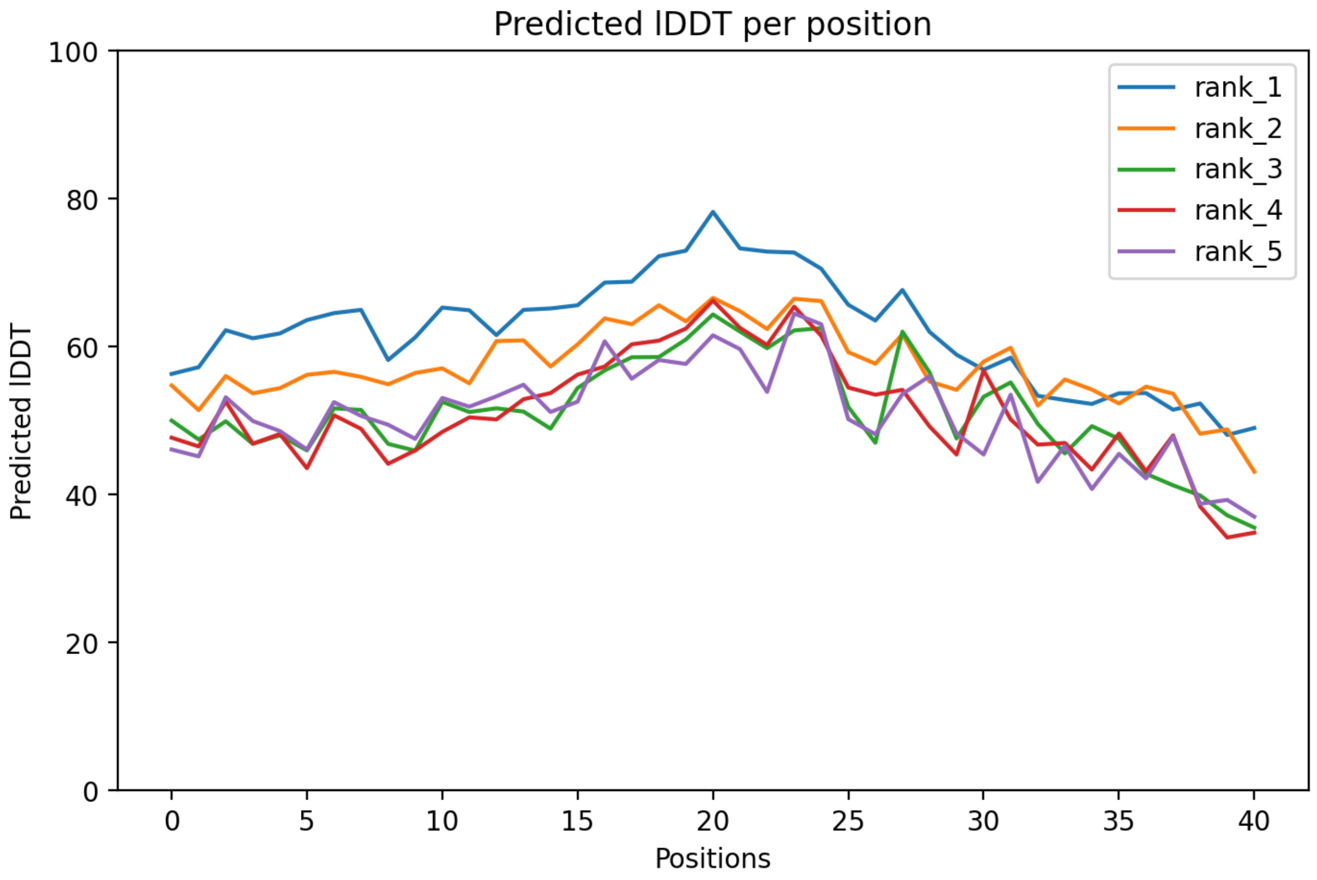

CP80: MtrunA17_Chr4g0070011

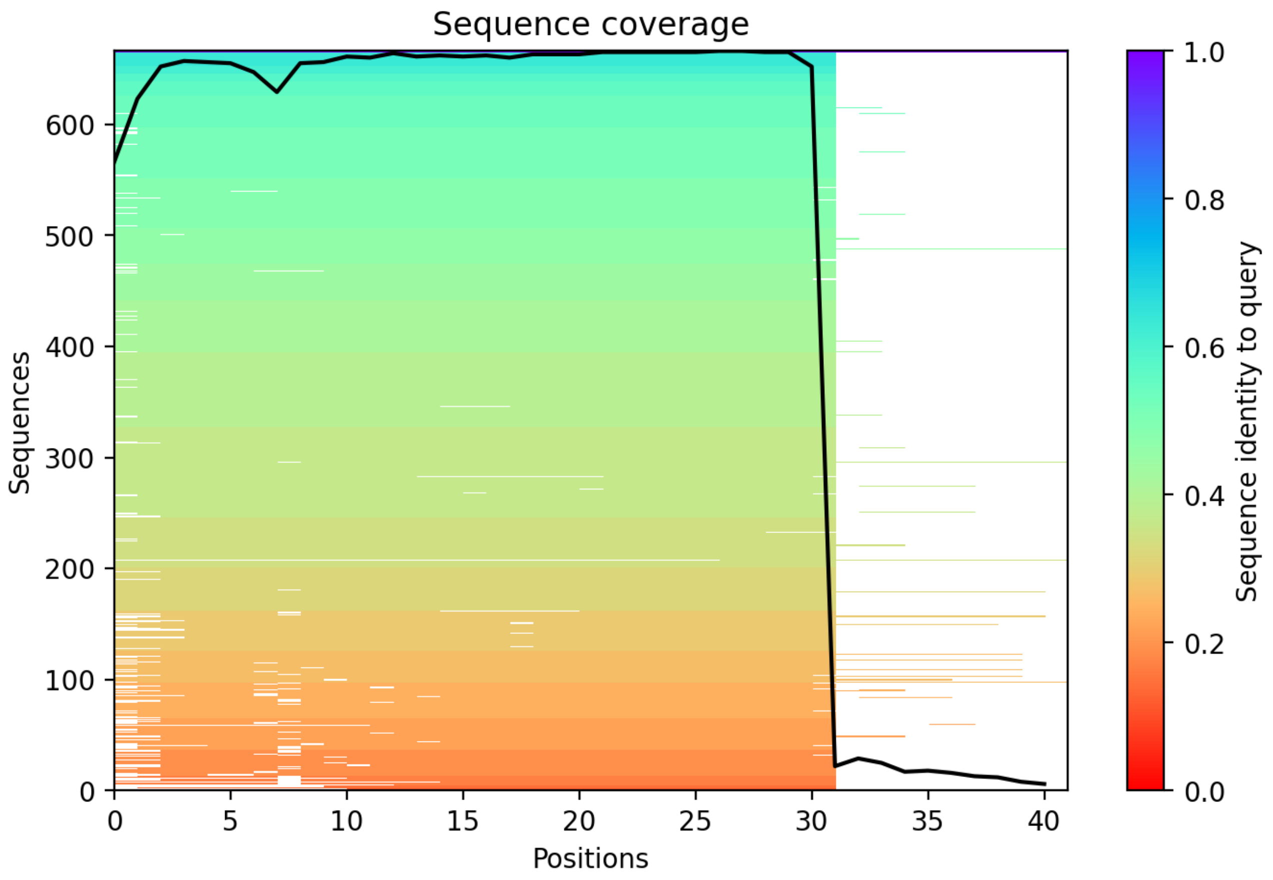

CP81: MtrunA17_Chr5g0393401

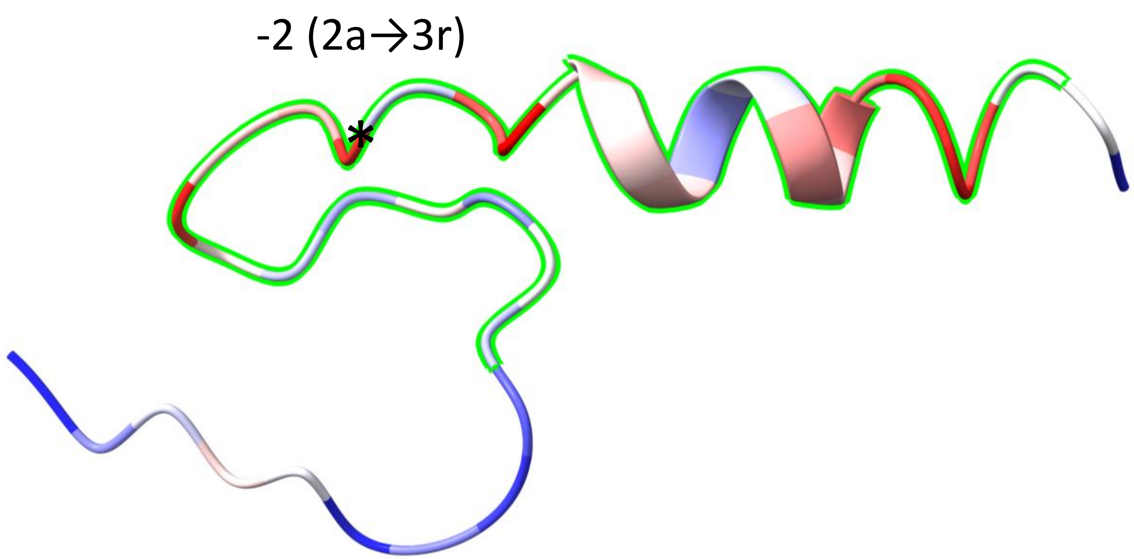

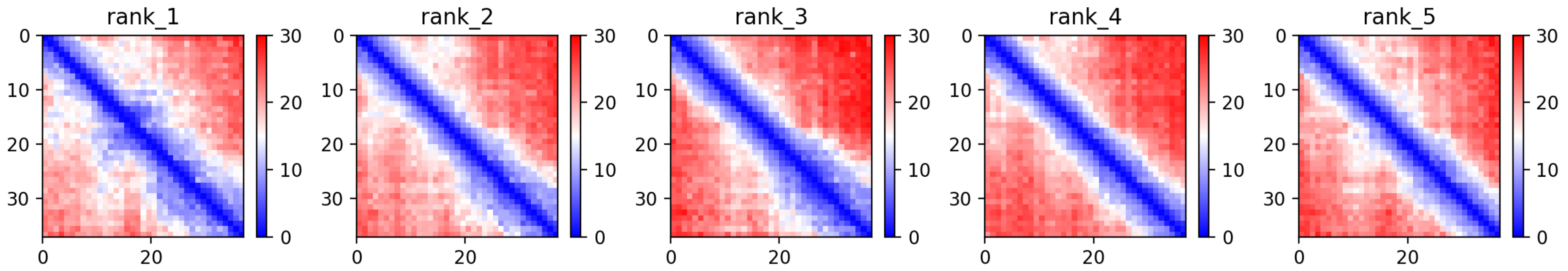

CP81: MtrunA17_Chr5g0393401

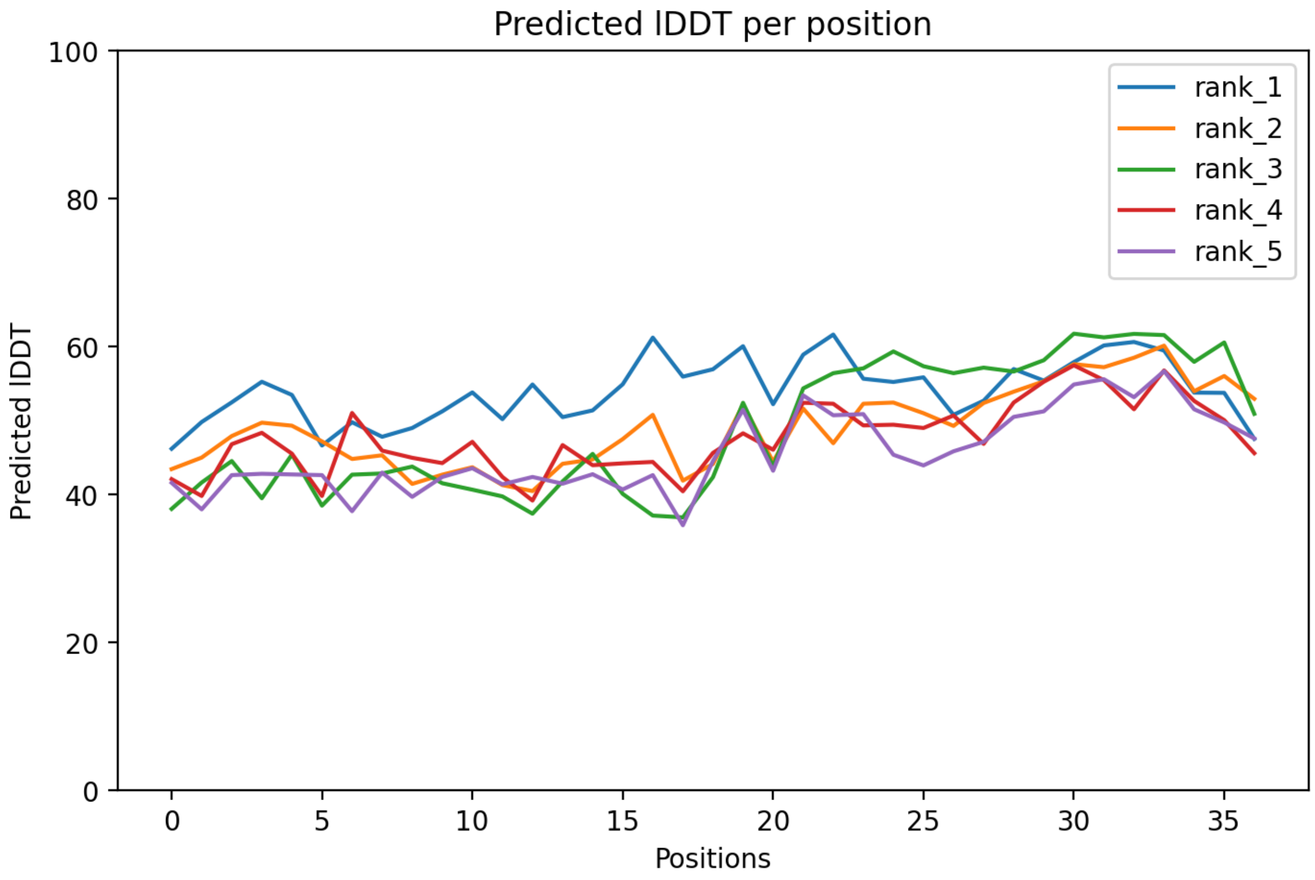

CP81: MtrunA17_Chr5g0393401

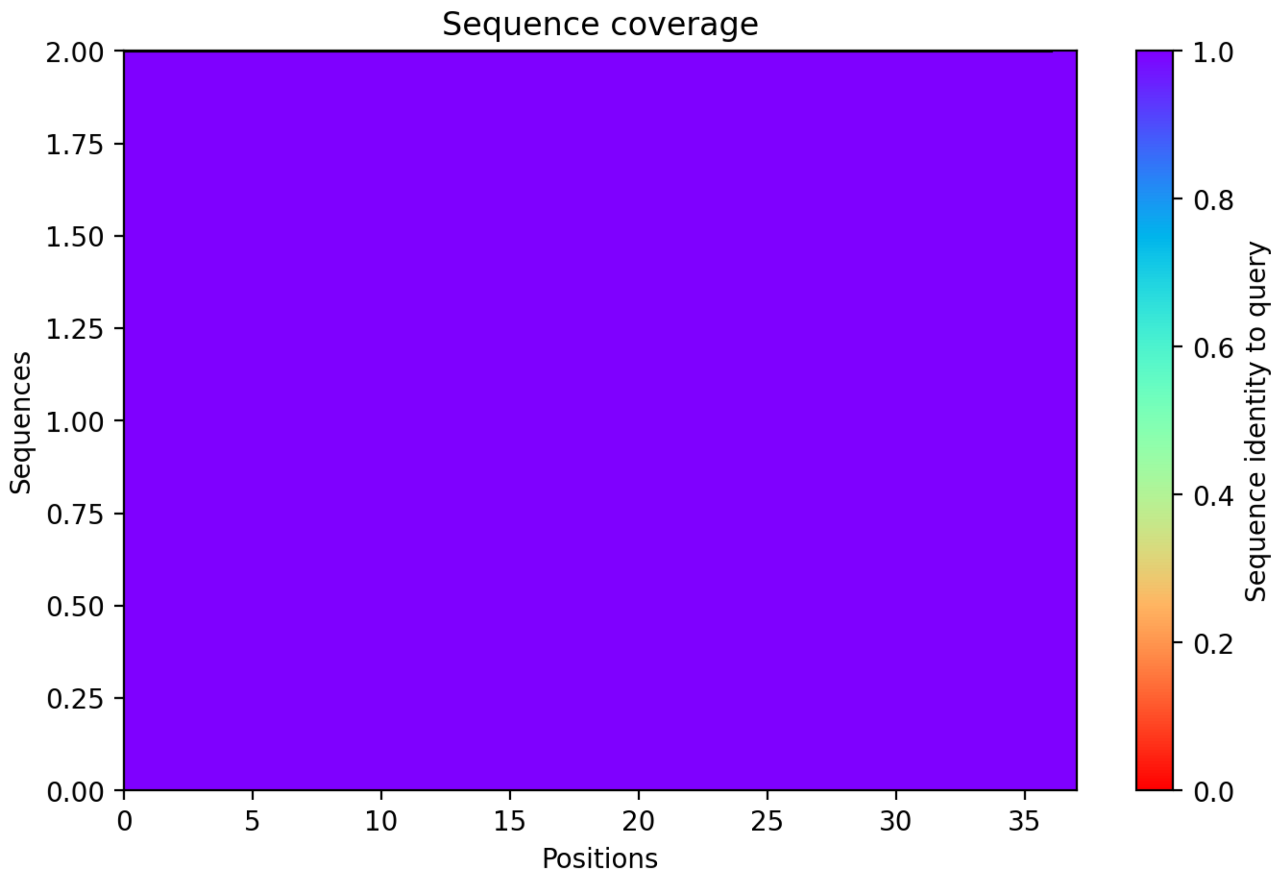

CP82: MtrunA17_Chr5g0400181

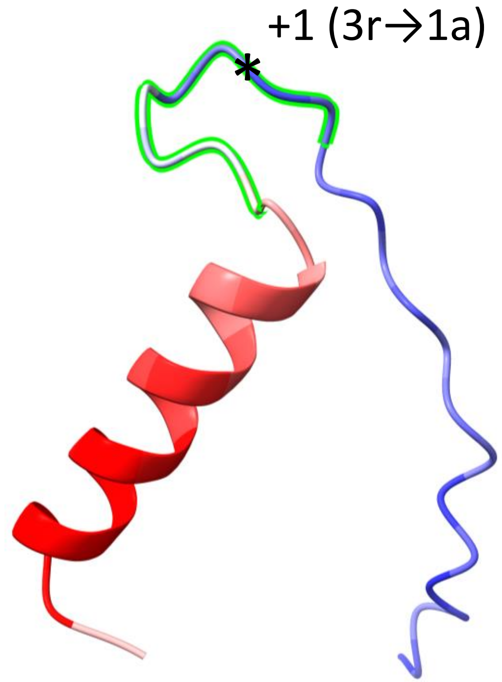

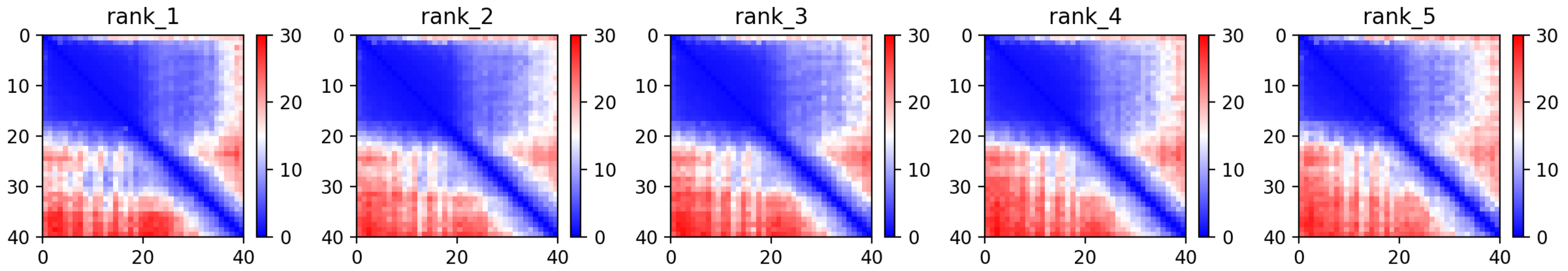

CP82: MtrunA17_Chr5g0400181

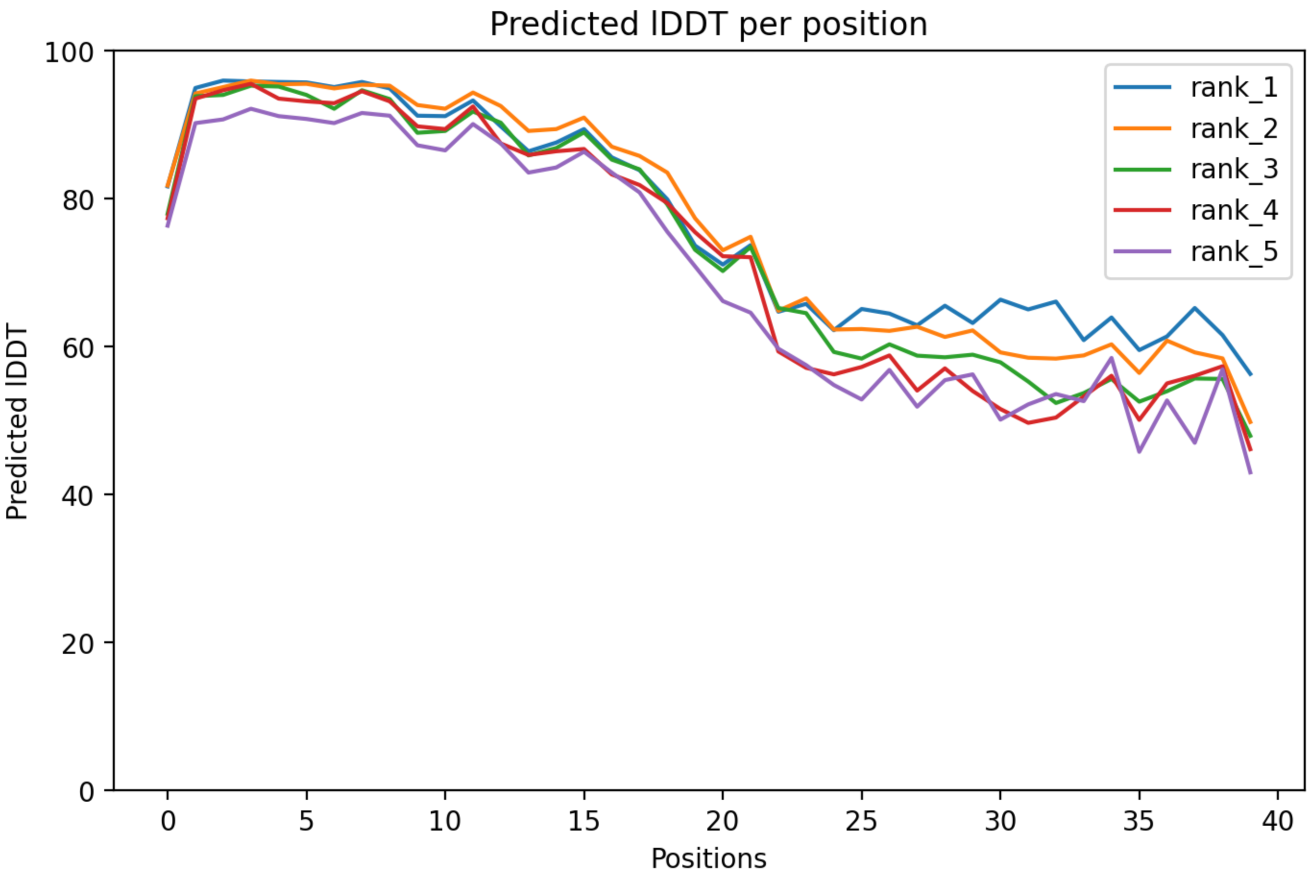

CP82: MtrunA17_Chr5g0400181

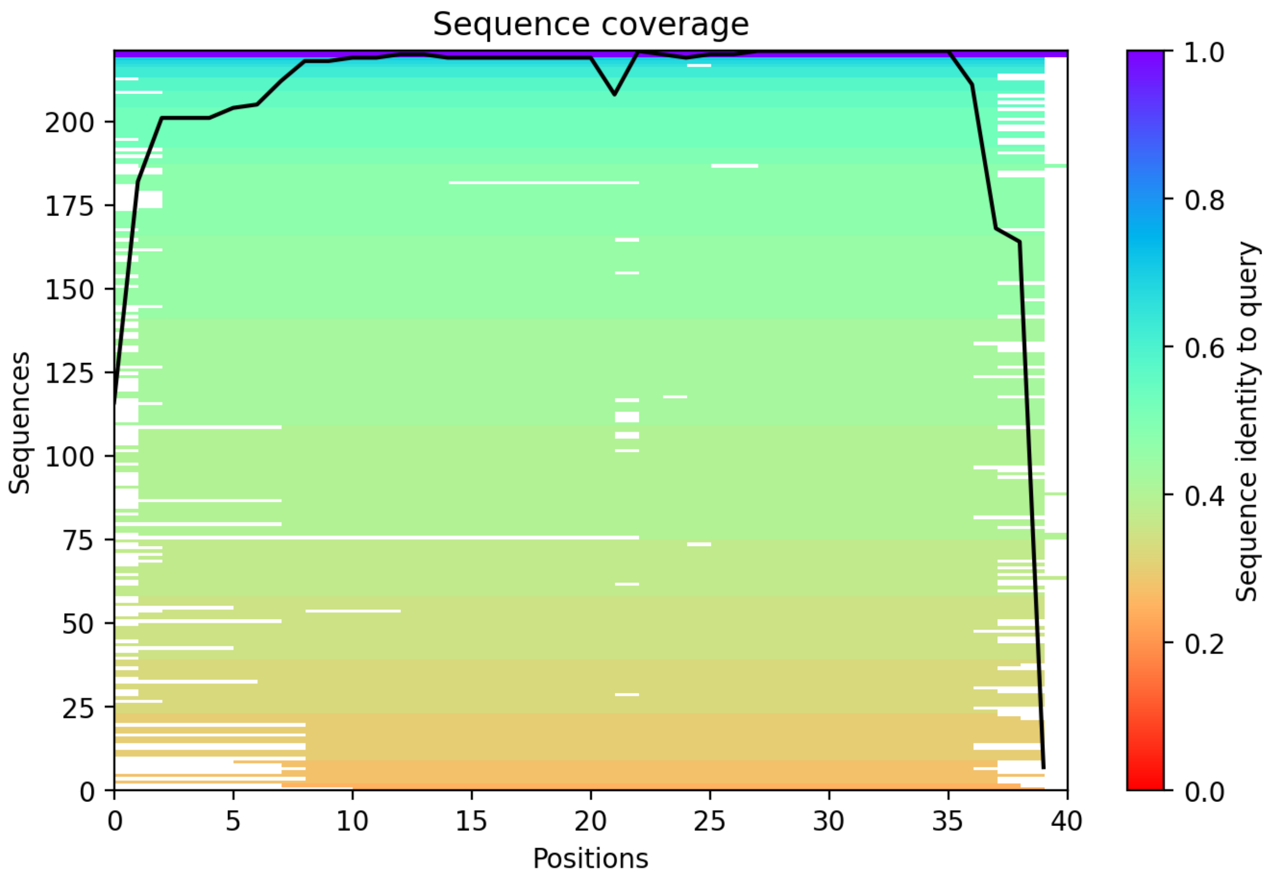

CP83: MtrunA17_Chr5g0405061

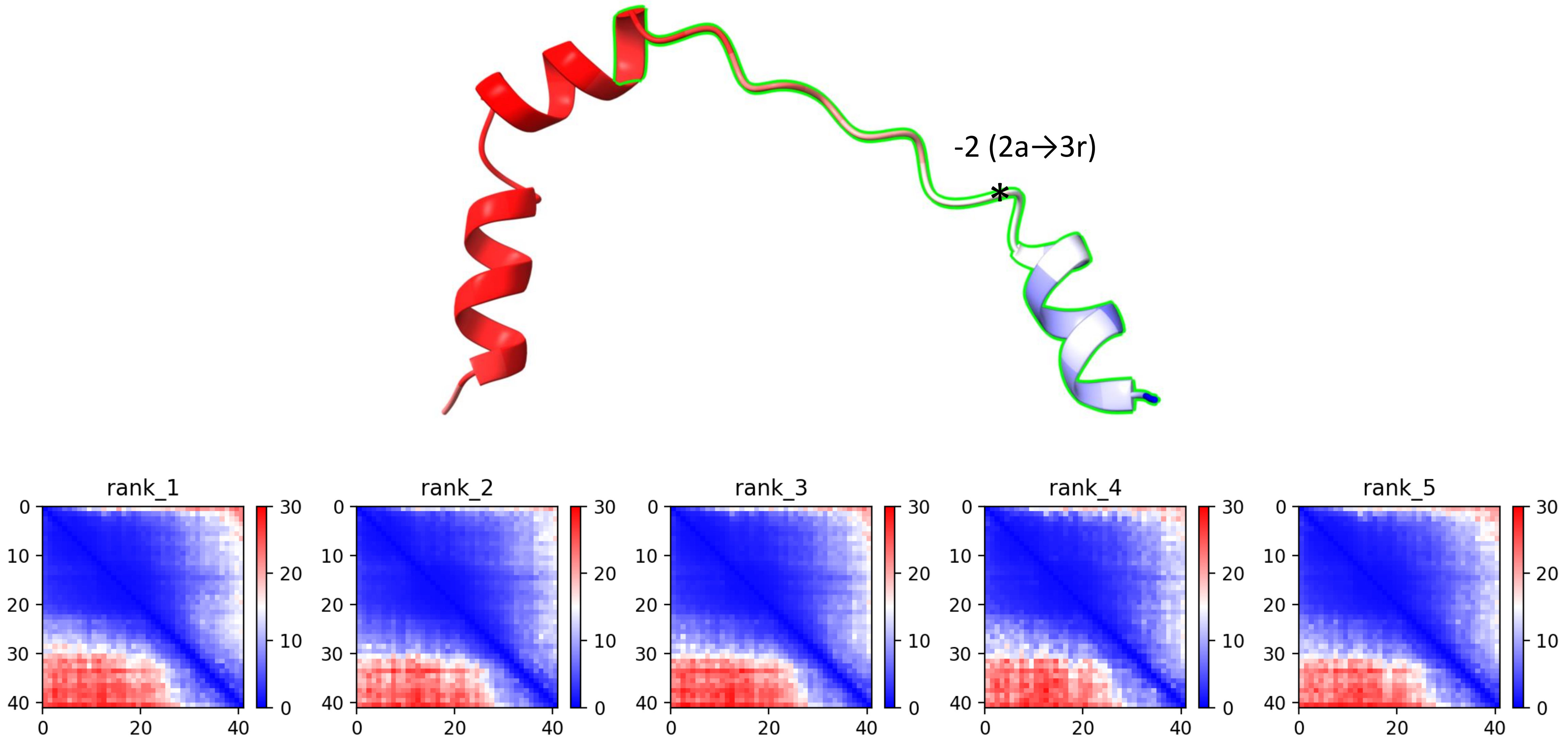

CP83: MtrunA17_Chr5g0405061

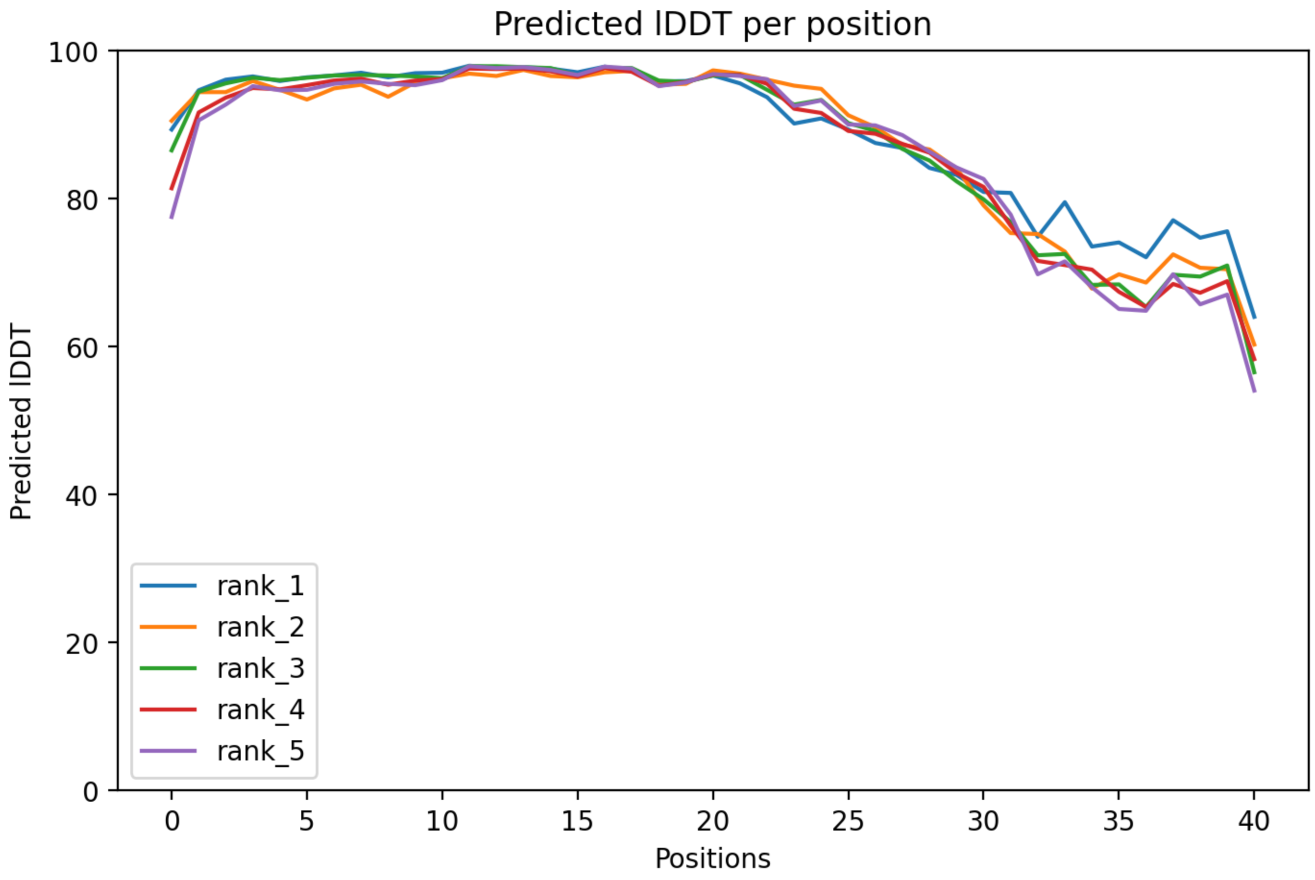

CP83: MtrunA17_Chr5g0405061

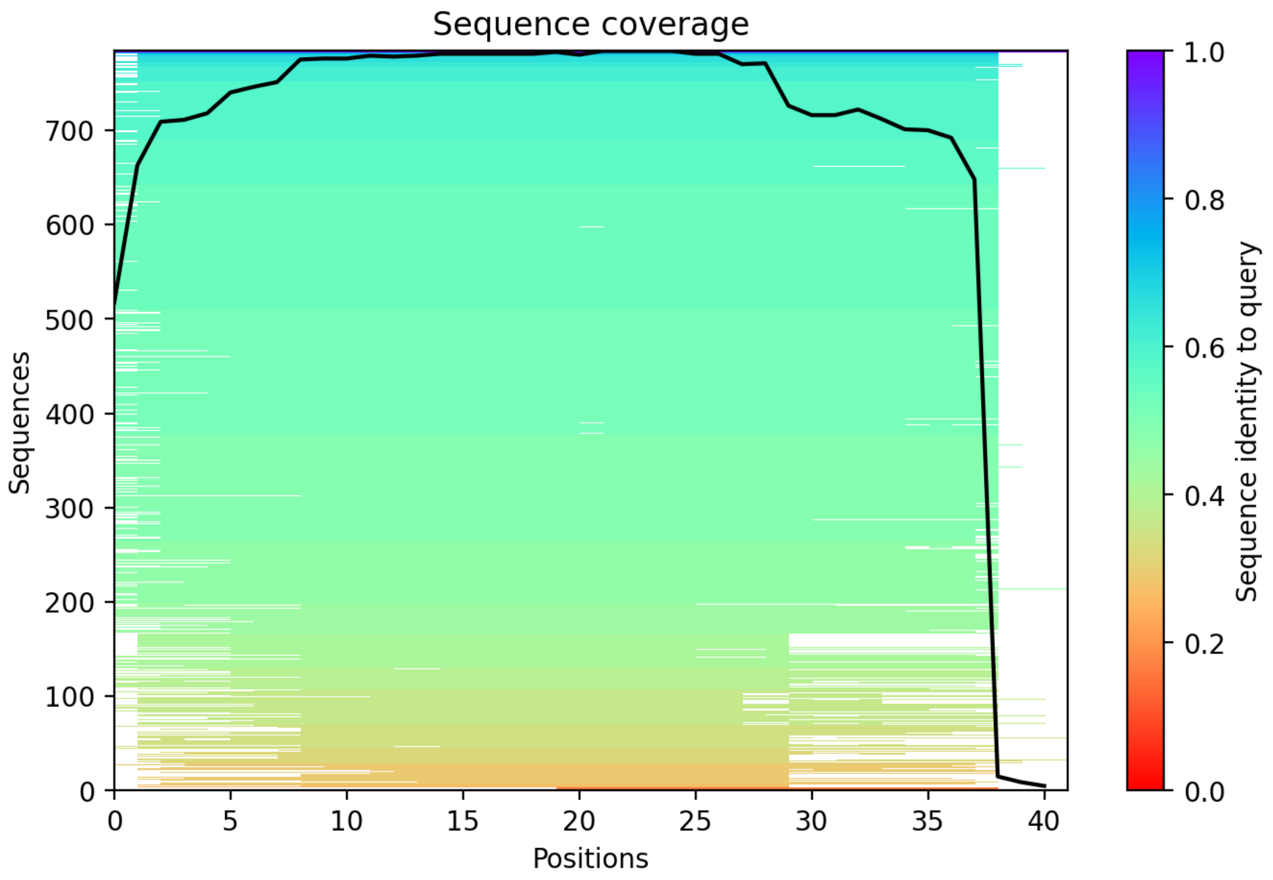

CP84: MtrunA17_Chr5g0408581

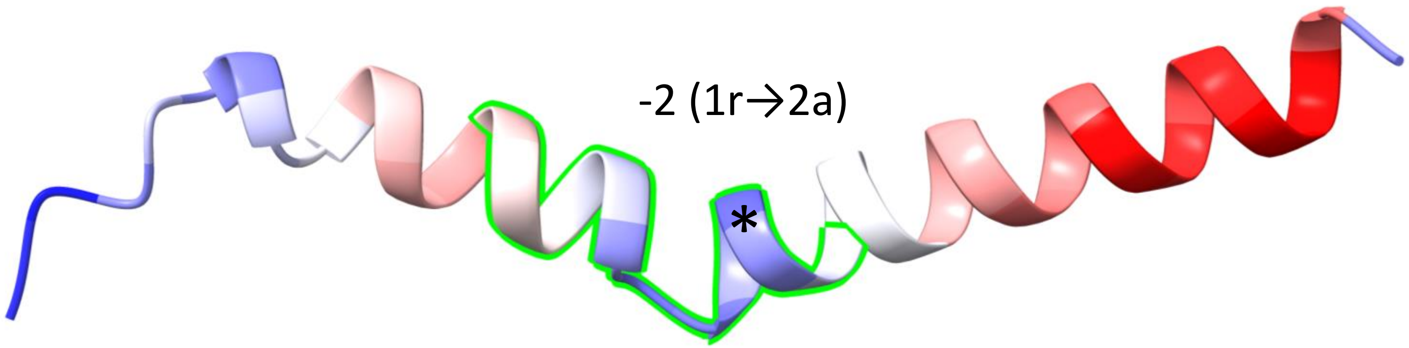

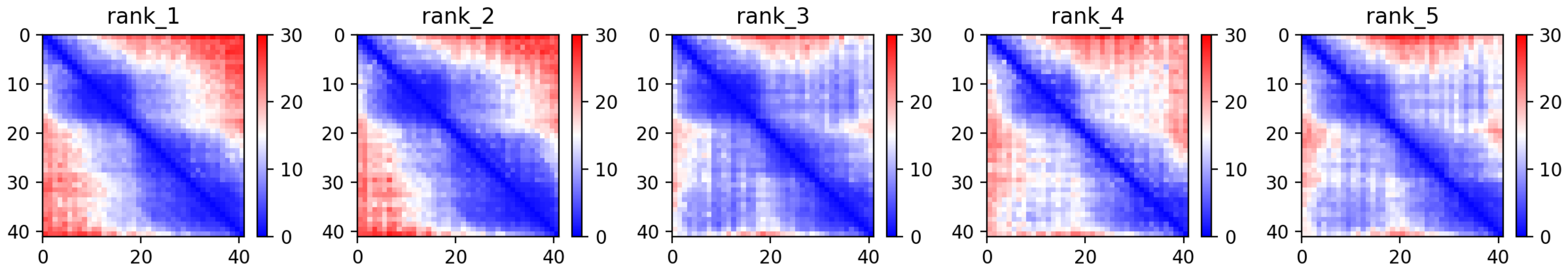

CP84: MtrunA17_Chr5g0408581

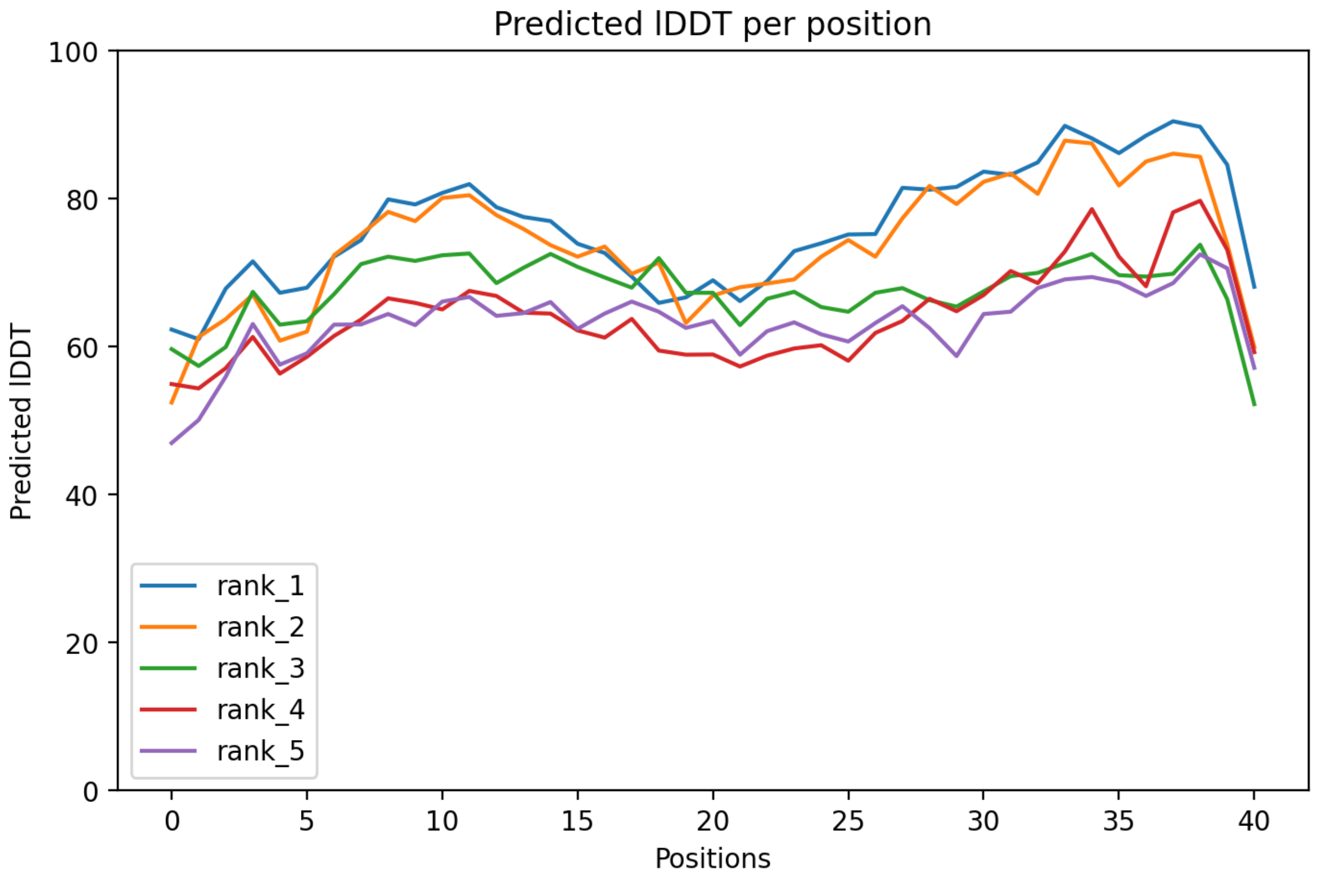

CP84: MtrunA17_Chr5g0408581

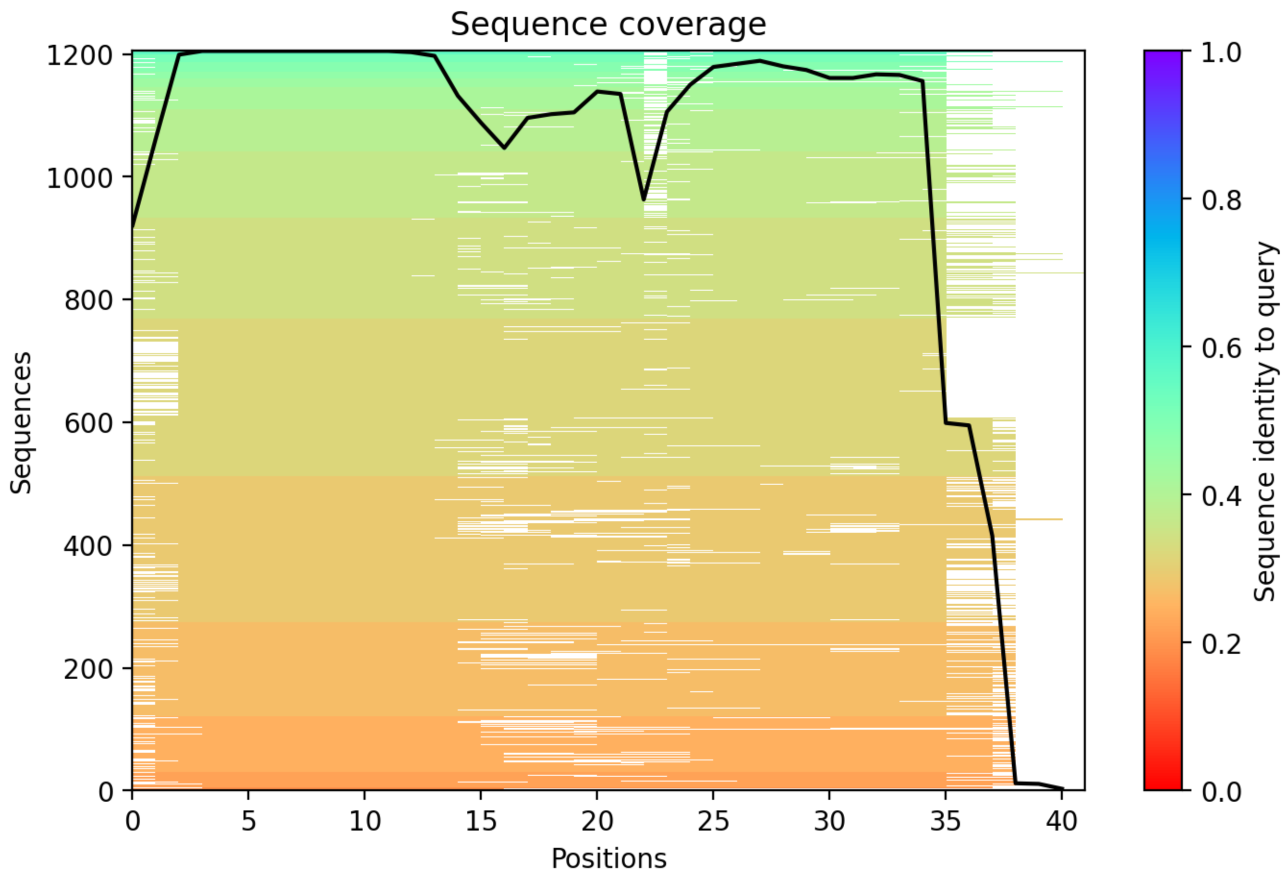

CP85: MtrunA17_Chr5g0415031

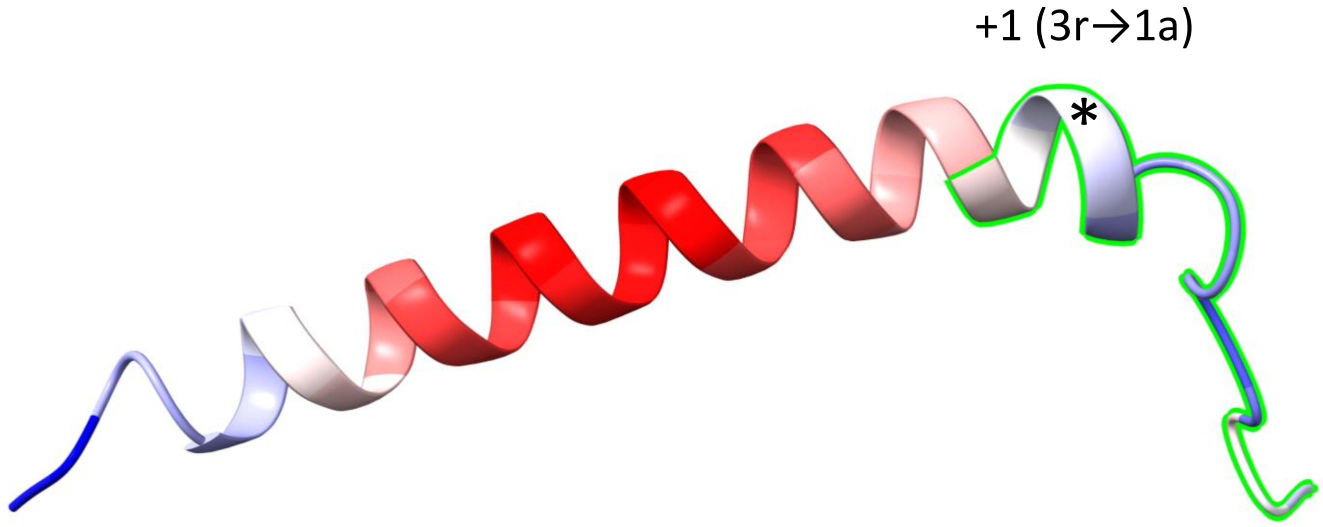

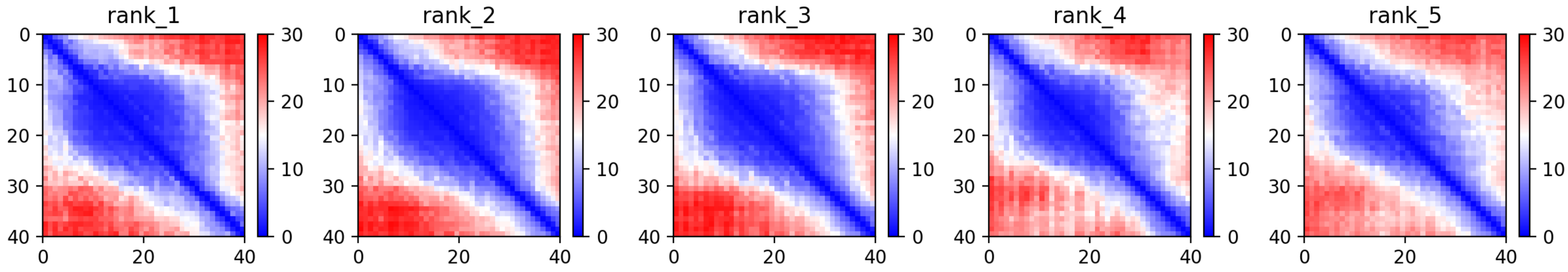

CP85: MtrunA17_Chr5g0415031

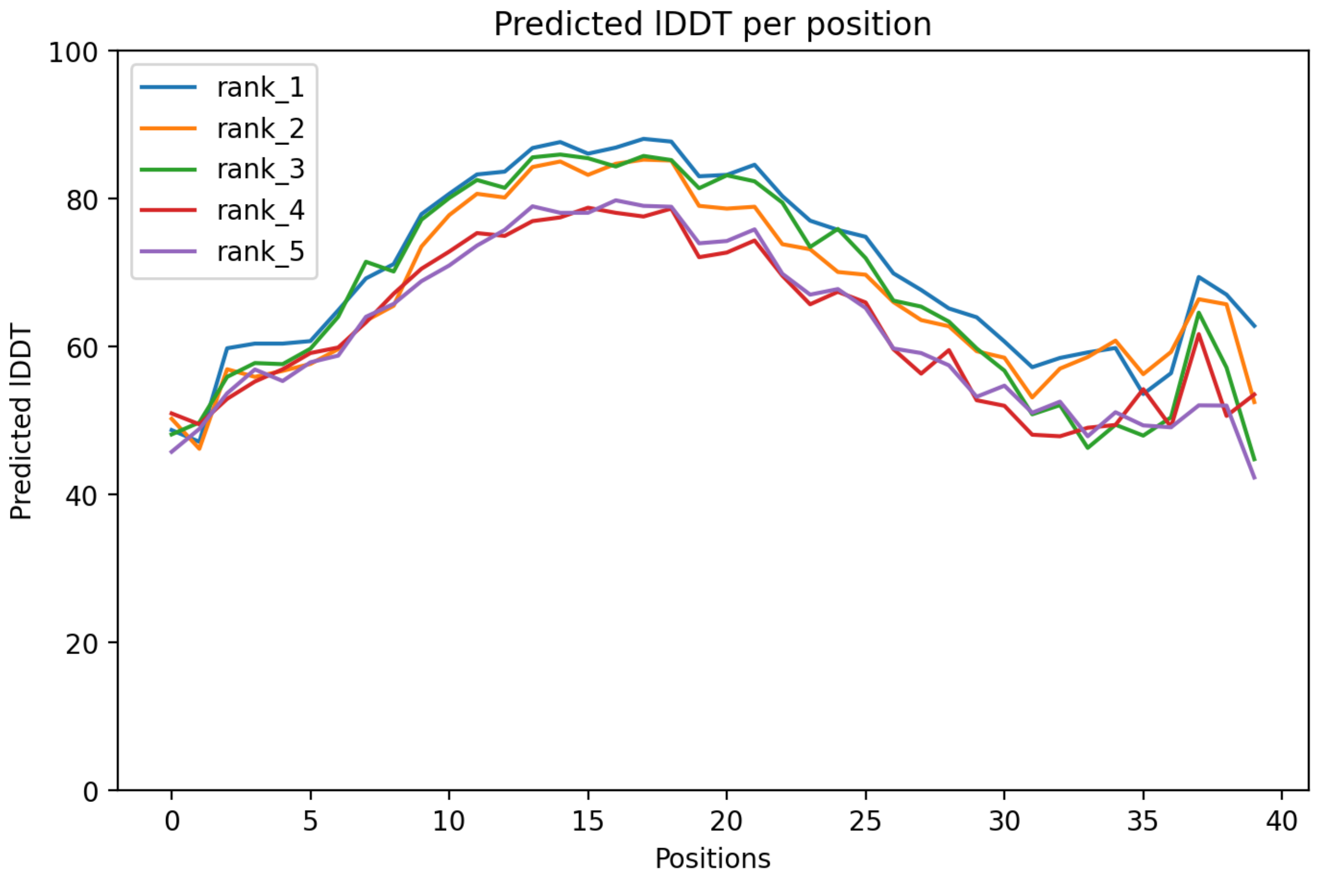

CP85: MtrunA17_Chr5g0415031

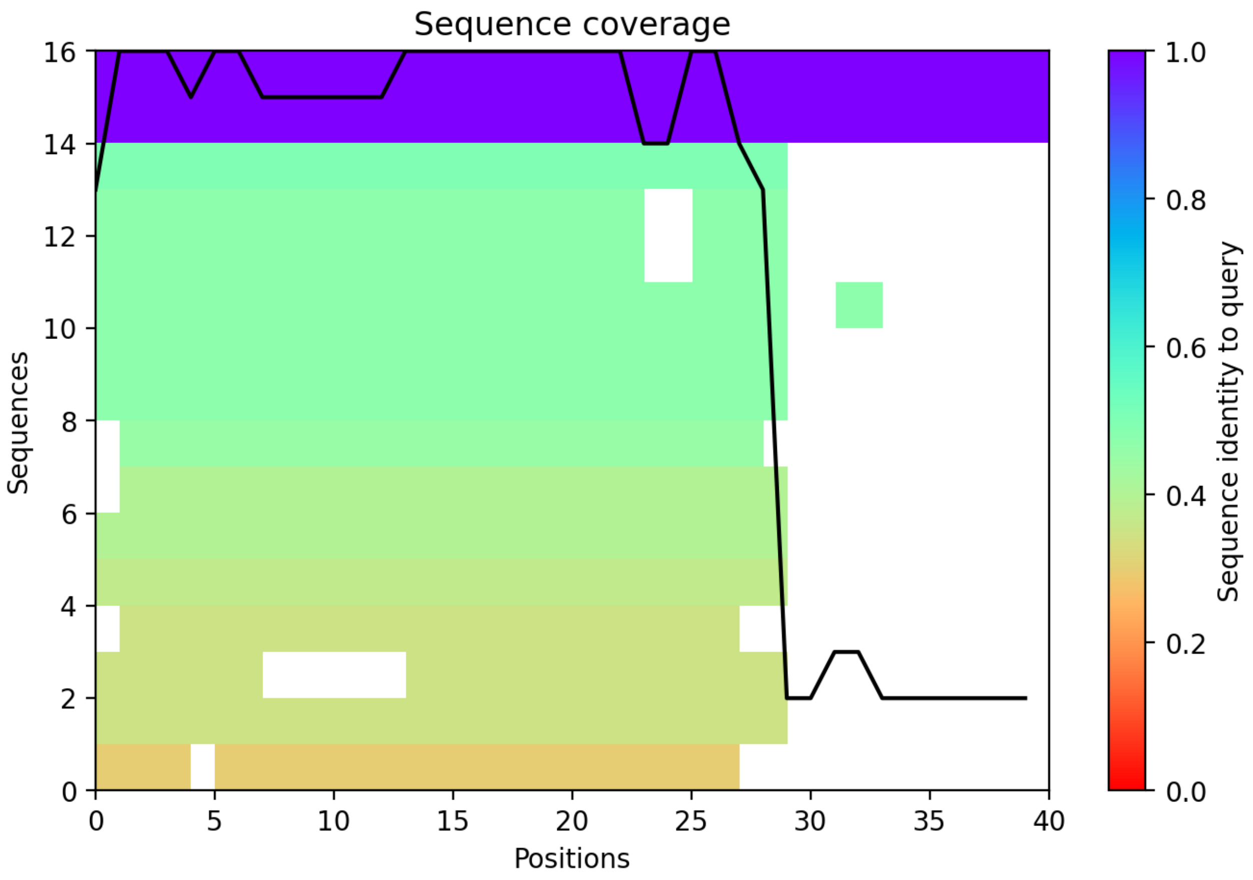

CP86: MtrunA17_Chr5g0415911

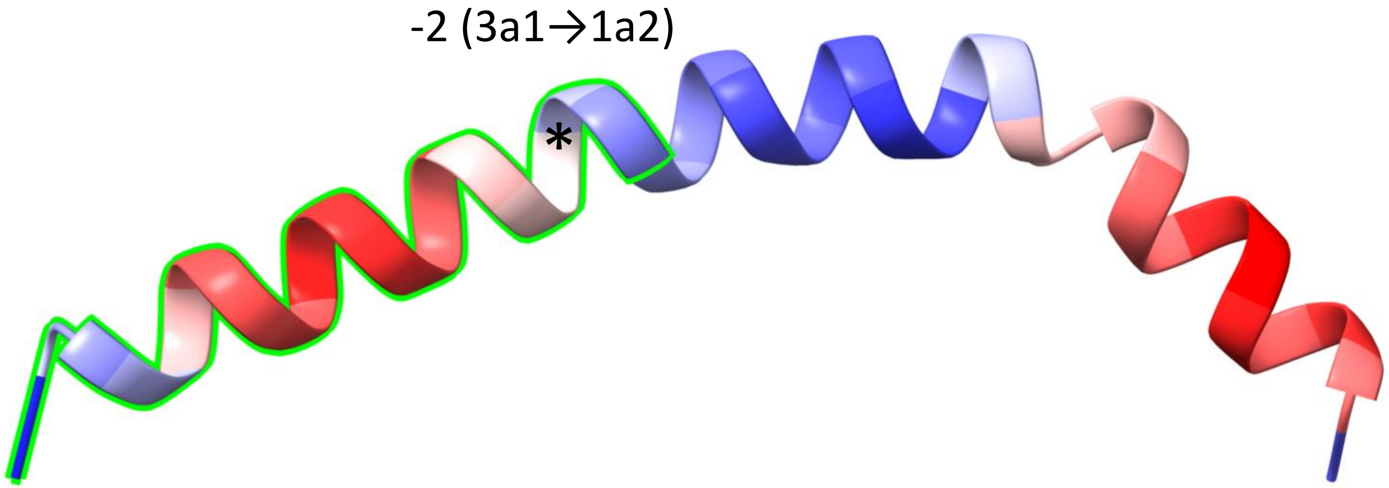

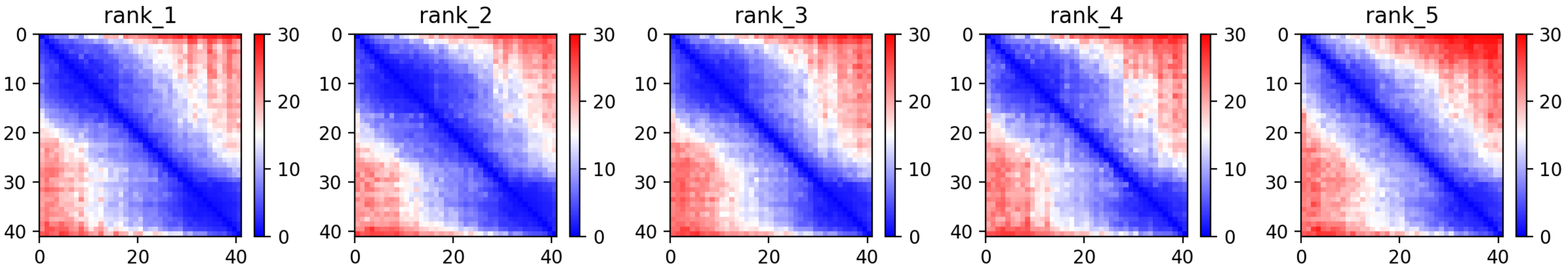

CP86: MtrunA17_Chr5g0415911

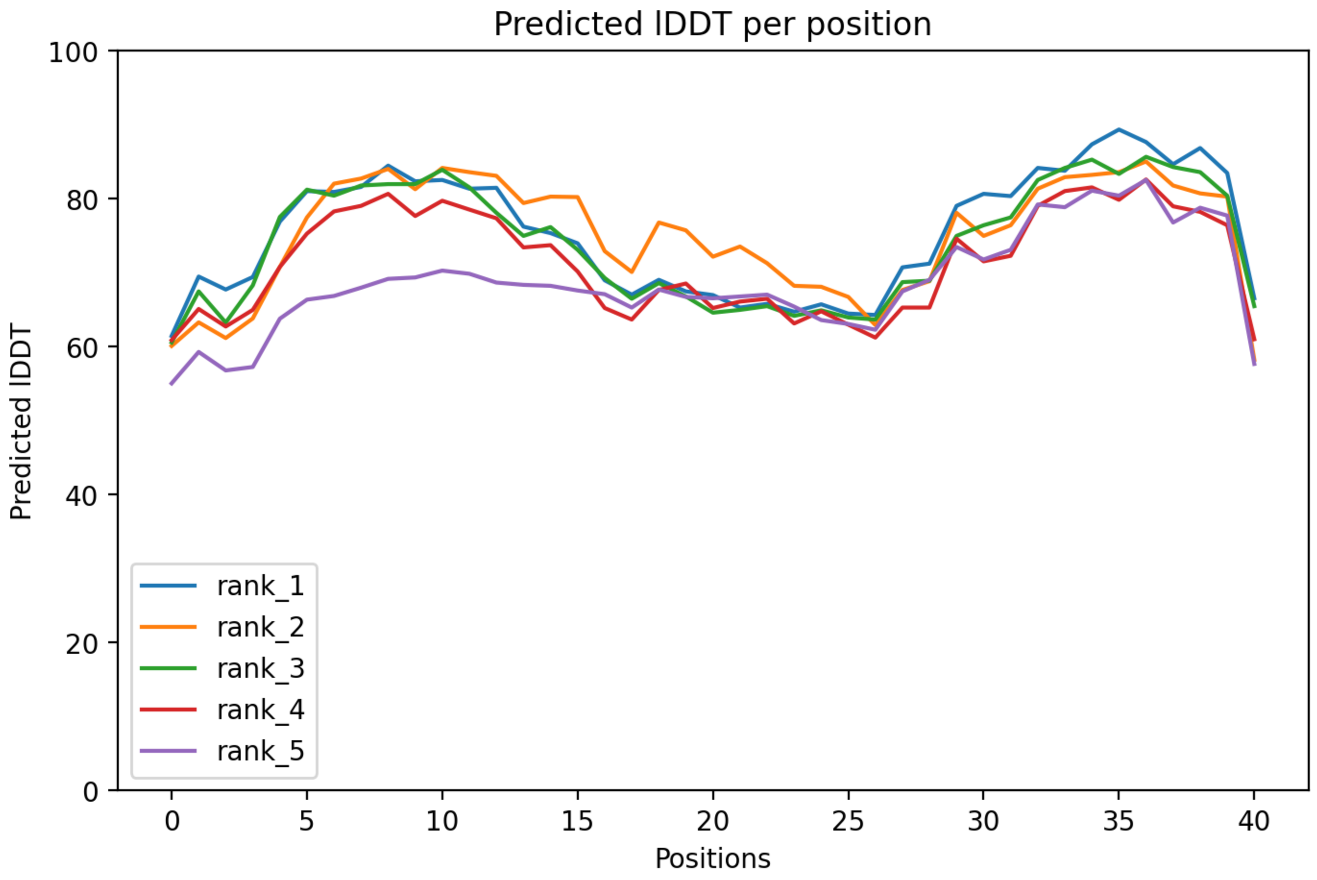

CP86: MtrunA17_Chr5g0415911

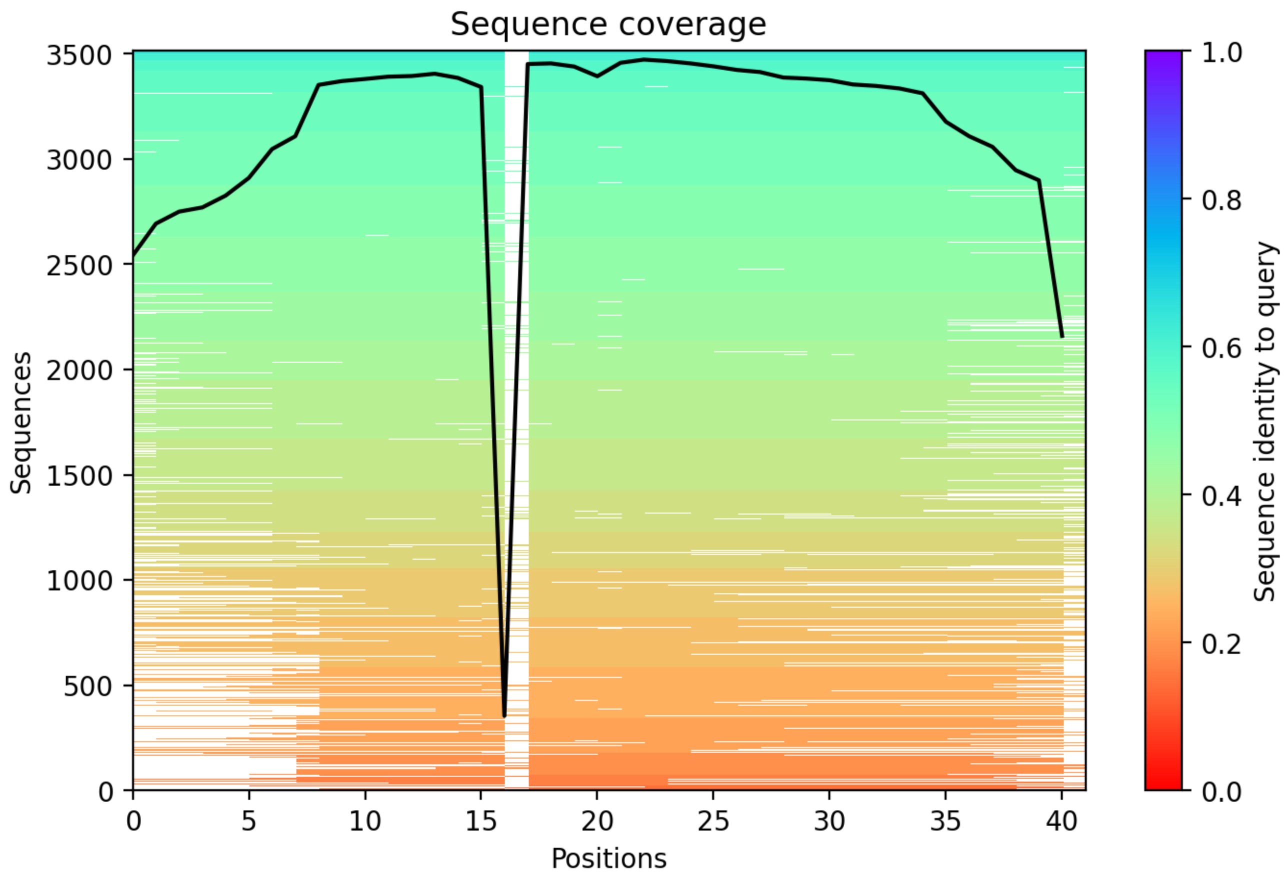

CP87: MtrunA17_Chr5g0421761

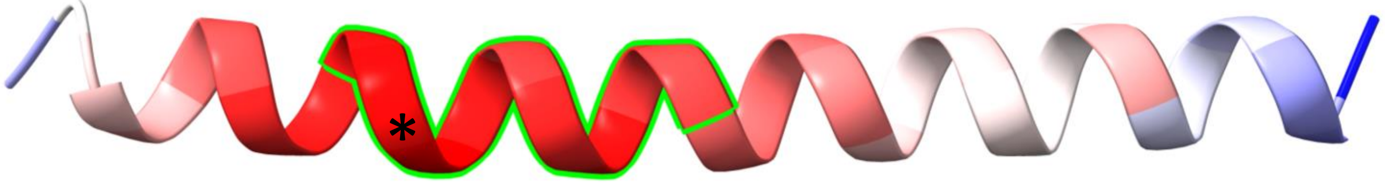

-1 (3a1→2a2)

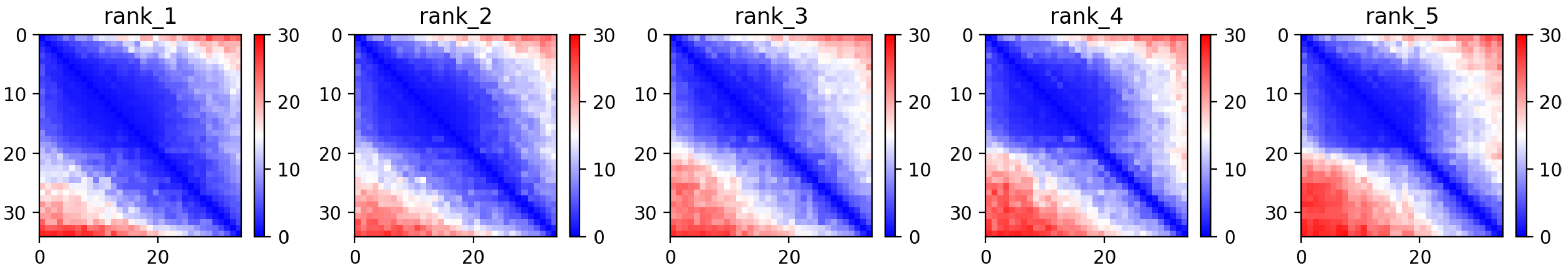

CP87: MtrunA17_Chr5g0421761

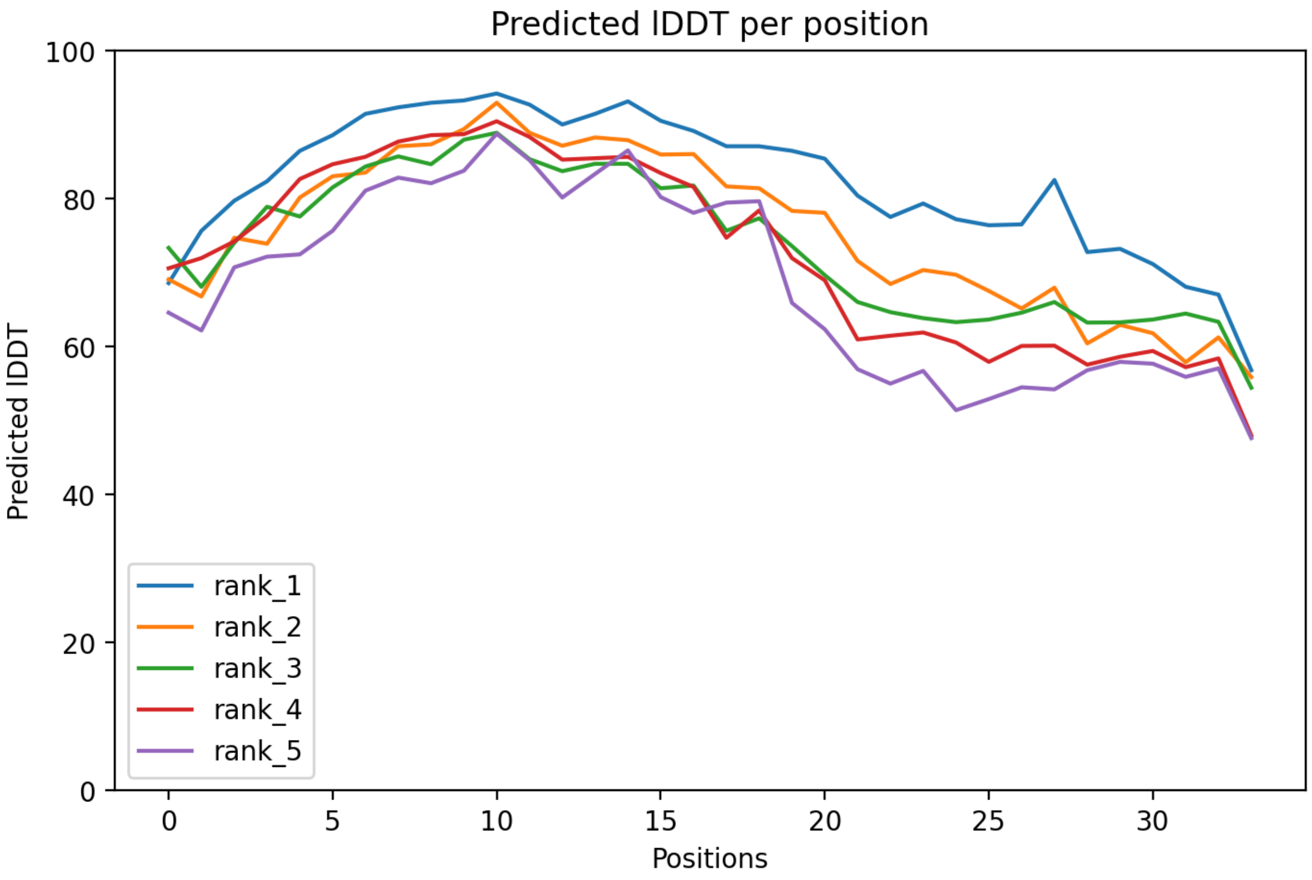

CP87: MtrunA17_Chr5g0421761

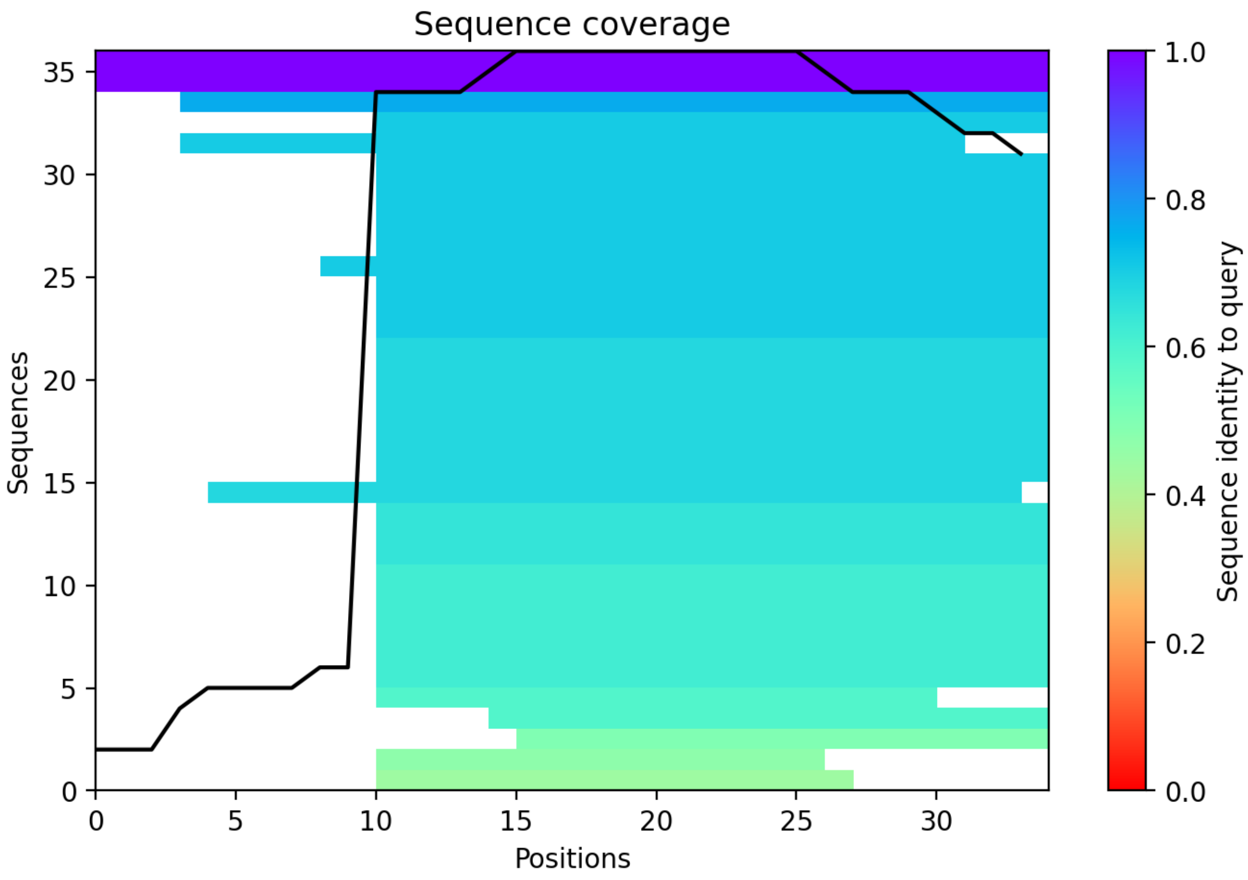

CP88: MtrunA17_Chr5g0422291

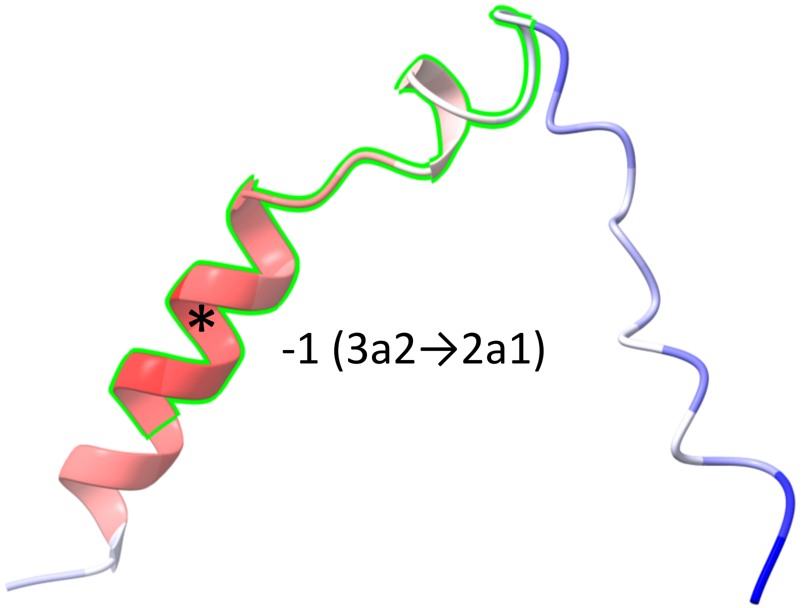

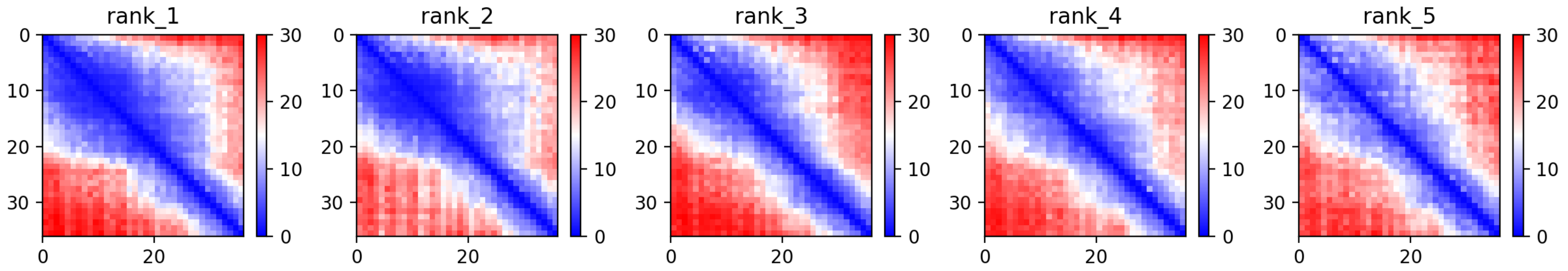

CP88: MtrunA17_Chr5g0422291

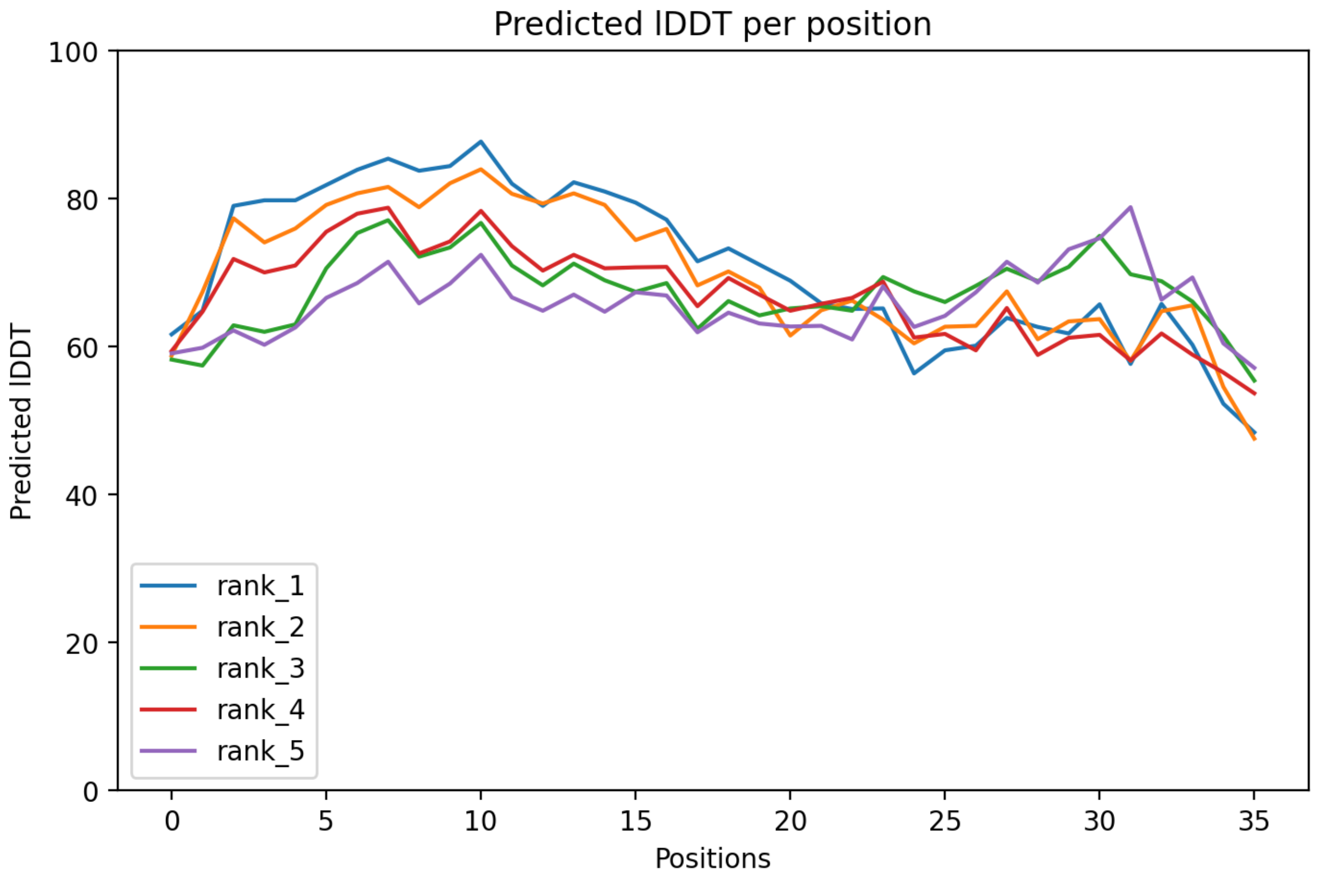

CP88: MtrunA17_Chr5g0422291

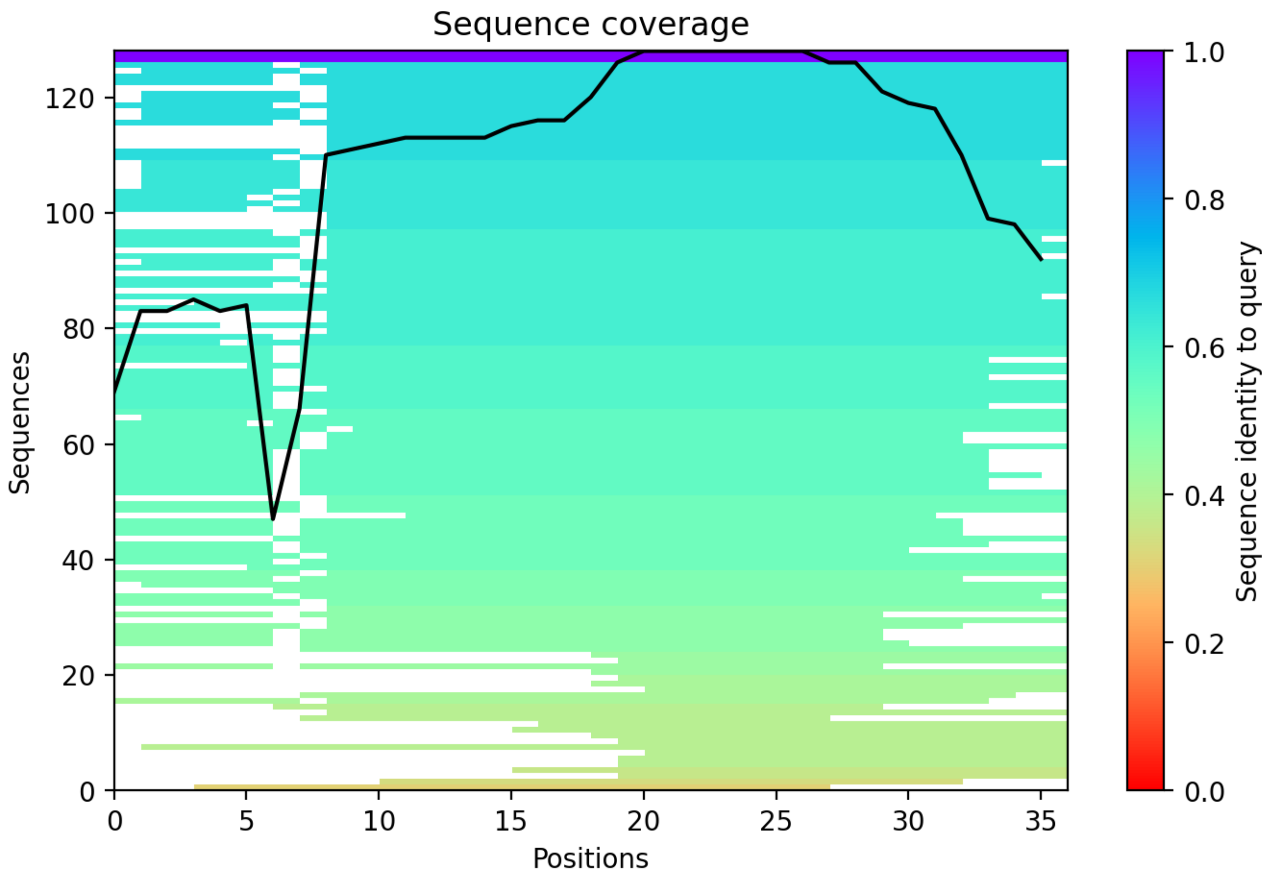

CP89: MtrunA17_Chr5g0422291

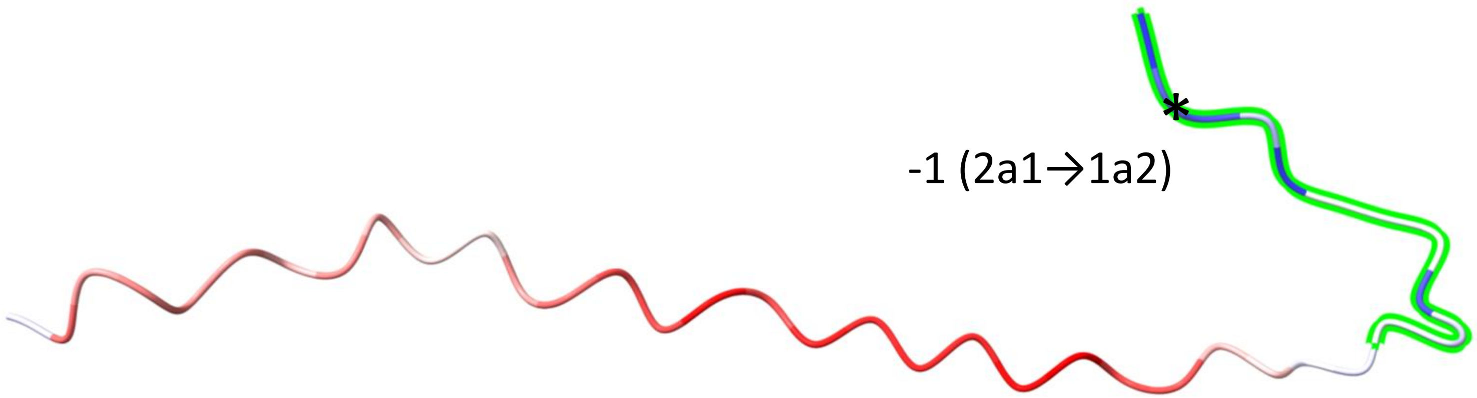

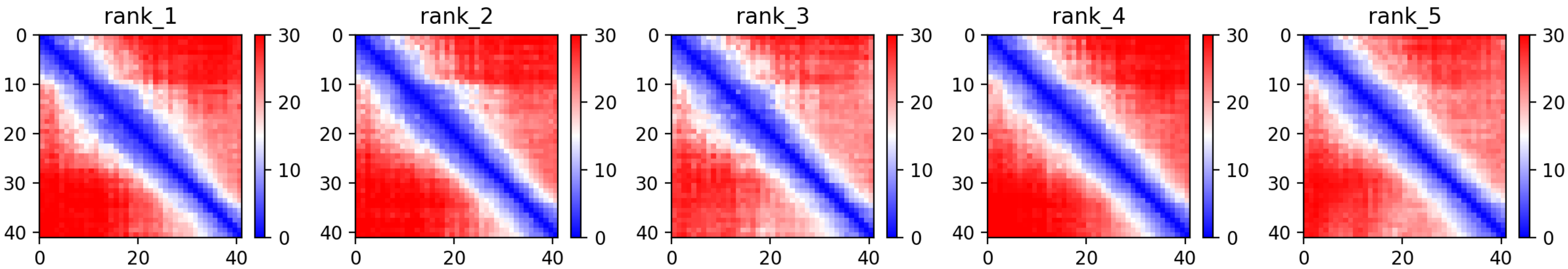

CP89: MtrunA17_Chr5g0422291

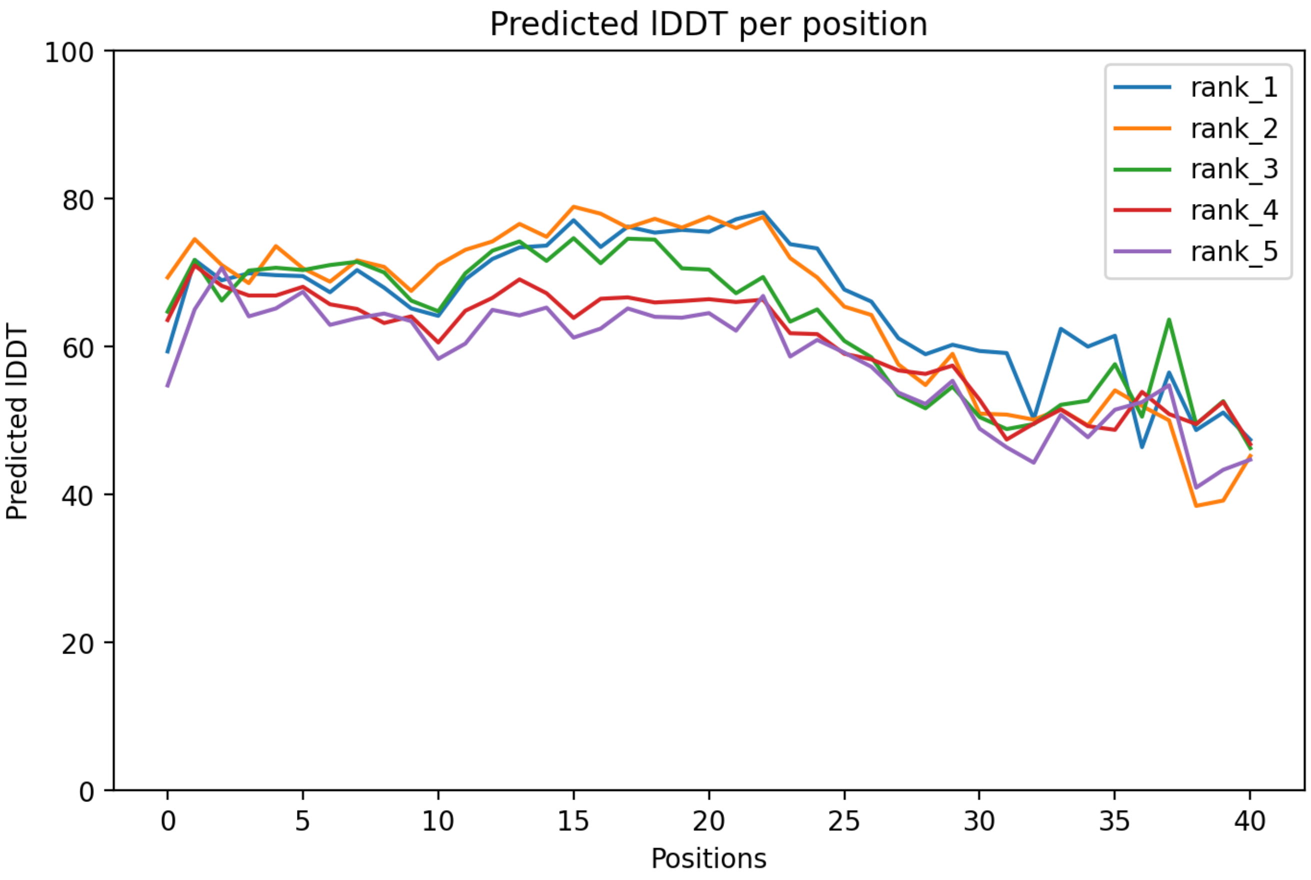

CP89: MtrunA17_Chr5g0422291

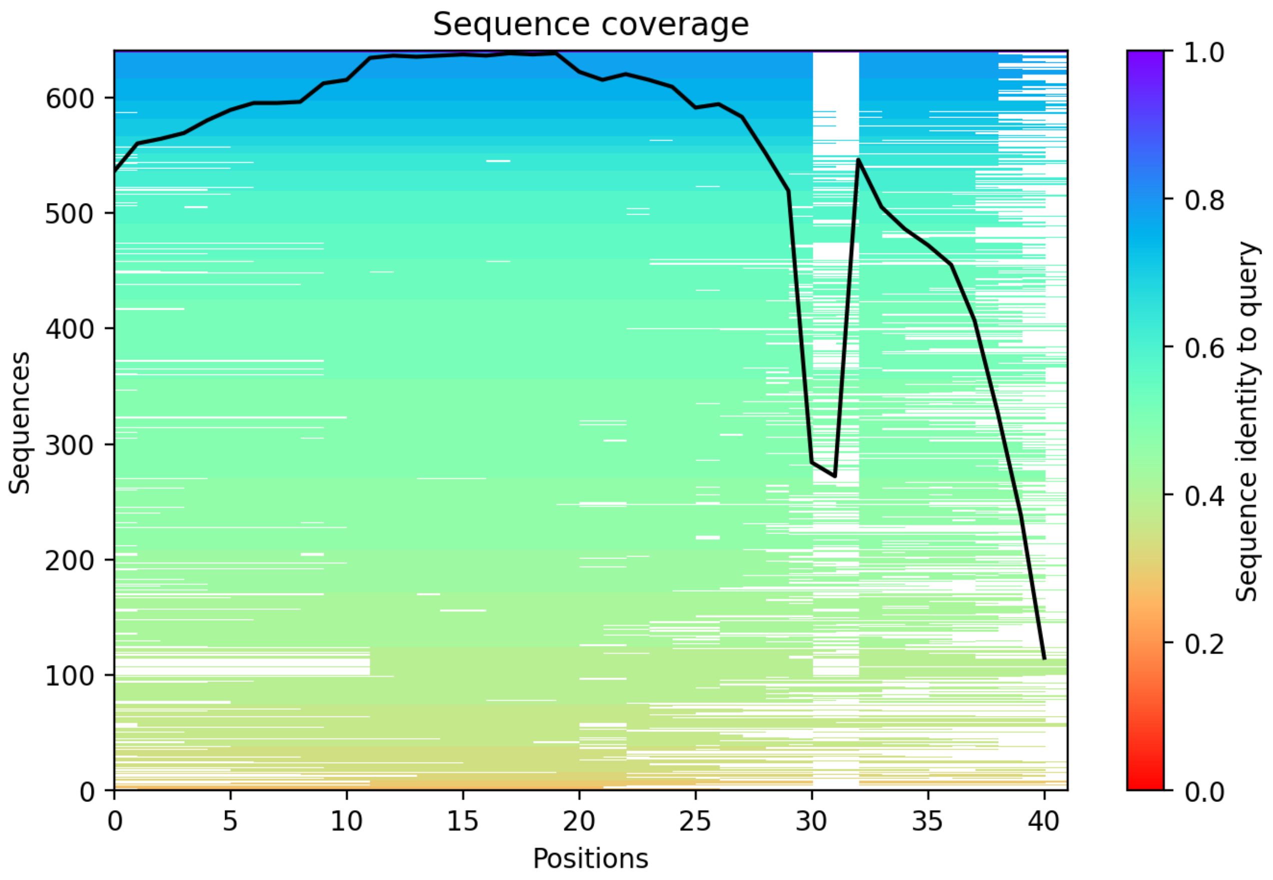

CP90: MtrunA17_Chr5g0430341

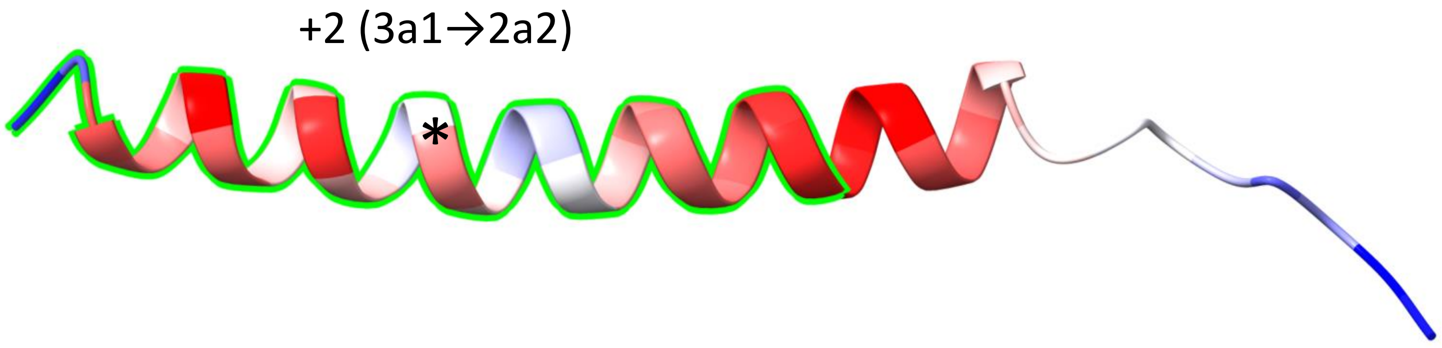

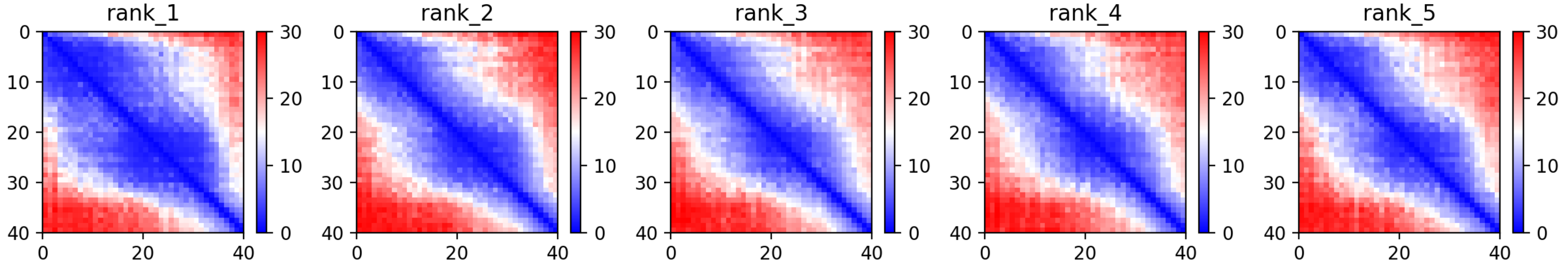

CP90: MtrunA17_Chr5g0430341

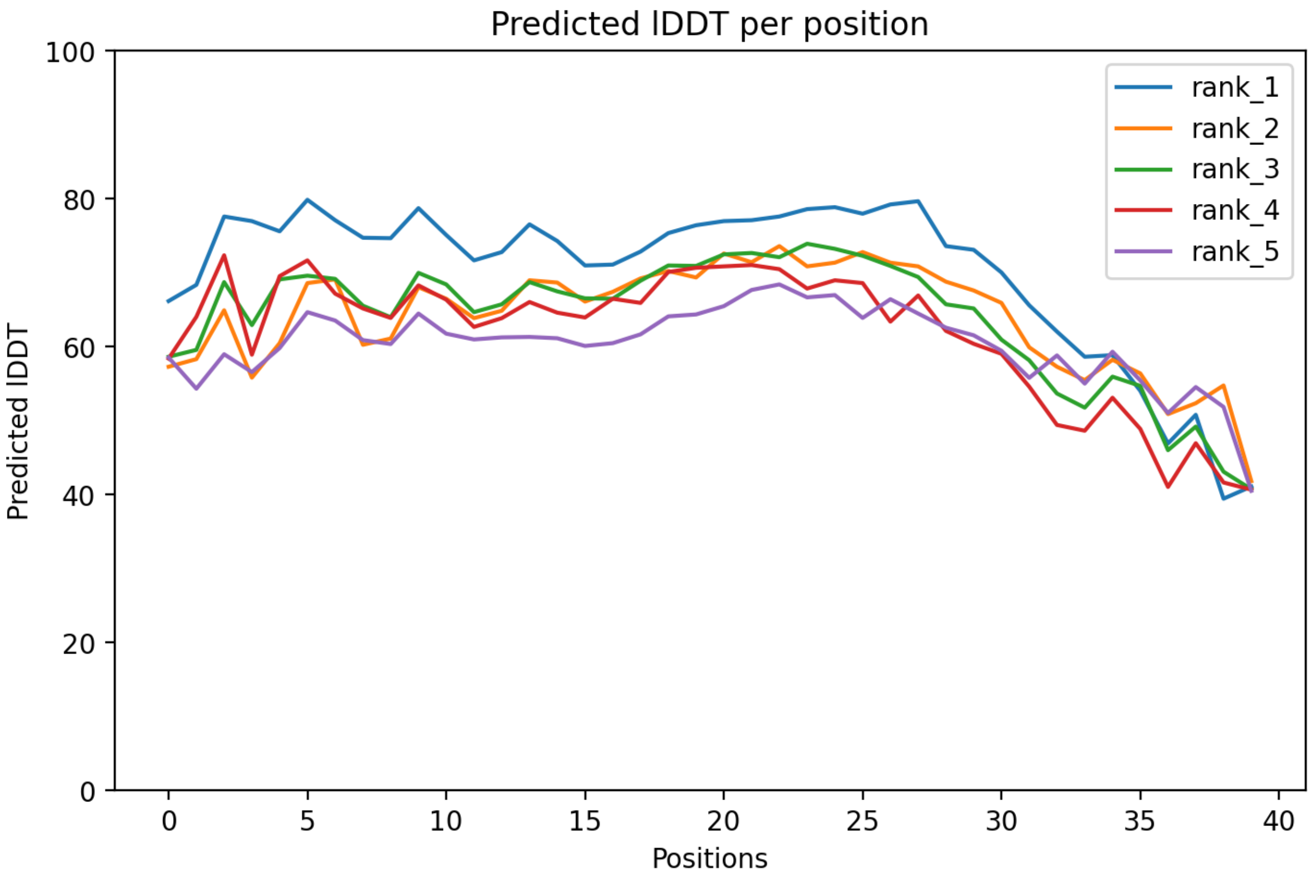

CP90: MtrunA17_Chr5g0430341

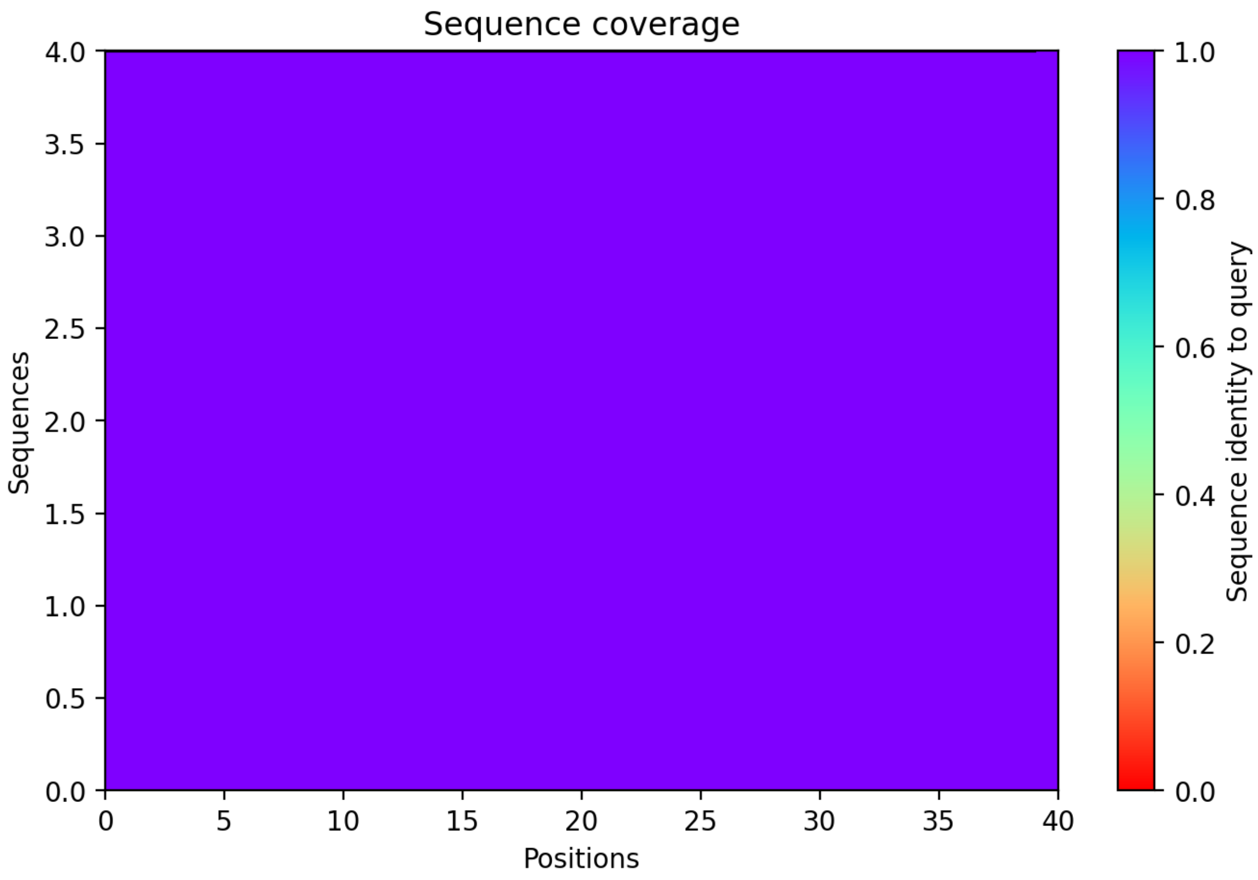

CP91: MtrunA17_Chr5g0430341

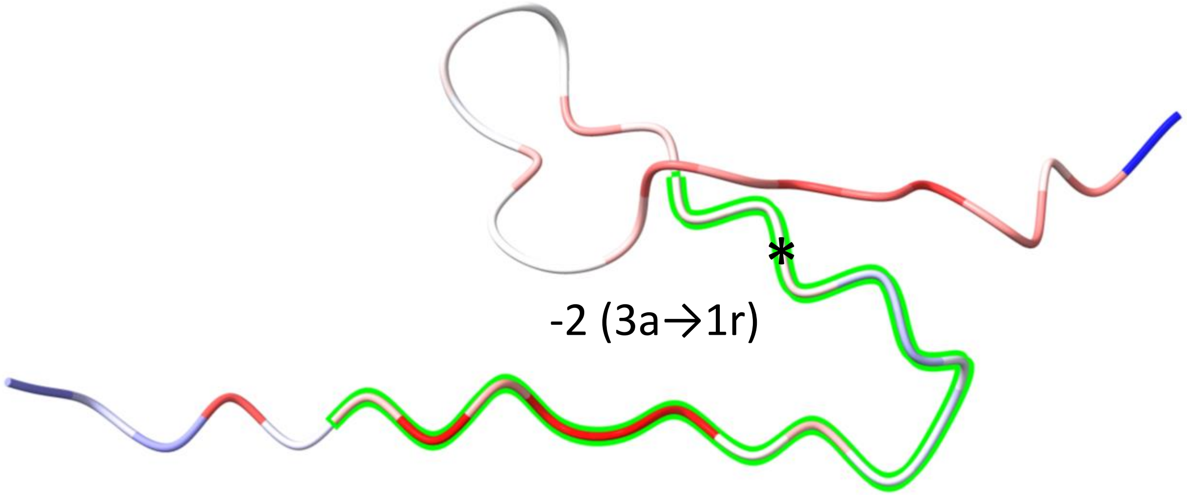

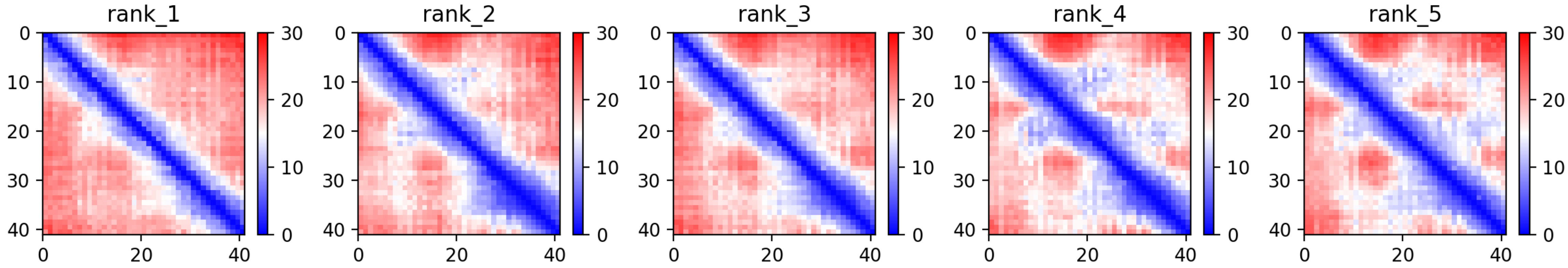

CP91: MtrunA17_Chr5g0430341

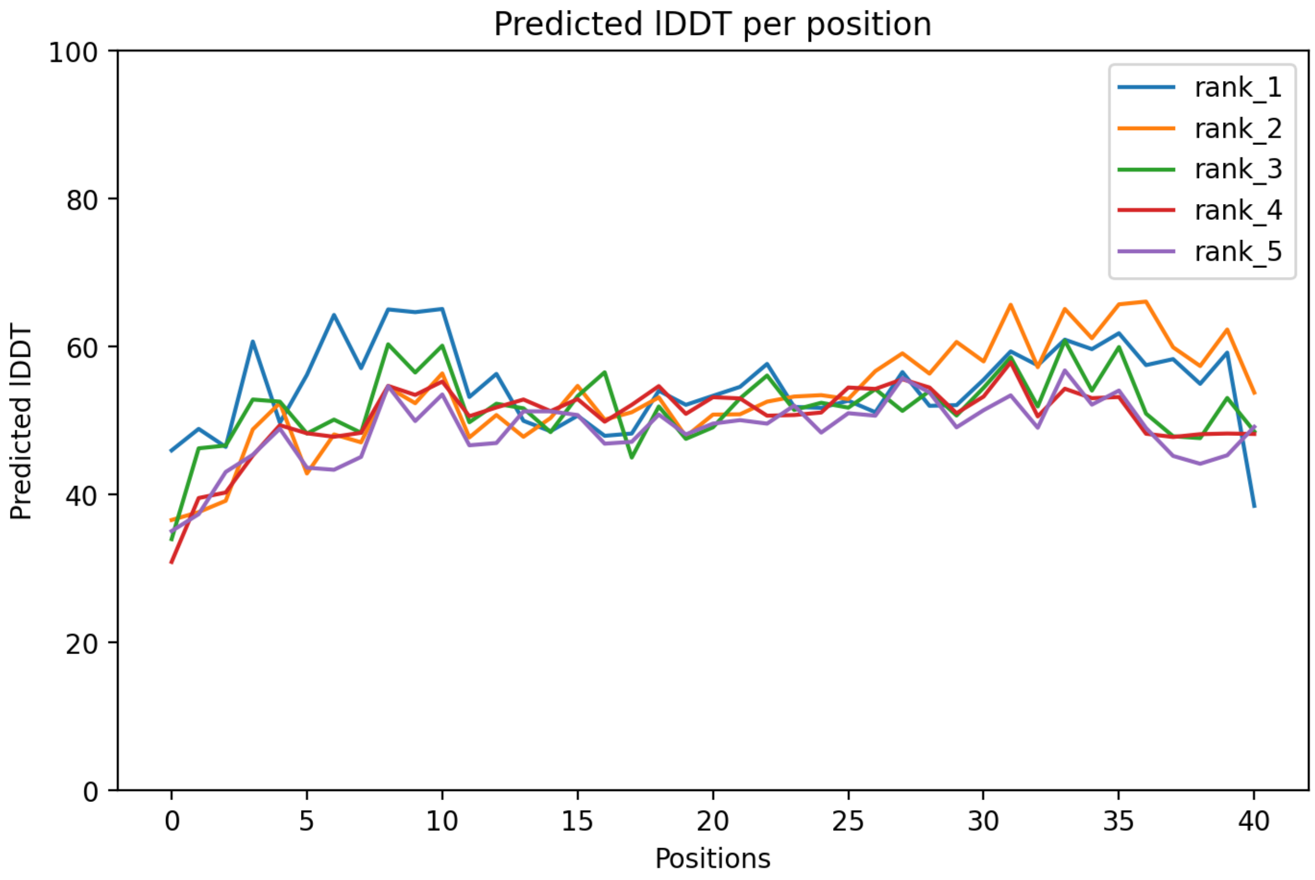

CP91: MtrunA17_Chr5g0430341

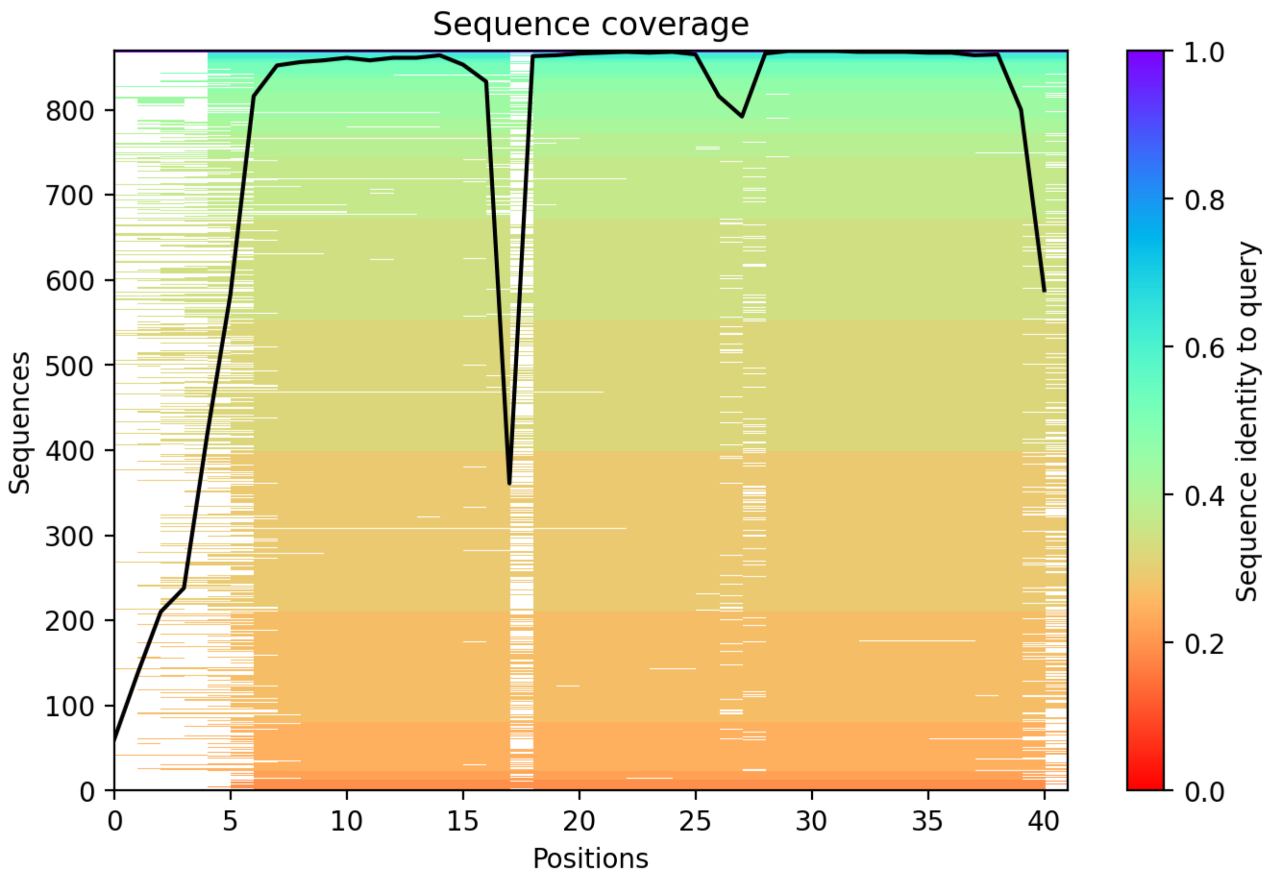

CP92: MtrunA17_Chr5g0431401

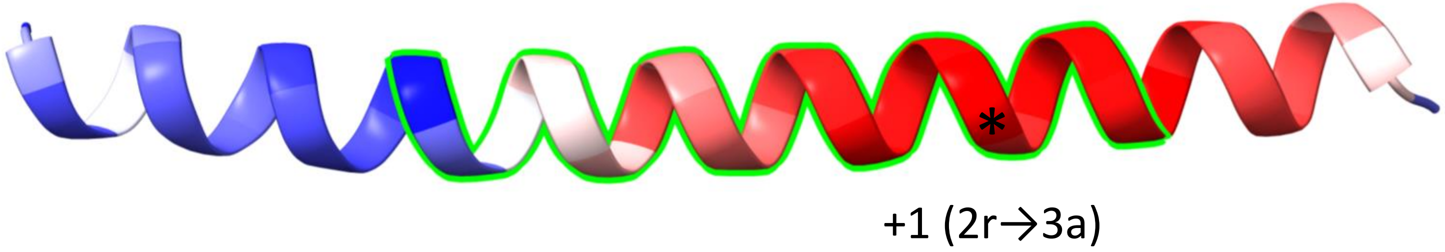

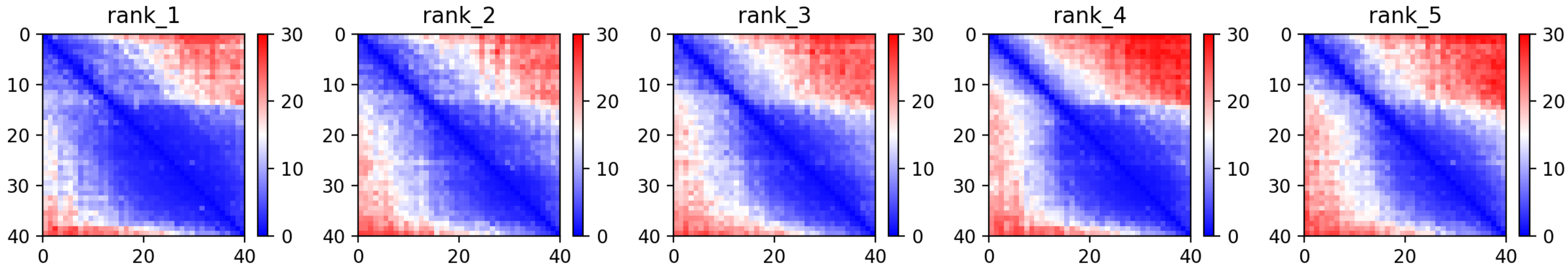

CP92: MtrunA17_Chr5g0431401

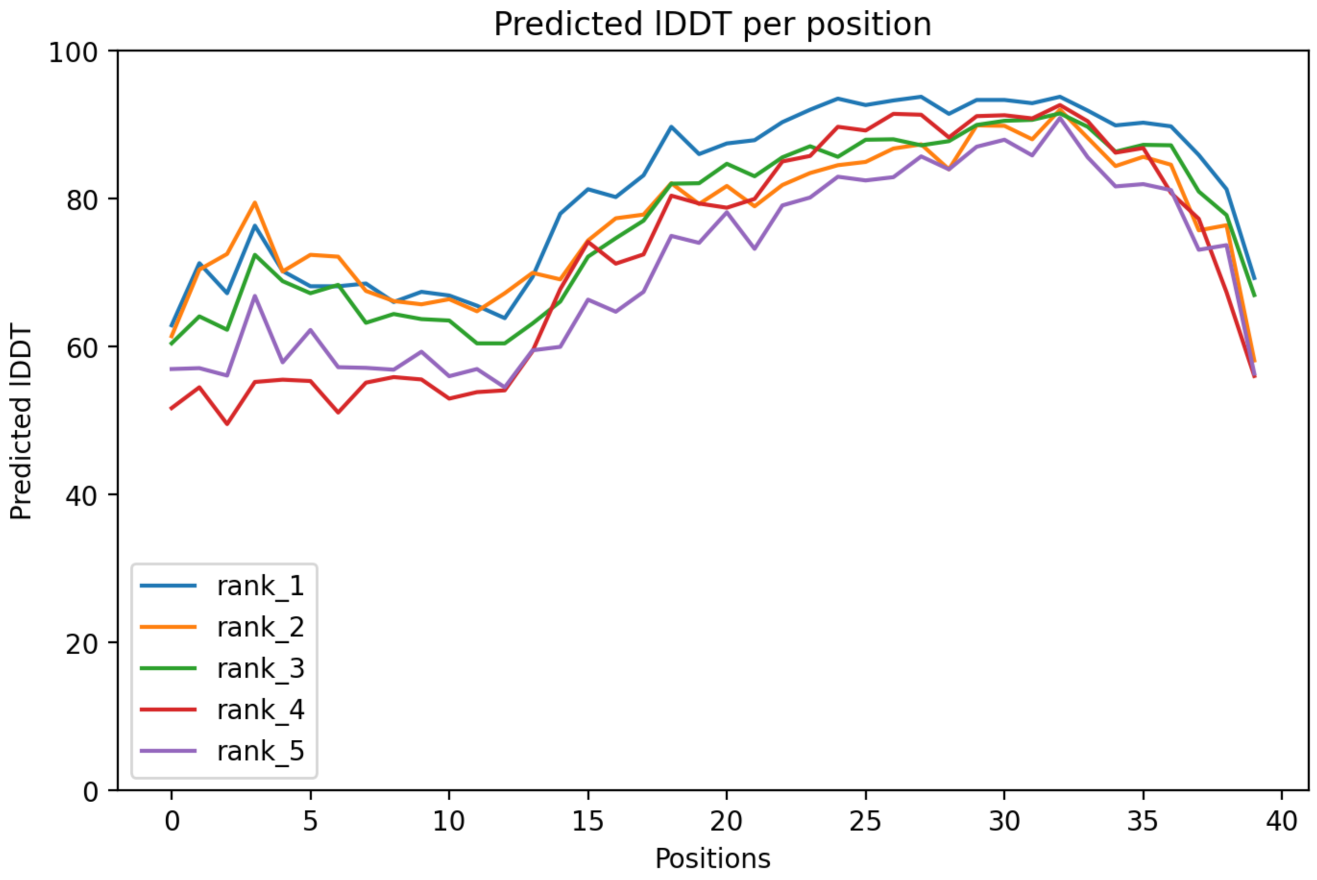

CP92: MtrunA17_Chr5g0431401

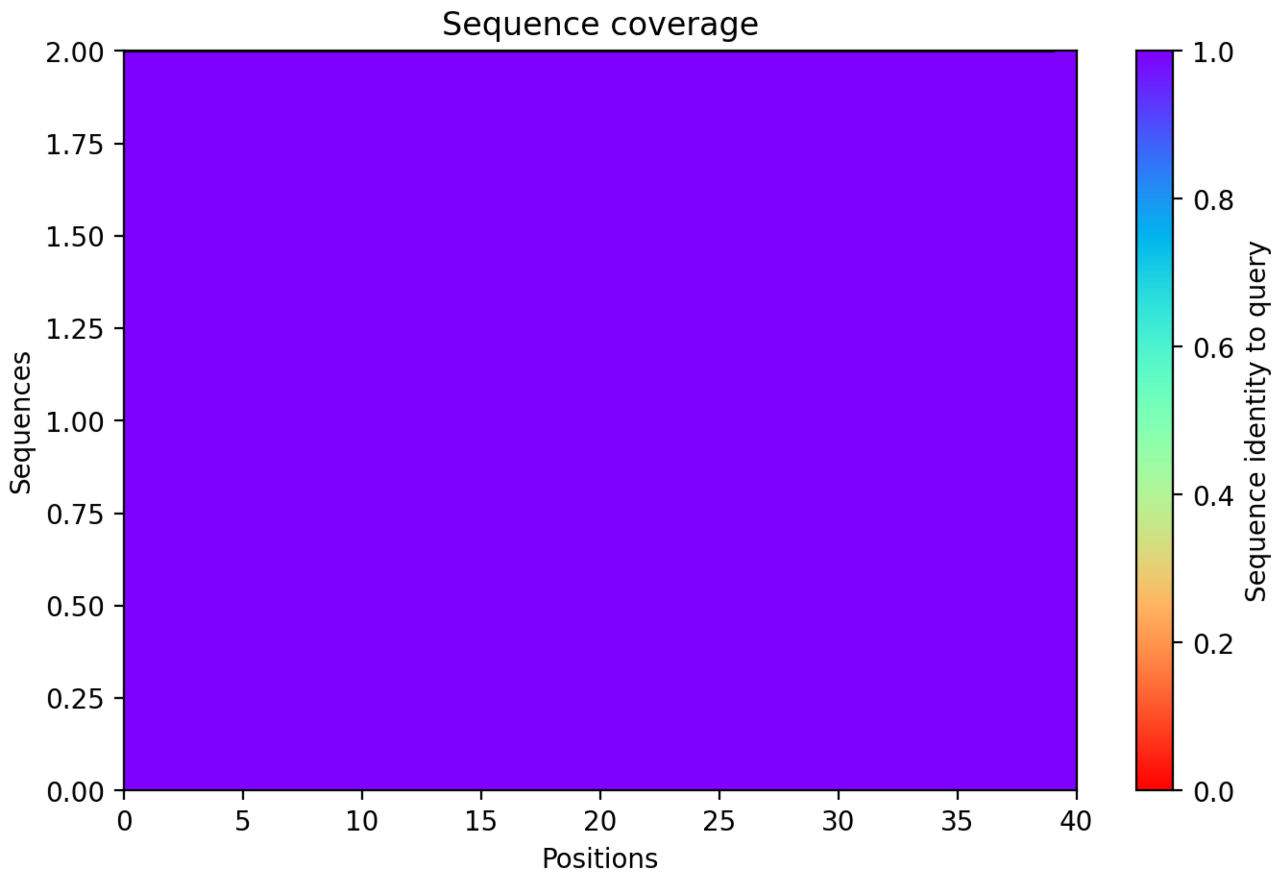

CP93: MtrunA17_Chr5g0435191

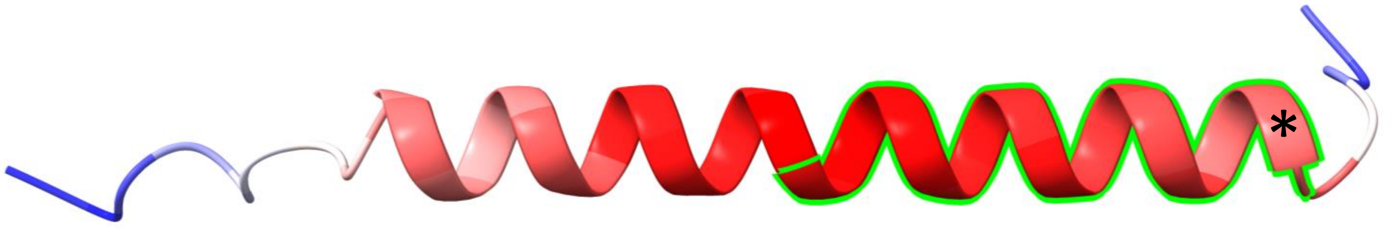

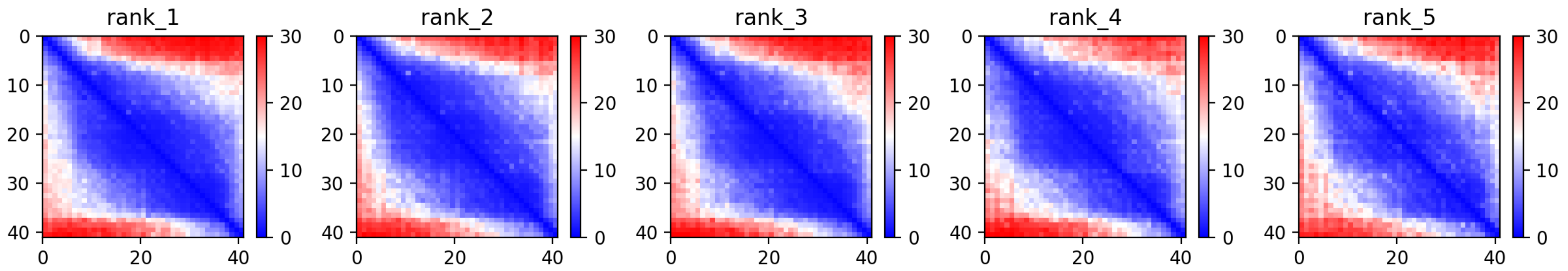

CP93: MtrunA17_Chr5g0435191

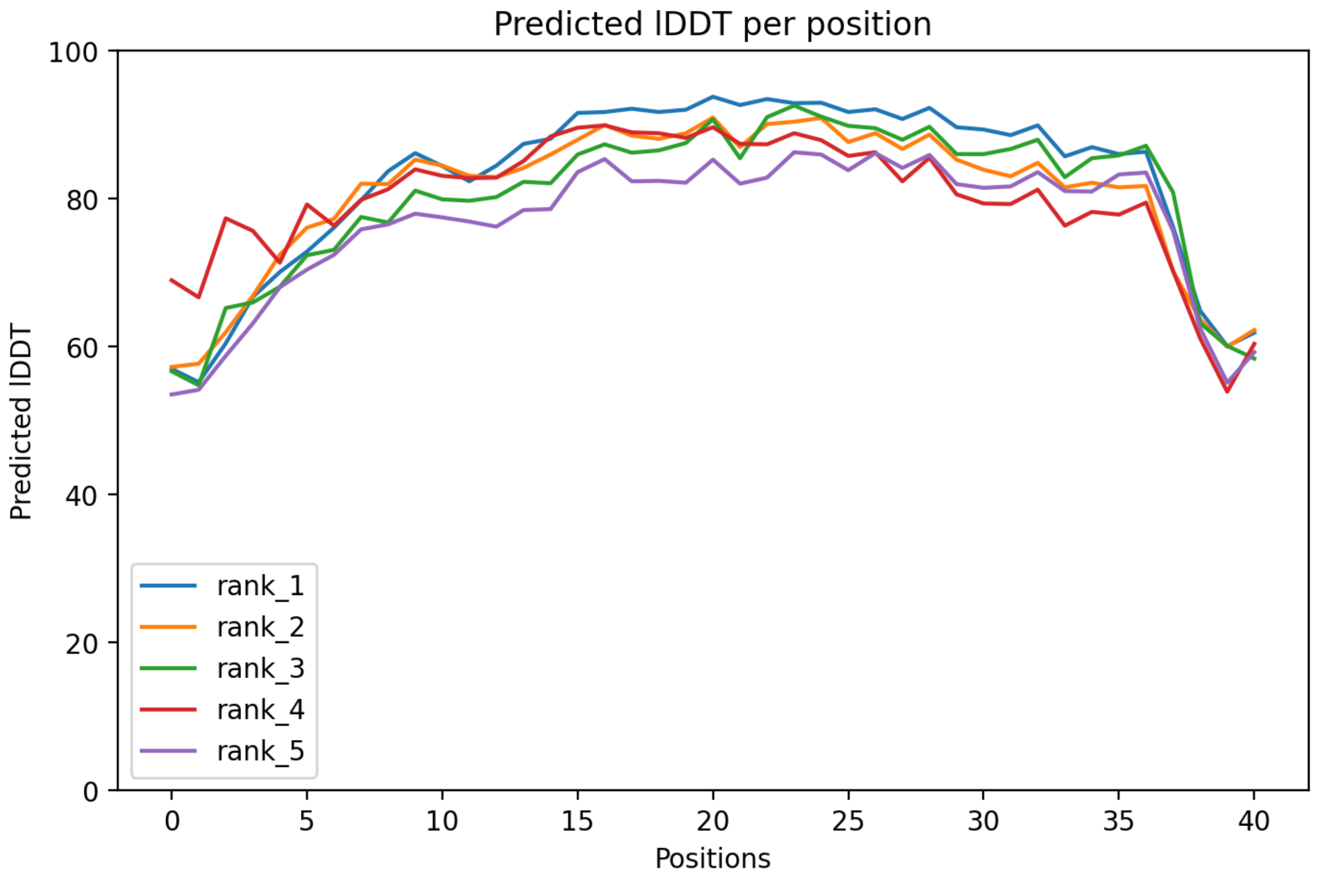

CP93: MtrunA17_Chr5g0435191

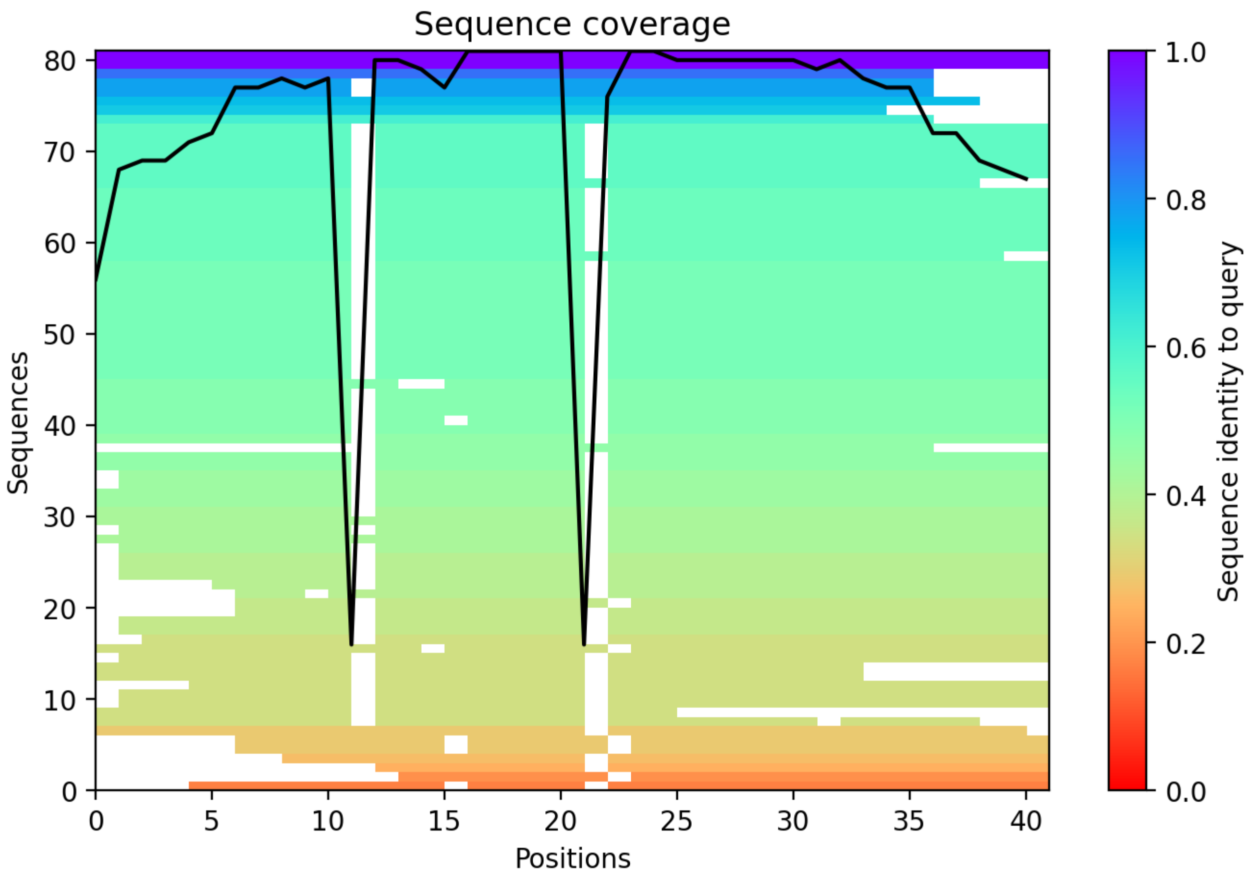

CP94: MtrunA17_Chr5g0444231

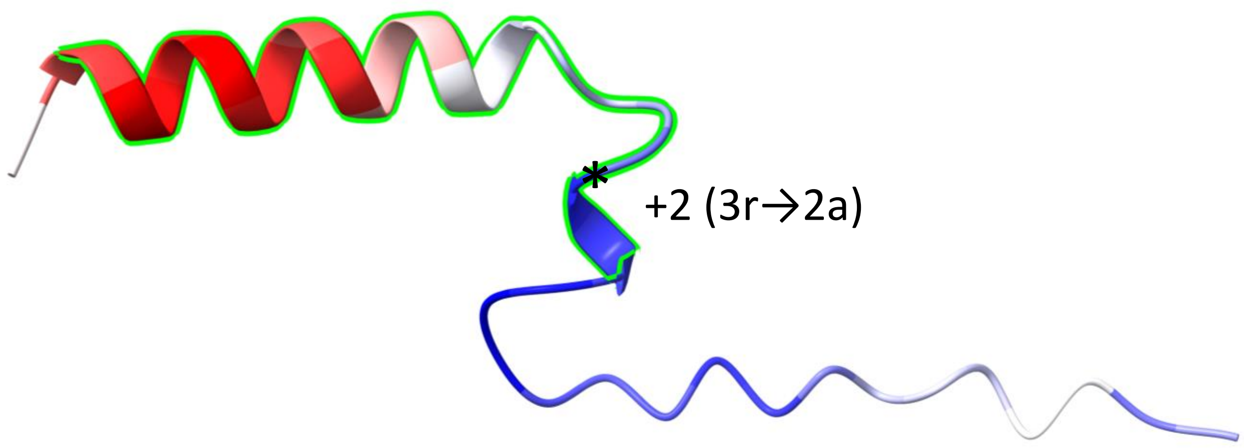

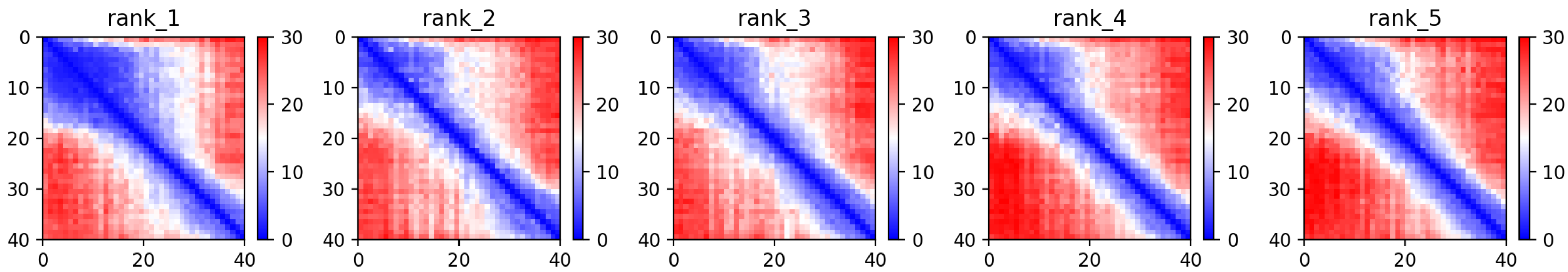

CP94: MtrunA17_Chr5g0444231

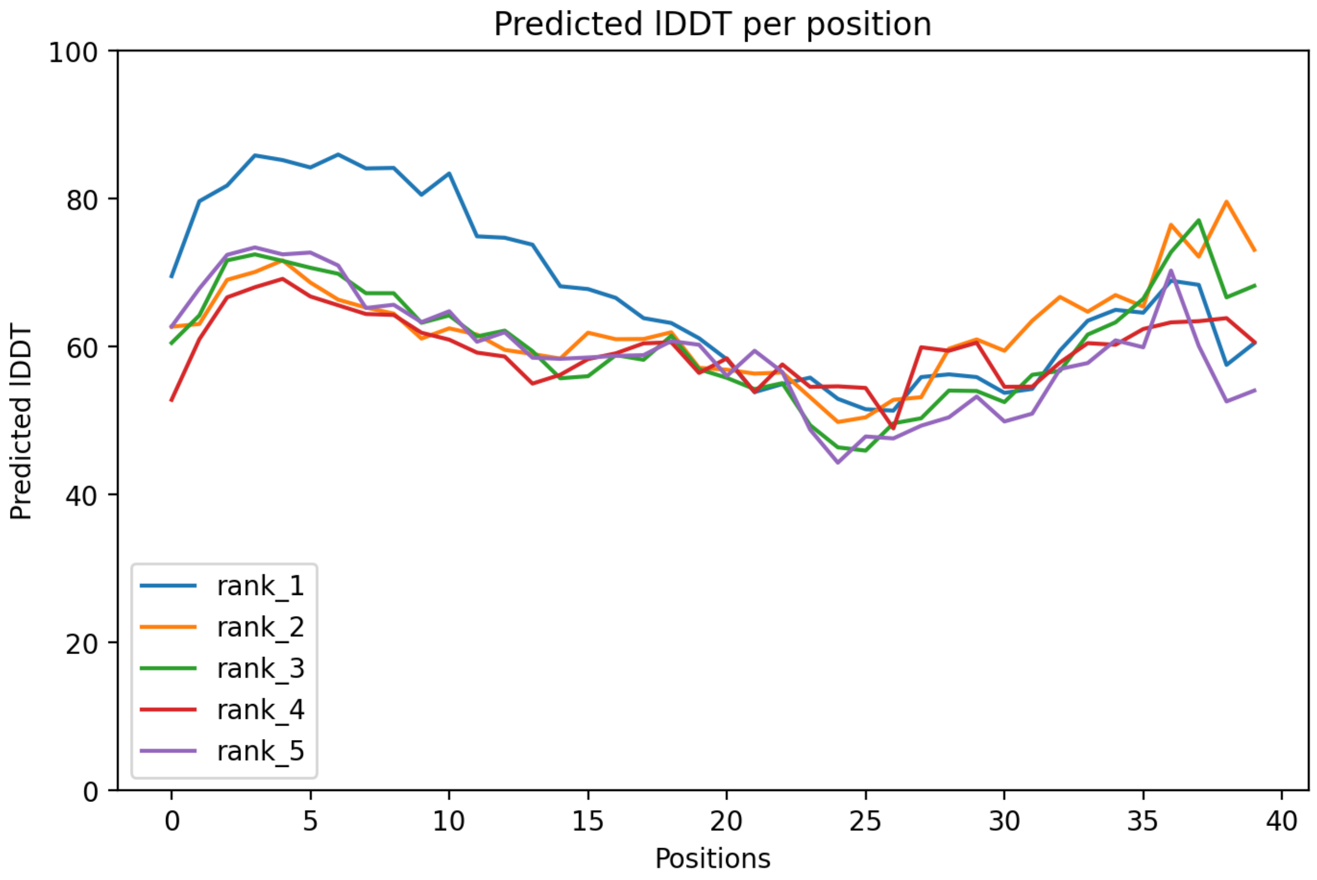

CP94: MtrunA17_Chr5g0444231

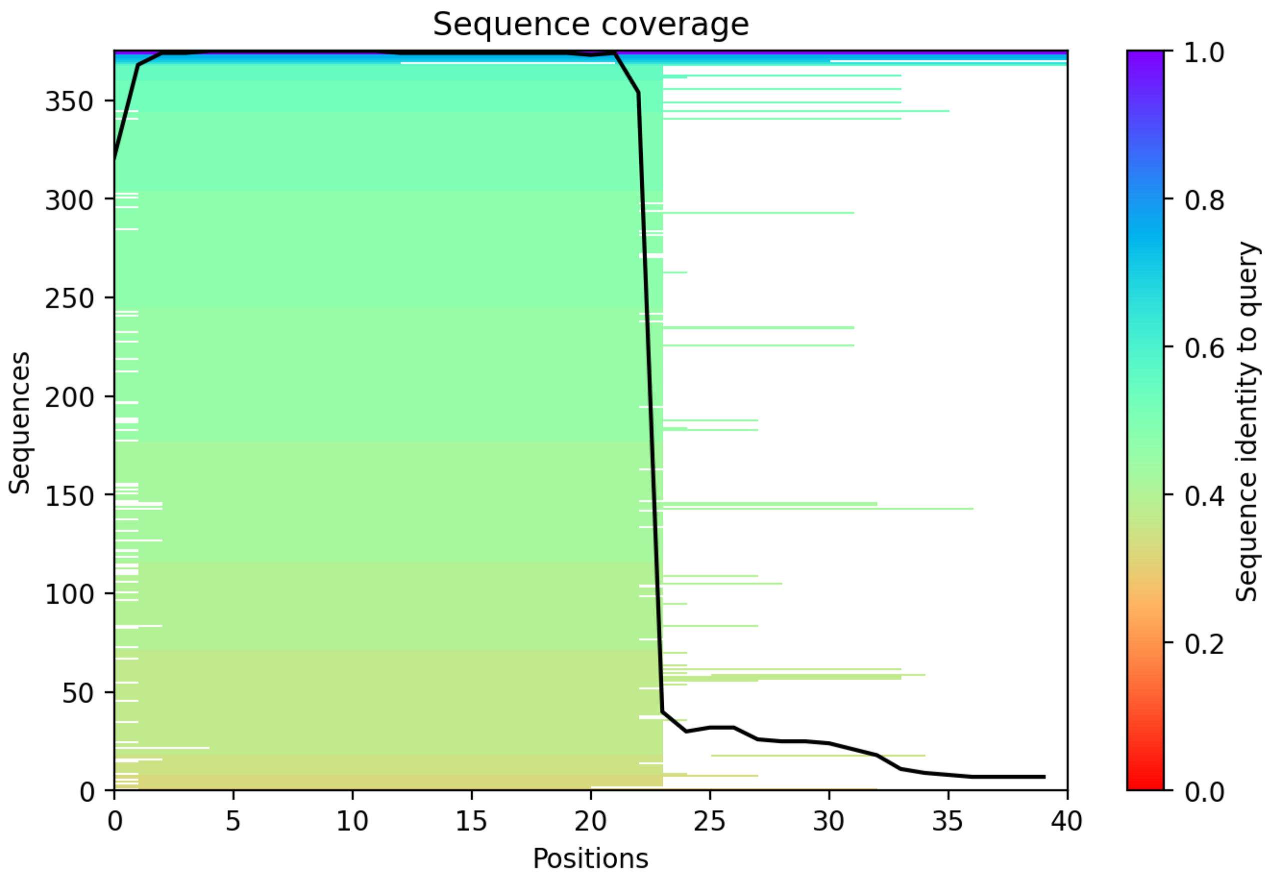

CP95: MtrunA17_Chr6g0451601

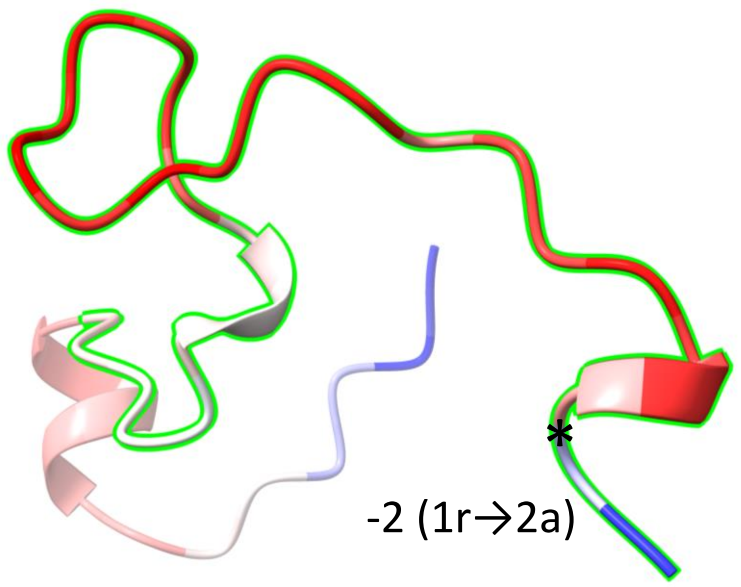

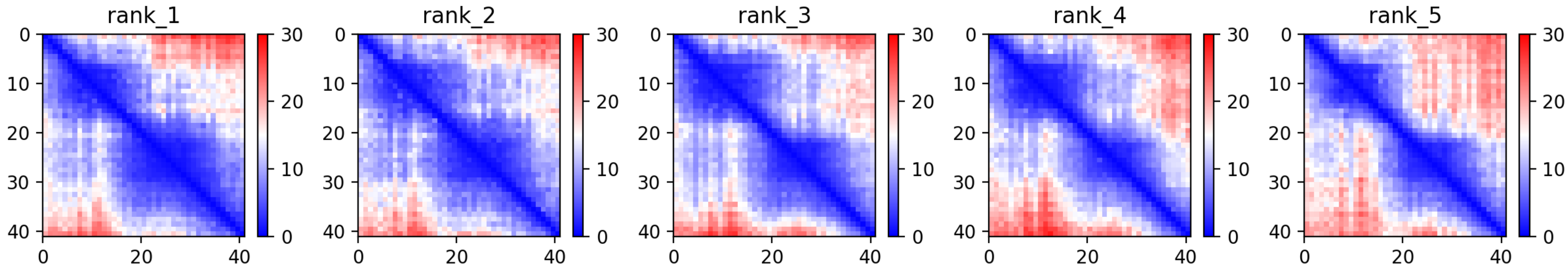

CP95: MtrunA17_Chr6g0451601

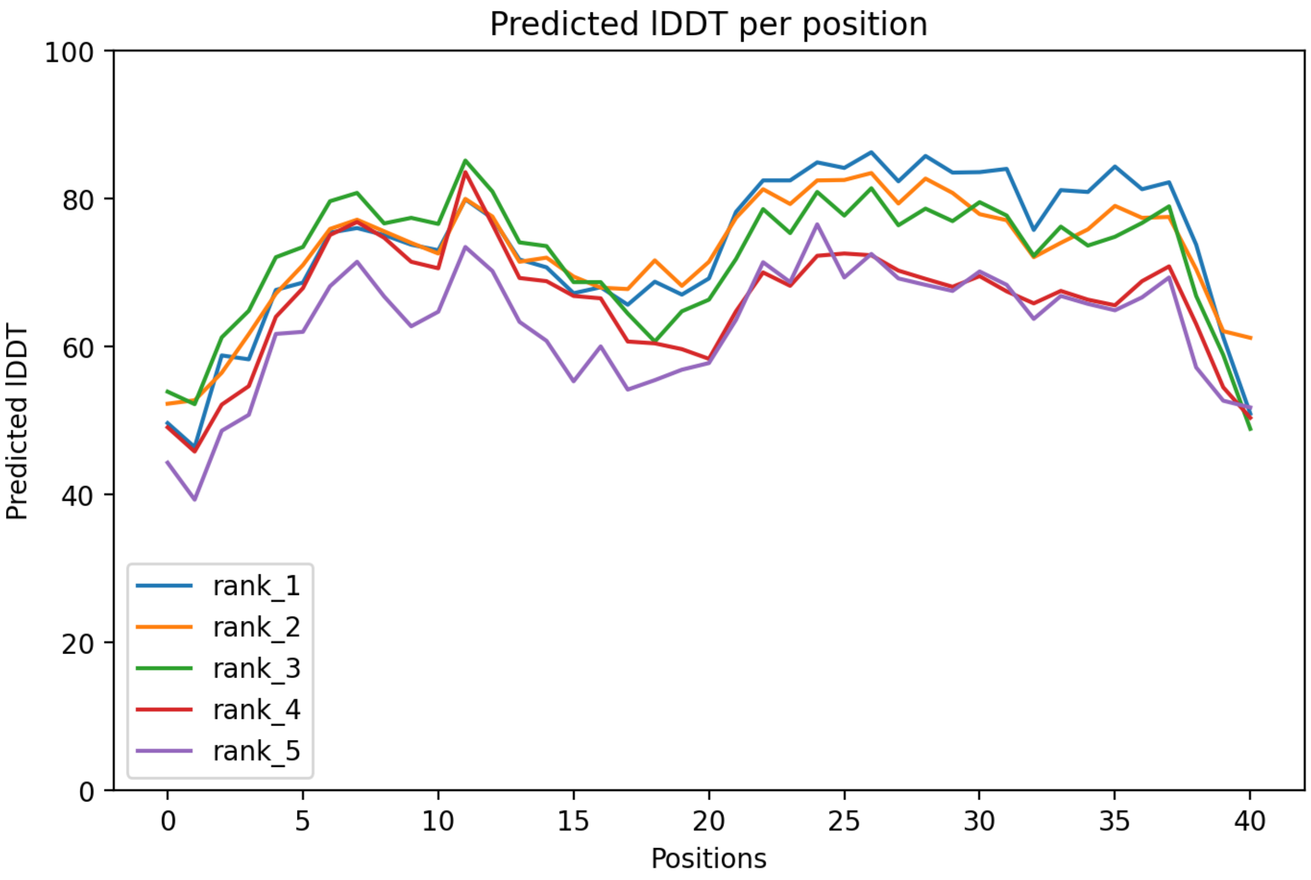

CP95: MtrunA17_Chr6g0451601

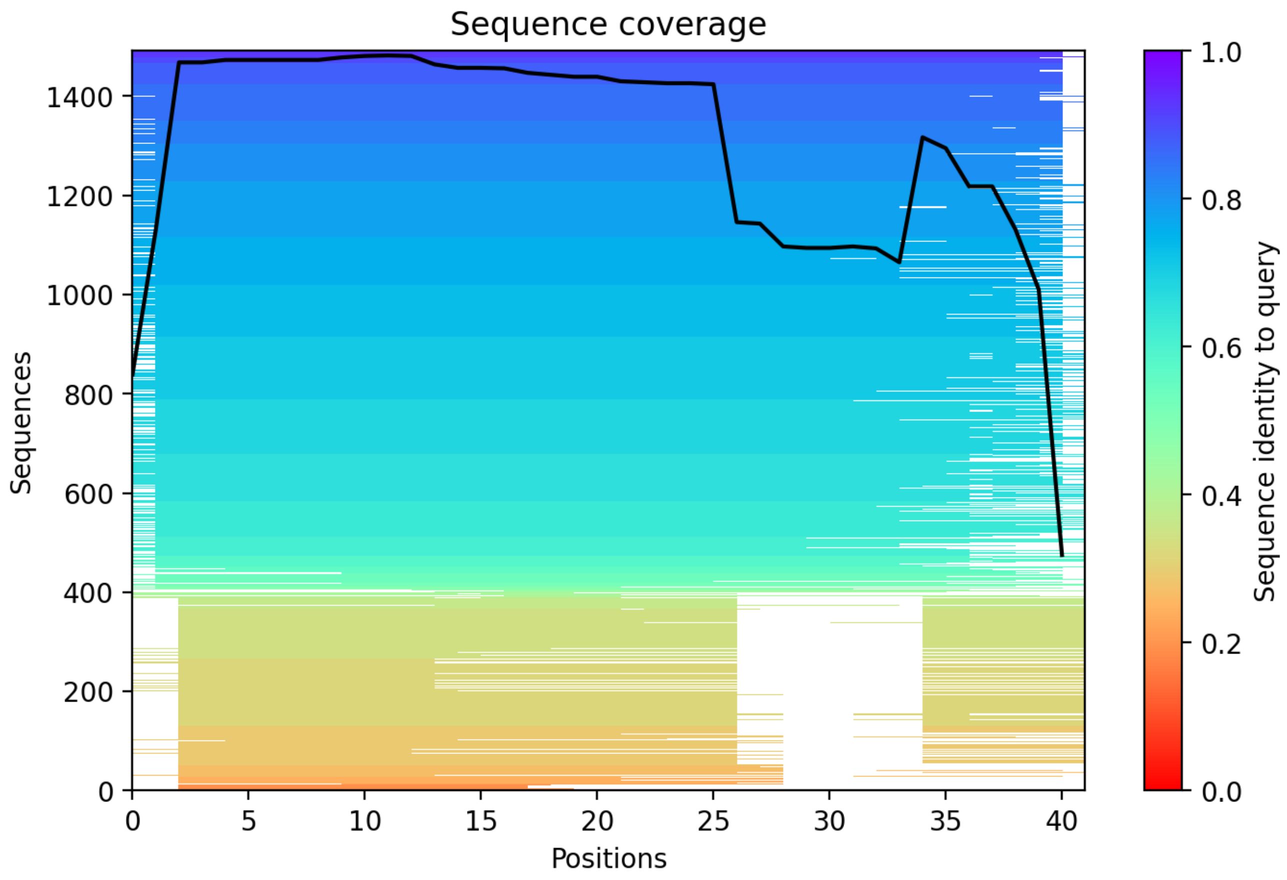

CP96: MtrunA17_Chr6g0452781

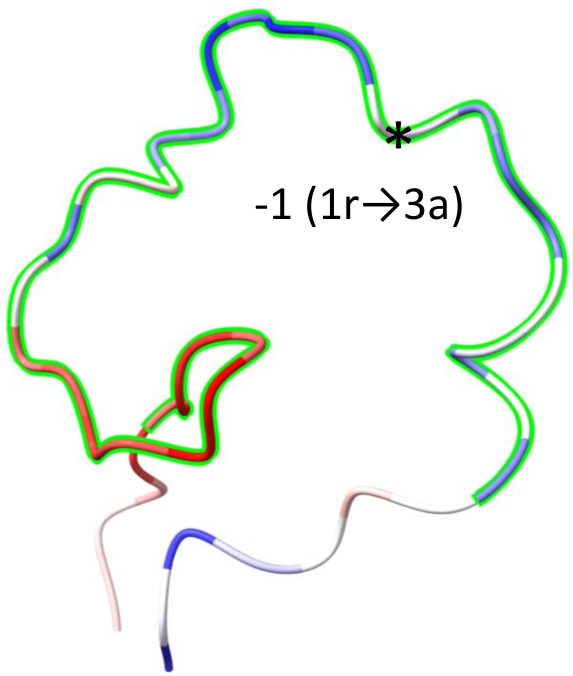

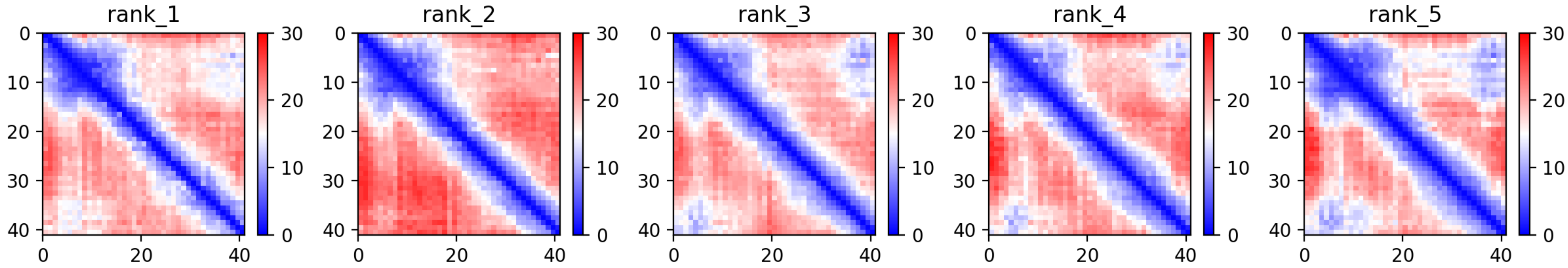

CP96: MtrunA17_Chr6g0452781

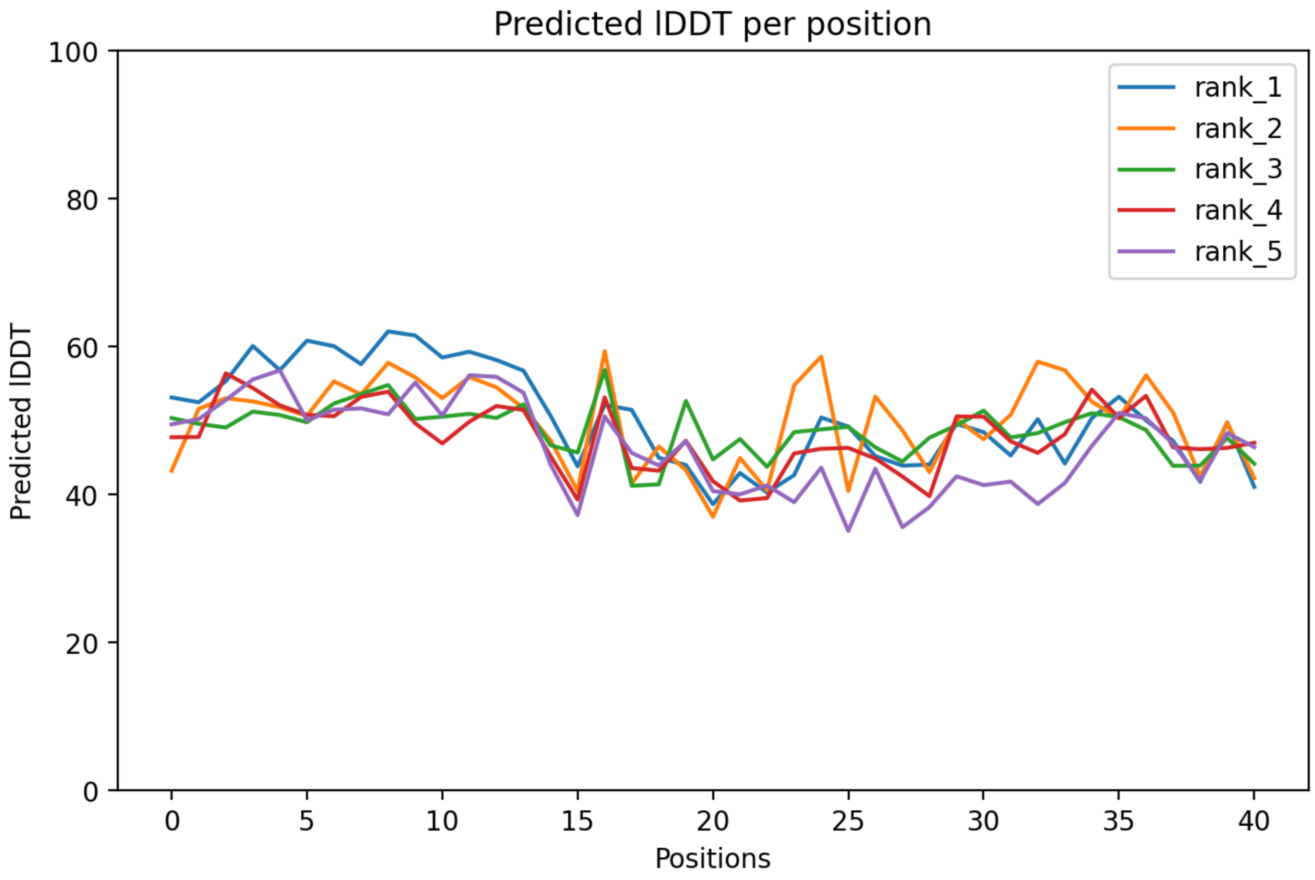

CP96: MtrunA17_Chr6g0452781

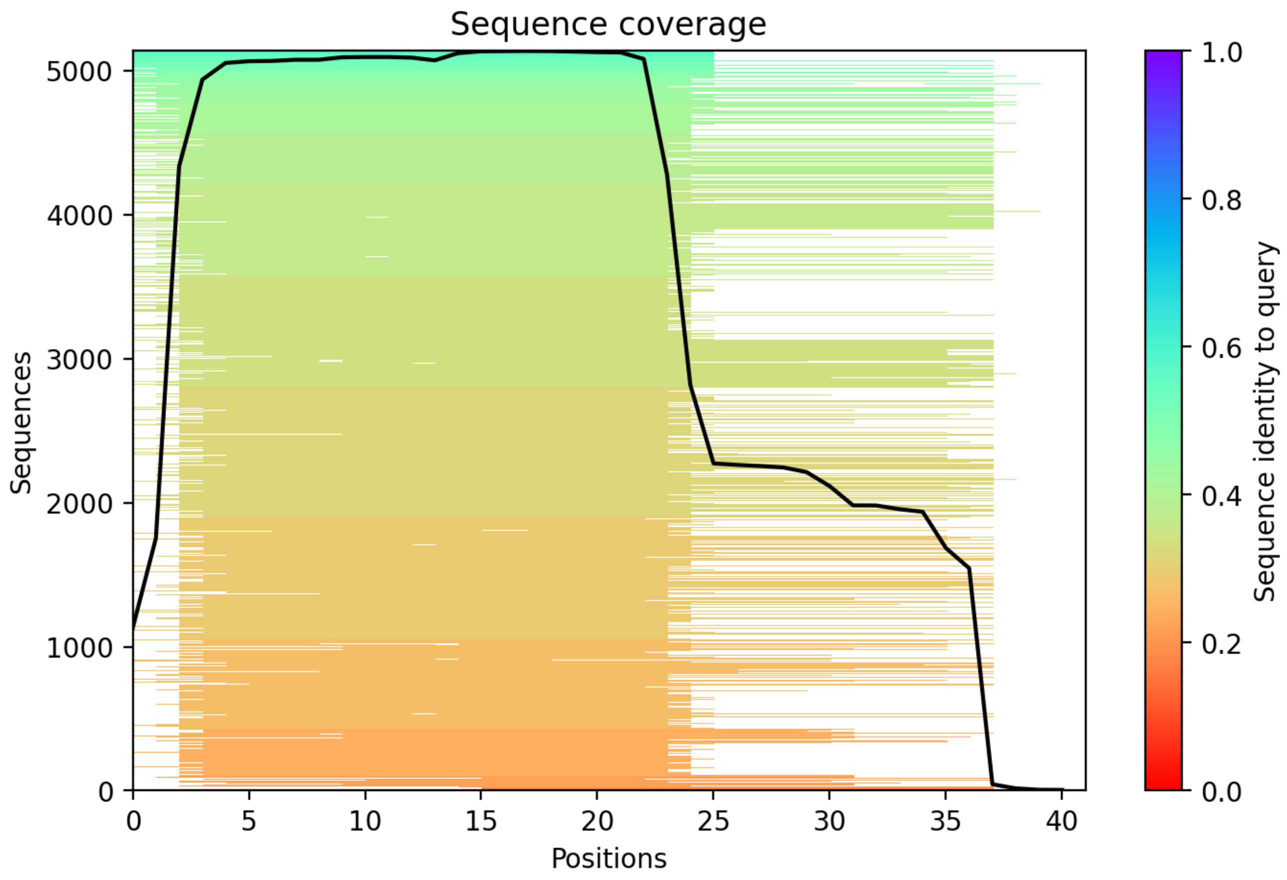

CP97: MtrunA17_Chr6g0457351

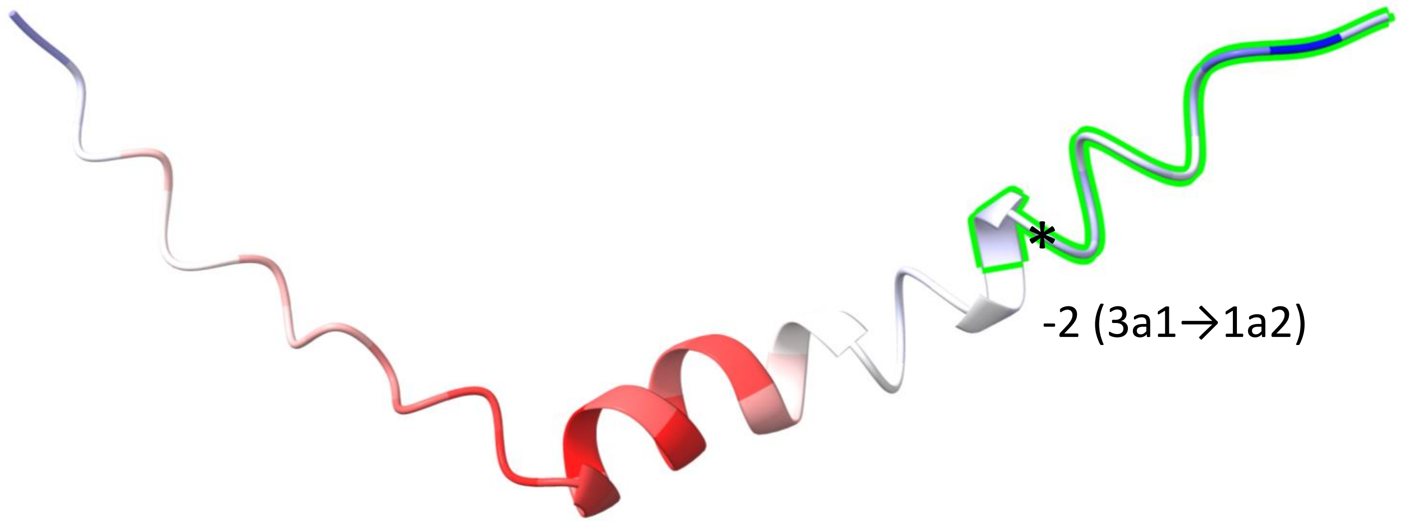

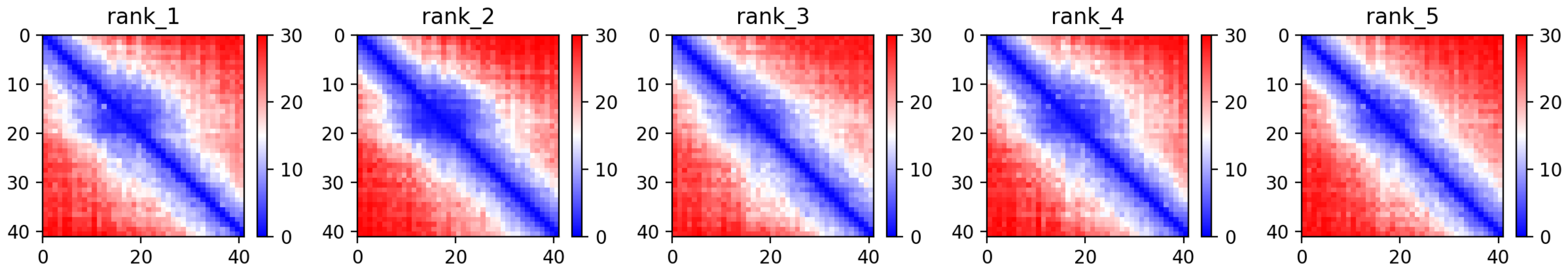

CP97: MtrunA17_Chr6g0457351

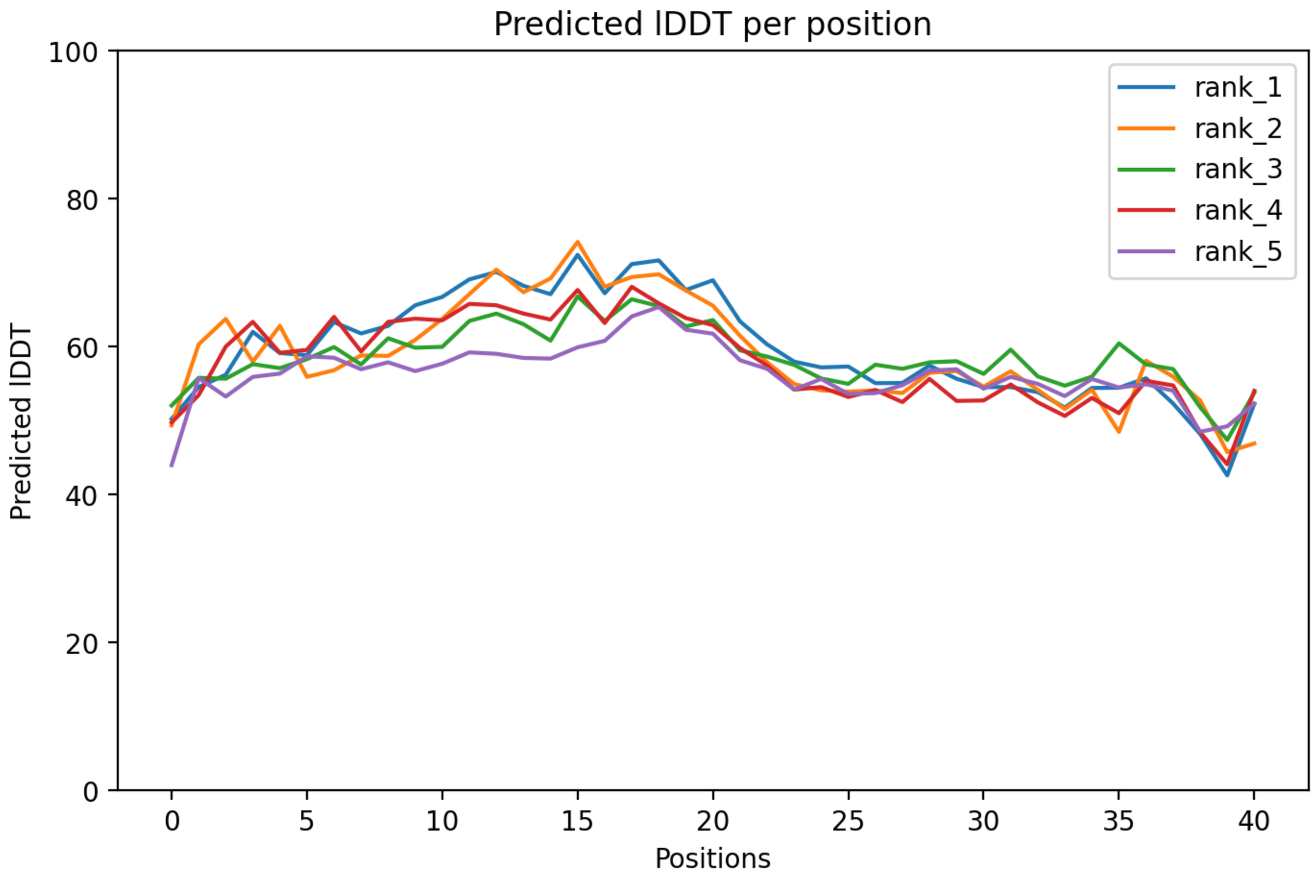

CP97: MtrunA17_Chr6g0457351

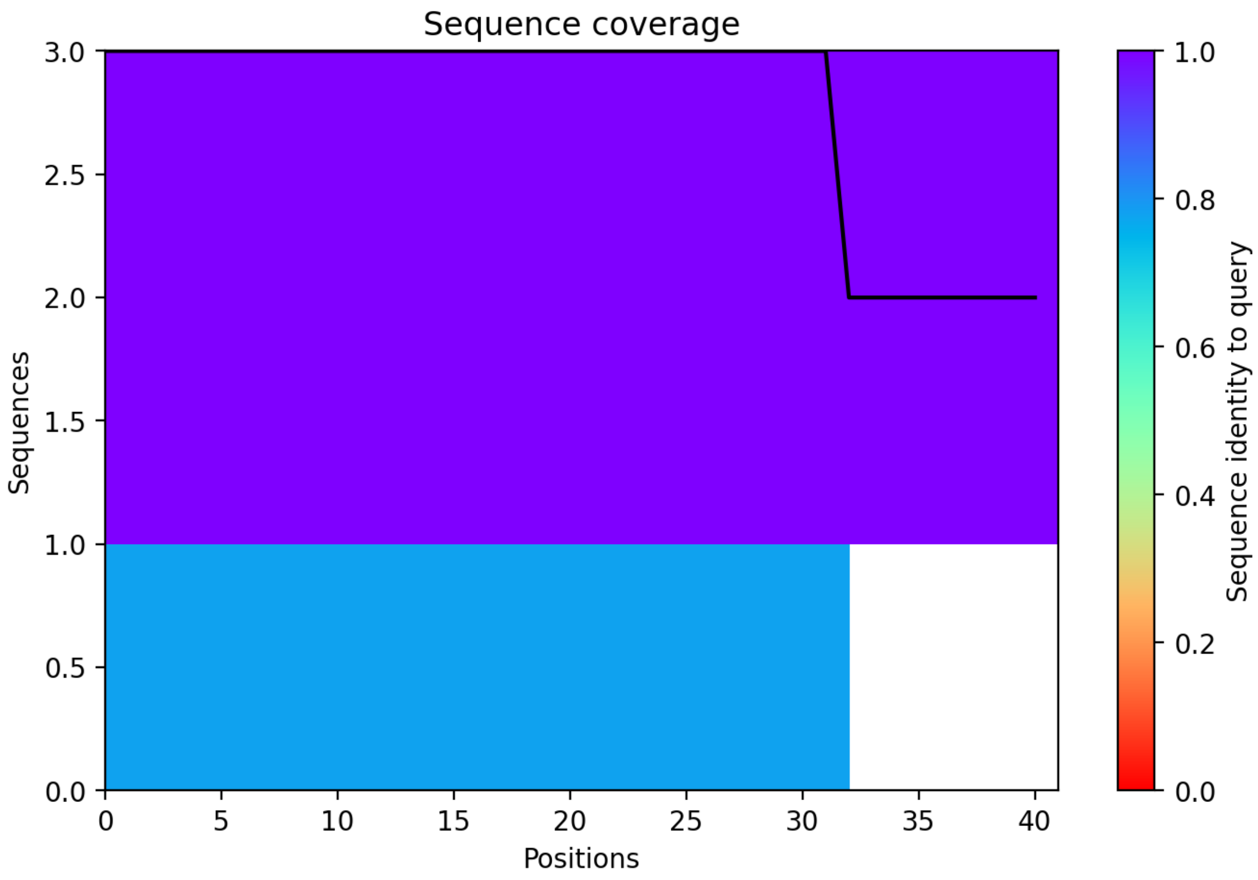

CP98: MtrunA17_Chr6g0457461

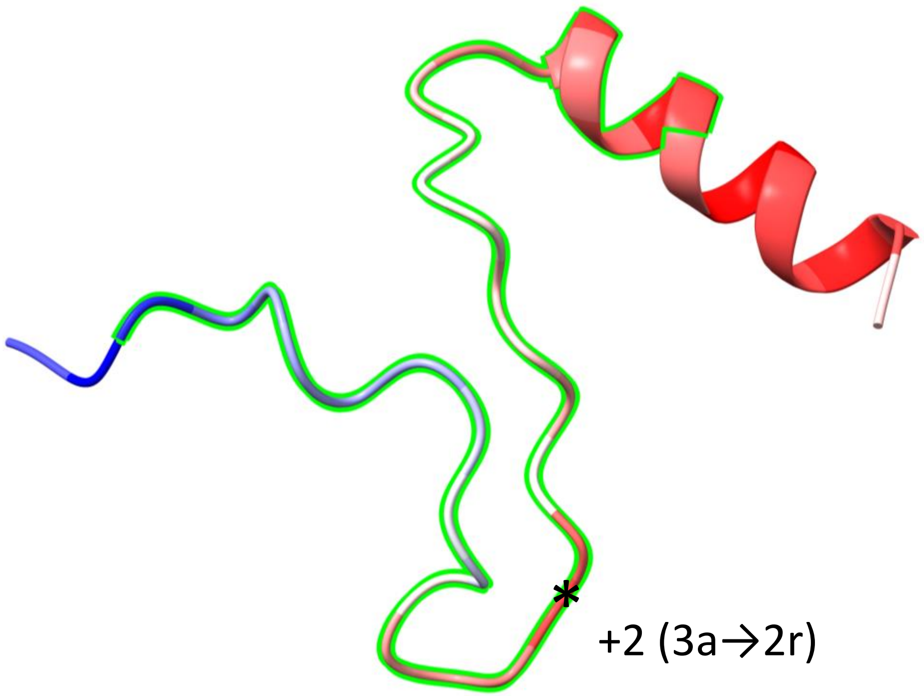

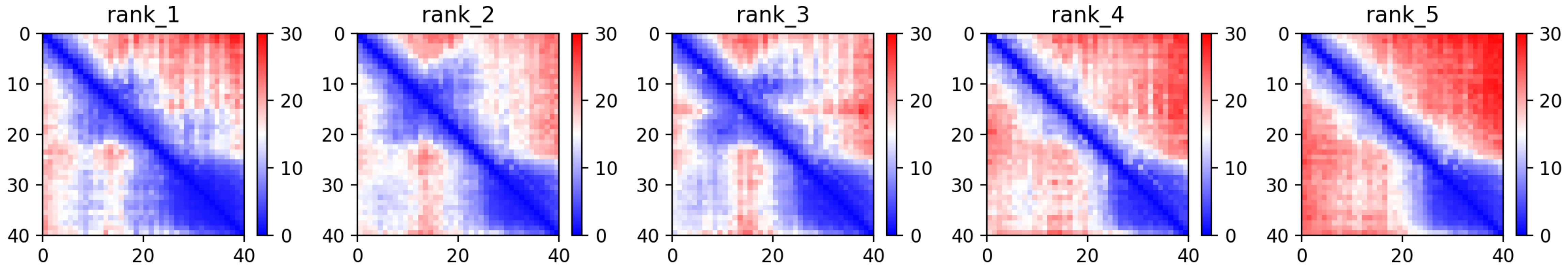

CP98: MtrunA17_Chr6g0457461

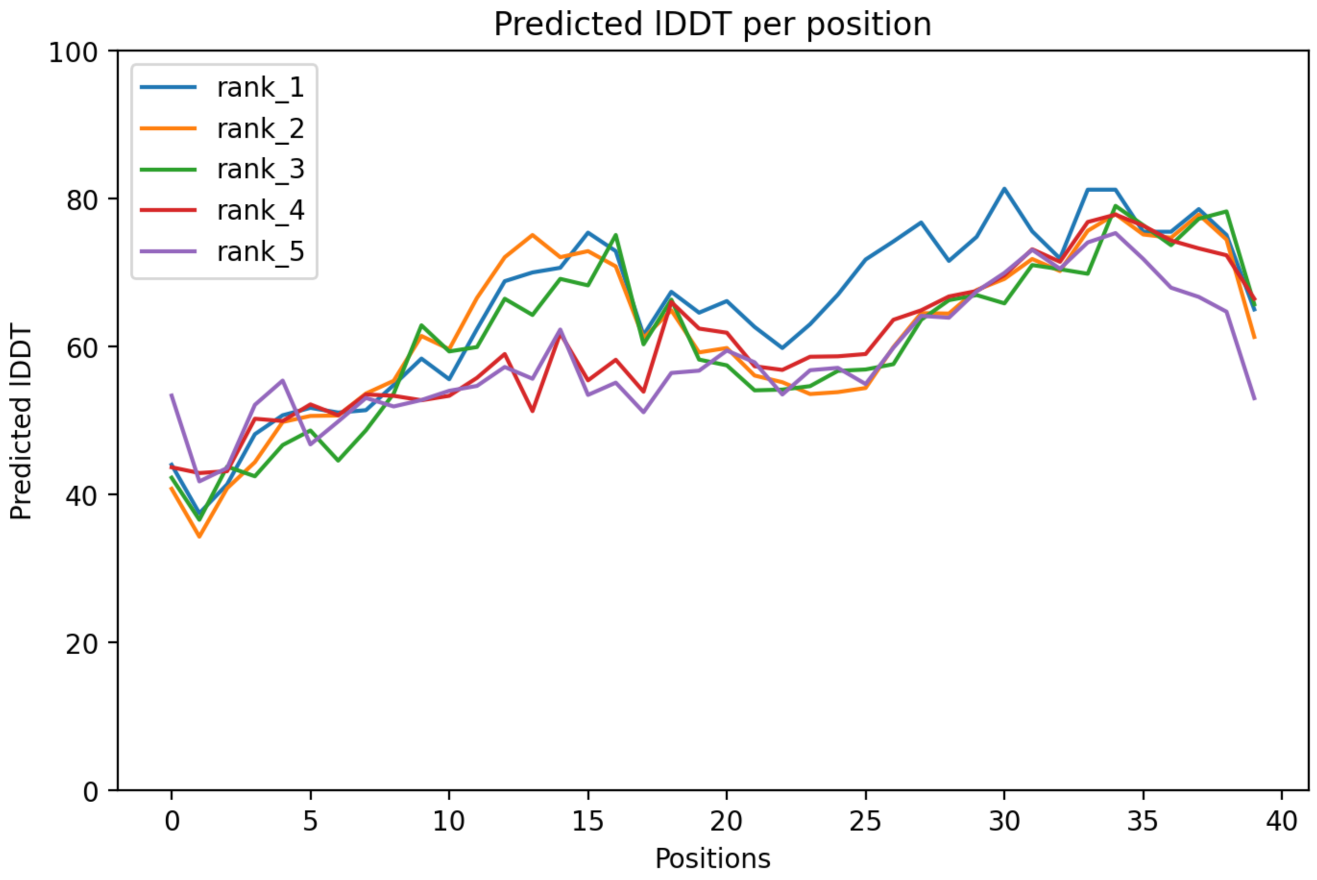

CP98: MtrunA17_Chr6g0457461

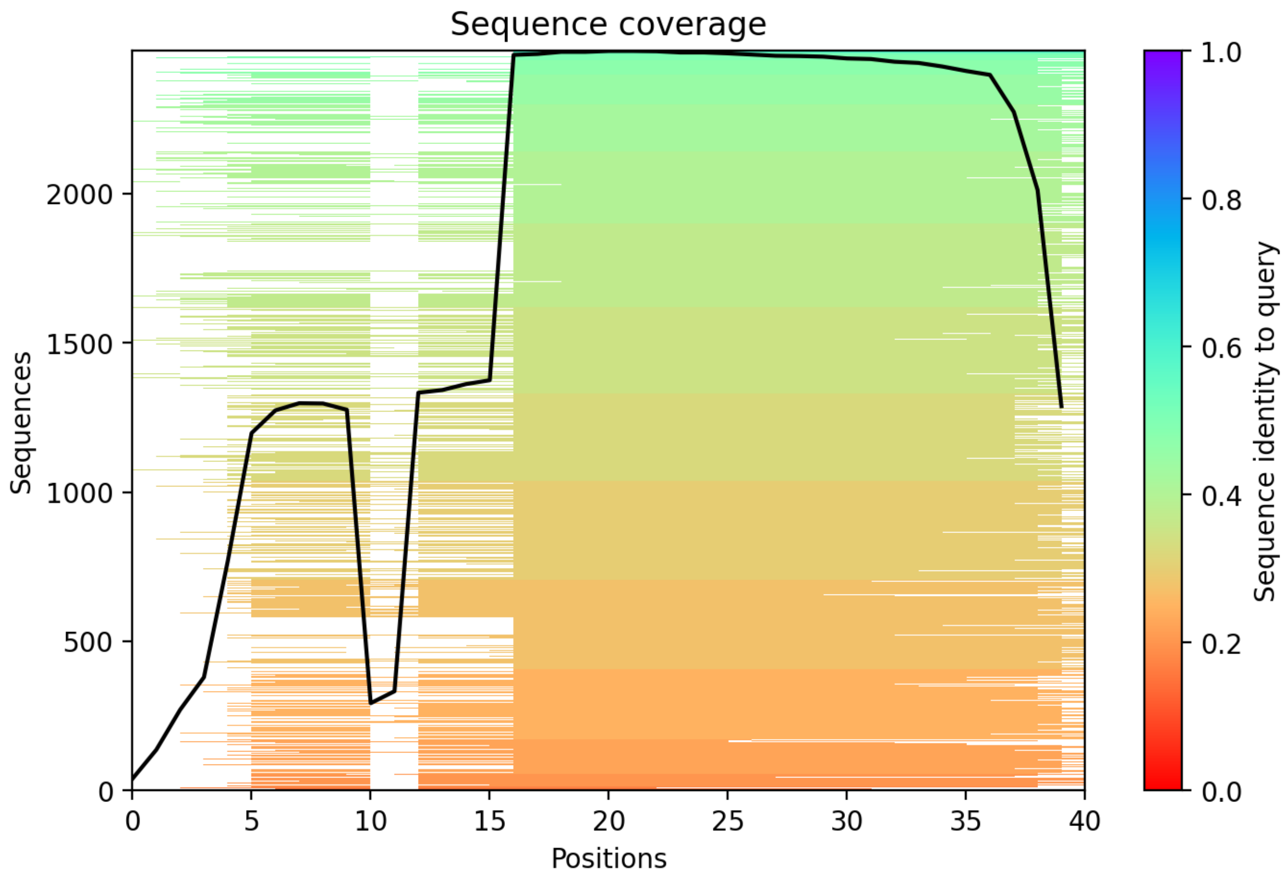

CP99: MtrunA17_Chr6g0457461

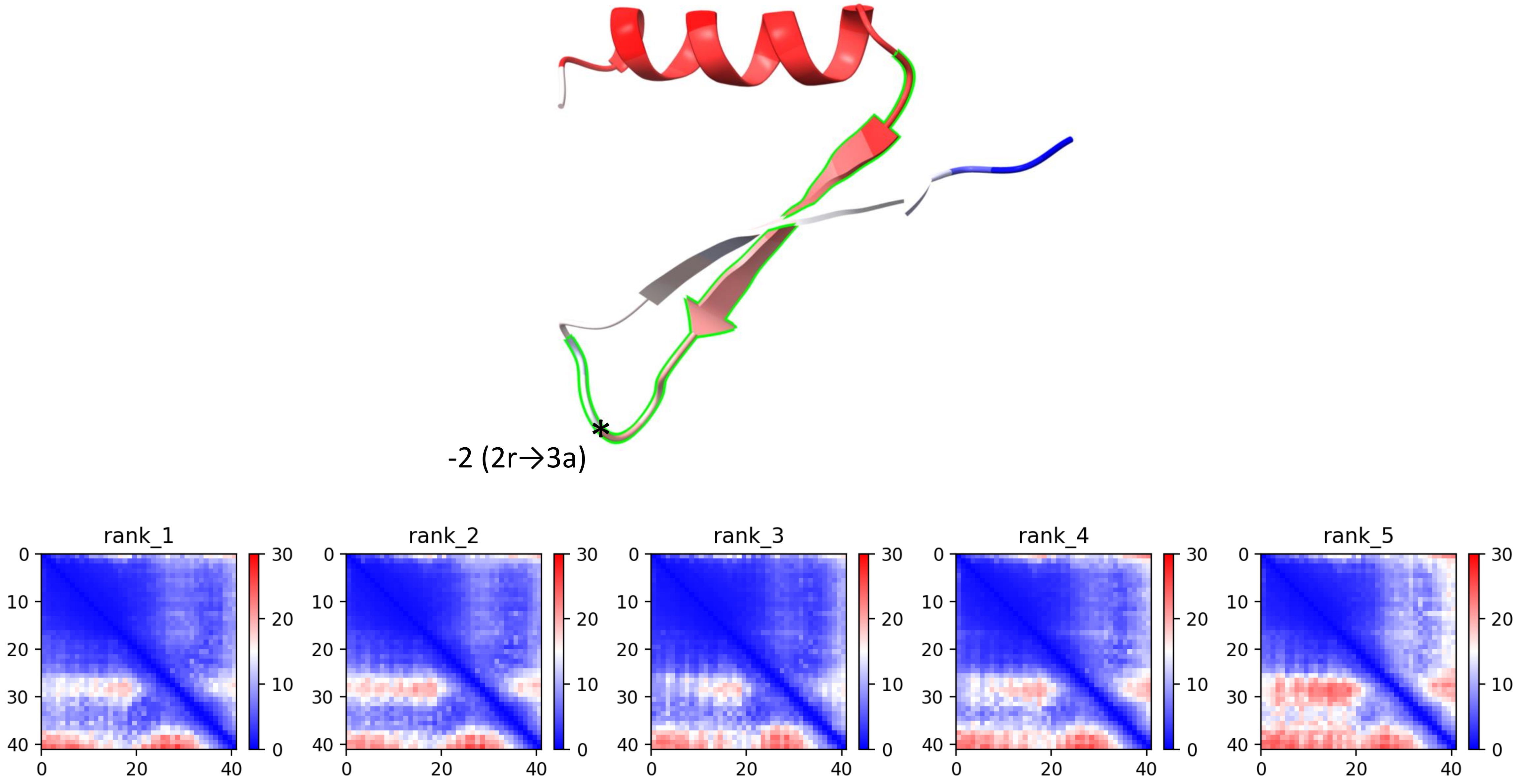

CP99: MtrunA17_Chr6g0457461

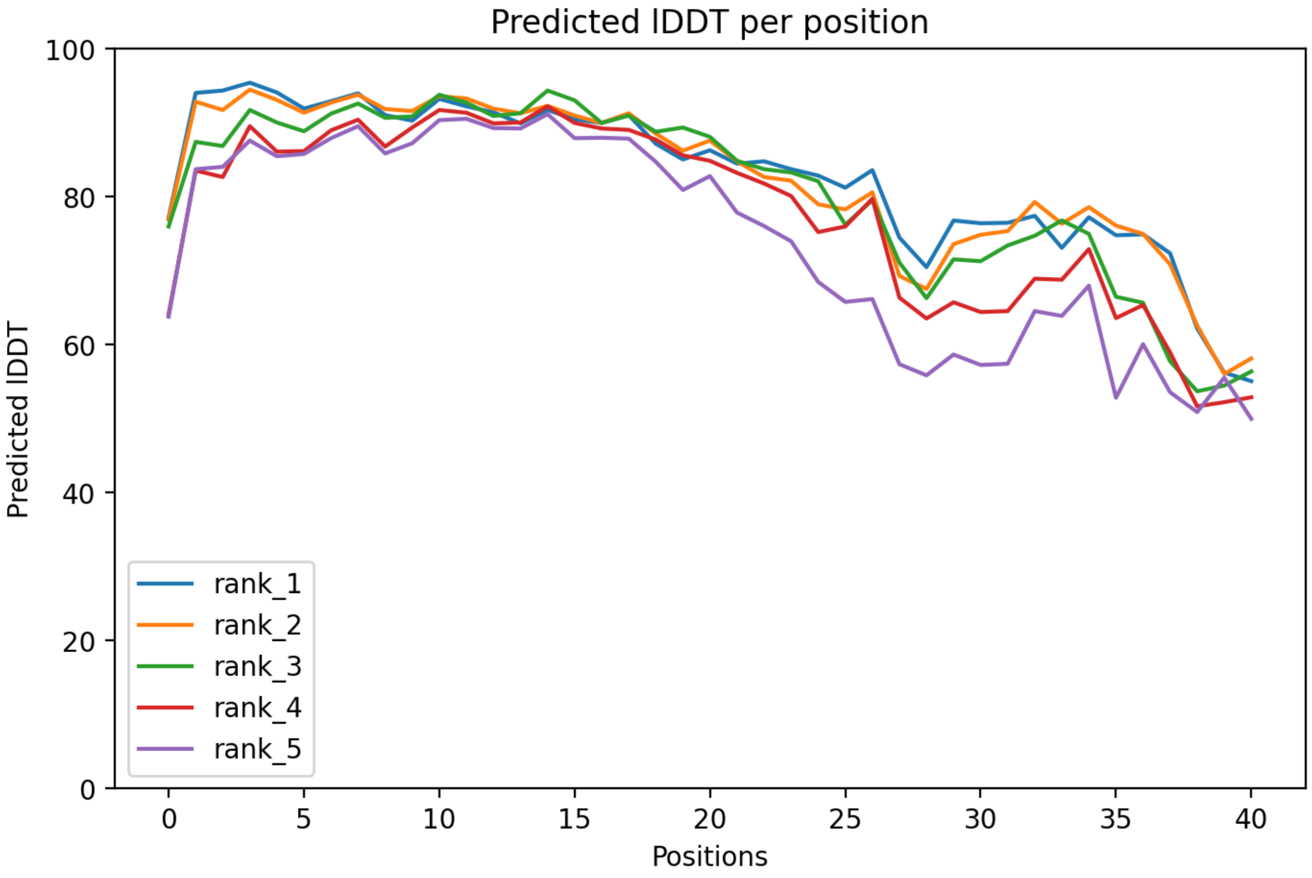

CP99: MtrunA17_Chr6g0457461

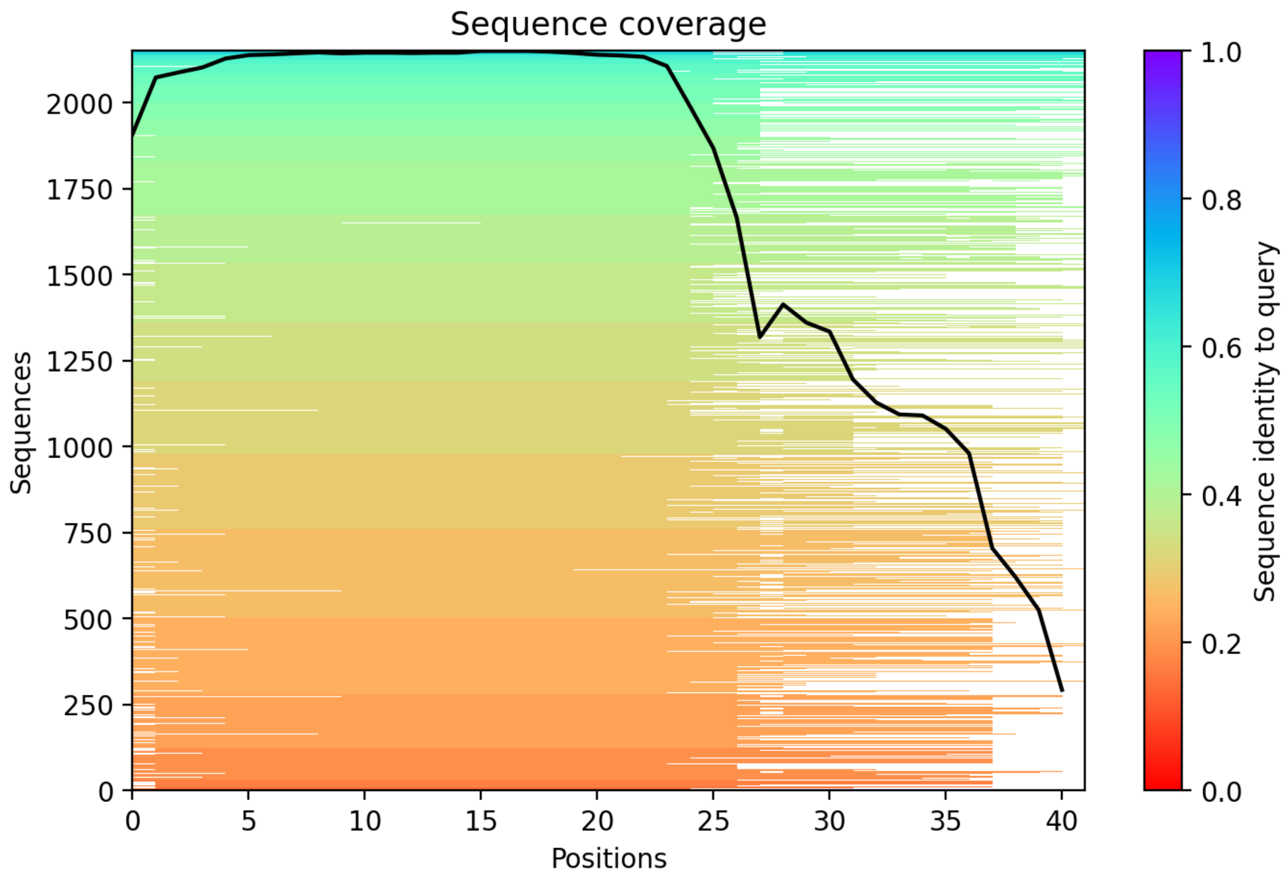

CP100: MtrunA17_Chr6g0457461

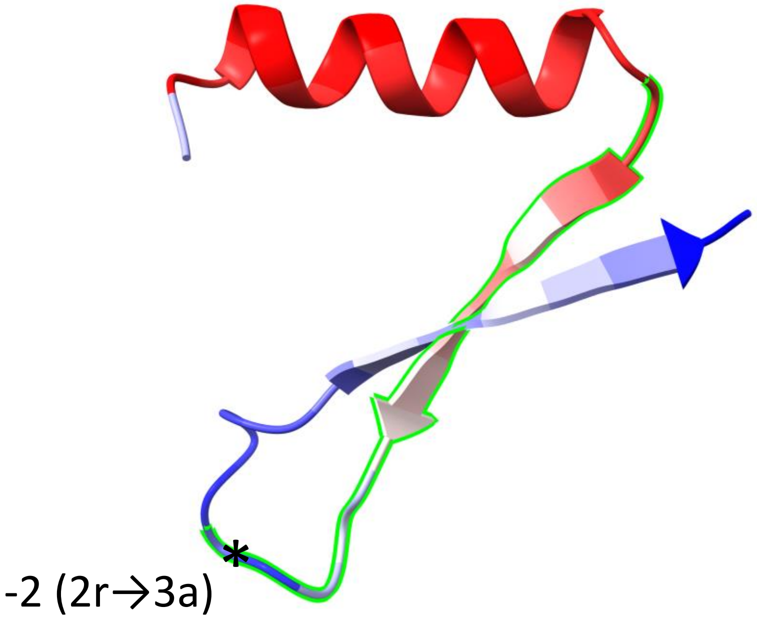

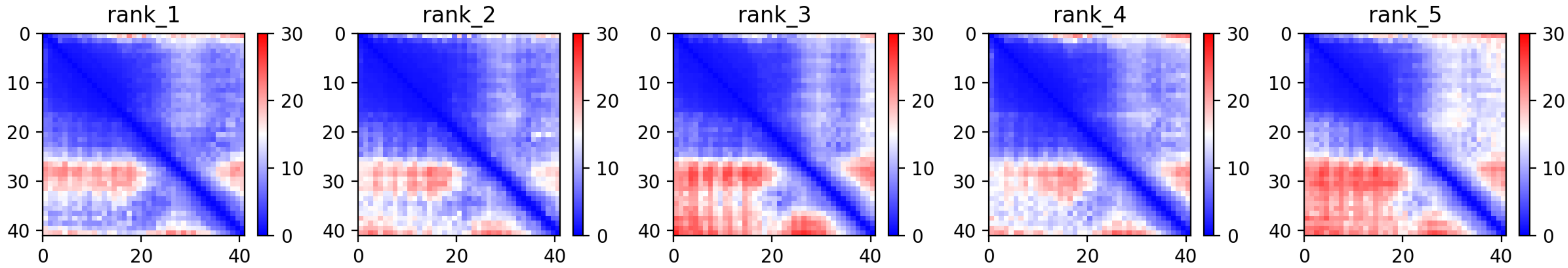

CP100: MtrunA17_Chr6g0457461

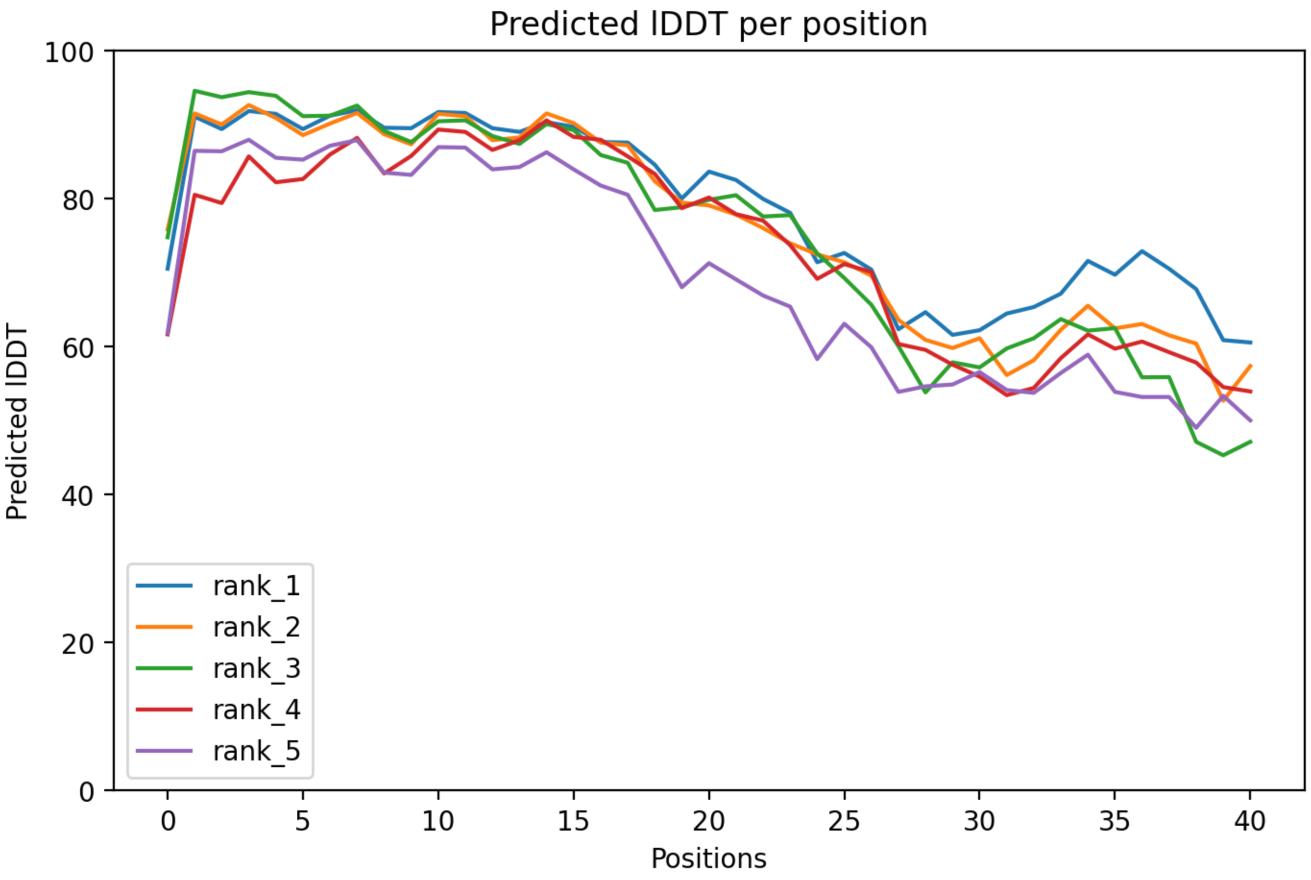

CP100: MtrunA17_Chr6g0457461

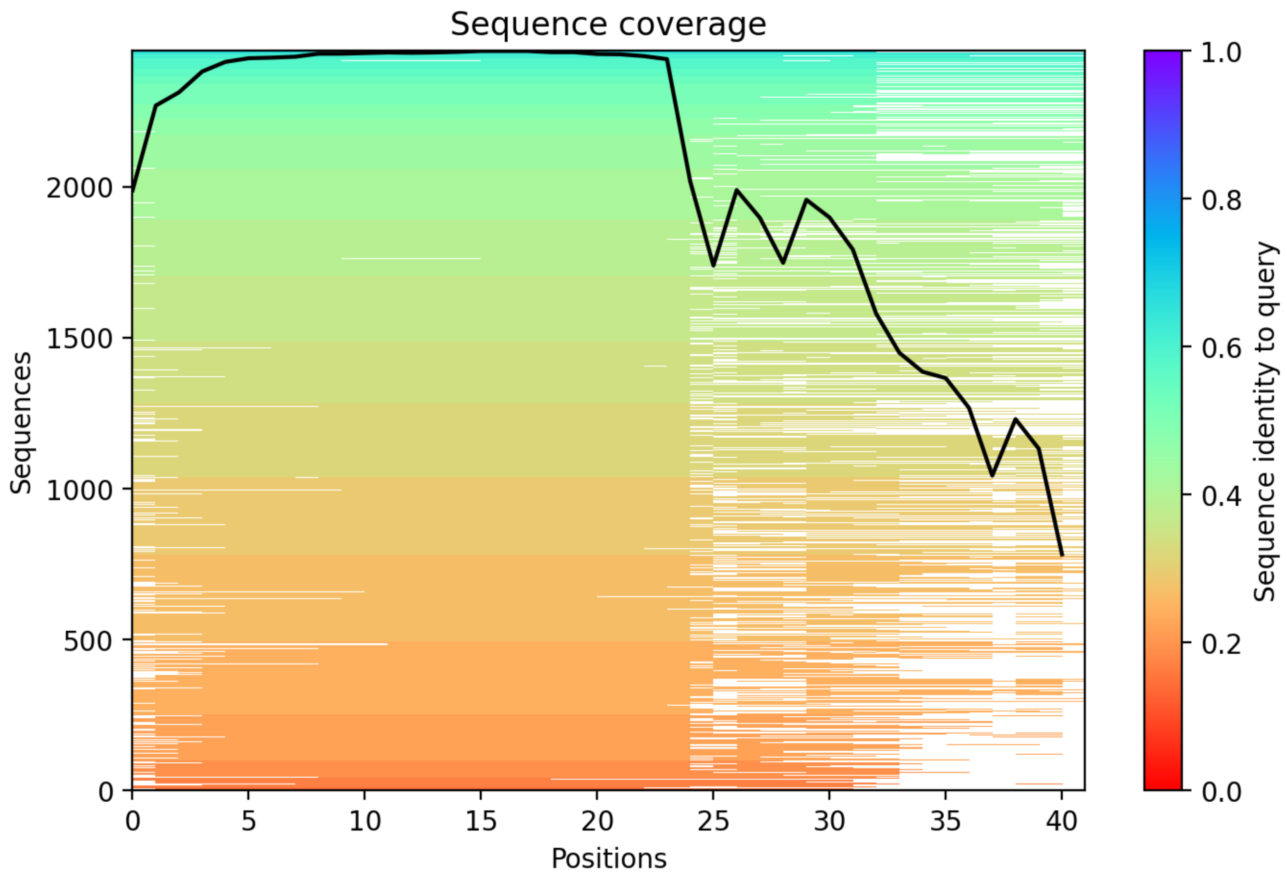

Supplementary Dataset S7 Part 2. A graphical summary on folding predictions for MS-supported chimeric peptide models (CPs) 51-100. The rest of the legend is the same as for Supplementary Dataset S7 Part 1.
