# Supplementary material for: Discovery of diverse chimeric peptides in a eukaryotic proteome sets the stage for experimental validation of the mosaic translation hypothesis
Source: Comput Struct Biotechnol J. 2025 Sep 12;27:4048–64. doi: 10.1016/j.csbj.2025.09.019 (PMC12481079; doi:10.1016/j.csbj.2025.09.019)
Supplement: Supplementary file 1 — Supplementary material [file mmc1.zip › Supplementary Datasets/Supplementary Dataset S7 Folding of chimeric protein models Part 3.pdf]

CP101: MtrunA17_Chr6g0458091

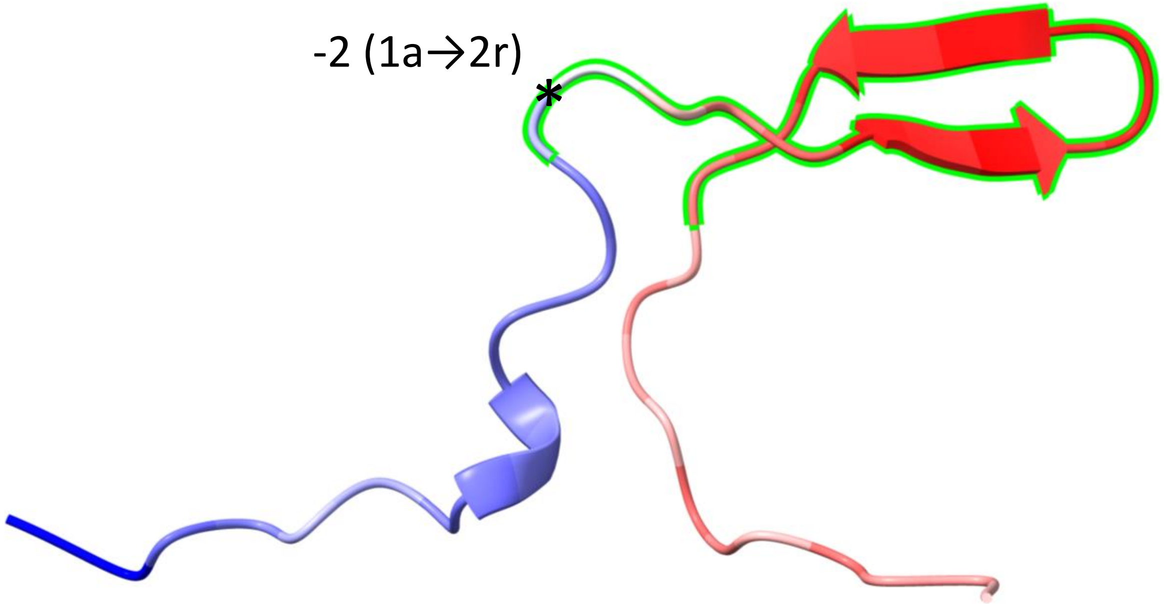

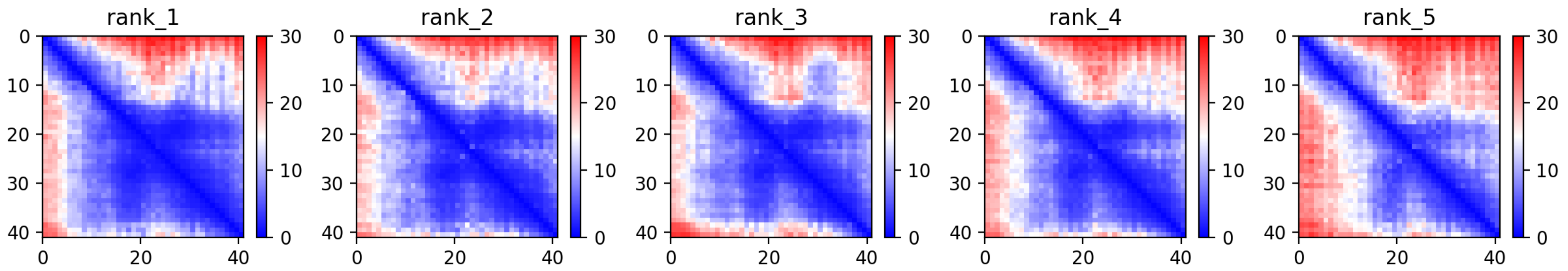

CP101: MtrunA17_Chr6g0458091

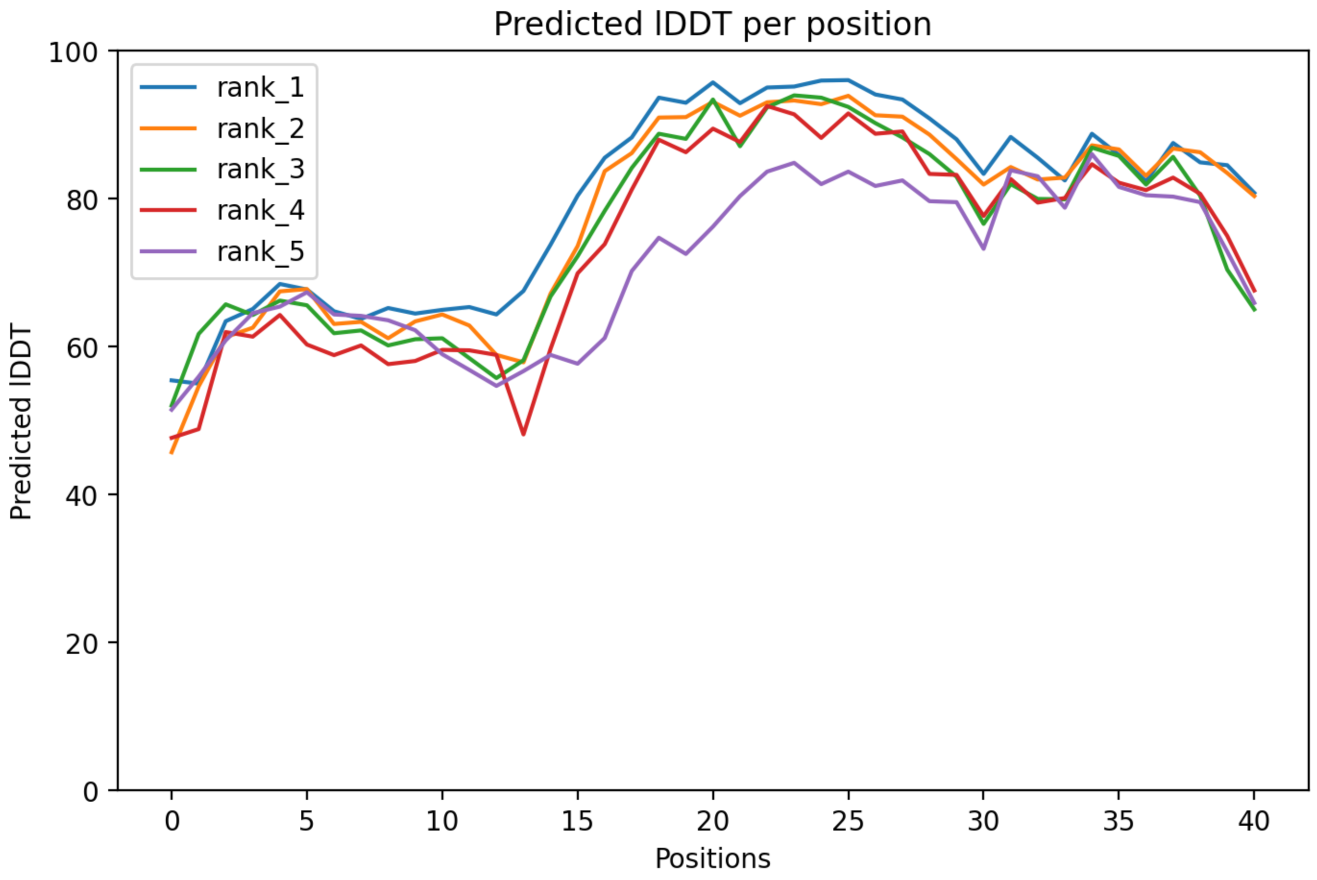

CP101: MtrunA17_Chr6g0458091

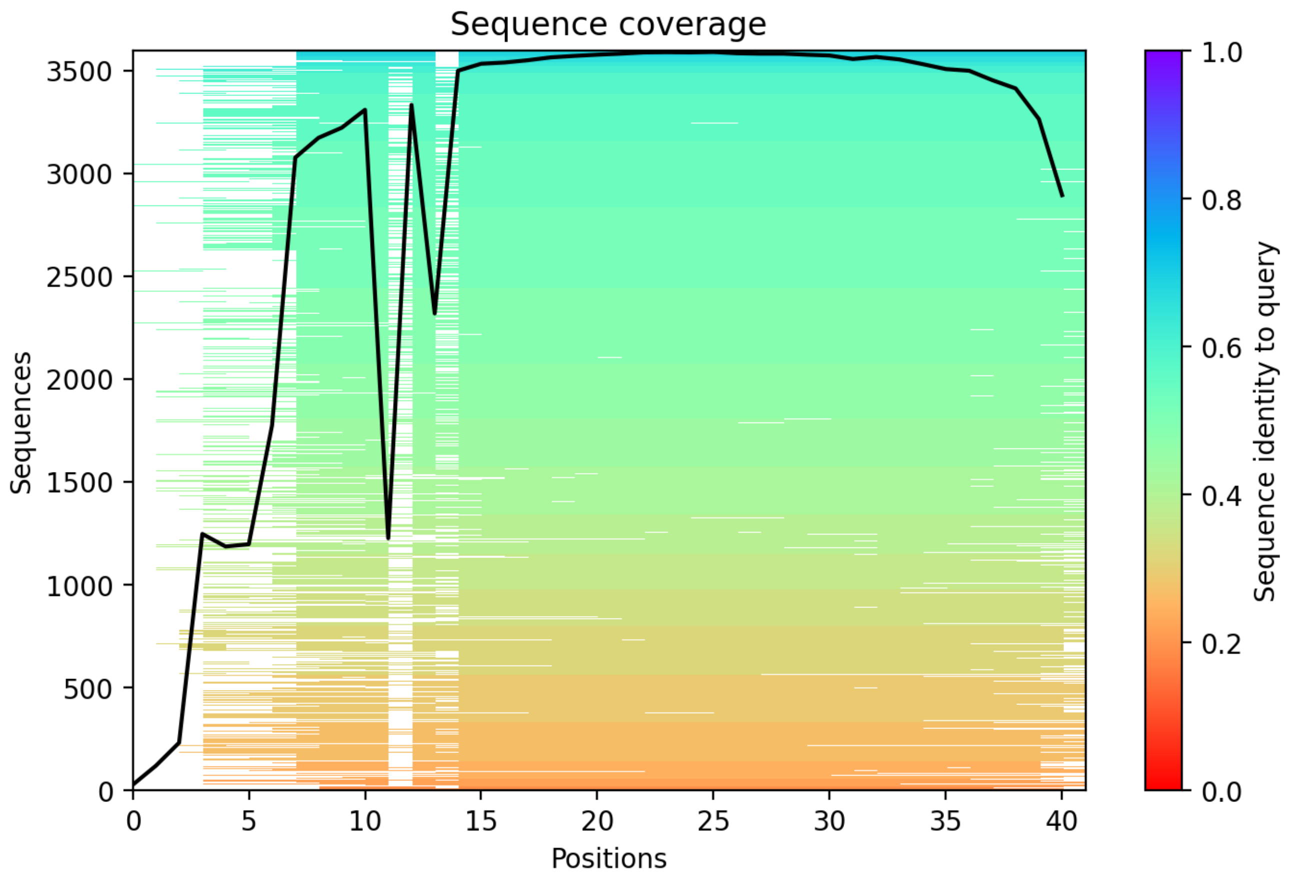

CP102: MtrunA17_Chr6g0459481

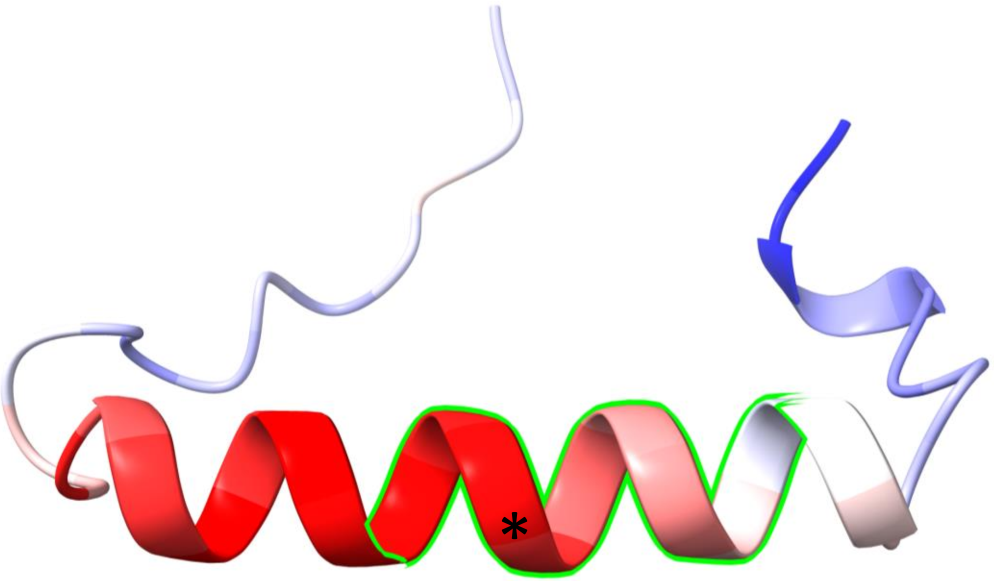

-1 (1r→3a)

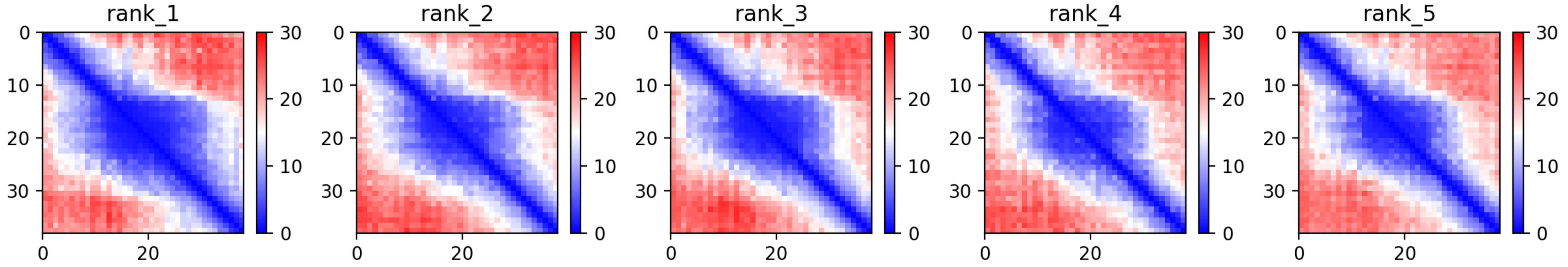

CP102: MtrunA17_Chr6g0459481

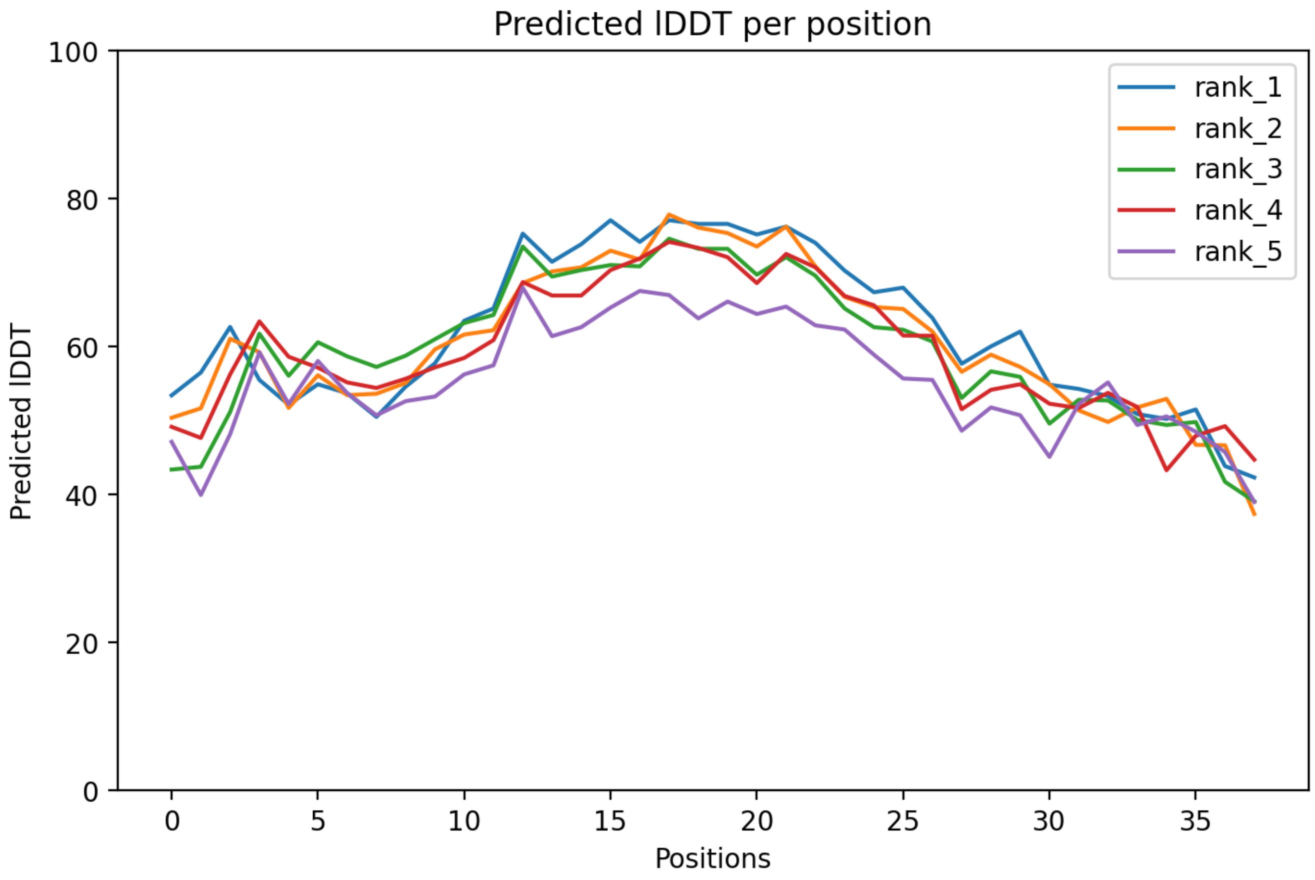

CP102: MtrunA17_Chr6g0459481

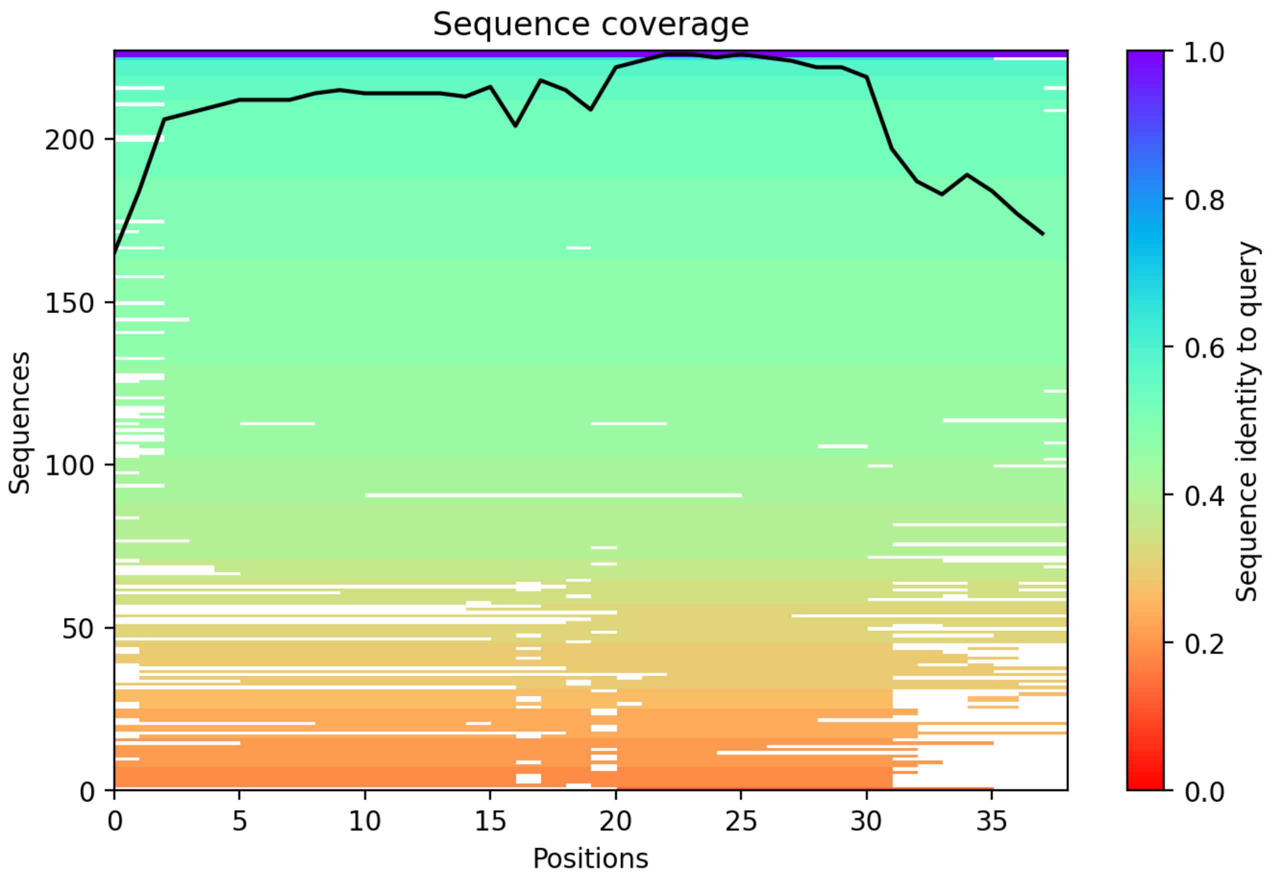

CP103: MtrunA17_Chr6g0461931

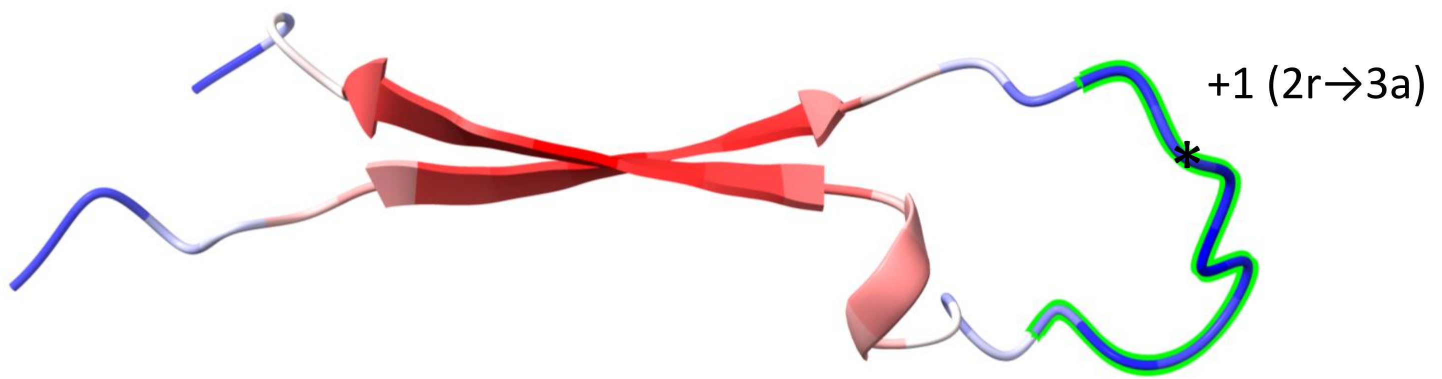

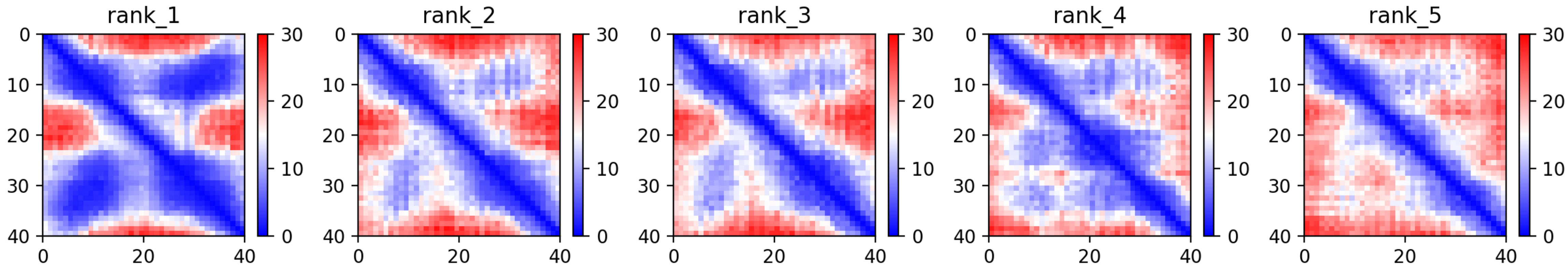

CP103: MtrunA17_Chr6g0461931

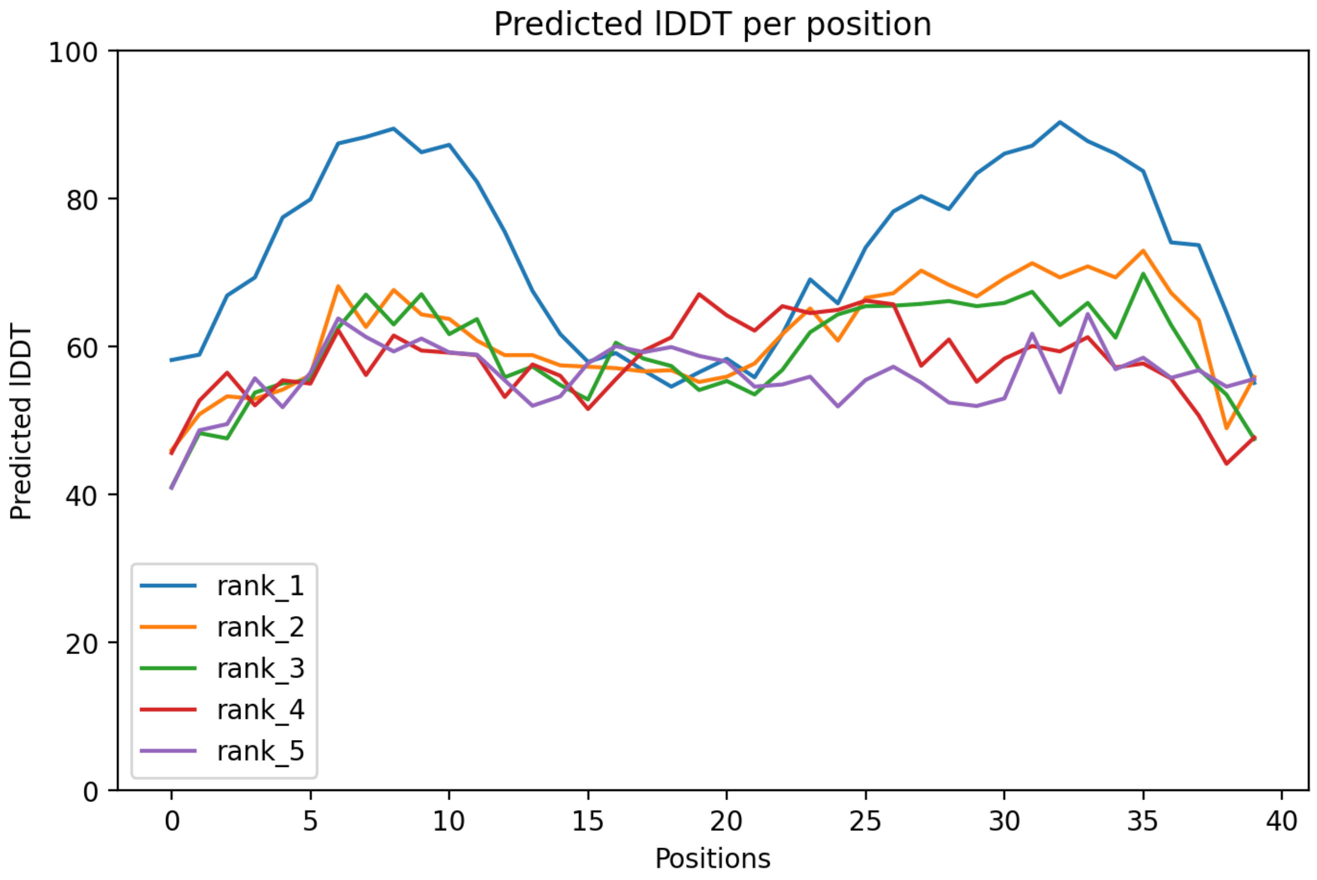

CP103: MtrunA17_Chr6g0461931

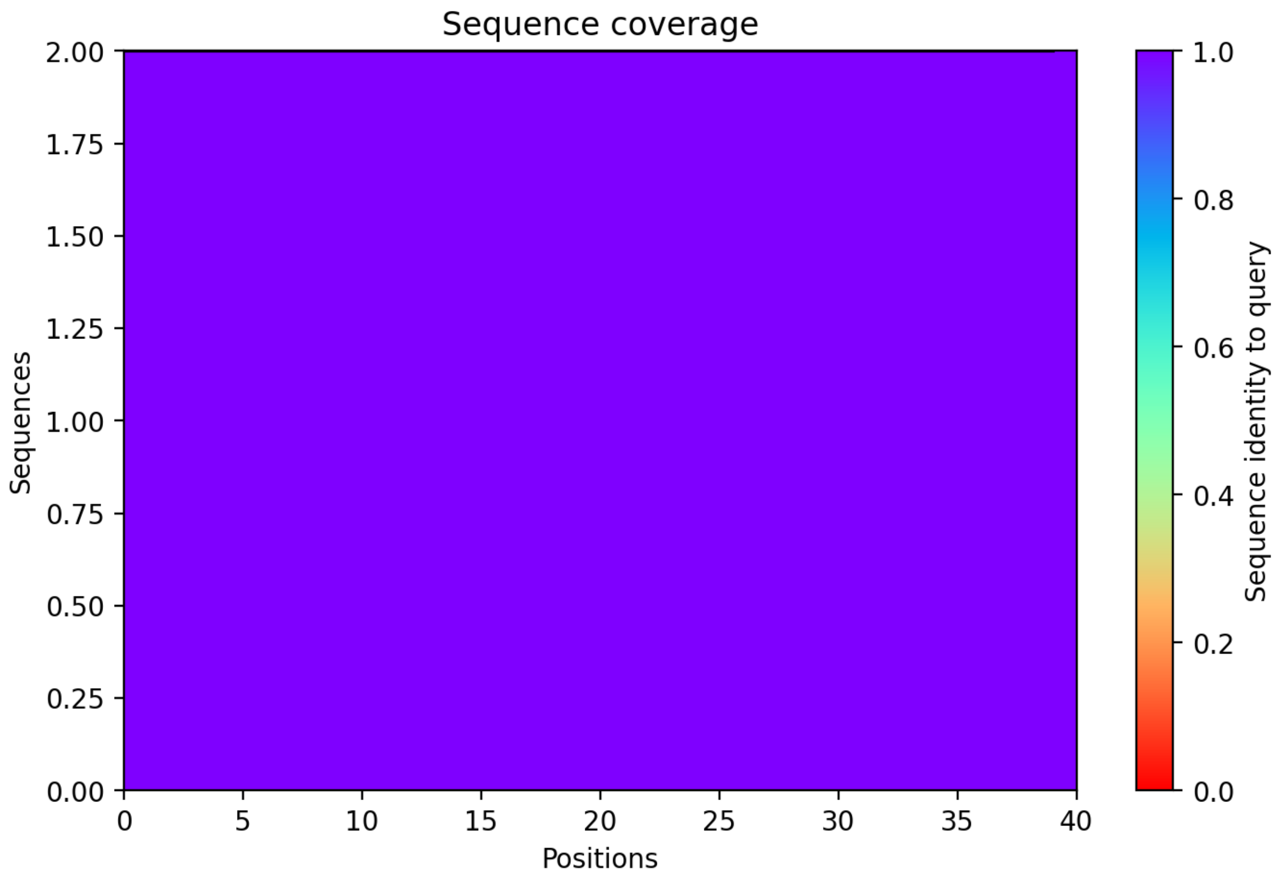

CP104: MtrunA17_Chr6g0462271

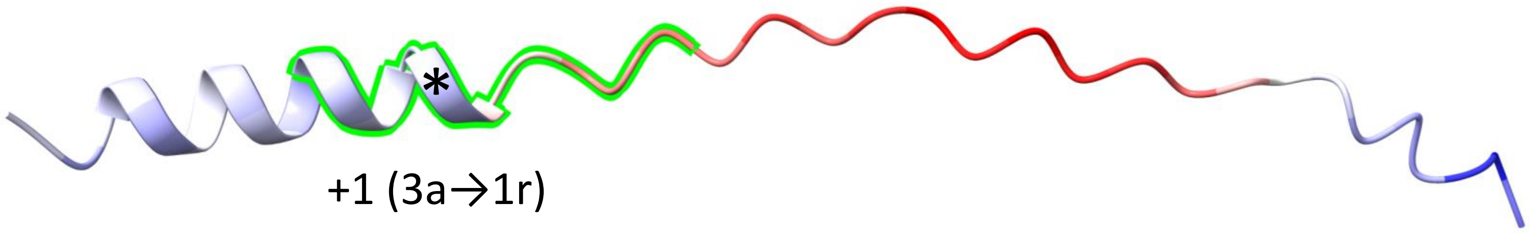

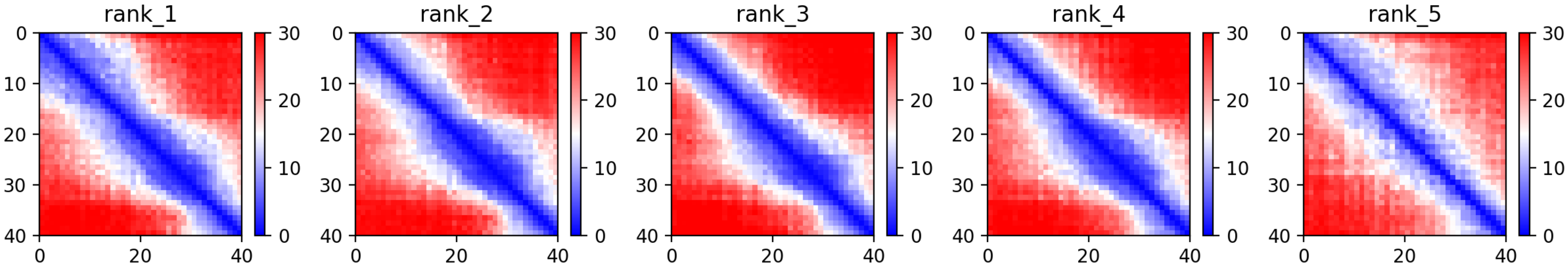

CP104: MtrunA17_Chr6g0462271

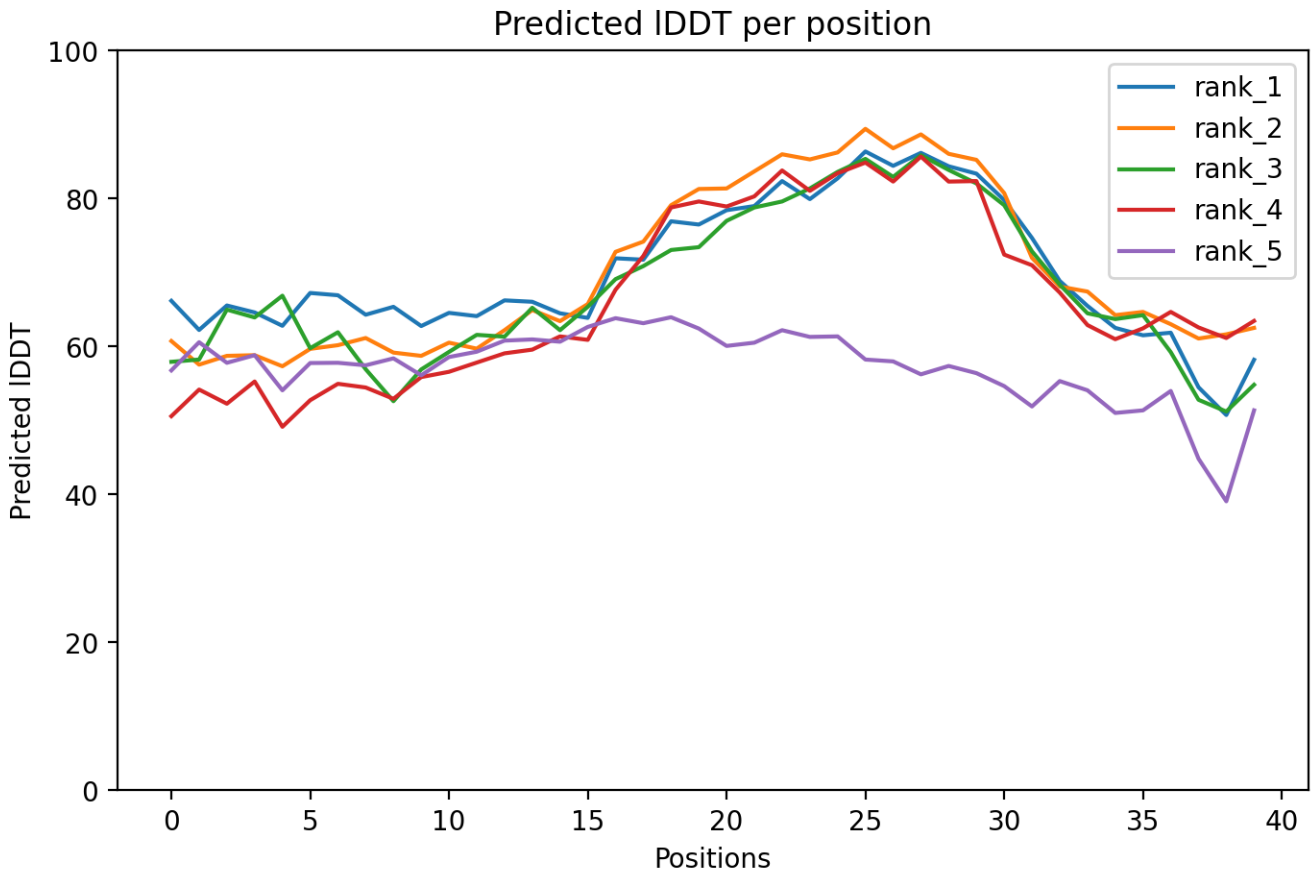

CP104: MtrunA17_Chr6g0462271

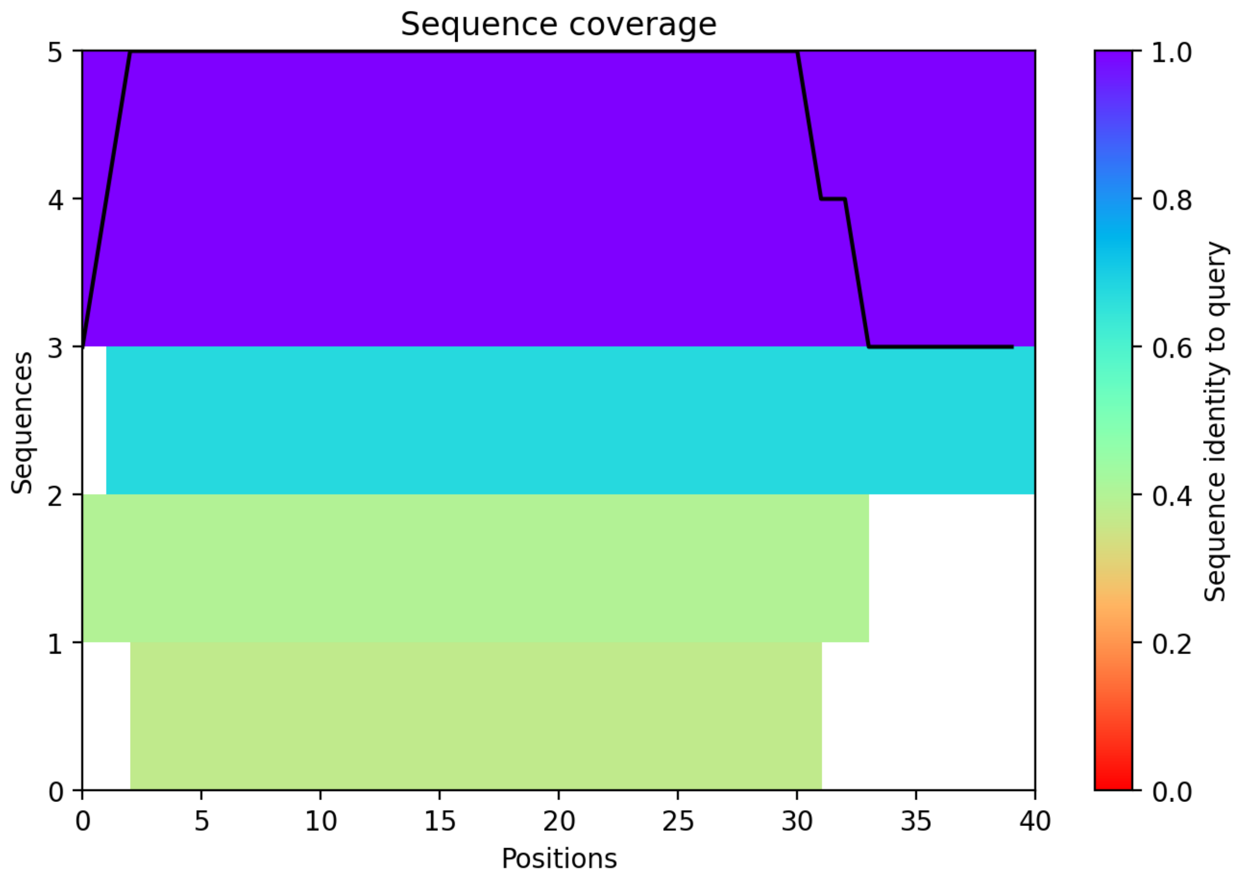

CP105: MtrunA17_Chr6g0468411

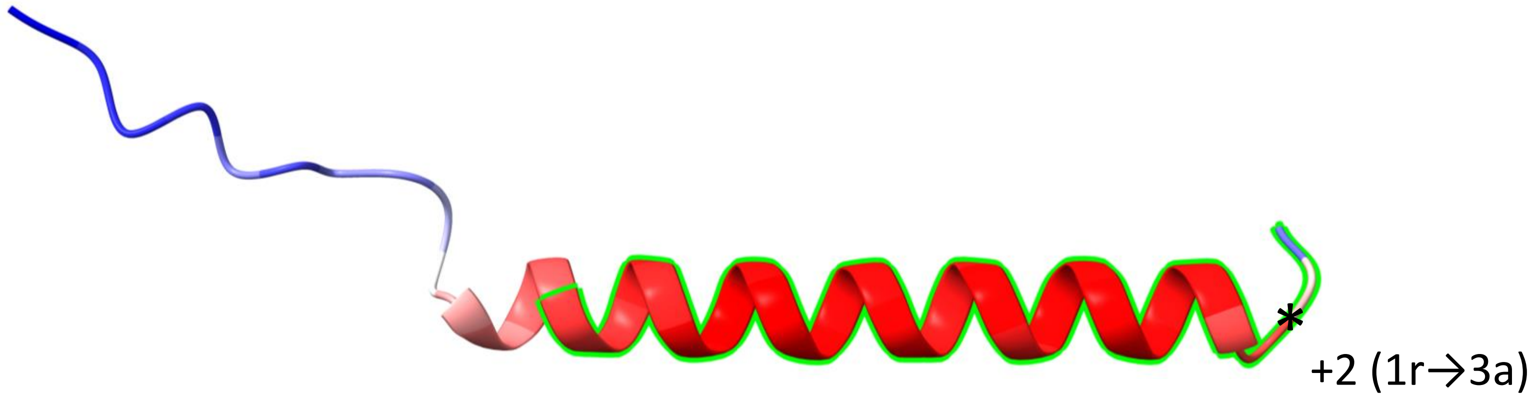

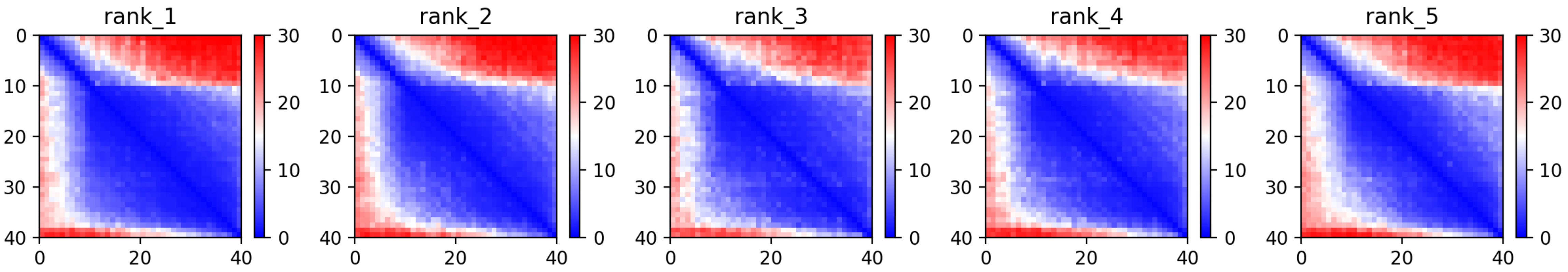

CP105: MtrunA17_Chr6g0468411

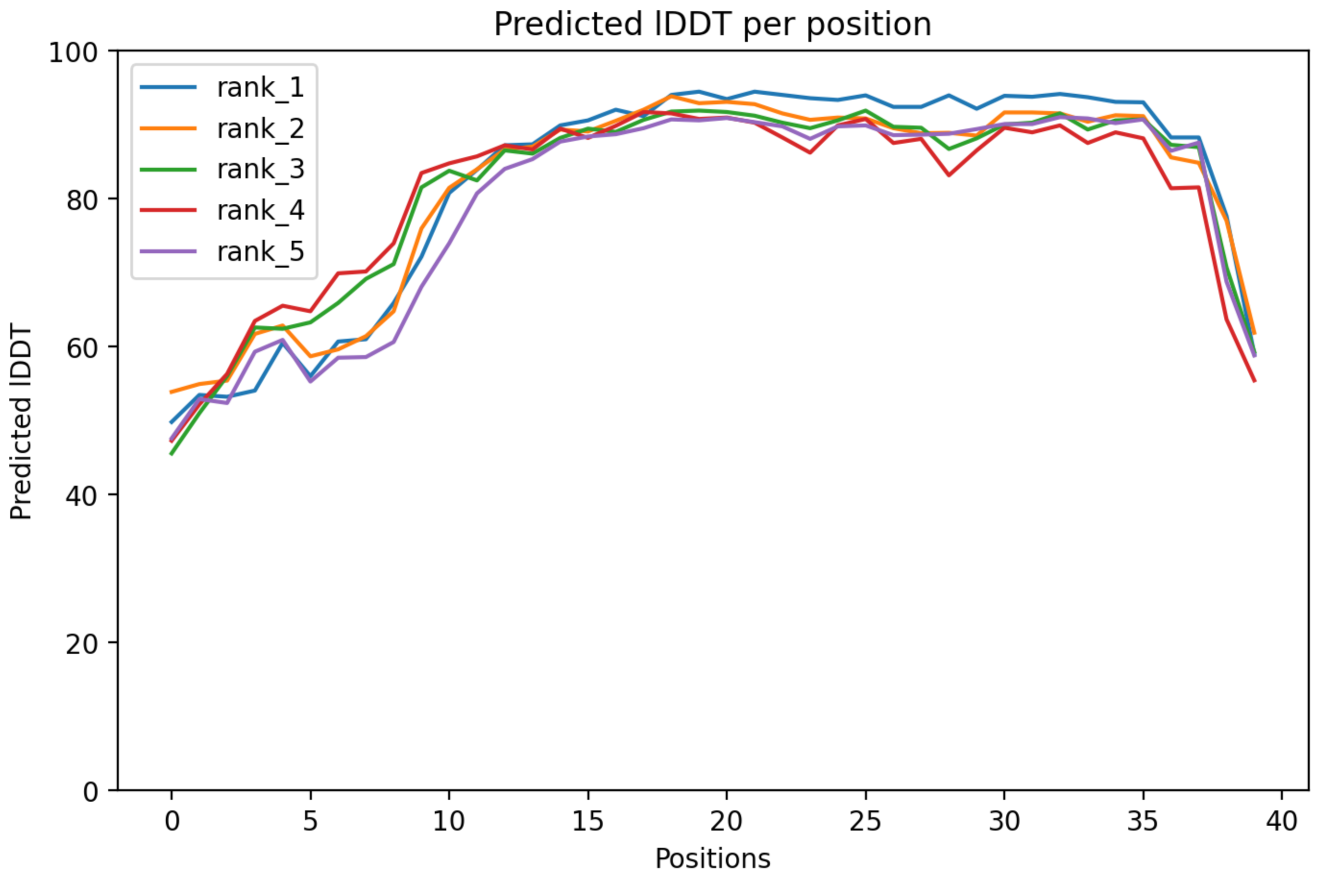

CP105: MtrunA17_Chr6g0468411

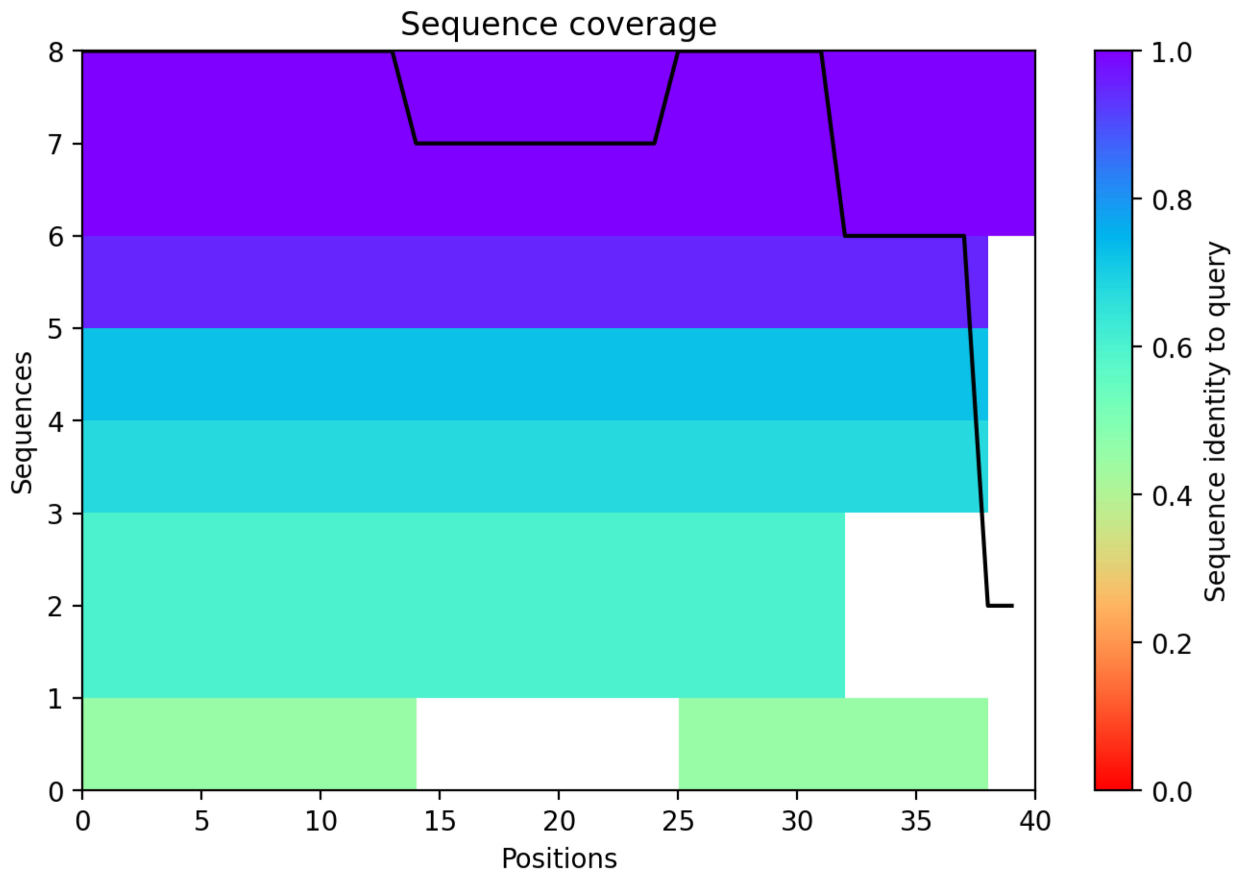

CP106: MtrunA17_Chr6g0476751

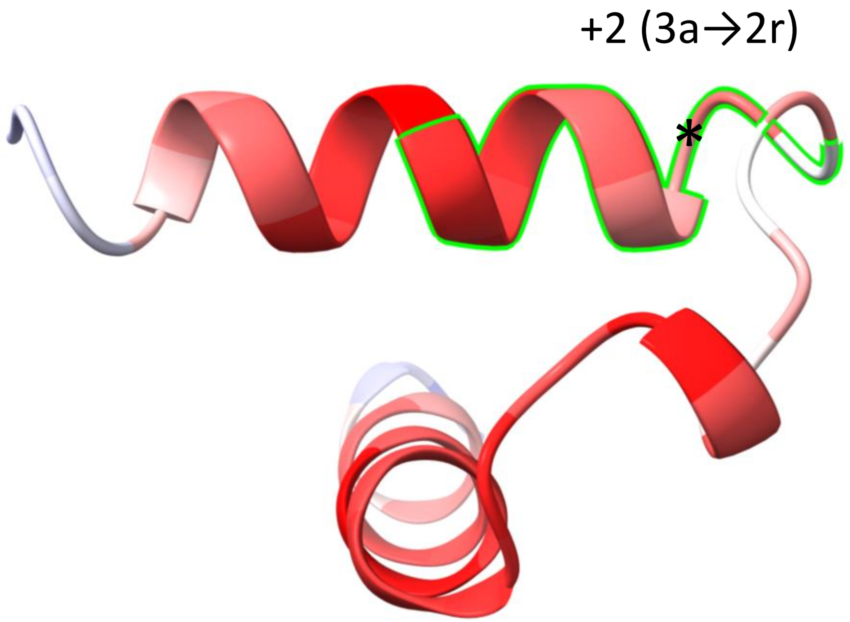

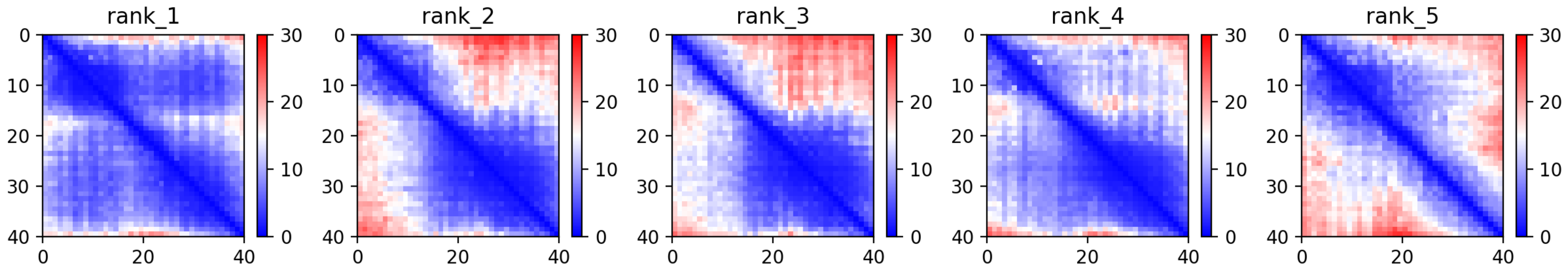

CP106: MtrunA17_Chr6g0476751

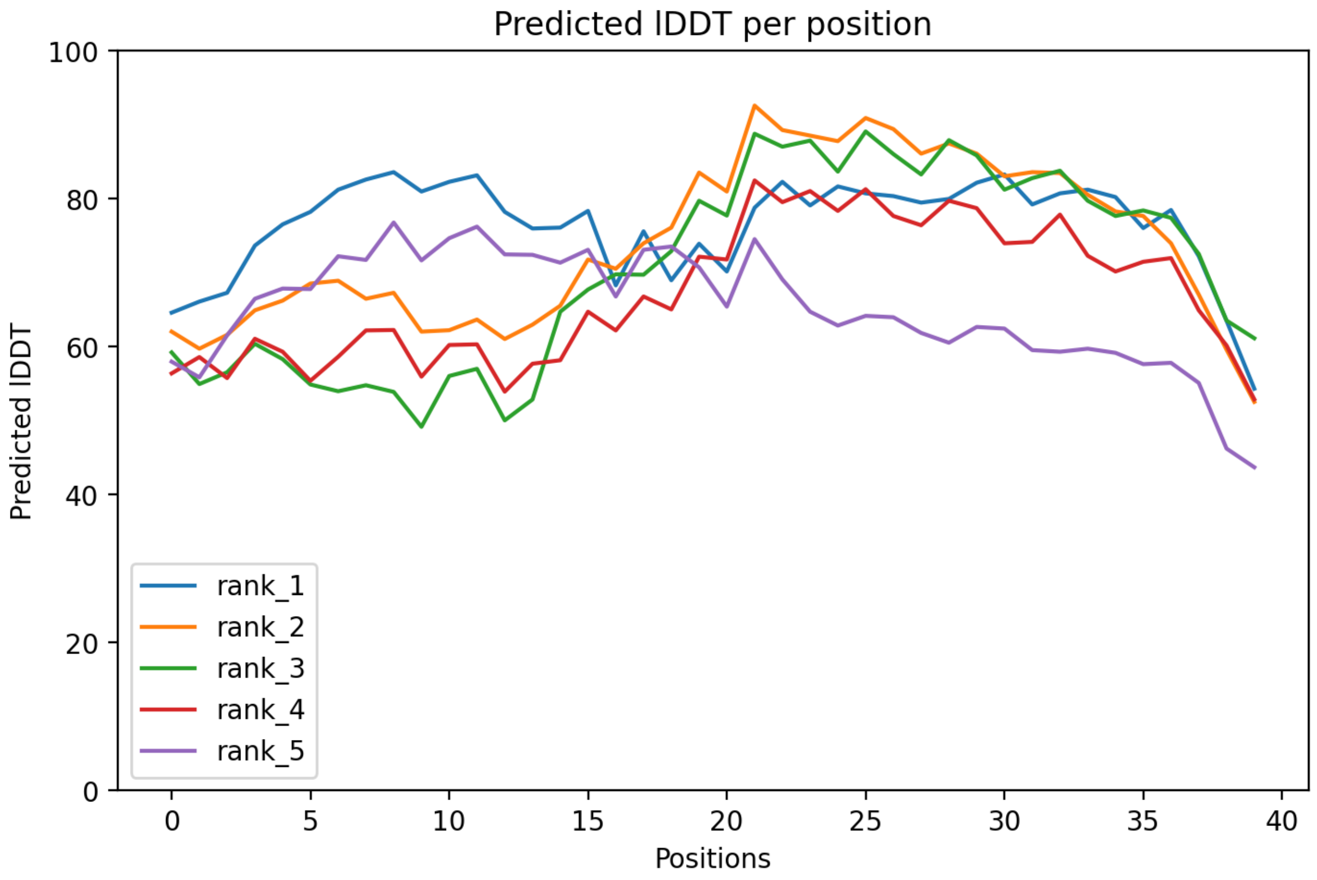

CP106: MtrunA17_Chr6g0476751

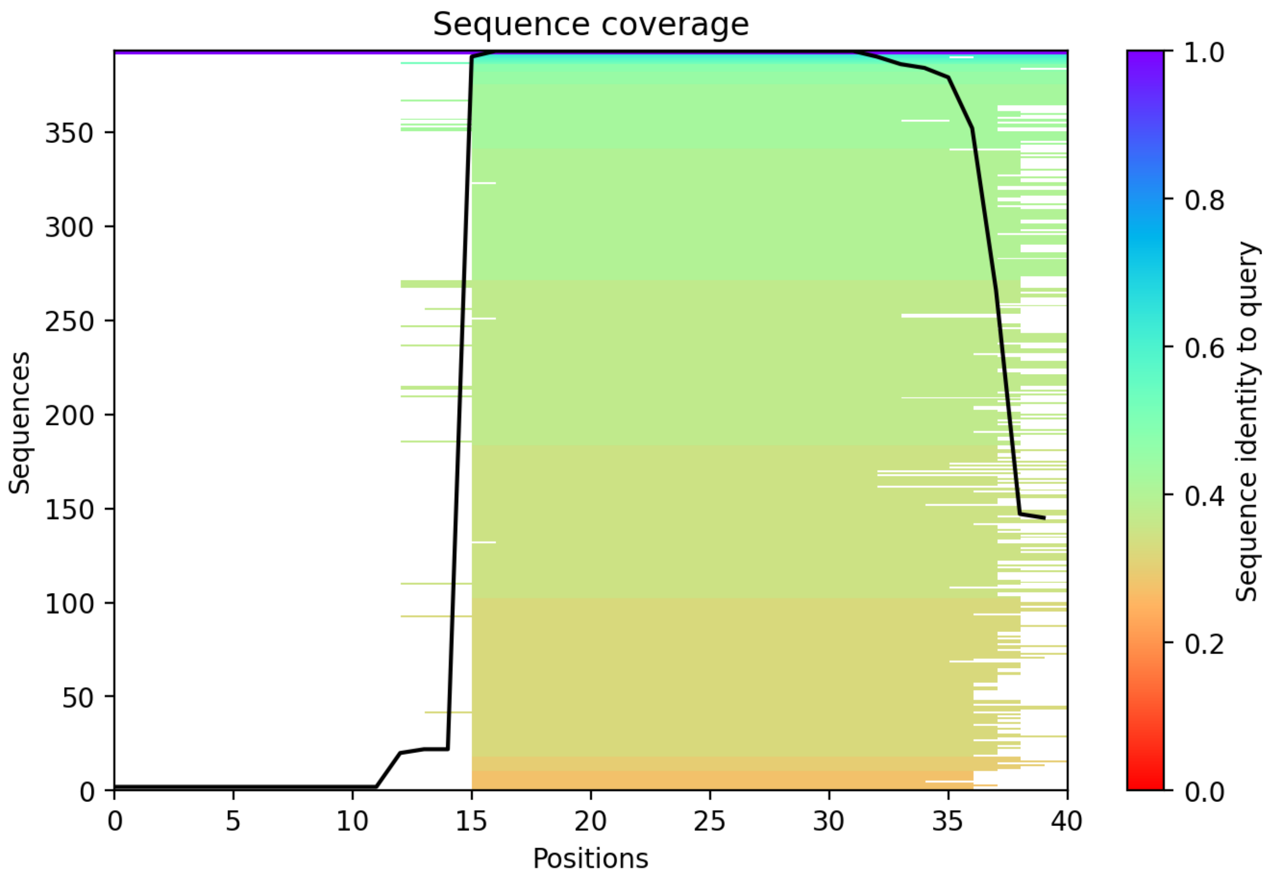

CP107: MtrunA17_Chr6g0479001

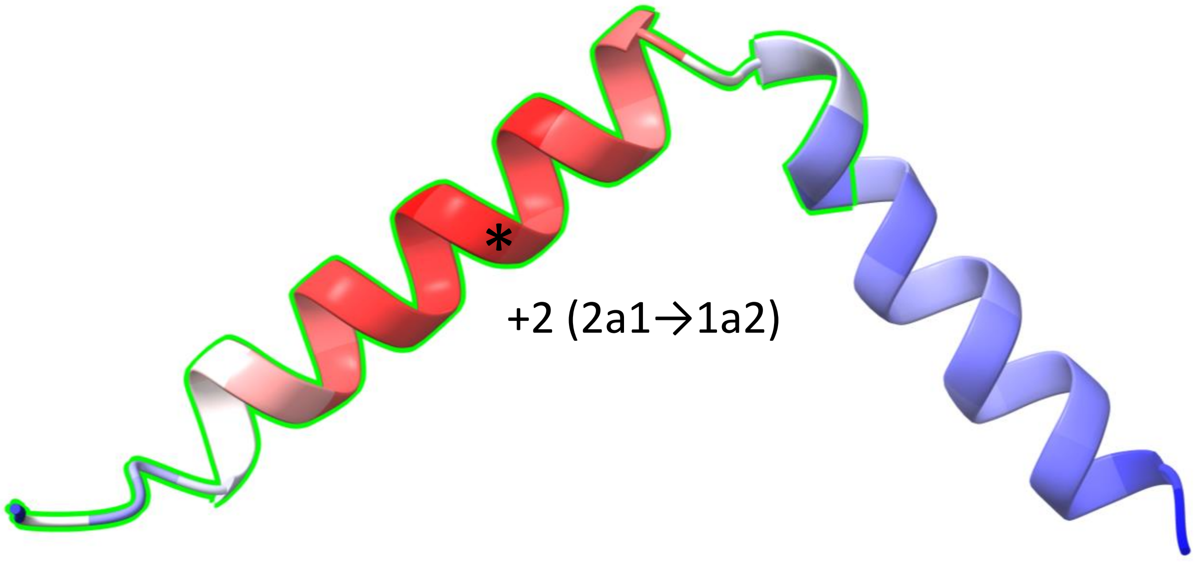

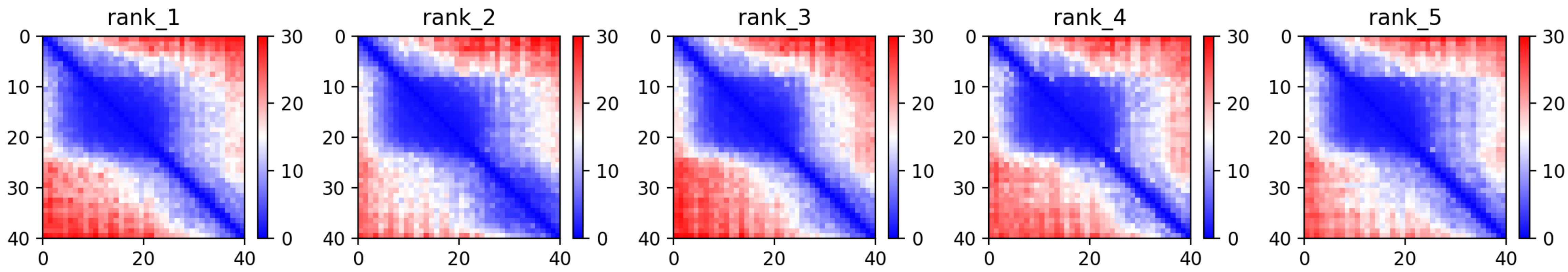

CP107: MtrunA17_Chr6g0479001

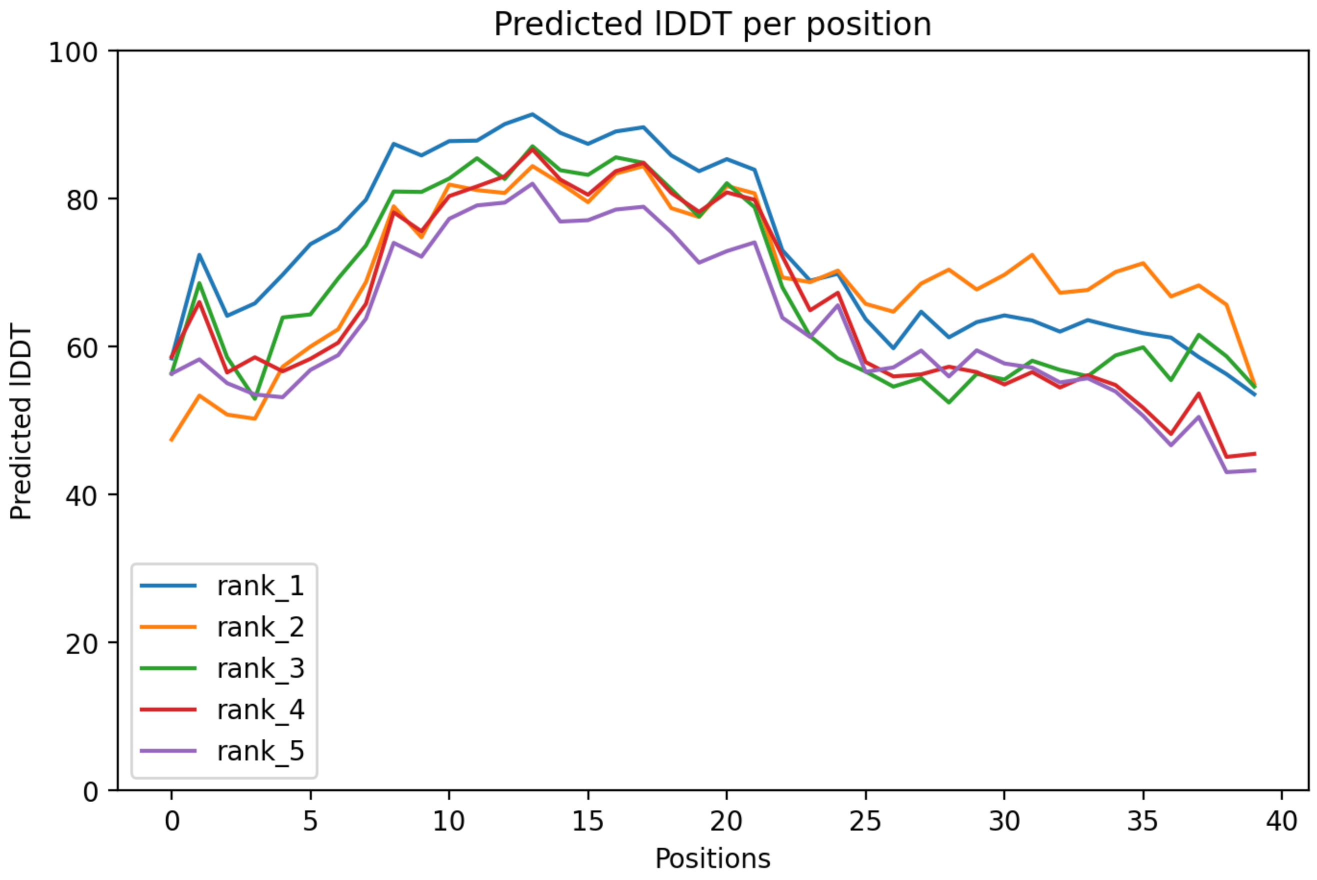

CP107: MtrunA17_Chr6g0479001

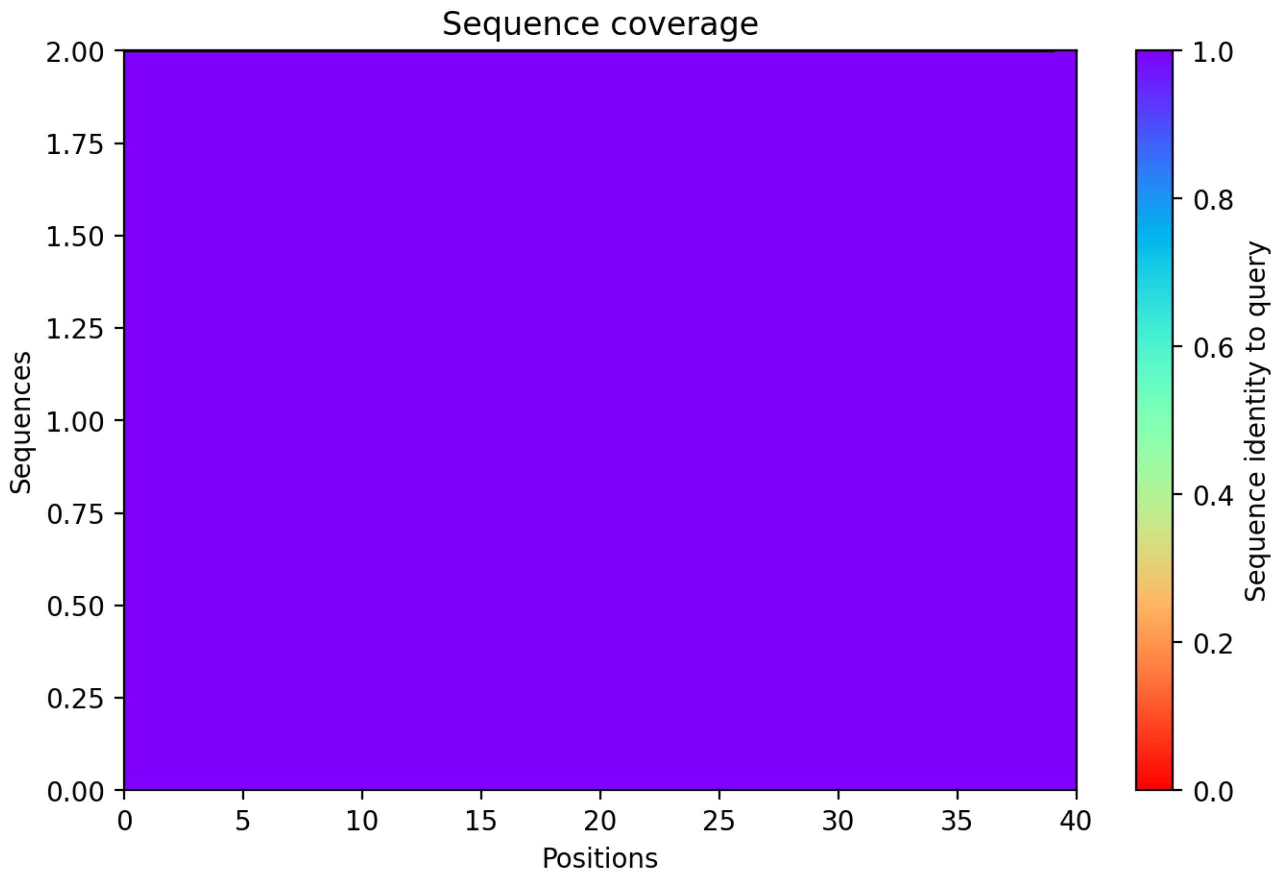

CP108: MtrunA17_Chr6g0485321

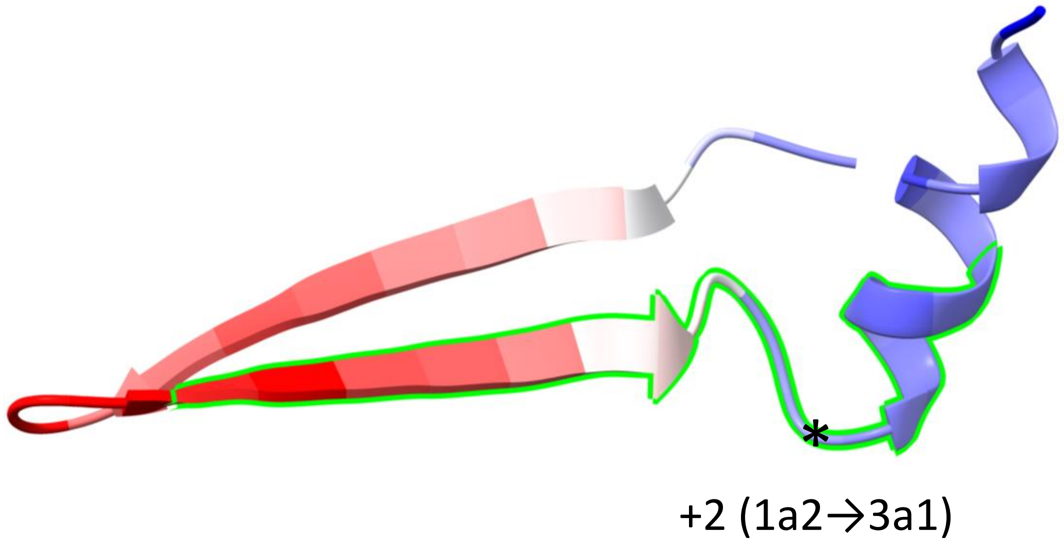

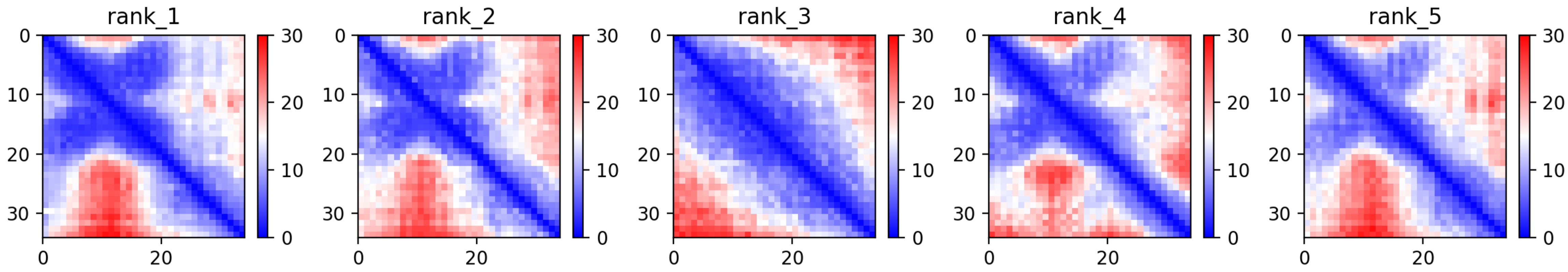

CP108: MtrunA17_Chr6g0485321

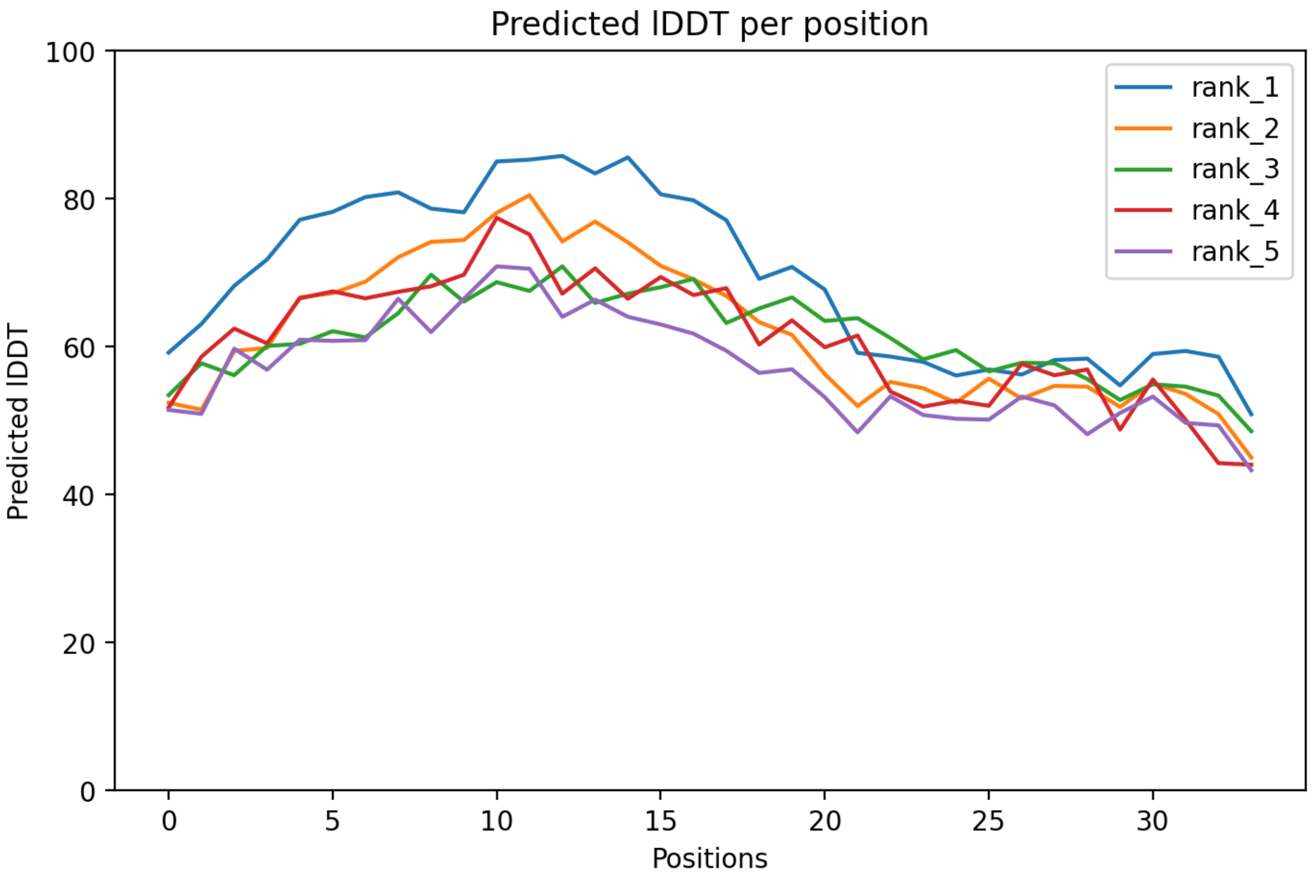

CP108: MtrunA17_Chr6g0485321

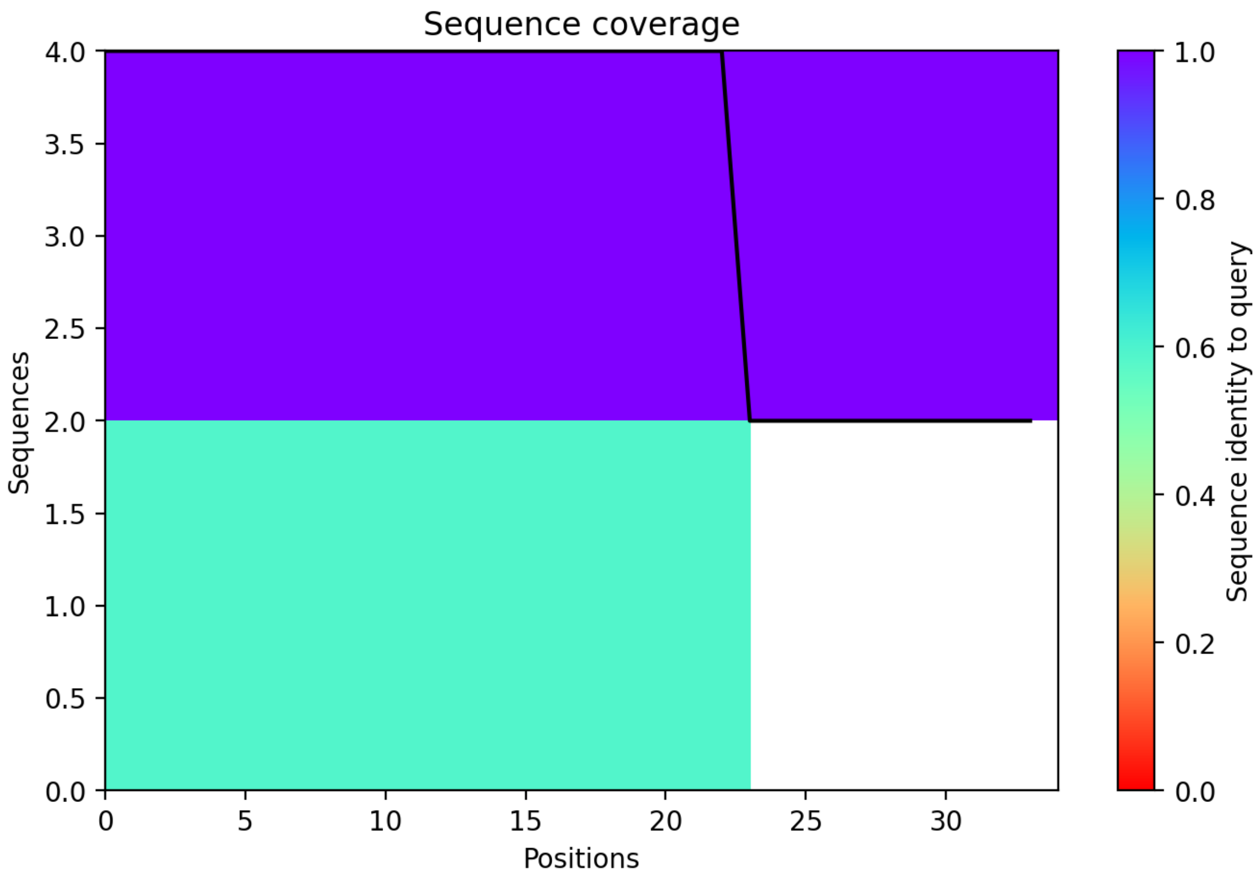

CP109: MtrunA17_Chr6g0486961

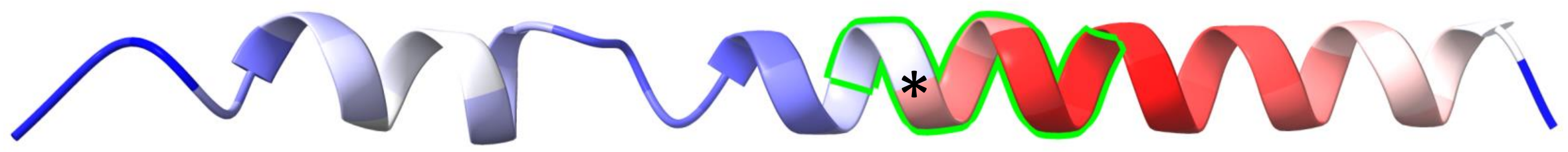

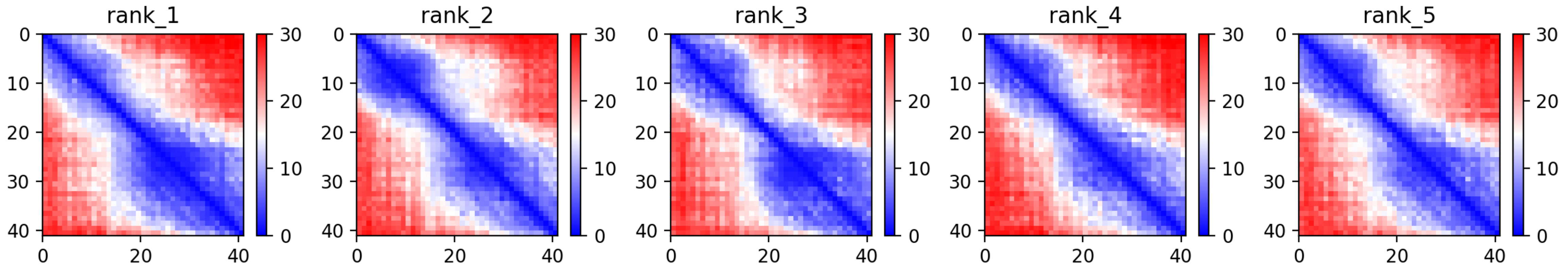

CP109: MtrunA17_Chr6g0486961

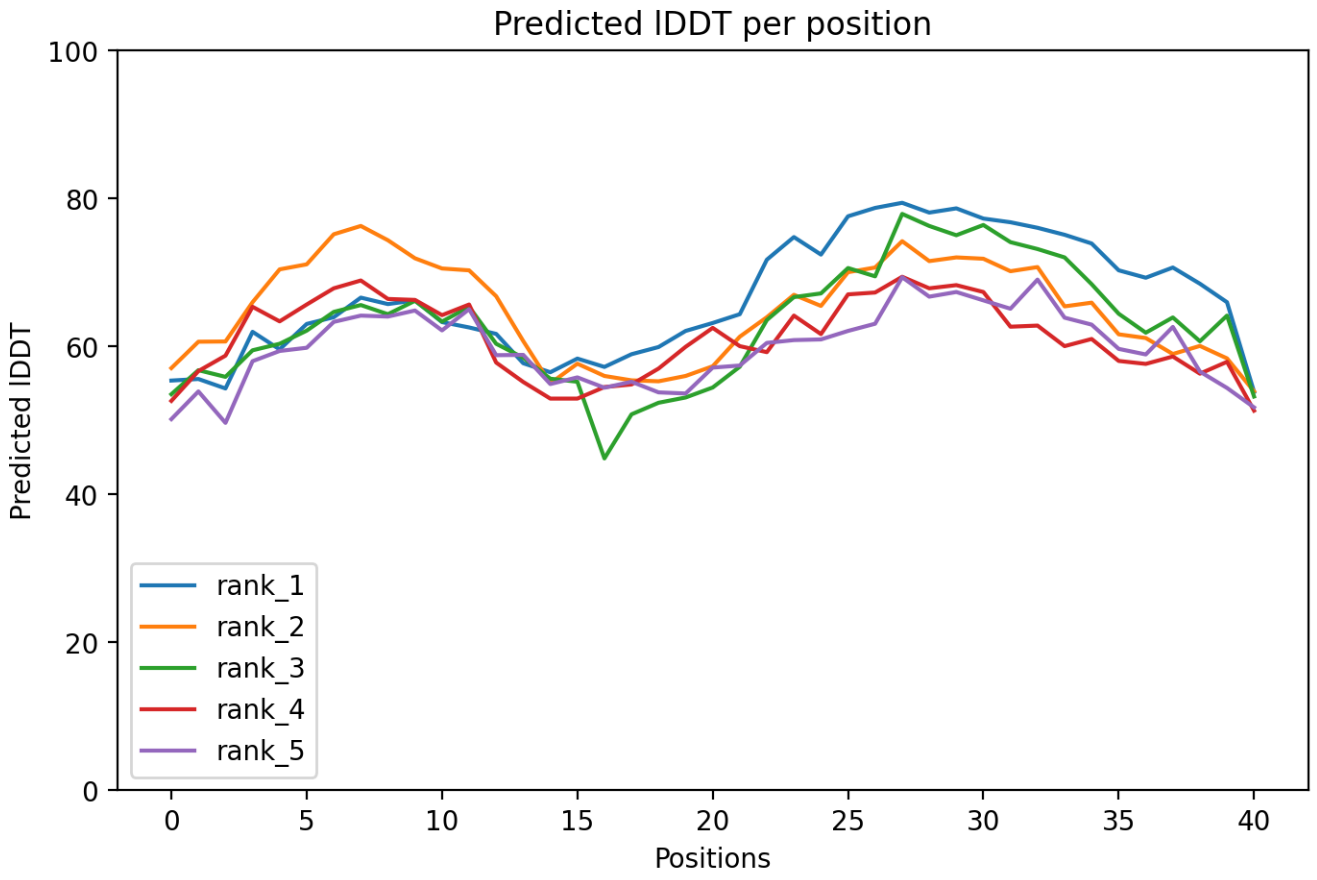

CP109: MtrunA17_Chr6g0486961

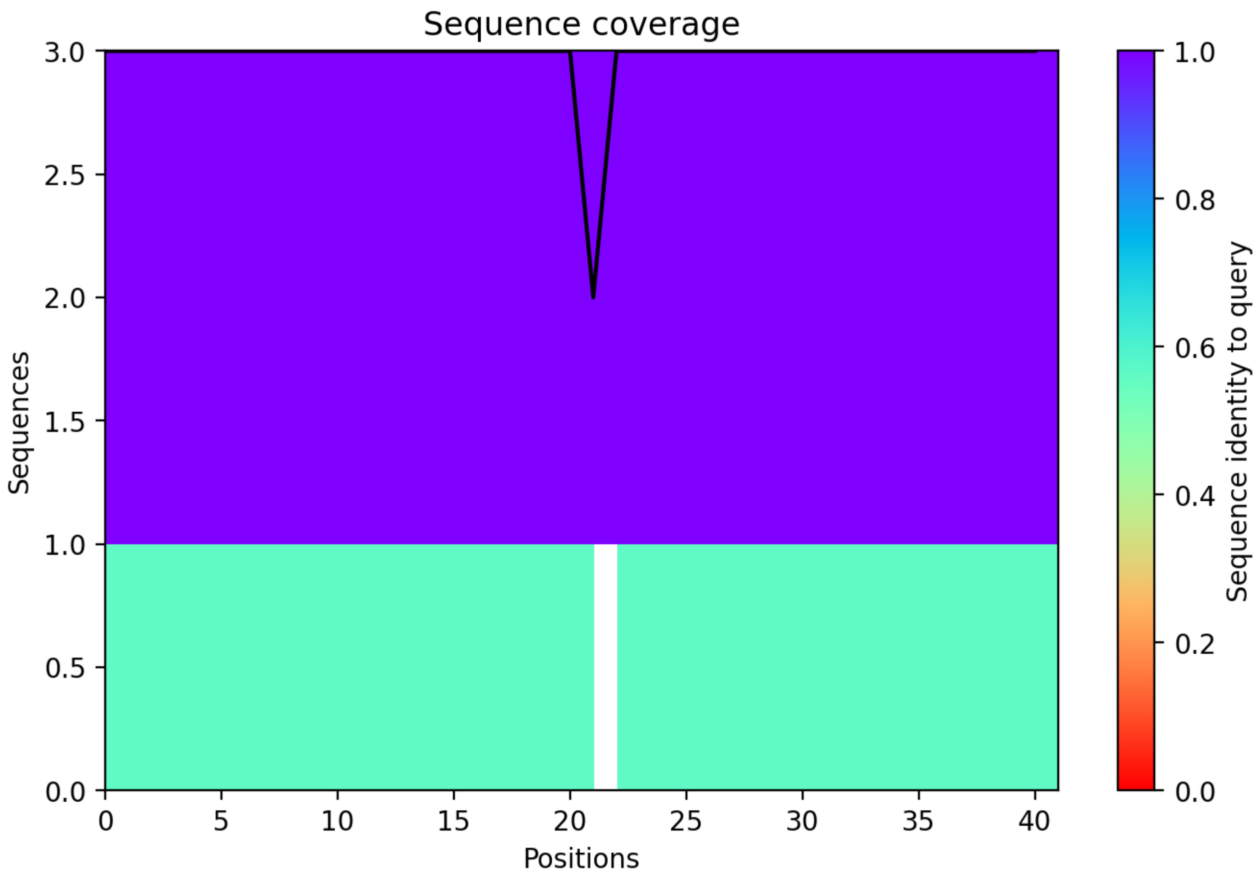

CP110: MtrunA17_Chr7g0214741

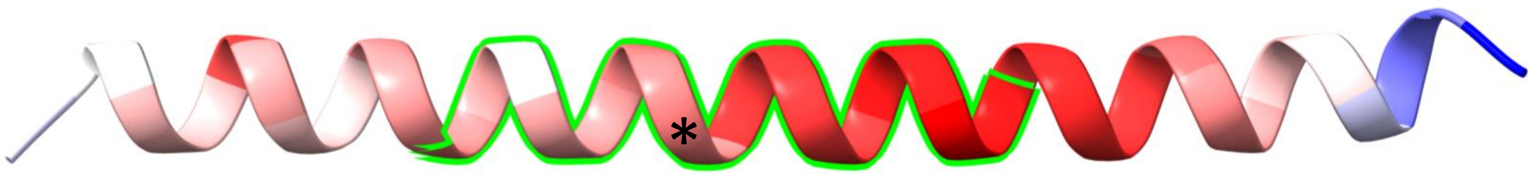

+1 (3r→1a)

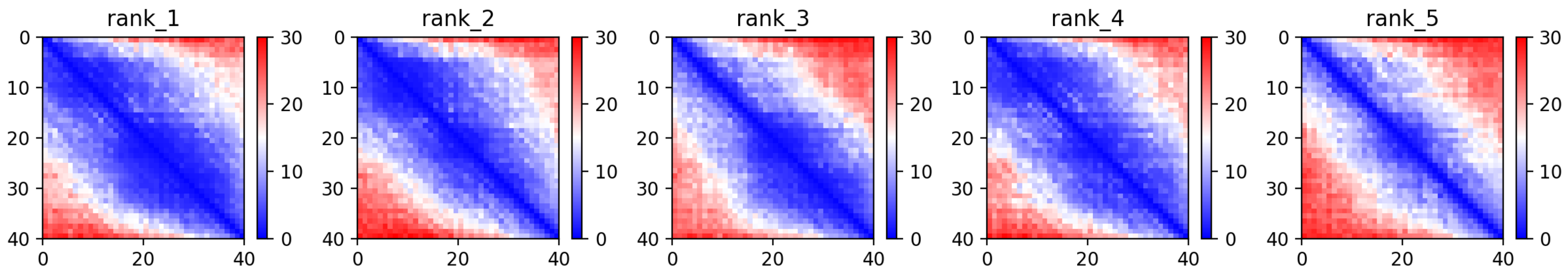

CP110: MtrunA17_Chr7g0214741

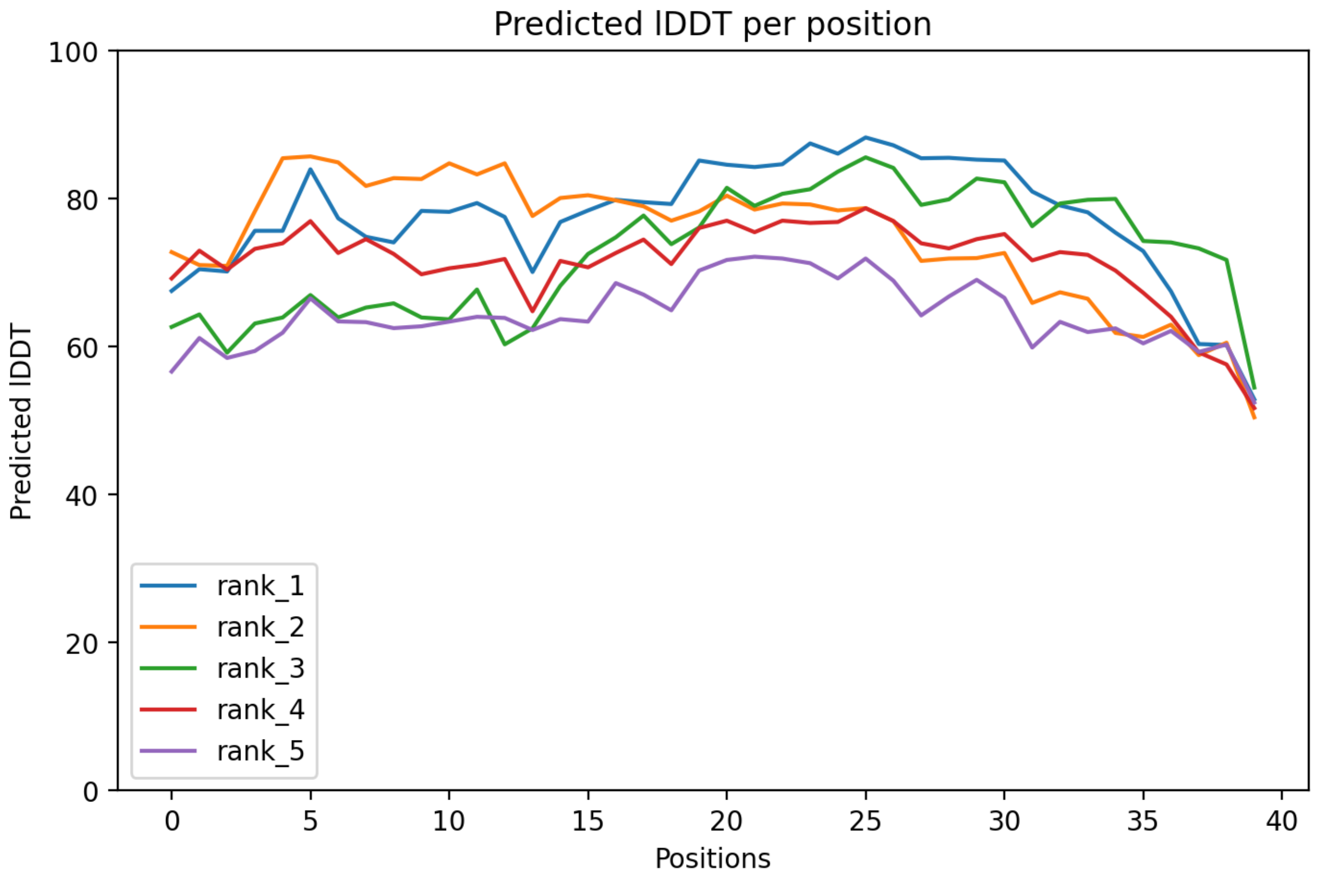

CP110: MtrunA17_Chr7g0214741

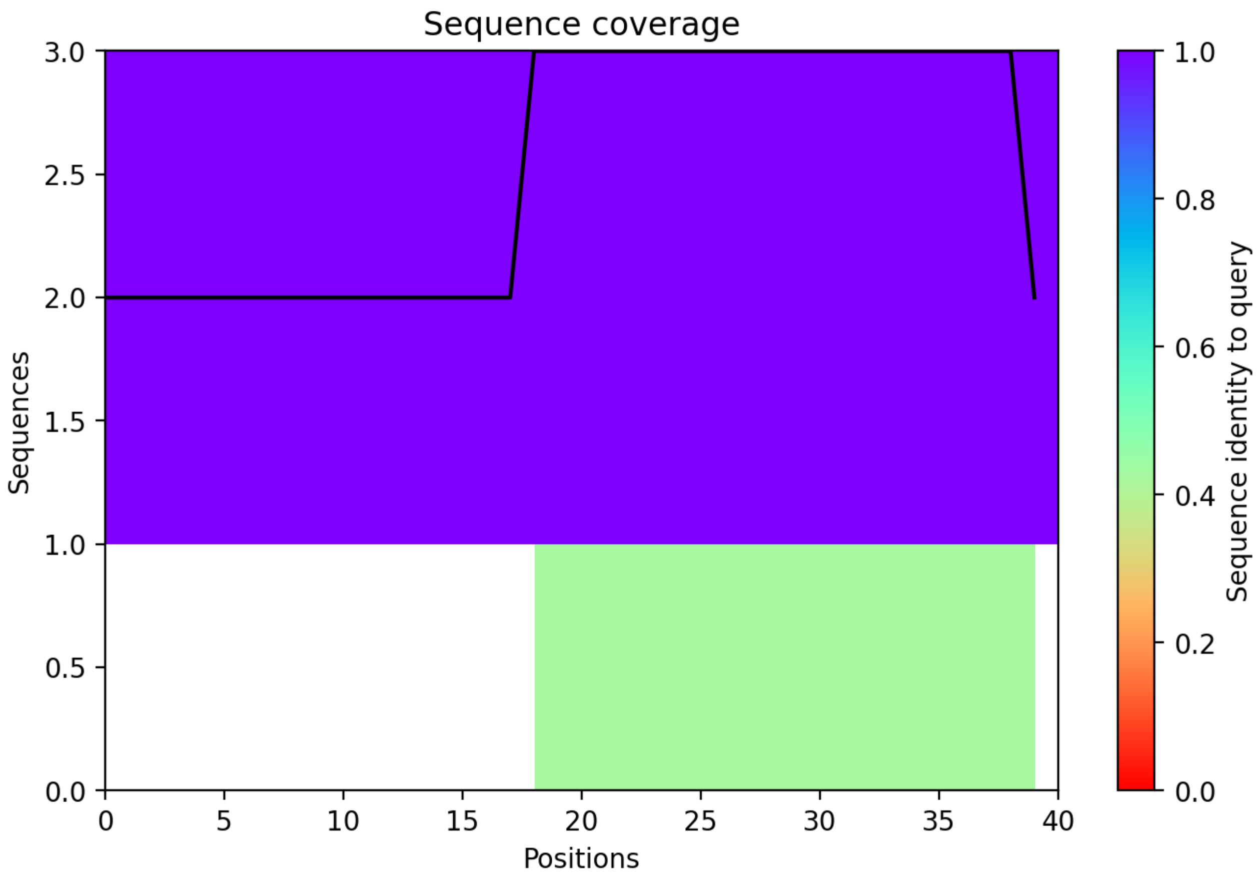

CP111: MtrunA17_Chr7g0214911

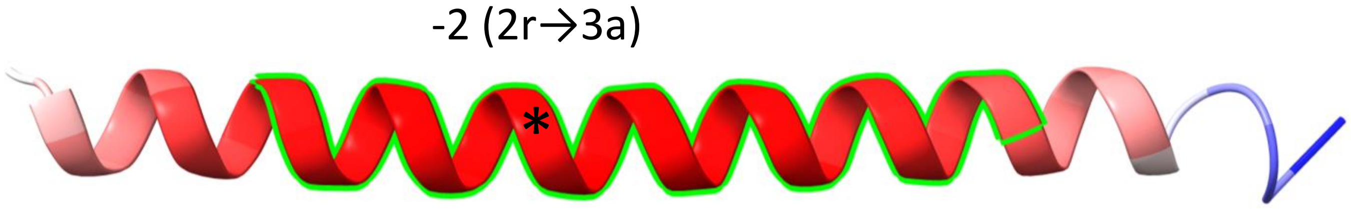

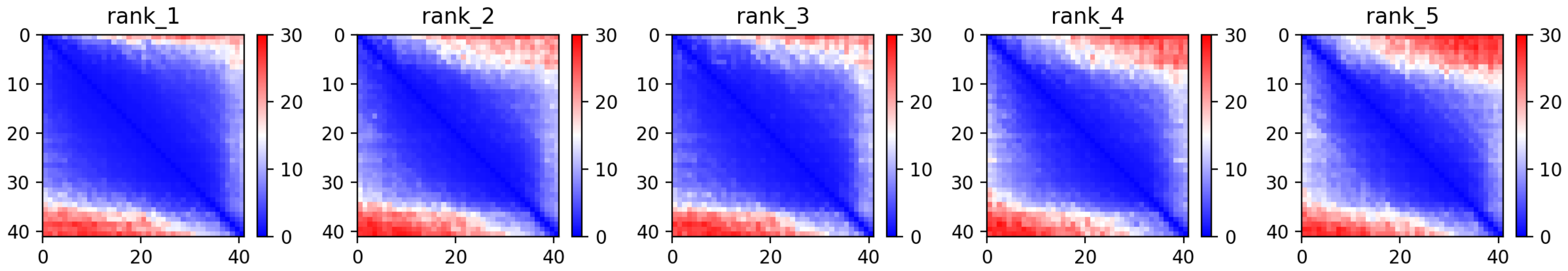

CP111: MtrunA17_Chr7g0214911

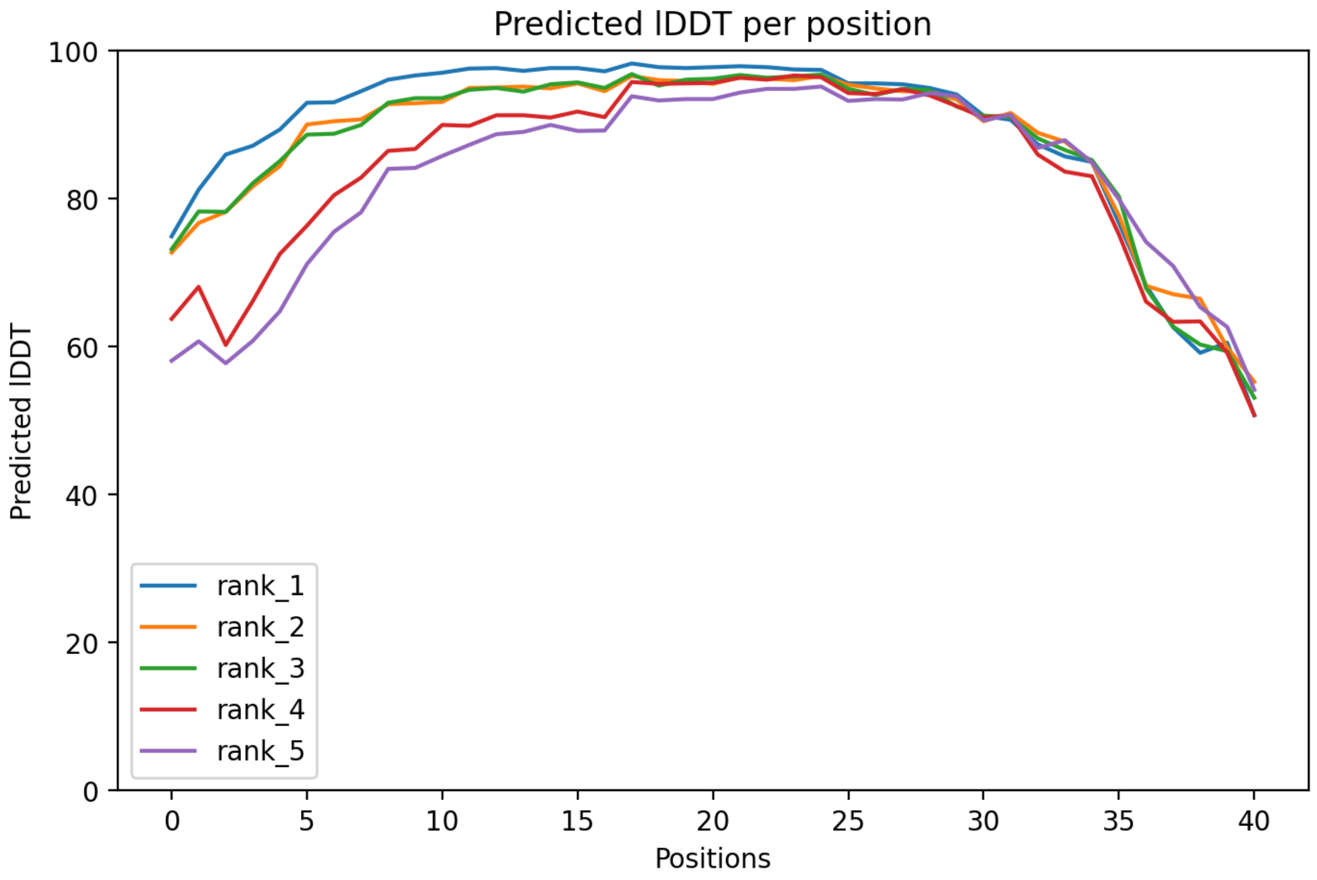

CP111: MtrunA17_Chr7g0214911

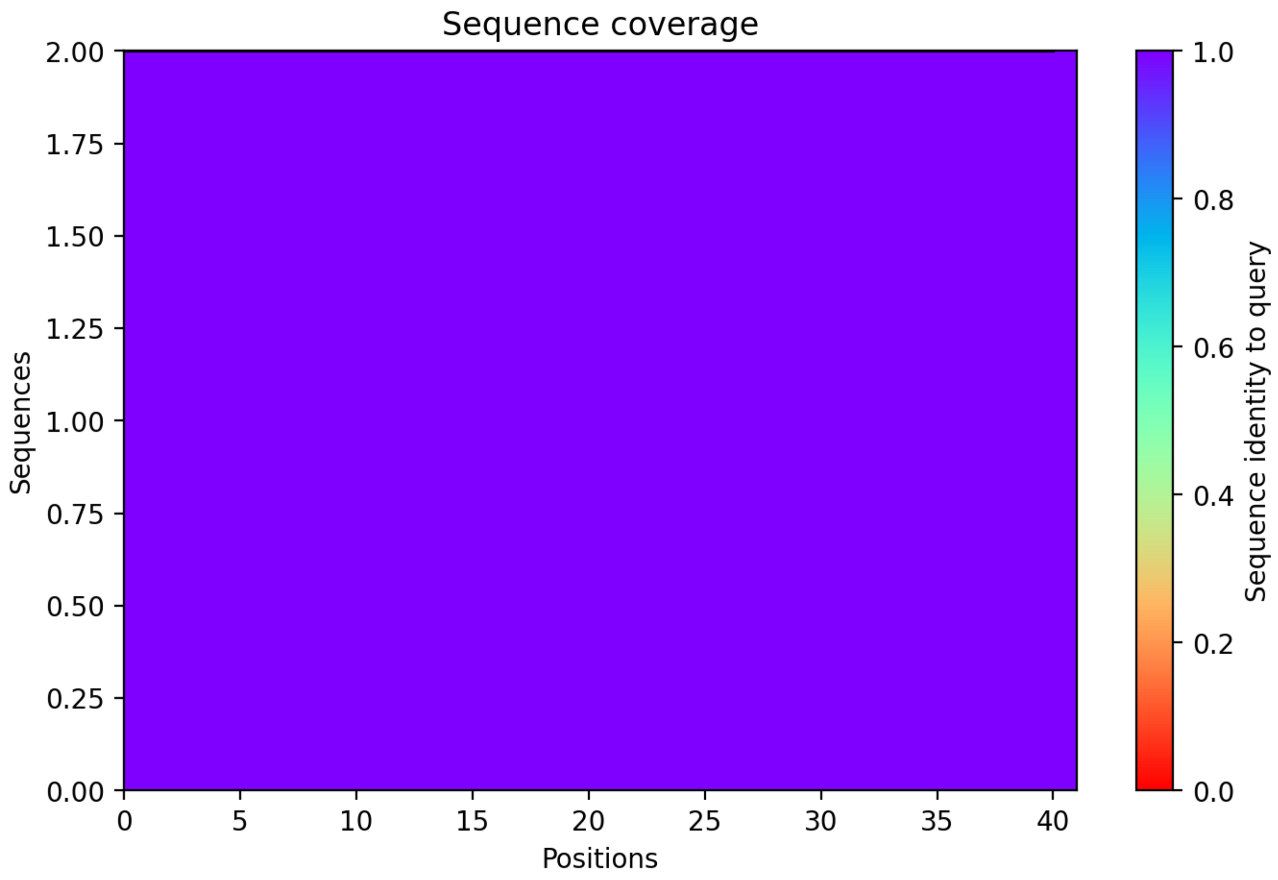

CP112: MtrunA17_Chr7g0219691

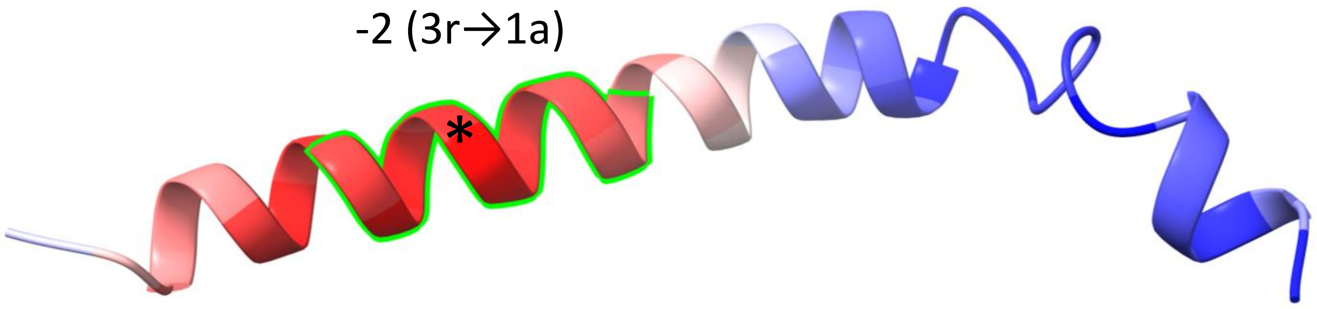

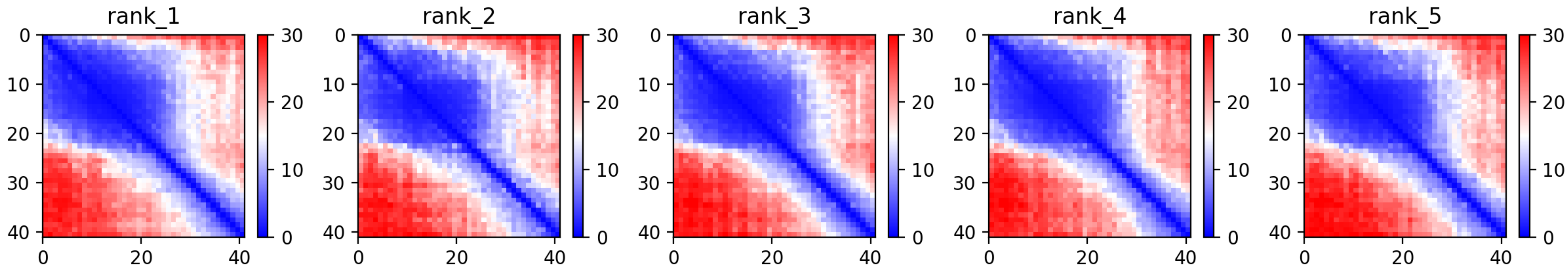

CP112: MtrunA17_Chr7g0219691

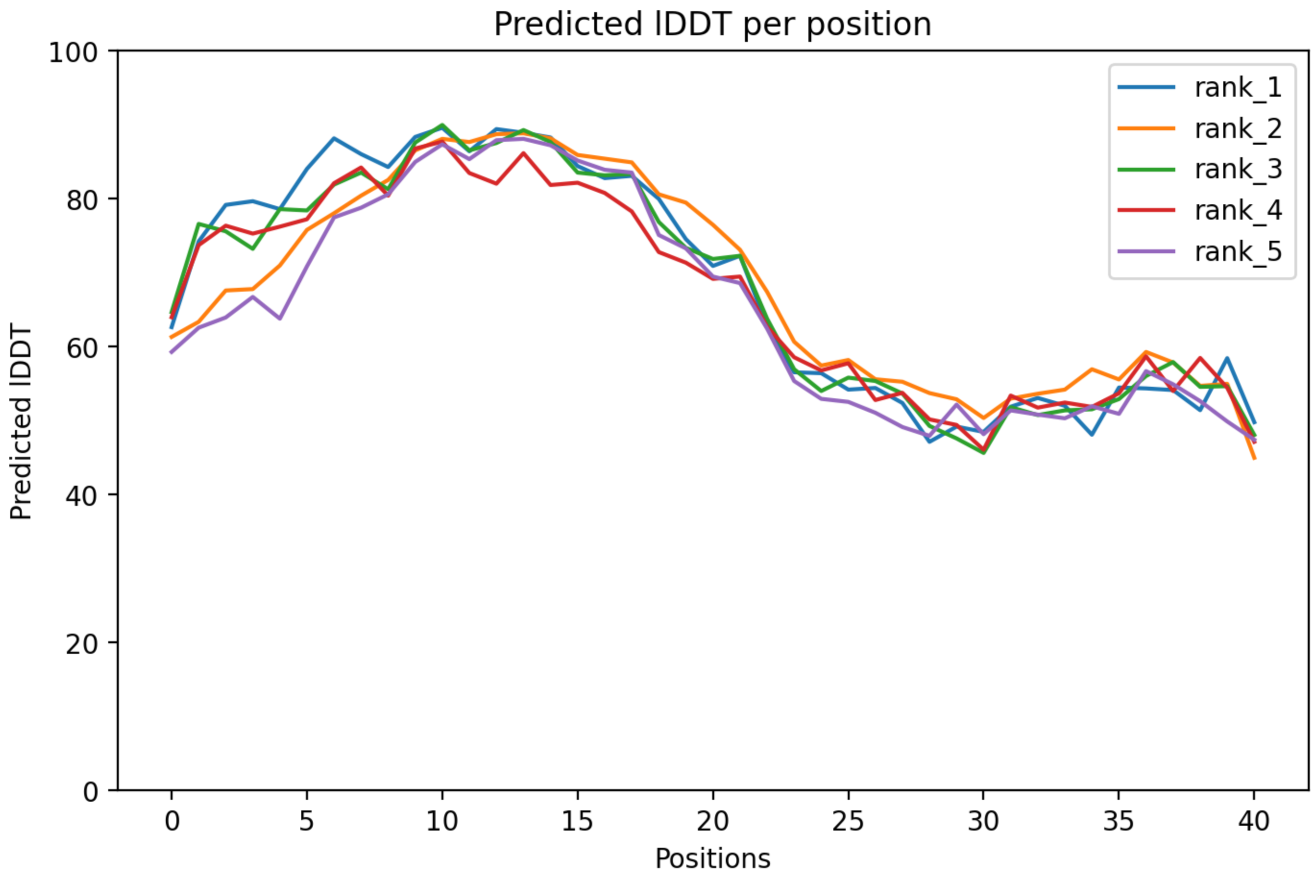

CP112: MtrunA17_Chr7g0219691

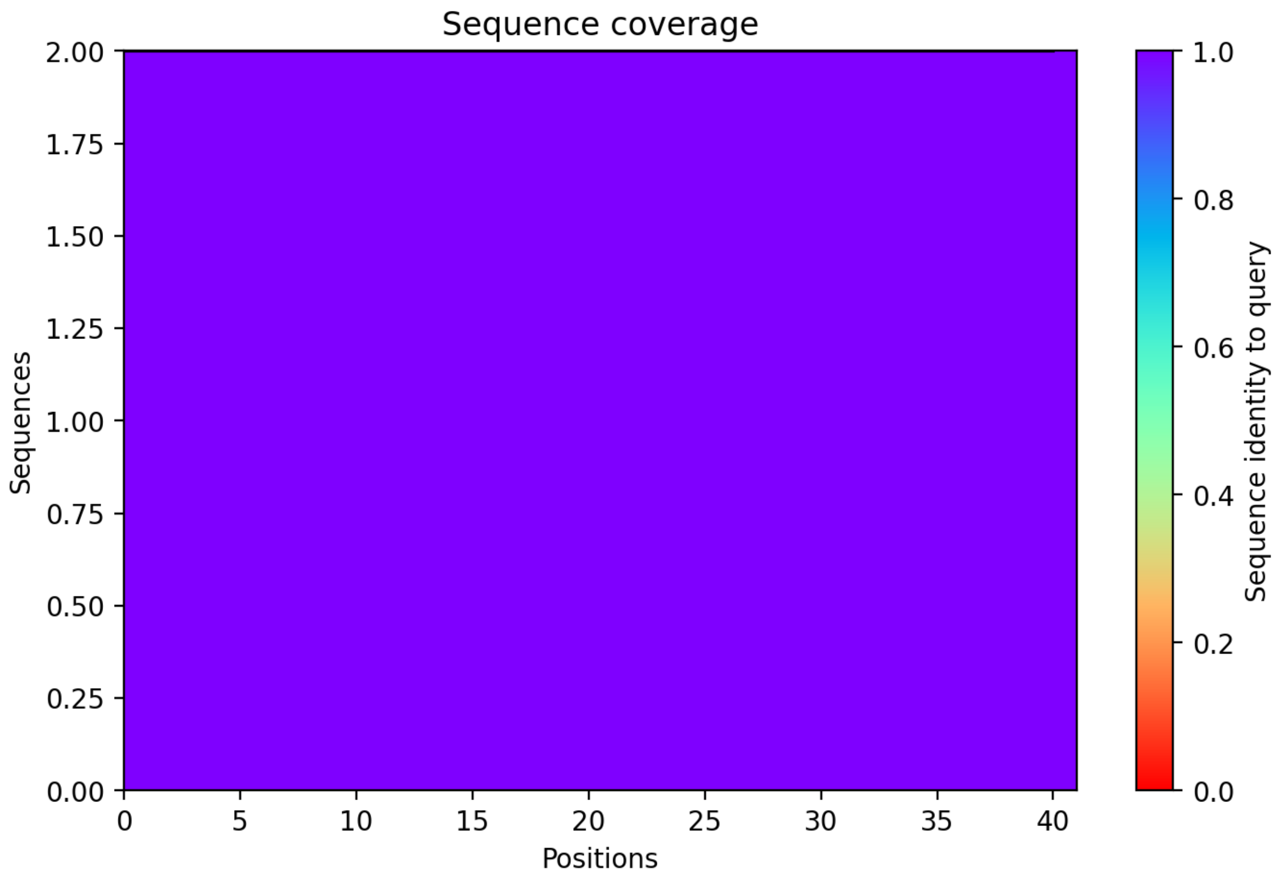

CP113: MtrunA17_Chr7g0221631

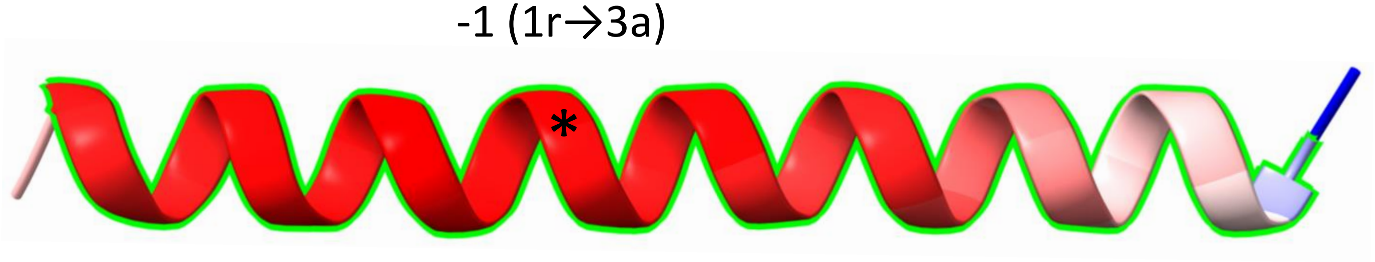

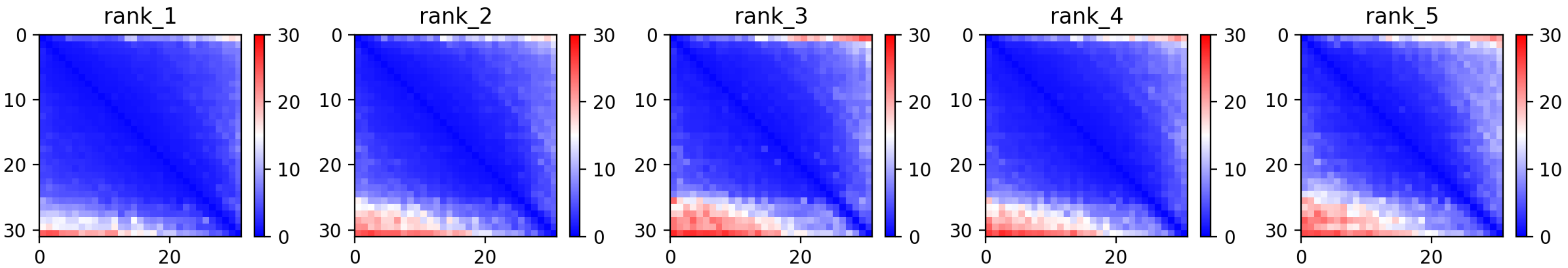

CP113: MtrunA17_Chr7g0221631

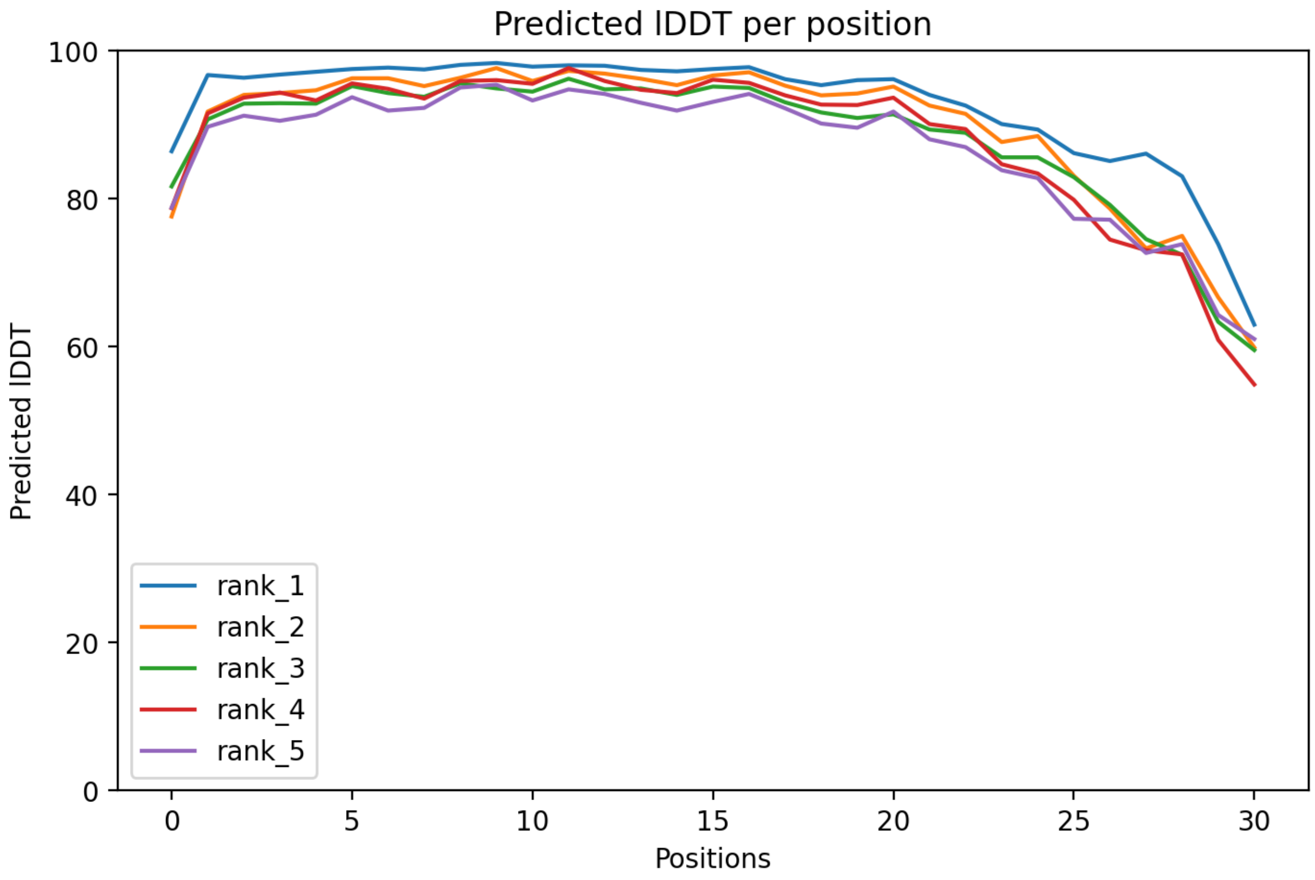

CP113: MtrunA17_Chr7g0221631

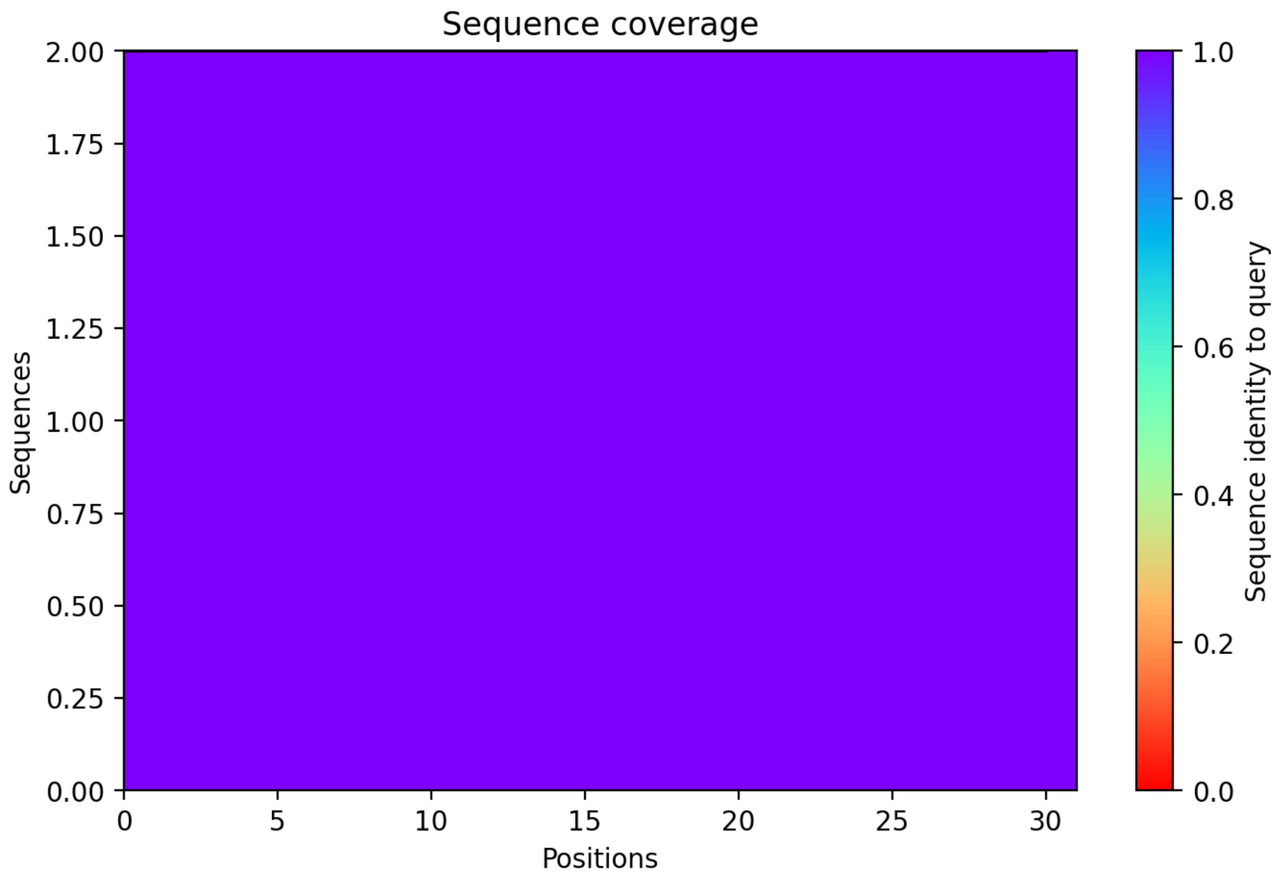

CP114: MtrunA17_Chr7g0229401

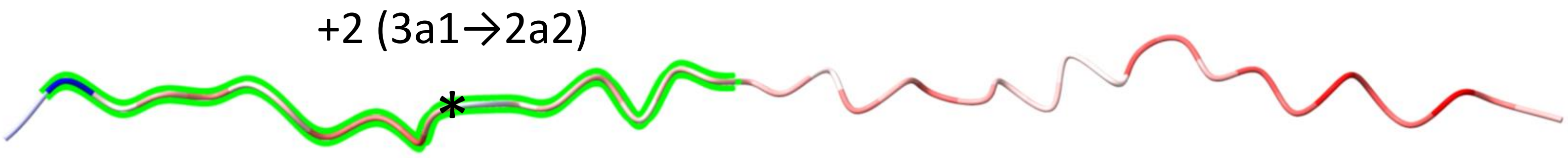

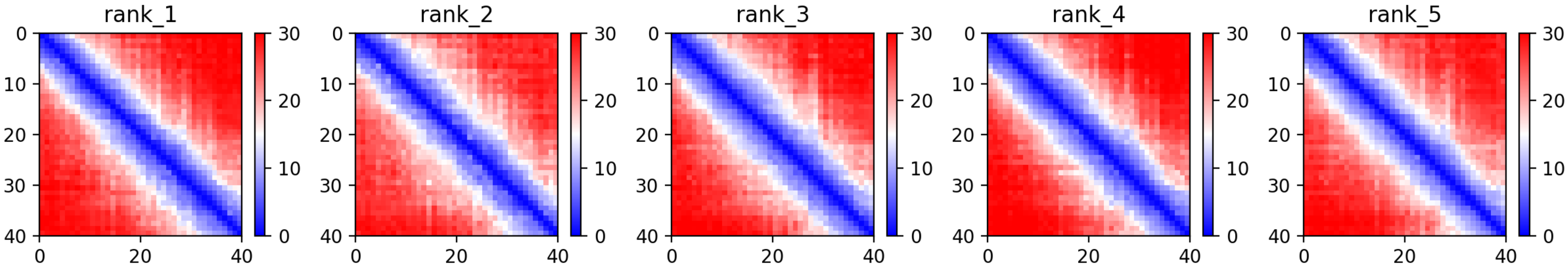

CP114: MtrunA17_Chr7g0229401

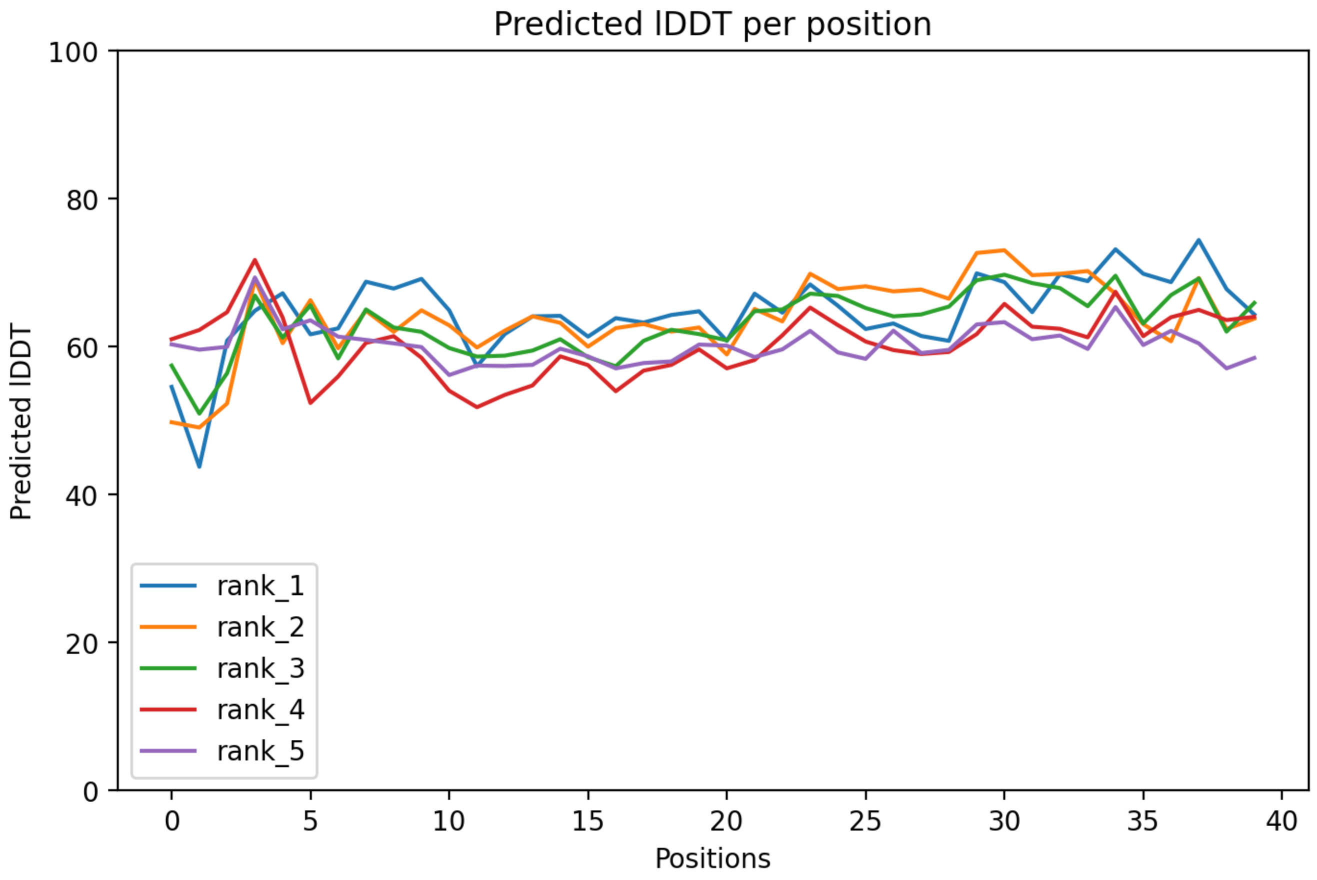

CP114: MtrunA17_Chr7g0229401

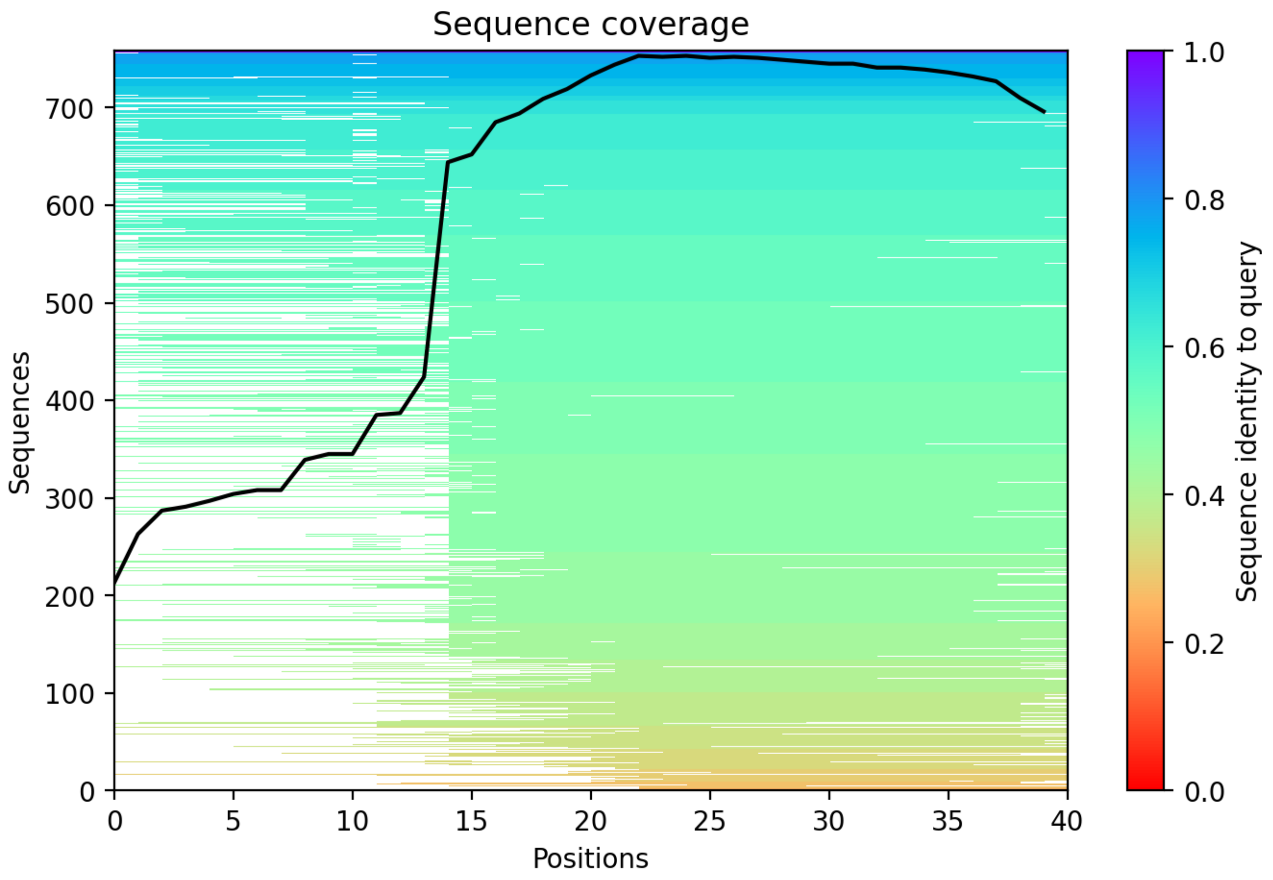

CP115: MtrunA17_Chr7g0230341

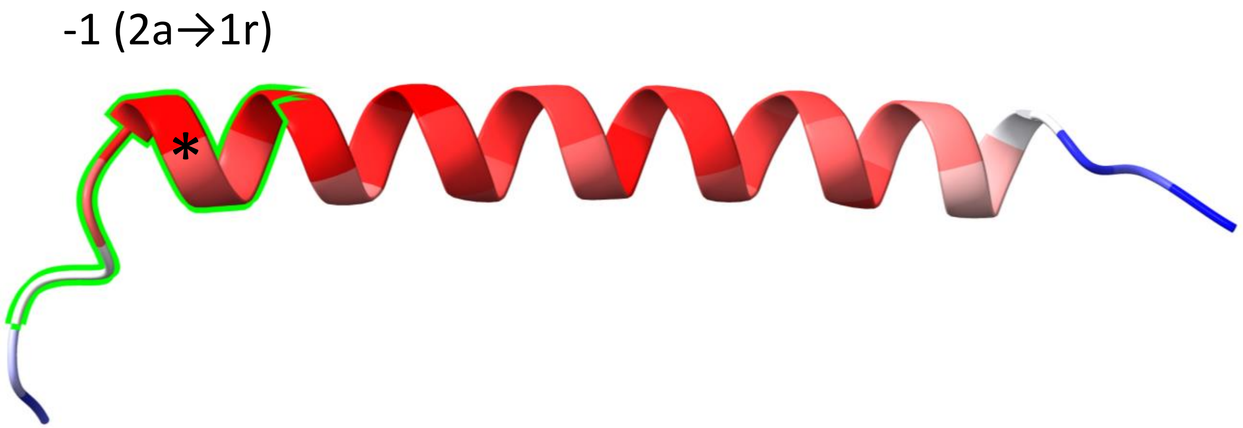

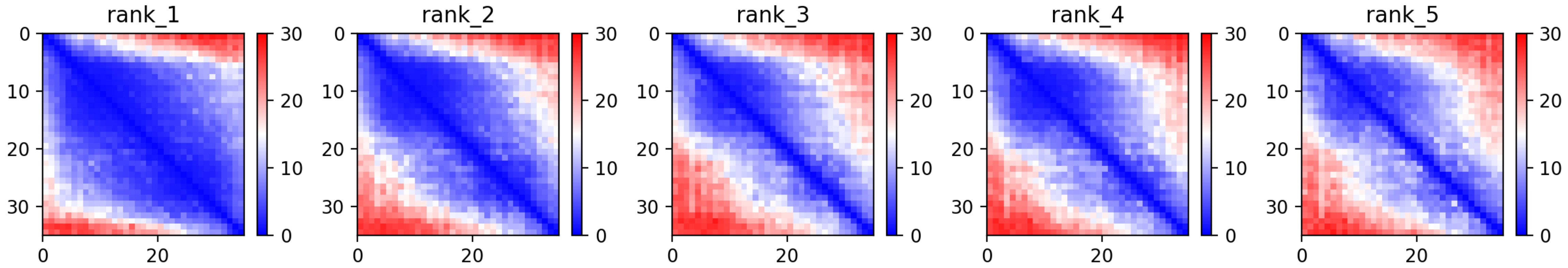

CP115: MtrunA17_Chr7g0230341

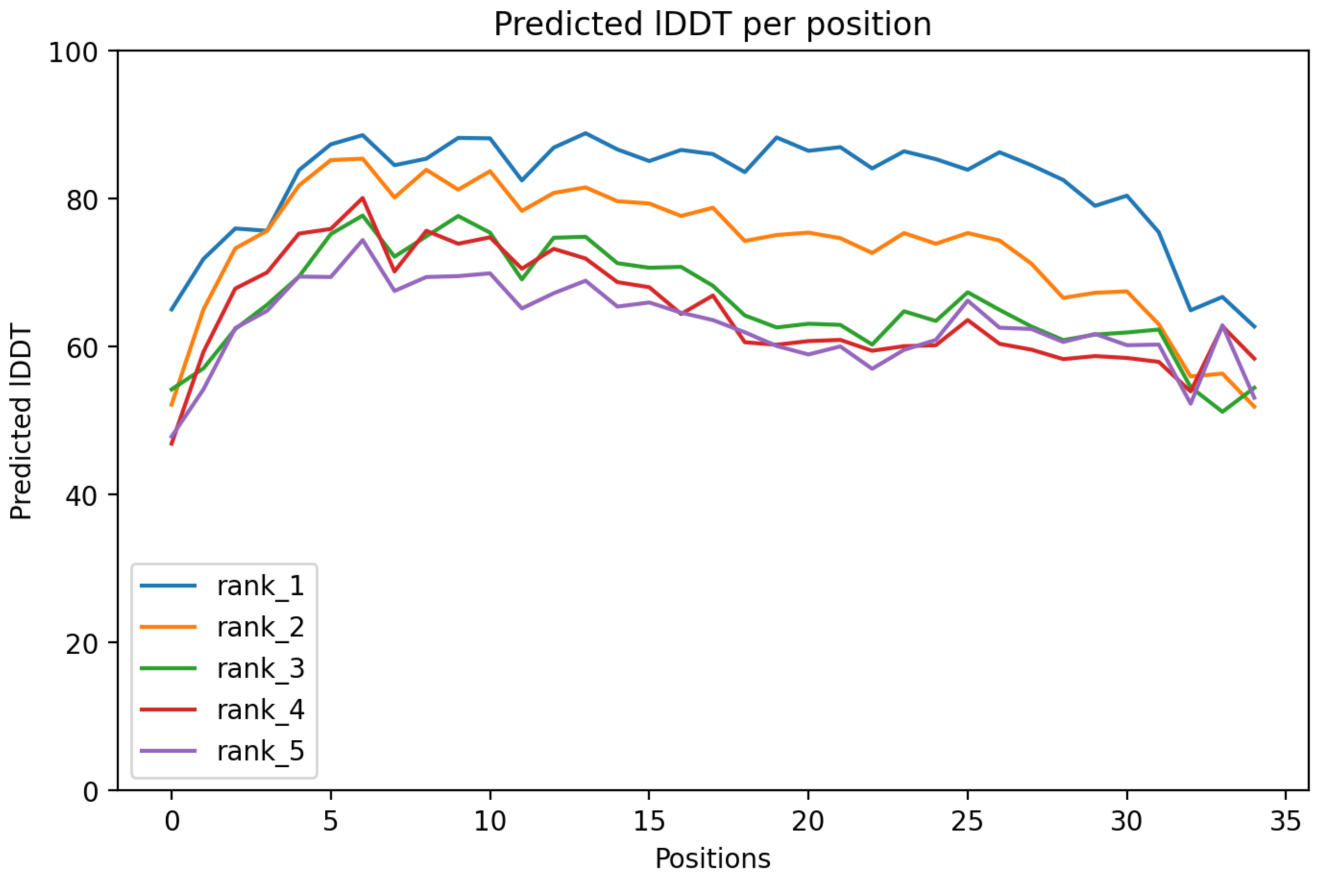

CP115: MtrunA17_Chr7g0230341

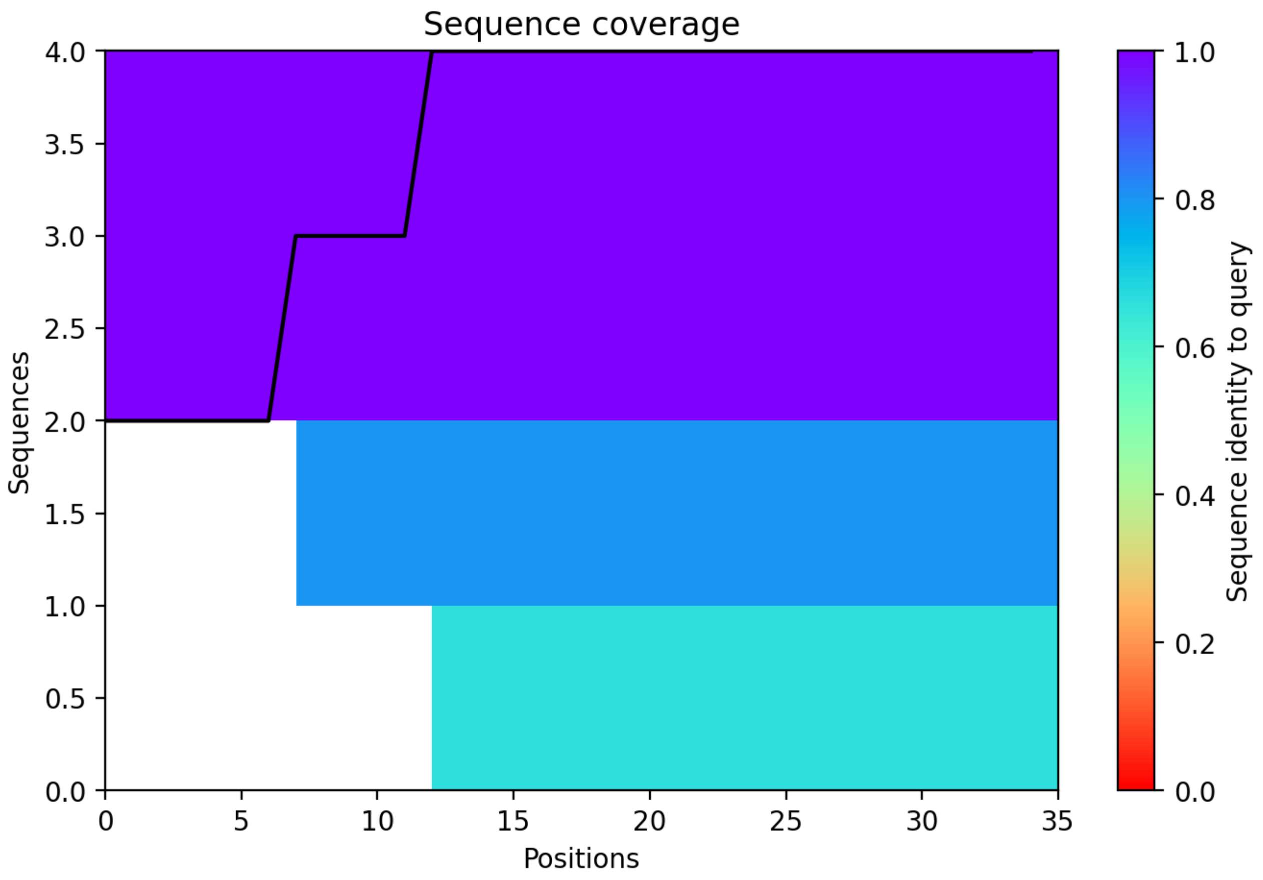

CP116: MtrunA17_Chr7g0232851

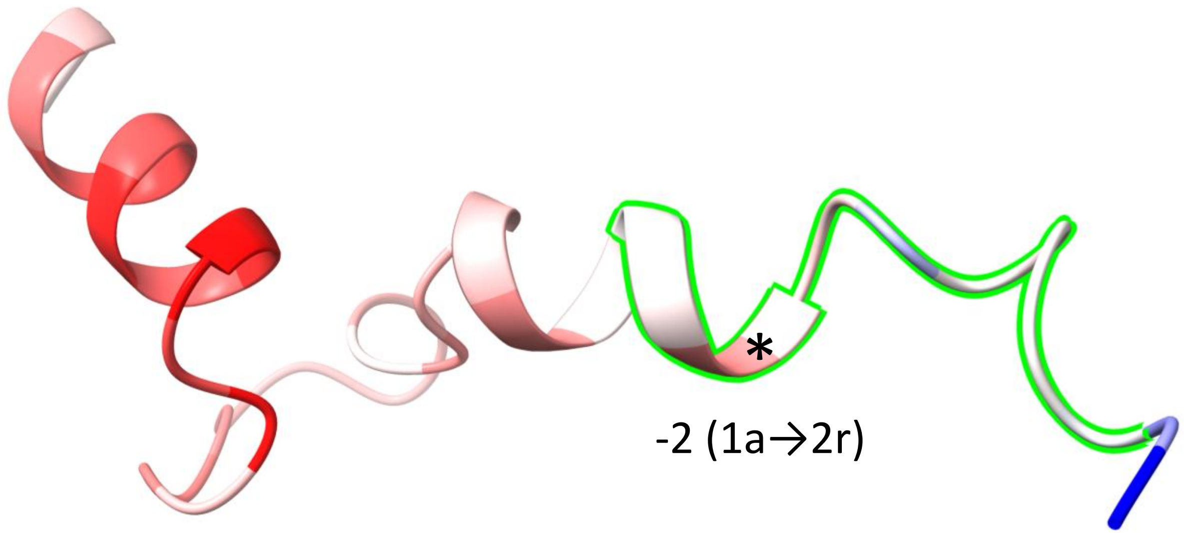

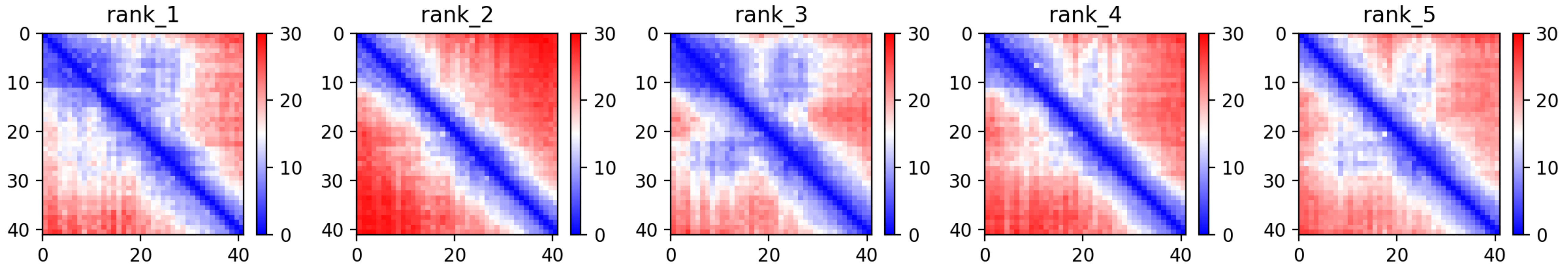

CP116: MtrunA17_Chr7g0232851

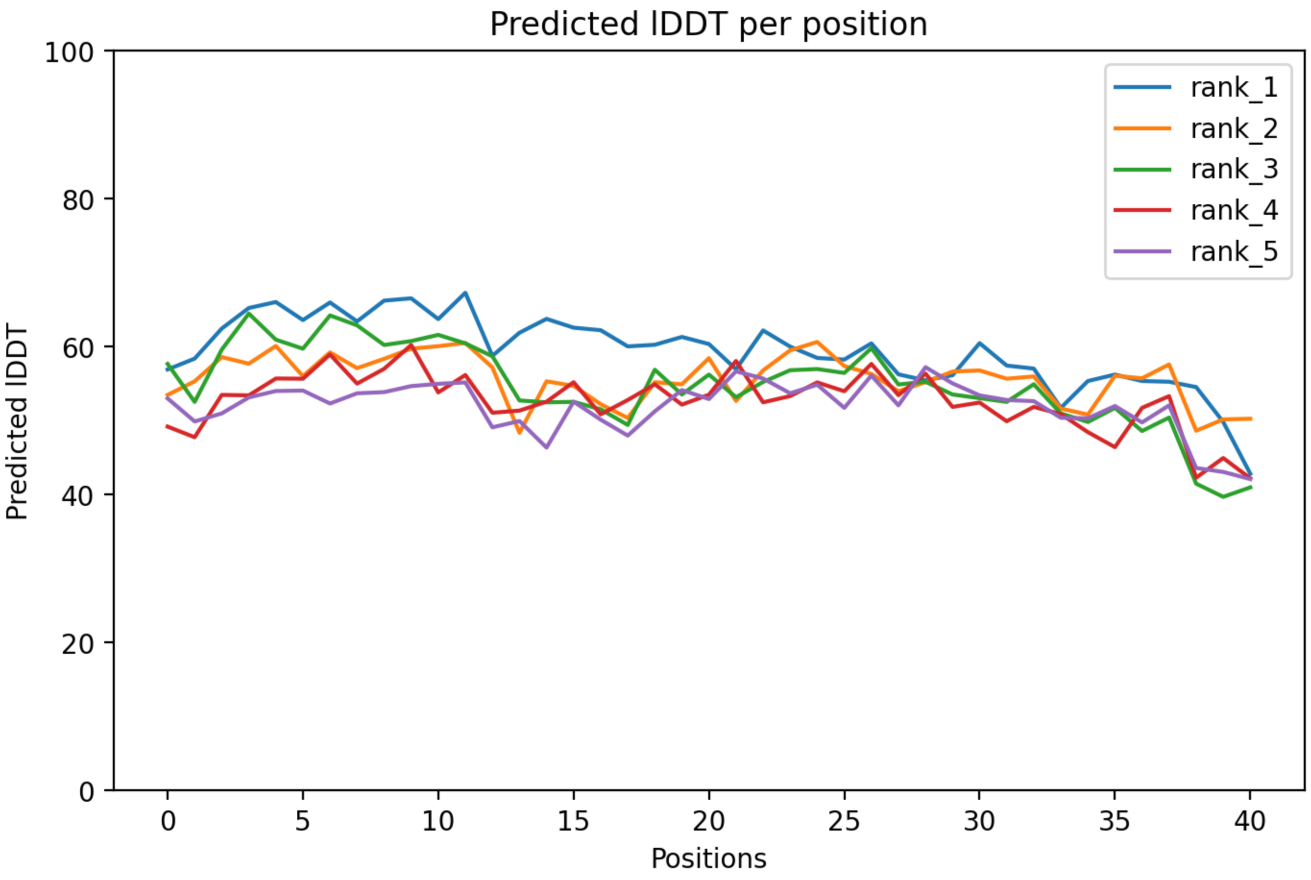

CP116: MtrunA17_Chr7g0232851

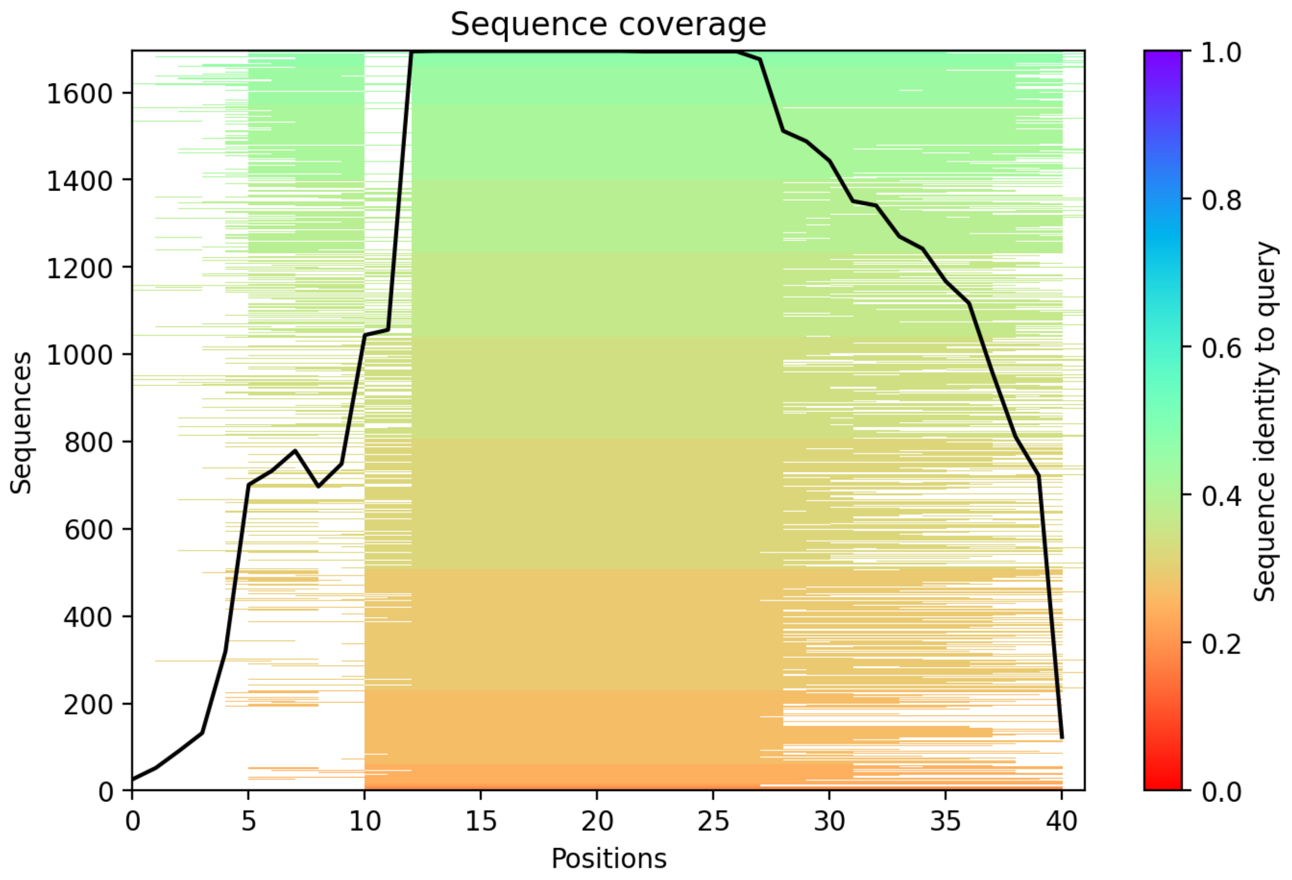

CP117: MtrunA17_Chr7g0237331

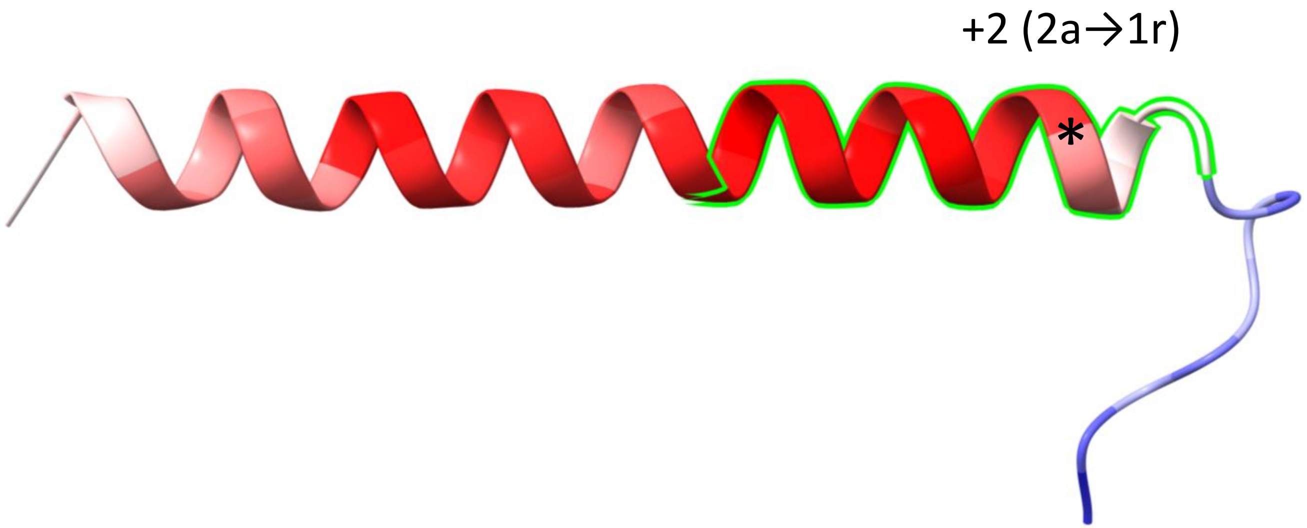

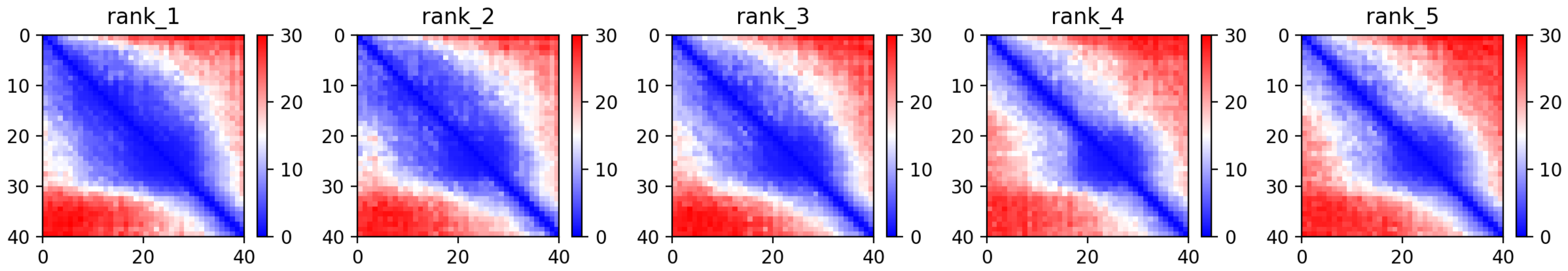

CP117: MtrunA17_Chr7g0237331

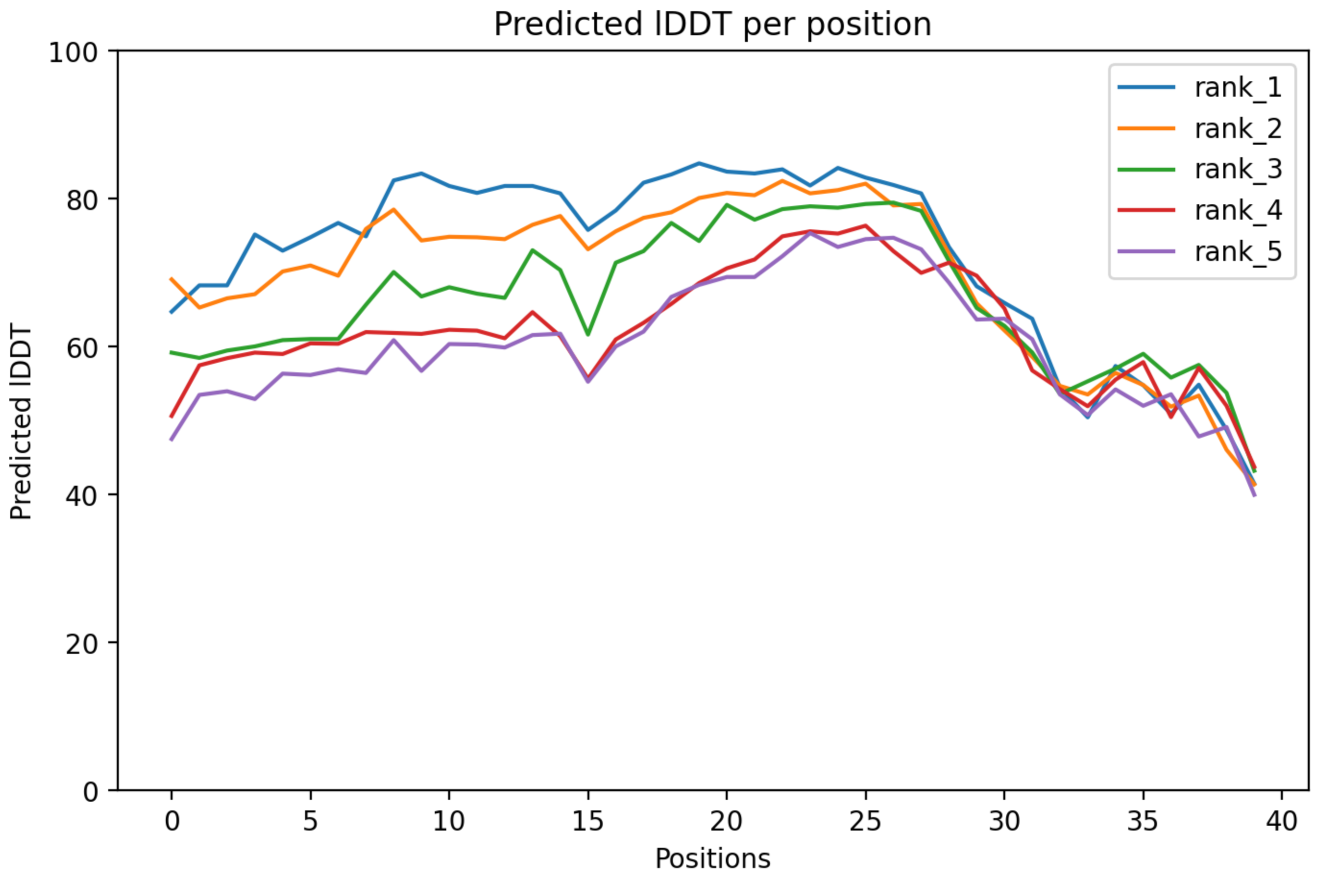

CP117: MtrunA17_Chr7g0237331

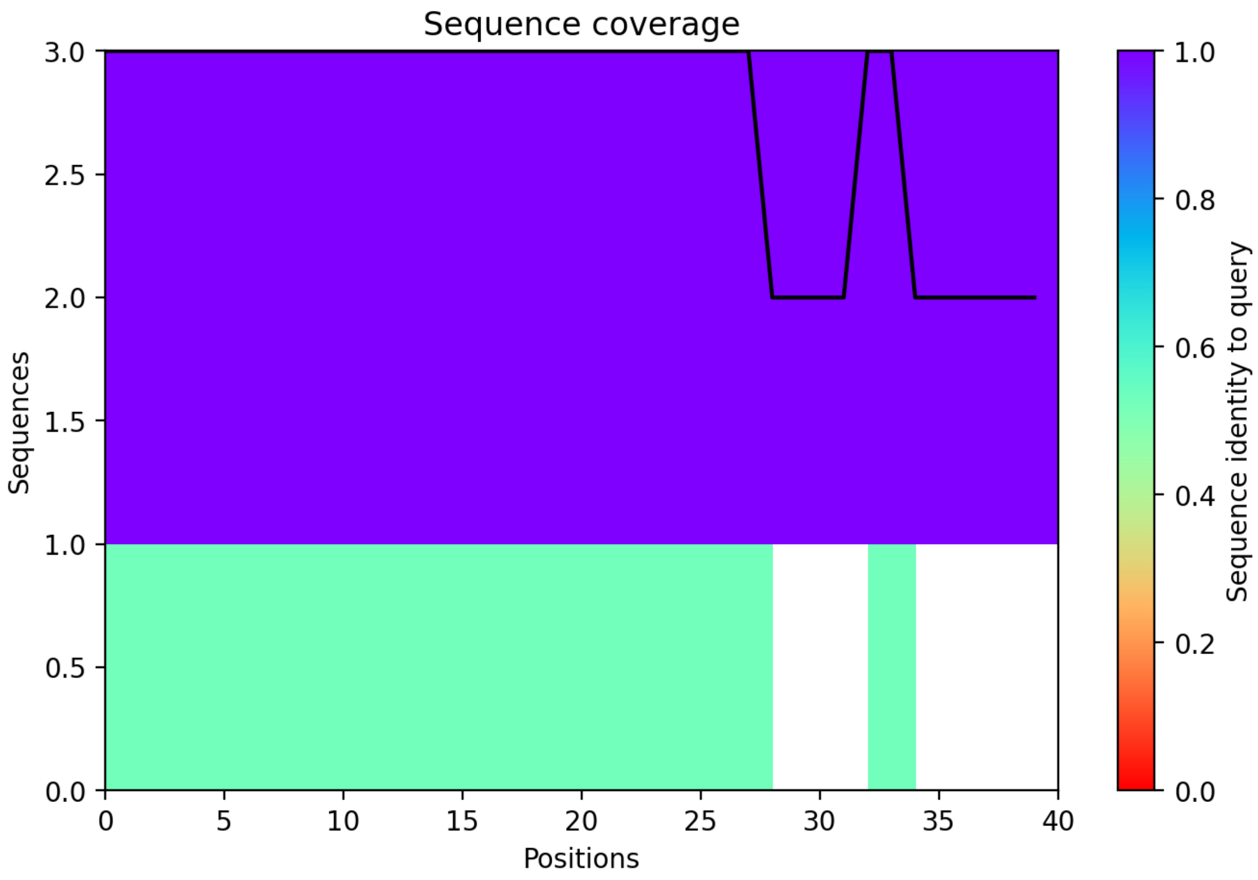

CP118: MtrunA17_Chr7g0251971

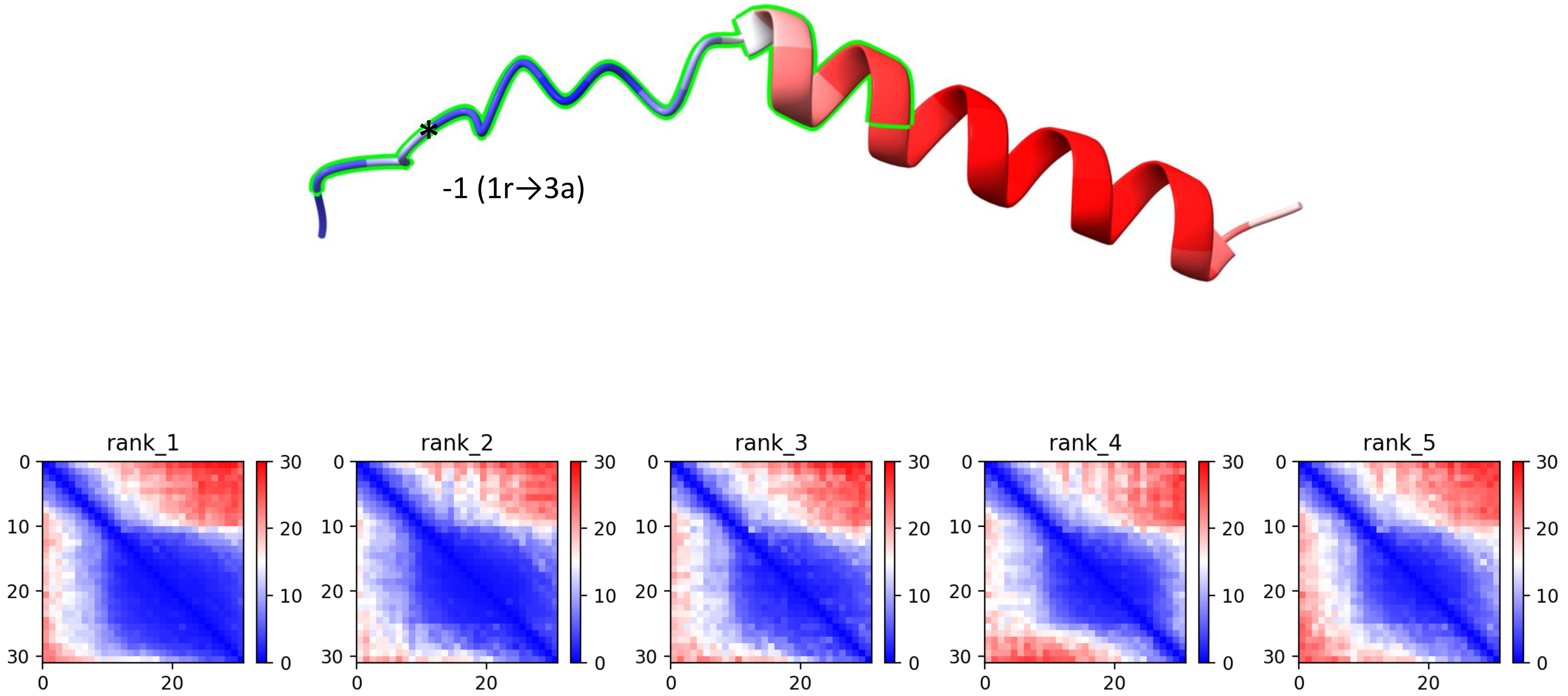

CP118: MtrunA17_Chr7g0251971

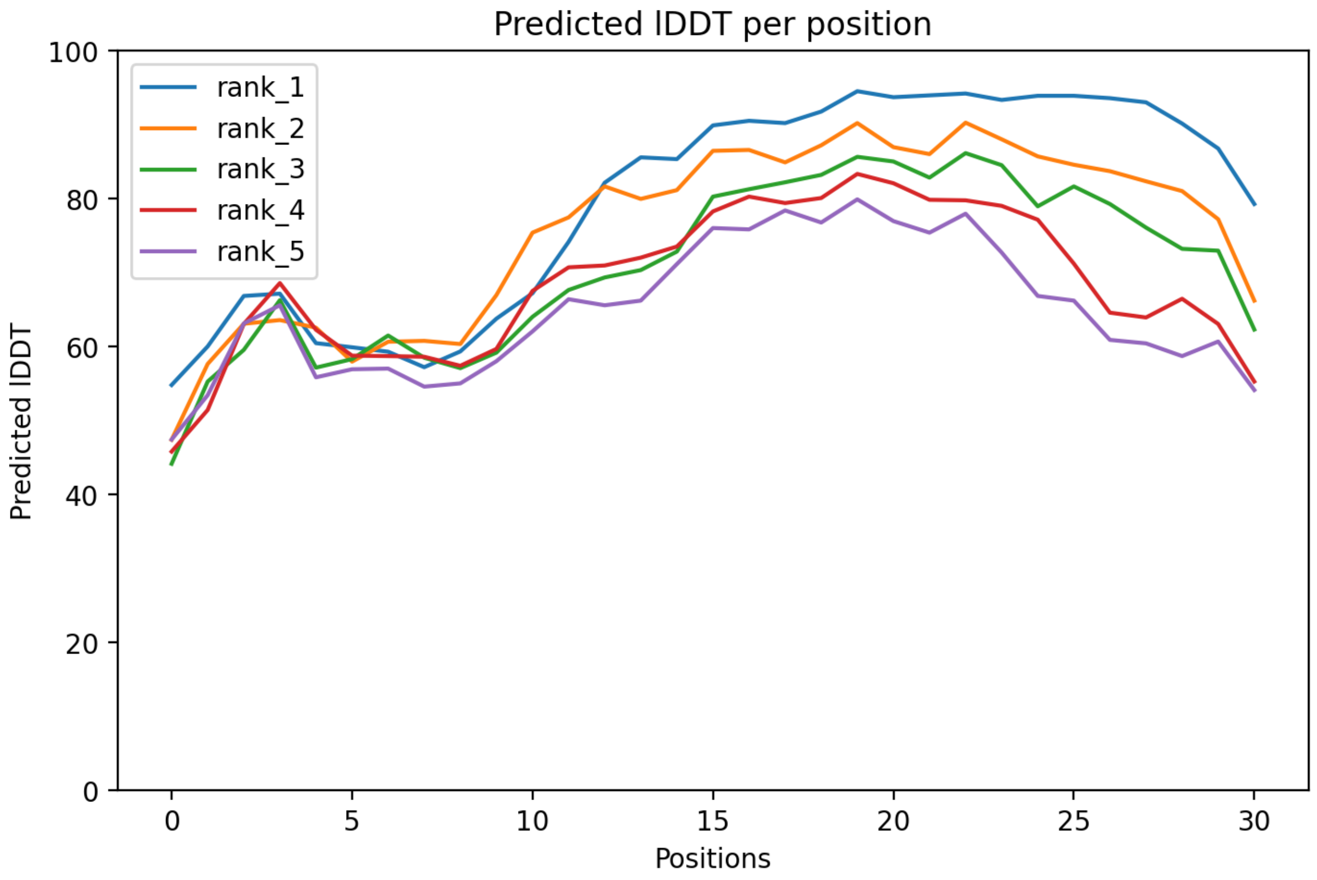

CP118: MtrunA17_Chr7g0251971

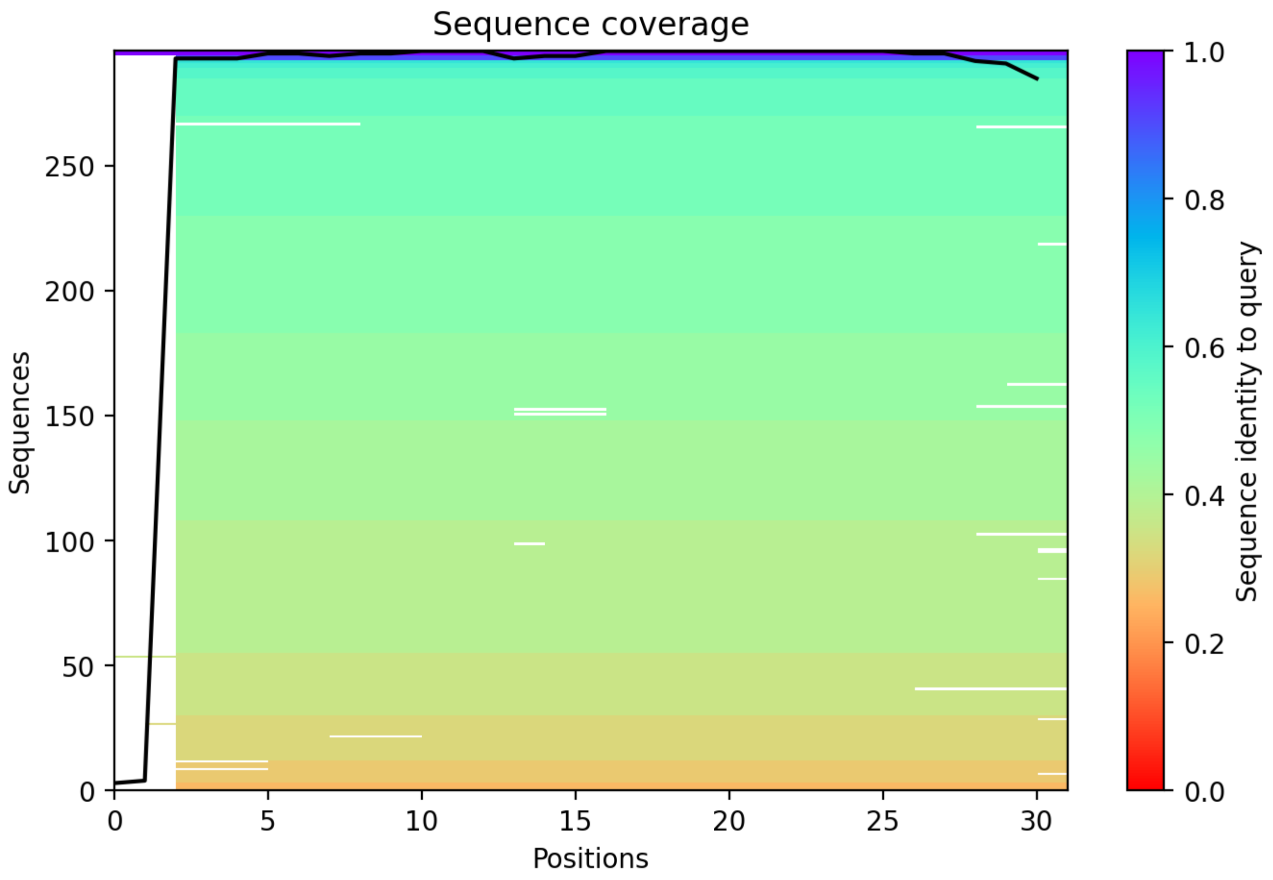

CP119: MtrunA17_Chr7g0259611

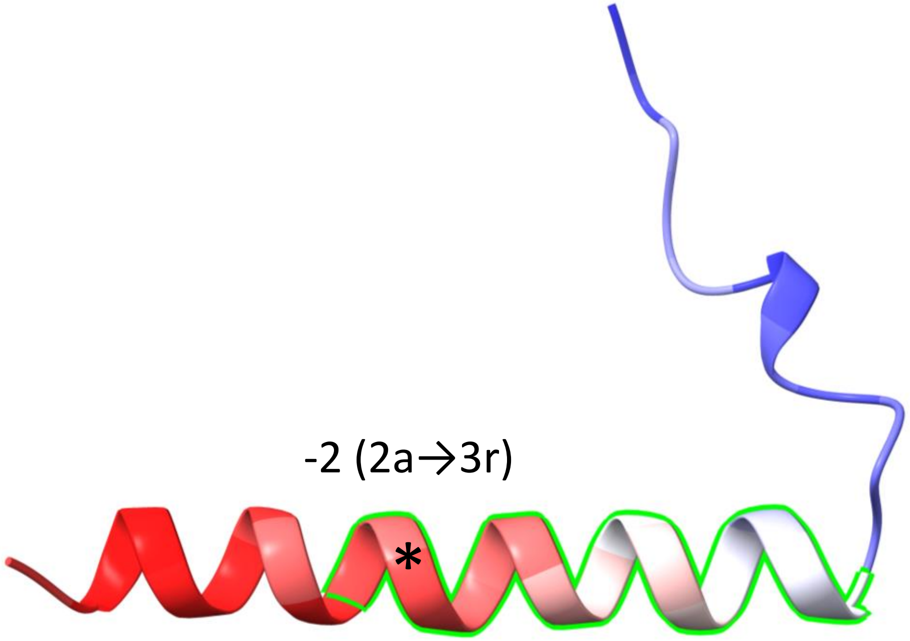

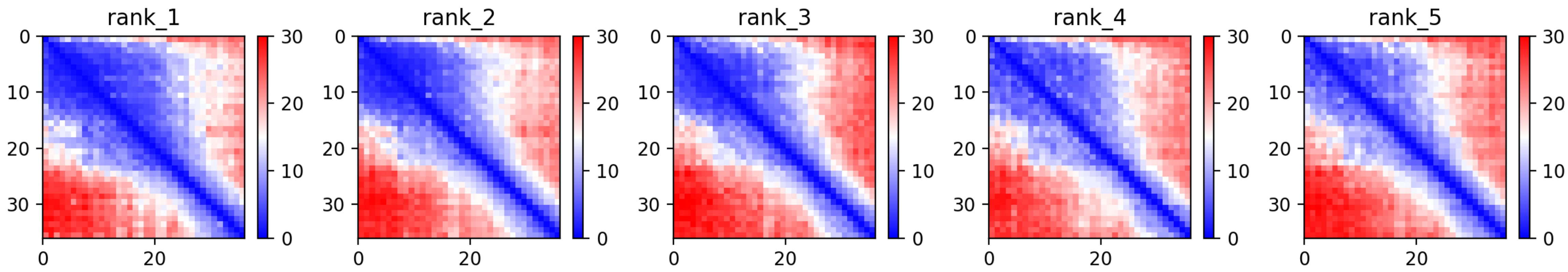

CP119: MtrunA17_Chr7g0259611

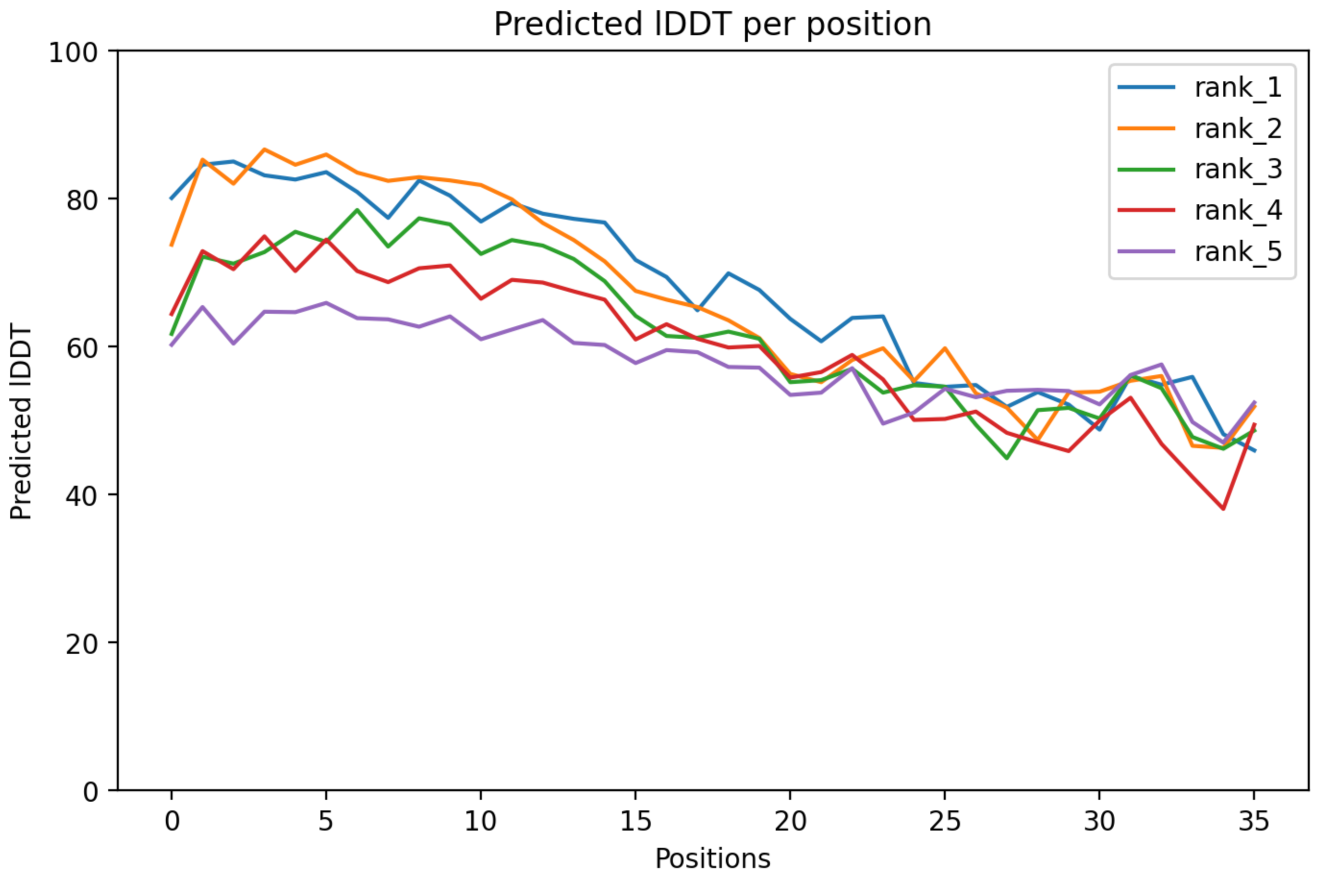

CP119: MtrunA17_Chr7g0259611

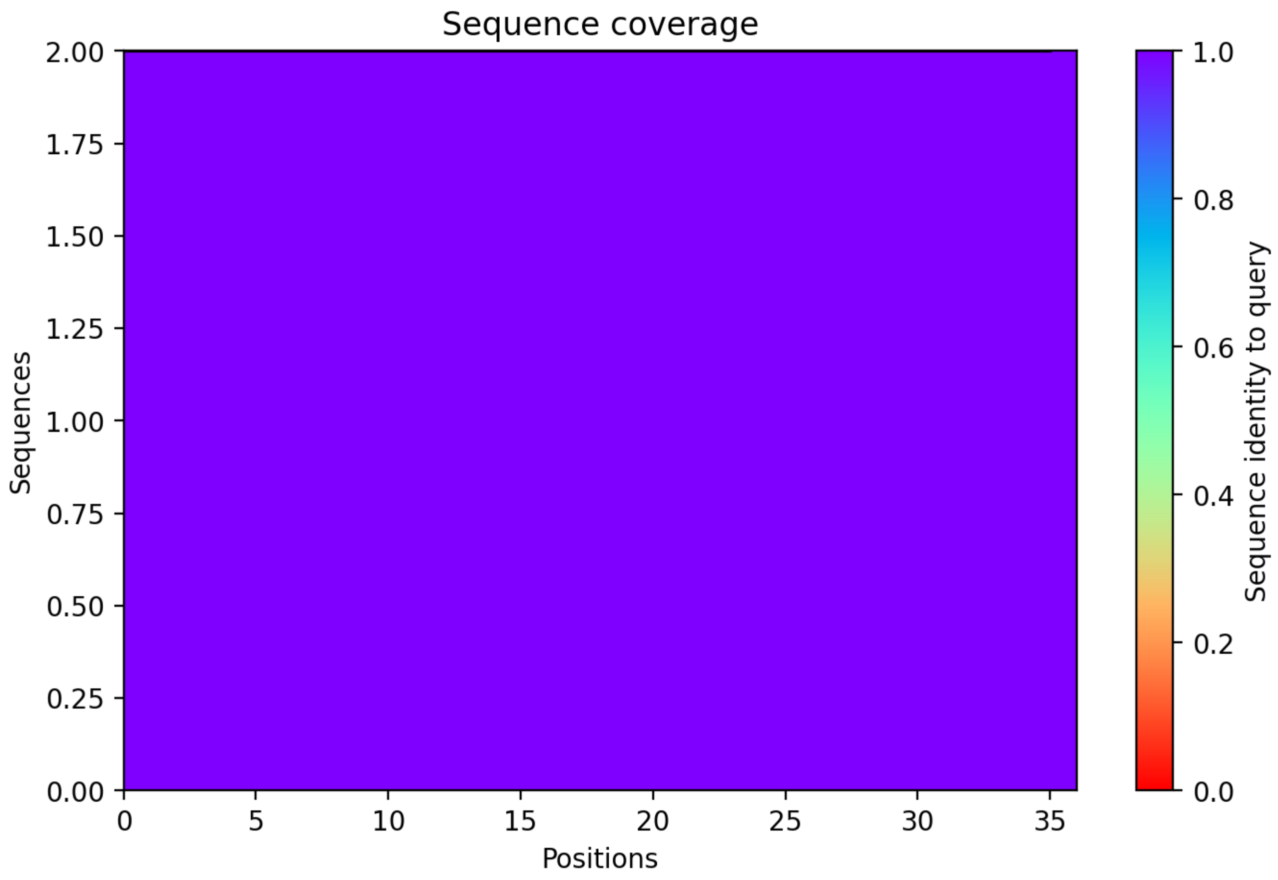

CP120: MtrunA17_Chr7g0262821

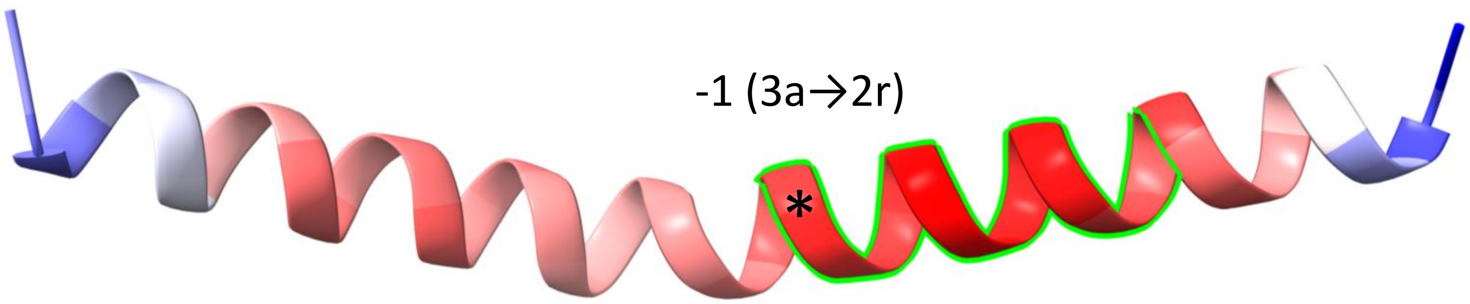

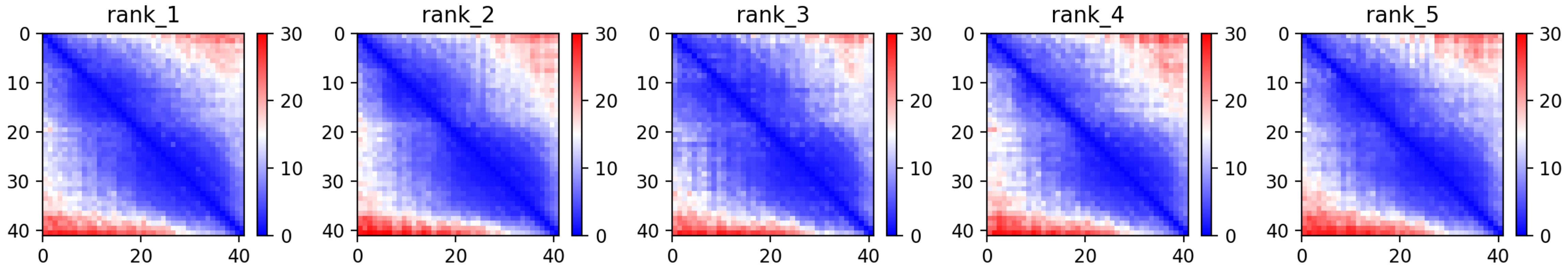

CP120: MtrunA17_Chr7g0262821

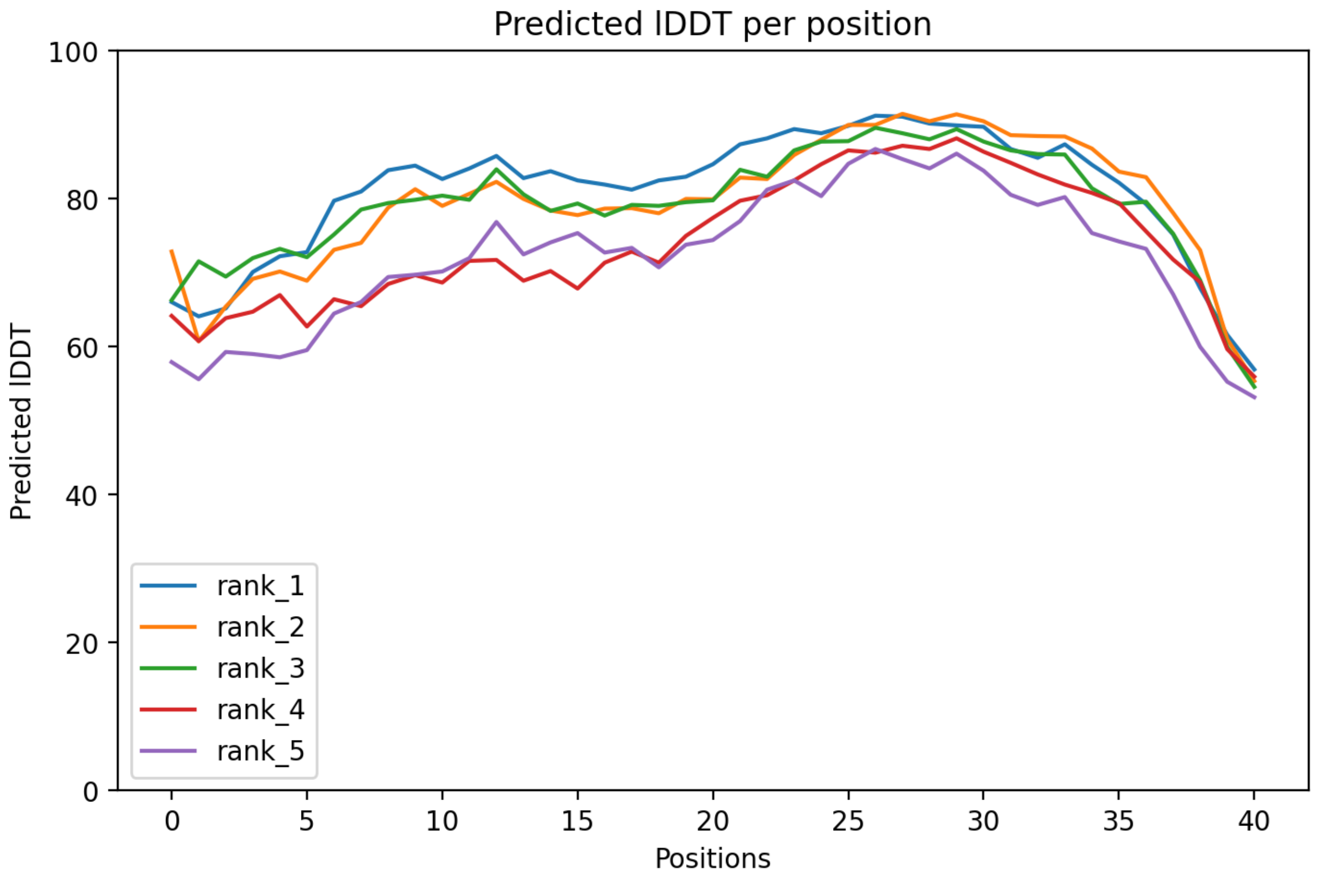

CP120: MtrunA17_Chr7g0262821

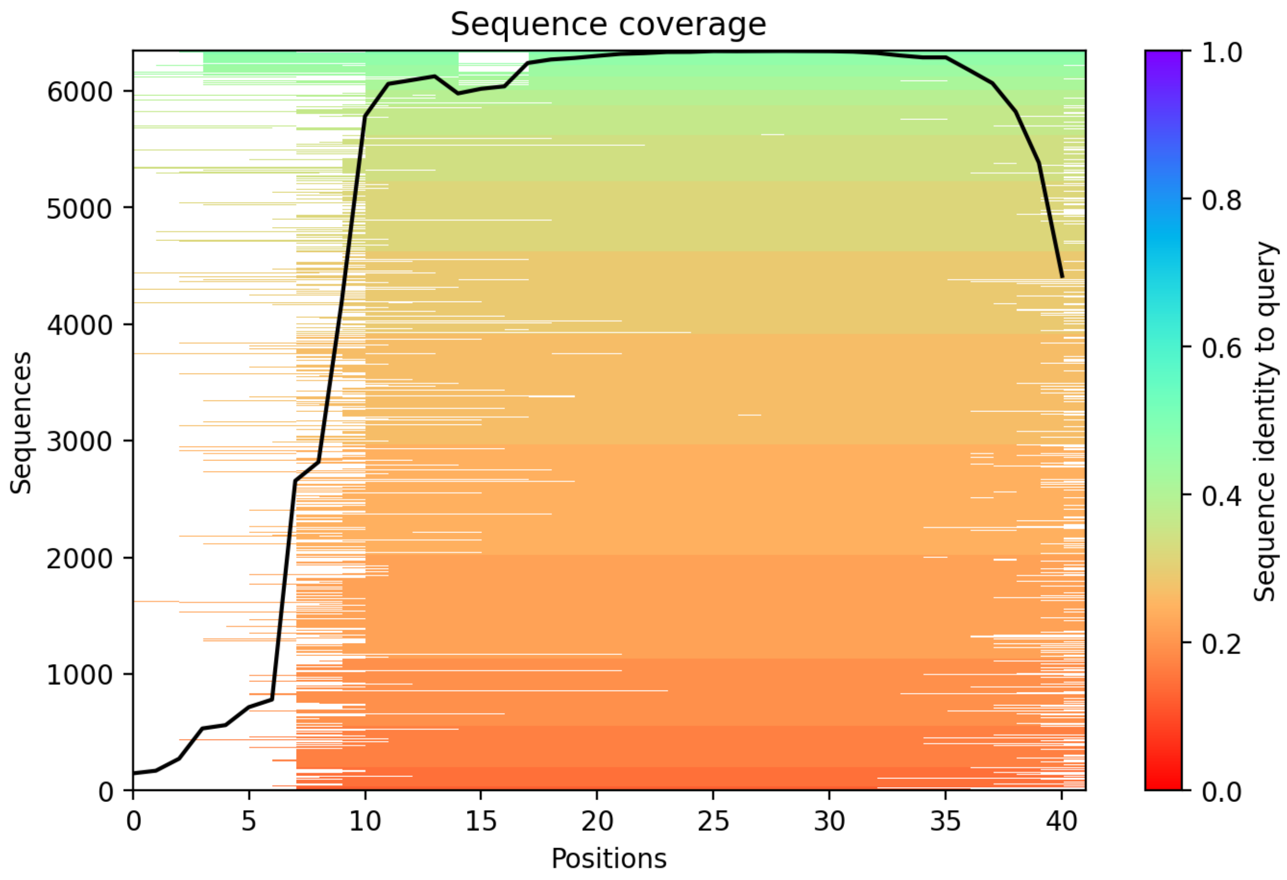

CP121: MtrunA17_Chr7g0270811

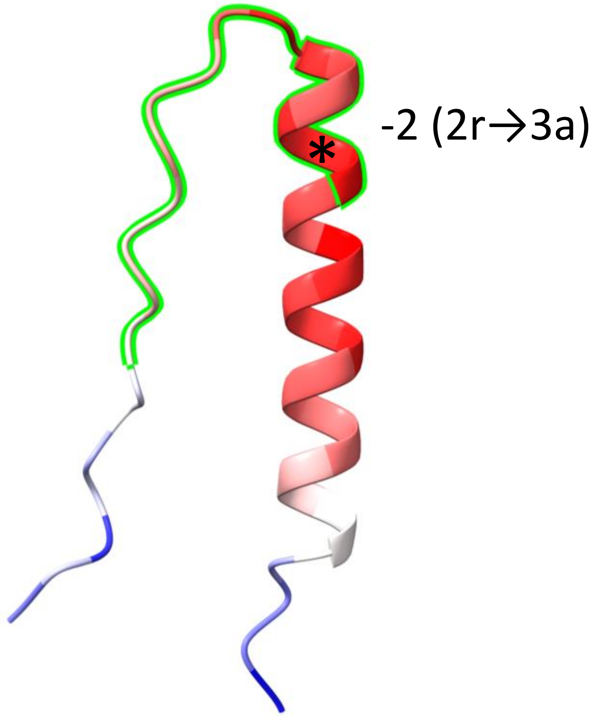

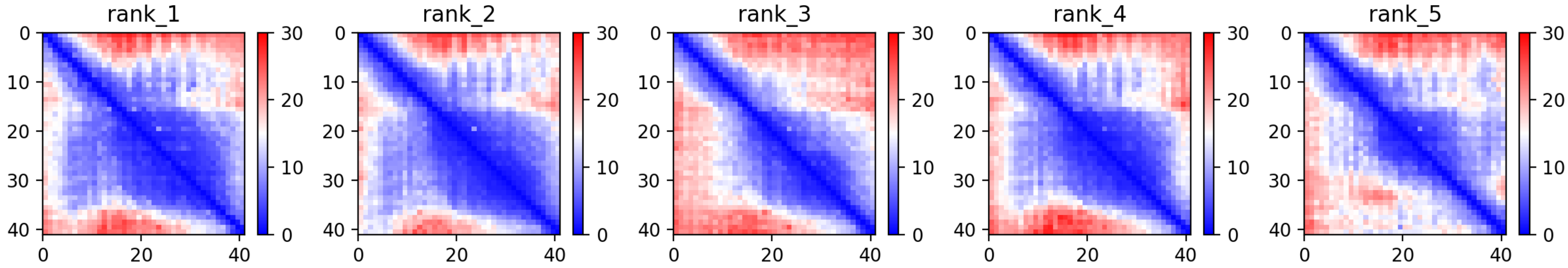

CP121: MtrunA17_Chr7g0270811

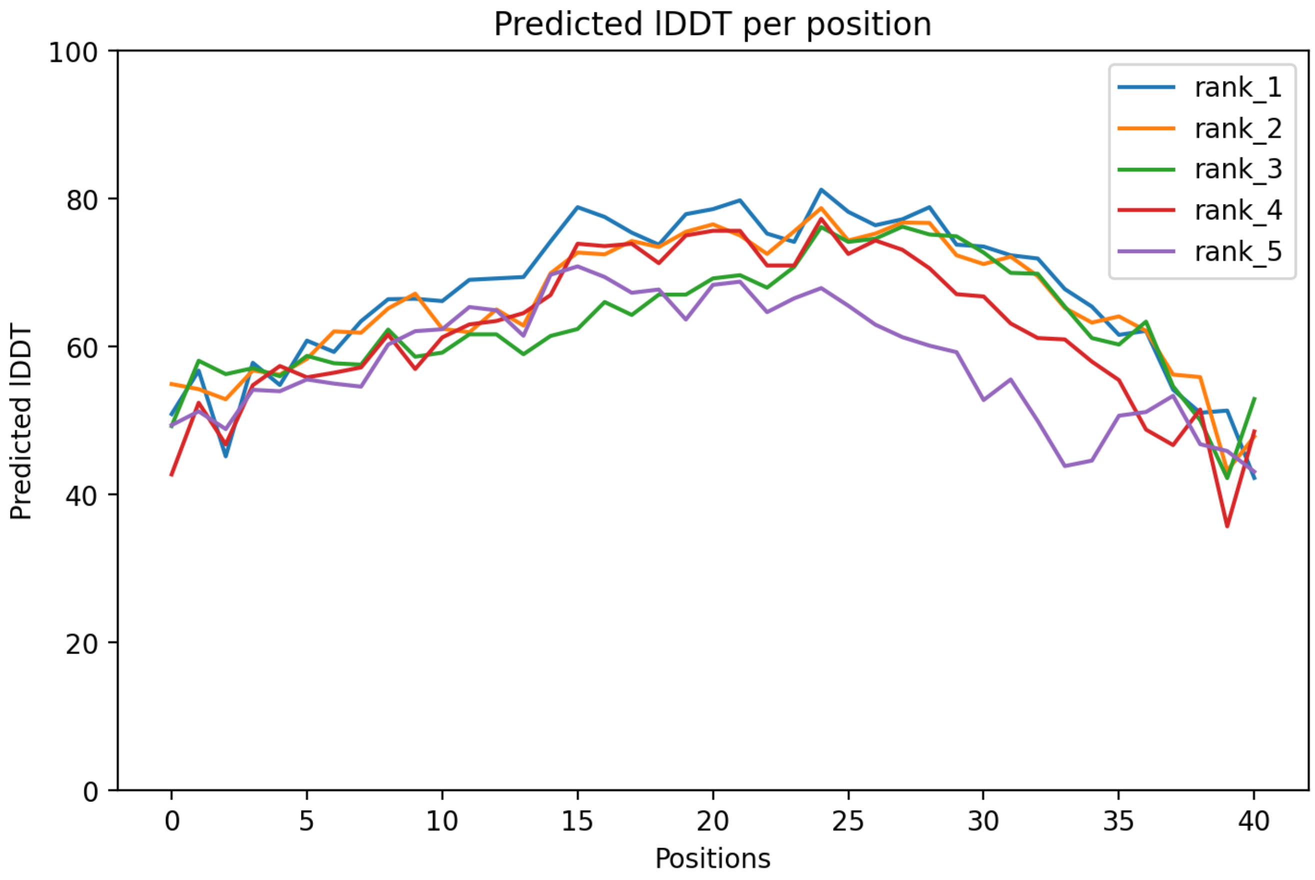

CP121: MtrunA17_Chr7g0270811

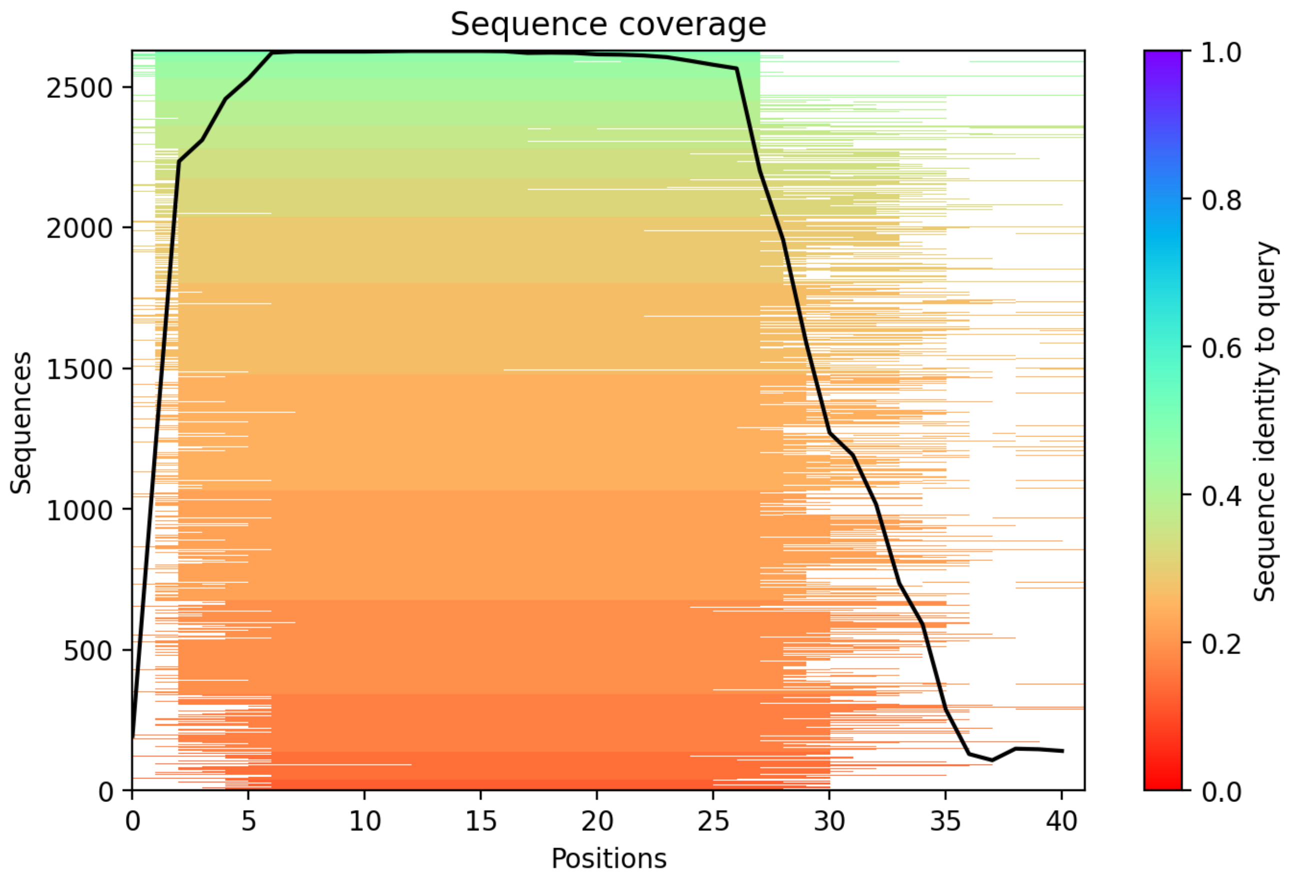

CP122: MtrunA17_Chr7g1034306

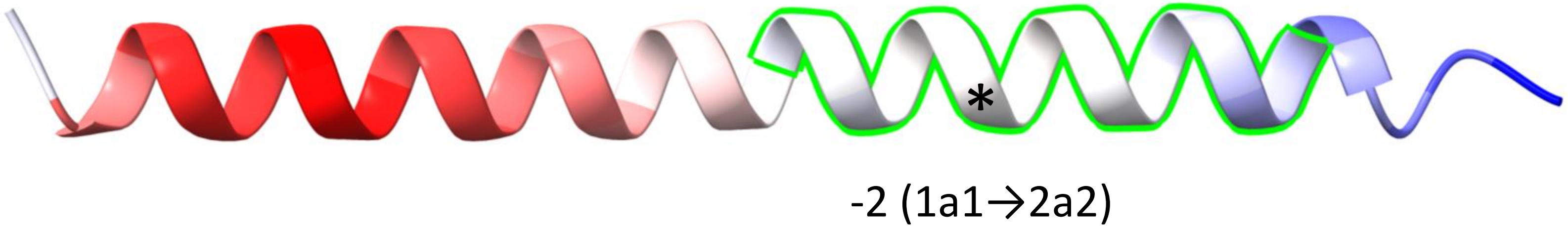

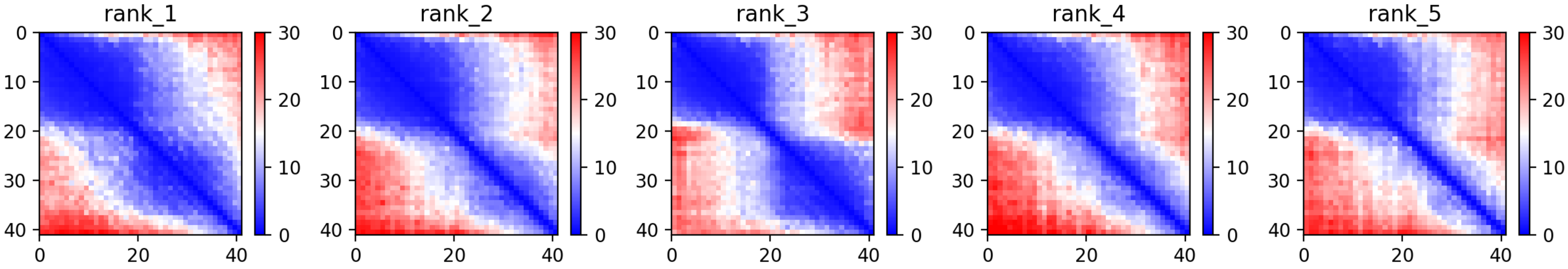

CP122: MtrunA17_Chr7g1034306

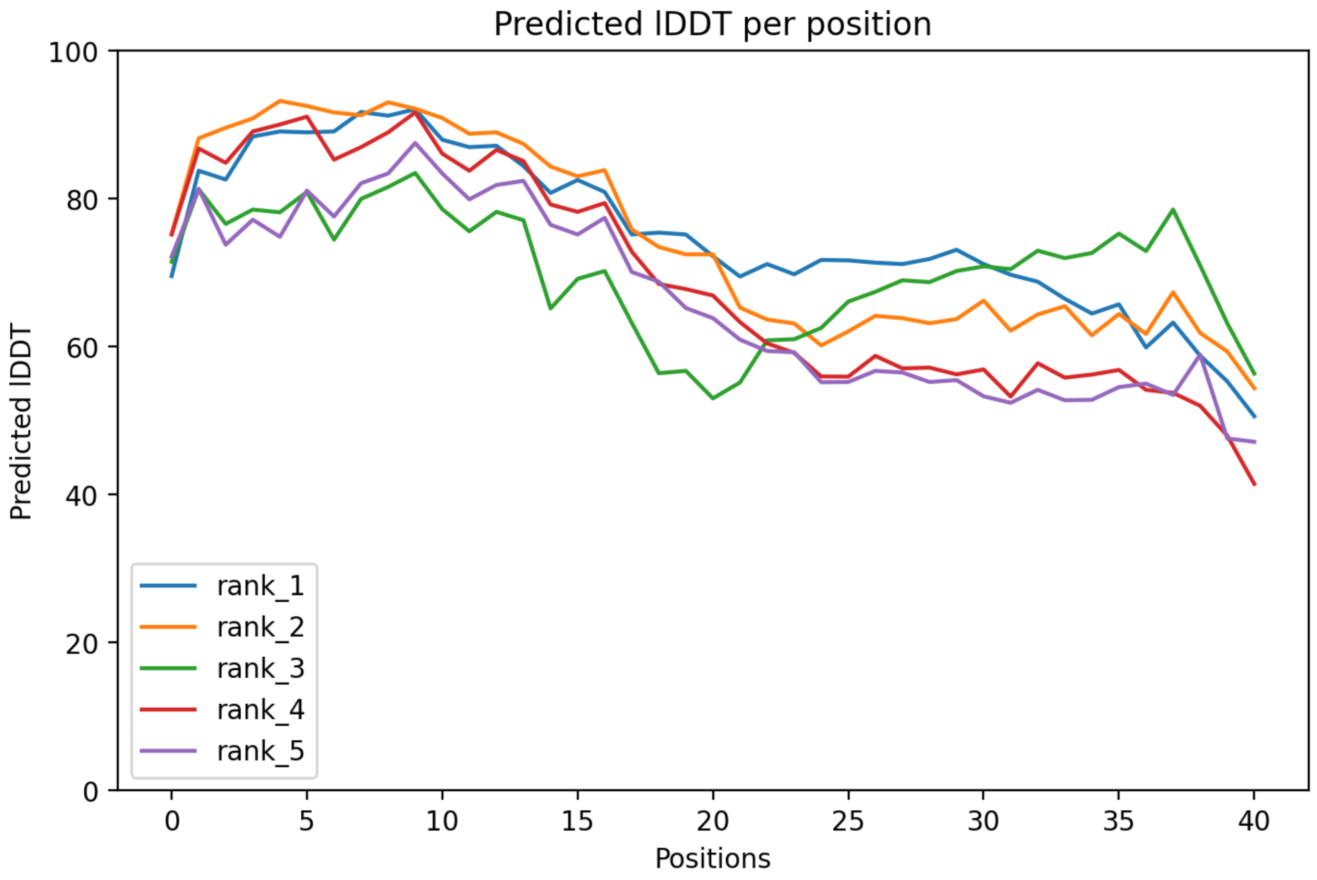

CP122: MtrunA17_Chr7g1034306

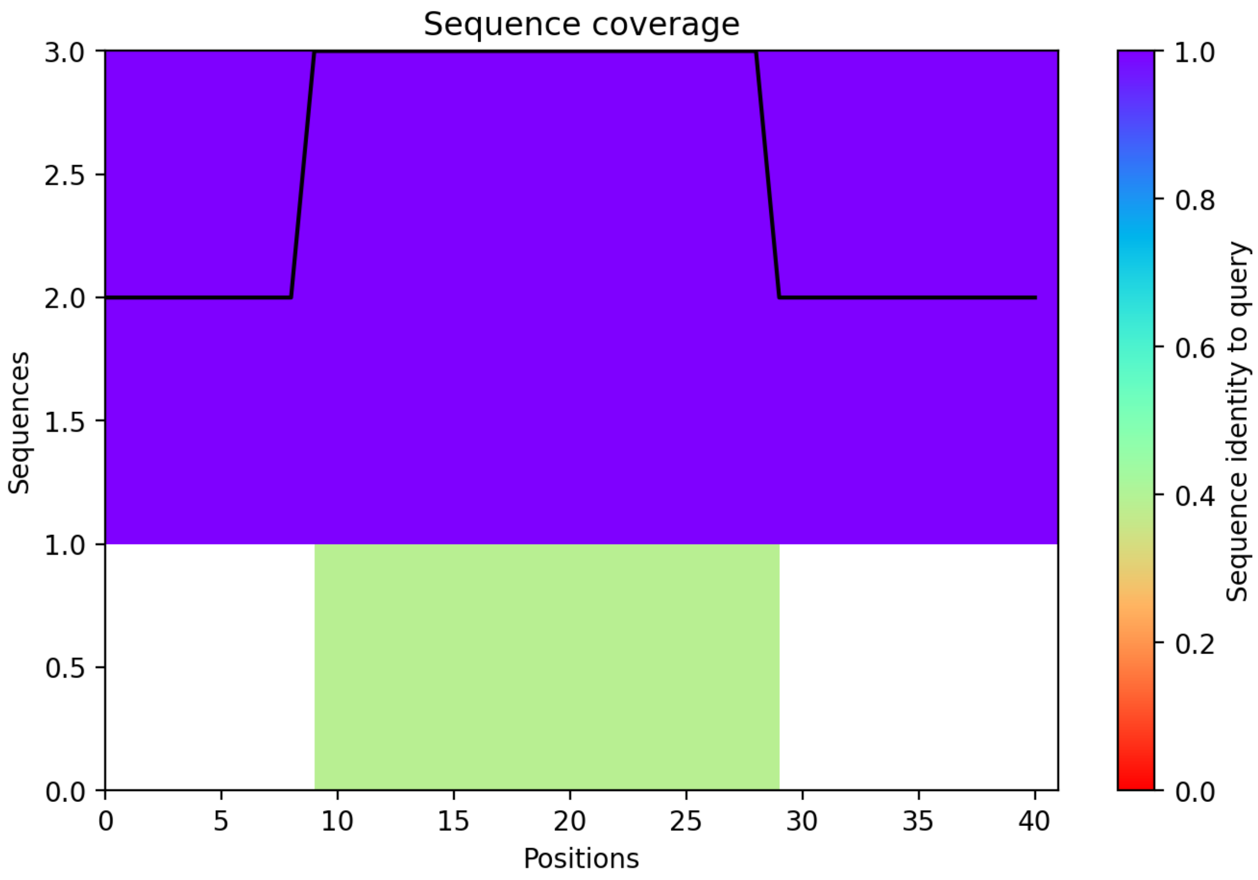

CP123: MtrunA17_Chr8g0338111

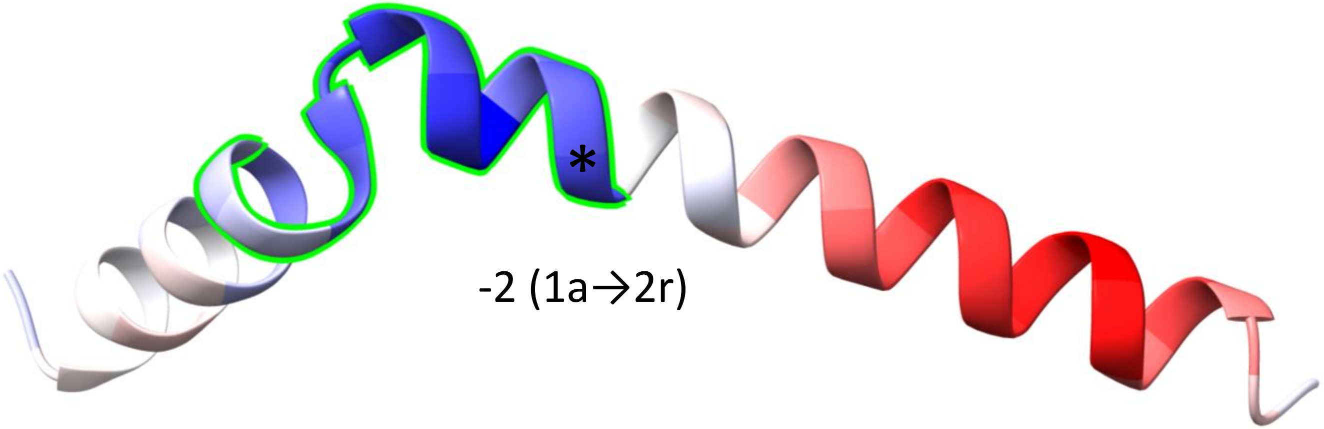

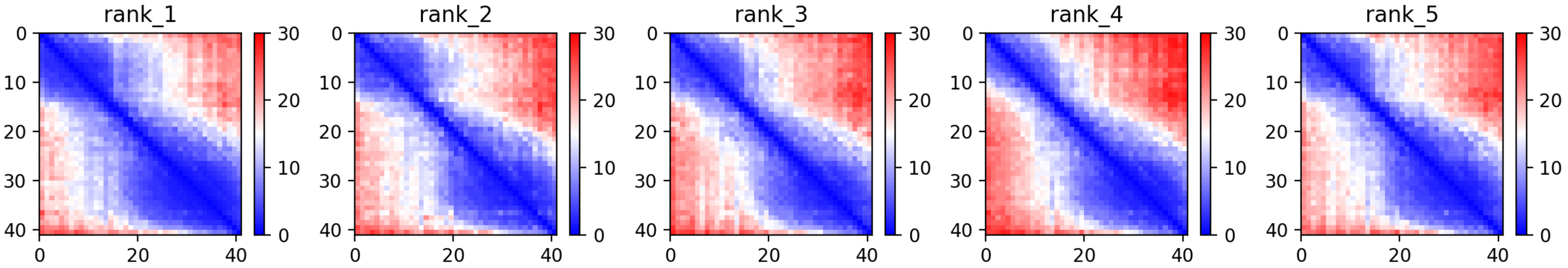

CP123: MtrunA17_Chr8g0338111

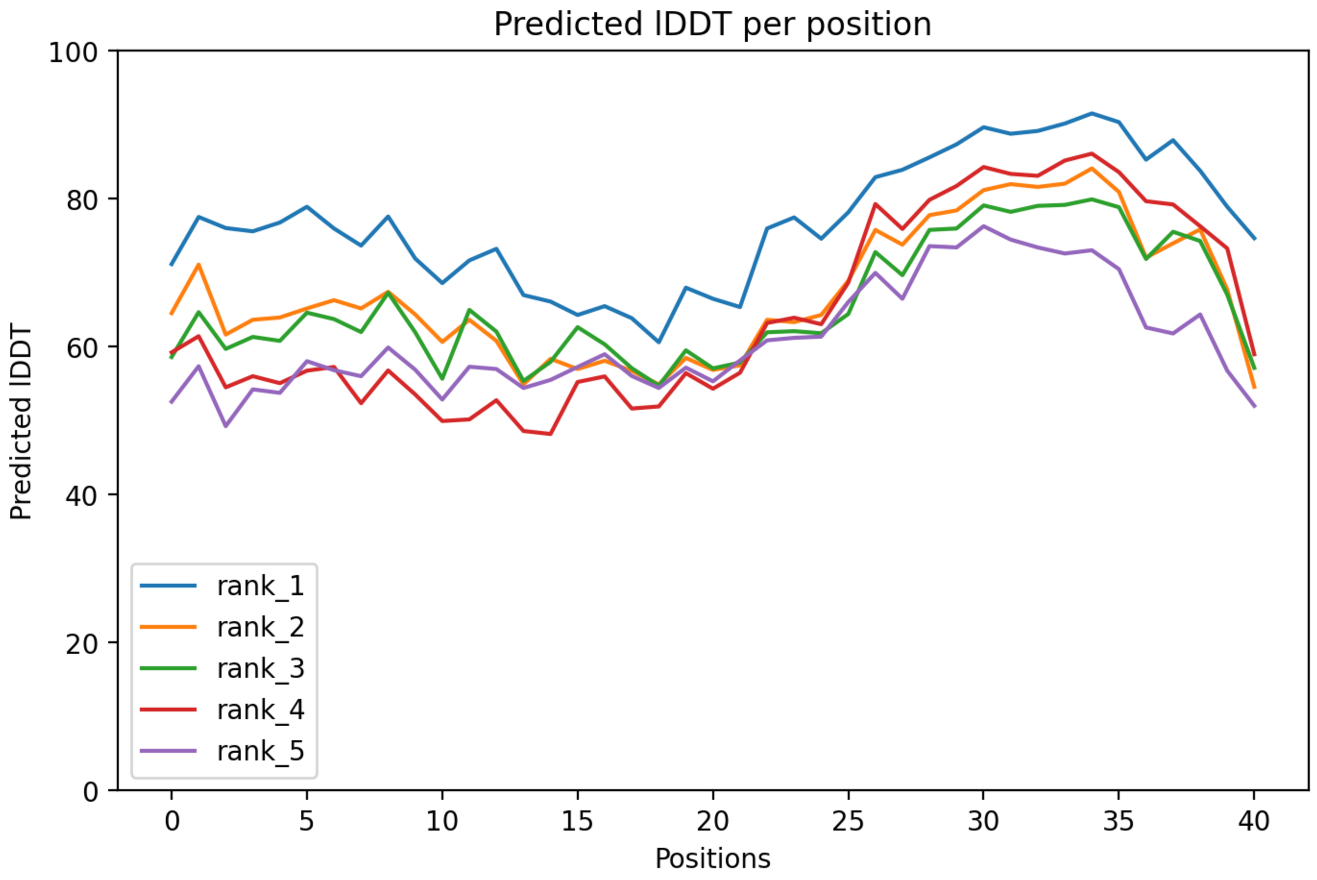

CP123: MtrunA17_Chr8g0338111

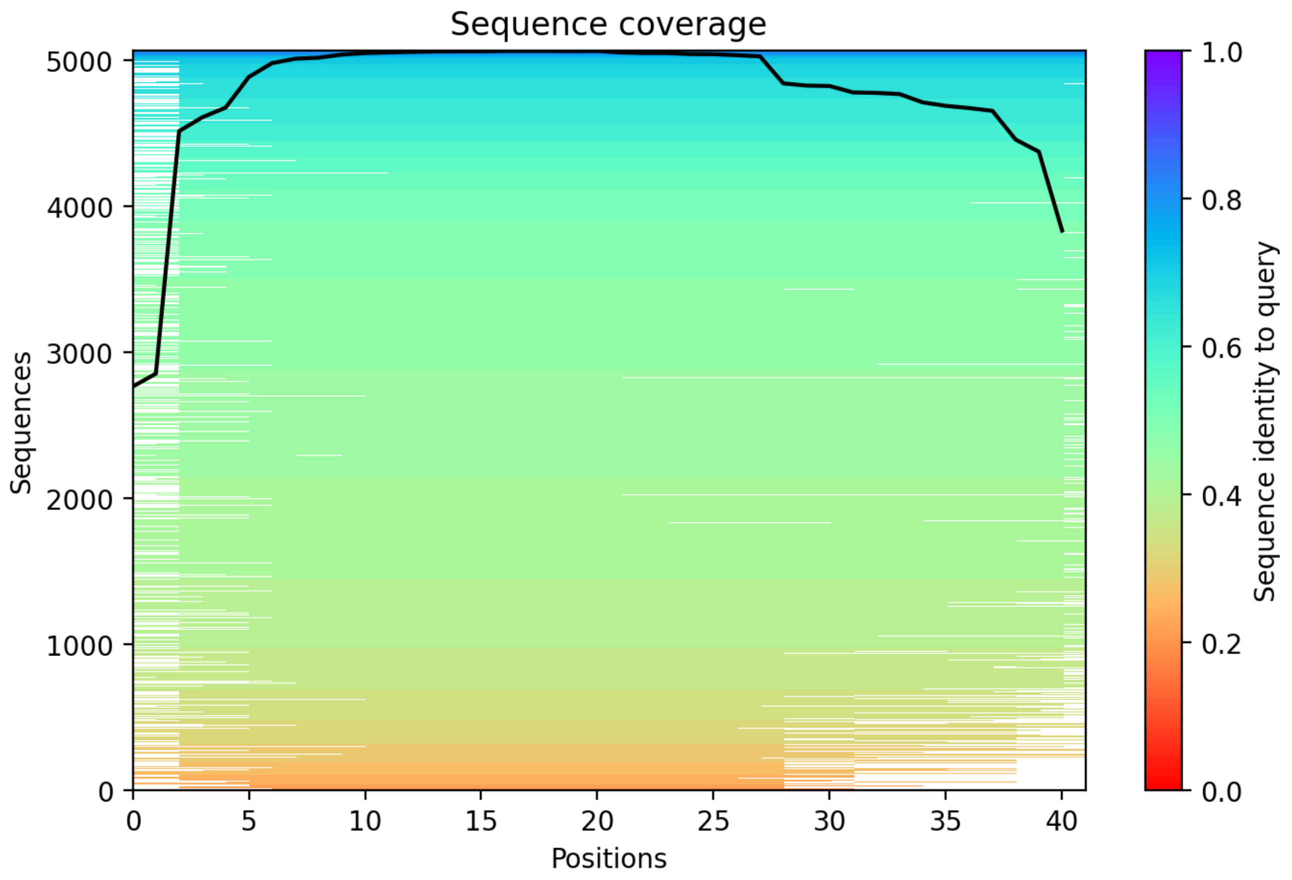

CP124: MtrunA17_Chr8g0338301

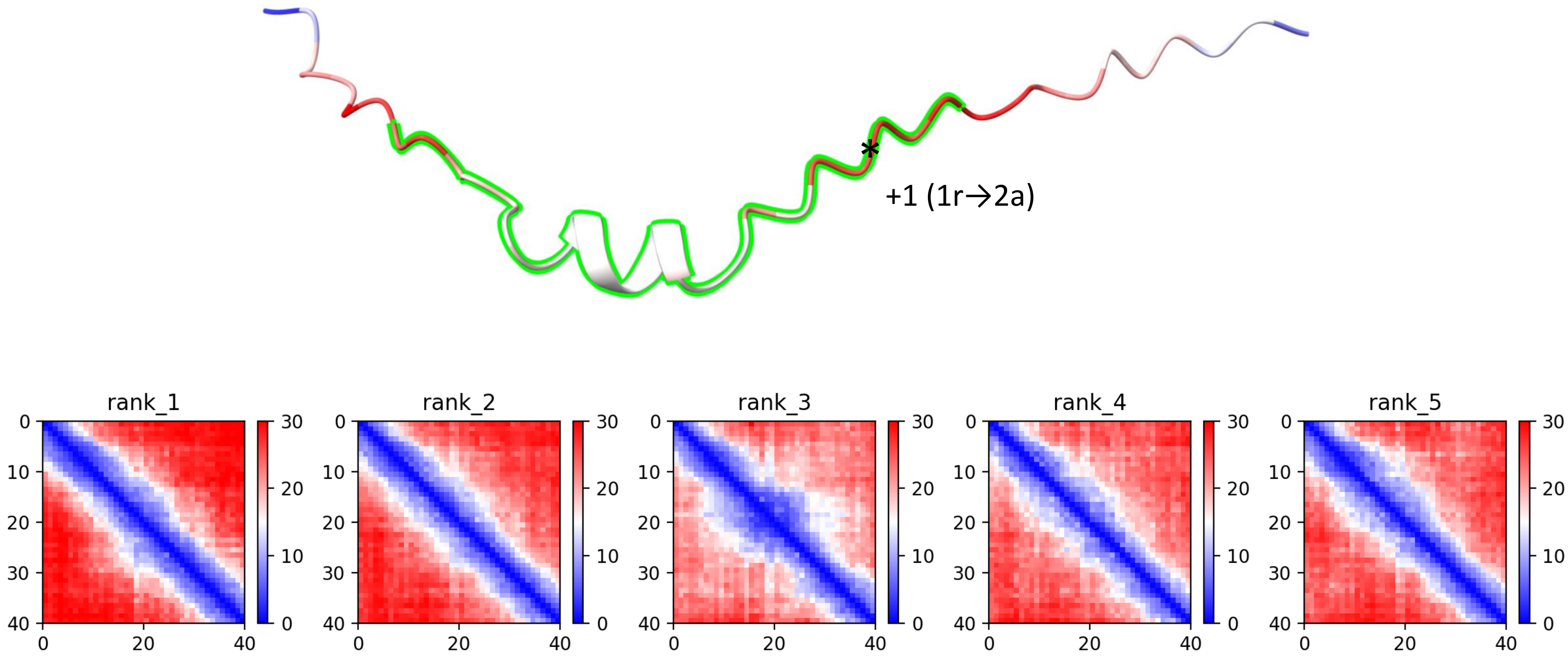

CP124: MtrunA17_Chr8g0338301

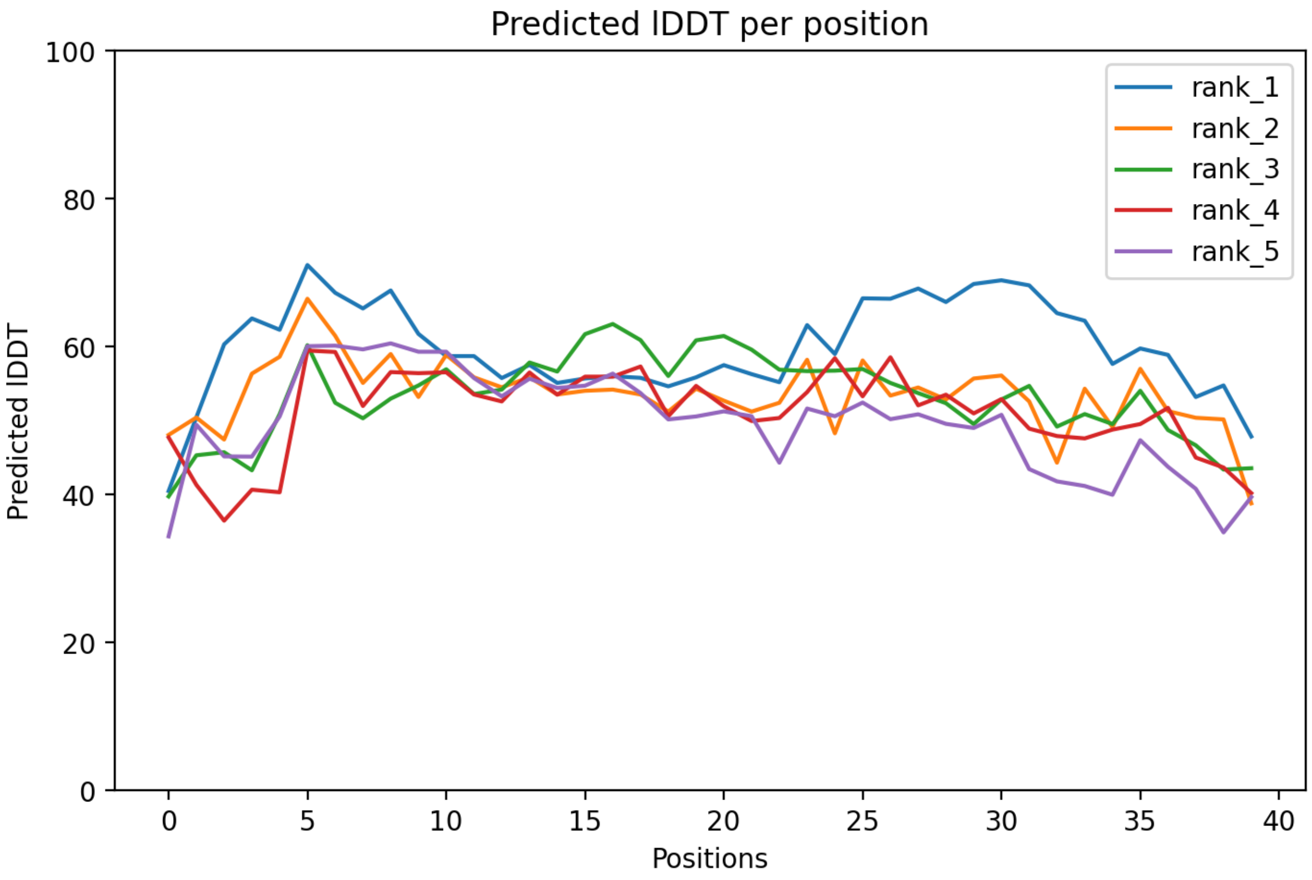

CP124: MtrunA17_Chr8g0338301

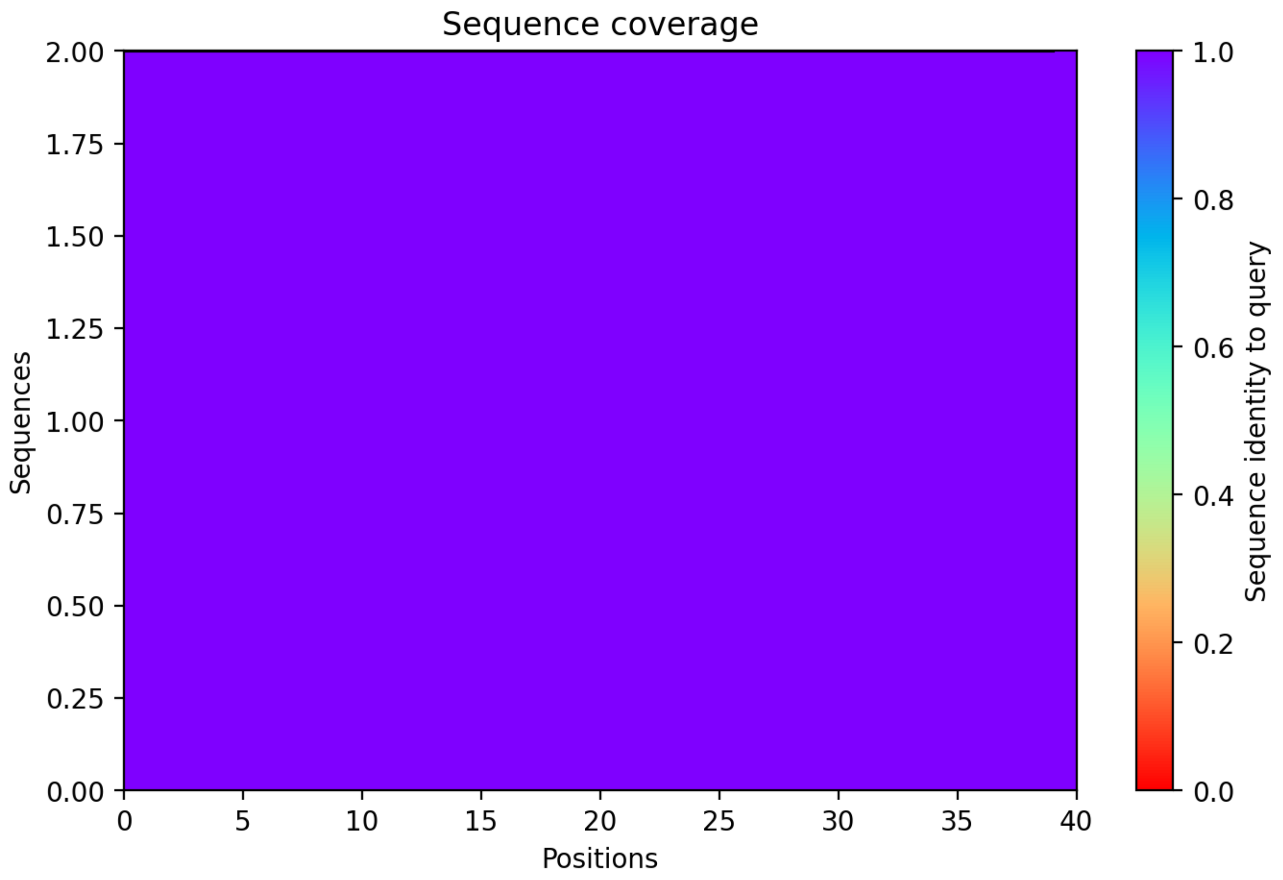

CP125: MtrunA17_Chr8g0339891

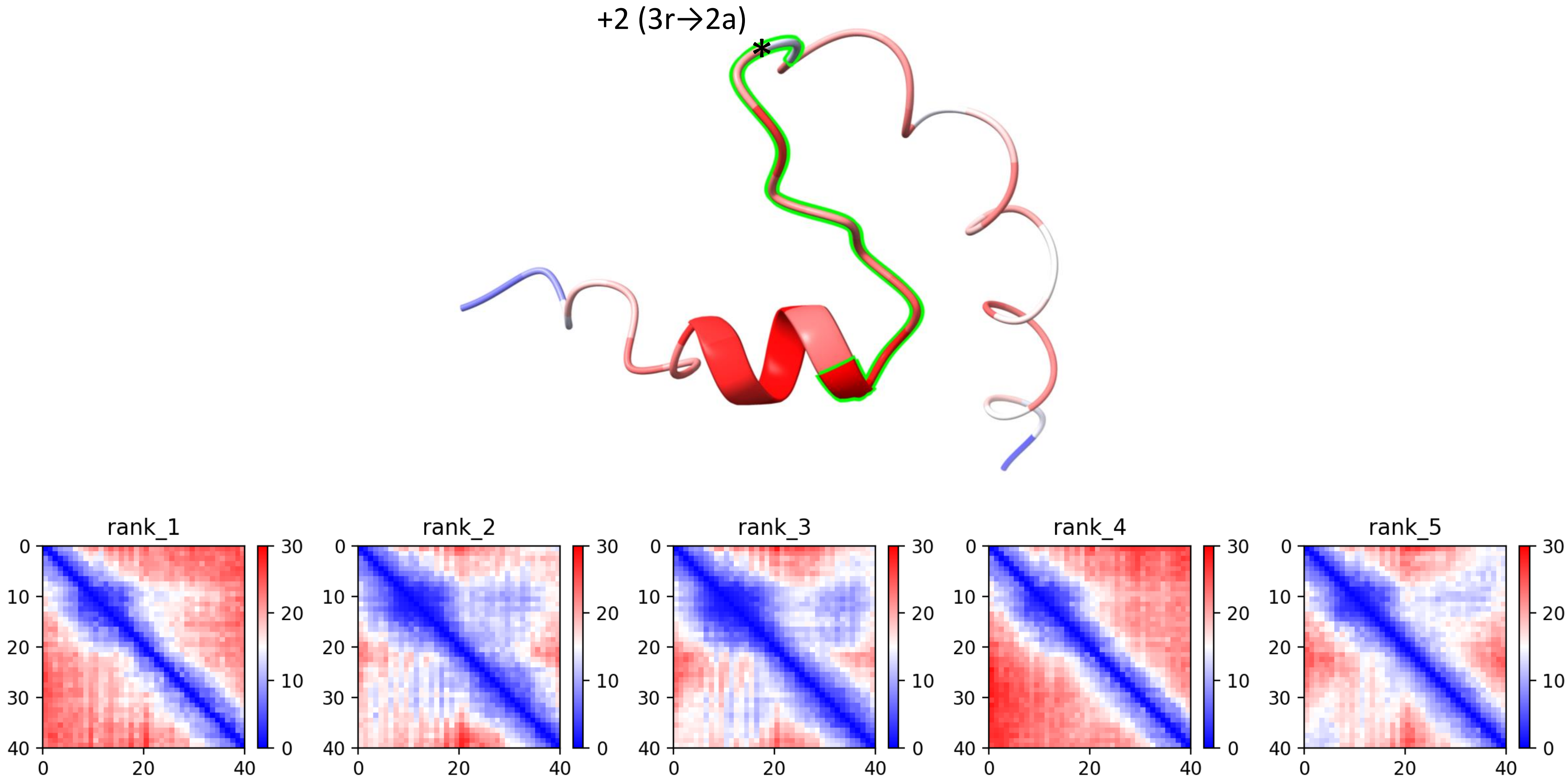

CP125: MtrunA17_Chr8g0339891

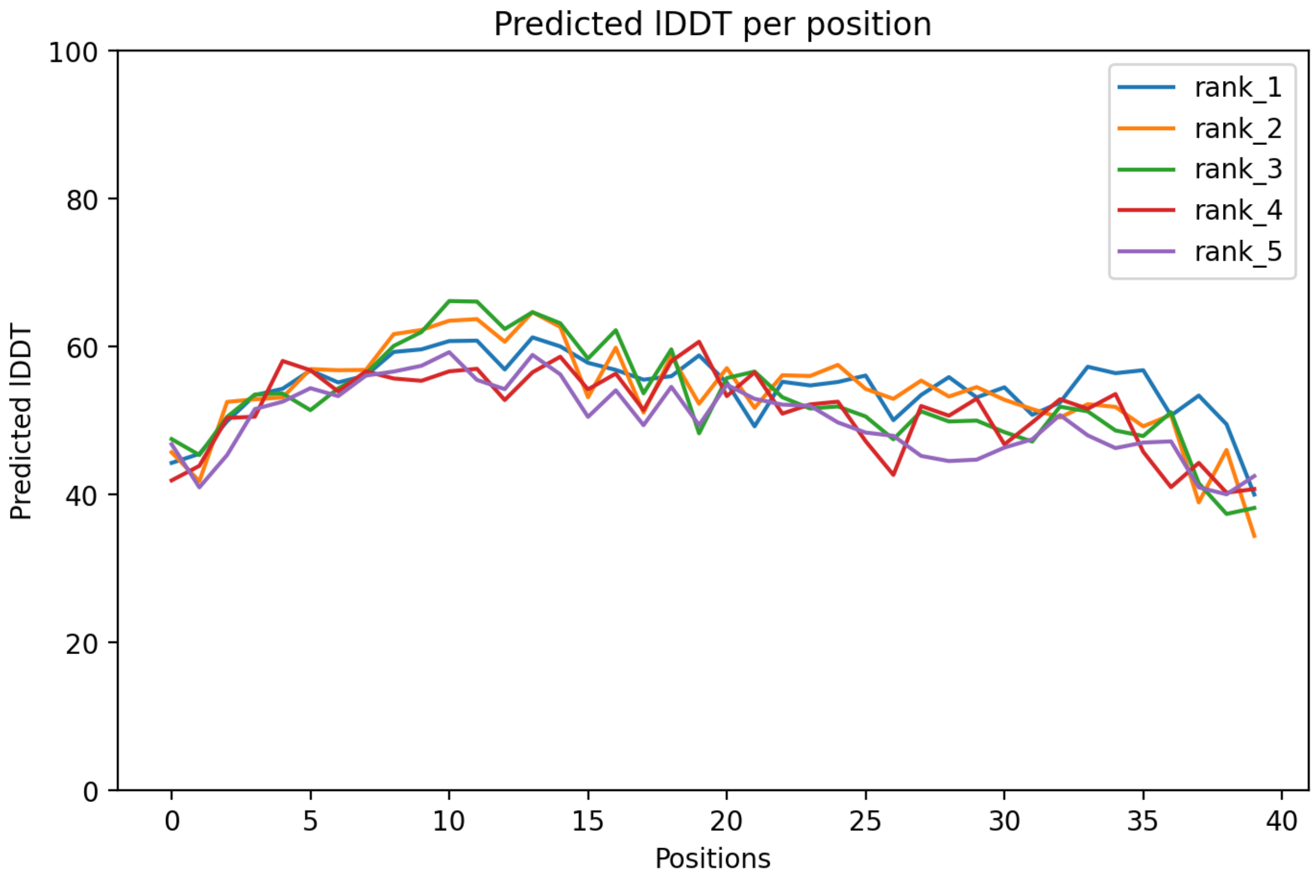

CP125: MtrunA17_Chr8g0339891

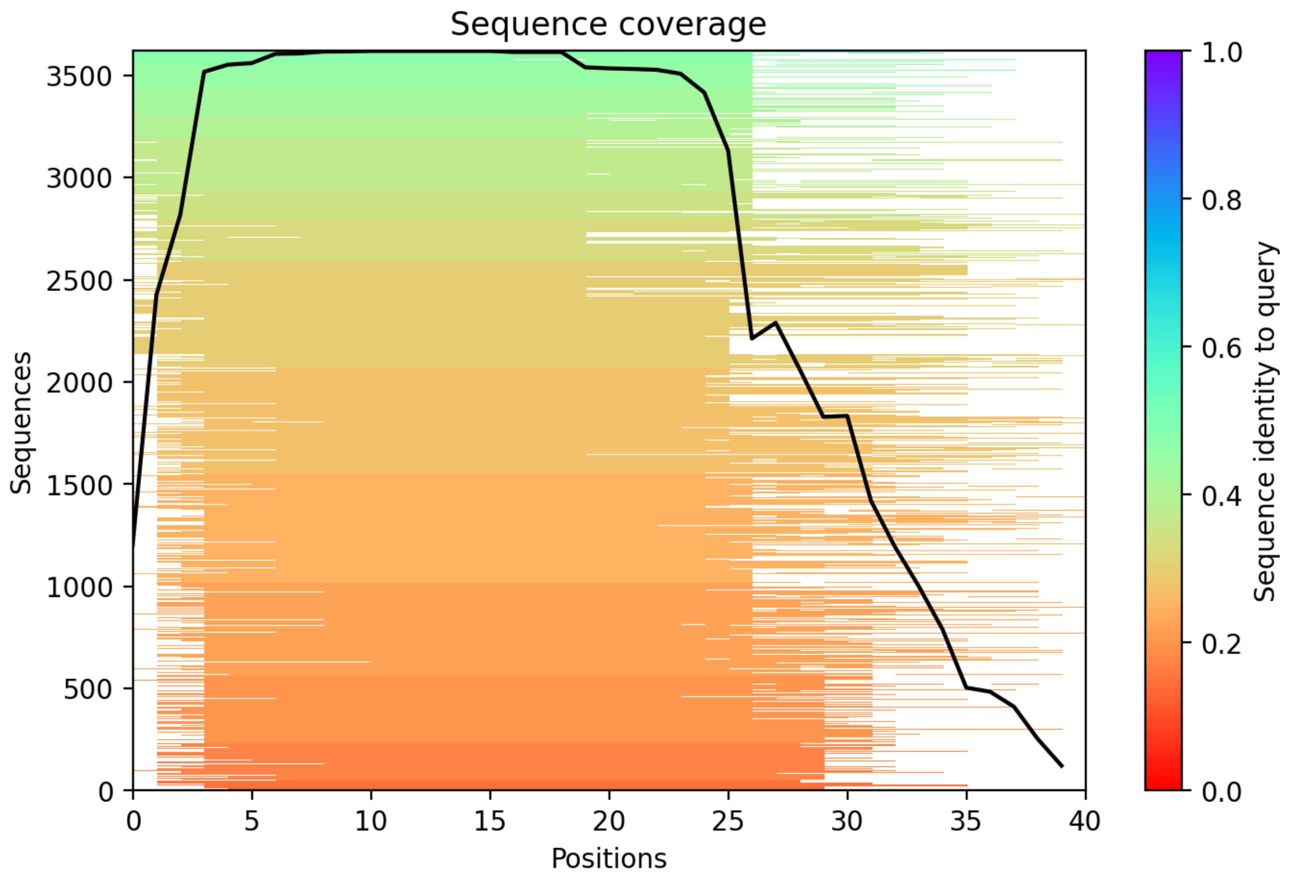

CP126: MtrunA17_Chr8g0342881

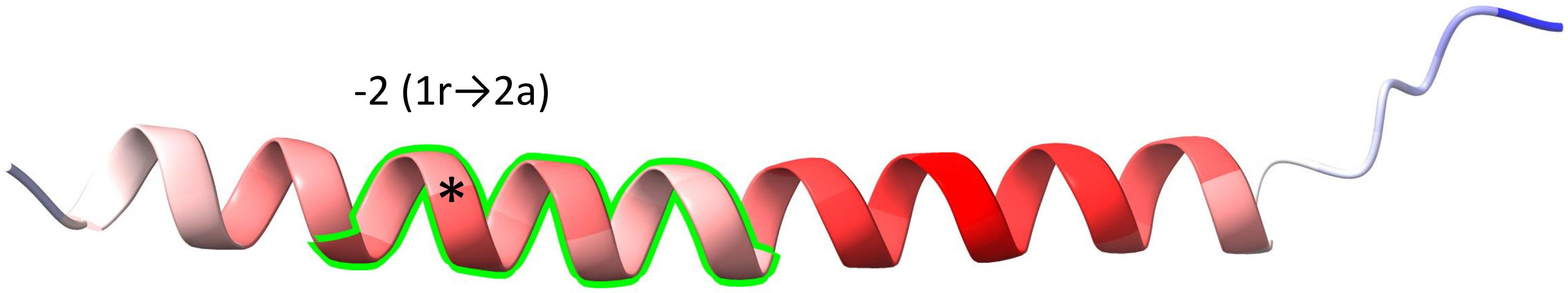

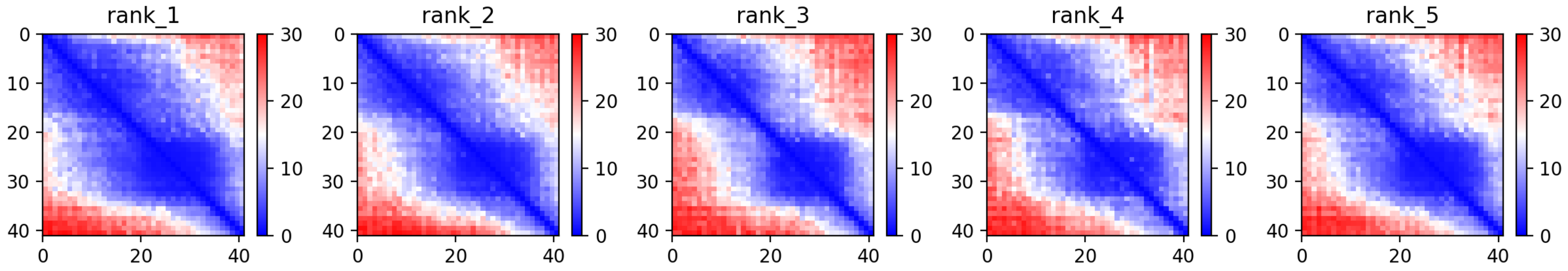

CP126: MtrunA17_Chr8g0342881

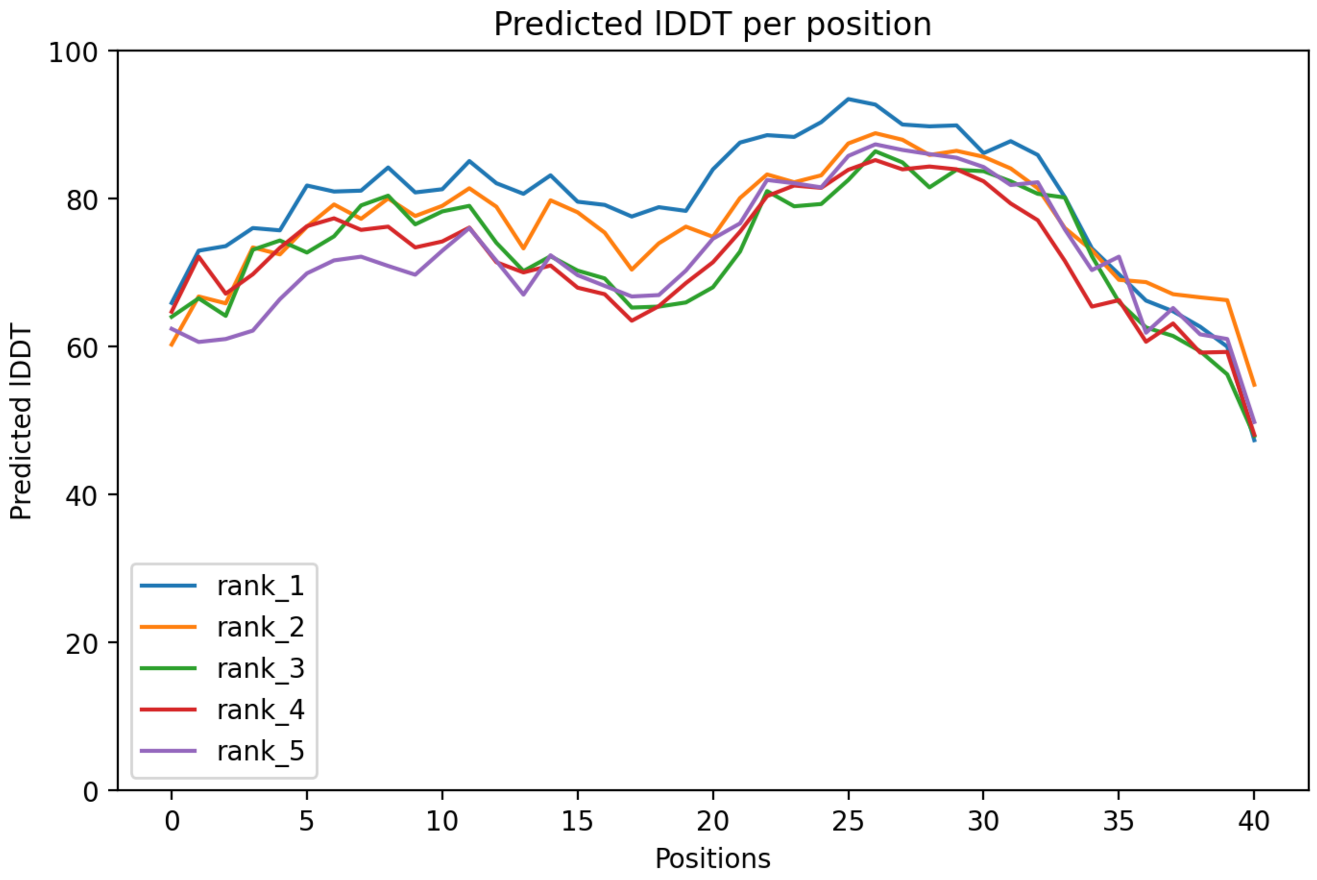

CP126: MtrunA17_Chr8g0342881

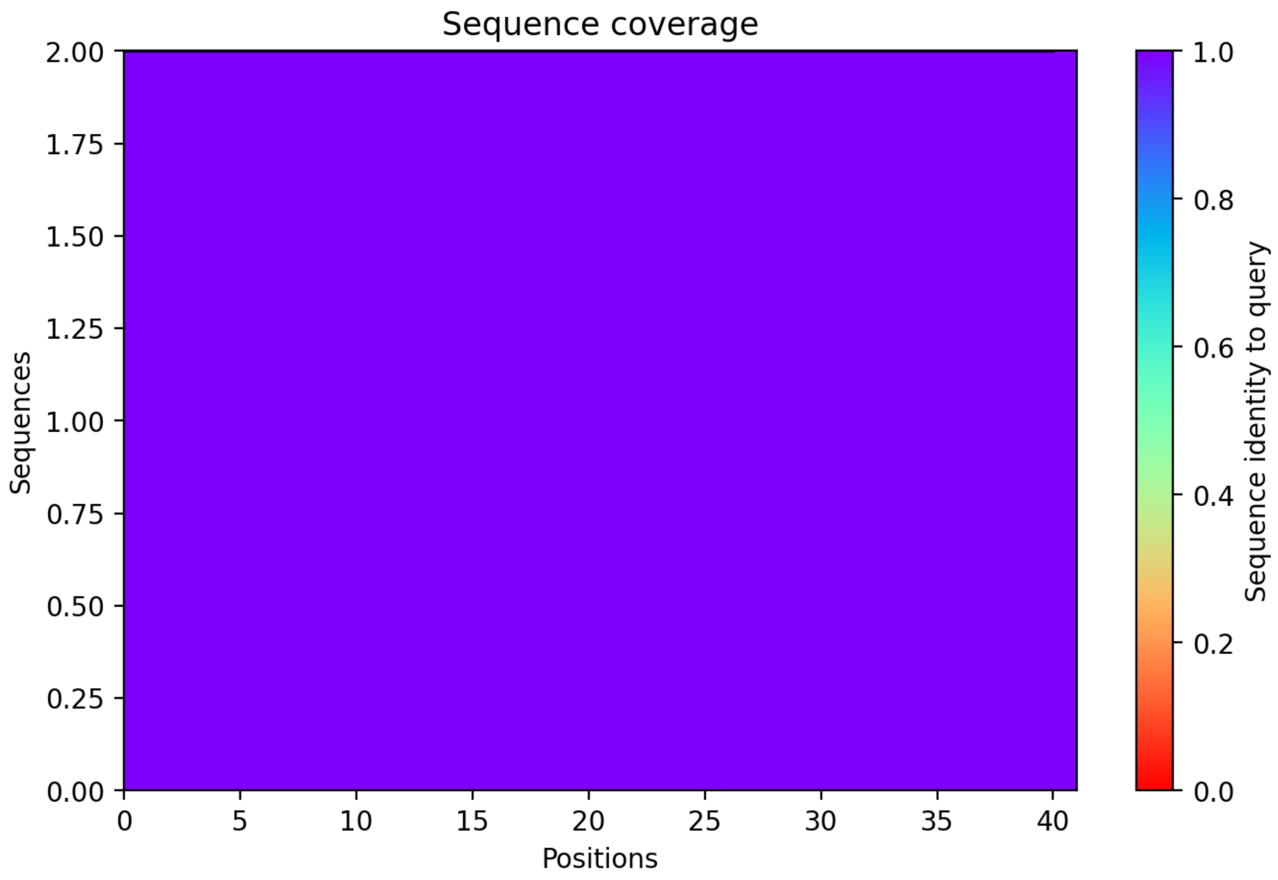

CP127: MtrunA17_Chr8g0345421

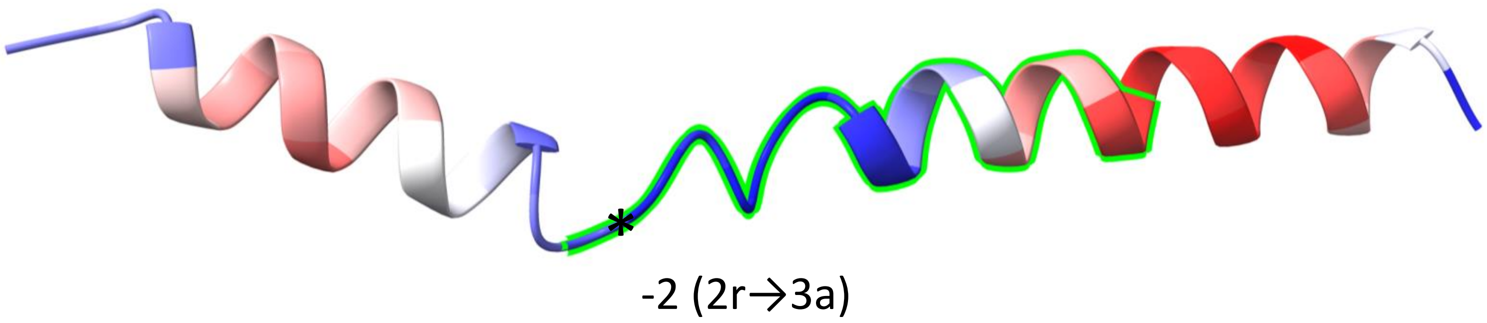

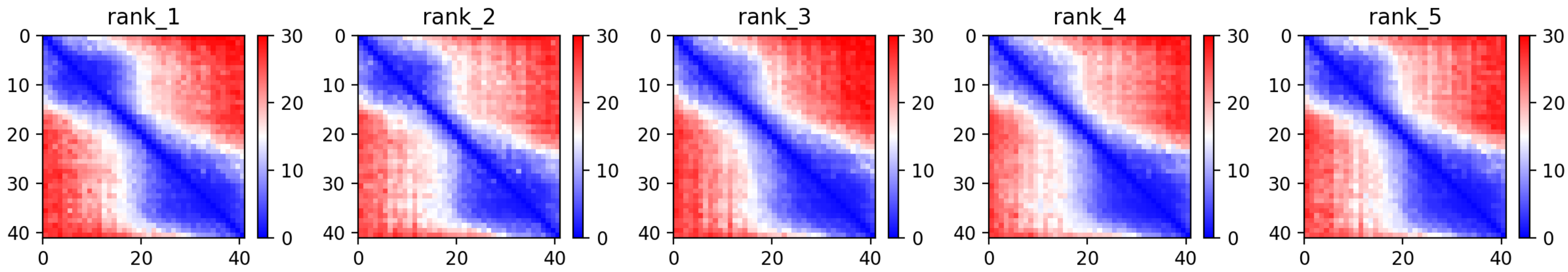

CP127: MtrunA17_Chr8g0345421

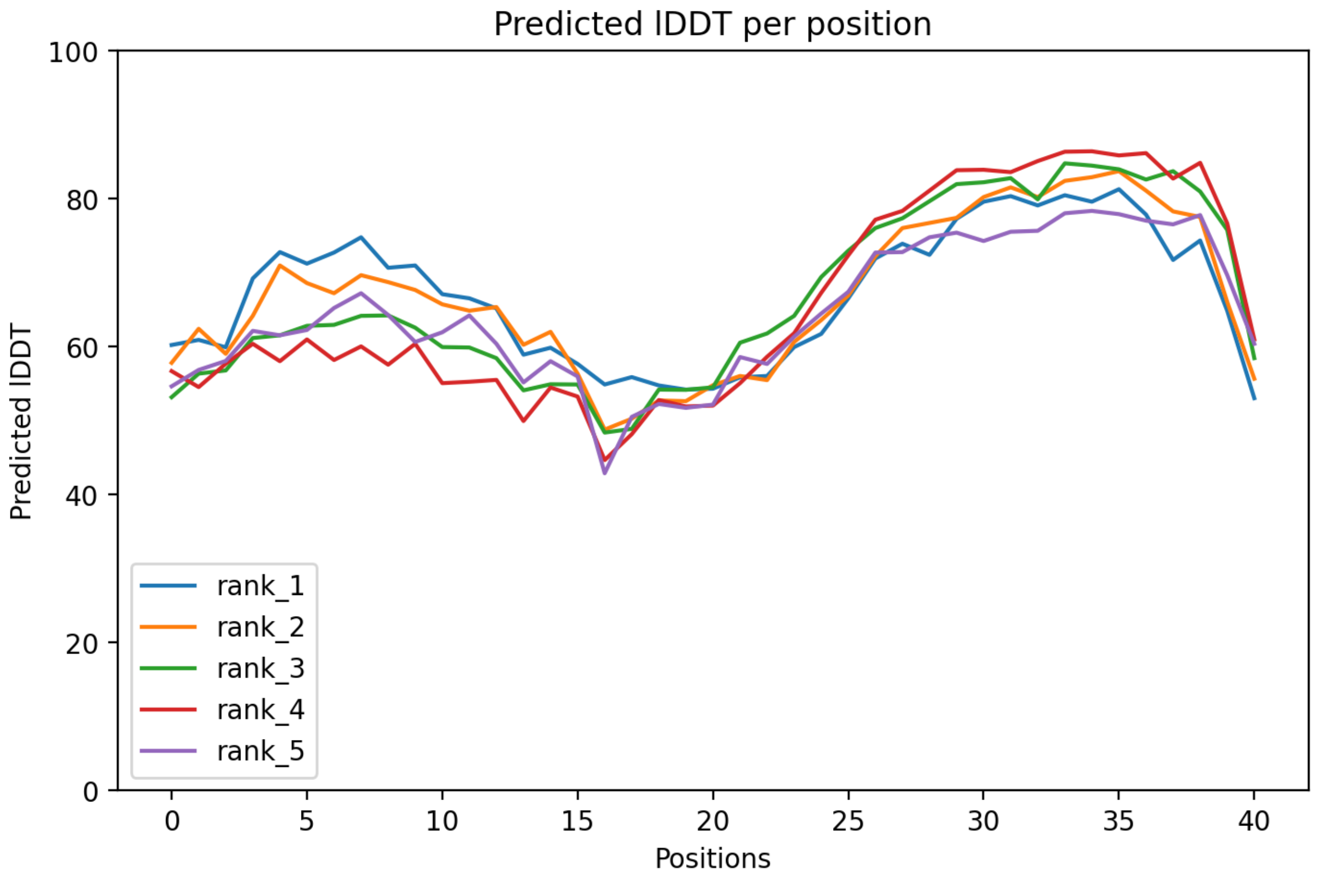

CP127: MtrunA17_Chr8g0345421

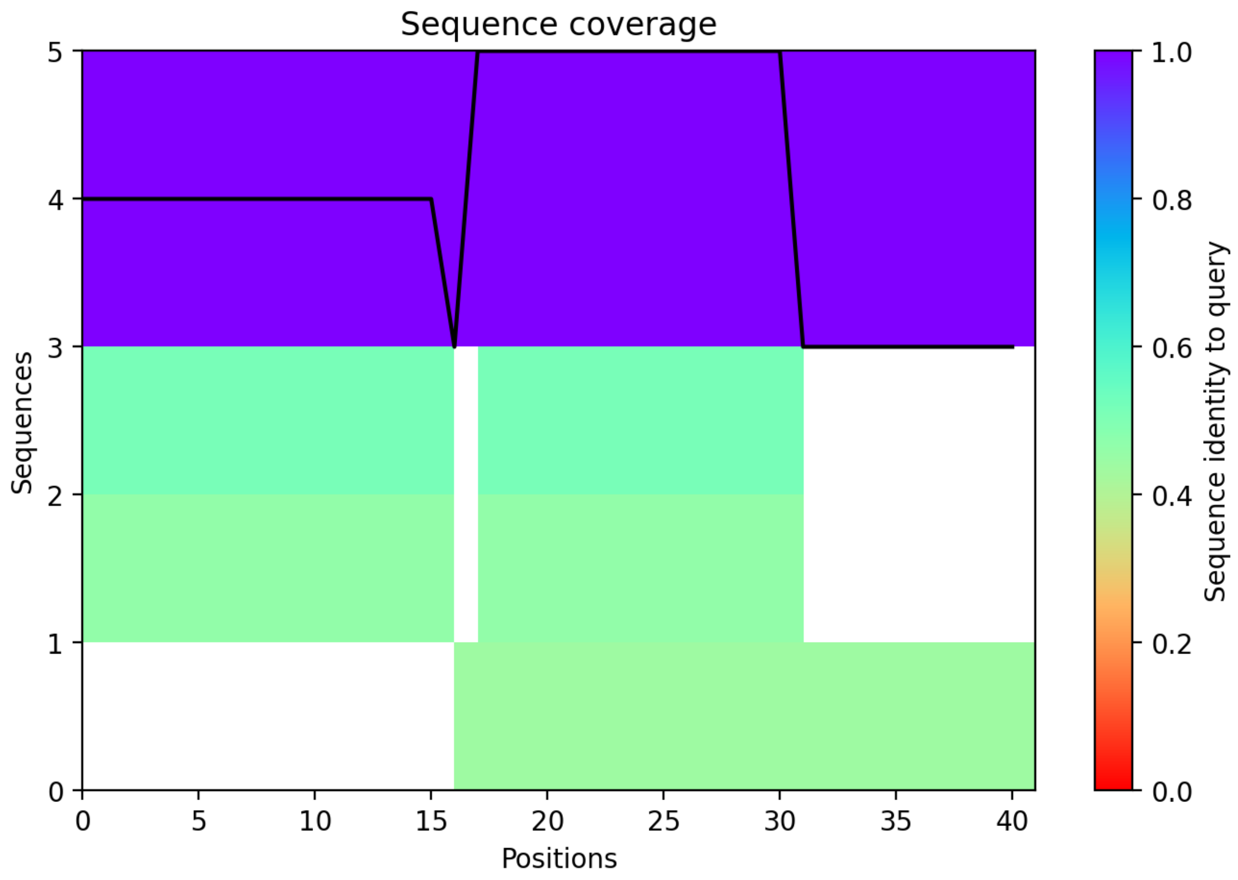

CP128: MtrunA17_Chr8g0353711

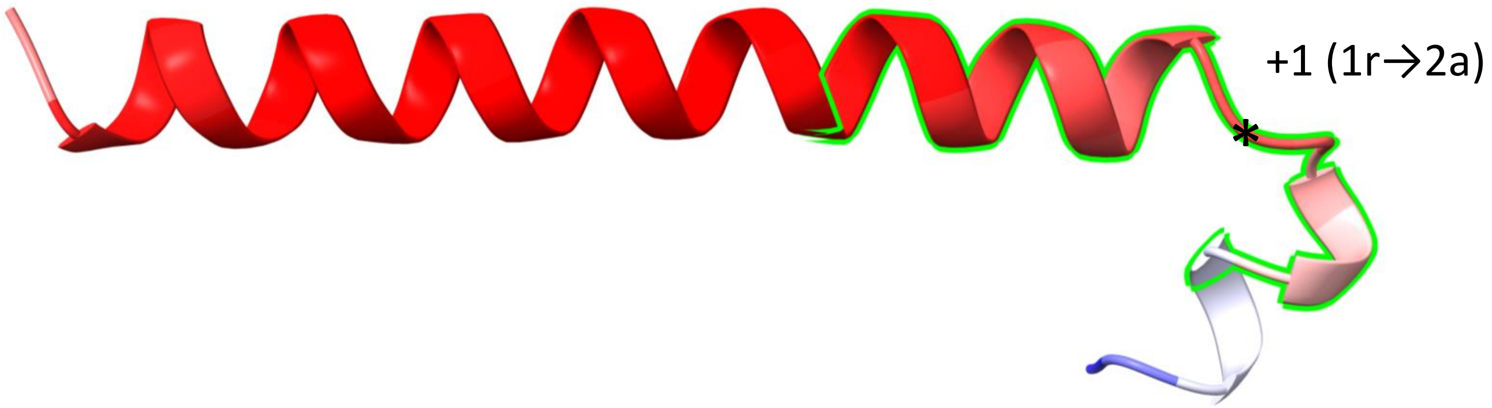

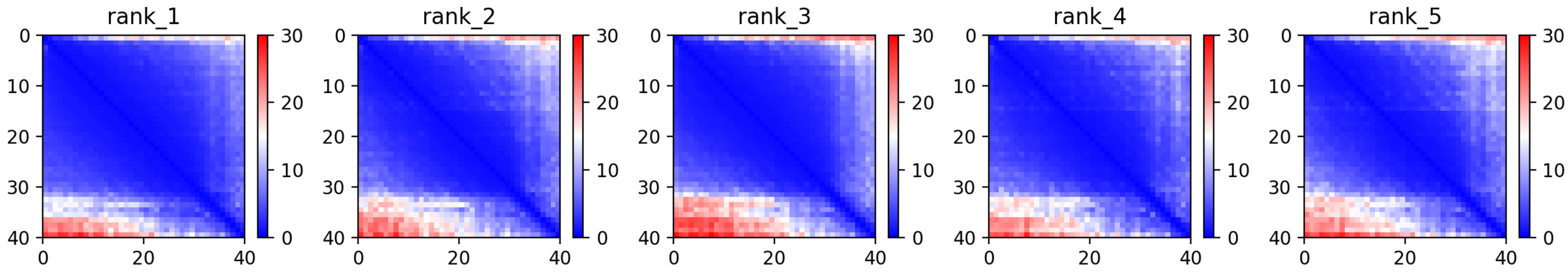

CP128: MtrunA17_Chr8g0353711

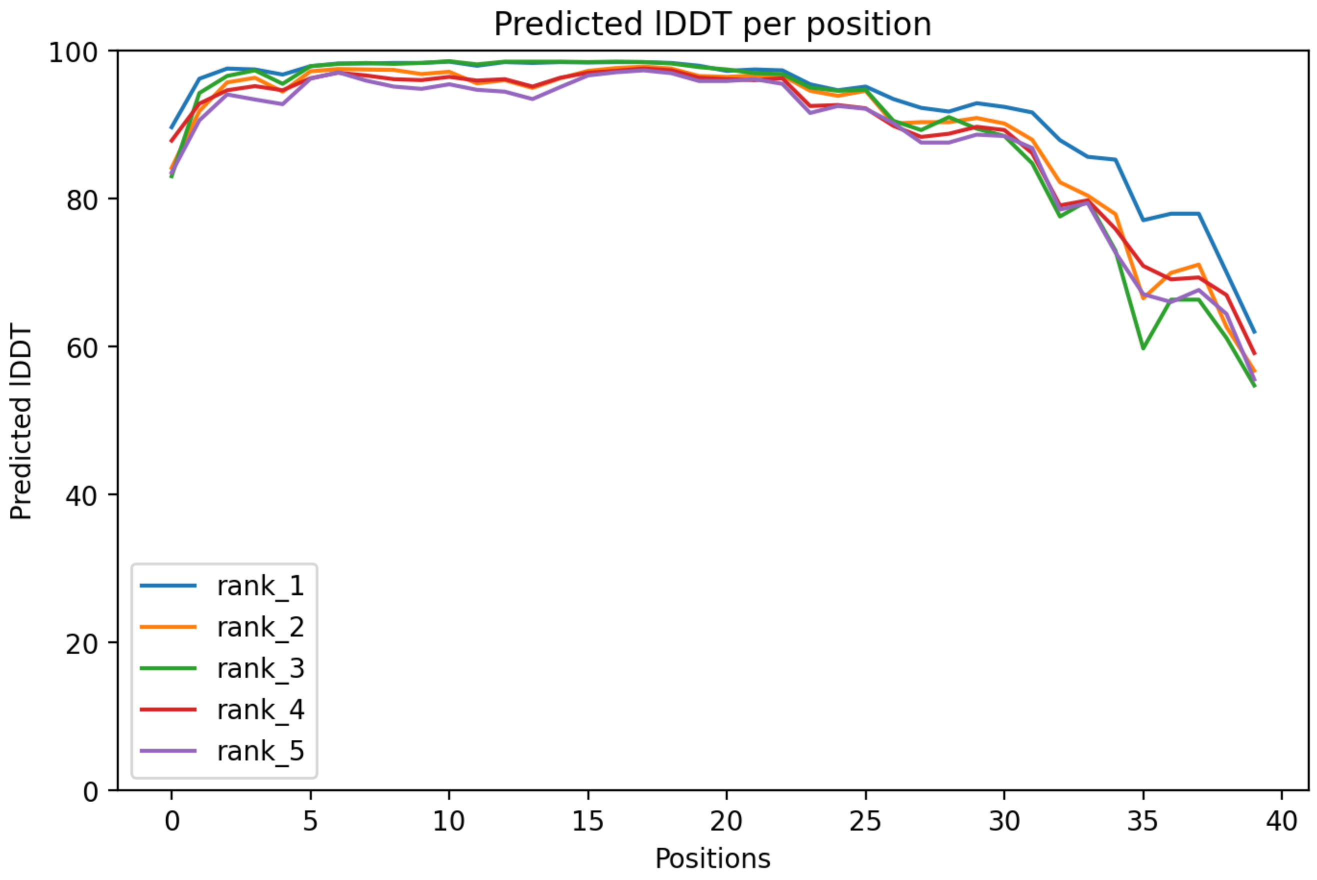

CP128: MtrunA17_Chr8g0353711

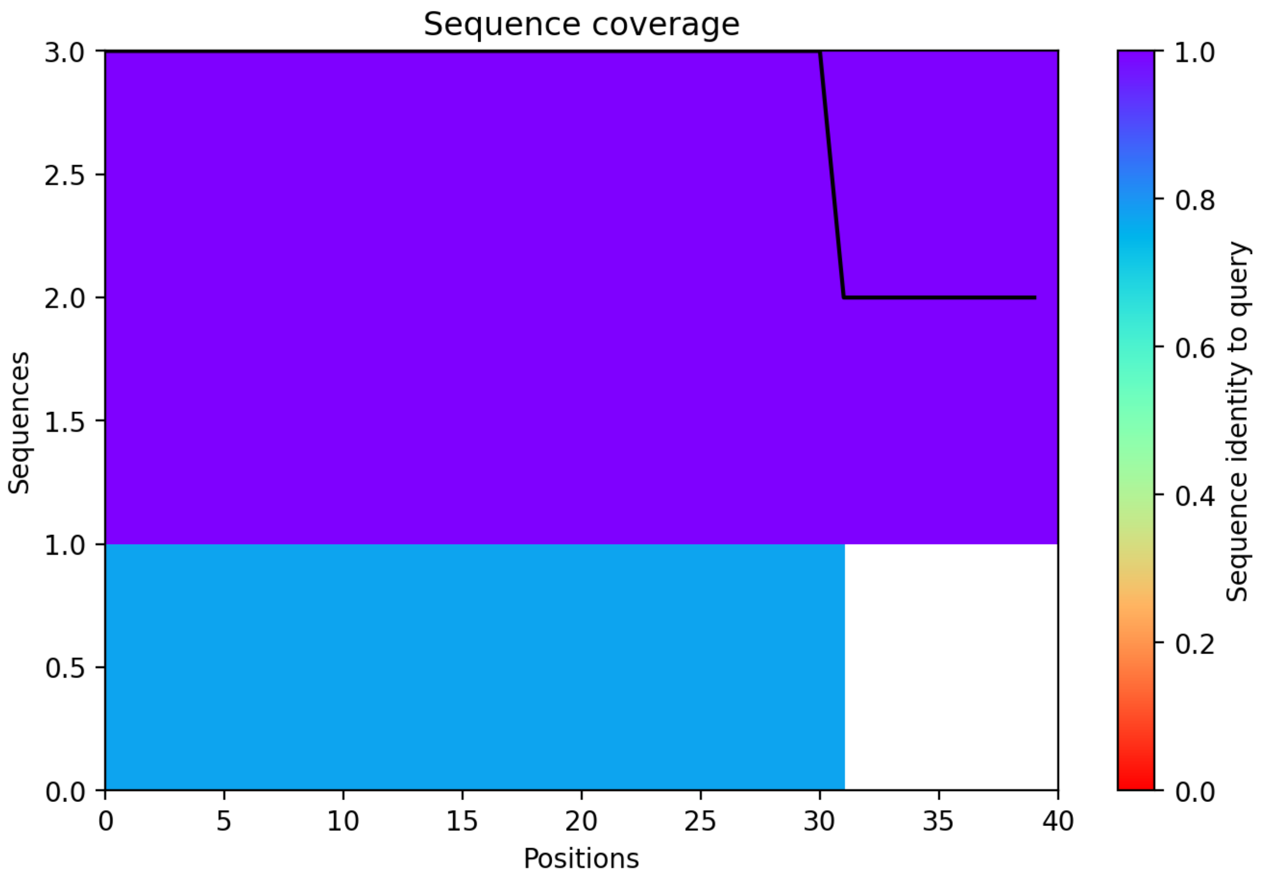

CP129: MtrunA17_Chr8g0355501

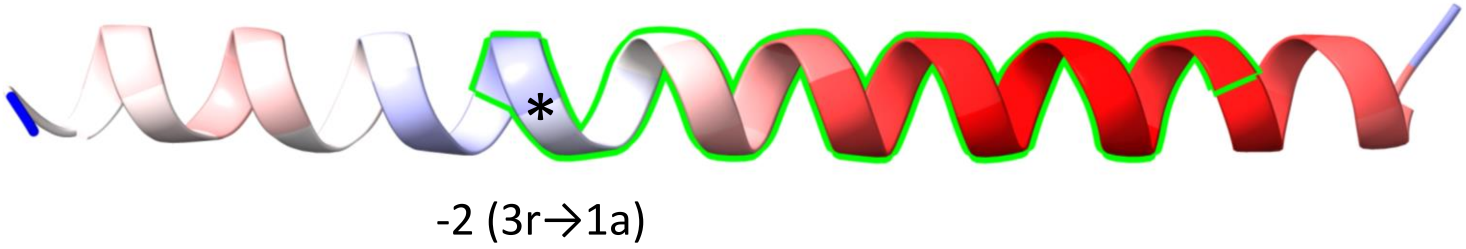

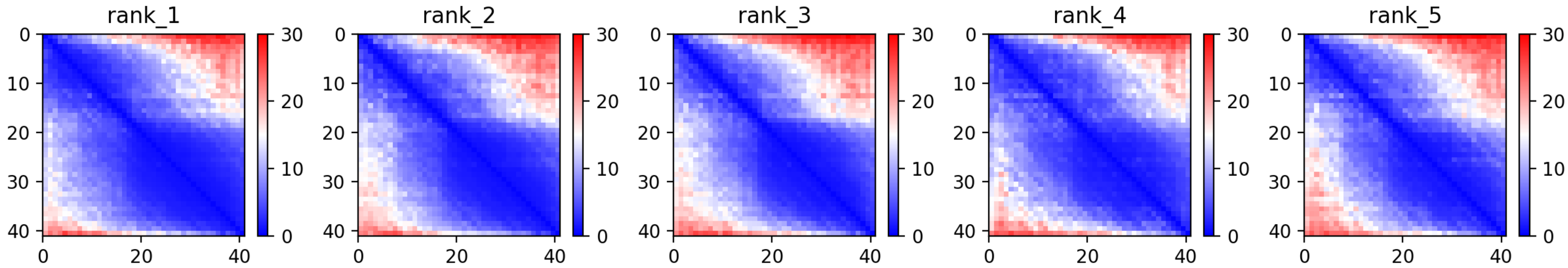

CP129: MtrunA17_Chr8g0355501

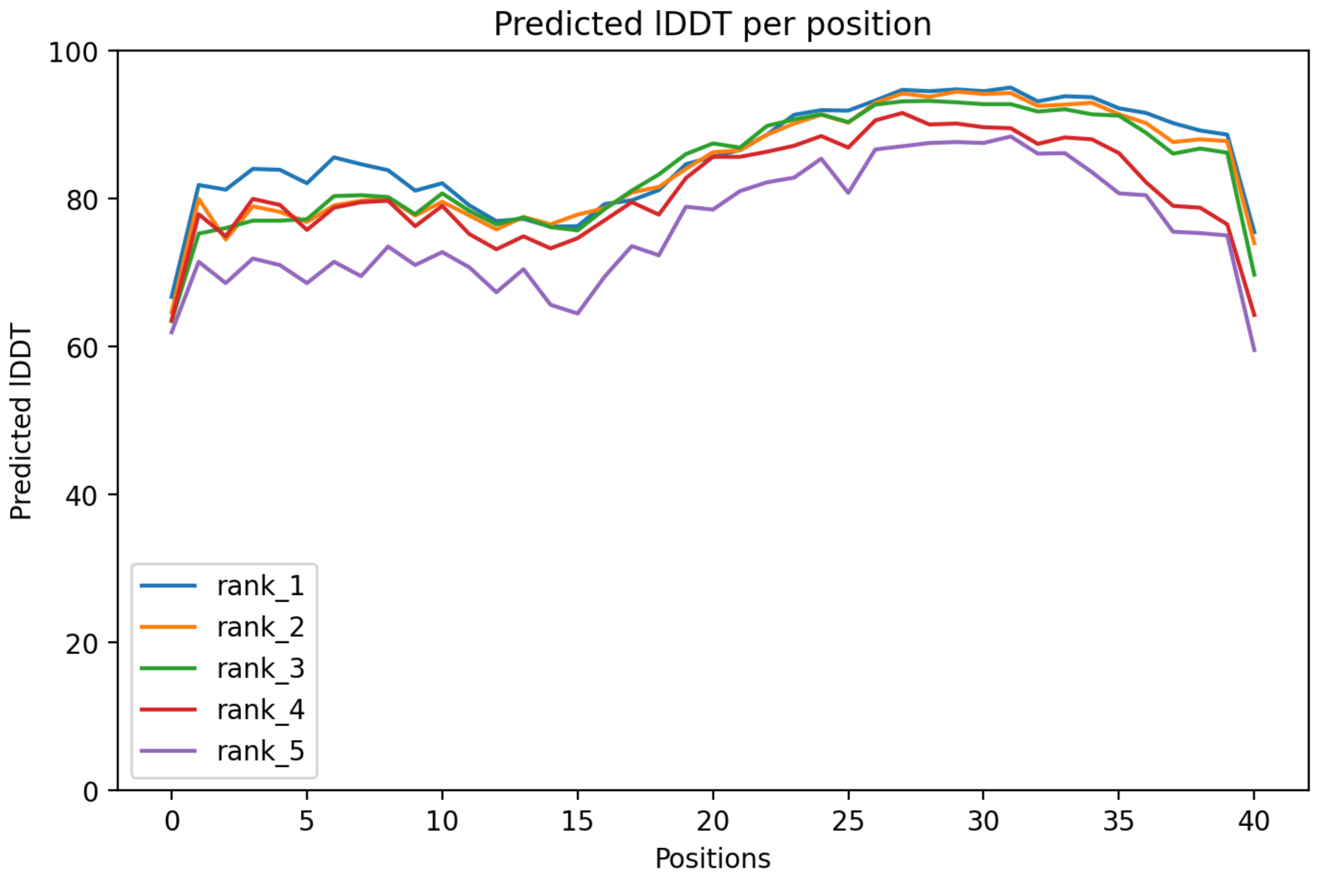

CP129: MtrunA17_Chr8g0355501

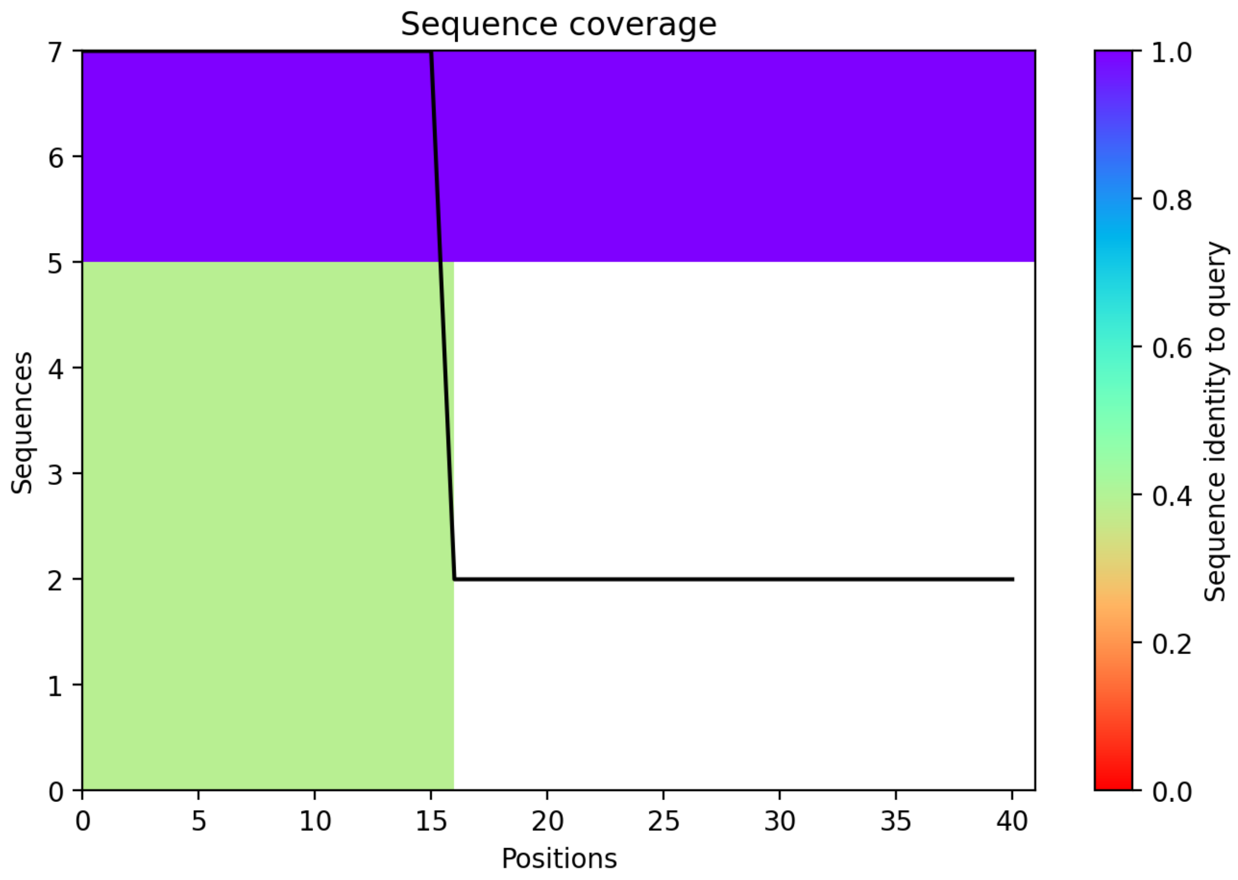

CP130: MtrunA17_Chr8g0356581

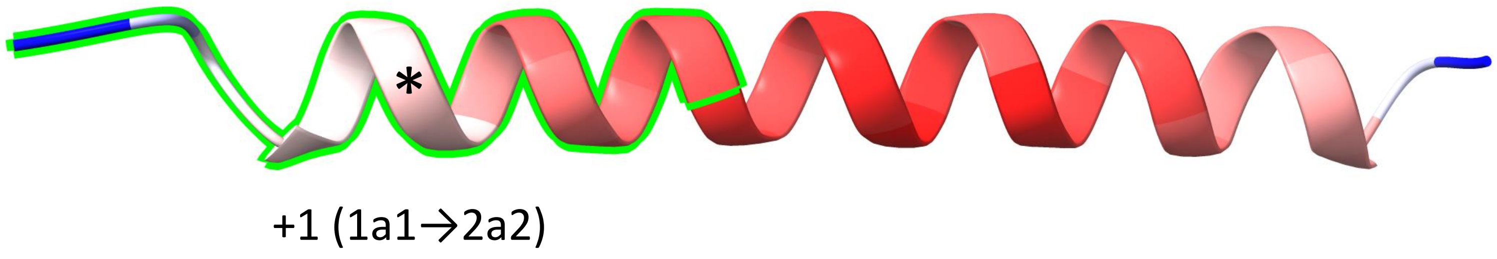

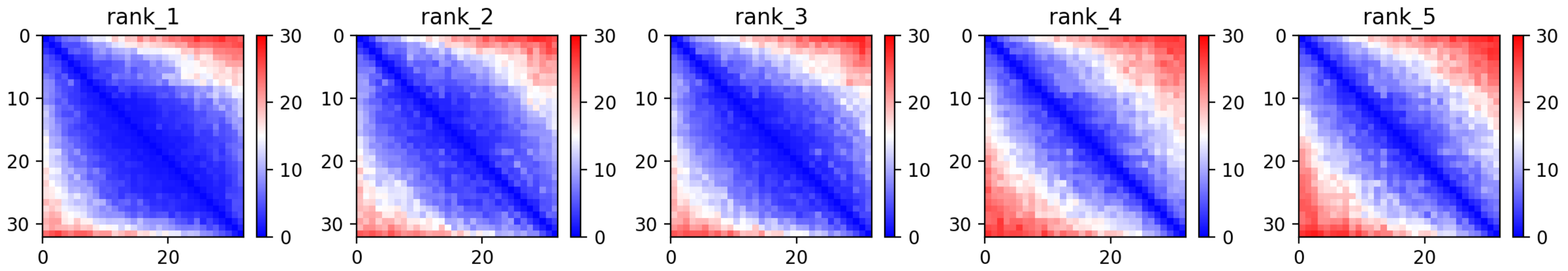

CP130: MtrunA17_Chr8g0356581

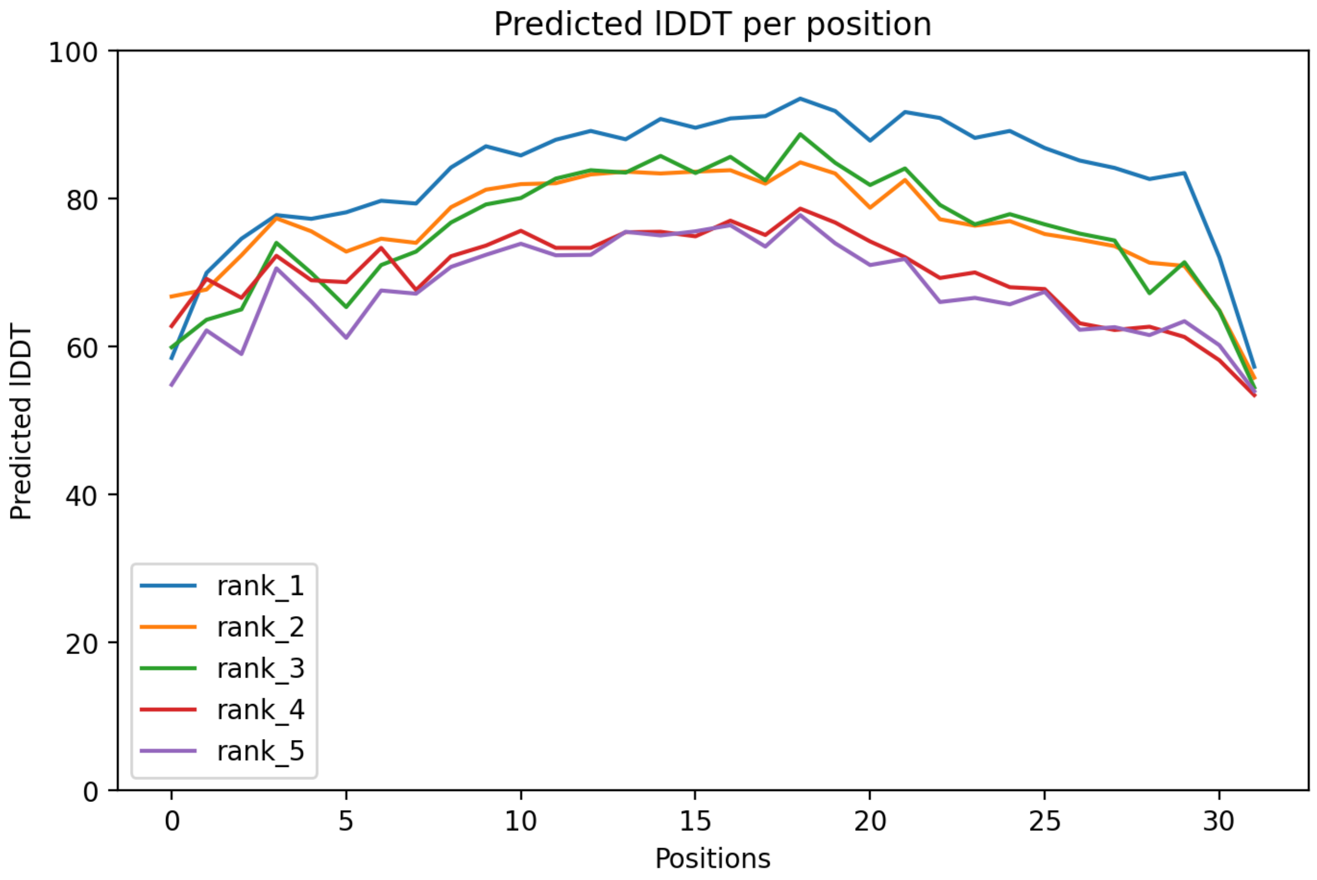

CP130: MtrunA17_Chr8g0356581

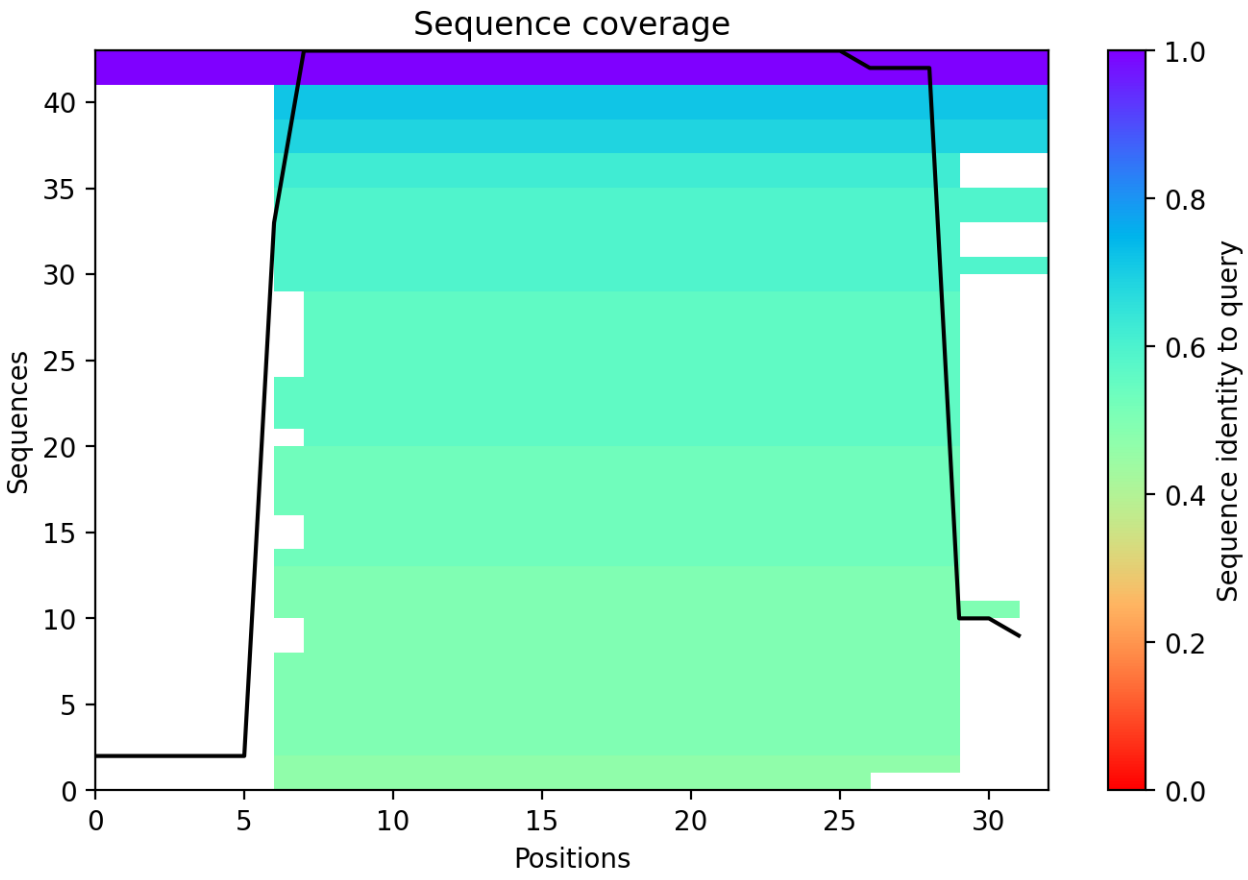

CP131: MtrunA17_Chr8g0365341

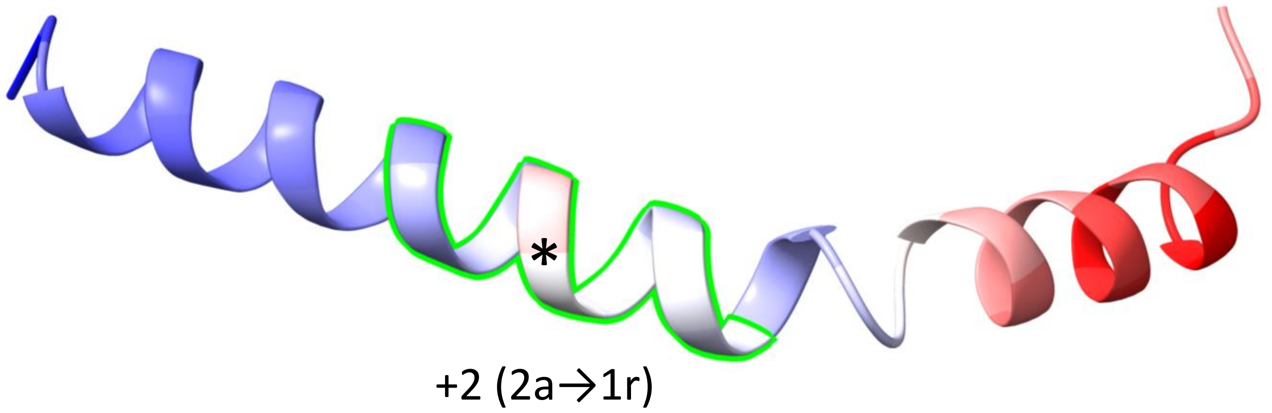

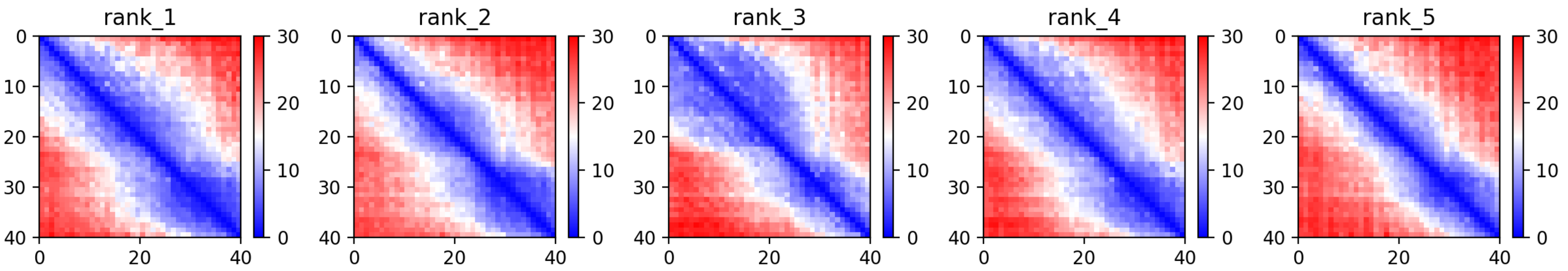

CP131: MtrunA17_Chr8g0365341

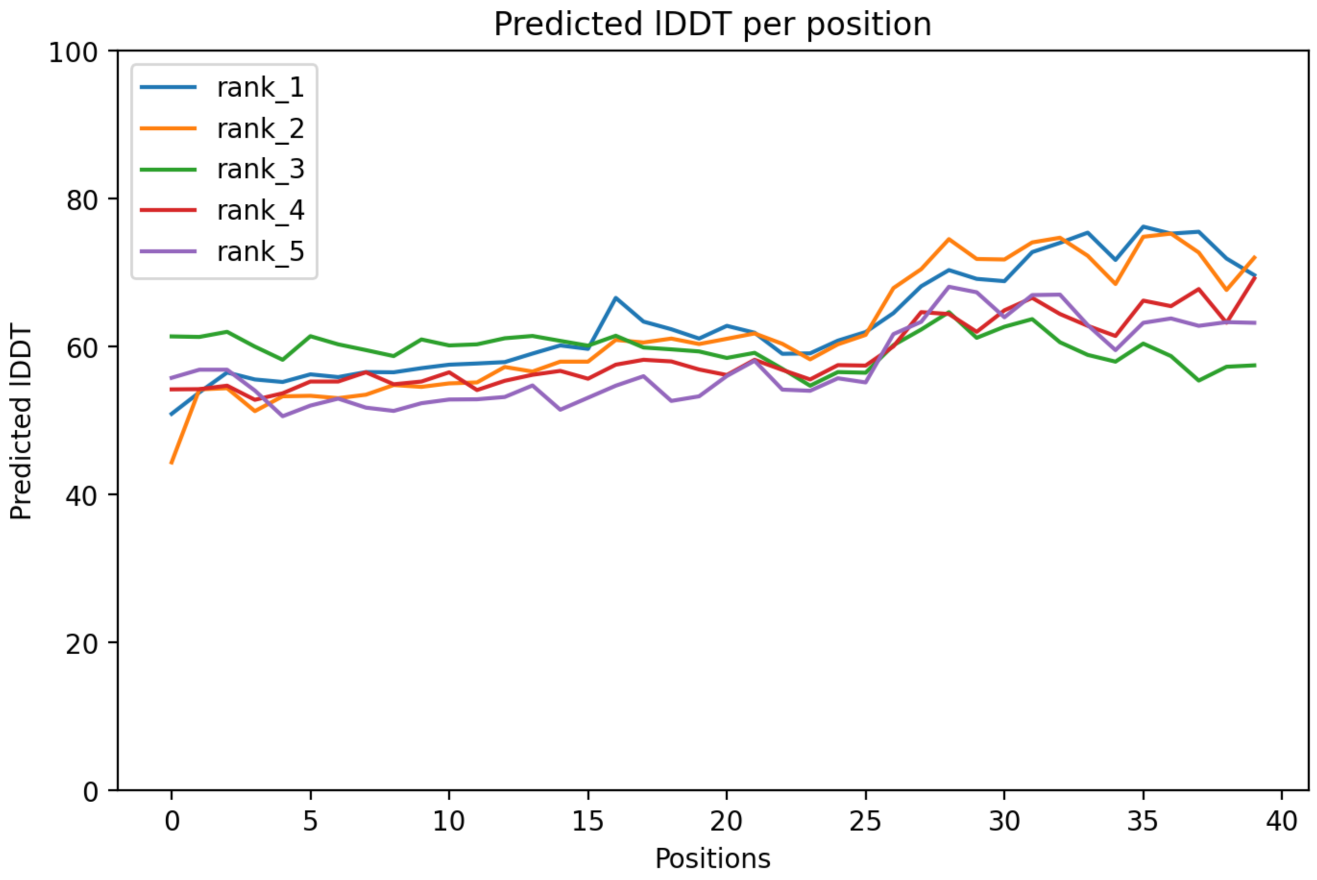

CP131: MtrunA17_Chr8g0365341

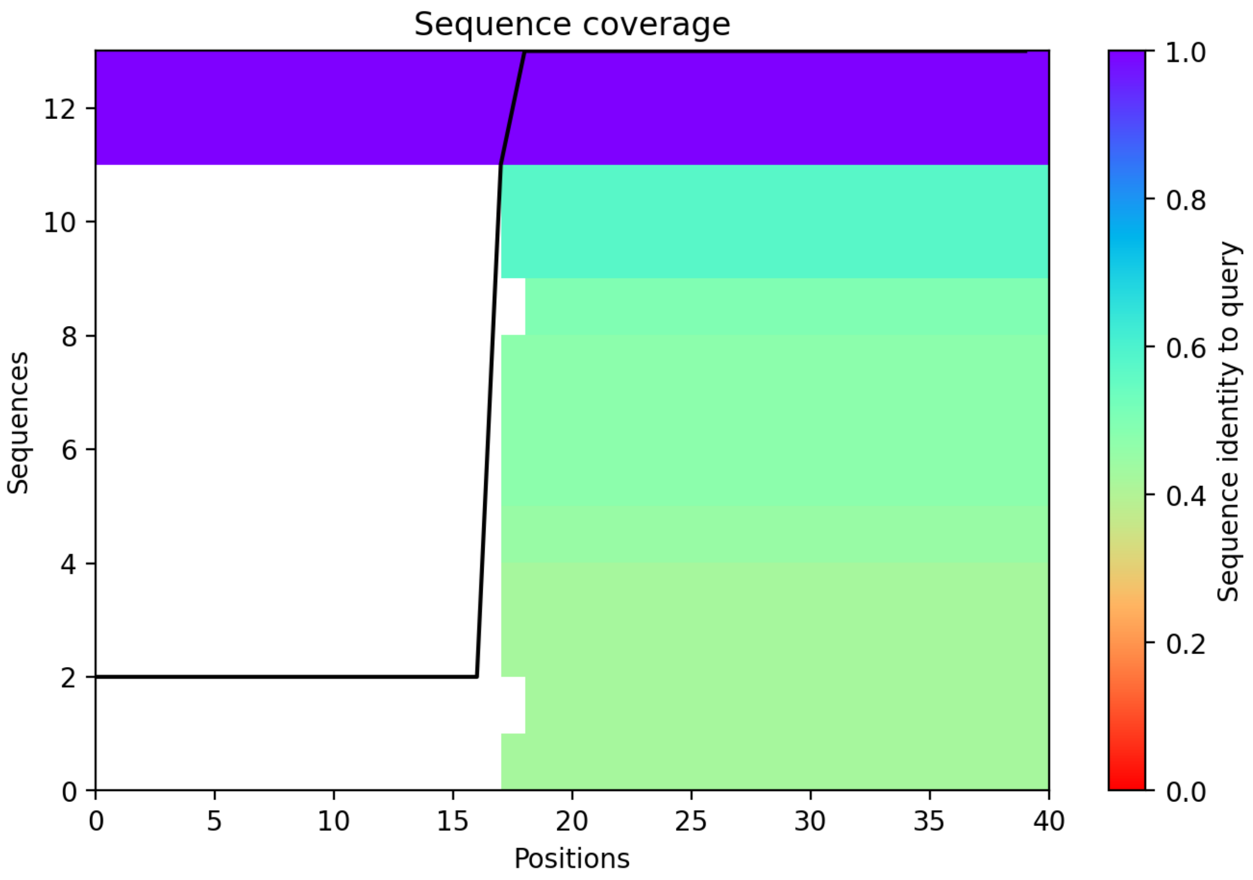

CP132: MtrunA17_Chr8g0368731

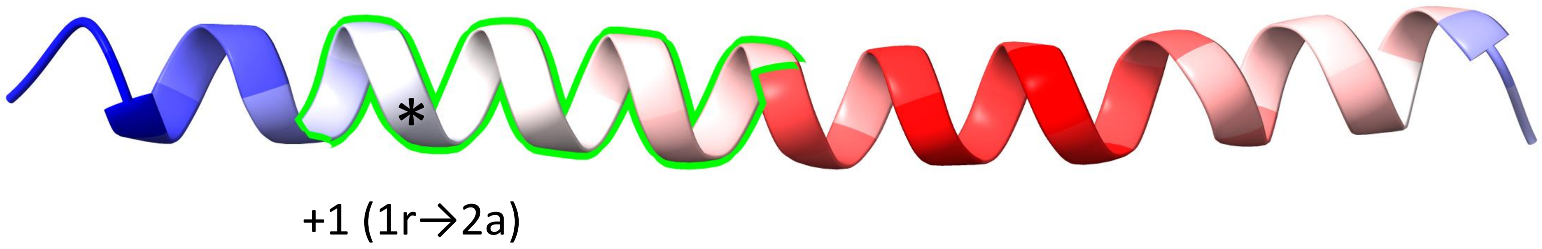

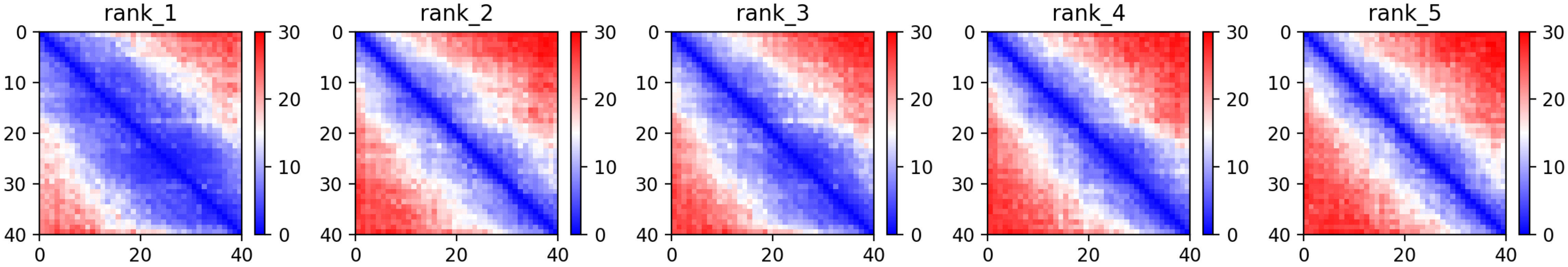

CP132: MtrunA17_Chr8g0368731

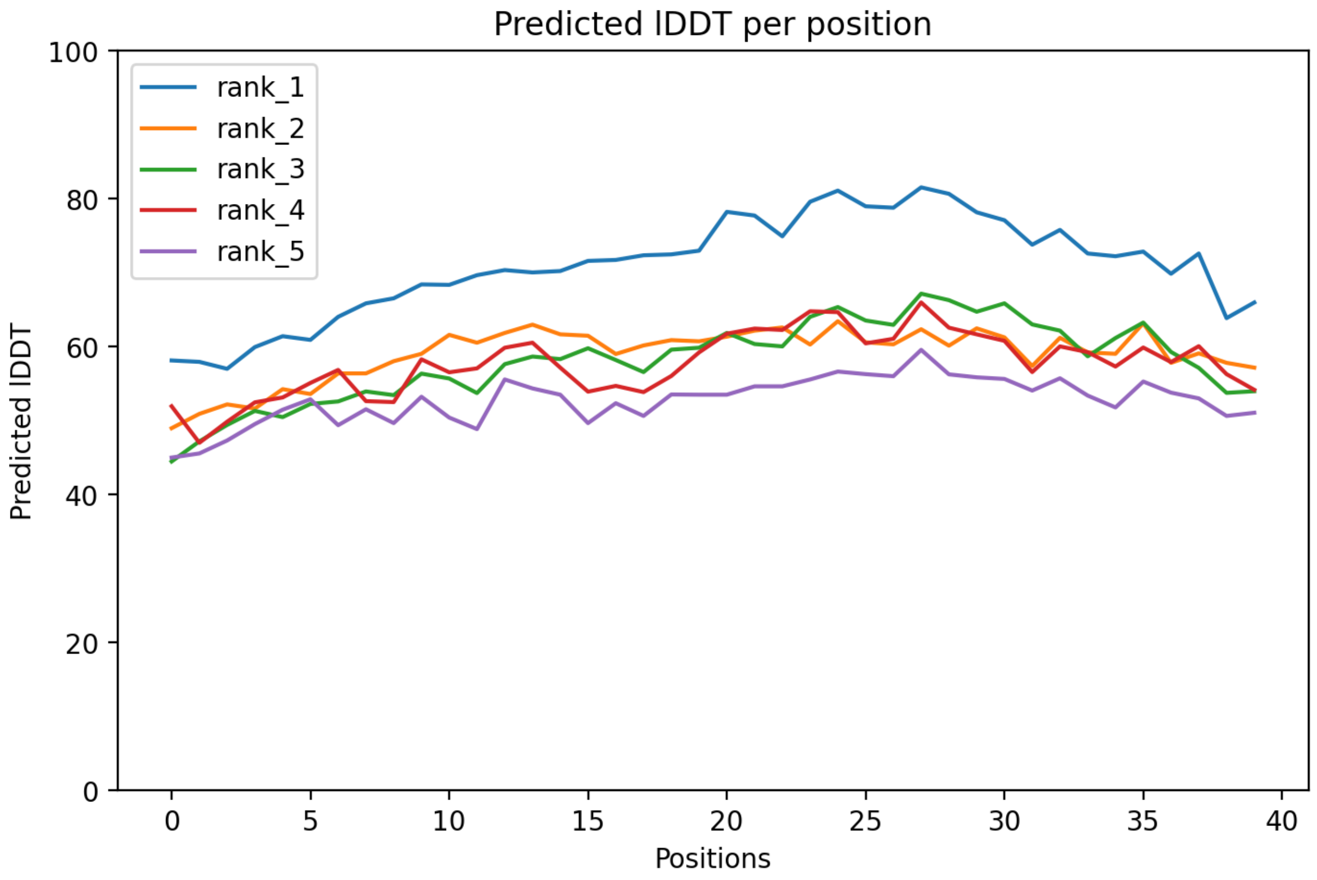

CP132: MtrunA17_Chr8g0368731

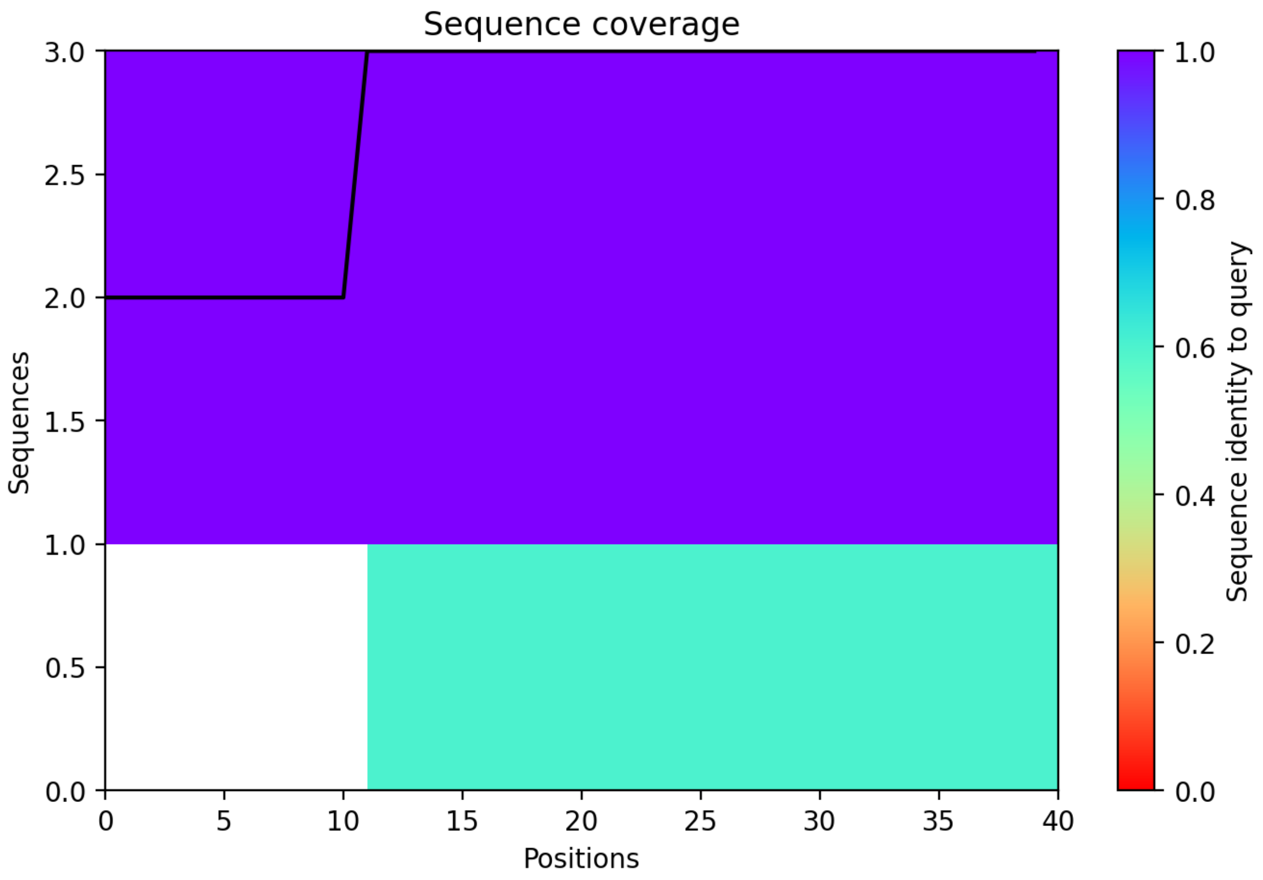

CP133: MtrunA17_Chr8g0368931

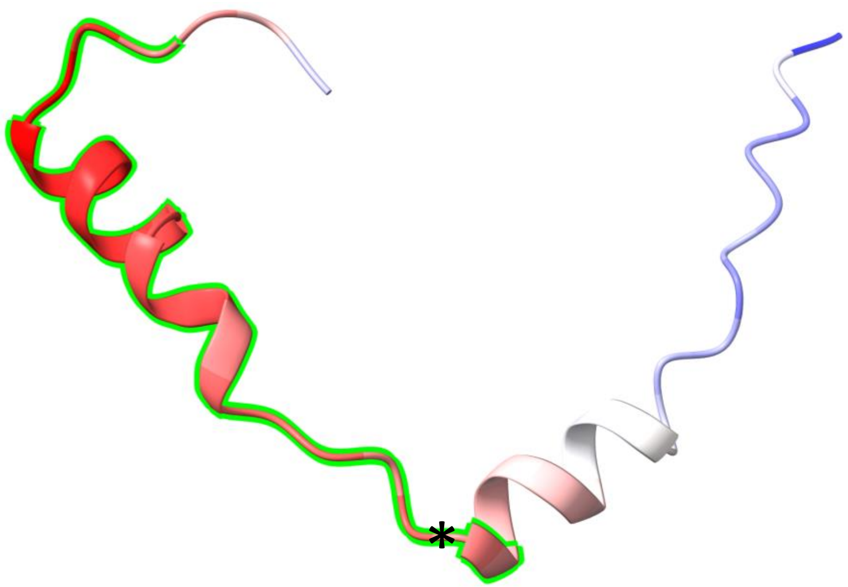

-2 (3r→1a)

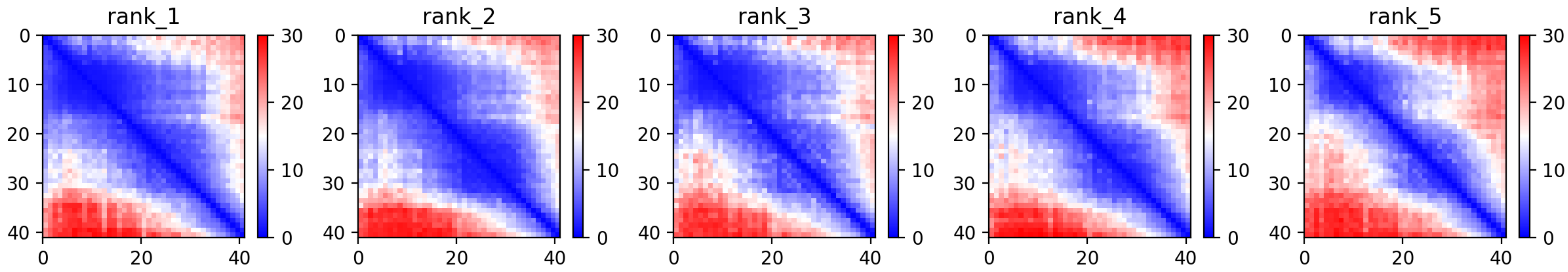

CP133: MtrunA17_Chr8g0368931

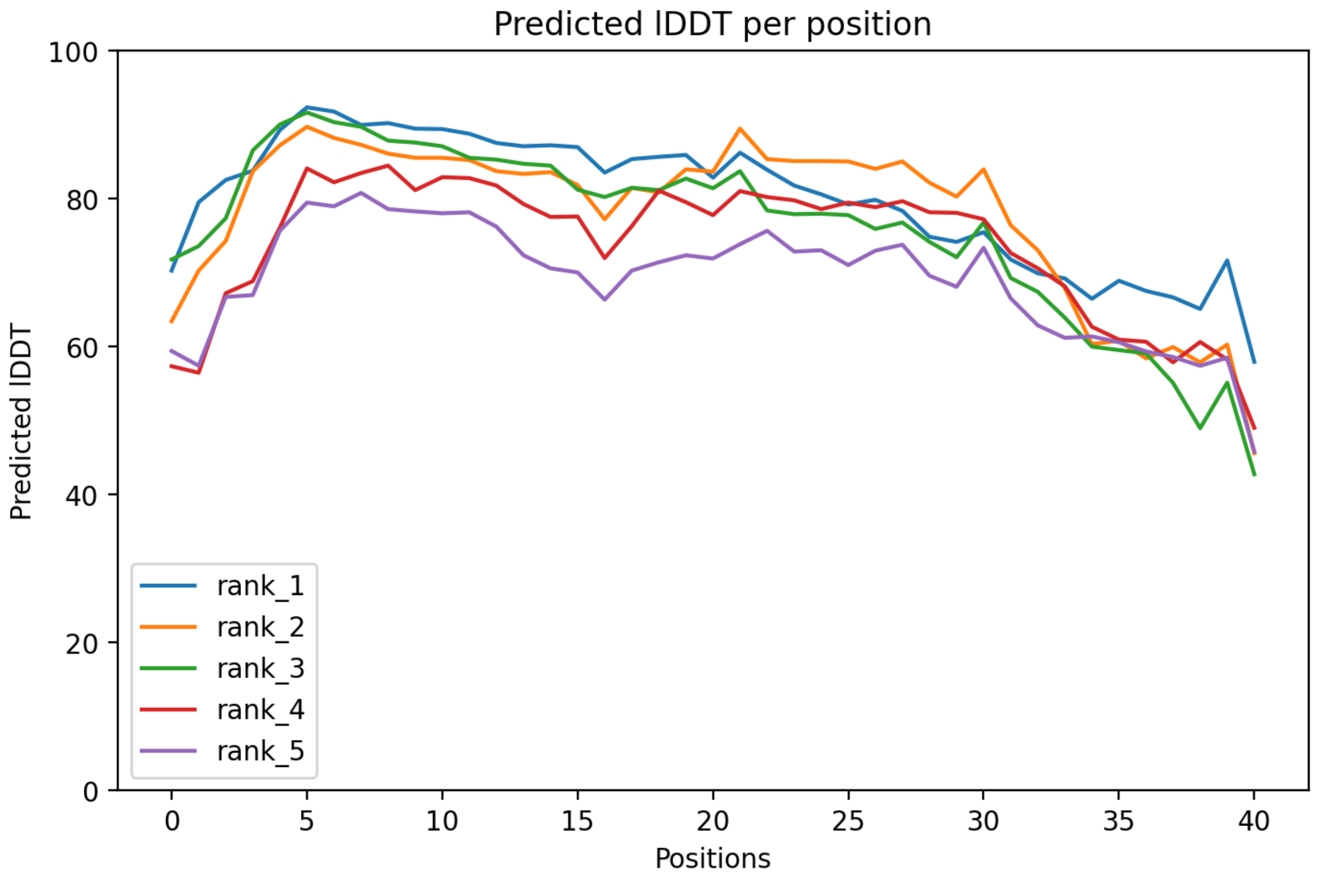

CP133: MtrunA17_Chr8g0368931

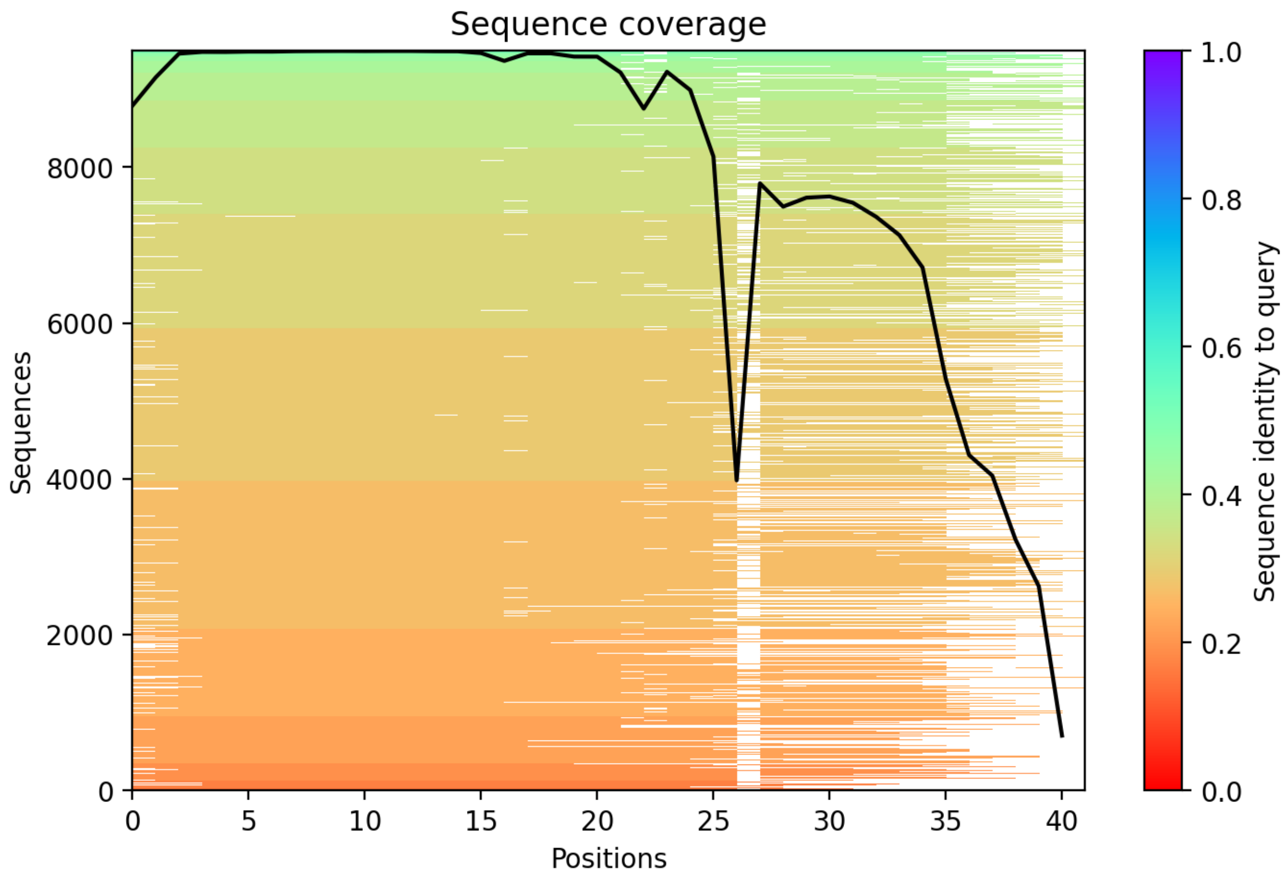

CP134: MtrunA17_Chr8g0371281

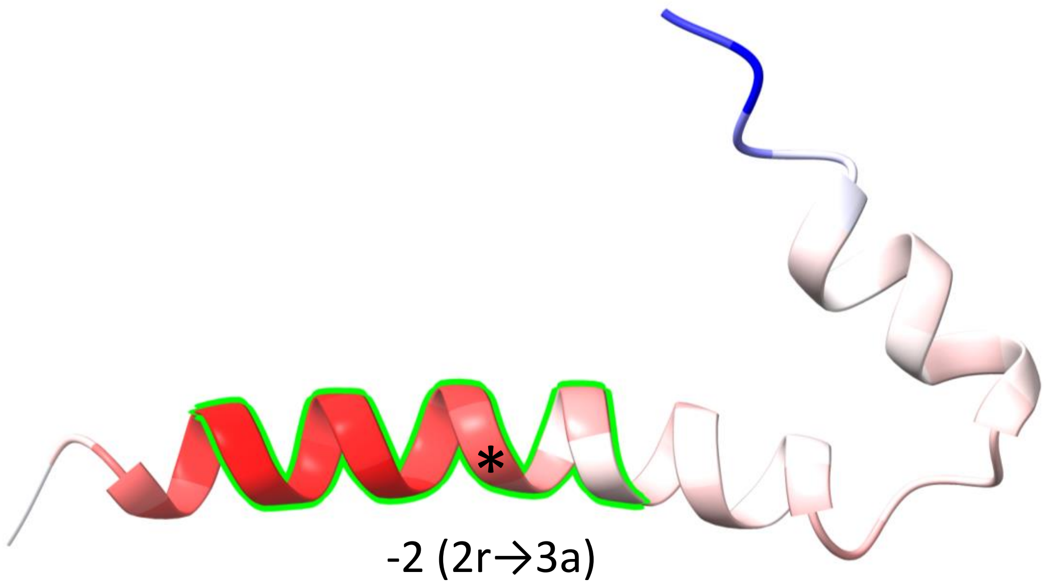

-2 (2r→3a)

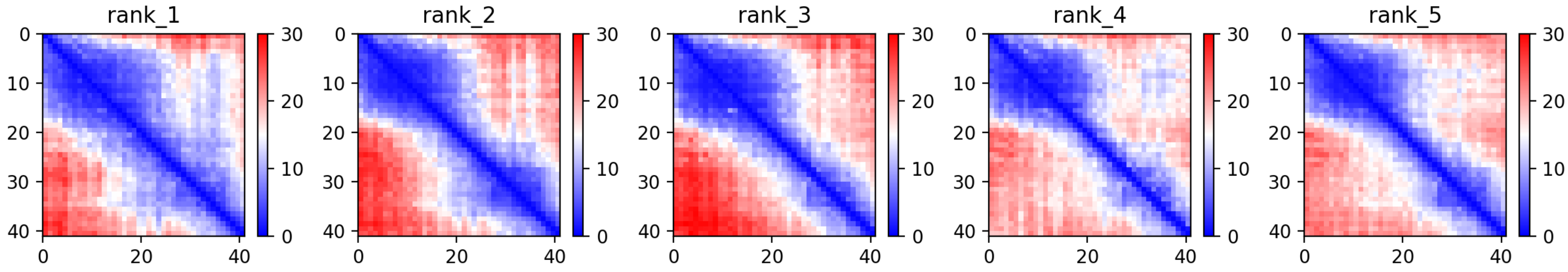

CP134: MtrunA17_Chr8g0371281

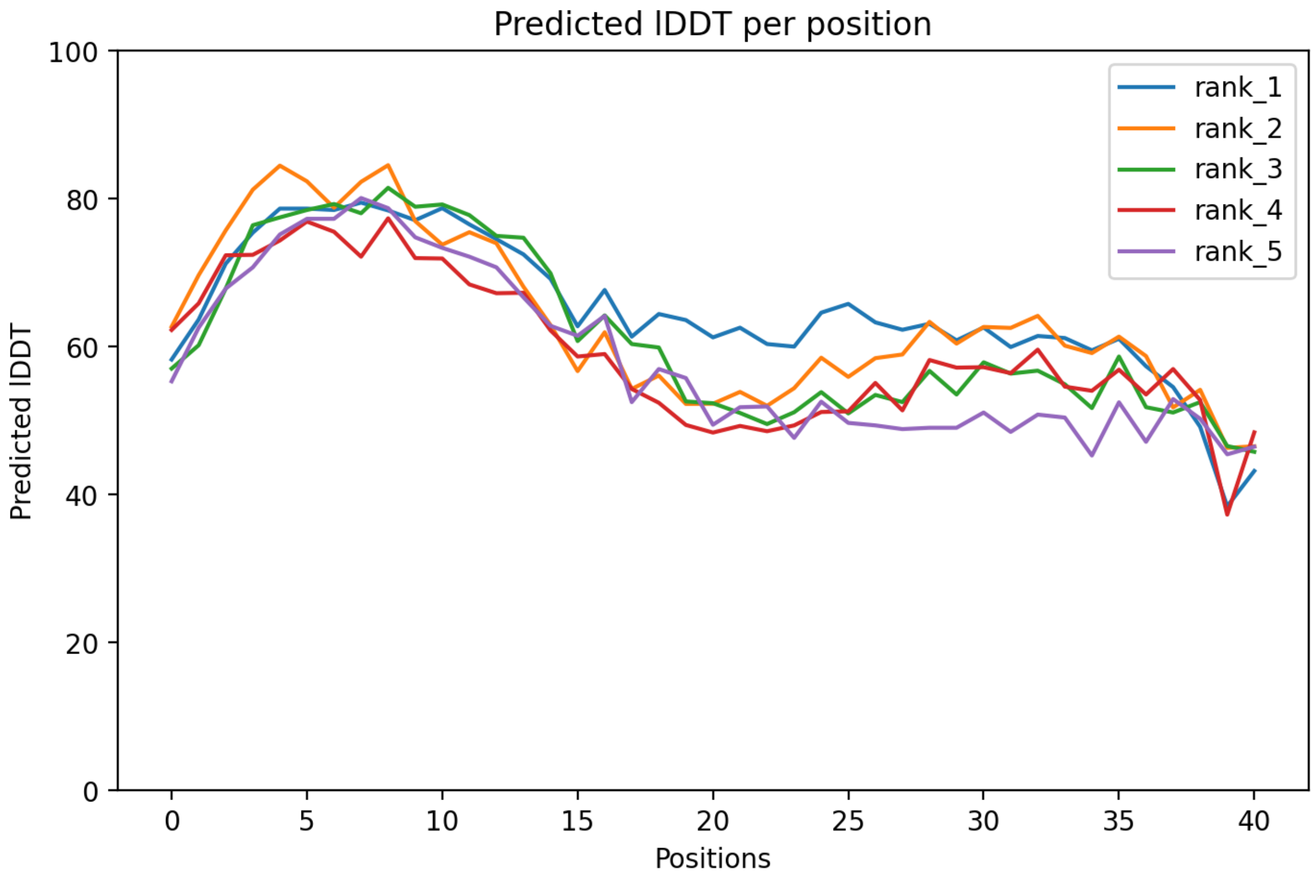

CP134: MtrunA17_Chr8g0371281

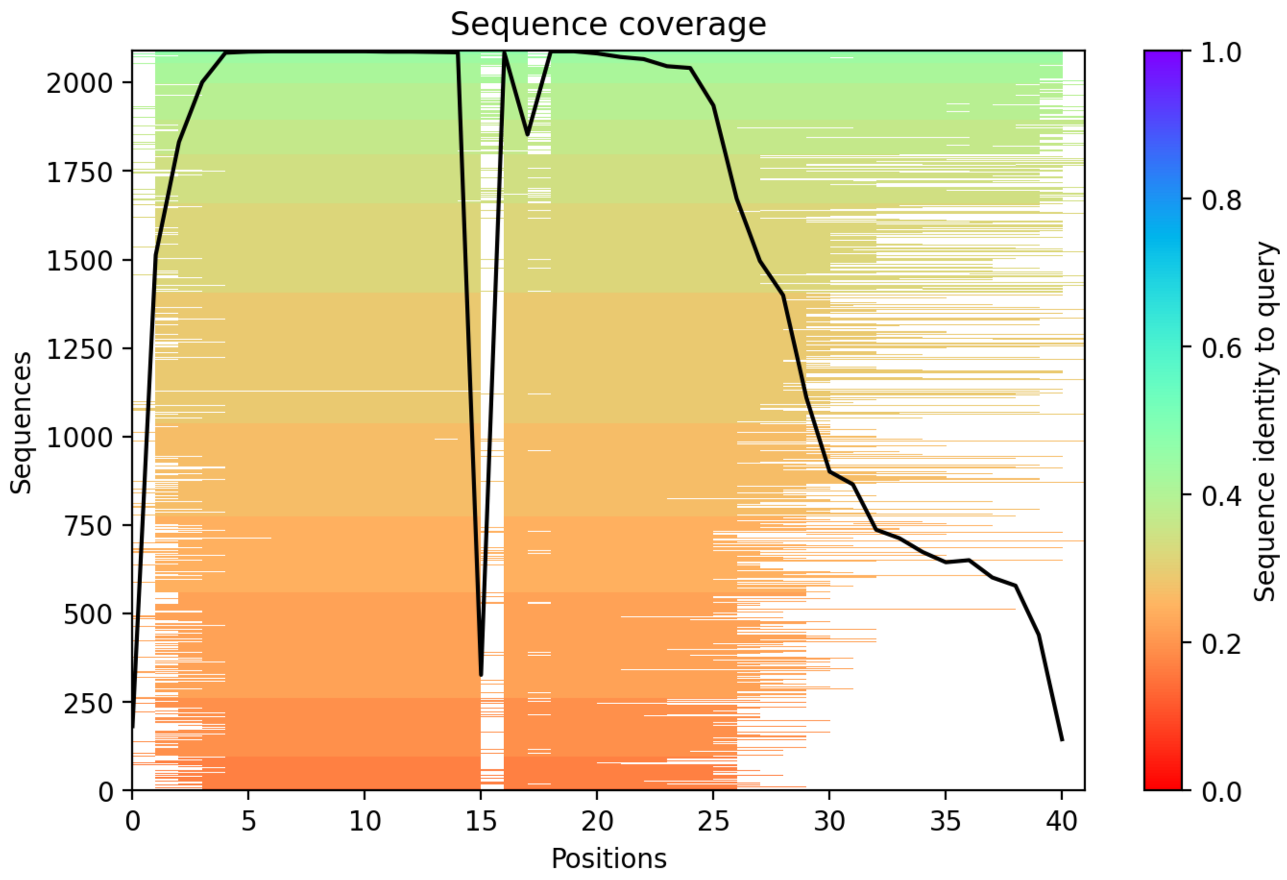

CP135: MtrunA17_Chr8g0371741

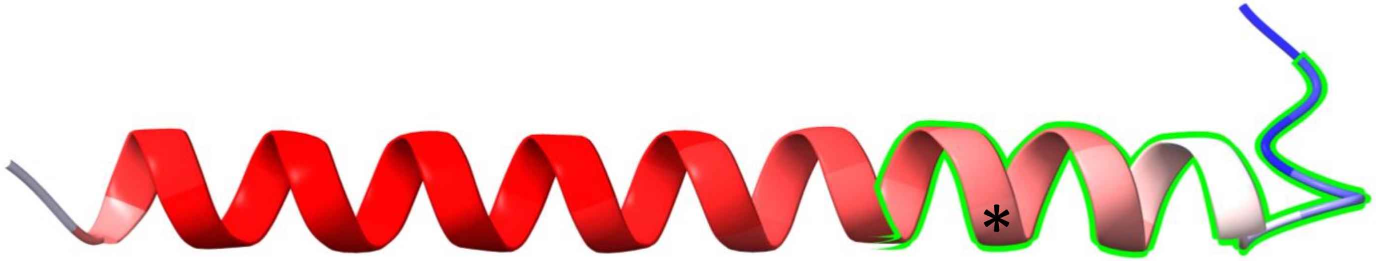

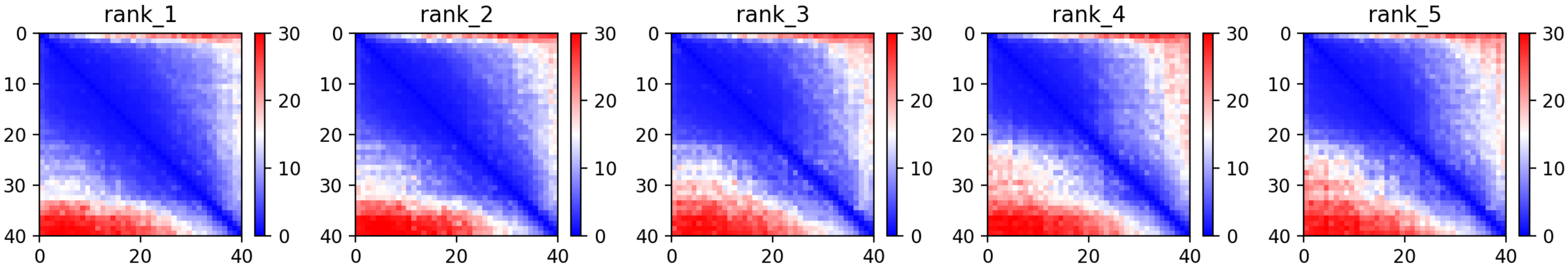

CP135: MtrunA17_Chr8g0371741

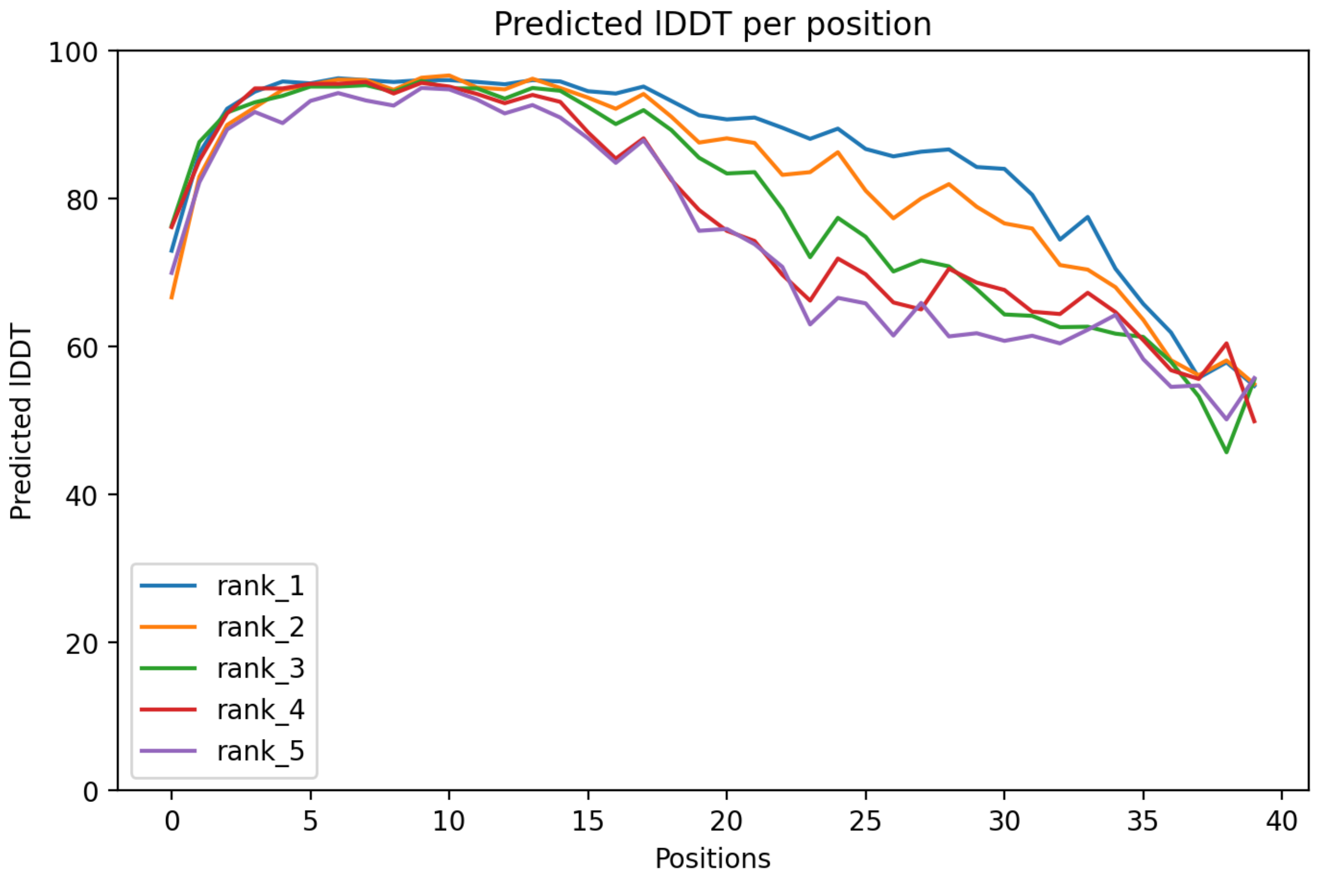

CP135: MtrunA17_Chr8g0371741

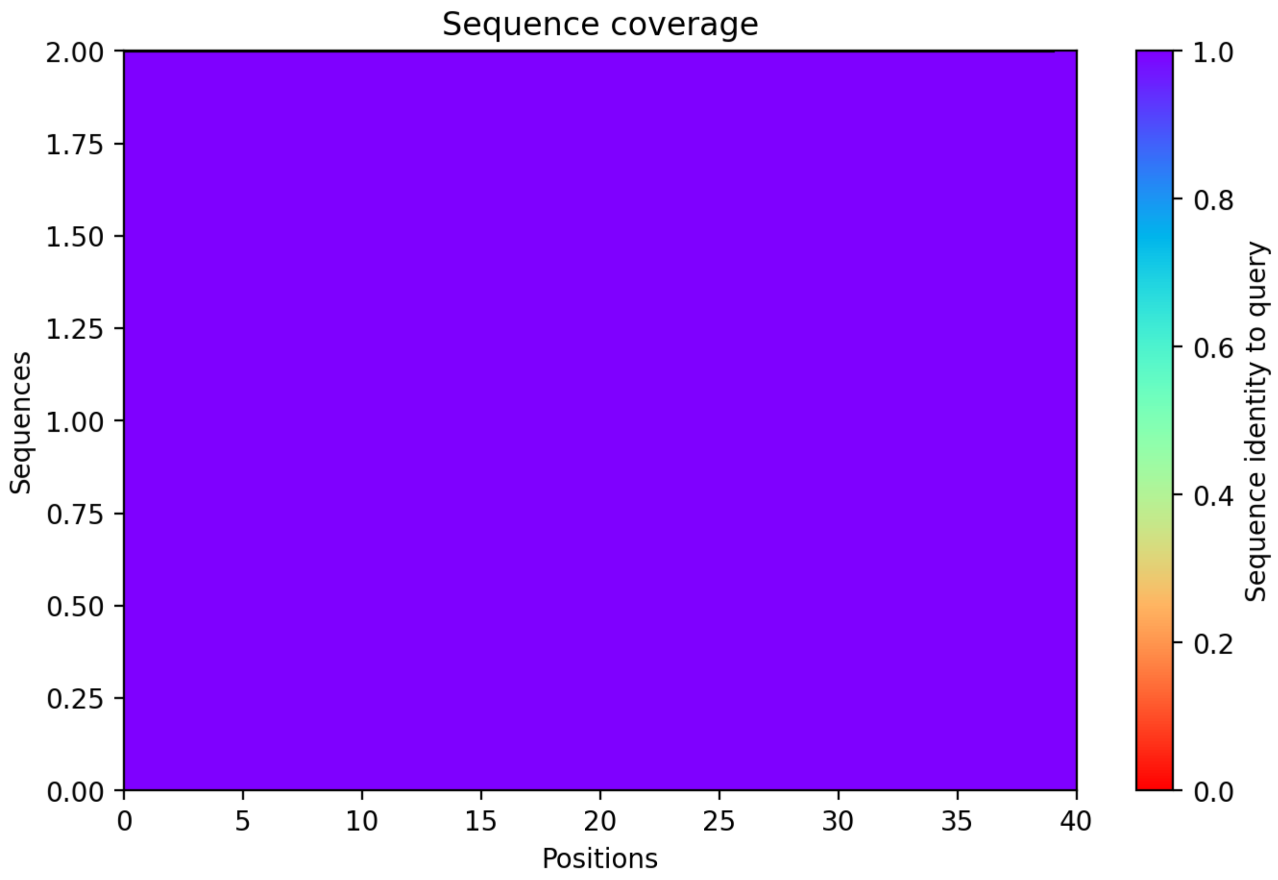

CP136: MtrunA17_Chr8g0373091

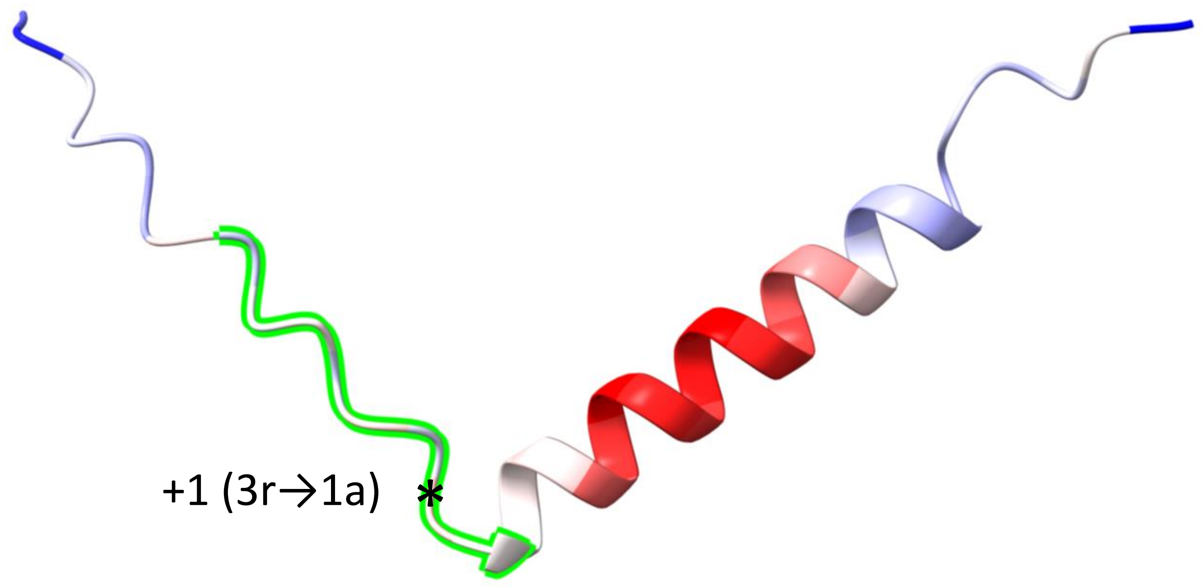

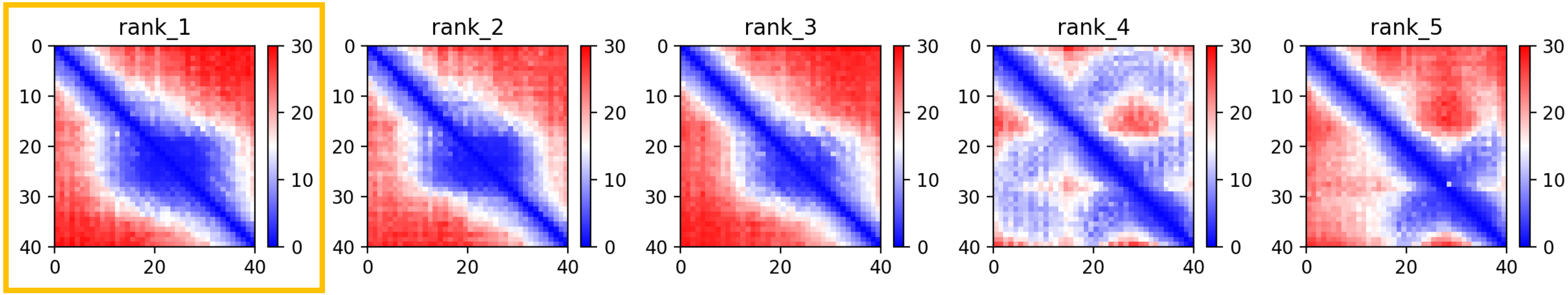

CP136: MtrunA17_Chr8g0373091

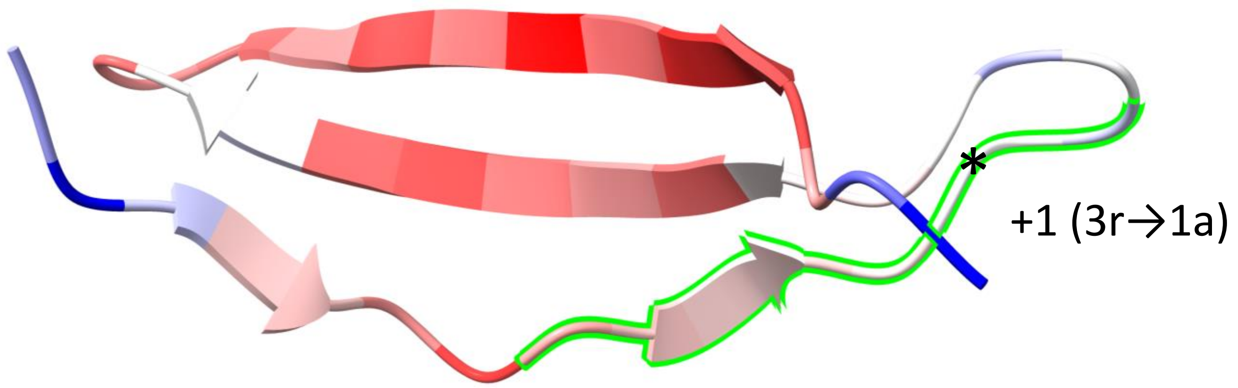

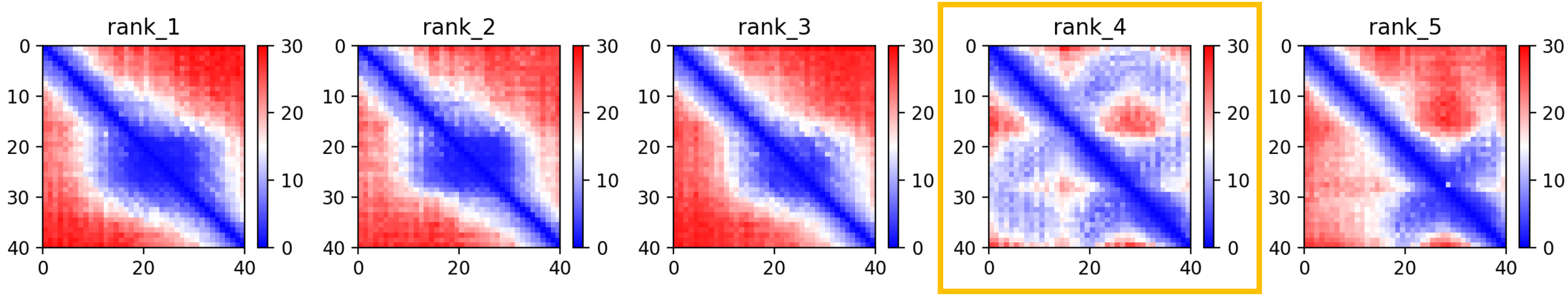

CP136: MtrunA17_Chr8g0373091

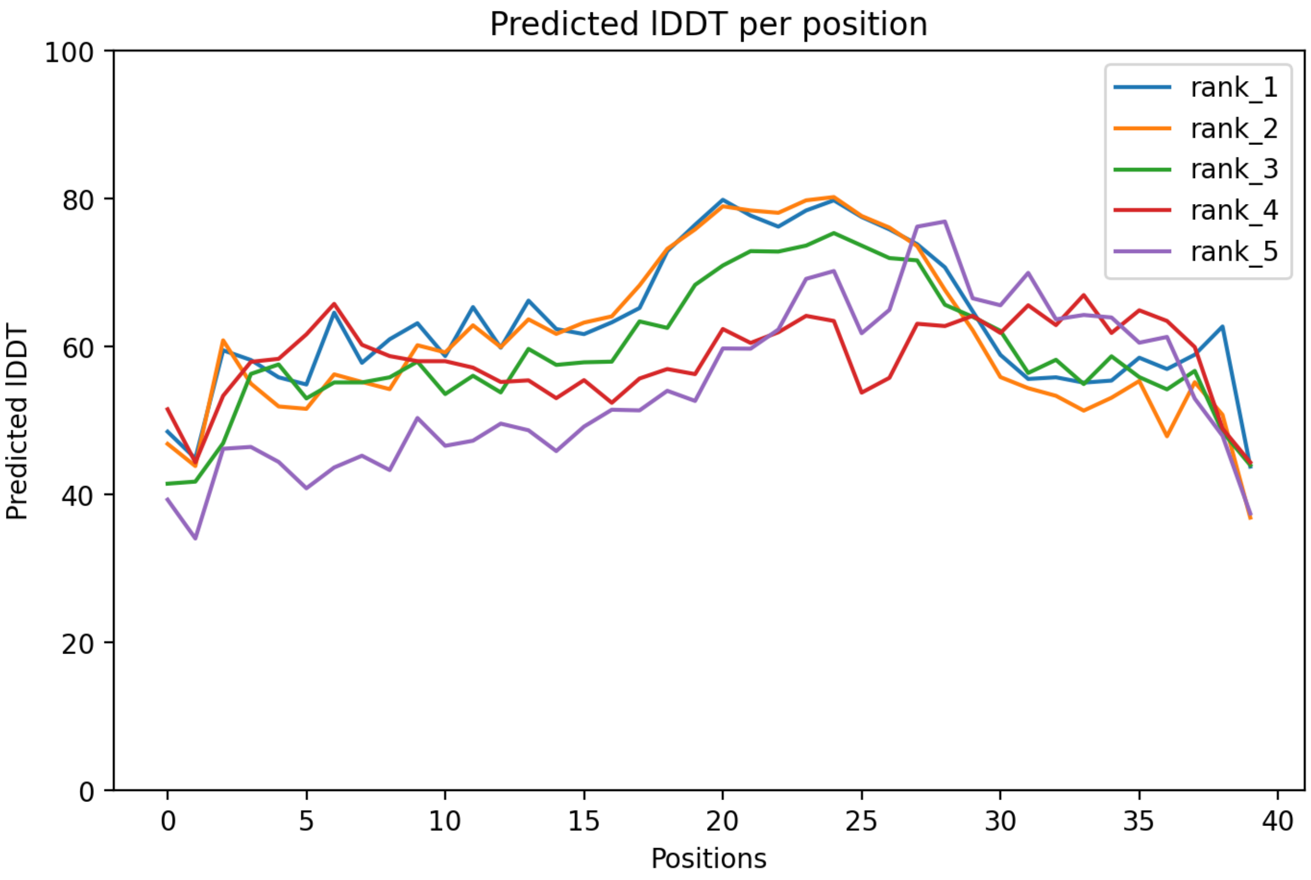

CP136: MtrunA17_Chr8g0373091

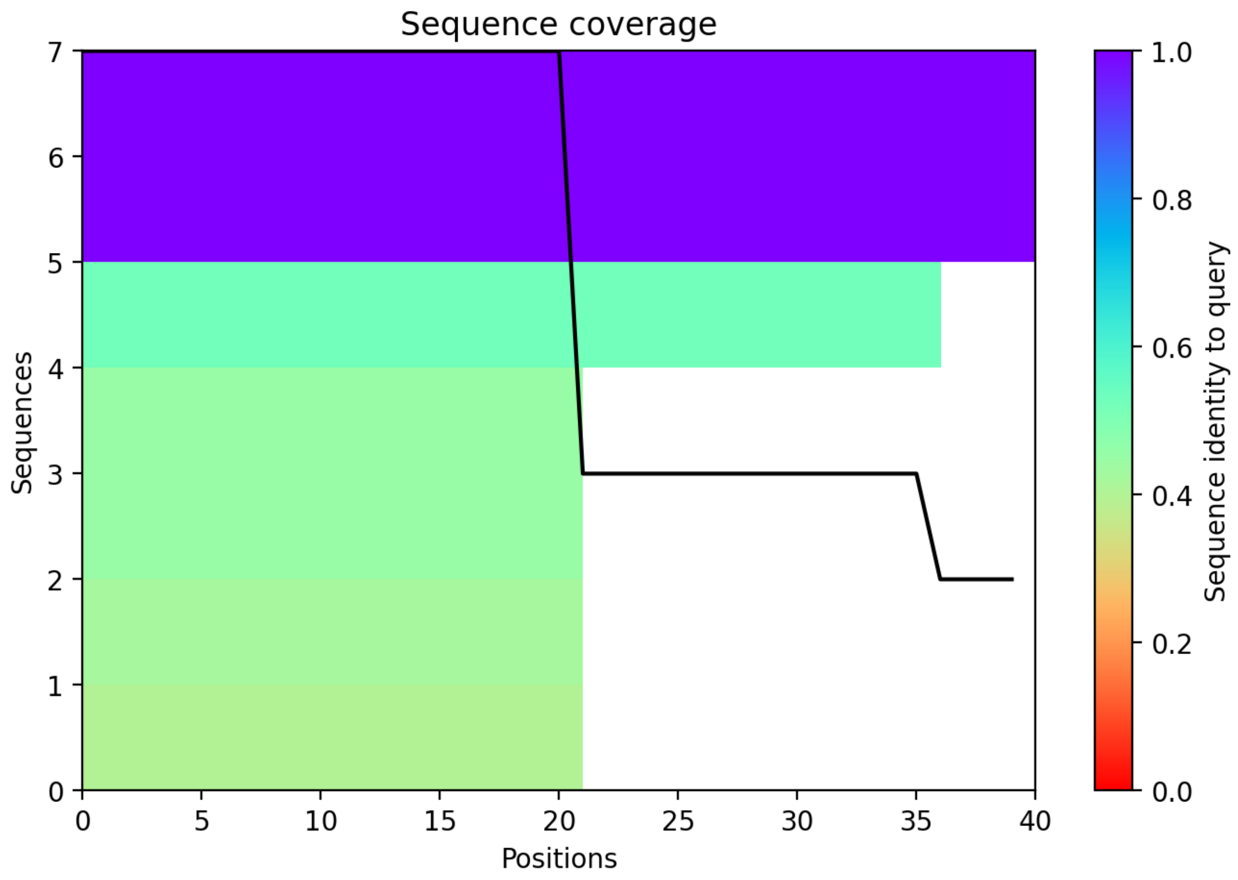

CP137: MtrunA17_Chr8g0376411

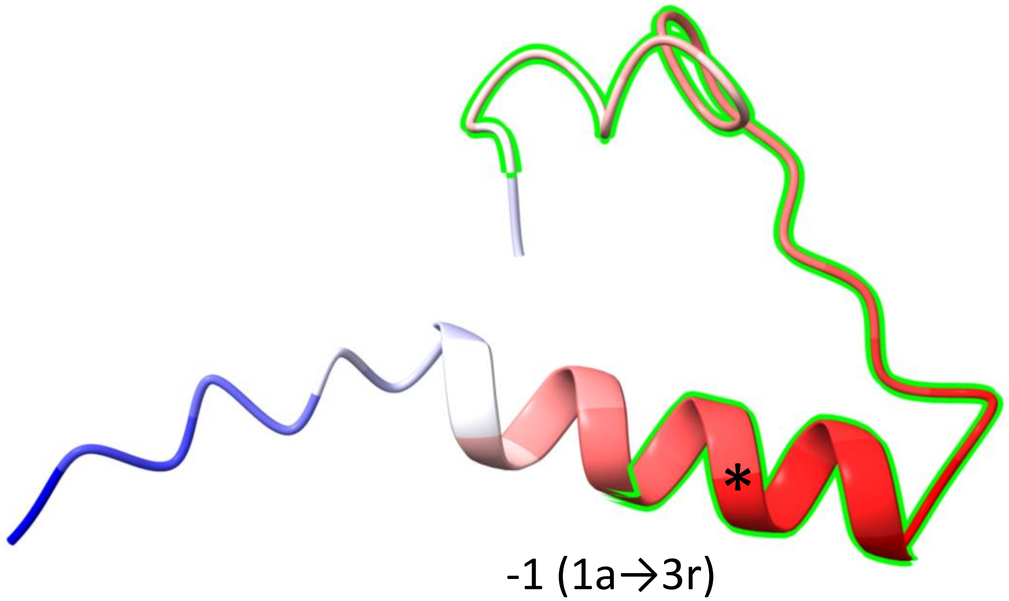

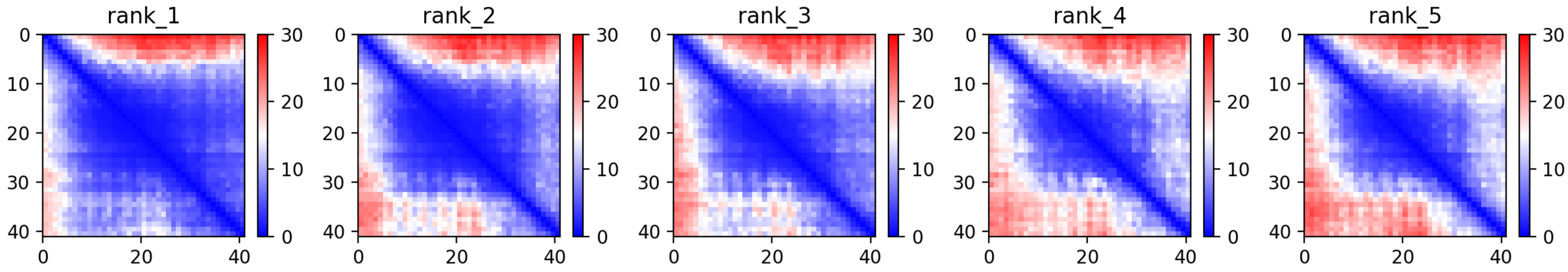

CP137: MtrunA17_Chr8g0376411

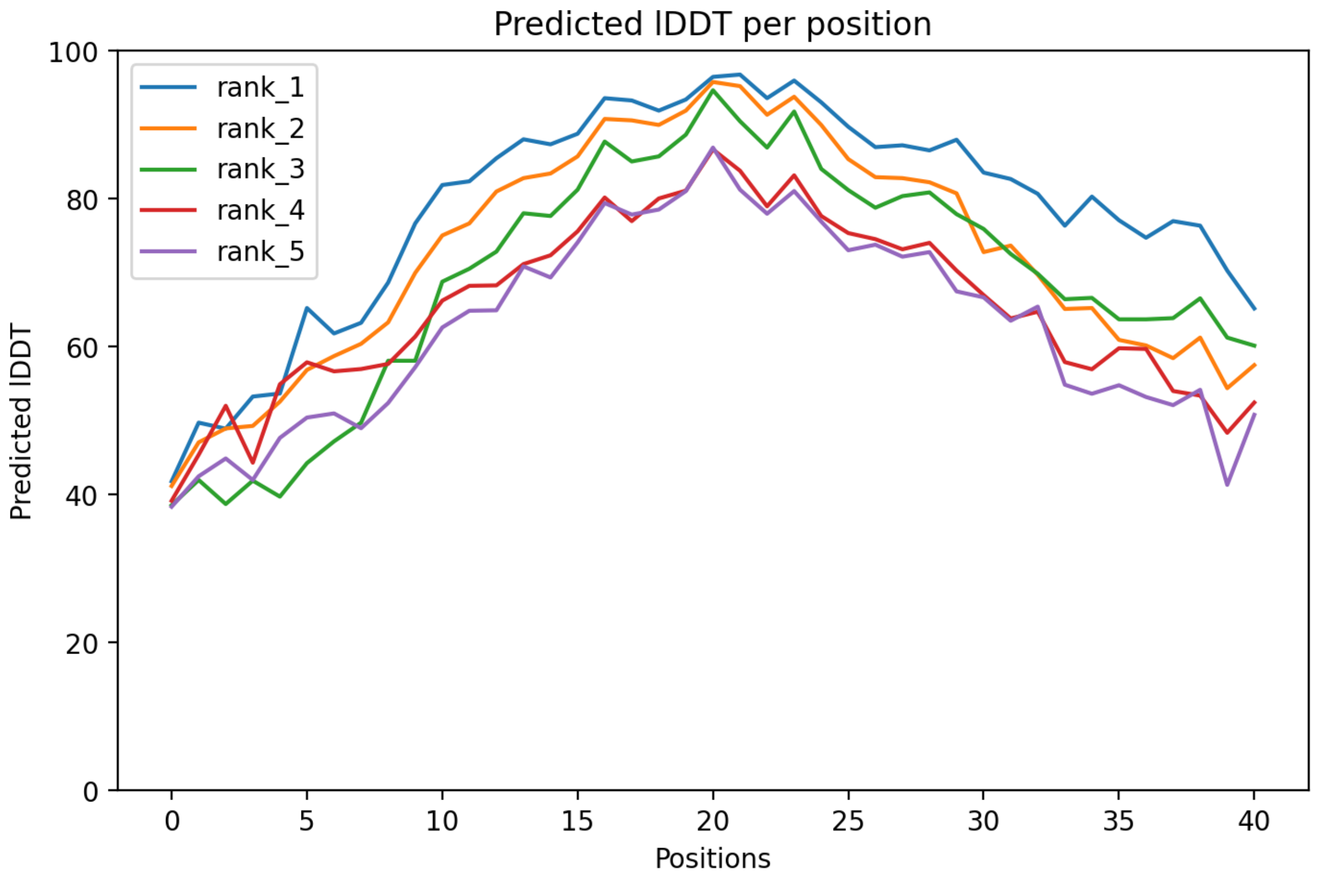

CP137: MtrunA17_Chr8g0376411

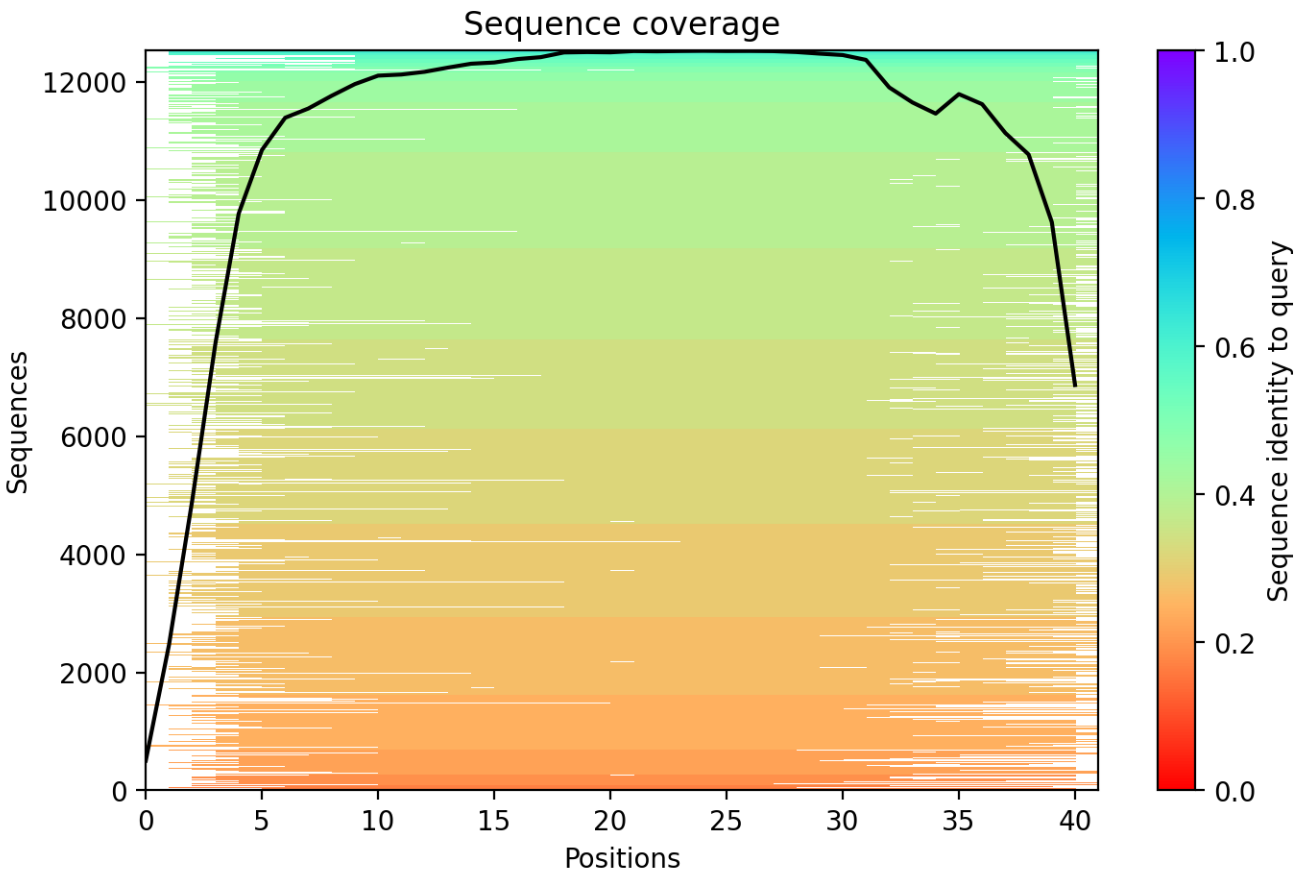

CP138: MtrunA17_Chr8g0377071

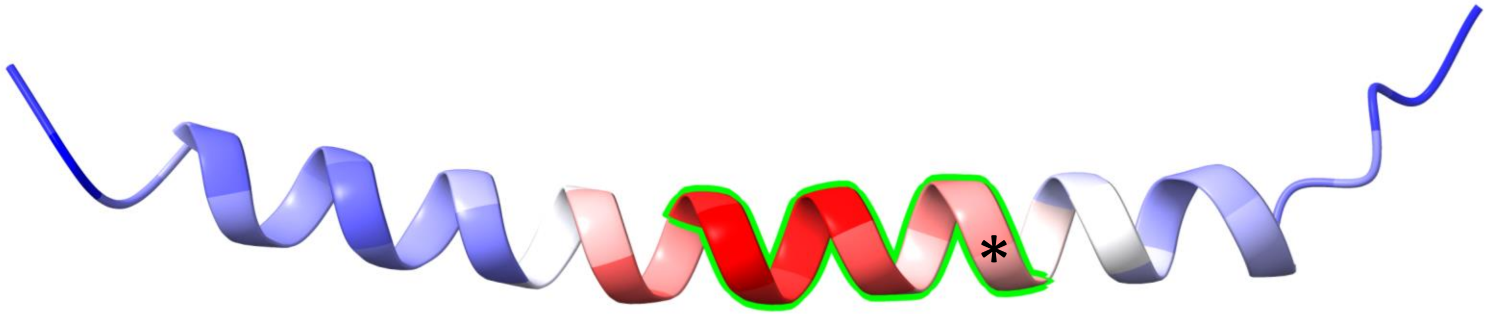

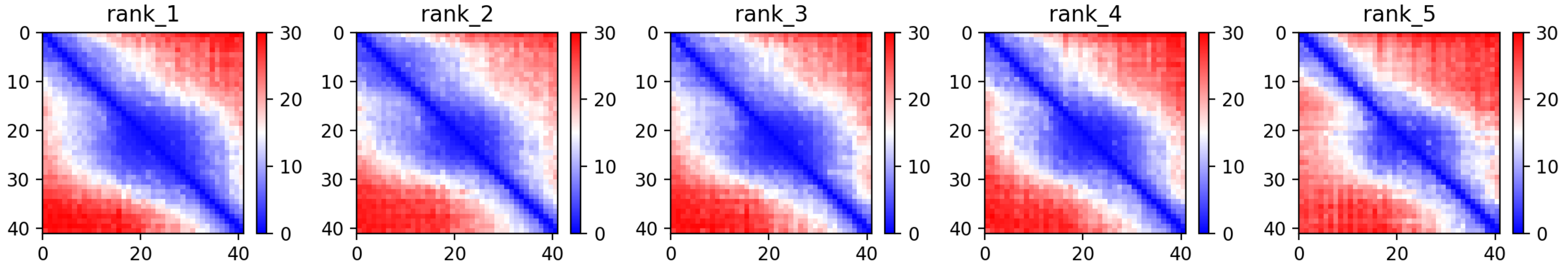

CP138: MtrunA17_Chr8g0377071

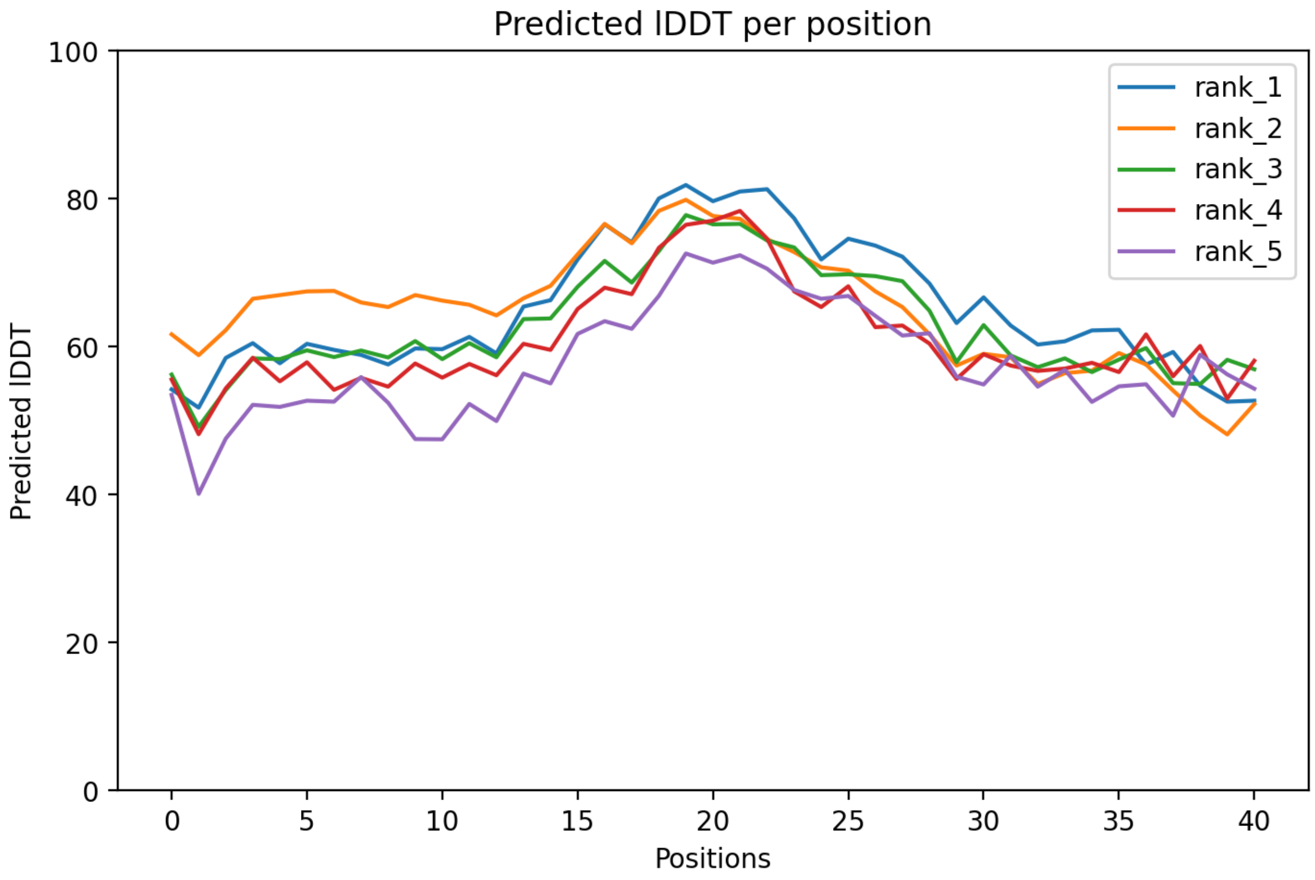

CP138: MtrunA17_Chr8g0377071

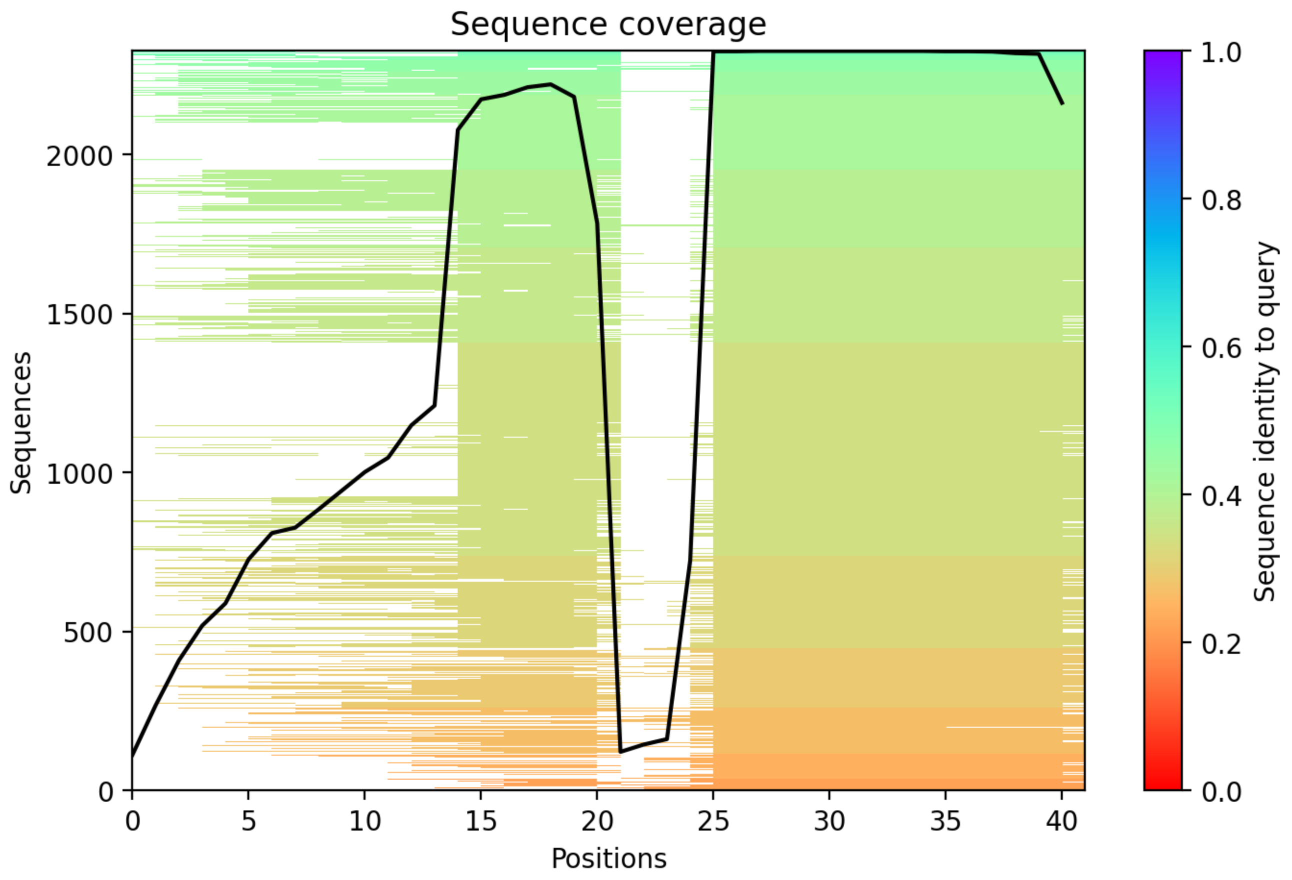

CP139: MtrunA17_Chr8g0385331

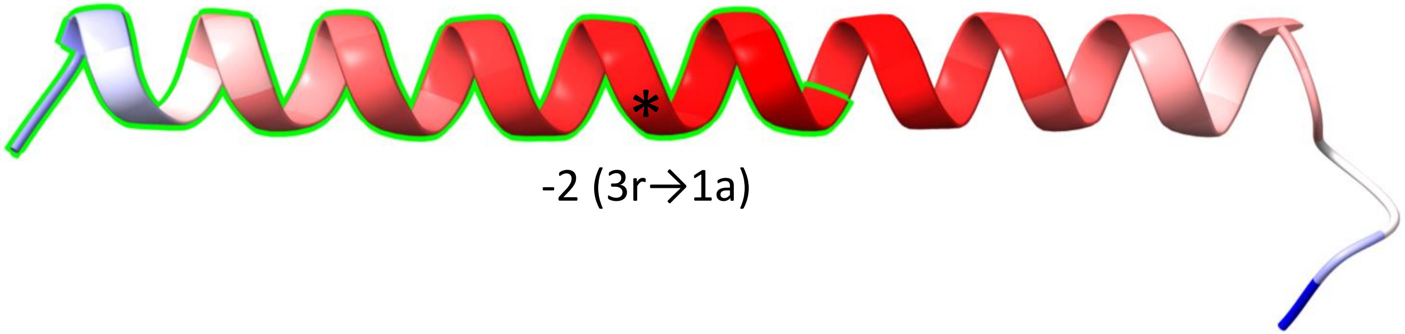

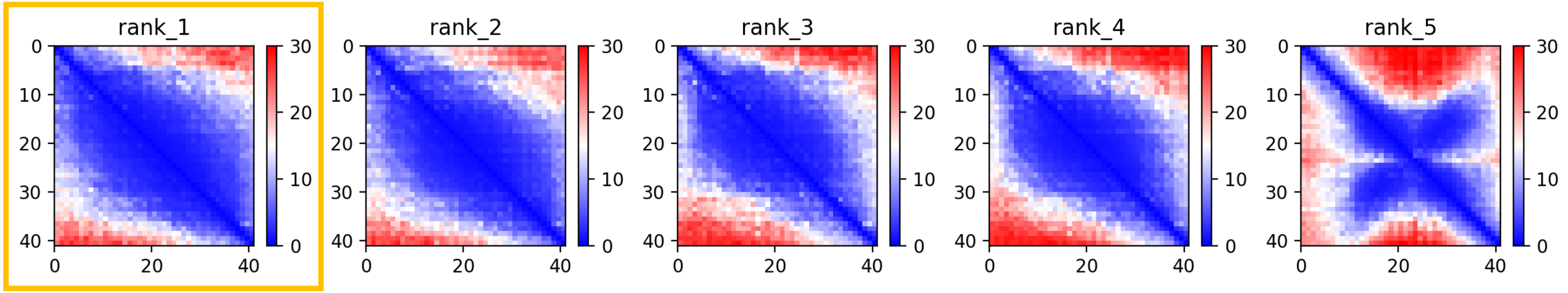

CP139: MtrunA17_Chr8g0385331

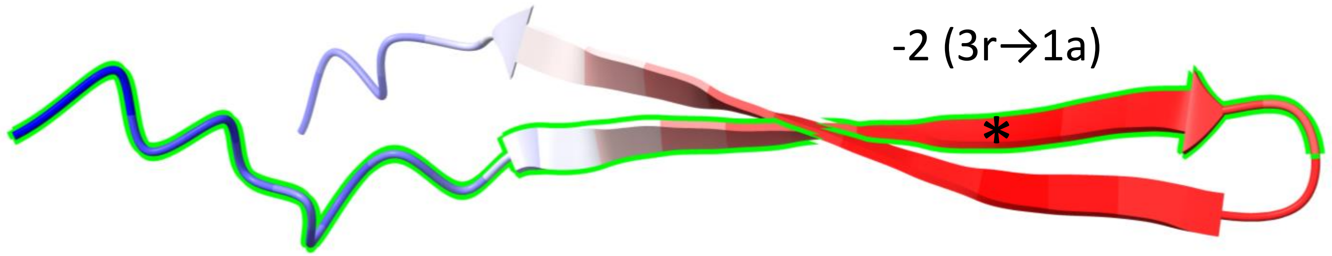

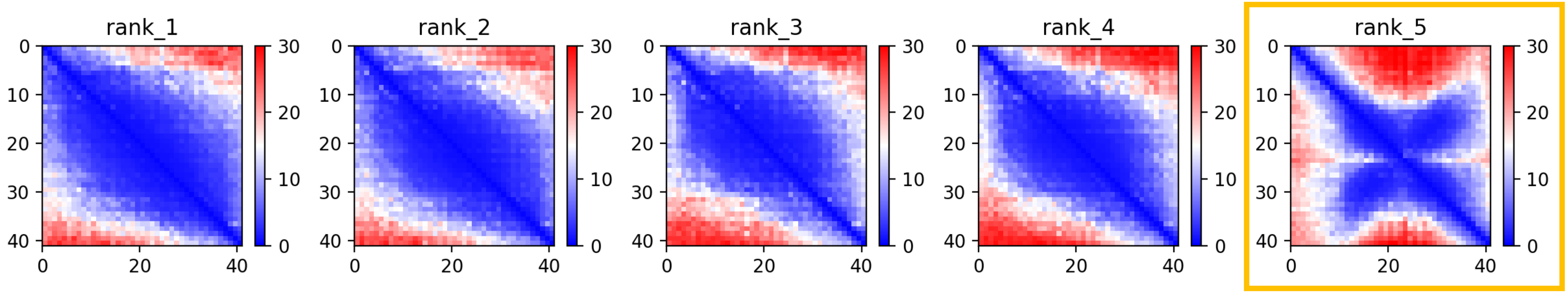

CP139: MtrunA17_Chr8g0385331

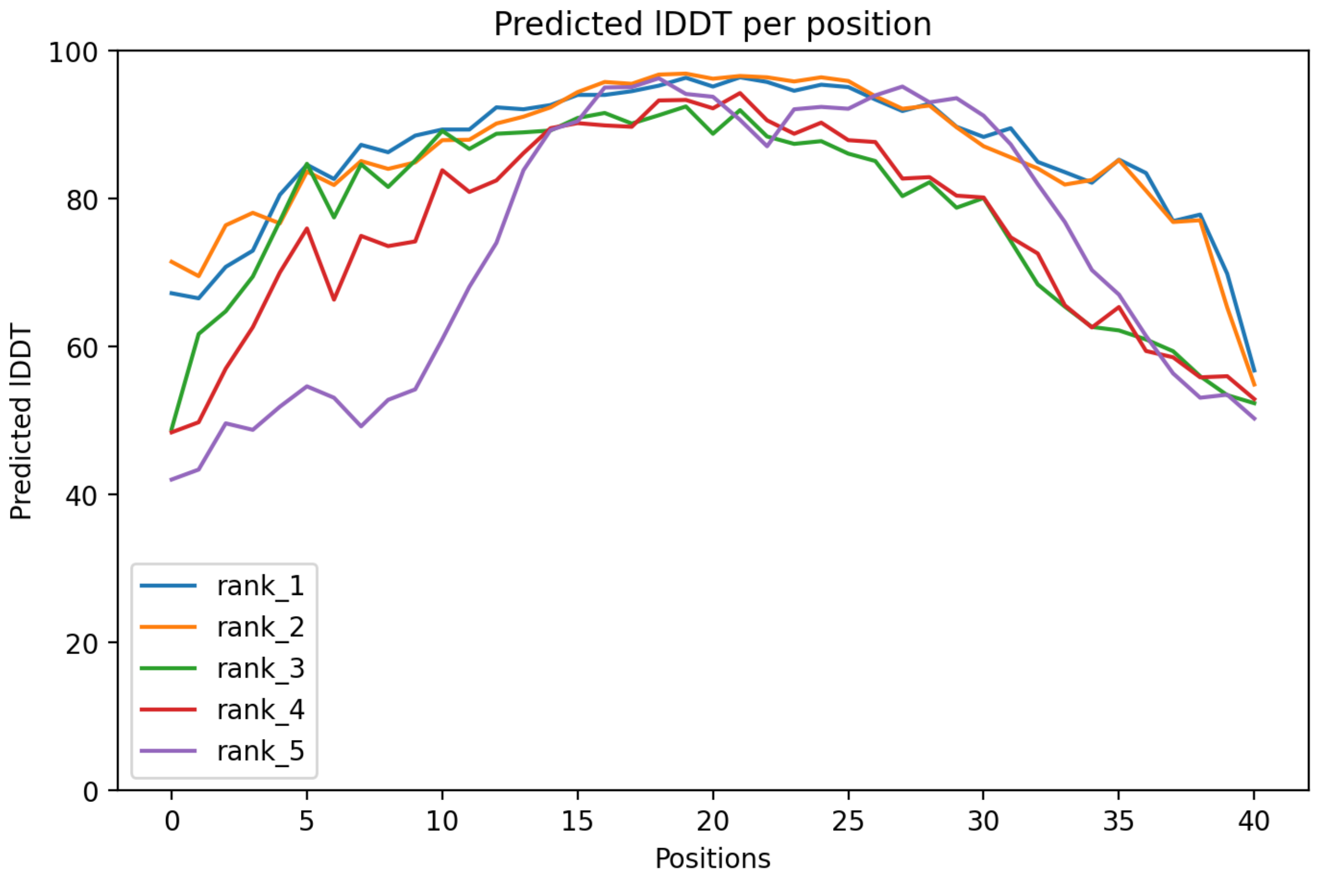

CP139: MtrunA17_Chr8g0385331

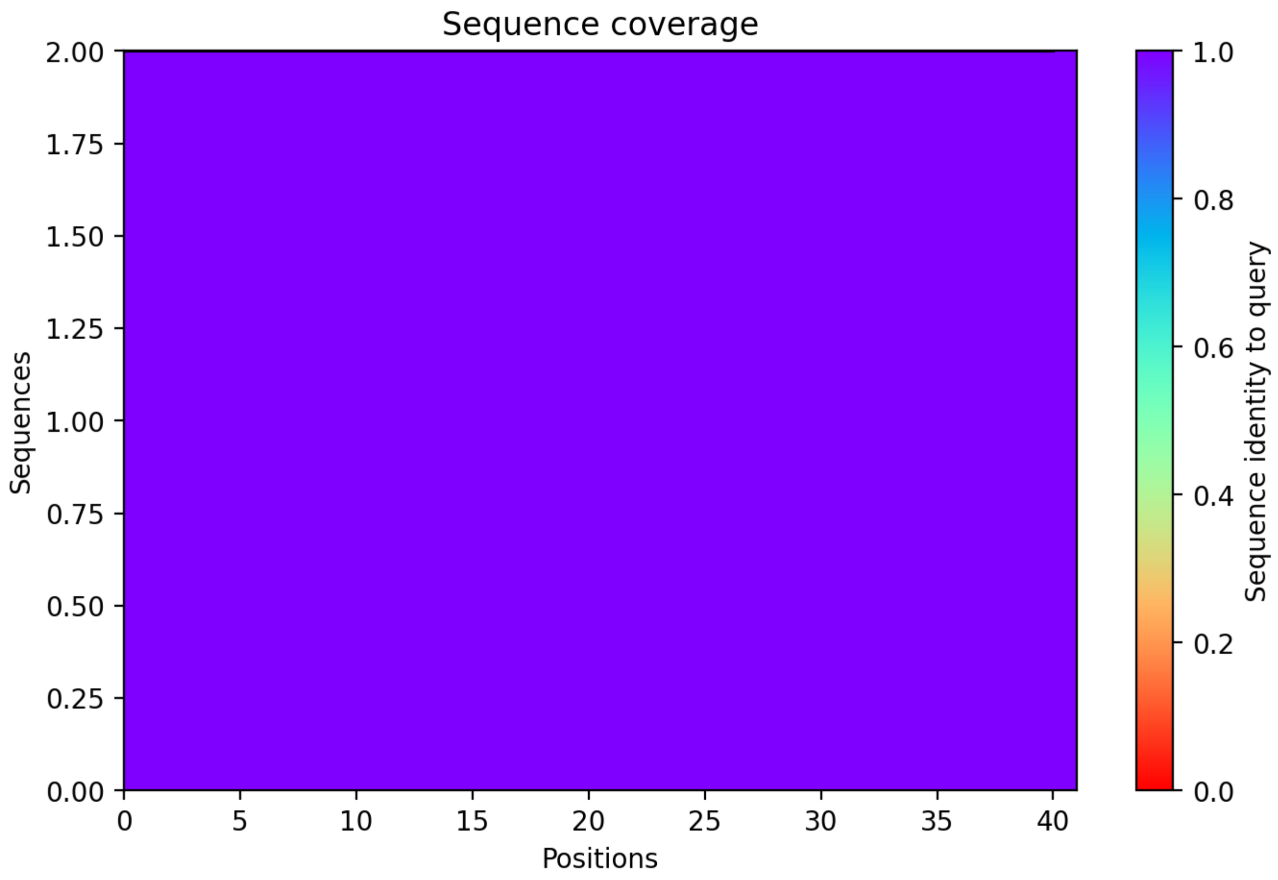

CP140: MtrunA17_Chr8g0392351

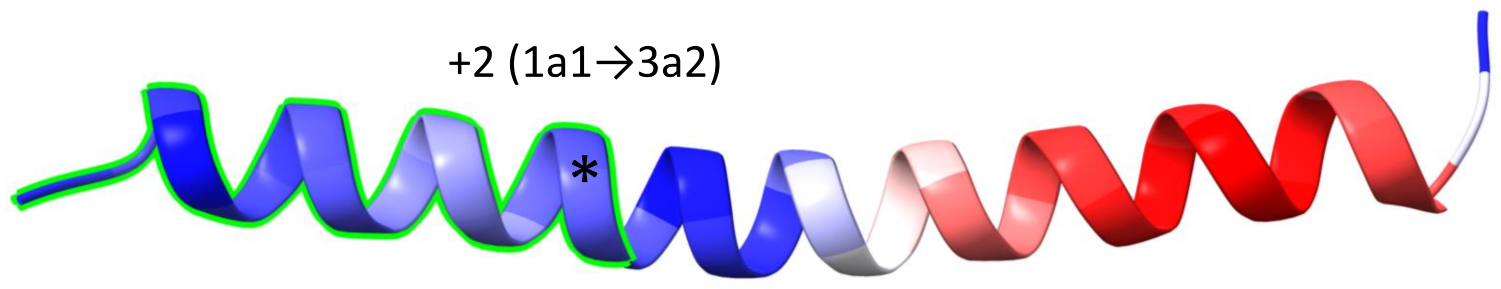

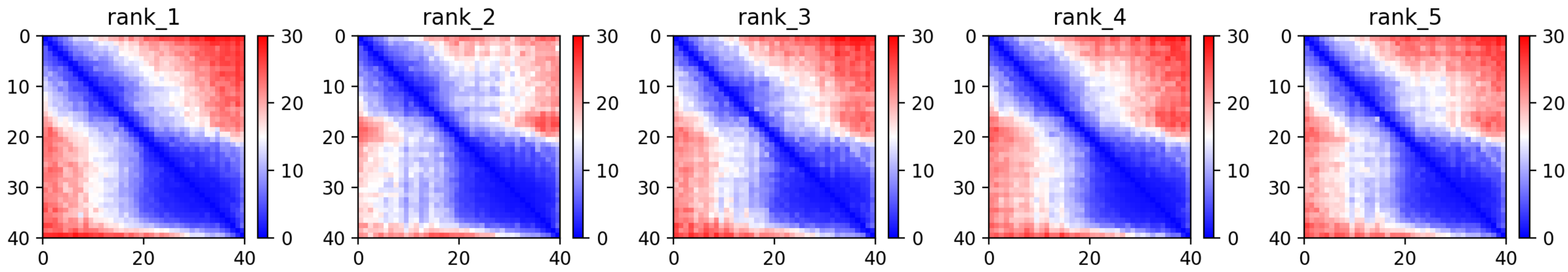

CP140: MtrunA17_Chr8g0392351

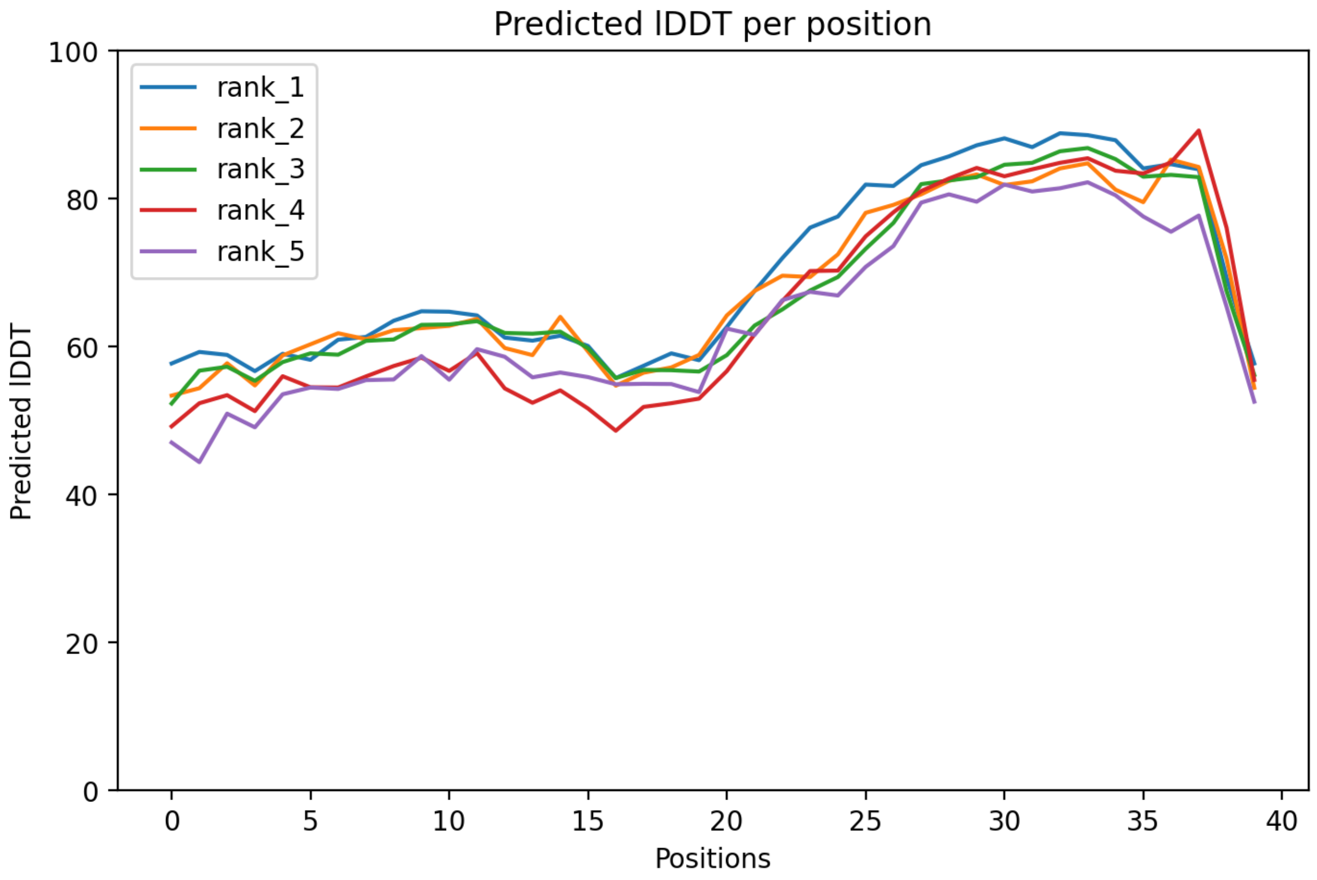

CP140: MtrunA17_Chr8g0392351

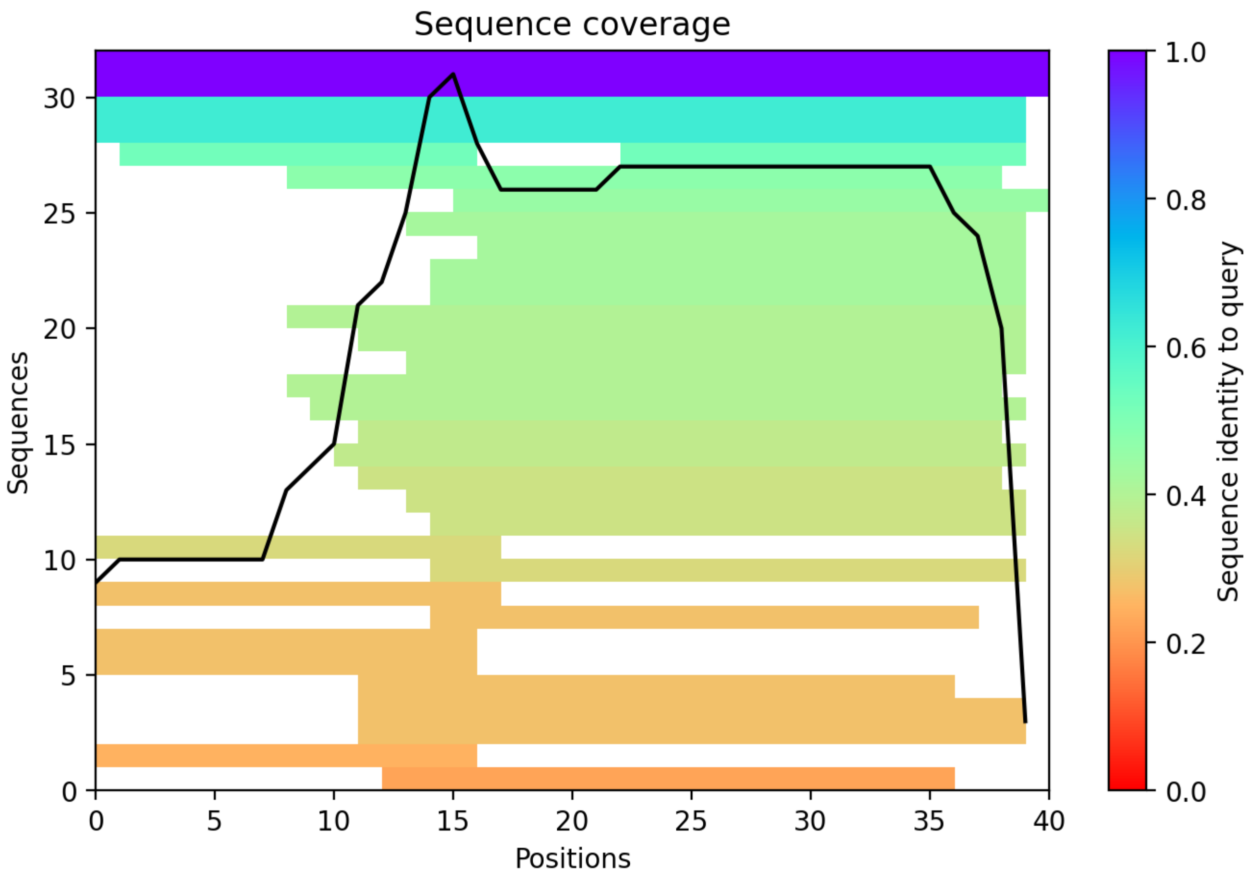

CP141: MtrunA17_CPg0492331

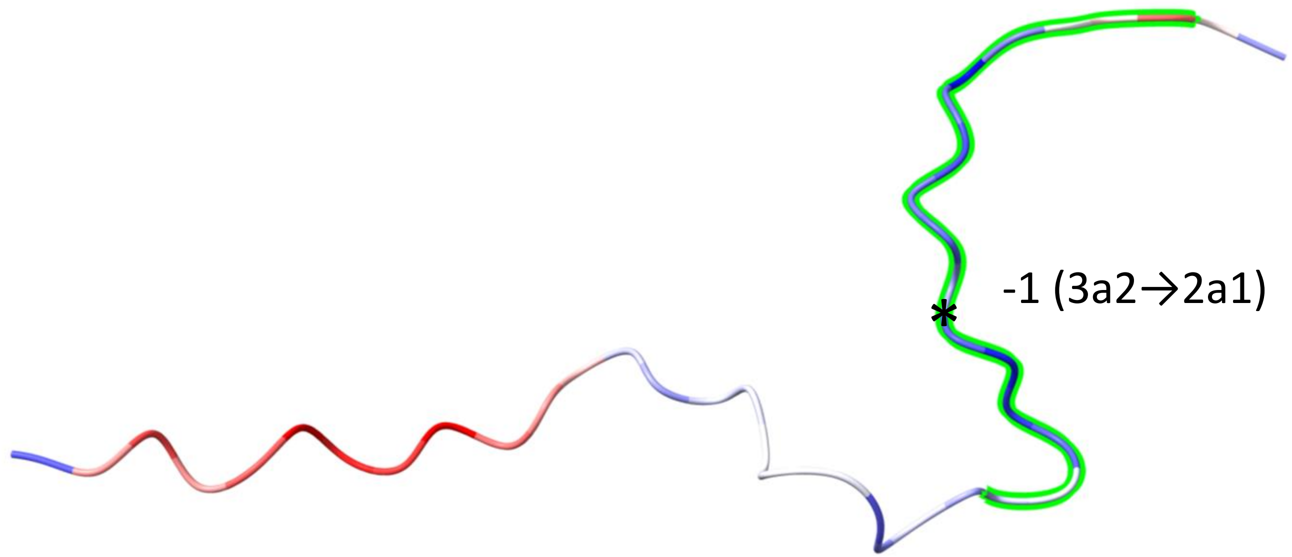

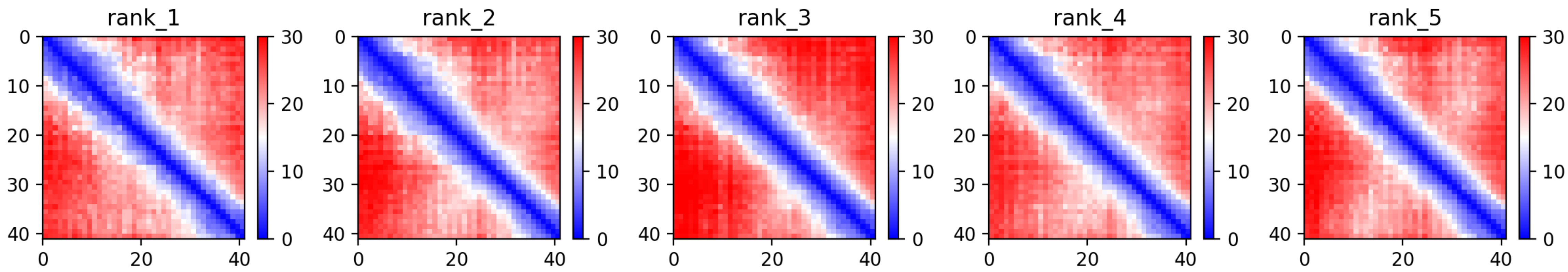

CP141: MtrunA17_CPg0492331

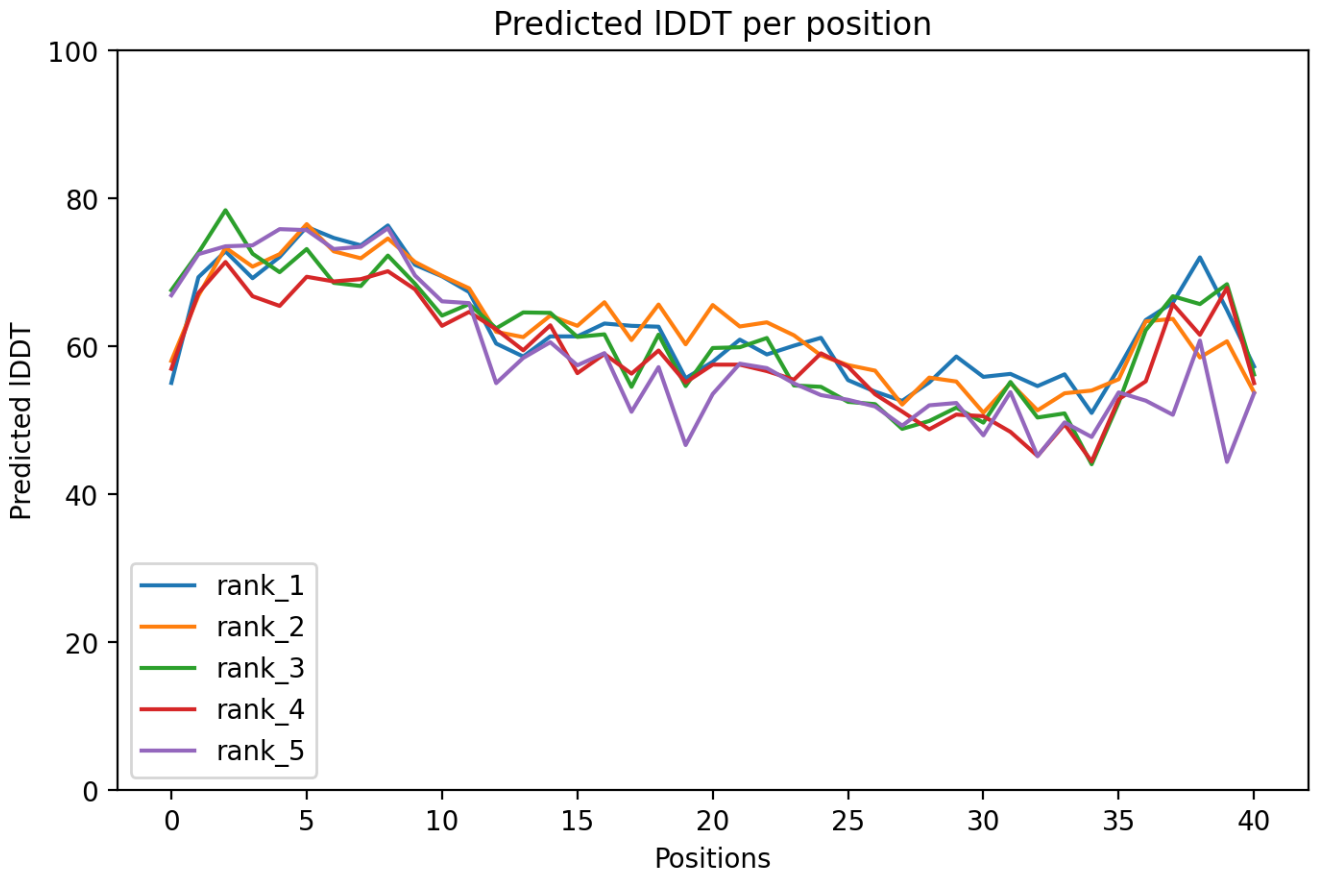

CP141: MtrunA17_CPg0492331

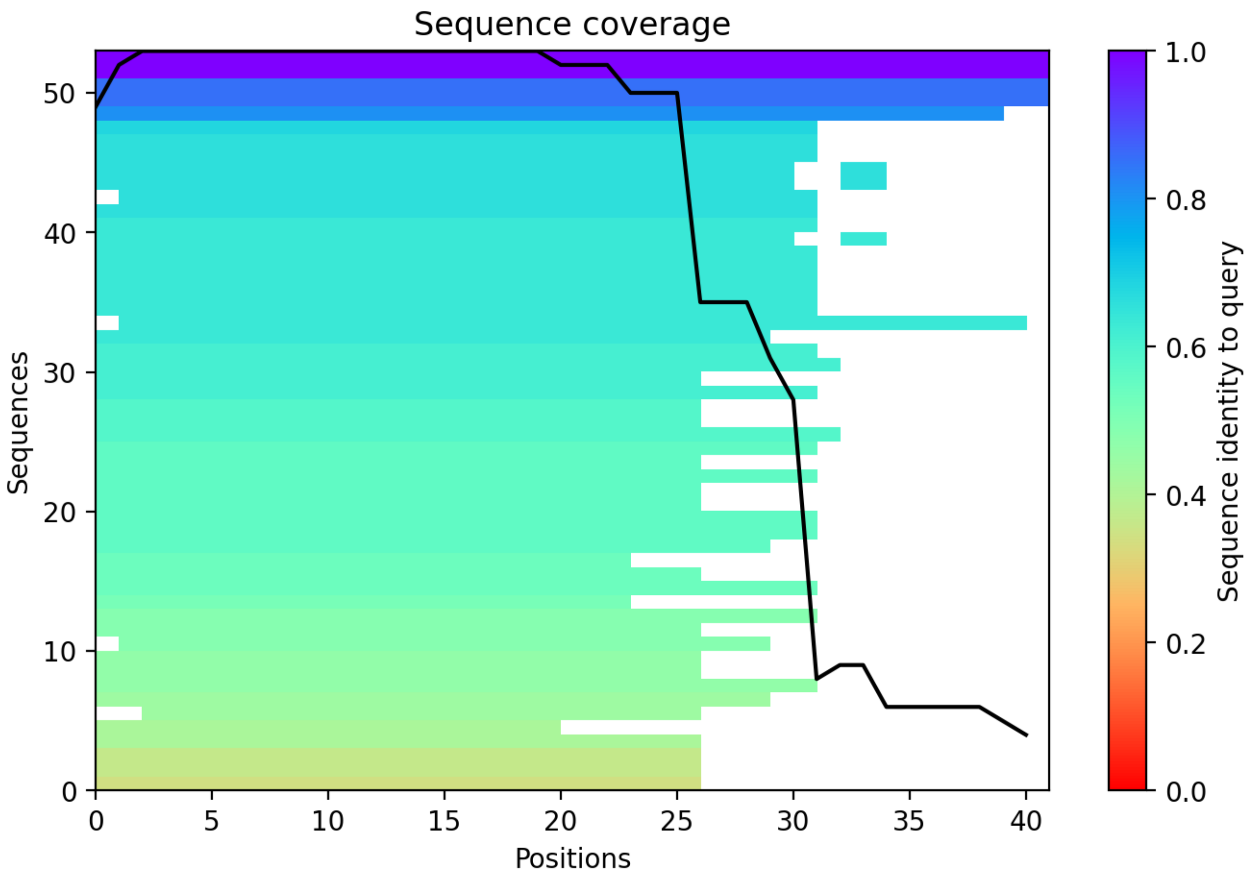

CP142: MtrunA17_CPg0492381

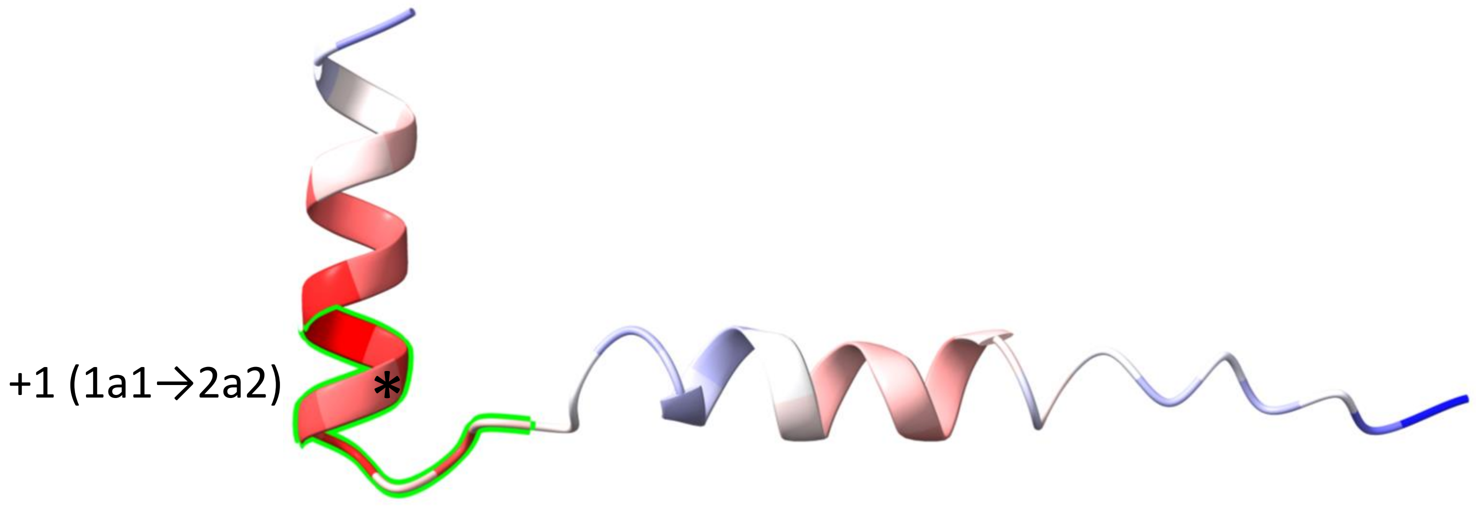

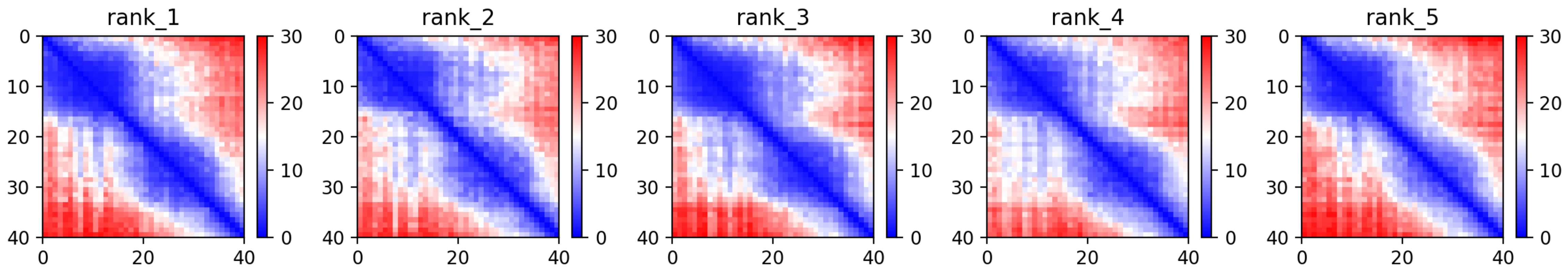

CP142: MtrunA17_CPg0492381

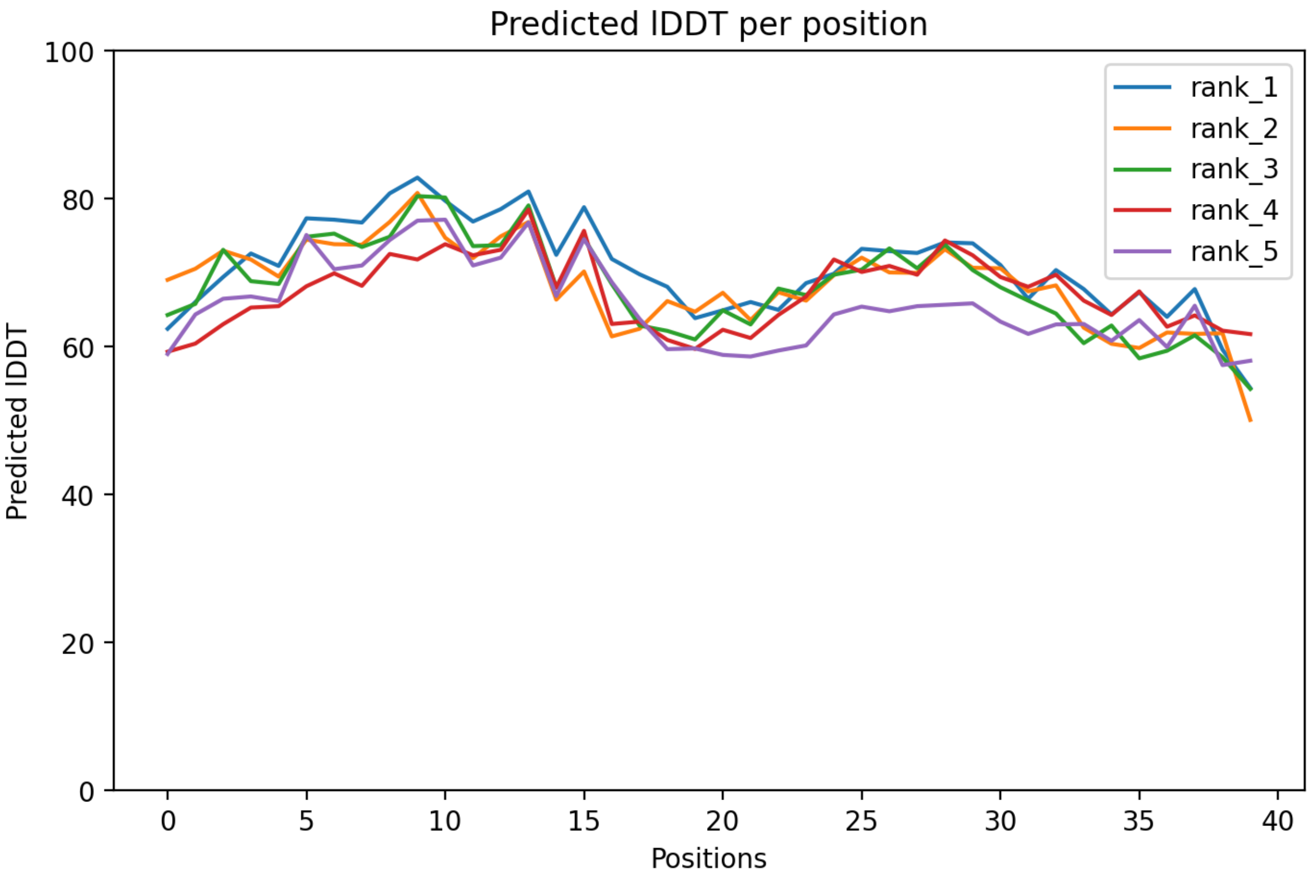

CP142: MtrunA17_CPg0492381

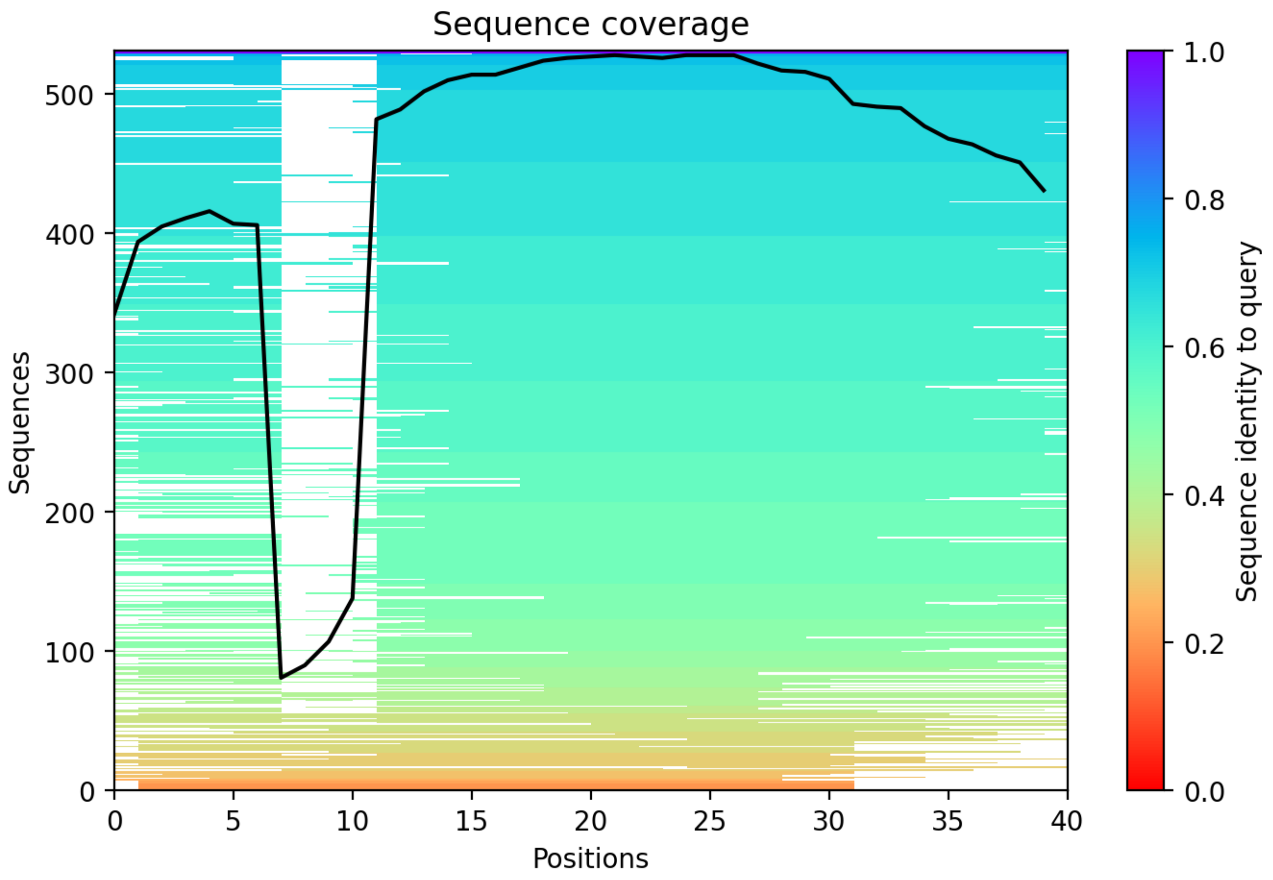

CP143: MtrunA17_CPg0492461

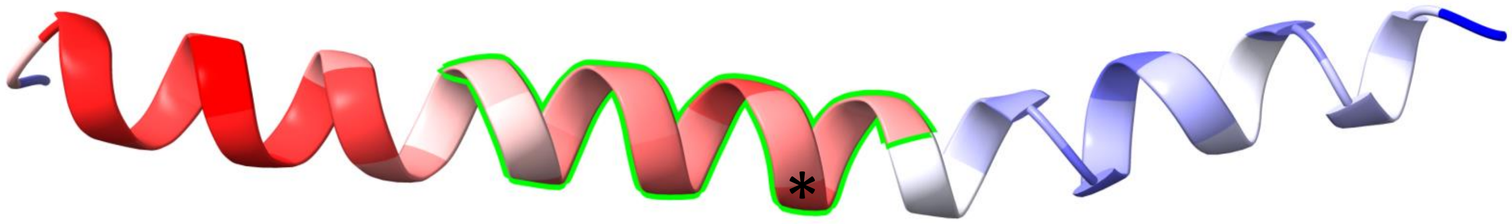

+1 (3r→1a)

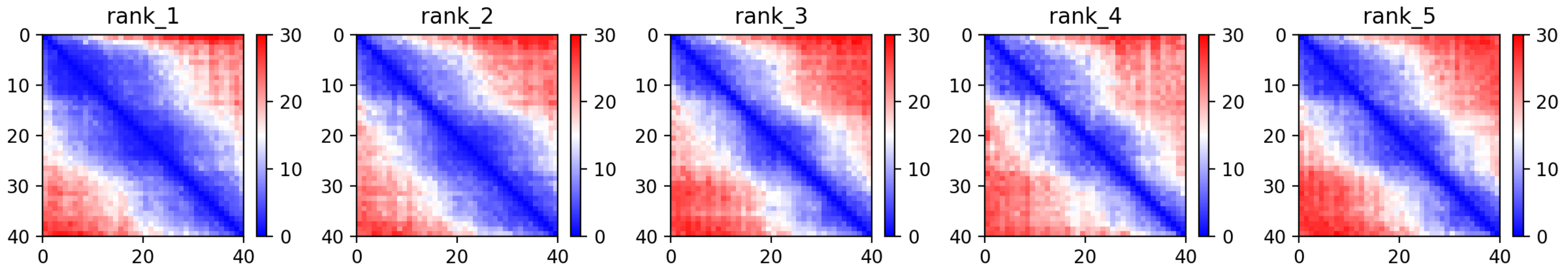

CP143: MtrunA17_CPg0492461

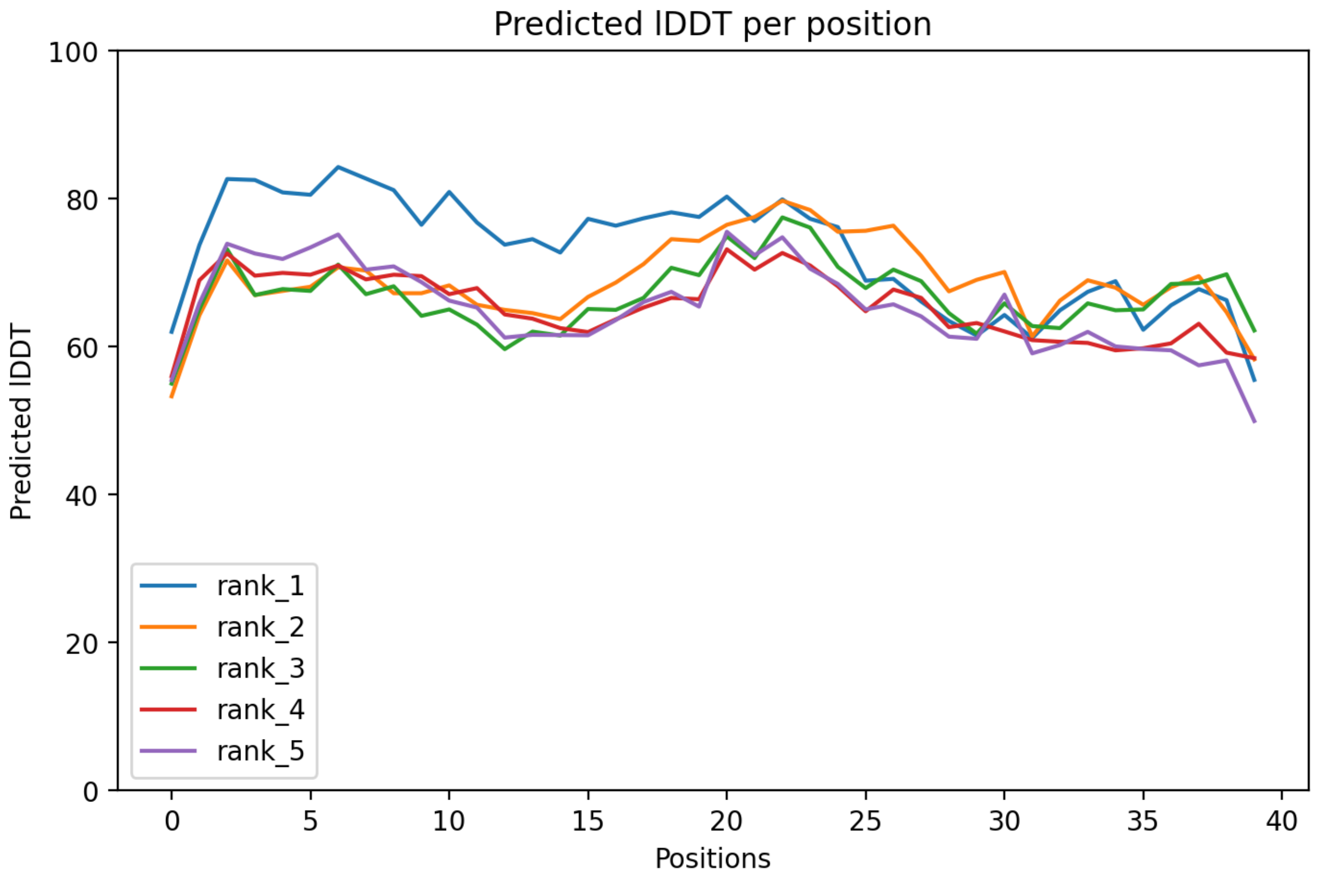

CP143: MtrunA17_CPg0492461

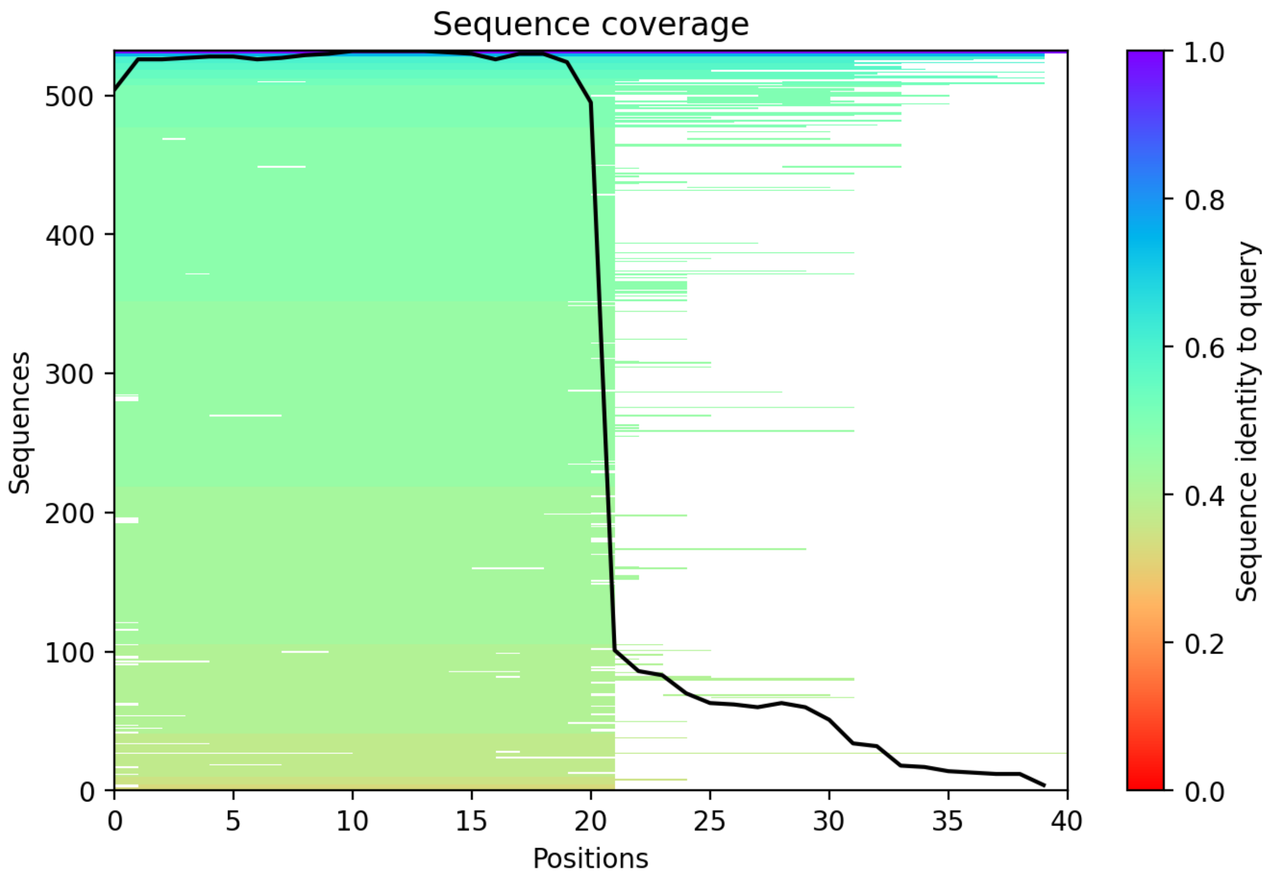

CP144: MtrunA17_CPg0492851

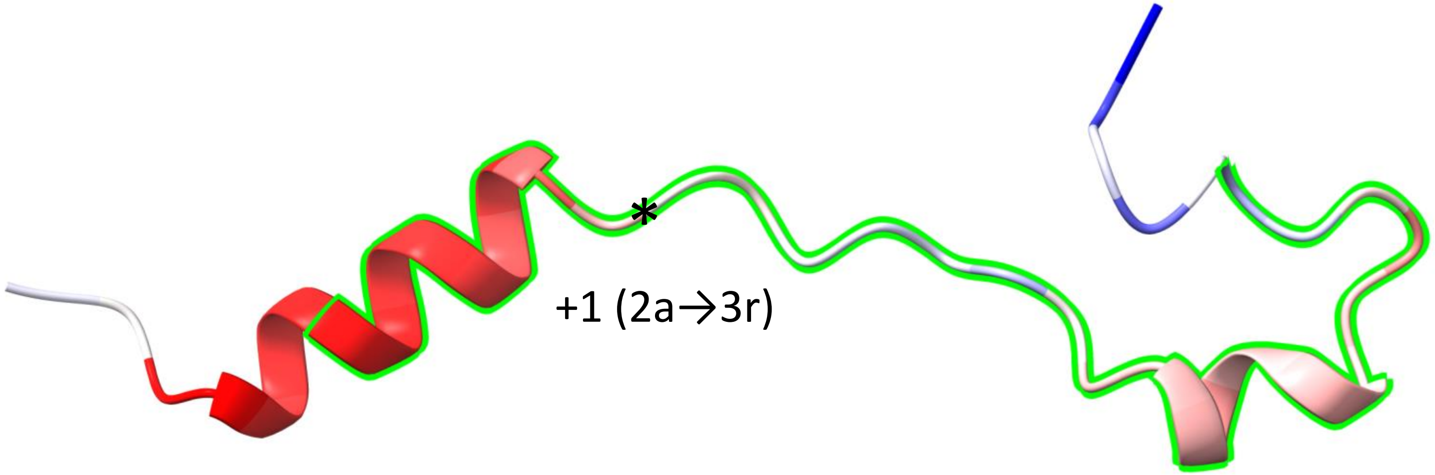

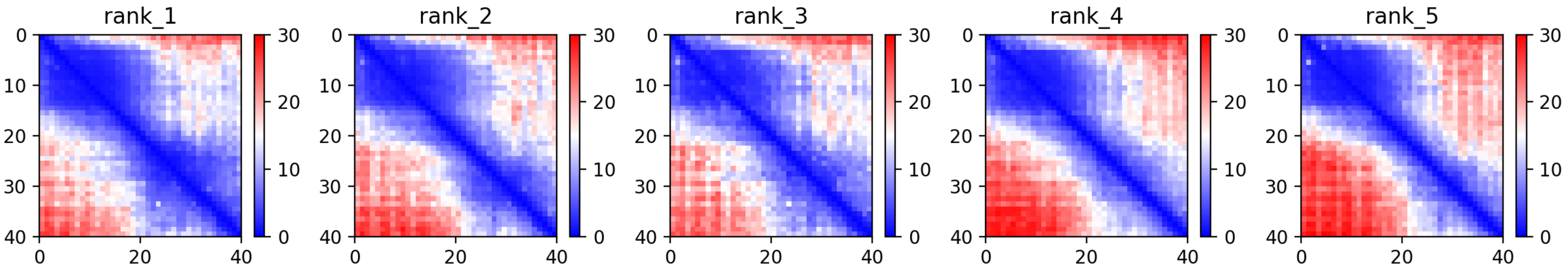

CP144: MtrunA17_CPg0492851

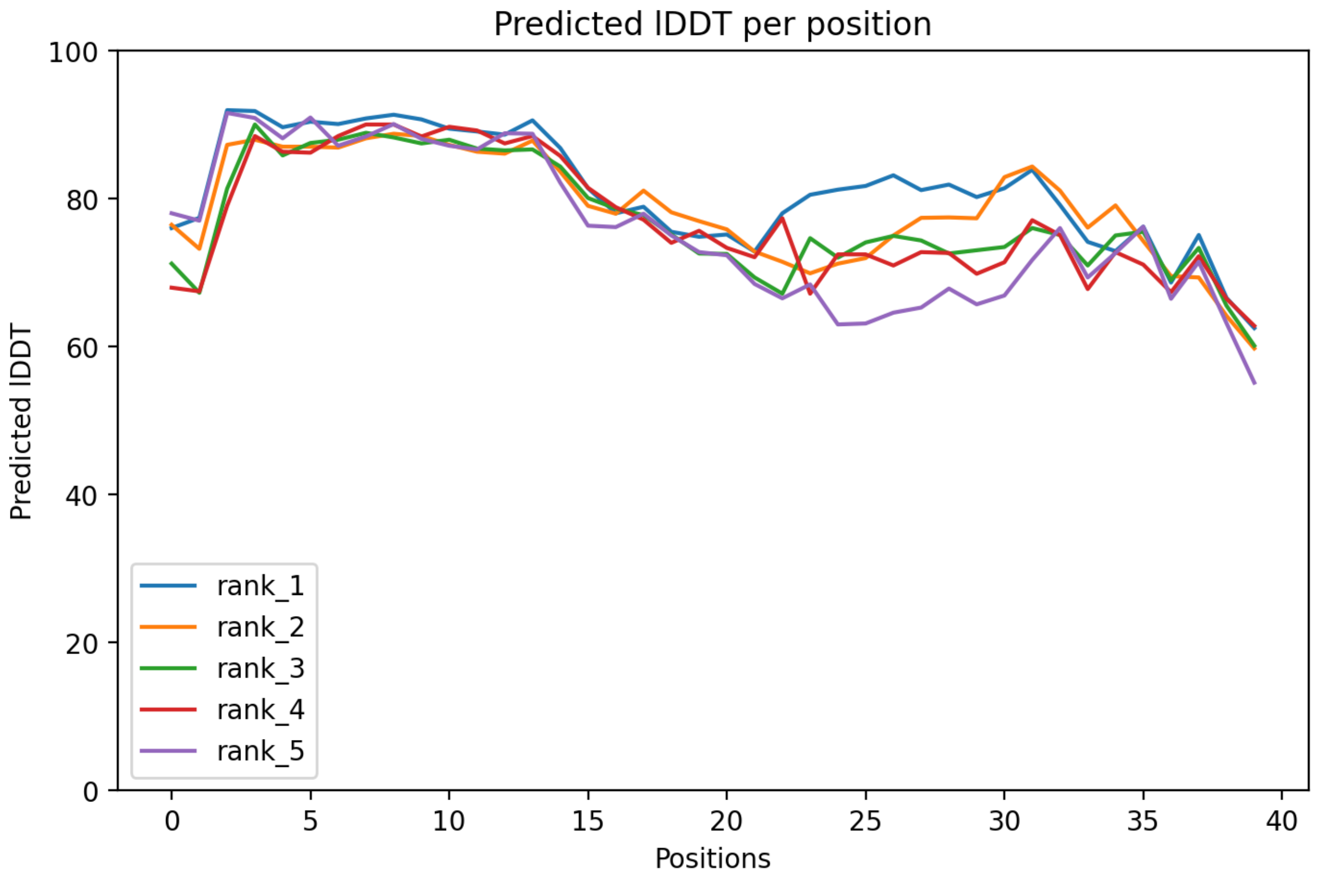

CP144: MtrunA17_CPg0492851

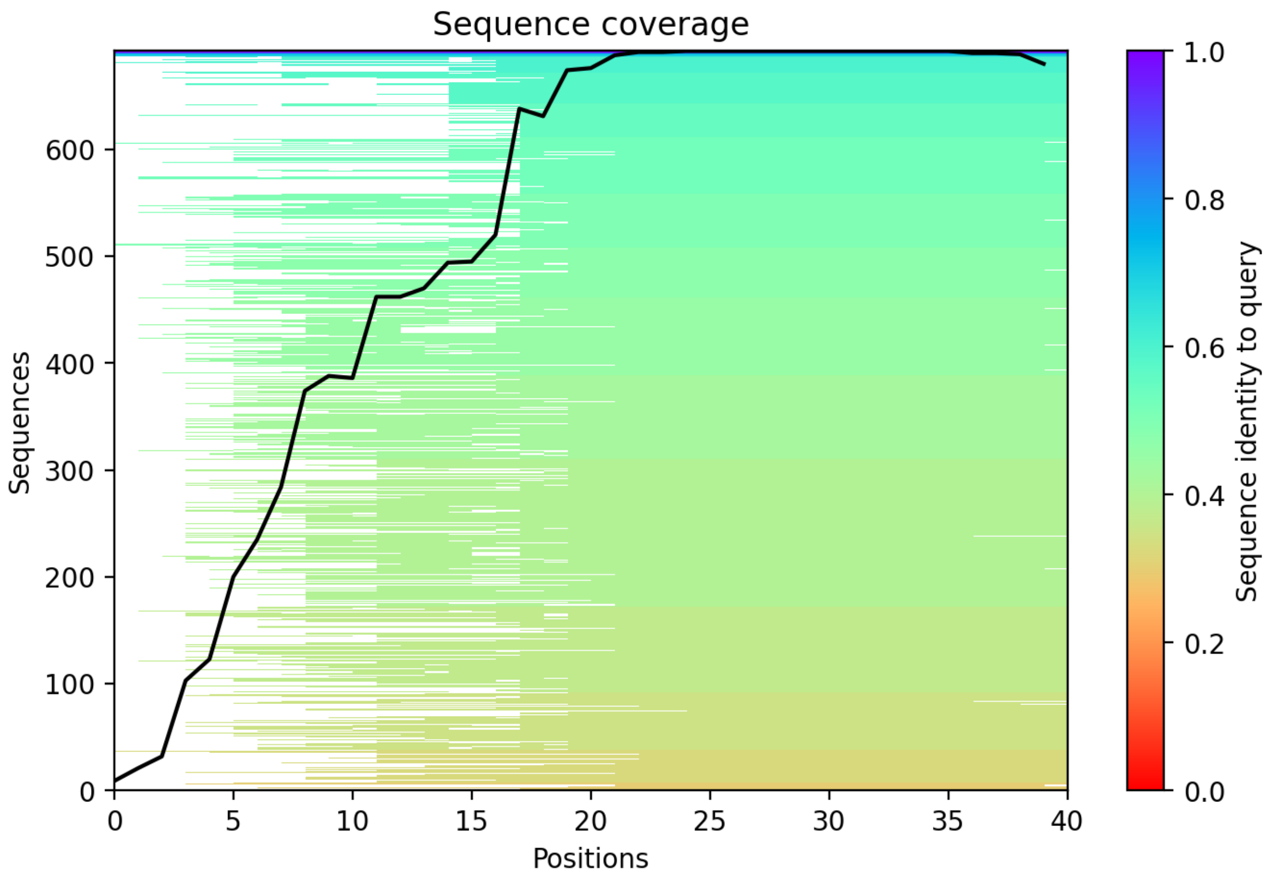

CP145: MtrunA17_CPg0492941

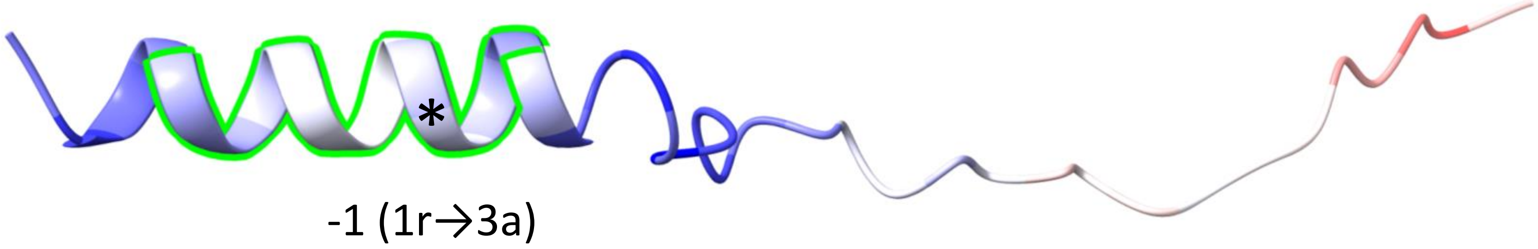

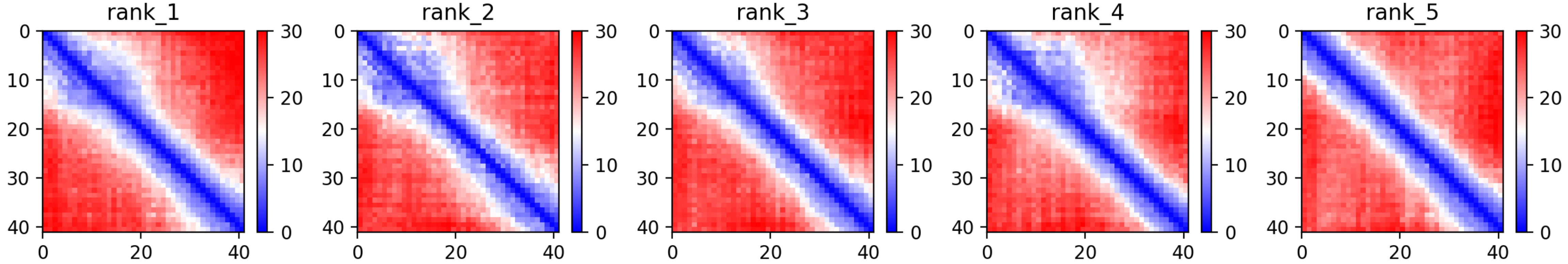

CP145: MtrunA17_CPg0492941

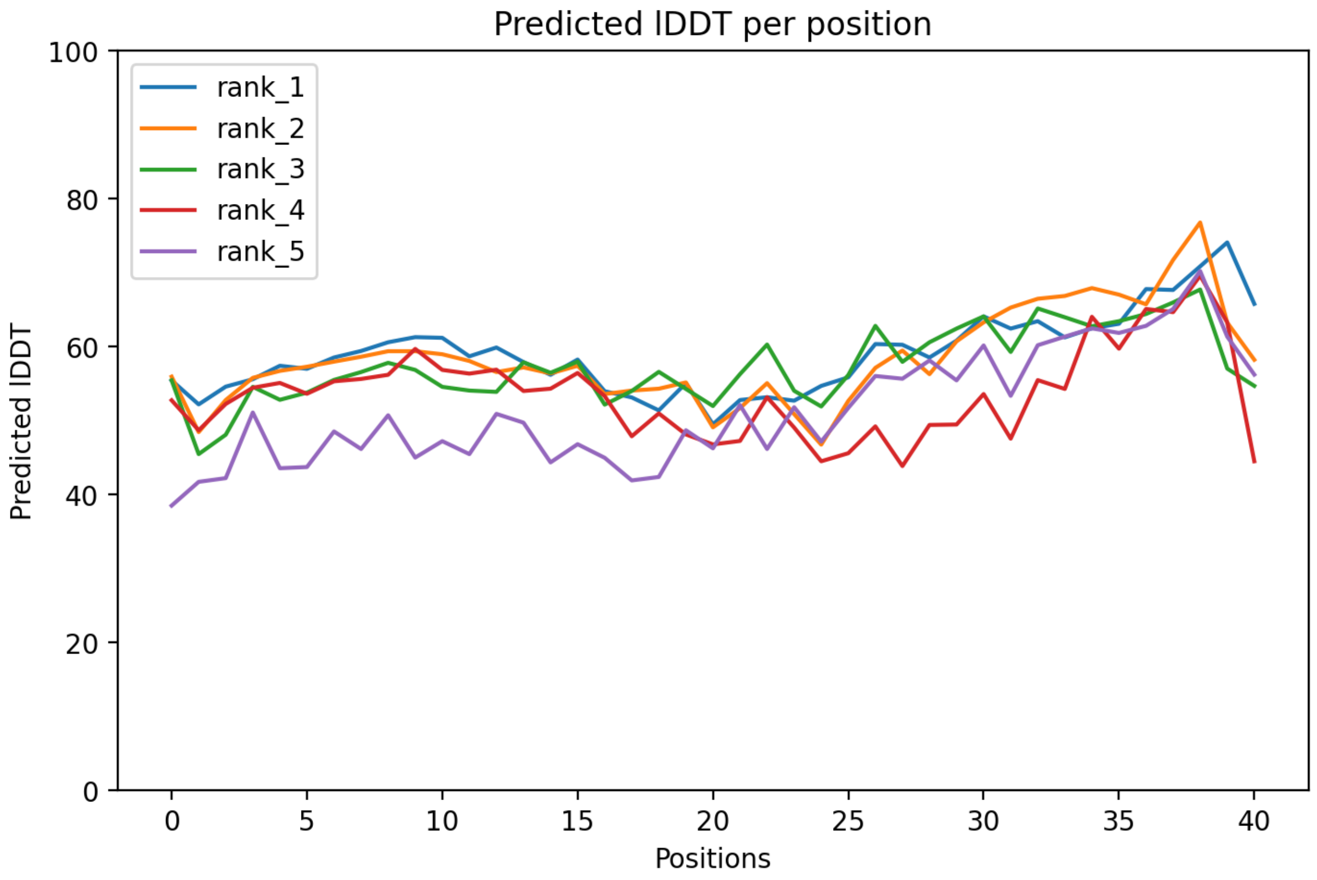

CP145: MtrunA17_CPg0492941

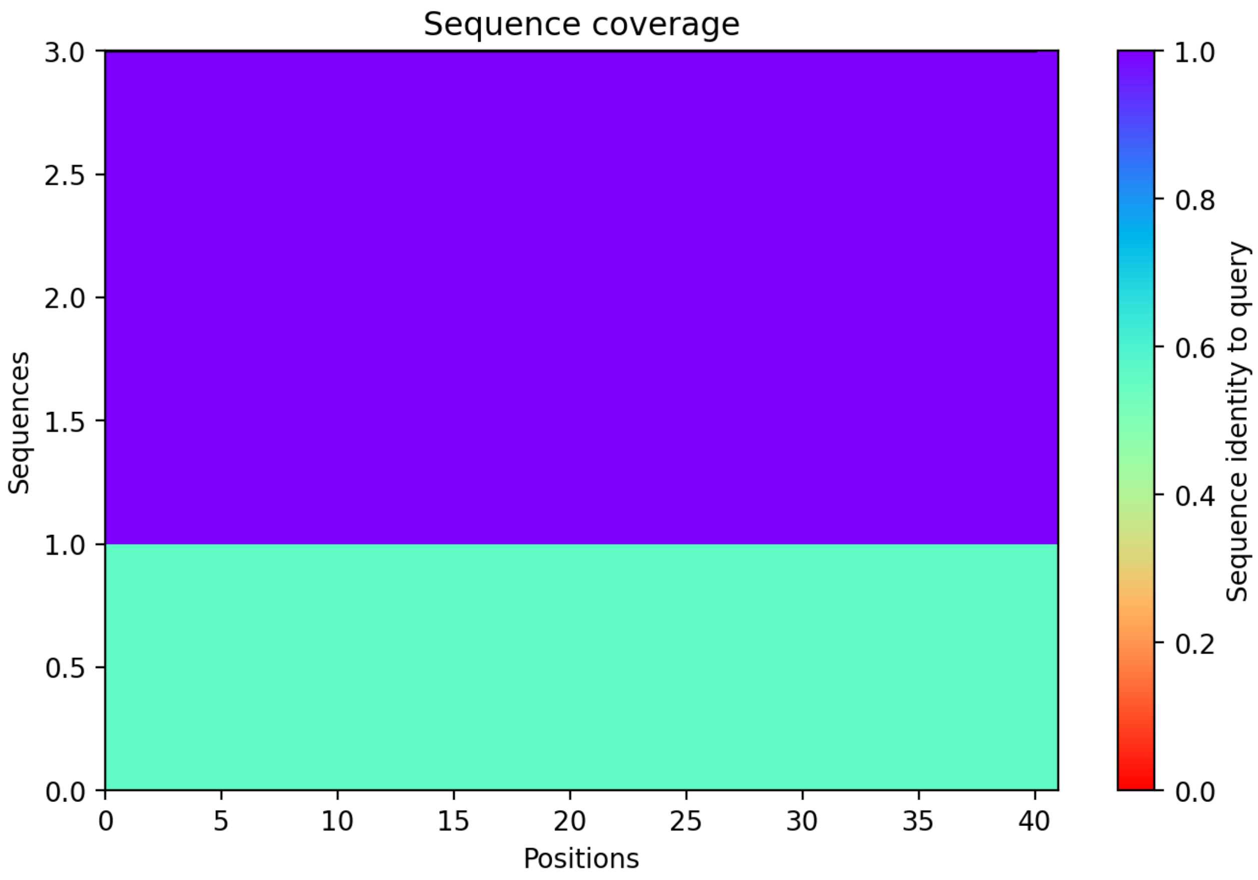

CP146: MtrunA17_CPg0493291

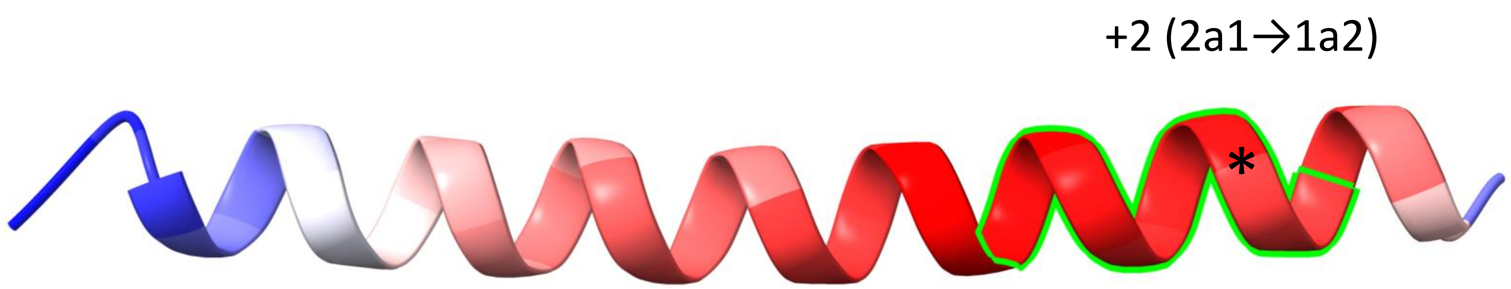

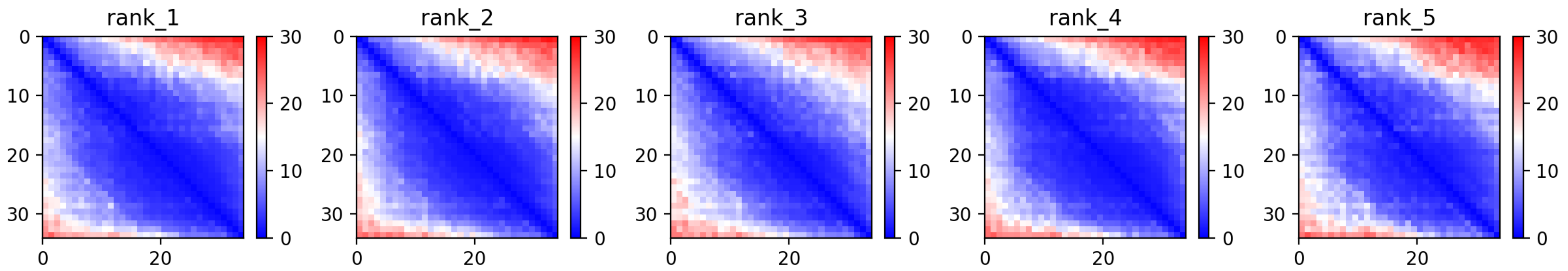

CP146: MtrunA17_CPg0493291

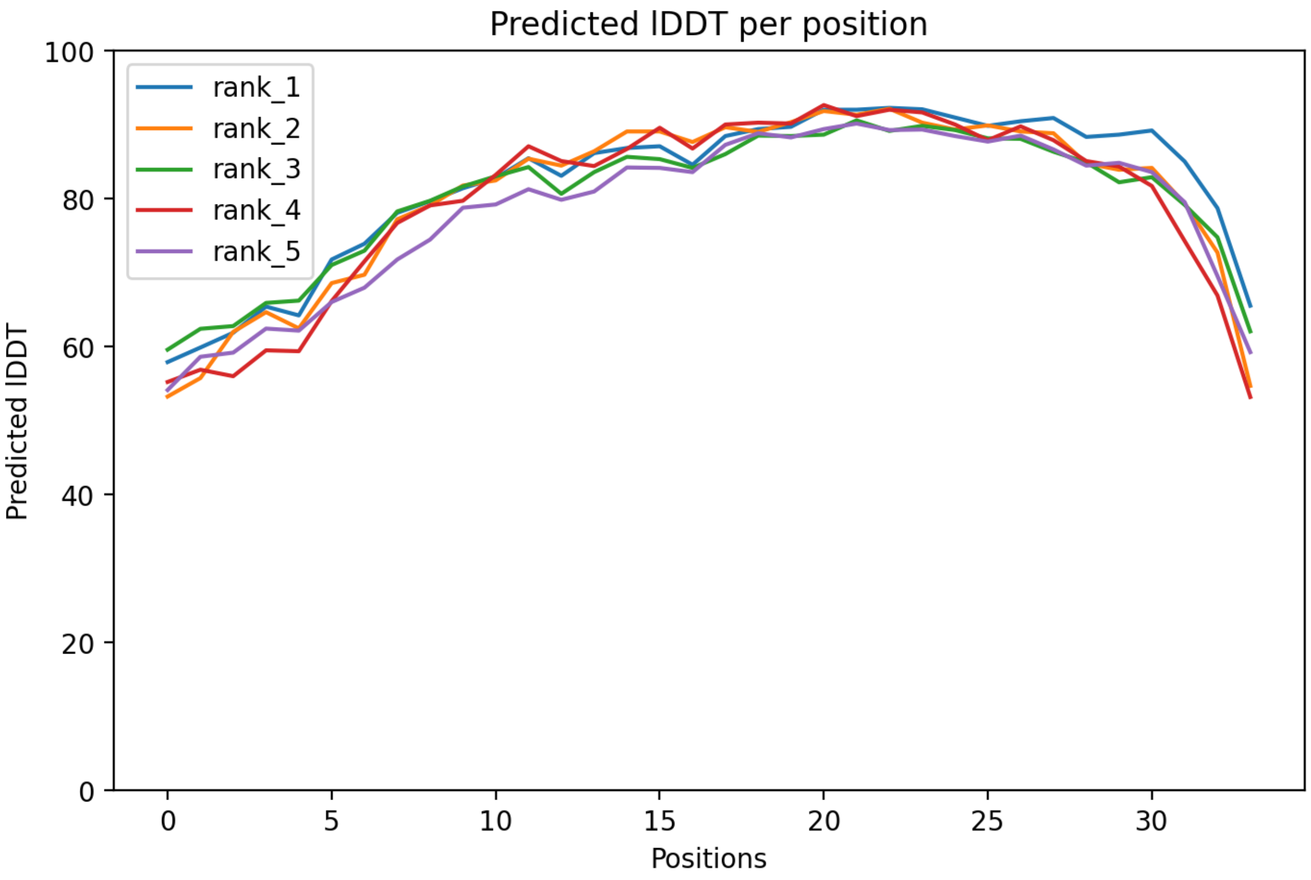

CP146: MtrunA17_CPg0493291

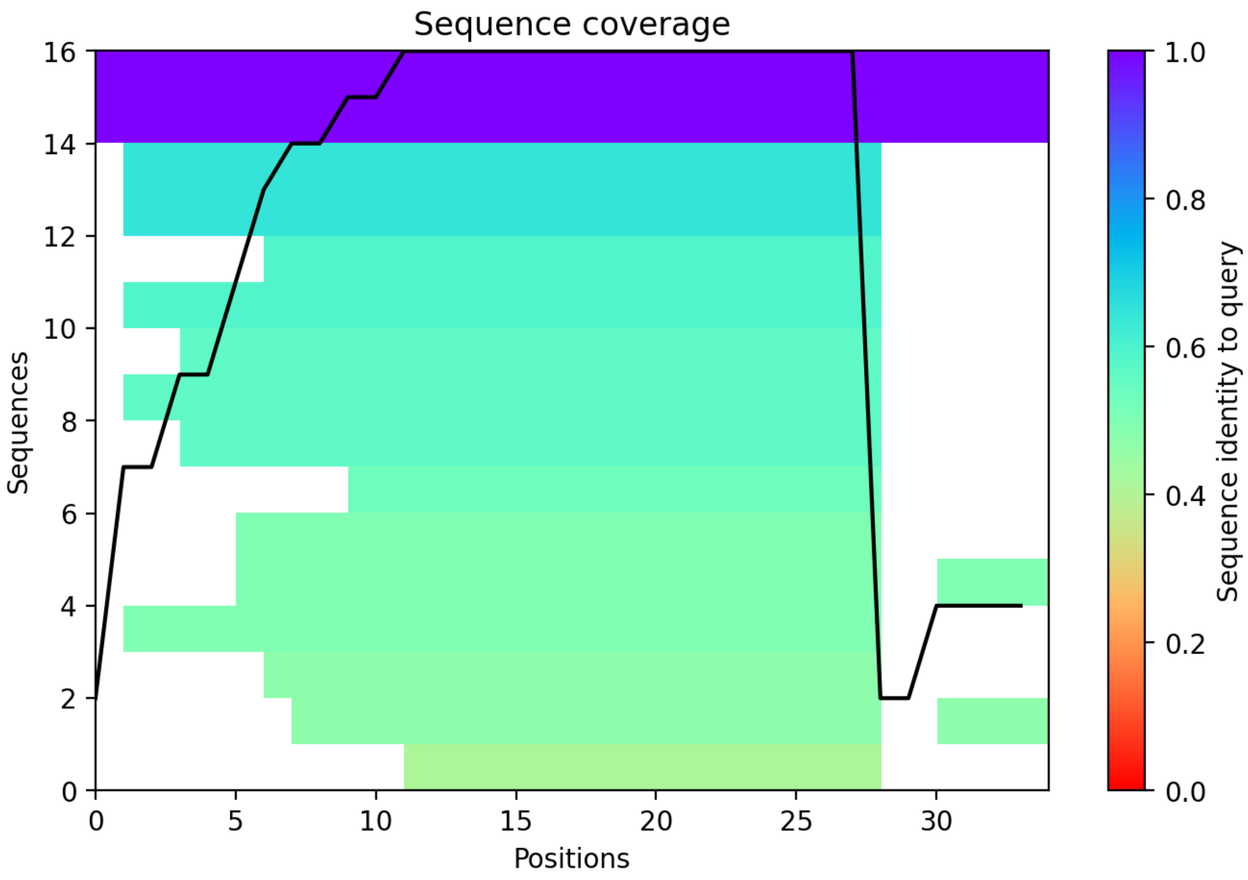

CP147: MtrunA17_CPg0493401

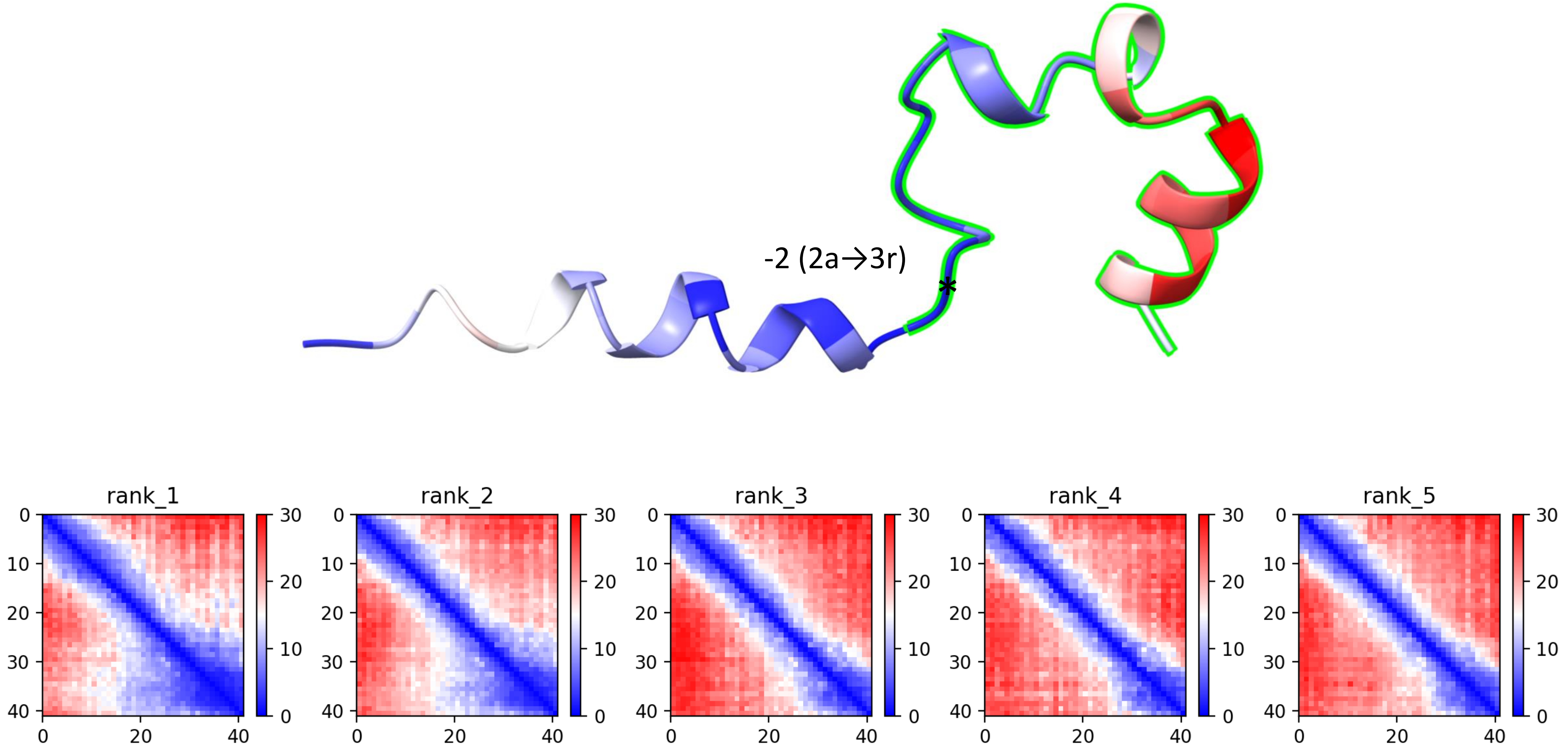

CP147: MtrunA17_CPg0493401

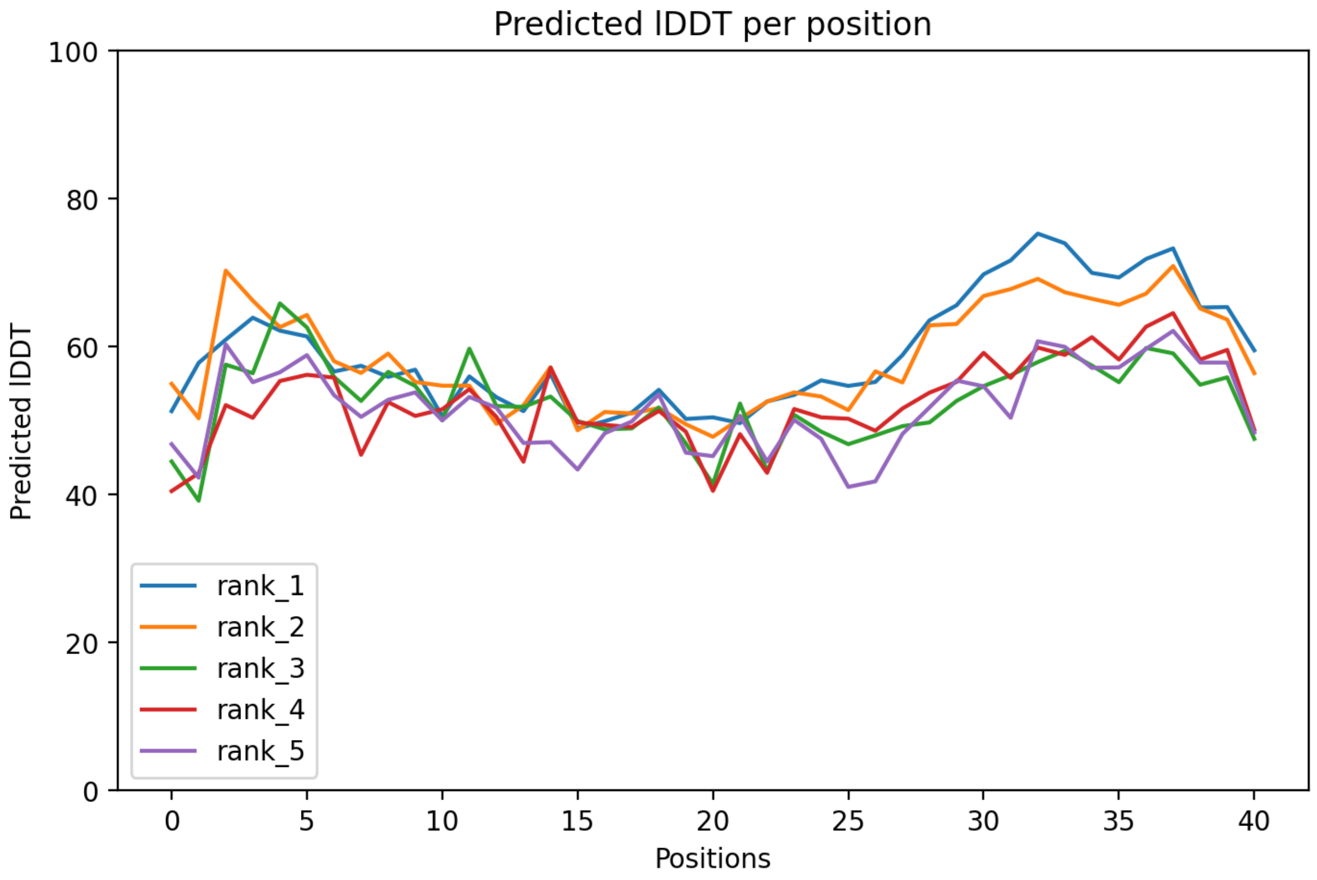

CP147: MtrunA17_CPg0493401

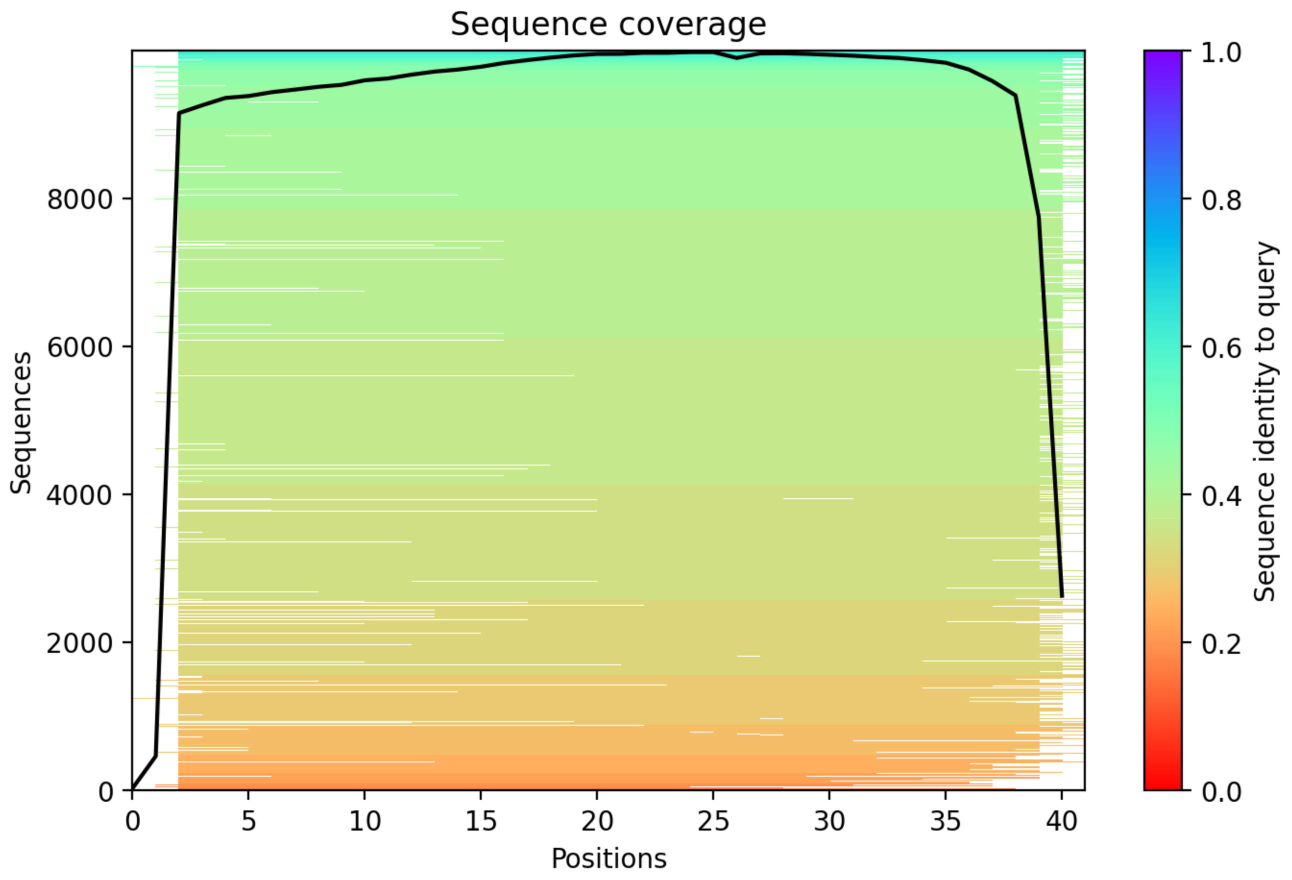

CP148: MtrunA17_MTg0490471

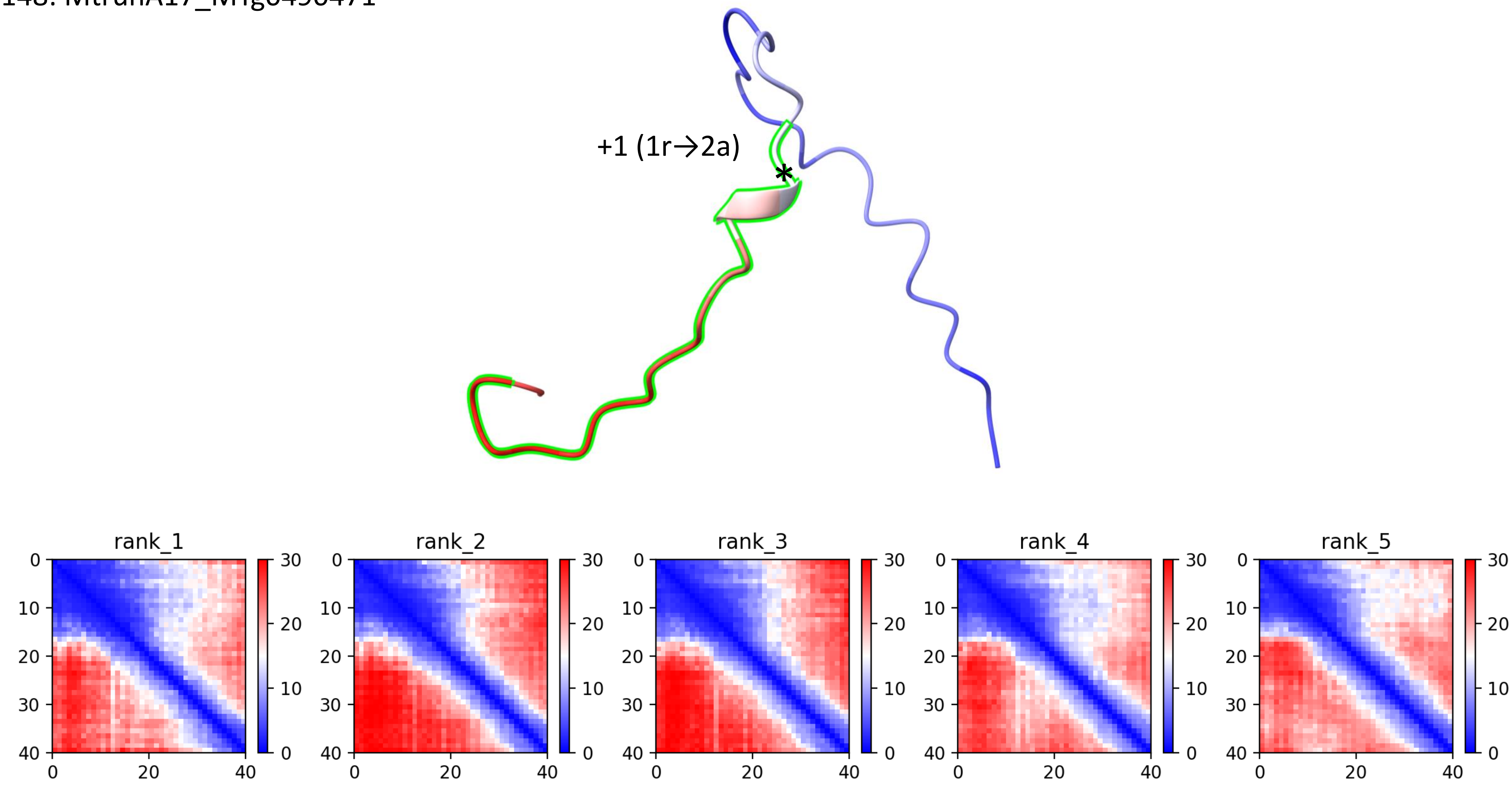

CP148: MtrunA17_MTg0490471

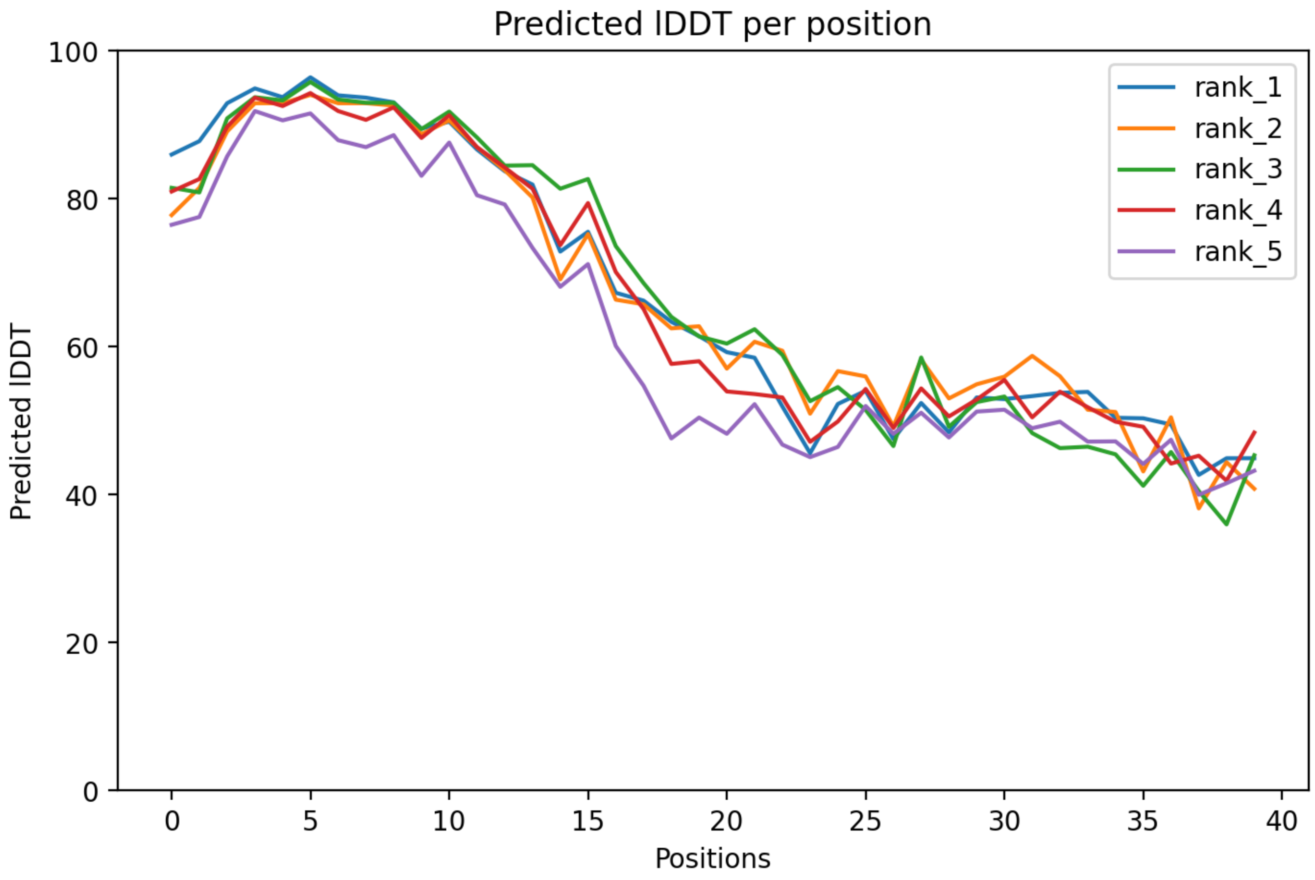

CP148: MtrunA17_MTg0490471

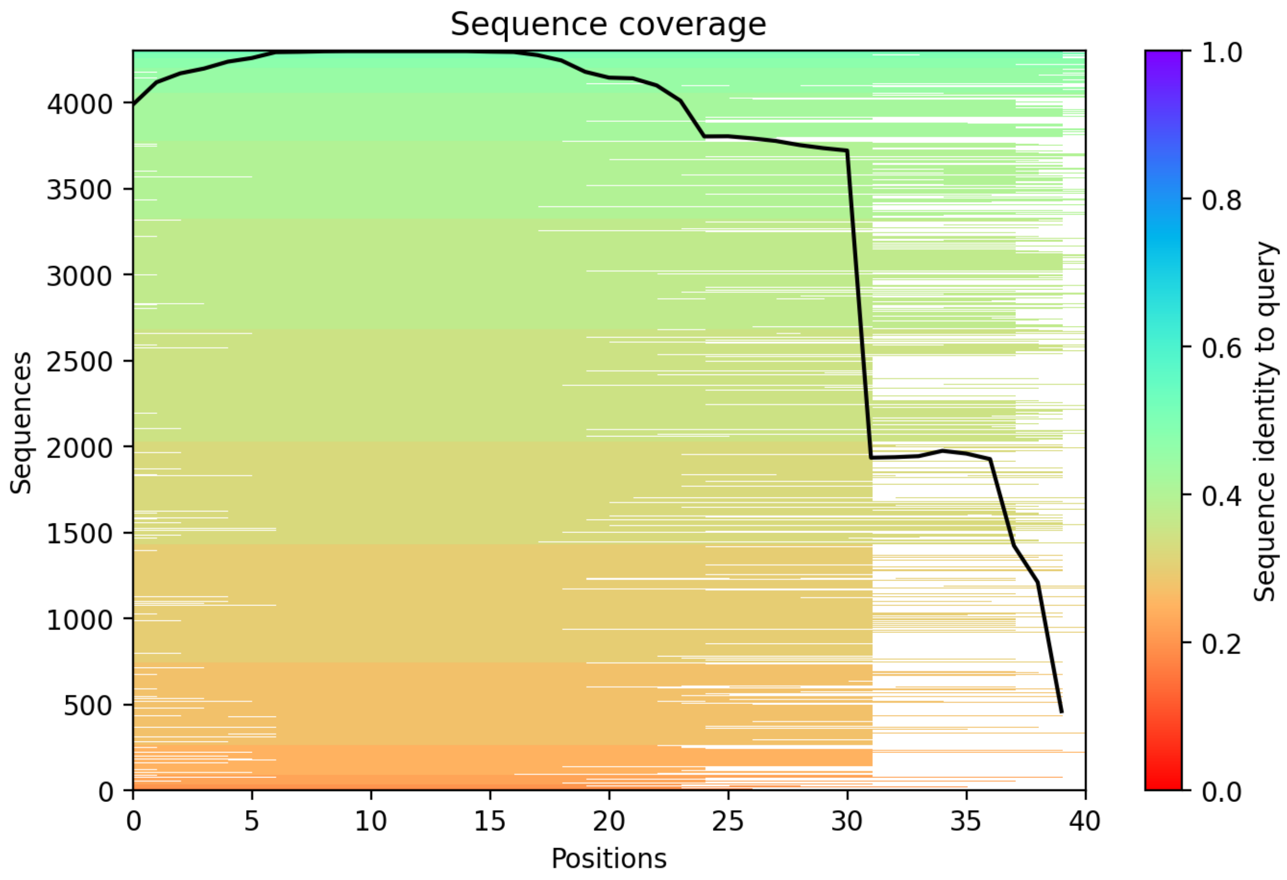

CP149: MtrunA17_MTg0490471

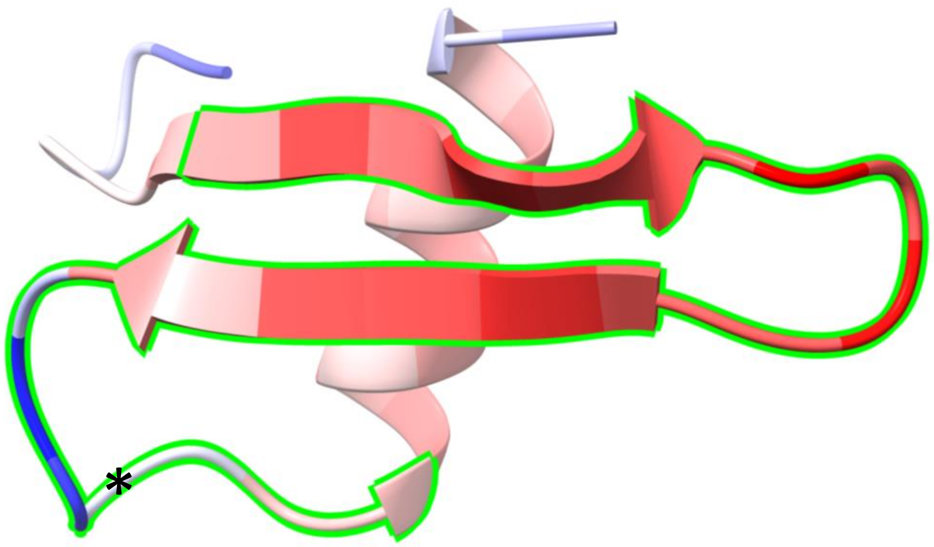

+1 (1r→2a)

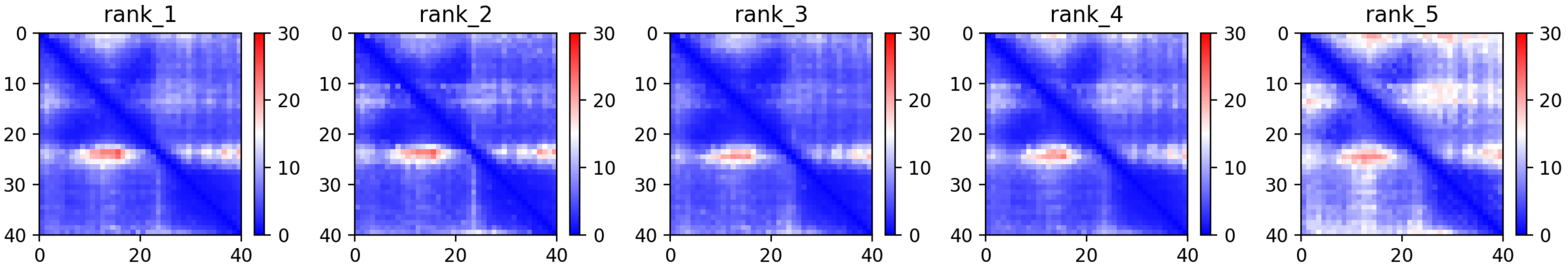

CP149: MtrunA17_MTg0490471

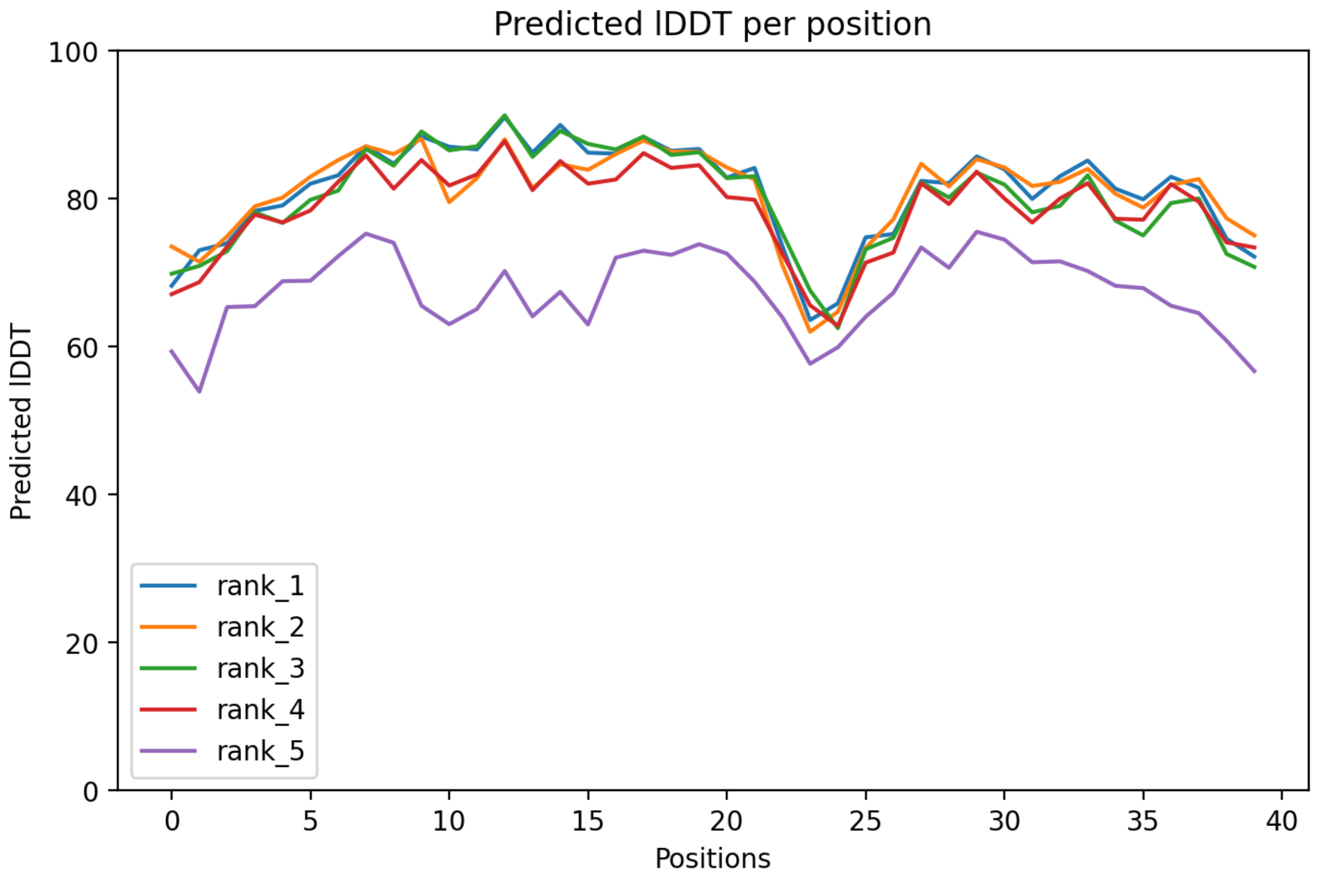

CP149: MtrunA17_MTg0490471

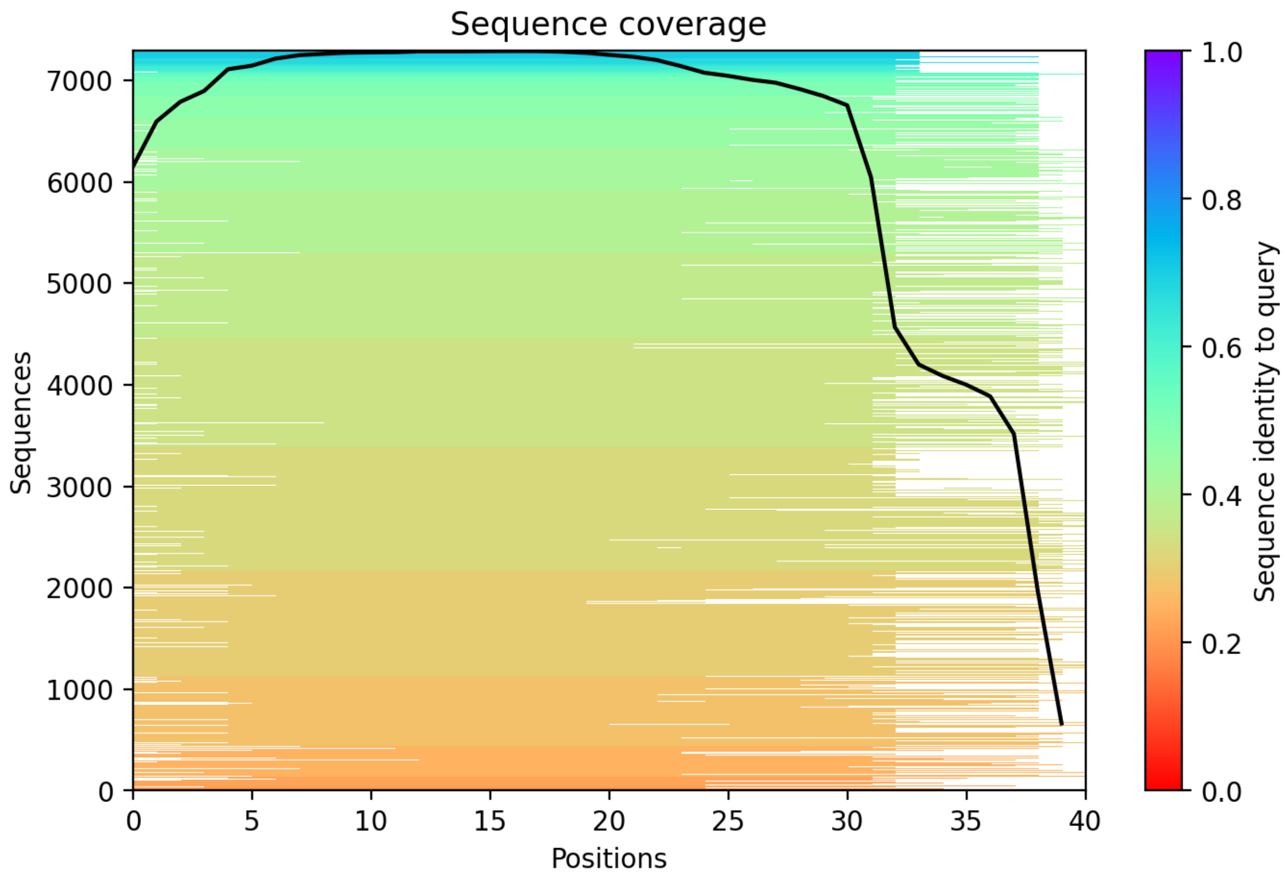

CP150: MtrunA17_MTg0490971

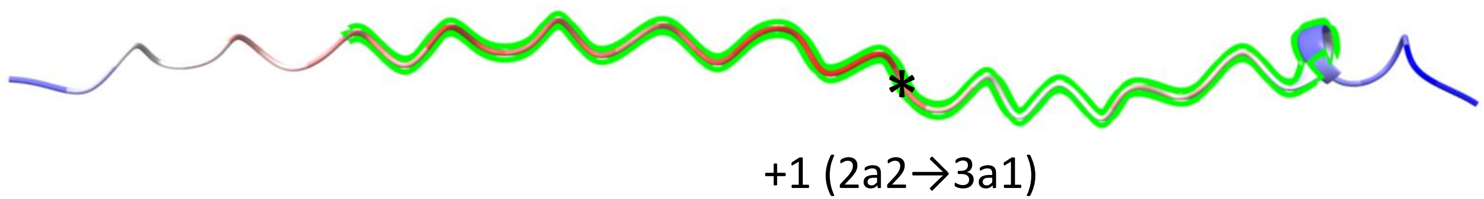

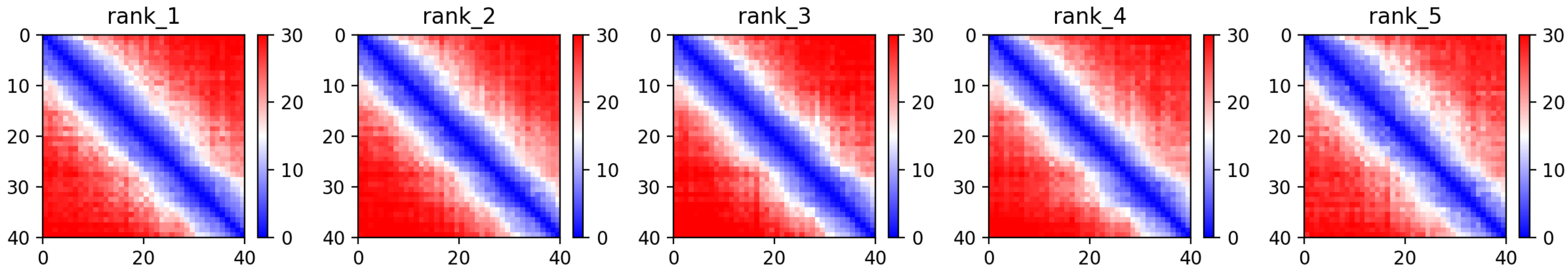

CP150: MtrunA17_MTg0490971

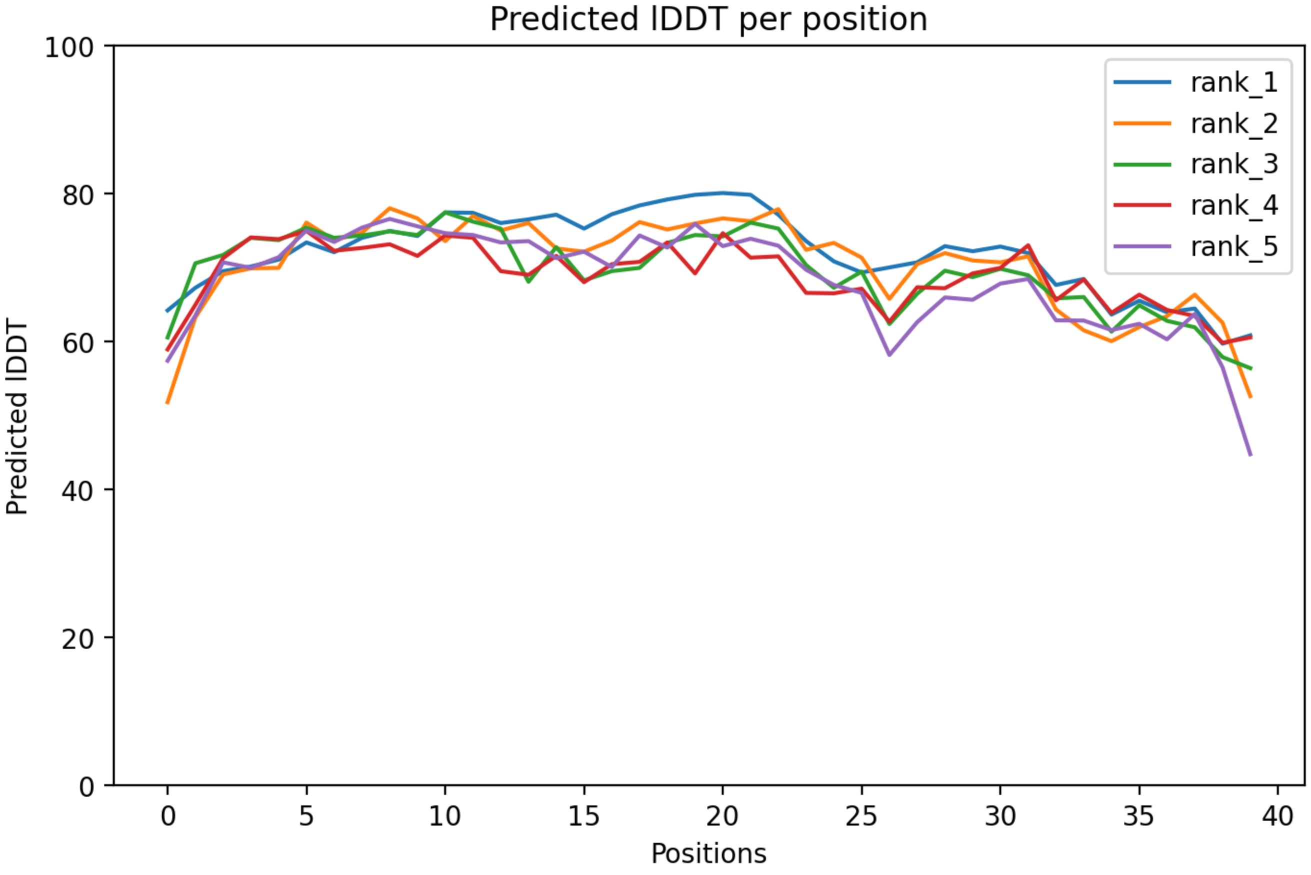

CP150: MtrunA17_MTg0490971

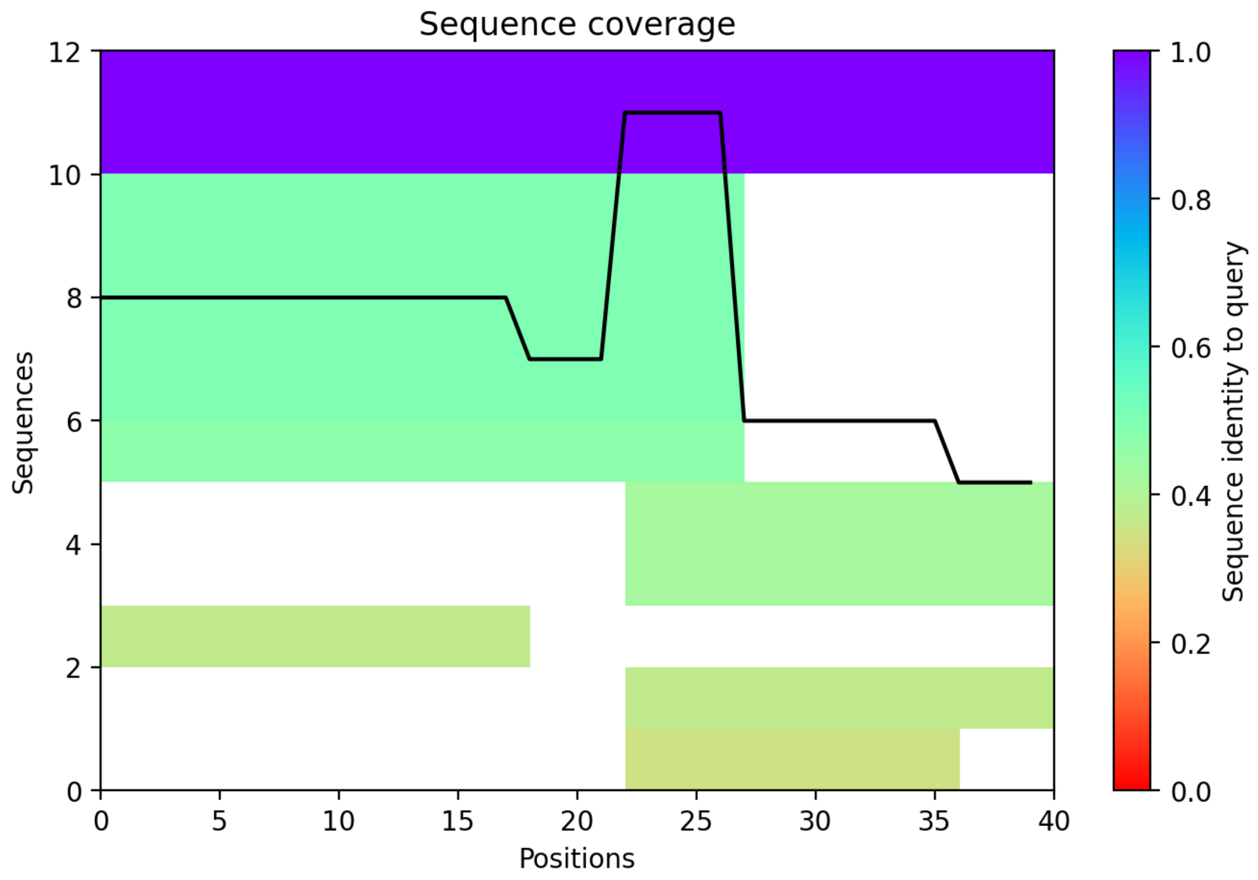

CP151: MtrunA17_MTg0490971

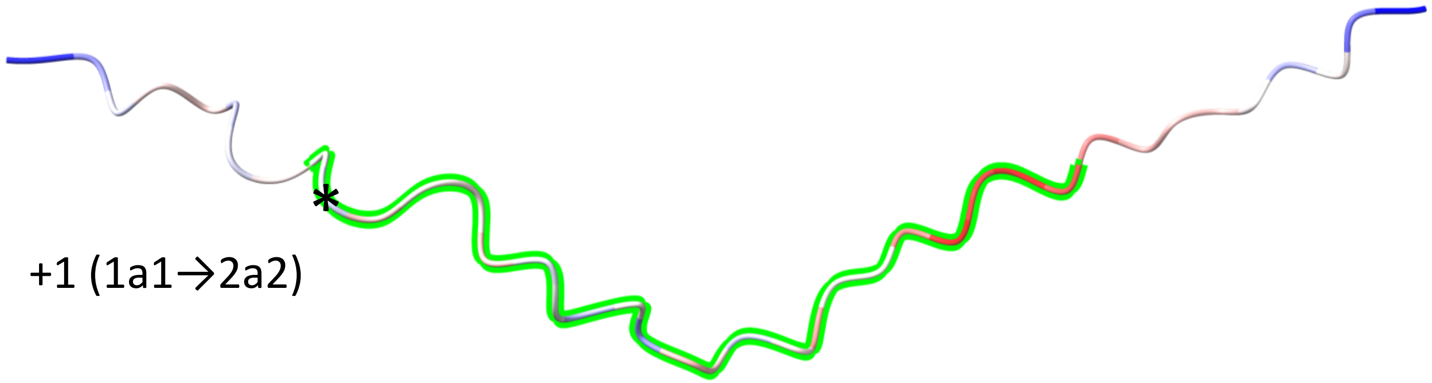

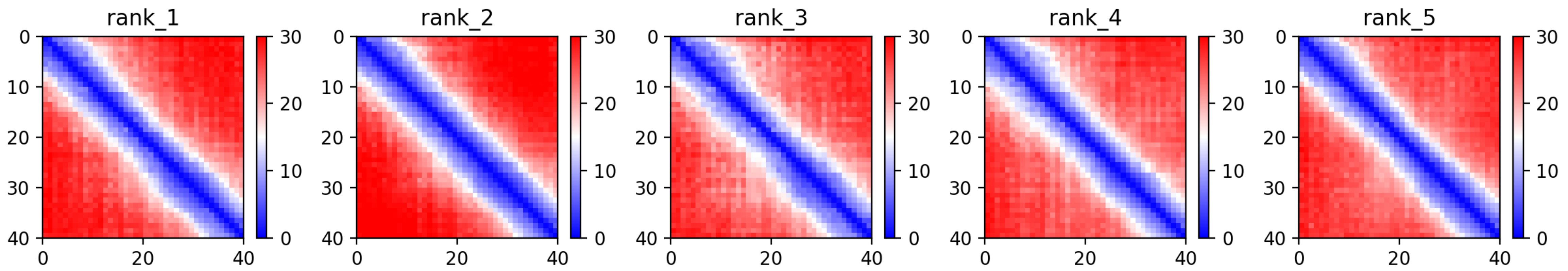

CP151: MtrunA17_MTg0490971

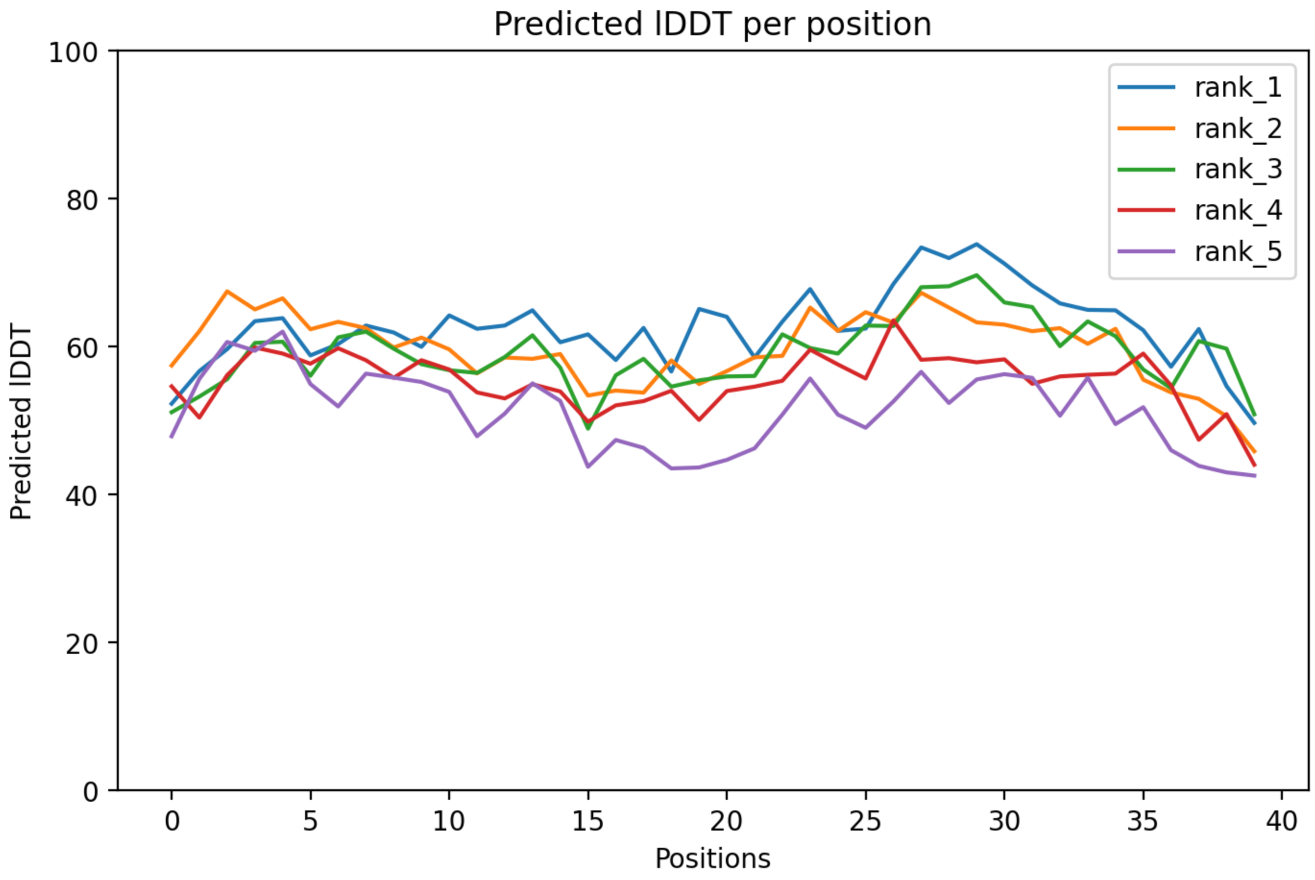

CP151: MtrunA17_MTg0490971

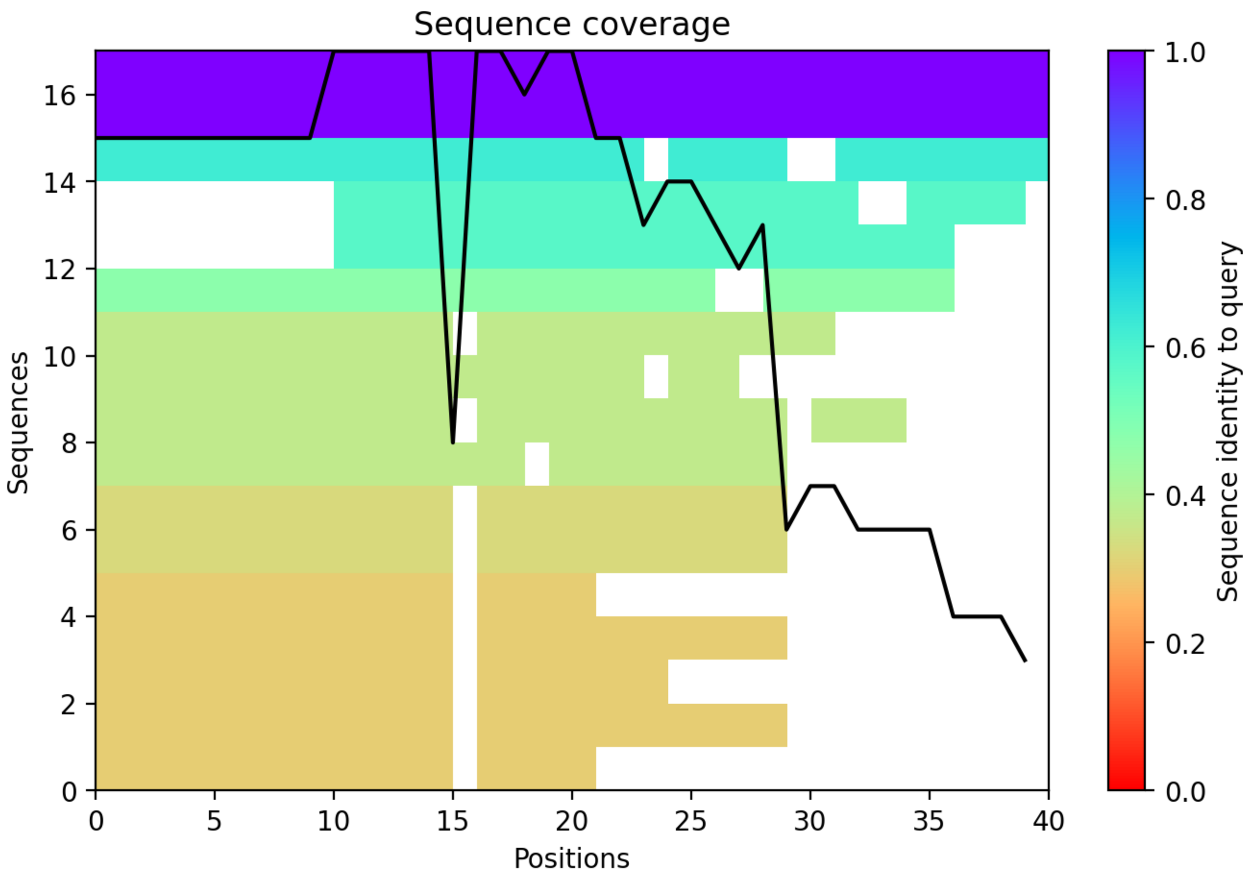

CP152: MtrunA17_MTg0491151

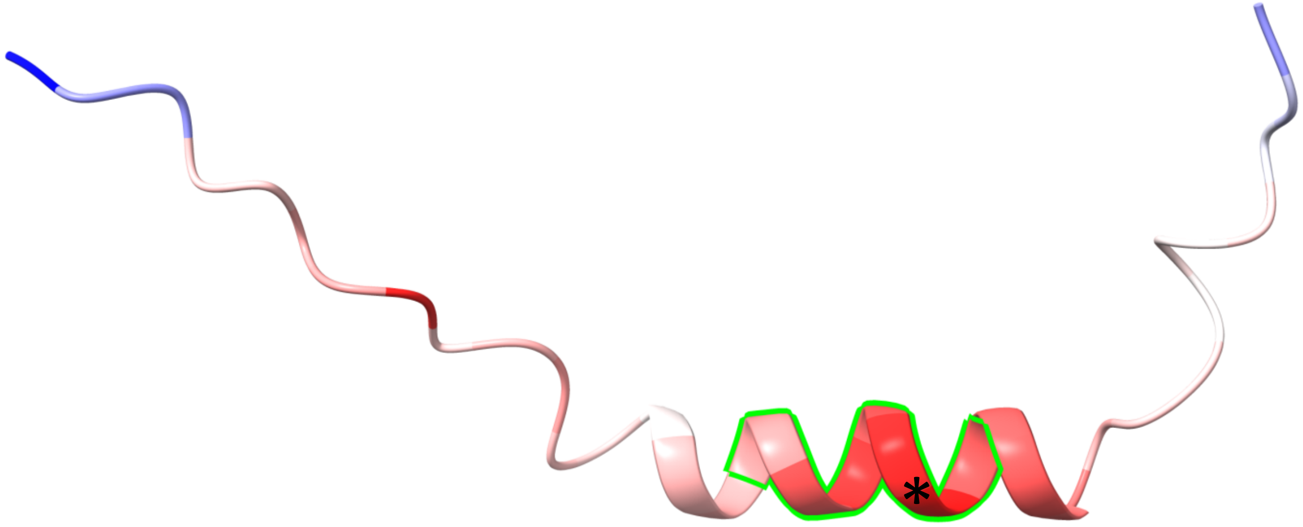

+2 (2a1→1a2)

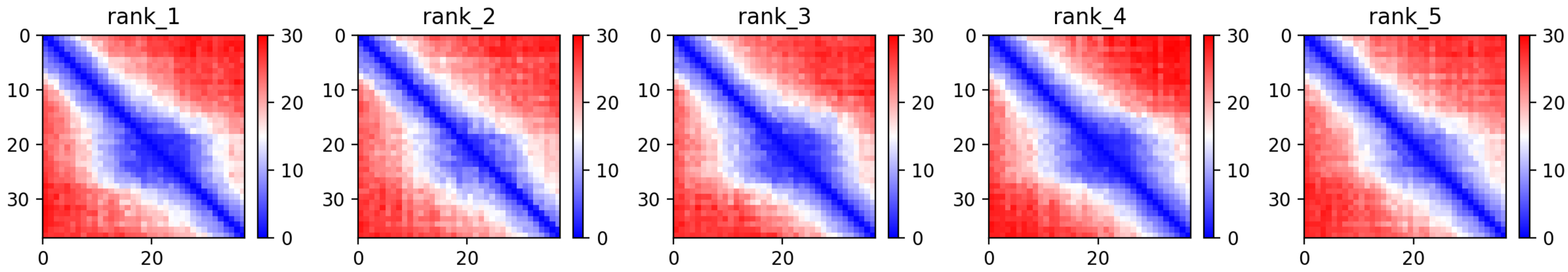

CP152: MtrunA17_MTg0491151

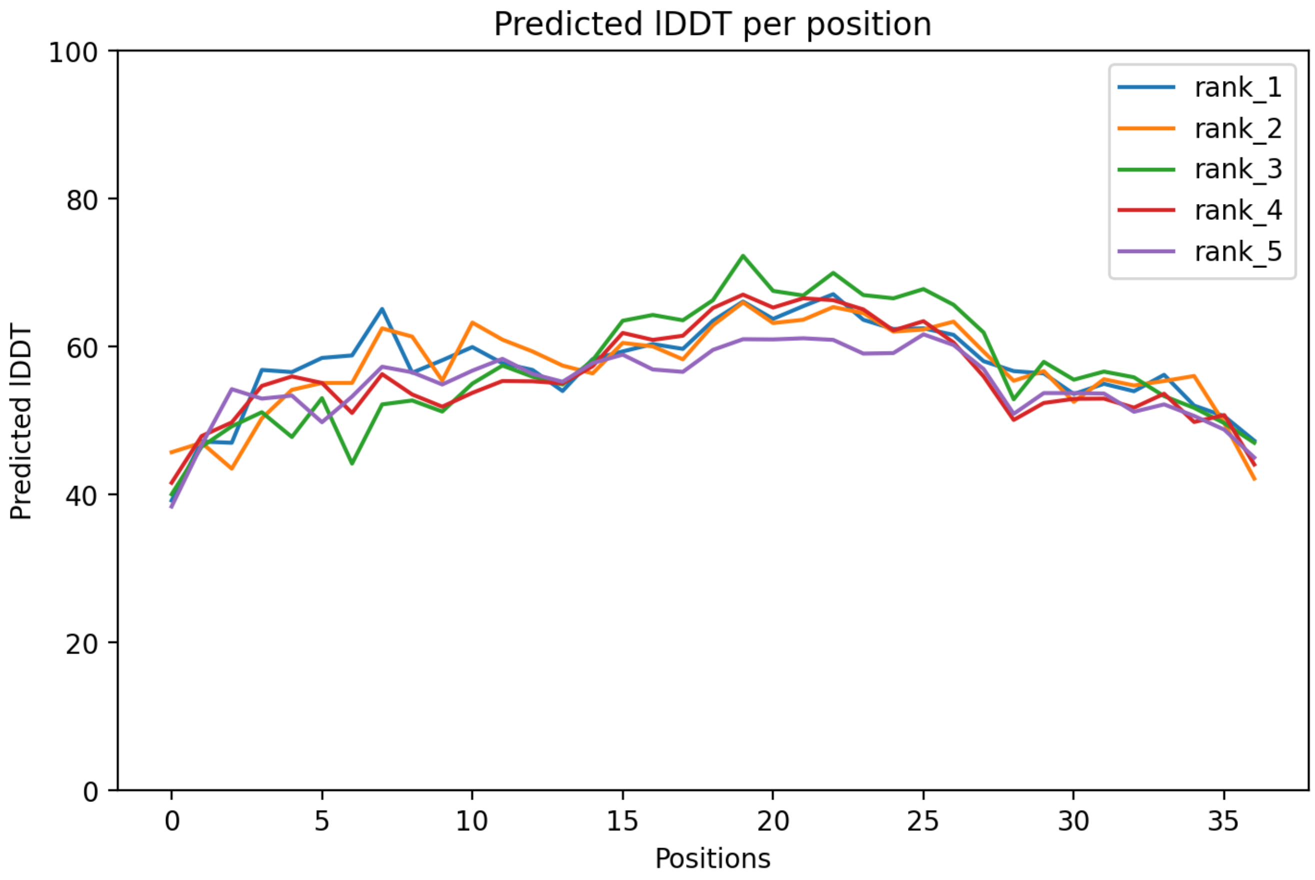

CP152: MtrunA17_MTg0491151

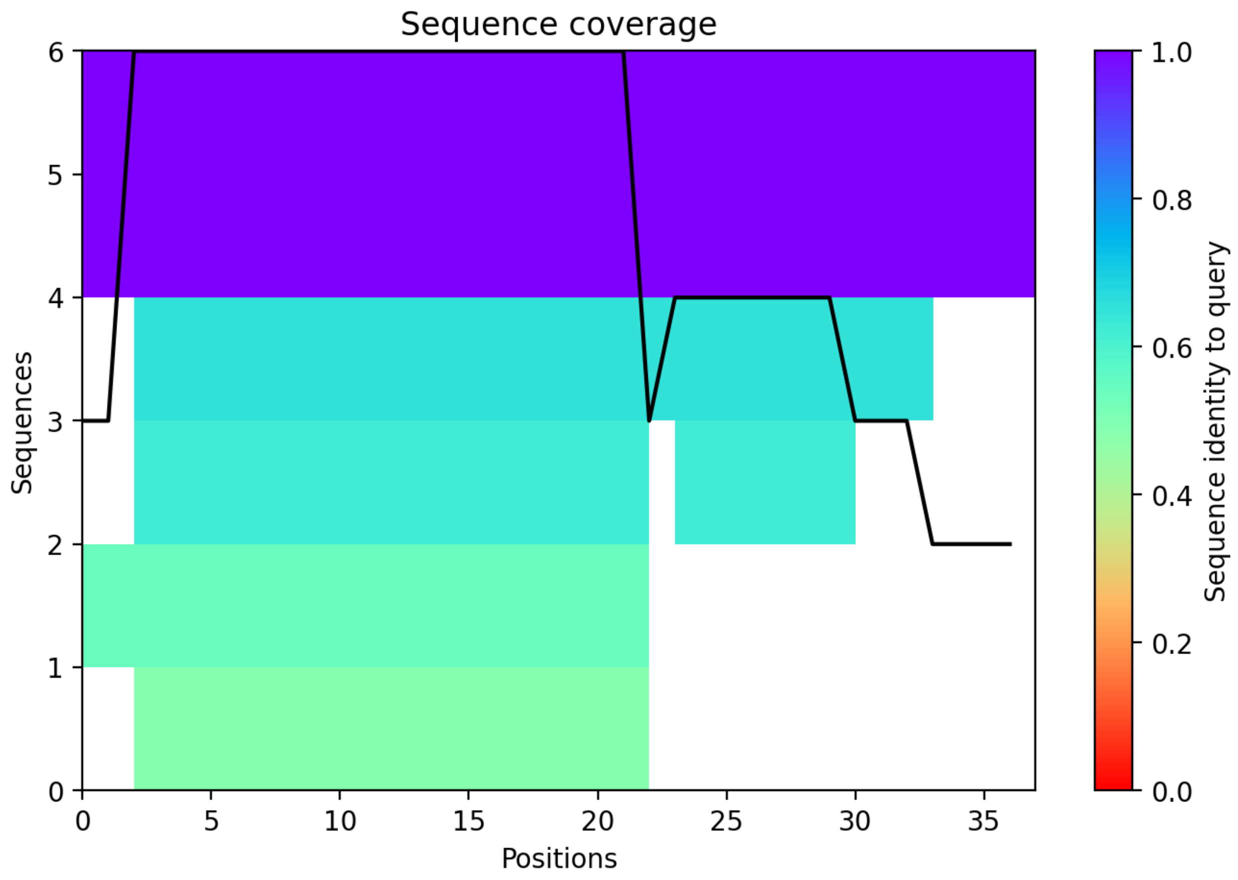

CP153: MtrunA17_MTg0491291

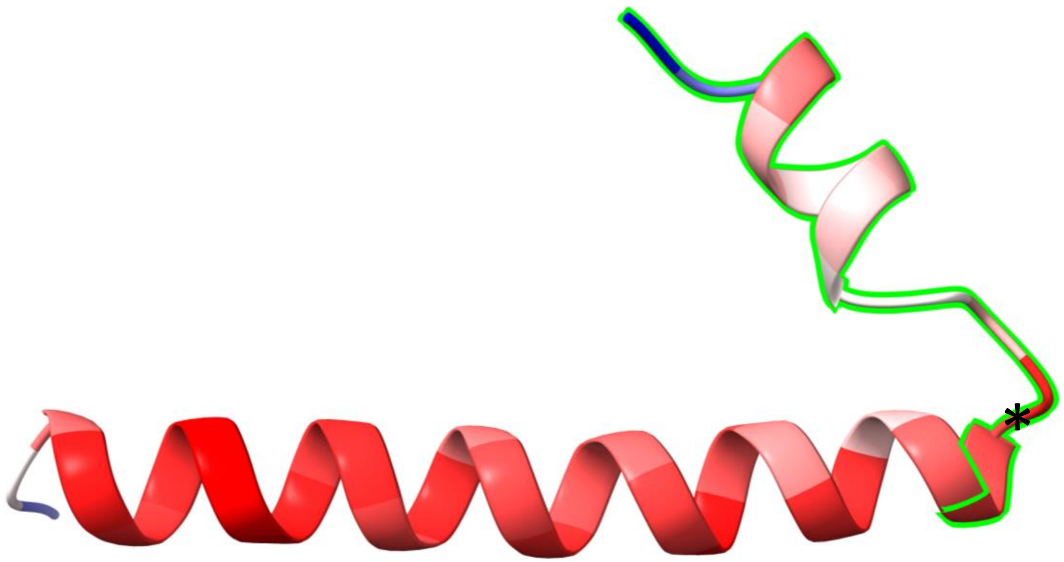

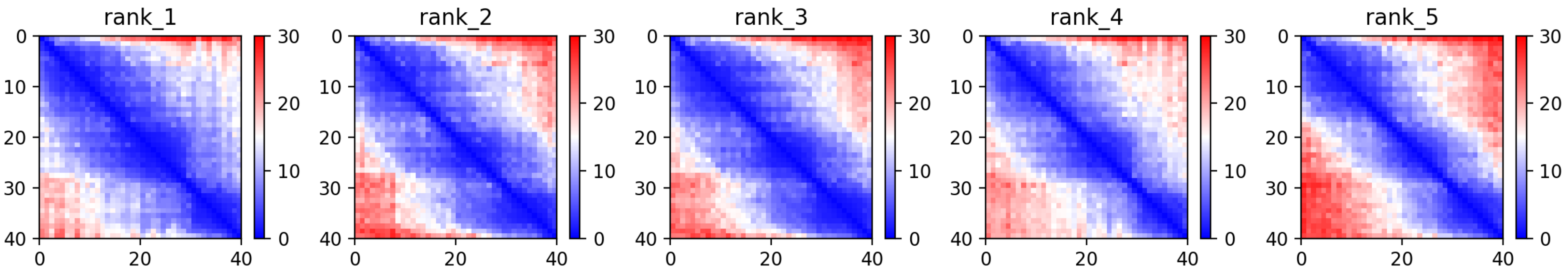

CP153: MtrunA17_MTg0491291

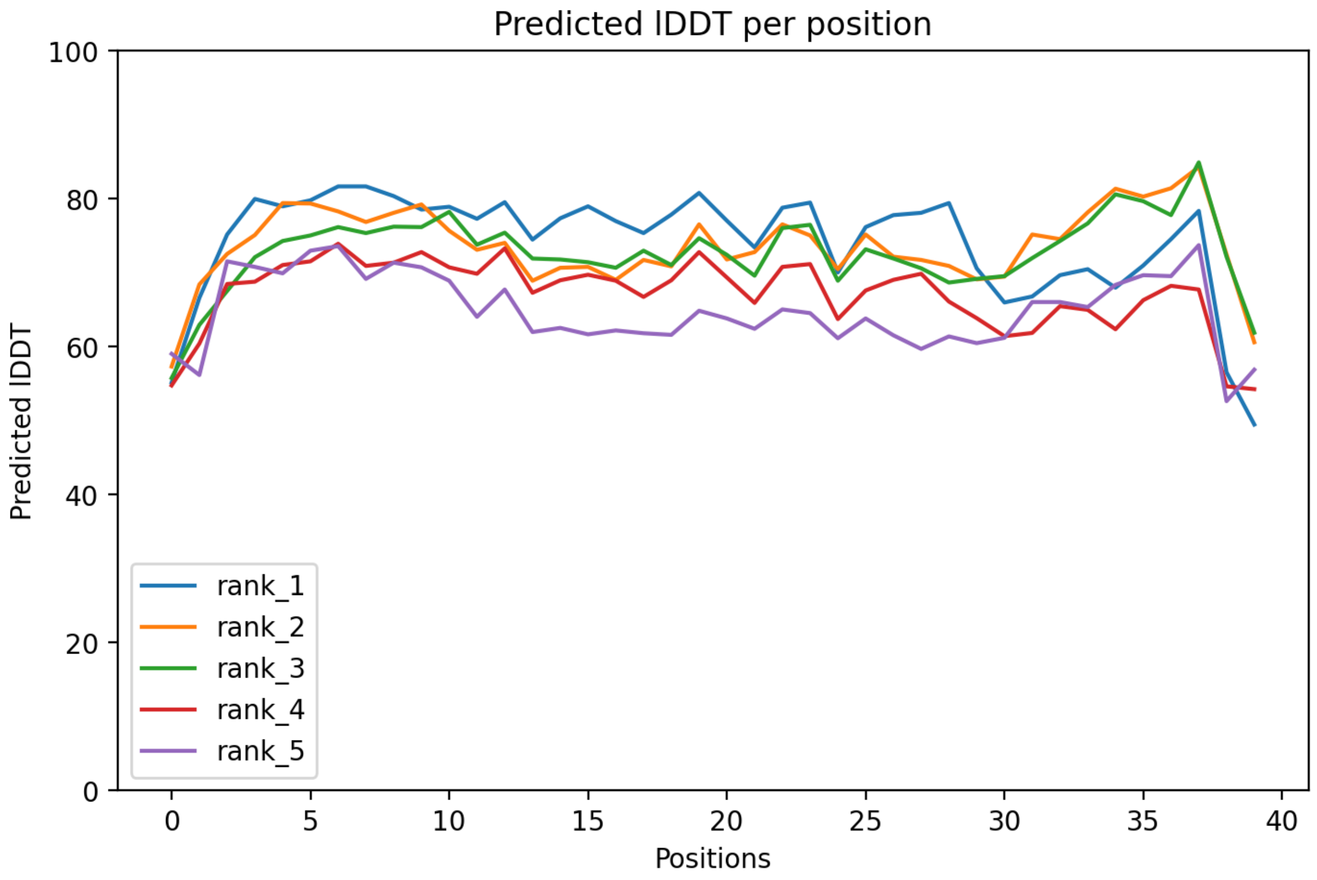

CP153: MtrunA17_MTg0491291

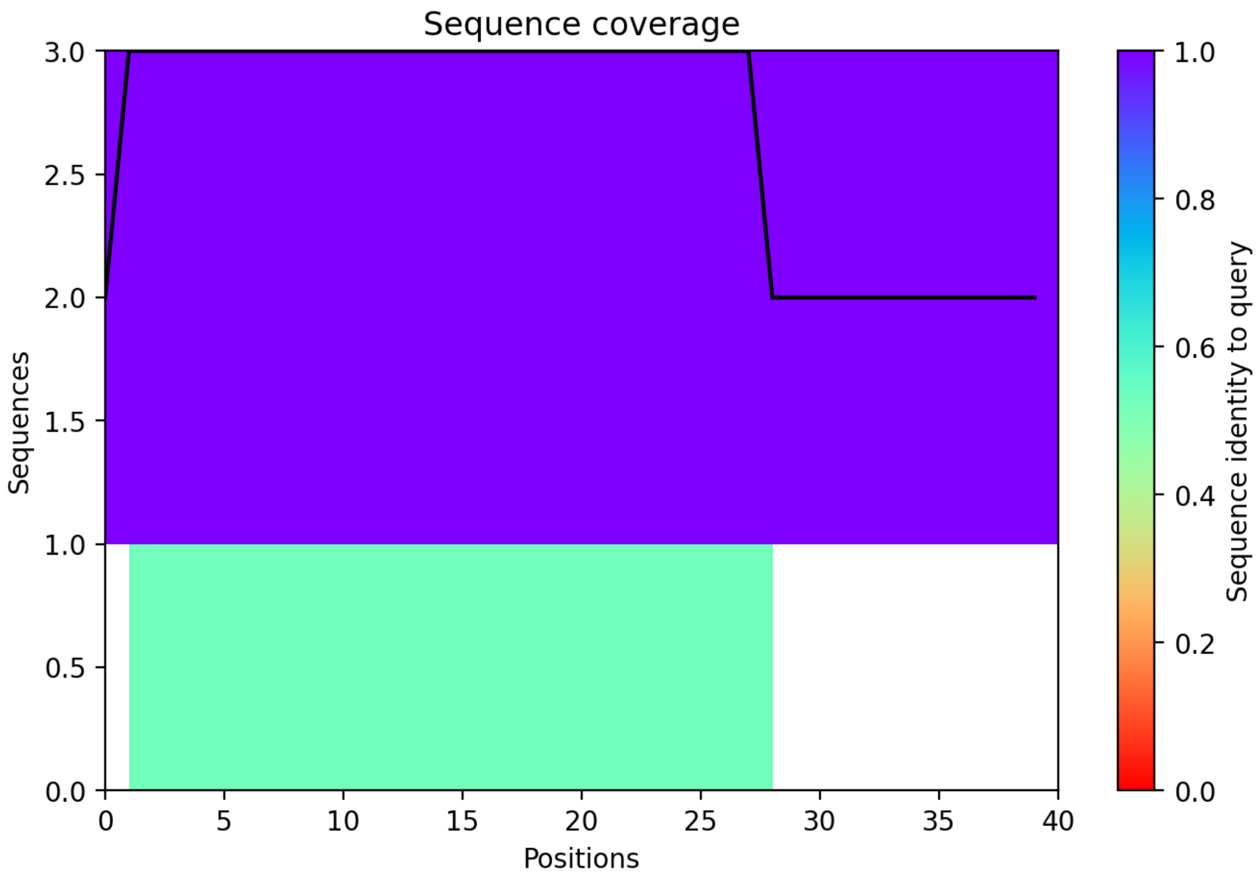

CP154: MtrunA17_MTg0491501

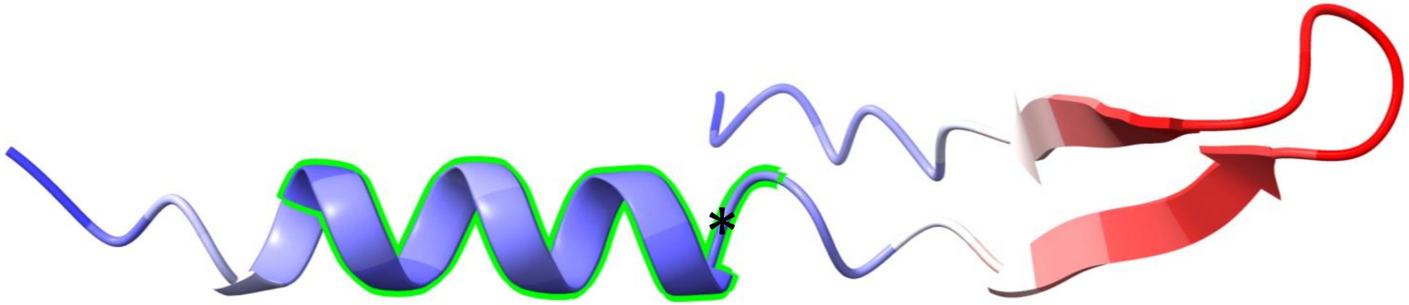

-2 (3a1→1a2)

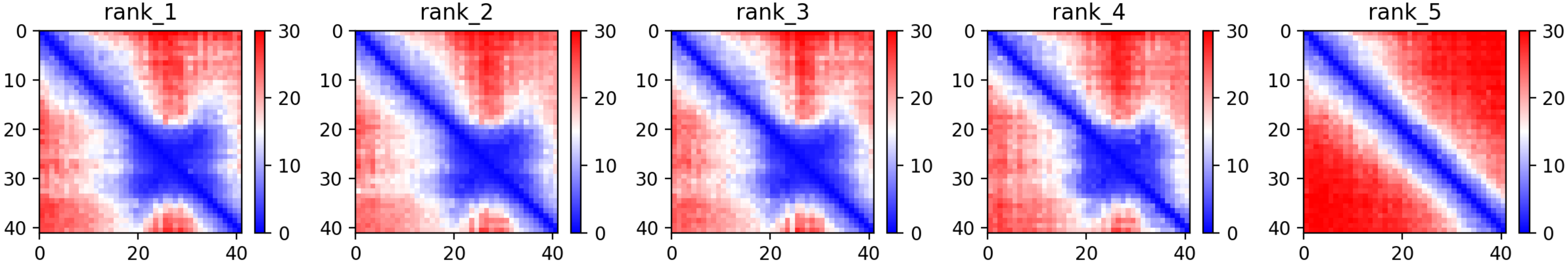

CP154: MtrunA17_MTg0491501

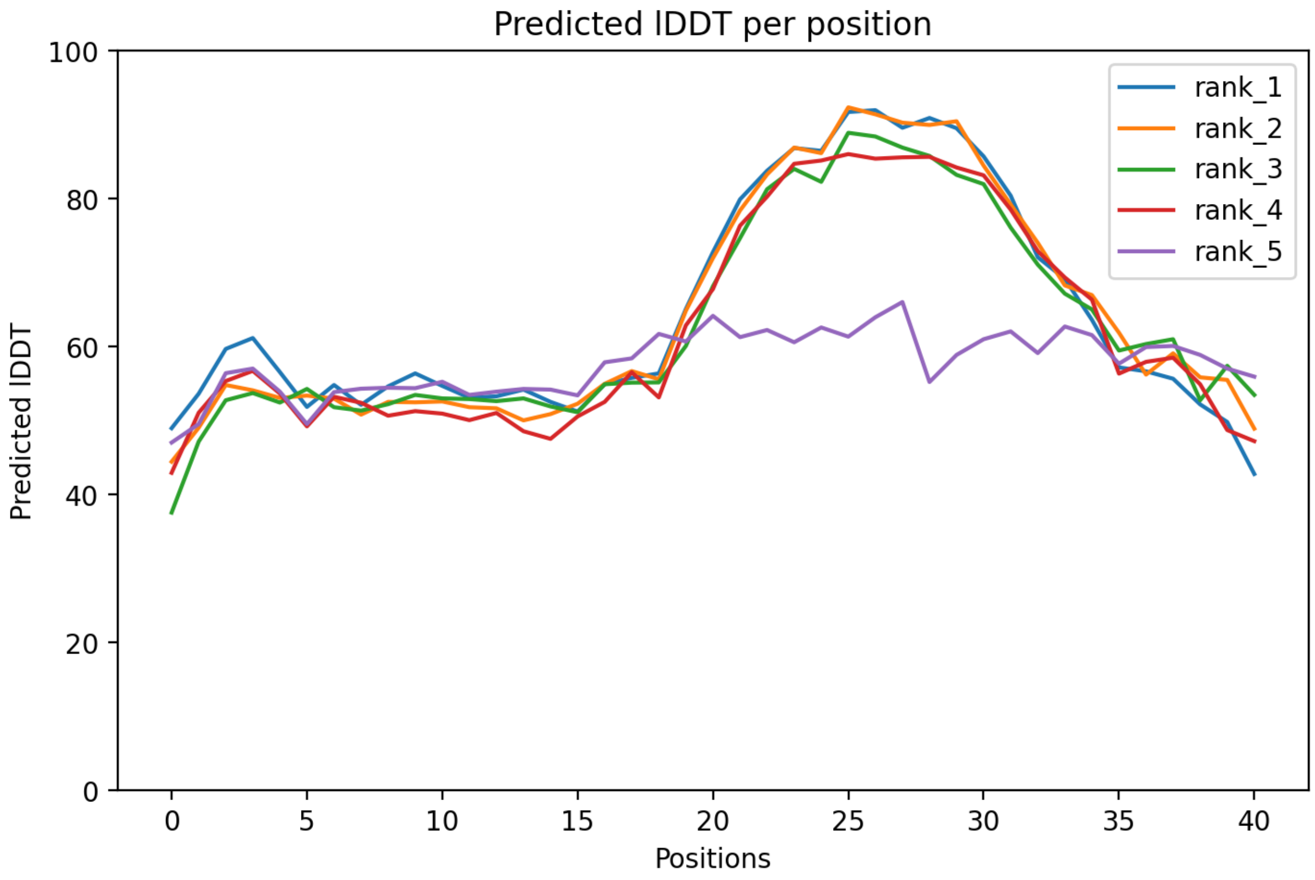

CP154: MtrunA17_MTg0491501

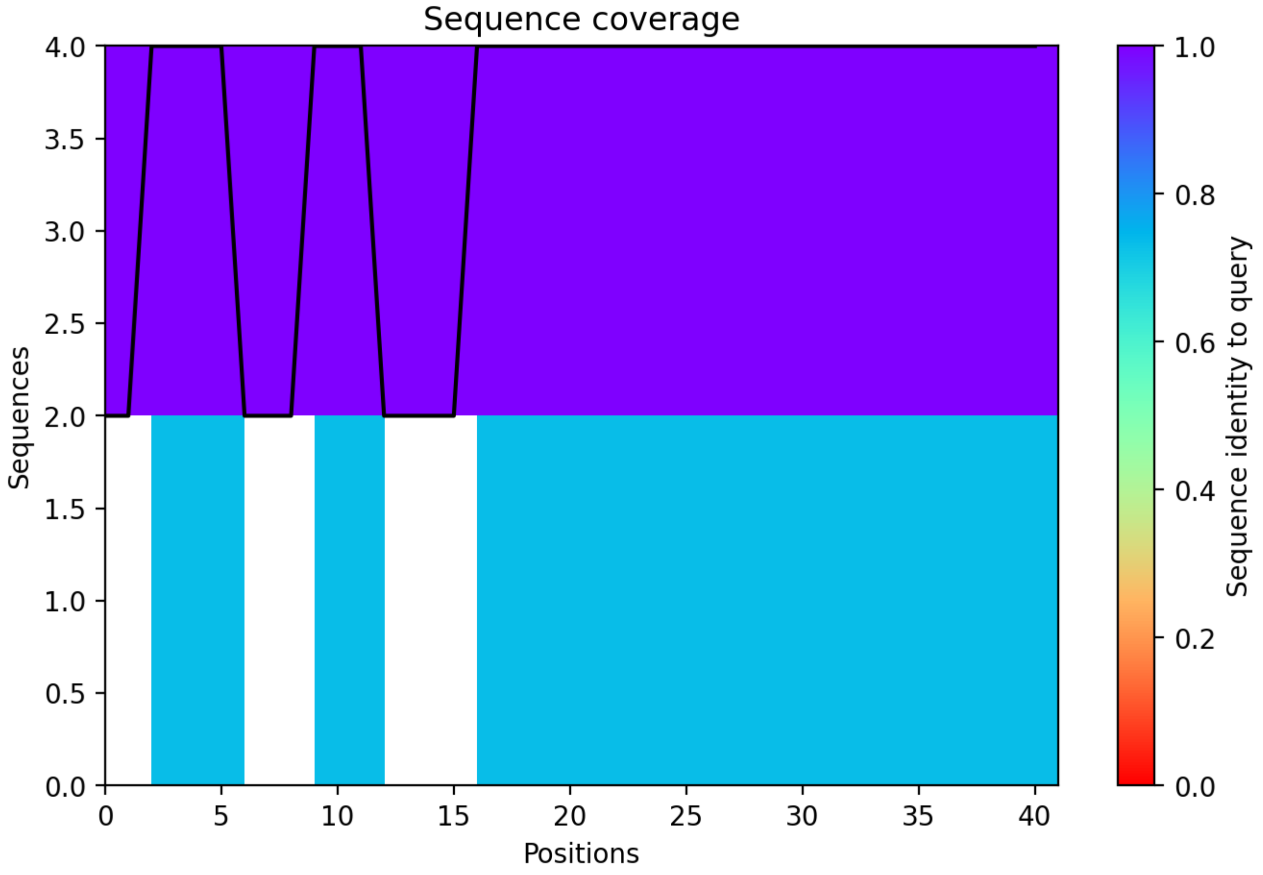

CP155: MtrunA17_MTg0491621

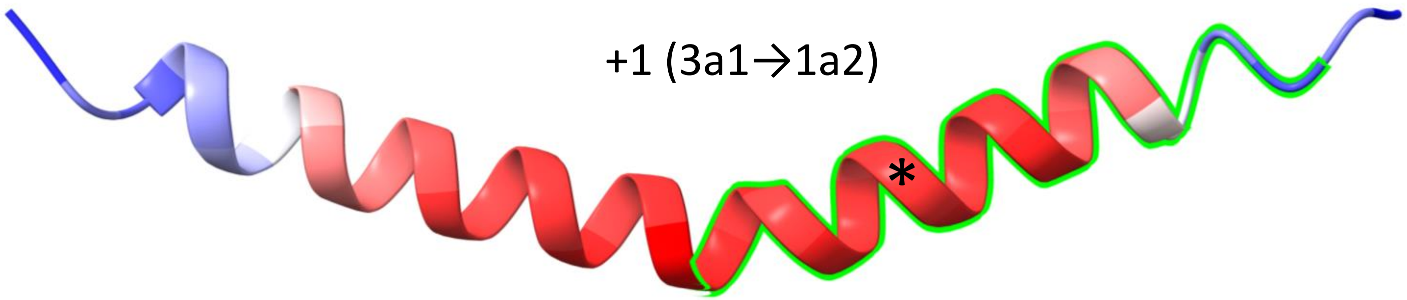

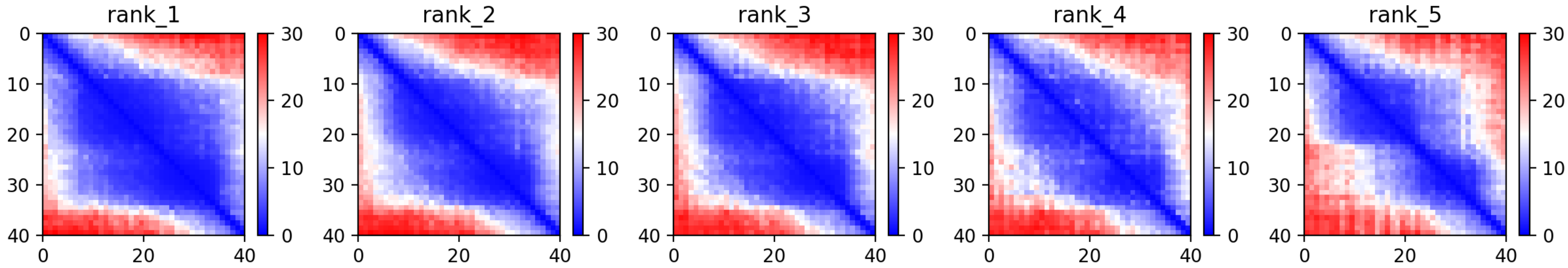

CP155: MtrunA17_MTg0491621

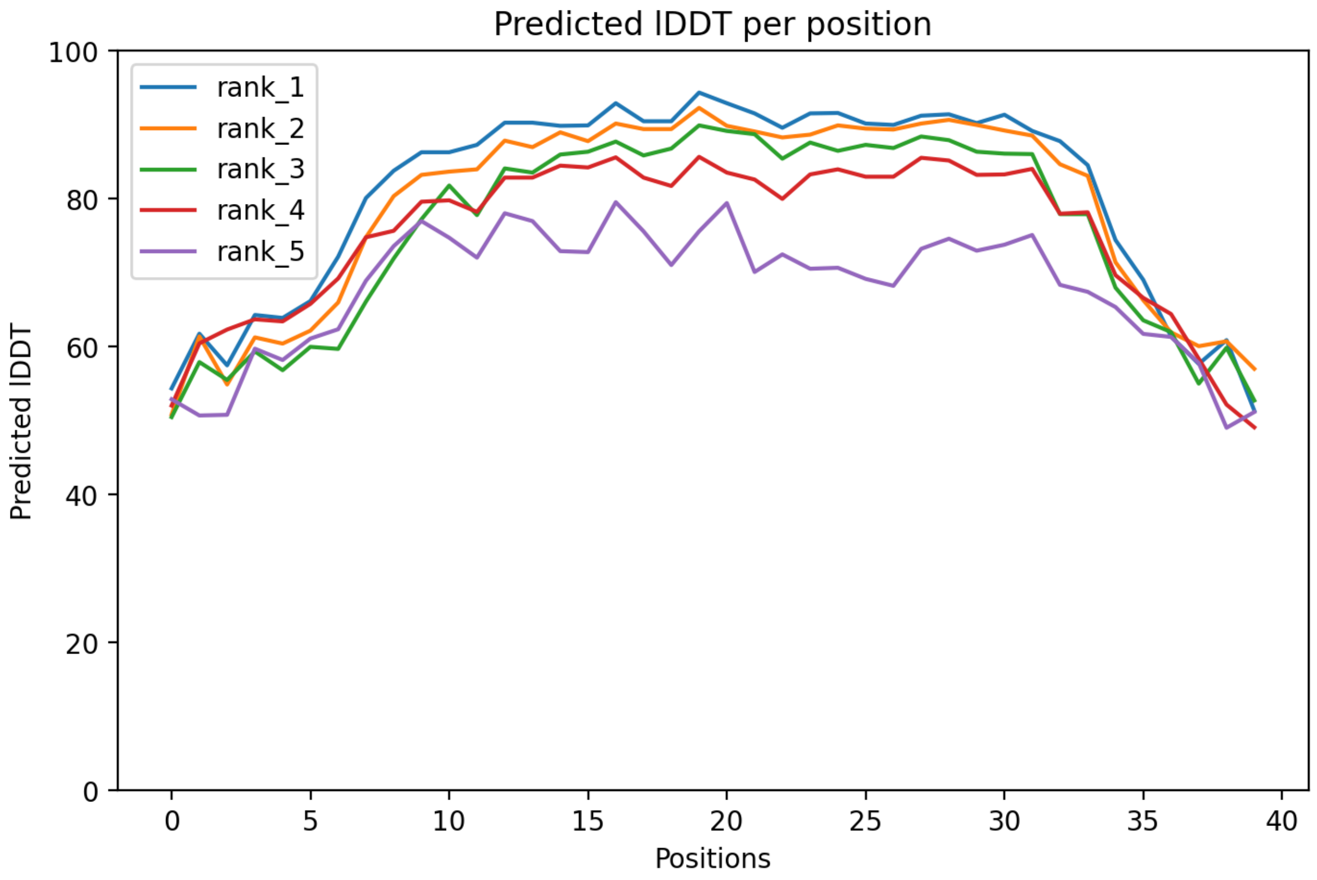

CP155: MtrunA17_MTg0491621

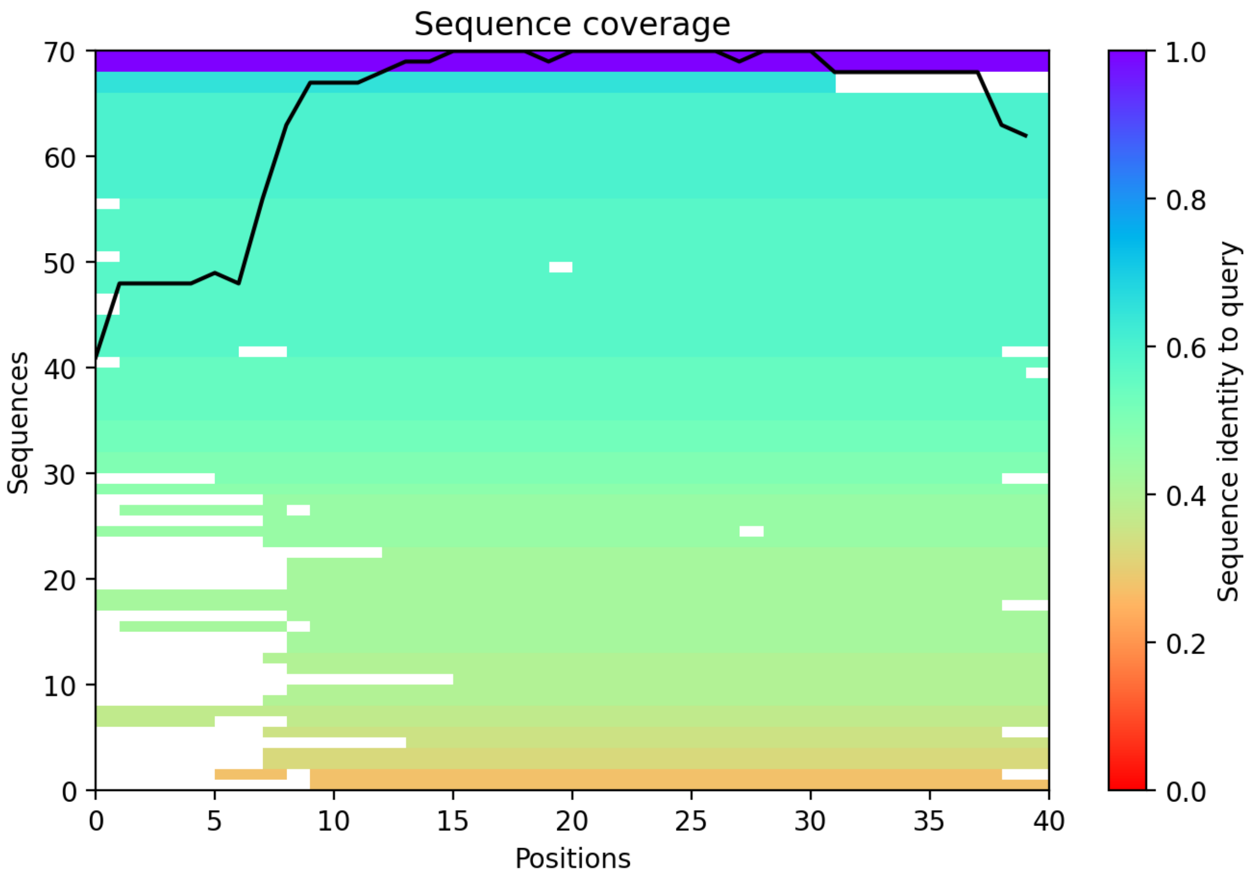

CP156: MtrunA17_MTg0491711

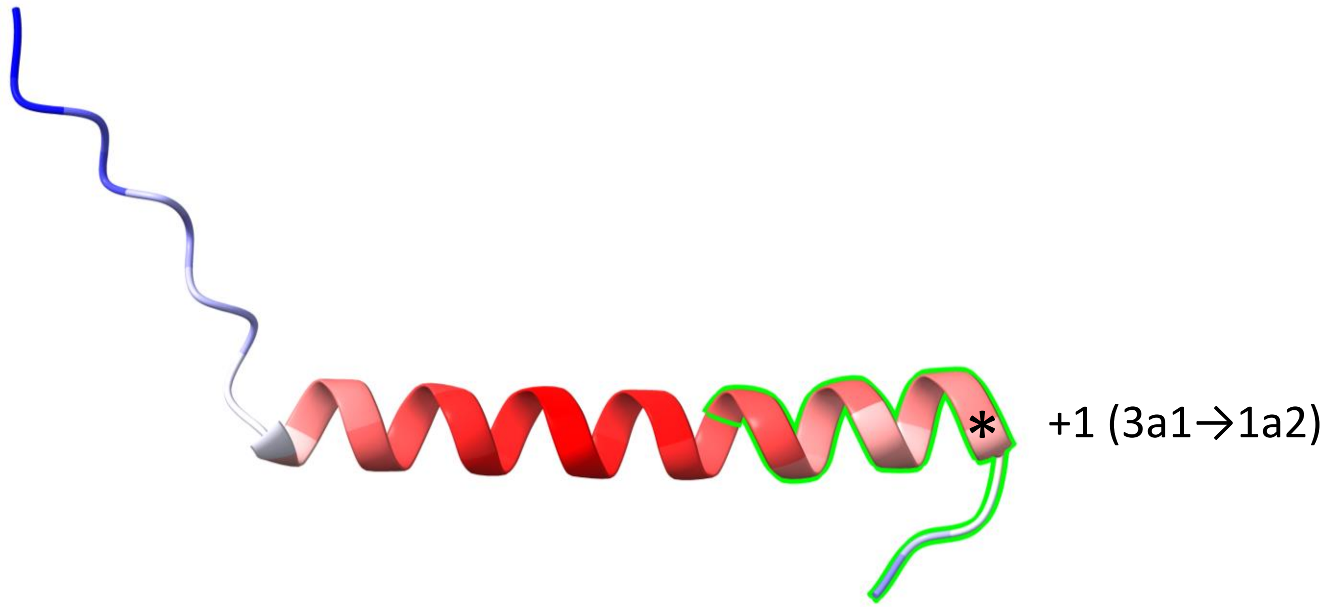

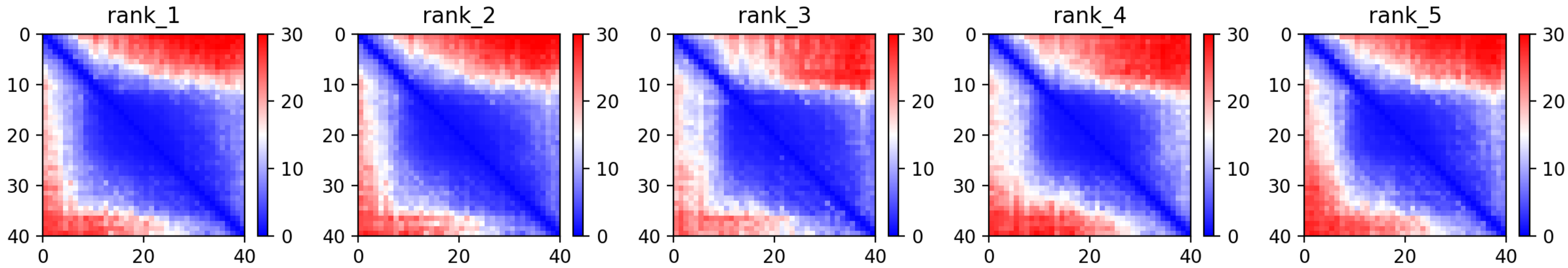

CP156: MtrunA17_MTg0491711

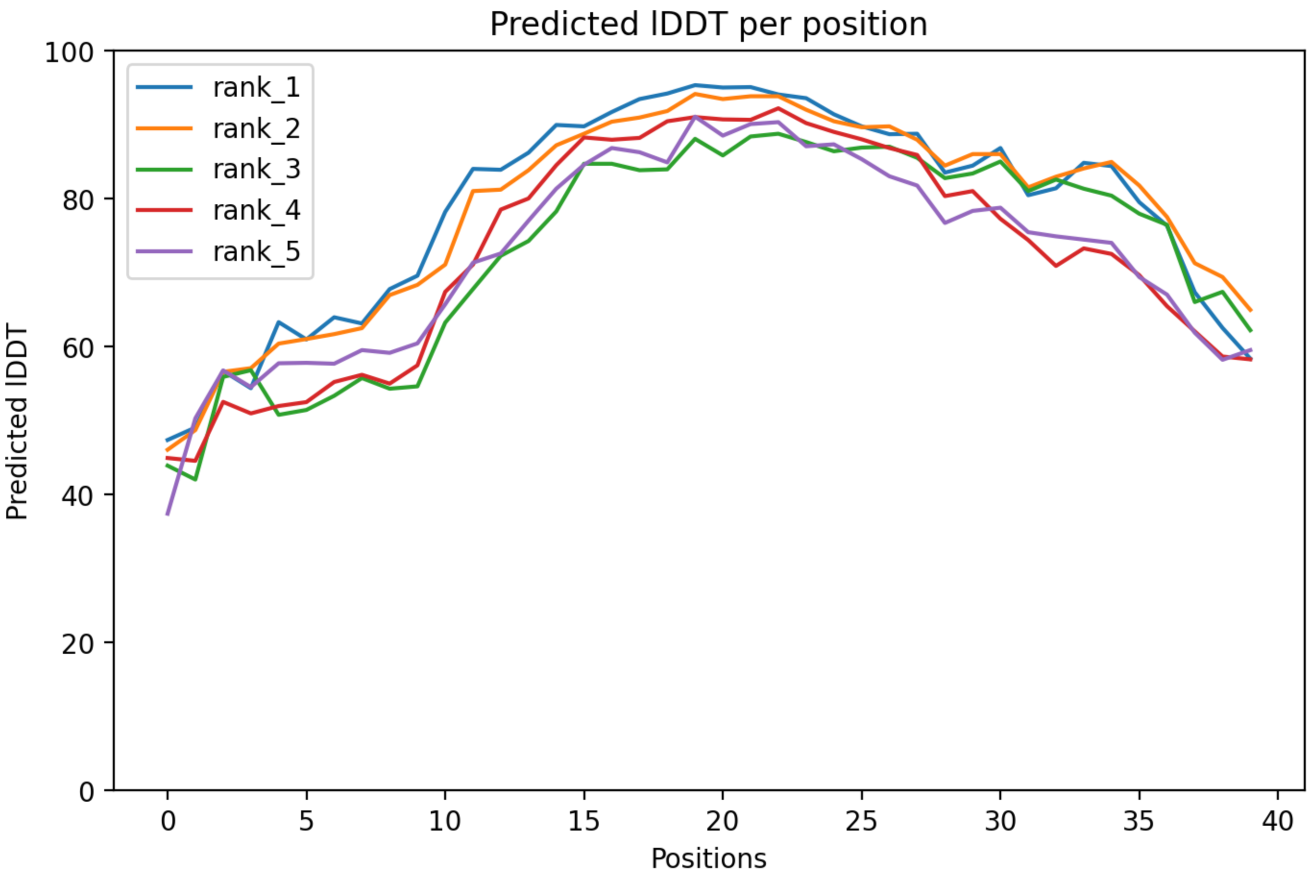

CP156: MtrunA17_MTg0491711

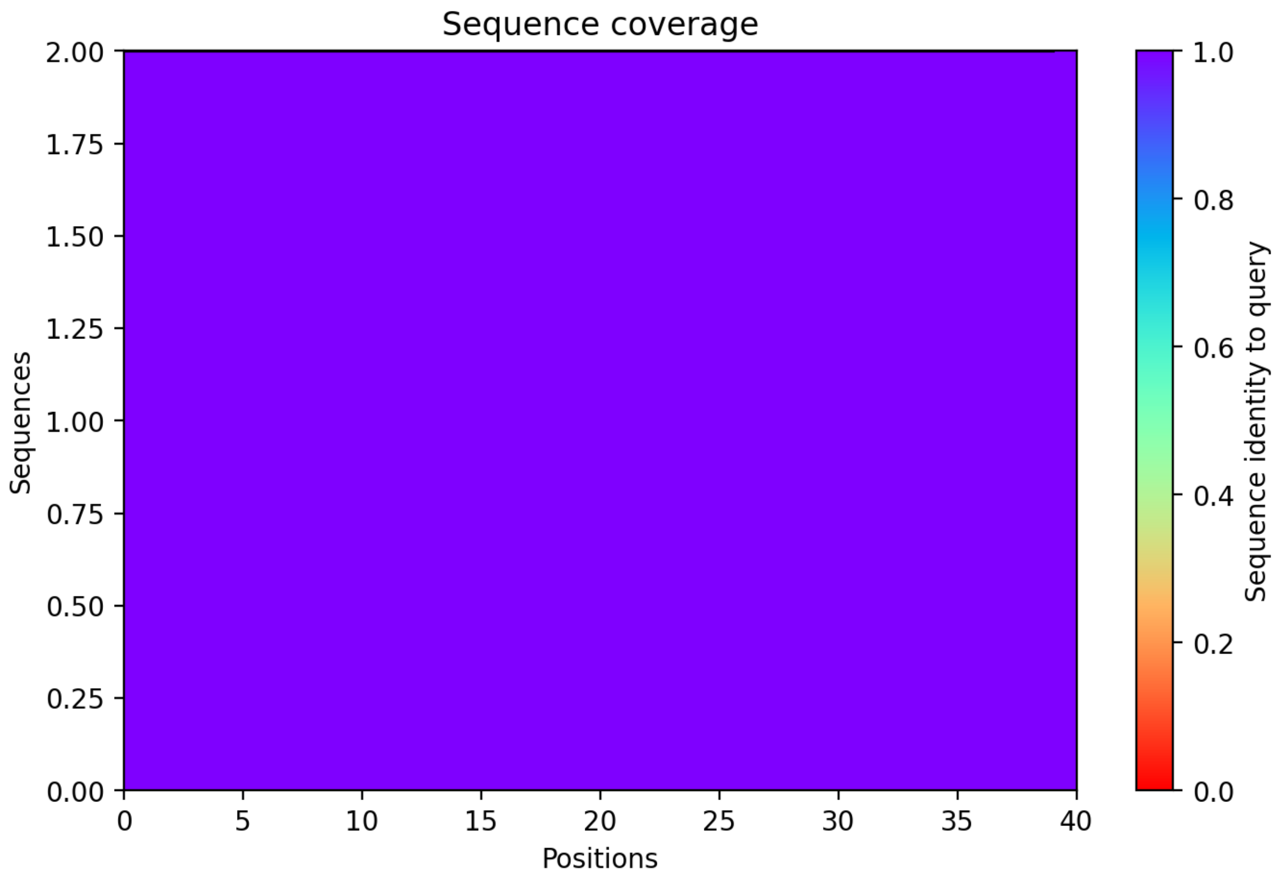

Supplementary Dataset S7 Part 3. A graphical summary on folding predictions for MS-supported chimeric peptide models (CPs) 101-156. The rest of the legend is the same as for Supplementary Dataset S7 Part 1.
