# Supplementary material for: Discovery of diverse chimeric peptides in a eukaryotic proteome sets the stage for experimental validation of the mosaic translation hypothesis
Source: Comput Struct Biotechnol J. 2025 Sep 12;27:4048–64. doi: 10.1016/j.csbj.2025.09.019 (PMC12481079; doi:10.1016/j.csbj.2025.09.019)
Supplement: Supplementary file 1 — Supplementary material [file mmc1.zip › Supplementary Datasets/Supplementary Dataset S7 Folding of chimeric protein models Part 4 Alpha-helices only.pdf]

CP1: MtrunA17_Chr0c01g0489091

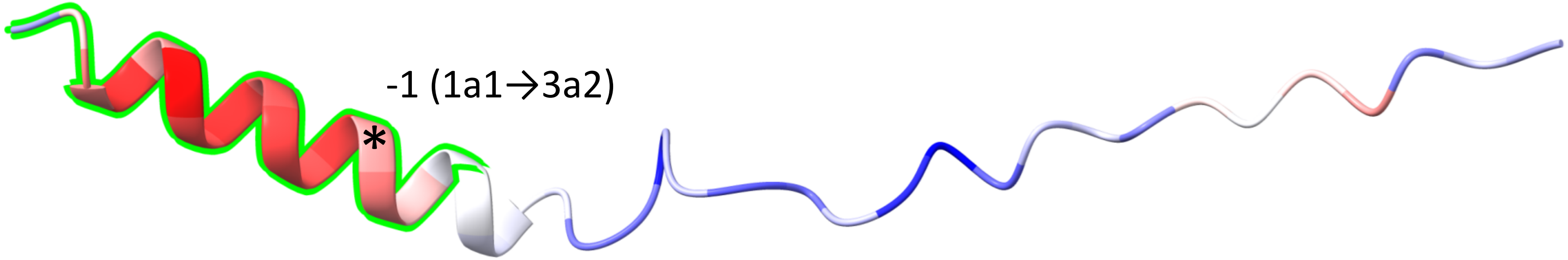

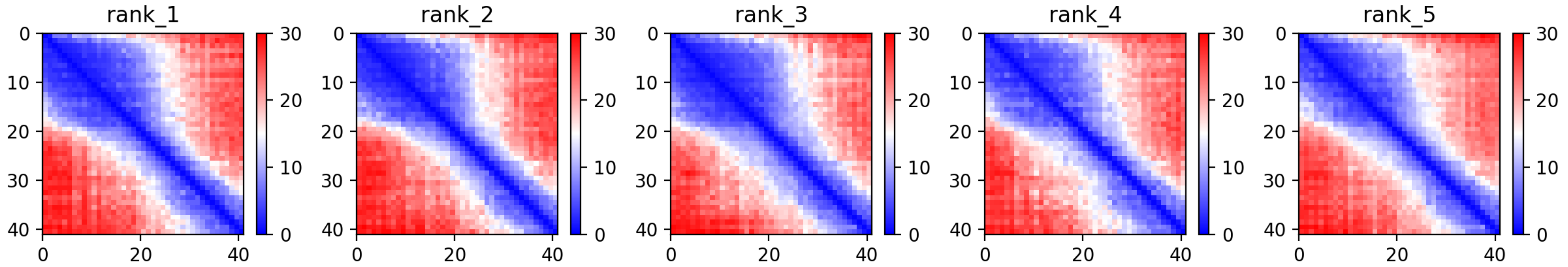

CP2: MtrunA17_Chr0c28g0493951

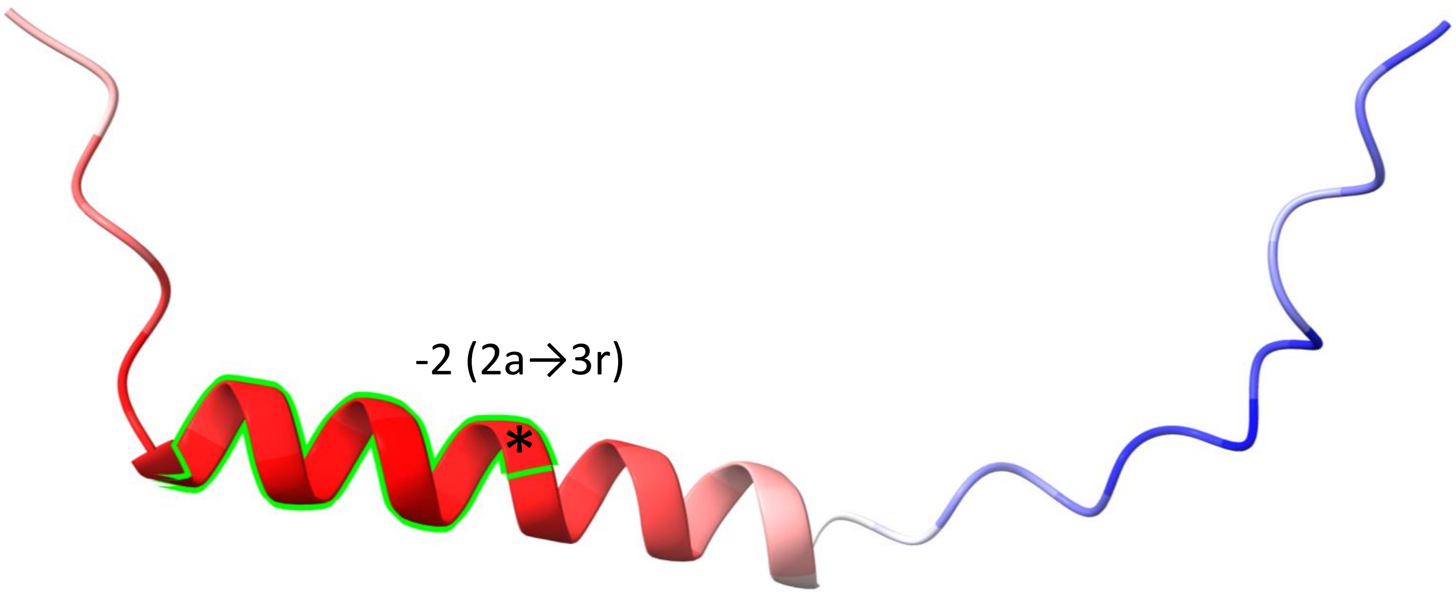

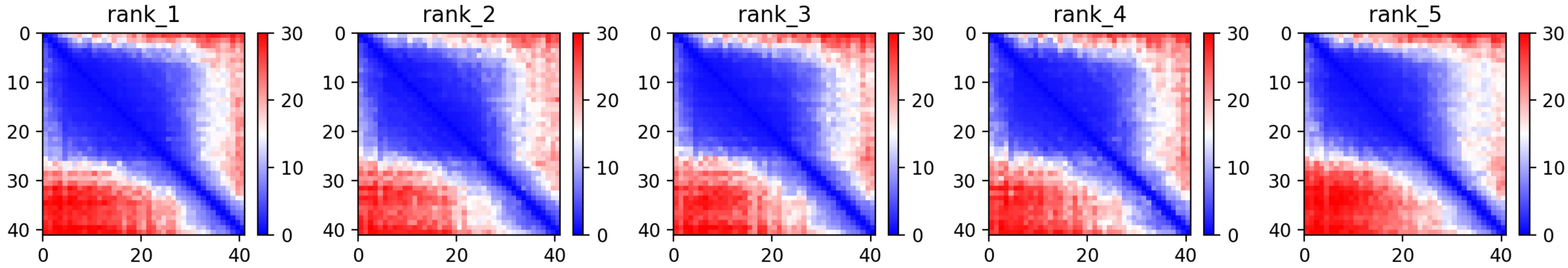

CP3: MtrunA17_Chr1g0148371

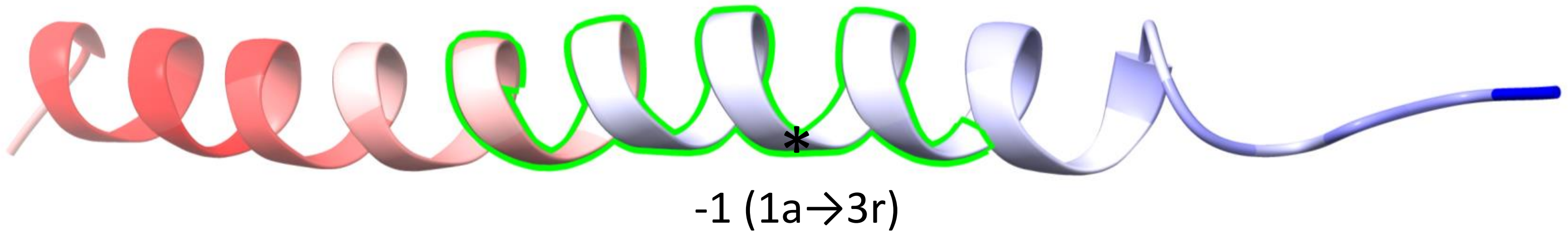

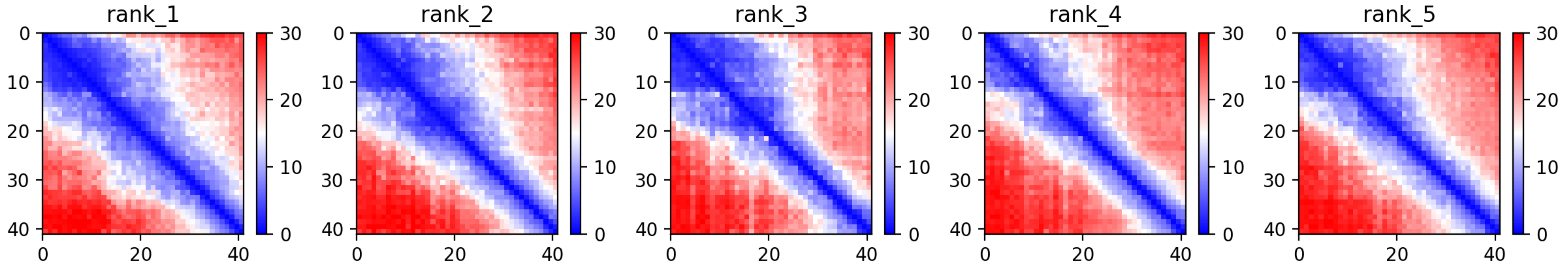

CP4: MtrunA17_Chr1g0149991

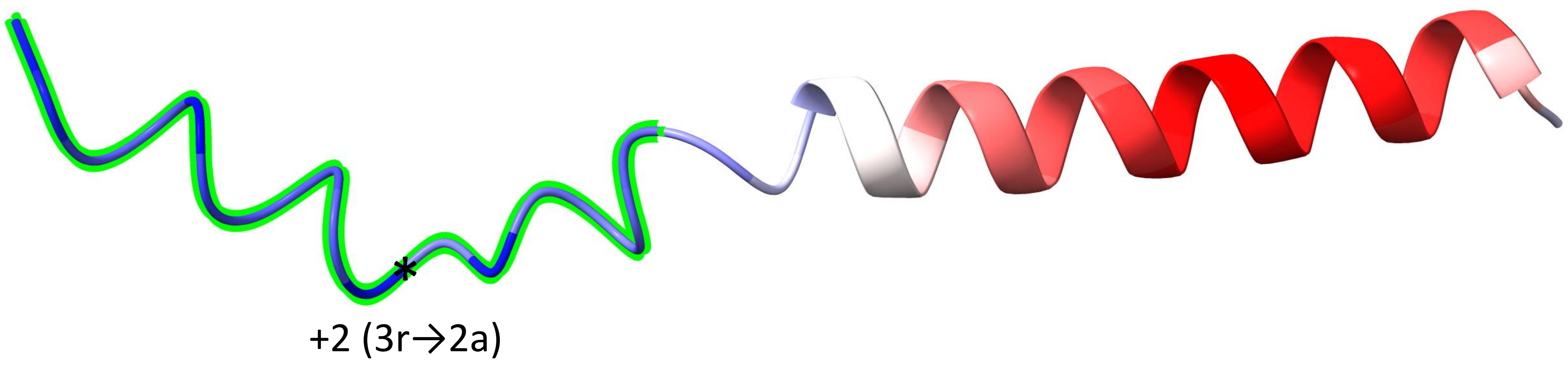

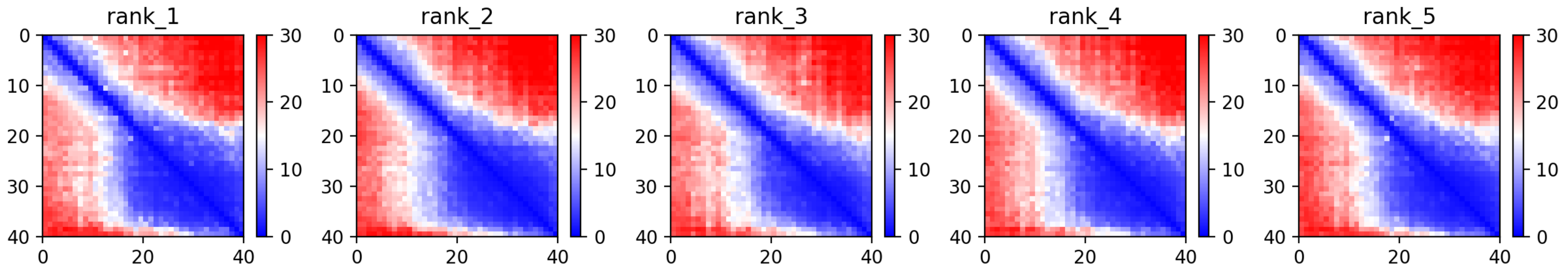

CP7: MtrunA17_Chr1g0153001

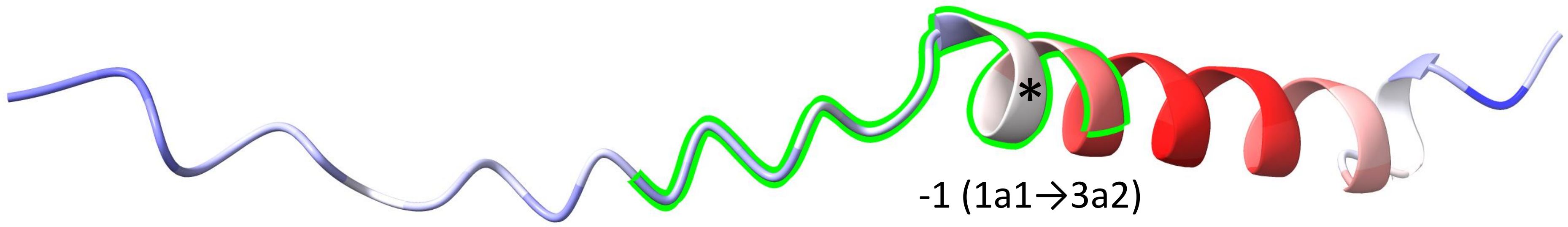

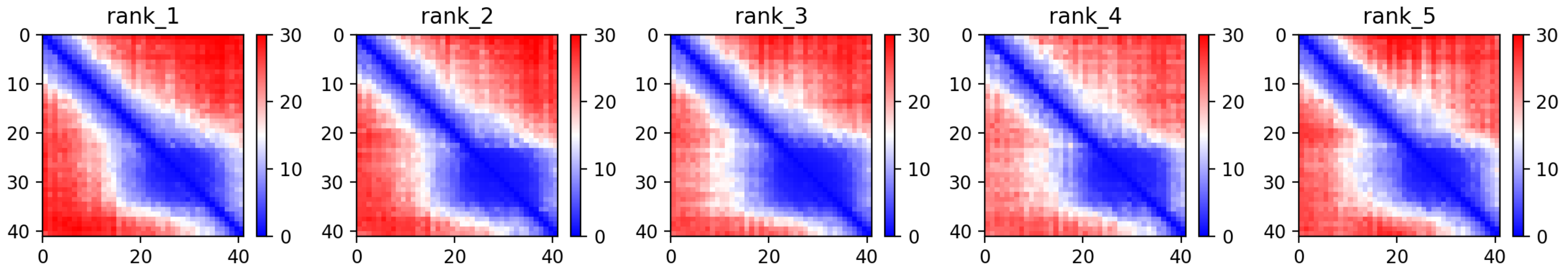

CP8: MtrunA17_Chr1g0155251

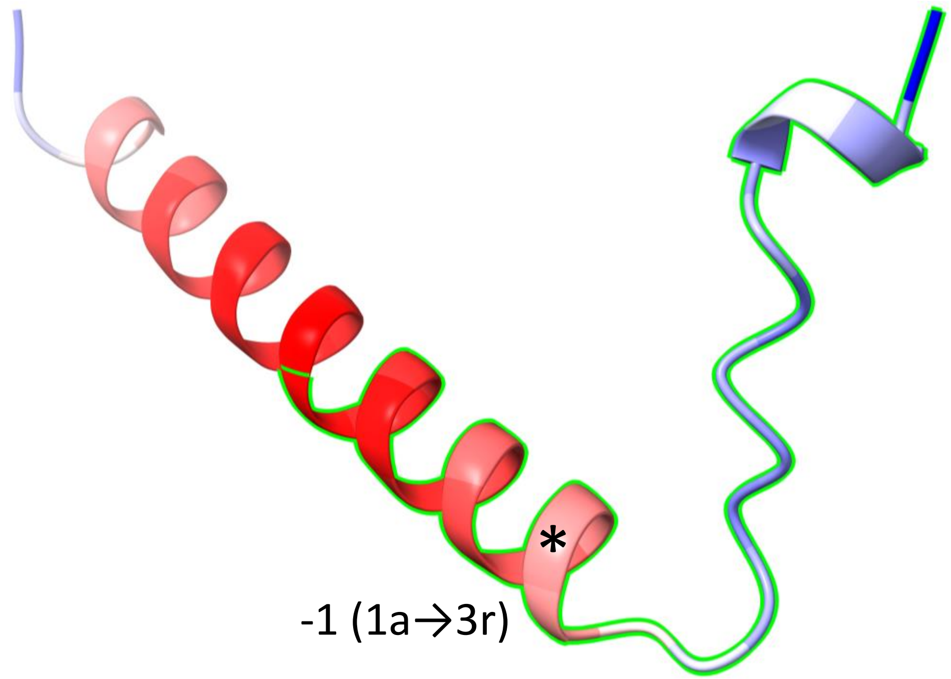

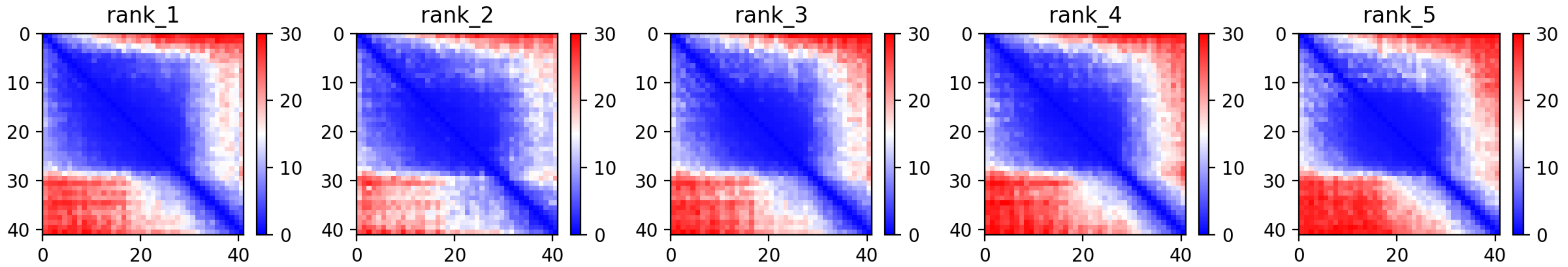

CP9: MtrunA17_Chr1g0158341

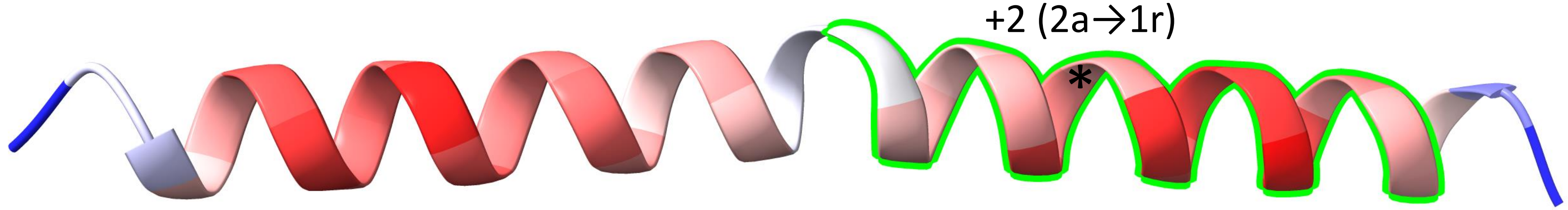

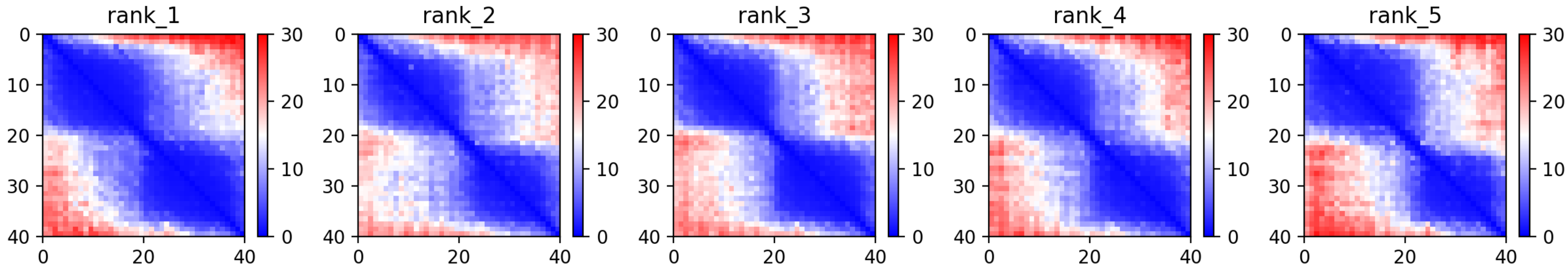

CP12: MtrunA17_Chr1g0178361

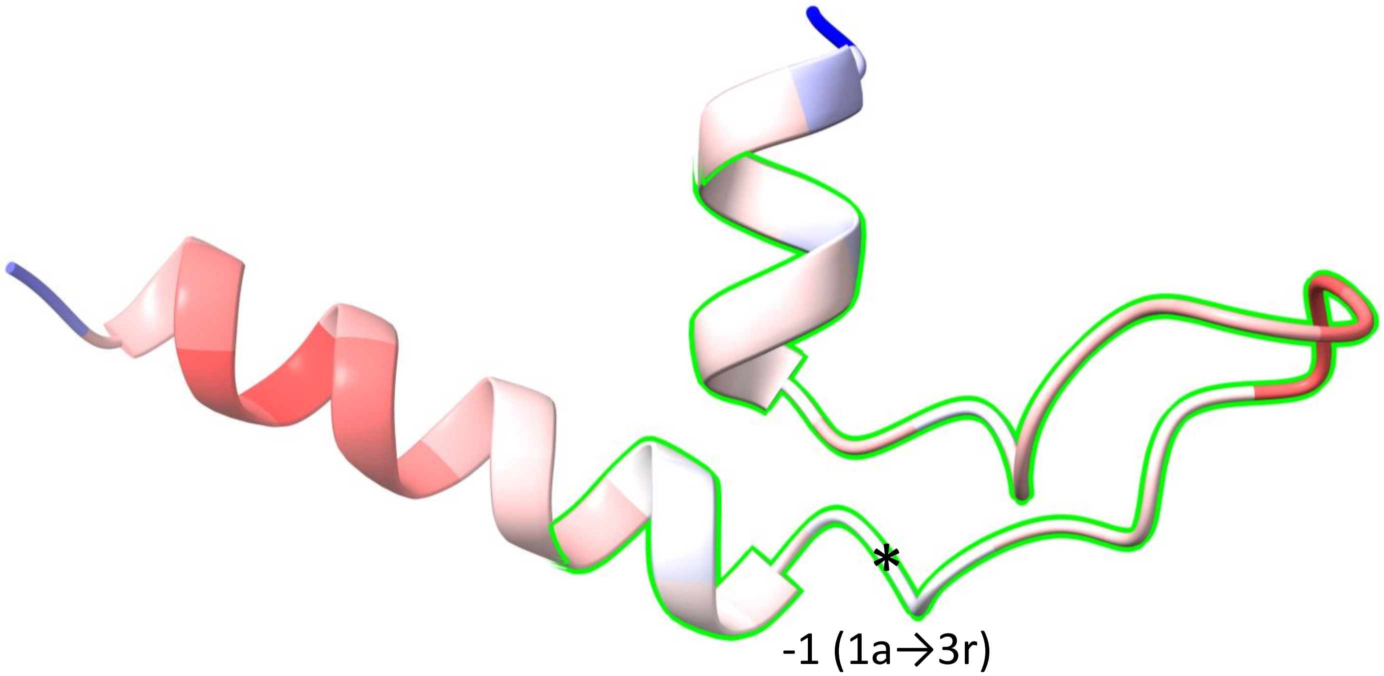

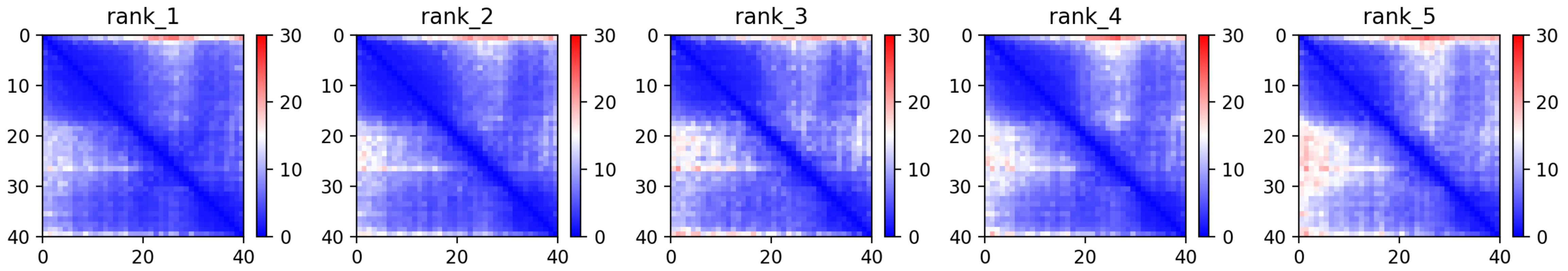

CP15: MtrunA17_Chr1g0183001

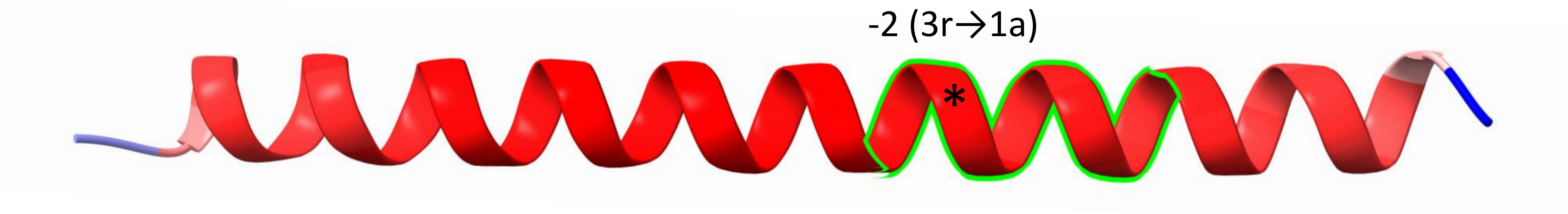

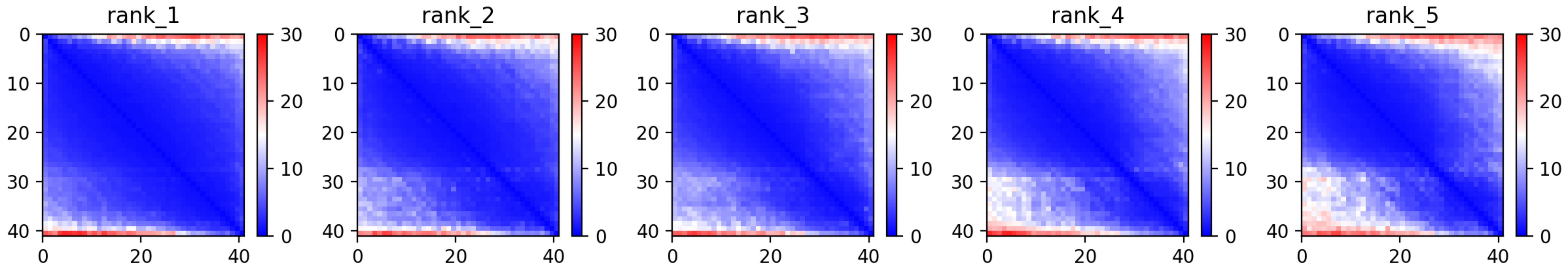

CP22: MtrunA17_Chr1g0198091

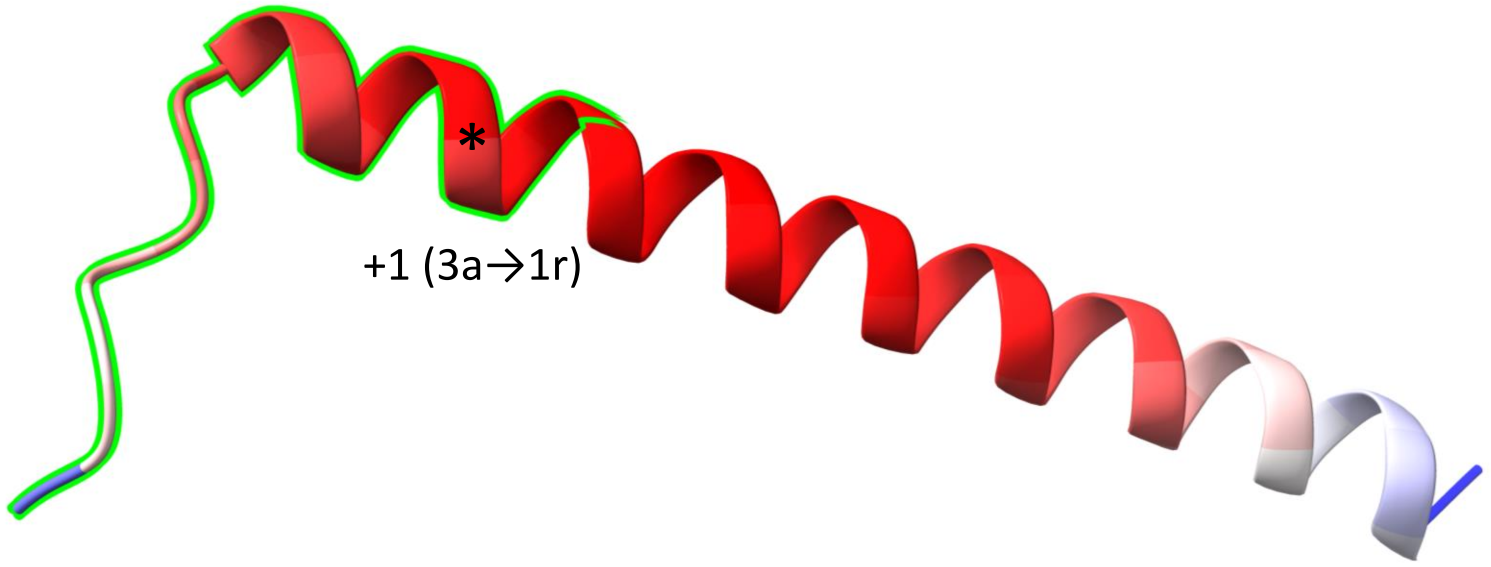

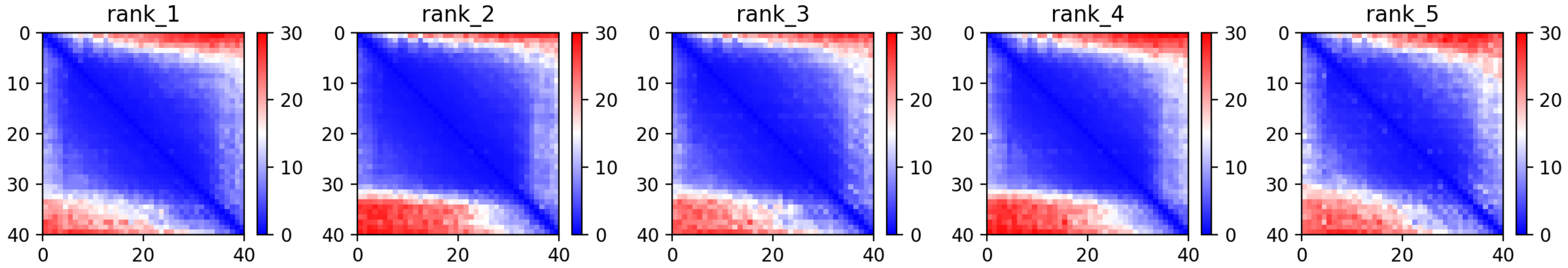

CP25: MtrunA17_Chr1g0202001

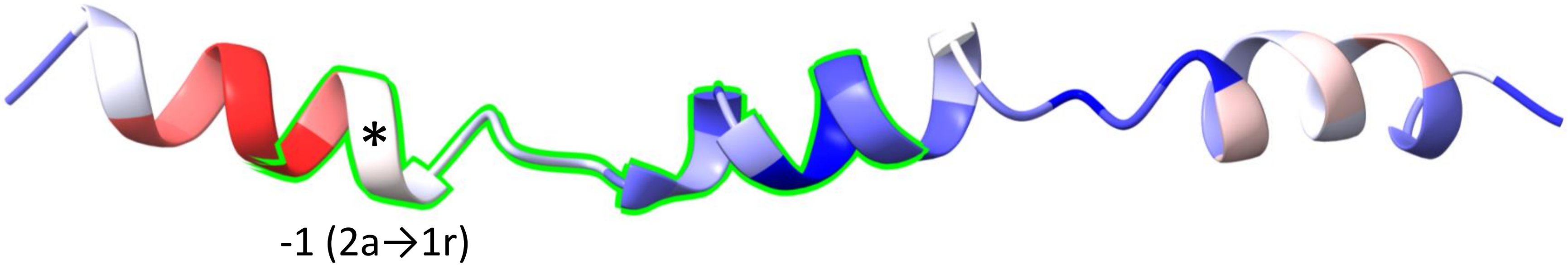

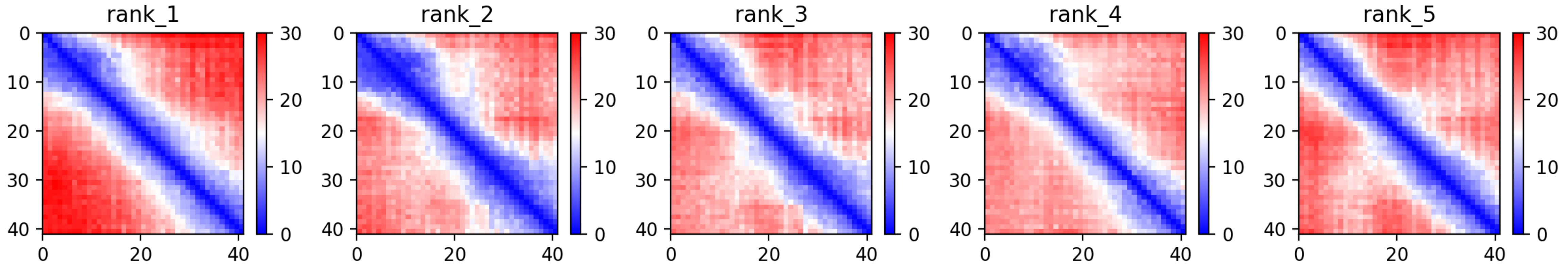

CP27: MtrunA17_Chr1g0207811

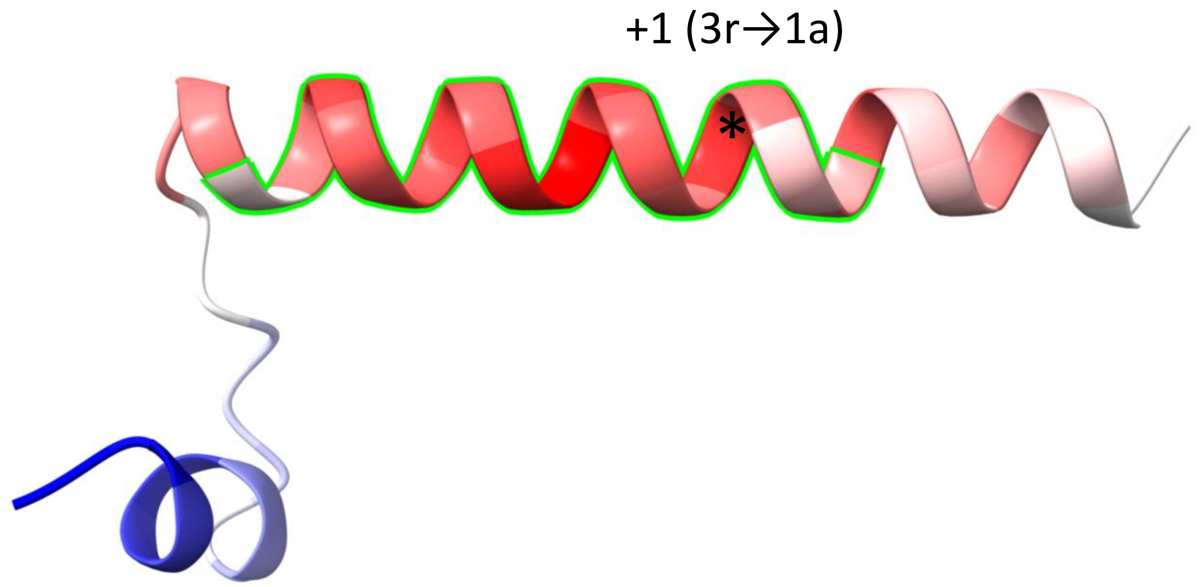

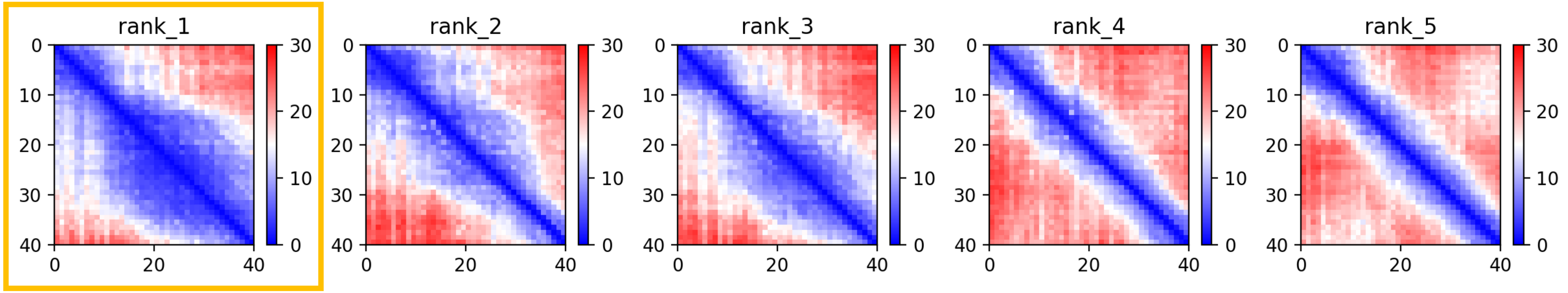

CP30: MtrunA17_Chr1g0210521

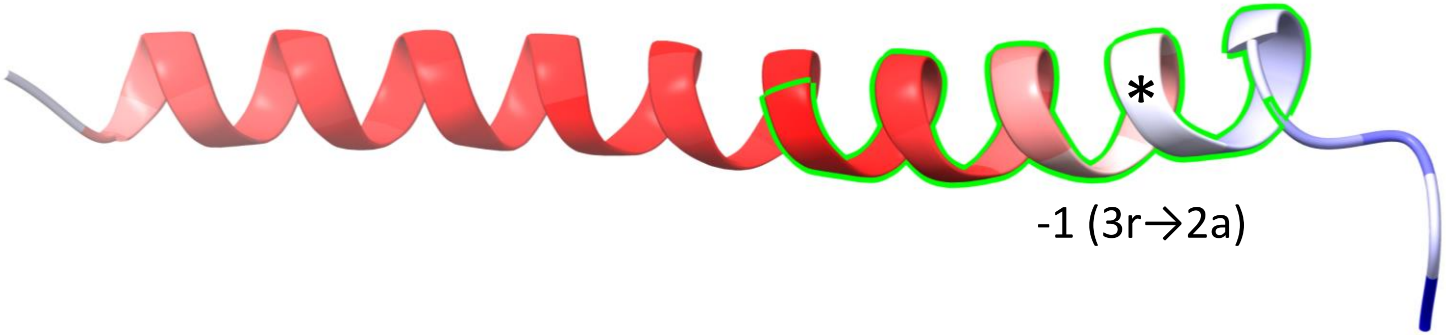

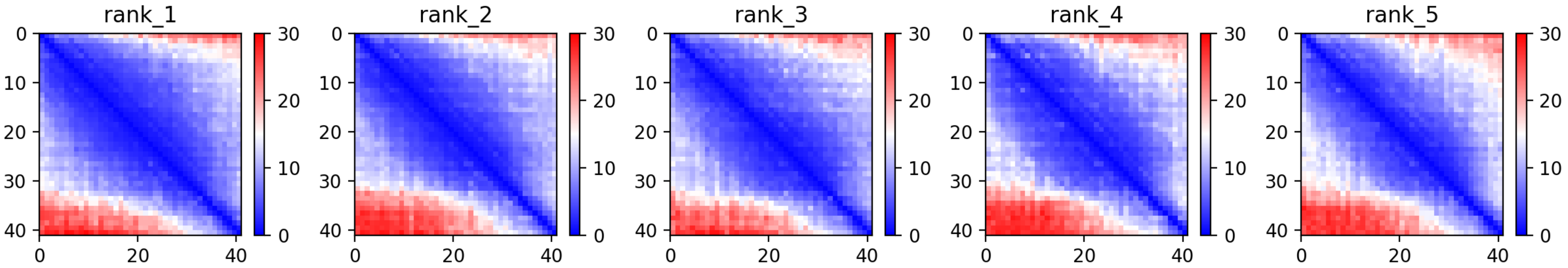

CP32: MtrunA17_Chr1g1004575

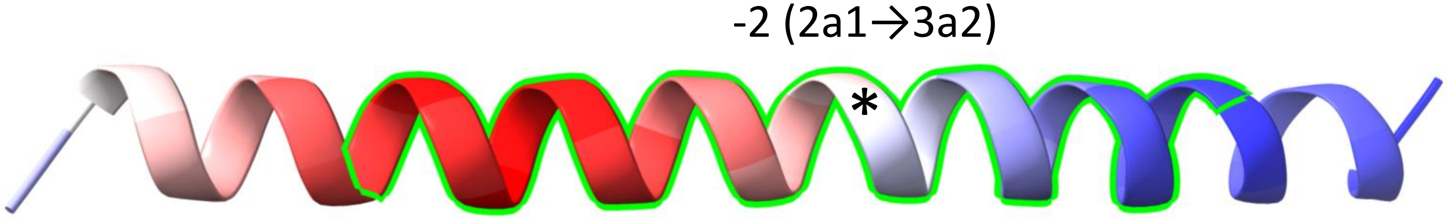

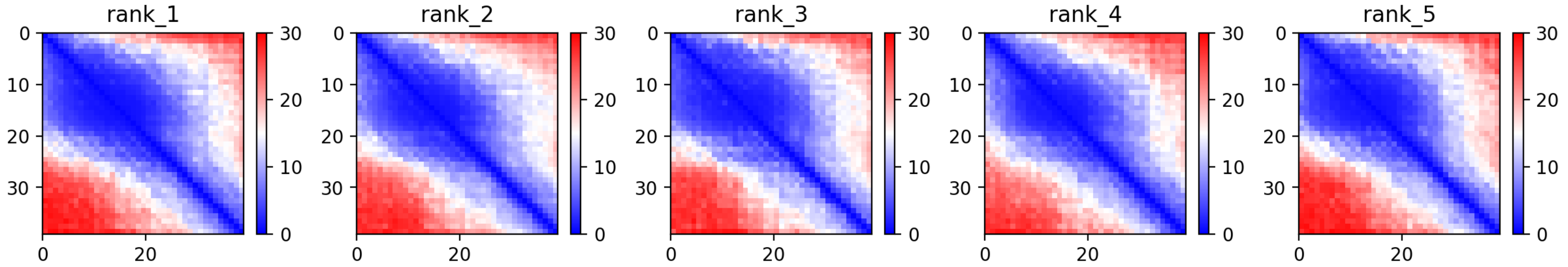

CP35: MtrunA17_Chr2g0292921

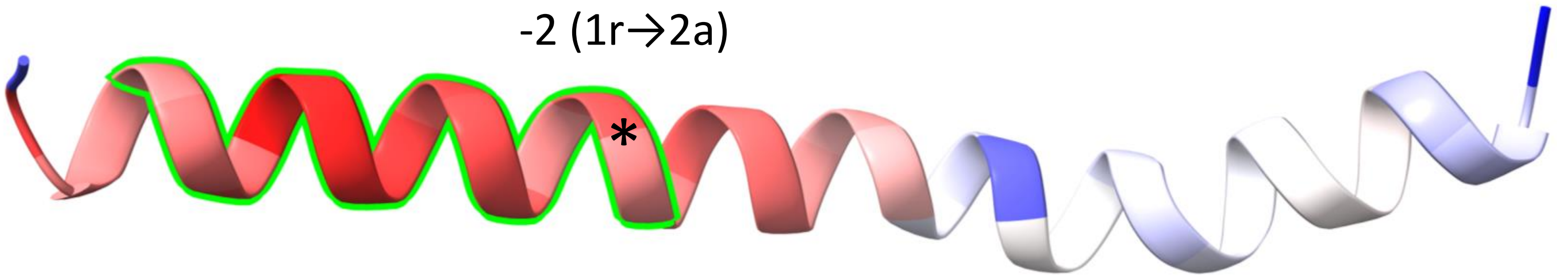

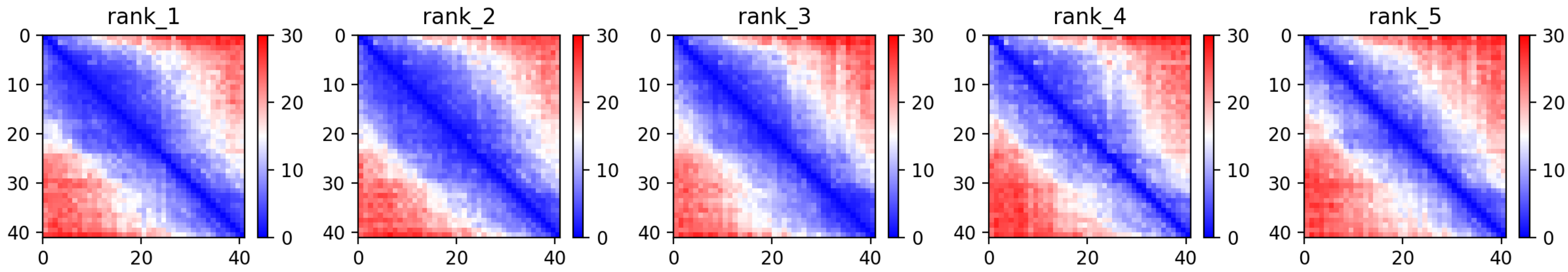

CP37: MtrunA17_Chr2g0299561

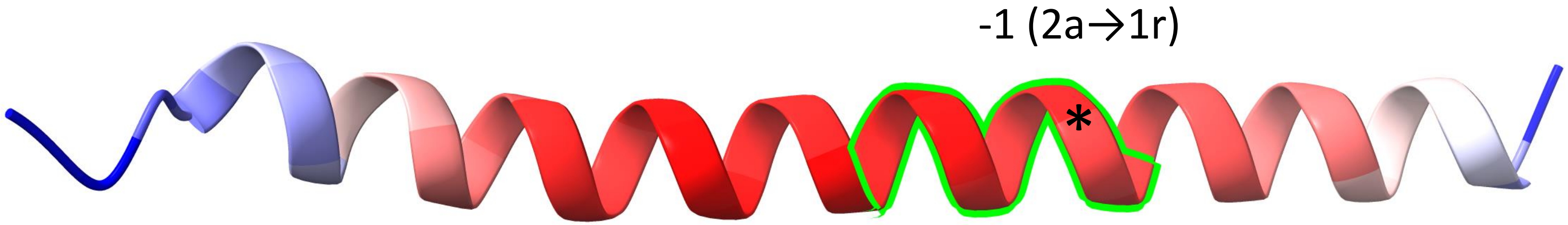

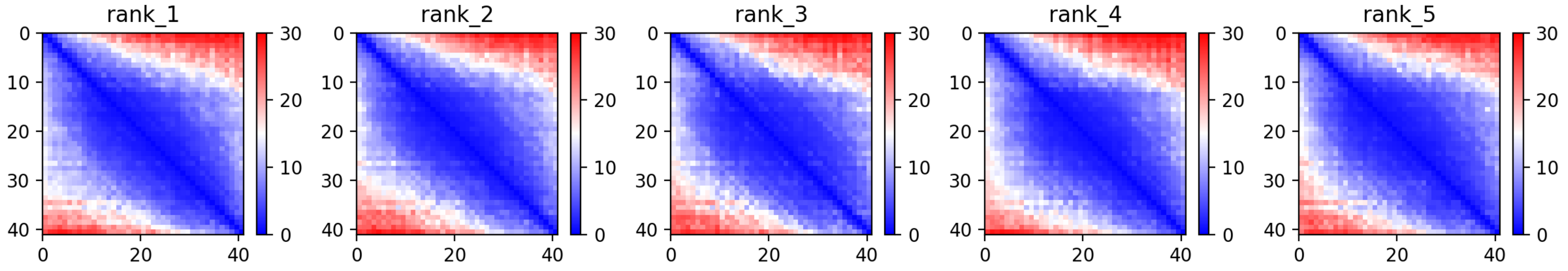

CP43: MtrunA17_Chr2g0316291

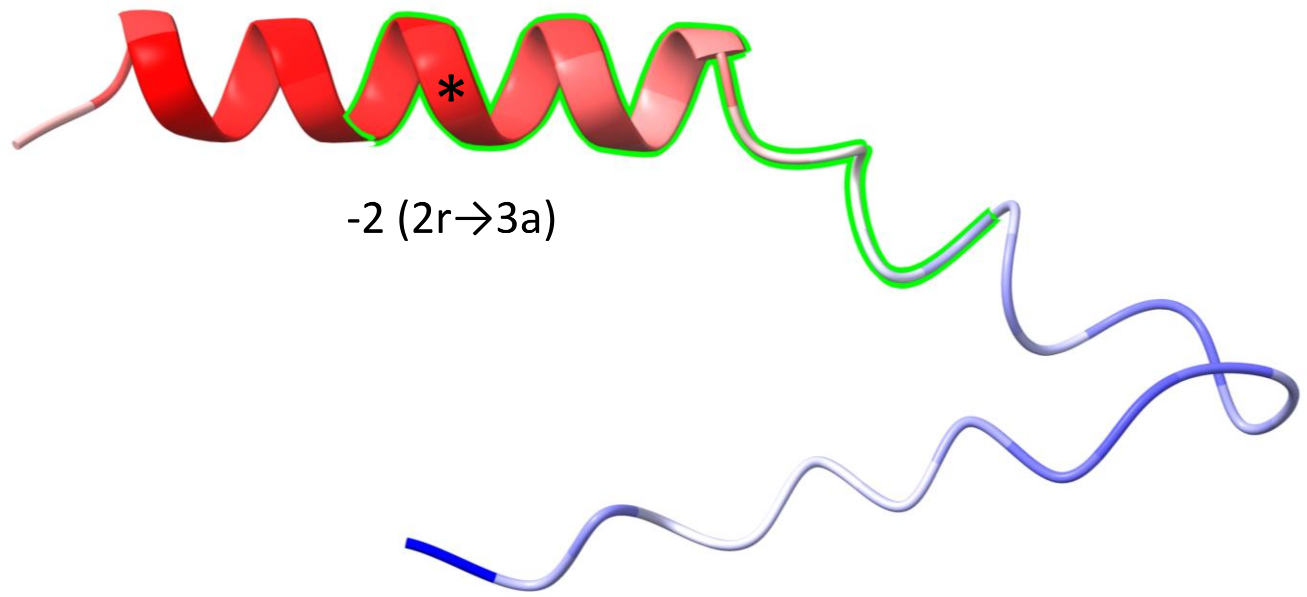

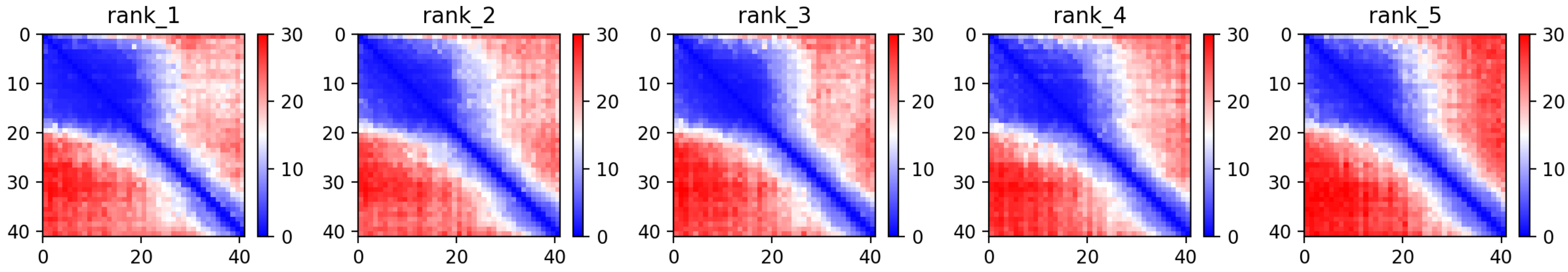

CP44: MtrunA17_Chr2g0326801

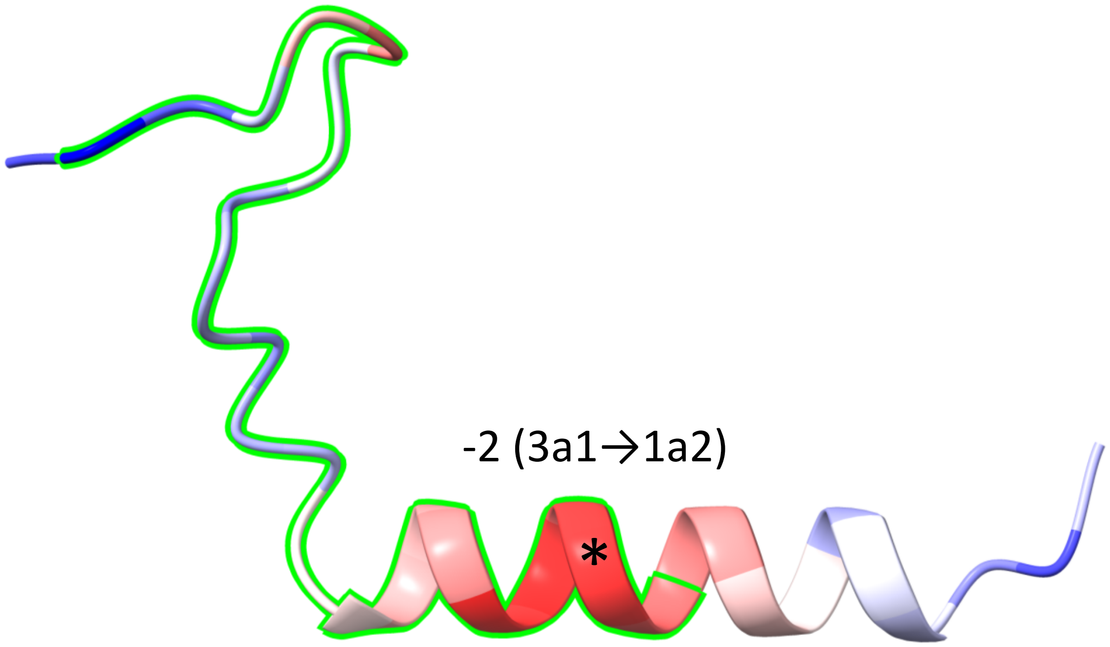

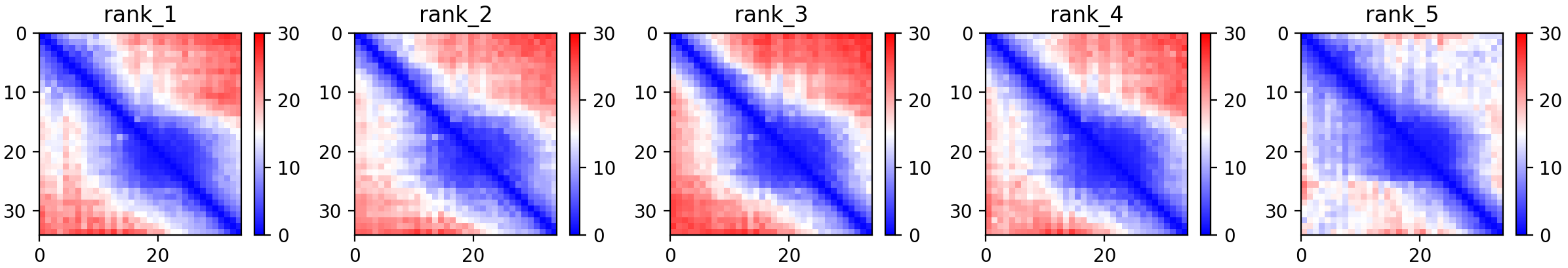

CP47: MtrunA17_Chr3g0079521

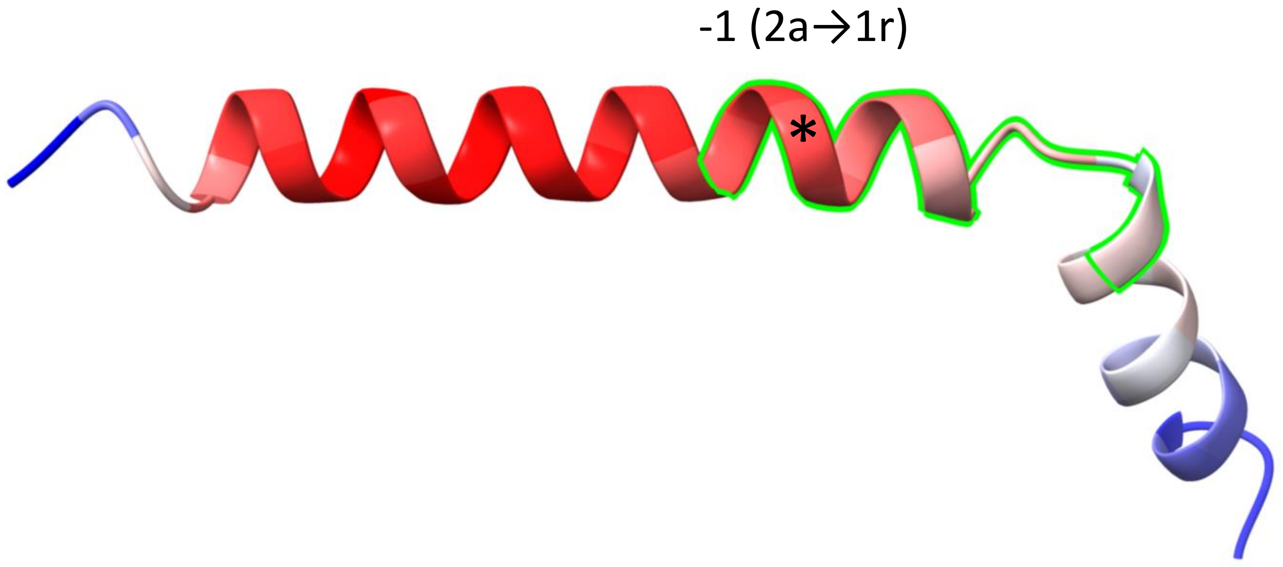

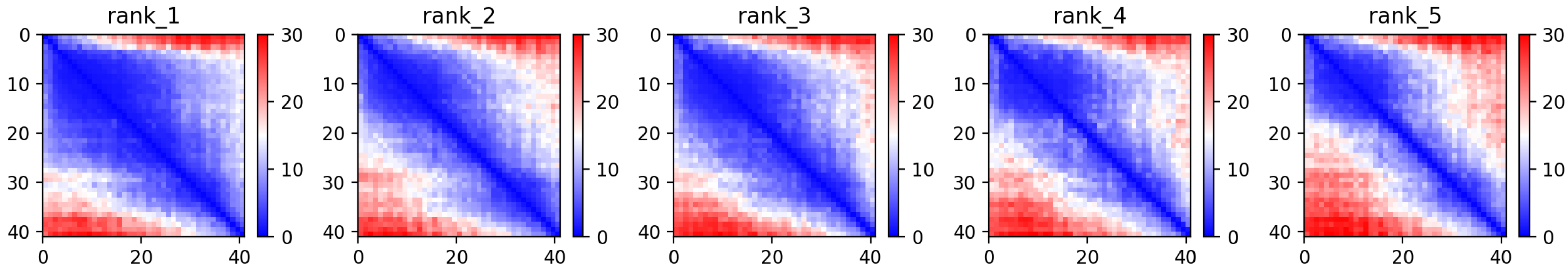

CP48: MtrunA17_Chr3g0083801

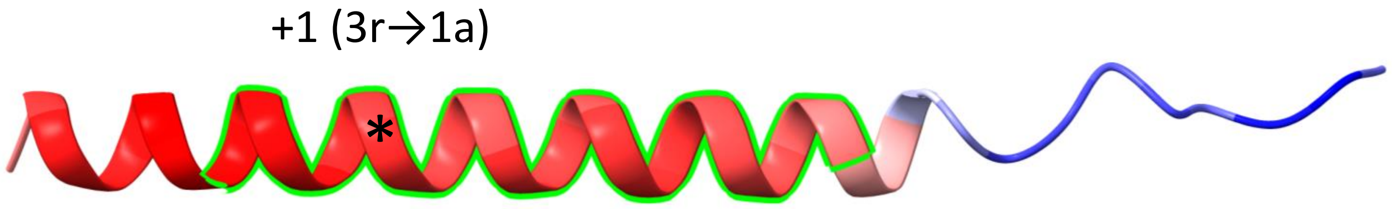

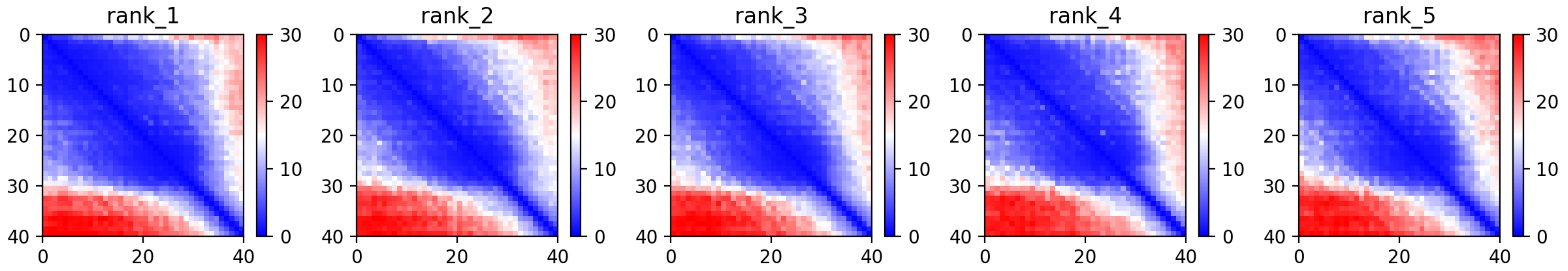

CP49: MtrunA17_Chr3g0091141

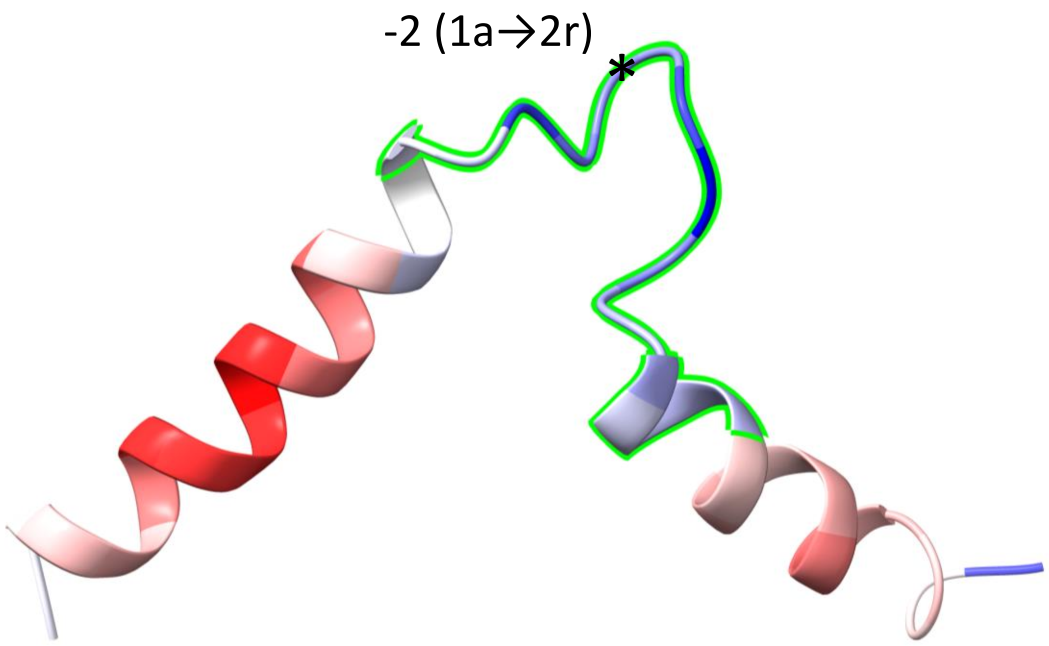

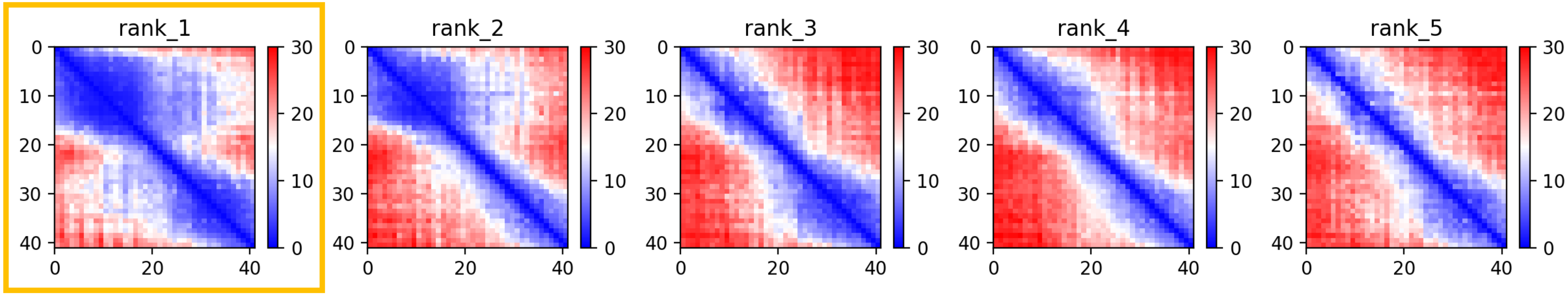

CP53: MtrunA17_Chr3g0102171

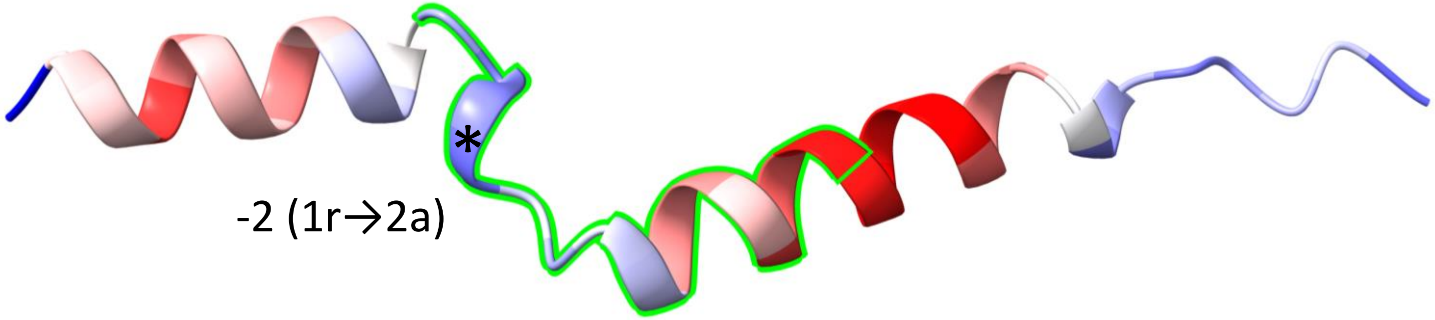

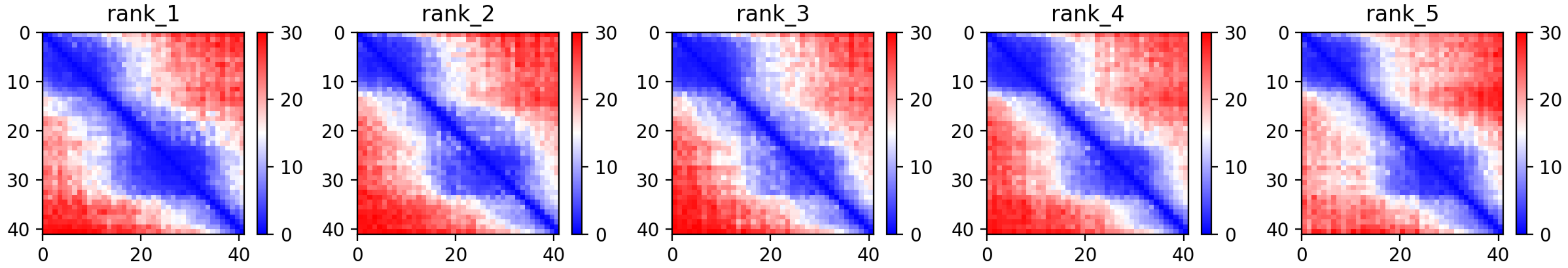

CP58: MtrunA17_Chr3g0127321

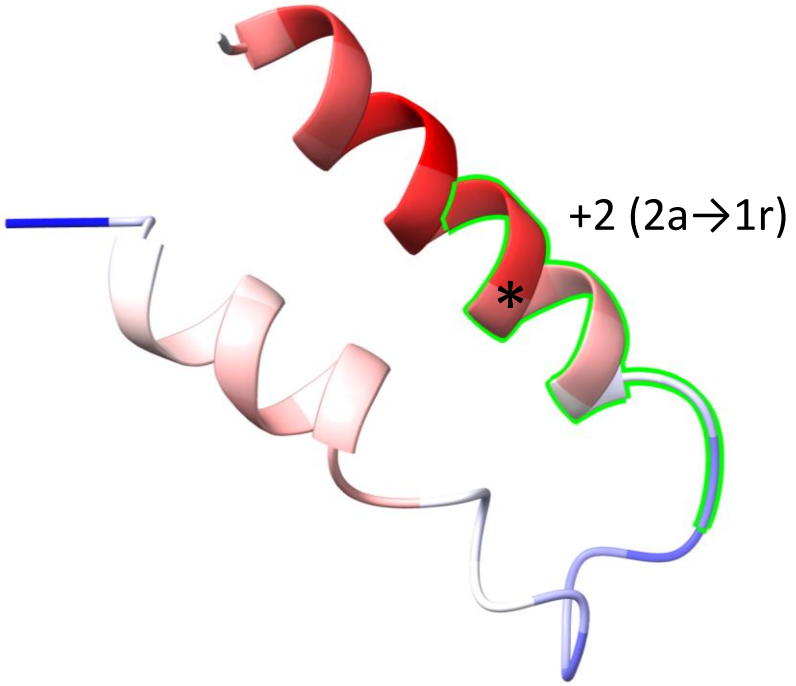

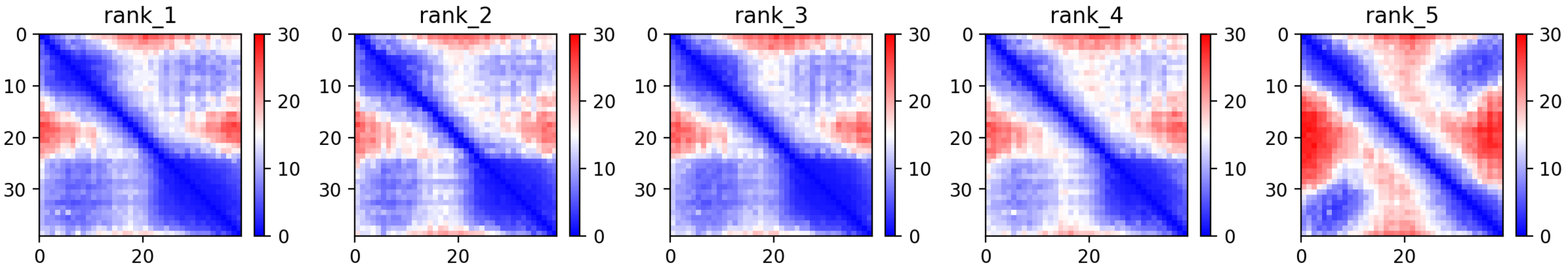

CP59: MtrunA17_Chr3g0130671

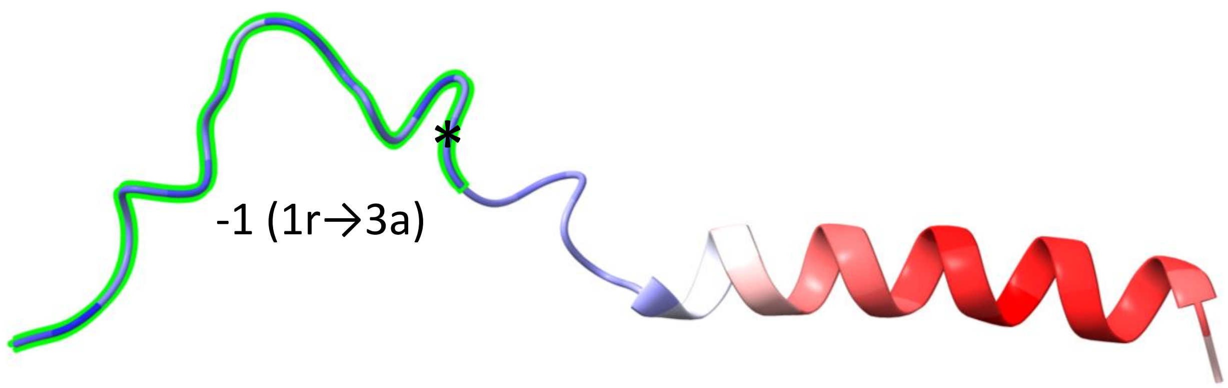

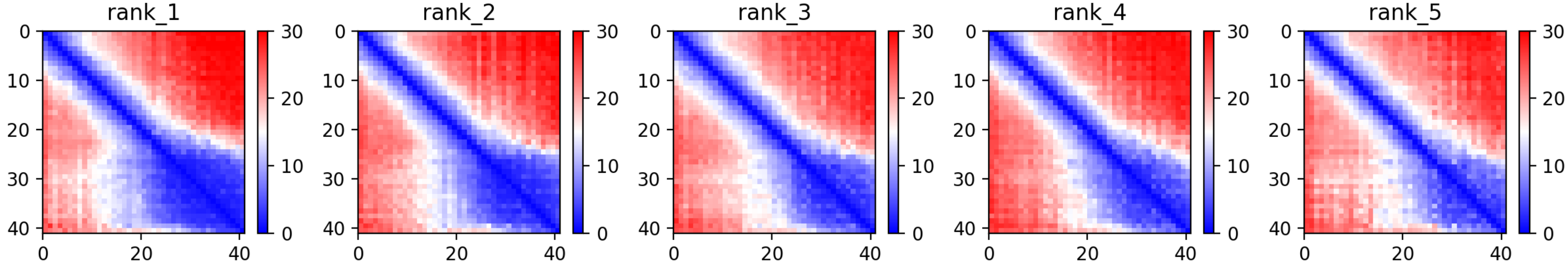

CP61: MtrunA17_Chr3g0137391

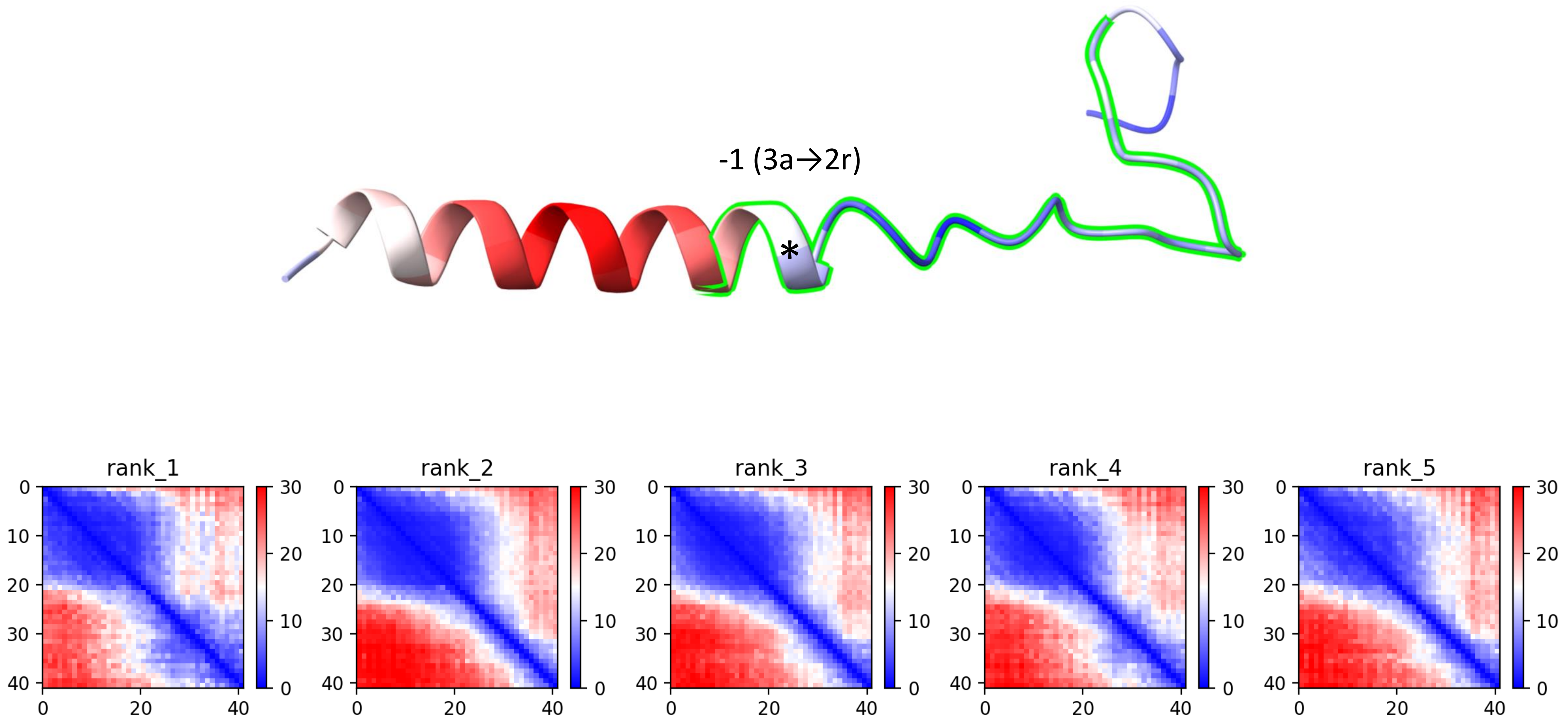

CP62: MtrunA17_Chr3g0141901

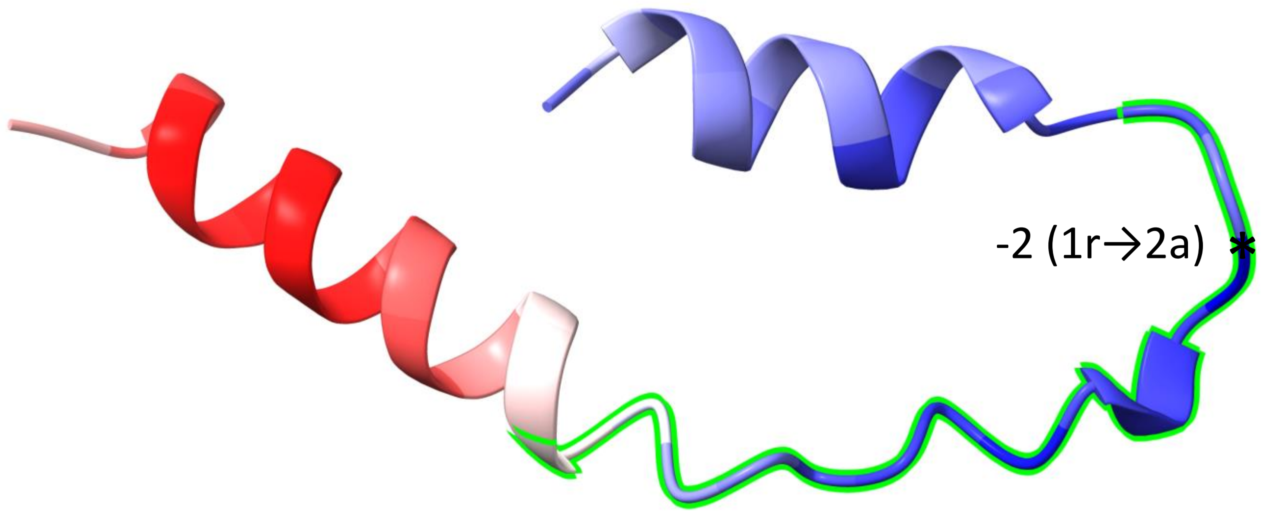

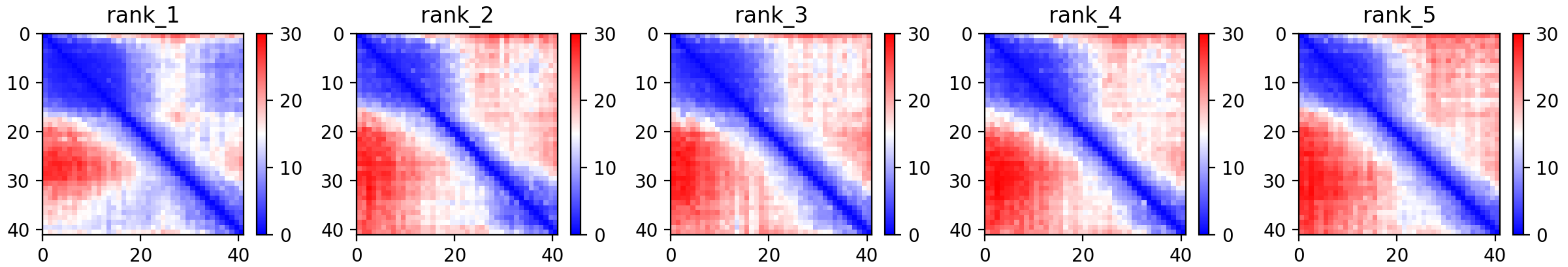

CP63: MtrunA17_Chr3g0144151

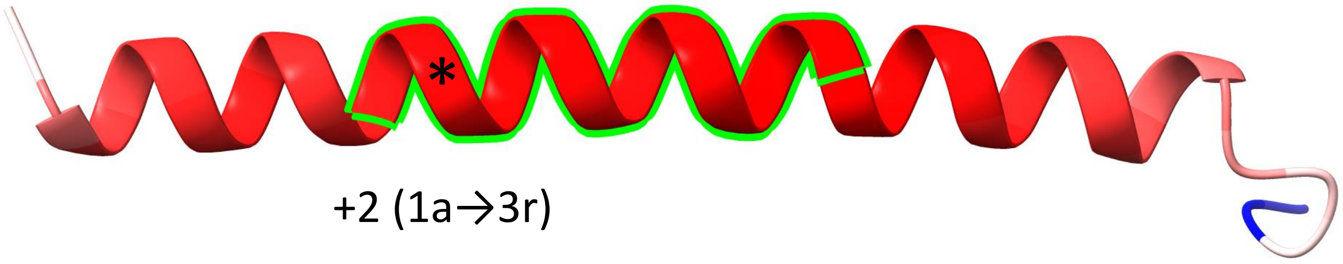

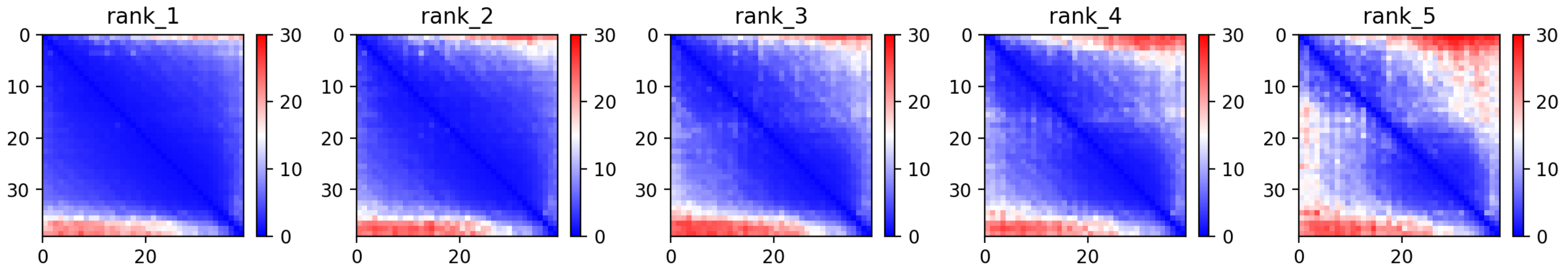

CP64: MtrunA17_Chr3g0144151

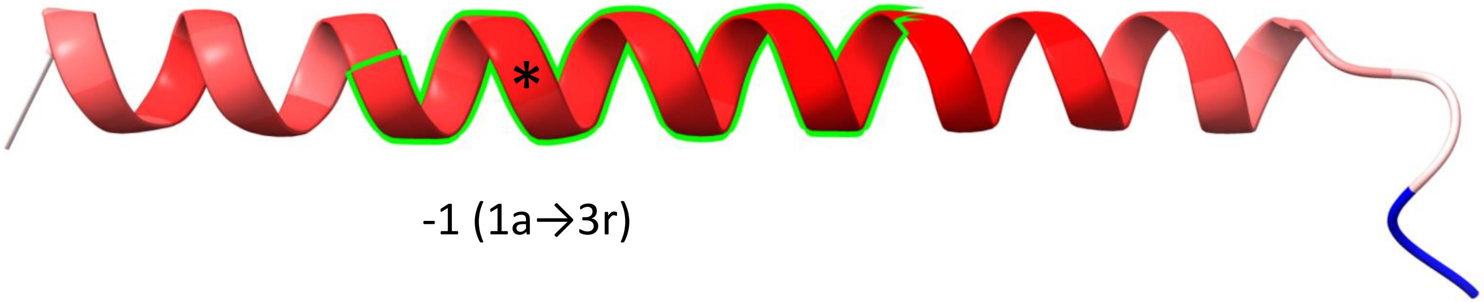

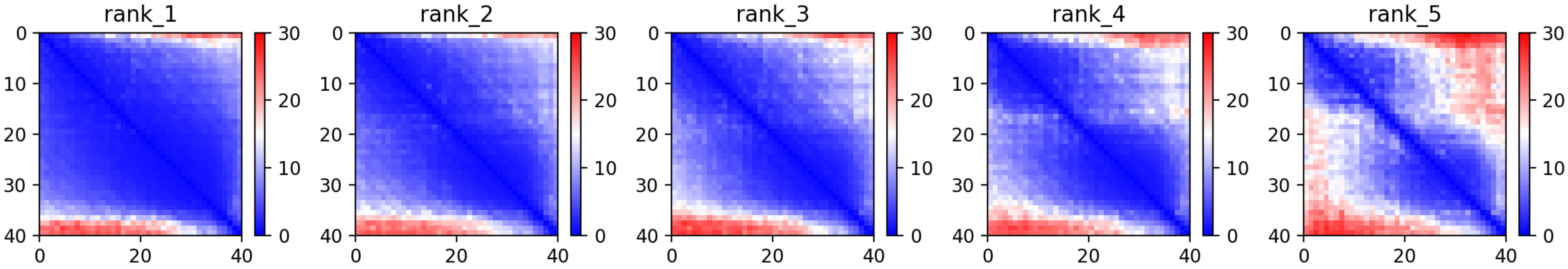

CP65: MtrunA17_Chr3g0144151

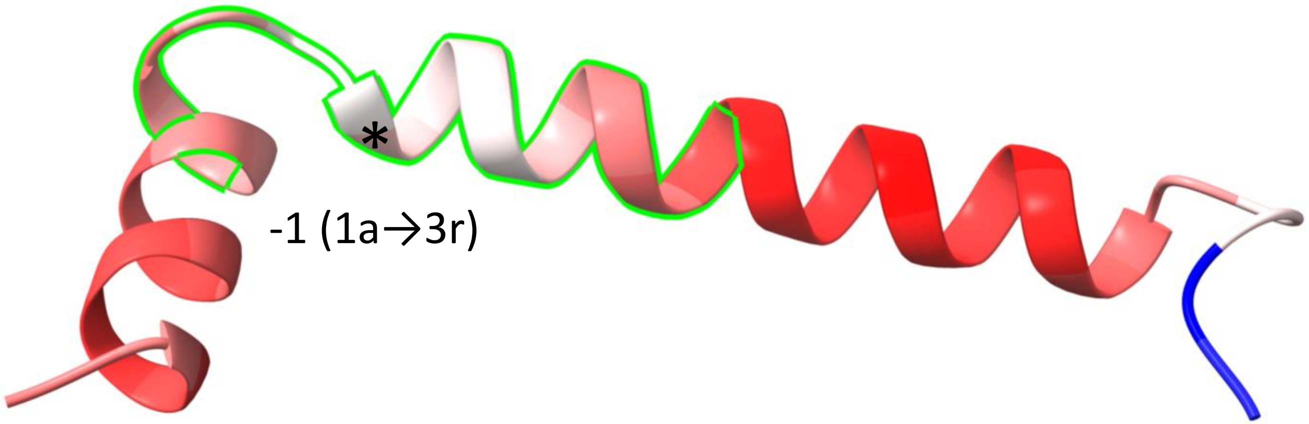

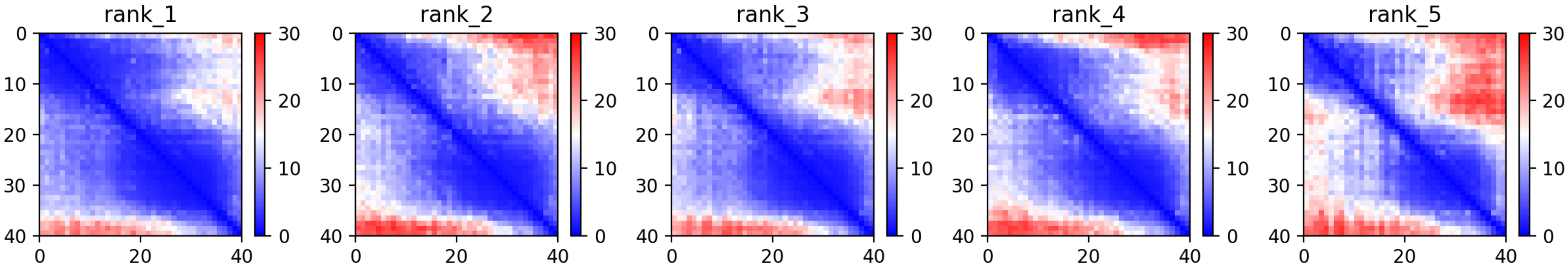

CP66: MtrunA17_Chr3g1011650

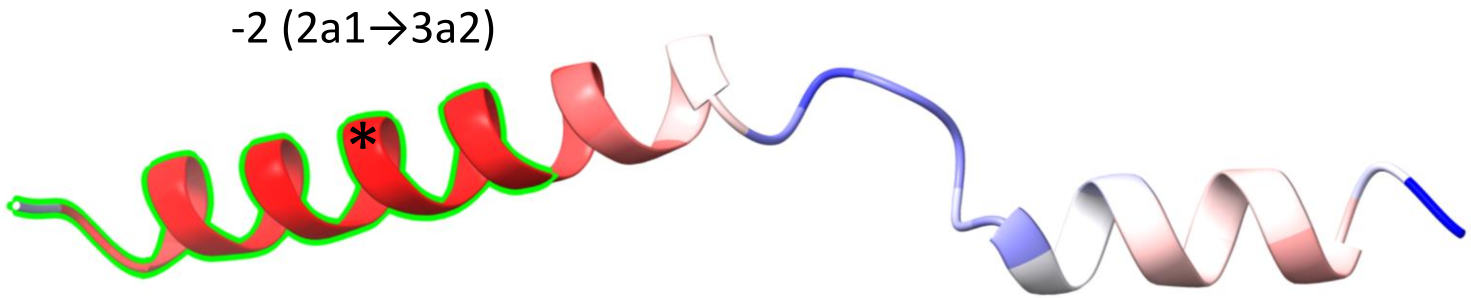

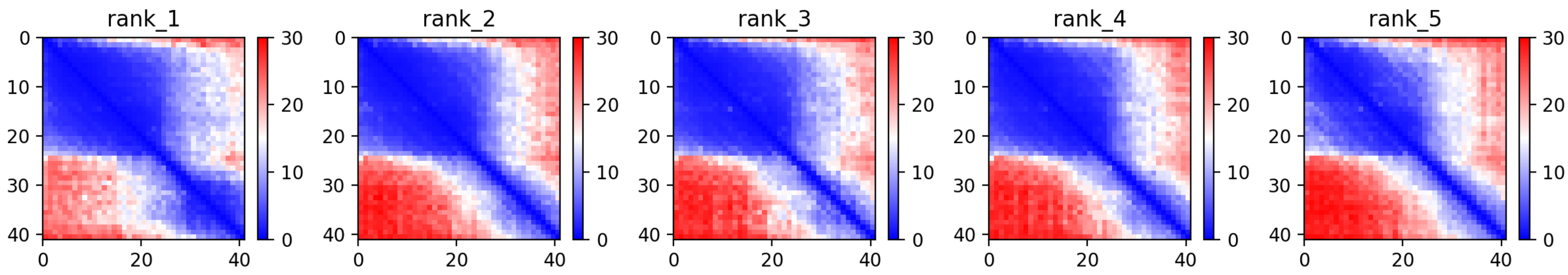

CP67: MtrunA17_Chr4g0000131

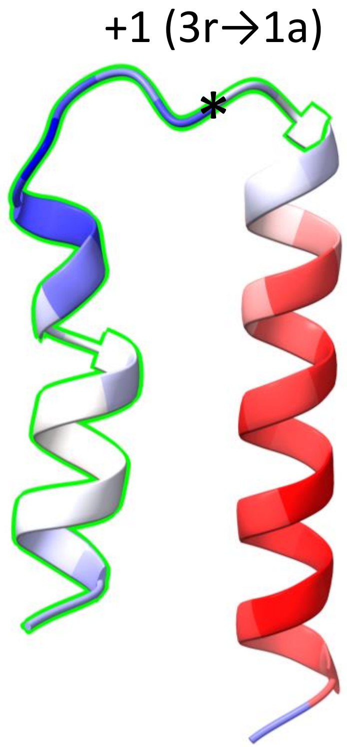

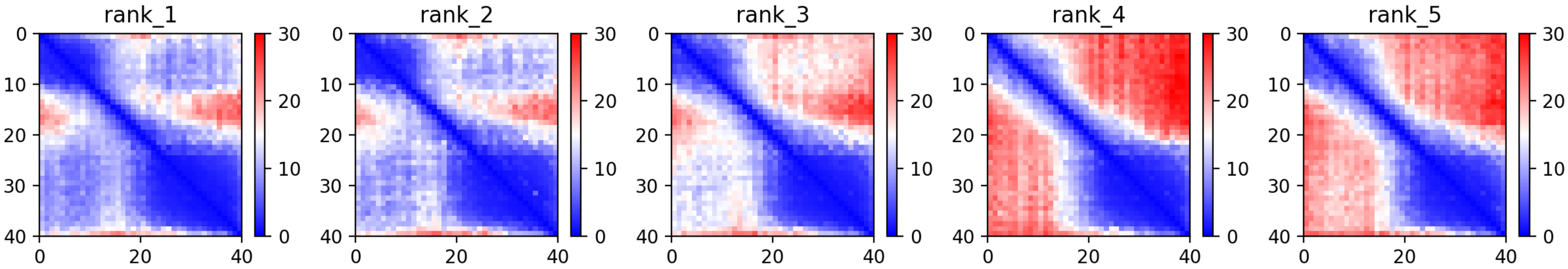

CP68: MtrunA17_Chr4g0000891

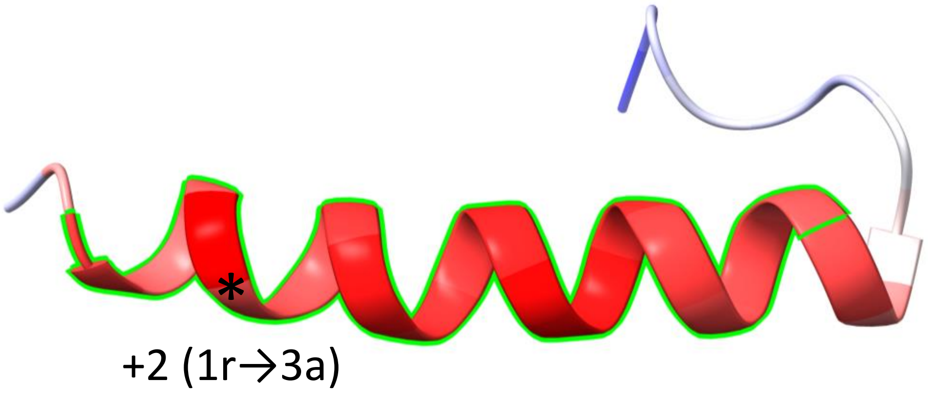

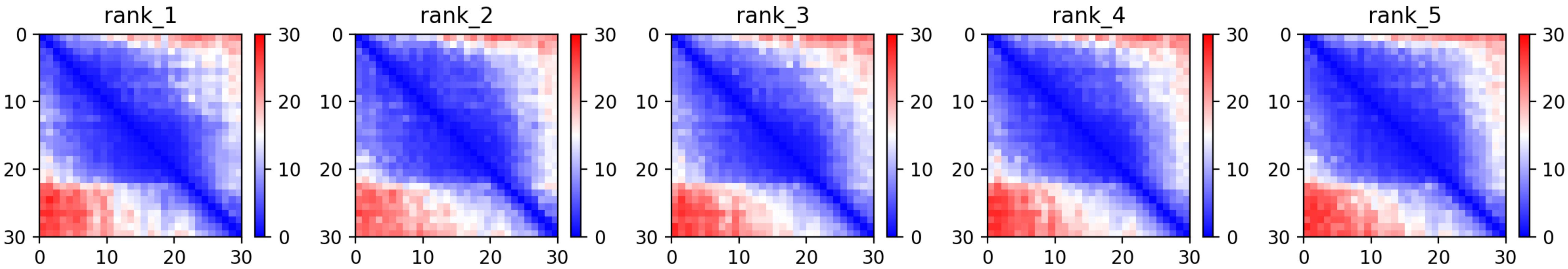

CP70: MtrunA17_Chr4g0014251

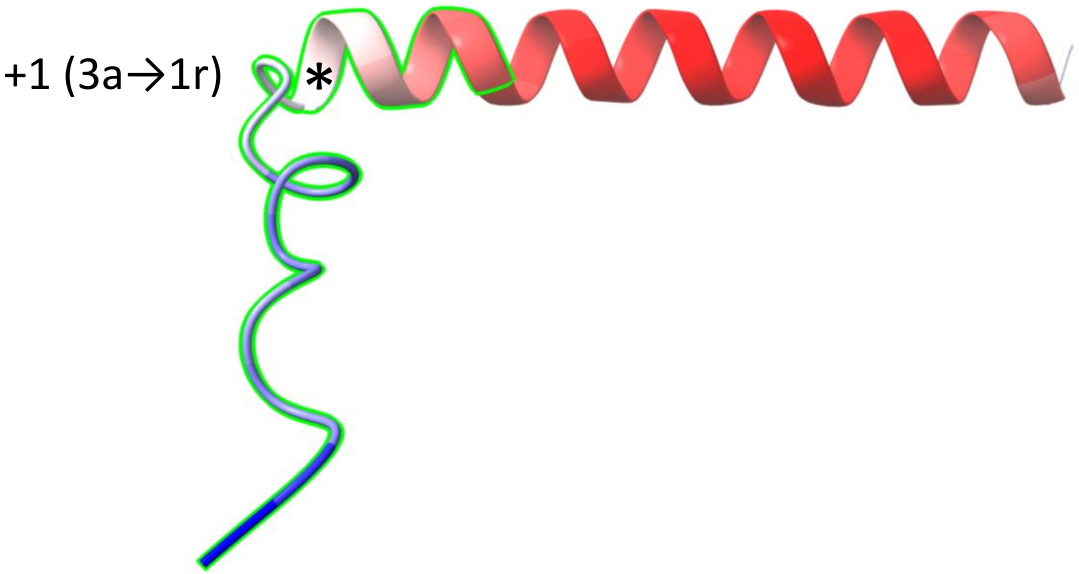

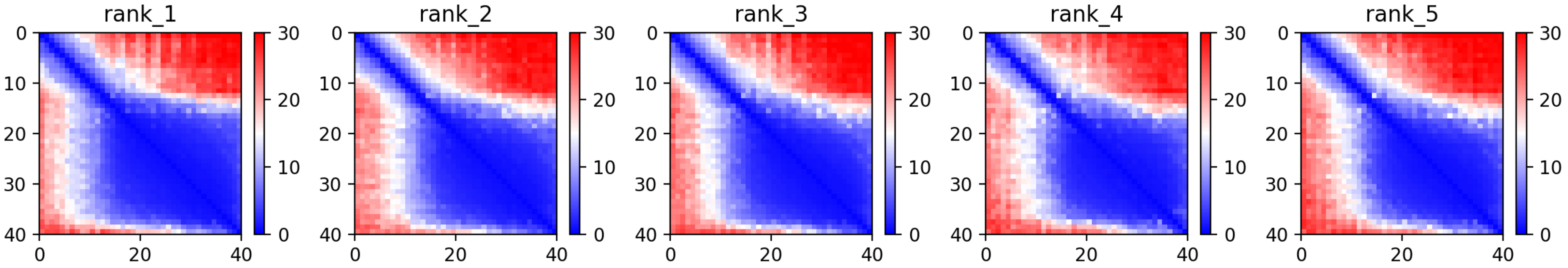

CP72: MtrunA17_Chr4g0023791

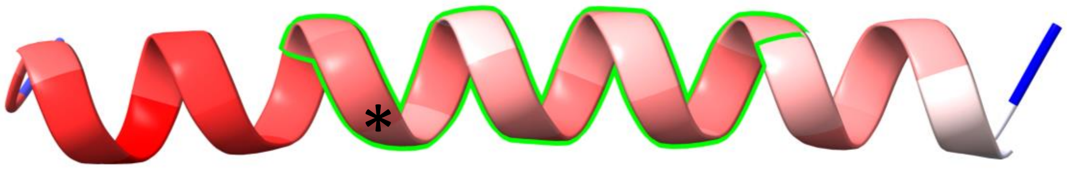

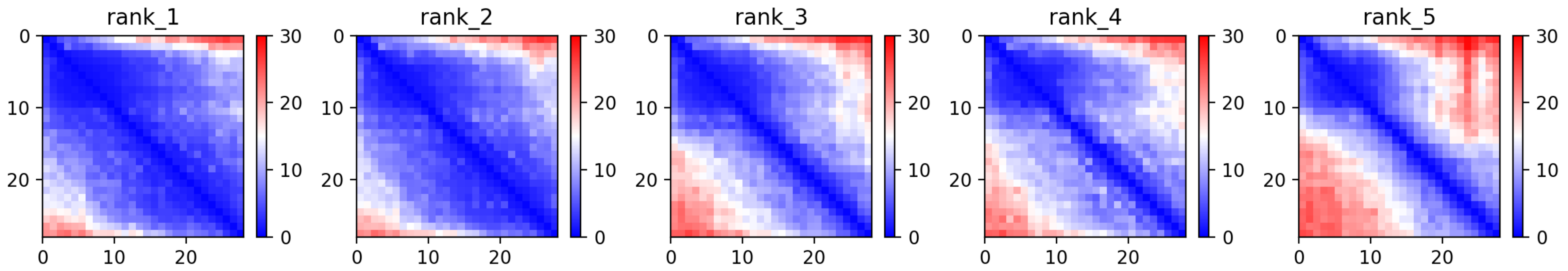

CP73: MtrunA17_Chr4g0034271

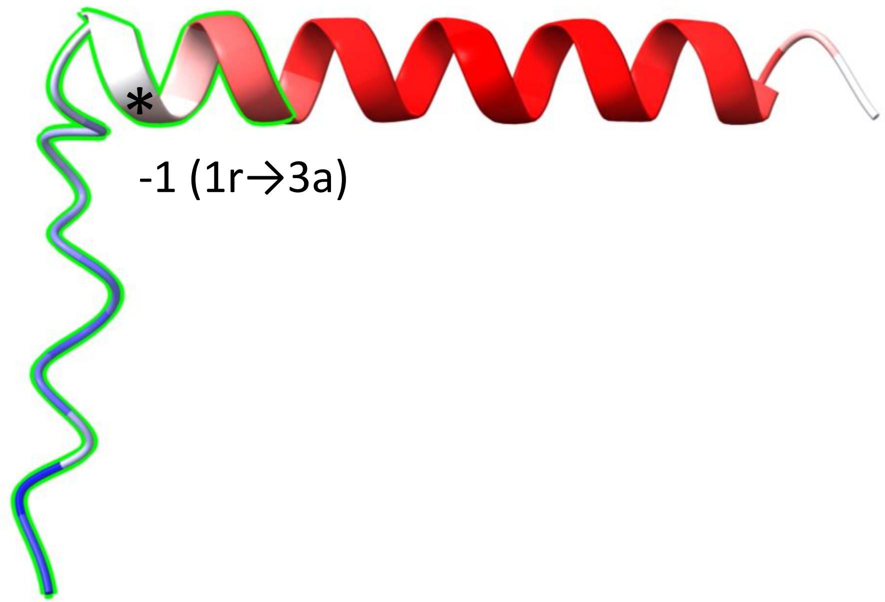

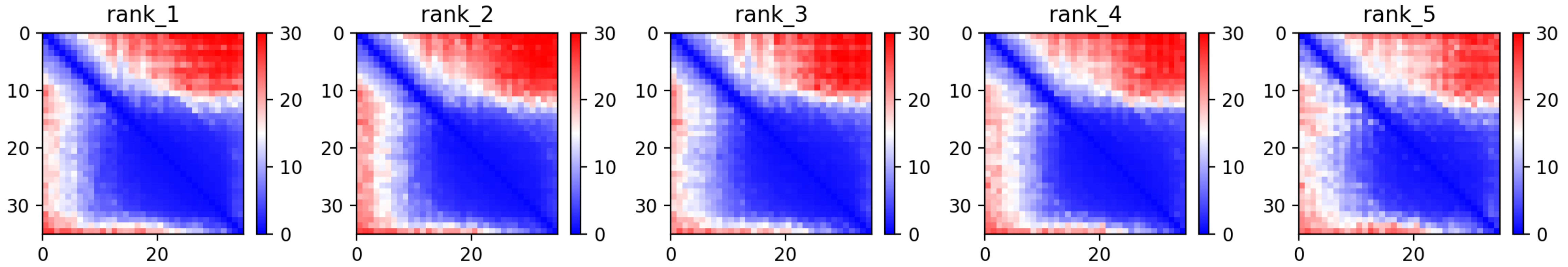

CP74: MtrunA17_Chr4g0034491

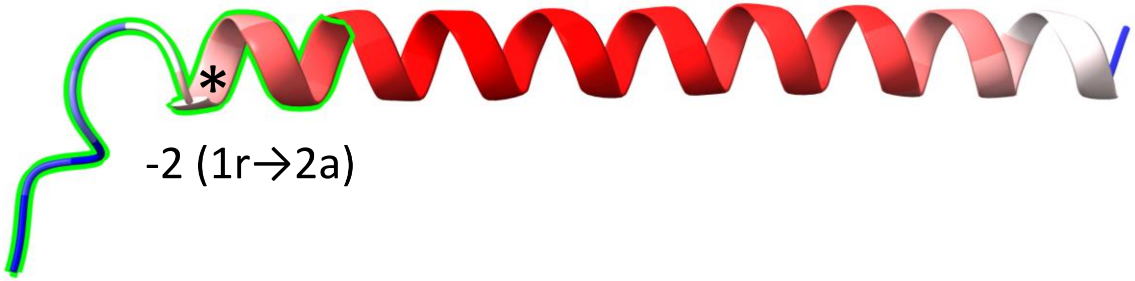

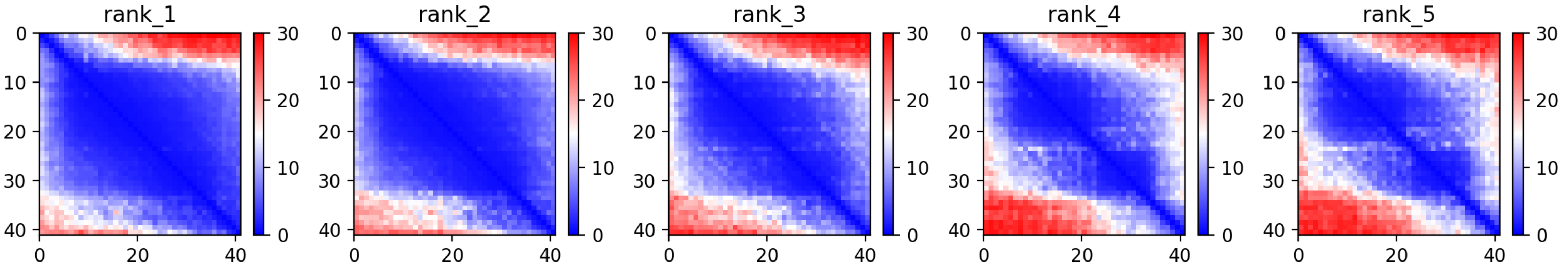

CP75: MtrunA17_Chr4g0037381

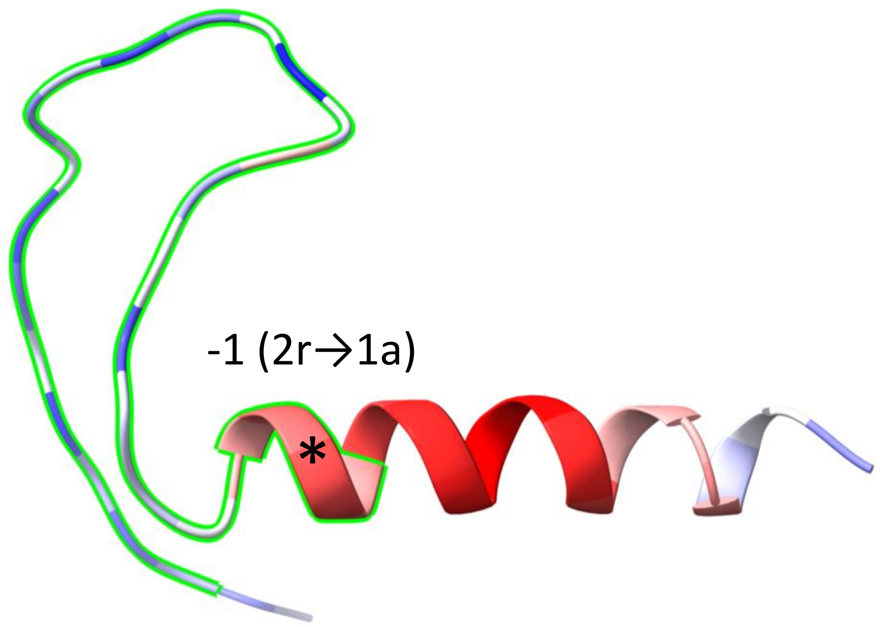

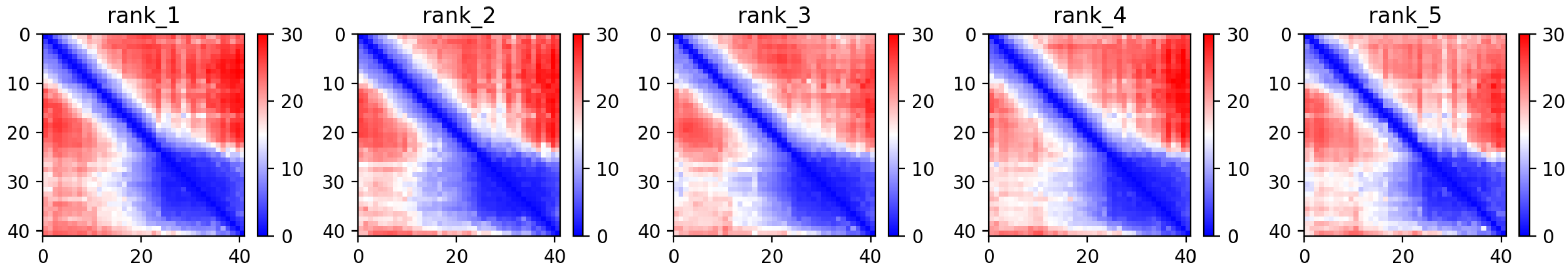

CP76: MtrunA17_Chr4g0040471

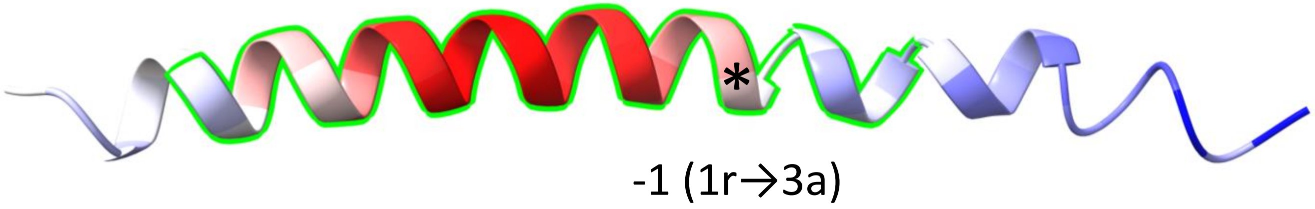

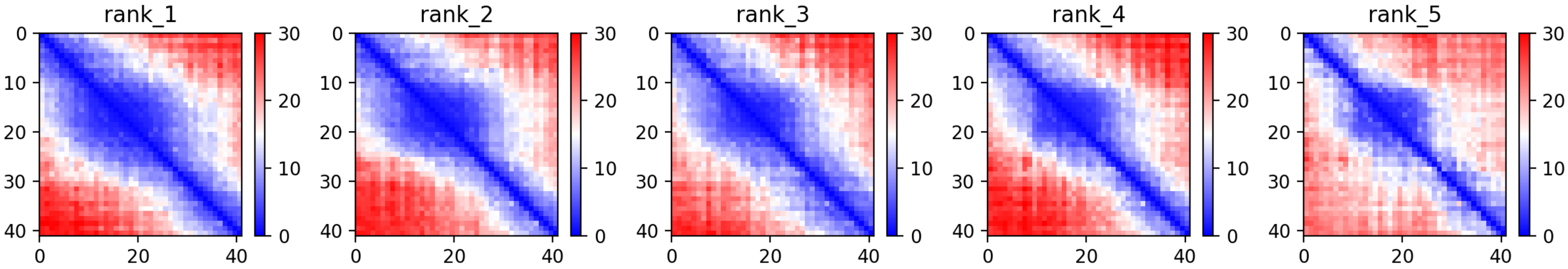

CP77: MtrunA17_Chr4g0055331

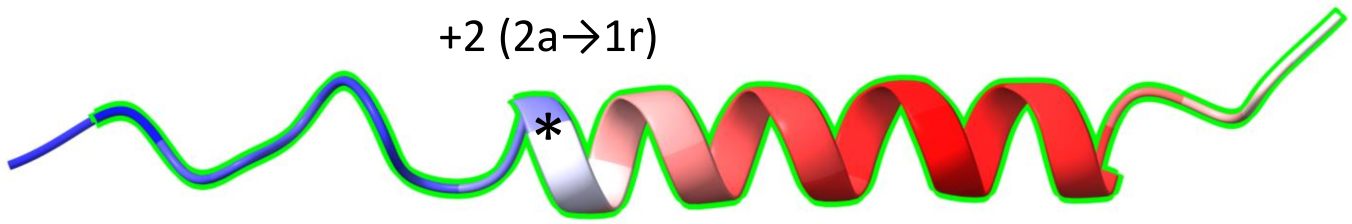

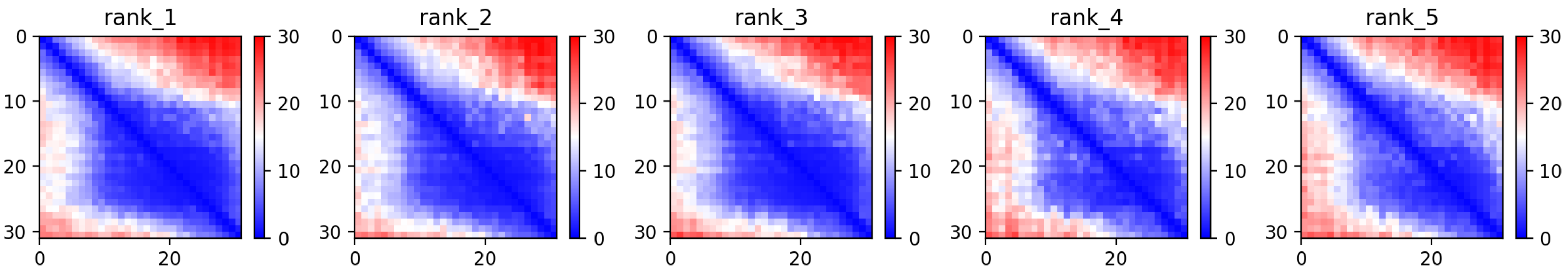

CP82: MtrunA17_Chr5g0400181

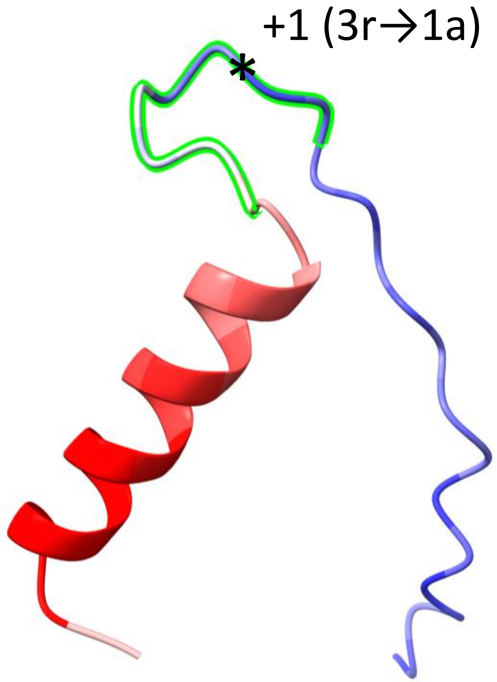

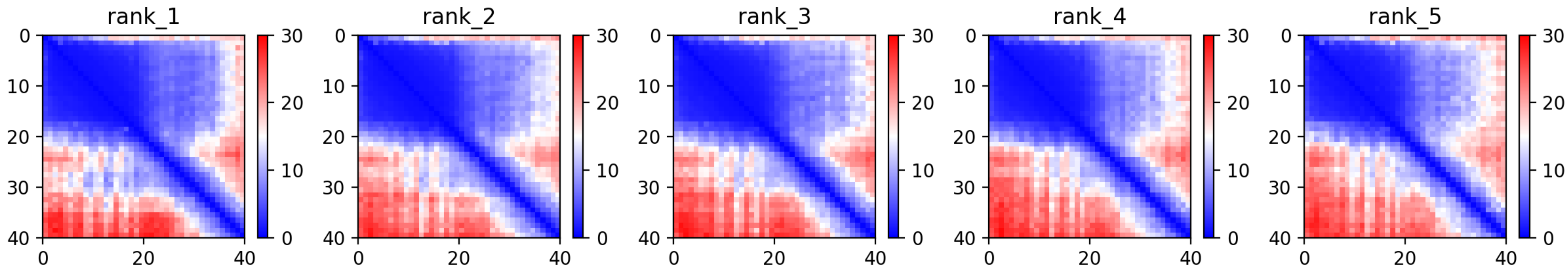

CP83: MtrunA17_Chr5g0405061

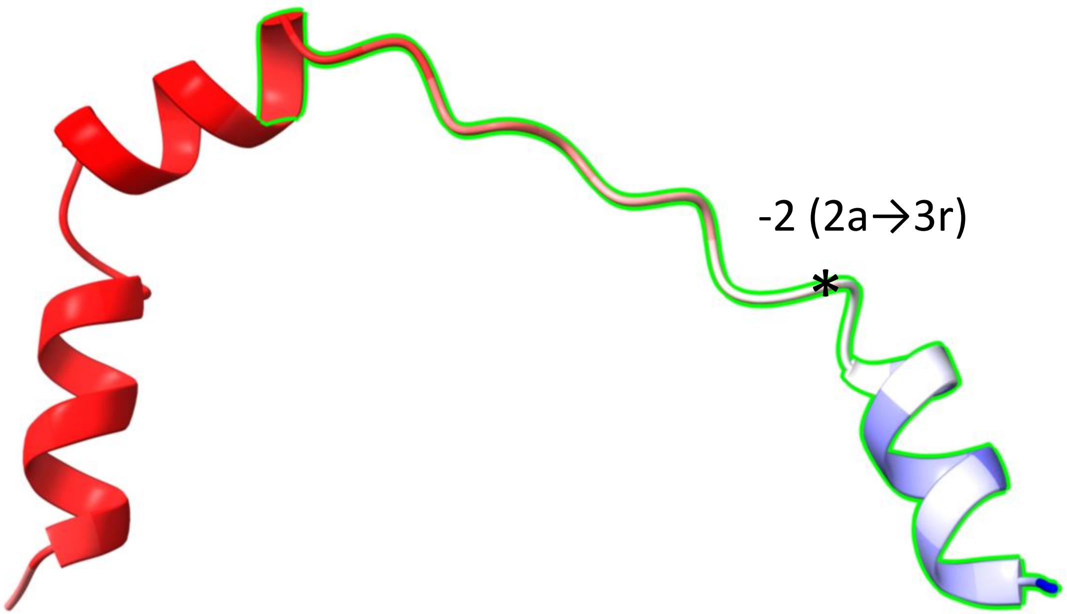

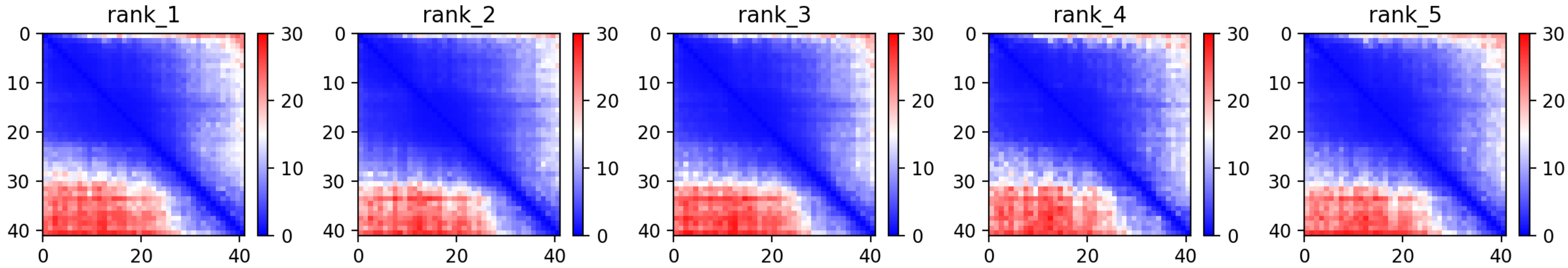

CP84: MtrunA17_Chr5g0408581

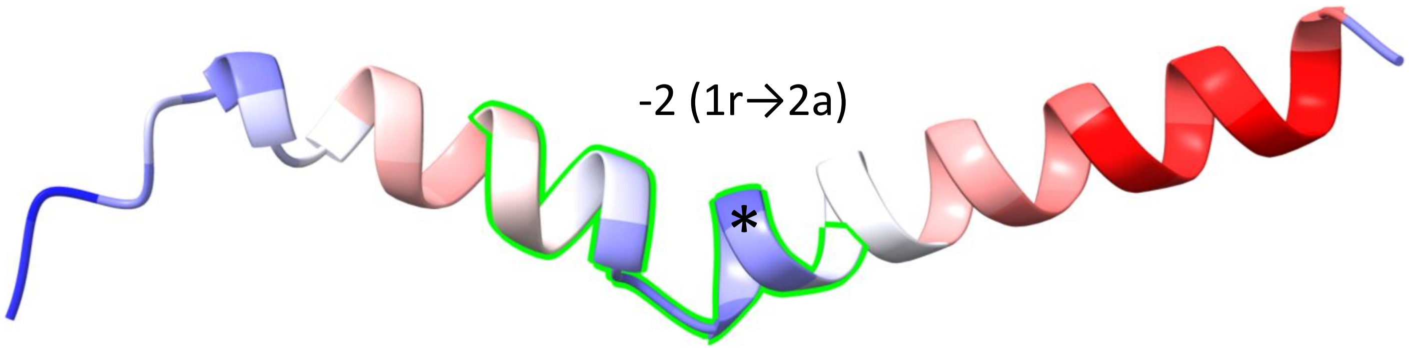

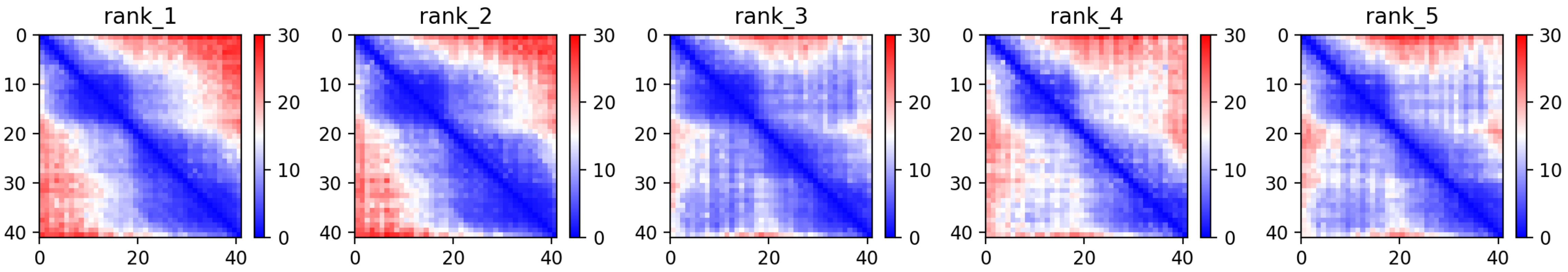

CP85: MtrunA17_Chr5g0415031

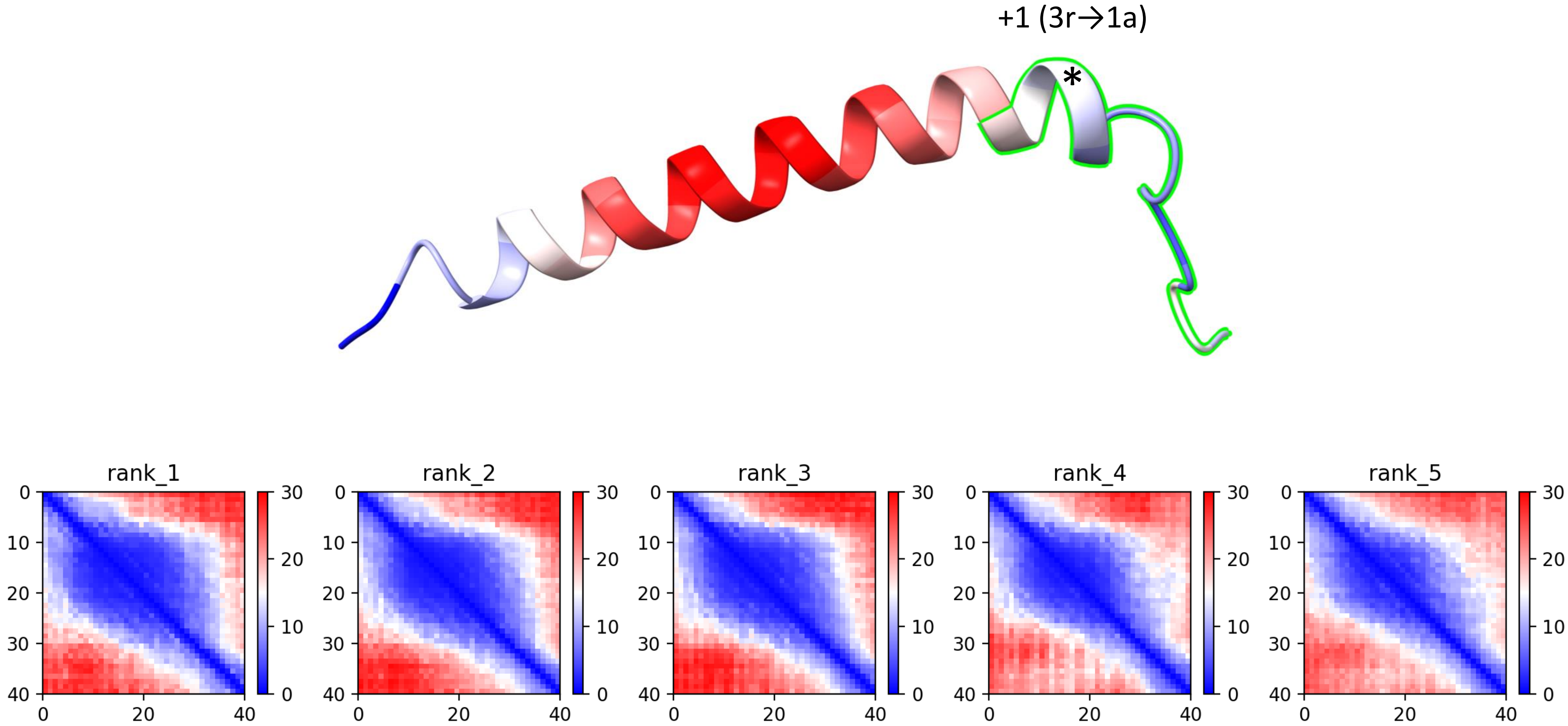

CP86: MtrunA17_Chr5g0415911

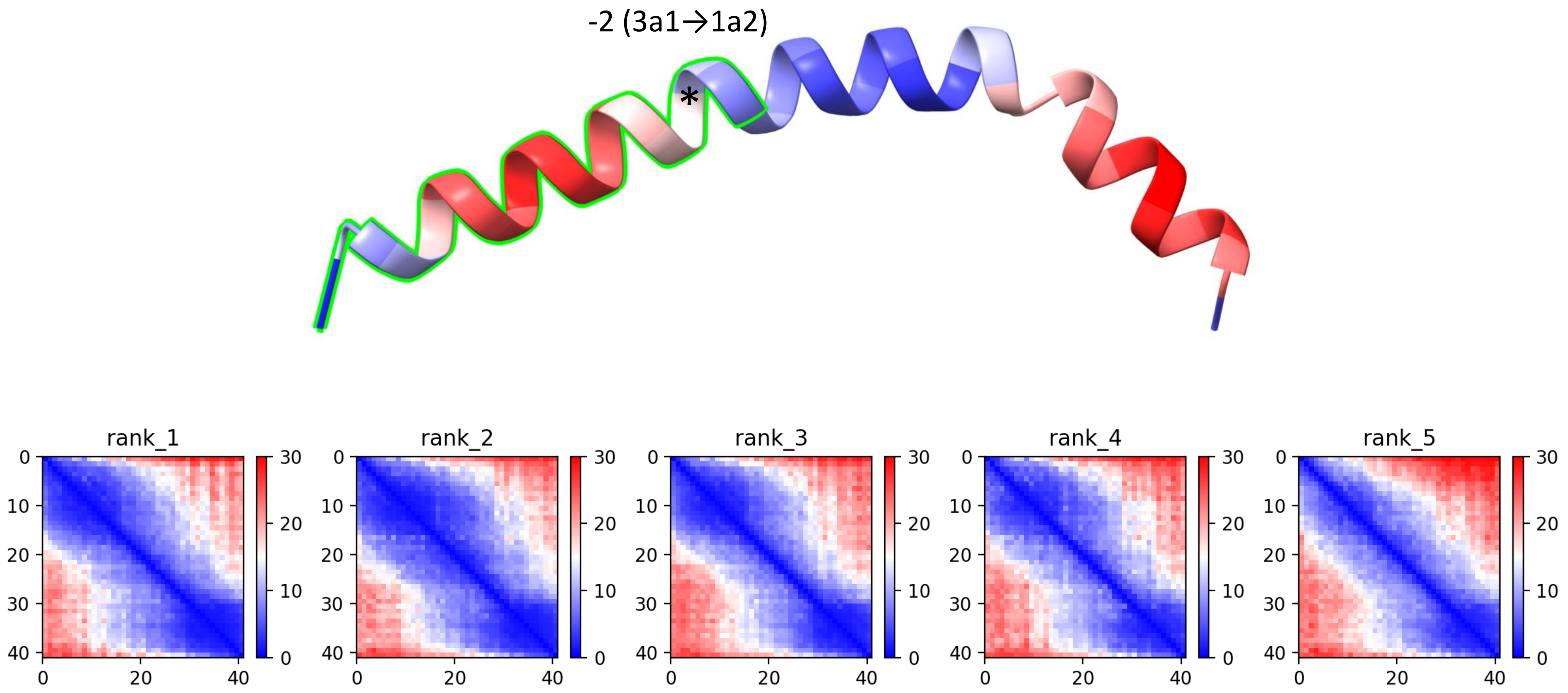

CP87: MtrunA17_Chr5g0421761

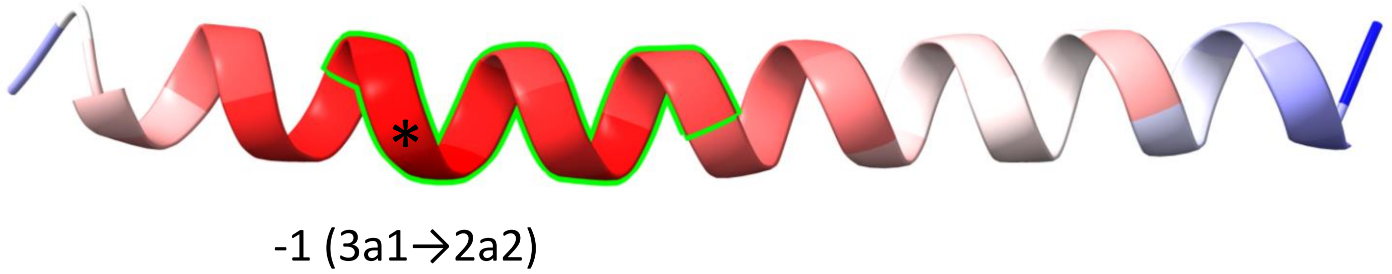

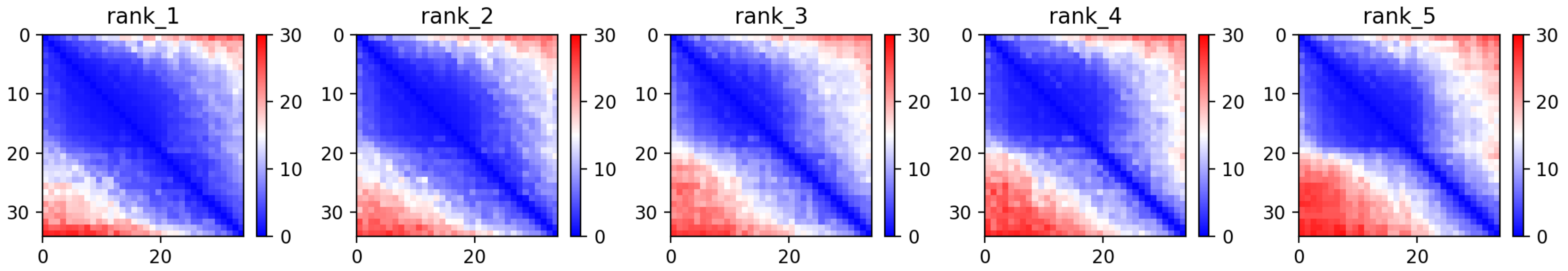

CP88: MtrunA17_Chr5g0422291

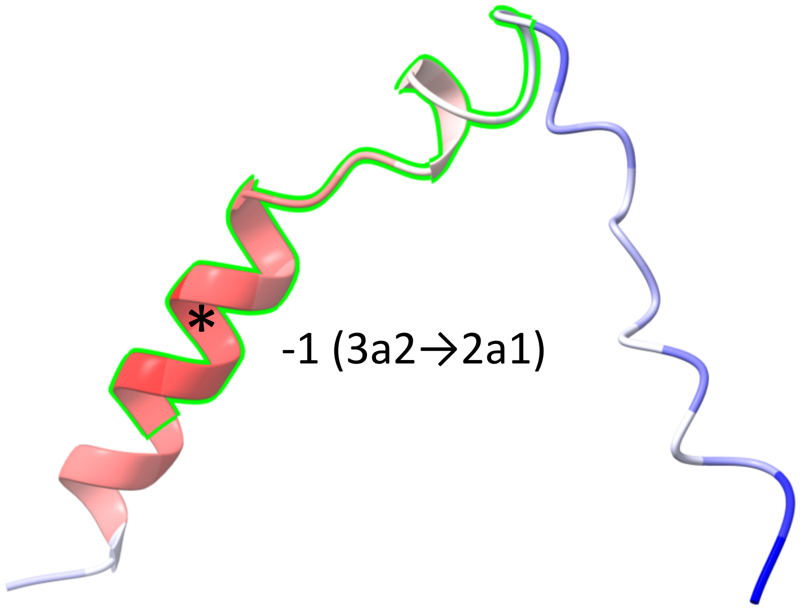

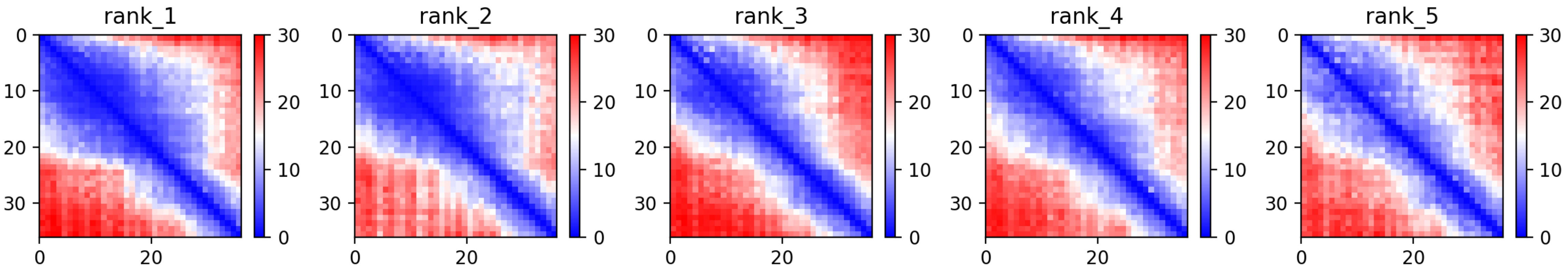

CP90: MtrunA17_Chr5g0430341

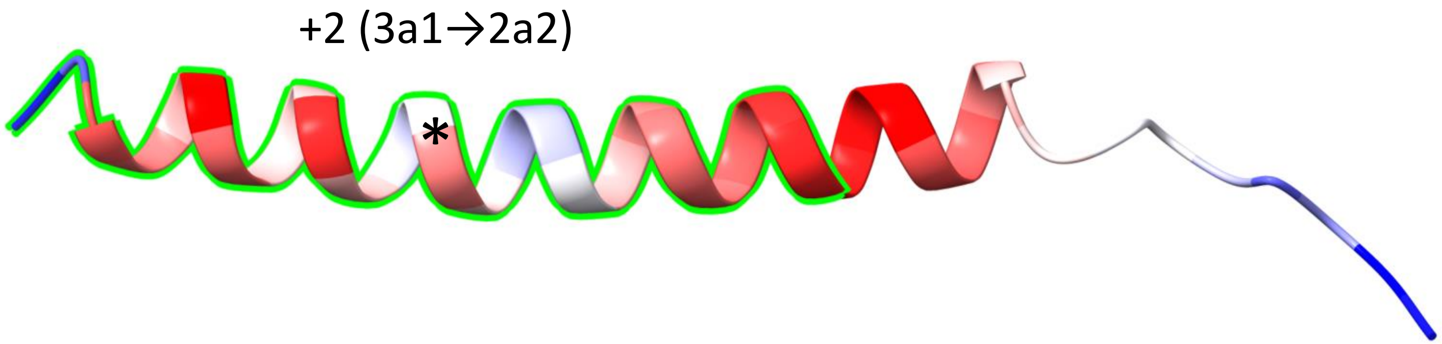

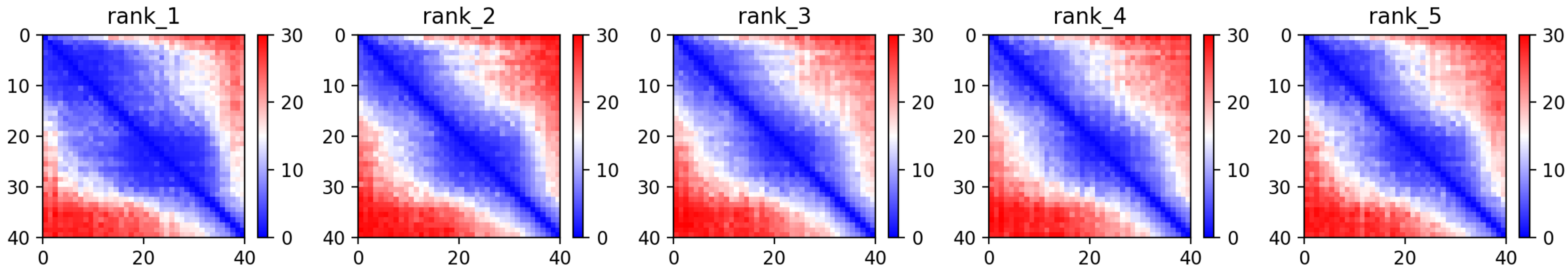

CP92: MtrunA17_Chr5g0431401

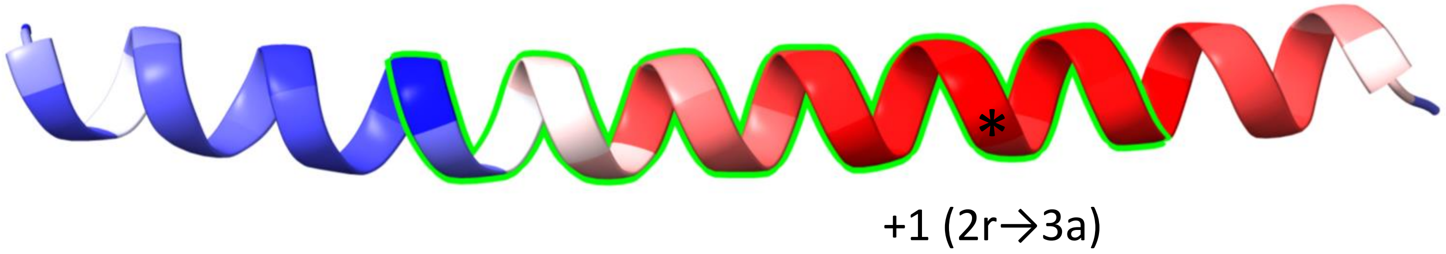

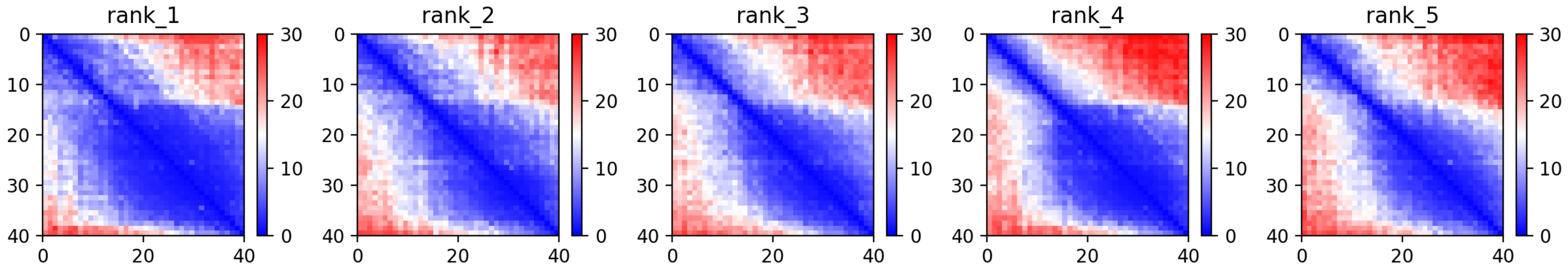

CP93: MtrunA17_Chr5g0435191

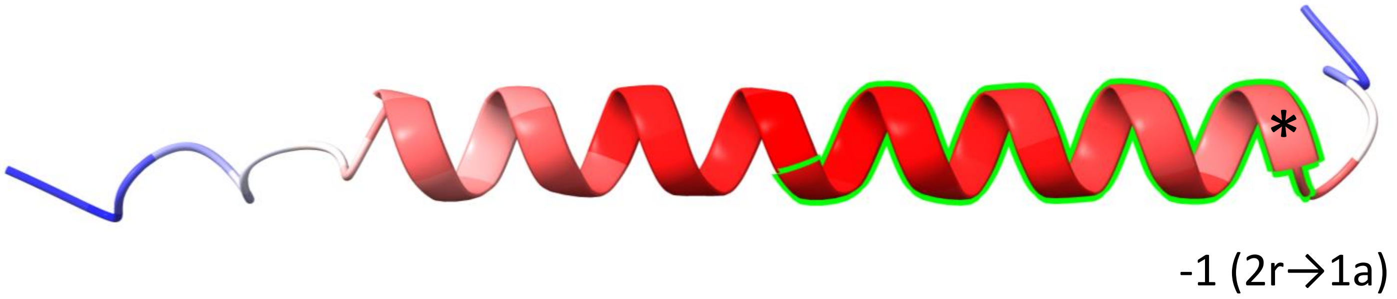

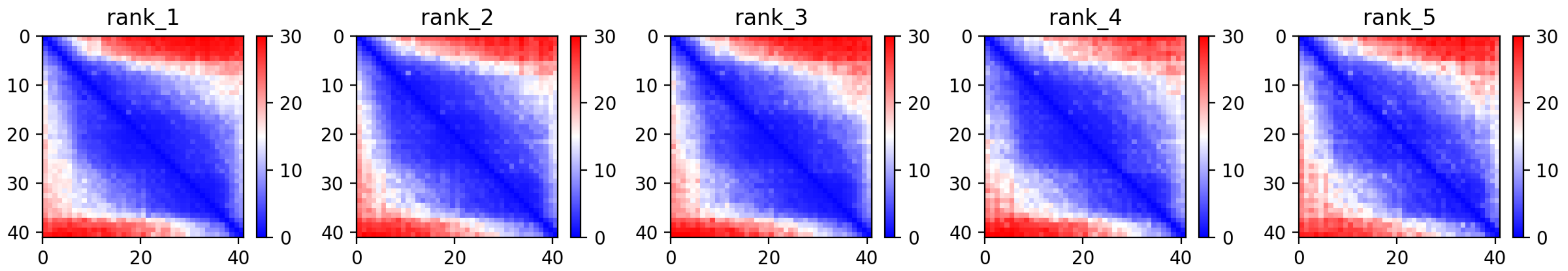

CP94: MtrunA17_Chr5g0444231

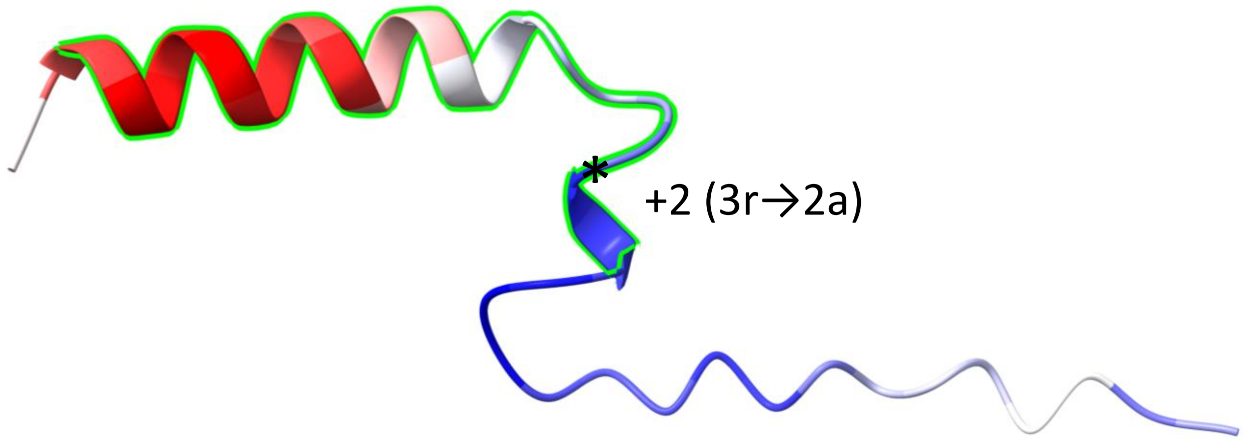

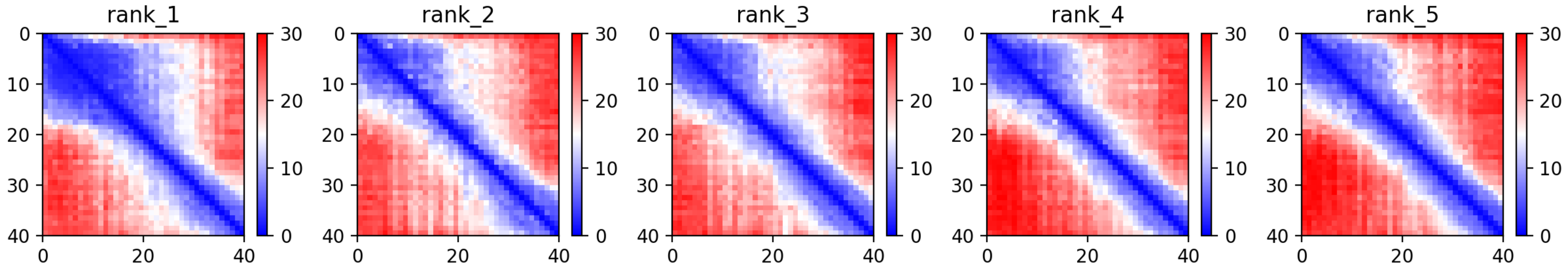

CP98: MtrunA17_Chr6g0457461

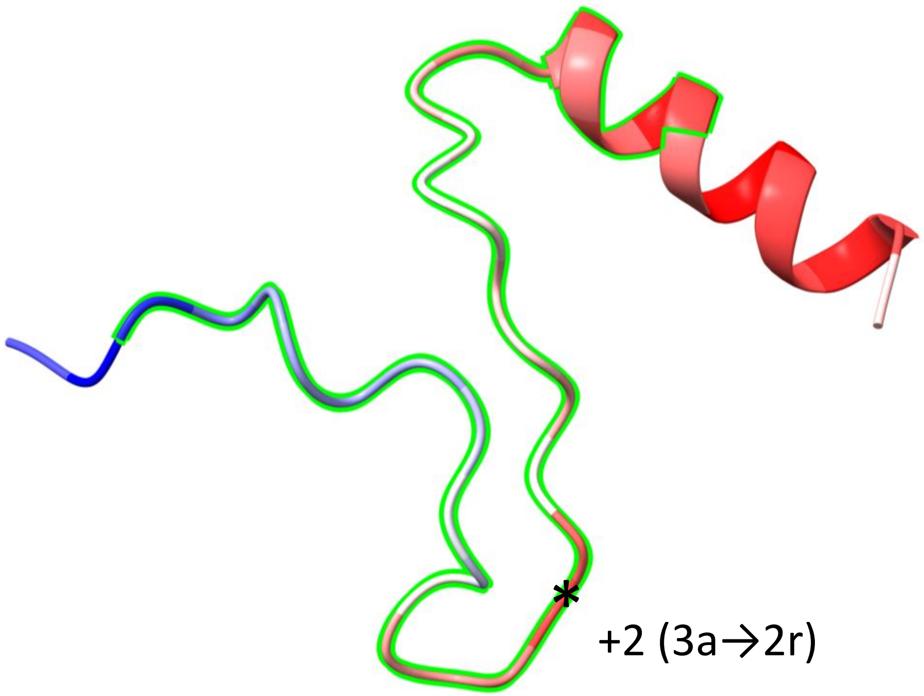

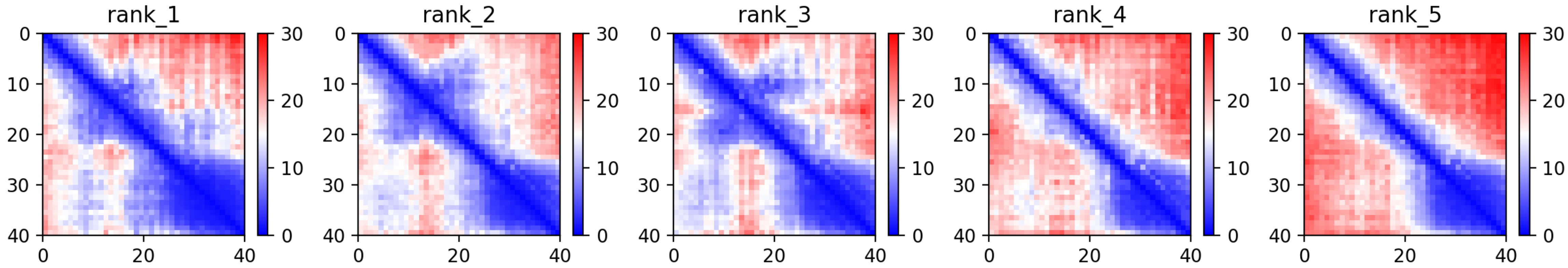

CP102: MtrunA17_Chr6g0459481

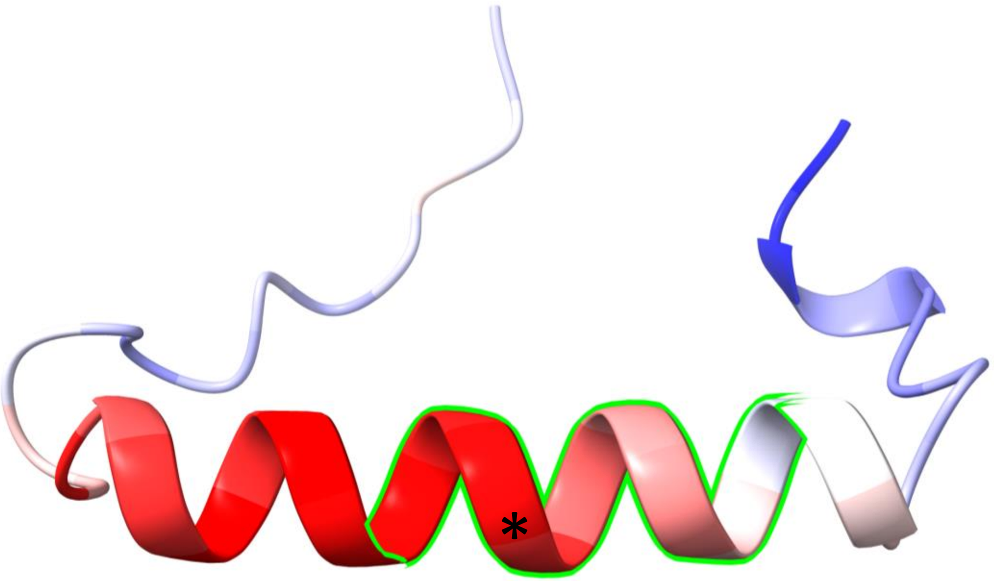

-1 (1r→3a)

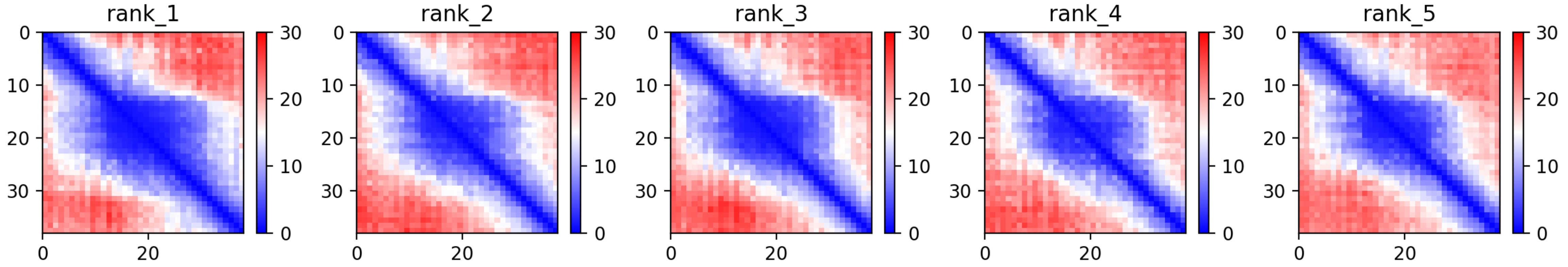

CP104: MtrunA17_Chr6g0462271

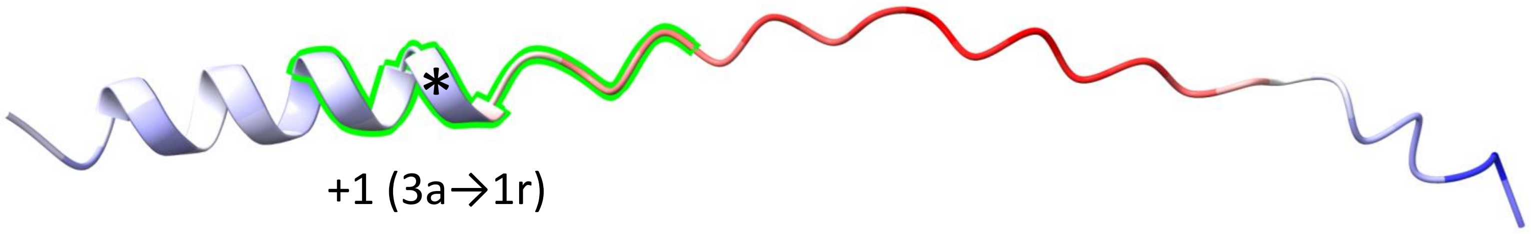

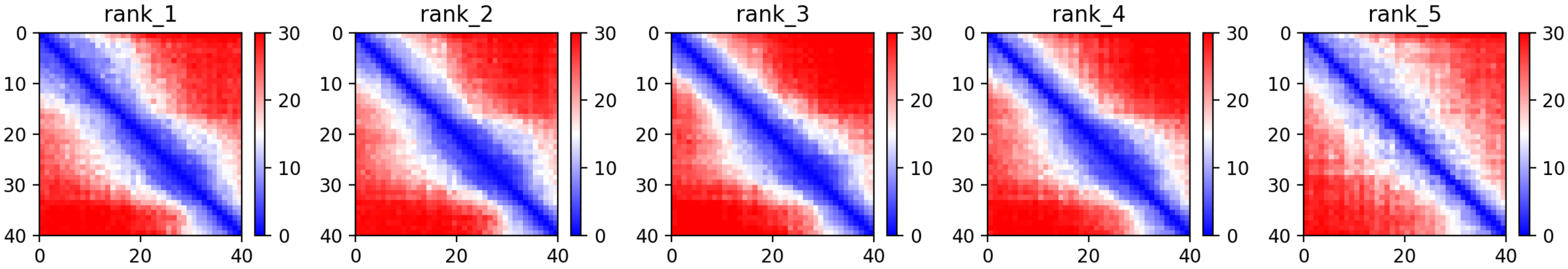

CP105: MtrunA17_Chr6g0468411

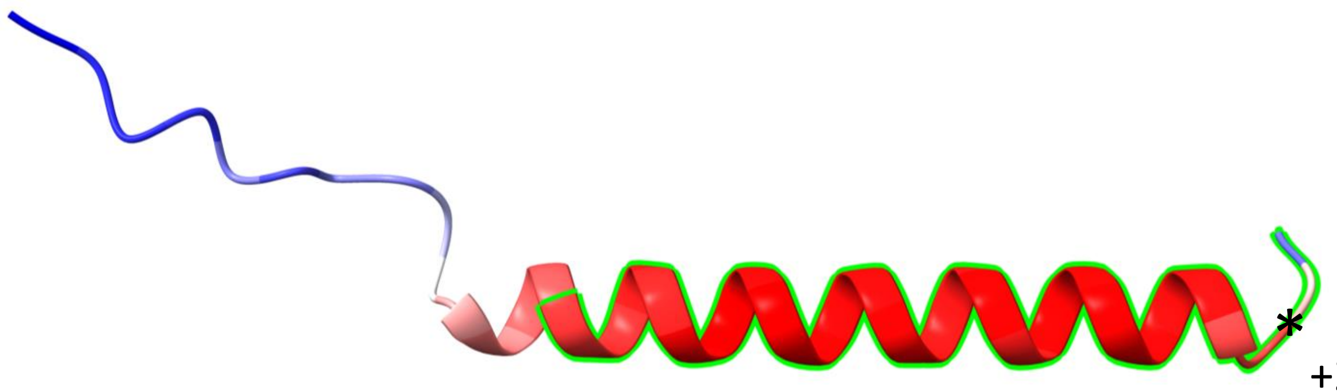

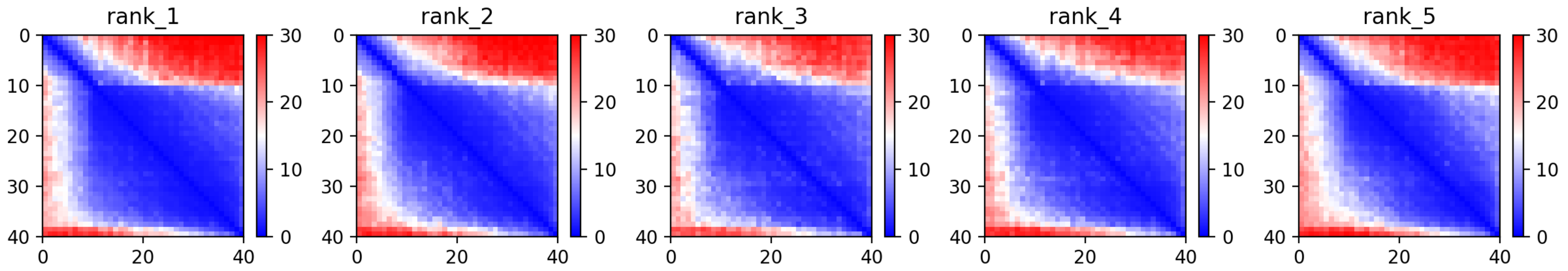

CP106: MtrunA17_Chr6g0476751

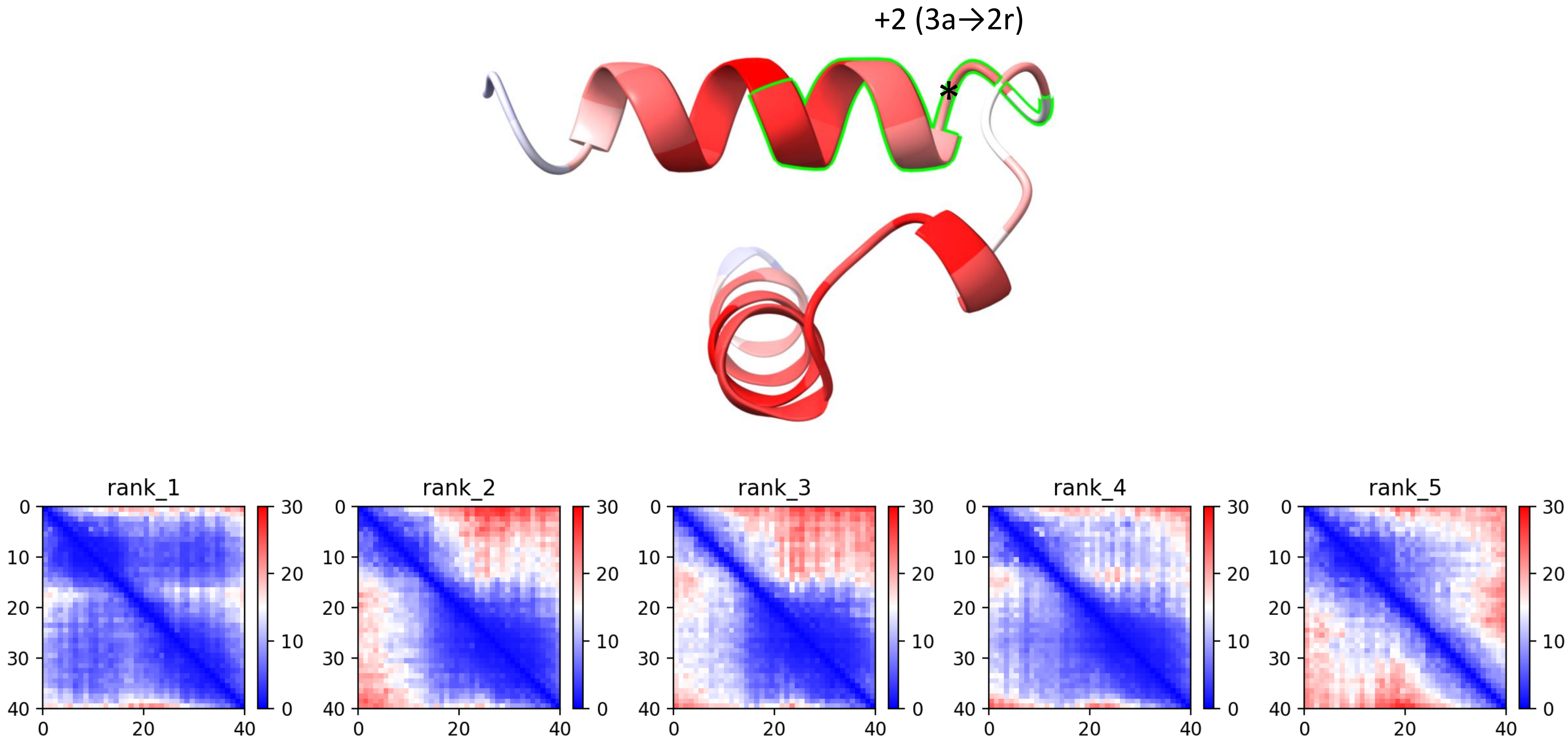

CP107: MtrunA17_Chr6g0479001

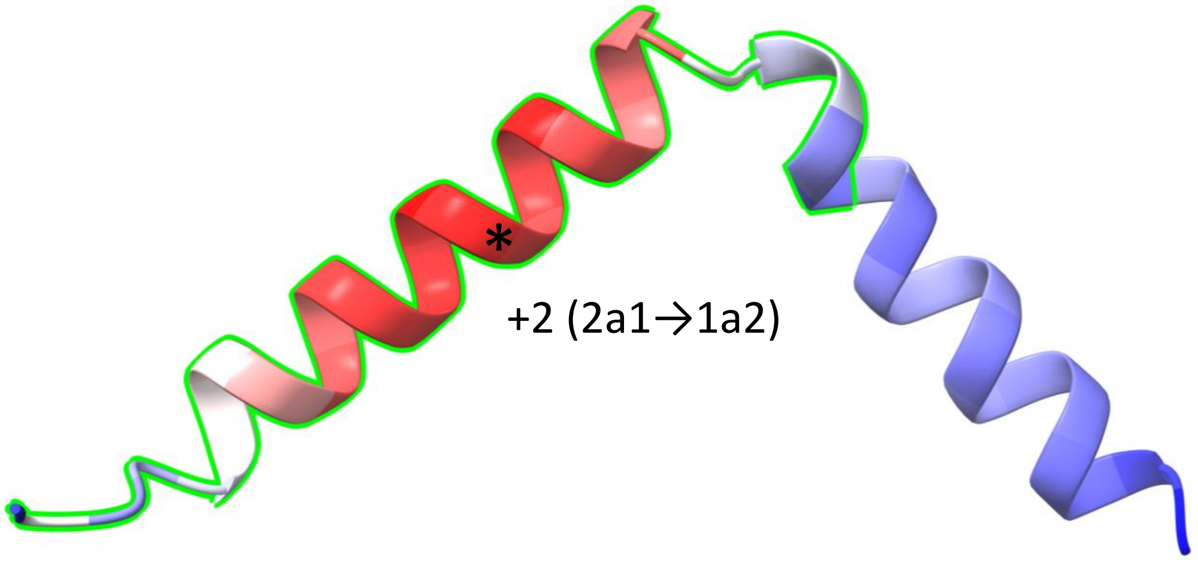

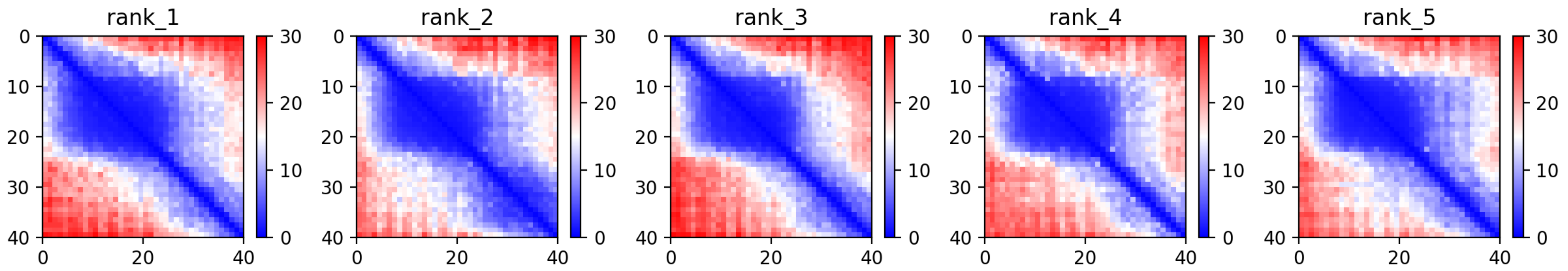

CP109: MtrunA17_Chr6g0486961

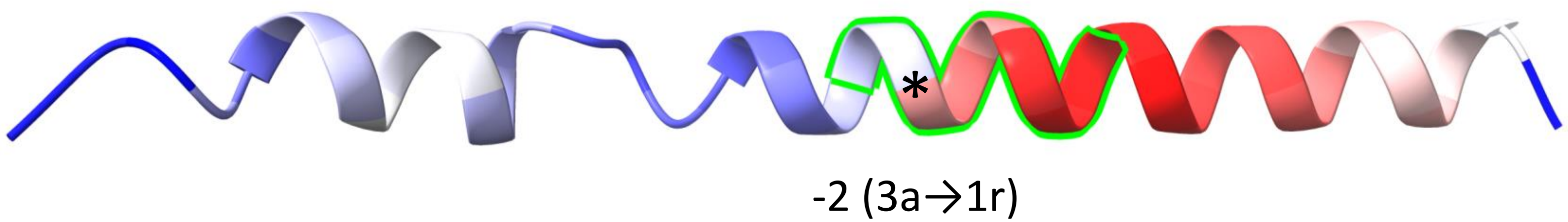

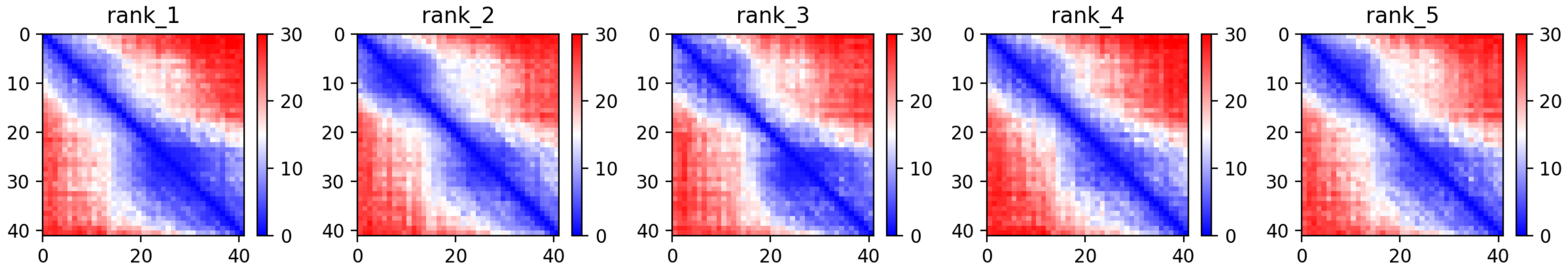

CP110: MtrunA17_Chr7g0214741

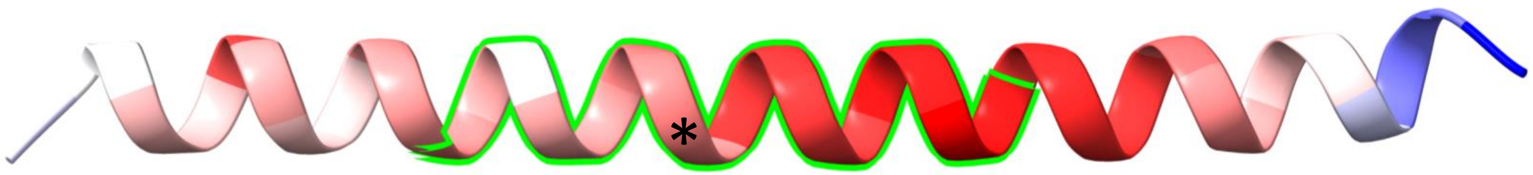

+1 (3r→1a)

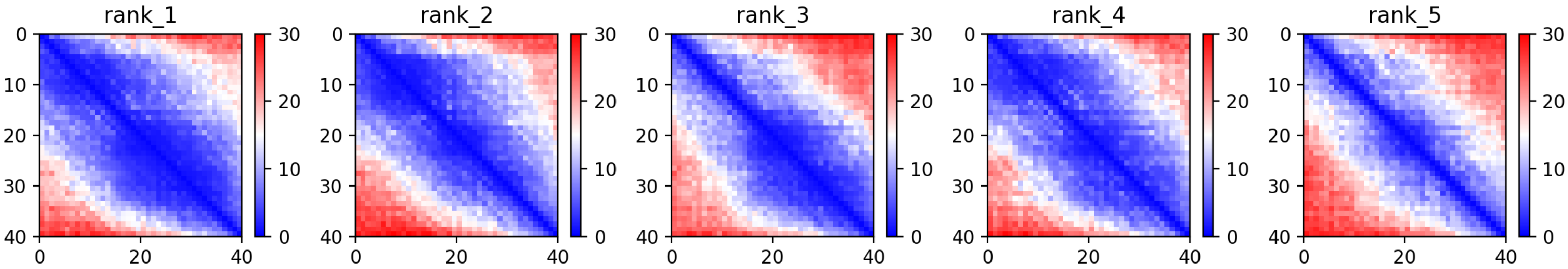

CP111: MtrunA17_Chr7g0214911

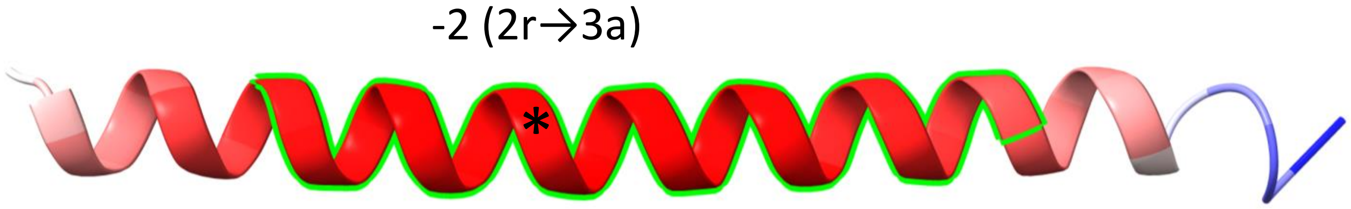

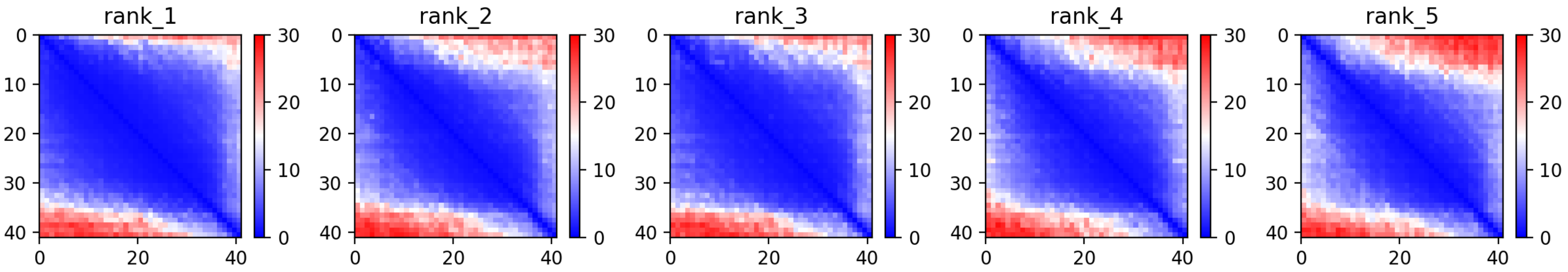

CP112: MtrunA17_Chr7g0219691

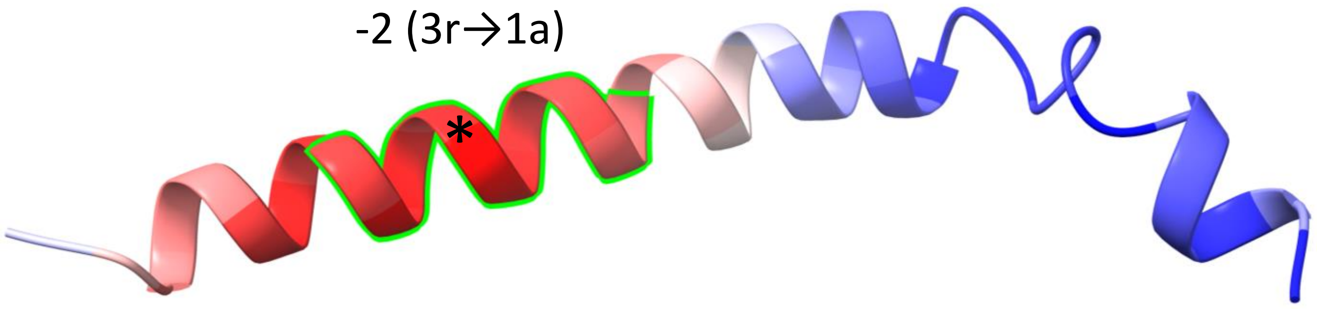

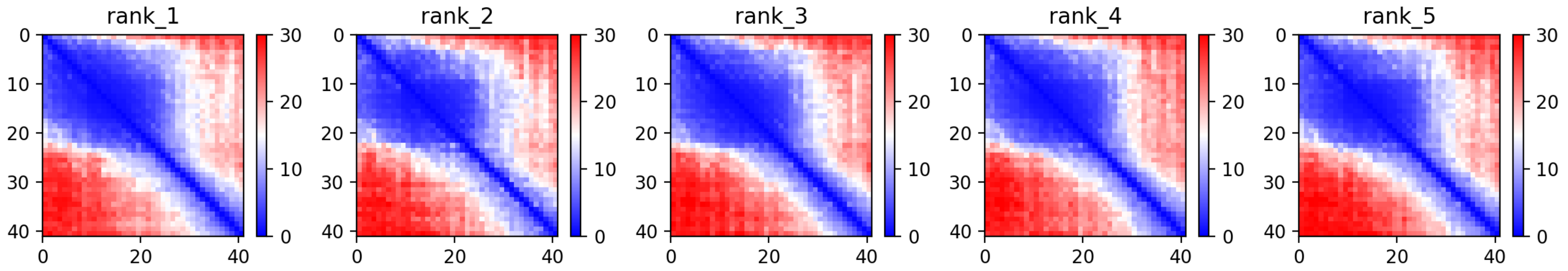

CP113: MtrunA17_Chr7g0221631

-1 (1r→3a)

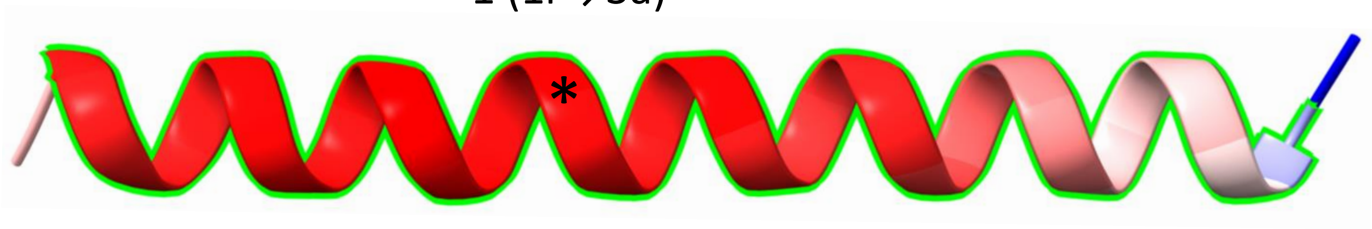

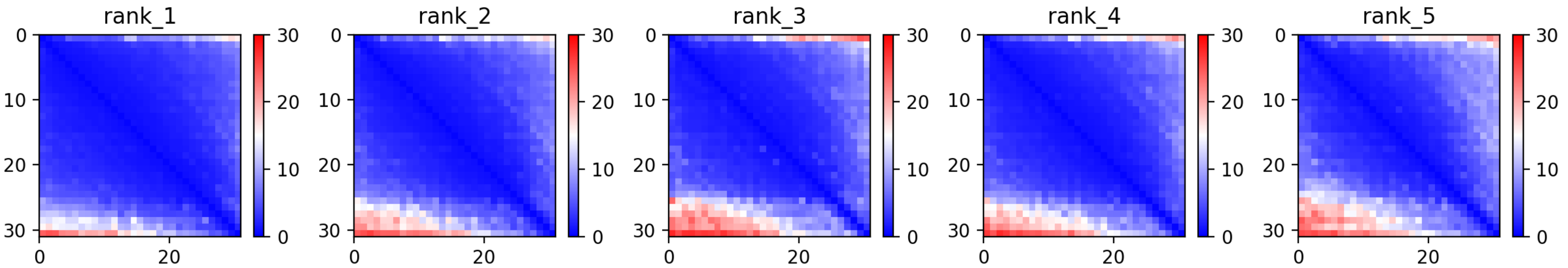

CP115: MtrunA17_Chr7g0230341

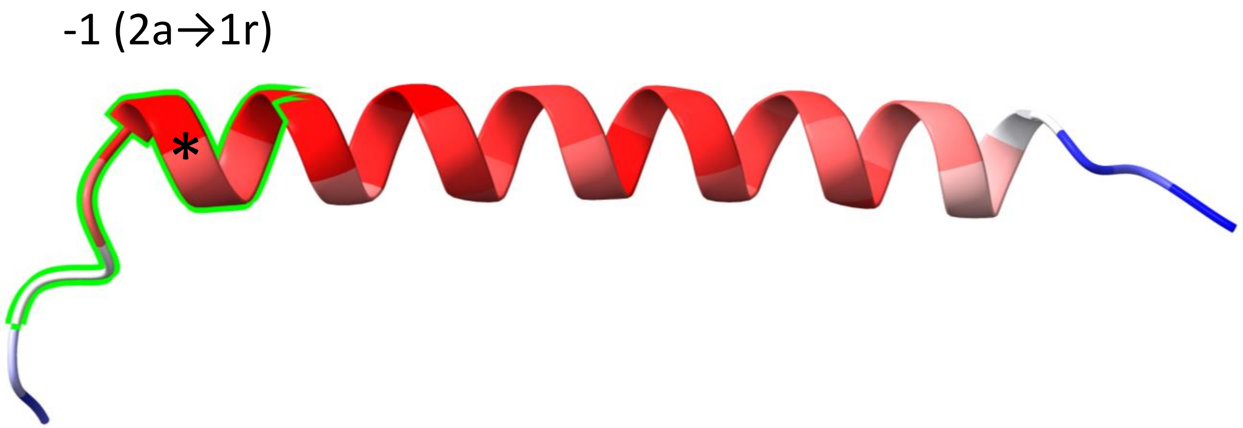

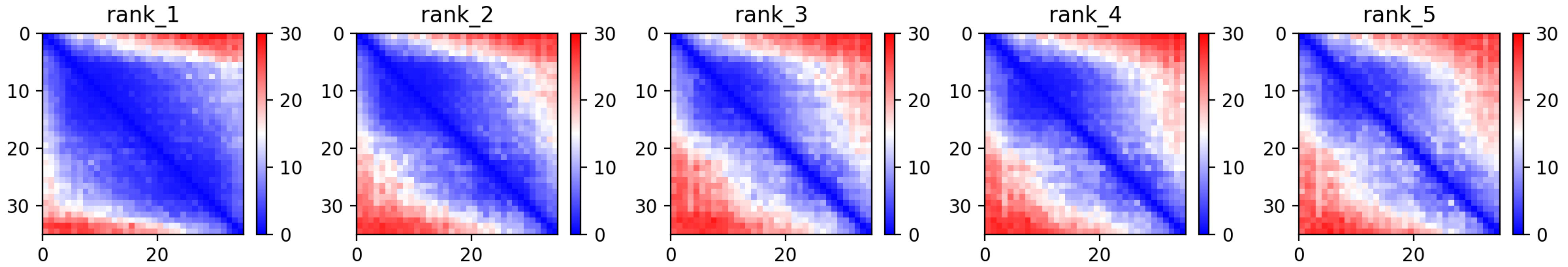

CP117: MtrunA17_Chr7g0237331

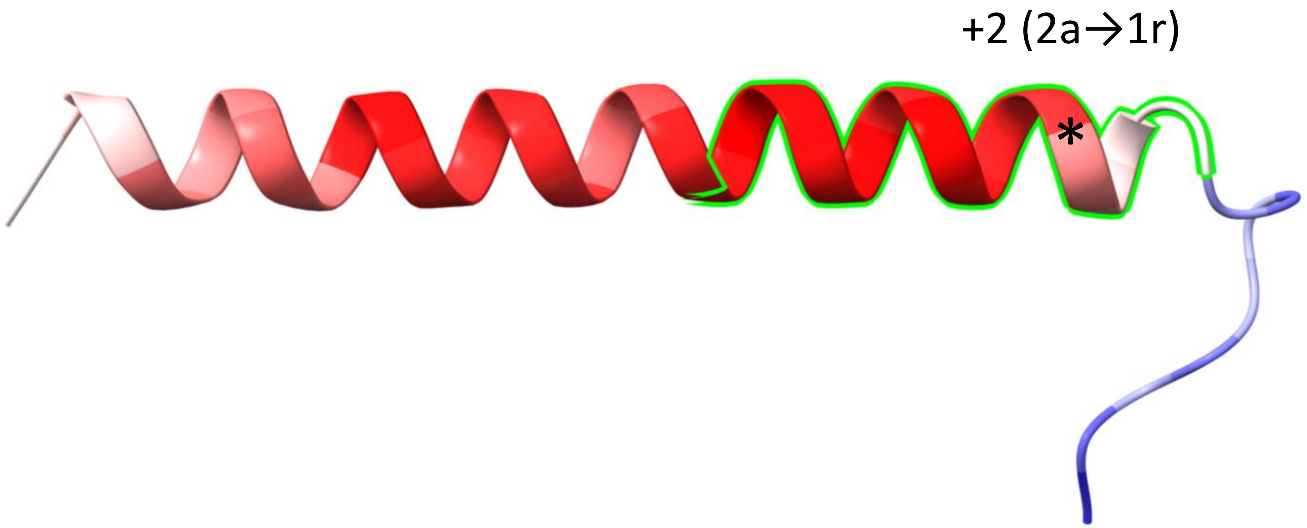

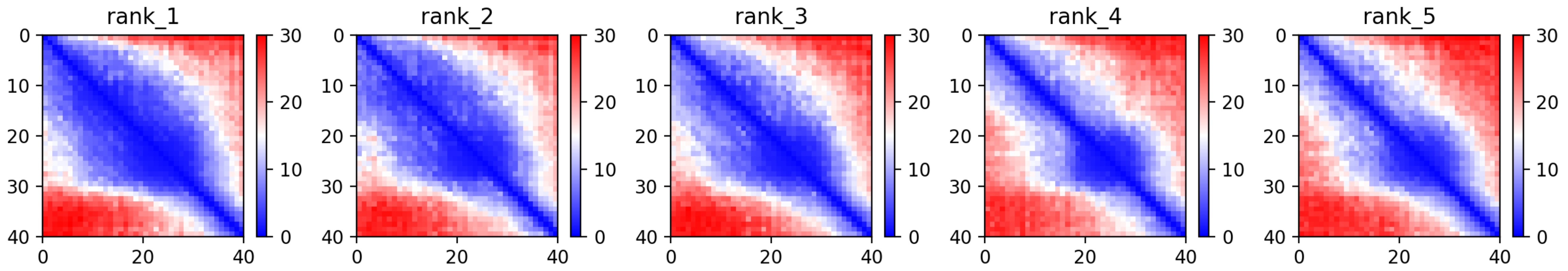

CP118: MtrunA17_Chr7g0251971

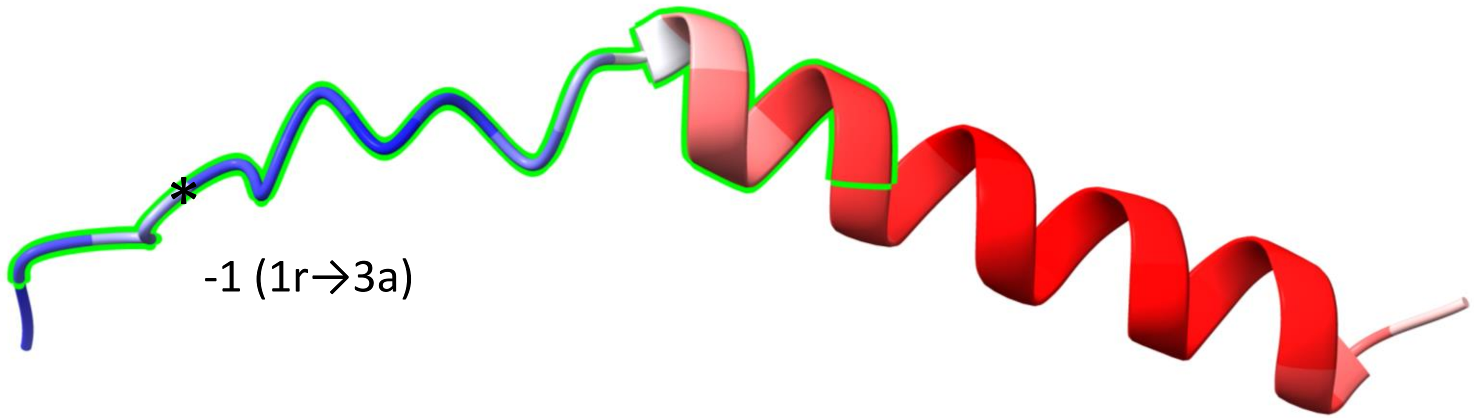

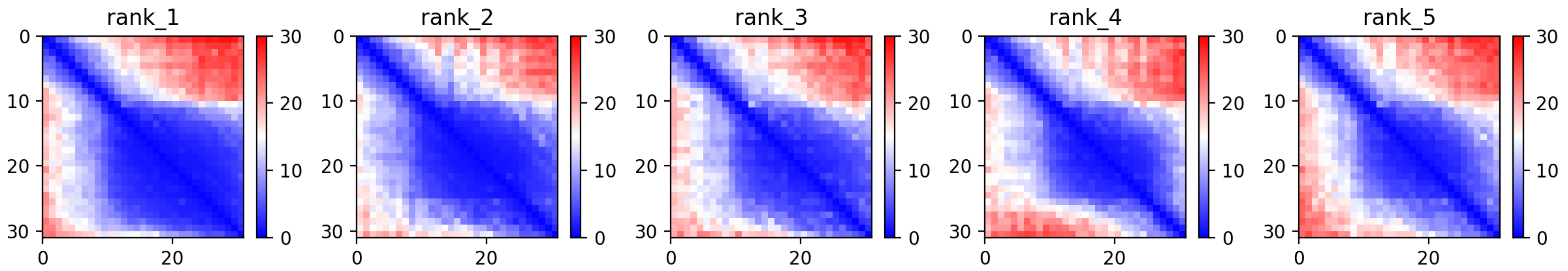

CP119: MtrunA17_Chr7g0259611

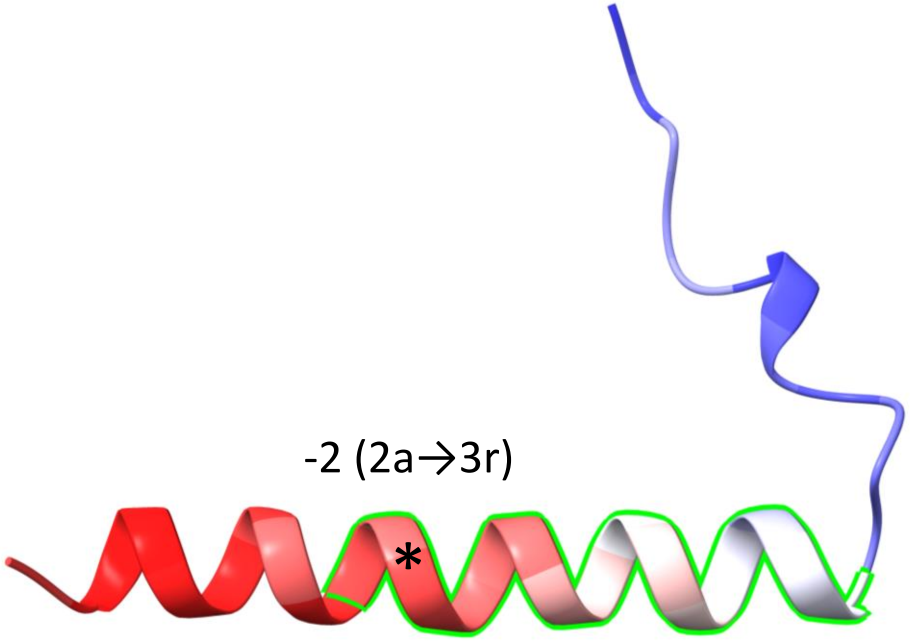

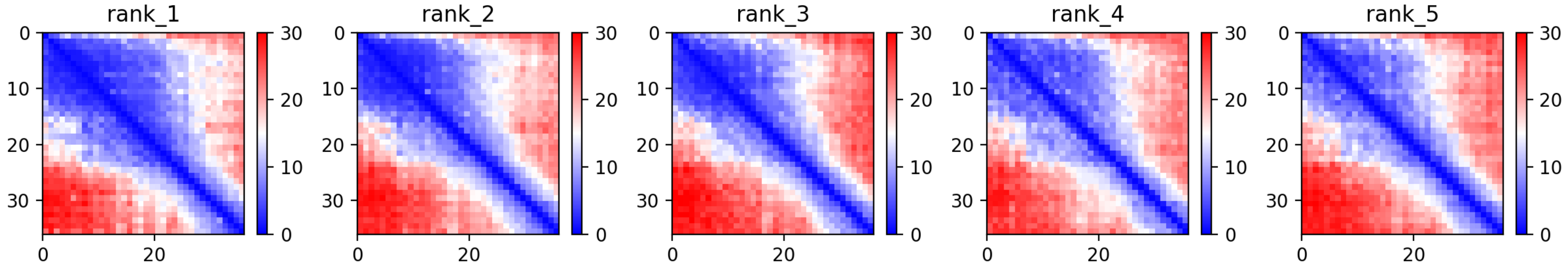

CP120: MtrunA17_Chr7g0262821

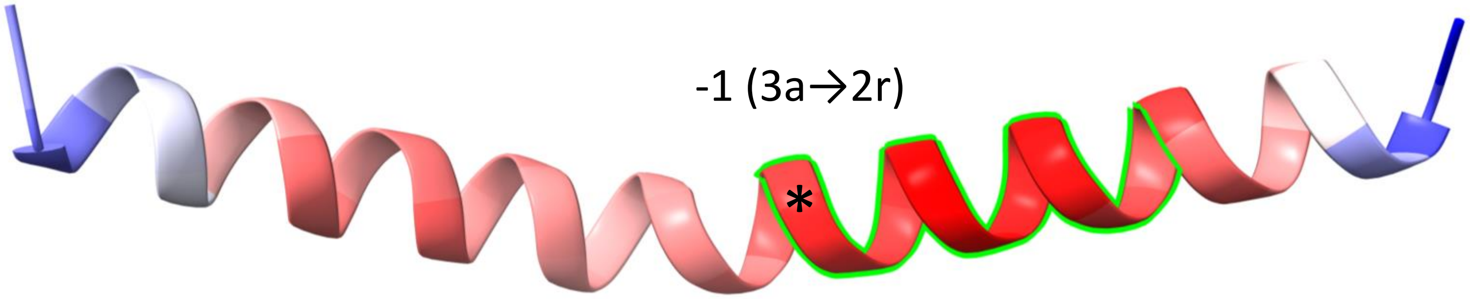

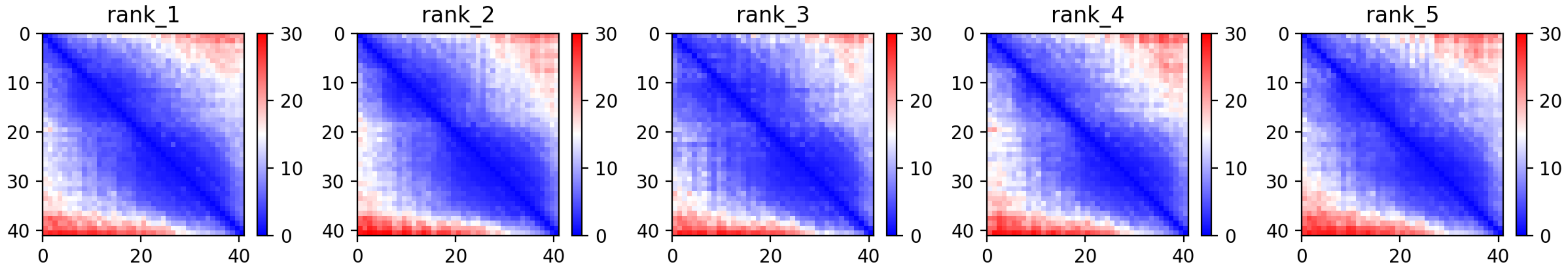

CP121: MtrunA17_Chr7g0270811

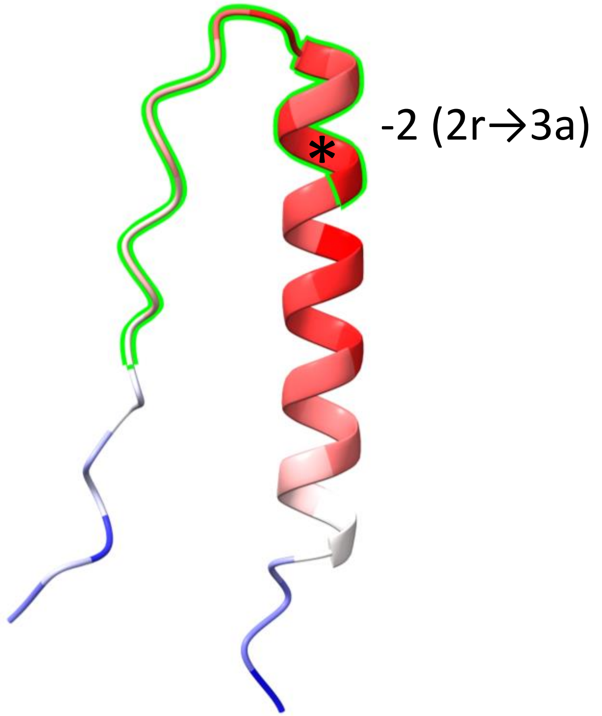

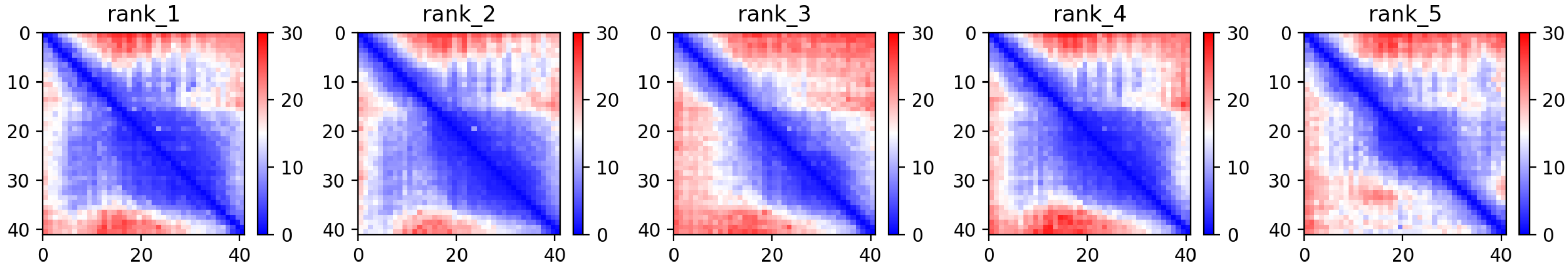

CP122: MtrunA17_Chr7g1034306

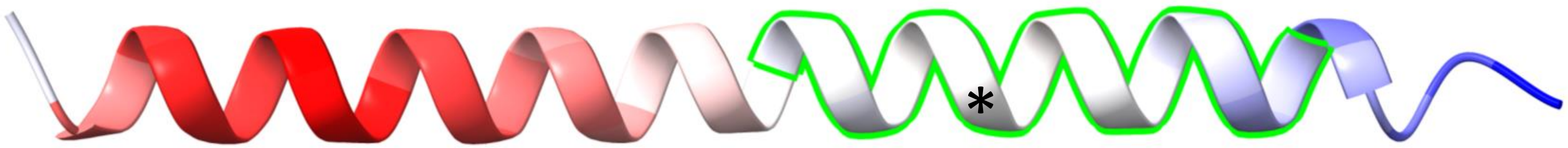

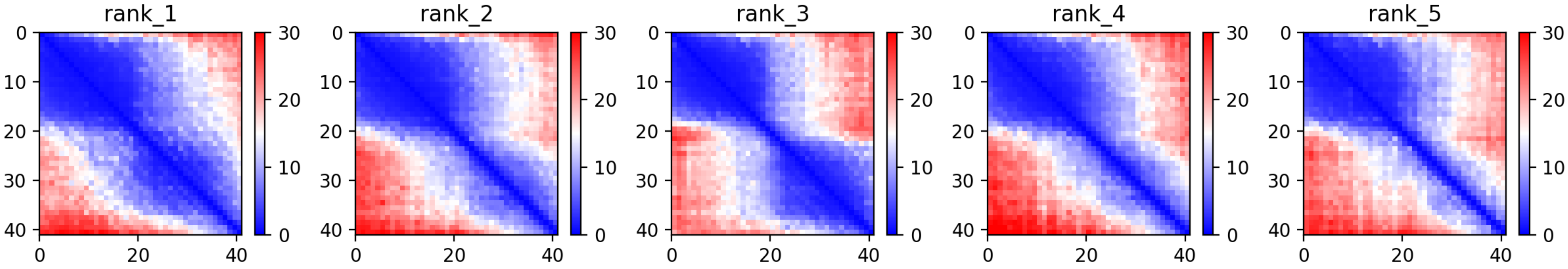

CP123: MtrunA17_Chr8g0338111

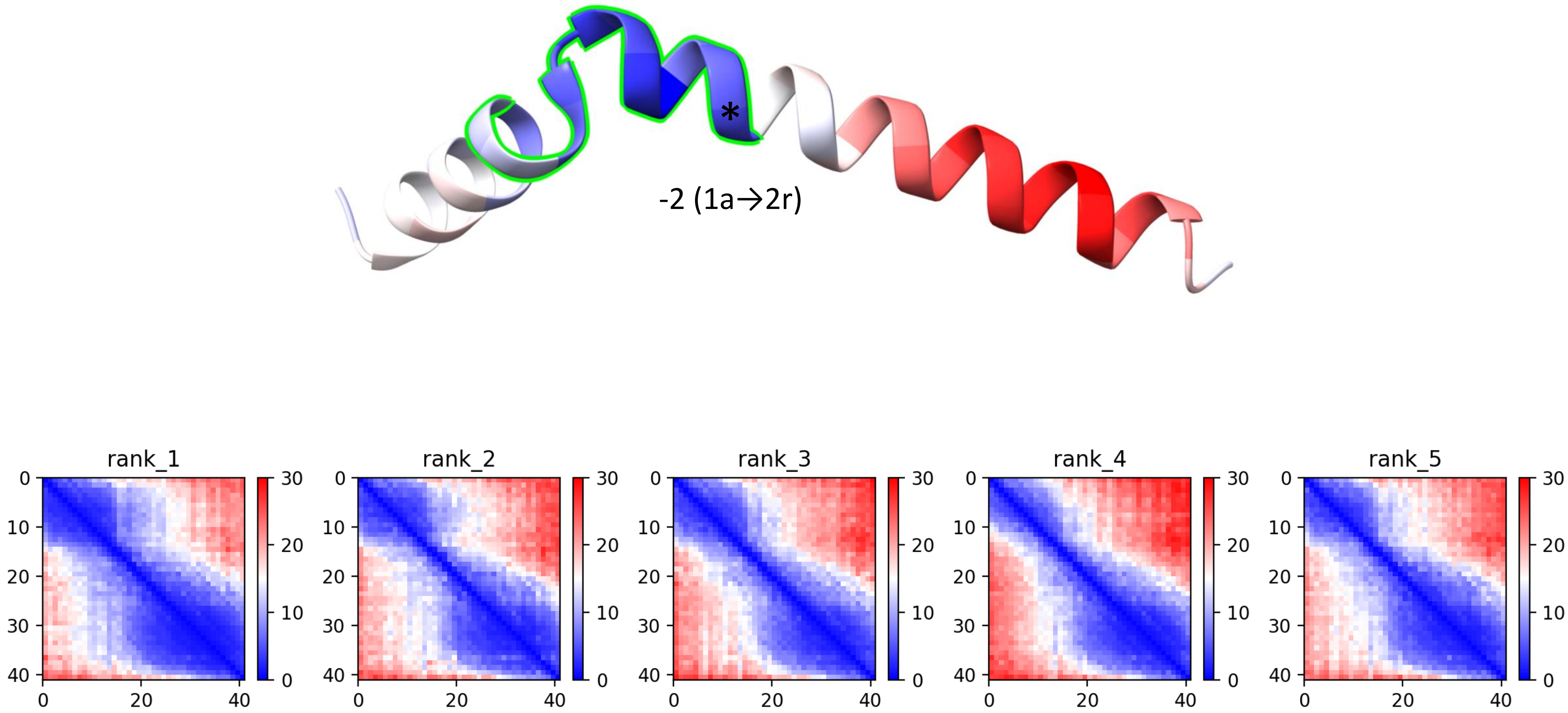

CP126: MtrunA17_Chr8g0342881

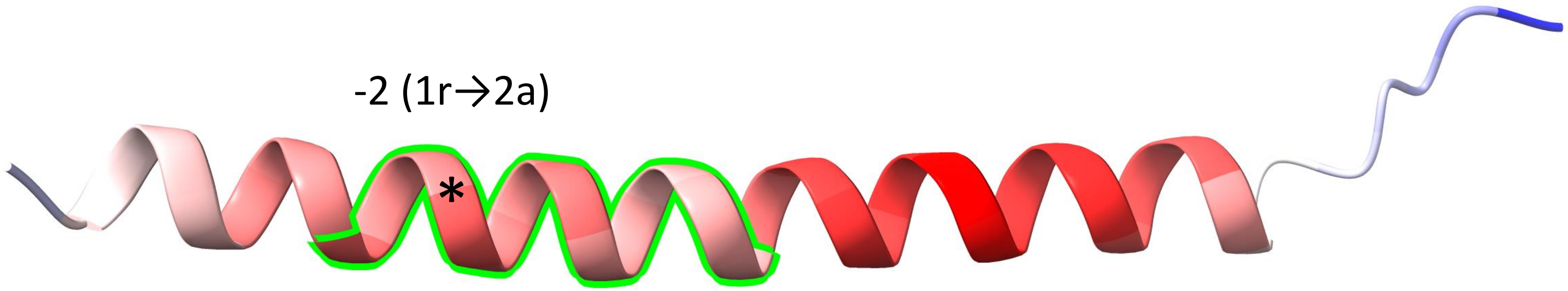

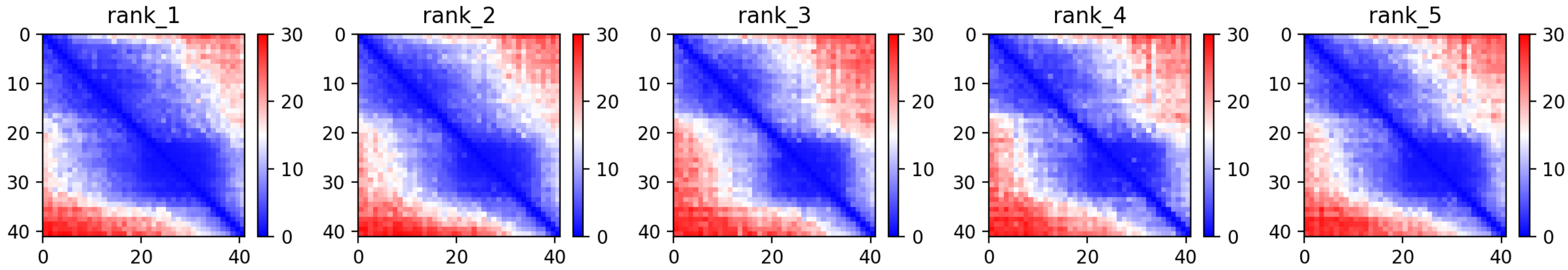

CP127: MtrunA17_Chr8g0345421

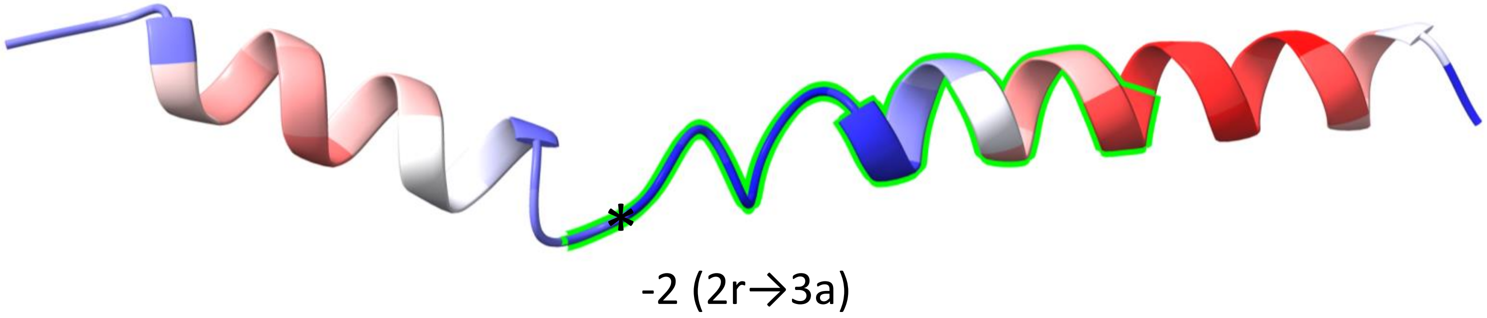

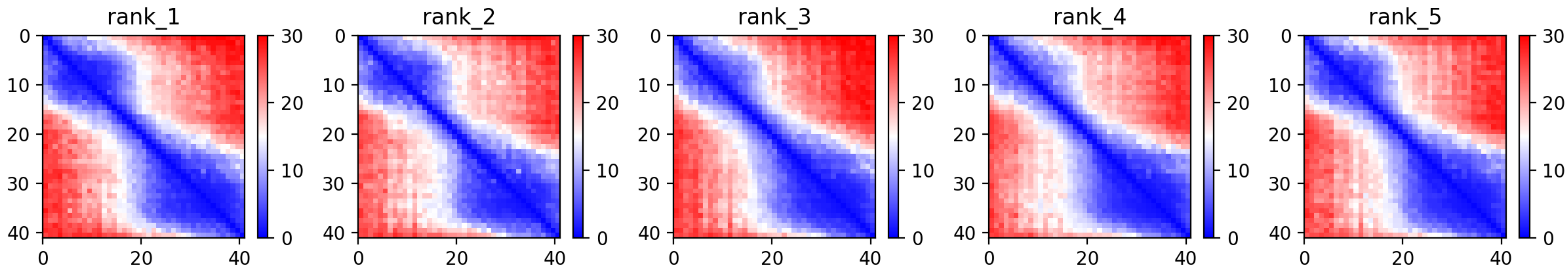

CP128: MtrunA17_Chr8g0353711

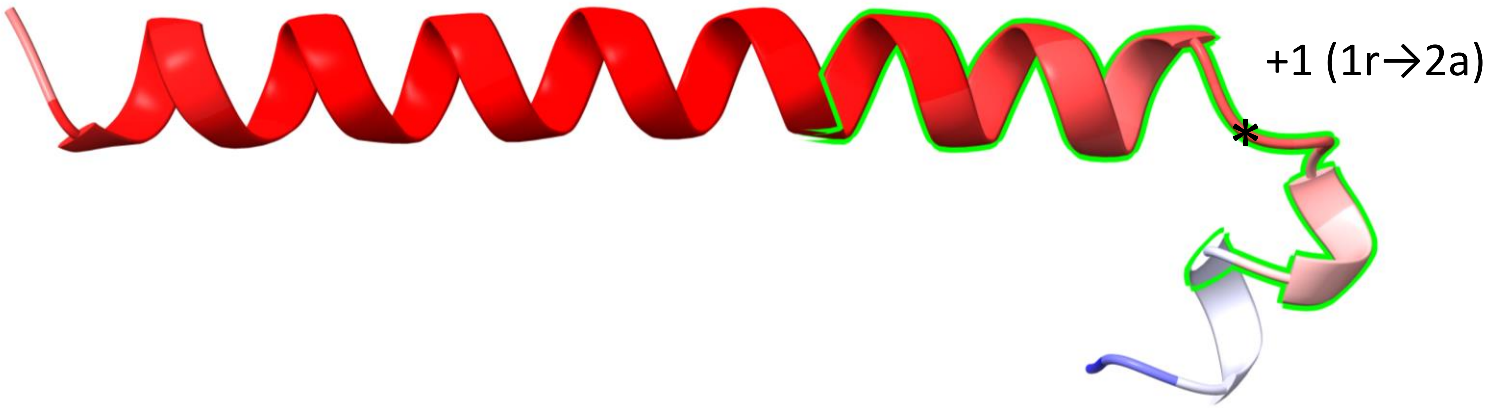

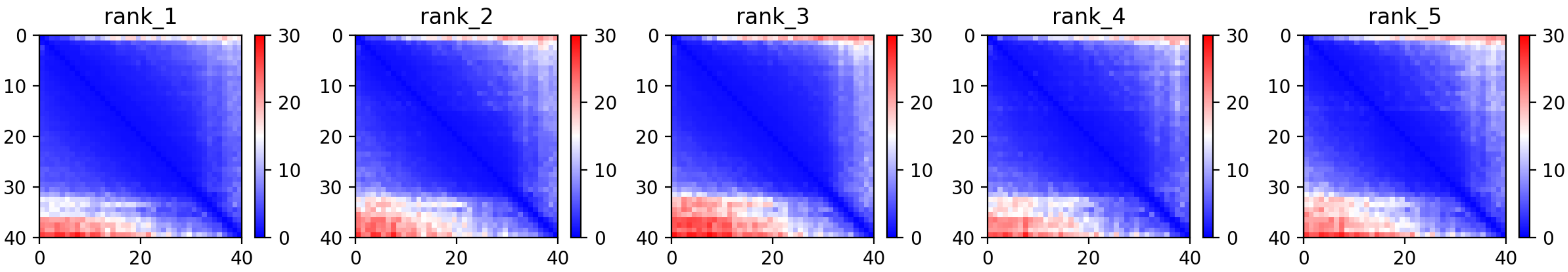

CP129: MtrunA17_Chr8g0355501

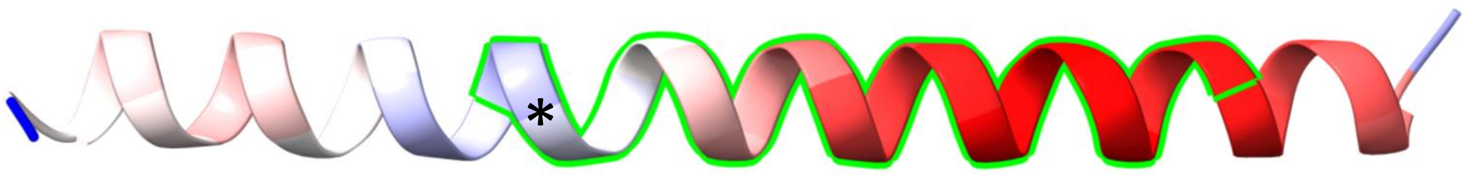

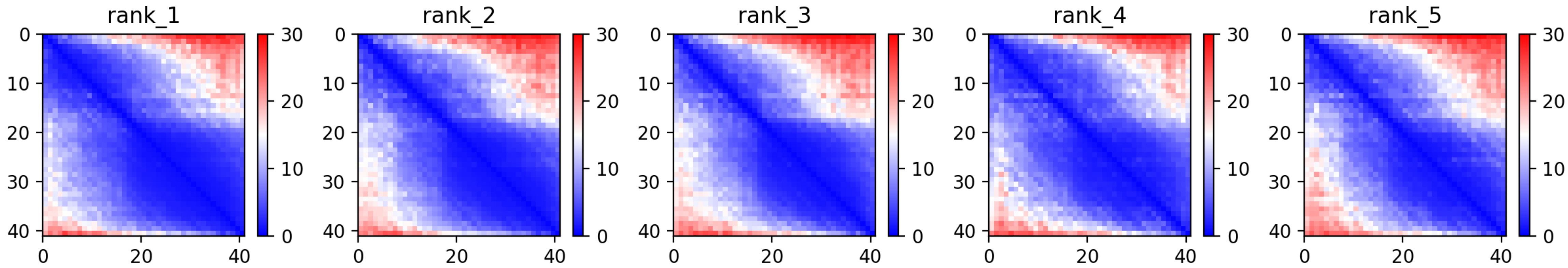

CP130: MtrunA17_Chr8g0356581

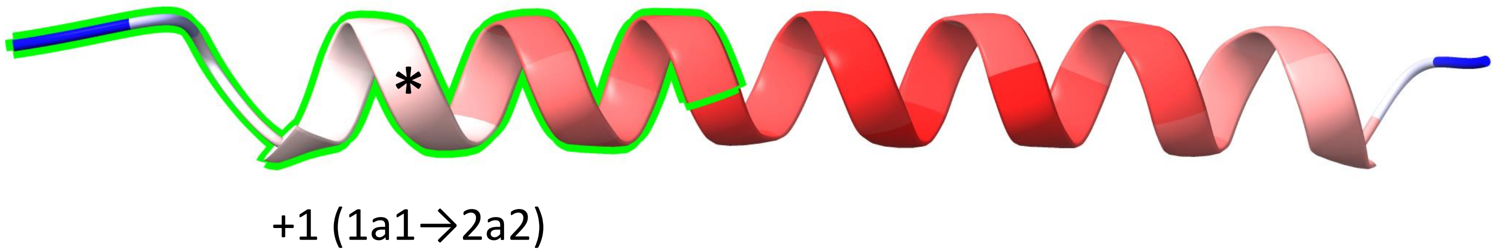

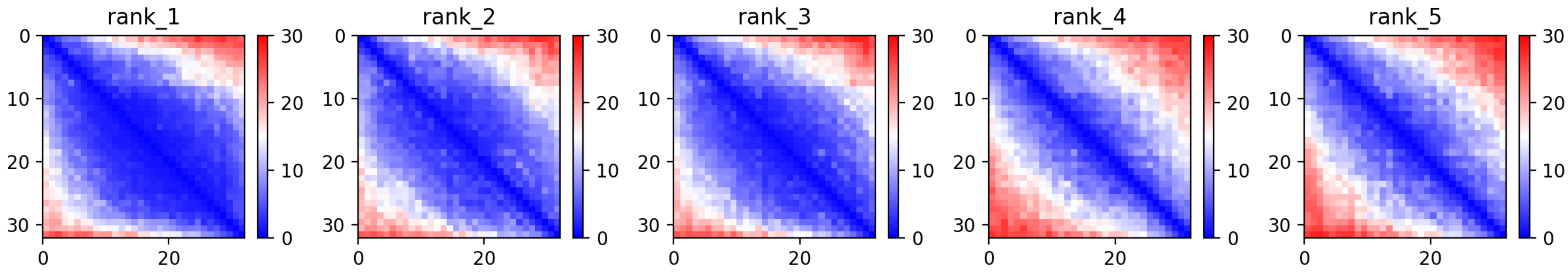

CP131: MtrunA17_Chr8g0365341

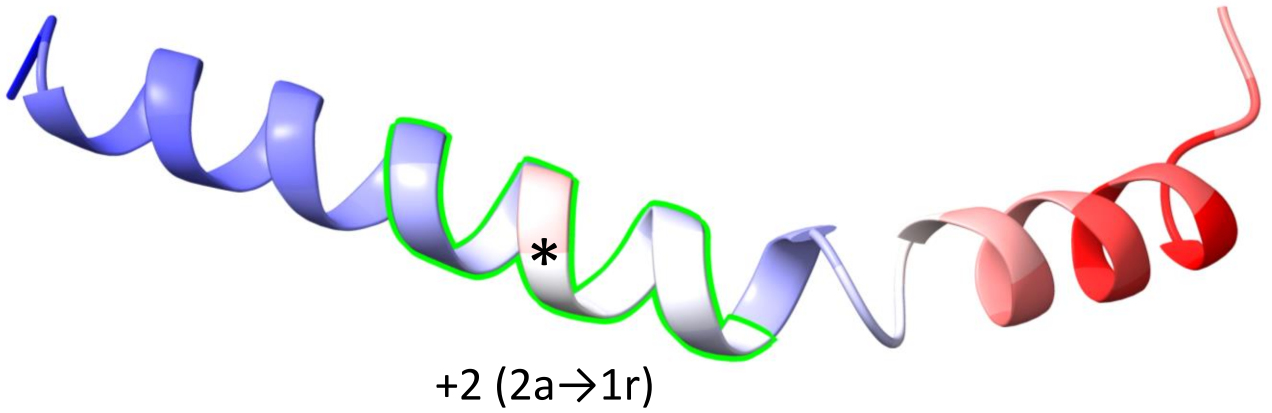

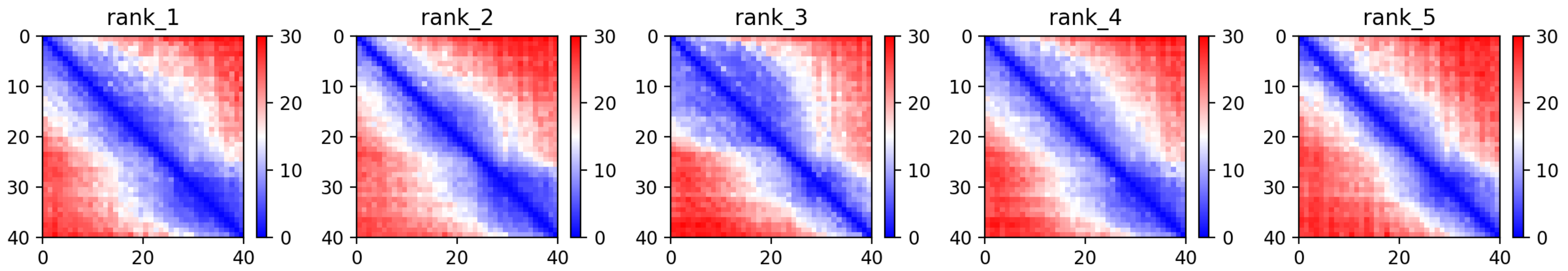

CP132: MtrunA17_Chr8g0368731

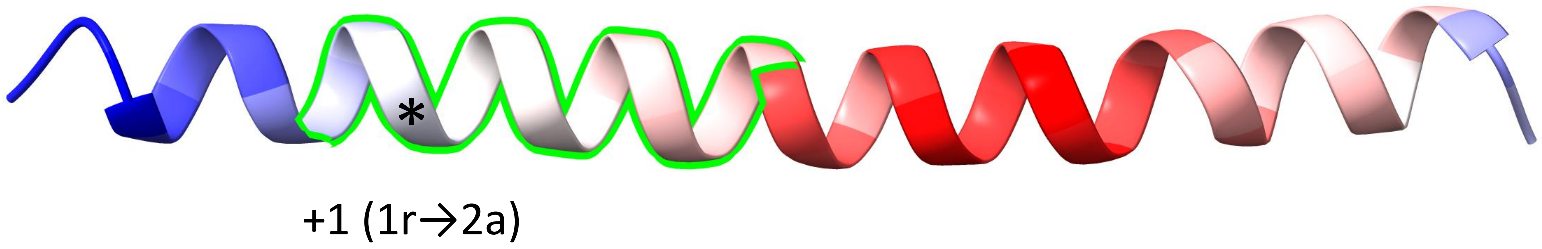

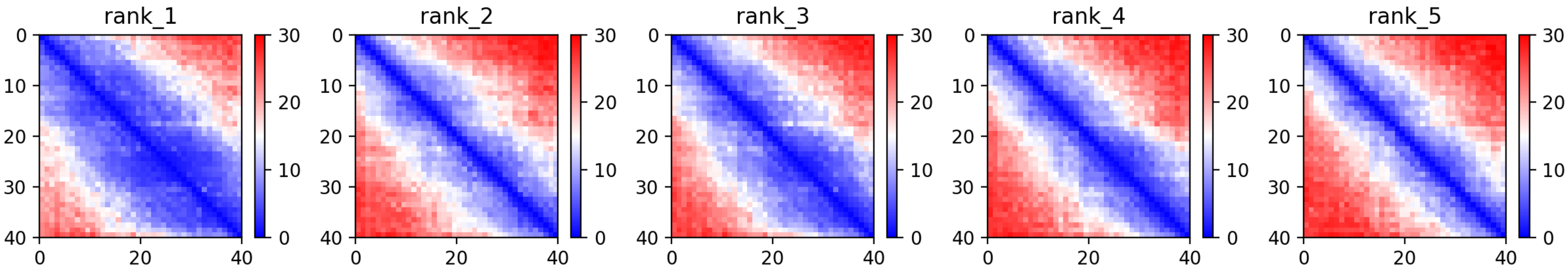

CP133: MtrunA17_Chr8g0368931

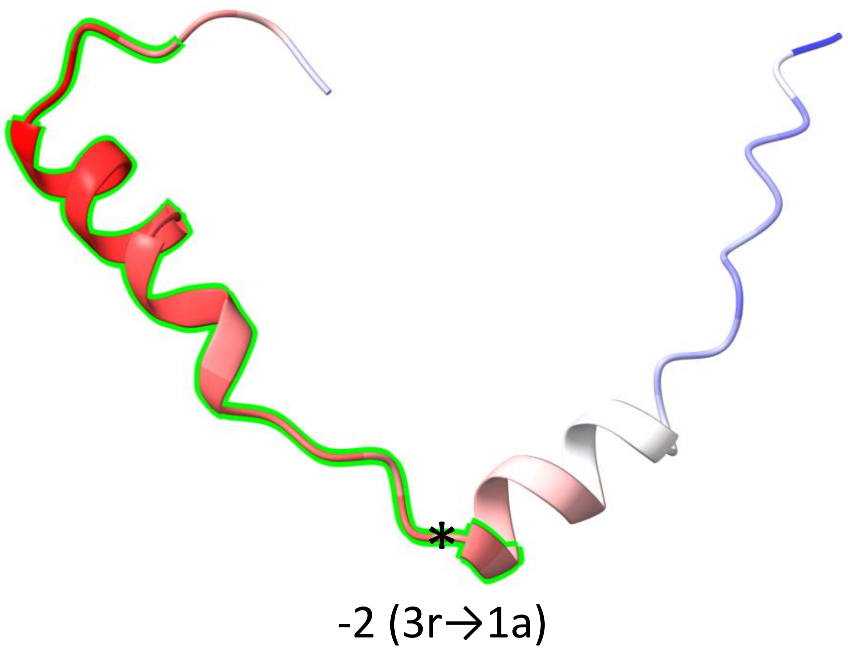

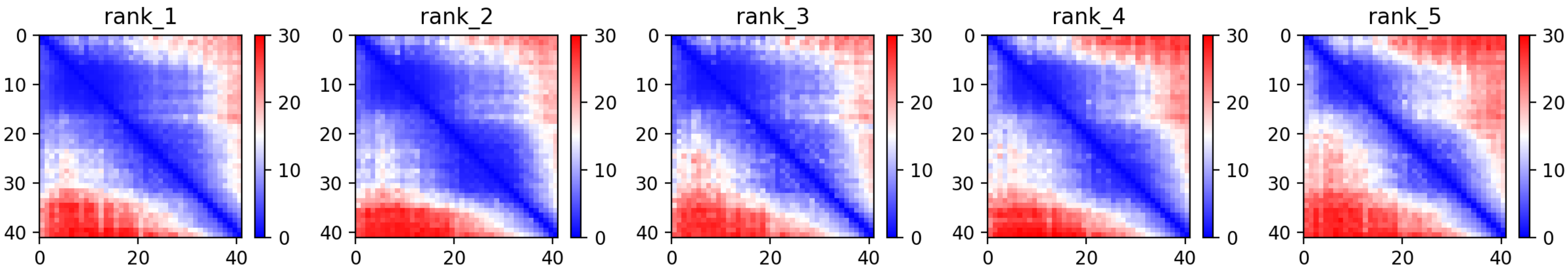

CP134: MtrunA17_Chr8g0371281

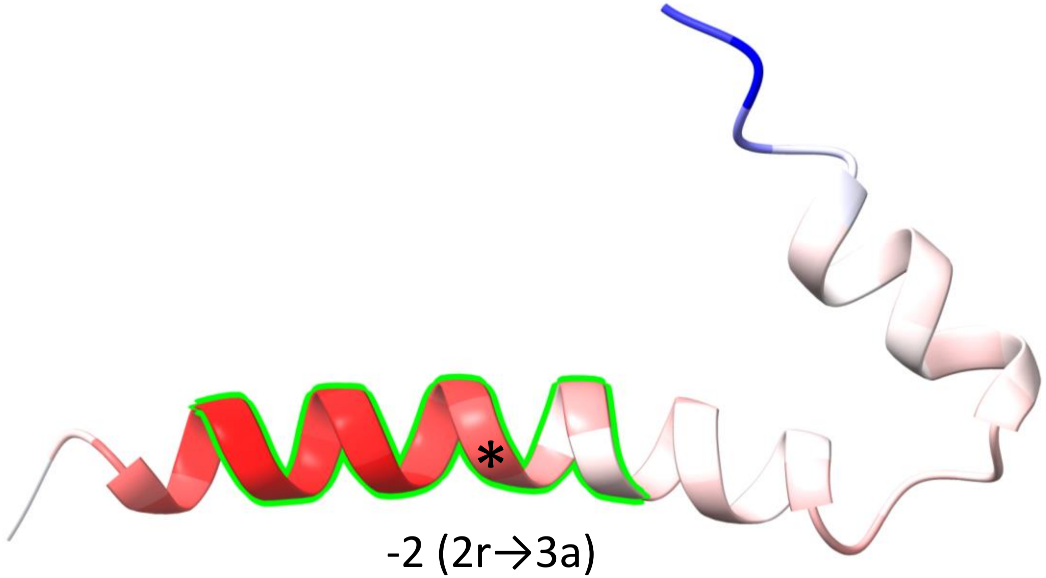

-2 (2r→3a)

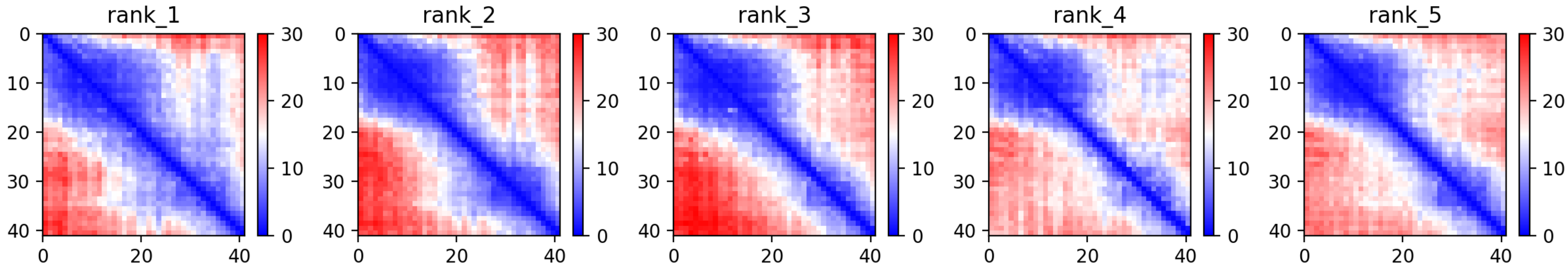

CP135: MtrunA17_Chr8g0371741

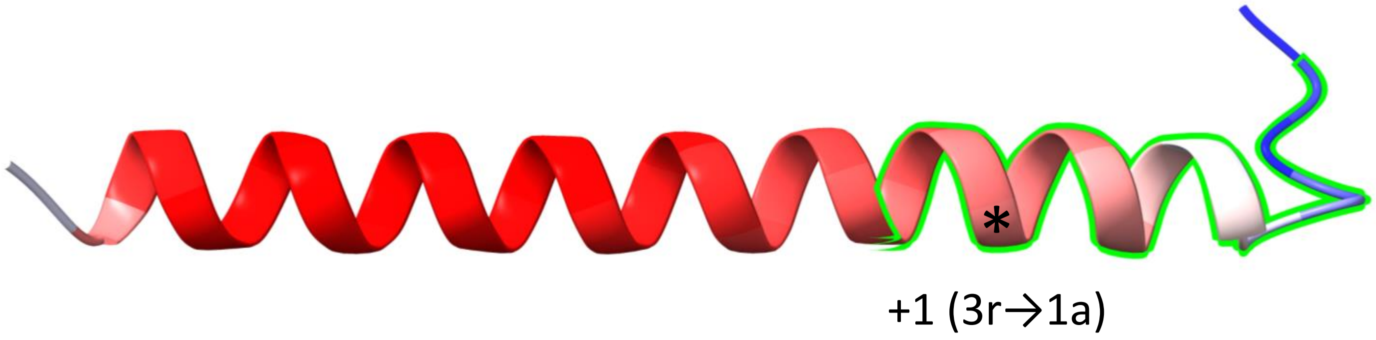

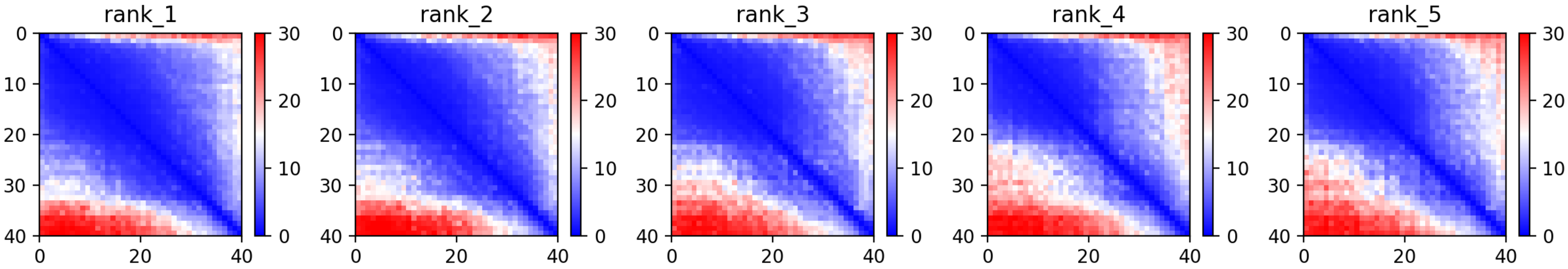

CP137: MtrunA17_Chr8g0376411

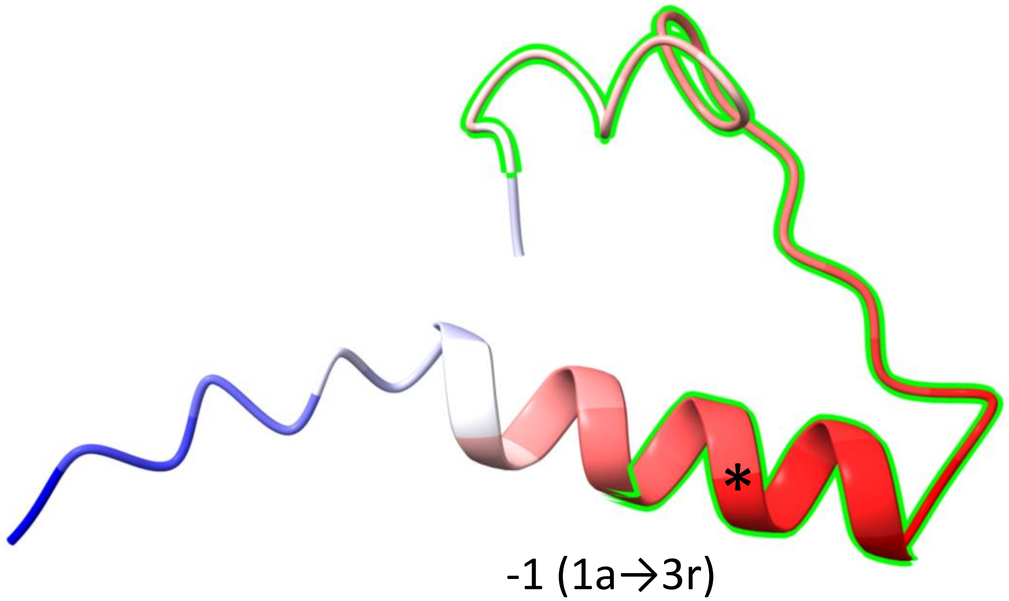

-1 (1a→3r)

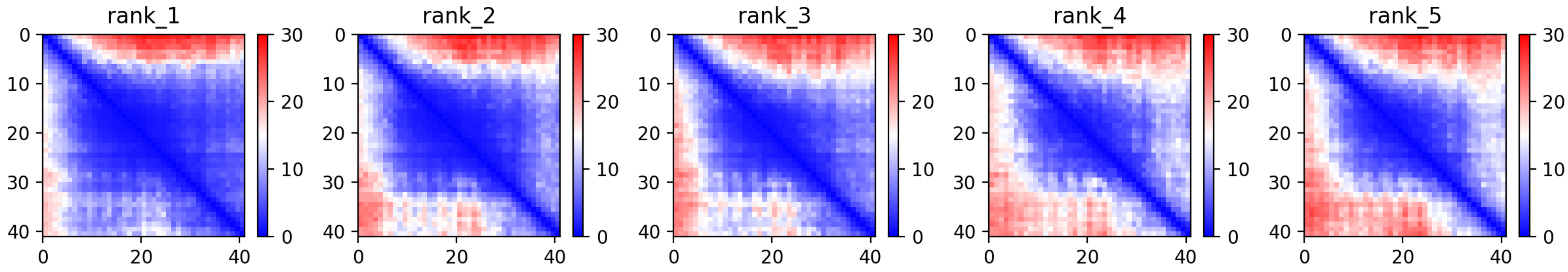

CP138: MtrunA17_Chr8g0377071

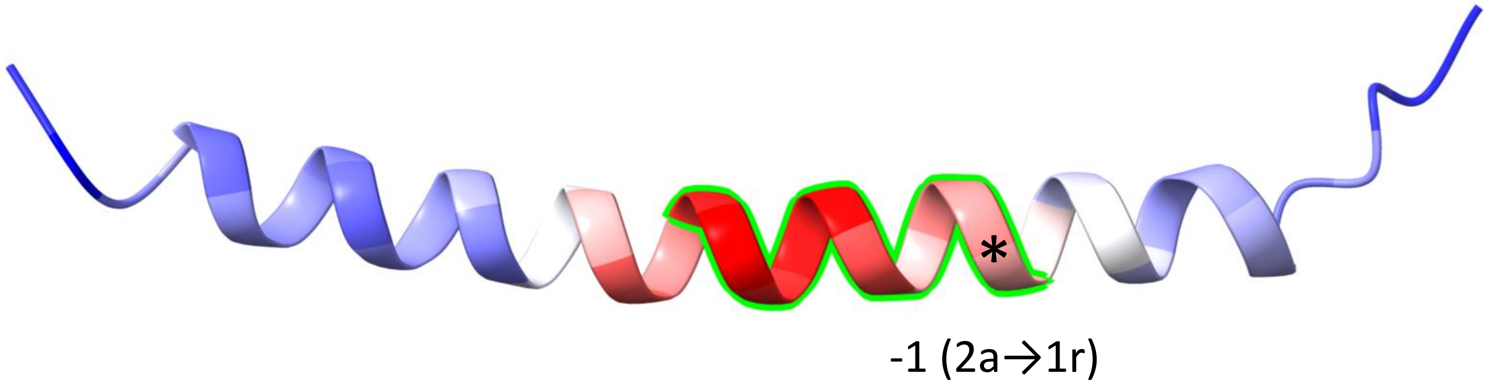

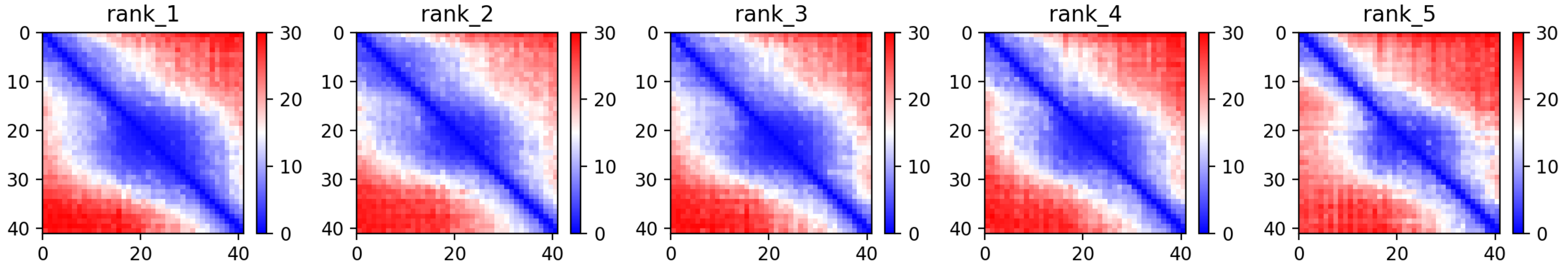

CP140: MtrunA17_Chr8g0392351

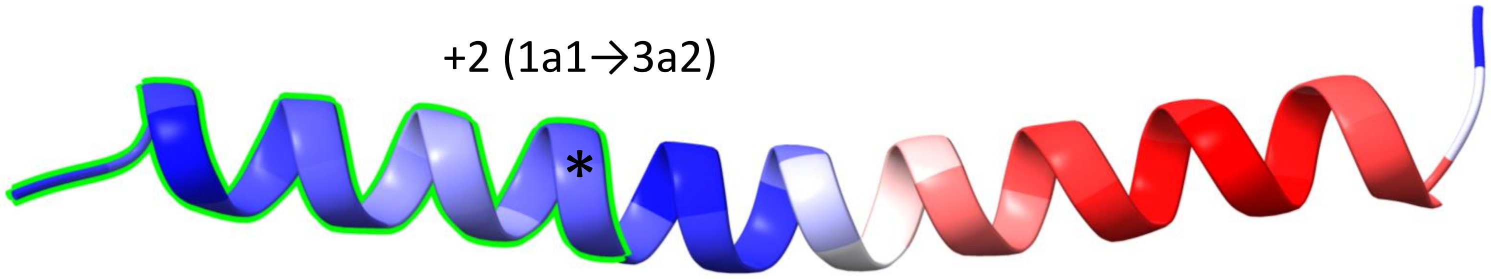

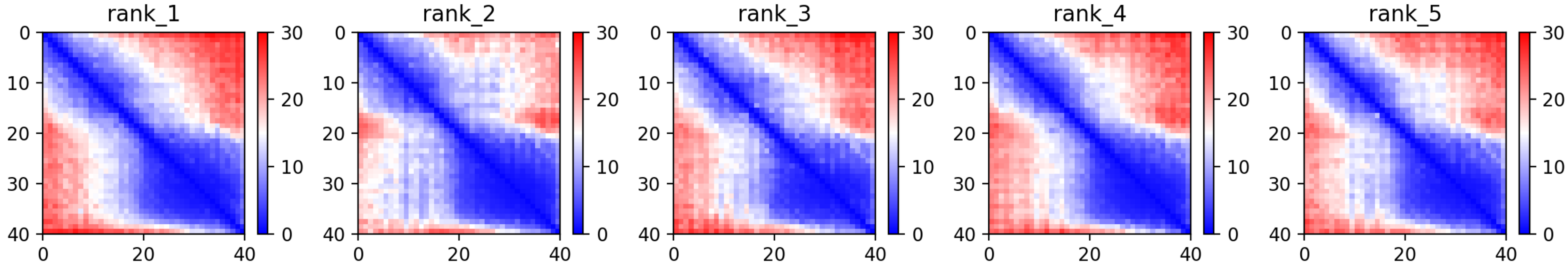

CP142: MtrunA17_CPg0492381

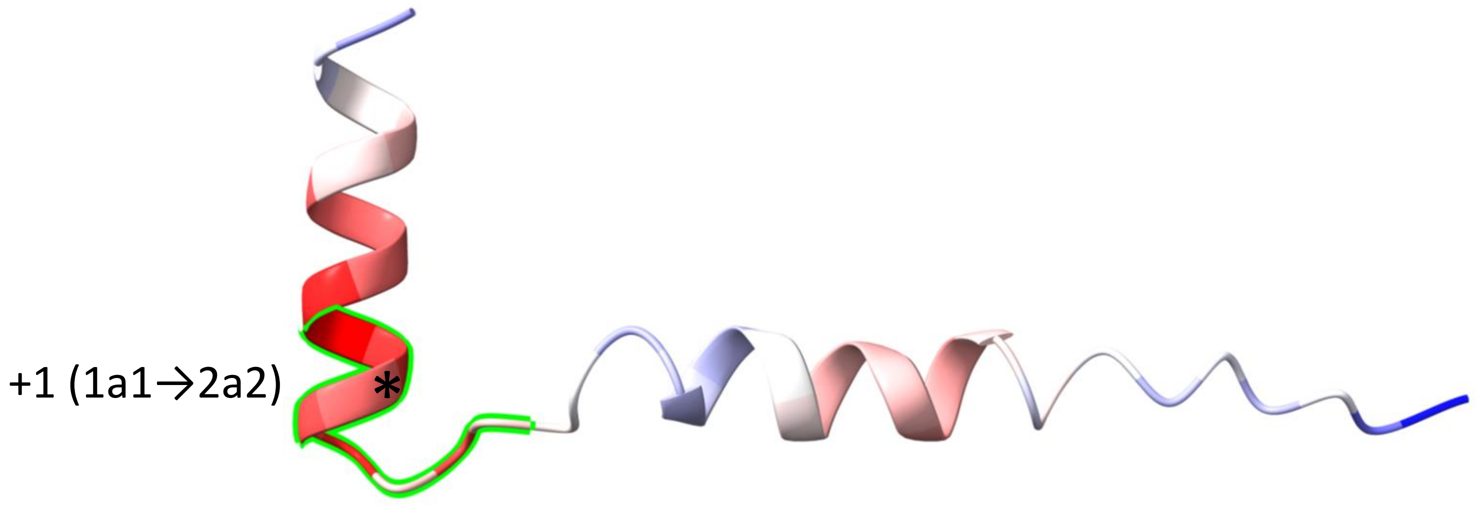

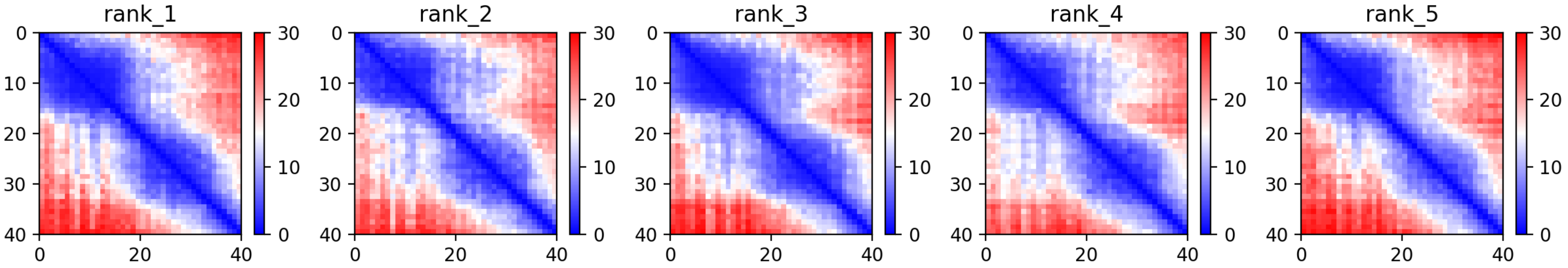

CP143: MtrunA17_CPg0492461

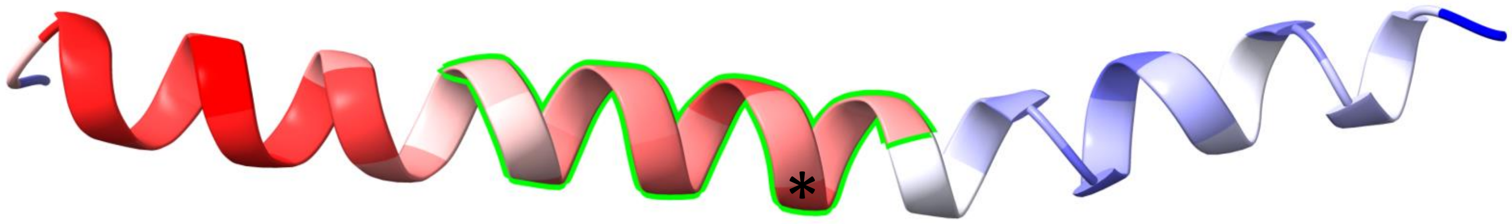

+1 (3r→1a)

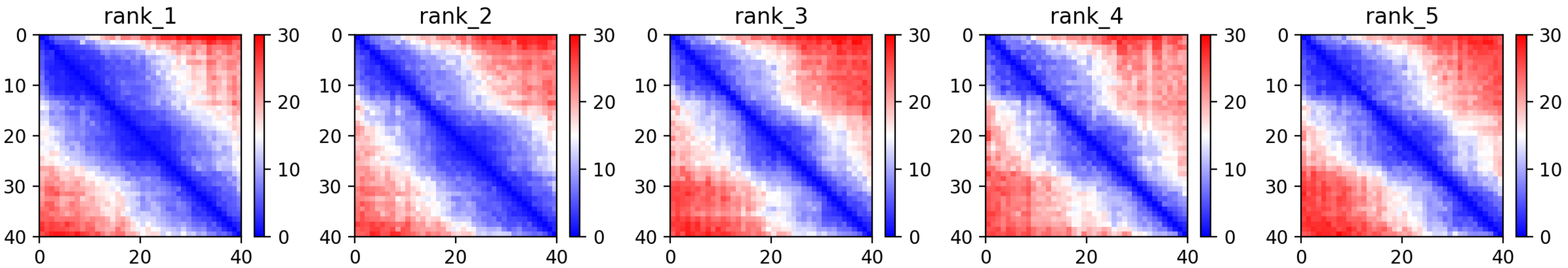

CP145: MtrunA17_CPg0492941

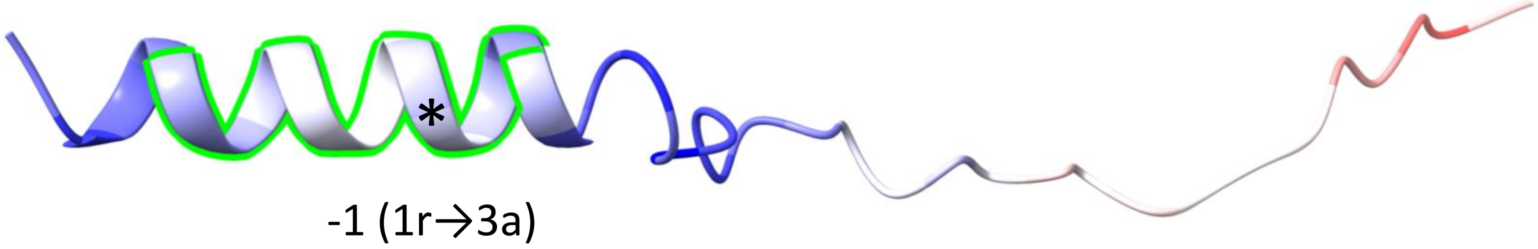

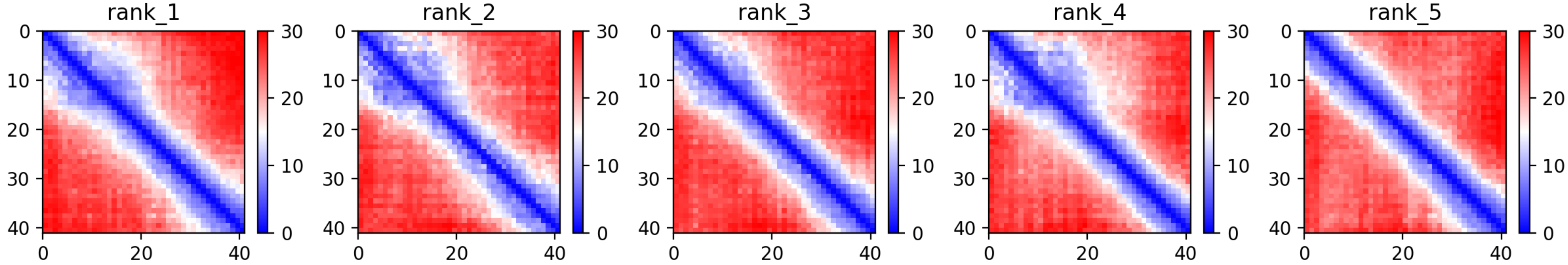

CP146: MtrunA17_CPg0493291

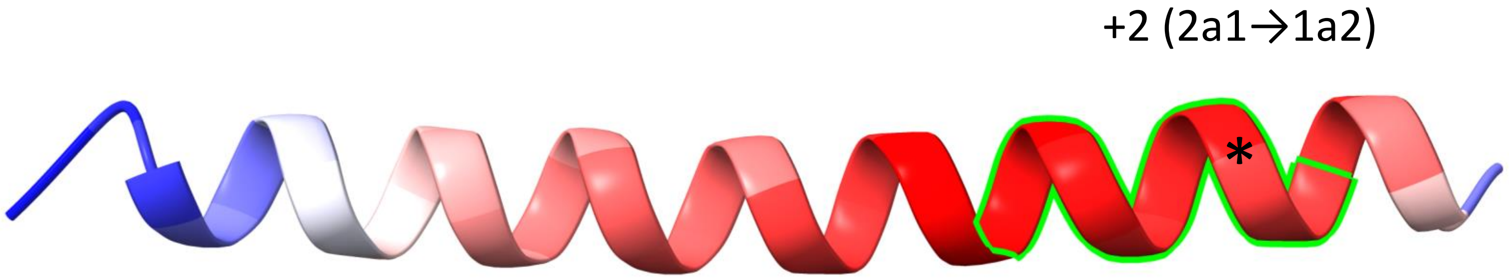

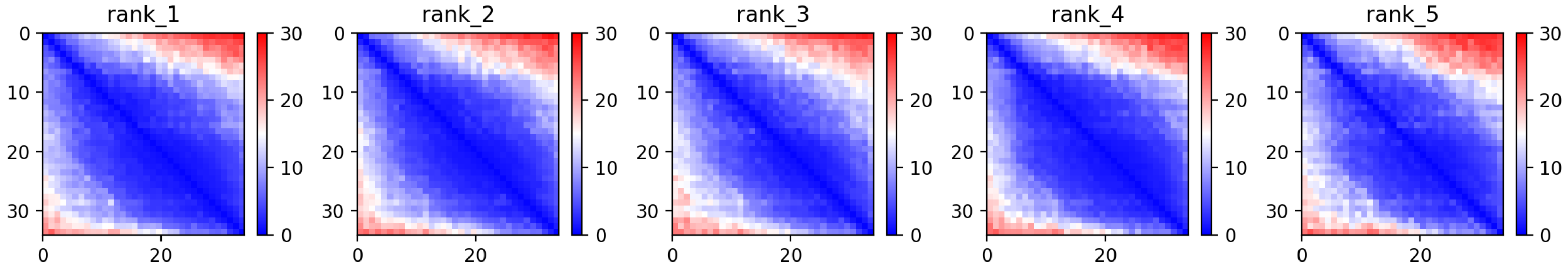

CP152: MtrunA17_MTg0491151

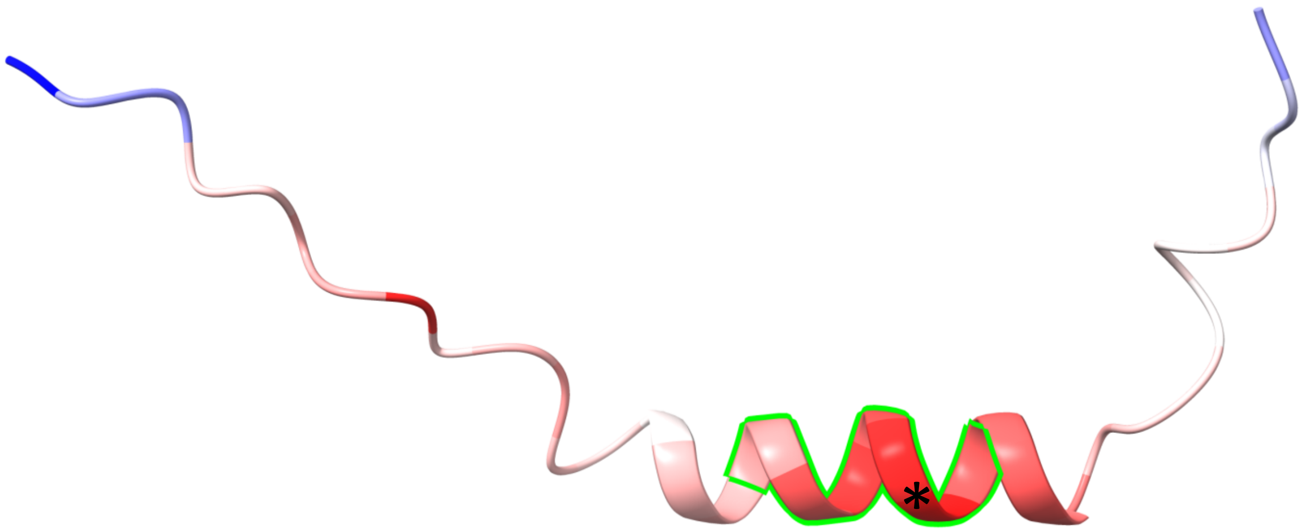

+2 (2a1→1a2)

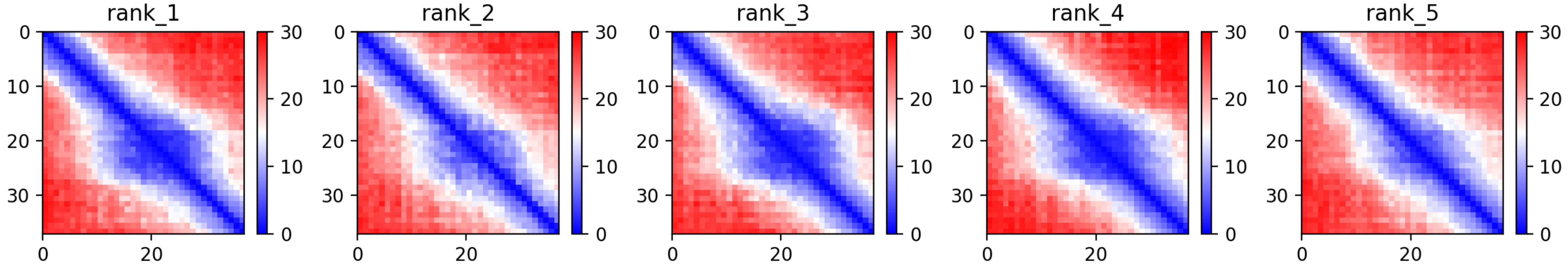

CP153: MtrunA17_MTg0491291

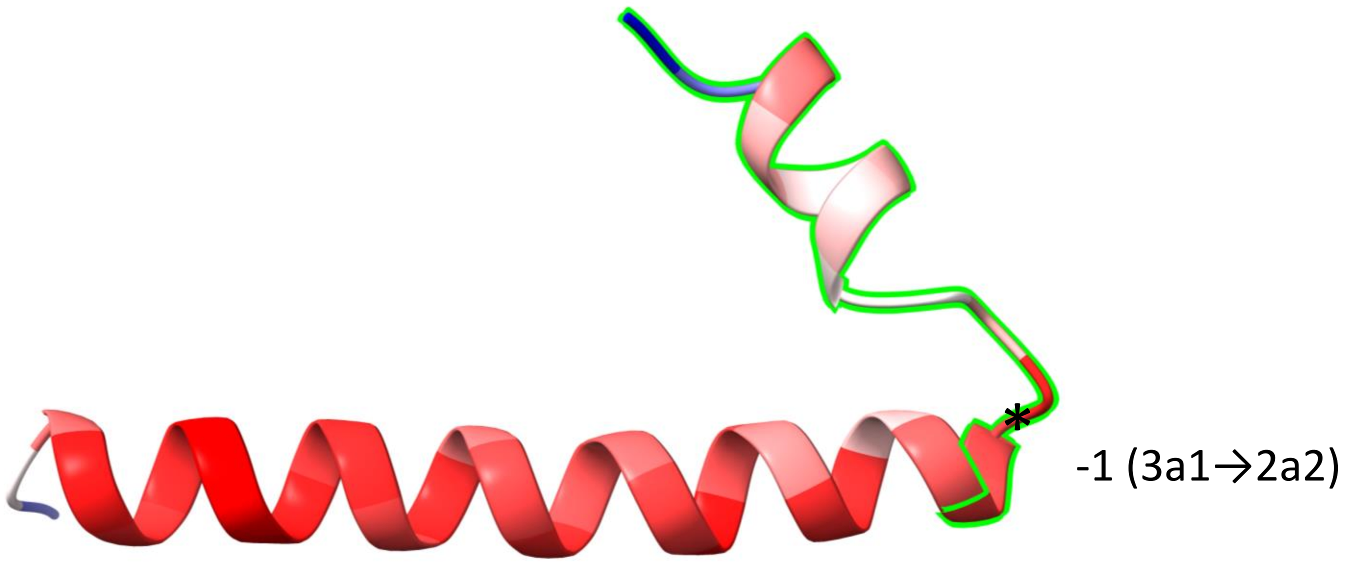

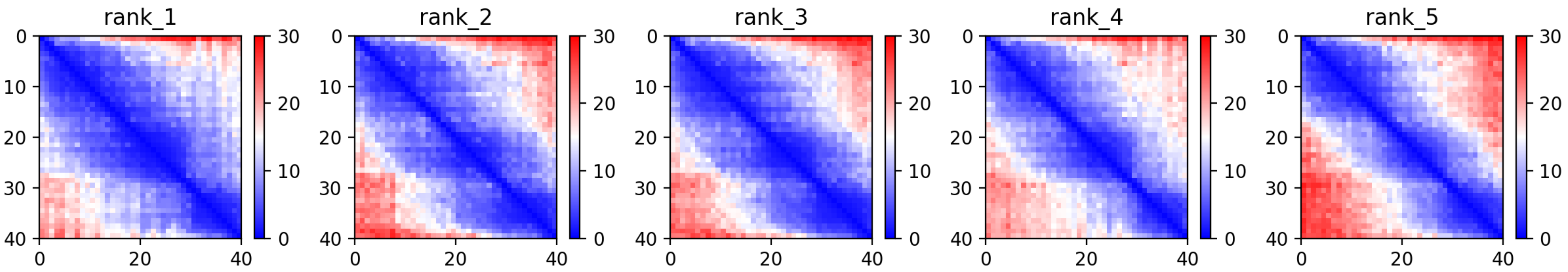

CP155: MtrunA17_MTg0491621

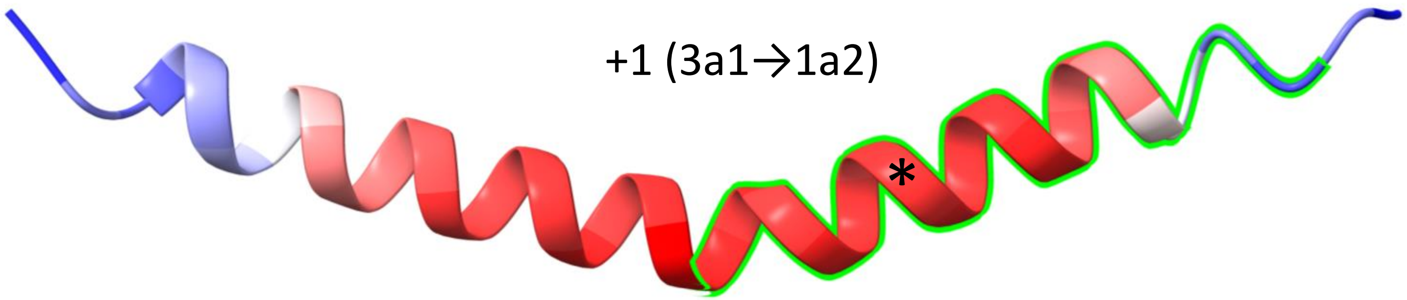

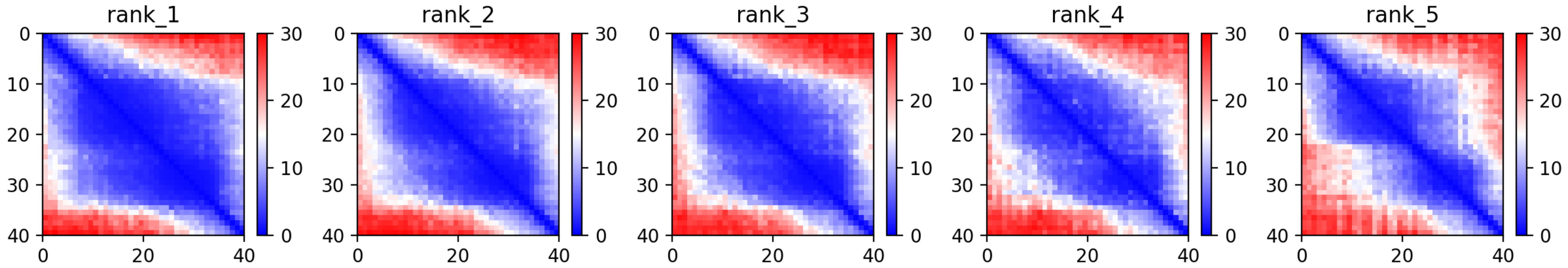

CP156: MtrunA17_MTg0491711

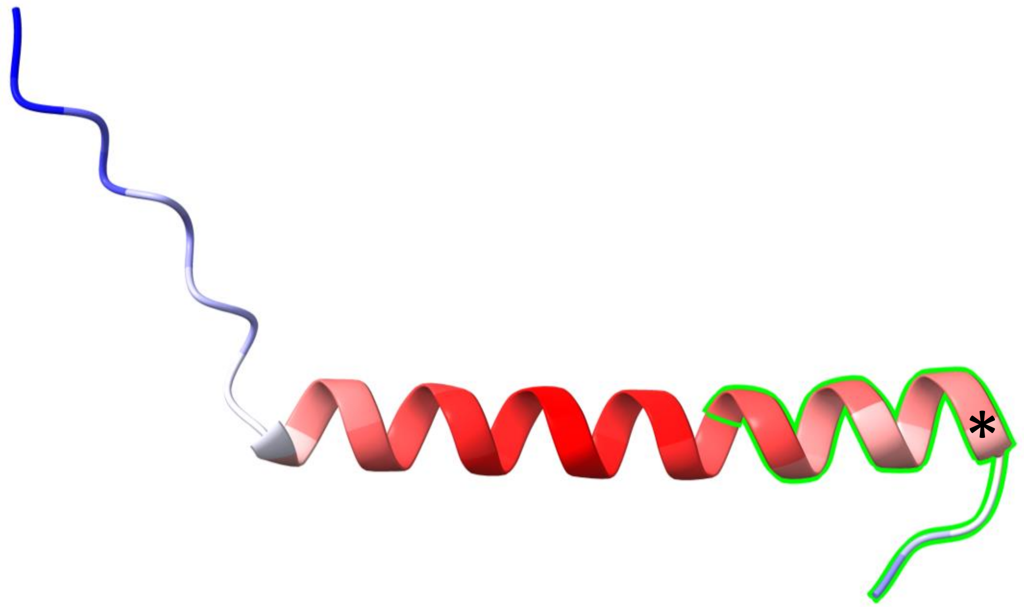

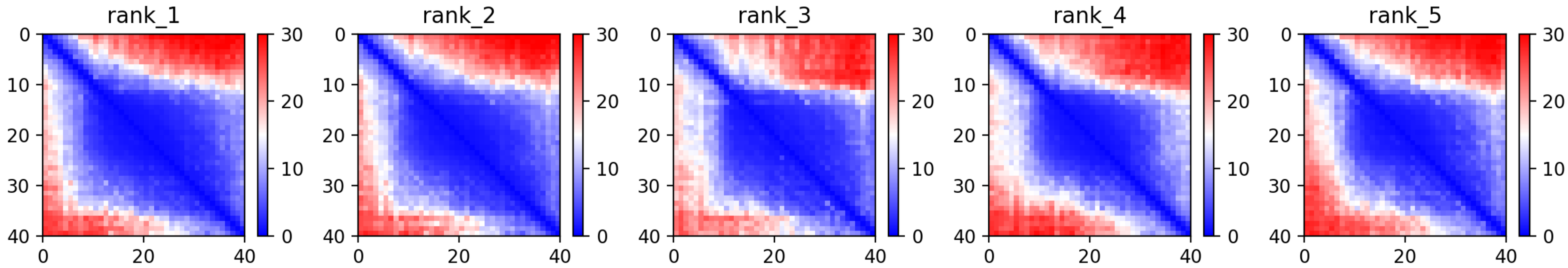

Supplementary Dataset S7 Part 4. A graphical summary on folding predictions for 90 MS-supported chimeric peptide models (CPs) that contain alpha-helices and no beta-sheets. For each CP, one image is displayed: the predicted folding structure (top) along with heat maps of five predictions (bottom). The rest of the legend is the same as for Supplementary Dataset S7 Part 1.
