# Supplementary material for: Discovery of diverse chimeric peptides in a eukaryotic proteome sets the stage for experimental validation of the mosaic translation hypothesis
Source: Comput Struct Biotechnol J. 2025 Sep 12;27:4048–64. doi: 10.1016/j.csbj.2025.09.019 (PMC12481079; doi:10.1016/j.csbj.2025.09.019)
Supplement: Supplementary file 1 — Supplementary material [file mmc1.zip › Supplementary Datasets/Supplementary Dataset S7 Folding of chimeric protein models Part 5 Beta-sheets only.pdf]

CP11: MtrunA17_Chr1g0164591

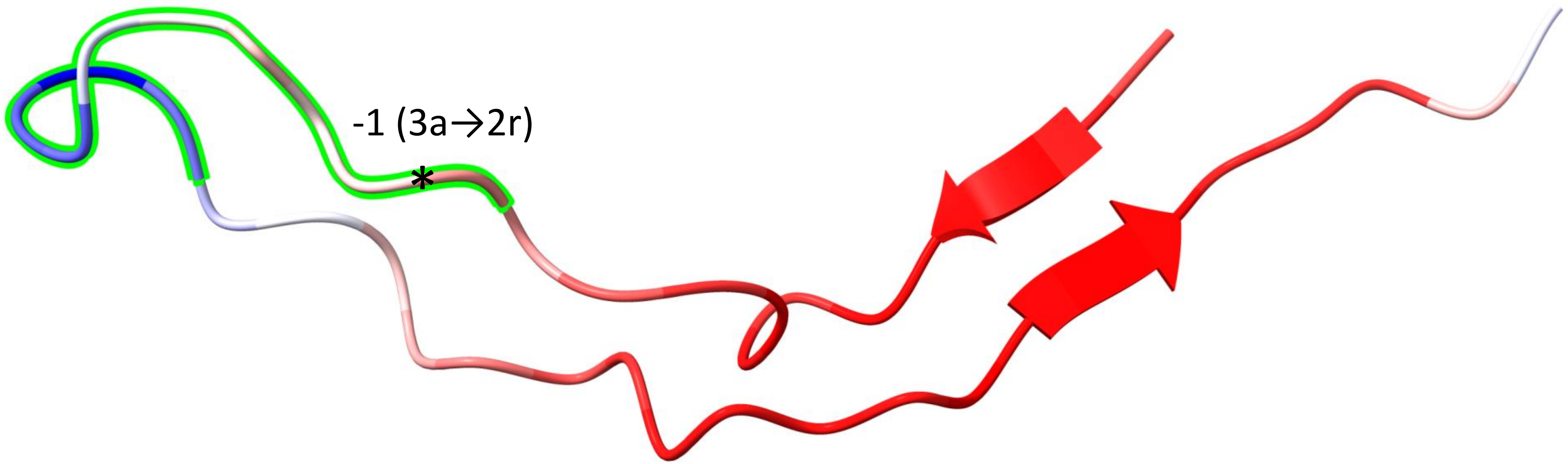

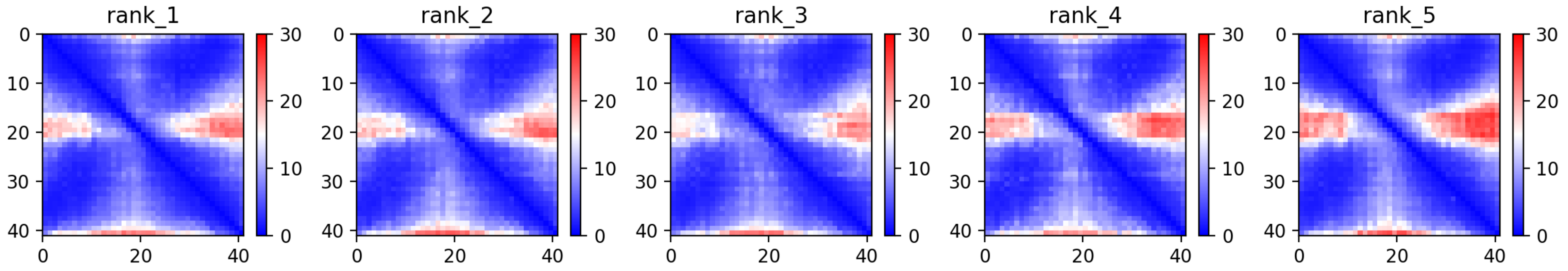

CP19: MtrunA17_Chr1g0185871

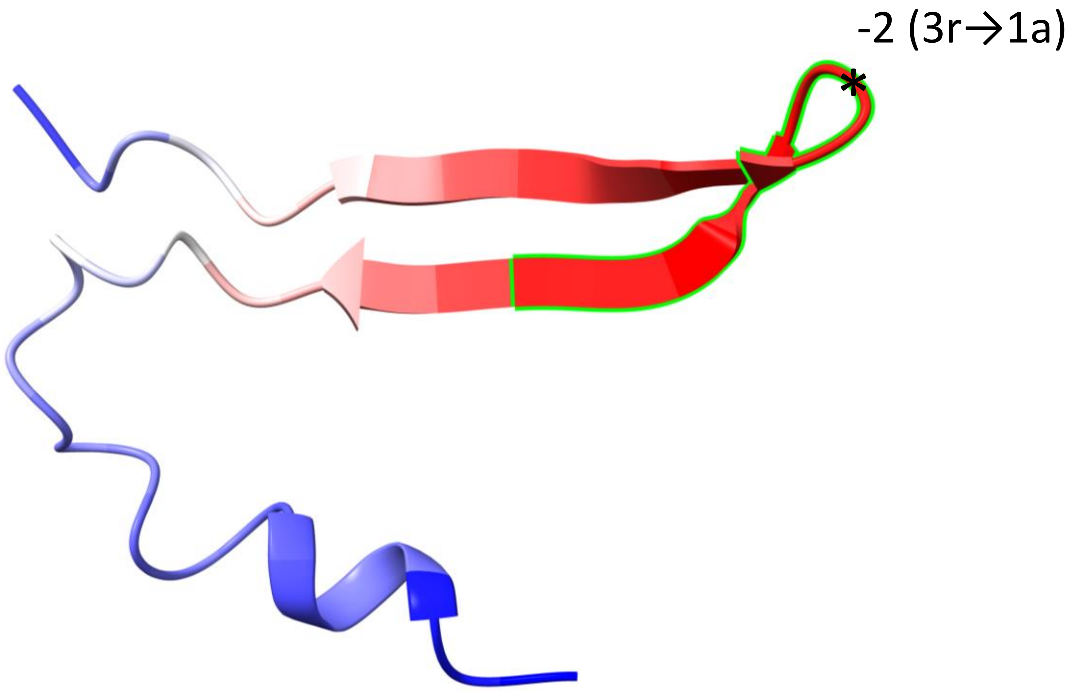

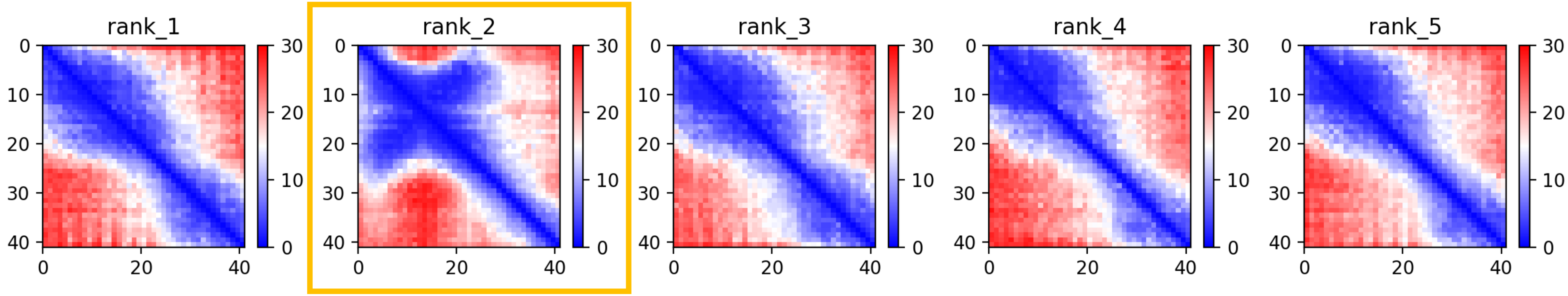

CP20: MtrunA17_Chr1g0190571

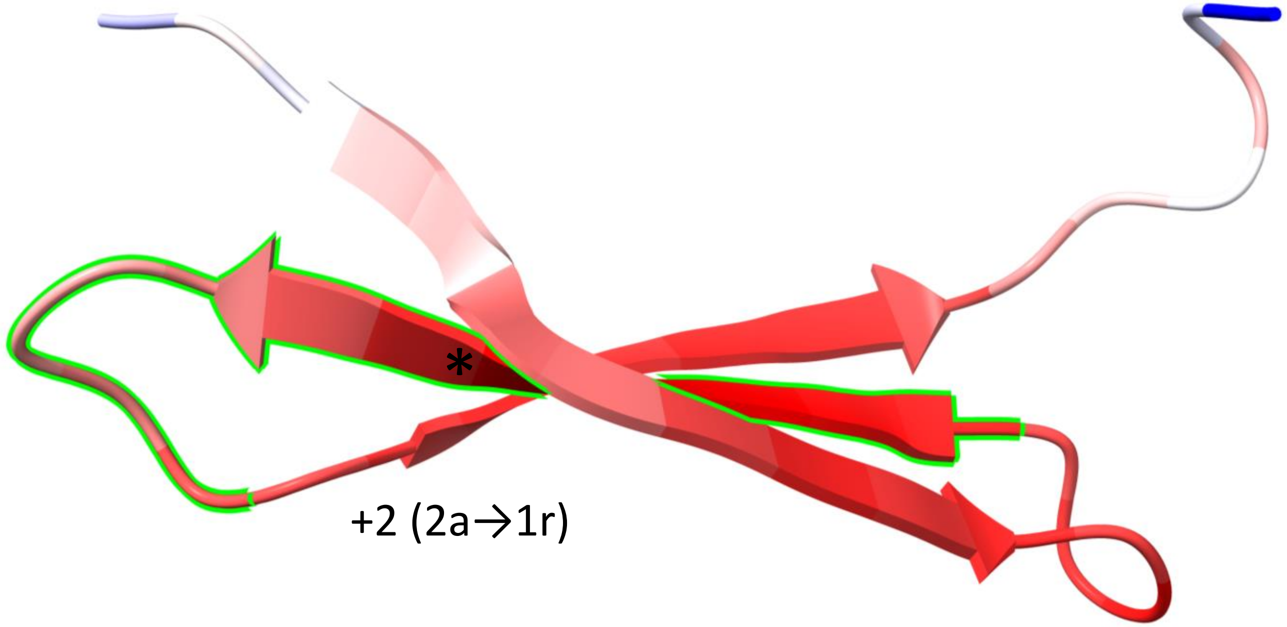

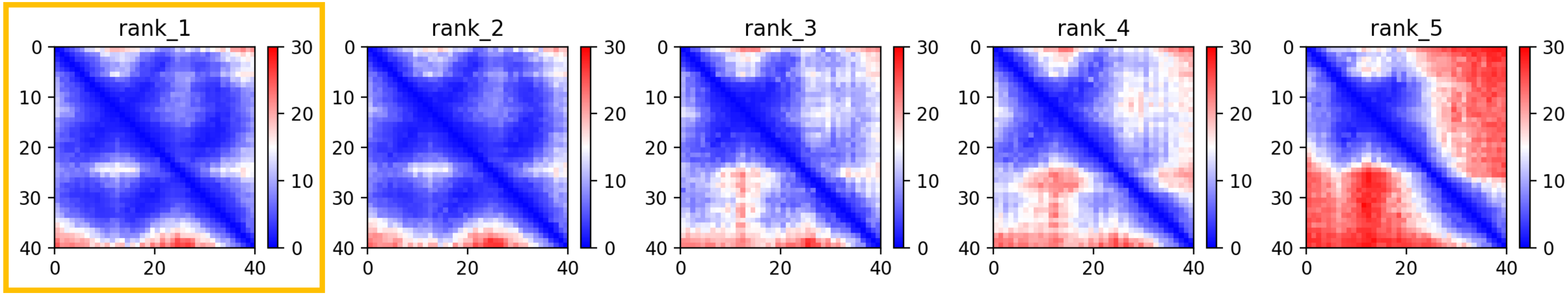

CP20: MtrunA17_Chr1g0190571

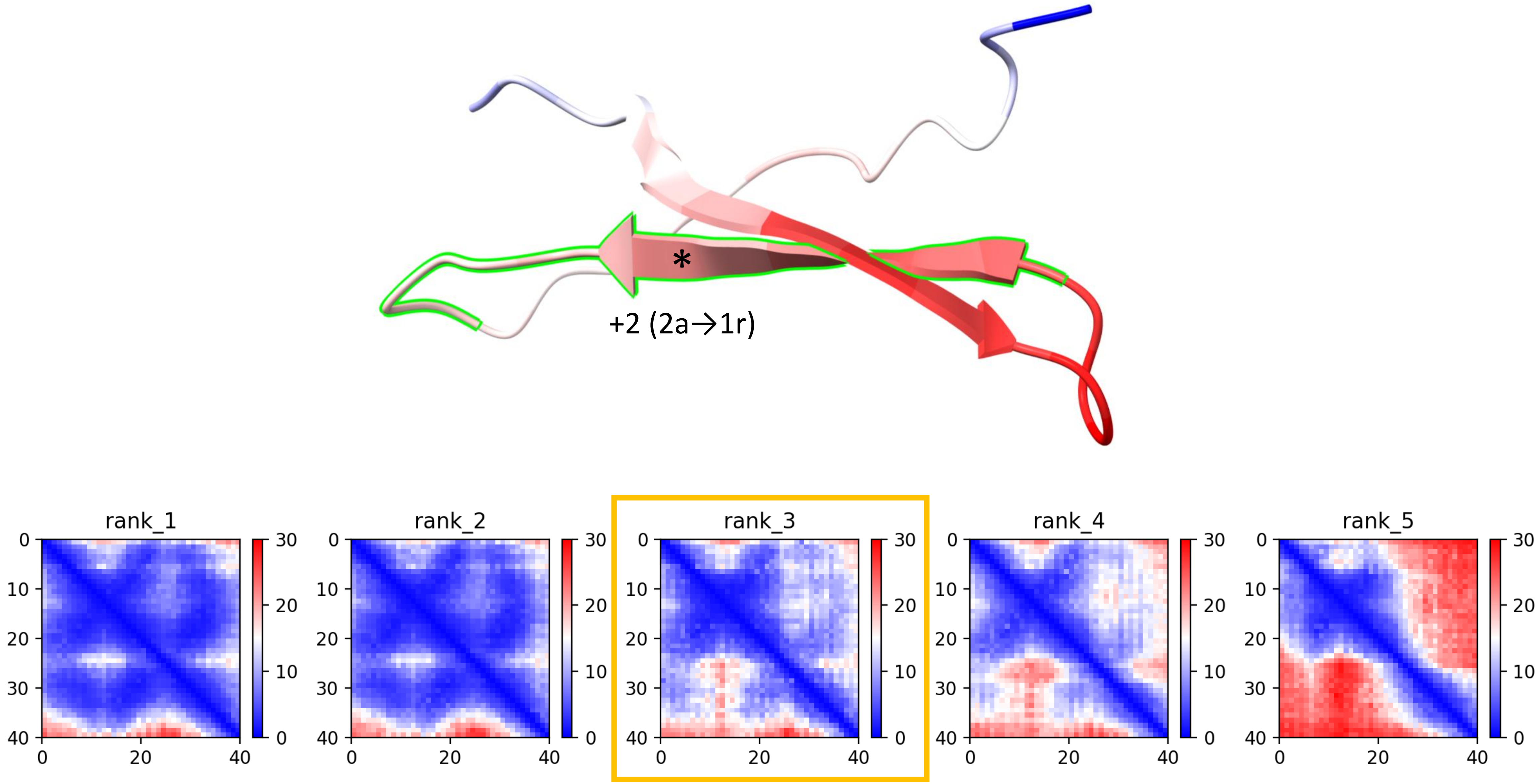

CP20: MtrunA17_Chr1g0190571

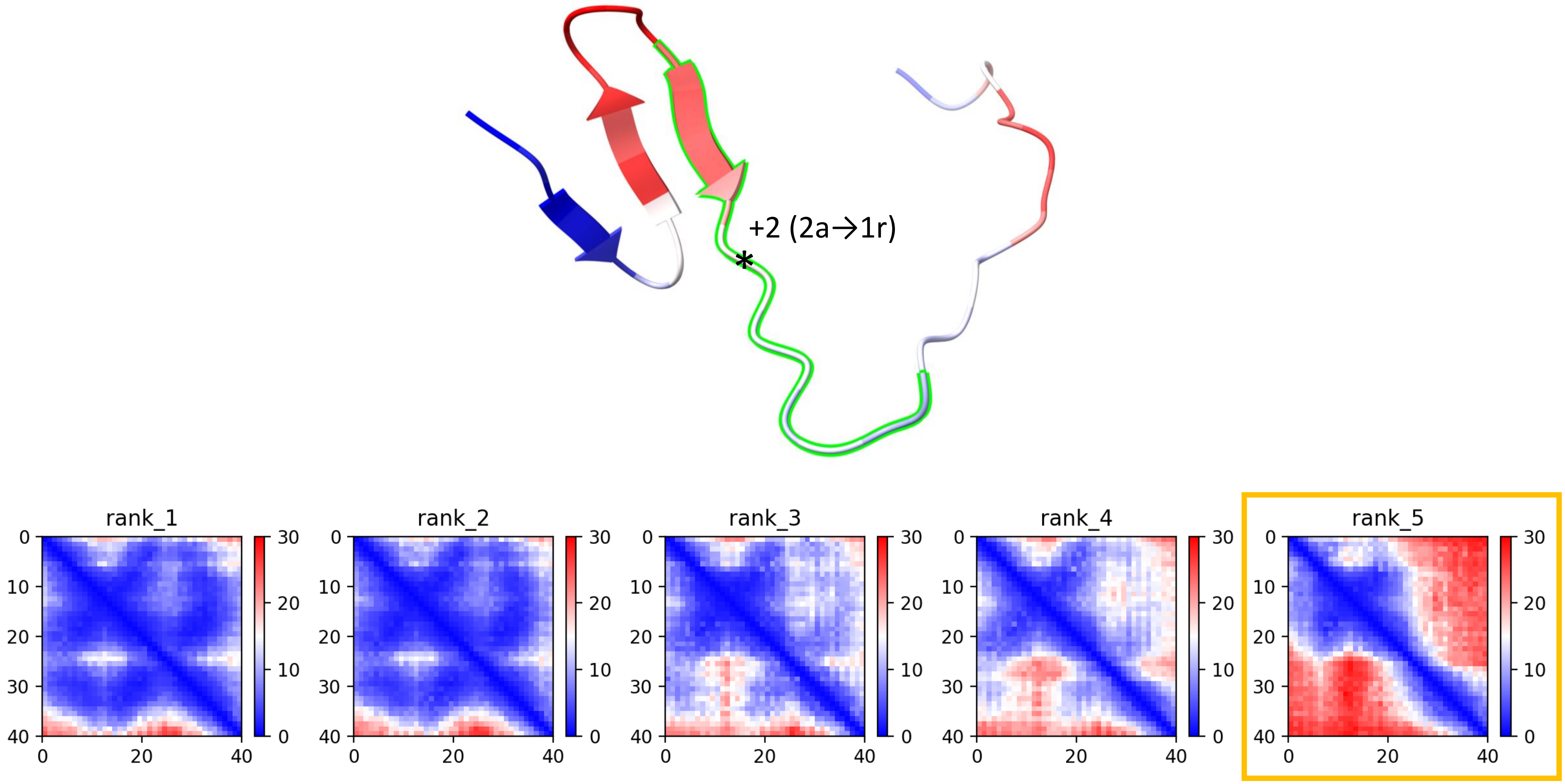

CP23: MtrunA17_Chr1g0200071

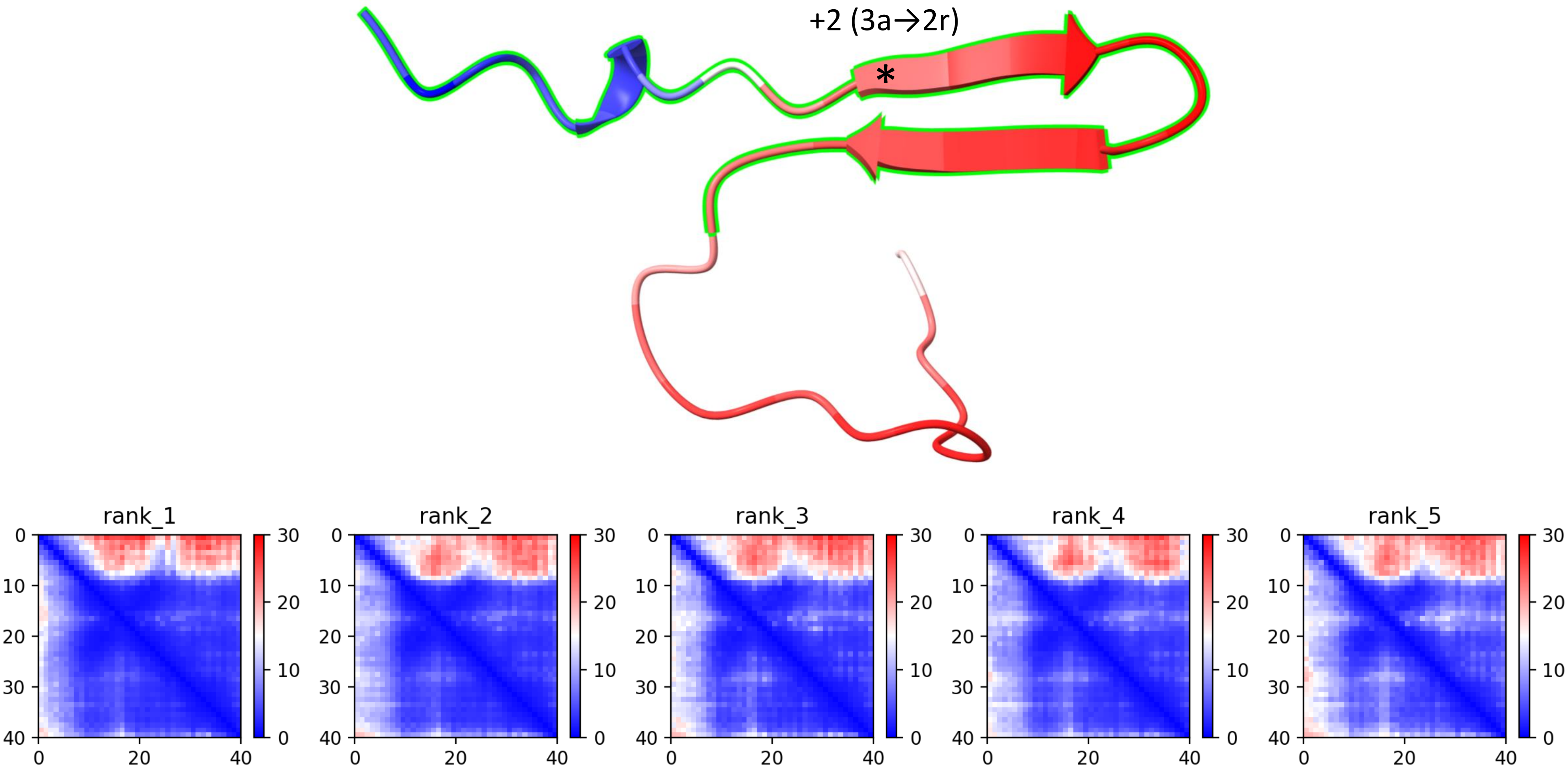

CP28: MtrunA17_Chr1g0207921

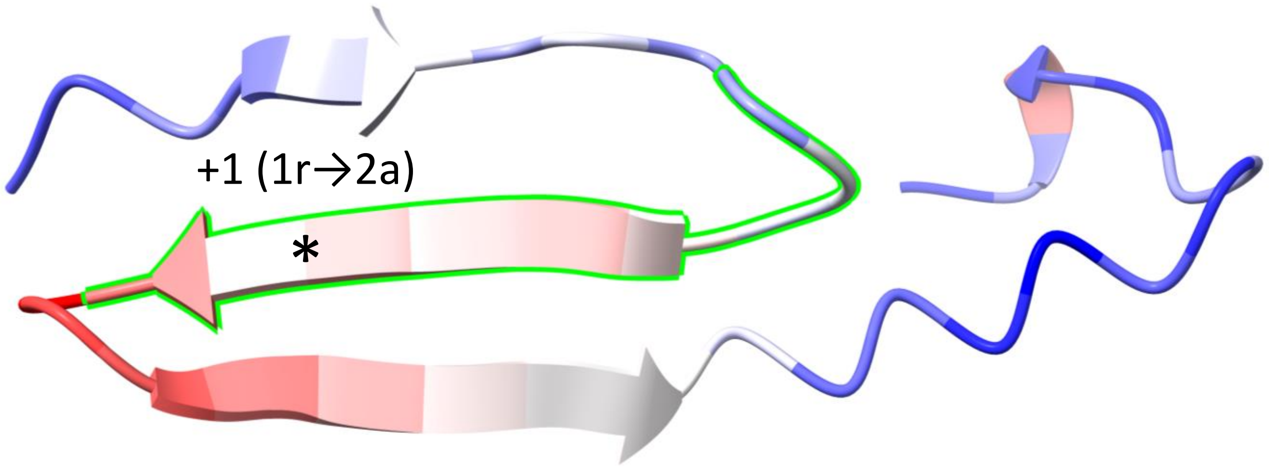

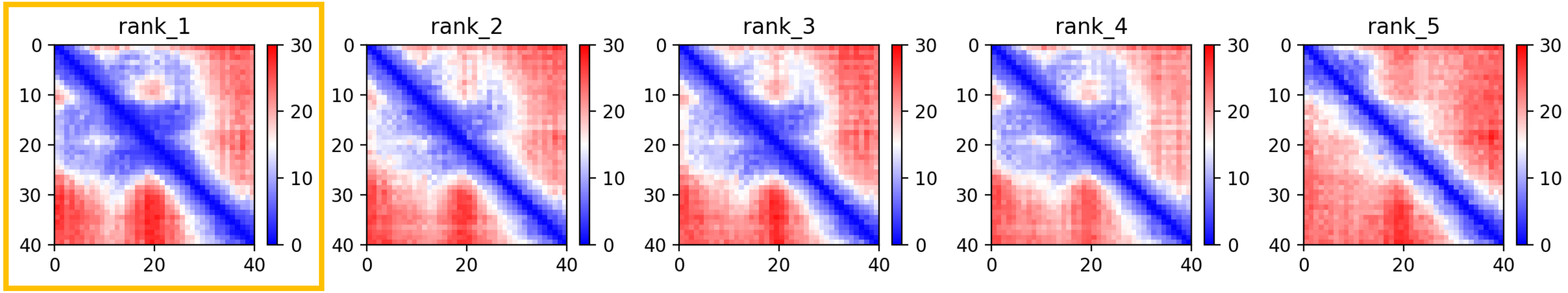

CP31: MtrunA17_Chr1g0212961

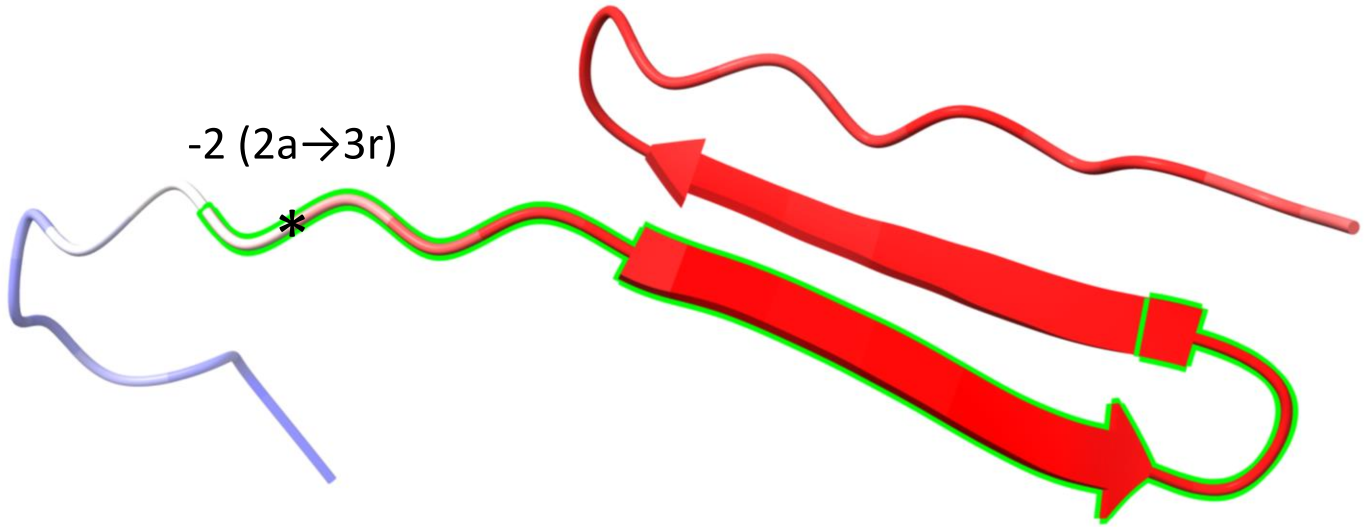

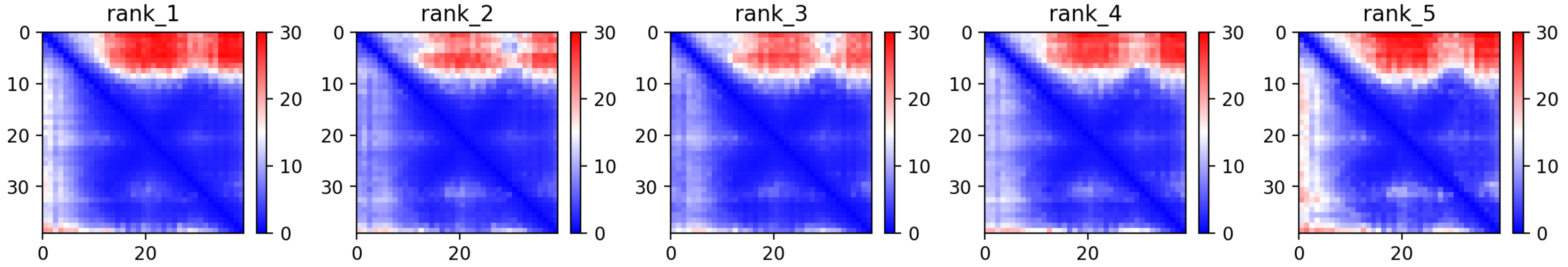

CP34: MtrunA17_Chr2g0285461

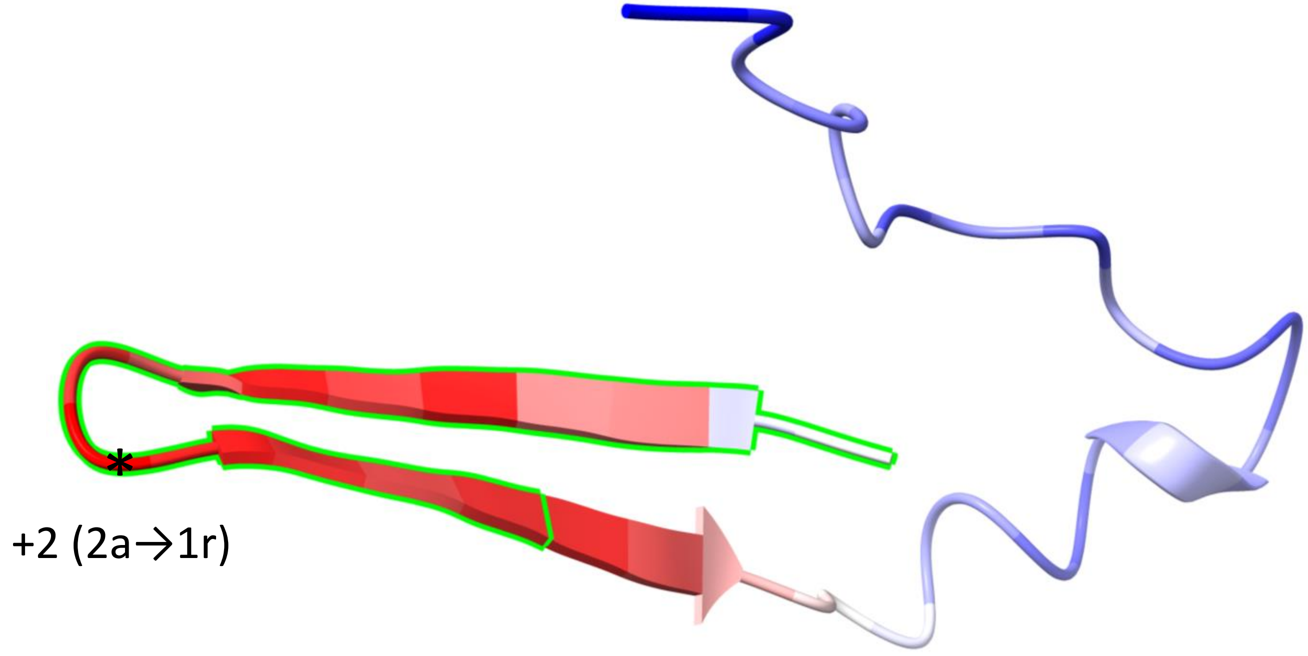

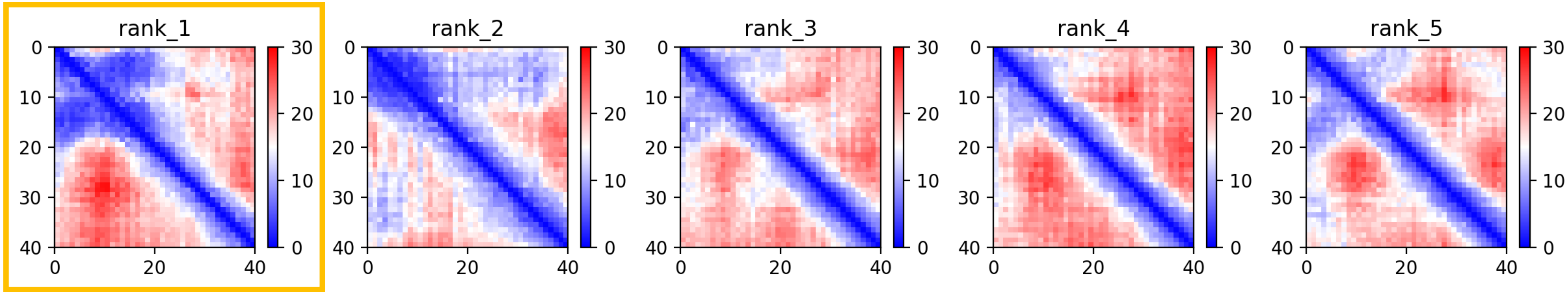

CP38: MtrunA17_Chr2g0304891

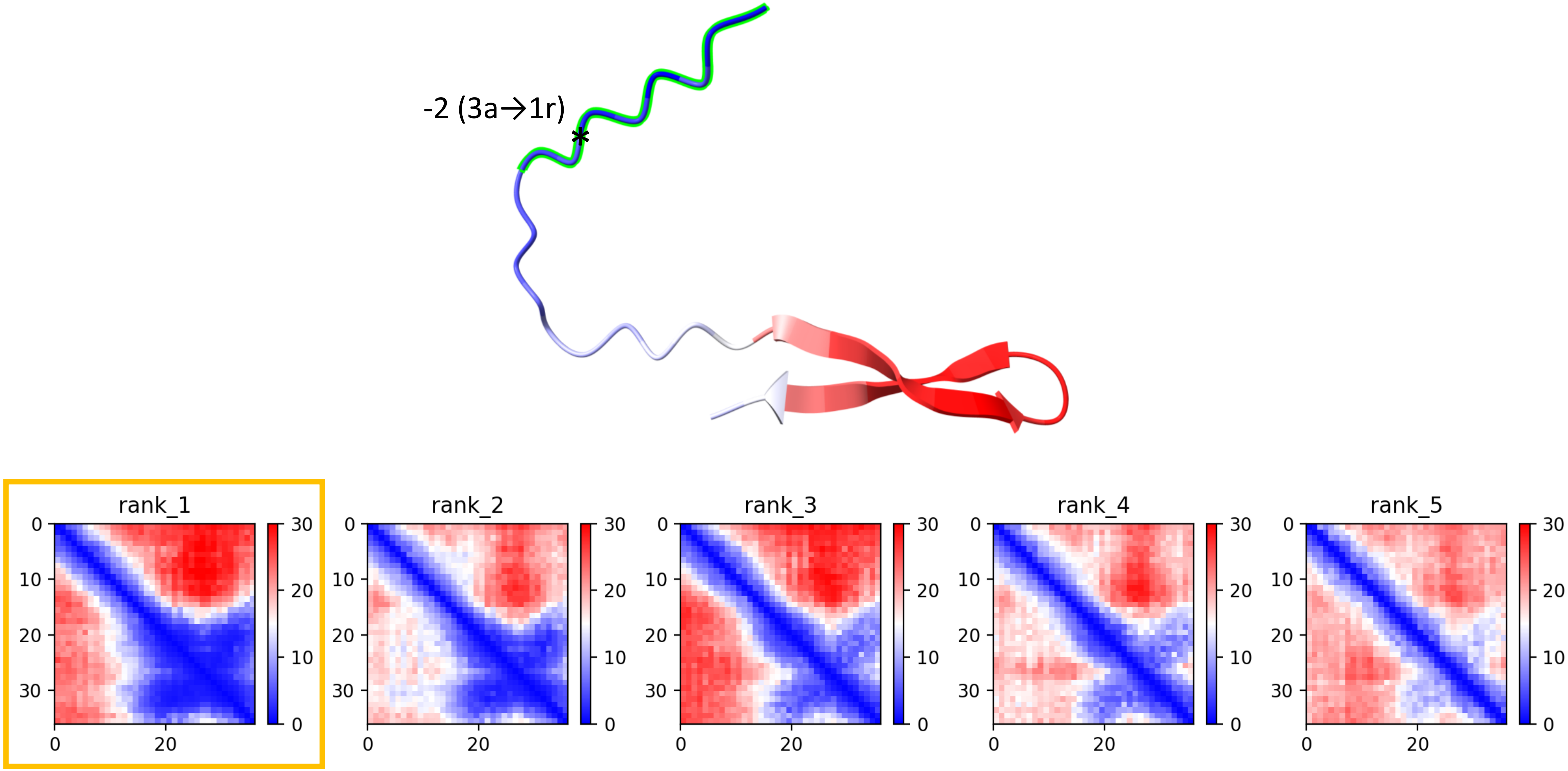

CP42: MtrunA17_Chr2g0312631

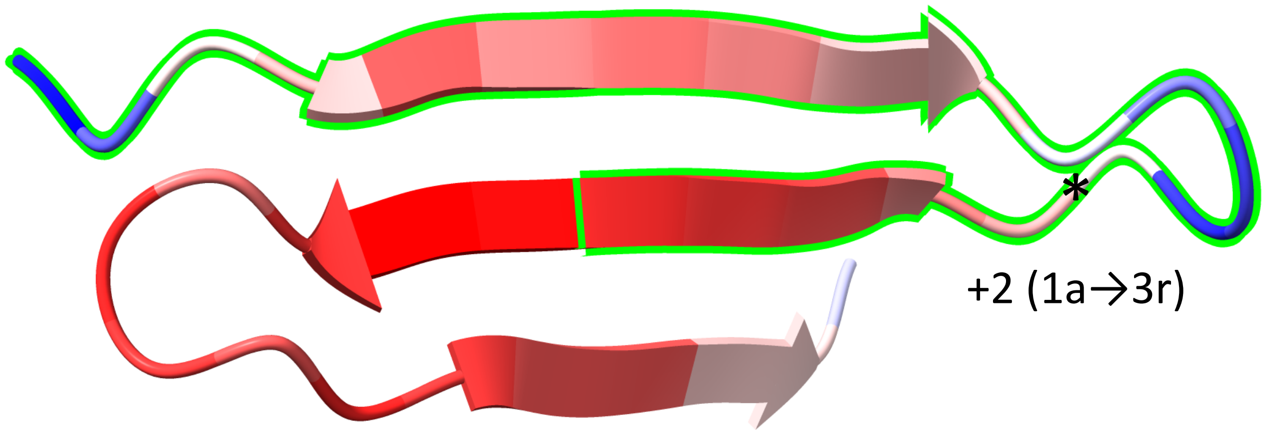

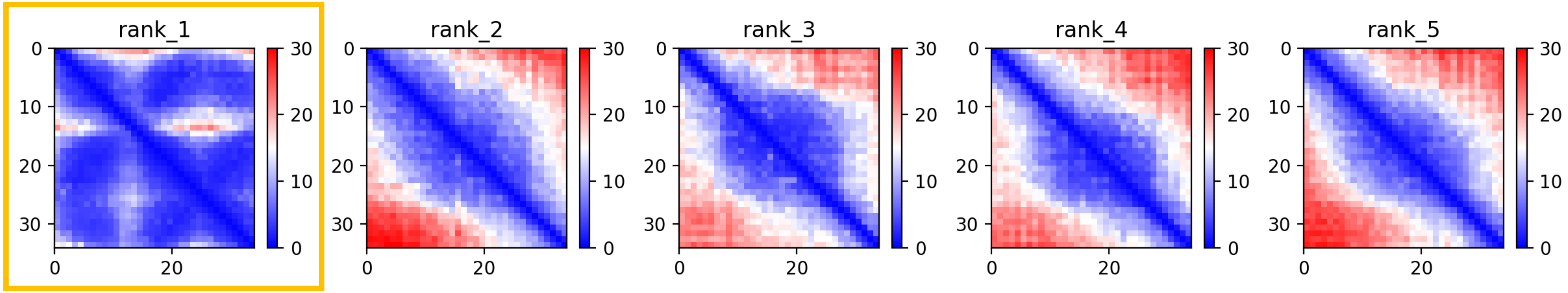

CP45: MtrunA17_Chr2g0328091

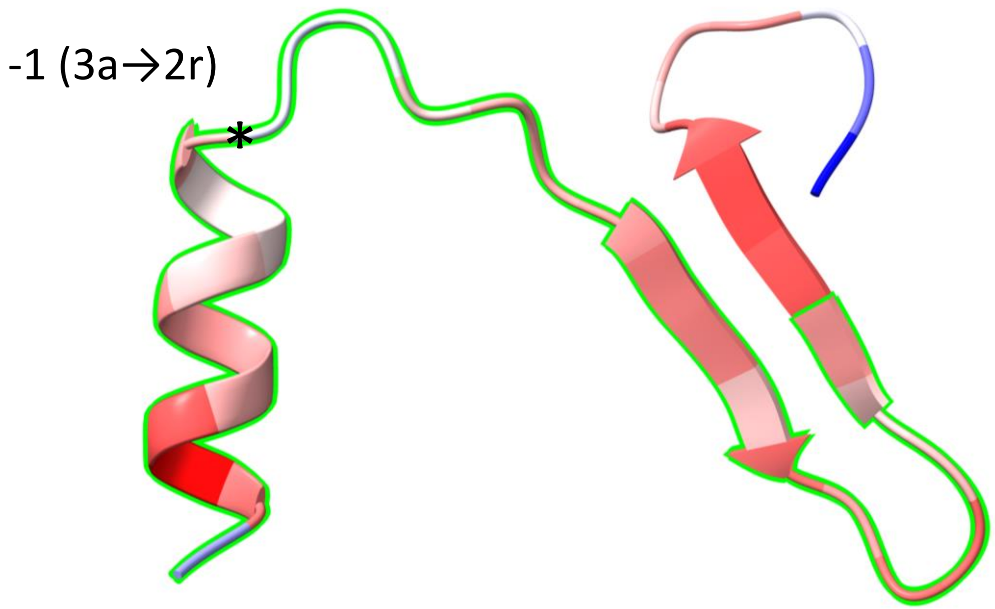

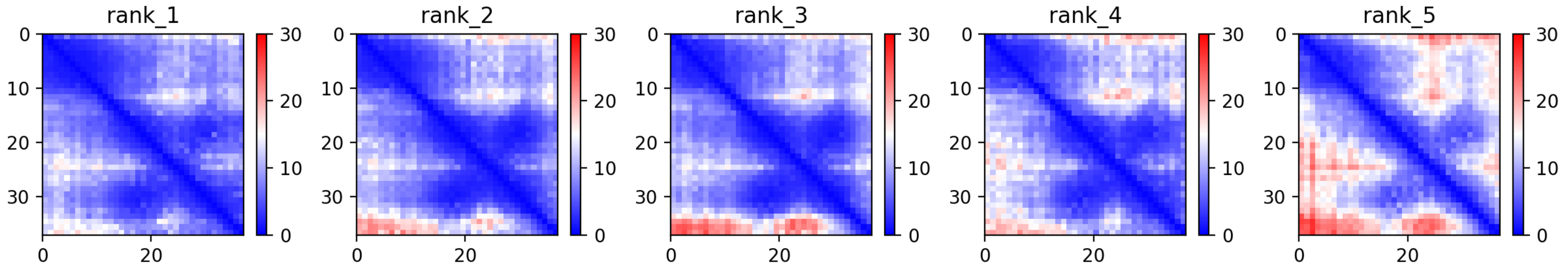

CP46: MtrunA17_Chr2g0329031

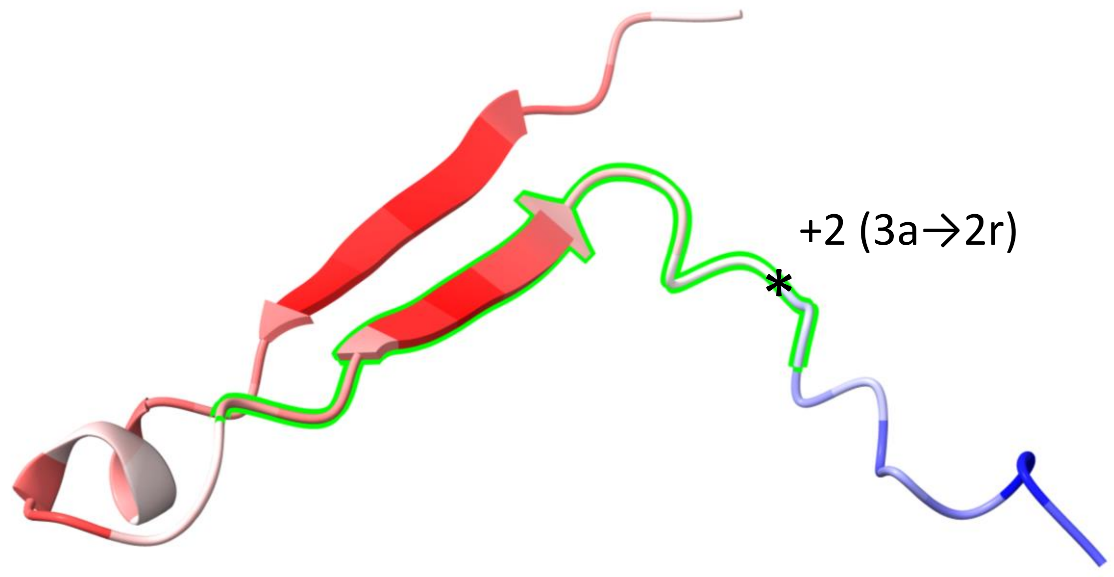

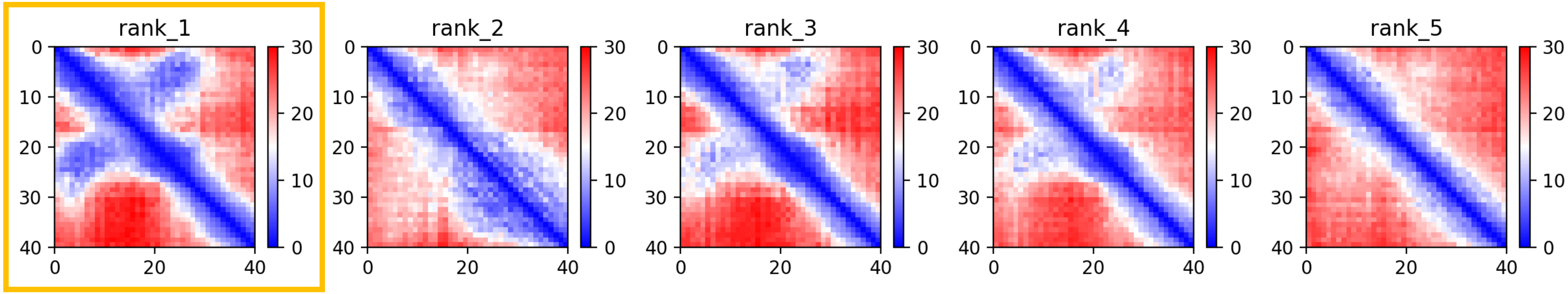

CP60: MtrunA17_Chr3g0135761

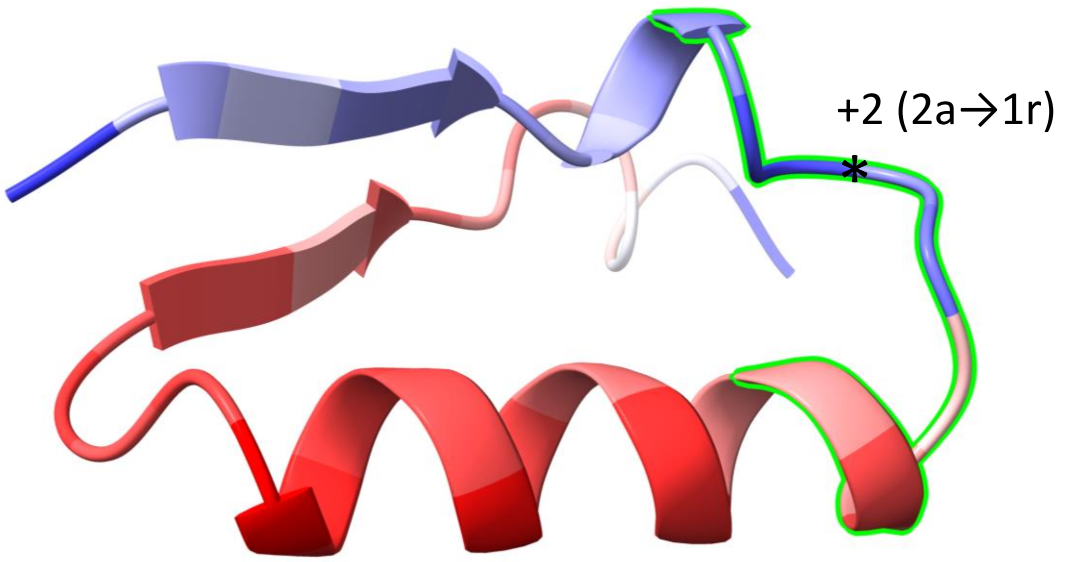

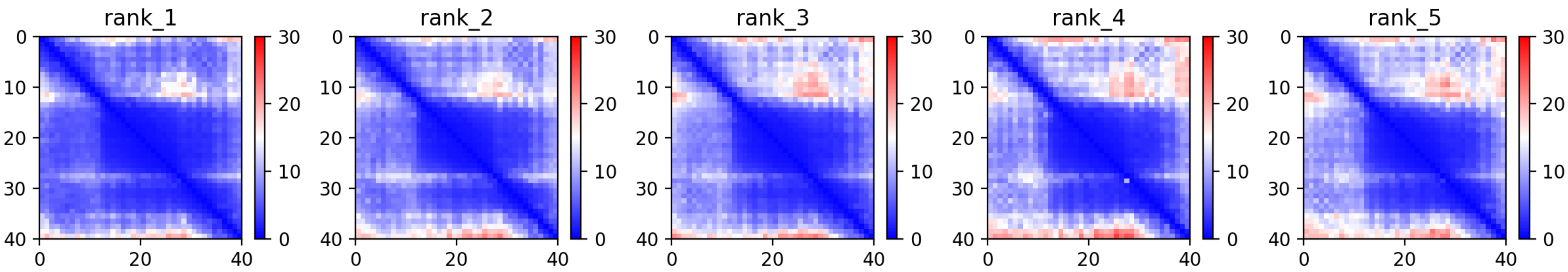

CP71: MtrunA17_Chr4g0022491

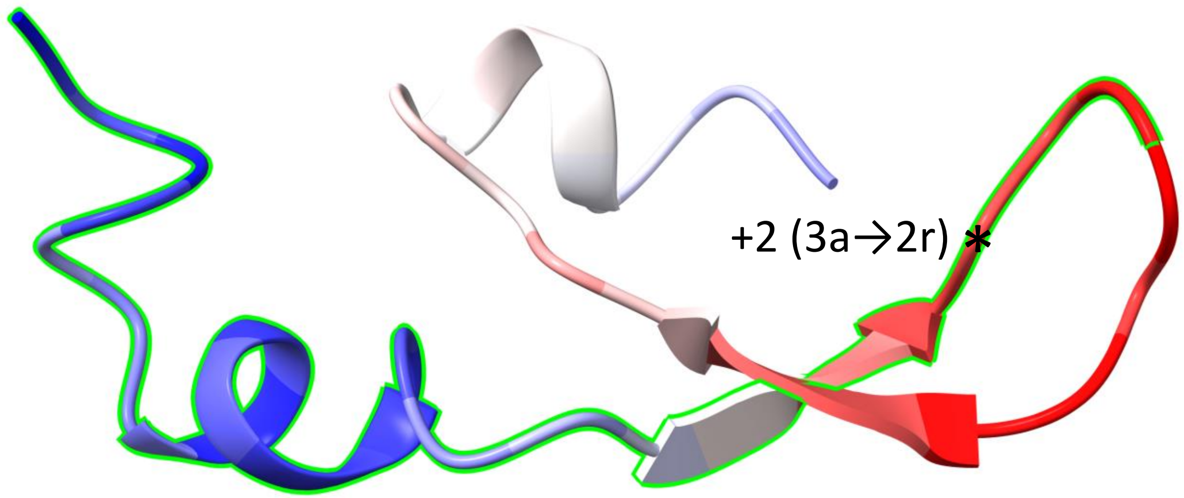

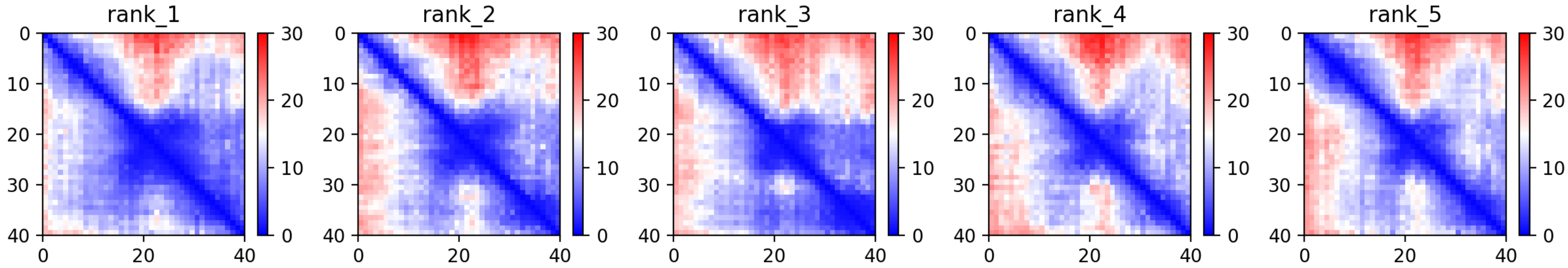

CP78: MtrunA17_Chr4g0059001

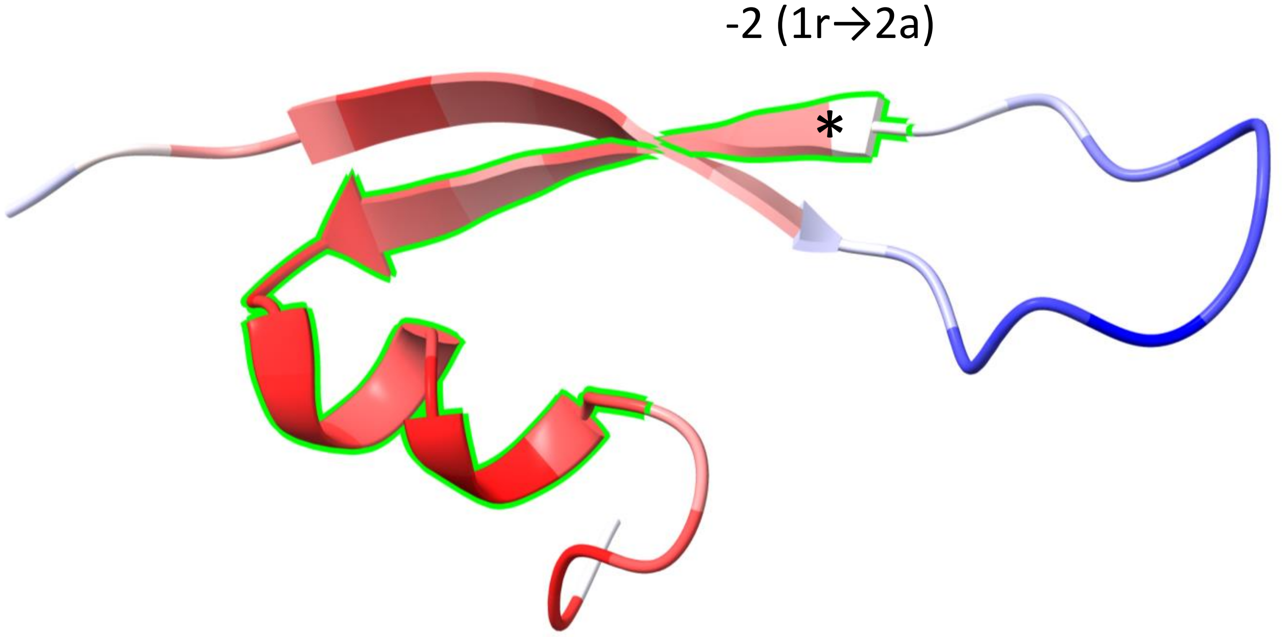

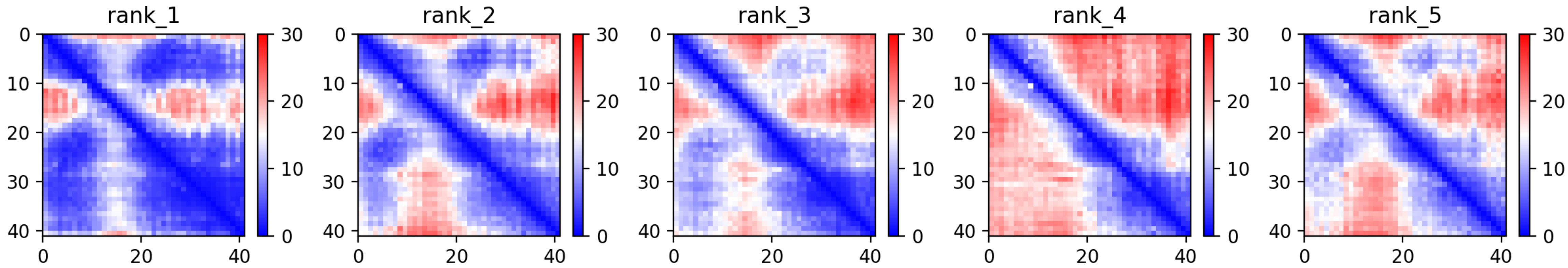

CP79: MtrunA17_Chr4g0063201

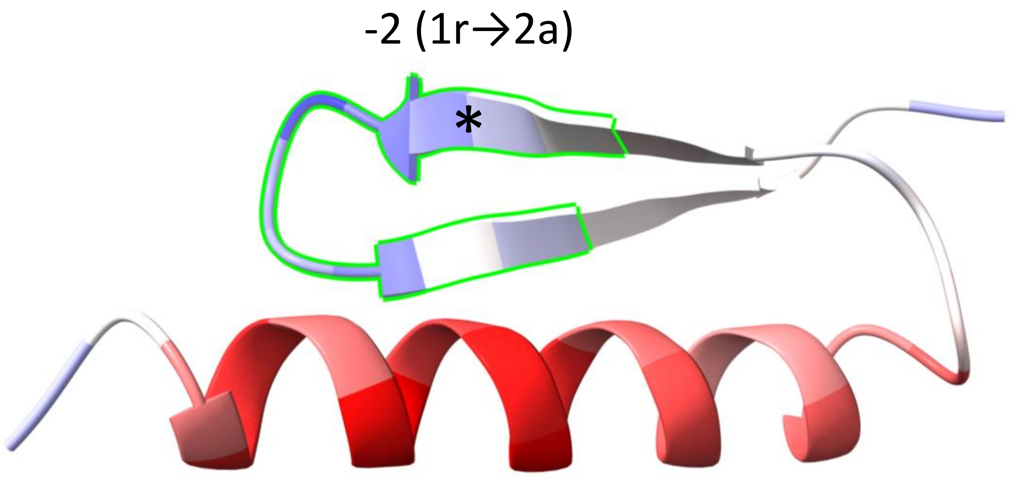

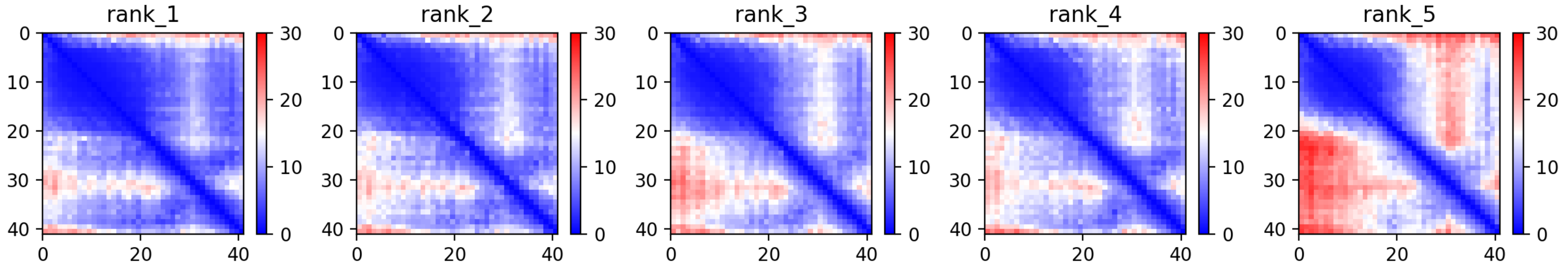

CP99: MtrunA17_Chr6g0457461

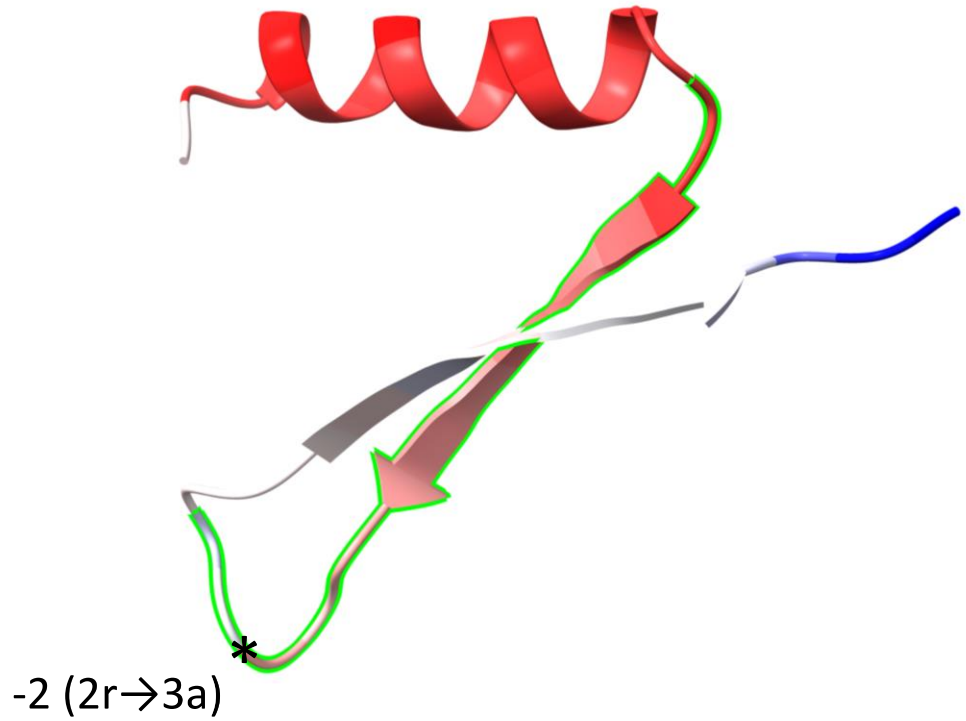

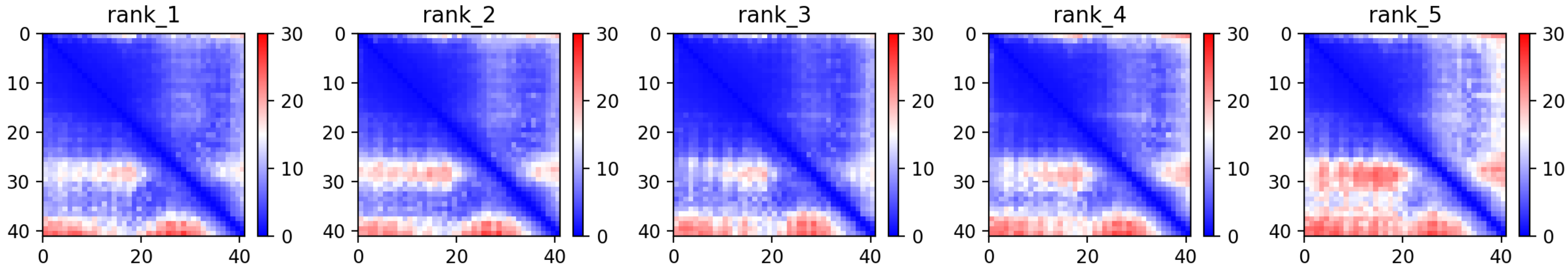

CP100: MtrunA17_Chr6g0457461

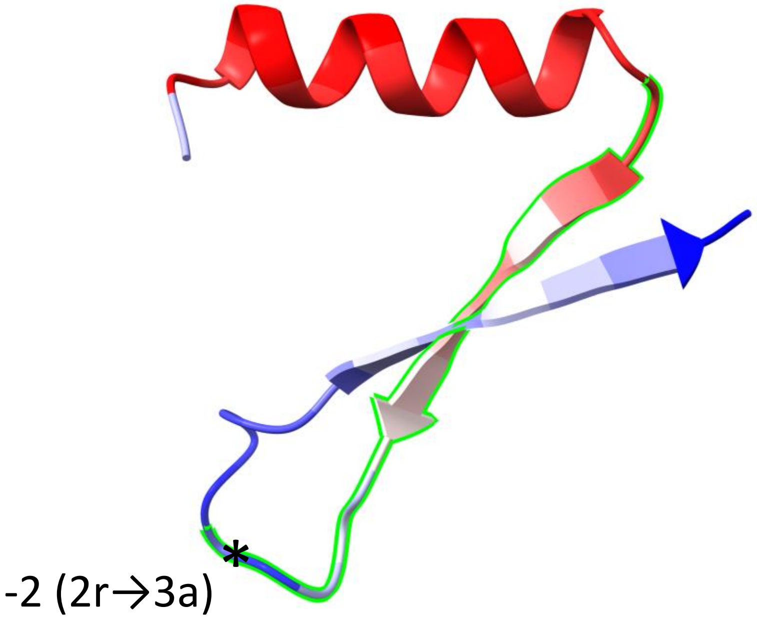

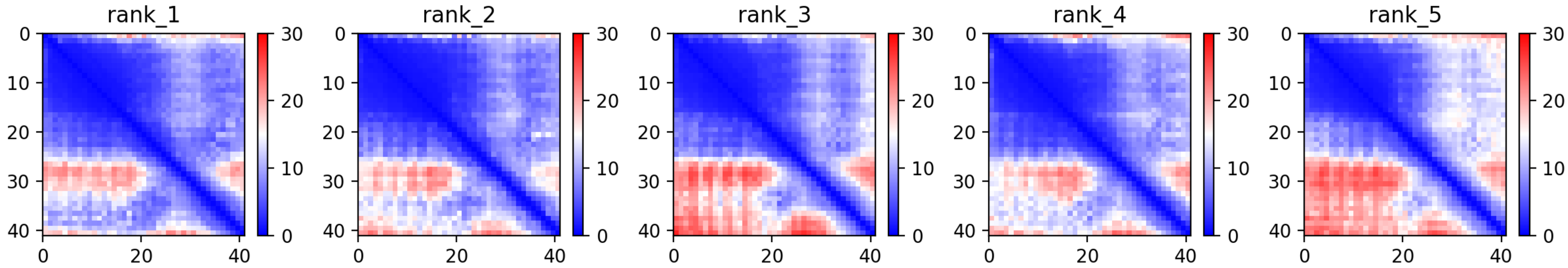

CP101: MtrunA17_Chr6g0458091

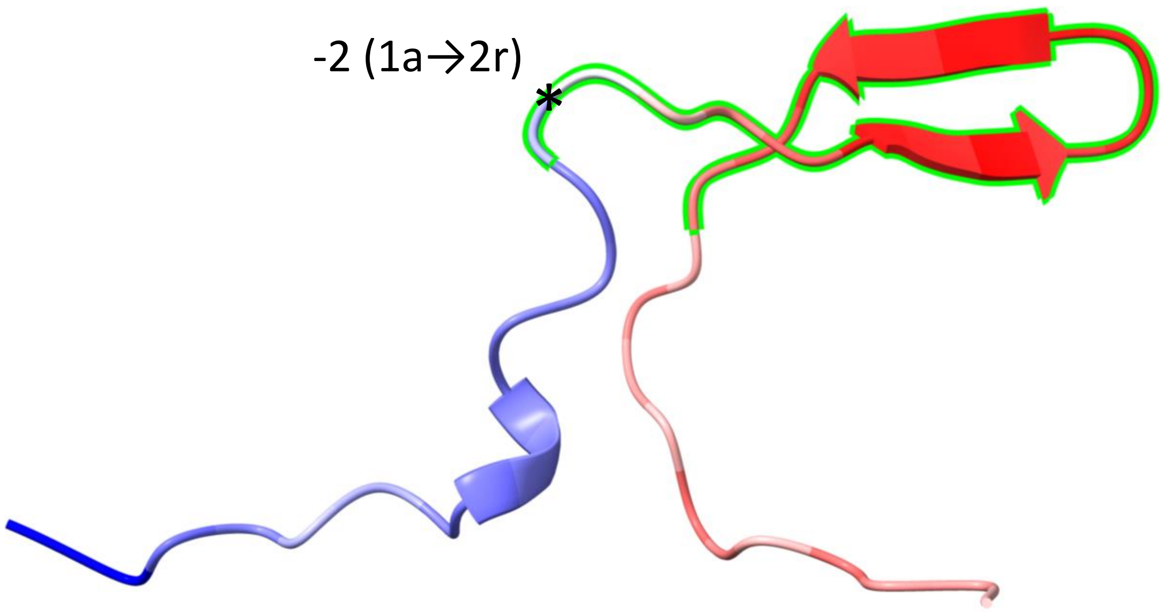

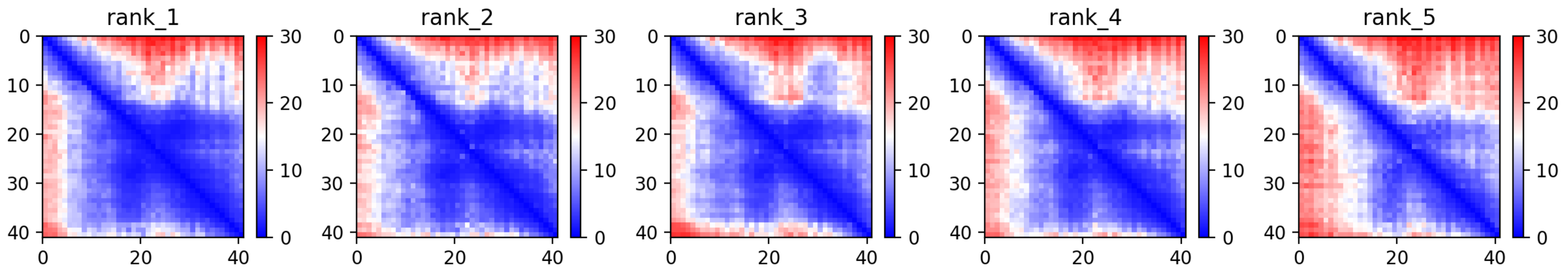

CP103: MtrunA17_Chr6g0461931

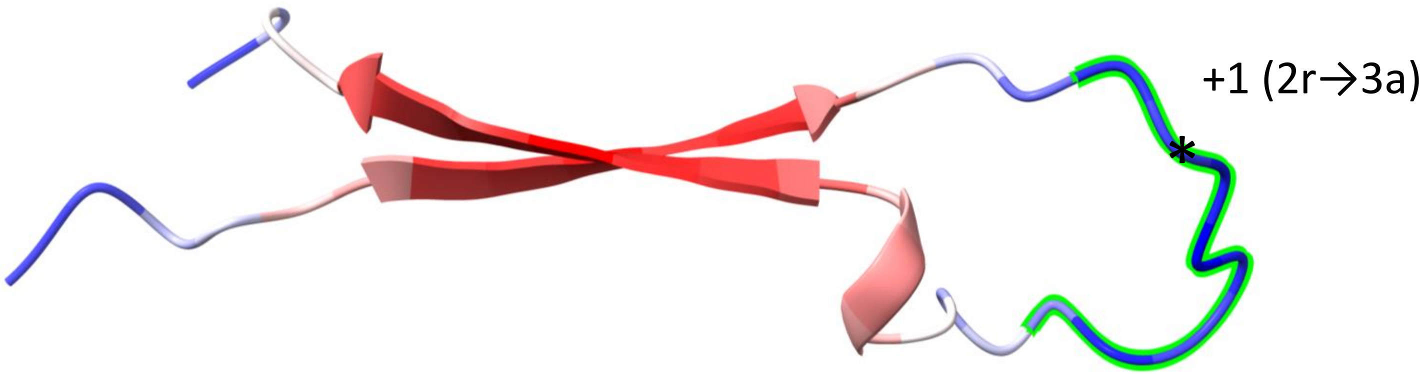

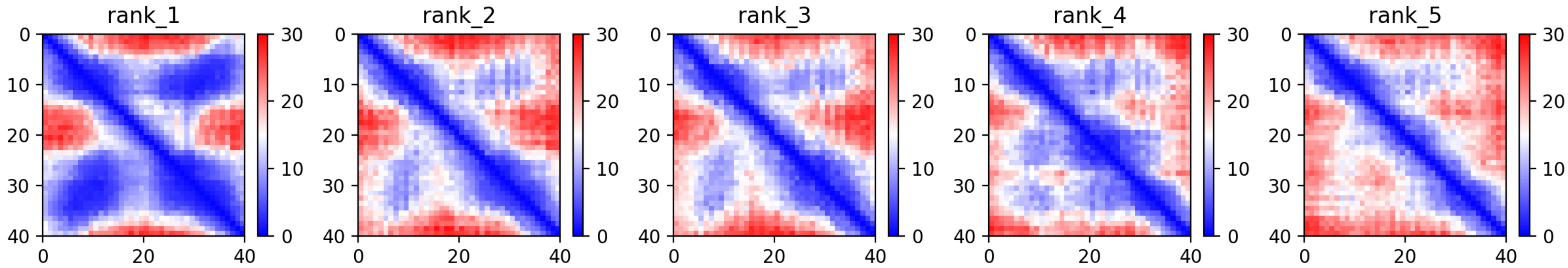

CP108: MtrunA17_Chr6g0485321

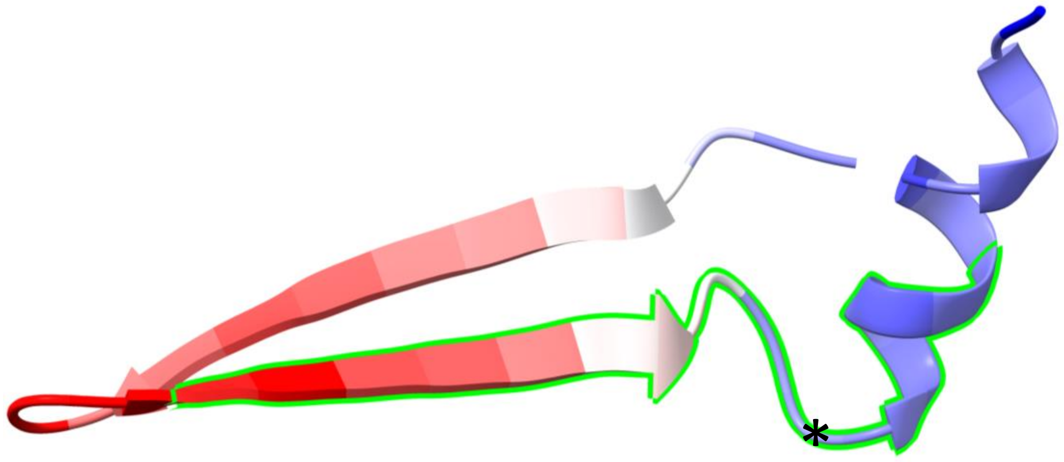

+2 (1a2→3a1)

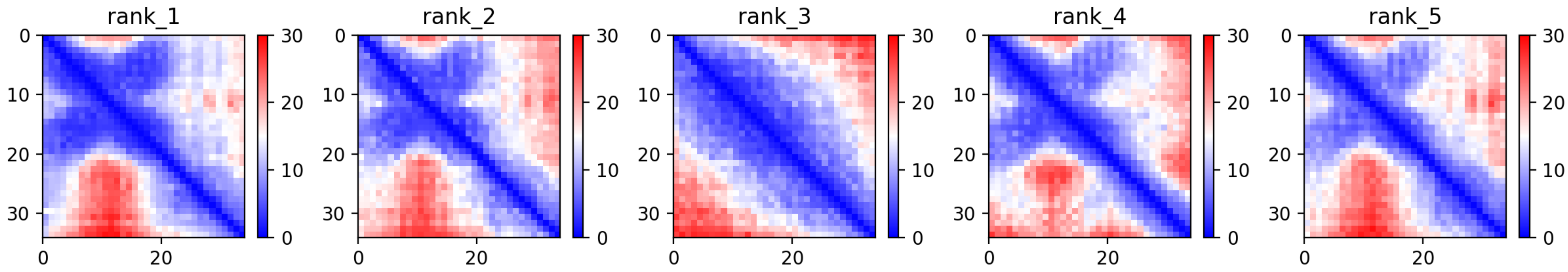

CP136: MtrunA17_Chr8g0373091

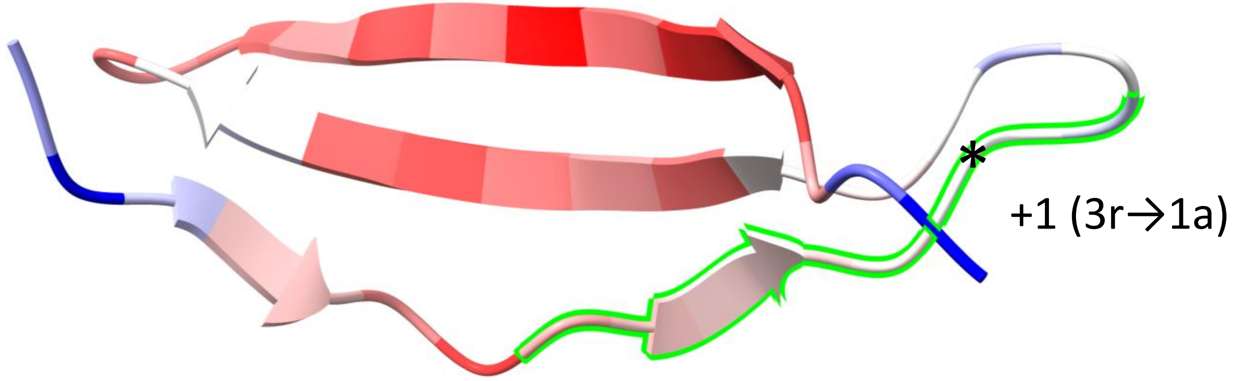

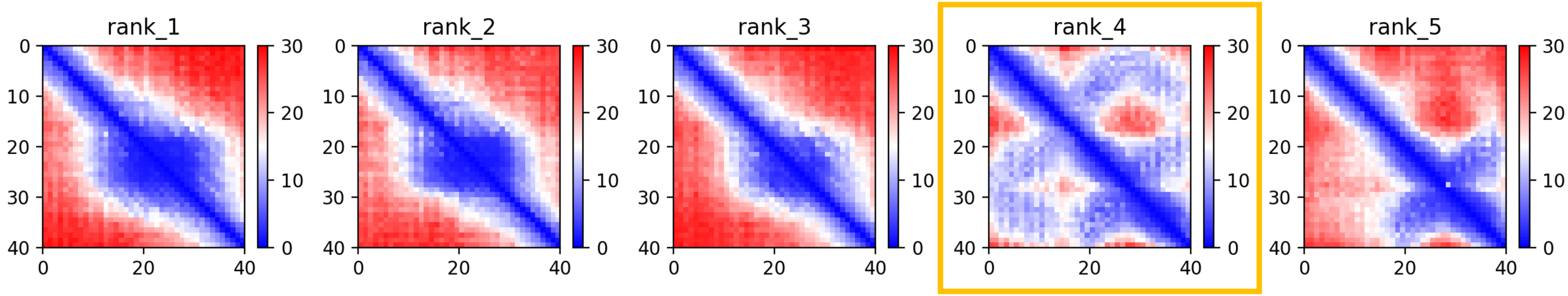

CP139: MtrunA17_Chr8g0385331

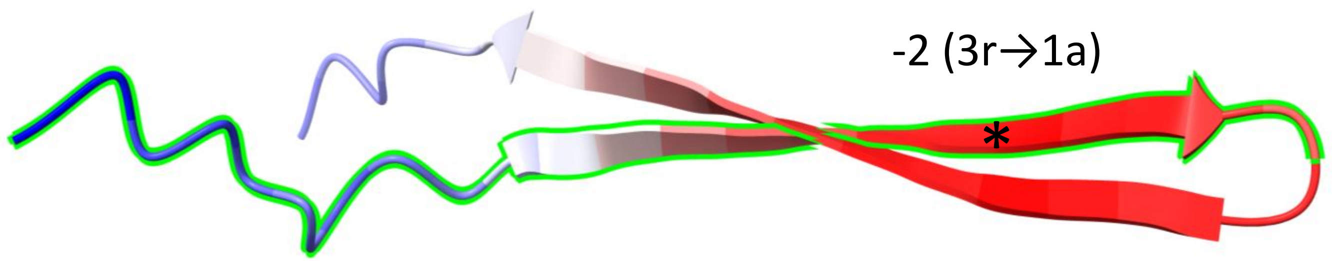

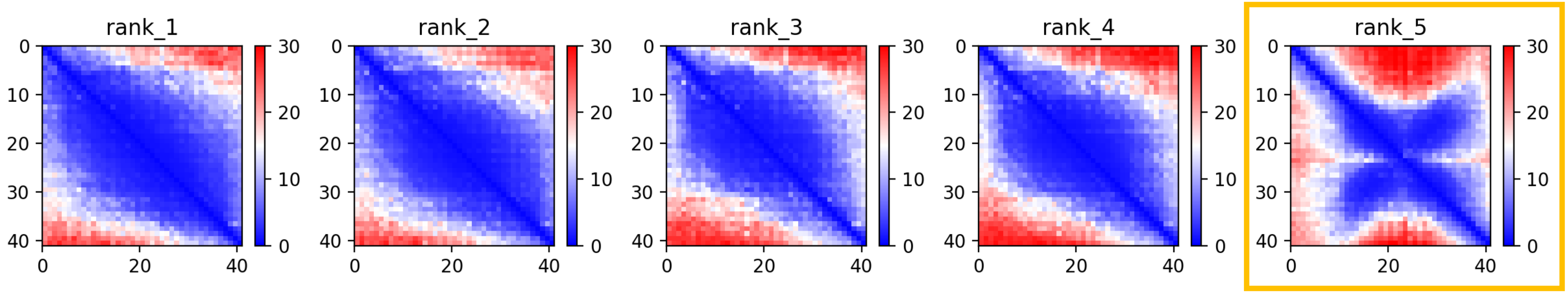

CP149: MtrunA17_MTg0490471

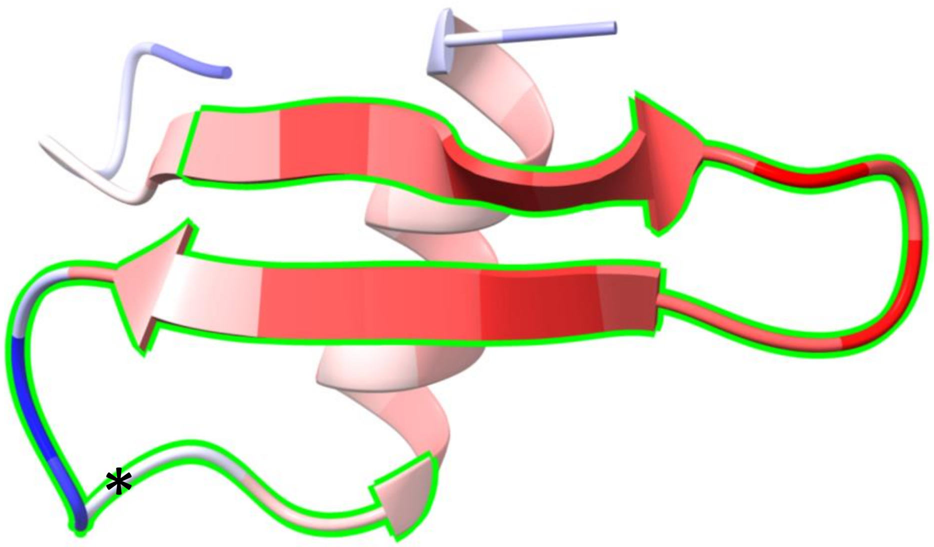

+1 (1r→2a)

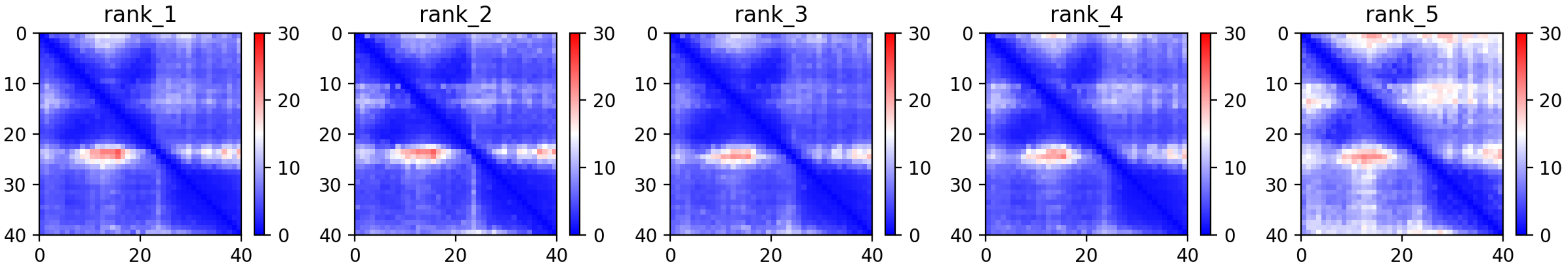

CP154: MtrunA17_MTg0491501

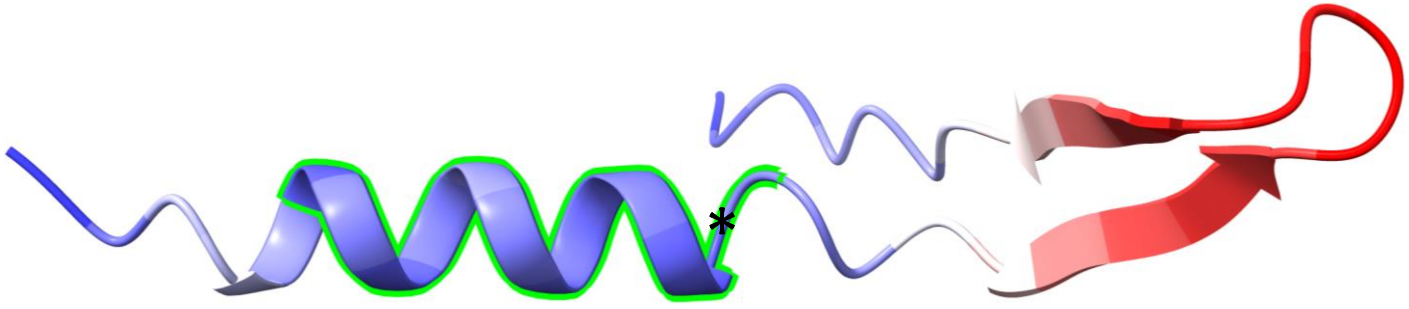

-2 (3a1→1a2)

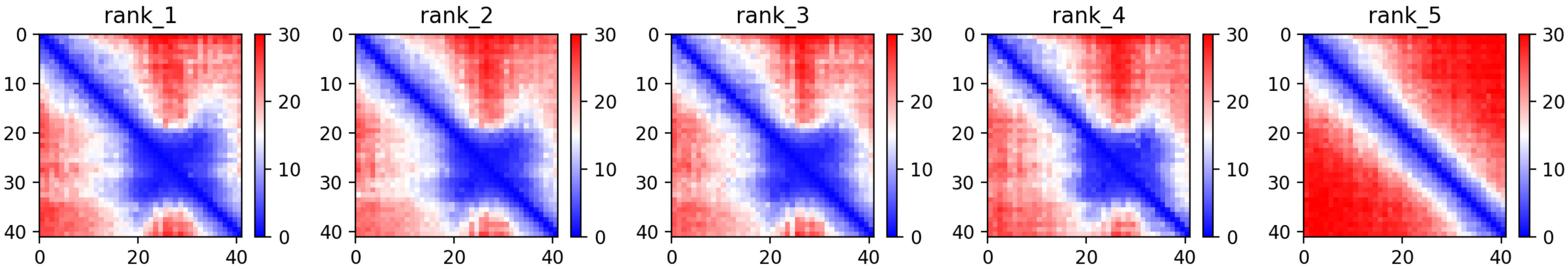

Supplementary Dataset S7 Part 5. A graphical summary on folding predictions for 24 MS-supported chimeric peptide models (CPs) that contain beta-sheets regardless of the presence of alpha-helices. For each CP, one image is displayed: the predicted folding structure (top) along with heat maps of five predictions (bottom). The rest of the legend is the same as for Supplementary Dataset S7 Part 1.
